# Why is a dicationic digallene so reactive towards activation of strong covalent bonds? Scope and mechanistic investigations

**DOI:** 10.1039/d5sc09508e

**Published:** 2026-02-02

**Authors:** Antoine Barthélemy, Nico Gino Kub, Celine Regnat, Harald Scherer, Ingo Krossing

**Affiliations:** a Institut für Anorganische und Analytische Chemie and Freiburger Materialforschungszentrum (FMF), Universität Freiburg Albertstr. 21 79104 Freiburg Germany krossing@uni-freiburg.de

## Abstract

Herein, we investigate the reactivity of the *trans*-bent Ga⇄Ga double bond in the dicationic digallene [{Ga(dcpe)}_2_]^2+^ (dcpe = bis(dicyclohexylphosphino)ethane) as its [*pf*]^−^ salt ([*pf*]^−^ = [Al(OR^F^)_4_]^−^; R^F^ = C(CF_3_)_3_), which is formed *in situ* within seconds. Unusually, this digallene is highly reactive towards covalent bonds and oxidatively adds even to strong E–Y σ-bonds, *e.g.*, H–O, H–N, H–C and C–F bonds, under mild conditions, often at room temperature. Their bond activation at any cationic subvalent group 13 compound is unprecedented and the C–H bond activation is the first oxidative addition reported between any subvalent gallium compound and a neutral substrate. The scope and mechanism of the bond activation reactions were experimentally investigated by interaction with selected substrates and *via* isotope labelling experiments, as well as using high-level quantum chemical calculations. Mechanistically, the pronounced reactivity of the digallene can be attributed to an easily accessible asymmetric conformer with one (Lewis-acidic) planarized and one (Lewis-basic) pyramidalized reactive Ga-site, allowing for cooperative E–Y bond cleavage. In addition, the [2 + 2] cycloaddition of the Ga⇄Ga bond to C=C double and triple bonds was studied: it follows a stepwise, non-concerted reaction mechanism, which allows for the catalytic isomerization of *cis*-olefins and may serve as the basis for follow-up functionalization reactions.

## Abstract 2

The reactivity of a dicationic digallene towards C=C double bonds and very strong single bonds is explored. The underlying reaction mechanisms involve an asymmetric dimer and were investigated and rationalized with quantum chemical calculations.

## Graphical abstract

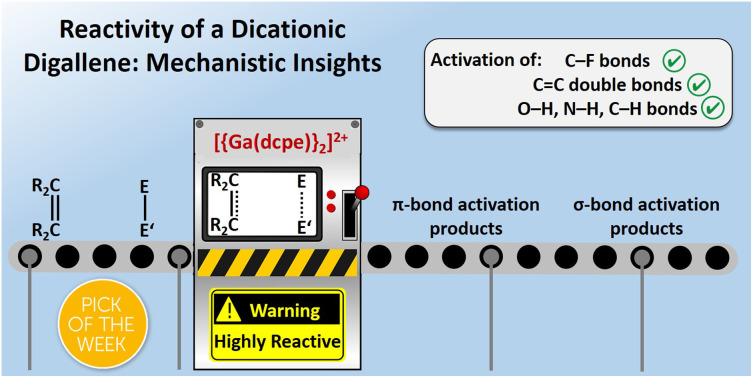

## Introduction

**Scheme 1:**
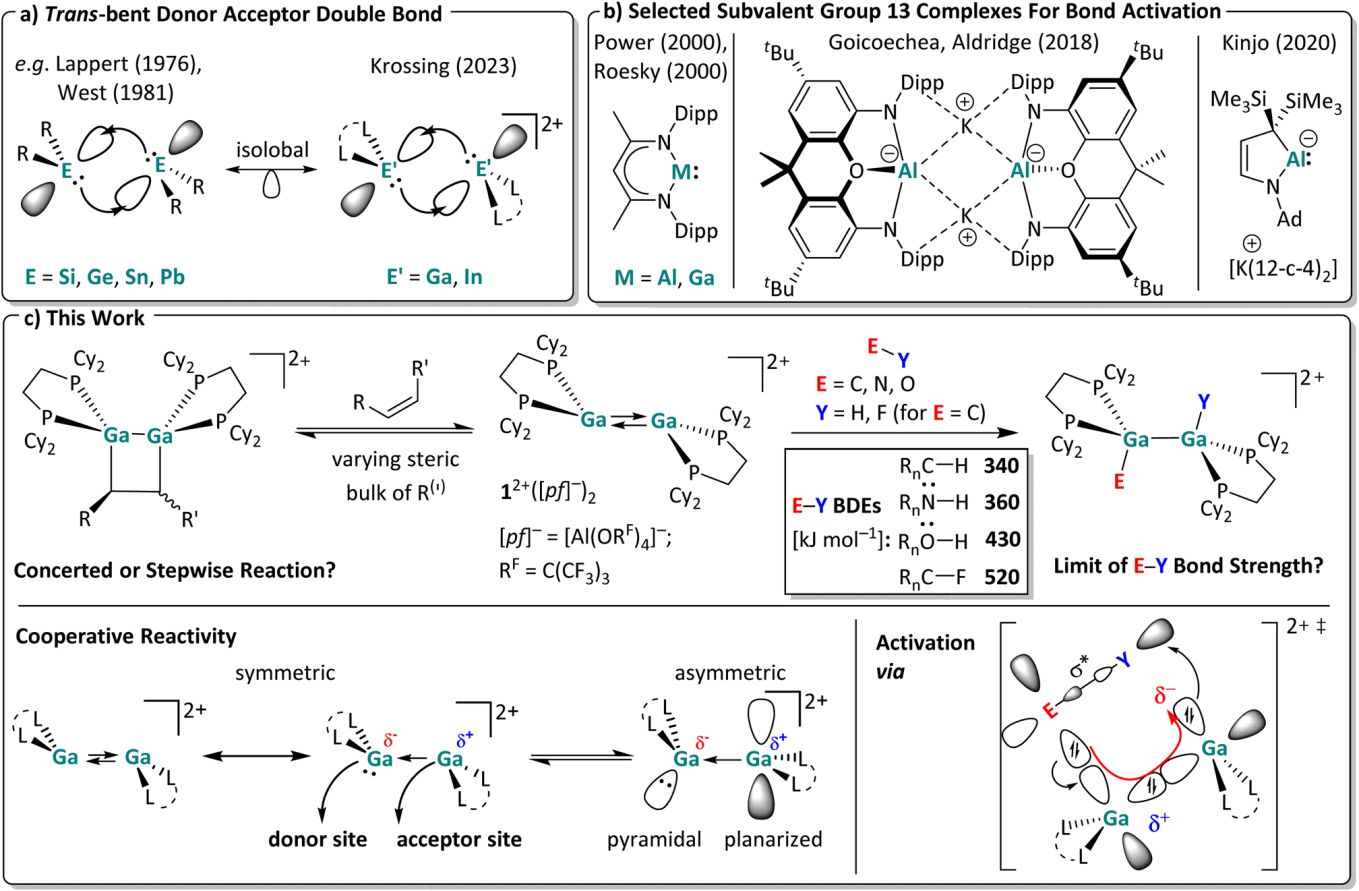
(a) Orbital interactions of two singlet fragments in a *trans*-bent double bond for ditetrylenes and ditrielenes.^7,28,29^ (b) Selected examples for subvalent group 13 complexes used for bond activation reactions (Dipp = 2,6-^*i*^Pr_2_C_6_H_3_; Ad = adamantyl; 12-*c*-4 = 12-crown-4).^23–26^ (c) Scope of this work. The activation of both π-bonds and very strong σ-bonds was investigated with the dicationic digallene [{Ga(dcpe)}_2_]^2+^. Published average bond dissociation energies (BDEs) are also included,^30–32^ which are heavily influenced by the substituents R_*n*_; only rough average values are given. At the bottom, the crystallographically found symmetric and an accessible asymmetric conformer relevant for the cooperative reactivity of the digallene 1^2+^ are shown. Trends of their partial charges and relevant orbital fragments are drawn in for the Ga atoms, as well as the suggested transition state important for the σ-bond activation reactions.

Although transition metal catalysts are widely employed in industry, their scarcity, high cost, and the environmental burdens associated with their mining continuously drive research towards more sustainable metal alternatives. In this context, low valent main group metal compounds have demonstrated their potential as viable alternatives to transition metal catalysts, as main group elements tend to be more abundant and cost efficient.^1–4^ With orbital hybridization between valence orbitals getting less pronounced as one descends in any given *p*-block group, most heavier low valent main group carbene analogues occupy a singlet ground state. In this state, the combination of a filled ns-type orbital and vacant np-type orbital leads to both, electrophilic and nucleophilic properties, which enable insertion into various σ-bonds by oxidative addition.^3,4^ Analogous to carbenes, heavier low valent group 13 and 14 compounds can dimerize into ditrielenes or ditetrylenes, respectively.^5^ Yet, the nature of the thereby formed formal double bond differs greatly from typical C=C double bonds. Their bonding situation is best described as a double-dative bond with a *trans*-bent geometry, in which the heavier singlet trielene/tetrylene fragments reciprocally donate electron density from the filled n*s*-orbital to the empty np-orbital of the adjacent fragment,^5–7^ as visualized in Scheme 1a. Oligomerization is only suppressed with suitable sterically demanding substituents R, as first demonstrated by Lappert and West with a stable distannene^8^ and a disilene.^9^ Considerable follow-up research efforts led to the isolation of a multitude of *trans*-bent digermene,^10^ distannene^11^ and diplumbene structures.^8,12^ Notably, these heavier ditetrylenes, in particular disilenes and digermenes, readily activate covalent σ- and π-bonds.^13,14^ After the isolation of the first molecular digallene by Power *et al.*,^15^ the chemistry of (neutral) ditrielenes also evolved into a major research field.^16–18^ However, reports of σ-bond activation reactions with group 13 dimers are still rather scarce.^17,19,20^ Instead, oxidative addition reactions with subvalent group 13 compounds are usually performed at monomeric complexes, or, if dinuclear, those without a direct group 13 metal–metal interaction. Such compounds are almost invariably neutral or anionic, exhibit high reactivity towards covalent bonds, but require the use of sophisticated ligands with multi-step syntheses.^21–26^ Selected examples are shown in Scheme 1b, with the anionic aluminyl anions, introduced by the Aldridge-group, ranking amongst the most reactive compounds that even activate C–H and C–C bonds, which is attributed to a high-lying HOMO and a small HOMO–LUMO gap.^27^

Yet, to allow the progress from mere bond activation/oxidative addition to true catalysis with relevant bonds, the reverse process – the reductive elimination – also needs to be energetically feasible. And, while anionic and neutral subvalent main group complexes typically undergo irreversible oxidative addition reactions impeding catalytic applications with such substrates, a few reductive elimination reactions at cationic gallium complexes have been reported.^33–36^ However, oxidative additions at cations are generally less favorable due to reduced orbital energies. Thus, a major goal in cationic group 13 chemistry is to identify metal/ligand combinations that enable reversible substrate activation, in order to realize true main group catalysis.^37,38^ However, bond activation at group 13 cations is still in its infancy, a fact to be overcome with this contribution.^29,35,39,40^

Favorably, and in contrast to other compounds with *trans*-bent double bonds requiring time-consuming multi-step syntheses, we recently obtained [{Ga(dcpe)}_2_][*pf*]_2_ ([*pf*]^−^ = [Al(OR^F^)_4_]^−^; R^F^ = C(CF_3_)_3_; dcpe = (bis(dicyclohexyl-phosphino)ethane)) *in situ* within a matter of minutes by mixing [Ga(PhF)_2_][*pf*],^41^ a stable source of univalent gallium, with the commercially available bisphosphine dcpe in the weakly coordinating solvent *o*DFB (*ortho*-difluorobenzene).^29^ The large, weakly coordinating [*pf*]^−^ anion^33,42^ prevents disproportionation of the dicationic *trans*-bent digallene [{Ga(dcpe)}_2_]^2+^ (1^2+^), which is isoelectronic and isostructural to neutral ditetrylenes (Scheme 1a). Note that changing the ligand from dcpe to sterically leaner systems led to the formation of polycationic gallium clusters with Ga–Ga single bonds.^37,43^ By contrast, the use of increasingly bulkier bisphosphine ligands allows for the formation of either dimeric or monomeric (di)gallenes, which, for example, with dipf as a ligand (dipf = 1,1′-(^*i*^Pr_2_P)_2_Fc), exhibit a temperature-dependent monomer–dimer equilibrium in solution.^40^ With the latter ligand, monomers and dimers already displayed Si–H and B–H bond activation potential, but the reactivity of the monomer and dimer is hard to separate for mechanistic studies.^40^

**Fig. 1:**
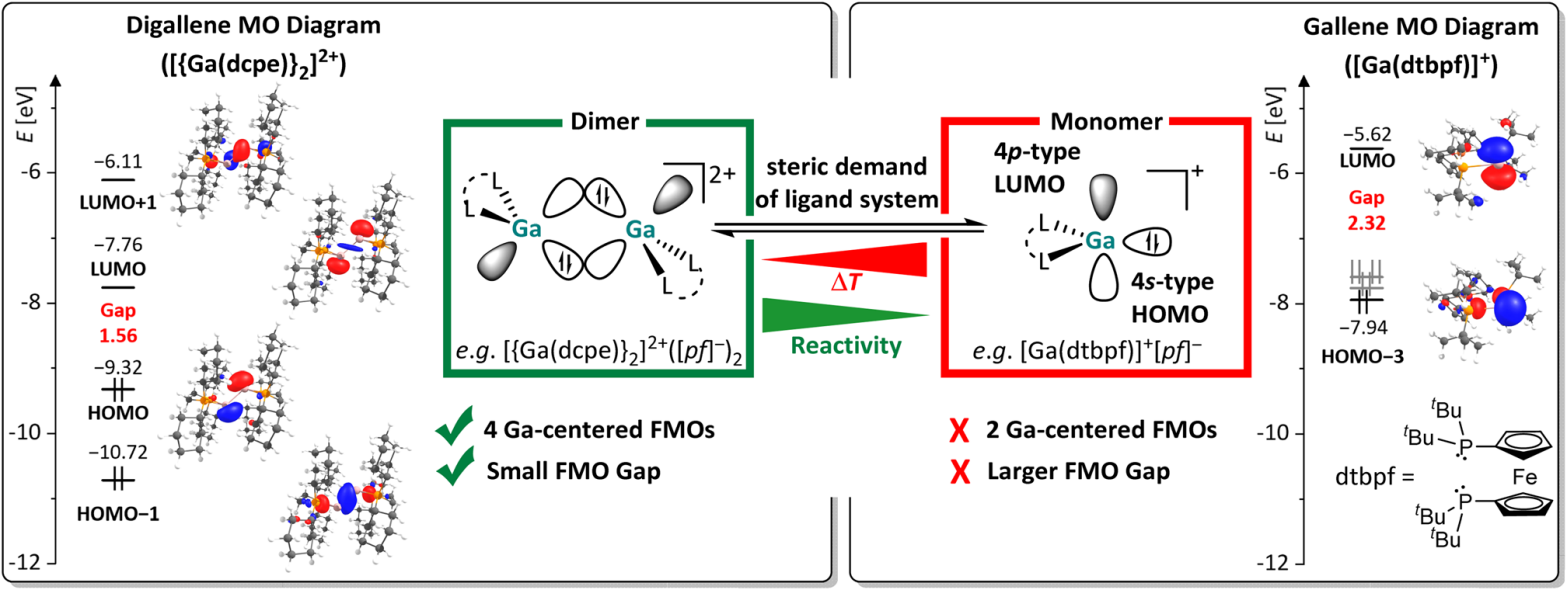
Comparison of the frontier molecular orbitals (FMOs) of monomeric and dimeric low valent cationic Ga^I^ compounds, using the example of the monomeric [Ga(dtbpf)]^+^ (1,1′-bis(ditertbutylphosphino)ferrocene) and dimeric [{Ga(dcpe)}_2_]^2+^ systems calculated at the RI-BP86(BJ)/def2-TZVPP level of theory (isosurface value: 0.05 au). The HOMO and HOMO(−1/−2) of [Ga(dtbpf)]^+^ are ligand-centered and only have small coefficients on the Ga atom. It should be noted that the HOMO–LUMO gap is even greater for the hypothetical [Ga(dcpe)]^+^ monomer (HOMO: −8.34 eV; LUMO: −5.61 eV; gap: 2.74 eV).

Hence, in this work we targeted reactions with the [{Ga(dcpe)}_2_]^2+^ (1^2+^) system that is a stable dimer under all conditions tested (Scheme 1c).††The previously calculated thermodynamic values for the dissociation of 1^2+^ in *o*DFB (Δ_r_*H*° ≈ +105 kJ mol^−1^ and Δ_r_*G*° ≈ +43 kJ mol^−1^ at 298 K)^29^ indicate that the complex largely remains dimeric in solution, not only at rt, but also at slightly elevated temperatures, *e.g.* at 60 °C. In line with this, diluting or heating the sample at 60 °C does not lead to a significant colour change of the dark red solution. By contrast, monocationic, monomeric gallenes are reported to produce yellow solutions.^40^ Therefore, in order to play a role in the bond activation reactions, the monomer, which may be present in extremely small quantities, would have to be considerably more reactive than the dimer. This contradicts not only the FMO analysis (Fig. 1) but also our experience with similar, cationic (di)gallene systems.^40^ Finally, the most important observation is that the reactions of 1^2+^ and one equivalent of the substrate E–Y yield exclusively dimeric products, as confirmed by NMR spectroscopy and scXRD. As demonstrated previously, monomeric Ga^III^ addition products can be obtained, but only with a second equivalent of the substrate E–Y and *via* the dimeric Ga^II^ addition products.^29^ In the present work, a monomeric intermediate was only observed with a slight surplus of HNPh_2_, but already at rt, implying that the bulky amine partially breaks down the dimer (Scheme 9). These results strongly suggest that no monomeric species are involved in the bond activation reactions presented herein and that the dimeric 1^2+^ acts as the active species in solution. We tested its limit for bond activation towards π and very strong σ bonds. In particular, the presence of an asymmetric conformer with separate donor and acceptor sites, as shown in Scheme 1c, might play a role in the observed bond activation reactions. In favor of this, preliminary work showed that 1^2+^ undergoes [2 + 2] cycloaddition reactions with unsaturated substrates and oxidative addition reactions, albeit only with rather weak S–S and P–H bonds, mimicking to some extent the reactivity of the well-known neutral ditetrylenes.^1,29^ Its full scope is analyzed here.

## Results and discussion

First, we describe all experiments, before analyzing the underlying mechanistic aspects in the second part, further supported by double-hybrid DFT calculations and bonding analyses. However, where sensible, the energetics underlying a given reaction is already included with its first description.

### Part I – bond activation reactions with the dicationic digallene

#### Reactions of [{Ga(dcpe)}_2_][*pf*]_2_ with C=C double and C≡C triple bonds

**Scheme 2:**
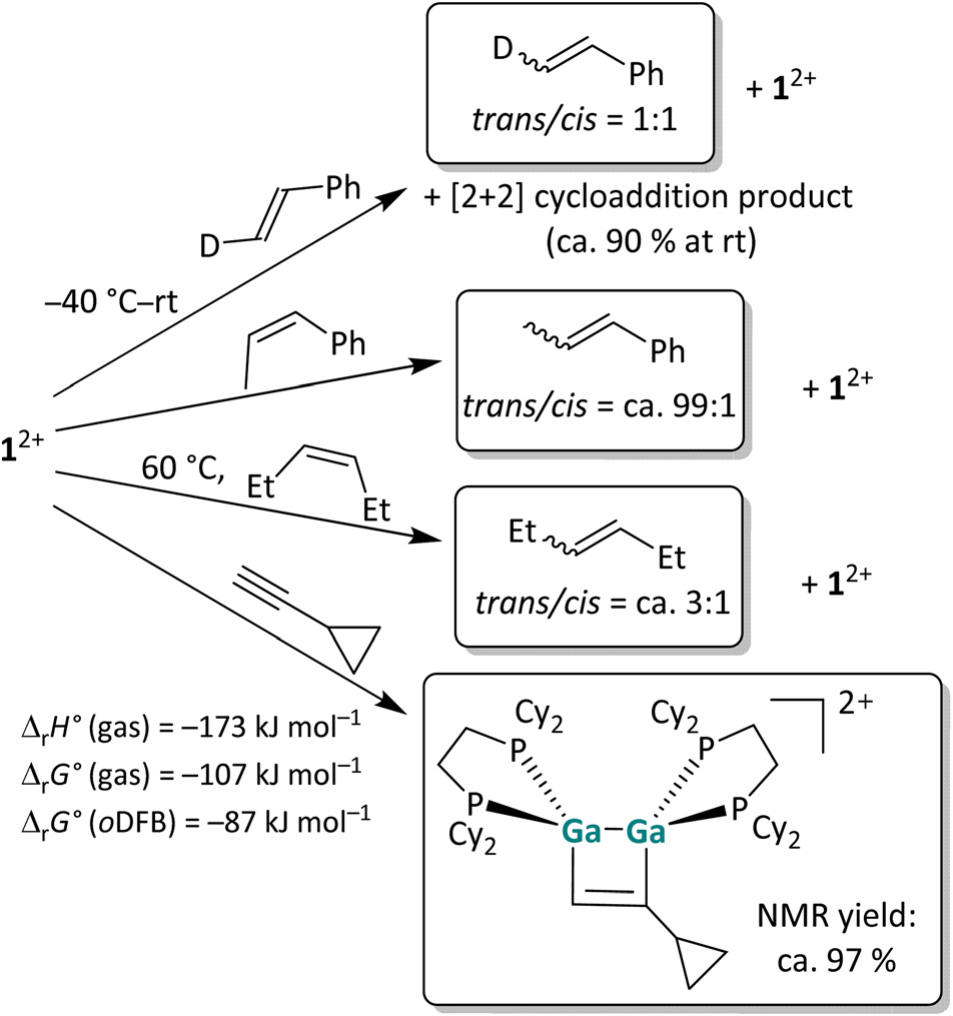
Overview of the reactivity of 1^2+^ towards three olefins with increasing steric protection of the double bond and the “radical clock” ethinylcyclopropane in *o*DFB. The [*pf*]^−^ anion is omitted for clarity. The reactions were carried out at rt in *o*DFB and examined *via* NMR spectroscopy. The calculated reaction enthalpies and Gibbs free energies in the gas phase and in *o*DFB are also given for the cycloaddition reaction (accurate^44^ DSD-PBEP86-D3(BJ)/def2-QZVPP(C) single point calculations on RI-BP86(D3BJ)/def2-TZVPP optimized structures; *ε*_r_ = 13.38).^32^

To analyze the reaction mechanism of the [2 + 2] cycloaddition reaction, digallene 1^2+^ was reacted with selected alkenes and alkynes to determine whether the reaction follows a stepwise or a concerted mechanism. *Trans*-deutero-styrene, *cis*-β-methyl-styrene, *cis*-3-hexene and ethinyl-cyclopropane were chosen as suitable substrates. The reaction outcomes are summarized in Scheme 2. Unless otherwise stated, the reactions were carried out at room temperature (rt) in *o*DFB.

##### *Trans*-deutero-styrene

In the reaction of a dipf-substituted dicationic digallene with styrene, an equilibrium between the starting material and the [2 + 2] cycloaddition product was observed.^40^ Also with 1^2+^, no full conversion of the olefin is observed and a 9 : 1 ratio between the cycloaddition product and the free olefin is obtained at rt in solution. More importantly, ^1^H NMR spectroscopy reveals that already at −40 °C, before any cycloaddition products are observable, *trans*-deutero-styrene is converted to a 1 : 1 mixture of the *trans* and *cis* isomers in the presence of 1^2+^ (Section 4.1.1 in the SI). The fast isomerization, even with a sterically unhindered olefin, presents evidence that the [2 + 2] cycloaddition reaction of dicationic digallenes does not follow a concerted but rather a stepwise mechanism. Consequently, the reaction needs to proceed *via* intermediates where free rotation around the former C=C double bond is possible.

##### Other olefins

Also, the *cis*-methyl-styrene and *cis*-3-hexene substrates yield a mixture of *cis*- and *trans*-isomers in the presence of 1^2+^, while no cycloaddition product is observed with these substrates. Note that the olefin isomerization can also be performed with catalytic amounts, *i.e.* 10 mol%, of 1^2+^, which makes this reaction an example of Lewis-acid catalyzed *cis*/*trans* olefin isomerization.^45^ The observed *cis*/*trans* ratios depend on the relative stabilities of the stereoisomers in comparison to one another and imply thermodynamic control. Hence, *cis*- and *trans*-deutero-styrene are similarly stable for steric and electronic reasons, while the *trans* isomers of 3-hexene and especially of β-methyl-styrene are more stable than the respective *cis* isomers. As a consequence, the *cis*/*trans* equilibrium ratio increases in this order as determined by ^1^H NMR spectroscopy (Scheme 2). Intriguingly, no [2 + 2] cycloaddition products form with these substrates, probably due to steric constraints and the steric bulk of the cyclohexyl groups. Finally, the clean [2 + 2] cycloaddition reaction with the “radical clock” substrate ethinyl-cyclopropane indicates that the cycloaddition does not proceed *via* radical intermediates, since otherwise a very fast ring opening of the 3-membered ring would be expected.^46^

##### Reaction with a butadiene

**Scheme 3:**
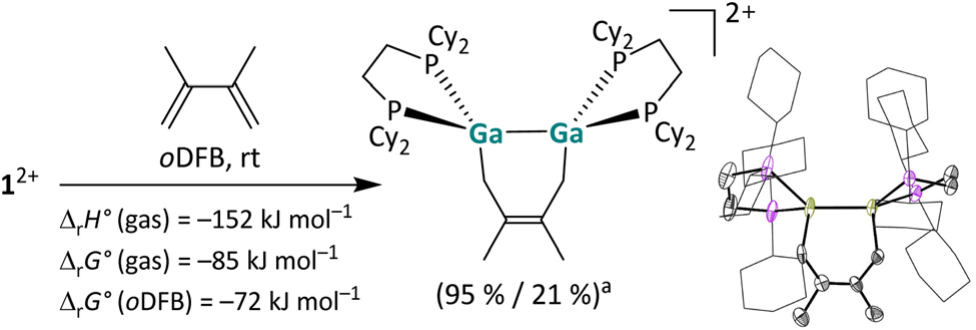
[2 + 4] Cycloaddition of 1^2+^ with 2,3-dimethylbutadiene and the molecular structure of the product [{Ga(dcpe)}_2_(C_6_H_10_)]^2+^ in the solid state (^*a*^ NMR yield/isolated yield). The [*pf*]^−^ anions and hydrogen atoms are omitted for clarity. The cyclohexyl groups are depicted in wireframe for simplicity. Thermal ellipsoids are set at the 50% probability level. The calculated reaction enthalpies and Gibbs free energies in the gas phase and in *o*DFB are also given (DSD-PBEP86-D3(BJ)/def2-QZVPP(C) single point calculation on RI-BP86(D3BJ)/def2-TZVPP optimized structures; *ε*_r_ = 13.38).^32^

In addition, dicationic digallenes not only undergo [2 + 2] cycloaddition reactions, but also [2 + 4] cycloaddition reactions, *i.e.* hetero Diels–Alder reactions,^47^ as described in Scheme 3.^48^ The reaction with 2,3-dimethylbutadiene yields a stable digalla-cyclohexene, which is highly exothermic and exergonic as suggested by quantum chemical calculations and notably thermodynamically more favorable than the [2 + 2] cycloaddition with the non-conjugated olefin 1-hexene (for comparison: Δ_r_*H*° (gas) = −107 kJ mol^−1^, Δ_r_*G*° (gas) = −41 kJ mol^−1^, Δ_r_*G*° (*o*DFB) = −31 kJ mol^−1^).^29^

We already reported a [1 + 4] cycloaddition reaction of a 1,3-diene with a monocationic, gallene monomer.^35^ Furthermore, [2 + 4] cycloaddition reactions are well known for neutral digallenes,^49^ thus emphasizing the structural and electronic relationship of [{Ga(dcpe)}_2_]^2+^ between these substance classes.

#### Reactions of [{Ga(dcpe)}_2_][*pf*]_2_ with strong σ-single bonds and (un)functionalized nitriles

**Scheme 4:**
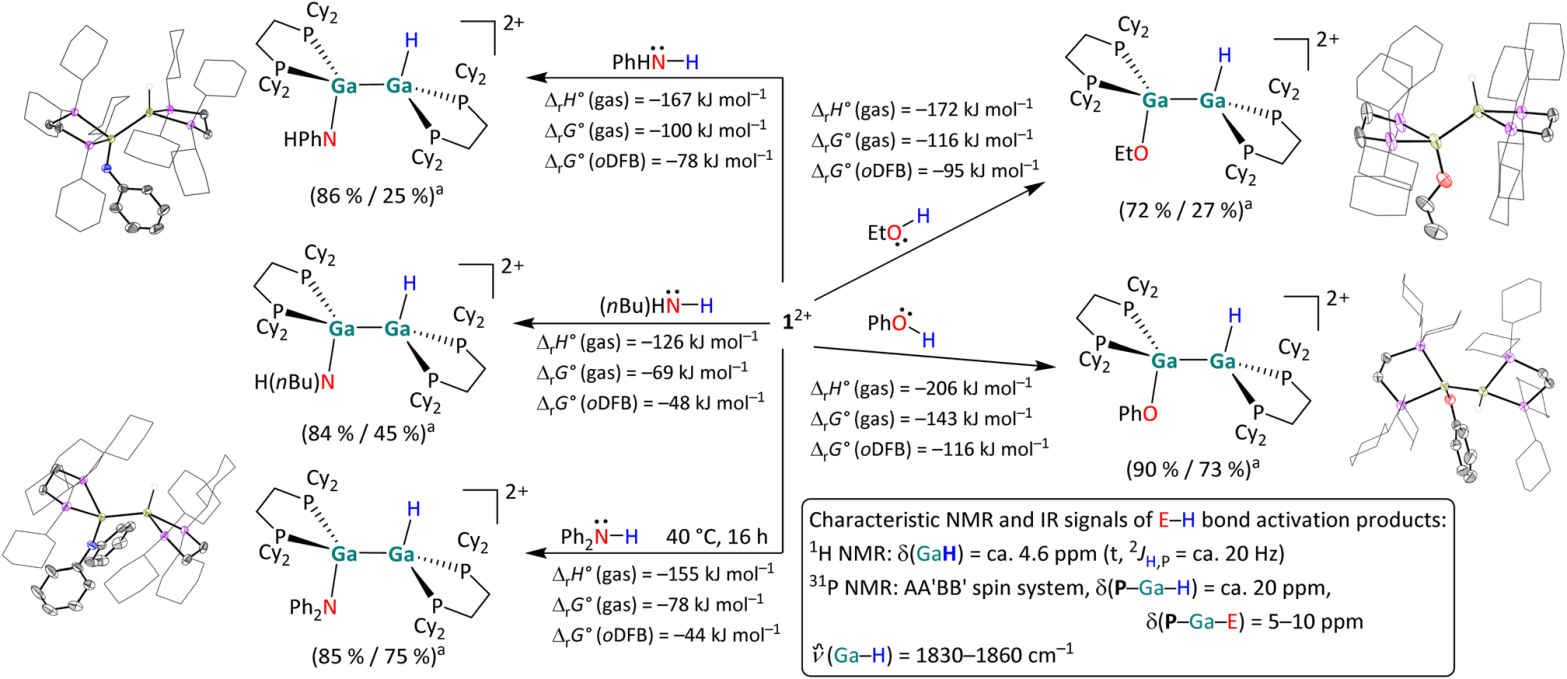
Overview of the reactivity of 1^2+^ towards ethanol, phenol, *n*-butylamine, aniline, and diphenylamine in *o*DFB. Unless otherwise stated, the reactions were carried out at rt (^*a*^ NMR yield/isolated yield). The molecular structures of [H{Ga(dcpe)}_2_OEt]^2+^, [H{Ga(dcpe)}_2_OPh]^2+^, [H{Ga(dcpe)}_2_NHPh]^2+^ and [H{Ga(dcpe)}_2_NPh_2_]^2+^ are included. The [*pf*]^−^ anions and hydrogen atoms – except those bound to Ga – are omitted for clarity. The cyclohexyl groups are depicted in wireframe for simplicity. Thermal ellipsoids are set at the 50% probability level. The calculated reaction enthalpies and Gibbs free energies in the gas phase and in *o*DFB are also given (DSD-PBEP86-D3(BJ)/def2-QZVPP(C) single point calculation on RI-BP86(D3BJ)/def2-TZVPP optimized structures, *ε*_r_ = 13.38).^32^

**Scheme 5:**
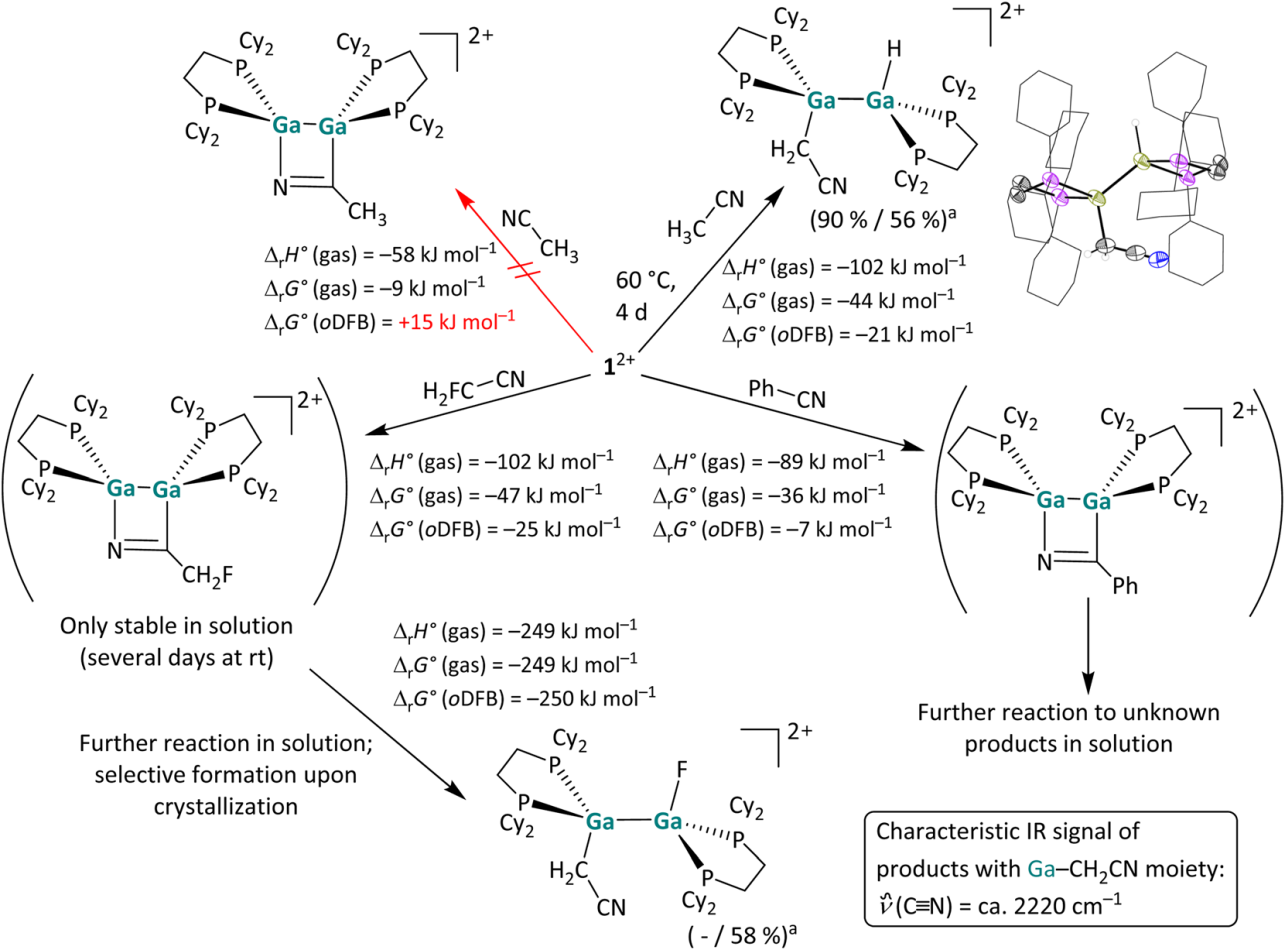
Overview of the reactivity of 1^2+^ towards FH_2_C–CN, Ph–CN and H_3_C–CN. Unless otherwise stated, the reactions were carried out at rt (^*a*^ NMR yield/isolated yield) in *o*DFB. The hypothetical [2 + 2] cycloaddition product with acetonitrile is also shown as well as the actually formed C–H bond activation product. The molecular structure of the resulting [H{Ga(dcpe)}_2_CH_2_CN]^2+^ is included. The [*pf*]^−^ anions and hydrogen atoms – except those bound to Ga and the adjacent methylene group – are omitted for clarity. The cyclohexyl groups are depicted in wireframe for simplicity. Thermal ellipsoids are set at the 50% probability level. The calculated reaction enthalpies and Gibbs free energies in the gas phase and in *o*DFB are given (DSD-PBEP86-D3(BJ)/def2-QZVPP(C) single point calculation on RI-BP86(D3BJ)/def2-TZVPP optimized structures).

**Scheme 6:**
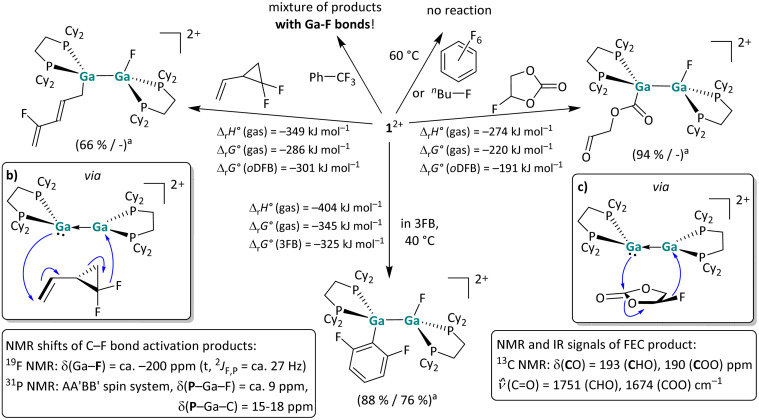
(a) Overview of the reactivity of 1^2+^ towards hexafluorobenzene, 1-fluorobutane, trifluorotoluene, 1,1-difluoro-2-vinylcyclopropane and fluoroethylenecarbonate (FEC) in *o*DFB and towards 3FB in the neat solvent (^*a*^ NMR yield/isolated yield). The [*pf*]^−^ anion is omitted for clarity. Unless otherwise stated, the reactions were carried out at rt. It should be noted that the C–F bond activation with 1,1-difluoro-2-vinylcyclopropane is somewhat unselective and that the structure of the main product is shown. The suggested electron flow in the adducts of 1^2+^ and 1,1-difluoro-2-vinylcyclopropane (b), as well as of 1^2+^ and FEC (c) accounts for the formation of the respective products.

In order to assess the bond activation potential of 1^2+^ towards single bonds, it was reacted with selected alcohols, amines, (un)functionalized nitriles, as well as C–F containing substrates. The respective reaction products are summarized in Schemes 4–6 and were observed *via* NMR spectroscopy and/or single crystal X-ray diffraction (scXRD). Note that the Ga–E (E = Ga, P, F, O, N, C, H) bond lengths (Section 3 in the SI) in the compounds presented are not discussed herein, since they are inconspicuous and in good agreement with other known and quantum chemically calculated bond lengths of their kind.

##### H–E (E = N, O) bond activation reactions

The digallene 1^2+^ reacts both with aliphatic and aromatic alcohols and amines, as summarized in Scheme 4. To the best of our knowledge, the H–O bond activation reactions lead to the first examples of group 13 dimers with the structural motif H–E–E–O^alkoxy/aryloxy^ (E = group 13 metal). Interestingly, the simple reduction of the polar amine/alcohol by the subvalent digallene and with evolution of dihydrogen according to R_*n*_E–H + Ga^I^ → R_*n*_E–Ga^III^ + 0.5 H_2_ (E = N, O), was never observed. By contrast, an oxidative addition reaction of the digallene into the H–O bond of ethanol and phenol takes place already at rt. The insertion of 1^2+^ into the H–N bond also occurs at rt for *n*-butylamine and aniline, but the addition of diphenylamine required additional heating of the reaction mixture to 40 °C for 16 hours. Cleavage reactions of O–H bonds have previously been reported with neutral, monomeric NHC analogues of Al(i)^50^ and Ga(i).^36^ However, the addition of a H–O bond across a ditrielene double bond is, to the best of our knowledge, unprecedented. Similarly, the activation of both primary and secondary amines has been reported *via* neutral ditetrylenes,^14,51^ and the activation of ammonia has been demonstrated with anionic Al(i) compounds and a neutral digallene.^19,52^ Yet, the activation of H–N bonds with ditrielenes is rather rare.^17,19^

##### Characterization

^1^H NMR and IR spectroscopy are useful tools to monitor the E–H bond activation: the H–Ga proton resonates at *ca.* 4.6 ppm and exhibits a triplet splitting pattern with ^2^*J*_H,P_ ≈ 20 Hz, while the H–Ga bond produces a broad band at *ca.* 1850 cm^−1^ in vibrational spectra. The scXRD measurements reveal an anti-periplanar geometry of the products, with H–Ga–Ga–E torsion angles ranging from 114° to *ca.* 175°. It can be assumed that the large range of torsion angles can be attributed to different dispersion interactions between the Cy rings and the respective organic substituents of the substrate. Furthermore, the trigonal planar coordination of the N atom in the activation products of H_2_NPh and HNPh_2_ is noteworthy and agrees with a formal sp^2^ hybridization of the N atoms in the products.

All reactions in Scheme 4 are exothermic and exergonic, with O–H insertions including the more electronegative atom being more favored than N–H activations. It should be pointed out that the HNPh_2_ and H_2_N*n*Bu activations are favored in solution only by −44 to –48 kJ mol^−1^, which might hint at some reversibility.

##### Reactions with (un)functionalized nitriles

Next, the reactivity of 1^2+^ towards a C≡N triple bond was investigated by reactions with acetonitrile, fluoroacetonitrile and benzonitrile (Scheme 5). Of special interest was whether one would observe simple coordination compounds with the nitrile donor, like in the [Ga(NCMe)_2_]^+^ complex salt recently reported,^53^ or whether one would observe a cycloaddition or even another bond activation reaction. The NMR spectra indicate that benzonitrile and 1^2+^ form a [2 + 2] cycloaddition product at rt in a slightly exergonic reaction, which decays to unknown decomposition products within 24 hours, while no reaction was observed between 1^2+^ and acetonitrile at rt. Interestingly, after increasing the reaction temperature to 60 °C the C–H insertion product rather than the coordination or [2 + 2] cycloaddition product with the C≡N triple bond was observed. DFT calculations suggest that the formation of a [2 + 2] cycloaddition product is thermodynamically unfavored (Δ_r_*G*° (*o*DFB) = +15 kJ mol^−1^), as is the simple formation of a coordination compound (Δ_r_*G*° (*o*DFB) = +14 kJ mol^−1^; see Fig. 4).

The observed activation of a C–H bond, representing the initial step of C–H functionalization reactions for the introduction of functional groups to abundant, simple starting materials, is even more challenging and has long been the domain of transition metal chemistry.^54^ Considerable progress in the field of subvalent main group element chemistry revealed that certain subvalent main group complexes are also able to cleave C–H bonds, *e.g.* neutral or anionic Al(i) compounds.^25,50,55^ However, the reaction with MeCN described herein is, to the best of our knowledge, the first example of an oxidative addition of Ga^I^ into a C–H bond of a neutral substrate. Such a bond activation was only described between an anionic Ga^I^-NHC analogue and an imidazolium cation.^56^

Intriguingly, the reaction with monofluoro-acetonitrile does not initially yield the analogous C–H or C–F bond activation product, but instead the [2 + 2] cycloaddition product formed in a very fast reaction at rt (Scheme 5), according to NMR spectroscopy: the product has two pairs of chemically non-equivalent P atoms, the CH_2_F group is still intact, and the ^13^C signal of the adjacent carbon atom is significantly low-field shifted. Notably, the ^13^C shift is comparable to the shift of the formally *sp*^2^-hybridized carbon atom in the [2 + 2] cycloaddition product of 1^2+^ with cyclooctyne (*ca.* 215 ppm *vs.* 180 ppm).^29^ Unfortunately, crystallization of the [2 + 2] cycloaddition product with H_2_FC–CN did not yield suitable crystals for scXRD. However, NMR spectra of the dissolved crystalline precipitate revealed that the product reacts further and yields the C–F activation product (Scheme 5).‡‡Note that the CN stretching mode *ν̃*_C≡N_ in the C–H activation product of H_3_CCN and in the C–F activation product of FH_2_CCN is red-shifted (*ca.* 2222 cm^−1^, see Scheme 5) compared to free H_3_CCN (2253 and 2293 cm^−1^).^67^ For both insertion products, the vibrational Stark effect, *i.e.* an electric field effect of the nitrile substituents, should account for the weakening of the CN triple bond.^68^ Concomitantly, we found that the [2 + 2] cycloaddition product also slowly decomposes in solution at rt, forming a range of unidentified compounds, including the simple C–F activation product. It can be concluded that, with H_2_FC–CN, cycloaddition and C–F bond activation are competing reaction pathways. DFT calculations reveal that the C–F bond cleavage is remarkably exergonic (Scheme 5), leading to the conclusion that the cycloadduct is the kinetic product, while the C–F bond insertion product can be considered the thermodynamic product. It should be noted that the [2 + 2] cycloaddition products of PhCN and H_2_FCN with 1^2+^ are considerably less stable than the cycloaddition products of 1^2+^ with alkenes and alkynes, which is reflected by the less exergonic or even endergonic formation of the nitrile cycloadducts (*cf.* the values in Schemes 2, 3, 5 and ref. 29). Nevertheless, the observed C–F bond activation with CH_2_FCN prompted us to explore the reactivity of 1^2+^ towards C–F bonds in nitrile-free substrates.

##### C–F bond activation reactions

Fluorinated organic compounds are interesting and challenging compounds for bond activation reactions due to the inherent strength of C–F bonds.^57^ Recently, C–F bond cleavage in fluorinated benzenes with a neutral, dinuclear Ga^I^ compound as well as neutral dialumenes was reported.^18,22^ As summarized in Scheme 6, the digallene 1^2+^ was reacted with several fluorinated substrates. No reaction was observed with hexafluorobenzene and 1-fluorobutane, whereas the reaction with trifluorotoluene yielded a mixture of Ga–F-containing products. Gratifyingly, the somewhat surprising molecular structures of C–F bond activation products with 1,1-difluoro-2-vinylcyclopropane and fluoroethylenecarbonate (FEC) could be determined unambiguously *via* NMR spectroscopy and are both shown in Scheme 6. In addition, 1^2+^ readily and selectively activates the central C–F bond in 1,2,3-trifluorobenzene (3FB) under mild conditions at room temperature. For all the substrates investigated herein, we found that characteristic triplets (^2^*J*_F,P_ ≈ 27 Hz) in the ^19^F NMR spectra at *ca.* −200 ppm serve as an indicator of a successful fluoride abstraction. The respective ^31^P{^1^H} spectra are characterized by a higher order AA′BB′ spin system with chemical shifts at 15–18 ppm and at *ca.* 9 ppm for the P–Ga^II^–C and P–Ga^II^–F phosphorus atoms respectively.

As expected, the ^13^C NMR shifts of the Ga-bound carbon atom are less characteristic and depend on the nature of the substituent: for the C–F bond activation products reported in this work, they range from 20 to 114 and 190 ppm for the alkyl, aryl and acyl substituents, respectively (Sections 4.12–4.15 in the SI). The results presented in Scheme 6 strongly suggest that the substrate coordination towards the dicationic digallene 1^2+^ is a prerequisite for C–F bond cleavage. While hexafluorobenzene and 1-fluorobutane are very poor ligands, trifluorotoluene, 1,1-difluoro-2-vinylcyclopropane and FEC can coordinate to metal cations through their comparatively electron rich aromatic π-system, double bond and carbonate group, respectively. It is reasonable to assume that the coordination of the substrate is followed by formal F^−^ transfer, which is only enabled by the close Ga–F proximity. In this context, it is important to mention that the Ga–F bond is the strongest gallium–element single bond (*ca.* 580 kJ mol^−1^),^30,32^ accounting for the pronounced thermodynamic driving force of the F^−^ transfer reactions (see the calculated thermodynamic values in Scheme 6). Consequently, the C–F bond cleavage with trifluorotoluene, leaving a fluorine atom on the first gallium atom and a –CF_2_Ph moiety on the second gallium atom, is also calculated to be highly exothermic and exergonic (Δ_r_*H*° (gas) = −232 kJ mol^−1^, Δ_r_*G*° (gas) = −159 kJ mol^−1^, Δ_r_*G*° (*o*DFB) = −150 kJ mol^−1^). The initial product probably undergoes side reactions due to the remaining and reactive C^benzyl^–F bonds, accounting for the observed mixture of products. A clean C–F bond cleavage without accompanying rearrangement reactions was observed for the reaction of 1^2+^ and 3FB after four days at 40 °C (Scheme 6, bottom). The molecular scXRD structure of the product is well reproduced by DFT calculations and is in accordance with NMR spectroscopic results as shown below in Fig. 5, in Part II. Note that [Ga(PhF)_2_][*pf*] without a donor ligand is compatible with 3FB.^53^ In line with previous transition metal mediated reactions, 1^2+^ selectively cleaves the central C^aromatic^–F bond, confirming that F substituents at *ortho* positions activate a C^aromatic^–F bond.^58–61^ One of the remaining fluorine atoms at the *ortho*-position is oriented almost vertically above the Ga–Ga bond (Fig. 5b and c), thereby probably stabilizing the formal gallium cations with its free lone pair orbitals (intramolecular C^*ortho*^F–Ga distance: *ca.* 320 pm).

### Part II – understanding the reactivity of the dicationic digallene

The reactions with substrates that bear lone pair orbitals at the reactive bond (*e.g.*, N–H and O–H, but also NC–CH_3_ or F–C), suggested the chance that, prior to the reaction, a substrate/dimer adduct might be relevant. An asymmetric conformer with one more accessible planarized Ga atom may serve as an “anchor” for the substrate, while the other more pyramidal Ga atom could cooperatively perform the subsequent oxidative addition. These hypotheses prompted us to first analyze the frontier MOs (FMOs) of 1^2+^ and scan for other possible conformers, potentially relevant for adduct formation.

#### Frontier MOs of [{Ga(dcpe)}_2_]^2+^ and the accessibility of an asymmetric conformer

The FMOs of the strictly dimeric^29^ dication 1^2+^ are compared to the FMOs of the known monomeric gallene [Ga(dtbpf)]^+^ (1,1′-bis(ditertbutyl-phosphino)ferrocene)^40^ in Fig. 1. Apparently, the availability of four frontier molecular orbitals, as well as the considerably smaller HOMO–LUMO gap of 1.56 eV as in the gallene with 2.32 eV endows digallene 1^2+^ with a significantly higher reactivity compared to its monomeric counterparts. This may enable bond activation under milder conditions and also the activation of stronger σ-bonds compared to monomeric low valent gallium species. Note that the HOMO and LUMO have large lobes residing on the Ga atoms, making them a prime target for interaction with substrates.

**Scheme 7:**
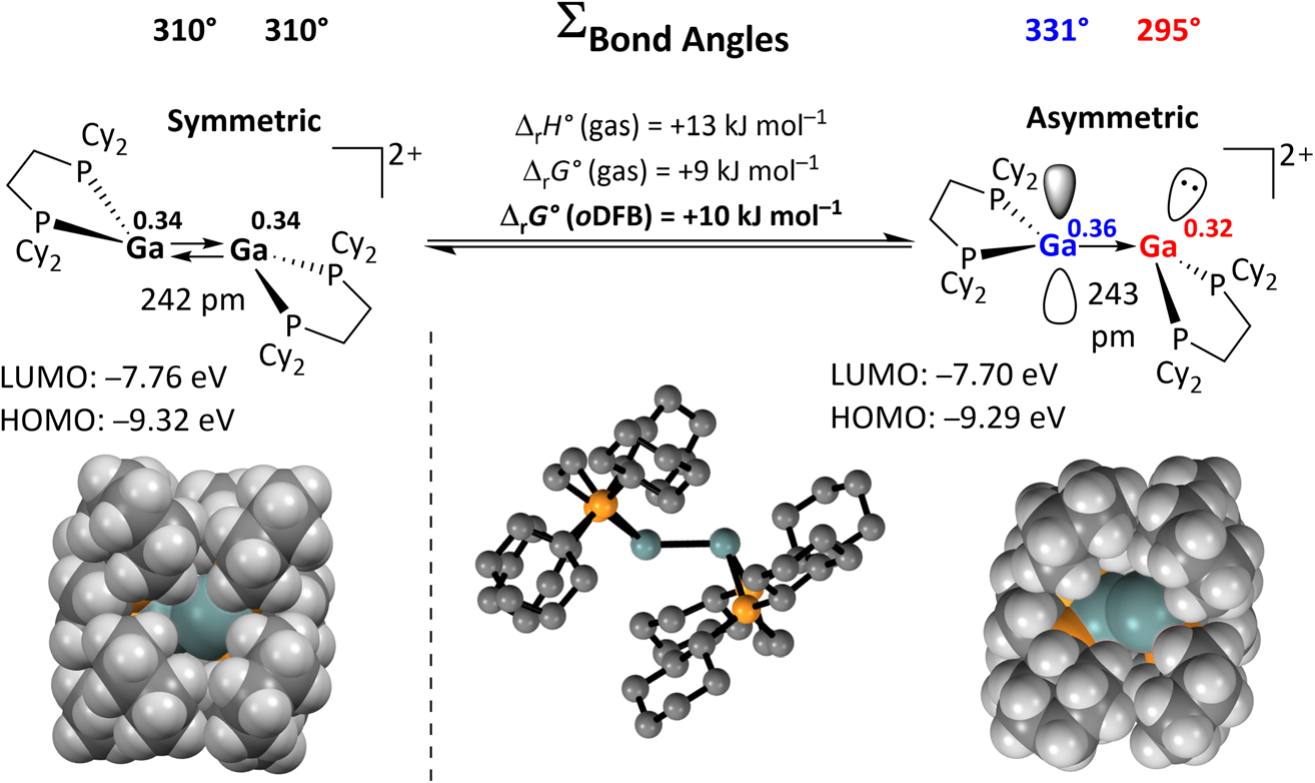
Calculated reaction enthalpies and Gibbs free energies in the gas phase and in *o*DFB for the conversion of the symmetric conformation of 1^2+^ into the asymmetric conformation (DSD-PBEP86-D3(BJ)/def2-QZVPP(C) single point calculation on RI-BP86(D3BJ)/def2-TZVPP optimized structures; *ε*_r_ = 13.38).^32^ The space-filling model and selected calculated parameters for both conformers are also shown, including QTAIM charges for the Ga atoms (superscripts).

Apart from the accessible FMOs of 1^2+^, the available conformations of the digallene in solution need to be considered in more detail. Interestingly, the asymmetric conformer shown in Scheme 7 is less stable than the symmetric, crystallographically found conformer only by 10 kJ mol^−1^. With Δ_r_*G*° = –*RT* ln *K*, this implies that *K* = 10^−1.7^ at 25 °C, or that out of 50 digallenes, one is in the asymmetric conformation. Note that such a briefly mentioned asymmetric conformation for [{Ga(dipf)}_2_]^2+^ is considerably higher in energy at +45 kJ mol^−1^ and, hence, almost irrelevant for reactions.^40^ The conformational change is well reflected in the bond angles around the Ga atoms: while the sum of the bond angles increases from 310° in the symmetric conformer to 331° for the planarized Ga atom, it decreases to 295° for the pyramidalized Ga atom. The QTAIM (Quantum Theory of Atoms in Molecules) charges indicate the planarized Ga atom to have a slightly higher positive charge than in the symmetric conformer (0.36*e vs.* 0.34*e*), while the pyramidal geometry of the second Ga atom indicates the presence of an electron lone pair and thus a more negative partial charge (0.32*e*). However, the HOMO/LUMO positions and the Ga–Ga bond length are barely affected by this conformational change.

The space-filling models of the optimized structures for both conformers nicely illustrate that the Ga atoms in the asymmetric conformer are more accessible (Scheme 7), probably indicating that the conformational change may facilitate the reaction with a given substrate for steric reasons. Hence, although the symmetric global minimum conformer was chosen as a starting point for the calculated thermodynamic values throughout this work, it should be kept in mind that the asymmetric conformer is available and may also serve as the reactive species.

#### Mechanistic aspects of the interaction with CC multiple bonds

**Scheme 8:**
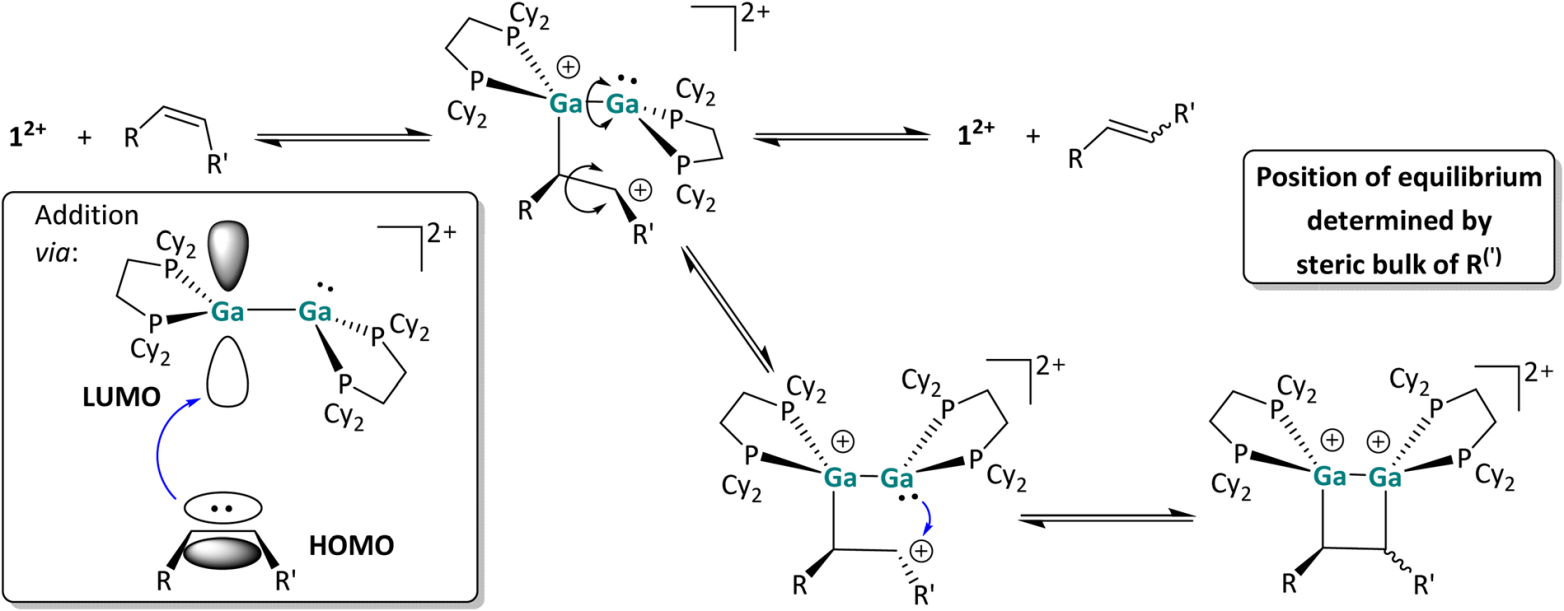
Postulated reaction mechanism of the [2 + 2] cycloaddition reaction of 1^2+^ with C=C double bonds. For the sake of clarity, the [*pf*]^−^ anion is omitted and, for chiral species, only one enantiomer is shown. The most relevant postulated orbital interaction between 1^2+^ and an olefinic substrate is also shown schematically.

The FMOs shown in Fig. 1 suggest a hypothetical cycloaddition mechanism, which is in accordance with all considerations and is described in Scheme 8. The reaction is presumably initiated *via* a nucleophilic attack of the π-MO of the olefin towards the LUMO of 1^2+^, which is more available at one Ga atom in the asymmetric conformer, and leads to the formation of a C–Ga bond as well as the conversion of the Ga⇄Ga and C=C double bonds to Ga–Ga and C–C single bonds.

In agreement with the low-lying frontier orbitals, the digallene most likely acts as an electrophile in this reaction step, accepting electron density from the olefin and forming a carbocation following Markovnikov's rule. In the resulting carbocation intermediate, rapid rotation around the former C=C double bond is possible, accounting for the formation of different stereoisomers in the reaction course. This short-lived carbocation intermediate could not be identified *via in situ* low temperature NMR spectroscopy; instead, as already described, the *cis*/*trans* isomerization was observed immediately in reactions performed at temperatures as low as −40 °C (around the melting point of *o*DFB), indicating a very low activation barrier for the initial addition of the olefinic species. The carbocationic intermediate next can follow two reaction pathways: it can either undergo ring closure, yielding the [2 + 2] cycloaddition product, or regenerate the dicationic digallene and the (isomerized) olefin *via* C–Ga bond cleavage. Ring-closure requires rotation around the Ga–Ga bond of the first olefin adduct. Apparently, all species are in equilibrium with one another, and the steric demand of the substrate as well as the capability of the residue to stabilize the carbocation determine whether the [2 + 2] cycloaddition reaction is complete (1-hexene), incomplete (styrene) or does not take place at all (*cis* 3-hexene and *cis* methylstyrene).

#### Mechanistic aspects of the H–E (E = N, O) bond activation reactions

**Fig. 2:**
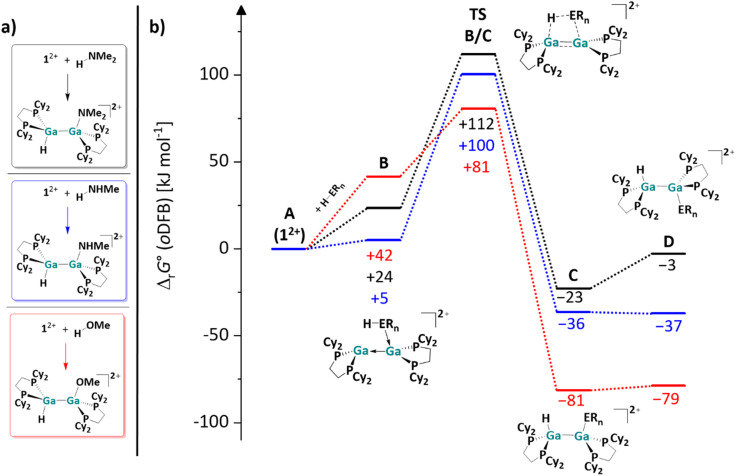
(a) Computationally analyzed H–O and H–N bond activation reactions of 1^2+^ with the model substrates HNMe_2_, H_2_NMe and HOMe. (b) Energy landscape and intermediates for a hypothetical mechanism of the H–E (E = O, N) bond activation reaction of HOMe (red line), H_2_NMe (blue line) and HNMe_2_ (black line) with 1^2+^ (DSD-PBEP86-D3(BJ)/def2-QZVPP(C) single point calculation on RI-BP86(D3BJ)/def2-TZVPP optimized structures; all values are given in kJ mol^−1^). The Gibbs free energies were calculated for an *o*DFB solution (*ε*_r_ = 13.38).^32^

While the primary amines *n*-butylamine and aniline reacted at room temperature within *ca.* 30 minutes, the increased steric bulk of diphenylamine required additional heating at 40 °C for several hours. Since the substrates with an aliphatic sidechain directly form the dimeric addition products, it is highly likely that the E–H bond activation *via*1^2+^ occurs directly from the cooperative donor–acceptor conformation of 1^2+^ shown in Scheme 7. The DFT-calculated energy landscapes for the one-step H–E (E = N, O) bond activation of the model substrates MeOH, MeNH_2_ and Me_2_NH with 1^2+^ are shown in Fig. 2. In the digallene/substrate adduct B, the digallene adopts the asymmetric donor–acceptor conformation of 1^2+^. Following the formation of B, the hydrogen atom is transferred to the second Ga atom in a concerted reaction. Here, the activation barrier is considerably higher for the amines than for HOMe (*ca.* 90 kJ mol^−1^*vs.* 40 kJ mol^−1^).

With the small NHMe, NMe_2_ and OMe substituents, the initially formed *syn*-periplanar addition products C are similarly stable or even more stable than the *anti*-periplanar addition products D. The calculated values are in agreement with the observed reaction rates, as H_2_NBu and H_2_NPh react within 30 to 60 minutes, while HOEt and HOPh, react within seconds at rt. However, it should be kept in mind that the exact activation barriers are probably highly influenced by steric effects and dispersion interactions. In line with this, the reaction rate with HNPh_2_ is considerably slower.

**Fig. 3:**
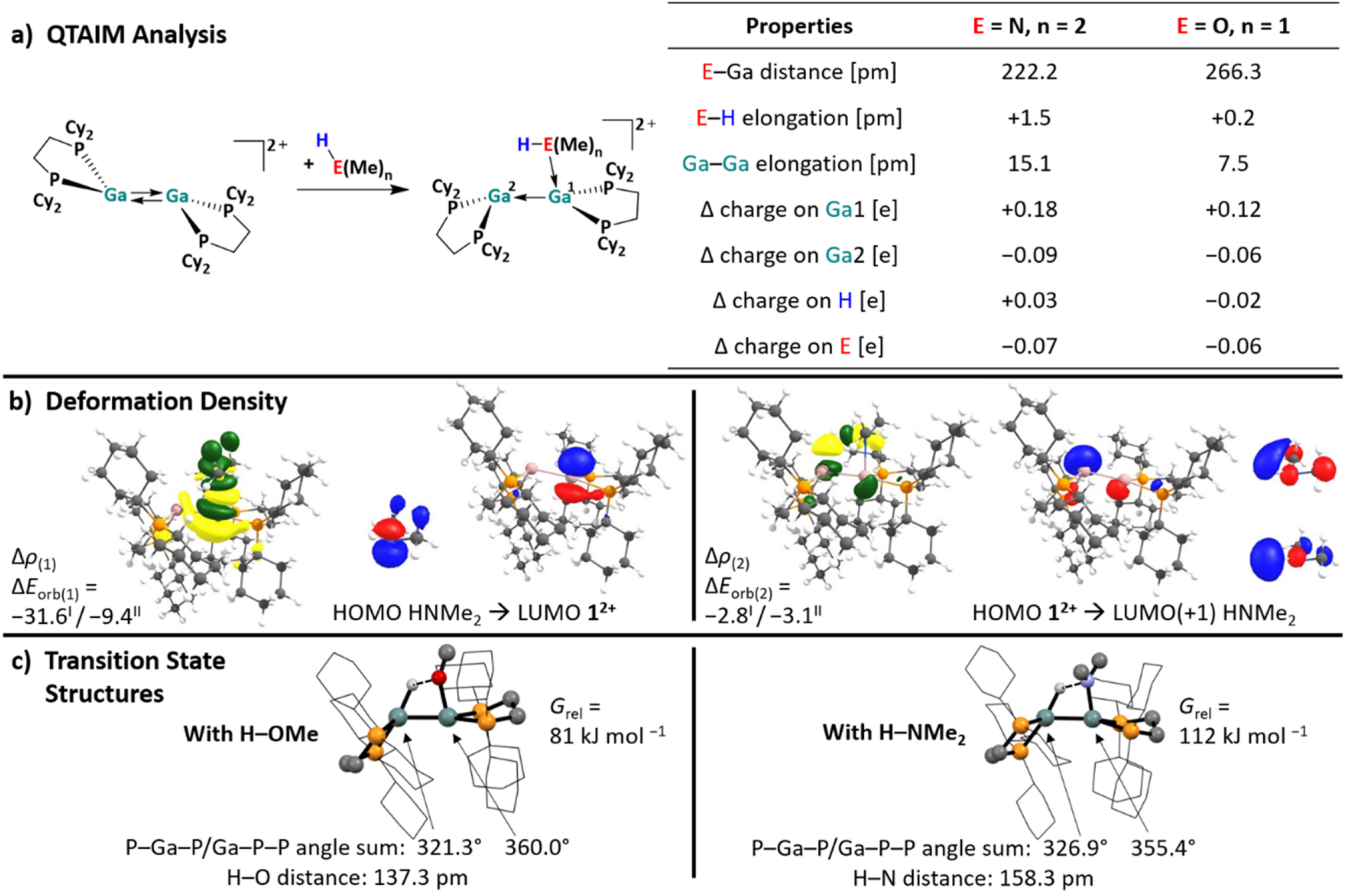
(a) Calculated structures and comparison of selected structural/bond parameters from QTAIM analysis in the Lewis acid–base complexes of 1^2+^ and the model substrates HOMe/HNMe_2_. *Δ* = difference in the QTAIM partial charge after coordination of the Lewis base. (b) EDA-NOCV results for the two complexes using 1^2+^ and HOMe or HNMe_2_ as interacting fragments. The shapes of the deformation densities Δ*ρ*_(1)–(2)_ as obtained by the EDA-NOCV analysis, correspond to Δ*E*_orb(1)–(2)_ for the interaction between 1^2+^ and HNMe_2_. The fragment orbitals of 1^2+^ and HNMe_2_ are shown exemplarily (isosurface values: 0.0008 au for Δ*ρ*_(1)–(2)_, 0.06 au for the molecular orbitals of 1^2+^, 0.08 au for the HOMO of HNMe_2_ and 0.05 au for the LUMO(+1) of HNMe_2_). All other interactions are shown in Section 6.2 of the SI. The direction of the charge flow of the deformation densities is green → yellow. All calculations were performed at the RI-BP86(D3BJ)/def2-TZVPP level of theory; the orbital interaction energies Δ*E*_orb(1)–(2)_ (for HNMe^I^_2_ and HOMe^II^) are given in kcal mol^−1^ instead of kJ mol^−1^ for better comparability with other EDA-NOCV analyses in the literature. (c) Calculated transition state structures of the H–O and H–N bond breakage for HOMe and HNMe_2_, including selected structural parameters. The value *G*_rel_ refers to the Gibbs free energies of the transition state structures relative to the free substrates and 1^2+^.

In order to better understand the fundamental electronic structure of the reactive 1^2+^/substrate conformation, it was further analyzed with QTAIM and EDA-NOCV calculations (energy decomposition analysis with the combination of natural orbitals for chemical valence). The small molecules MeOH and Me_2_NH were chosen as model substrates. The results, summarized in Fig. 3, reveal that the coordination of the substrates leads to significant Ga–Ga bond elongation (15.1 pm and 7.5 pm for the HNMe_2_ and HOMe complexes, respectively), H–E bond weakening (elongation by 1.5 and 0.2 pm) and considerably more positive charge residing on the coordinating Ga^I^ atom, compared to the free, symmetric conformer of 1^2+^ (*Δ* charge = 0.18 and 0.12 e). Concurrently, the second Ga atom (*Δ* charge = −0.09 and −0.06) and the donor atom E (*Δ* charge = −0.07 and −0.06) become more negatively polarized than in the free species. Note that the charge redistribution calculated for the conformer change of the digallene in the absence of a ligand (Scheme 7) is greatly intensified upon substrate coordination. The effects are more pronounced for the Me_2_NH adduct than with HOMe, agreeing with the generally stronger donor character of amines compared to the analogous alcohols.

In accordance with the described charge redistribution in Fig. 3, the electrostatic interactions between 1^2+^ and HOMe/HNMe_2_ make up the strongest contribution to the total attractive interaction, as confirmed by the EDA-NOCV analysis (*ca.* 50%, Section 6.1 in the SI). By contrast, the dispersion interactions between HOMe/HNMe_2_ and 1^2+^ are considerably weaker, due to the small organic substituents in the chosen model substrates.

As expected, the electron donation from the HOMO(−1) of the ligand to the LUMO of the asymmetric digallene is the most relevant interaction (Fig. 3b). Interestingly, the second most important interaction is characterized by a reverse electron flow: the HOMO of the asymmetric digallene, which is mainly located on the non-coordinated Ga atom, donates electron density to the LUMO(+1) of the substrates, *i.e.*, the σ*(H–E) orbitals, thus weakening the H–E bonds. Consequently, the cationic digallene primarily acts as a Lewis acid towards substrates with H–E bonds; however, its effect as an electron donor is also relevant. In summary, and as already sketched in Scheme 1b, the EDA-NOCV analysis underlines the importance of both unoccupied and occupied orbitals of 1^2+^ in interactions with σ-bonds and thus confirms the cooperative bond activation mode of the asymmetric conformer of 1^2+^. Notably, also the planarized/pyramidalized asymmetric geometry of the two Ga atoms is retained in the structure of the H–E bond breakage transition states shown in Fig. 3c. This results from the described electron flow.

**Scheme 9:**
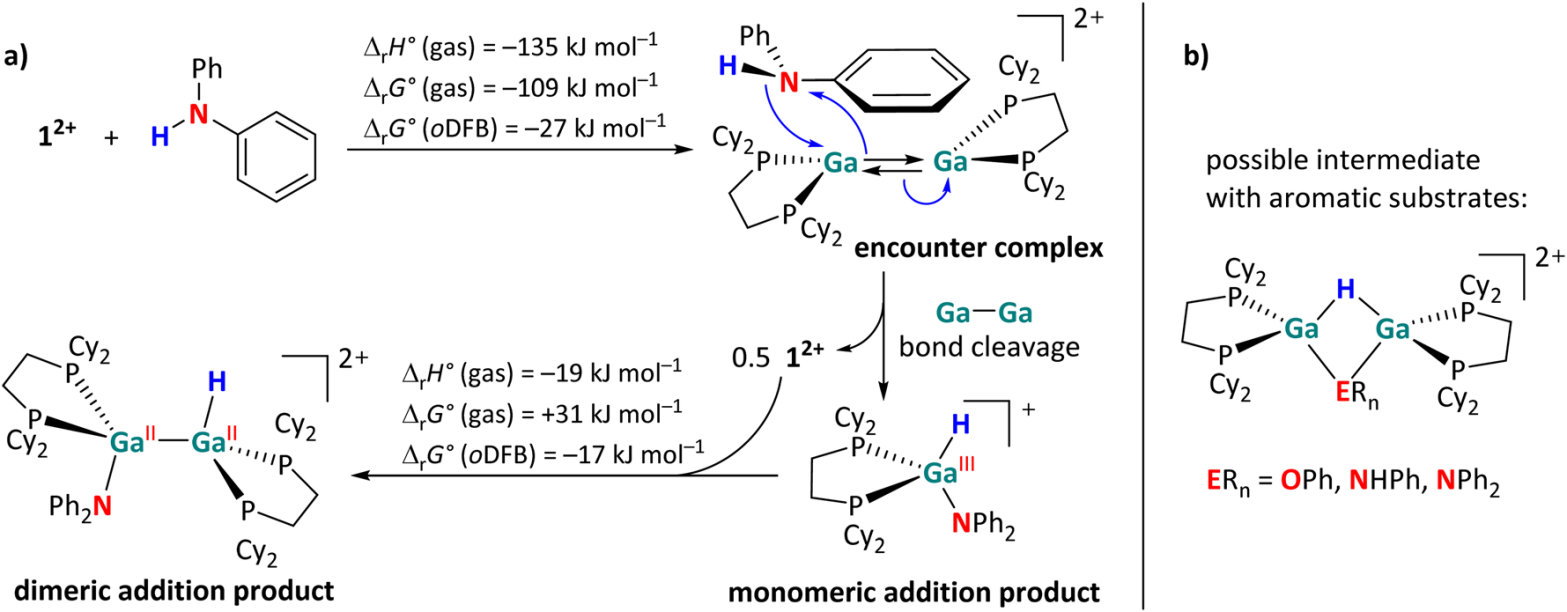
(a) Sketch of the proposed, main activation mechanism of HNPh_2_, with calculated reaction enthalpies and Gibbs free energies in the gas phase and in *o*DFB (DSD-PBEP86-D3(BJ)/def2-QZVPP(C) single point calculation on RI-BP86(D3BJ)/def2-TZVPP optimized structures). (b) Possible structure of NMR-spectroscopically observed intermediates during the E–H (E = O, N) bond activation reactions with the aromatic substrates HOPh, H_2_NPh and HNPh_2_. Taking into account that the ^31^P NMR and the ^1^H NMR shifts of the P–Ga–H units in these intermediates are very similar, it cannot be ruled out that the ER_*n*_ groups act as bridging ligands with their aromatic π-systems or that the ER_*n*_ groups are only weakly associated.

However, the activation of aromatic substrates may be more complicated than described in Fig. 2. Unlike with the aliphatic substrates, a monomeric, H–Ga containing intermediate was observed *via* NMR spectroscopy during the reactions of 1^2+^ with diphenylamine. The monomeric species is identified on the basis of a singlet in the ^31^P{^1^H} NMR spectrum and its large ^2^*J*_H,P_ coupling constant of 50 Hz, which is rather indicative of a Ga^III^–H species.^29,39^ By contrast, the ^2^*J*_H,P_ coupling constants in the dimeric Ga^II^ addition products are significantly smaller (*ca.* 20 Hz, see Scheme 4). Since the presence of the electron-rich aromatic π-system probably plays a crucial role, we propose the initial formation of an encounter complex, as visualized in Scheme 9a. The affinity of 1^2+^ towards aromatic π-systems is further discussed in the context of the reaction with 3FB (intermediate B in Fig. 5).

Due to the close proximity of the N–H bond to only one Ga atom in the encounter complex, the –NPh_2_ and –H fragments are transferred to the same Ga atom, while the other Ga^+^ cation interacts with the π-system of the aromatic substituent and reforms 1^2+^ after dissociation (Scheme 9a). The monomeric addition product further reacts with 1^2+^, ultimately leading to the dimeric addition products shown in Scheme 4. Interestingly, the formation of a monomeric intermediate was not observed during the H–P activation of HPPh_2_.^29^ This is most likely due to the fact that phosphines are (towards Ga^+^) stronger σ-donors than amines, making the η^6^ coordination mode very uncommon for arylphosphines.^62^

In addition, another intermediate can be identified during the reactions of 1^2+^ with HOPh, H_2_NPh and, to a smaller extent, with HNPh_2_. The singlets in the ^31^P{^1^H} NMR spectrum, display a *J*_H,P_ coupling constant of *ca.* 27 Hz. Since these species are directly converted to the final addition product, when no more 1^2+^ is present in solution, we suggest these intermediates to have a symmetric, but dimeric structure as proposed in Scheme 9b. The formation mechanism of these bridged species is not entirely clear. They could form after a H atom migration of the initially formed *syn*-periplanar product, which would allow the formation of the *anti*-periplanar product without the rotation around the sterically congested Ga–Ga bond.

#### Mechanistic aspects of the C–H insertion with acetonitrile

**Fig. 4:**
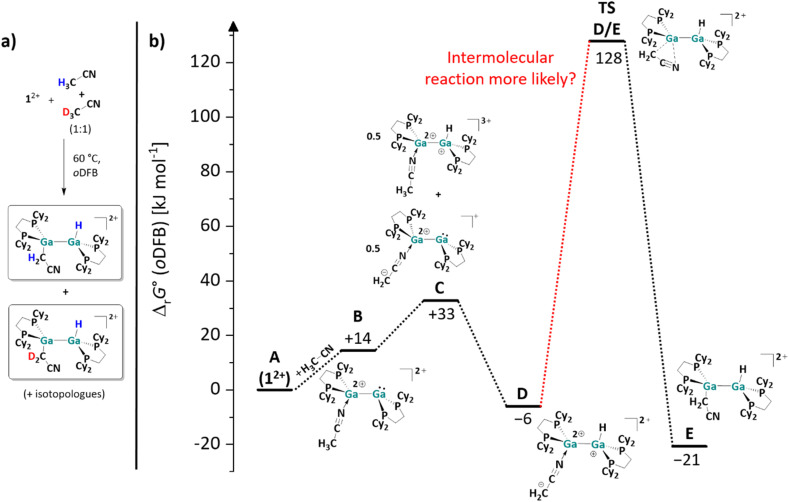
(a) Products formed when reacting 1^2+^ with a 1 : 1 mixture of acetonitrile and acetonitrile-*d*_3_. (b) Energy landscape and intermediates for a hypothetical mechanism of the C–H bond activation reaction of acetonitrile with 1^2+^ (DSD-PBEP86-D3(BJ)/def2-QZVPP(C) single point calculation on RI-BP86(D3BJ)/def2-TZVPP optimized structures; all values are given in kJ mol^−1^). The Gibbs free energies were calculated for an *o*DFB solution (*ε*_r_ = 13.38).^32^

To gain further insights into the reaction mechanism of the C–H activation of acetonitrile, the reaction was repeated with a 1 : 1 mixture of acetonitrile and acetonitrile-*d*_3_. As revealed by ^1^H, ^13^C HMBC NMR spectroscopy, two different products with a Ga–H bond and an adjacent Ga–C bond were formed. Since the other ^1^H, ^13^C and ^31^P chemical shifts are very similar or even virtually identical in both compounds, this can only be explained by the formation of mixed species, *i.e.*, products with H–Ga–Ga–CD_2_ moieties (Fig. 4a).

Upon heating a 1 : 1 mixture of separately synthesized [H{Ga(dcpe)}_2_(CH_2_CN)]^2+^ and [D{Ga(dcpe)}_2_(CD_2_CN)]^2+^ for 4 days at 60 °C, no formation of [H{Ga(dcpe)}_2_(CD_2_CN)]^2+^ was observed. Thus, for the reaction of H_3_C–CN and D_3_C–CN with [{Ga(dcpe)}_2_][*pf*]_2_, scrambling of Ga–H and Ga–D can be ruled out. This leads to the conclusion that the –H and –CH_2_CN fragments in the C–H activation product of H_3_CCN (Scheme 5) are not transferred from the same acetonitrile molecule, *i.e.*, the activation is an intermolecular reaction. A calculated energy landscape of a hypothetical intermolecular reaction mechanism is presented in Fig. 4b, as well as the structures of the corresponding intermediates. The reaction sequence is probably initiated by the coordination of the ligand MeCN to one of the two Ga atoms of the dicationic digallene. The formation of the adduct (B in Fig. 4b) and shift of electron density result in an increased Brønsted basicity of the second Ga atom as well as an increased Brønsted acidity of the methyl group. A subsequent slightly endergonic Brønsted acid–base reaction would yield a tricationic digallane and a zwitterionic, monocationic digallane (C). A second Brønsted acid–base reaction could then lead to isomer D of the observed product E, which is finally formed *via* a 1,3-rearrangement reaction. The activation barrier for this final reaction step is probably prohibitively high (*ca.* 135 kJ mol^−1^), even when taking into account that the reaction requires prolonged heating at 60 °C for 4 days. Thus, it is more likely that the [H_2_C–CN]^−^ fragment in D is transferred to another gallium dimer in an intermolecular reaction, rather than undergoing a rearrangement reaction. In line with this, no intermediate D is observed spectroscopically during the reaction of 1^2+^ and MeCN, which would be expected if the mechanism presented in Fig. 4b was operative, considering its relative inertness. In conclusion, the calculations underpin that both the C–H bond activation and the [H_2_C–CN]^−^ migration probably follow a bimolecular mechanism and that (de)protonated MeCN/1^2+^ complexes similar to C and D may be involved.

Overall, the different reactivity of 1^2+^ towards H_3_C–CN and H_2_F–CN was unexpected. However, the different routes can be rationalized with quantum chemical calculations: as shown in Scheme 5, the hypothetical [2 + 2] cycloaddition reaction of acetonitrile with 1^2+^ is endergonic in solution, whereas the C–H activation is exergonic, just like the [2 + 2] cycloaddition with the monofluorinated substrate (*cf.*Scheme 5). These results not only demonstrate that the dicationic digallene shows a versatile chemistry, but also that subtle changes in the substrate can favor different reaction pathways.

#### C–F bond activations

**Fig. 5:**
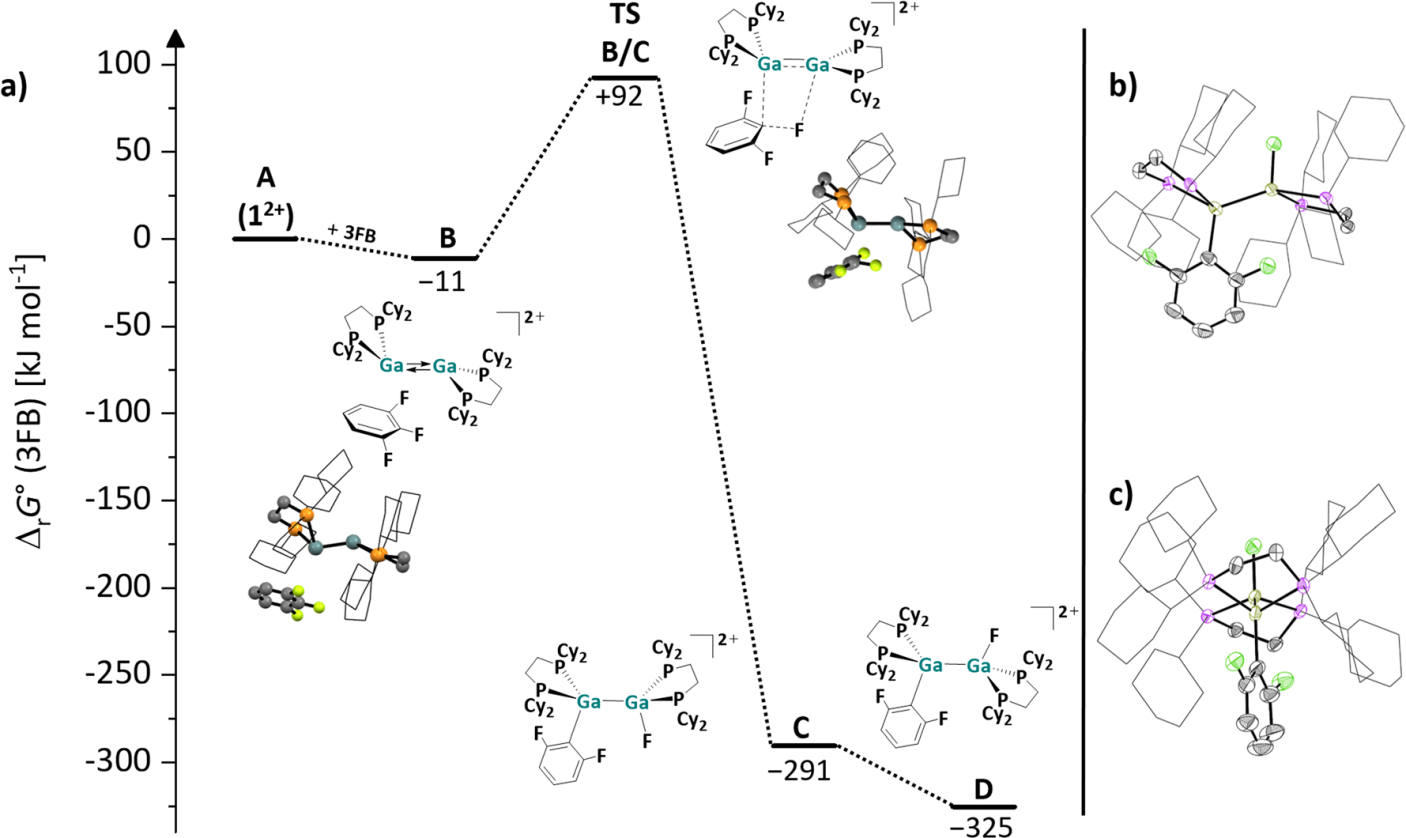
(a) Energy landscape and intermediates for a hypothetical mechanism of the C–F bond activation reaction of 3FB with 1^2+^ (DSD-PBEP86-D3(BJ)/def2-QZVPP(C) single point calculation on RI-BP86(D3BJ)/def2-TZVPP optimized structures); all values are given in kJ mol^−1^. The Gibbs free energies were calculated for an 3FB solution (*ε*_r_ = 22.1).^63^ (b + c) Molecular structure in the solid state of the cation in the reaction product of [1][*pf*]_2_ with 3FB. The two different perspectives (b) and (c) illustrate the *anti*-periplanar arrangement of the F atom and the C_6_F_2_H_3_ moiety in the product. Only one of the three crystallographically independent [F{Ga(dcpe)}_2_C_6_F_2_H_3_]^2+^ units is shown. Hydrogen atoms, counteranions and disordered ligand molecules are omitted for clarity; the cyclohexyl groups are depicted in wireframe for simplicity. Thermal ellipsoids are set at the 50% probability level.

The observed main C–F bond activation products of 1,1-difluoro-2-vinylcyclopropane and FEC are particularly interesting and revealing. A plausible formation path for the respective products necessitates pre-coordination, possibly from the asymmetric conformer and is sketched in the middle-left and middle-right of insets in Scheme 6. It is evident that the initial fluoride transfer to 1^2+^ has several consequences: it induces a positive charge on the substrate and a negative polarization of the second Ga atom. Thus, the F^−^ transfer is probably accompanied by a nucleophilic attack of the second Ga atom on the functional group, *i.e.*, the double bond and the carbonate moiety of 1,1-difluoro-2-vinylcyclopropane and FEC, respectively. For both reactions, no structures of zwitterionic intermediates, as above with the olefins, could be calculated with DFT methods, indicating that the bonds involved are broken and formed in a concerted reaction. By contrast, the reaction with the arene 3FB apparently again proceeds *via* an encounter complex; a calculated energy landscape for the insertion of 1^2+^ into the central C–F bond of 3FB is shown in Fig. 5. Notably and unexpectedly, the π-coordination of one solvent molecule 3FB to the dicationic digallene is slightly exergonic and results in the formation of encounter-complex B. It should be pointed out that the formation of complex B is more favorable than the formation of the donor–acceptor complex with HOMe and HNMe_2_ (Fig. 2), even though 3FB is relatively electron poor. This finding again underlines the affinity of the *trans*-bent Ga⇄Ga double bond towards π-systems and also suggests that the aromatic anilines and phenols primarily coordinate *via* their electron-rich π-system, as proposed in the reaction mechanism in Scheme 9.

Starting from the complex B, the F atom and the C_6_F_2_H_3_ moiety are transferred to the two Ga atoms of the digallene in a concerted fashion. The Ga–C and Ga–F bonds are formed and the C–F bond is broken in the transition state TSB/C, which is *ca.* 100 kJ mol^−1^ higher in energy than B. Subsequently, a thermodynamically favorable rotation around the Ga–Ga bond in the initially formed *syn*-periplanar product C diminishes the steric repulsion between the cyclohexyl groups and yields the *anti*-periplanar product D in the crystallized compound.

A comparison of the reactivity of 1^2+^ towards fluorinated benzenes reveals that the reactivity of the digallene is pronounced towards 3FB, while the reactions with both higher and lower fluorinated aromatics are considerably slower. Solutions of [1][*pf*]_2_ in *o*DFB are stable over several days to weeks at rt before the (very) slow formation of Ga–F bonds, which can be observed *via* NMR spectroscopy. By contrast, even at 60 °C, the digallene is virtually unreactive towards hexafluorobenzene (6FB) (Scheme 6), even though the C–F bonds in 6FB are easily cleaved with a bimetallic Mg(i) complex^64^ and a neutral alumene.^65,66^ It should be mentioned that 1^2+^ is similarly reactive towards 1,2,3,4-tetrafluorobenzene (4FB) as towards 3FB. However, the reaction is considerably less selective, as multiple C–F bond activation products are observed spectroscopically after 3 days at 40 °C (Section 4.16.2 in the SI).

Different and opposing effects may contribute to the observed reactivity trend: on the one hand, it is well known that C^aromatic^–F bonds are weakened by additional fluorine substituents, accounting for the fact that C–F bonds in higher fluorinated aromatics are usually easier to activate than in lower fluorinated aromatics.^58–60^ This was experimentally confirmed with main group element compounds, *e.g.* a neutral alumene^65,66^ and a neutral, dinuclear bisgallene.^22^ On the other hand, the coordinating ability decreases with a higher degree of fluorination. It may also be significant that the steric demand of the aromatic substrate increases with every F substituent and that the polarity of the fluorinated benzenes reaches a maximum with 3FB with a large dipole moment of 4.7 Debye,^63^ before decreasing with increasing degree of fluorination.^63^ Thus, the reaction outcomes with fluorinated arenes nicely illustrate that the reactivity of 1^2+^ towards fluorinated compounds is influenced by the strength and polarity of the C–F bond, as well as the coordinating ability of the substrate, which in turn is affected by electronic and steric effects. These partly opposing effects lead to the observed high reactivity and selective reaction of 1^2+^ towards 3FB, *i.e.*, a polar arene with a medium degree of fluorination. It is noteworthy that the reaction between a neutral alumene and 3FB yields both conceivable C–F activation products and requires prolonged heating at 80 °C for several days,^66^ which is in contrast to the selective, facile bond cleavage with 1^2+^ at 40 °C.

Overall, the results imply that C–F bond activation with dicationic digallenes is possible, if the substrate has an additional “donor” functionality or a reasonably electron rich π bond or aromatic π system. Interestingly, in the case of fluorinated aromatics, the dicationic digallene shows a different reactivity trend than neutral subvalent group 13 compounds, suggesting that the cationic congeners may complement the reactivity of the neutral agents.

## Conclusion

**Scheme 10:**
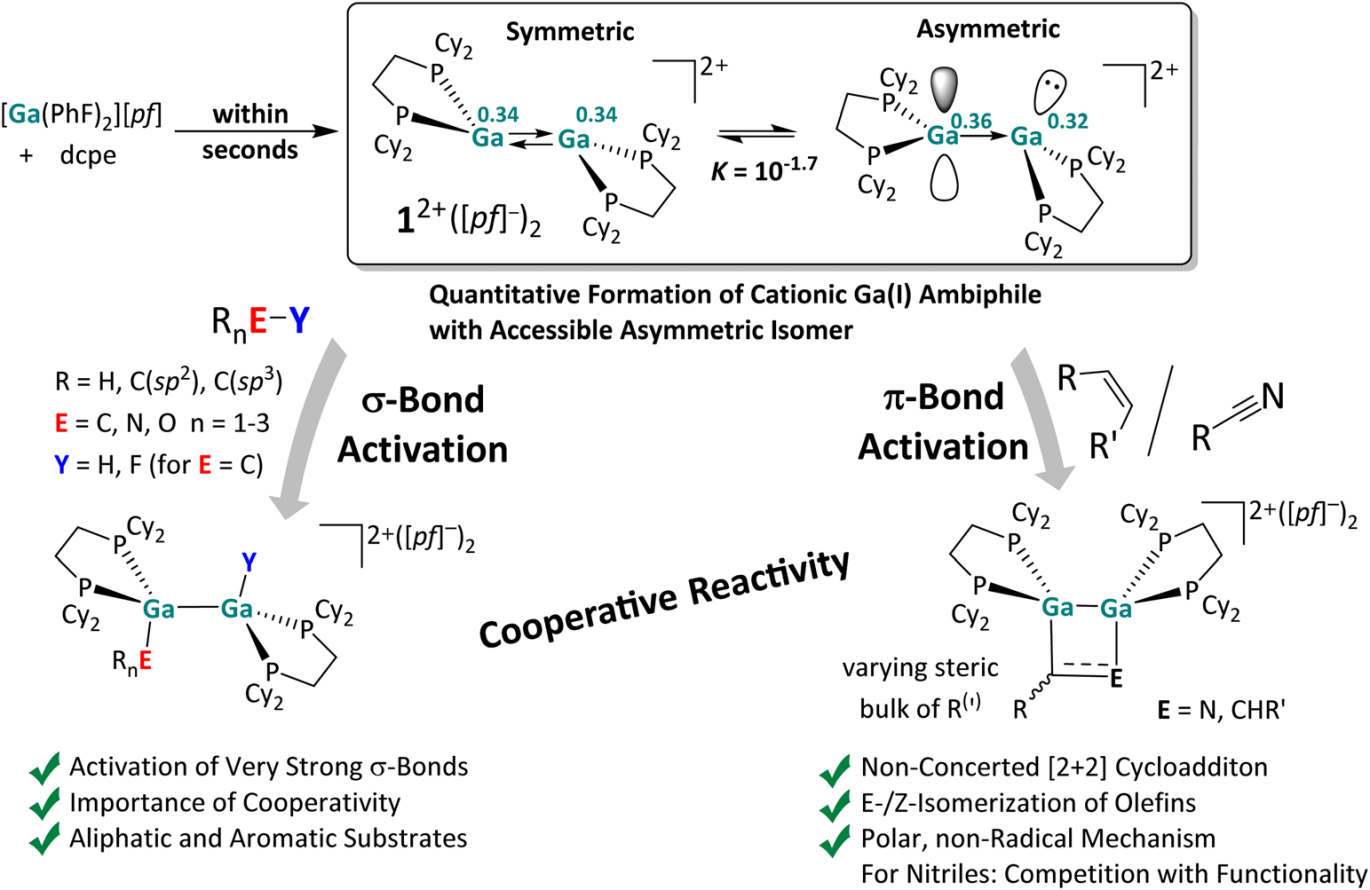
Results of this work. The *in situ* formed reactive digallene 1^2+^ accessible from [Ga(PhF)_2_][*pf*] and commercial dcpe,^29^ reacts cooperatively and with strong contributions from the accessible asymmetric conformer (QTAIM charges on Ga atoms are written in superscript) with olefins, nitriles and strong C–F, C–H, N–H, and O–H σ-bonds. The herein derived mechanistic insight will help to pick up selected activations and to transform them into parts of catalytic cycles, especially in those cases, where the energetics underlying the activations is only moderately exergonic or even on the edge (olefins, N–H, O–H, and CN-cycloaddition).

Herein, the reactivity of a dicationic digallene towards CC multiple bonds and very strong element–element E–Y single bonds was thoroughly investigated. The instantaneously formed, strictly dimeric [{Ga(dcpe)}_2_][*pf*]_2_ with a *trans*-bent double bond served as a model compound, as summarized in Scheme 10.

To the best of our knowledge, our results include the first additions of H–O and H–C bonds to a *trans*-bent ditrielene double bond and the first C–F bond activation with a subvalent cationic group 13 complex. Notably, the [2 + 2] cycloaddition reaction between a dicationic digallene and an alkene is a stepwise reaction and most likely a polar addition, without radical intermediates. The starting materials, cycloaddition products and open-chain intermediates are in equilibrium with each other, with the steric bulk of the substrate determining the position of the equilibrium. Consequently, employing doubly substituted *cis*-alkenes does not yield the expected cycloaddition product, but instead *cis*/*trans* mixtures of the starting material, which may also be obtained catalytically.

In addition, we demonstrated the bond cleavage of very strong single bonds, *i.e.*, H–O, H–N, H–C and F–C bonds with [{Ga(dcpe)}_2_]^2+^, under mild conditions. The H–O and H–N bonds are even cleaved under ambient conditions. These results underline the pronounced reactivity of dicationic digallenes – probably proceeding through an accessible asymmetric conformer, which was shown to be only 10 kJ mol^−1^ higher in energy than the ground state using DFT calculations. The electronic influence of substrate/digallene adduct formation is clearly visible from the QTAIM and EDA-NOCV analyses, revealing asymmetric charge redistribution facilitating the bond activation. Quantum chemical calculations indicate that the H–N, H–O and C–F bonds are likely cleaved in a concerted, cooperative mechanism, promoted by substrate coordination. The oxidative addition into the H–C bond of acetonitrile probably also proceeds *via* initial coordination of MeCN and a series of protonation/deprotonation steps. It appears that coordination of the substrate to the Ga⇄Ga double bond is a prerequisite for activation of strong bonds, as similar observations were made with fluorinated organic compounds: while [{Ga(dcpe)}_2_]^2+^ cleaves the C–F bonds in substrates with reasonably electron rich aromatic π-systems or double bonds in a highly exergonic reaction, it does not react with very poor ligands. Notably, the facile C–F cleavage in 3FB, which is difficult to activate with neutral, subvalent group 13 species, implies that the positive charge of the digallene may induce a reactivity, which is complementary to that of the neutral congeners.

These results shall spark the application of cationic digallenes as *in situ* accessible catalysts in organic reactions, very much like in classical transition metal chemistry. To this end, further reversible bond activation reactions have to be achieved. Olefin/digallene or nitrile/digallene combinations are the most promising candidates for a catalytic cycle, in light of the reversibility of the [2 + 2] cycloaddition. While the thermodynamic stability of the C–F activation products probably impedes follow-up reactions, the N–H and O–H addition products are less thermodynamically stable and may thus serve as starting points for catalytic reactions. Further experiments are under way in order to fine tune the experimental conditions, find a suitable ligand/substrate combination, and to eventually perform main group metal catalysis with subvalent gallium.

## Author contributions

A. B., N. G. K. and I. K. conceived the experiments. N. G. K., A. B., and C. R. performed the experiments. H. S. measured the NMR spectra. All authors analyzed and discussed the experimental data. A. B. and I. K. co-wrote the paper and edited the manuscript.

## Conflicts of interest

There are no conflicts to declare.

## Supplementary Material

SC-OLF-D5SC09508E-s001

SC-OLF-D5SC09508E-s002

## Data Availability

Supplementary information (SI): experimental details, procedures, weights, and IR- and NMR spectra of the reactions; details of the quantum chemical calculations together with crystallographic details. See DOI: https://doi.org/10.1039/d5sc09508e. CCDC 2488257–2488261 and 2490061 contain the supplementary crystallographic data for this paper.^69*a*–*f*^

## References

[cit1] Power P. P. (2010). Nature.

[cit2] Weetman C., Inoue S. (2018). ChemCatChem.

[cit3] Melen R. L. (2019). Science.

[cit4] Chu T., Nikonov G. I. (2018). Chem. Rev..

[cit5] Malrieu J. P., Trinquier G. (1989). J. Am. Chem. Soc..

[cit6] Trinquier G., Malrieu J. P., Riviere P. (1982). J. Am. Chem. Soc..

[cit7] Power P. P. (1999). Chem. Rev..

[cit8] Davidson P. J., Lappert M. F. (1973). J. Chem. Soc., Chem. Commun..

[cit9] West R., Fink M. J., Michl J. (1981). Science.

[cit10] Snow J. T., Murakami S., Masamune S., Williams D. J. (1984). Tetrahedron Lett..

[cit11] Goldberg D. E., Harris D. H., Lappert M. F., Thomas K. M. (1976). J. Chem. Soc., Chem. Commun..

[cit12] Stürmann M., Weidenbruch M., Klinkhammer K. W., Lissner F., Marsmann H. (1998). Organometallics.

[cit13] Batcheller S. A., Masamune S. (1988). Tetrahedron Lett..

[cit14] Wendel D., Szilvási T., Henschel D., Altmann P. J., Jandl C., Inoue S., Rieger B. (2018). Angew. Chem., Int. Ed..

[cit15] Hardman N. J., Wright R. J., Phillips A. D., Power P. P. (2002). Angew. Chem., Int. Ed..

[cit16] Hardman N. J., Wright R. J., Phillips A. D., Power P. P. (2003). J. Am. Chem. Soc..

[cit17] García-Romero Á., Fernández I., Goicoechea J. M. (2025). Angew. Chem., Int. Ed..

[cit18] Liu X., Dong S., Zhu J., Inoue S. (2024). J. Am. Chem. Soc..

[cit19] Zhu Z., Wang X., Peng Y., Lei H., Fettinger J. C., Rivard E., Power P. P. (2009). Angew. Chem., Int. Ed..

[cit20] Weetman C., Porzelt A., Bag P., Hanusch F., Inoue S. (2020). Chem. Sci..

[cit21] Schmidt E. S., Jockisch A., Schmidbaur H. (1999). J. Am. Chem. Soc..

[cit22] Kysliak O., Görls H., Kretschmer R. (2021). J. Am. Chem. Soc..

[cit23] Hardman N. J., Eichler B. E., Power P. P. (2000). Chem. Commun..

[cit24] Cui C., Roesky H. W., Schmidt H.-G., Noltemeyer M., Hao H., Cimpoesu F. (2000). Angew. Chem., Int. Ed..

[cit25] Hicks J., Vasko P., Goicoechea J. M., Aldridge S. (2018). Nature.

[cit26] Koshino K., Kinjo R. (2020). J. Am. Chem. Soc..

[cit27] Hicks J., Vasko P., Goicoechea J. M., Aldridge S. (2021). Angew. Chem., Int. Ed..

[cit28] Driess M., Grützmacher H. (1996). Angew. Chem., Int. Ed..

[cit29] Barthélemy A., Scherer H., Daub M., Bugnet A., Krossing I. (2023). Angew. Chem., Int. Ed..

[cit30] Luo Y.-R. , Comprehensive Handbook of Chemical Bond Energies, CRC Press, 2007

[cit31] Ruscic B., Feller D., Peterson K. A. (2014). Theor. Chem. Acc..

[cit32] Wohlfarth C. and Luo Y.-R., in CRC handbook of chemistry and physics, ed. W. M. Haynes, D. R. Lide, T. J. Bruno, and W. M. Haynes, CRC Press, Boca Raton, 97th edn, 2017, 6-199–6-220 and 9-73–6-86

[cit33] Barthélemy A. , Dabringhaus P., Jacob E., Koger H., Röhner D., Schmitt M., Sellin M., and Krossing I. , in Comprehensive Inorganic Chemistry III, ed. J. Reedijk, and K. R. Poeppelmeier, Elsevier, Amsterdam, 3rd edn, 2023, pp. 378–438

[cit34] Wehmschulte R. J., Peverati R., Powell D. R. (2019). Inorg. Chem..

[cit35] Dabringhaus P., Heizmann T., Krossing I. (2023). Chem.–Eur. J..

[cit36] Seifert A., Scheid D., Linti G., Zessin T. (2009). Chem.–Eur. J..

[cit37] Barthélemy A., Krossing I. (2024). Inorg. Chem..

[cit38] Dabringhaus P., Barthélemy A., Krossing I. (2021). Z. Anorg. Allg. Chem..

[cit39] Schorpp M., Tamim R., Krossing I. (2021). Dalton Trans..

[cit40] Dabringhaus P., Scherer H., Krossing I. (2024). Nat. Synth..

[cit41] Slattery J. M., Higelin A., Bayer T., Krossing I. (2010). Angew. Chem., Int. Ed..

[cit42] Krossing I., Reisinger A. (2006). Coord. Chem. Rev..

[cit43] Glootz K., Kratzert D., Himmel D., Kastro A., Yassine Z., Findeisen T., Krossing I. (2018). Angew. Chem., Int. Ed..

[cit44] Goerigk L., Hansen A., Bauer C., Ehrlich S., Najibi A., Grimme S. (2017). Phys. Chem. Chem. Phys..

[cit45] Dunning H. N. (1953). Ind. Eng. Chem..

[cit46] Bergman R. G., Sherrod S. A. (1971). J. Am. Chem. Soc..

[cit47] Tietze L. F. , and Kettschau G., in Topics in Current Chemistry, ed. A. de Meijere, K. N. Houk, J.-M. Lehn, S. V. Ley, J. Thiem, B. M. Trost, F. Vögtle, H. Yamamoto, and P. Metz, Springer, Berlin, Heidelberg, 1997, vol. 189, pp. 1–120

[cit48] Barthélemy A. , Investigations of the Ligand-Dependent Clustering Tendency and Reactivity of Cationic, Subvalent Gallium and Indium Complexes, PhD thesis, Albert-Ludwigs University Freiburg, 2024

[cit49] Caputo C. A., Guo J.-D., Nagase S., Fettinger J. C., Power P. P. (2012). J. Am. Chem. Soc..

[cit50] Chu T., Korobkov I., Nikonov G. I. (2014). J. Am. Chem. Soc..

[cit51] McOnie S. L., Özpınar G. A., Bourque J. L., Müller T., Baines K. M. (2021). Dalton Trans..

[cit52] Sarkar D., Vasko P., Ying L., Struijs J. J. C., Griffin L. P., Aldridge S. (2025). Angew. Chem., Int. Ed..

[cit53] Barthélemy A., Scherer H., Weller H., Krossing I. (2024). Chem.–Eur. J..

[cit54] (b) Goldberg K. I. , Goldman A. S., in Organometallic C-H Bond Activation: An Introduction, ed. K. I. Goldberg and A. S. Goldman, ACS symposium series, American Chemical Society, Washington DC, 2004, vol. 885, pp. 1–43

[cit55] Hicks J., Vasko P., Heilmann A., Goicoechea J. M., Aldridge S. (2020). Angew. Chem., Int. Ed..

[cit56] Jones C., Mills D. P., Rose R. P. (2006). J. Organomet. Chem..

[cit57] Chu T., Nikonov G. I. (2018). Chem. Rev..

[cit58] Johnson S. A. , Hatnean J. A., and Doster M. E. in Progress in Inorganic Chemistry, ed. K. D. Karlin, Wiley, 2011, 255–352

[cit59] Kuehnel M. F., Lentz D., Braun T. (2013). Angew. Chem., Int. Ed..

[cit60] Macgregor S. A., McKay D., Panetier J. A., Whittlesey M. K. (2013). Dalton Trans..

[cit61] Eisenstein O., Milani J., Perutz R. N. (2017). Chem. Rev..

[cit62] Luck R. L., Morris R. H. (1984). J. Am. Chem. Soc..

[cit63] Armbruster C., Sellin M., Seiler M., Würz T., Oesten F., Schmucker M., Sterbak T., Fischer J., Radtke V., Hunger J. (2024). et al.. Nat. Commun..

[cit64] Bakewell C., White A. J. P., Crimmin M. R. (2016). J. Am. Chem. Soc..

[cit65] Crimmin M. R., Butler M. J., White A. J. P. (2015). Chem. Commun..

[cit66] Chu T., Boyko Y., Korobkov I., Nikonov G. I. (2015). Organometallics.

[cit67] Hijazi A. K., Al Hmaideen A., Syukri S., Radhakrishnan N., Herdtweck E., Voit B., Kühn F. E. (2008). Eur. J. Inorg. Chem..

[cit68] Andrews S. S., Boxer S. G. (2000). J. Phys. Chem. A.

[cit69] (a) CCDC 2488257: Experimental Crystal Structure Determination, 2026, 10.5517/ccdc.csd.cc2pj7cv

