# Supplementary material for: Why is a dicationic digallene so reactive towards activation of strong covalent bonds? Scope and mechanistic investigations
Source: Chem Sci. 2026 Feb 2;17(8):4004–20. doi: 10.1039/d5sc09508e (PMC12914561; doi:10.1039/d5sc09508e)
Supplement: SC-017-D5SC09508E-s001 [file SC-017-D5SC09508E-s001.pdf]

Supplementary Information (SI) for Chemical Science.
This journal is © The Royal Society of Chemistry 2026

Supplementary Information to

Why is a Dicationic Digallene so Reactive towards Activation of Strong Covalent Bonds? Scope and Mechanistic Investigations

Dr. Antoine Barthélemy,[a] M.Sc. Nico Gino Kub,[a] M.Sc. Celine Regnat,[a] Dr. Harald Scherer,[a] Prof. Dr. Ingo Krossing*[a]

[a]Institut für Anorganische und Analytische Chemie and Freiburger Materialforschungszentrum (FMF), Universität Freiburg, Albertstr. 21, 79104 Freiburg, Germany. *Correspondence to krossing@uni-freiburg.de.

Table of Contents

1 General Remarks 1

1.1 Single Crystal X-Ray Diffraction 1

1.2 NMR Spectroscopy 2

1.3 IR Spectroscopy 3

1.4 Quantum Chemical Calculations 4

2 Syntheses 6

2.1 NMR Investigations of [2+2] Cycloaddition Reactions with $[\{Ga(dcpe)\}_2]^{2+}$ 6

2.1.1 Reactions with *trans*-β-deutero-styrene, *cis*-β-methyl-styrene, *cis*-3-hexene and ethinylcyclopropane 6

2.2 $[\{Ga(dcpe)\}_2(C_6H_{10})]$[*pf*]$_2$ 6

2.3 $[H\{Ga(dcpe)\}_2(OEt)]$[*pf*]$_2$ 7

2.4 $[H\{Ga(dcpe)\}_2(OPh)]$[*pf*]$_2$ 7

2.5 $[H\{Ga(dcpe)\}_2(NHC_4H_9)]$[*pf*]$_2$ 7

2.6 $[H\{Ga(dcpe)\}_2(NHPh)]$[*pf*]$_2$ 8

2.7 $[H\{Ga(dcpe)\}_2(NPh_2)]$[*pf*]$_2$ 8

2.8 $[H\{Ga(dcpe)\}_2(CH_2CN)]$[*pf*]$_2$ 8

2.9 Reaction between H_3C–CN and D_3C–CN with $[\{Ga(dcpe)\}_2]$[*pf*]$_2$ 9

2.10 $[\{Ga(dcpe)\}_2(NCCH_2F)]$[*pf*]$_2$ and $[F\{Ga(dcpe)\}_2(CH_2CN)]$[*pf*]$_2$ 9

2.11 $[F\{Ga(dcpe)\}_2(C_5H_6F)]$[*pf*]$_2$ 10

2.12 $[F\{Ga(dcpe)\}_2(COOCH_2CHO)]$[*pf*]$_2$ 10

2.13 $[F\{Ga(dcpe)\}_2(C_6F_2H_3)]$[*pf*]$_{5/3}$[*alfal*]$_{1/3}$ 11

3 Crystal Structures 12

3.1 $[\{Ga(dcpe)\}_2(C_6H_{10})]$[*pf*]$_2$ * C_6H_{10} 15

3.2 $[H\{Ga(dcpe)\}_2(OEt)]$[*pf*]$_2$ 16

3.3 $[H\{Ga(dcpe)\}_2(OPh)]$[*pf*]$_2$ 17

3.4 $[H\{Ga(dcpe)\}_2(NHPh)]$[*pf*]$_2$ 18

3.5 $[H\{Ga(dcpe)\}_2(NPh_2)]$[*pf*]$_2$ 20

3.6 $[H\{Ga(dcpe)\}_2(CH_2CN)]$[*pf*]$_2$ 21

3.7 $[F\{Ga(dcpe)\}_2(CH_2CN)]$[*pf*]$_2$ 22

3.8 $[F\{Ga(dcpe)\}_2(C_6F_2H_3)]$[*pf*]$_{5/3}$[*alfal*]$_{1/3}$ 25

4 NMR Spectroscopy 27

4.1 NMR Investigations of [2+2] Cycloaddition Reactions with $[\{Ga(dcpe)\}_2]^{2+}$ 27

4.1.1 Reaction with *trans*-β-deutero-styrene 27

4.1.2 Reaction with *cis*-β-methyl-styrene 31

4.1.3 Reaction with *cis*-3-hexene 33

4.1.4 $[\{Ga(dcpe)\}_2(C_5H_6)]$[*pf*]$_2$ 35

4.2 [{Ga(dcpe)}$_2$(C_6H_{10})][*pf*]$_2$ 40
4.3 [H{Ga(dcpe)}$_2$(OEt)][*pf*]$_2$ 45
4.4 [H{Ga(dcpe)}$_2$(OPh)][*pf*]$_2$ 51
4.4.1 Observed Intermediates during the Formation of [H{Ga(dcpe)}$_2$OPh]$^{2+}$ 56
4.5 [H{Ga(dcpe)}$_2$(NH*n*Bu)][*pf*]$_2$ 58
4.6 [H{Ga(dcpe)}$_2$(NHPh)][*pf*]$_2$ 64
4.6.1 Observed Intermediates during the Formation of [H{Ga(dcpe)}$_2$NHPh]$^{2+}$ 70
4.7 [H{Ga(dcpe)}$_2$(NPh_2)][*pf*]$_2$ 72
4.7.1 Observed Intermediates during the Formation of [H{Ga(dcpe)}$_2$$NPh_2$]$^{2+}$ 77
4.8 [H{Ga(dcpe)}$_2$(CH_2CN)][*pf*]$_2$ 83
4.9 Reaction between H_3C–CN and D_3C–CN with [{Ga(dcpe)}$_2$][*pf*]$_2$ 89
4.10 [{Ga(dcpe)}$_2$($NCCH_2F$)][*pf*]$_2$ and its Decomposition in Solution 92
4.10.1 [{Ga(dcpe)}$_2$($NCCH_2F$)][*pf*]$_2$ 92
4.10.2 Decomposition of [{Ga(dcpe)}$_2$($NCCH_2F$)]$^{2+}$ in Solution 97
4.11 [{Ga(dcpe)}$_2$(NCPh)][*pf*]$_2$ and its Decomposition in Solution 99
4.11.1 [{Ga(dcpe)}$_2$(NCPh)][*pf*]$_2$ 99
4.11.2 Decomposition of [{Ga(dcpe)}$_2$(NCPh)]$^{2+}$ in Solution 101
4.12 [F{Ga(dcpe)}$_2$(CH_2CN)][*pf*]$_2$ 102
4.13 [F{Ga(dcpe)}$_2$(C_5H_6F)][*pf*]$_2$ 109
4.14 [F{Ga(dcpe)}$_2$($COOCH_2CHO$)][*pf*]$_2$ 114
4.15 [F{Ga(dcpe)}$_2$($C_6F_2H_3$)][*pf*]$_{5/3}$[*alfal*]$_{1/3}$ 120
4.16 Unselective Bond Activation Reactions 126
4.16.1 Reaction Between [{Ga(dcpe)}$_2$]$^{2+}$ and $PhCF_3$ 126
4.16.2 Reaction Between [{Ga(dcpe)}$_2$]$^{2+}$ and 4FB 127
5 Vibrational Spectroscopy 129
5.1 [{Ga(dcpe)}$_2$(C_6H_{10})][*pf*]$_2$ 129
5.2 [H{Ga(dcpe)}$_2$(OEt)][*pf*]$_2$ 131
5.3 [H{Ga(dcpe)}$_2$(OPh)][*pf*]$_2$ 133
5.4 [H{Ga(dcpe)}$_2$(NH*n*Bu)][*pf*]$_2$ 135
5.5 [H{Ga(dcpe)}$_2$(NHPh)][*pf*]$_2$ 137
5.6 [H{Ga(dcpe)}$_2$(NPh_2)][*pf*]$_2$ 139
5.7 [H{Ga(dcpe)}$_2$(CH_2CN)][*pf*]$_2$ 141
5.8 [F{Ga(dcpe)}$_2$(CH_2CN)][*pf*]$_2$ 143
5.9 [F{Ga(dcpe)}$_2$($COOCH_2CHO$)][*pf*]$_2$ 145
5.10 [F{Ga(dcpe)}$_2$($C_6F_2H_3$)][*pf*]$_{5/3}$[*alfal*]$_{1/3}$ 147
6 Quantum Chemical Calculations 149

6.1 EDA-NOCV Analysis ... 149
6.1.1 [{Ga(dcpe)}$_2$]$^{2+}$ + HOMe ... 149
6.1.2 [{Ga(dcpe)}$_2$]$^{2+}$ + $HNMe_2$ ... 151
6.1.3 Summary of the EDA-NOCV Analysis ... 153
6.2 Optimized Geometries ... 154
6.2.1 [{Ga(dcpe)}$_2$]$^{2+}$ (symmetric) ... 154
6.2.2 [{Ga(dcpe)}$_2$]$^{2+}$ (asymmetric) ... 164
6.2.3 [Ga(dtbpf)]$^{+}$ ... 175
6.2.4 [Ga(dcpe)]$^{+}$ ... 181
6.2.5 $HCCC_3H_5$ ... 187
6.2.6 [{Ga(dcpe)}$_2$($HCCC_3H_5$)]$^{2+}$ ... 188
6.2.7 C_6H_{10} ... 200
6.2.8 [{Ga(dcpe)}$_2$(C_6H_{10})]$^{2+}$ ... 201
6.2.9 EtOH ... 214
6.2.10 [H{Ga(dcpe)}$_2$(OEt)]$^{2+}$ ... 215
6.2.11 PhOH ... 227
6.2.12 [H{Ga(dcpe)}$_2$(OPh)]$^{2+}$ ... 228
6.2.13 $HNPh_2$ ... 240
6.2.14 [H{Ga(dcpe)}$_2$(NPh_2)]$^{2+}$ ... 242
6.2.15 [H{Ga(dcpe)}(NPh_2)]$^{+}$ ... 254
6.2.16 H_2NPh ... 261
6.2.17 [H{Ga(dcpe)}$_2$(HNPh)]$^{2+}$ ... 263
6.2.18 H_2N*n*Bu ... 275
6.2.19 [H{Ga(dcpe)}$_2$(HN*n*Bu)]$^{2+}$ ... 276
6.2.20 HOMe ... 289
6.2.21 [{Ga(dcpe)}$_2$(HOMe)]$^{2+}$ ... 290
6.2.22 [{Ga(dcpe)}$_2$(H–OMe)]$^{2+}$ (Transition State) ... 301
6.2.23 [H{Ga(dcpe)}$_2$(OMe)]$^{2+}$ (*syn*-periplanar) ... 313
6.2.24 [H{Ga(dcpe)}$_2$(OMe)]$^{2+}$ (*anti*-periplanar) ... 325
6.2.25 $HNMe_2$ ... 336
6.2.26 [{Ga(dcpe)}$_2$($HNMe_2$)]$^{2+}$ ... 337
6.2.27 [{Ga(dcpe)}$_2$(H–NMe_2)]$^{2+}$ (Transition State) ... 349
6.2.28 [H{Ga(dcpe)}$_2$(NMe_2)]$^{2+}$ (*syn*-periplanar) ... 361
6.2.29 [H{Ga(dcpe)}$_2$(NMe_2)]$^{2+}$ (*anti*-periplanar) ... 373
6.2.30 H_2NMe ... 385
6.2.31 [{Ga(dcpe)}$_2$(H_2NMe)]$^{2+}$ ... 386

6.2.32 [{Ga(dcpe)}$_2$(H–NHMe)]$^{2+}$ (Transition State) 398
6.2.33 [H{Ga(dcpe)}$_2$(NHMe)]$^{2+}$ (*syn*-periplanar) 409
6.2.34 [H{Ga(dcpe)}$_2$(NHMe)]$^{2+}$ (*anti*-periplanar) 421
6.2.35 H_3CCN 433
6.2.36 [{Ga(dcpe)}$_2$($NCCH_3$)]$^{2+}$ (Adduct) 434
6.2.37 [H{Ga(dcpe)}$_2$($NCCH_3$)]$^{3+}$ 445
6.2.38 [{Ga(dcpe)}$_2$($NCCH_2$)]$^{+}$ 457
6.2.39 [H{Ga(dcpe)}$_2$($NCCH_2$)]$^{2+}$ 469
6.2.40 [H{Ga(dcpe)}$_2$($NCCH_2$)]$^{2+}$/[H{Ga(dcpe)}$_2$(CH_2CN)]$^{2+}$ Transition State 480
6.2.41 [H{Ga(dcpe)}$_2$(CH_2CN)]$^{2+}$ 492
6.2.42 [{Ga(dcpe)}$_2$($NCCH_3$)]$^{2+}$ (Cycloadduct) 504
6.2.43 PhCN 515
6.2.44 [{Ga(dcpe)}$_2$(NCPh)]$^{2+}$ (Cycloadduct) 517
6.2.45 H_2FCCN 529
6.2.46 [{Ga(dcpe)}$_2$($NCCH_2F$)]$^{2+}$ (Cycloadduct) 530
6.2.47 [F{Ga(dcpe)}$_2$(CH_2CN)]$^{2+}$ 541
6.2.48 $PhCF_3$ 553
6.2.49 [F{Ga(dcpe)}$_2$(CF_2Ph)]$^{2+}$ 554
6.2.50 $C_5H_6F_2$ 567
6.2.51 [F{Ga(dcpe)}$_2$(C_5H_6F)]$^{2+}$ 568
6.2.52 FEC 579
6.2.53 [F{Ga(dcpe)}$_2$($COOCH_2CHO$)]$^{2+}$ 581
6.2.54 1,2,3-$C_6F_3H_3$ (3FB) 593
6.2.55 [{Ga(dcpe)}$_2$($C_6F_3H_3$)]$^{2+}$ (Adduct) 594
6.2.56 [{Ga(dcpe)}$_2$(F–$C_6F_2H_3$)]$^{2+}$ Transition State 606
6.2.57 [F{Ga(dcpe)}$_2$($C_6F_2H_3$)]$^{2+}$ (*syn*-periplanar) 618
6.2.58 [F{Ga(dcpe)}$_2$($C_6F_2H_3$)]$^{2+}$ (*anti*-periplanar) 630
6.2.59 [*pf*]$^{-}$ 642
7 References 647

1 General Remarks

All manipulations were carried out under exclusion of moisture and air through usage of a *MBraun* glovebox filled with nitrogen (O_2/H_2O < 1 ppm) and standard Schlenk technique. All glassware used in reactions have been stored overnight in an oven at 180 °C and were additionally dried with a heat gun prior to usage.

Ortho-difluorobenzene (*o*DFB) was dried over CaH_2 for two days, distilled and degassed prior to use. and stored under an atmosphere of argon or nitrogen in sealed vessels and over 4 Å molecular sieves. The water content of the solvents was below 8 ppm, as determined by Carl Fischer titration.

Li[*pf*],[1] Ag[*pf*] (synthesis in SO_2, instead of CH_2Cl_2 or perfluorohexane, under ambient conditions),[1,2] $[Ga(PhF)_2]$[*pf*],[3] $[\{Ga(dcpe)\}_2]$[*pf*]$_2$[4] (dcpe = *bis*(dicyclohexylphosphino)ethane; [*pf*]$^-$ = $[Al(OR^F)_4]^-$, R^F = $C(CF_3)_3$) and *trans*-β-deutero-styrene[5] were prepared according to literature protocols. Note that, in the fluorobenzene complex of Ga^+, the number of fluorobenzene molecules coordinated to Ga^+ can vary, depending on the vacuum applied when drying the product. Thus, the formula $[Ga(PhF)_2]$[*pf*] is used for the sake of simplicity instead of $[Ga(PhF)_x]$[*pf*] (1 < x < 3). The exact ratio x was determined *via* ^{19}F NMR spectroscopy.

1.1 Single Crystal X-Ray Diffraction

Crystals were obtained by layering a concentrated *o*DFB solution with *n*-pentane. Single crystal X-ray diffraction data were collected from shock-cooled single crystals at 100(2) K on a Bruker *D8 VENTURE* dual wavelength Mo/Cu three-circle diffractometer with a microfocus sealed X-ray tube using a mirror optics as monochromator and a Bruker *PHOTON III* detector. Crystals were selected under perfluoropolyether oil, mounted on 0.1 to 0.2 mm diameter CryoLoops and quench-cooled using an *Oxford Cryostream 800* open flow N_2 cooling device.[6] Data were collected at 100 K using monochromated Mo K_α radiation (λ = 0.71073 Å).

All data were integrated with SAINT and a multi-scan absorption correction using SADABS was applied.[7] The structure was solved by direct methods using SHELXT and refined by full-matrix least-squares methods against F^2 by SHELXL-2018/3[8] employing shelXle.[9] All non-hydrogen atoms were refined with anisotropic displacement parameters. The hydrogen atoms were refined isotropically on calculated positions using a riding model with their U_{iso} values constrained to 1.5 times the U_{eq} of their pivot atoms for terminal sp^3 carbon atoms and 1.2 times for all other carbon atoms. Disordered

moieties were refined using bond lengths restraints and displacement parameter restraints. Some parts of the disorder model were introduced by the program DSR.[10] Graphical representations were prepared using Olex2-1.3.[11]

Crystallographic data for the structure reported in this paper have been deposited with the Cambridge Crystallographic Data Centre.[12] CCDC numbers 2488257–2488261 and 2490061 contain the supplementary crystallographic data for this paper. Copies of the data can be obtained free of charge from the Cambridge Crystallographic Data Centre *via* www.ccdc.cam.ac.uk/structures. This report and the CIF file were generated using FinalCif.[13]

1.2 NMR Spectroscopy

^{1}H-, ^{2}D-, ^{13}C-, ^{19}F-, ^{27}Al-, ^{31}P, ^{71}Ga, and 2D NMR spectra were detected on an *Avance DPX 200* (200 MHz), *Avance III HD* (300 MHz) and *Avance II+ 400* (400 MHz) NMR spectrometer from *Bruker*. NMR spectra were analyzed using *Bruker TopSpin 3.2.* ^{1}H NMR chemical shifts are given with respect to tetramethylsilane (TMS). The ^{1}H NMR spectrum was calibrated using the solvent signal as secondary standard. The chemical shifts of *o*DFB and 3FB were determined experimentally by adding TMS to the aromatic solvents. The most intensive signal of the downfield multiplet of these solvents appear at 6.96 and 6.86 ppm, respectively, when the signal of TMS is at 0.00 ppm (**Figure S 1** and **Figure S *2***).

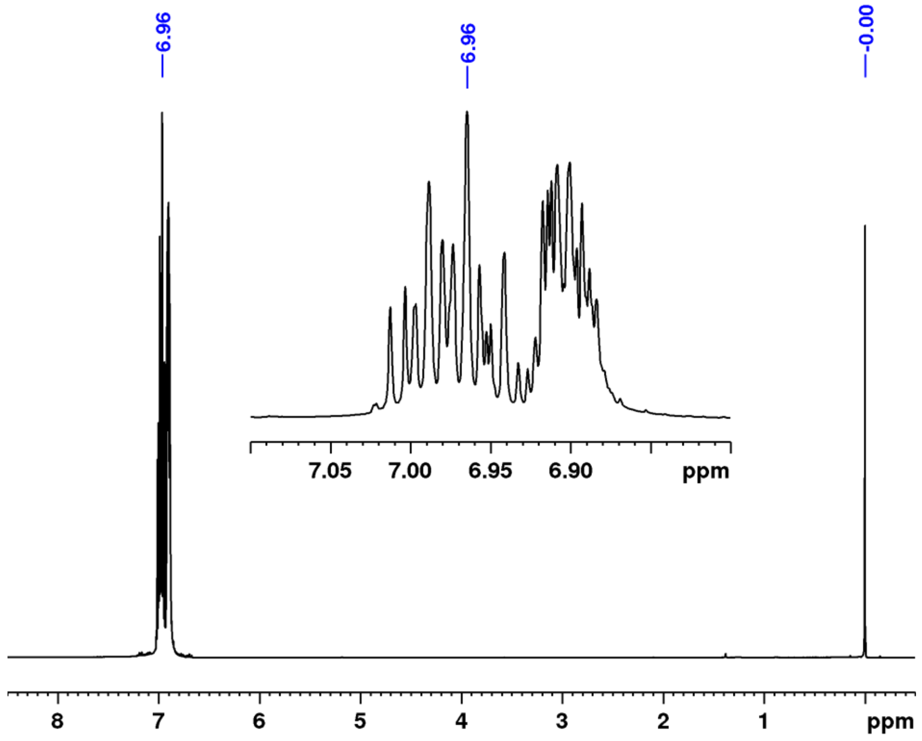

Figure S 1: ^{1}H NMR spectrum (400.17 MHz, *o*DFB, 298 K) of TMS in *o*DFB.

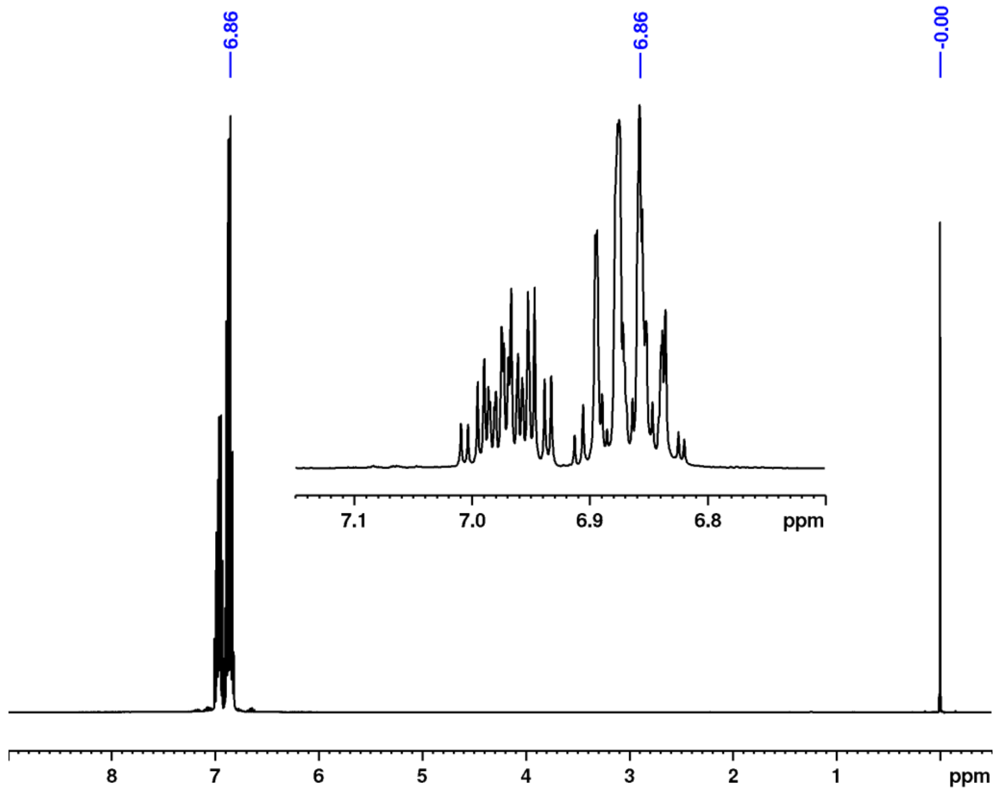

Figure S 2: ^{1}H NMR spectrum (400.17 MHz, 3FB, 298 K) of TMS in 3FB.

Heteronuclear spectra were calibrated according to the IUPAC Ξ-table.[14] The ^{19}F and ^{27}Al NMR spectra show signals at ca. –75 ppm and 35 ppm, respectively, indicating the presence of non-decomposed $[Al(OR^F)_4]^-$ anion. The broad resonance between 50 and 100 ppm in the ^{27}Al NMR spectra is caused by the probe head. Note that ^{71}Ga spectra without a detected signal, due to the quadrupolar relaxation of ^{71}Ga, are not shown here.

1.3 IR Spectroscopy

IR spectra FT-IR spectra were recorded on a FT-IR Bruker Alpha, equipped with a Quick Snap Platinum ATR (diamond or ZnSe crystal) unit inside a glove box under an atmosphere of nitrogen at room temperature (rt). The spectra were recorded with a resolution of 2 cm^{-1} and 64 scans. For measurements and data processing, the software *OPUS 7.5* (Bruker Optic GmbH) was used. All spectra were ATR corrected and base line corrections (5 cycles) were employed. By setting the most intense peak to 100 %, the other IR bands were given the following intensity assignment: very weak (vw) < 20 %, weak (w) < 40 %; medium (m) < 60 %, strong (s) < 80 % and very strong (vs) for ≥ 80 %.

1.4 Quantum Chemical Calculations

Quantum chemical calculations were performed with *Turbomole* (version 7.5.1).[15,16] Structures were optimized using density functional theory (DFT),[15] internal coordinates, resolution of identity-approximation (RI),[17,18] D3(BJ)-dispersion correction[19] and a fine integration gridsize (gridsize = m5). Calculations were performed on the RI-BP86[20](D3BJ)/def2-TZVPP[21] level of theory. Thermal and entropic contributions to the Gibbs energy were calculated without scaling factor at standard conditions (298.15 K, 0.1 MPa) with the FREEH module. Every species presented herein were checked in terms of reasonable geometry and electronic occupation with the EIGER module. Vibrational analyses were performed with the AOFORCE module, in order to detect imaginary frequencies.[22] To counteract imaginary frequencies, the convergence criteria for geometry optimization were modified from the default 10^{-7} H (energy) and 10^{-4} a.u. (gradient) to 10^{-8} H und 10^{-5} a.u., respectively. The coordinates of the herein investigated compounds are listed in section 6.

Due to the size of the dcpe-complexes, the geometry of the herein investigated molecules was optimized at the RI-BP86(D3BJ)/def2-TZVPP level of theory. However, in order to obtain more reliable electronic energy values, the thereby optimized structures were further analysed employing a double-hybrid functional[23] using the ORCA program (version 4.2.1).[24] Single point calculations at the DSD-PBEP86[25]-D3(BJ) level of theory were performed, with the RIJCOSX[26] approximation, the def2-QZVPP[21] orbital basis set and the def2-QZVPP/C[27] auxiliary basis set. The DSD-PBEP86 double-hybrid functional is known to yield accurate thermochemistry results for various types of reactions involving main group elements.[28] The results from the vibrational analysis were taken from the respective gas phase calculations at the RI-BP86(D3BJ)/def2-TZVPP level.

For each molecular species, the standard enthalpy $H°$ at 298.15 K and 0.1 MPa was calculated from the electronic energy obtained by the double-hybrid energy E_{DH} and the sum of translational, rotational, and vibrational energy including zero-point energy E_{vrt} (FREEH energy) using the following equation:

$$H° = E_{\mathrm{DH}} + E_{\mathrm{vrt}} + \mathrm{R} \cdot \mathrm{T} \qquad (1)$$

E_{DH}: electronic double-hybrid energy

E_{vrt}: sum of translational, rotational, and vibrational energy including zero-point energy

R: universal gas constant (8.314 J K^{-1} mol^{-1})

T: temperature in Kelvin (298.15 K)

The Gibbs free energy $G°$ follows from the standard enthalpy $H°$ and the standard entropy $S°$:

$$G° = H° - S° \cdot T \tag{2}$$

Solvation effects were incorporated using the conductor like screening model (COSMO).[29] A dielectric constant of 13.38 D for *o*DFB[30] and of 22.1 D for 3FB[31] was assumed at 298 K. Single point calculations were performed on the optimized gas phase structures and the results from the vibrational analysis were taken from the respective gas phase calculations.

In order to find transition state structures, relaxed potential energy surface scans along selected bond lengths or bond angles were performed with the ORCA program on the RI-BP86(D3BJ)/def2-SVP[17,21] level of theory. The geometry of the most energy-rich species was chosen as the starting geometry for the transition state structure search in *Turbomole* at the RI-BP86(D3BJ)/def2-TZVPP level of theory. The transition states thus obtained display one imaginary frequency and were distorted along the reaction coordinate to make sure that the transition state describes the desired transformation.

The calculated energies, entropies and vibrations of all the species presented herein are summarized in section 6.

2 Syntheses

2.1 NMR Investigations of [2+2] Cycloaddition Reactions with $[\{Ga(dcpe)\}_2]^{2+}$

2.1.1 Reactions with *trans*-β-deutero-styrene, *cis*-β-methyl-styrene, *cis*-3-hexene and ethinylcyclopropane

$[Ga(PhF)_x][pf]$ (for x = 2.0: 50 mg, 40.7 µmol, 1.0 eq.) and dcpe (17.2 mg, 40.7 µmol, 1.0 eq.) were mixed in *o*DFB (ca. 0.6 ml). A stock solution of *trans*-β-deutero-styrene, *cis*-β-methyl-styrene, *cis*-3-hexene or ethinylcyclopropane in *o*DFB (0.09 mL, *c* = 0.225 M, 20.2 µmol, 0.5 eq.) was added, respectively. The reaction solutions remained red with *trans*-β-deutero-styrene, *cis*-β-methyl-styrene and *cis*-3-hexene. Only the reaction solution with ethinylcyclopropane turned colourless with minutes, indicating the complete consumption of the dicationic digallene. The reaction mixtures were analyzed *via* multinuclear NMR spectroscopy (see section 4).

2.2 $[\{Ga(dcpe)\}_2(C_6H_{10})][pf]_2$

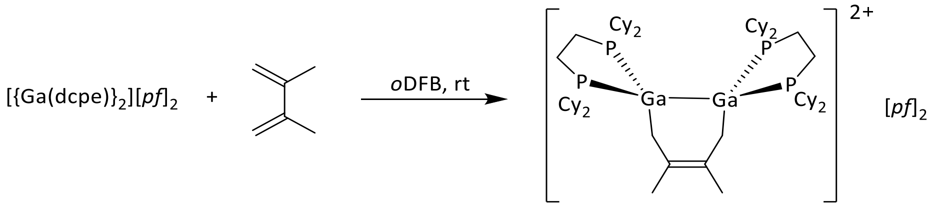

Scheme S 1: Synthesis $[\{Ga(dcpe)\}_2(C_6H_{10})][pf]_2$.

$[Ga(PhF)_x][pf]$ (for x = 2.0: 50 mg, 40.7 µmol, 1.0 eq.) and dcpe (17.2 mg, 40.7 µmol, 1.0 eq.) were mixed in *o*DFB (ca. 0.8 ml). A stock solution of 2,3-dimethylbutadiene in *o*DFB (0.06 mL, *c* = 0.43 M, 25.8 µmol, 0.63 eq.) was added. The initially red reaction solution turned pink within minutes and was stirred overnight at rt. Layering the then colourless solution with *n*-pentane (ca. 8 mL), filtration and drying the filter residue in *vacuo* (1×10^{-3} mbar) yielded white crystals of $[\{Ga(dcpe)\}_2(C_6H_{10})][pf]_2$ (13.0 mg, 4.33 µmol, 21 %).

Note that a co-crystallized 2,3-dimethylbutadiene molecule is found in the crystals which have directly been picked from the mother liquor (section 3.1). However, this molecule is probably removed when drying the product in *vacuo*, since no traces of 2,3-dimethylbutadiene are found in the NMR and IR spectra of the thoroughly dried product.

2.3 [H{Ga(dcpe)}$_2$(OEt)][*pf*]$_2$

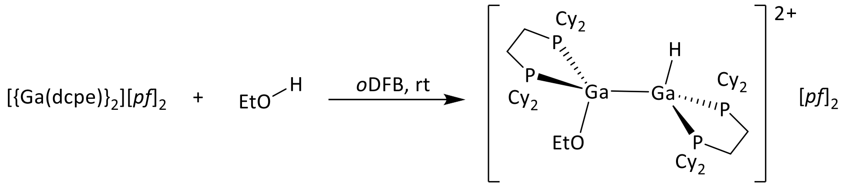

Scheme S 2: Synthesis of [H{Ga(dcpe)}$_2$(OEt)][*pf*]$_2$.

[Ga(PhF)$_x$][*pf*] (for x = 2.0: 50 mg, 40.7 µmol, 1.0 eq.) and dcpe (17.2 mg, 40.7 µmol, 1.0 eq.) were mixed in *o*DFB (ca. 0.8 ml). A stock solution of ethanol in *o*DFB (0.08 mL, *c* = 0.26 M, 20.8 µmol, 0.5 eq.) was added. The initially red reaction solution immediately turned colourless. Layering the solution with *n*-pentane (ca. 8 mL), filtration and drying the filter residue in *vacuo* (1×10^{-3} mbar) yielded colorless crystals of [H{Ga(dcpe)}$_2$(OEt)][*pf*]$_2$ (16.5 mg, 5.57 µmol, 27 %).

2.4 [H{Ga(dcpe)}$_2$(OPh)][*pf*]$_2$

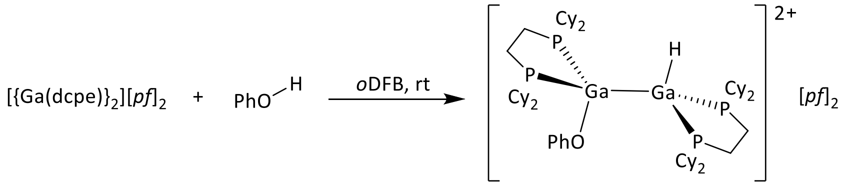

Scheme S 3: Synthesis of [H{Ga(dcpe)}$_2$(OPh)][*pf*]$_2$.

[Ga(PhF)$_x$][*pf*] (for x = 2.0: 50 mg, 40.7 µmol, 1.0 eq.) and dcpe (17.2 mg, 40.7 µmol, 1.0 eq.) were mixed in *o*DFB (ca. 0.8 ml). A stock solution of phenol in *o*DFB (0.09 mL, *c* = 0.225 M, 20.3 µmol, 0.5 eq.) was added. The initially red reaction solution immediately turned colourless. Layering the solution with *n*-pentane (ca. 8 mL), filtration and drying the filter residue in *vacuo* (1×10^{-3} mbar) yielded colorless crystals of [H{Ga(dcpe)}$_2$(OPh)][*pf*]$_2$ (45.0 mg, 14.9 µmol, 73 %).

2.5 [H{Ga(dcpe)}$_2$(NHC$_4$H$_9$)][*pf*]$_2$

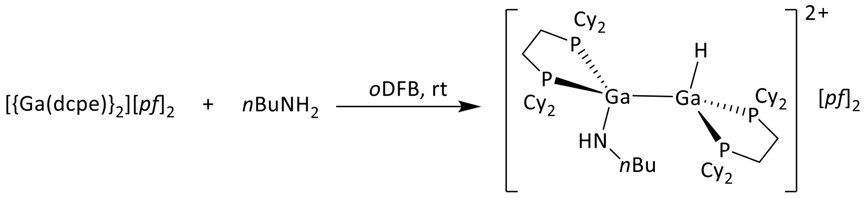

Scheme S 4: Synthesis of [H{Ga(dcpe)}$_2$(NHC$_4$H$_9$)][*pf*]$_2$.

[Ga(PhF)$_x$][*pf*] (for x = 1.0: 40 mg, 32.5 µmol, 1.0 eq.) and dcpe (13.5 mg, 31.9 µmol, 1.0 eq.) were mixed in *o*DFB (ca. 0.5 ml) upon which a stock solution of *n*butylamine in *o*DFB (0.16 mL, *c* = 0.10 M, 16.0 µmol, 0.5 eq.) was added. The initially red solution mixture turned colorless within 30 minutes. Layering the reaction solution with *n*-pentane (ca. 8 mL) yielded colorless crystals, which were not suitable for scXRD (21.3 mg, 7.12 µmol, 45 %).

2.6 [H{Ga(dcpe)}$_2$(NHPh)][*pf*]$_2$

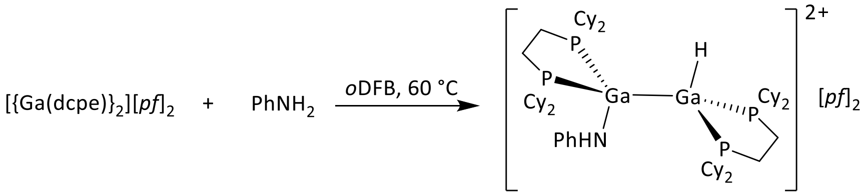

Scheme S 5: Synthesis of [H{Ga(dcpe)}$_2$(NHPh)][*pf*]$_2$.

[Ga(PhF)$_x$][*pf*] (for x = 1.0: 40 mg, 32.5 µmol, 1.0 eq.) and dcpe (13.5 mg, 31.9 µmol, 1.0 eq.) were mixed in *o*DFB (ca. 0.5 ml) upon which a stock solution of aniline in *o*DFB (0.16 mL, *c* = 0.1 M, 16.0 µmol, 0.5 eq.) was added. The initially red solution mixture turned colorless within 30 minutes. Layering the then colourless solution with *n*-pentane (ca. 8 mL), filtration and drying the filter residue in *vacuo* (1×10^{-3} mbar) yielded colorless crystals of [H{Ga(dcpe)}$_2$(NHPh)][*pf*]$_2$ (11.8 mg, 3.92 µmol, 25 %).

2.7 [H{Ga(dcpe)}$_2$(NPh$_2$)][*pf*]$_2$

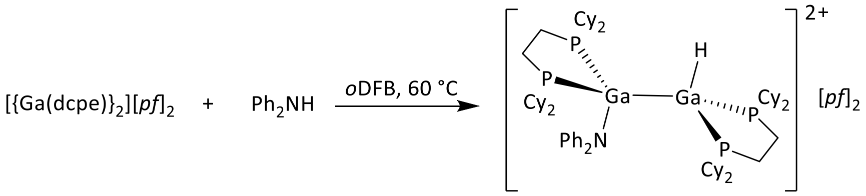

Scheme S 6: Synthesis of [H{Ga(dcpe)}$_2$(NPh$_2$)][*pf*]$_2$.

[Ga(PhF)$_x$][*pf*] (for x = 1.0: 40 mg, 32.5 µmol, 1.0 eq.) and dcpe (13.5 mg, 31.9 µmol, 1.0 eq.) were mixed in *o*DFB (ca. 0.5 ml) upon which a stock solution of diphenylamine in *o*DFB (0.16 mL, *c* = 0.1 M, 16.0 µmol, 0.5 eq.) was added. The reaction mixture was stirred for 24 hours at 60 °C. Layering the then colourless solution with *n*-pentane (ca. 8 mL), filtration and drying the filter residue in *vacuo* (1×10^{-3} mbar) yielded colorless crystals of [H{Ga(dcpe)}$_2$(NPh$_2$)][*pf*]$_2$ (37.0 mg, 12.0 µmol, 75 %).

2.8 [H{Ga(dcpe)}$_2$(CH$_2$CN)][*pf*]$_2$

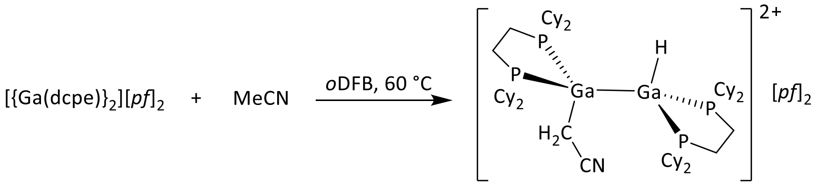

Scheme S 7: Synthesis of [H{Ga(dcpe)}$_2$(CH$_2$CN)][*pf*]$_2$.

[Ga(PhF)$_x$][*pf*] (for x = 2.0: 50 mg, 40.7 µmol, 1.0 eq.) and dcpe (17.2 mg, 40.7 µmol, 1.0 eq.) were mixed in *o*DFB (ca. 0.8 ml). A stock solution of acetonitrile in *o*DFB (0.08 mL, *c* = 0.225 M, 20.2 µmol, 0.5 eq.) was added. The red reaction solution was stirred for 4 days at 60 °C. Layering the then colourless solution with *n*-pentane (ca. 8 mL), filtration and drying the filter residue in *vacuo* (1×10^{-3} mbar) yielded white crystals of [H{Ga(dcpe)}$_2$(CH$_2$CN)][*pf*]$_2$ (33.8 mg, 11.4 µmol, 56 %).

2.9 Reaction between H_3C–CN and D_3C–CN with [{Ga(dcpe)}$_2$][*pf*]$_2$

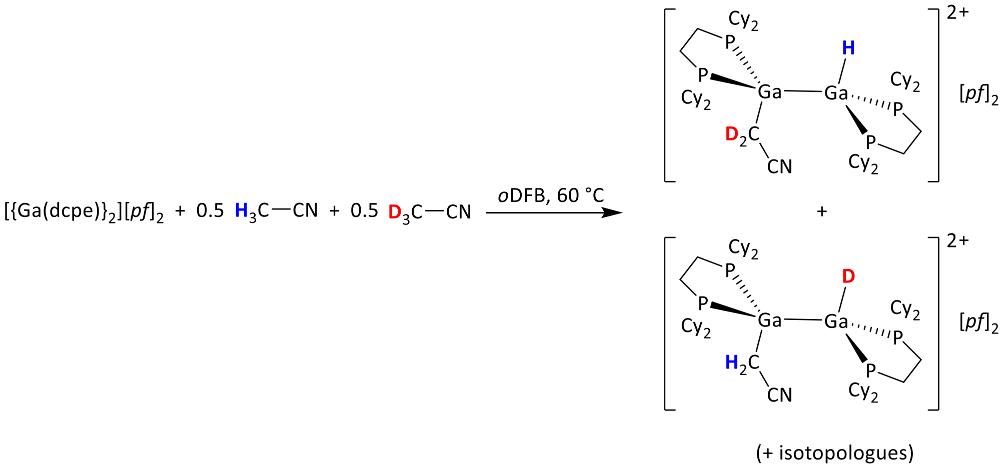

Scheme S 8: Reaction of [{Ga(dcpe)}$_2$][*pf*]$_2$ with an equimolar solution of acetonitrile and acetonitrile-*d*$_3$.

[Ga(PhF)$_x$][*pf*] (for x = 2.0: 50 mg, 45.0 µmol, 1.0 eq.) and dcpe (17.2 mg, 45.0 µmol, 1.0 eq.) were mixed in *o*DFB (ca. 0.6 ml). Two stock solutions of acetonitrile and of acetonitrile-*d*$_3$ in *o*DFB (0.05 mL, *c* = 0.225 M, 11.2 µmol, 0.25 eq., respectively) were added. The red reaction solution was stirred for 4 days at 60 °C. The then colourless reaction mixture was analyzed *via* multinuclear NMR spectroscopy (see section 4.9).

2.10 [{Ga(dcpe)}$_2$(NCCH$_2$F)][*pf*]$_2$ and [F{Ga(dcpe)}$_2$(CH$_2$CN)][*pf*]$_2$

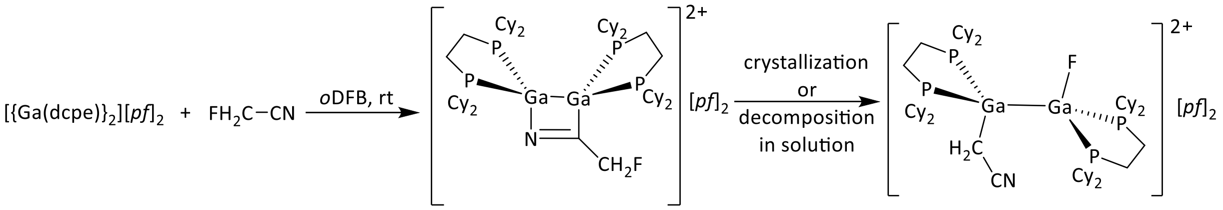

Scheme S 9: Synthesis of [H{Ga(dcpe)}$_2$(CH$_2$CN)][*pf*]$_2$.

[Ga(PhF)$_x$][*pf*] (for x = 2.0: 50 mg, 40.7 µmol, 1.0 eq.) and dcpe (17.2 mg, 40.7 µmol, 1.0 eq.) were mixed in *o*DFB (ca. 0.8 ml). A stock solution of monofluoroacetonitrile in *o*DFB (0.09 mL, *c* = 0.225 M, 20.2 µmol, 0.50 eq.) was added. The initially red reaction solution immediately turned yellow. NMR spectroscopy confirms that the CH$_2$F moiety is intact in the freshly prepared solution, while the ^{13}C NMR shift of 215 ppm and the AA'BB' spin system in the ^{31}P{^{1}H} NMR spectrum strongly suggest that the [2+2] cycloaddition product is present in solution (see section **4.10**).

Layering the solution with *n*-pentane (ca. 8 mL), filtration and drying the filter residue in *vacuo* (1×10^{-3} mbar) yielded white crystals of the C–F activation product [F{Ga(dcpe)}$_2$(CH$_2$CN)][*pf*]$_2$ (34.9 mg, 11.7 µmol, 58 %), as suggested by NMR and IR spectroscopy (see section **4.12**). Unfortunatley, the crystals were not suitable for sc-XRD

2.11 [F{Ga(dcpe)}$_2$(C_5H_6F)][*pf*]$_2$

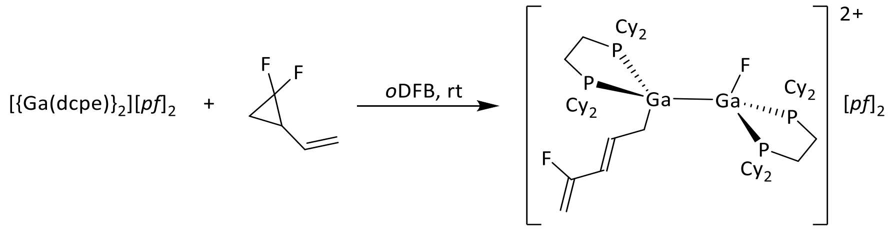

Scheme S 10: Synthesis of [F{Ga(dcpe)}$_2$(C_5H_6F)][*pf*]$_2$.

[Ga(PhF)$_x$][*pf*] (for x = 1.3: 60 mg, 51.6 µmol, 1.0 eq.) and dcpe (21.8 mg, 51.6 µmol, 1.0 eq.) were mixed in oDFB (ca. 0.6 ml). A stock solution of 1,1-difluoro-2-vinylcyclopropane in *o*DFB (0.08 mL, c = 0.43 M, 34.4 µmol, 0.7 eq.) was added. The colourless reaction solution was analyzed *via* NMR spectroscopy and the structure of the main product was determined unambiguously. Layering the reaction solution with *n*-pentane (ca. 8 mL) did not yield crystals suitable for sc-XRD (the yield was not determined).

2.12 [F{Ga(dcpe)}$_2$($COOCH_2CHO$)][*pf*]$_2$

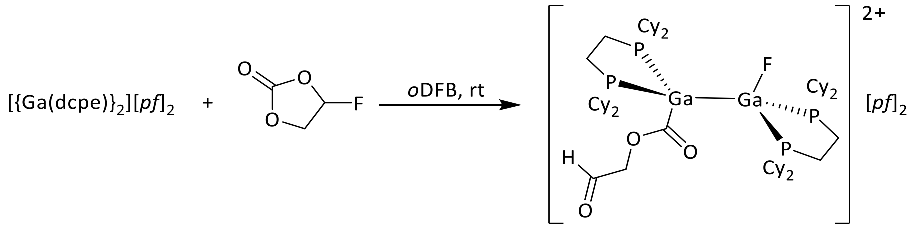

Scheme S 11: Synthesis of [F{Ga(dcpe)}$_2$($COOCH_2CHO$)][*pf*]$_2$.

[Ga(PhF)$_x$][*pf*] (for x = 2.0: 50 mg, 40.7 µmol, 1.0 eq.) and dcpe (17.2 mg, 40.7 µmol, 1.0 eq.) were mixed in *o*DFB (ca. 0.6 ml). A stock solution of FEC in *o*DFB (0.1 mL, c = 0.225 M, 22.5 µmol, 0.55 eq.) was added. The colourless reaction solution was analyzed *via* NMR spectroscopy and the structure of the main product was determined unambiguously. Layering the reaction solution with *n*-pentane (ca. 8 mL) did not yield crystals suitable for sc-XRD (the yield was not determined).

2.13 [F{Ga(dcpe)}$_2$($C_6F_2H_3$)][*pf*]$_{5/3}$[*alfal*]$_{1/3}$

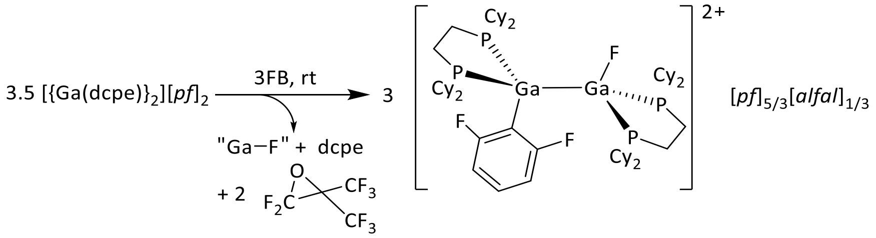

Scheme S 12: Synthesis of [F{Ga(dcpe)}$_2$($C_6F_2H_3$)][*pf*]$_{5/3}$[*alfal*]$_{1/3}$.

[Ga(PhF)$_x$][*pf*] (for x = 1.0: 40 mg, 32.5 µmol, 1.0 eq.) and dcpe (13.5 mg, 31.9 µmol, 1.0 eq.) were mixed in 3FB (ca. 0.8 ml). The red reaction solution was stirred for 4 days at 40 °C. The reaction is best performed under the exclusion of light. This observation may indicate that the formation of the encounter complex is impeded by the irradiation of light, in analogy to the well-known displacement of arene ligands in transition metal complexes.[32]

Layering the then colourless solution with *n*-pentane (ca. 8 mL), filtration and drying the filter residue in *vacuo* (1×10^{-3} mbar) yielded colorless crystals of [F{Ga(dcpe)}$_2$($C_6F_2H_3$)][*pf*]$_{5/3}$[*alfal*]$_{1/3}$ (33.5 mg, 10.4 µmol, 76 %).

3 Crystal Structures

Table S 1: Crystal data and summary of the data collection and refinement for [{Ga(dcpe)}$_2$(C_6H_{10})][*pf*]$_2$ *C_6H_{10} and [H{Ga(dcpe)}$_2$(OEt)][*pf*]$_2$.

CCDC	-	2488257
Empirical formula	$C_{96}H_{116}Al_2F_{72}Ga_2O_8P_4$	$C_{86}H_{102}Al_2F_{72}Ga_2O_9P_4$
Formula weight	3083.16	2964.95
Temperature [K]	100(2)	100(2)
Crystal system	triclinic	triclinic
Space group (number)	$P\bar{1}$ (2)	$P\bar{1}$ (2)
a [Å]	14.919(4)	17.168(4)
b [Å]	14.959(4)	17.984(5)
c [Å]	29.752(9)	22.621(8)
α [°]	97.393(8)	69.090(8)
β [°]	102.118(11)	67.933(9)
γ [°]	98.894(11)	63.812(8)
Volume [Å^3]	6324(3)	5653(3)
Z	2	2
ρ_{calc} [gcm^{-3}]	1.619	1.742
μ [mm^{-1}]	0.647	0.720
F(000)	3104	2972
Crystal size [mm^3]	0.168×0.254×0.309	0.202×0.248×0.4413
Crystal colour	yellow	colourless
Crystal shape	block	plate
Radiation	MoK_α (λ=0.71073 Å)	MoK_α (λ=0.71073 Å)
2θ range [°]	2.79 to 55.50 (0.76 Å)	2.59 to 54.37 (0.78 Å)
Index ranges	−19 ≤ h ≤ 18<br>−19 ≤ k ≤ 19<br>0 ≤ l ≤ 38	−22 ≤ h ≤ 22<br>−23 ≤ k ≤ 23<br>−29 ≤ l ≤ 29
Reflections collected	27494	217054
Independent reflections	27494<br>R_{int} = 0.1315<br>R_{sigma} = 0.1554	25055<br>R_{int} = 0.0603<br>R_{sigma} = 0.0370
Completeness to θ = 25.242°	93.9 %	100.0 %
Data / Restraints / Parameters	27494 / 47512 / 2867	25055 / 48279 / 2726
Absorption correction T_{min}/T_{max} (method)	0.6174 / 0.7456 (multi-scan)	0.6821 / 0.7455 (multi-scan)
Goodness-of-fit on F^2	1.761	0.874
Final *R* indexes [I≥2σ(I)]	R_1 = 0.1462<br>wR_2 = 0.4157	R_1 = 0.0676<br>wR_2 = 0.1788
Final *R* indexes [all data]	R_1 = 0.1895<br>wR_2 = 0.4303	R_1 = 0.0952<br>wR_2 = 0.2133
Largest peak/hole [eÅ^{-3}]	2.30/−1.63	2.27/−0.99

Table S 2: Crystal data and summary of the data collection and refinement for [H{Ga(dcpe)}$_2$(OPh)][*pf*]$_2$, [H{Ga(dcpe)}$_2$(NHPh)][*pf*]$_2$ and [H{Ga(dcpe)}$_2$(NPh$_2$)][*pf*]$_2$.

CCDC	2488258	2488259	2488260
Empirical formula	$C_{90}H_{102}Al_2F_{72}Ga_2O_9P_4$	$C_{90}H_{103}Al_2F_{72}Ga_2NO_8P_4$	$C_{96}H_{107}Al_2F_{72}Ga_2NO_8P_4$
Formula weight	3012.99	3012.01	3088.10
Temperature [K]	100(2)	100(2)	100(2)
Crystal system	orthorhombic	monoclinic	triclinic
Space group (number)	$P2_12_12_1$ (19)	Cc (9)	$P\bar{1}$ (2)
a [Å]	15.677(2)	18.260(3)	15.468(5)
b [Å]	23.421(4)	29.497(5)	18.034(4)
c [Å]	31.572(5)	22.081(4)	22.835(5)
α [°]	90	90	81.311(12)
β [°]	90	96.514(9)	72.120(5)
γ [°]	90	90	86.298(8)
Volume [Å^3]	11592(3)	11816(4)	5992(2)
Z	4	4	2
ρ_{calc} [gcm^{-3}]	1.726	1.693	1.712
μ [mm^{-1}]	0.704	0.690	0.683
F(000)	6040	6040	3100
Crystal size [mm^3]	0.118×0.134×0.419	0.200×0.210×0.369	0.214×0.263×0.44
Crystal colour	colourless	colourless	colourless
Crystal shape	block	plate	block
Radiation	MoK_α (λ=0.71073 Å)	MoK_α (λ=0.71073 Å)	MoK_α (λ=0.71073 Å)
2θ range [°]	2.90 to 55.07 (0.77 Å)	2.64 to 56.76 (0.75 Å)	2.77 to 55.18 (0.77 Å)
Index ranges	−20 ≤ h ≤ 20<br>−30 ≤ k ≤ 30<br>−41 ≤ l ≤ 41	−24 ≤ h ≤ 24<br>−39 ≤ k ≤ 39<br>−29 ≤ l ≤ 29	−20 ≤ h ≤ 20<br>−23 ≤ k ≤ 23<br>−29 ≤ l ≤ 29
Reflections collected	553135	473329	412837
Independent reflections	26698<br>R_{int} = 0.0617<br>R_{sigma} = 0.0237	29446<br>R_{int} = 0.0467<br>R_{sigma} = 0.0233	27702<br>R_{int} = 0.0503<br>R_{sigma} = 0.0211
Completeness to θ = 25.242°	99.9 %	100.0 %	100.0 %
Data / Restraints / Parameters	26698 / 33678 / 2379	29446 / 33743 / 2382	27702 / 24108 / 2160
Absorption correction T_{min}/T_{max} (method)	0.6901 / 0.7456 (multi-scan)	0.6743 / 0.7457 (multi-scan)	0.6343 / 0.7456 (multi-scan)
Goodness-of-fit on F^2	1.018	1.039	1.060
Final *R* indexes [I≥2σ(I)]	R_1 = 0.0487<br>wR_2 = 0.1271	R_1 = 0.0291<br>wR_2 = 0.0786	R_1 = 0.0447<br>wR_2 = 0.1171
Final *R* indexes [all data]	R_1 = 0.0527<br>wR_2 = 0.1314	R_1 = 0.0302<br>wR_2 = 0.0798	R_1 = 0.0526<br>wR_2 = 0.1255
Largest peak/hole [eÅ^{-3}]	0.81/−0.52	0.68/−0.58	2.58/−0.57
Flack X parameter	0.017(9)	0.047(5)	-

Table S 3: Crystal data and summary of the data collection and refinement for [H{Ga(dcpe)}$_2$(CH$_2$CN)][*pf*]$_2$ and [F{Ga(dcpe)}$_2$(C$_6$F$_2$H$_3$)][*pf*]$_{5/3}$[*alfal*]$_{1/3}$.

CCDC	2488261	2490061
Empirical formula	$C_{86}H_{99}Al_2F_{72}Ga_2NO_8P_4$	$C_{278}H_{297}Al_7F_{244}Ga_6O_{26}P_{12}$
Formula weight	2959.94	9668.99
Temperature [K]	100(2)	100(2)
Crystal system	monoclinic	triclinic
Space group (number)	$P2_1$ (4)	$P\overline{1}$ (2)
a [Å]	13.550(4)	18.462(5)
b [Å]	27.099(5)	27.562(6)
c [Å]	15.504(4)	37.435(9)
α [°]	90	86.961(6)
β [°]	91.200(7)	88.862(7)
γ [°]	90	75.387(10)
Volume [Å^3]	5692(3)	18406(8)
Z	2	2
ρ_{calc} [gcm^{-3}]	1.727	1.745
μ [mm^{-1}]	0.715	0.684
F(000)	2964	9652
Crystal size [mm^3]	0.319×0.366×0.477	0.210×0.322×0.389
Crystal colour	colourless	colourless
Crystal shape	block	block
Radiation	MoK_α (λ=0.71073 Å)	MoK_α (λ=0.71073 Å)
2θ range [°]	2.63 to 55.26 (0.77 Å)	2.53 to 55.26 (0.77 Å)
Index ranges	$-17 \le h \le 17$<br>$-35 \le k \le 35$<br>$-20 \le l \le 20$	$-24 \le h \le 24$<br>$-35 \le k \le 35$<br>$-48 \le l \le 48$
Reflections collected	218575	1009603
Independent reflections	26263<br>R_{int} = 0.0611<br>R_{sigma} = 0.0402	85023<br>R_{int} = 0.0663<br>R_{sigma} = 0.0343
Completeness to θ = 25.242°	99.8 %	99.9 %
Data / Restraints / Parameters	26263 / 44139 / 3022	85023 / 328226 / 8698
Absorption correction T_{min}/T_{max} (method)	0.6500 / 0.7084 (multi-scan)	0.6849 / 0.7456 (multi-scan)
Goodness-of-fit on F^2	1.044	1.100
Final *R* indexes [$I \ge 2\sigma(I)$]	R_1 = 0.0889<br>wR_2 = 0.2511	R_1 = 0.0990<br>wR_2 = 0.2598
Final *R* indexes [all data]	R_1 = 0.1063<br>wR_2 = 0.2781	R_1 = 0.1243<br>wR_2 = 0.2809
Largest peak/hole [eÅ^{-3}]	1.00/−0.64	1.45/−1.09
Flack X parameter	0.12(2)	

3.1 [{Ga(dcpe)}$_2$(C_6H_{10})][*pf*]$_2$ * C_6H_{10}

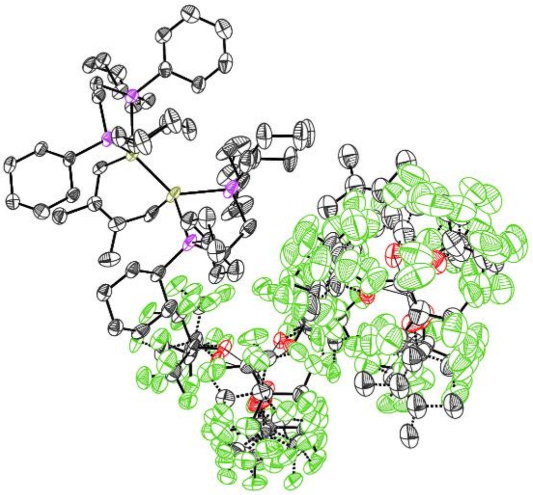

Figure S 3: Molecular structure of [{Ga(dcpe)}$_2$(C_6H_{10})][*pf*]$_2$. * C_6H_{10}. Hydrogen atoms are omitted for clarity. The disordered anion fragments are shown. Thermal ellipsoids are set at 50 % probability level.

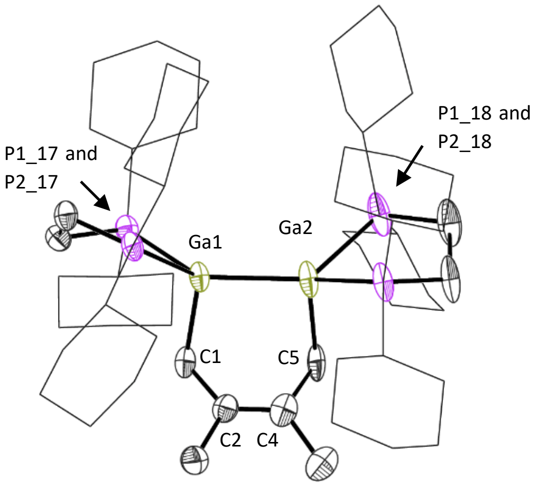

Figure S 4: Molecular structure of [H{Ga(dcpe)}$_2$(C_6H_{10})]$^{2+}$ in [{Ga(dcpe)}$_2$(C_6H_{10})][*pf*]$_2$. * C_6H_{10}. Hydrogen atoms are omitted and the cyclohexyl groups are depicted in wireframe for simplicity. Thermal ellipsoids are set at 50 % probability level.

Table S 4: Selected bond lengths in [{Ga(dcpe)}$_2$(C_6H_{10})][*pf*]$_2$. * C_6H_{10}.

Atom–Atom	Bond Length [pm]
Ga1–C1	201.5(6)
Ga1–P1_17	242.18(19)
Ga1–P2_17	243.6(2)
Ga1–Ga2	243.90(11)
Ga2–C5	203.9(7)
Ga2–P2_18	242.2(2)
Ga2–P1_18	244.0(2)
C2–C1	1.500(9)
C2–C4	1.314(11)
C4–C5	1.510(10)

It has to be noted that the compound crystallizes in a superstructure, as previously observed for cycloaddition products of [{Ga(dcpe)}$_2$][*pf*]$_2$ with an alkene and an alkyne. Thus, the scXRD data were not uploaded in the CCDC data base and only allow to show atom connectivities. The bond lengths in **Table S 4** are only shown to underline the good agreement with the computed values and with the Ga–Ga and Ga–P bond lengths in the other crystalline compounds presented herein. Interestingly, the determined C1–C2 and C4–C5 bond lengths are not only in good agreement with the calculated distances (ca. 150 pm) but are also significantly longer than the C2–C4 distance (calculated bond length: 136 pm). This strongly implies that the cycloaddition leads to the formation of a C=C double bond between C2 and C4 and a concomitant formation of C1–C2 and C4–C5 single bonds, which is expected for the product of a hetero-Diels-Alder reaction.

3.2 [H{Ga(dcpe)}$_2$(OEt)][*pf*]$_2$

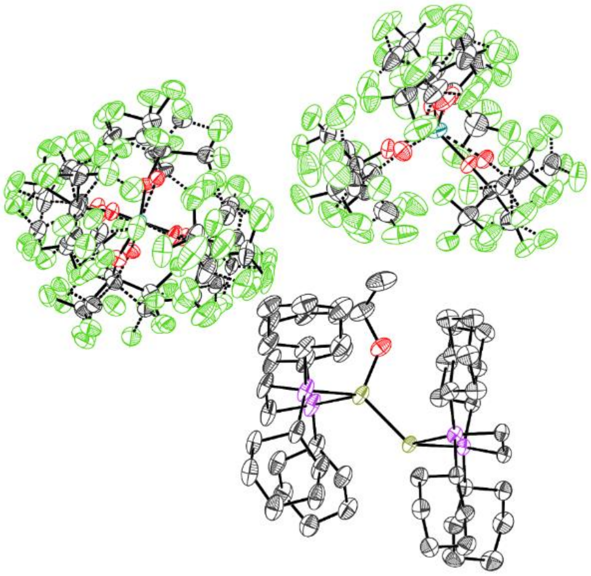

Figure S 5: Molecular structure of the asymmetric unit of [H{Ga(dcpe)}$_2$(OEt)][*pf*]$_2$ in the solid state from scXRD. Hydrogen atoms are omitted for the sake of clarity. Disordered anion fragments are shown. Thermal ellipsoids are set at 50 % probability level.

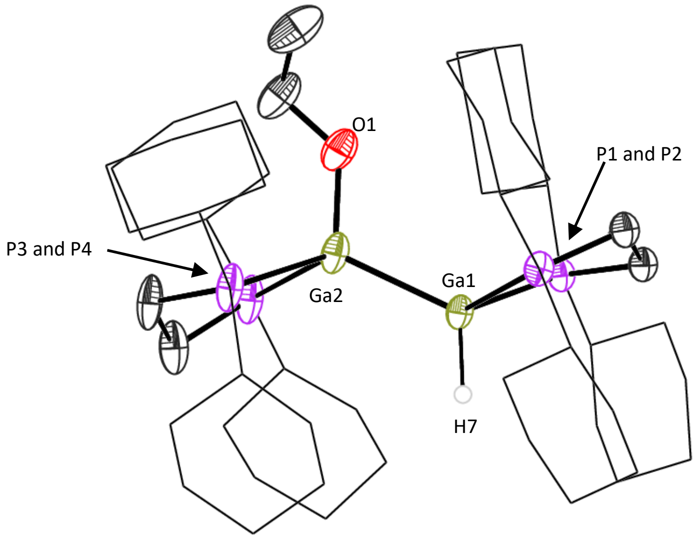

Figure S 6: Molecular structure of [H{Ga(dcpe)}$_2$(OEt)]$^{2+}$ in [H{Ga(dcpe)}$_2$(OEt)][*pf*]$_2$. Hydrogen atoms are omitted (except for the hydrogen atom bound to Ga) and the cyclohexyl groups are depicted in wireframe for simplicity. Thermal ellipsoids are set at 50 % probability level.

Table S 5: Selected bond lengths in [H{Ga(dcpe)}$_2$(OEt)][*pf*]$_2$.

Atom–Atom	Bond Length [pm]
Ga1–P2	241.57(12)
Ga1–P1	241.70(11)
Ga1–Ga2	243.35(9)
Ga1–H7	147(4)
Ga2–O1	181.5(4)
Ga2–P3	247.82(13)
Ga2–P4	247.88(13)

3.3 [H{Ga(dcpe)}$_2$(OPh)][*pf*]$_2$

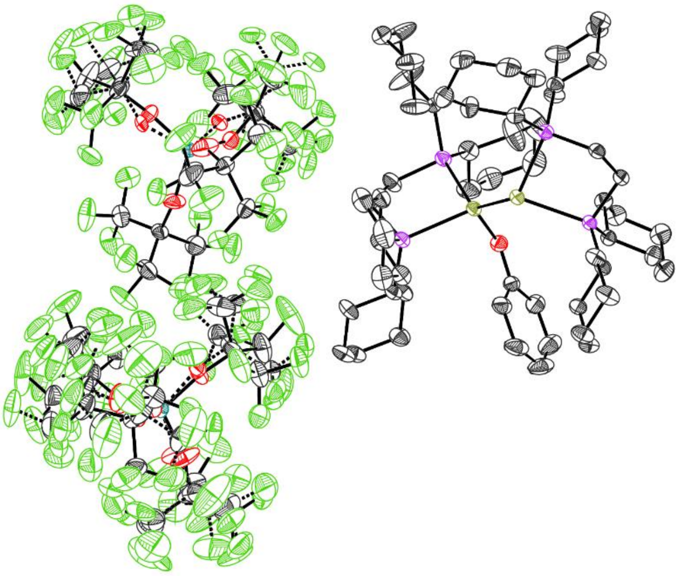

Figure S 7: Molecular structure of the asymmetric unit of [H{Ga(dcpe)}$_2$(OPh)][*pf*]$_2$ in the solid state from scXRD. Hydrogen atoms are omitted for the sake of clarity. Disordered anion fragments are shown. Thermal ellipsoids are set at 50 % probability level.

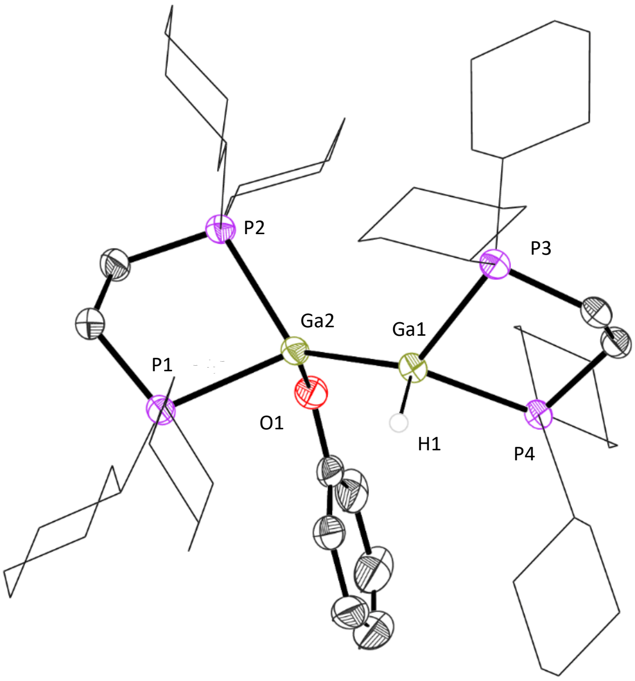

Figure S 8: Molecular structure of [H{Ga(dcpe)}$_2$(OPh)]$^{2+}$ in [H{Ga(dcpe)}$_2$(OPh)][*pf*]$_2$. Hydrogen atoms are omitted (except for the hydrogen atom bound to Ga) and the cyclohexyl groups are depicted in wireframe for simplicity. Thermal ellipsoids are set at 50 % probability level.

Table S 6: Selected bond lengths in [H{Ga(dcpe)}$_2$(OPh)][*pf*]$_2$.

Atom–Atom	Bond Length [pm]
Ga1–P3	241.81(13)
Ga1–P4	242.64(12)
Ga1–Ga2	246.28(7)
Ga1–H1	143(6)
Ga2–O1	185.9(4)
Ga2–P2	242.83(13)
Ga2–P1	247.39(12)

3.4 [H{Ga(dcpe)}$_2$(NHPh)][*pf*]$_2$

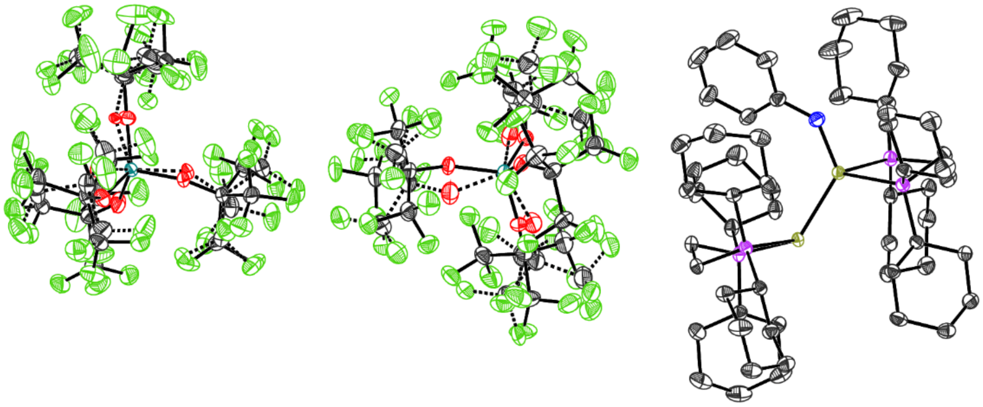

Figure S 9: Molecular structure of the asymmetric unit of [H{Ga(dcpe)}$_2$(NHPh)][*pf*]$_2$ in the solid state from scXRD. Hydrogen atoms are omitted for the sake of clarity. Disordered anion fragments are shown. Thermal ellipsoids are set at 50 % probability level.

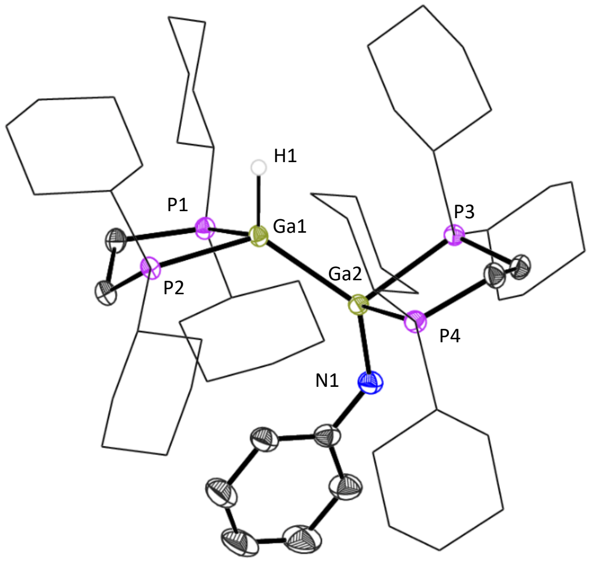

Figure S 10: Molecular structure of $[H\{Ga(dcpe)\}_2(NHPh)]^{2+}$ in $[H\{Ga(dcpe)\}_2(NHPh)][pf]_2$. Hydrogen atoms are omitted (except for the hydrogen atom bound to Ga) and the cyclohexyl groups are depicted in wireframe for simplicity. Thermal ellipsoids are set at 50 % probability level.

Table S 7: Selected bond lengths in $[H\{Ga(dcpe)\}_2(NHPh)][pf]_2$.

Atom–Atom	Bond Length [pm]
Ga1–P2	240.78(8)
Ga1–P1	241.16(8)
Ga1–Ga2	243.21(5)
Ga1–H1	149(6)
Ga2–N1	187.1(2)
Ga2–P3	242.68(8)
Ga2–P4	246.97(8)

3.5 [H{Ga(dcpe)}$_2$(NPh$_2$)][*pf*]$_2$

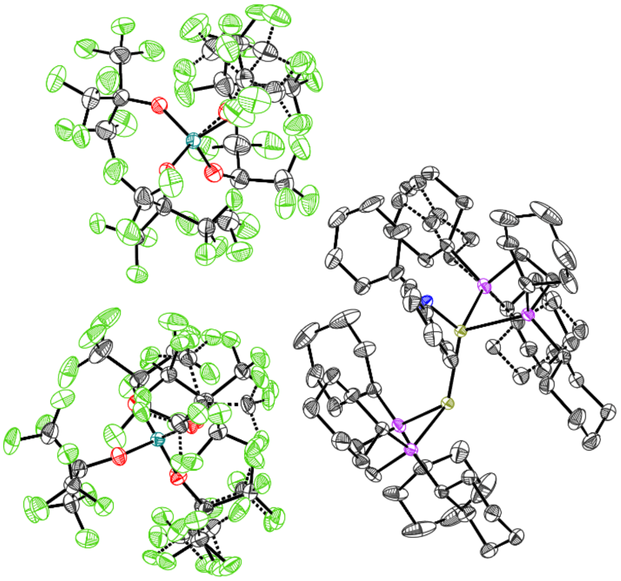

Figure S 11: Molecular structure of the asymmetric unit of [H{Ga(dcpe)}$_2$(NPh$_2$)][*pf*]$_2$ in the solid state from scXRD. Hydrogen atoms are omitted for the sake of clarity. Disordered anion fragments are shown. Thermal ellipsoids are set at 50 % probability level.

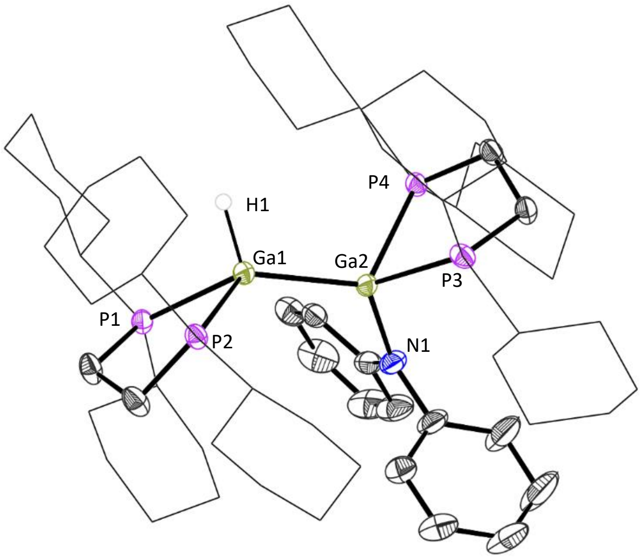

Figure S 12: Molecular structure of [H{Ga(dcpe)}$_2$(NPh$_2$)]$^{2+}$ in [H{Ga(dcpe)}$_2$(NPh$_2$)][*pf*]$_2$. Hydrogen atoms are omitted (except for the hydrogen atom bound to Ga) and the cyclohexyl groups are depicted in wireframe for simplicity. Thermal ellipsoids are set at 50 % probability level.

Table S 8: Selected bond lengths in [H{Ga(dcpe)}$_2$(NPh$_2$)][*pf*]$_2$.

Atom–Atom	Bond Length [pm]
Ga1–P2	244.37(7)
Ga1–P1	245.72(8)
Ga1–Ga2	248.53(6)
Ga1–H1	144(3)
Ga2–N1	191.9(2)
Ga2–P3	247.32(9)
Ga2–P4	250.50(7)

3.6 [H{Ga(dcpe)}$_2$(CH$_2$CN)][*pf*]$_2$

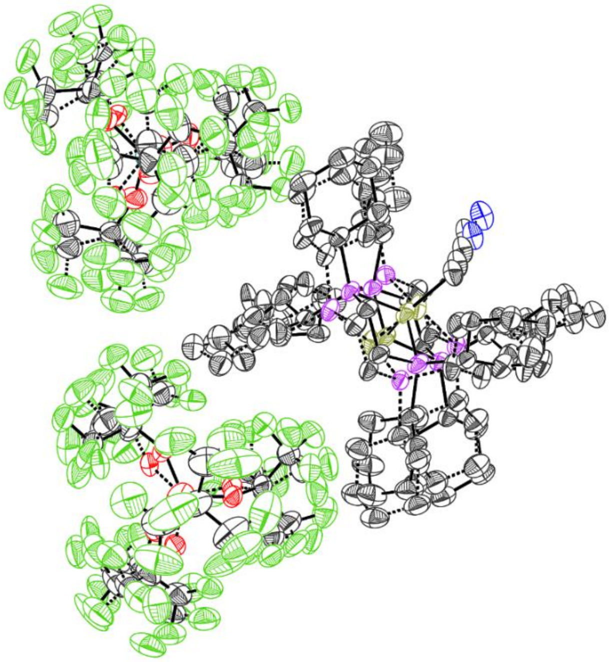

Figure S 13: Molecular structure of the asymmetric unit of [H{Ga(dcpe)}$_2$(CH$_2$CN)][*pf*]$_2$ in the solid state from scXRD. Hydrogen atoms are omitted for the sake of clarity. Disordered ligand and anion fragments are shown. Thermal ellipsoids are set at 50 % probability level.

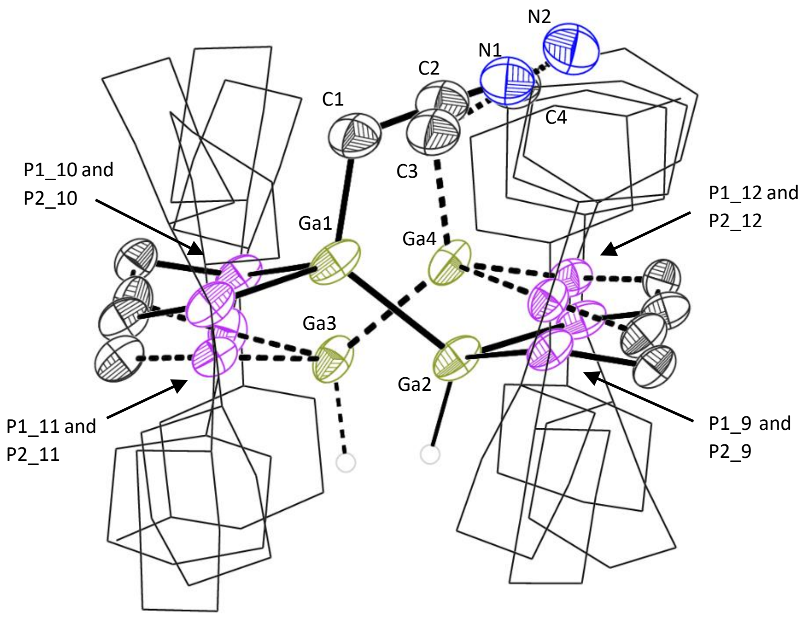

Figure S 14: Molecular structure of [H{Ga(dcpe)}$_2$(CH$_2$CN)]$^{2+}$ in [H{Ga(dcpe)}$_2$(CH$_2$CN)][*pf*]$_2$. Hydrogen atoms are omitted (except for the hydrogen atoms bound to Ga) and the cyclohexyl groups are depicted in wireframe for simplicity. Disordered ligand fragments are also shown. Thermal ellipsoids are set at 50 % probability level.

Table S 9: Selected bond lengths in [H{Ga(dcpe)}$_2$(CH$_2$CN)][*pf*]$_2$.

Atom–Atom	Bond Length [pm]
Ga1–C1	205.4(16)
Ga1–Ga2	244.64(18)
Ga1–P2_10	243.7(4)
Ga1–P1_10	246.4(5)
Ga2–P2_9	241.5(4)
Ga2–P1_9	243.5(5)
Ga4–C3	208(2)
Ga4–Ga3	244.1(3)
Ga3–P2_11	241.7(5)
Ga3–P1_11	242.6(4)
Ga4–P2_12	242.0(5)
Ga4–P1_12	244.8(5)

It has to be noted that the quality of the scXRD data only allow to show atom connectivities, and not to discuss bond lengths. The bond lengths in **Table S 9Table S 4** are only shown to underline the good agreement with the computed values and with the Ga–Ga, Ga–P and Ga–C bond lengths in the other crystalline compounds presented herein. Since the Ga–H distance cannot be determined reliably, the bond distance is not included in **Table S 9**. However, the Ga–**H** signal in the ^{1}H NMR spectrum as well as the Ga–H band in the IR spectrum unequivocally confirm the presence of this proton in the reaction product.

3.7 [F{Ga(dcpe)}$_2$(CH$_2$CN)][*pf*]$_2$

A structural model for the crystals obtained upon layering a mixture of [{Ga(dcpe)}$_2$][*pf*]$_2$ and FH$_2$C–CN in oDFB with *n*-pentane is shown in **Figure S 15**. Unfortunately, the quality of the scXRD data (R_1 = 0.1241; wR_2 = 0.4161; reflection/parameter ration = 4.78) does not allow to upload the structural model in the CCDC database. The handling of the data (space group: $P2_1/n$) is complicated by the fact that the whole cation is disordered on a special position, i.e. the surface centre of the unit cell (**Figure S 16** and **Figure S 17**).

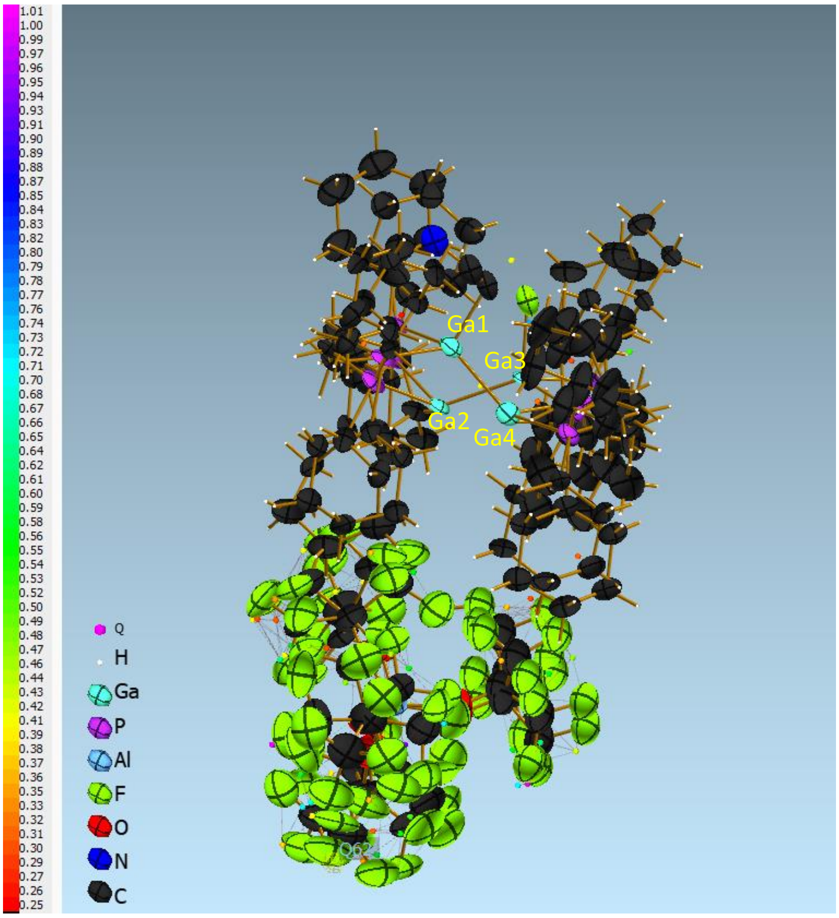

Figure S 15: Structural model for the crystals obtained upon layering a mixture of [{Ga(dcpe)}$_2$][*pf*]$_2$ and FH_2C–CN in *o*DFB with *n*-pentane.

However, it is reasonable to assume that the positions of the heavy, electron rich gallium atoms within the unit cell can be determined reliably, as well as the Ga–Ga distances. The main question that arises when assigning the disordered fragments is, whether Ga1 is bound to Ga4 or to Ga3. The bond distances Ga1–Ga3 and Ga2–Ga4 are unreasonably short (213.6 and 191.1 pm), considering that the Ga–Ga bond length typically ranges from 235–255 pm[33] in elemental gallium and organometallic gallium compounds. Thus, in the structural model presented in **Figure S *15***, Ga1 has to be bound to Ga4 and Ga2 has to be bound to Ga3 (bond lengths: 242.2 and 243.8 pm). This is an important finding, since the “zigzag” arrangement of the central “P_2GaGaP_2“-unit is not consistent with the structure of a [2+2] cycloaddition product.

As a consequence, the scXRD data of the crystaline product of the reaction between [{Ga(dcpe)}$_2$]$^{2+}$ and FH_2C–CN are more in line with the *anti*-periplanar C–F activation product, which is also confirmed by NMR spectroscopy (section 4.12).

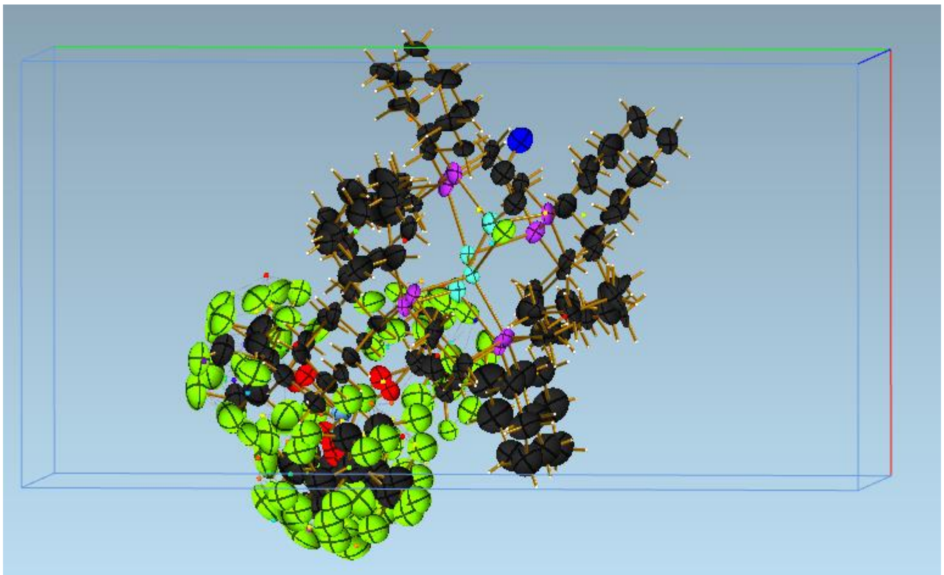

Figure S 16: Structural model, and unit cell, for the crystals obtained upon layering a mixture of [{Ga(dcpe)}$_2$][*pf*]$_2$ and FH_2C–CN in *o*DFB with *n*-pentane.

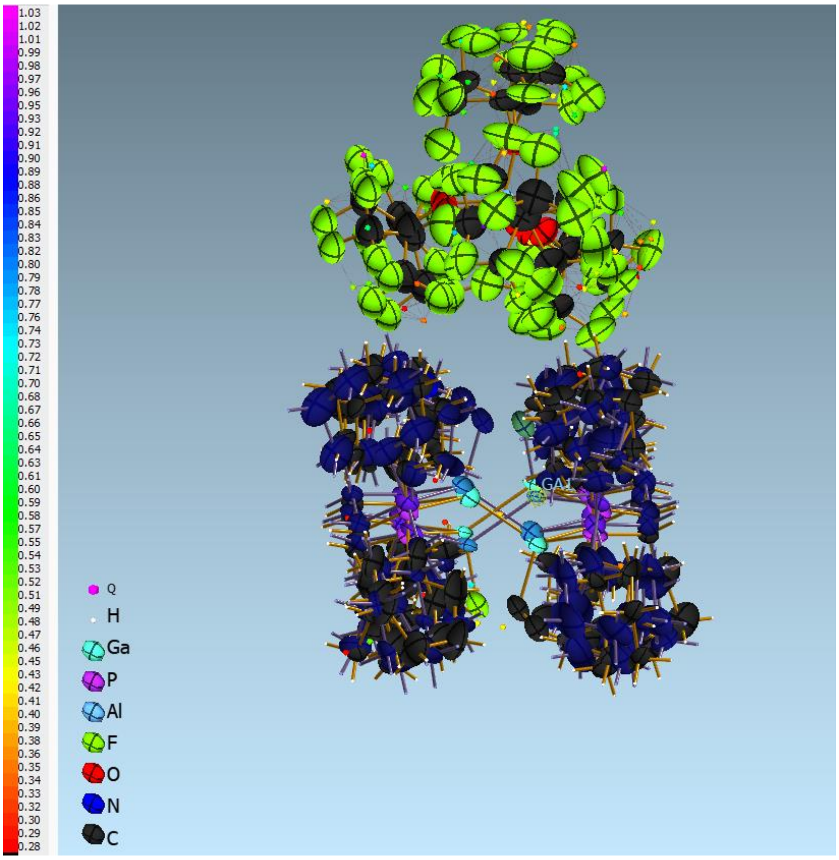

Figure S 17: Structural model for the crystals obtained upon layering a mixture of [{Ga(dcpe)}$_2$][*pf*]$_2$ and FH_2C–CN in *o*DFB with *n*-pentane. The disordered and symmetry-generated cation fragments are shown.

3.8 [F{Ga(dcpe)}$_2$(C$_6$F$_2$H$_3$)][*pf*]$_{5/3}$[*alfal*]$_{1/3}$

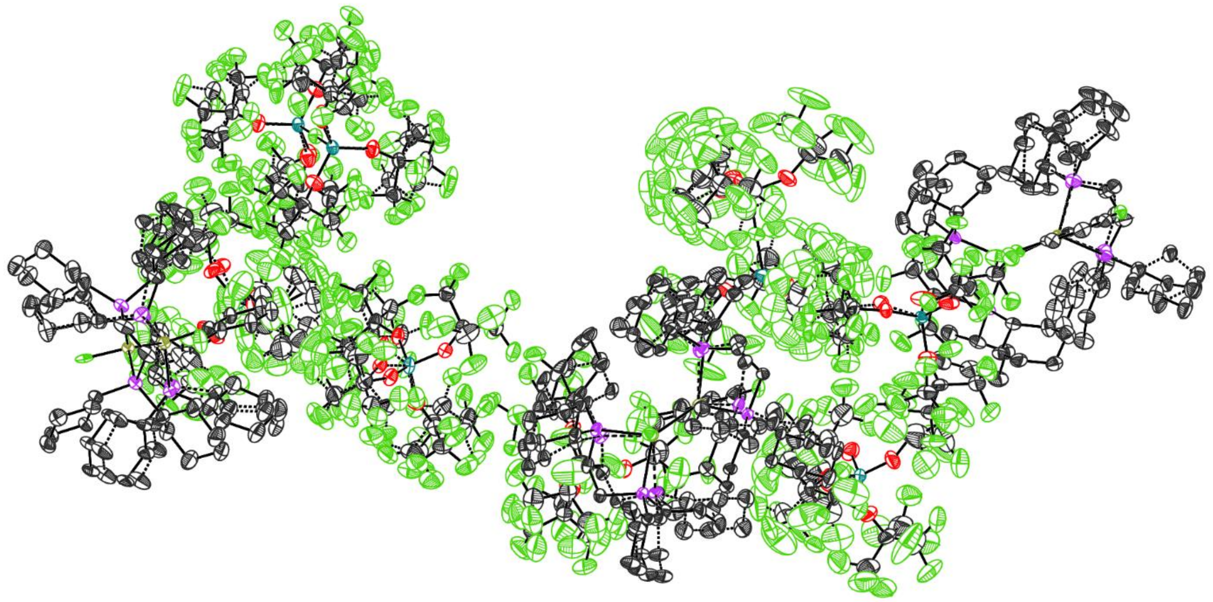

Figure S 18: Molecular structure of the asymmetric unit of [F{Ga(dcpe)}$_2$(C$_6$F$_2$H$_3$)][*pf*]$_{5/3}$[*alfal*]$_{1/3}$ in the solid state from scXRD. Hydrogen atoms are omitted for the sake of clarity. The three crystallographically independent [F{Ga(dcpe)}$_2$(C$_6$F$_2$H$_3$)]$^{2+}$ units are shown, as well as disordered ligand and anion fragments. The second [Al{O(CF$_3$)$_3$}$_3$] fragments of the two [*alfal*]$^-$ anions are symmetry-generated (by an inversion operation). Thermal ellipsoids are set at 50 % probability level

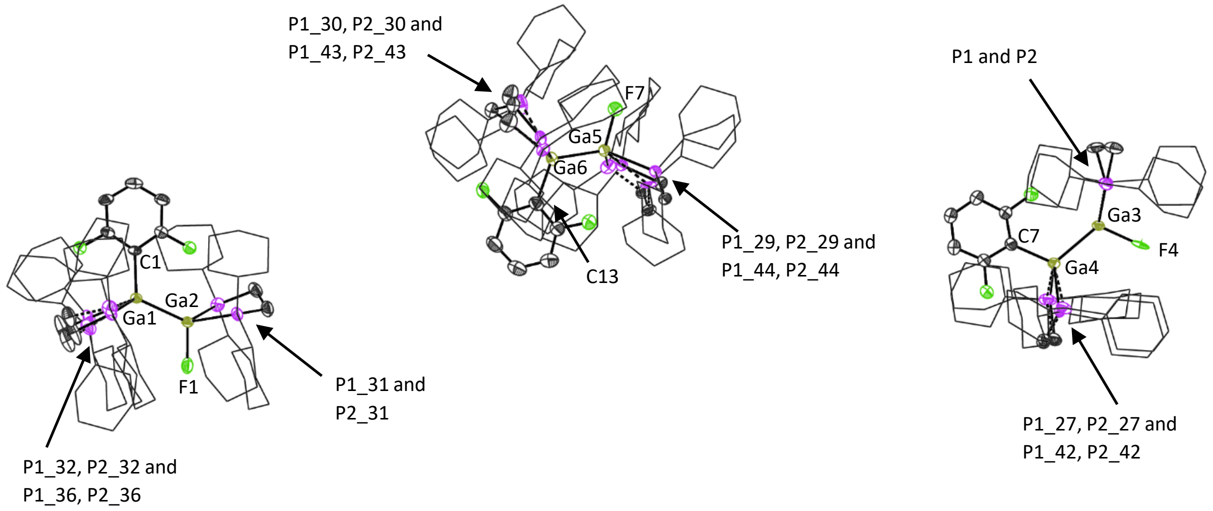

Figure S 19: Molecular structure of the three crystallographically independent [F{Ga(dcpe)}$_2$(C$_6$F$_2$H$_3$)]$^{2+}$ fragments in [F{Ga(dcpe)}$_2$(C$_6$F$_2$H$_3$)][*pf*]$_{5/3}$[*alfal*]$_{1/3}$. Hydrogen atoms are omitted and the cyclohexyl groups are depicted in wireframe for simplicity. Disordered ligand fragments are also shown. Thermal ellipsoids are set at 50 % probability level.

Table S 10: Selected bond lengths in [F{Ga(dcpe)}$_2$($C_6F_2H_3$)][*pf*]$_{5/3}$[*alfal*]$_{1/3}$.

Atom–Atom	Bond Length [pm]
Ga1–C1	200.8(5)
Ga1–P1_32	243.4(3)
Ga1–P2_32	244.2(3)
Ga1–Ga2	245.83(9)
Ga1–P2_36	247(3)
Ga1–P1_36	254(3)
Ga2–F1	185.0(4)
Ga2–P1_31	245.00(16)
Ga2–P2_31	246.28(16)
Ga3–F4	195.0(4)
Ga3–P1	243.37(15)
Ga3–Ga4	245.41(10)
Ga3–P2	247.51(16)
Ga4–C7	199.6(5)
Ga4–P2_42	236.8(15)
Ga4–P1_42	240.5(13)
Ga4–P2_27	241.6(10)
Ga4–P1_27	251.1(11)
Ga5–F7	180.9(4)
Ga5–P1_44	239.6(15)
Ga5–P2_29	242.87(18)
Ga5–Ga6	244.38(9)
Ga5–P2_44	245(2)
Ga5–P1_29	247.6(2)
Ga6–C13	201.0(6)
Ga6–P2_43	230(2)
Ga6–P1_30	241.22(17)
Ga6–P2_30	245.6(2)
Ga6–P1_43	266.2(15)

4 NMR Spectroscopy

4.1 NMR Investigations of [2+2] Cycloaddition Reactions with $[\{Ga(dcpe)\}_2]^{2+}$

4.1.1 Reaction with *trans*-β-deutero-styrene

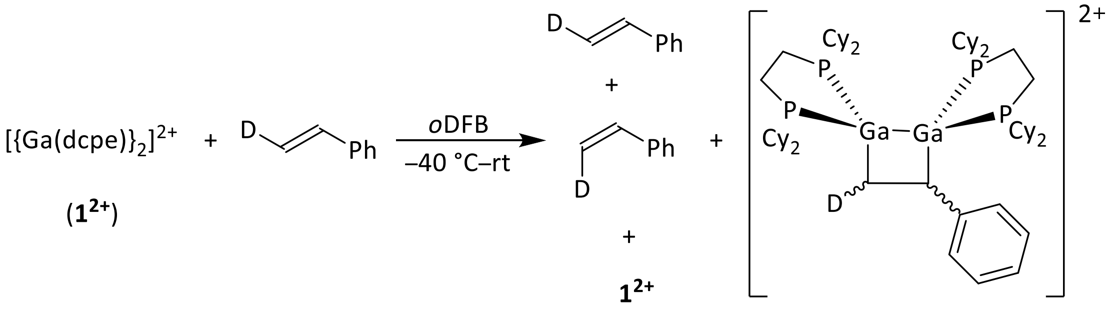

Scheme S 13: Reaction of $[\{Ga(dcpe)\}_2][pf]_2$ ($[\mathbf{1}][pf]_2$) and *trans*-β-deutero-styrene in *o*DFB at different temperatures. The $[pf]^-$ anions are omitted for clarity. Note that the [2+2] cycloaddition products are only formed very slowly below 0 °C.

The NMR spectra in **Figure S 20**–**Figure S 26** show that *trans*-β-deutero-styrene is converted to a 1:1 mixture of the *trans* and *cis* isomers in the presence of $\mathbf{1}^{2+}$ in an *o*DFB solution, already at 233 K (**Figure S 22**). At this temperature, only the isomerization takes place and no [2+2] cycloaddition products are formed (**Figure S 24**). However, at rt, the formation of the stereoisomeric [2+2] cycloaddition products is indicated by a broad signal at ca. 2.1 ppm in the ^{2}D NMR spectrum (**Figure S 20** and **Figure S 21**) and by the AA'BB' spin system in the $^{31}P\{^{1}H\}$ NMR spectrum (**Figure S 26**).

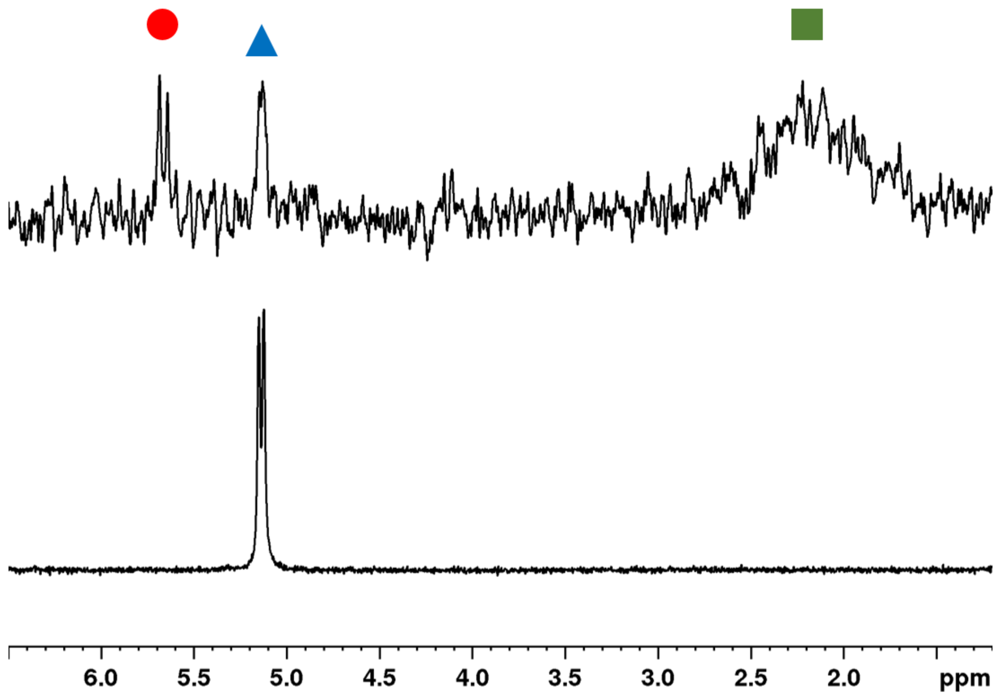

Figure S 20: ^{2}D NMR spectrum of *trans*-β-deutero-styrene in *o*DFB (61.43 MHz, 298 K, bottom) and of a mixture of $[\{Ga(dcpe)\}_2][pf]_2$ and *trans*-β-deutero-styrene in *o*DFB (61.43 MHz, 298 K, top). The ^{2}D NMR signals of the olefinic deuterium atoms of *trans*-β-deutero-styrene (blue triangle), of *cis*-β-deutero-styrene (red point) and of the deuterium atoms in the stereoisomeric [2+2] cycloaddition products (green square) are shown.

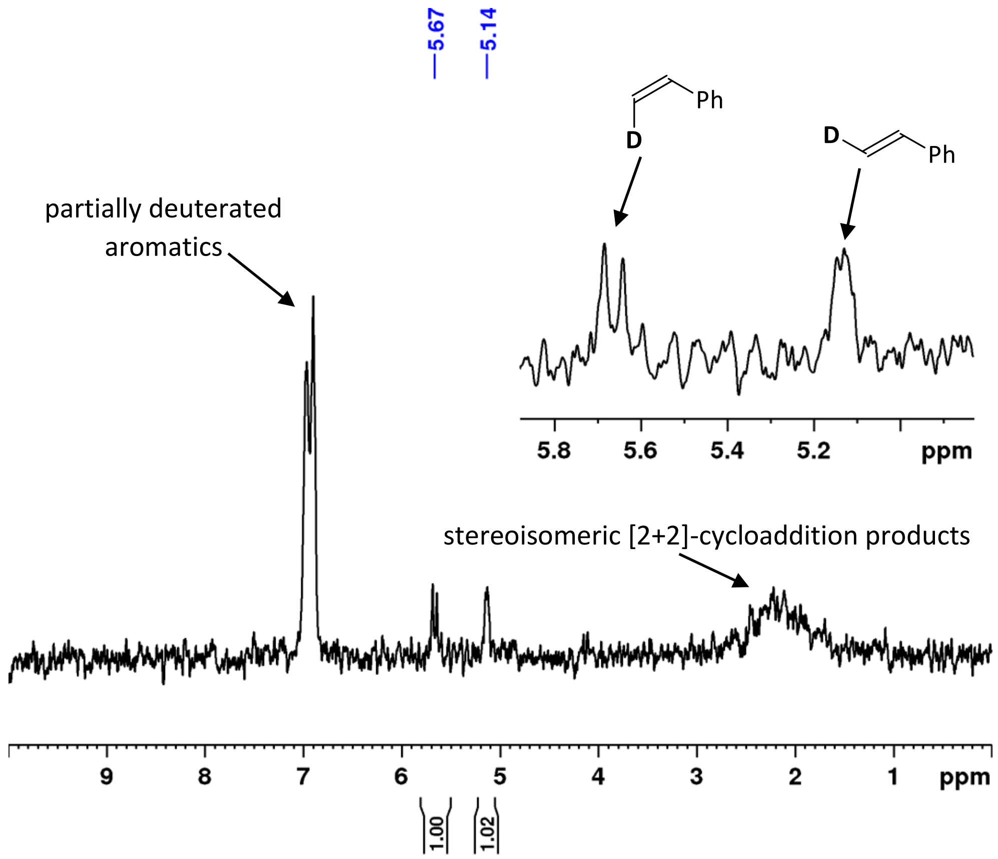

Figure S 21: ^{2}D NMR spectrum of a mixture of [{Ga(dcpe)}$_2$][*pf*]$_2$ and *trans*-β-deutero-styrene in *o*DFB (61.43 MHz, 298 K), with integrated signal intensities for the olefinic deuterium atoms for both products.

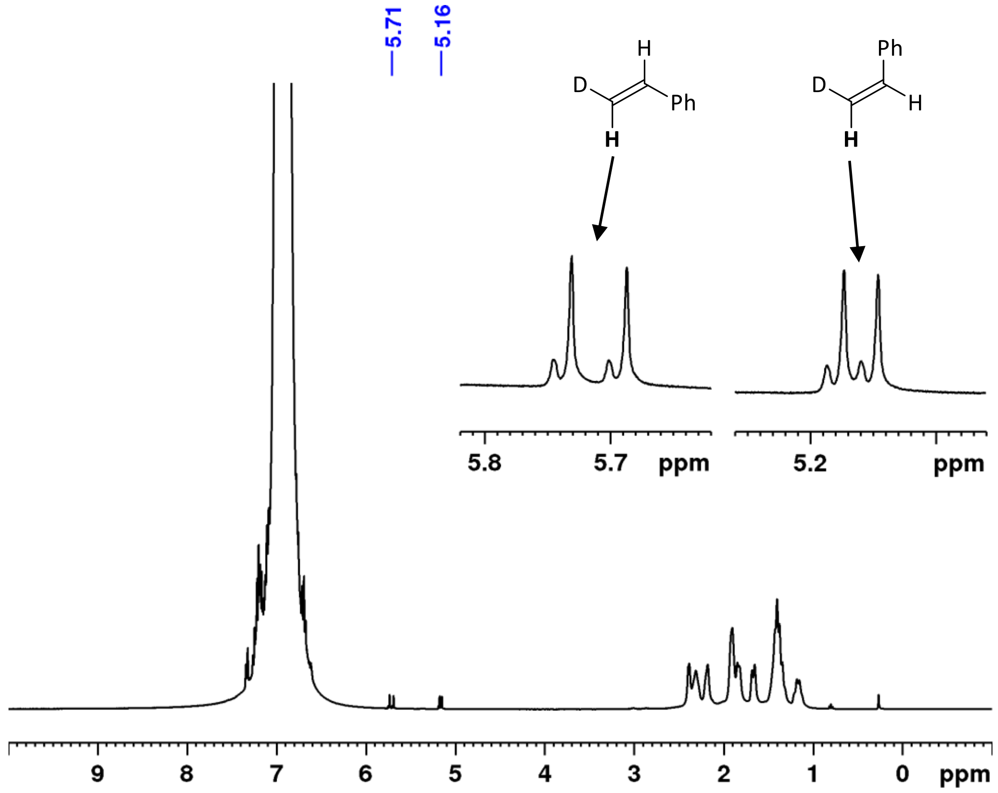

Figure S 22: ^{1}H NMR spectrum (400.17 MHz, 233 K) of a mixture of [{Ga(dcpe)}$_2$][*pf*]$_2$ and *trans*-β-deutero-styrene in *o*DFB, measured directly after mixing the components at low temperatures.

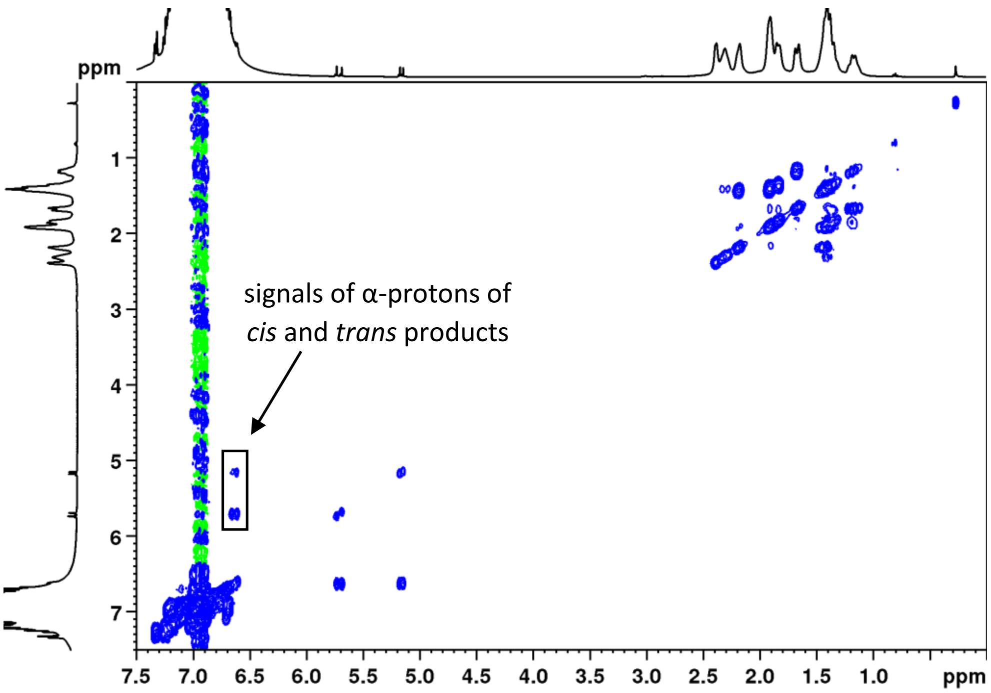

Figure S 23: ^{1}H-COSY NMR spectrum (400.17 MHz, 233 K) of a mixture of [{Ga(dcpe)}$_2$][*pf*]$_2$ and *trans*-β-deutero-styrene in *o*DFB, measured directly after mixing the components at low temperatures.

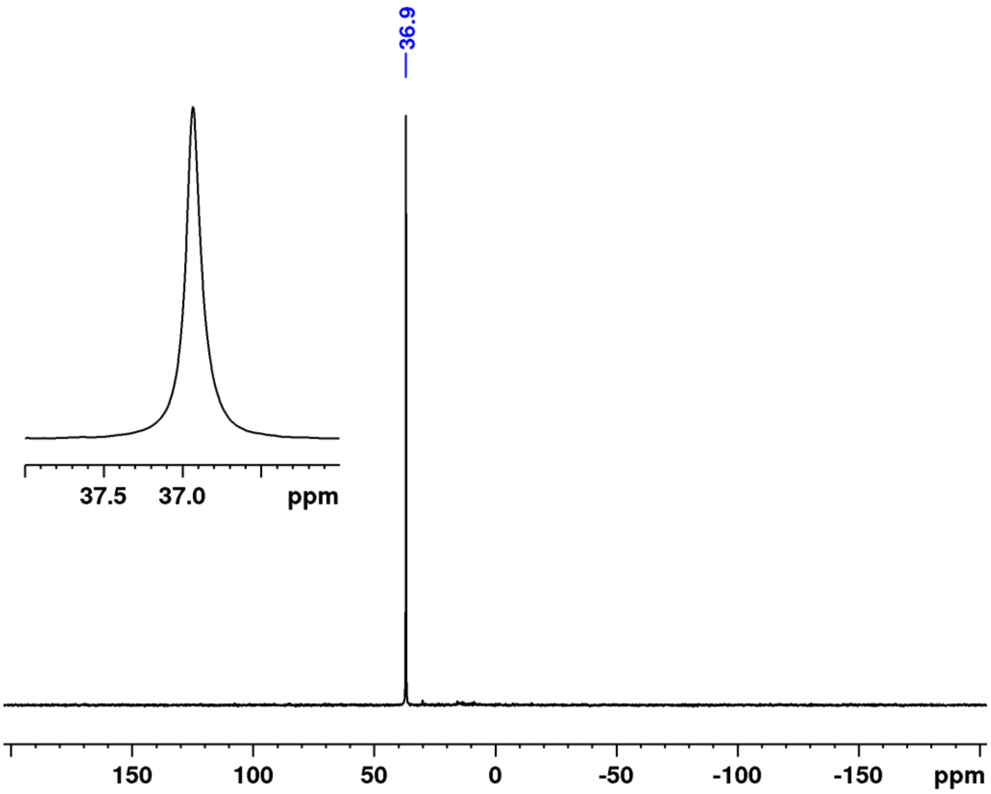

Figure S 24: ^{31}P{^{1}H} NMR spectrum (161.99 MHz, 233 K) of a mixture of [{Ga(dcpe)}$_2$][*pf*]$_2$ and *trans*-β-deutero-styrene in *o*DFB, measured directly after mixing the components at low temperatures.

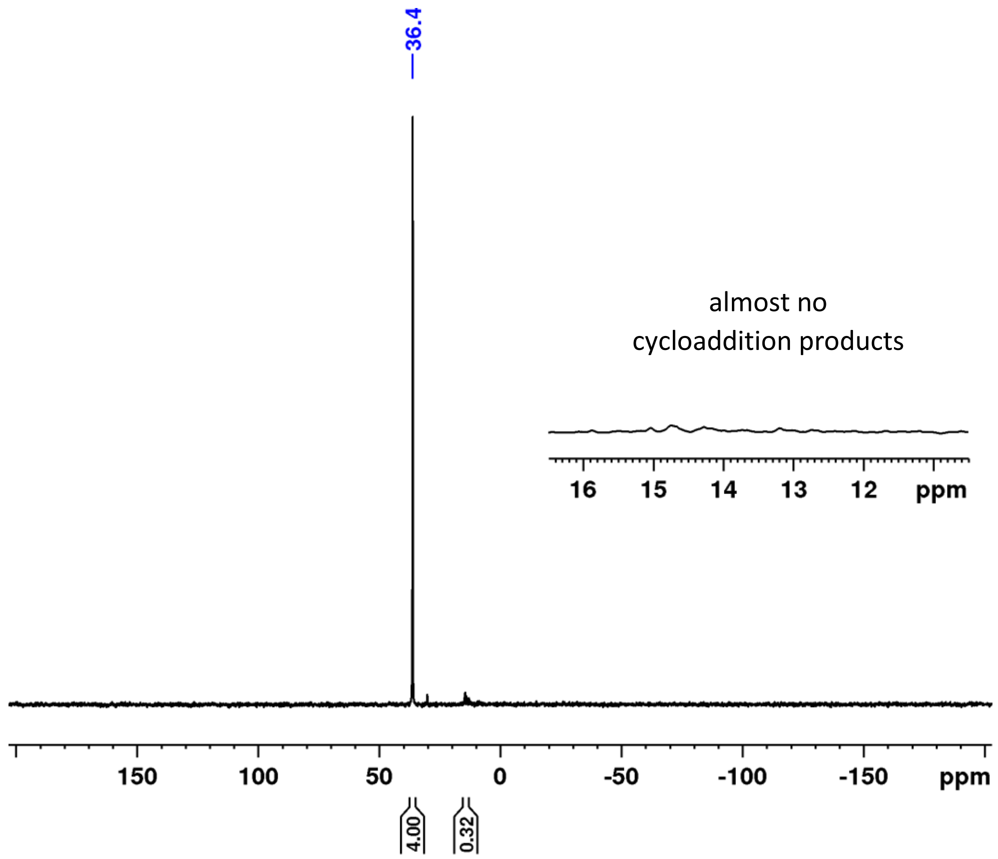

Figure S 25: $^{31}P\{^{1}H\}$ NMR spectrum (161.99 MHz, 263 K) of a mixture of [{Ga(dcpe)}$_2$][*pf*]$_2$ and *trans*-β-deutero-styrene in *o*DFB, measured directly after allowing the sample to warm up to 263 K.

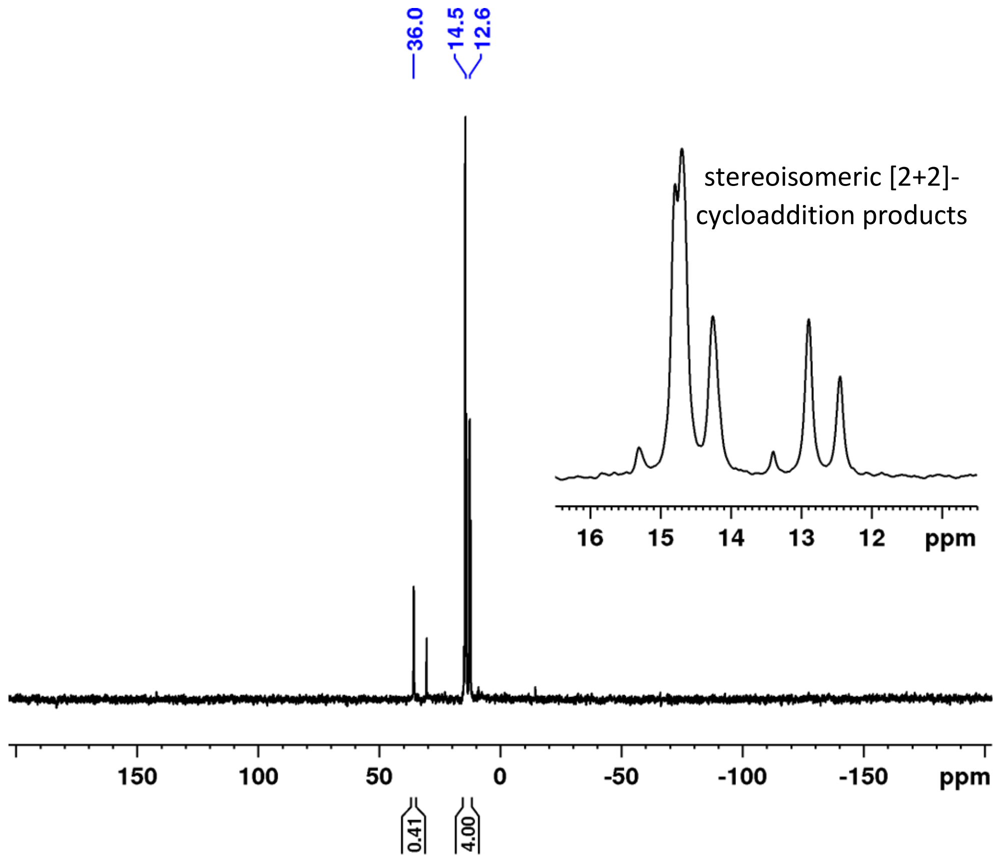

Figure S 26: $^{31}P\{^{1}H\}$ NMR spectrum (161.99 MHz, 298 K) of a mixture of [{Ga(dcpe)}$_2$][*pf*]$_2$ and *trans*-β-deutero-styrene in *o*DFB, measured 1 d after mixing the components.

4.1.2 Reaction with *cis*-β-methyl-styrene

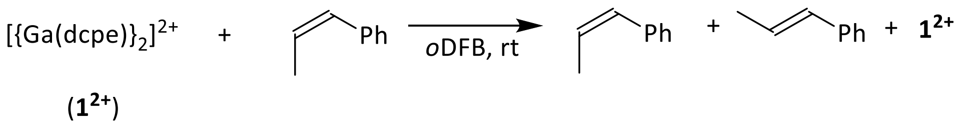

Scheme S 14: Reaction of $[\{Ga(dcpe)\}_2][pf]_2$ ($[\mathbf{1}][pf]_2$) and *cis*-β-methyl-styrene in *o*DFB at rt. The $[pf]^-$ anions are omitted for clarity.

The NMR spectra in **Figure S 27**–**Figure S 29** show that *cis*-β-methyl-styrene is converted to a ≈99:1 mixture of the *trans* and *cis* isomers in the presence of $\mathbf{1}^{2+}$ in an *o*DFB solution. No [2+2] cycloaddition product can be detected in the $^{31}P\{^1H\}$ NMR spectrum.

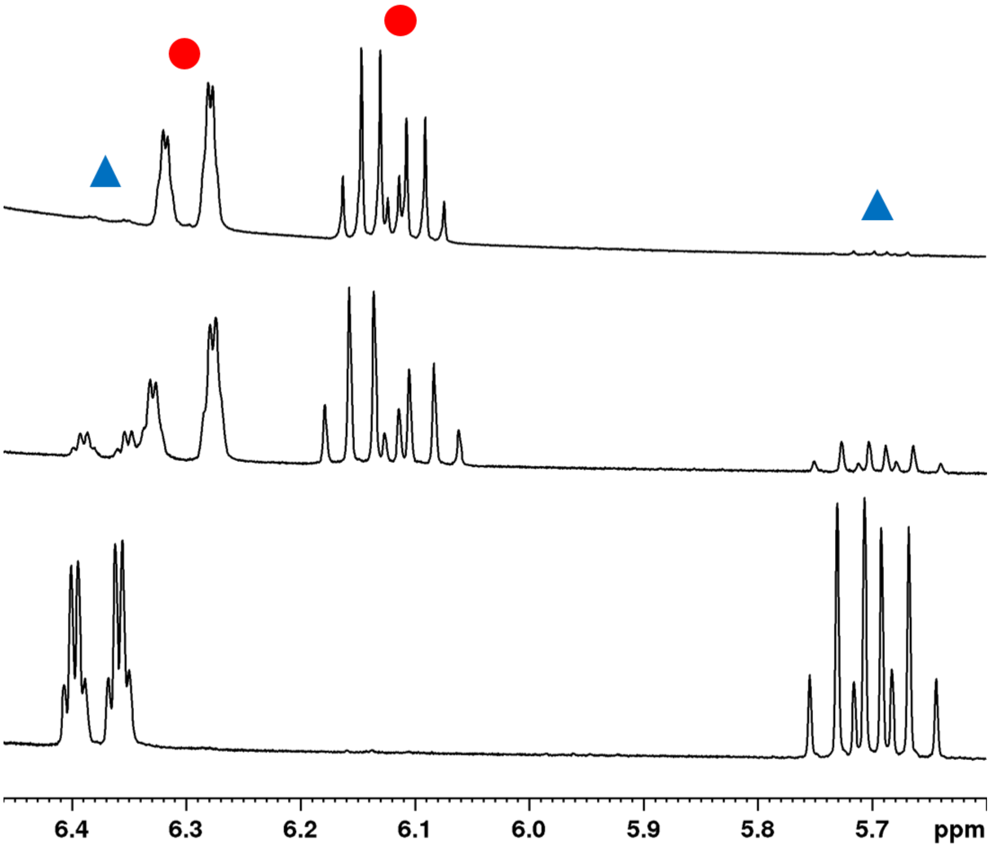

Figure S 27: ^1H NMR spectrum of *cis*-β-methyl-styrene in *o*DFB (300.18 MHz, 298 K, bottom) and of a mixture of $[\{Ga(dcpe)\}_2][pf]_2$ and *cis*-β-methyl-styrene in *o*DFB, 3 h (300.18 MHz, 298 K, middle) and 1 d (400.17 MHz, 298 K, top) after mixing the components. The ^1H NMR signals of the olefinic protons of *cis*-β-methyl-styrene (blue triangles) and of *trans*-β-methyl-styrene (red points) are shown.

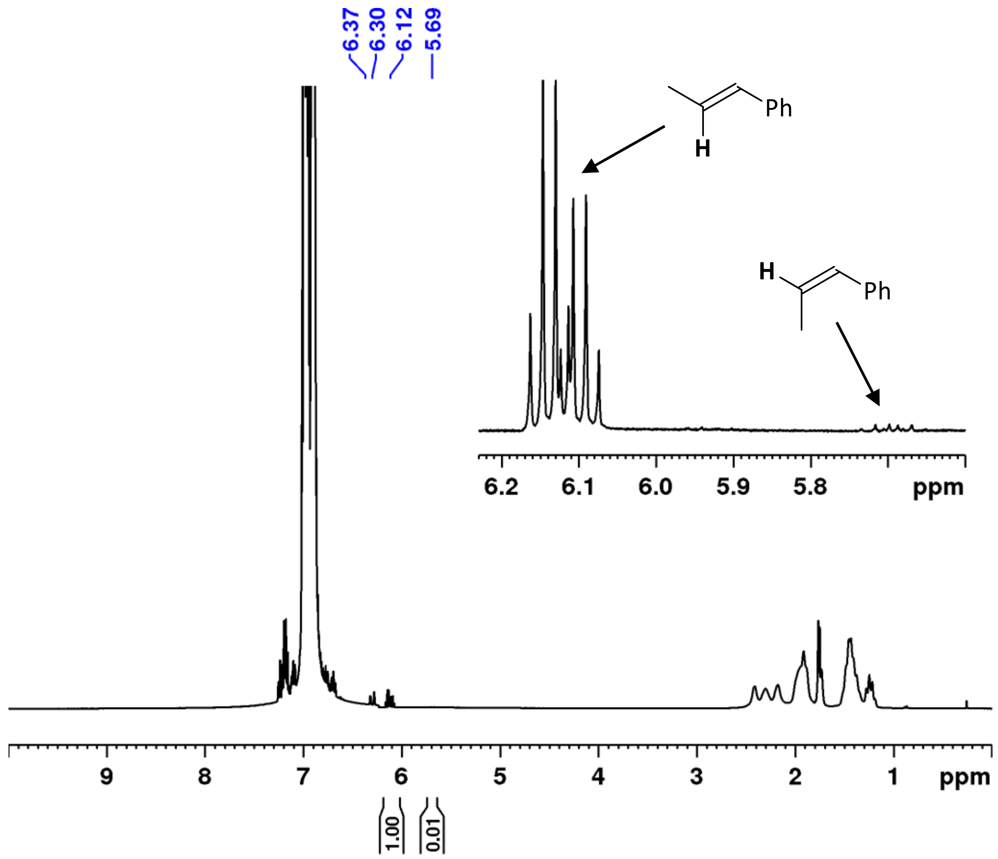

Figure S 28: ^{1}H NMR spectrum of a mixture of [{Ga(dcpe)}$_2$][*pf*]$_2$ and *cis*-β-methyl-styrene in *o*DFB, 1 d (400.17 MHz, 298 K) after mixing the components, with integrated signal intensities for the olefinic protons in β position for both products.

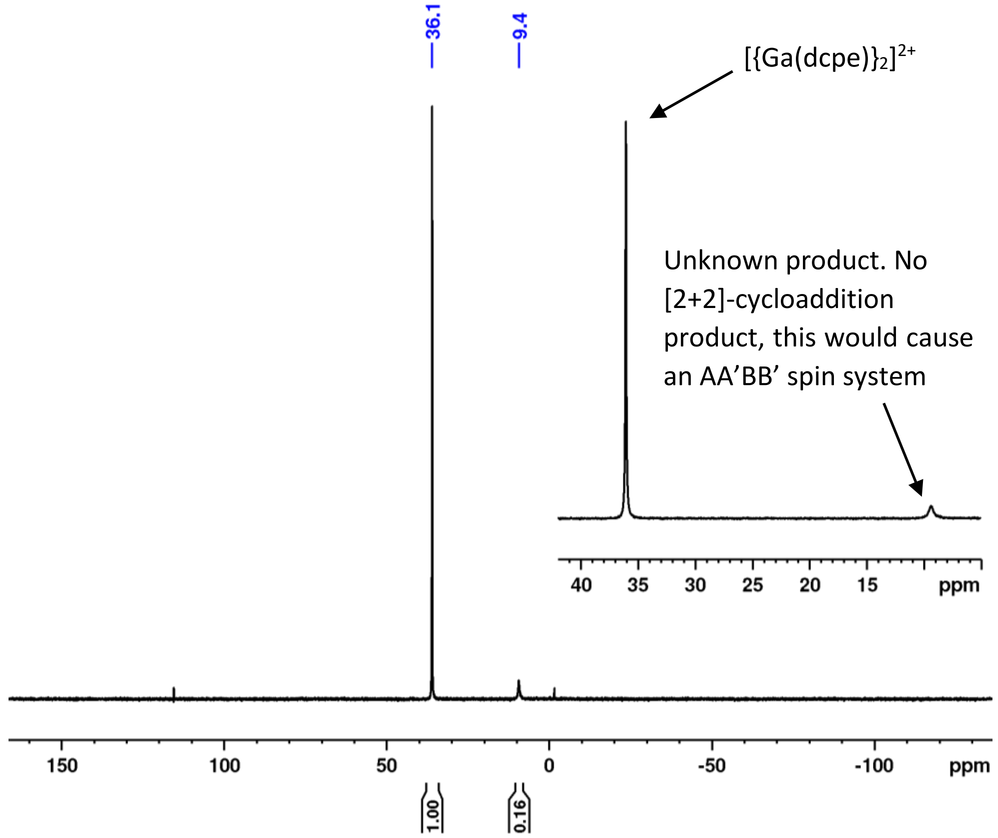

Figure S 29: $^{31}P\{^{1}H\}$ NMR spectrum of a mixture of [{Ga(dcpe)}$_2$][*pf*]$_2$ and *cis*-β-methyl-styrene in *o*DFB, 1 d (161.99 MHz, 298 K) after mixing the components.

4.1.3 Reaction with *cis*-3-hexene

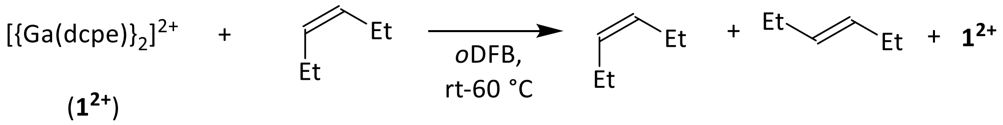

Scheme S 15: Reaction of [{Ga(dcpe)}$_2$][*pf*]$_2$ ([**1**][*pf*]$_2$) and *cis*-3-hexene in *o*DFB at rt–60 °C. The [*pf*]$^-$ anions are omitted for clarity.

The NMR spectra in **Figure S 30**–**Figure S 32** show that *cis*-3-hexene is converted to a ≈3:1 mixture of the *trans* and *cis* isomers in the presence of **1**$^{2+}$ in an *o*DFB solution. No [2+2] cycloaddition product can be detected in the $^{31}P\{^1H\}$ NMR spectrum.

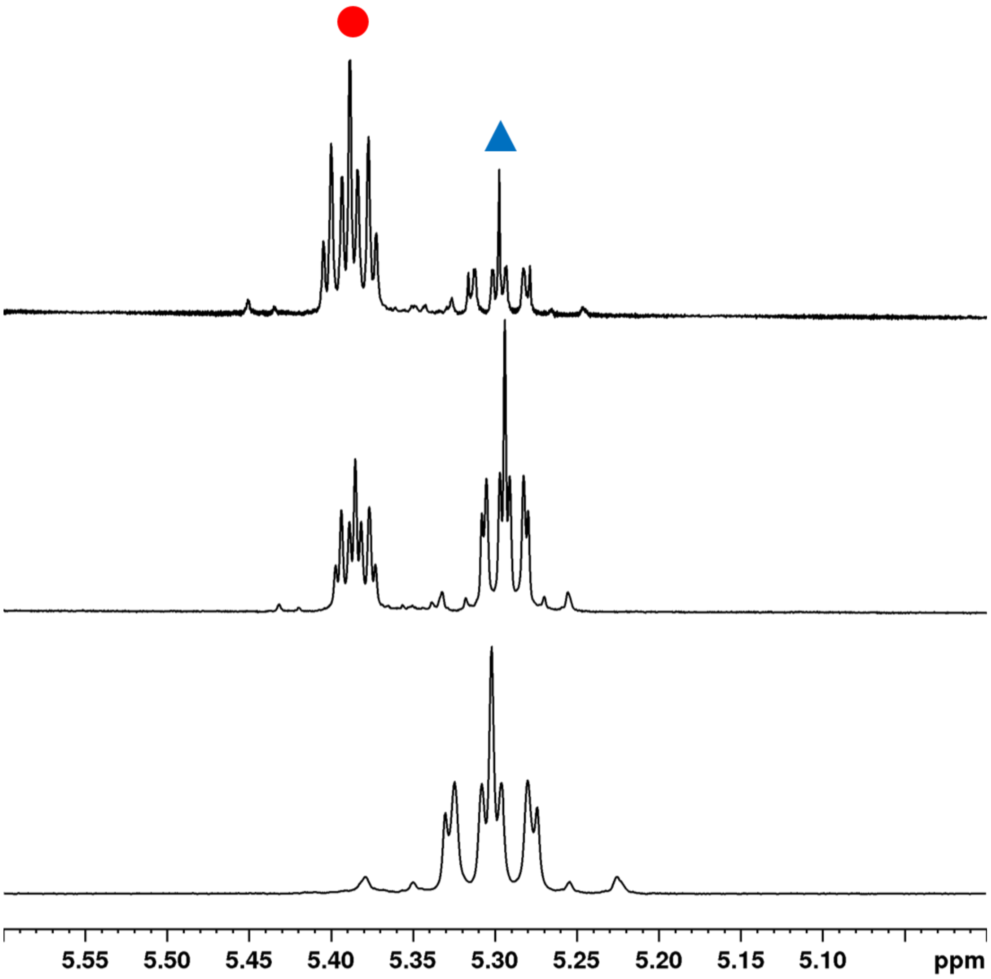

Figure S 30: ^{1}H NMR spectrum of *cis*-3-hexene in *o*DFB (200.13 MHz, 298 K, bottom) and of a mixture of [{Ga(dcpe)}$_2$][*pf*]$_2$ and *cis*-3-hexene in *o*DFB, after 18 h at 60 °C (400.17 MHz, 298 K, middle) and 8 weeks (300.18 MHz, 298 K, top) after mixing the components. The ^{1}H NMR signals of the olefinic protons of *cis*-3-hexene (blue triangle) and of *trans*-3-hexene (red point) are shown.

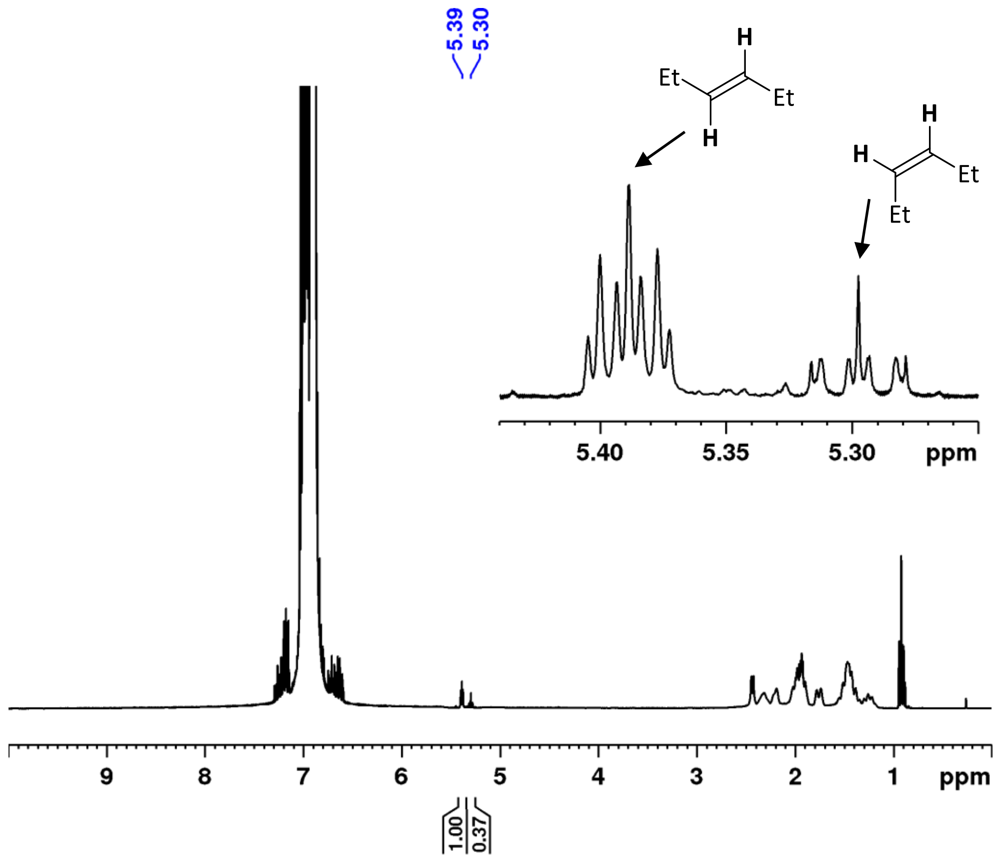

Figure S 31: ^{1}H NMR spectrum of a mixture of [{Ga(dcpe)}$_2$][*pf*]$_2$ and *cis*-3-hexene in *o*DFB, 8 weeks (400.17 MHz, 298 K) after mixing the components, with integrated signal intensities for the olefinic protons for both products.

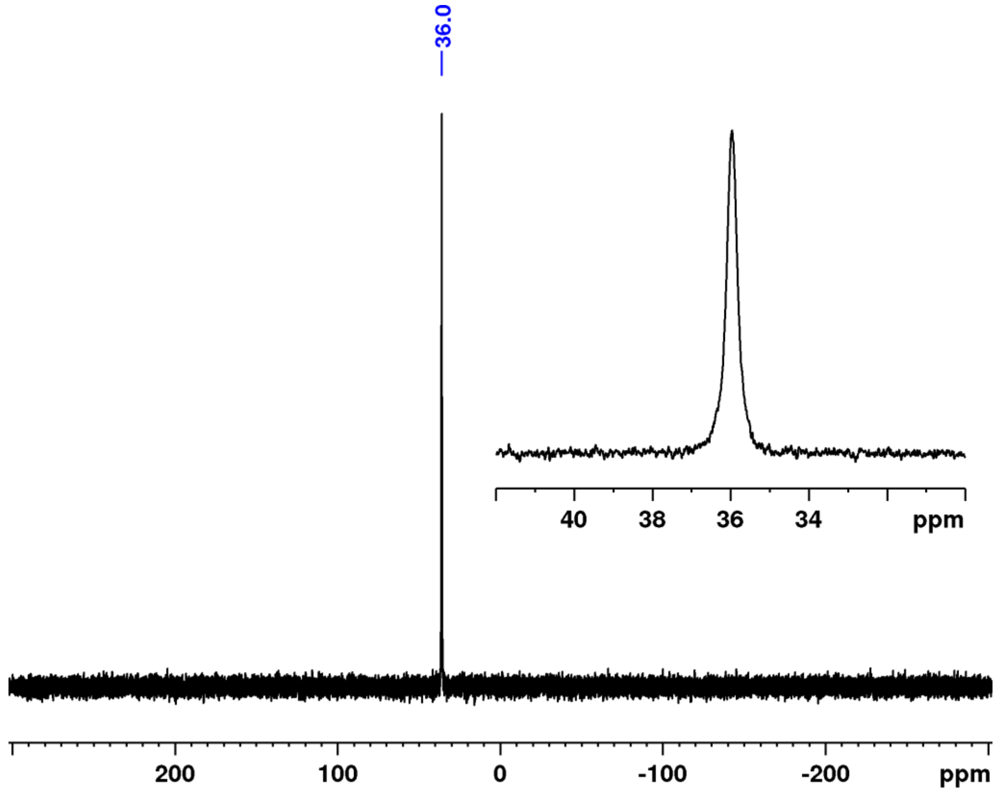

Figure S 32: $^{31}P\{^{1}H\}$ NMR spectrum of a mixture of [{Ga(dcpe)}$_2$][*pf*]$_2$ and *cis*-3-hexene in *o*DFB (121.52 MHz, 298 K), 1 d after mixing the components (the mixture was heated at 60 °C for 18 h).

4.1.4 [{Ga(dcpe)}$_2$(C_5H_6)][*pf*]$_2$

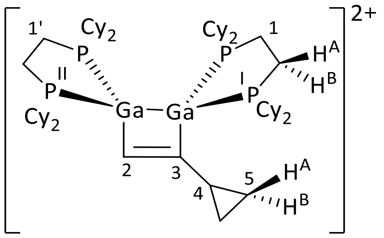

^{1}H NMR [400.17 MHz, *o*DFB, calibrated at *o*$C_6F_2\mathbf{H}_4$ = 6.96 ppm, 298 K]: δ = 7.26 (t, 1 H, C^{2}**H**, $^{3}J_{H,P}$ = 16.7 Hz), 2.53 and 2.38 (m, 8 H, C^{1}**H**^{A}, C$^{1'}$**H**^{A}, C^{1}**H**^{B}, C$^{1'}$**H**^{B}), 2.46–2.15 (m, 8 H, C**H** protons of the Cy groups), 2.06–1.28 (m, 80 H, C**H**$_2$ protons of the Cy groups), 1.72 (m, 1 H, C^{4}**H**), 1.01 and 0.81 (m, 4 H, C^{5}**H**^{A} and C^{5}**H**^{B}) ppm.

^{13}C NMR [100.62 MHz, *o*DFB, 298 K]: δ = 190.5 (1 C, **C**^{3}), 152.2 (1 C, **C**^{2}), 33.4–31.7 (8 C, **C**H carbon atoms of the Cy groups), 29.2–25.0 (40 C, **C**H$_2$ carbon atoms of the Cy groups), 20.0 (1 C, **C**^{4}), 15.8 (4 C, **C**^{1}), 12.6 (2 C, **C**^{5}) ppm.

^{19}F NMR [376.54 MHz, *o*DFB, 298 K]: δ = –75.3 (s, 36 F, [Al(OC(C**F**$_3$)$_3$)$_4$]$^-$), –113.8 (m, Ph**F**), –139.5 (m, *o*$C_6\mathbf{F}_2H_4$) ppm.

^{27}Al NMR [78.22 MHz, *o*DFB, 298 K]: δ = 35.0 (s, 1 Al, [**Al**(OC(CF$_3$)$_3$)$_4$]$^-$) ppm.

^{31}P NMR [161.99 MHz, *o*DFB, 298 K]: δ = 12.57 (m, 2 P, **P**^{II}), 12.52 (m, 2 P, **P**^{I}) ppm.

^{71}Ga NMR [91.55 MHz, *o*DFB, 298 K]: signal probably too broad to be detected, due to the quadrupolar relaxation of ^{71}Ga.

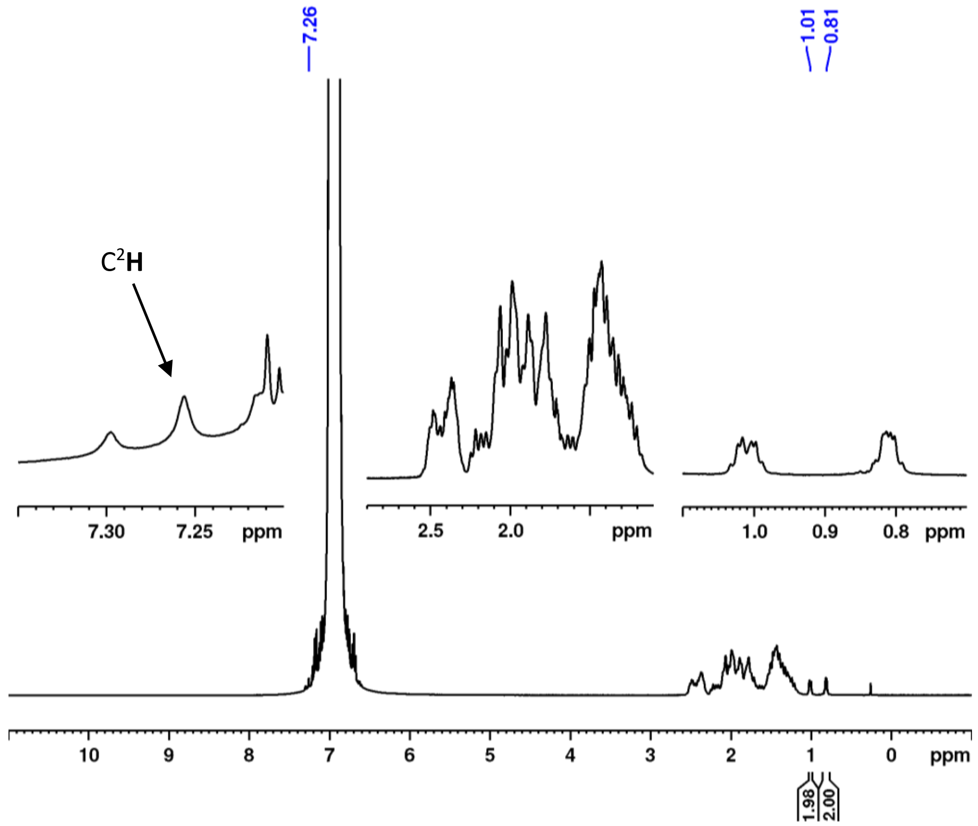

Figure S 33: ^{1}H NMR spectrum (400.17 MHz, *o*DFB, 298 K) of [{Ga(dcpe)}$_2$(C_5H_6)][*pf*]$_2$.

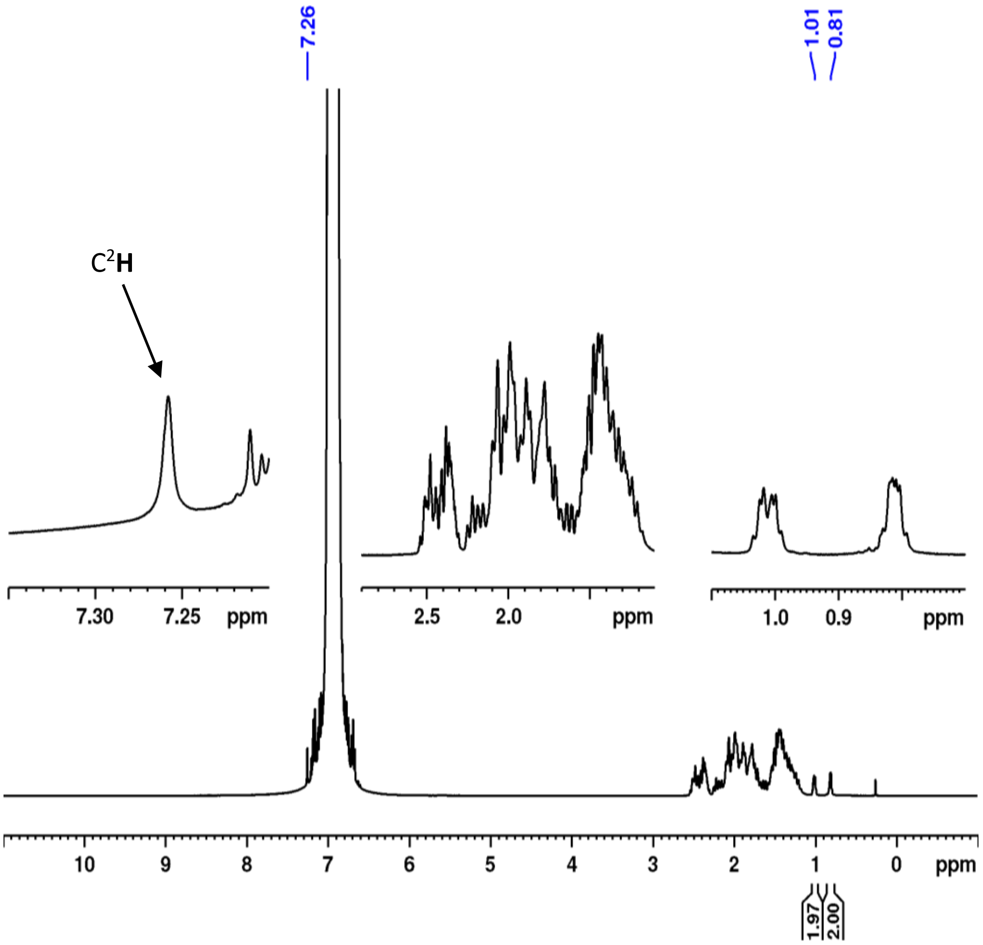

Figure S 34: $^{1}H\{^{31}P\}$ NMR spectrum (400.17 MHz, *o*DFB, 298 K) of [{Ga(dcpe)}$_2$(C_5H_6)][*pf*]$_2$.

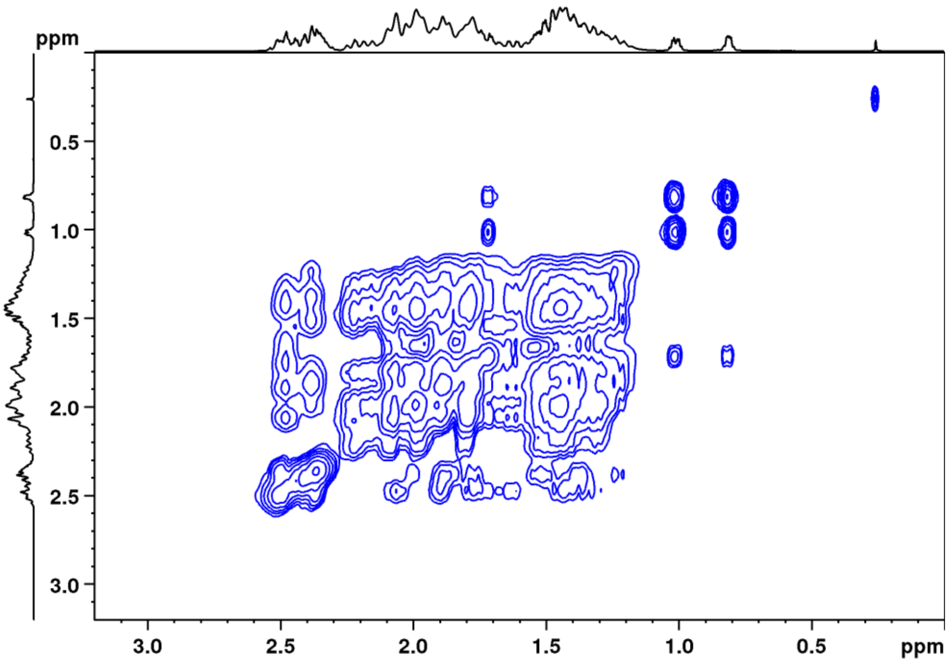

Figure S 35: ^{1}H-TOCSY NMR spectrum (400.17 MHz, *o*DFB, 298 K, mixing time: 0.12 s) of [{Ga(dcpe)}$_2$(C_5H_6)][*pf*]$_2$.

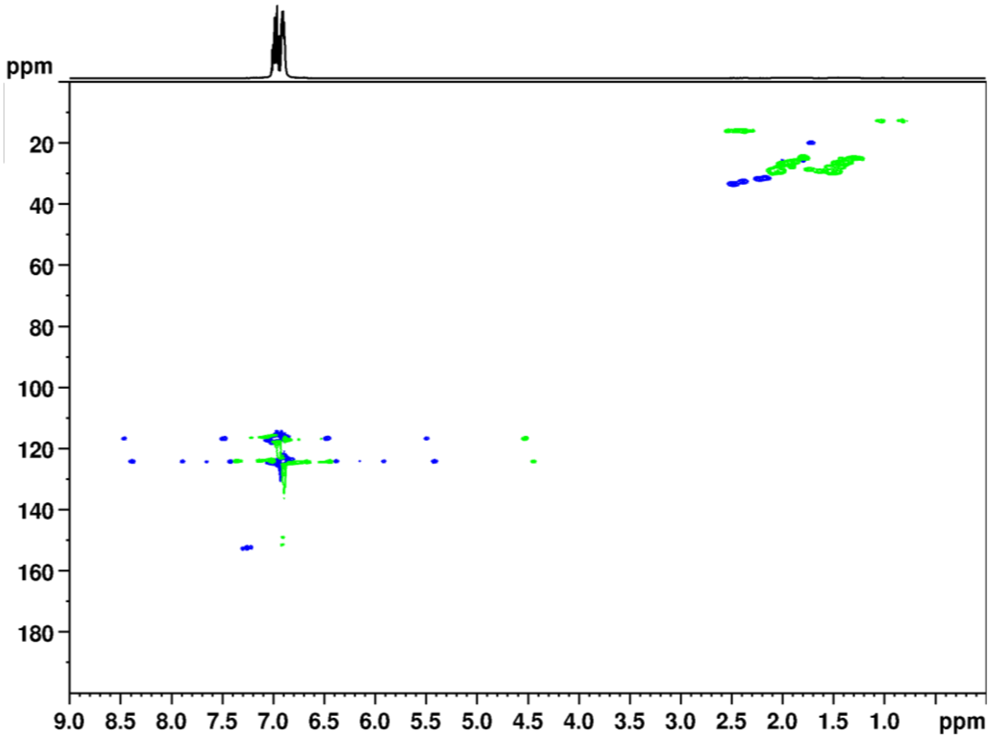

Figure S 36: Edited ^{1}H,^{13}C-HSQC NMR spectrum (400.17 MHz, *o*DFB, 298 K, optimized for J = 145 Hz) of [{Ga(dcpe)}$_2$(C_5H_6)][*pf*]$_2$.

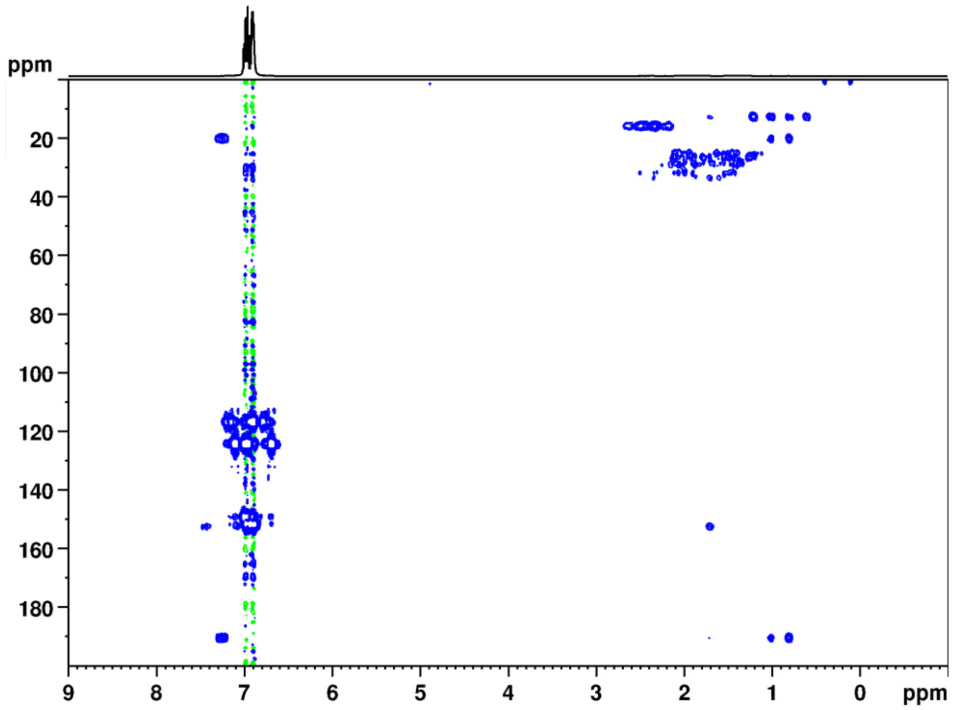

Figure S 37: $^{1}H,^{13}C$-HMBC NMR spectrum (400.17 MHz, *o*DFB, 298 K, optimized for *J* = 8 Hz) of [{Ga(dcpe)}$_2$(C_5H_6)][*pf*]$_2$.

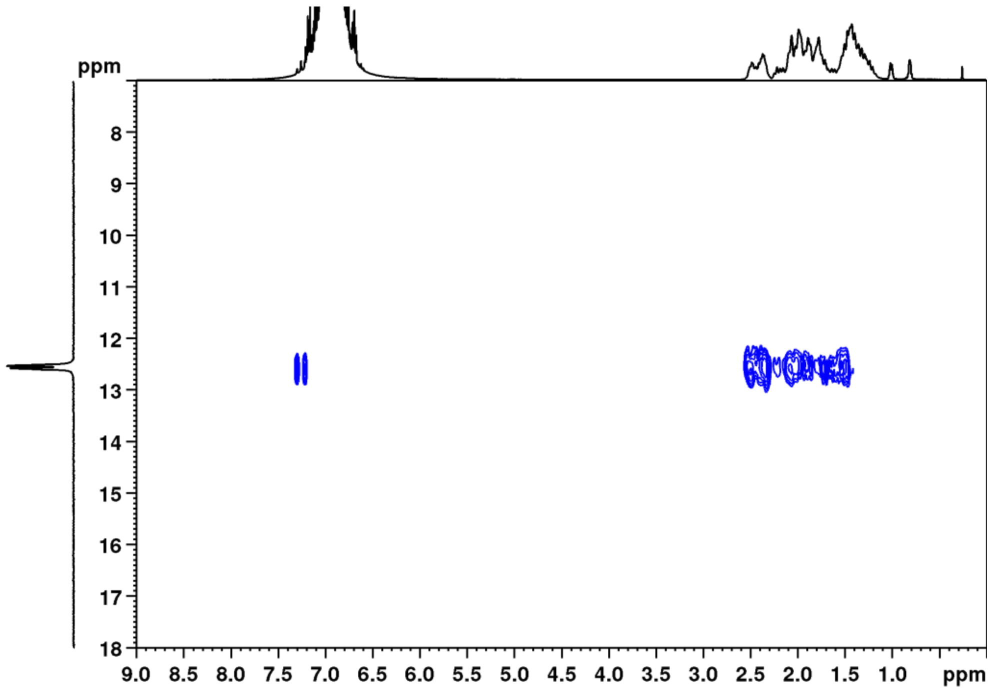

Figure S 38: $^{1}H,^{31}P$-HMBC NMR spectrum (400.17 MHz, *o*DFB, 298 K, optimized for *J* = 15 Hz) of [{Ga(dcpe)}$_2$(C_5H_6)][*pf*]$_2$.

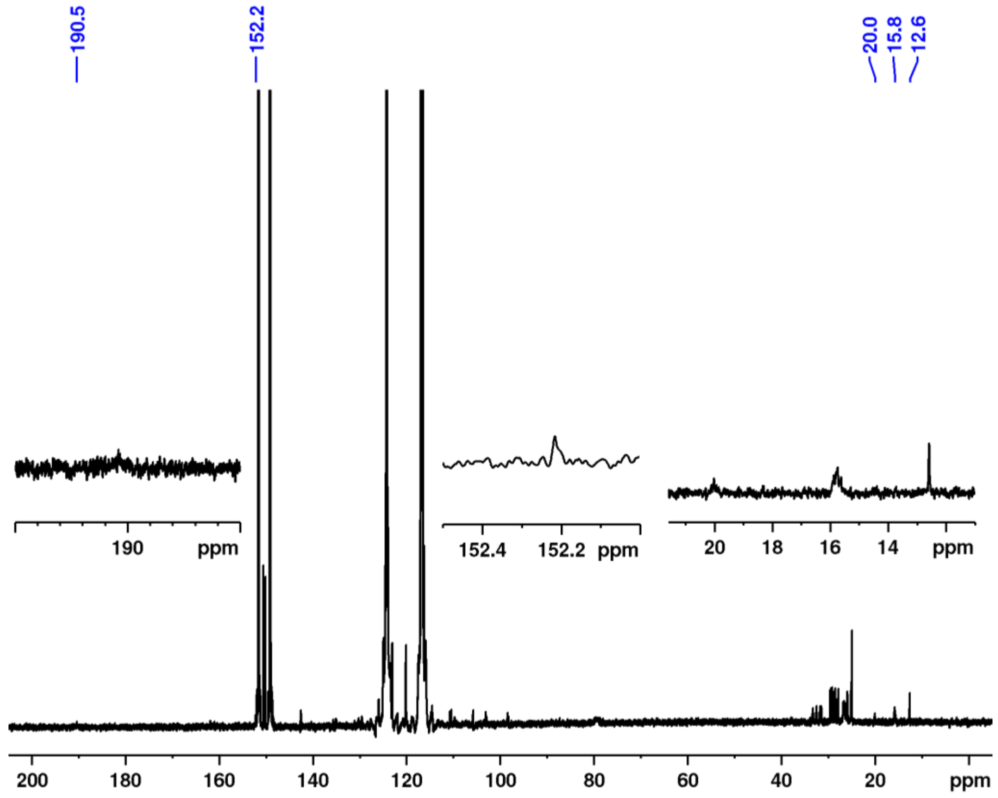

Figure S 39: $^{13}C\{^1H\}$ NMR spectrum (100.62 MHz, *o*DFB, 298 K) of $[\{Ga(dcpe)\}_2(C_5H_6)][pf]_2$.

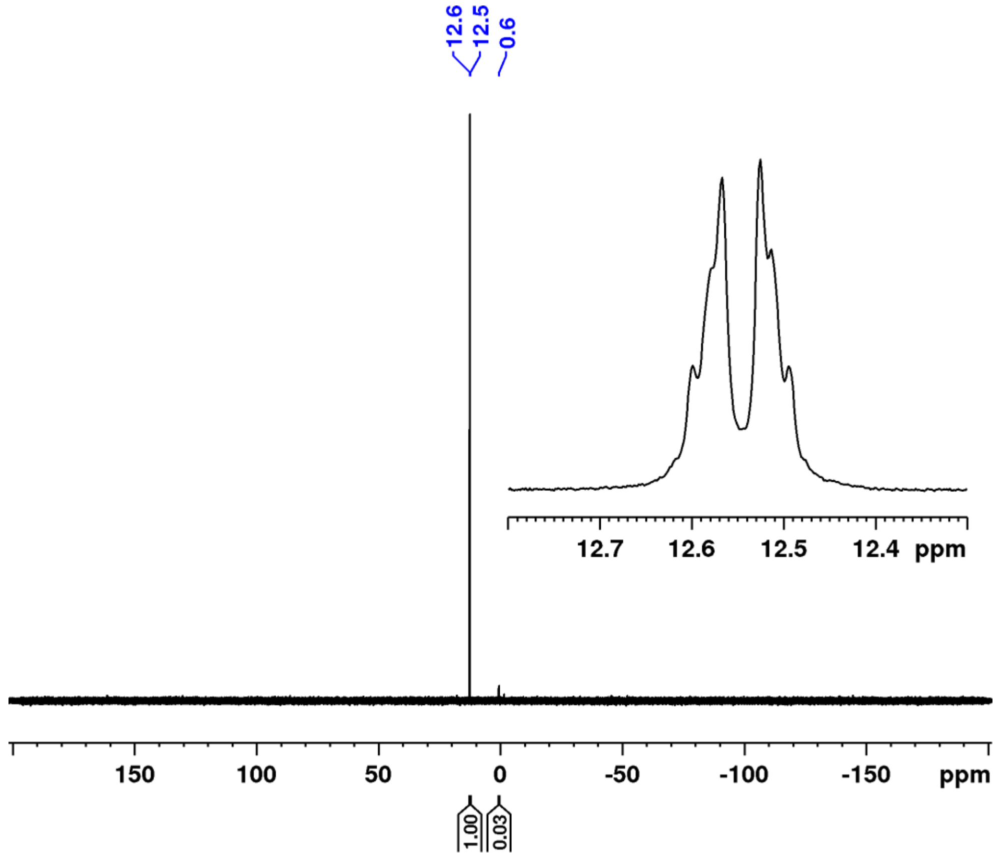

Figure S 40: $^{31}P\{^1H\}$ NMR spectrum (121.52 MHz, *o*DFB, 298 K) of a mixture of $[\{Ga(dcpe)\}_2][pf]_2$ and ethinylcyclopropane in *o*DFB.

4.2 [{Ga(dcpe)}$_2$(C_6H_{10})][*pf*]$_2$

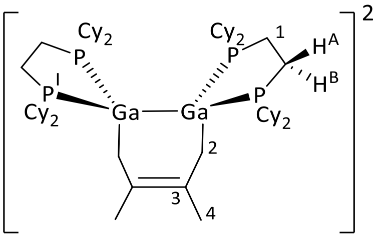

^{1}**H NMR** [300.18 MHz, *o*DFB, calibrated at *o*C_6F_2**H**$_4$ = 6.96 ppm, 298 K]: δ = 2.22 (m, 8 H, C^1**H**^{A} and C^1**H**^{B}), 2.32 and 2.12 (m, 8 H, C**H** protons of the Cy groups), 2.01–1.26 (m, 80 H, C**H**$_2$ protons of the Cy groups), 1.81 (s, 6 H, C^4**H**$_3$), 1.64 (s, 4 H, C^2**H**$_2$) ppm.

^{13}**C NMR** [75.48 MHz, *o*DFB, 298 K]: δ = 132.3 (2 C, **C**^{3}), 33.1 and 31.6 (8 C, **C**H carbon atoms of the Cy groups), 29.1–25.0 (40 C, **C**H$_2$ carbon atoms of the Cy groups), 22.4 (2 C, **C**^{2}), 19.9 (2 C, **C**^{4}), 15.5 (4 C, **C**^{1}) ppm.

^{19}**F NMR** [282.45 MHz, *o*DFB, 298 K]: δ = –75.3 (s, 36 F, [Al(OC(C**F**$_3$)$_3$)$_4$]$^-$), –113.8 (m, Ph**F**), –139.5 (m, *o*C_6**F**$_2$H$_4$) ppm.

^{27}**Al NMR** [78.22 MHz, *o*DFB, 298 K]: δ = 35.0 (s, 1 Al, [**Al**(OC(CF$_3$)$_3$)$_4$]$^-$) ppm.

^{31}**P NMR** [121.52 MHz, *o*DFB, 298 K]: δ = 14.0 (minor impurity), 5.7 (s, 4 P, **P**^{I}) ppm.

^{71}**Ga NMR** [91.55 MHz, *o*DFB, 298 K]: signal probably too broad to be detected, due to the quadrupolar relaxation of ^{71}Ga.

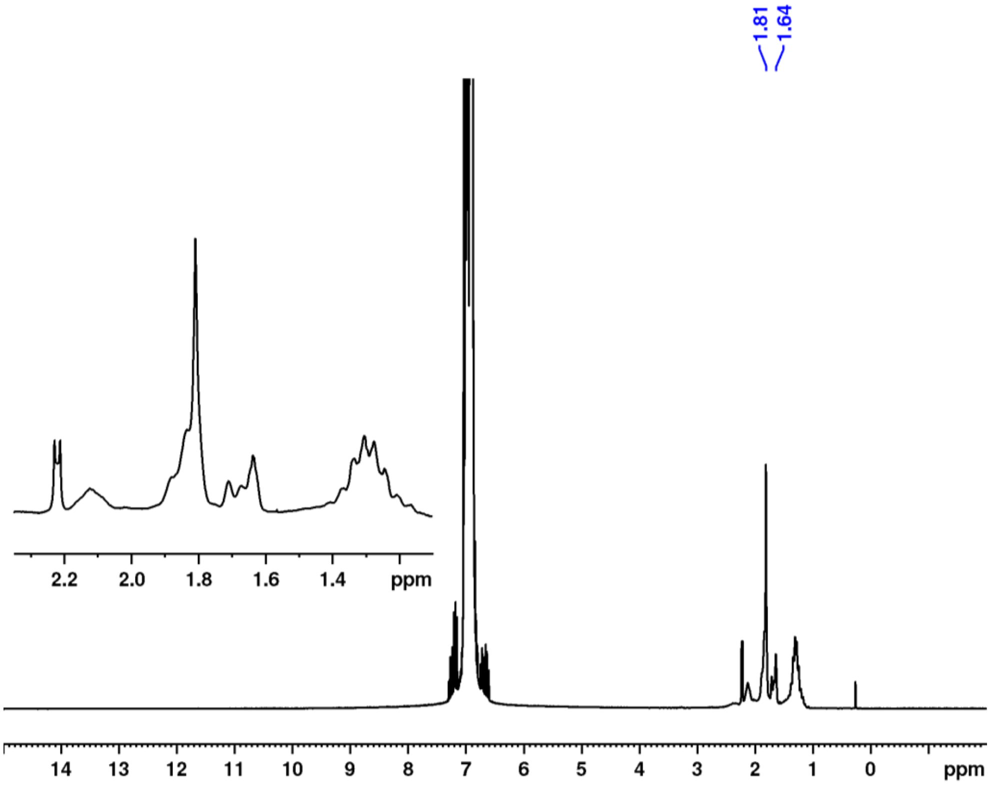

Figure S 41: ^{1}H NMR spectrum (300.18 MHz, *o*DFB, 298 K) of [{Ga(dcpe)}$_2$(C$_6$H$_{10}$)][*pf*]$_2$.

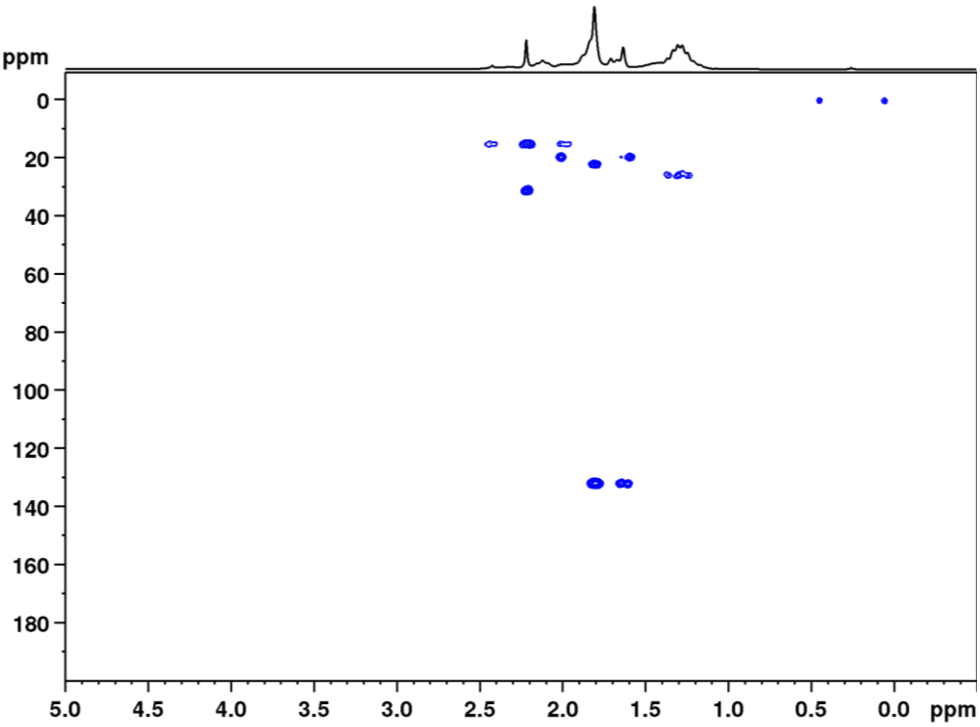

Figure S 42: ^{1}H,^{13}C-HMBC NMR spectrum (300.18 MHz, *o*DFB, 298 K, optimized for *J* = 8 Hz) of [{Ga(dcpe)}$_2$(C$_6$H$_{10}$)][*pf*]$_2$.

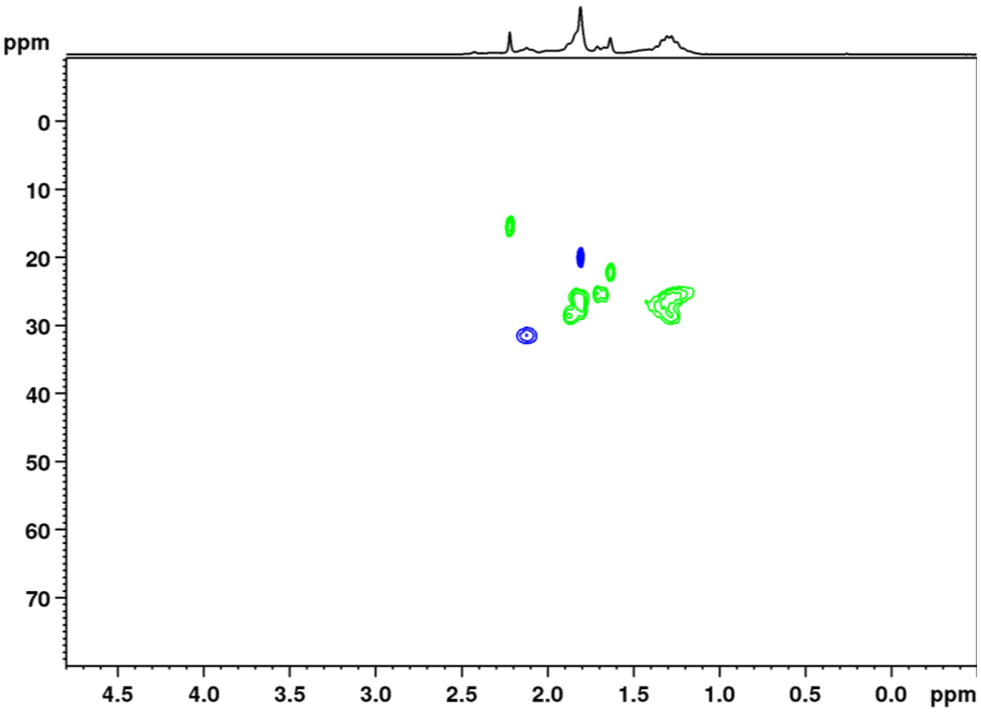

Figure S 43: Edited $^{1}H,^{13}C$-HSQC NMR spectrum (300.18 MHz, *o*DFB, 298 K, optimized for J = 145 Hz) of [{Ga(dcpe)}$_2$(C_6H_{10})][*pf*]$_2$.

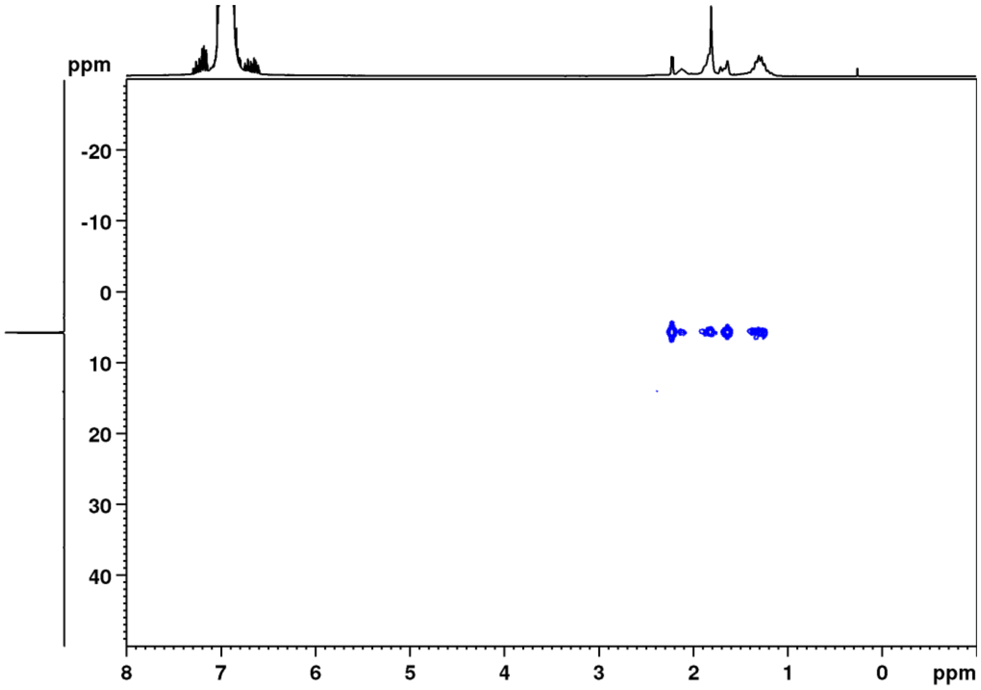

Figure S 44: $^{1}H,^{31}P$-HMBC NMR spectrum (300.18 MHz, *o*DFB, 298 K, optimized for J = 15 Hz) of [{Ga(dcpe)}$_2$(C_6H_{10})][*pf*]$_2$.

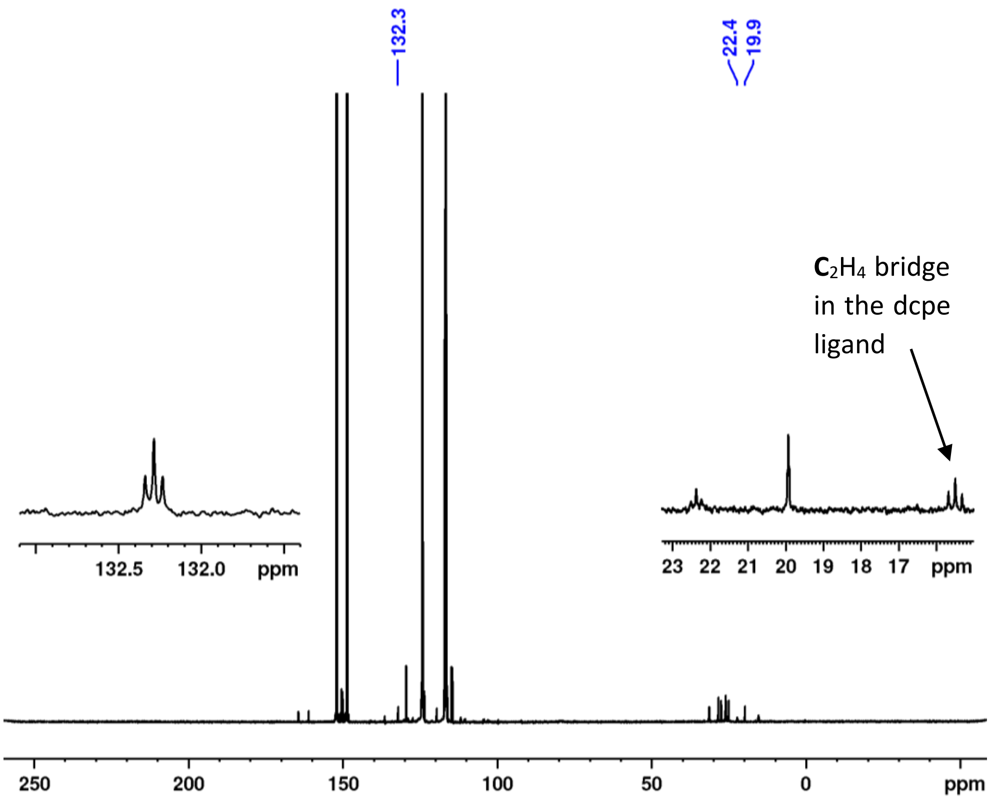

Figure S 45: $^{13}C\{^1H\}$ NMR spectrum (75.48 MHz, *o*DFB, 298 K) of [{Ga(dcpe)}$_2$(C_6H_{10})][*pf*]$_2$.

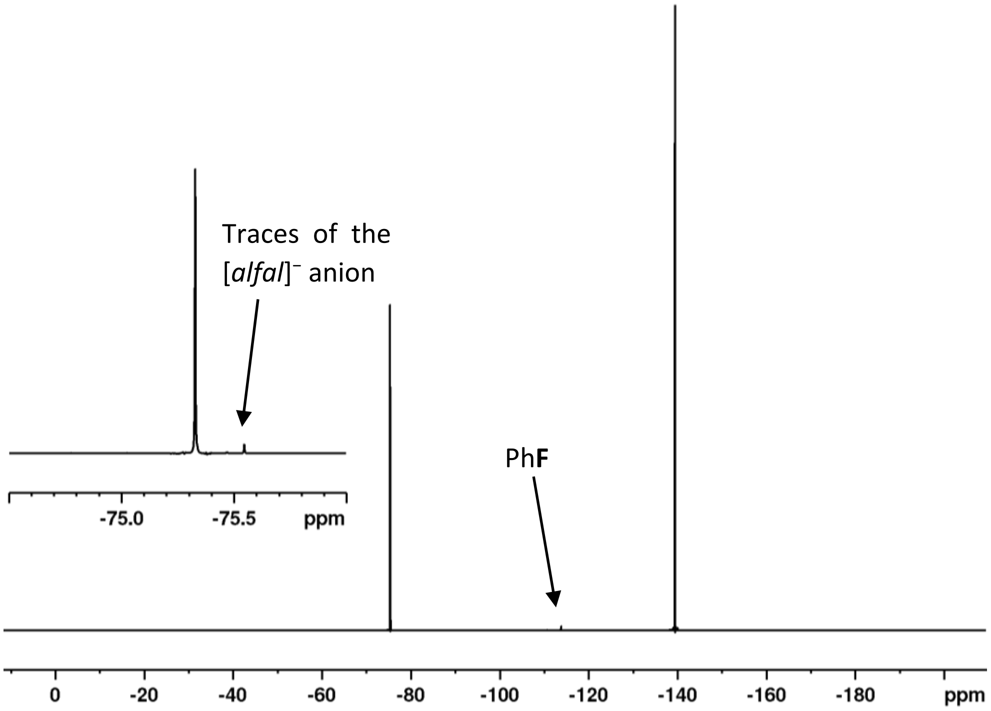

Figure S 46: ^{19}F NMR spectrum (282.45 MHz, *o*DFB, 298 K) of [{Ga(dcpe)}$_2$(C_6H_{10})][*pf*]$_2$.

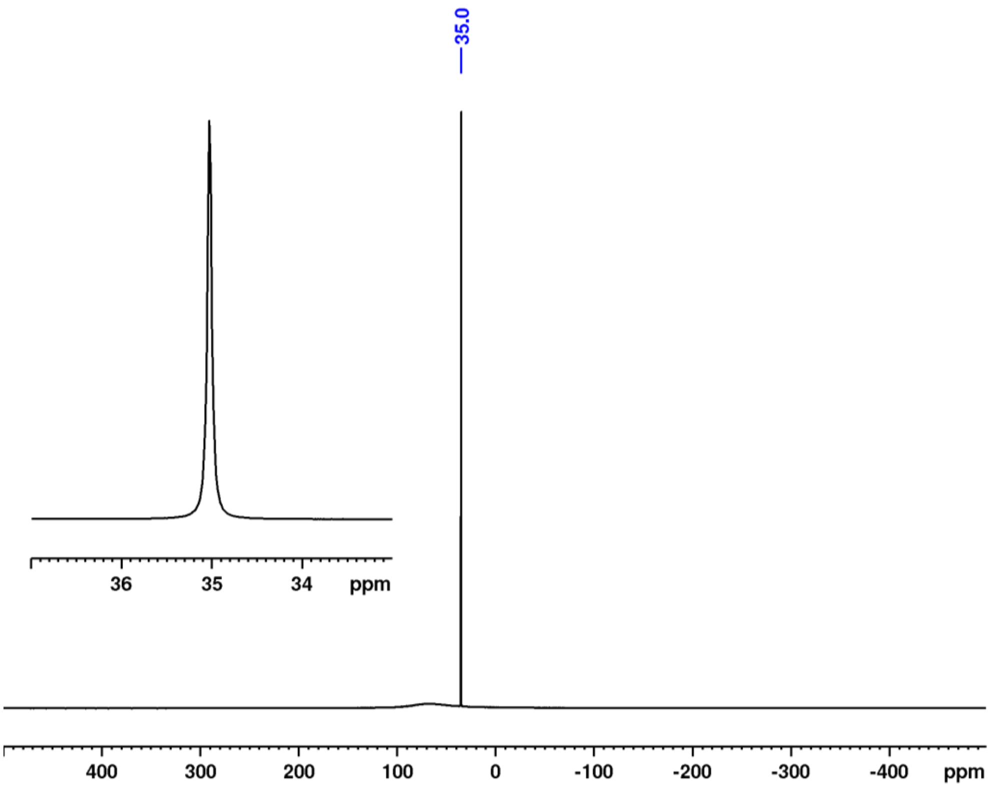

Figure S 47: ^{27}Al NMR spectrum (78.22 MHz, *o*DFB, 298 K) of [{Ga(dcpe)}$_2$(C$_6$H$_{10}$)][*pf*]$_2$.

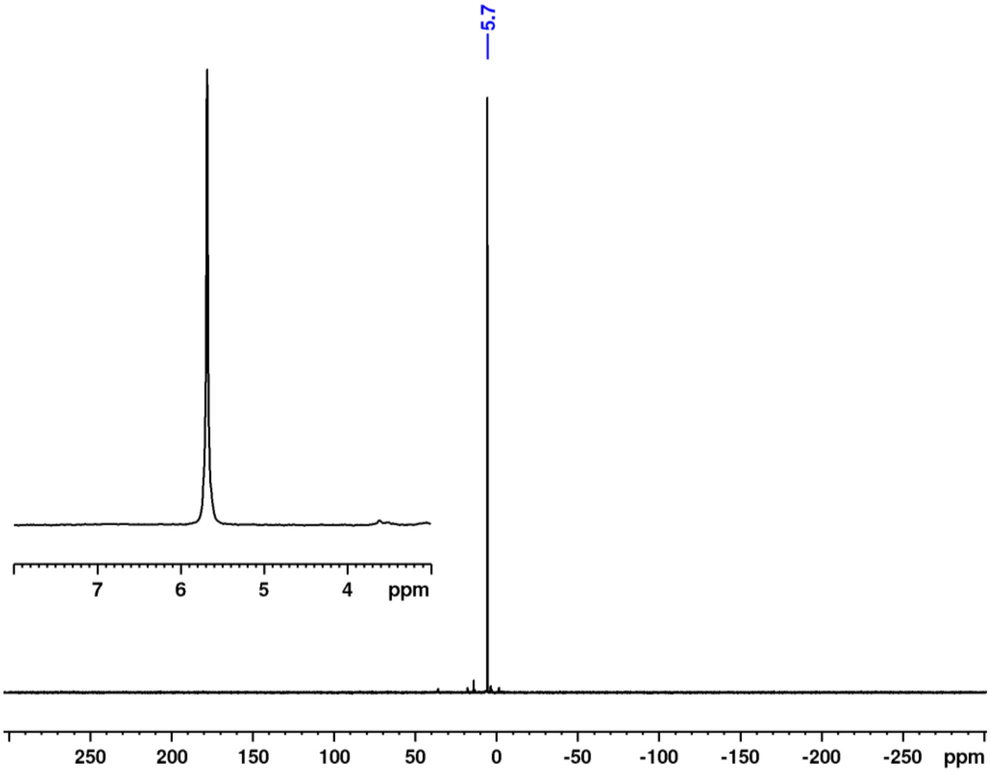

Figure S 48: ^{31}P{^{1}H} NMR spectrum (121.52 MHz, *o*DFB, 298 K) of [{Ga(dcpe)}$_2$(C$_6$H$_{10}$)][*pf*]$_2$.

4.3 [H{Ga(dcpe)}$_2$(OEt)][*pf*]$_2$

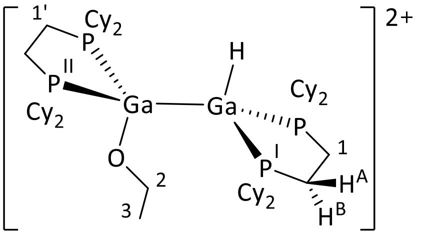

^{1}H NMR [400.17 MHz, *o*DFB, calibrated at $oC_6F_2\mathbf{H}_4$ = 6.96 ppm, 298 K]: δ = 4.53 (t, 1 H, Ga**H**, $^2J_{H,P}$ = 19.6 Hz), 3.91 (q, 2 H, $C^2\mathbf{H}_2$, $^3J_{H,H}$ = 6.8 Hz), 2.45–2.22 (m, 8 H, C**H** protons of the Cy groups), 2.13–1.29 (m, 80 H, C**H**$_2$ protons of the Cy groups), 2.45–2.19 (m, 8 H, $C^1\mathbf{H}^A$, $C^1\mathbf{H}^B$, $C^{1'}\mathbf{H}^A$ and $C^{1'}\mathbf{H}^B$), 1.36 (t, 2 H, $C^3\mathbf{H}_3$, $^3J_{H,H}$ = 6.8 Hz) ppm.

^{13}C NMR [100.62 MHz, *o*DFB, 298 K]: δ = 64.0 (1 C, **C**^{2}), 20.2 (1 C, **C**^{3}) 33.1–31.5 (8 C, **C**H carbon atoms of the Cy groups), 29.3–25.1 (40 C, **C**H$_2$ carbon atoms of the Cy groups), 16.8 and 15.2 (4 C, **C**^{1} and **C**$^{1'}$) ppm.

^{19}F NMR [376.54 MHz, *o*DFB, 298 K]: δ = –75.3 (s, 36 F, $[Al(OC(C\mathbf{F}_3)_3)_4]^-$), –113.9 (m, Ph**F**), –139.5 (m, $oC_6\mathbf{F}_2H_4$) ppm.

^{27}Al NMR [104.27 MHz, *o*DFB, 298 K]: δ = 35.0 (s, 1 Al, $[\mathbf{Al}(OC(CF_3)_3)_4]^-$) ppm.

^{31}P NMR [161.99 MHz, *o*DFB, 298 K]: δ = 27.6 and –2.0 (unknown impurity), 21.4 (m, 2 P, **P**^{I}), 9.4 (m, 2 P, **P**^{II}), –1.5 (unknown impurity) ppm.

^{71}Ga NMR [122.04 MHz, *o*DFB, 298 K]: signal probably too broad to be detected, due to the quadrupolar relaxation of ^{71}Ga.

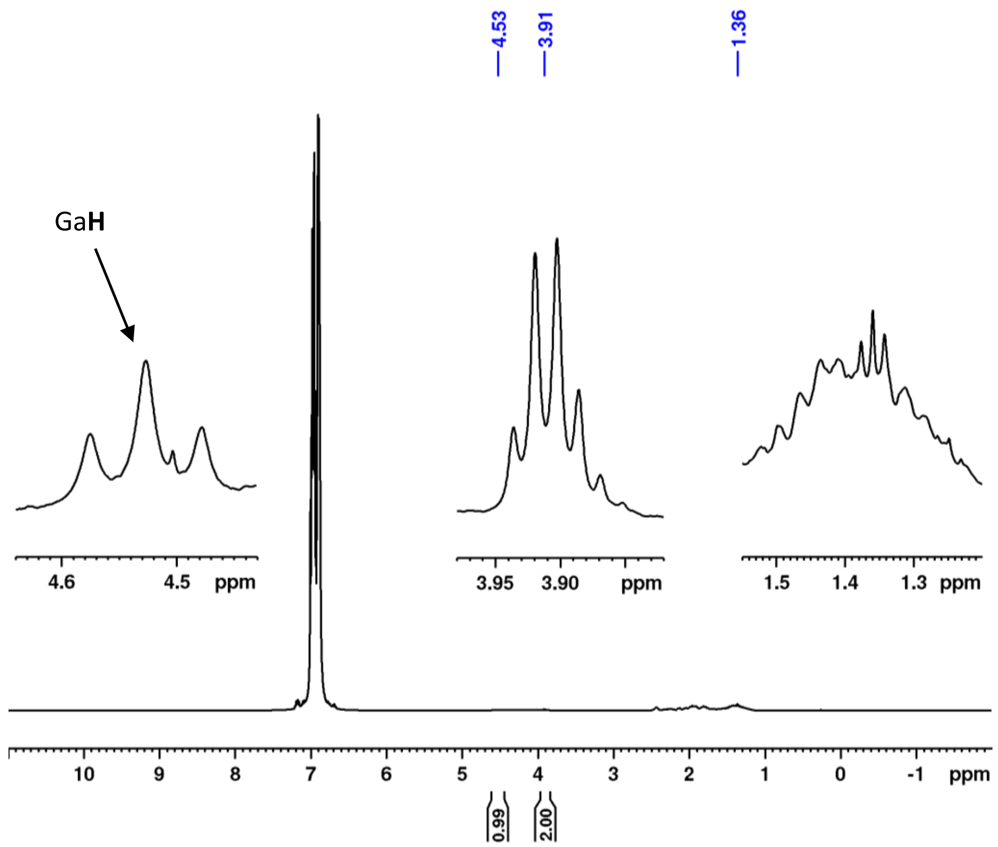

Figure S 49: ^{1}H NMR spectrum (400.17 MHz, *o*DFB, 298 K) of a mixture of [{Ga(dcpe)}$_2$][*pf*]$_2$ and HOEt in *o*DFB.

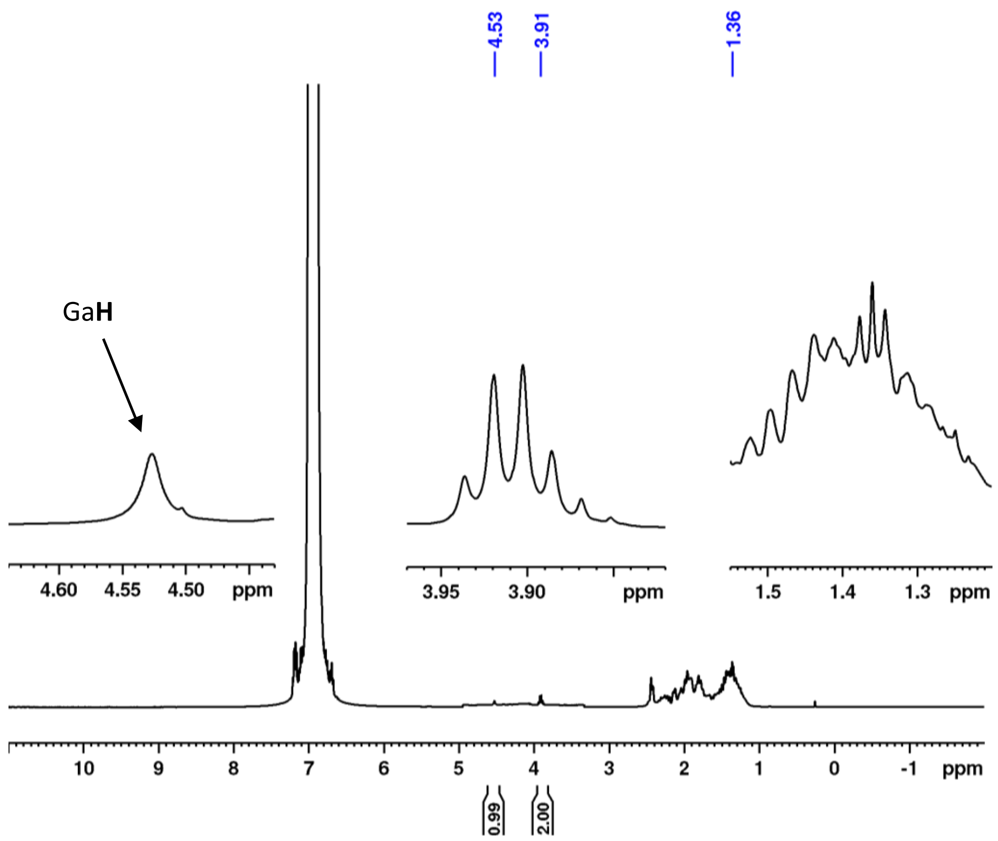

Figure S 50: ^{1}H{^{31}P} NMR spectrum (400.17 MHz, *o*DFB, 298 K) of a mixture of [{Ga(dcpe)}$_2$][*pf*]$_2$ and HOEt in *o*DFB.

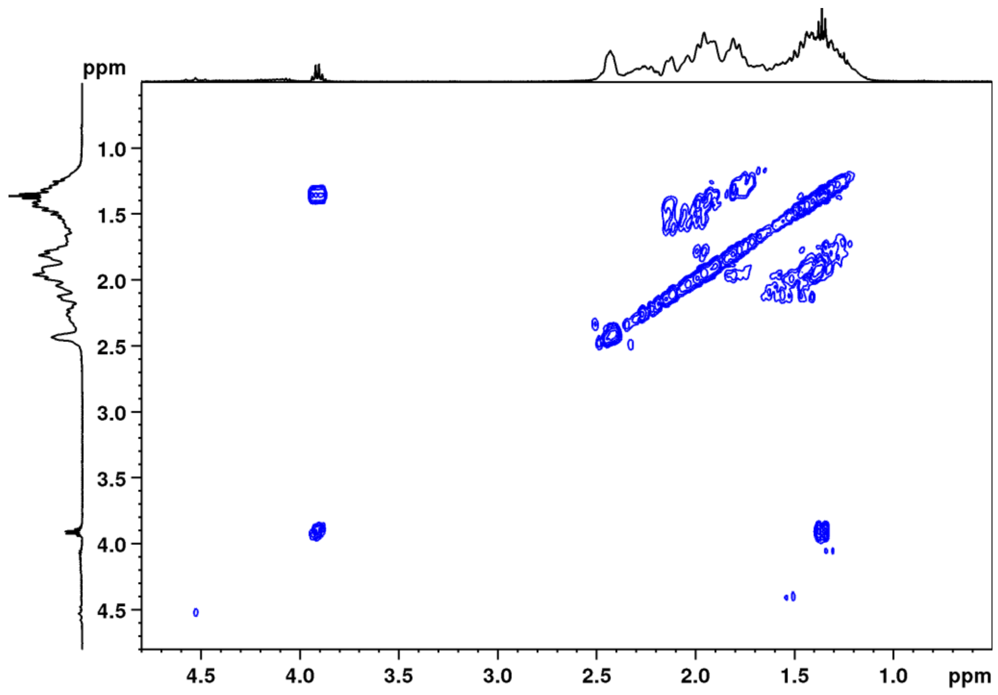

Figure S 51: ^{1}H-COSY NMR spectrum (400.17 MHz, *o*DFB, 298 K) of a mixture of [{Ga(dcpe)}$_2$][*pf*]$_2$ and HOEt in *o*DFB.

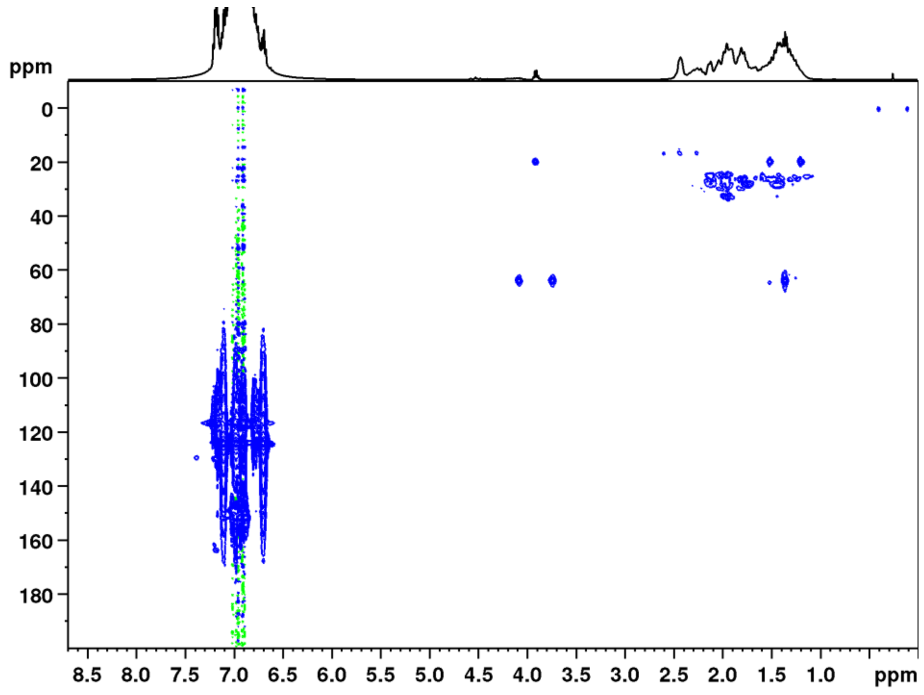

Figure S 52: ^{1}H,^{13}C-HMBC NMR spectrum (400.17 MHz, *o*DFB, 298 K, optimized for *J* = 8 Hz) of a mixture of [{Ga(dcpe)}$_2$][*pf*]$_2$ and HOEt in *o*DFB.

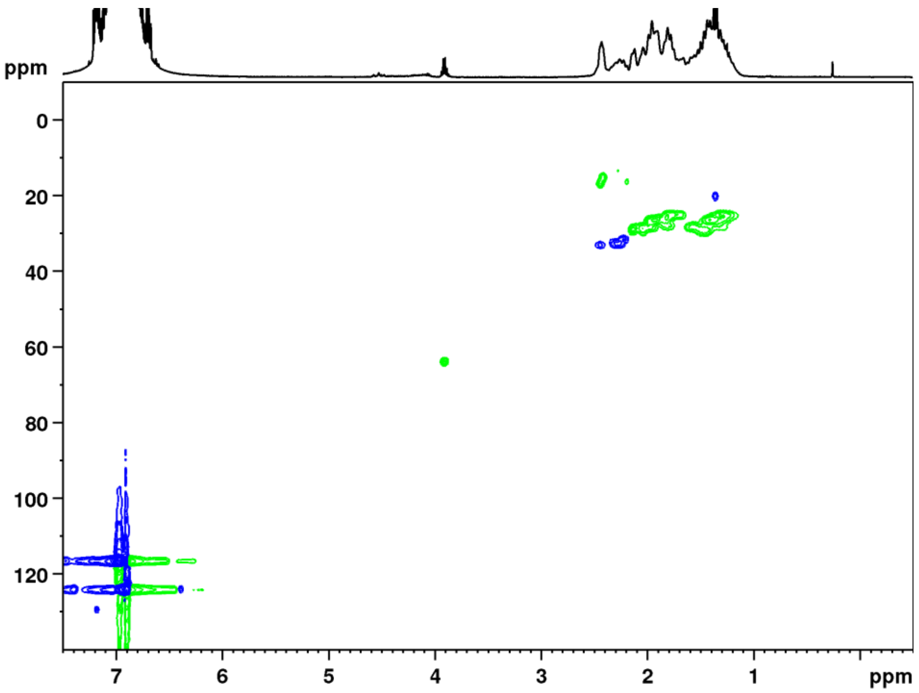

Figure S 53: Edited $^{1}H,^{13}C$-HSQC NMR spectrum (400.17 MHz, *o*DFB, 298 K, optimized for $J = 145$ Hz) of a mixture of $[\{Ga(dcpe)\}_2][pf]_2$ and HOEt in *o*DFB.

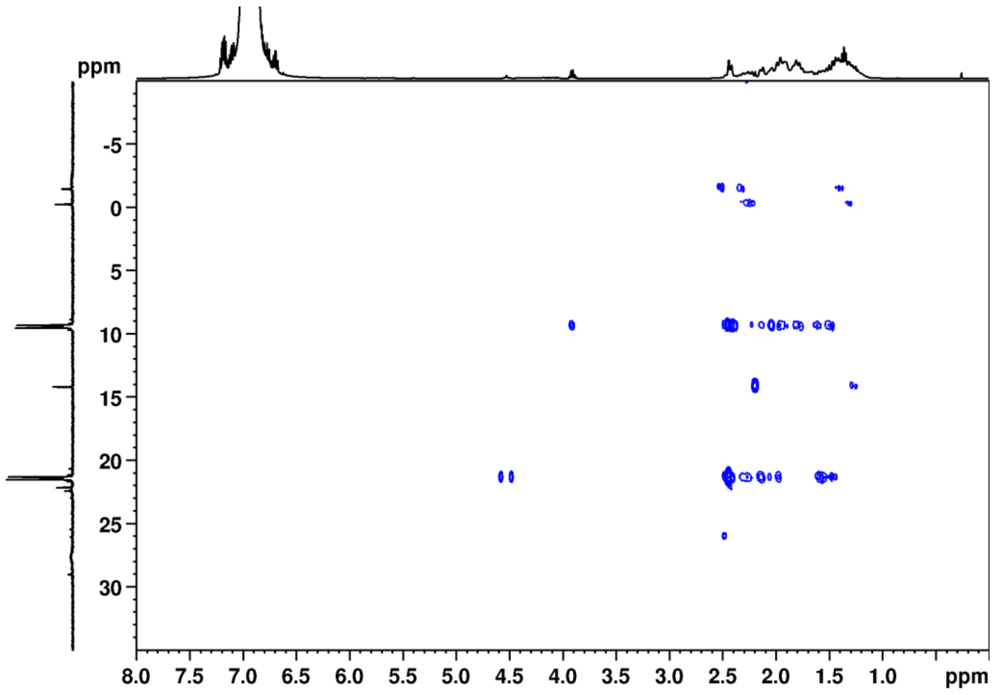

Figure S 54: $^{1}H,^{31}P$-HMBC NMR spectrum (400.17 MHz, *o*DFB, 298 K, optimized for $J = 15$ Hz) of a mixture of $[\{Ga(dcpe)\}_2][pf]_2$ and HOEt in *o*DFB.

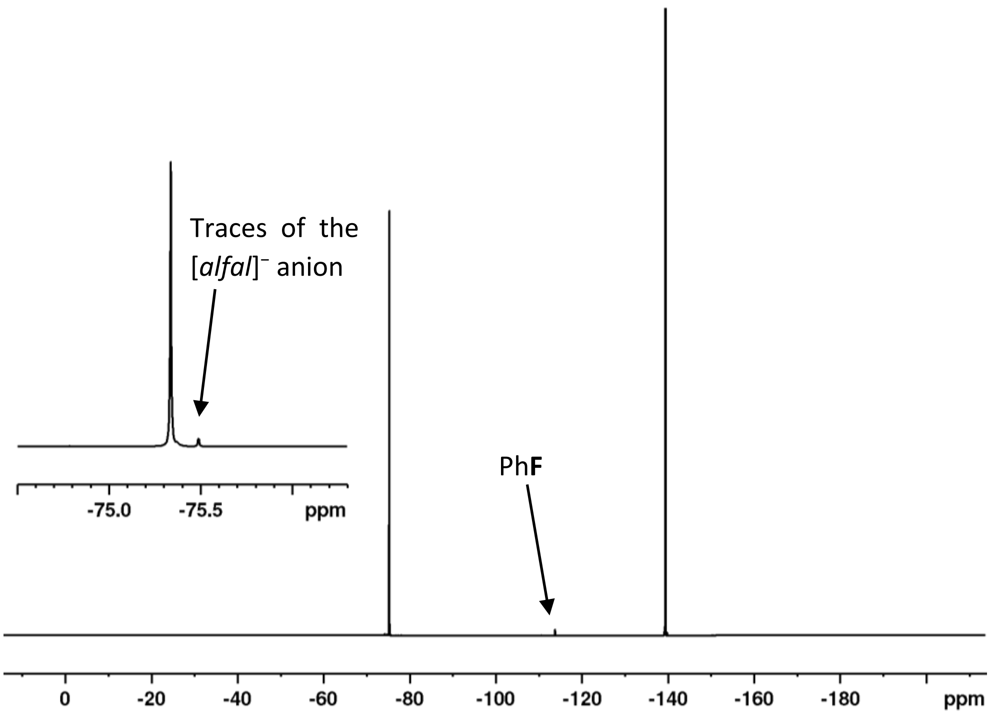

Figure S 55: ^{19}F NMR spectrum (376.54 MHz, *o*DFB, 298 K) of a mixture of [{Ga(dcpe)}$_2$][*pf*]$_2$ and HOEt in *o*DFB.

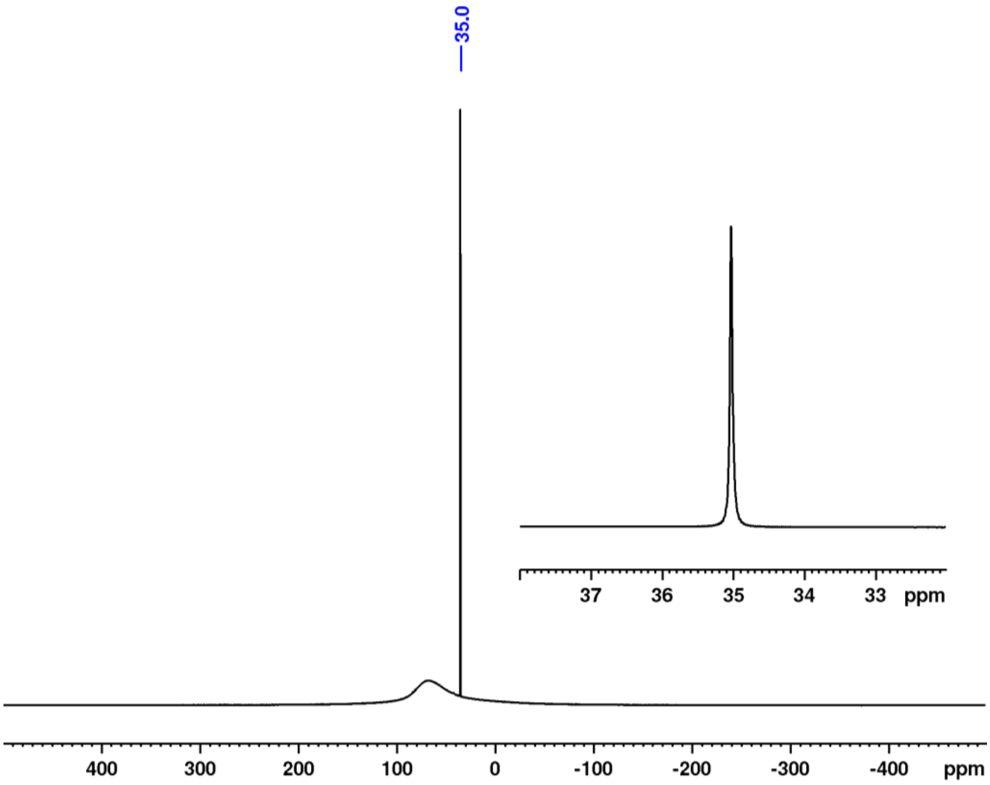

Figure S 56: ^{27}Al NMR spectrum (104.27 MHz, *o*DFB, 298 K) of a mixture of [{Ga(dcpe)}$_2$][*pf*]$_2$ and HOEt in *o*DFB.

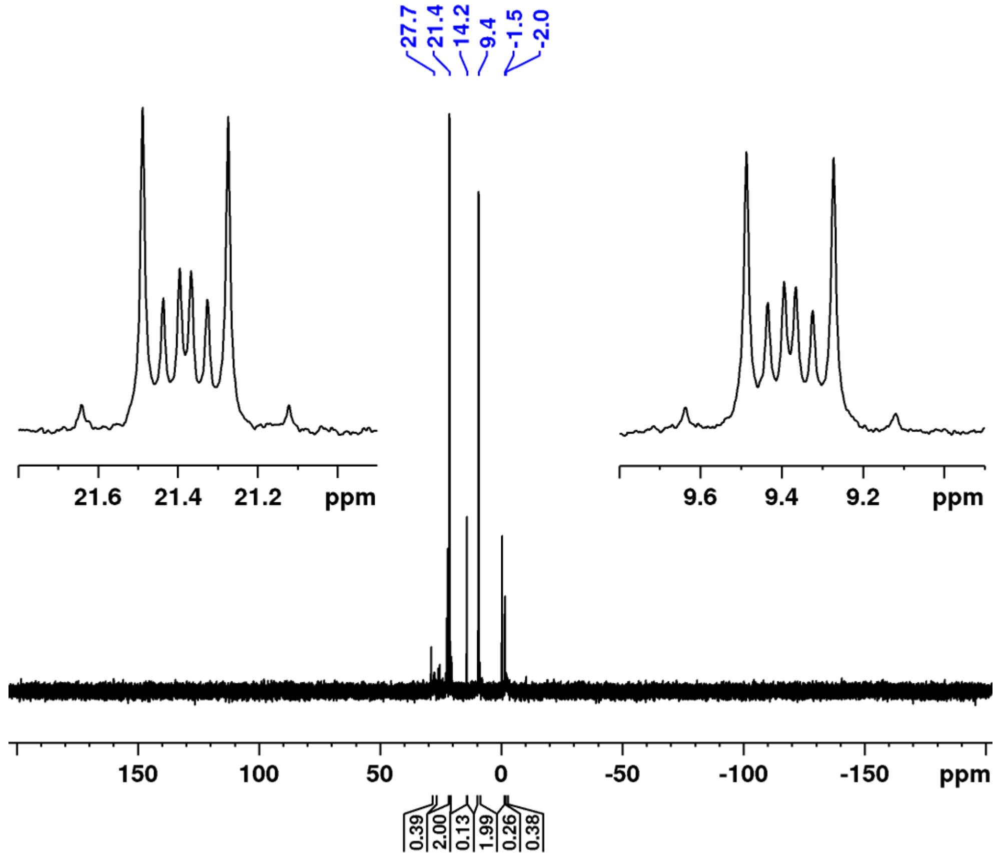

Figure S 57: $^{31}P\{^{1}H\}$ NMR spectrum (161.99 MHz, *o*DFB, 298 K) of a mixture of [{Ga(dcpe)}$_2$][*pf*]$_2$ and HOEt in *o*DFB.

4.4 [H{Ga(dcpe)}$_2$(OPh)][*pf*]$_2$

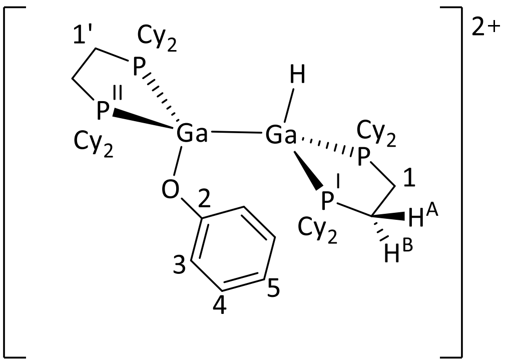

^{1}H NMR [300.18 MHz, *o*DFB, calibrated at *o*C$_6$F$_2$**H**$_4$ = 6.96 ppm, 298 K]: δ = 7.32 (m, 2 H, C^4**H**), ca. 6.92 (m, 1 H, C^5**H**), 6.77 (m, 2 H, C^3**H**), 4.78 (t, 1 H, Ga**H**, $^2J_{H,P}$ = 20.6 Hz), 2.54−2.44 (m, 8 H, C^1**H**^{A}, C^1**H**^{B}, C$^{1'}$**H**^{A}, C$^{1'}$**H**^{B}), 2.50−2.16 (m, 8 H, C**H** protons of the Cy groups), 2.21−1.12 (m, 80 H, C**H**$_2$ protons of the Cy groups) ppm.

^{13}C NMR [75.48 MHz, *o*DFB, 298 K]: δ = 160.8 (1 C, **C**^{2}), 130.0 (2 C, **C**^{4}), 119.7 (1 C, **C**^{5}), 118.0 (2 C, **C**^{3}), 33.1−31.5 (8 C, **C**H carbon atoms of the Cy groups), 29.1−25.0 (40 C, **C**H$_2$ carbon atoms on the Cy groups), 16.9−15.1 (4 C, **C**^{1} and **C**$^{1'}$) ppm.

^{19}F NMR [282.45 MHz, *o*DFB, 298 K]: δ = −75.3 (s, 36 F, [Al(OC(C**F**$_3$)$_3$)$_4$]$^-$), −113.9 (m, Ph**F**), −139.5 (m, *o*C$_6$**F**$_2$H$_4$) ppm.

^{27}Al NMR [78.22 MHz, *o*DFB, 298 K]: δ = 35.0 (s, 1 Al, [Al(OC(C**F**$_3$)$_3$)$_4$]$^-$) ppm.

^{31}P NMR [121.51 MHz, *o*DFB, 298 K]: δ = 20.7 (m, 2 P, **P**^{I}), 10.5 (m, 2 P, **P**^{II}) ppm.

^{71}Ga NMR [91.54 MHz, *o*DFB, 298 K]: signal probably too broad to be detected, due to the quadrupolar relaxation of ^{71}Ga.

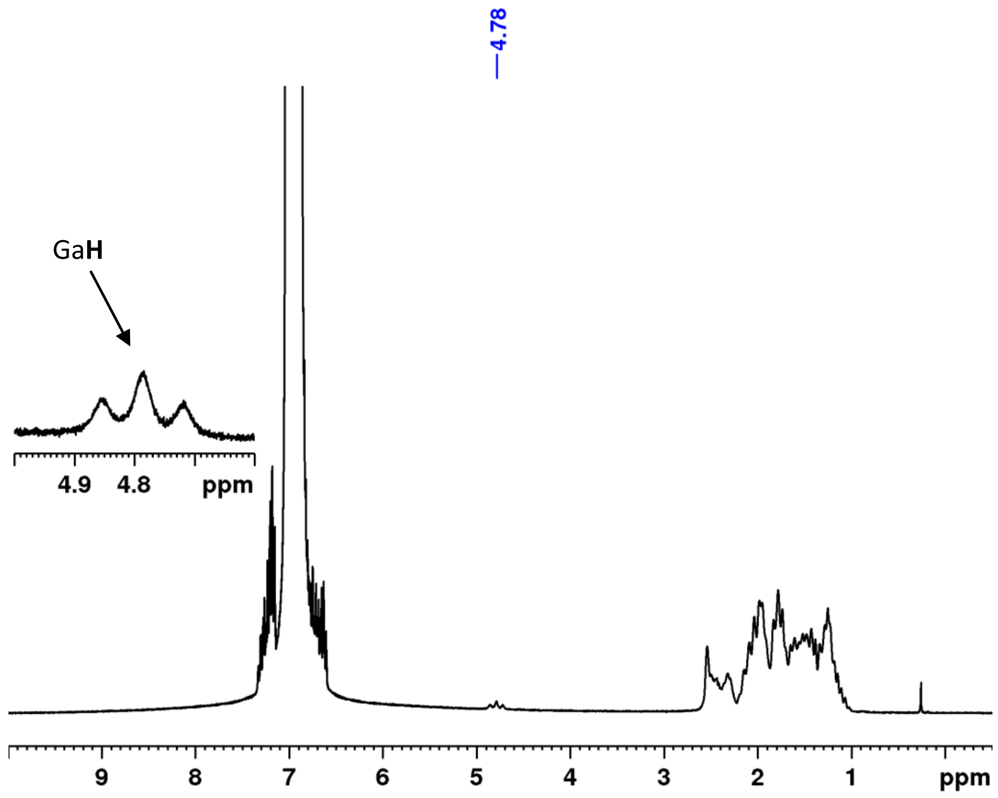

Figure S 58: ^{1}H NMR spectrum (300.18 MHz, *o*DFB, 298 K) of a mixture of [{Ga(dcpe)}$_2$][*pf*]$_2$ and PhOH in oDFB.

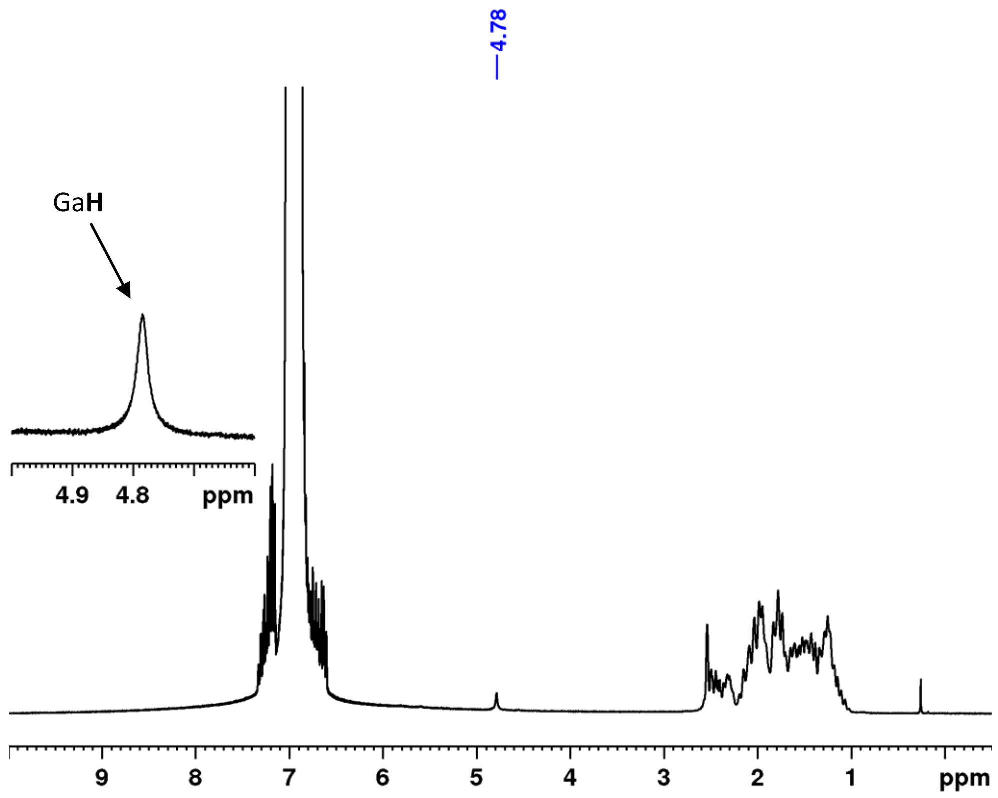

Figure S 59: $^{1}H\{^{31}P\}$ NMR spectrum (300.18 MHz, *o*DFB, 298 K) of a mixture of [{Ga(dcpe)}$_2$][*pf*]$_2$ and PhOH in *o*DFB.

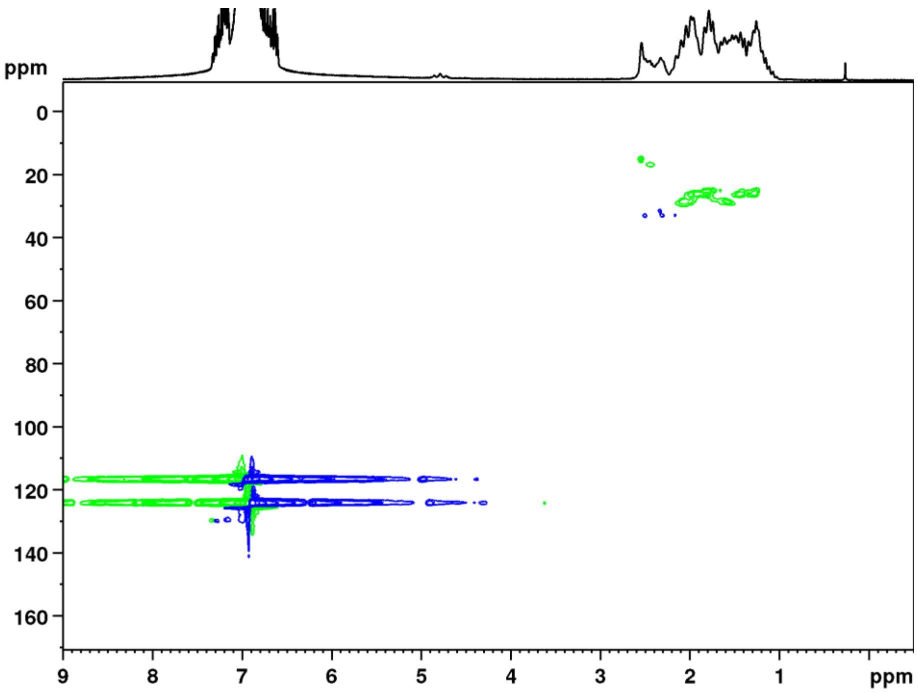

Figure S 60: Edited $^{1}H,^{13}C$-HSQC NMR spectrum (300.18 MHz, *o*DFB, 298 K, optimized for $J = 145$ Hz) of a mixture of [{Ga(dcpe)}$_2$][*pf*]$_2$ and PhOH in *o*DFB.

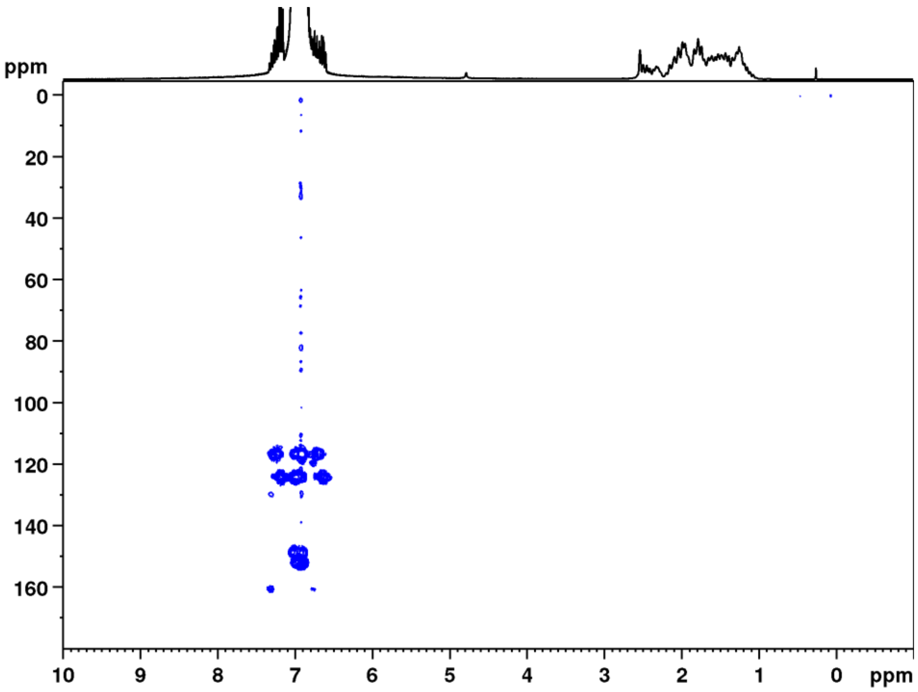

Figure S 61: $^{1}H,^{13}C$-HMBC NMR spectrum (300.18 MHz, *o*DFB, 298 K, optimized for $J = 8$ Hz) of a mixture of [{Ga(dcpe)}$_2$][*pf*]$_2$ and PhOH in *o*DFB.

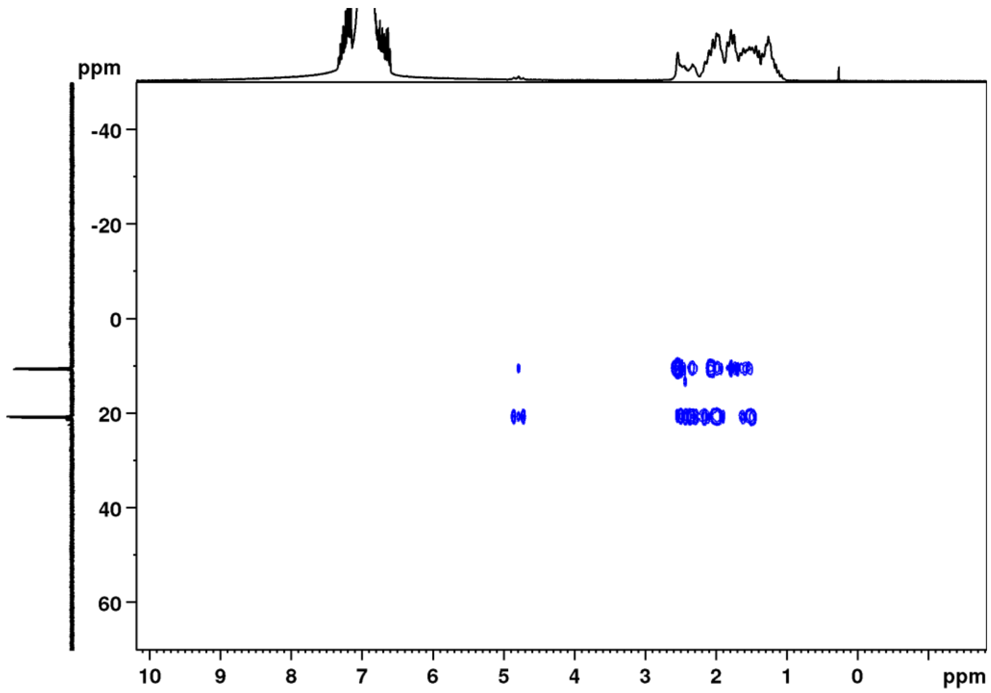

Figure S 62: $^{1}H,^{31}P$-HMBC NMR spectrum (300.18 MHz, *o*DFB, 298 K, optimized for J = 15 Hz) of a mixture of [{Ga(dcpe)}$_2$][*pf*]$_2$ and PhOH in *o*DFB.

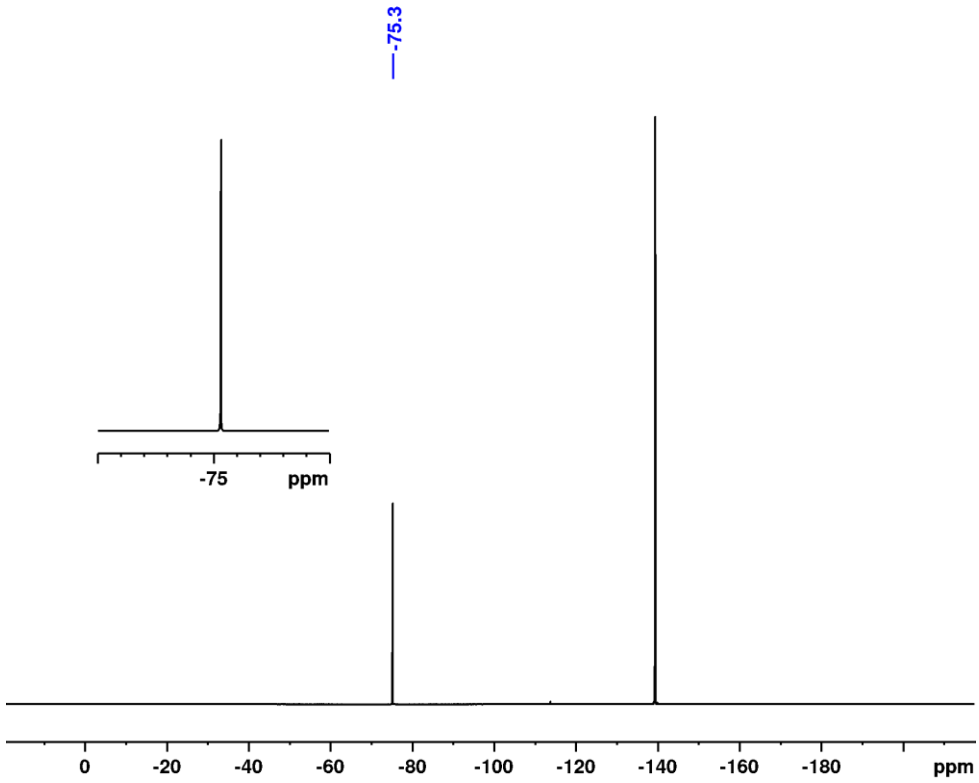

Figure S 63: ^{19}F NMR spectrum (282.45 MHz, *o*DFB, 298 K) of a mixture of [{Ga(dcpe)}$_2$][*pf*]$_2$ and PhOH in *o*DFB.

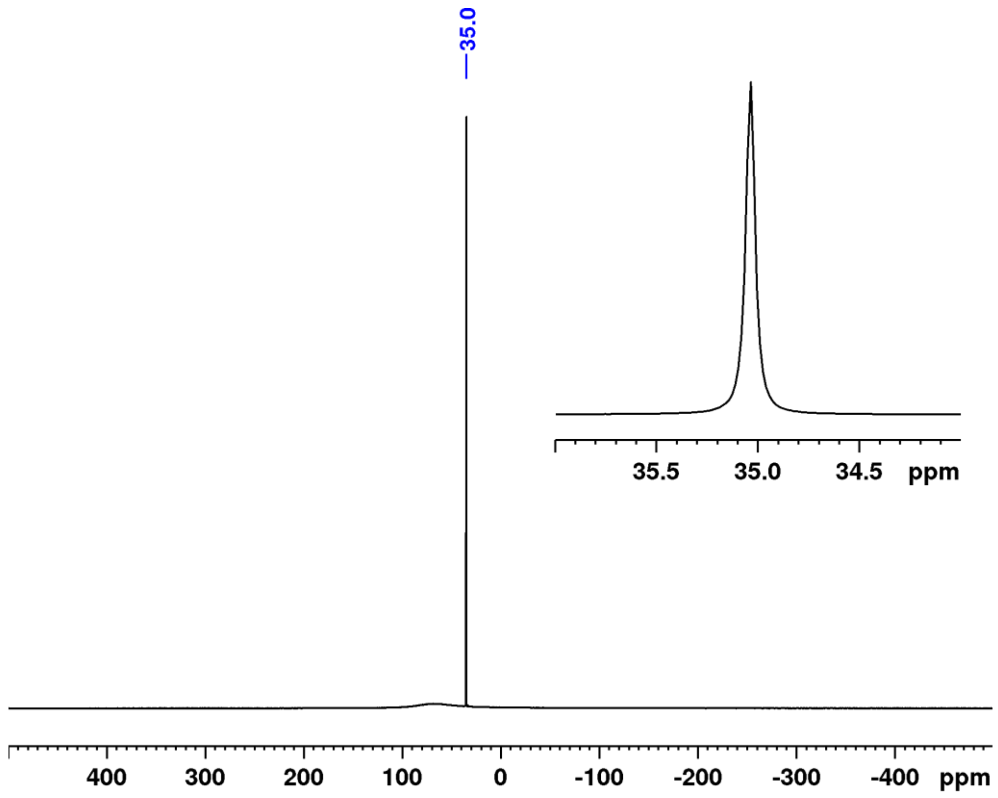

Figure S 64: ^{27}Al NMR spectrum (78.22 MHz, oDFB, 298 K) of a mixture of [{Ga(dcpe)}$_2$][*pf*]$_2$ and PhOH in *o*DFB.

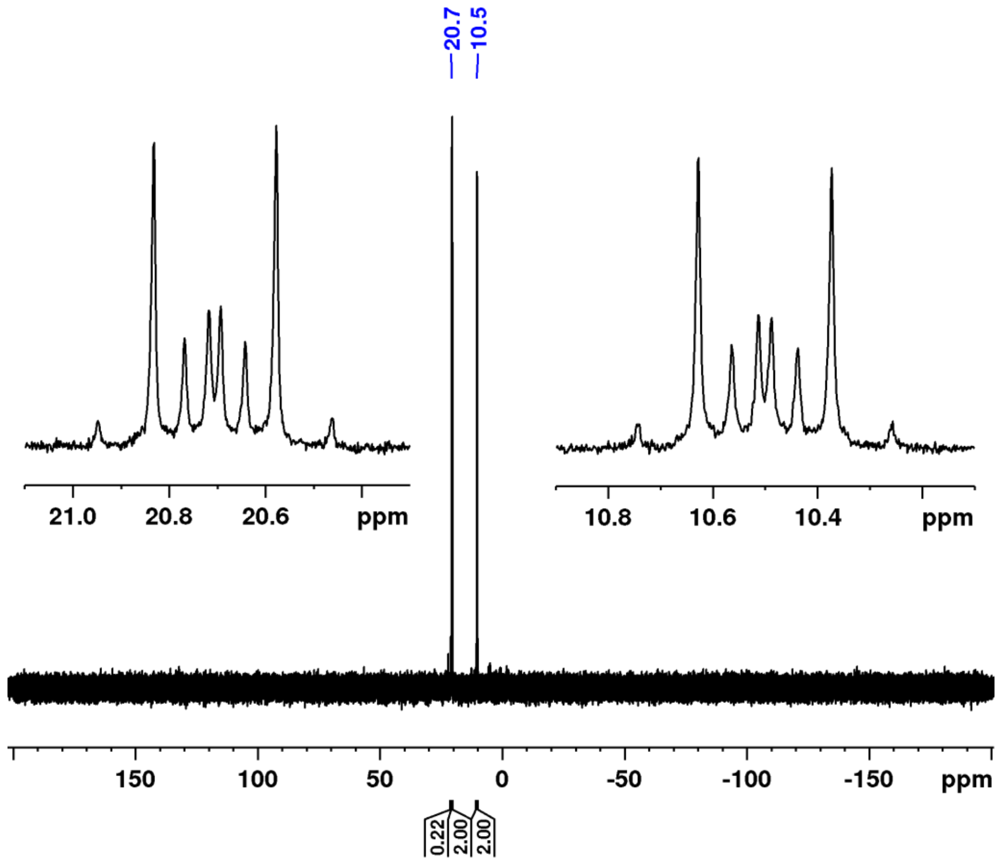

Figure S 65: ^{31}P{^{1}H} NMR spectrum (121.51 MHz, oDFB, 298 K) of a mixture of [{Ga(dcpe)}$_2$][*pf*]$_2$ and PhOH in oDFB, 5 h after mixing the components.

4.4.1 Observed Intermediates during the Formation of $[H\{Ga(dcpe)\}_2OPh]^{2+}$

During the H–O bond activation with HOPh, one intermediate is observed in the $^{31}P\{^1H\}$ NMR spectrum, when no more $\mathbf{1}^{2+}$ is present in solution. While the concentration of the dimer increases, the intermediate is consumed after ca. 1 h (*cf.* **Figure S 66** and **Figure S 65**). It is characterized by a singlet in the $^{31}P\{^1H\}$ NMR spectrum (δ (^{31}P) = 13.4 ppm) and $J_{H,P}$ coupling constants of ca. 27 Hz. The ^1H signal of the corrsponding Ga**H** proton at ca. 5.25 ppm is remarkably broad, so that it cannot be determined whether the ^1H signal has a triplet or quintet splitting pattern in the non-decoupled ^1H NMR spectrum. In summary, the fact that this compound produces a singlet in the $^{31}P\{^1H\}$ NMR spectrum and is directly converted to the final Ga^{II} addition product suggests that this intermediate has a symmetric, but dimeric structure. A plausible structure is presented in **Figure S 66**.

Nevertheless, taking into account that the ^{31}P NMR and ^1H NMR shift of the **P**–Ga–**H** units in the intermediates observed during the reactions with HOPh, H_2NPh and $HNPh_2$ (sections 4.4.1, 4.6.1 and 4.7.1) is very similar, it cannot be ruled out that the OPh group acts as a bridging ligand with its aromatic π-system or that the OPh group is only weakly associated.

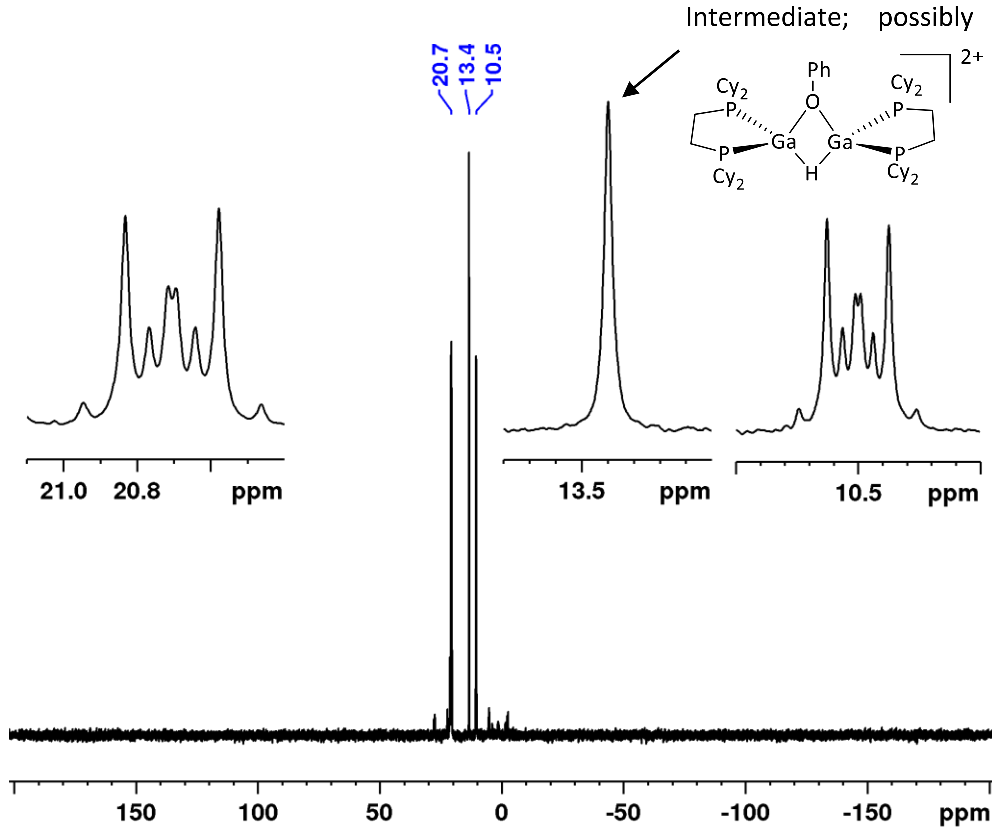

Figure S 66: $^{31}P\{^1H\}$ NMR spectrum (121.51 MHz, oDFB, 298 K) of a mixture of $[\{Ga(dcpe)\}_2][pf]_2$ and PhOH in oDFB, 30 minutes after mixing the components.

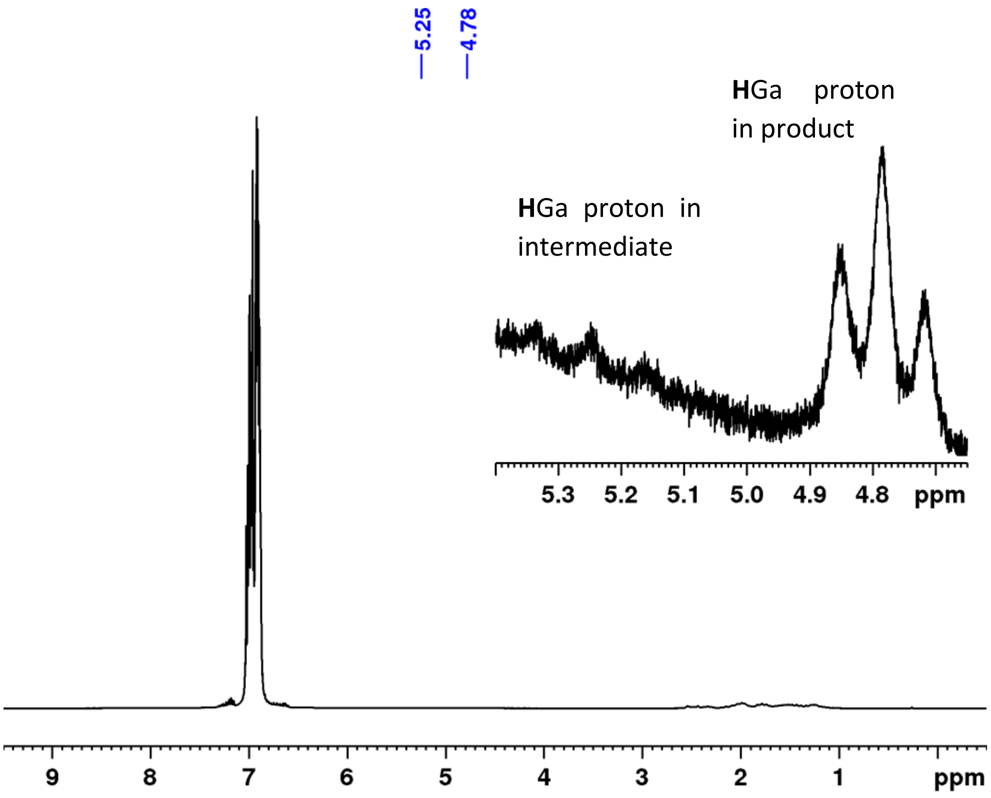

Figure S 67: ^{1}H NMR spectrum (300.18 MHz, oDFB, 298 K) of a mixture of [{Ga(dcpe)}$_2$][*pf*]$_2$ and PhOH in oDFB, 30 minutes after mixing the components.

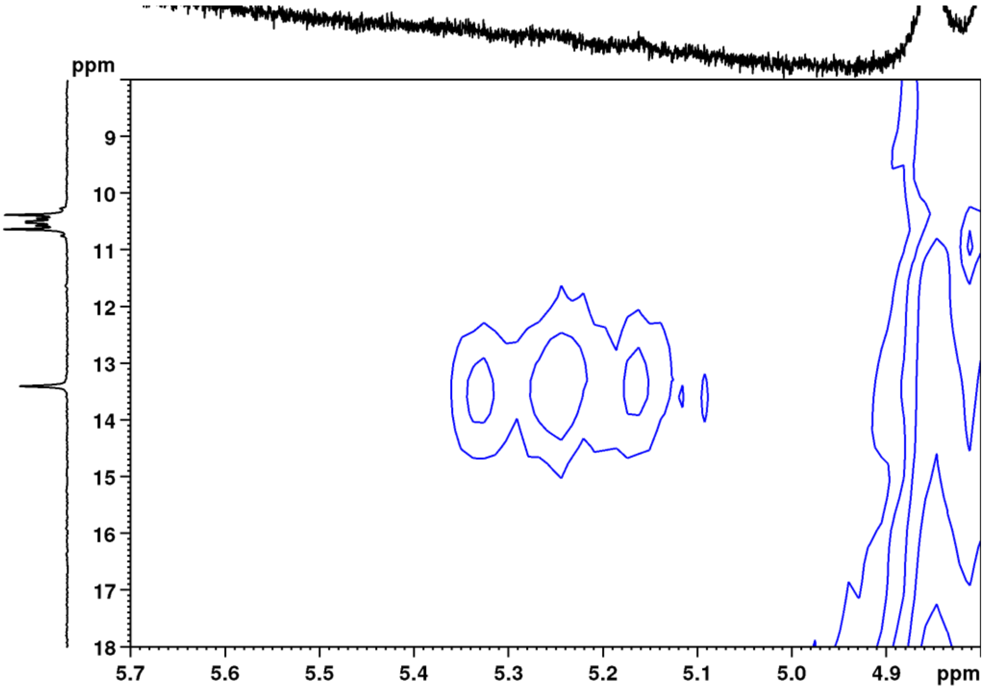

Figure S 68: ^{1}H,^{31}P-HMBC NMR spectrum (300.18 MHz, *o*DFB, 298 K, optimized for J = 15 Hz) of a mixture of [{Ga(dcpe)}$_2$][*pf*]$_2$ and PhOH in *o*DFB, 2 h after mixing the components.

4.5 [H{Ga(dcpe)}$_2$(NH*n*Bu)][*pf*]$_2$

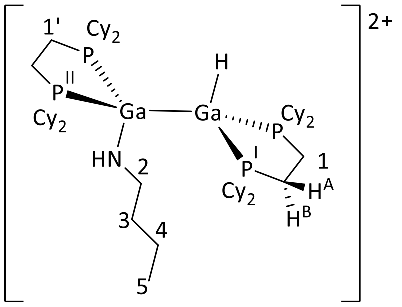

^{1}H NMR [400.17 MHz, *o*DFB, calibrated at *o*C_6F_2**H_4** = 6.96 ppm, 298 K]: δ = 4.55 (t, 1 H, Ga**H**, $^{2}J_{H,P}$ = 19.6 Hz), 3.05 (m, 2 H, C^2**H_2**, $^{3}J_{H,H}$ = 7.0 Hz), 2.49–2.37 (m, 8 H, C^1**H^A**, C^1**H^B**, $C^{1'}$**H^A**, $C^{1'}$**H^B**), 2.41–2.15 (m, 8 H, C**H** protons of the Cy groups), 2.21–1.18 (m, 80 H, C**H_2** protons of the Cy groups), 1.66–1.60 (m, 2 H, C^3**H_2**), 1.47–1.40 (m, 2 H, C^4**H_2**), 0.97 (t, 3 H, C^5**H_3**, $^{3}J_{H,H}$ = 7.5 Hz), 0.29 (m, 1 H, N–**H**, $^{3}J_{H,H}$ = 6.9 Hz)

^{13}C NMR [100.62 MHz, *o*DFB, 298 K]: δ = 50.3 (1 C, **C^2**), 38.0 (1 C, **C^3**), 20.1 (1 C, **C^4**), 35.0–30.7 (8 C, **C**H carbon atoms on the Cy groups), 30.7–24.1 (40 C, **C**H_2 carbon atoms on the Cy groups), 17.6–14.0 (4 C, **C^1** and **$C^{1'}$**), 13.2 (1 C, **C^5**) ppm.

^{19}F NMR [376.54 MHz, *o*DFB, 298 K]: δ = −75.3 (s, 36 F, $[Al(OC(C\mathbf{F}_3)_3)_4]^-$), −113.9 (m, Ph**F**), −139.5 (m, *o*C_6**F_2**H_4) ppm.

^{27}Al NMR [104.27 MHz, *o*DFB, 298 K]: δ = 35.0 (s, 1 Al, $[Al(OC(C\mathbf{F}_3)_3)_4]^-$) ppm.

^{31}P NMR [161.99 MHz, *o*DFB, 298 K]: δ = 21.0 (m, 2 P, **P^I**), 5.7 (m, 2 P, **P^{II}**) ppm.

^{71}Ga NMR [122.03 MHz, *o*DFB, 298 K]: signal probably too broad to be detected, due to the quadrupolar relaxation of ^{71}Ga.

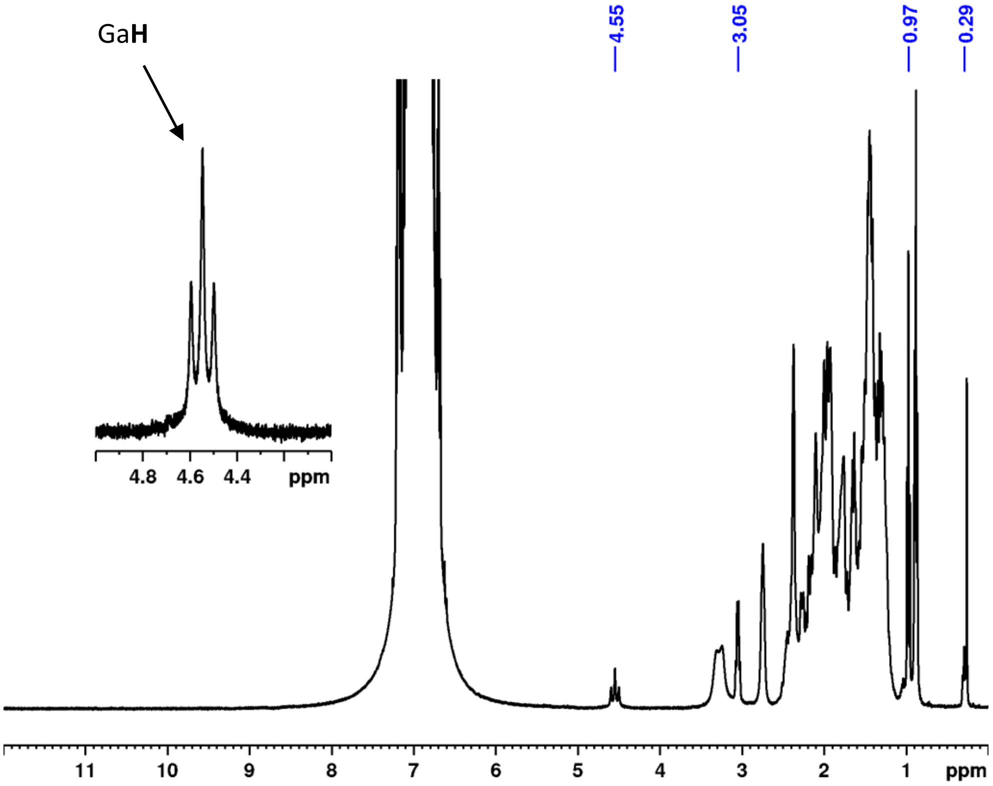

Figure S 69: ^{1}H NMR spectrum (400.17 MHz, *o*DFB, 298 K) of a mixture of [{Ga(dcpe)}$_2$][*pf*]$_2$ and NH_2*n*Bu in *o*DFB at room temperature, 0.5 h after mixing the components.

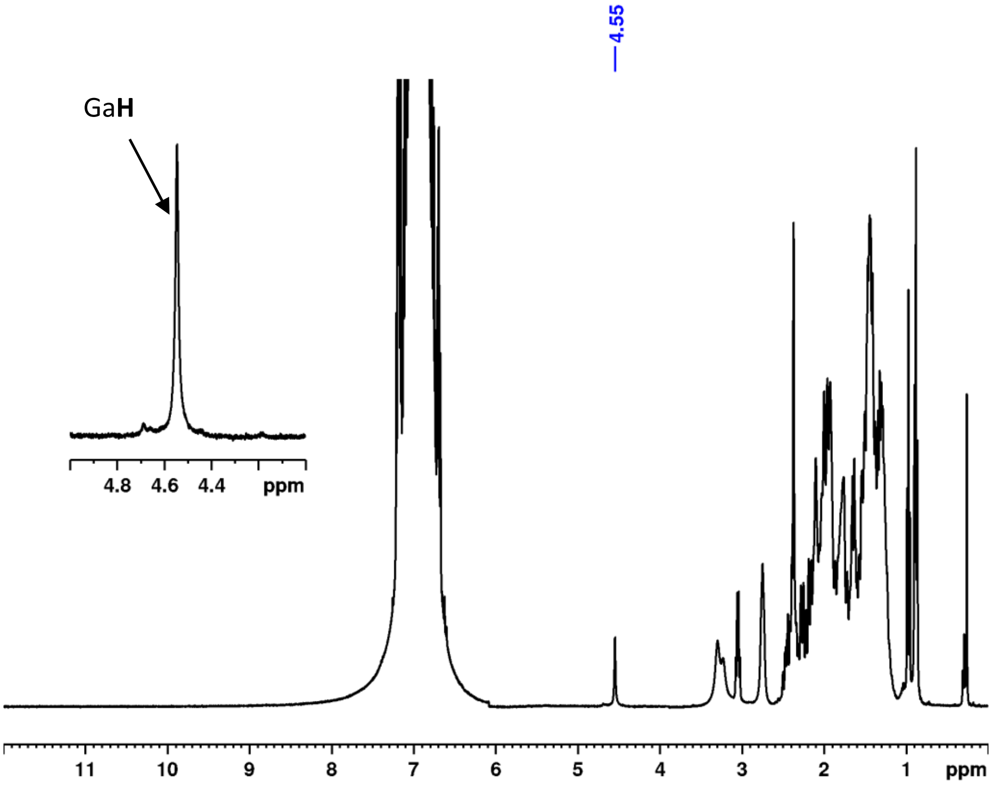

Figure S 70: $^{1}H\{^{31}P\}$ NMR spectrum (400.17 MHz, *o*DFB, 298 K) of a mixture of [{Ga(dcpe)}$_2$][*pf*]$_2$ and NH_2*n*Bu in *o*DFB at room temperature, 0.5 h after mixing the components.

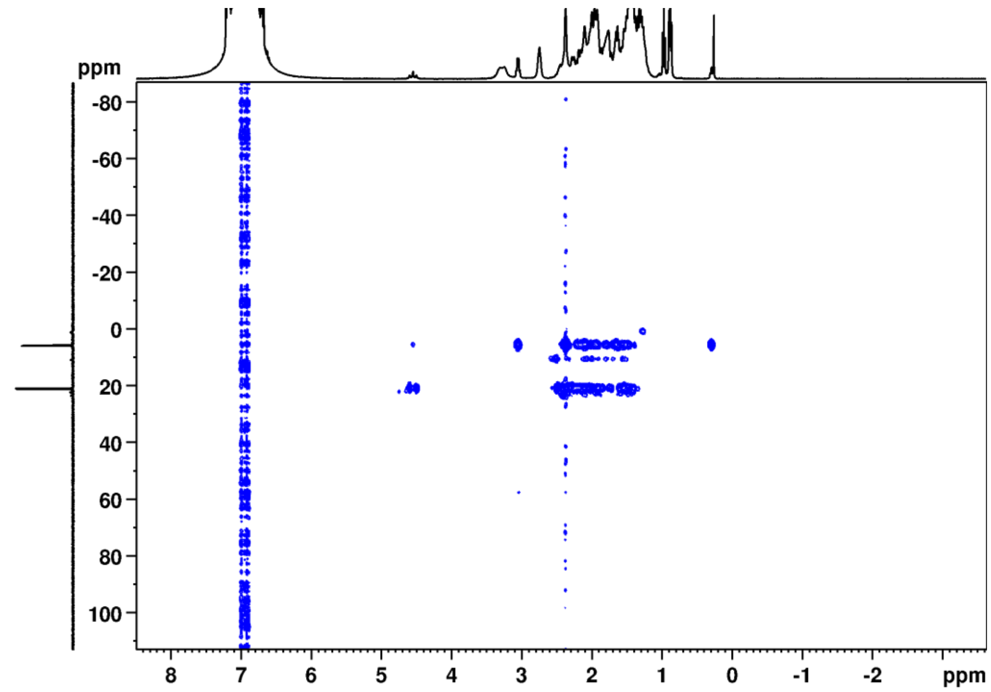

Figure S 71: ^{1}H,^{31}P-HMBC NMR spectrum (400.17 MHz, *o*DFB, 298 K, optimized for *J* = 15 Hz) of a mixture of [{Ga(dcpe)}$_2$][*pf*]$_2$ and NH_2*n*Bu in *o*DFB at room temperature, 0.5 h after mixing the components.

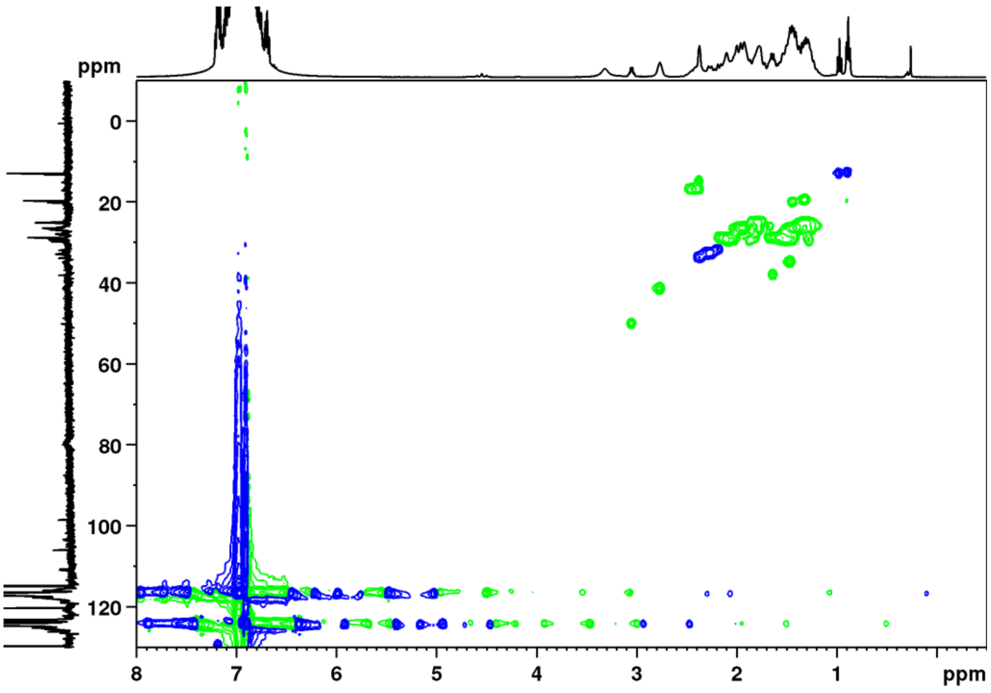

Figure S 72: Edited ^{1}H,^{13}C-HSQC NMR spectrum (400.17 MHz, *o*DFB, 298 K, optimized for *J* = 145 Hz) of a mixture of [{Ga(dcpe)}$_2$][*pf*]$_2$ and NH_2*n*Bu in *o*DFB at room temperature, 0.5 h after mixing the components.

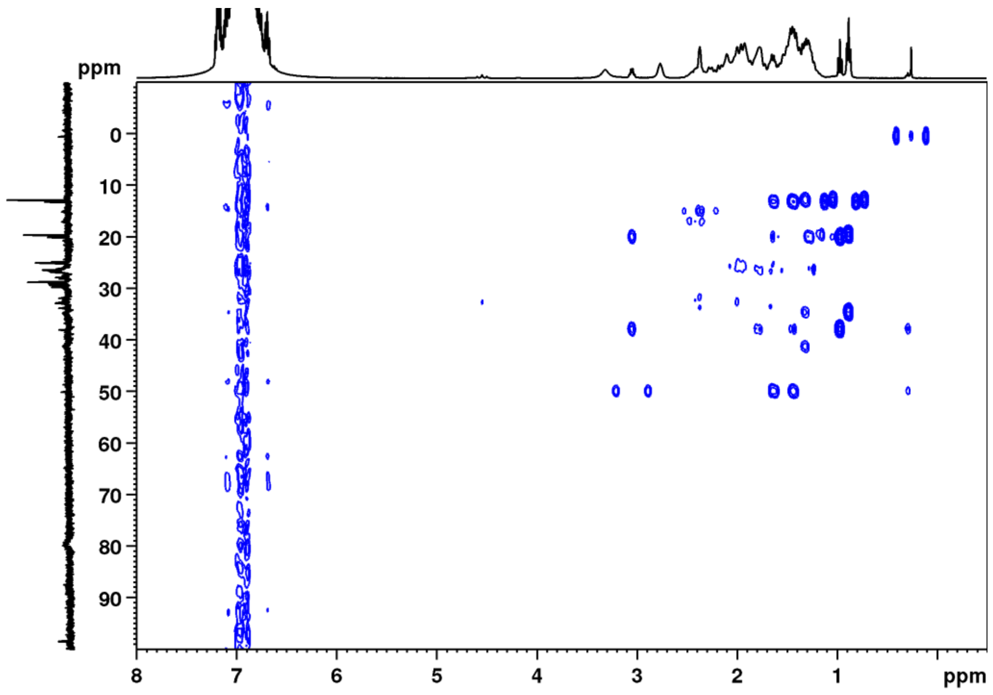

Figure S 73: $^{1}H,^{13}C$-HMBC NMR spectrum (400.17 MHz, *o*DFB, 298 K, optimized for J = 8 Hz) of a mixture of $[\{Ga(dcpe)\}_2][pf]_2$ and NH_2nBu in *o*DFB at room temperature, 0.5 h after mixing the components.

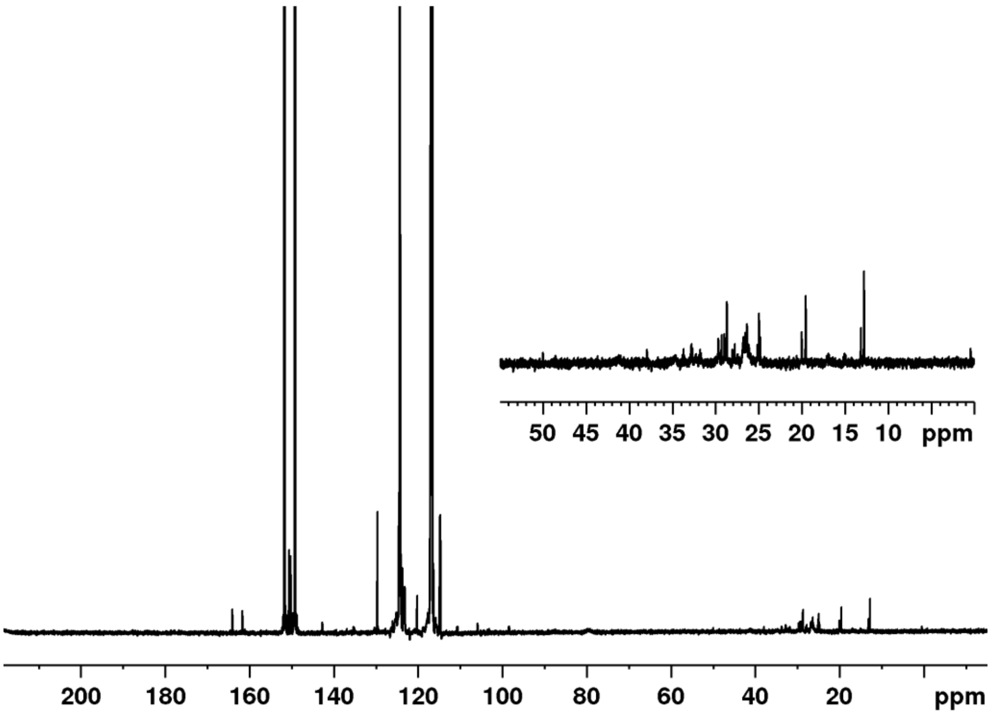

Figure S 74: $^{13}C\{^{1}H\}$ NMR spectrum (100.62 MHz, *o*DFB, 298 K) of a mixture of $[\{Ga(dcpe)\}_2][pf]_2$ and NH_2nBu in *o*DFB at room temperature, 0.5 h after mixing the components.

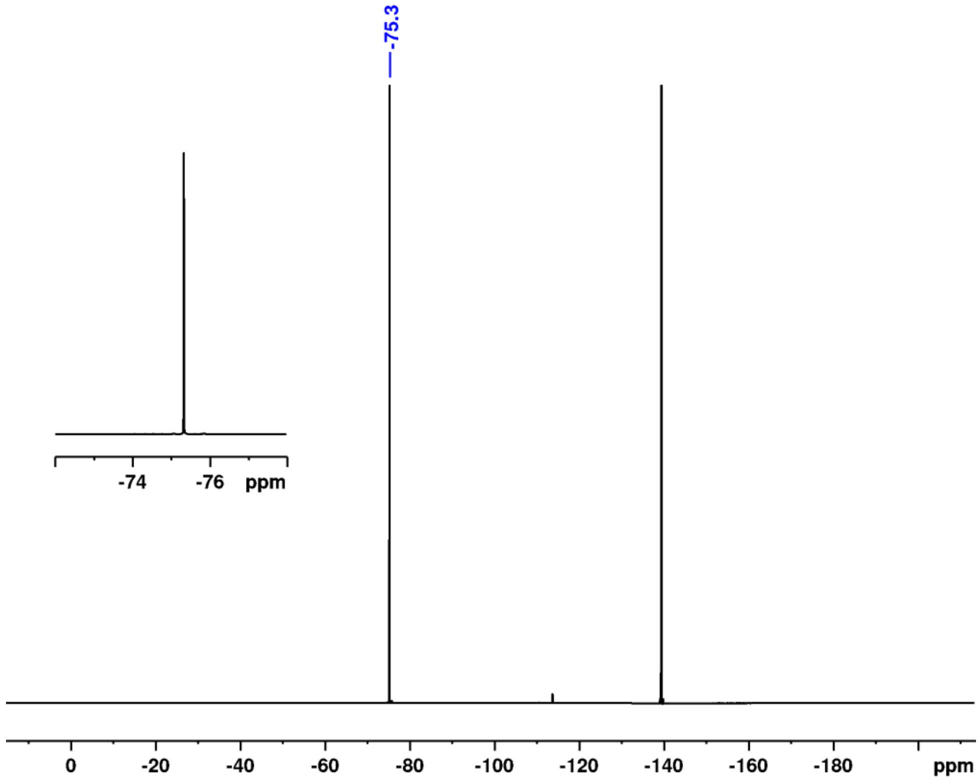

Figure S 75:^{19}F NMR spectrum (376.53 MHz, *o*DFB, 298 K) of a mixture of [{Ga(dcpe)}$_2$][*pf*]$_2$ and NH_2*n*Bu in *o*DFB at room temperature, 0.5 h after mixing the components.

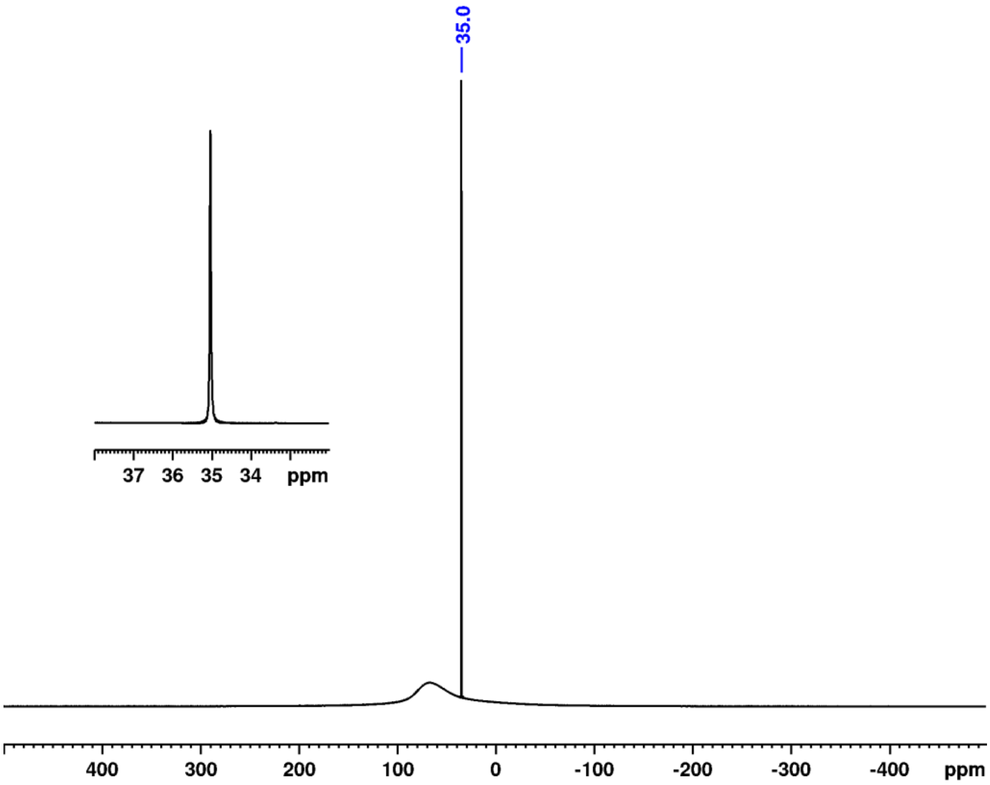

Figure S 76: ^{27}Al NMR spectrum (104.27 MHz, oDFB, 298 K) of a mixture of [{Ga(dcpe)}$_2$][*pf*]$_2$ and NH_2nBu in *o*DFB at room temperature, 0.5 h after mixing the components.

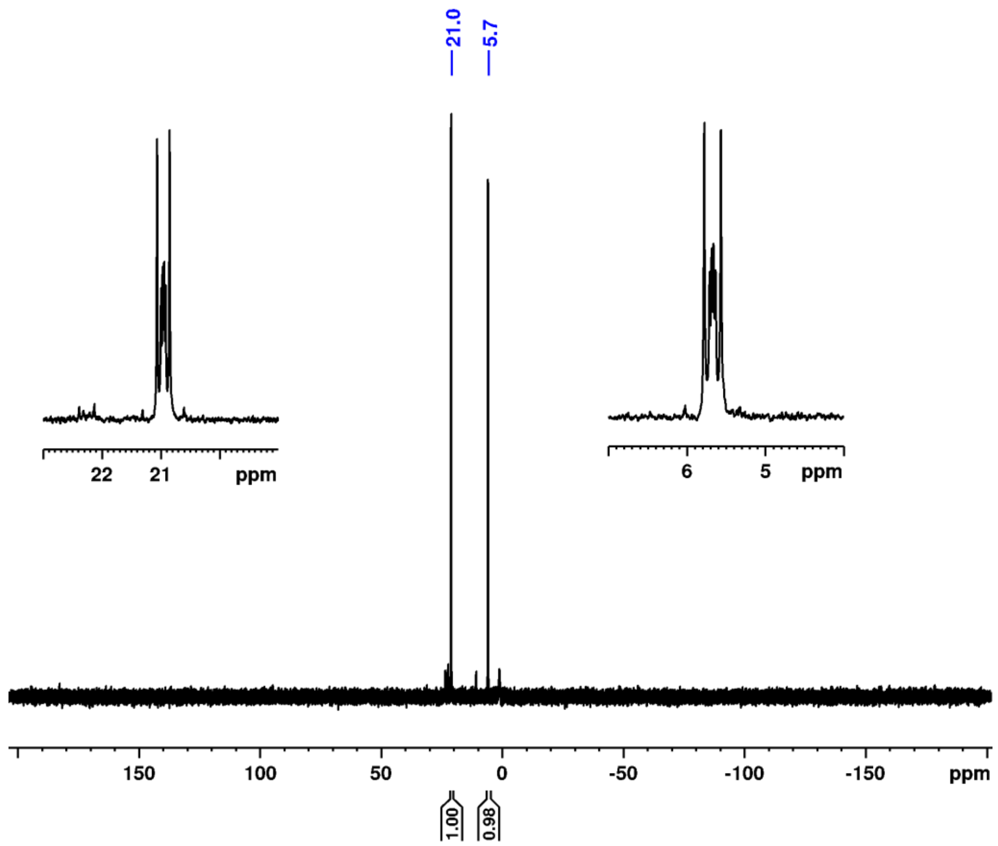

Figure S 77: $^{31}P\{^1H\}$ NMR spectrum (161.99 MHz, *o*DFB, 298 K) of a mixture of $[\{Ga(dcpe)\}_2][pf]_2$ and NH_2*n*Bu in *o*DFB at room temperature, 0.5 h after mixing the components.

4.6 [H{Ga(dcpe)}$_2$(NHPh)][*pf*]$_2$

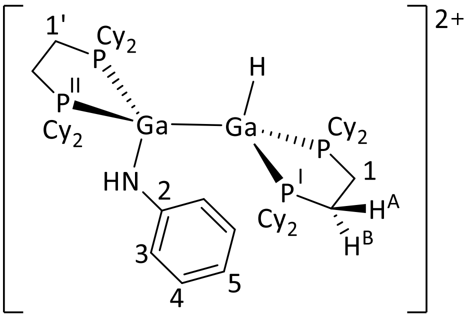

^{1}H NMR [300.18 MHz, *o*DFB, calibrated at *o*C_6F_2**H_4** = 6.96 ppm, 298 K]: δ = 7.20 (m, 2 H, C^4**H**), 6.73 (m, 1 H, C^5**H**), 6.62 (m, 2 H, C^3**H**), 4.73 (t, 1 H, Ga**H**, $^{2}J_{H,P}$ = 20.4 Hz), 3.30 (m, 1 H, N–**H**), 2.50−2.33 (m, 8 H, C^1**H^A**, C^1**H^B**, $C^{1'}$**H^A**, $C^{1'}$**H^B**), 2.42−2.24 (m, 8 H, C**H** protons of the Cy groups), 2.05−1.27 (m, 80 H, C**H_2** protons of the Cy groups) ppm.

^{13}C NMR [75.48 MHz, *o*DFB, 298 K]: δ = 150.9 (1 C, **C^2**), 129.6 (2 C, **C^4**), 117.9 (1 C, **C^5**), 115.3 (2 C, **C^3**), 33.4−31.7 (8 C, **C**H carbon atoms of the Cy groups), 29.1−25.3 (40 C, **C**H_2 carbon atoms on the Cy groups), 17.0−15.4 (4 C, **C^1** and **$C^{1'}$**) ppm.

^{19}F NMR [282.45 MHz, *o*DFB, 298 K]: δ = −75.3 (s, 36 F, $[Al(OC(C\mathbf{F}_3)_3)_4]^-$), −113.8 (m, Ph**F**), −139.5 (m, *o*C_6**F_2**H_4) ppm.

^{27}Al NMR [78.22 MHz, *o*DFB, 298 K]: δ = 35.0 (s, 1 Al, $[Al(OC(C\mathbf{F}_3)_3)_4]^-$) ppm.

^{31}P NMR [121.52 MHz, *o*DFB, 298 K]: δ = 20.1 (m, 2 P, **P^I**), 8.5 (m, 2 P, **P^{II}**) ppm.

^{71}Ga NMR [91.54 MHz, *o*DFB, 298 K]: signal probably too broad to be detected, due to the quadrupolar relaxation of ^{71}Ga.

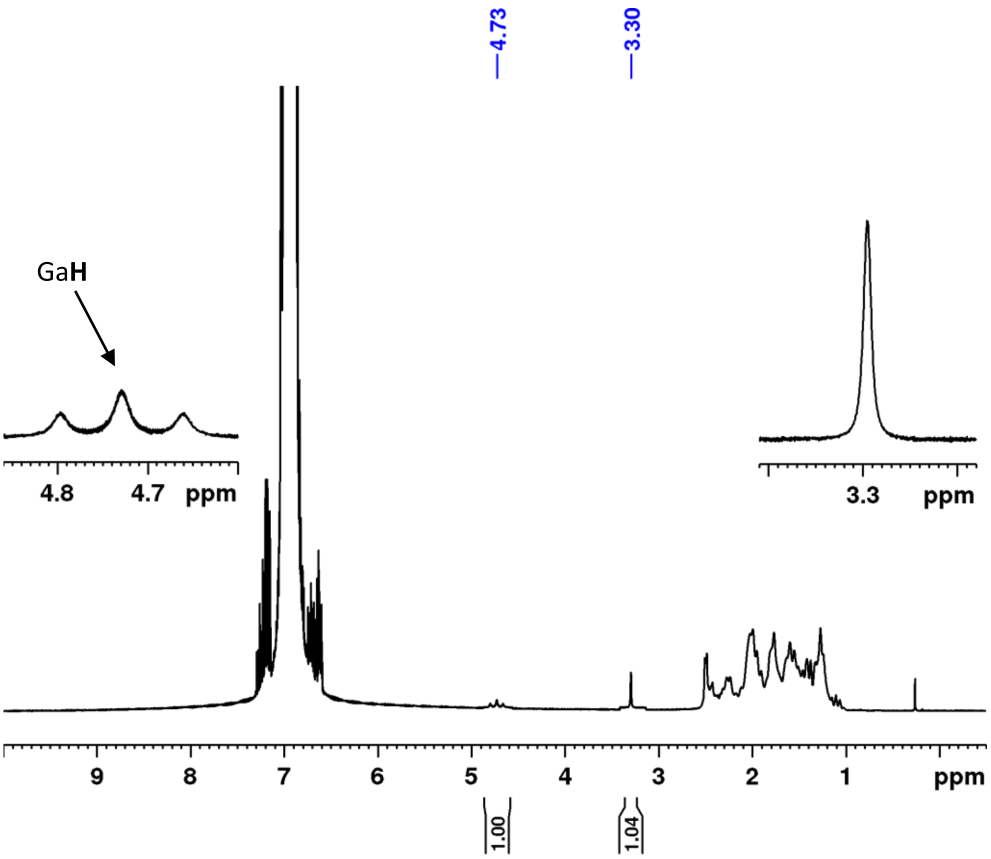

Figure S 78: ^{1}H NMR spectrum (300.18 MHz, *o*DFB, 298 K) of a mixture of [{Ga(dcpe)}$_2$][*pf*]$_2$ and NH_2Ph in *o*DFB.

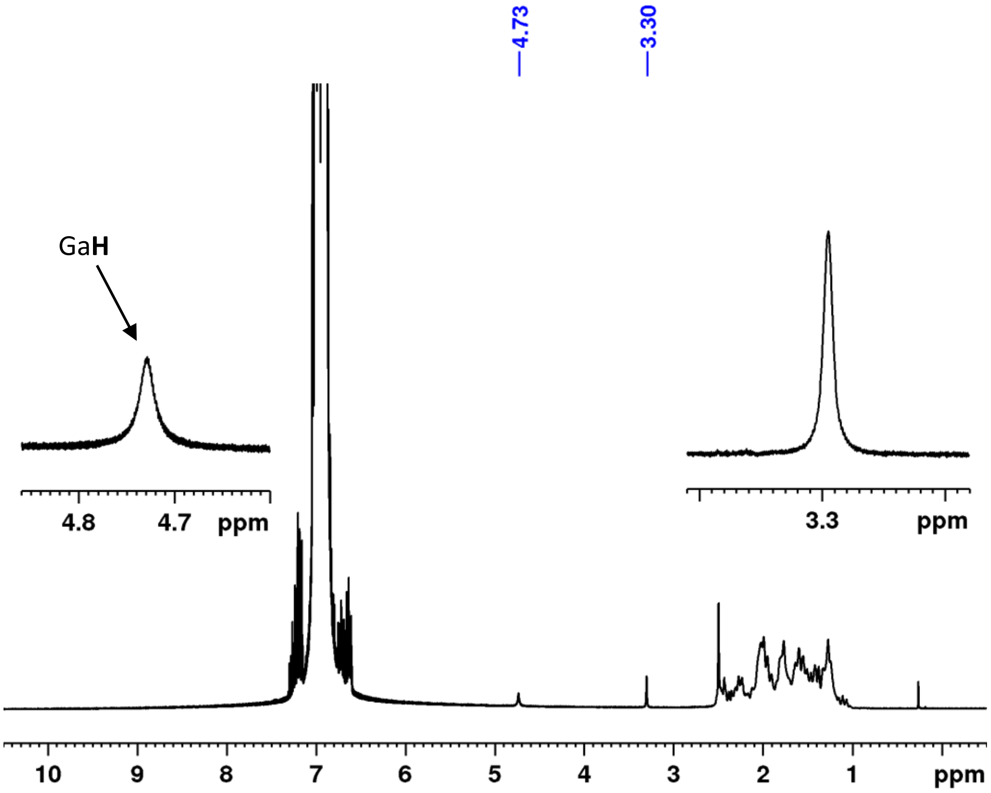

Figure S 79: $^{1}H\{^{31}P\}$ NMR spectrum (300.18 MHz, *o*DFB, 298 K) of a mixture of [{Ga(dcpe)}$_2$][*pf*]$_2$ and NH_2Ph in *o*DFB.

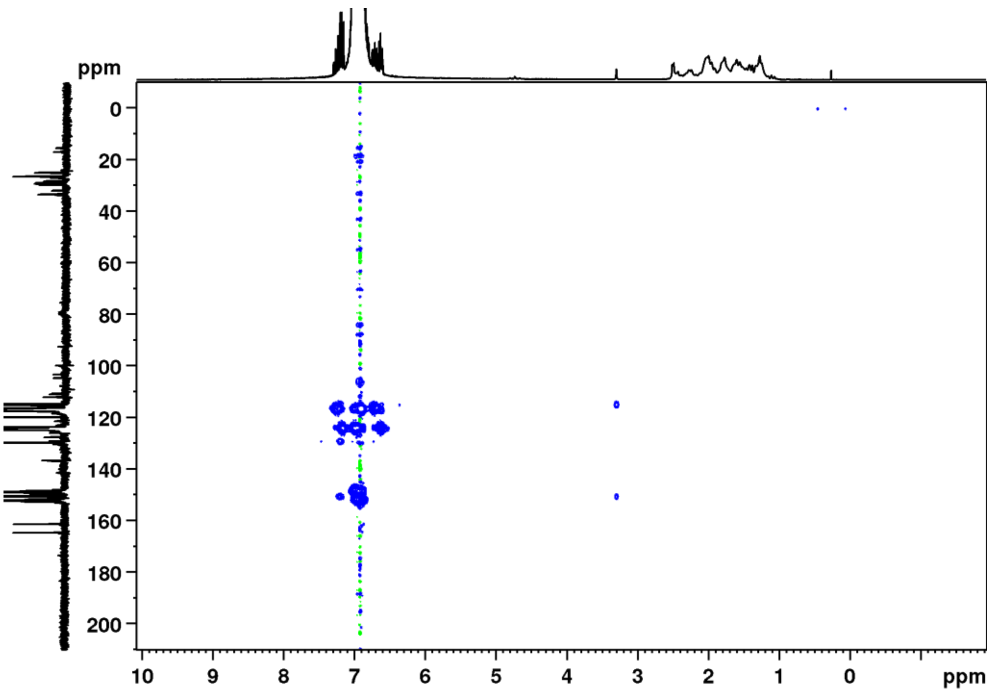

Figure S 80: $^{1}H,^{13}C$-HMBC NMR spectrum (300.18 MHz, *o*DFB, 298 K, optimized for *J* = 8.0 Hz) of a mixture of [{Ga(dcpe)}$_2$][*pf*]$_2$ and NH_2Ph in *o*DFB.

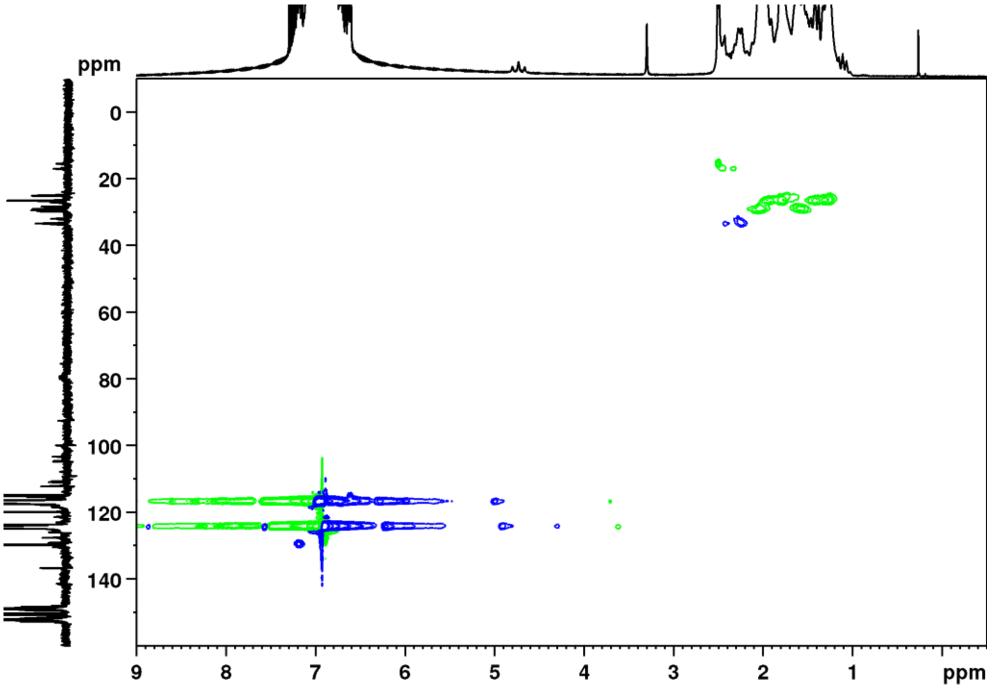

Figure S 81: Edited $^{1}H,^{13}C$-HSQC NMR spectrum (300.18 MHz, *o*DFB, 298 K, optimized for *J* = 145 Hz) of a mixture of [{Ga(dcpe)}$_2$][*pf*]$_2$ and NH_2Ph in *o*DFB.

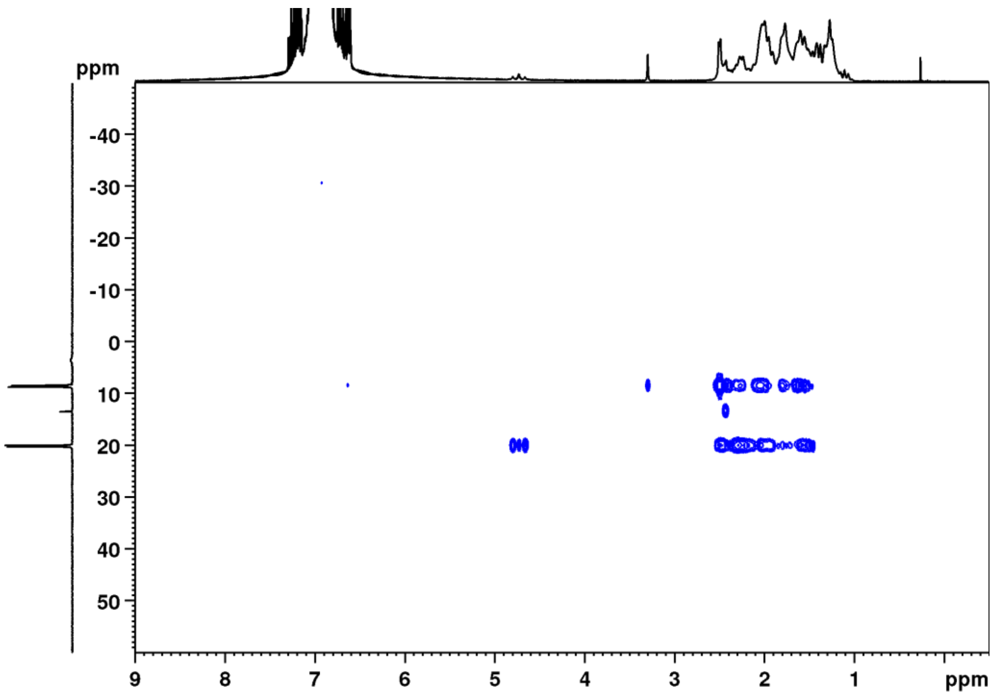

Figure S 82: $^{1}H,^{31}P$-HMBC NMR spectrum (300.18 MHz, *o*DFB, 298 K, optimized for J = 15 Hz) of a mixture of $[\{Ga(dcpe)\}_2][pf]_2$ and NH_2Ph in *o*DFB.

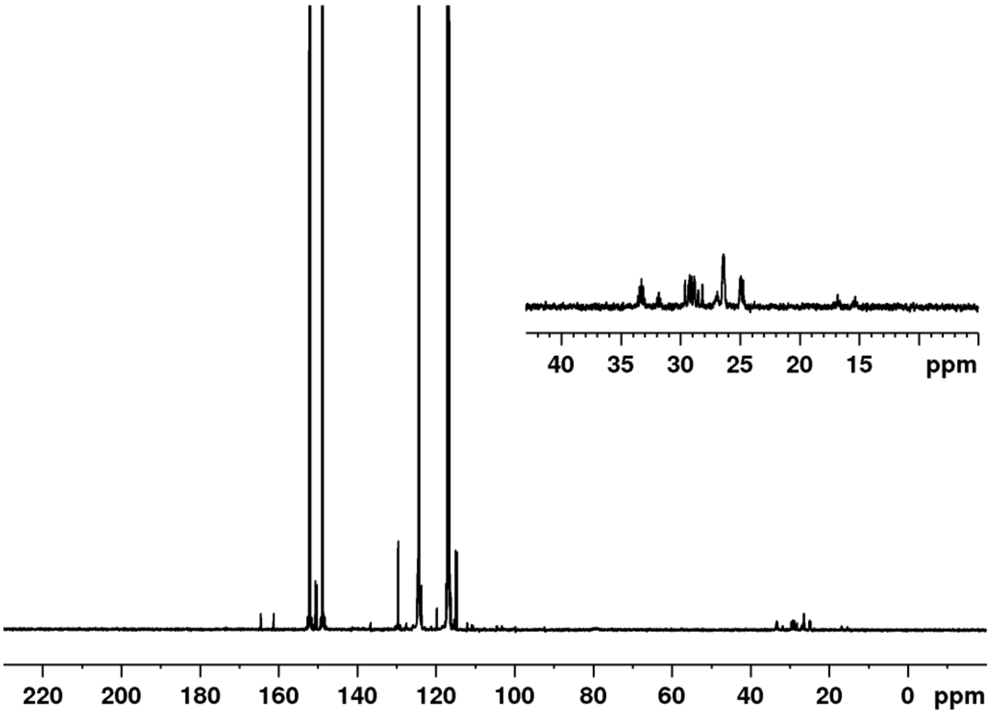

Figure S 83: $^{13}C\{^{1}H\}$ NMR spectrum (75.48 MHz, *o*DFB, 298 K) of a mixture $[\{Ga(dcpe)\}_2][pf]_2$ and NH_2Ph in *o*DFB.

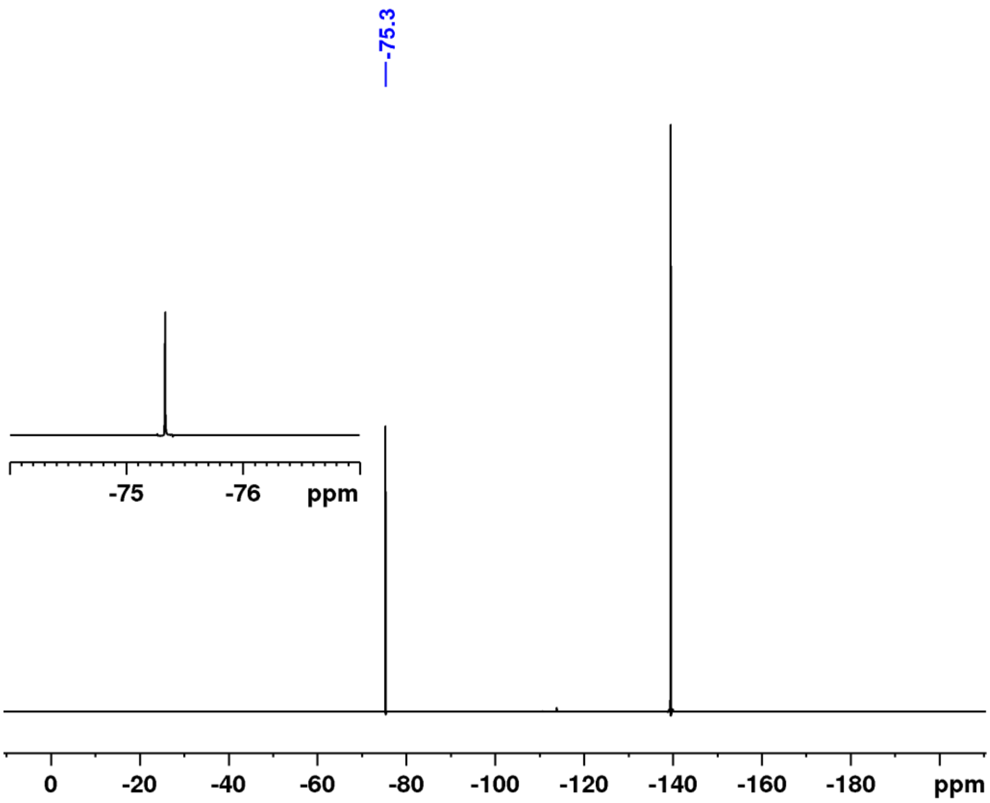

Figure S 84: ^{19}F NMR spectrum (188.31 MHz, *o*DFB, 298 K) of a mixture [{Ga(dcpe)}$_2$][*pf*]$_2$ and NH_2Ph in *o*DFB

.

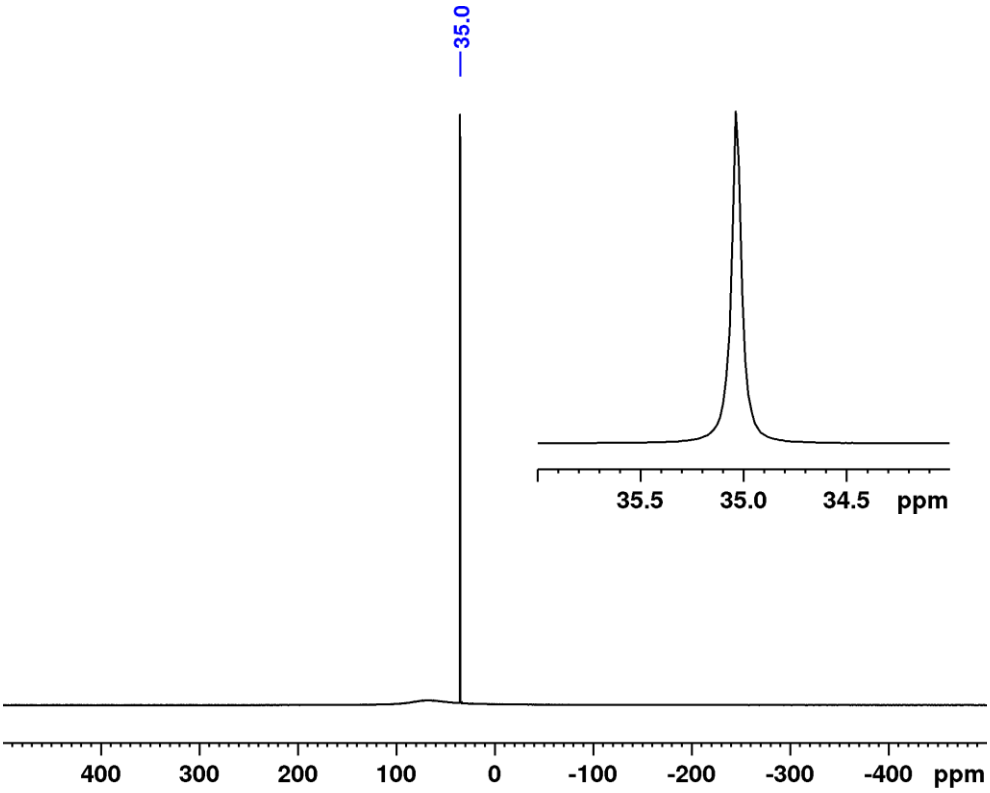

Figure S 85: ^{27}Al NMR spectrum (78.22 MHz, oDFB, 298 K) of a mixture [{Ga(dcpe)}$_2$][*pf*]$_2$ and NH_2Ph in *o*DFB.

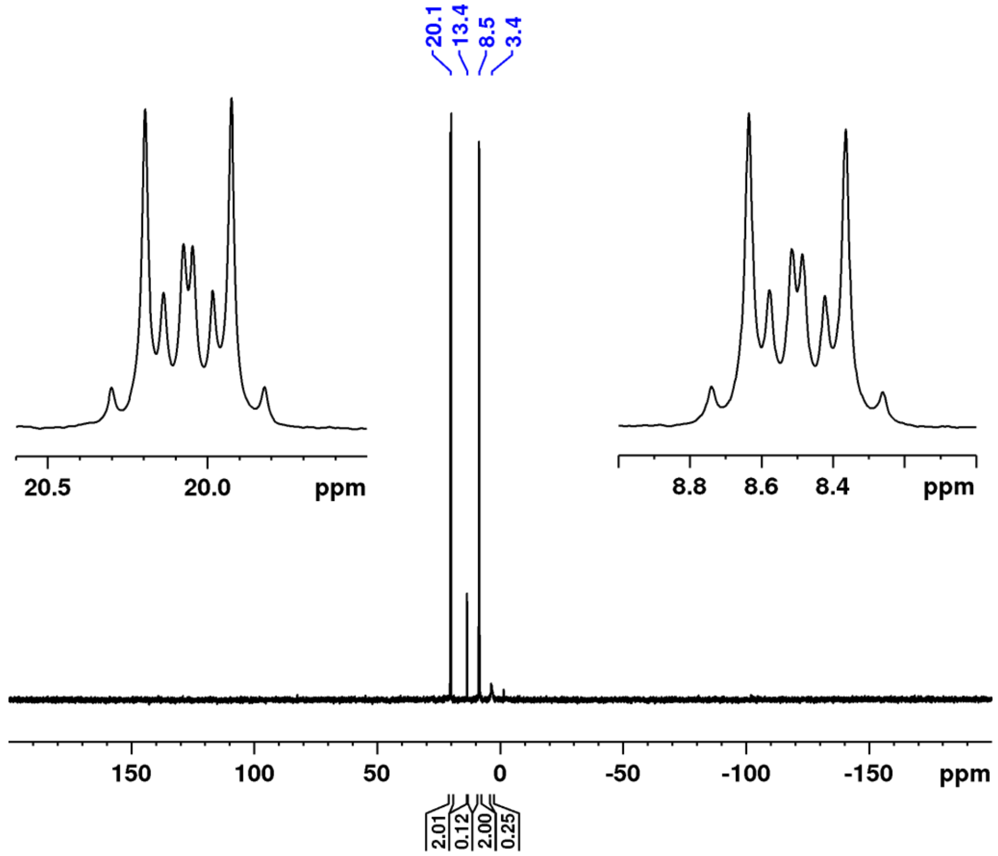

Figure S 86: $^{31}P\{^{1}H\}$ NMR spectrum (121.52 MHz, *o*DFB, 298 K) of a mixture [{Ga(dcpe)}$_2$][*pf*]$_2$ and NH_2Ph in *o*DFB.

4.6.1 Observed Intermediates during the Formation of [H{Ga(dcpe)}$_2$NHPh]$^{2+}$

During the H–N bond activation with H_2NPh, one intermediate is observed in the $^{31}P\{^1H\}$ NMR spectrum. The NMR findings for this species are similar to those for the intermediate with PhOH (section 4.4.1): It is characterized by a singlet in the $^{31}P\{^1H\}$ NMR spectrum (δ (^{31}P) = 13.4 ppm) and $J_{H,P}$ coupling constants of ca. 27 Hz. The ^1H signal of the corrsponding Ga**H** proton at ca. 5.25 ppm is remarkably broad, so that it cannot be determined whether the ^1H signal has a triplet or quintet splitting pattern in the non-decoupled ^1H NMR spectrum. Apparantly, the intermediate is more stable for H_2NPh than for HOPh, since it is still present in the reaction mixture after several hours (see **Figure S 87** and **Figure S 89**). However, the signal intensities indicate that the concentration of this species decreases while the dimeric addition product is formed. A plausible structure is presented in **Figure S 87**.

Nevertheless, taking into account that the ^{31}P NMR and ^1H NMR shift of the **P**–Ga–**H** units in the intermediates observed during the reactions with HOPh, H_2NPh and $HNPh_2$ (sections 4.4.1, 4.6.1 and 4.7.1) is very similar, it cannot be ruled out that the NHPh group acts as a bridging ligand with its aromatic π-system or that the NHPh group is only weakly associated.

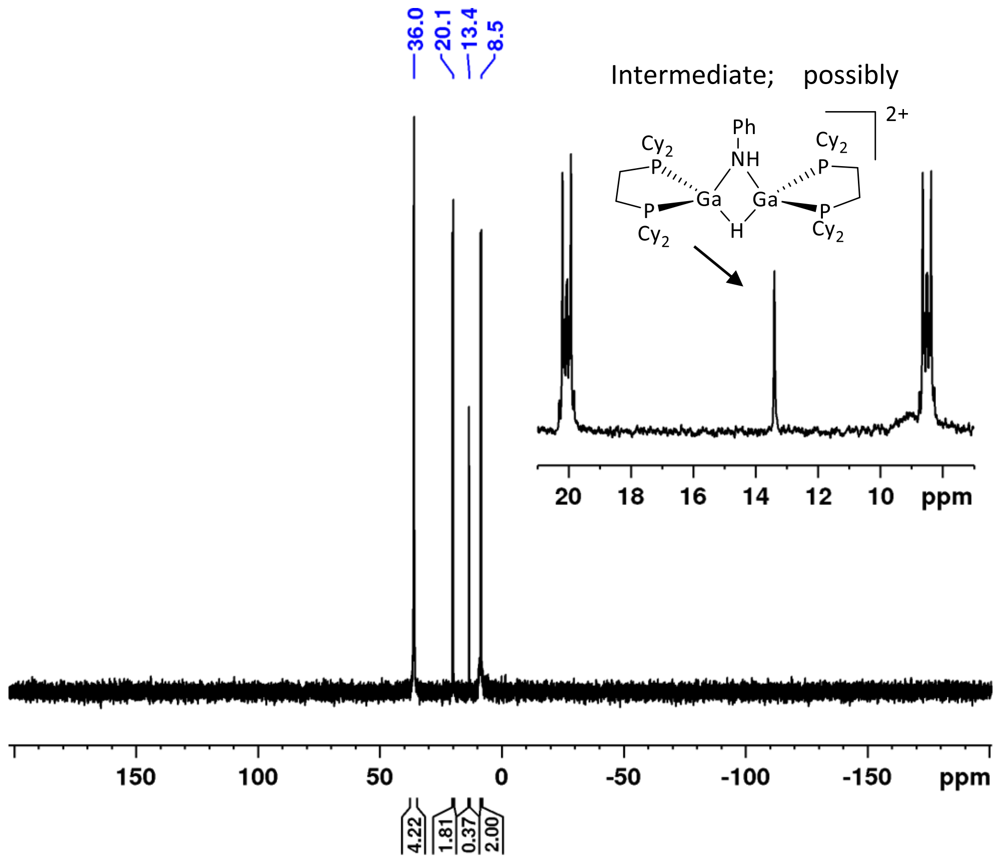

Figure S 87: $^{31}P\{^1H\}$ NMR spectrum (121.52 MHz, *o*DFB, 298 K) of a mixture [{Ga(dcpe)}$_2$][*pf*]$_2$ and NH_2Ph in *o*DFB, directly after mixing the components.

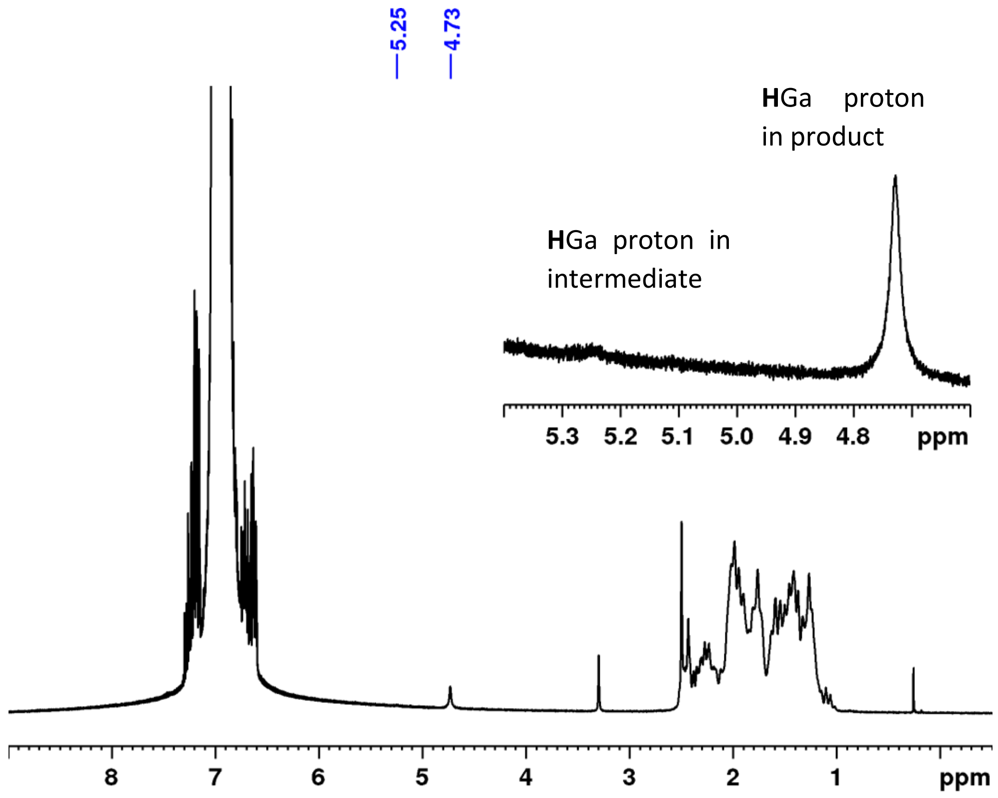

Figure S 88: $^{1}H\{^{31}P\}$ NMR spectrum (300.18 MHz, *o*DFB, 298 K) of a mixture [{Ga(dcpe)}$_2$][*pf*]$_2$ and NH_2Ph in *o*DFB, 3 h after mixing the components.

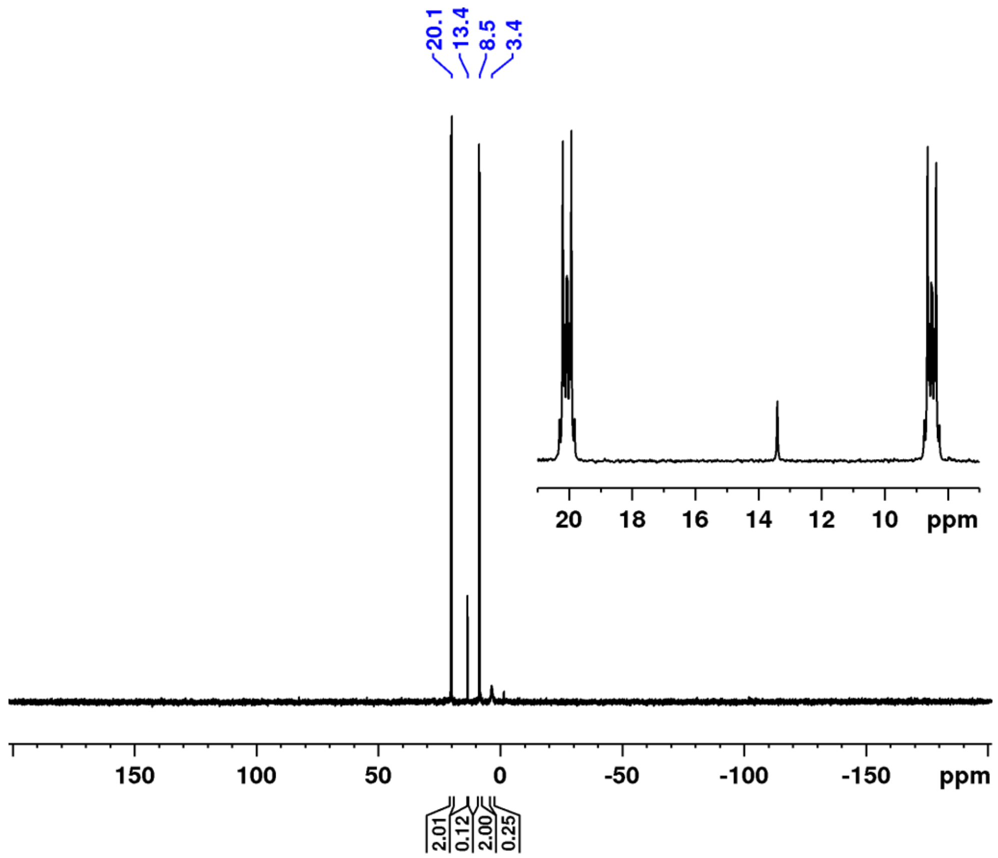

Figure S 89: $^{31}P\{^{1}H\}$ NMR spectrum (121.52 MHz, *o*DFB, 298 K) of a mixture [{Ga(dcpe)}$_2$][*pf*]$_2$ and NH_2Ph in *o*DFB, 8 h after mixing the components.

4.7 [H{Ga(dcpe)}$_2$(NPh$_2$)][*pf*]$_2$

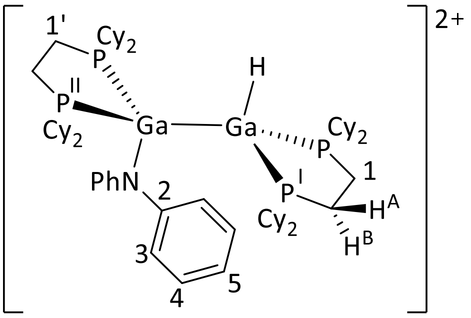

^{1}H NMR [300.18 MHz, *o*DFB, calibrated at *o*C_6F_2**H**$_4$ = 6.96 ppm, 298 K]: δ = 7.39 (m, 2 H, C^4**H**), 7.13 (1 H, C^5**H**), 6.85 (2 H, C^3**H**) 5.85 (br, **H**NPh$_2$, starting material), 5.00 (t, 1 H, Ga**H**, $^2J_{H,P}$ = 19.5 Hz), 2.44–2.36 (m, 8 H, C^1**H**^{A}, C^1**H**^{B}, $C^{1'}$**H**^{A} and $C^{1'}$**H**^{B}), 2.34–1.52 (m, 8 H, C**H** protons of the Cy groups), 2.06–0.83 (m, 80 H, C**H**$_2$ protons of the Cy groups) ppm.

^{13}C NMR [100.62 MHz, *o*DFB, 298 K]: δ = 150.7 (1 C, **C**^{2}), 130.6 (2 C, **C**^{4}), 122.5 (1 C, **C**^{5}), 121.5 (2 C, **C**^{3}), 33.7−32.4 (8 C, **C**H carbon atoms of the Cy groups), 30.4−25.4 (40 C, **C**H$_2$ carbon atoms on the Cy groups), 16.5−14.6 (4 C, **C**^{1} and **C**$^{1'}$) ppm.

^{19}F NMR [376.54 MHz, *o*DFB, 298 K]: δ = −75.3 (s, 36 F, [Al(OC(C**F**$_3$)$_3$)$_4$]$^-$, −113.8 (m, Ph**F**), −139.5 (m, *o*C_6**F**$_2H_4$) ppm.

^{27}Al NMR [78.22 MHz, *o*DFB, 298 K]: δ = 35.0 (s, 1 Al, [Al(OC(C**F**$_3$)$_3$)$_4$]$^-$) ppm.

^{31}P NMR [161.99 MHz, *o*DFB, 298 K]: δ = 30.7 ([HdcpeH]$^+$), 21.6 (m, 2 P, **P**^{I}), 17.8 (unknown impurity), 5.5 (m, 2 P, **P**^{II}), −1.5 (m, [H(dcpe)GaNPh$_2$]$^+$) ppm

^{71}Ga NMR [91.54 MHz, *o*DFB, 298 K]: signal probably too broad to be detected, due to the quadrupolar relaxation of ^{71}Ga.

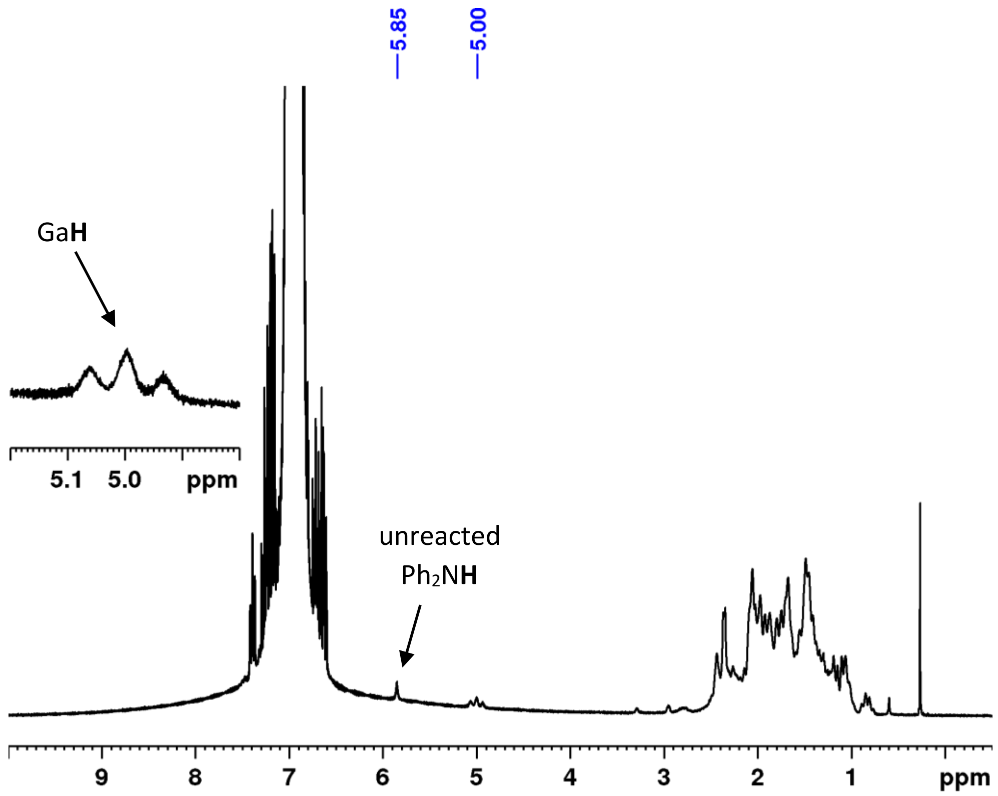

Figure S 90: ^{1}H NMR spectrum (300.18 MHz, *o*DFB, 298 K) of a mixture of [{Ga(dcpe)}$_2$][*pf*]$_2$ and $NHPh_2$ in *o*DFB after heating the reaction mixture to 60 °C for 24 h.

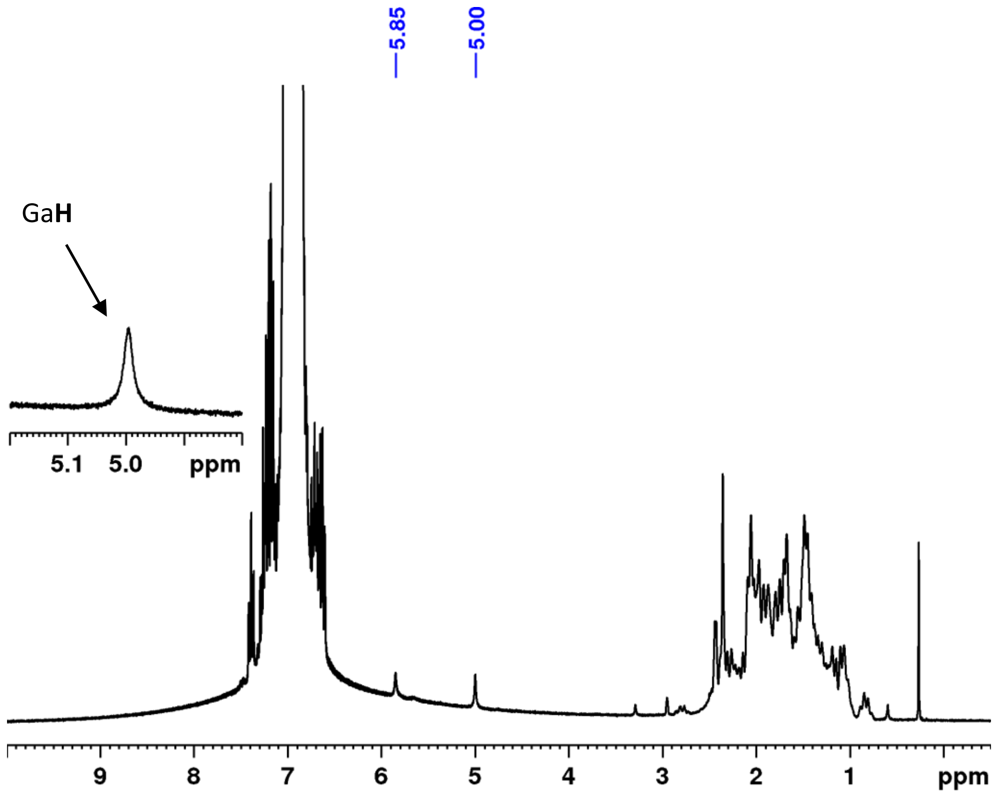

Figure S 91: $^{1}H\{^{31}P\}$ NMR spectrum (300.18 MHz, *o*DFB, 298 K) of a mixture of [{Ga(dcpe)}$_2$][*pf*]$_2$ and $NHPh_2$ in *o*DFB after heating the reaction mixture to 60 °C for 24 h.

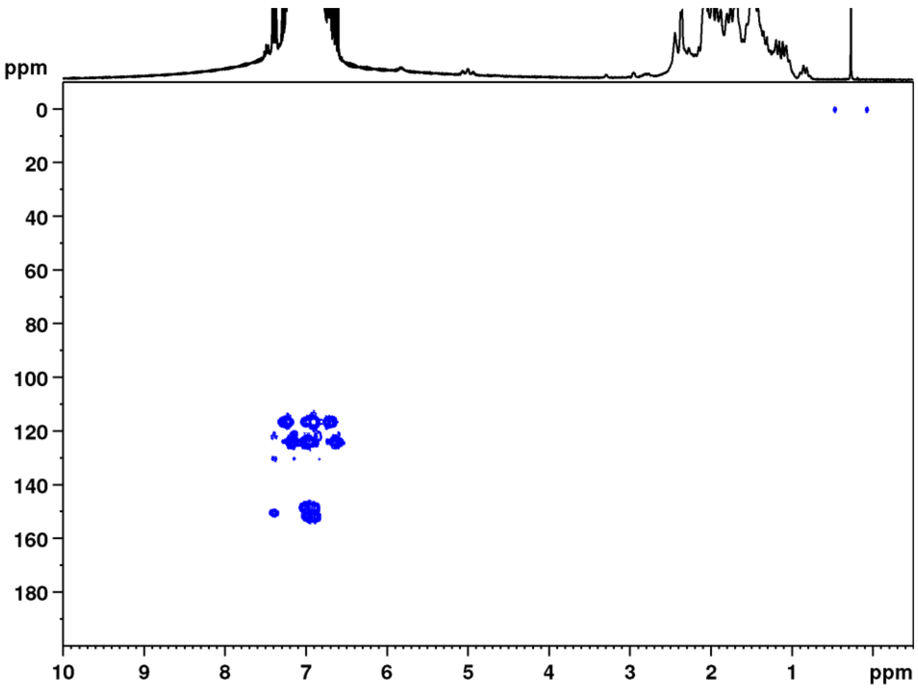

Figure S 92: $^{1}H,^{13}C$-HMBC NMR spectrum (300.18 MHz, *o*DFB, 298 K, optimized for J = 8.0 Hz) of a mixture of $[\{Ga(dcpe)\}_2][pf]_2$ and $NHPh_2$ in *o*DFB after heating the reaction mixture to 60 °C for 24 h.

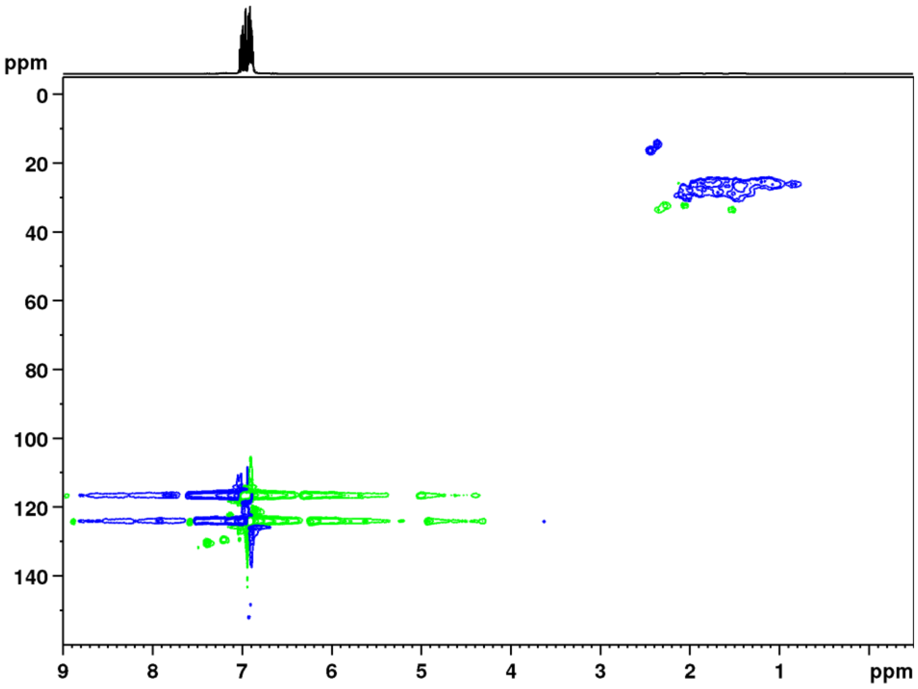

Figure S 93: Edited $^{1}H,^{13}C$-HSQC NMR spectrum (300.18 MHz, *o*DFB, 298 K, optimized for J = 145 Hz) of a mixture of $[\{Ga(dcpe)\}_2][pf]_2$ and $NHPh_2$ in *o*DFB after heating the reaction mixture to 60 °C for 24 h.

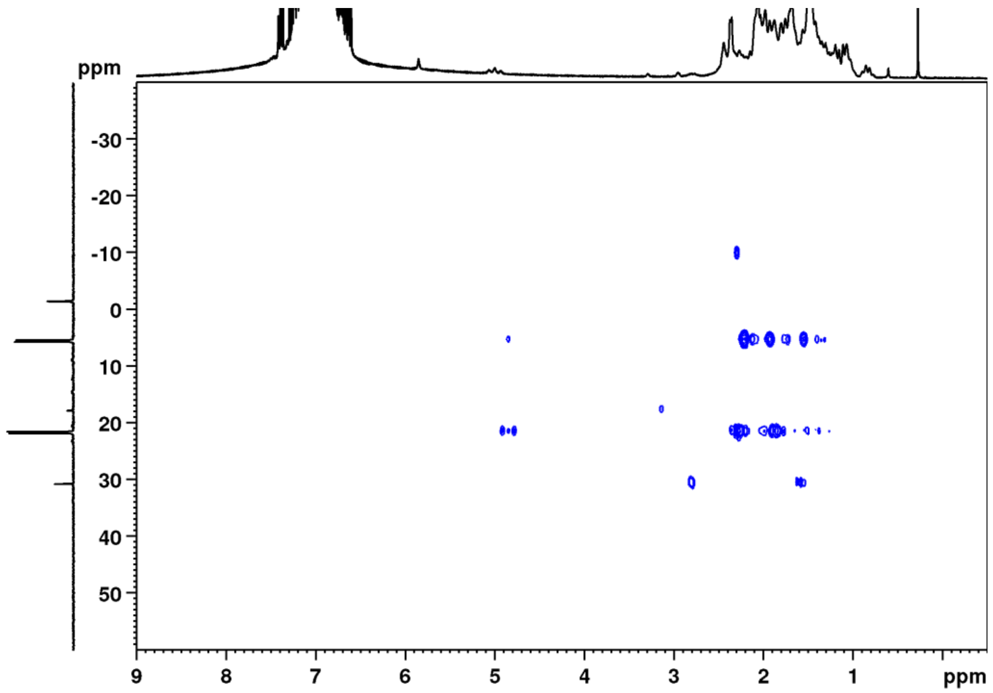

Figure S 94: ^{1}H,^{31}P-HMBC NMR spectrum (300.18 MHz, *o*DFB, 298 K, optimized for *J* = 15 Hz) of a mixture of [{Ga(dcpe)}$_2$][*pf*]$_2$ and $NHPh_2$ in *o*DFB after heating the reaction mixture to 60 °C for 24 h.

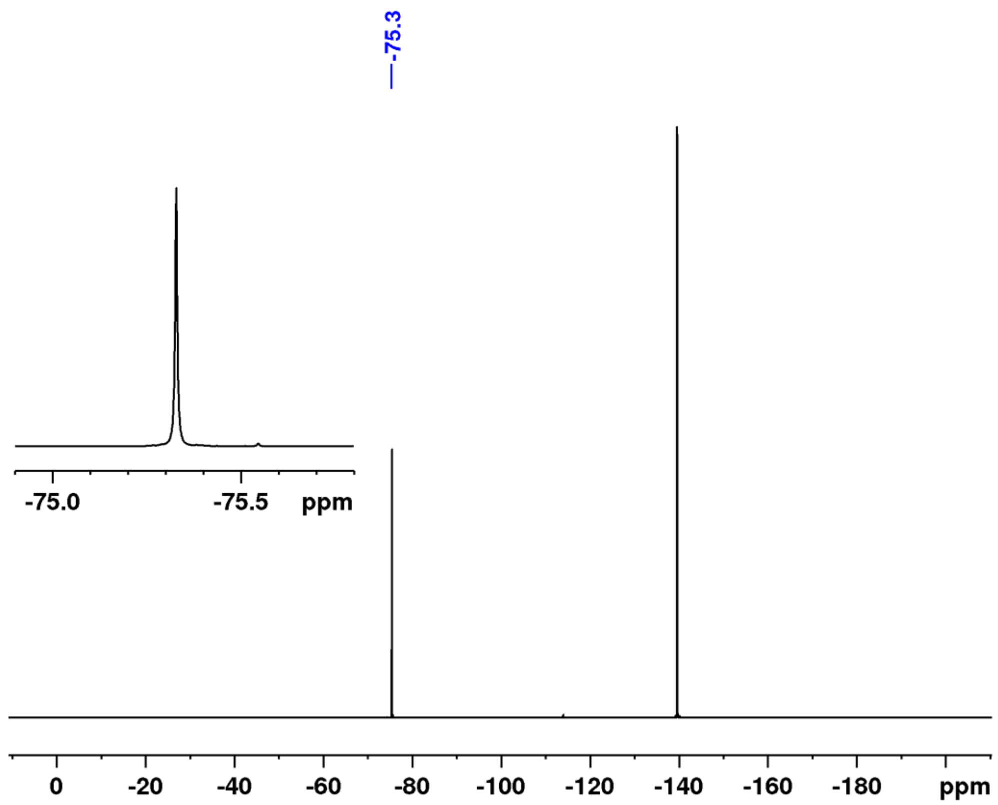

Figure S 95: ^{19}F NMR spectrum (282.45MHz, *o*DFB, 298 K) of a mixture of [{Ga(dcpe)}$_2$][*pf*]$_2$ and $NHPh_2$ in *o*DFB after heating the reaction mixture to 60 °C for 24 h.

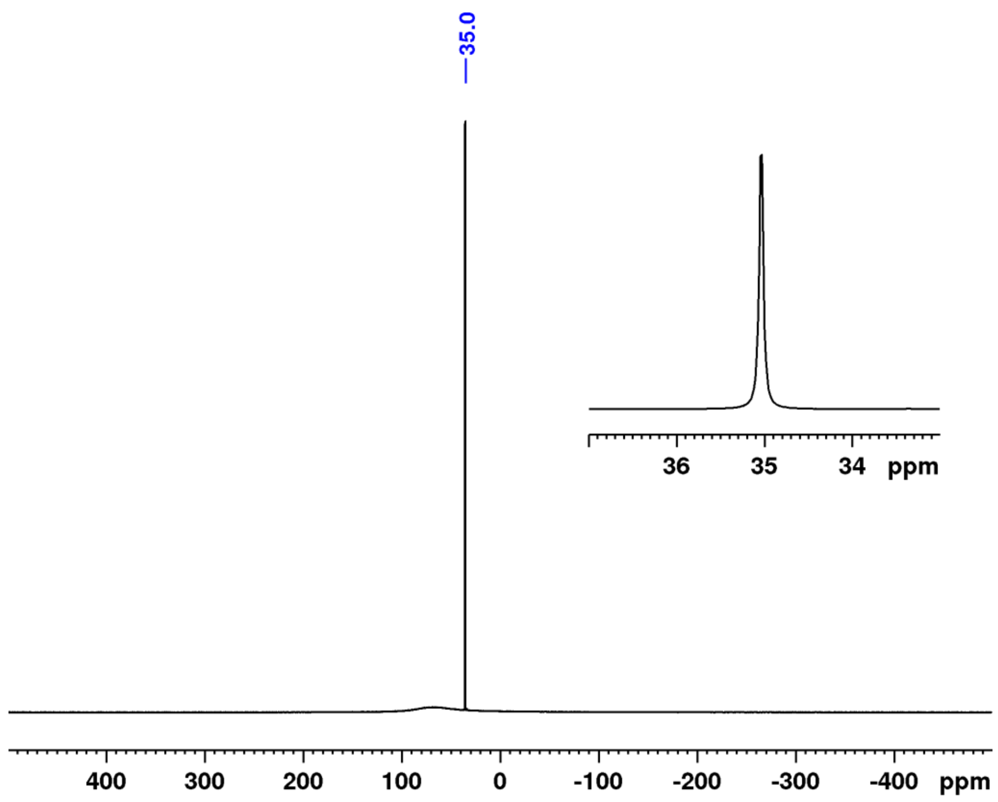

Figure S 96: ^{27}Al NMR spectrum (78.22 MHz, *o*DFB, 298 K) of a mixture of [{Ga(dcpe)}$_2$][*pf*]$_2$ and $NHPh_2$ in *o*DFB after heating the reaction mixture to 60 °C for 24 h.

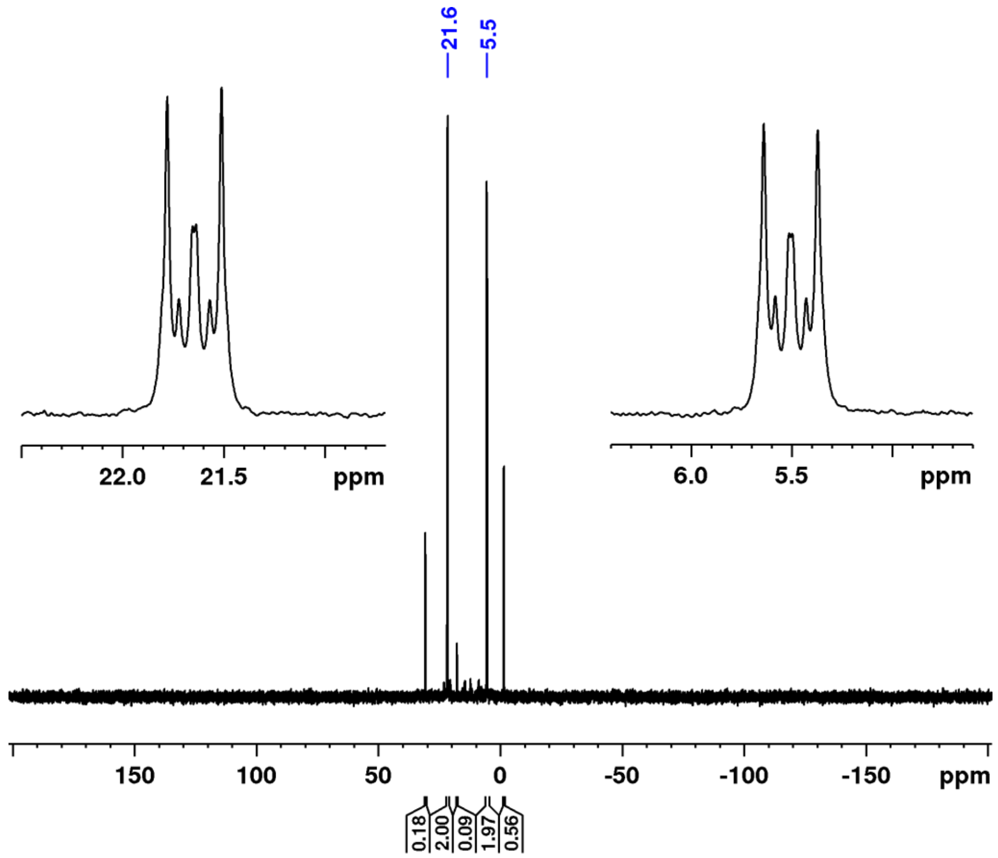

Figure S 97: $^{31}P\{^1H\}$ NMR spectrum (121.52 MHz, *o*DFB, 298 K) of a mixture of [{Ga(dcpe)}$_2$][*pf*]$_2$ and $NHPh_2$ in *o*DFB after heating the reaction mixture to 60 °C for 24 h.

4.7.1 Observed Intermediates during the Formation of $[H\{Ga(dcpe)\}_2NPh_2]^{2+}$

With $HNPh_2$, the reaction process is more complex than with HOPh and H_2NPh (section 4.4.1 and 4.6.1) and seems to depend on the stoichiometry. When using sub-stoichiometric amounts of $HNPh_2$, the main intermediate is similar to those observed with PhOH and H_2NPh (**Figure S 98** and **Figure S 100**). A plausible structure is presented in **Figure S 100**.

Nevertheless, taking into account that the ^{31}P NMR and ^{1}H NMR shift of the **P**–Ga–**H** units in the intermediates observed during the reactions with HOPh, H_2NPh and $HNPh_2$ (sections 4.4.1, 4.6.1 and 4.7.1) is very similar, it cannot be ruled out that the NPh_2 group acts as a bridging ligand with its aromatic π-system or that the NPh_2 group is only weakly associated.

When using a surplus of $HNPh_2$, the reaction proceeds considerably faster at room temperature (*cf*. **Figure S 100** and **Figure S 105**). Besides this, and more importantly, another main intermediate is formed. It is characterized by a singlet in the $^{31}P\{^{1}H\}$ NMR spectrum, a doublet in the ^{31}P NMR spectrum ($\delta(^{31}P) = -1.5$ ppm) and by a $J_{H,P}$ coupling constant of 50 Hz, which is indicative of a Ga^{III}–H species. The Ga**H** proton shows a signal at 5.65 ppm. On the basis of these findings (**Figure S 101**–**Figure S 104**), we suggest that this species is a monomeric $[HGa(dcpe)NPh_2]^+$ complex, since, in the dimeric Ga^{II} addition products, the $^{2}J_{H,P}$ coupling constants are significantly smaller (ca. 20 Hz) and all phosphorous atoms in the molecule are chemically and magnetically equivalent. The putative monomer is quickly formed in solution and at room temperature slowly reacts further as the concentration of $\mathbf{1}^{2+}$ decreases (**Figure S 105**), indicating that the monomeric addition product reacts with the digallene under the formation of the final, dimeric Ga^{II} addition product.

It should be noted that both intermediates are present in reaction solutions of $\mathbf{1}^{2+}$ and $HNPh_2$, and that the applied stoichiometry merely influences their relative concentrations, underlining that, with $HNPh_2$, different reaction mechanisms are likely opperative. In this context, it is unsurprising that a higher starting concentration of $HNPh_2$ leads to an increased formation of the monomeric intermediate. The somewhat more complex reaction mechanism compared to other substrates could be attributed to the high steric demand of $HNPh_2$, and a different coordination mode compared to other alcohols and amines: due to the electron delocalization over the Ph–N–Ph system, the π systems are electron rich, while the nitrogen atom is comparatively electron poor.

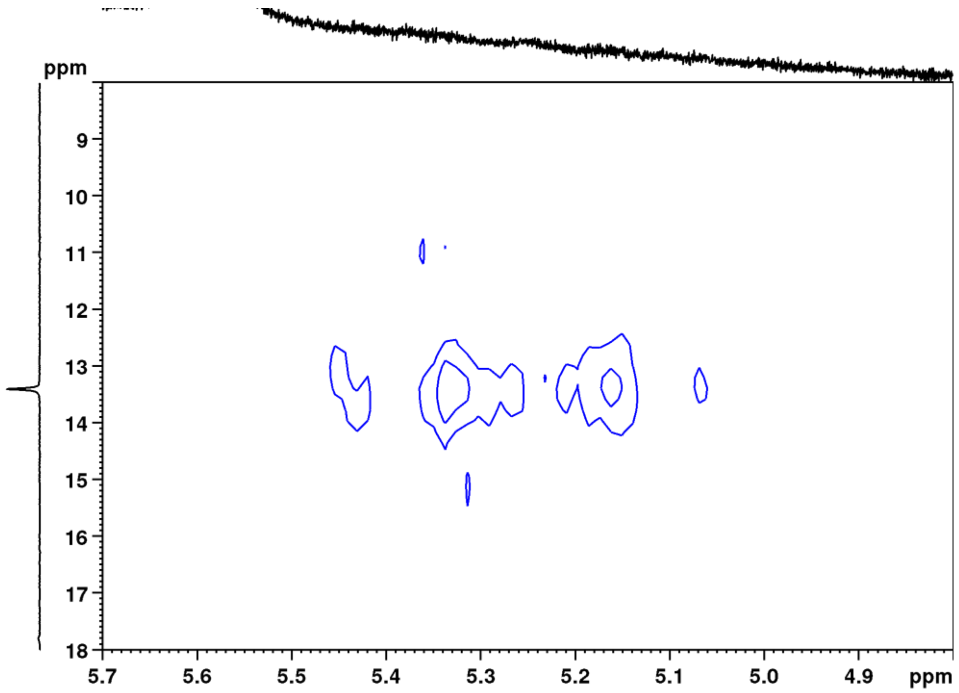

Figure S 98: $^{1}H,^{31}P$-HMBC NMR spectrum (300.18 MHz, *o*DFB, 298 K, optimized for *J* = 15 Hz) of a mixture of [{Ga(dcpe)}$_2$][*pf*]$_2$ and $NHPh_2$ (1 : 0.6) in *o*DFB, two days after mixing the components.

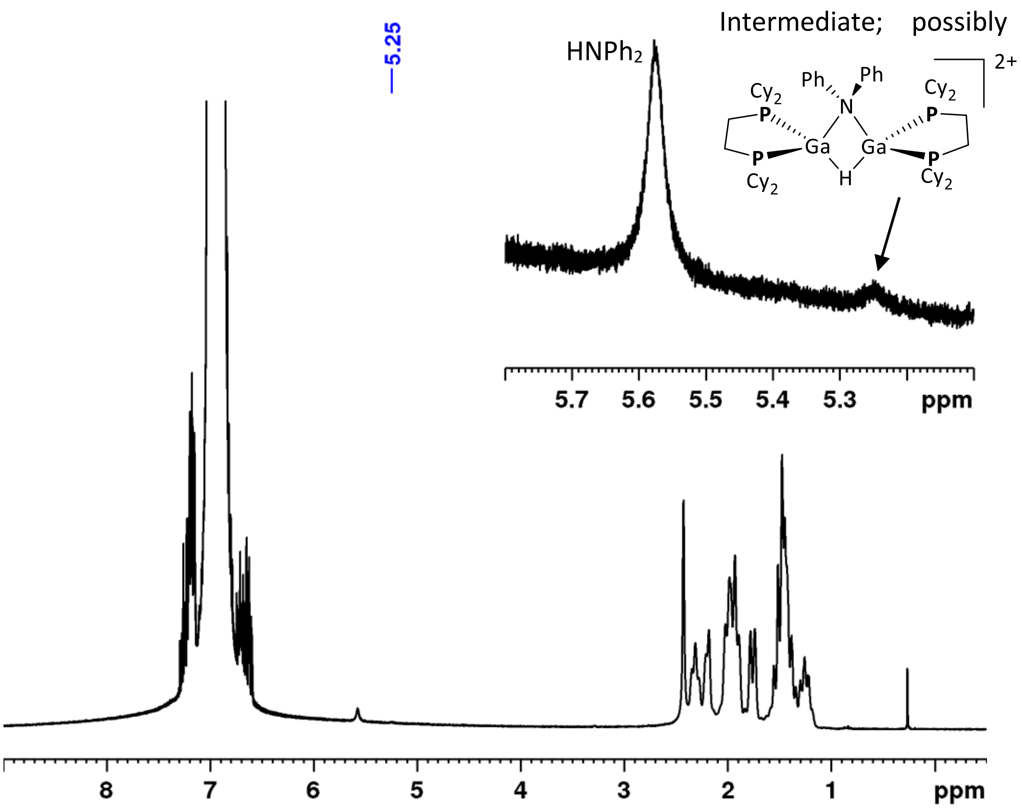

Figure S 99: $^{1}H\{^{31}P\}$ NMR spectrum (300.18 MHz, *o*DFB, 298 K) of a mixture of [{Ga(dcpe)}$_2$][*pf*]$_2$ and $NHPh_2$ (1 : 0.6) in *o*DFB, one day after mixing the components.

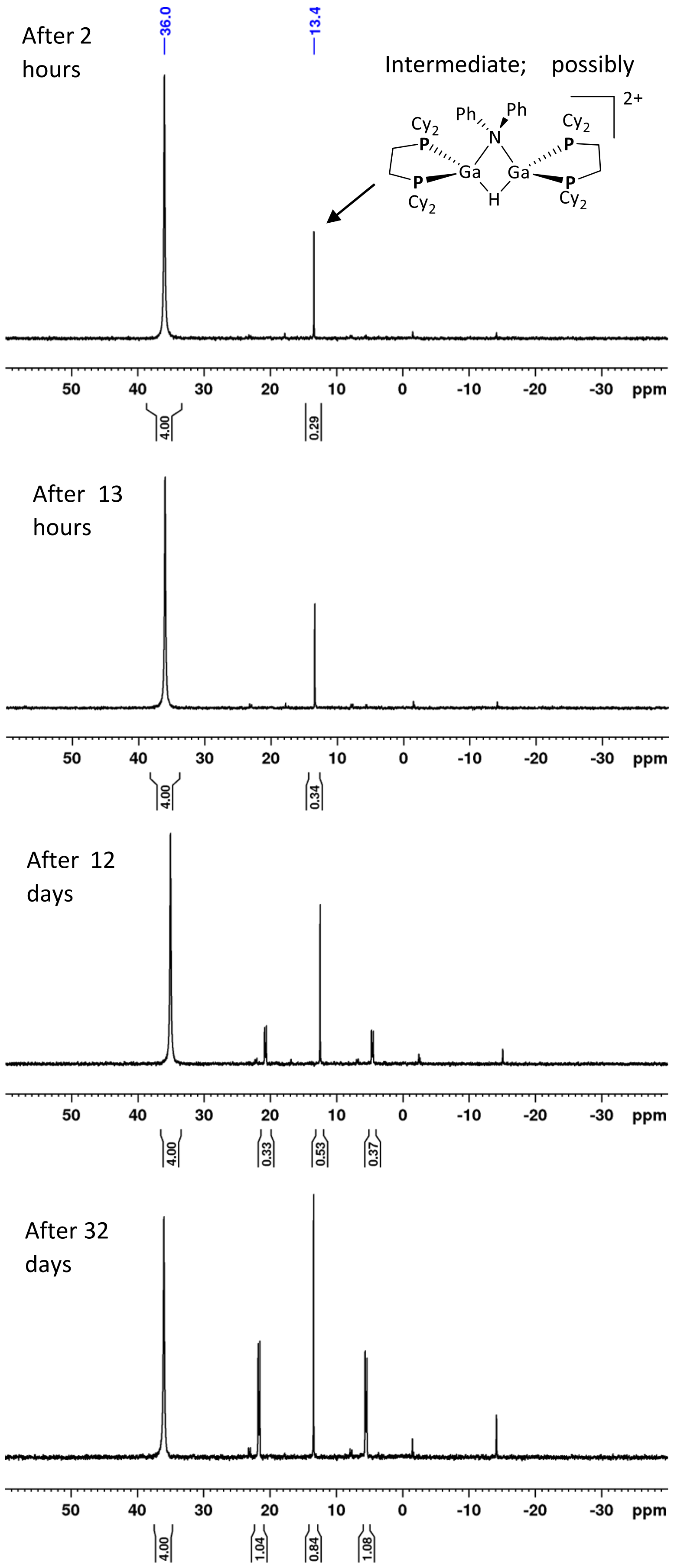

Figure S 100: $^{31}P\{^{1}H\}$ NMR spectrum (121.52 MHz, *o*DFB, 298 K) of a mixture of $[\{Ga(dcpe)\}_2][pf]_2$ and $NHPh_2$ (1 : 0.6) in *o*DFB at different times after mixing the components.

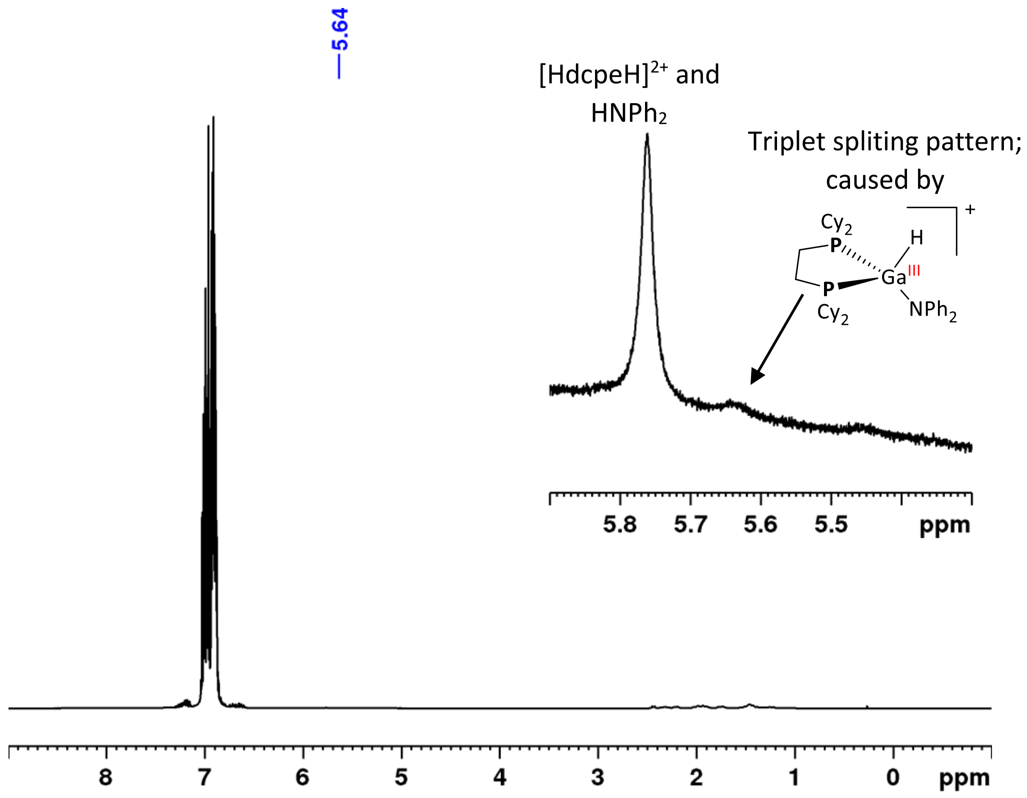

Figure S 101: ^{1}H NMR spectrum (300.18 MHz, *o*DFB, 298 K) of a mixture of $[\{Ga(dcpe)\}_2][pf]_2$ and $NHPh_2$ (1 : 1.2) in *o*DFB, 6 h after mixing the components.

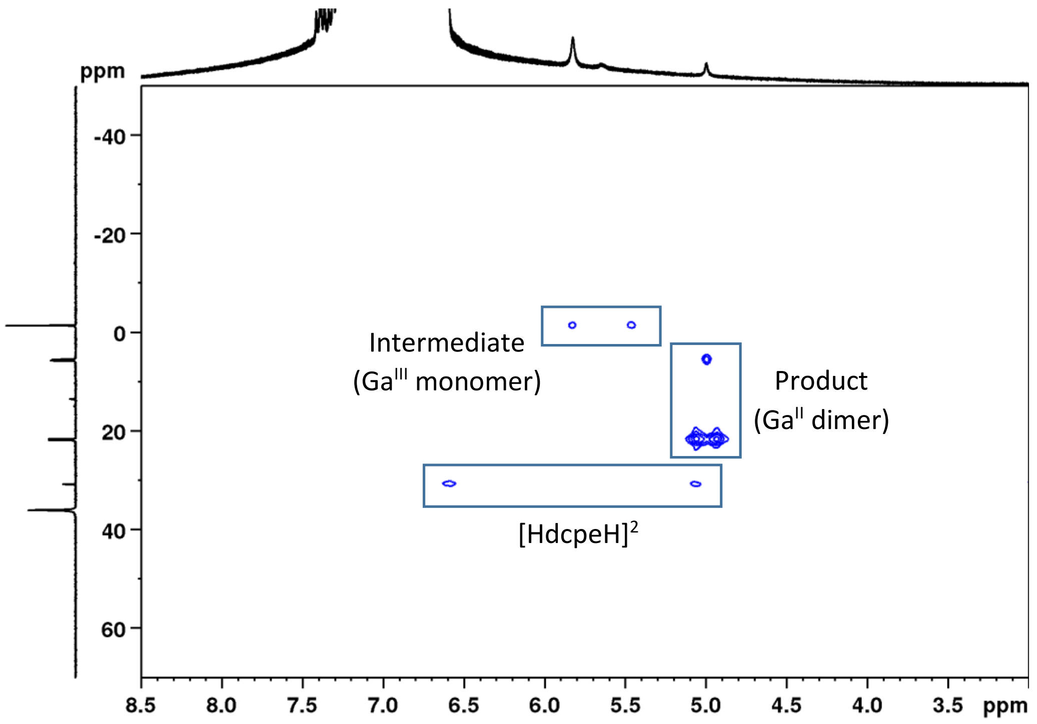

Figure S 102: $^{1}H,^{31}P$-HMBC NMR spectrum (300.18 MHz, *o*DFB, 298 K, optimized for $J = 15$ Hz) of a mixture of $[\{Ga(dcpe)\}_2][pf]_2$ and $NHPh_2$ (1 : 1.2) in *o*DFB, one day after mixing the components.

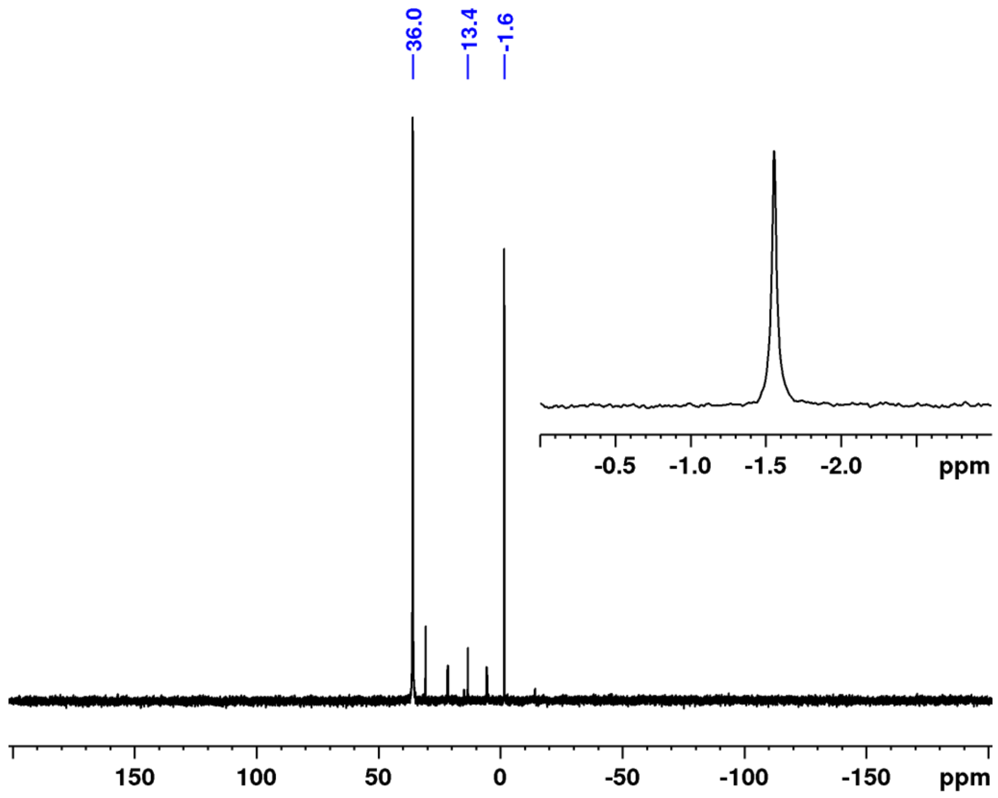

Figure S 103: $^{31}P\{^{1}H\}$ NMR spectrum (121.52 MHz, *o*DFB, 298 K) of a mixture of [{Ga(dcpe)}$_2$][*pf*]$_2$ and $NHPh_2$ (1 : 1.2) in *o*DFB, 7 h after mixing the components.

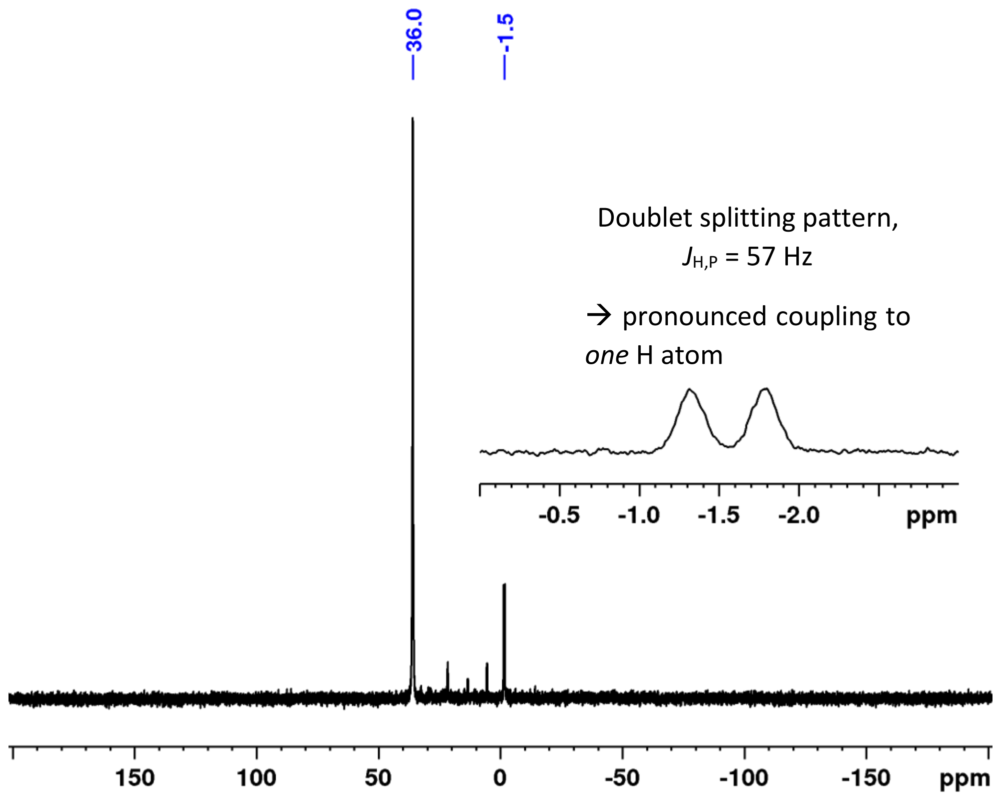

Figure S 104: ^{31}P NMR spectrum (121.52 MHz, *o*DFB, 298 K) of a mixture of [{Ga(dcpe)}$_2$][*pf*]$_2$ and $NHPh_2$ (1 : 1.2) in *o*DFB, 7 h after mixing the components.

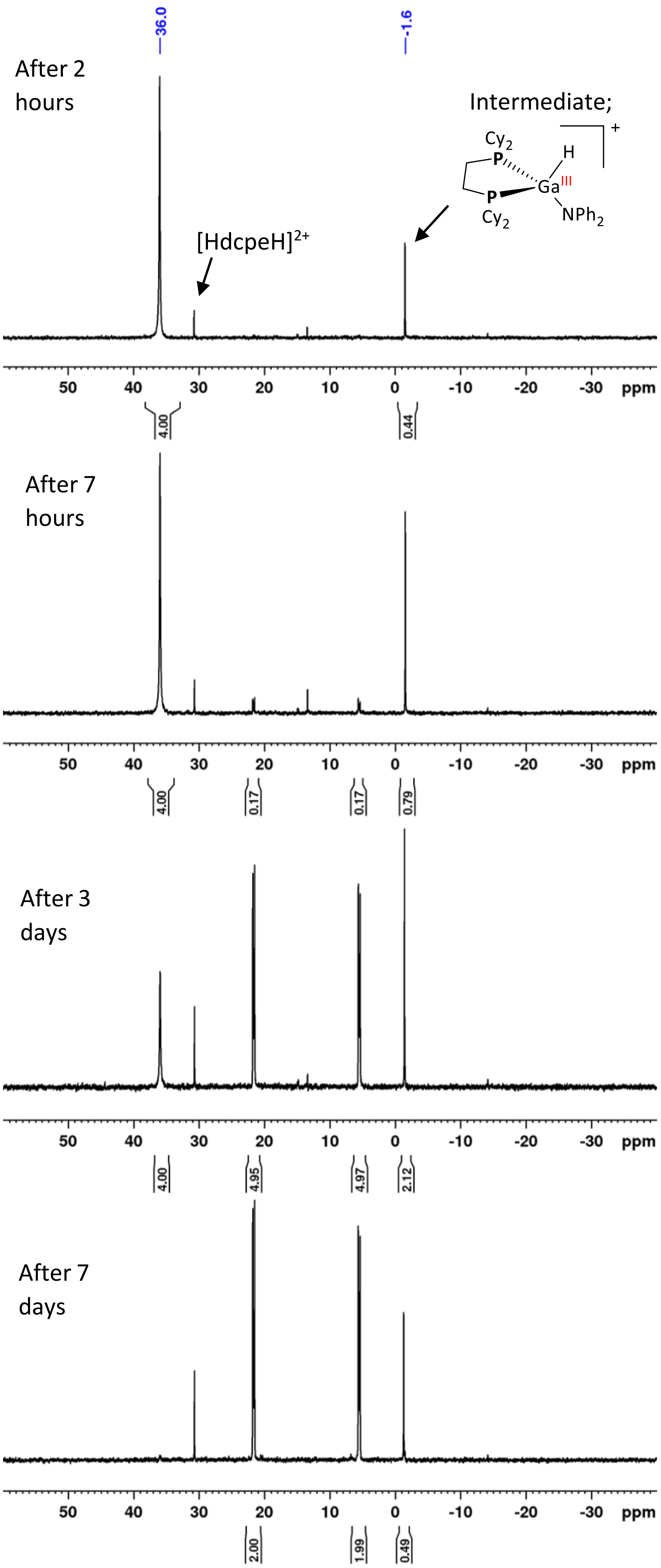

Figure S 105: $^{31}P\{^{1}H\}$ NMR spectrum (121.52 MHz, *o*DFB, 298 K) of a mixture of [{Ga(dcpe)}$_2$][*pf*]$_2$ and $NHPh_2$ (1 : 1.2) in *o*DFB at different times after mixing the components.

4.8 $[H\{Ga(dcpe)\}_2(CH_2CN)][pf]_2$

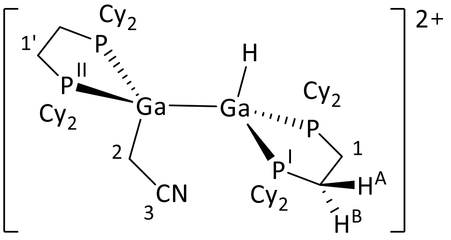

^{1}H NMR [300.18 MHz, *o*DFB, calibrated at $oC_6F_2H_4$ = 6.96 ppm, 298 K]: δ = 4.68 (t, 1 H, Ga**H**, $^{2}J_{H,P}$ = 20.8 Hz), 2.54–2.29 (m, 8 H, C**H** protons of the Cy groups), 2.50–2.44 (m, 8 H, C^1H^A, C^1H^B, $C^{1'}H^A$ and $C^{1'}H^B$), 2.15–1.29 (m, 8 H, C**H**$_2$ protons of the Cy groups), 1.80 (t, 2 H, C^2H_2, $^{3}J_{H,P}$ = 4.3 Hz) ppm.

^{13}C NMR [100.62 MHz, *o*DFB, 298 K]: δ = 122.7 (1 C, **C**^{3}N), 33.8–32.7 (8 C, **C**H carbon atoms of the Cy groups), 29.3–24.9 (40 C, **C**H$_2$ carbon atoms of the Cy groups), 16.6 and 15.6 (4 C, **C**^{1} and **C**$^{1'}$), –2.5 (1 C, **C**^{2}) ppm.

^{19}F NMR [376.54 MHz, *o*DFB, 298 K]: δ = –75.3 (s, 36 F, $[Al(OC(CF_3)_3)_4]^-$), –113.9 (m, Ph**F**), –139.5 (m, $oC_6F_2H_4$) ppm.

^{27}Al NMR [78.22 MHz, *o*DFB, 298 K]: δ = 35.0 (s, 1 Al, $[Al(OC(CF_3)_3)_4]^-$) ppm.

^{31}P NMR [161.99 MHz, *o*DFB, 298 K]: δ = 35.9 (digallene starting material), 23.1 (unknown impurity), 20.8 (m, 2 P, **P**^{I}), 14.9 (m, 2 P, **P**^{II}), 10.4 (unknown impurity) ppm.

^{71}Ga NMR [91.54 MHz, *o*DFB, 298 K]: signal probably too broad to be detected, due to the quadrupolar relaxation of ^{71}Ga.

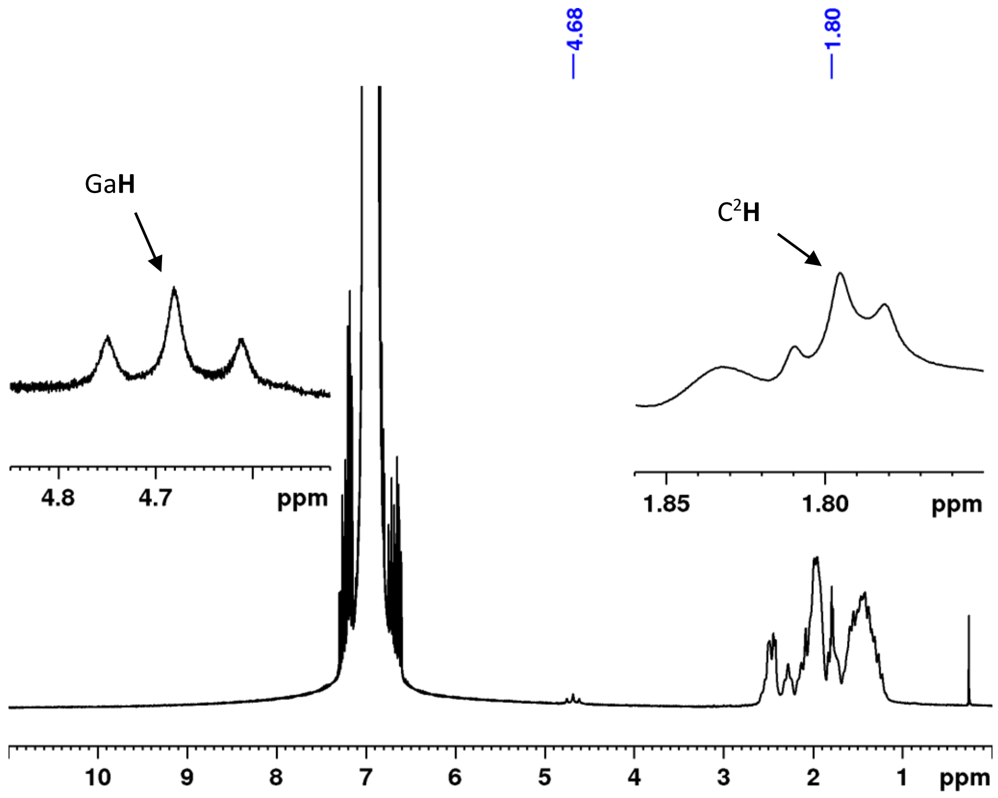

Figure S 106: ^{1}H NMR spectrum (300.18 MHz, *o*DFB, 298 K) of a mixture of [{Ga(dcpe)}$_2$][*pf*]$_2$ and NC–CH$_3$ in *o*DFB after heating the reaction mixture to 60 °C for 4 days.

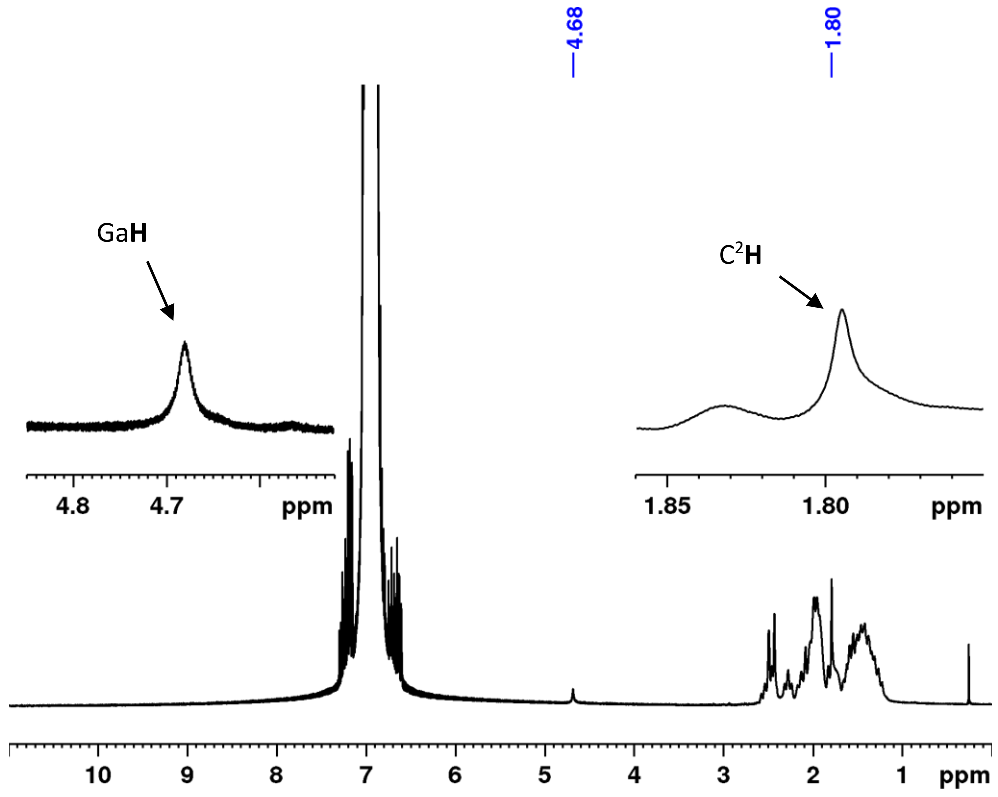

Figure S 107*:* $^{1}H\{^{31}P\}$ NMR spectrum (300.18 MHz, *o*DFB, 298 K) of a mixture of [{Ga(dcpe)}$_2$][*pf*]$_2$ and NC–CH$_3$ in *o*DFB after heating the reaction mixture to 60 °C for 4 days.

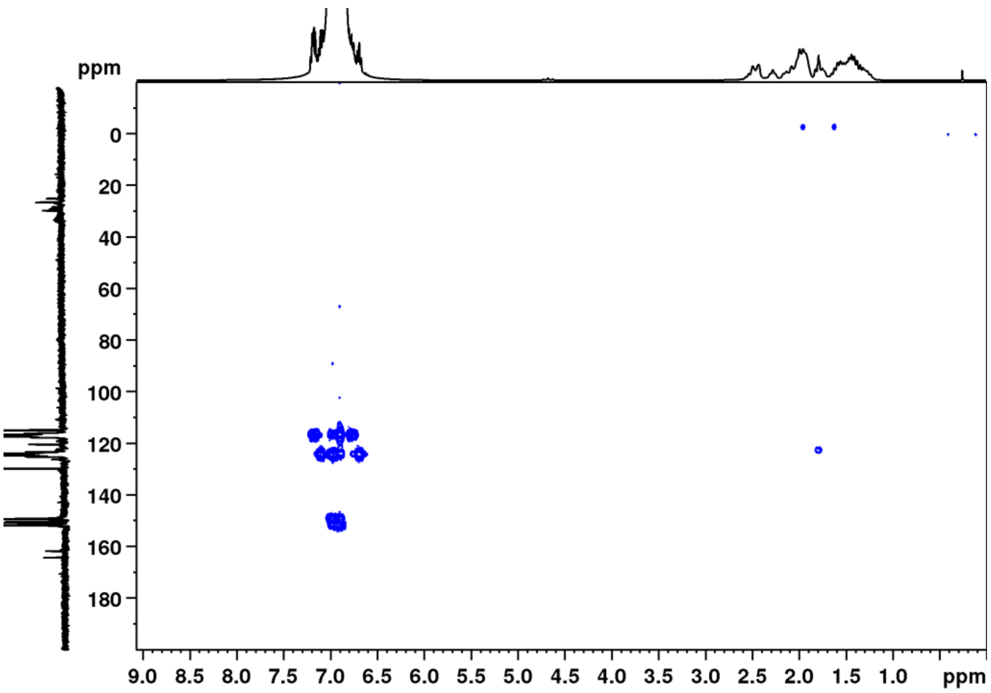

Figure S 108: $^{1}H,^{13}C$-HMBC NMR spectrum (400.17 MHz, *o*DFB, 298 K, optimized for J = 8 Hz) of a mixture of [{Ga(dcpe)}$_2$][*pf*]$_2$ and NC–CH_3 in *o*DFB after heating the reaction mixture to 60 °C for 4 days.

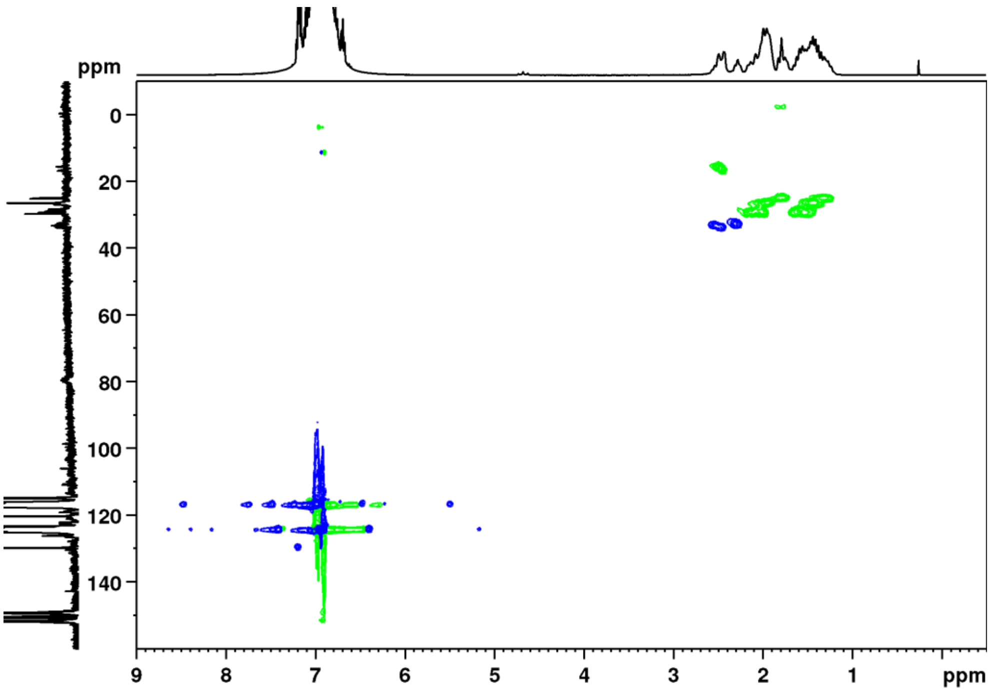

Figure S 109: Edited $^{1}H,^{13}C$-HSQC NMR spectrum (400.17 MHz, *o*DFB, 298 K, optimized for J = 145 Hz) of a mixture of [{Ga(dcpe)}$_2$][*pf*]$_2$ and NC–CH_3 in *o*DFB after heating the reaction mixture to 60 °C for 4 days.

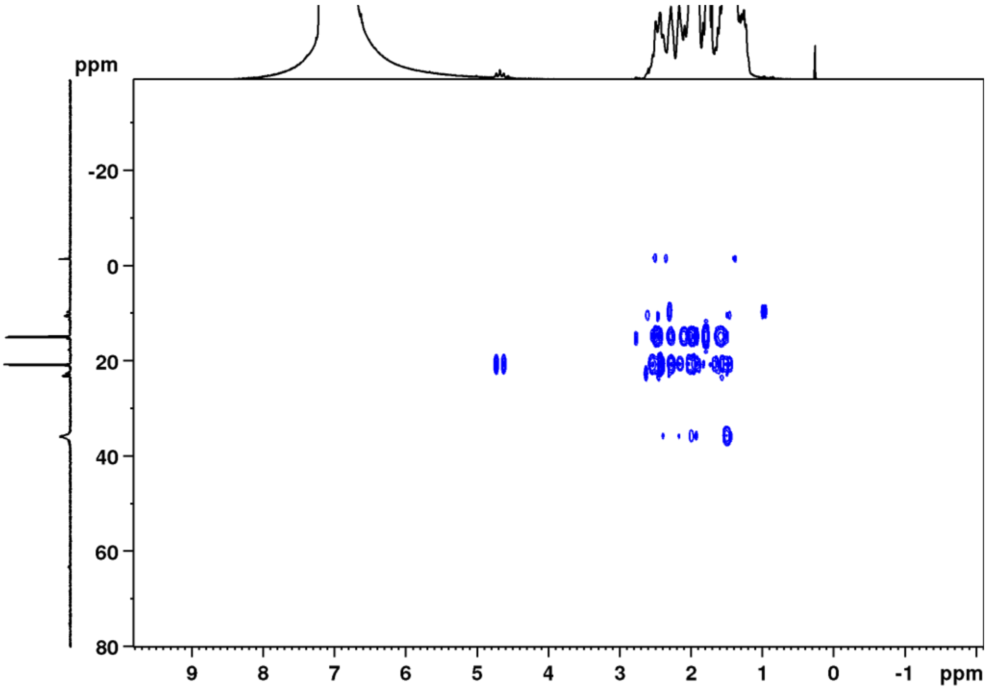

Figure S 110: $^{1}H,^{31}P$-HMBC NMR spectrum (400.17 MHz, *o*DFB, 298 K, optimized for $J = 15$ Hz) of a mixture of [{Ga(dcpe)}$_2$][*pf*]$_2$ and NC–CH_3 in *o*DFB after heating the reaction mixture to 60 °C for 4 days.

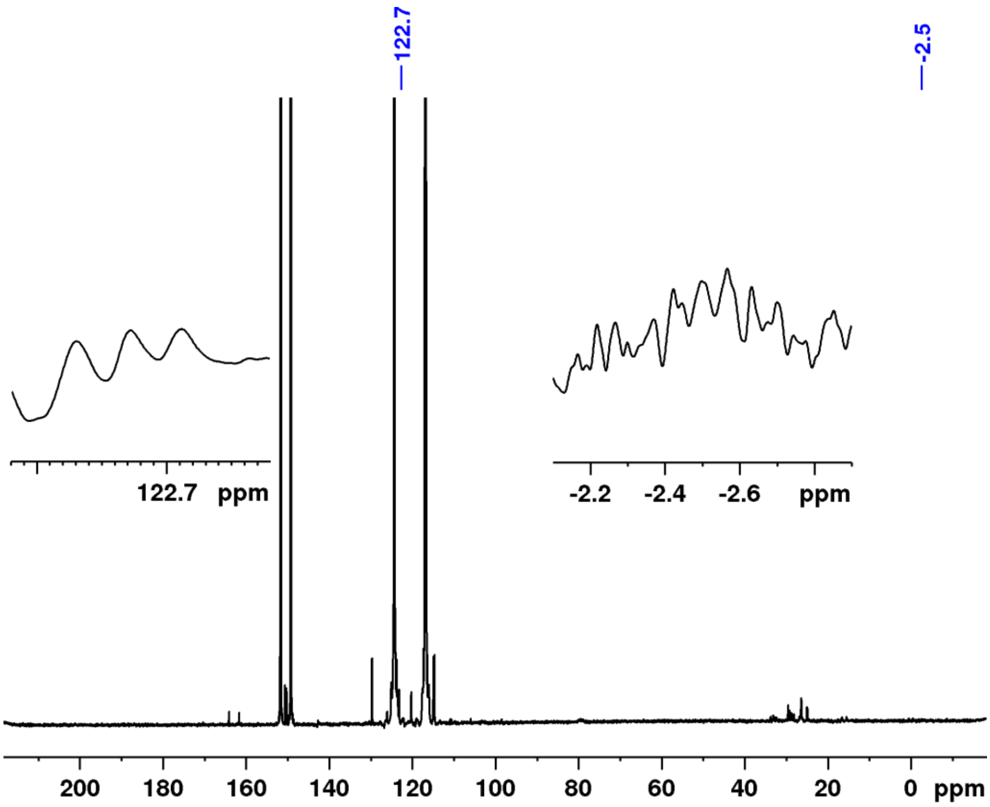

Figure S 111: $^{13}C\{^{1}H\}$ NMR spectrum (376.54 MHz, *o*DFB, 298 K) of a mixture of [{Ga(dcpe)}$_2$][*pf*]$_2$ and NC–CH_3 in *o*DFB after heating the reaction mixture to 60 °C for 4 days.

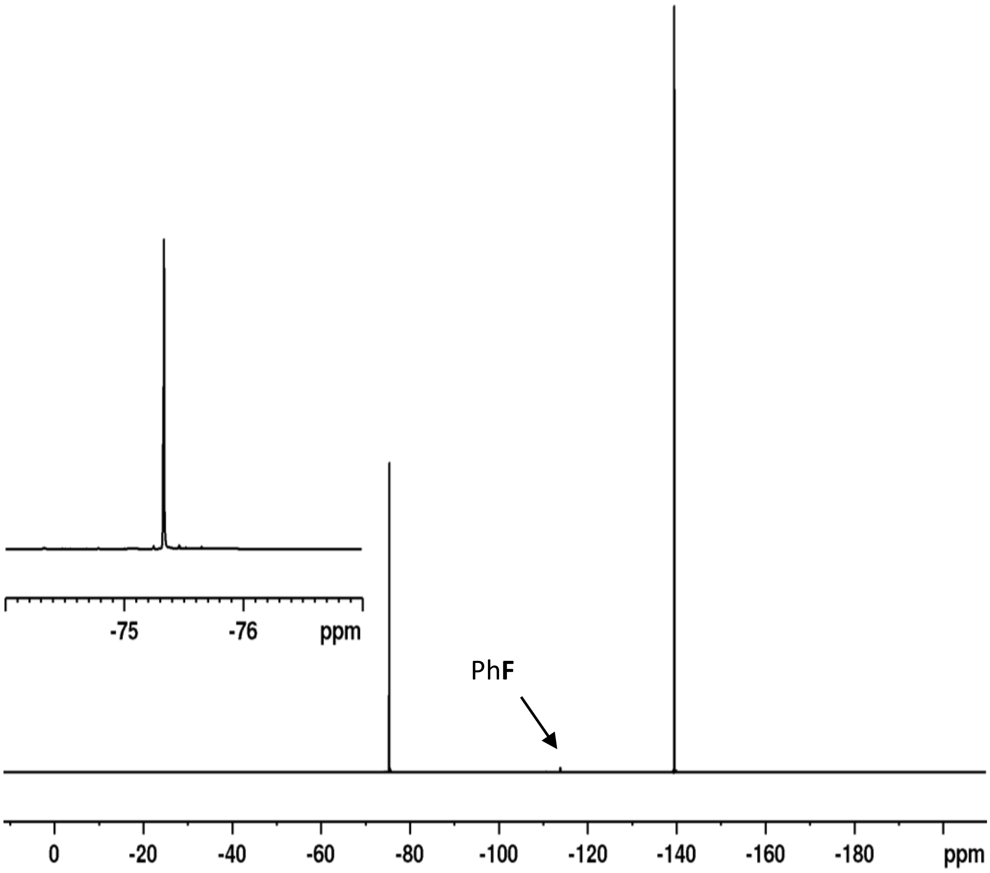

Figure S 112: ^{19}F NMR spectrum (376.54 MHz, *o*DFB, 298 K) of a mixture of [{Ga(dcpe)}$_2$][*pf*]$_2$ and NC–CH$_3$ in *o*DFB after heating the reaction mixture to 60 °C for 4 days.

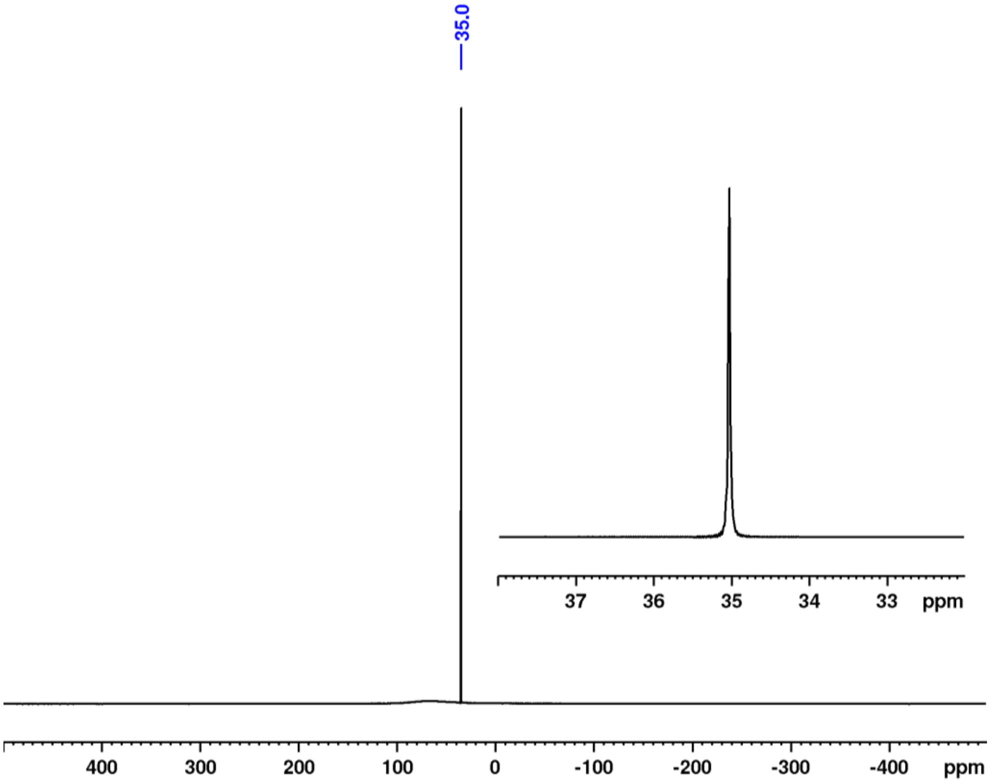

Figure S 113: ^{27}Al NMR spectrum (78.22 MHz, *o*DFB, 298 K) of a mixture of [{Ga(dcpe)}$_2$][*pf*]$_2$ and NC–CH$_3$ in *o*DFB after heating the reaction mixture to 60 °C for 4 days.

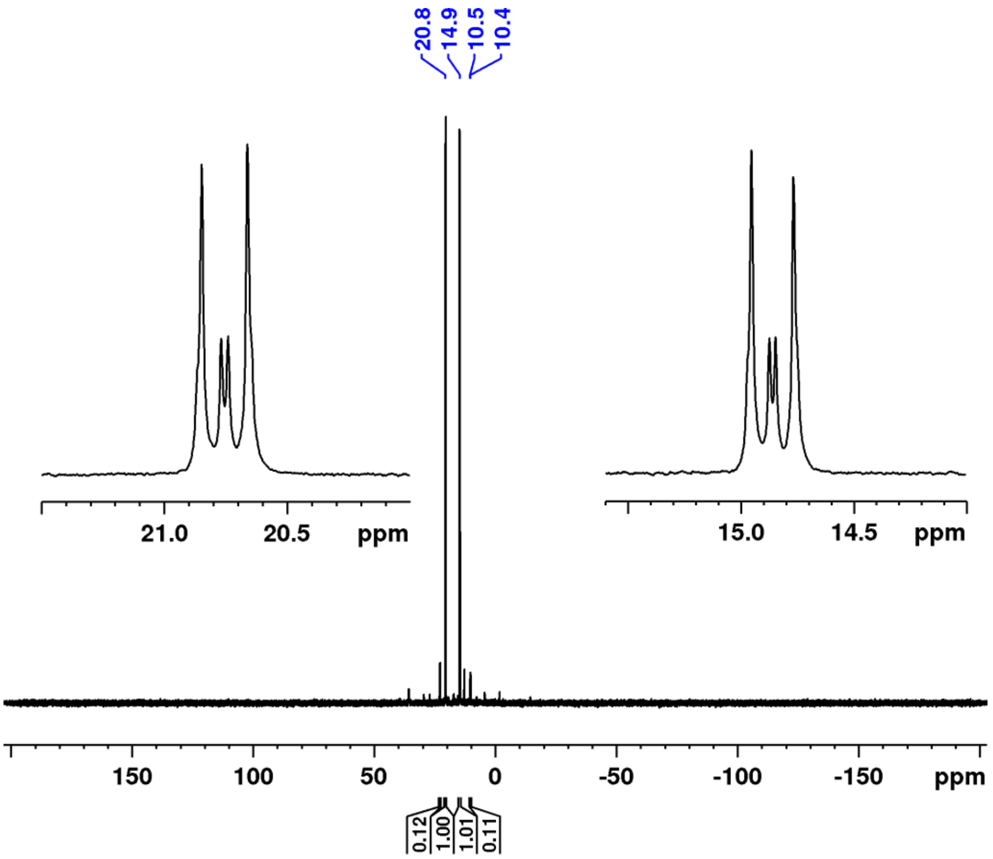

Figure S 114: $^{31}P\{^{1}H\}$ NMR spectrum (161.99 MHz, *o*DFB, 298 K) of a mixture of [{Ga(dcpe)}$_2$][*pf*]$_2$ and NC–CH_3 in *o*DFB after heating the reaction mixture to 60 °C for 4 days.

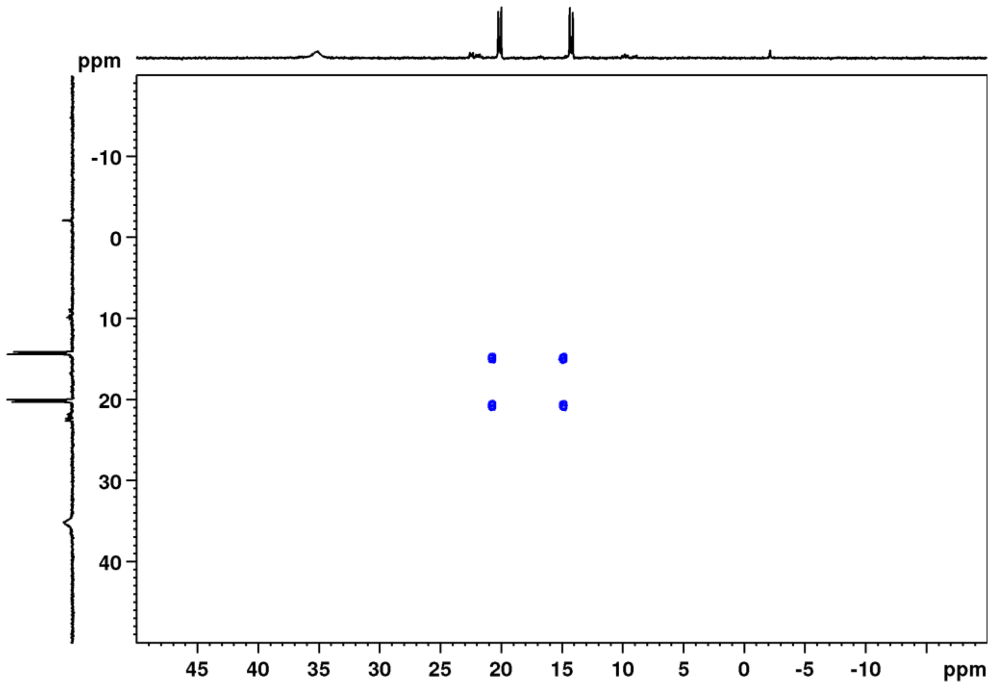

Figure S 115: $^{31}P,\{^{1}H\}$-COSY NMR spectrum (121.52 MHz, *o*DFB, 298 K, optimized for $J = 15$ Hz) of a mixture of [{Ga(dcpe)}$_2$][*pf*]$_2$ and NC–CH_3 in *o*DFB after heating the reaction mixture to 60 °C for 4 days**.**

4.9 Reaction between H_3C–CN and D_3C–CN with [{Ga(dcpe)}$_2$][*pf*]$_2$

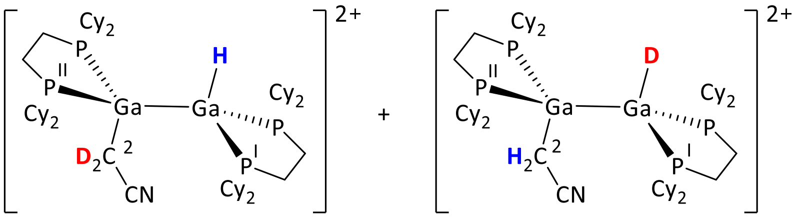

(+ isotopologues)

When heating a 2:1:1 mixture of [**1**][*pf*]$_2$, acetonitrile and acetonitrile-d_3, the ^1H and $^{31}P\{^1H\}$ spectra are similar to the spectra obtained for a 1:1 mixture of [**1**][*pf*]$_2$ and acetonitrile (see section 4.4). Note that the more complex signals in the $^{31}P\{^1H\}$ spectrum are caused by $^{31}P/^2D$ couplings and the isotope shifts on the ^{31}P NMR resonances (**Figure S 114** vs. **Figure S 117**). Obviously, the H/D exchange in the Ga–CH_2CN and/or the Ga–H group leads only to small isotope shifts of the resonances of the phosphorous atoms and has no significant effect on the chemical shift of the Ga**H** proton.

However, a noticeable isotope shift is observed for the carbon atom $\mathbf{C}^2H_2/D_2$ (ca. −3.1 vs −2.5 ppm). The fact that the Ga**H** proton, which resonates at 4.67 ppm, shows a cross peak in the $^1H,^{13}C$ NMR spectrum to *both* ^{13}C signals (*cf.* **Figure S 118** and **Figure S 119**) clearly indicates that two different products were formed: one product with GaH/GaCH_2 and the other product with GaH/GaCD_2 moieties.

Strictly speaking, the analogous isotopologues with GaD/GaCH_2 and with GaD/GaCD_2 moieties are not detected with this NMR experiment. However, since our results prove that the X and CX_2CN parts of one CX_3CN substrate molecule (X = H or D) are not transferred to the same digallene molecule, it is reasonable to assume that these isotopologues were also formed.

It is important to note that, when heating a 1:1 mixture of [H{Ga(dcpe)}$_2$(CH_2CN)][*pf*]$_2$ and [D{Ga(dcpe)}$_2$(CD_2CN)][*pf*]$_2$ to 60 °C for 4 days, no formation of [H{Ga(dcpe)}$_2$(CD_2CN)][*pf*]$_2$ was observed. Thus, for the reaction of H_3C–CN and D_3C–CN with [{Ga(dcpe)}$_2$][*pf*]$_2$, scrambling of Ga–H and Ga–D can be ruled out.

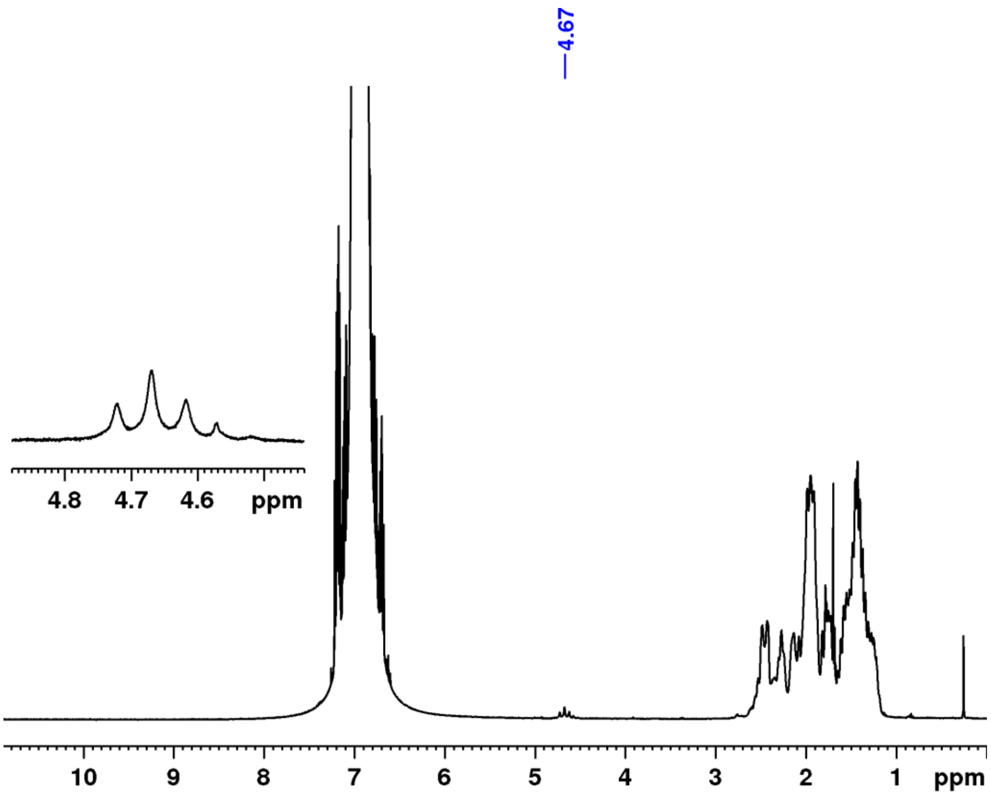

Figure S 116: ^{1}H NMR spectrum (400.17 MHz, *o*DFB, 298 K) of a mixture of [{Ga(dcpe)}$_2$][*pf*]$_2$, H_3C–CN and D_3C–CN (2 : 1 : 1) in *o*DFB after heating the reaction mixture to 60 °C for 2 days.

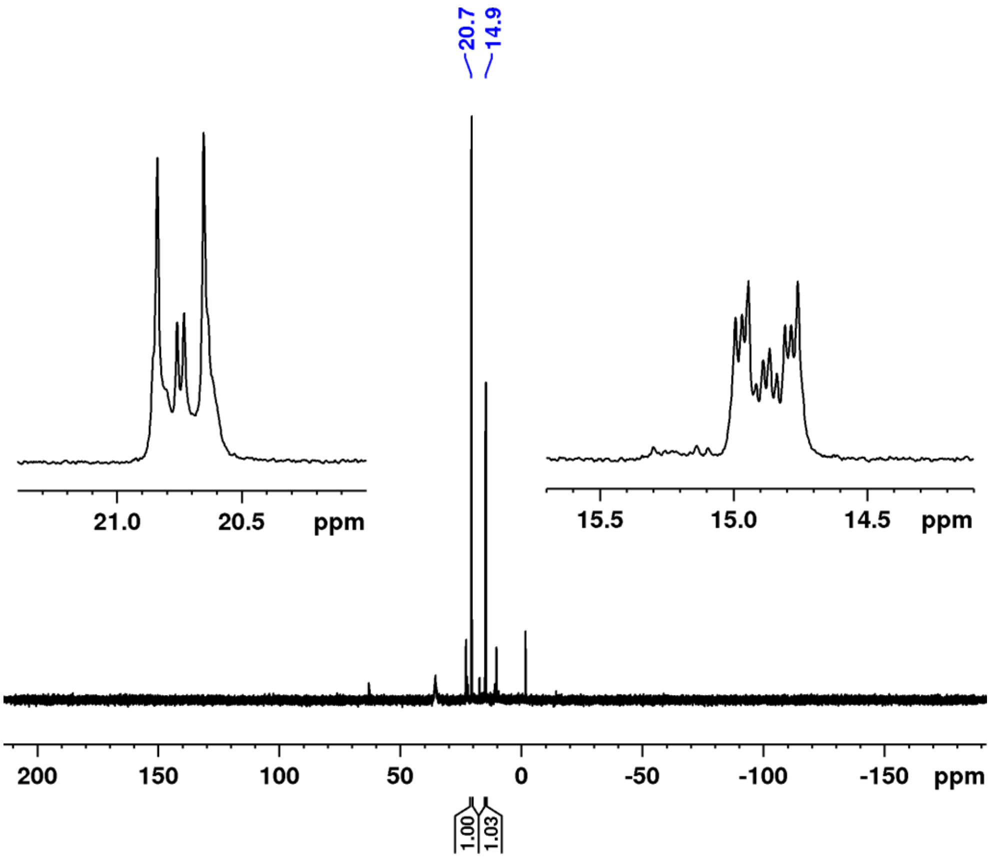

Figure S 117: $^{31}P\{^{1}H\}$ NMR spectrum (161.99 MHz, *o*DFB, 298 K) of a mixture of [{Ga(dcpe)}$_2$][*pf*]$_2$, H_3C–CN and D_3C–CN (2 : 1 : 1) in *o*DFB after heating the reaction mixture to 60 °C for 2 days.

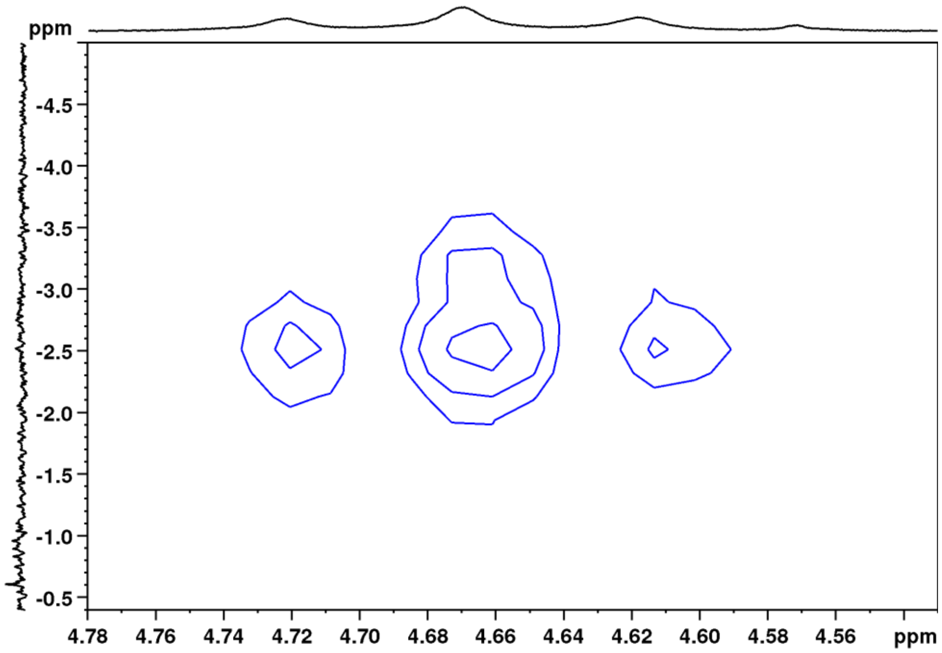

Figure S 118: $^{1}H,^{13}C$-HMBC NMR spectrum (400.17 MHz, *o*DFB, 298 K, optimized for $J = 8$ Hz) of a mixture of [{Ga(dcpe)}$_2$][*pf*]$_2$, H_3C–CN and D_3C–CN (2 : 1 : 1) in *o*DFB after heating the reaction mixture to 60 °C for 2 days.

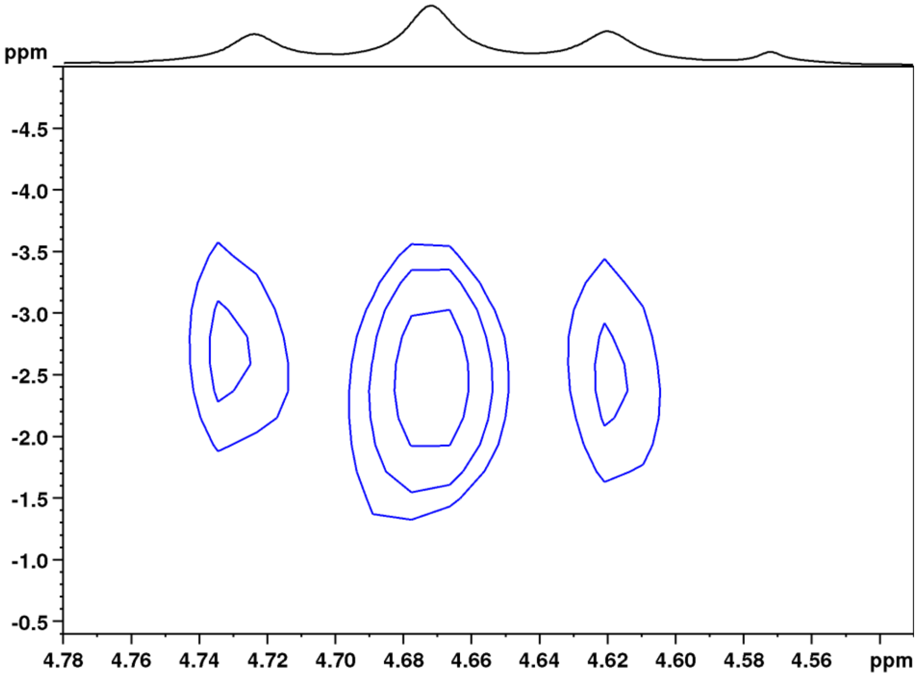

Figure S 119: $^{1}H,^{13}C$-HMBC NMR spectrum (400.17 MHz, *o*DFB, 298 K, optimized for $J = 16$ Hz) of a mixture of [{Ga(dcpe)}$_2$][*pf*]$_2$ and H_3C–CN in *o*DFB after heating the reaction mixture to 60 °C for 4 days (for comparison with **Figure S 118**).

4.10 [{Ga(dcpe)}$_2$(NCCH$_2$F)][*pf*]$_2$ and its Decomposition in Solution

4.10.1 [{Ga(dcpe)}$_2$(NCCH$_2$F)][*pf*]$_2$

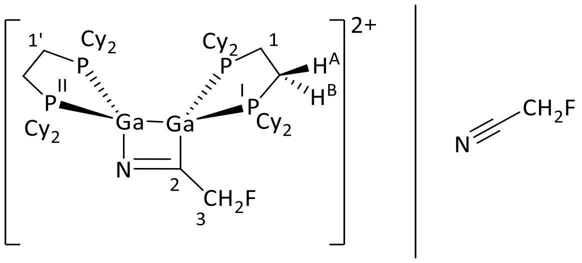

^{1}H NMR [400.17 MHz, *o*DFB, calibrated at *o*C_6F_2**H_4** = 6.96 ppm, 298 K]: δ = 4.90 (d, 2 H, C^3**H_2**, $^{2}J_{H,F}$ = 51.0 Hz), 4.76 (d, C**H_2**F in starting material, $^{2}J_{H,F}$ = 46.0 Hz), 2.72–2.40 (m, 8 H, C^1**H^A**, C^1**H^B**, $C^{1'}$**H^A** and $C^{1'}$**H^B**), 2.50–2.29 (m, 8 H, C**H** protons of the Cy groups), 2.10–1.26 (m, 80 H, C**H_2** protons of the Cy groups) ppm.

^{13}C NMR [100.62 MHz, *o*DFB, 298 K]: δ = 215.2 (1 C, **C^2**), 87.4 (1 C, **C^3**), 113.6 and 66.0 (N**C** and **C**H_2F in starting material), 33.8–31.6 (8 C, **C**H carbon atoms of the Cy groups), 30.1–25.0 (40 C, **C**H_2 carbon atoms of the Cy groups), 15.8 and 15.5 (4 C, **C^1** and **$C^{1'}$**) ppm.

^{19}F NMR [376.54 MHz, *o*DFB, 298 K]: δ = –75.3 (s, 36 F, [Al(OC(C**F_3**)$_3$)$_4$]$^-$), –113.9 (m, Ph**F**), –139.5 (m, *o*C_6**F_2**H_4), –202.3 (t, 1 F, C^3**F**, $^{2}J_{F,H}$ = 51.0 Hz), –232.5 (CH_2**F** in starting material) ppm.

^{27}Al NMR [104.27 MHz, *o*DFB, 298 K]: δ = 35.0 (s, 1 Al, [**Al**(OC(CF_3)$_3$)$_4$]$^-$) ppm.

^{31}P NMR [161.99 MHz, *o*DFB, 298 K]: δ = 13.9 (m, 2 P, **P^I**), 12.9 (m, 2 P, **P^{II}**) ppm.

^{71}Ga NMR [122.04 MHz, *o*DFB, 298 K]: signal probably too broad to be detected, due to the quadrupolar relaxation of ^{71}Ga.

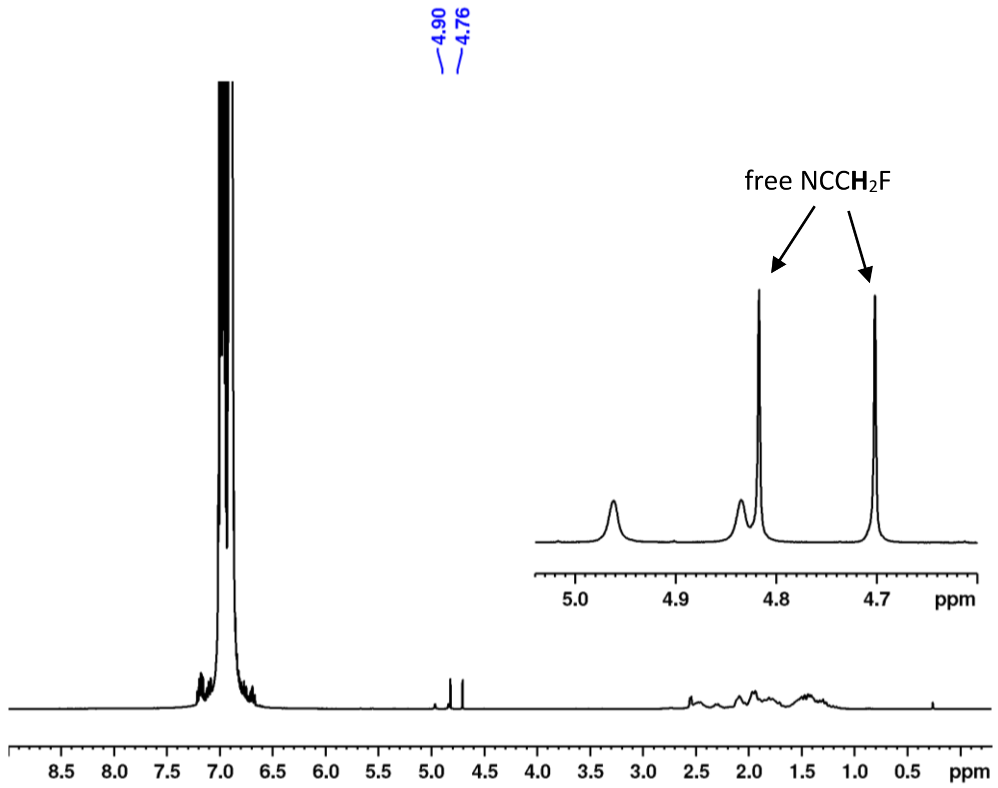

Figure S 120: ^{1}H NMR spectrum (400.17 MHz, *o*DFB, 298 K) of a mixture of [{Ga(dcpe)}$_2$][*pf*]$_2$ and NC–CH$_2$F, 3 h after mixing the components in *o*DFB.

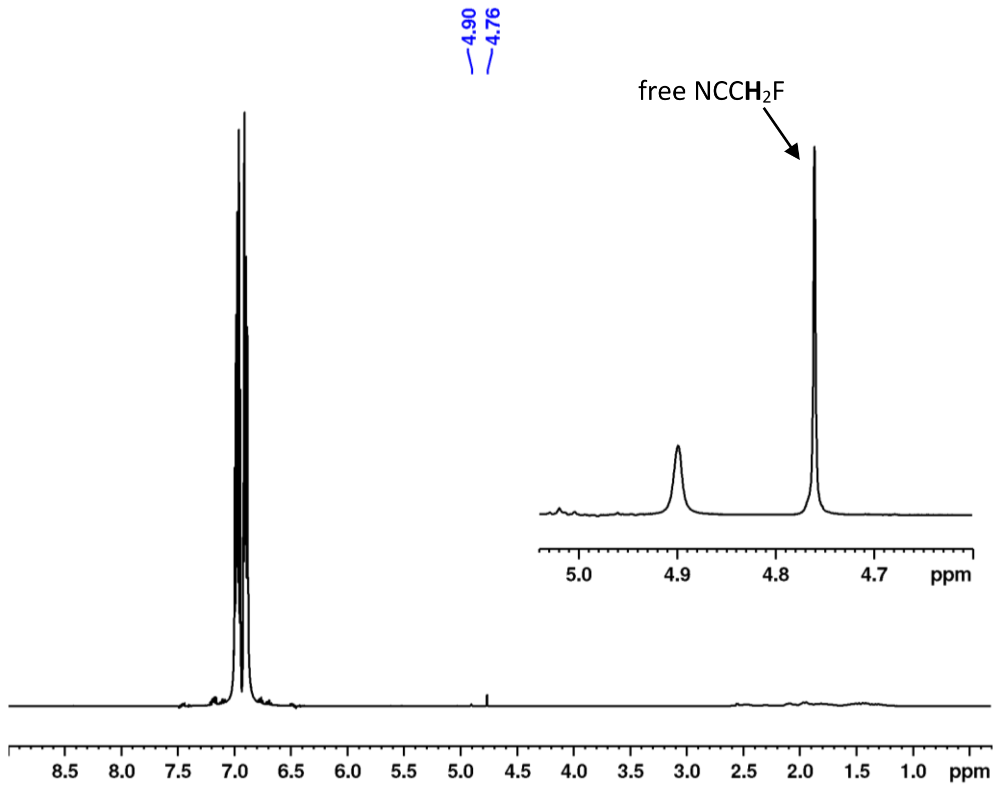

Figure S 121: $^{1}H\{^{19}F\}$ NMR spectrum (400.17 MHz, *o*DFB, 298 K) of a mixture of [{Ga(dcpe)}$_2$][*pf*]$_2$ and NC–CH$_2$F, 3 h after mixing the components in *o*DFB.

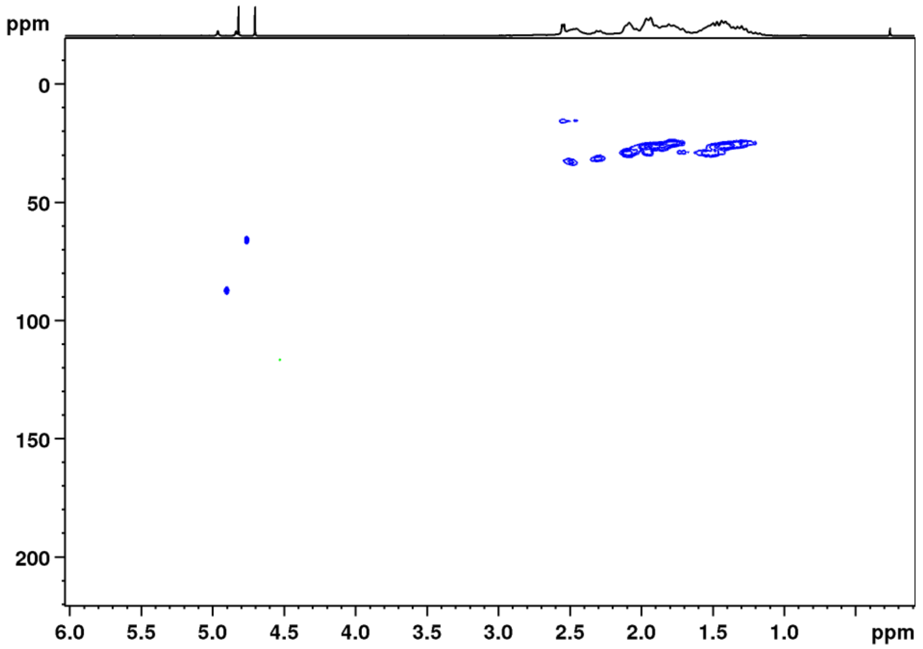

Figure S 122: ^{1}H,^{13}C-HSQC NMR spectrum (400.17 MHz, *o*DFB, 298 K, optimized for $J = 145$ Hz) of a mixture of [{Ga(dcpe)}$_2$][*pf*]$_2$ and NC–CH_2F, 3 d after mixing the components in *o*DFB.

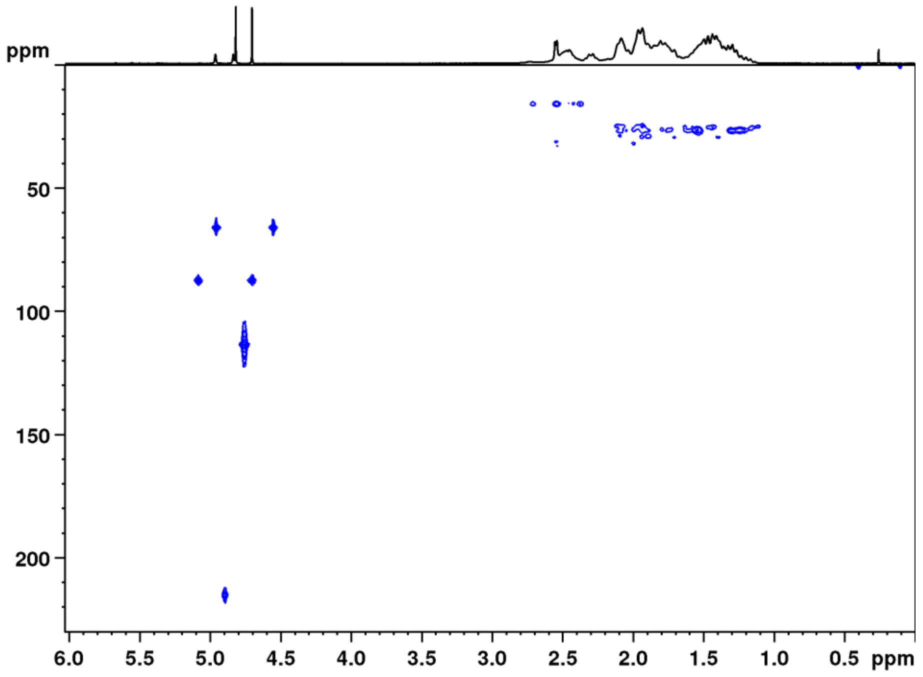

Figure S 123: ^{1}H,^{13}C-HMBC NMR spectrum (400.17 MHz, *o*DFB, 298 K, optimized for $J = 8$ Hz) of a mixture of [{Ga(dcpe)}$_2$][*pf*]$_2$ and NC–CH_2F, 3 d after mixing the components in *o*DFB.

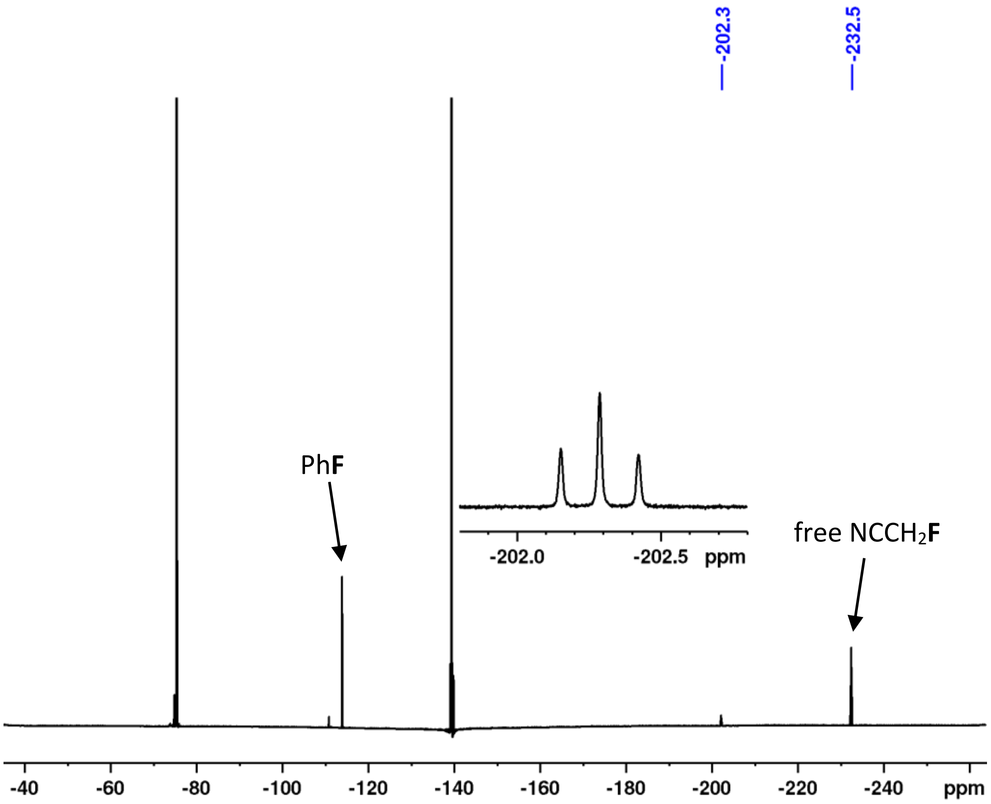

Figure S 124: ^{19}F NMR spectrum (376.54 MHz, *o*DFB, 298 K) of a mixture of [{Ga(dcpe)}$_2$][*pf*]$_2$ and NC–CH$_2$F, 3 h after mixing the components in *o*DFB.

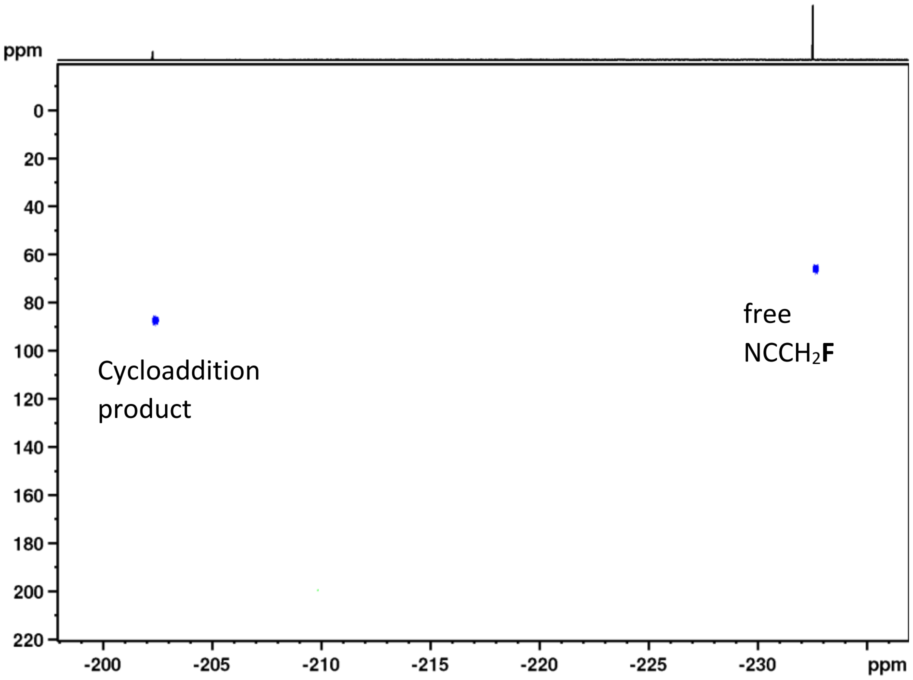

Figure S 125: ^{19}F,^{13}C{^{1}H}-HSQC NMR spectrum (376.54 MHz, *o*DFB, 298 K, optimized for *J* = 250 Hz) of a mixture of [{Ga(dcpe)}$_2$][*pf*]$_2$ and NC–CH$_2$F in *o*DFB.

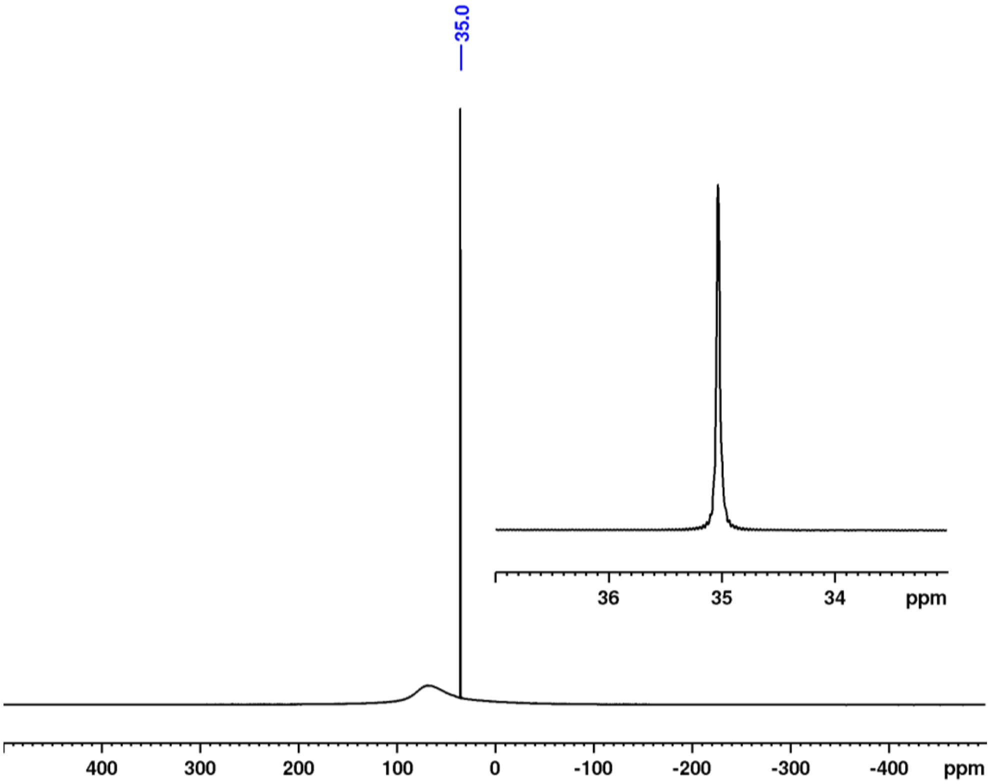

Figure S 126: ^{27}Al NMR spectrum (104.27 MHz, *o*DFB, 298 K) of a mixture of [{Ga(dcpe)}$_2$][*pf*]$_2$ and NC–CH$_2$F, 3 h after mixing the components in *o*DFB.

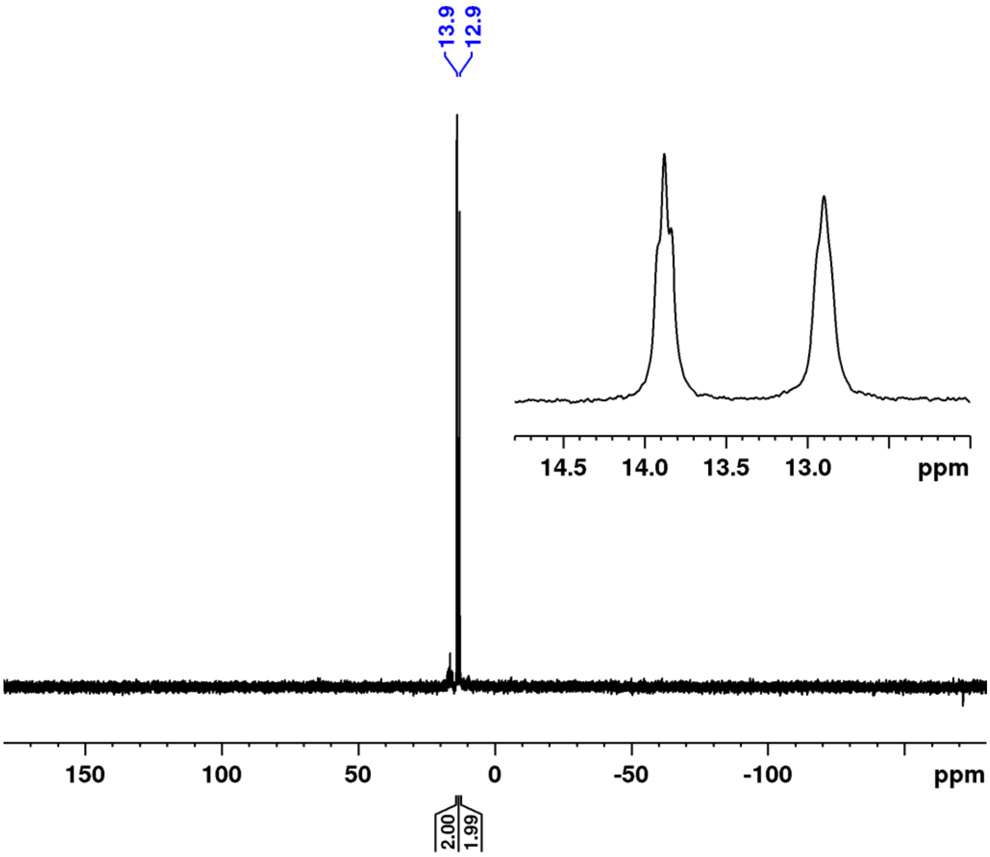

Figure S 127: ^{31}P{^{1}H} NMR spectrum (81.01 MHz, *o*DFB, 298 K) of a mixture of [{Ga(dcpe)}$_2$][*pf*]$_2$ and NC–CH$_2$F, 3 h after mixing the components in *o*DFB.

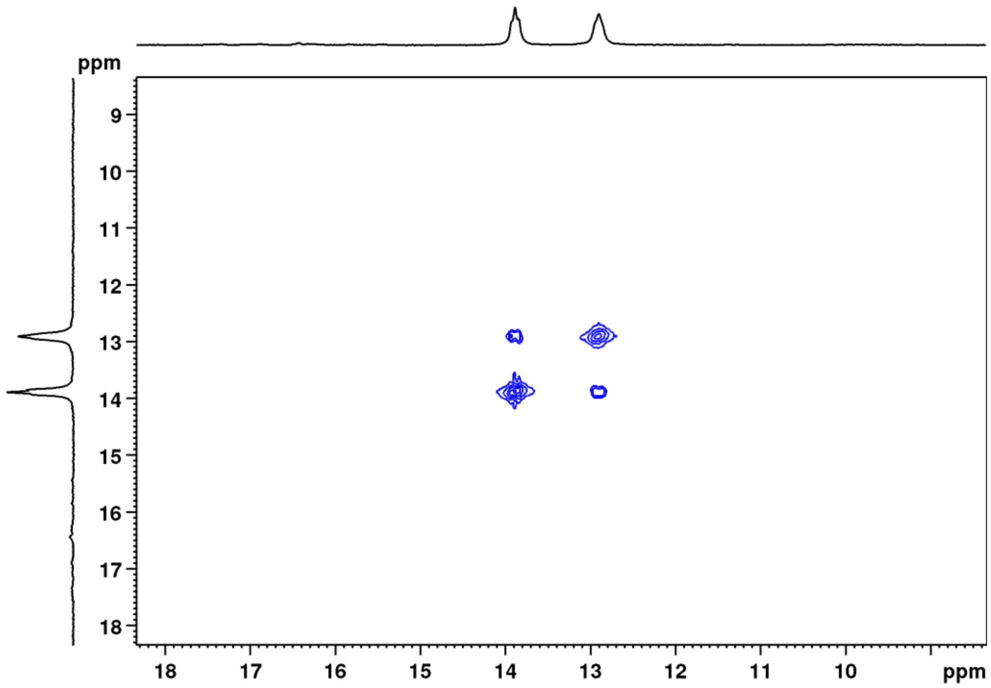

Figure S 128: $^{31}P\{^{1}H\}$-COSY NMR spectrum (81.01 MHz, *o*DFB, 298 K) of a mixture of [{Ga(dcpe)}$_2$][*pf*]$_2$ and NC–CH_2F, 4 h after mixing the components in *o*DFB.

4.10.2 Decomposition of [{Ga(dcpe)}$_2$(NCCH$_2$F)]$^{2+}$ in Solution

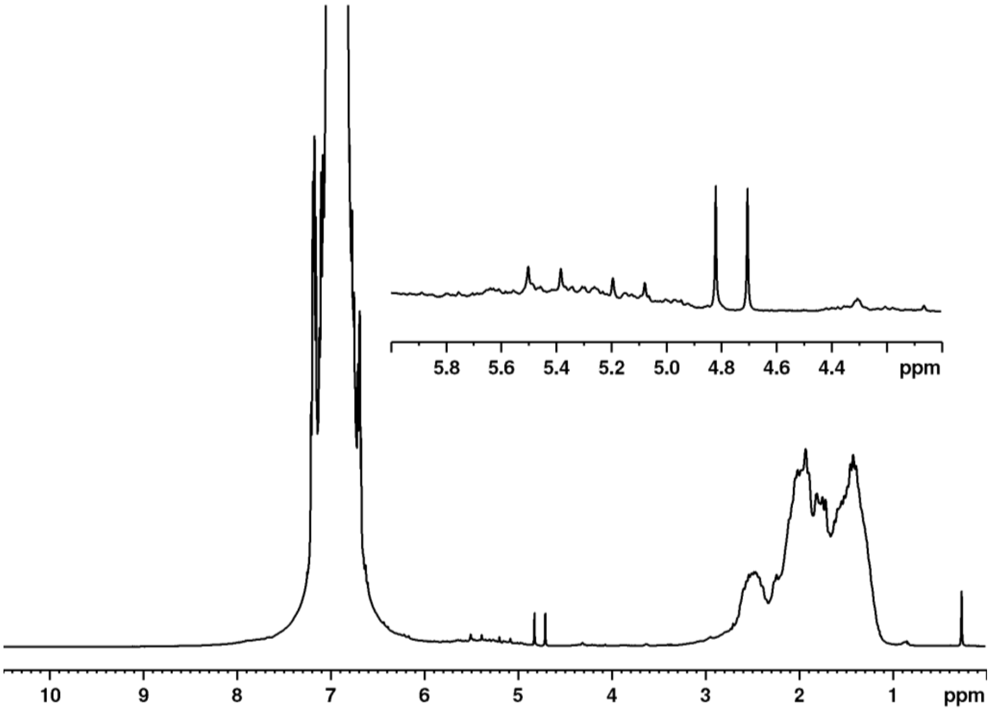

Figure S 129: ^{1}H NMR spectrum (400.17 MHz, *o*DFB, 298 K) of a mixture of [{Ga(dcpe)}$_2$][*pf*]$_2$ and NC–CH_2F in *o*DFB, 4 weeks after mixing the components.

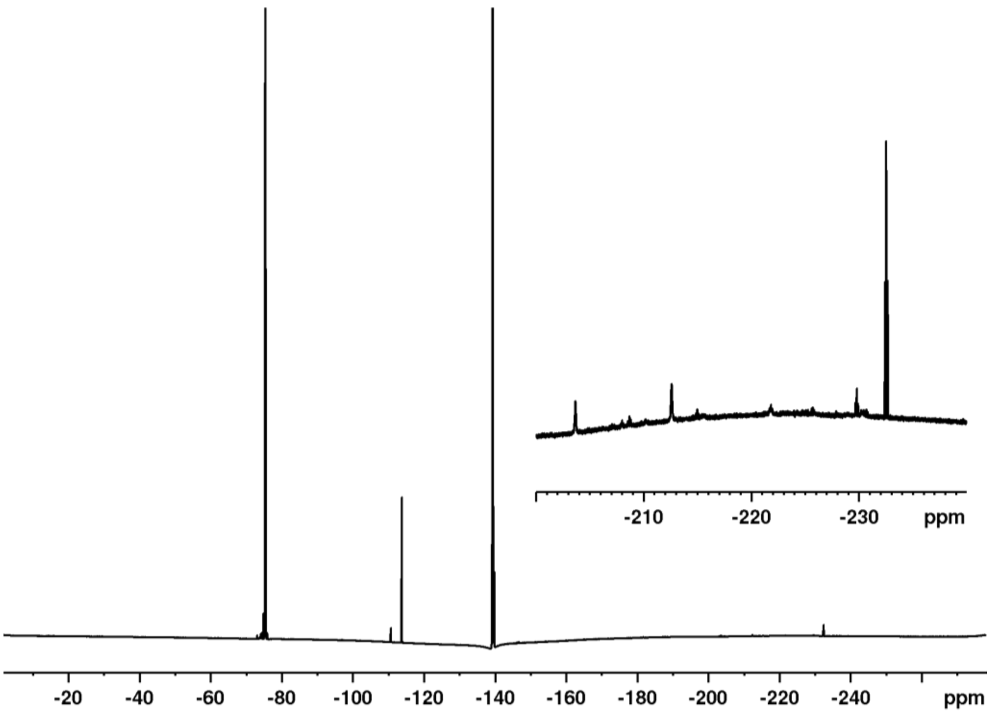

Figure S 130: ^{19}F NMR spectrum (376.54 MHz, *o*DFB, 298 K) of a mixture of [{Ga(dcpe)}$_2$][*pf*]$_2$ and NC–CH_2F in *o*DFB, 4 weeks after mixing the components.

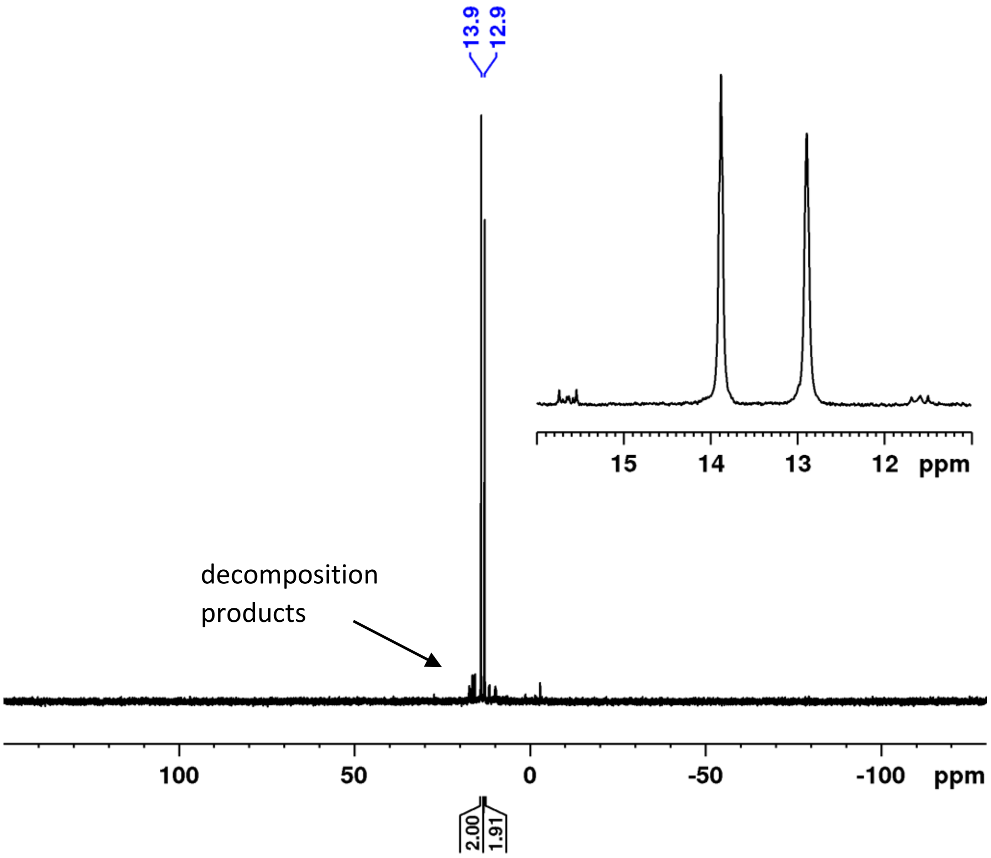

Figure S 131: $^{31}P\{^1H\}$ NMR spectrum (161.99 MHz, *o*DFB, 298 K) of a mixture of [{Ga(dcpe)}$_2$][*pf*]$_2$ and NC–CH_2F in *o*DFB, 2 days after mixing the components.

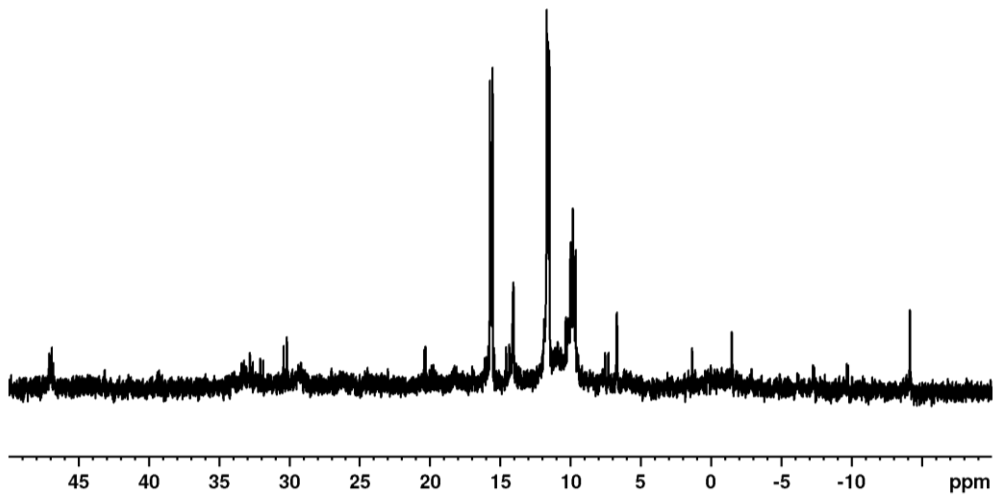

Figure S 132: $^{31}P\{^{1}H\}$ NMR spectrum (161.99 MHz, *o*DFB, 298 K) of a mixture of $[\{Ga(dcpe)\}_2][pf]_2$ and NC–CH_2F in *o*DFB, 4 weeks after mixing the components.

4.11 $[\{Ga(dcpe)\}_2(NCPh)][pf]_2$ and its Decomposition in Solution

4.11.1 $[\{Ga(dcpe)\}_2(NCPh)][pf]_2$

Due to the rapid decomposition of the putative $[\{Ga(dcpe)\}_2(NCPh)]^{2+}$, which is already noticeable after 2 h (see broad signal at 32.8 ppm in the $^{31}P\{^{1}H\}$ NMR spectrum in **Figure S 134**) and thus faster than for the cycloaddition product with $NCCH_2F$, the NMR signals could not be assigned in detail. However, the $^{1}H,^{31}P$ crosspeak in the aromatic region and the AA'BB' spin system in the $^{31}P\{^{1}H\}$ NMR spectrum strongly indicate the formation of the cycloaddition product.

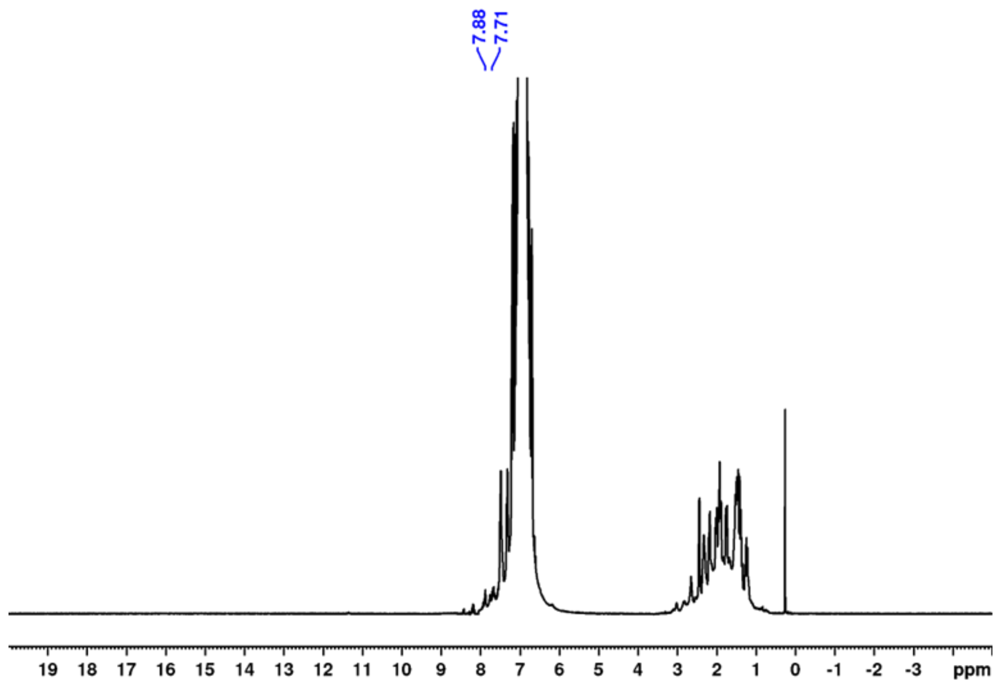

Figure S 133: ^{1}H NMR spectrum [400.17 MHz, 2FB, 298 K] of a mixture of $[\{Ga(dcpe)\}_2][pf]_2$ and benzonitrile, 2 h after mixing the components in *o*DFB.

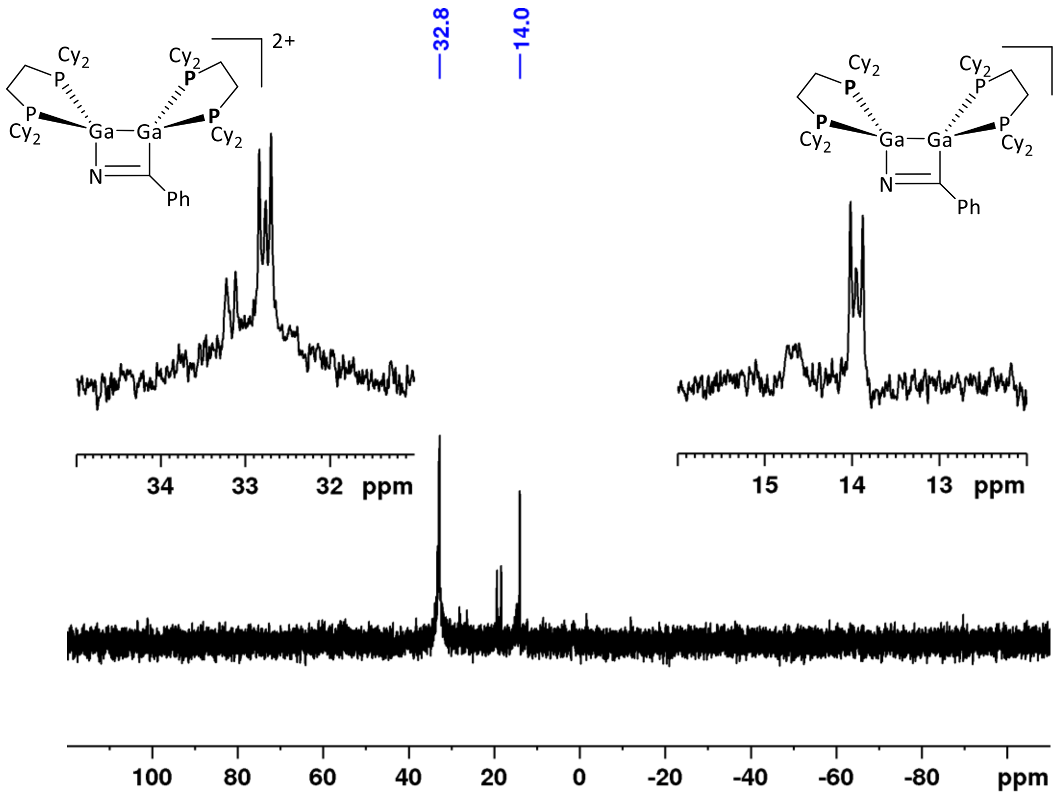

Figure S 134: $^{31}P\{^{1}H\}$ NMR spectrum [81.01 MHz, 2FB, 298 K] of a mixture of [{Ga(dcpe)}₂][*pf*]₂ and benzonitrile, 2 h after mixing the components in *o*DFB.

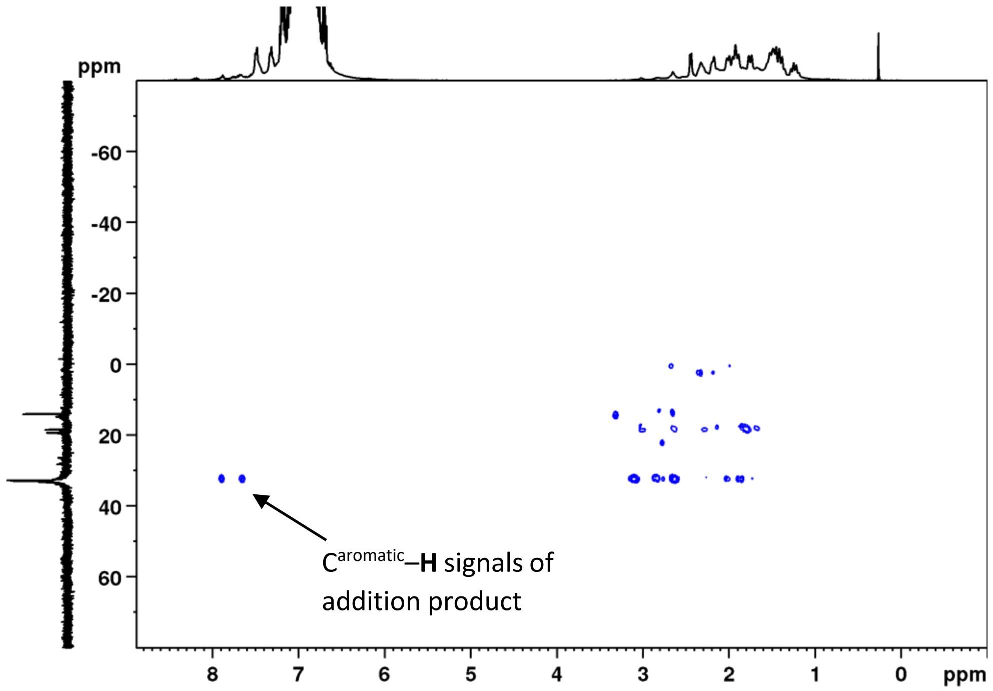

Figure S 135: $^{1}H,^{31}P$-HMBC NMR spectrum (400.17 MHz, *o*DFB, 298 K, optimized for *J* = 15 Hz) of a mixture of [{Ga(dcpe)}₂][*pf*]₂ and benzonitrile, 5 h after mixing the components in *o*DFB.

4.11.2 Decomposition of $[\{Ga(dcpe)\}_2(NCPh)]^{2+}$ in Solution

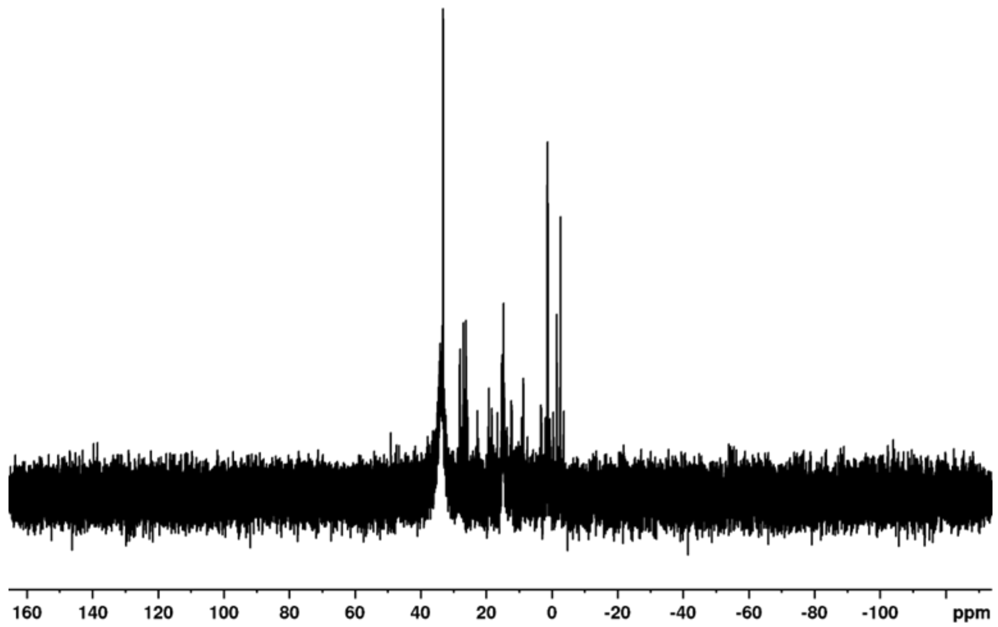

Figure S 136: $^{31}P\{^1H\}$ NMR spectrum [161.99 MHz, *o*FB, 298 K] of a mixture of $[\{Ga(dcpe)\}_2]$[*pf*]$_2$ and benzonitrile, 1 d after mixing the components in *o*DFB.

4.12 [F{Ga(dcpe)}$_2$(CH_2CN)][*pf*]$_2$

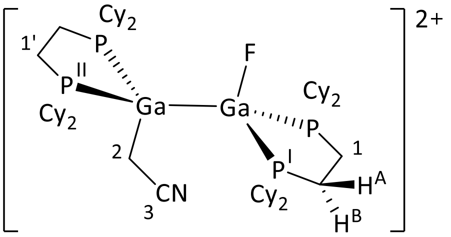

^{1}H NMR [300.18 MHz, *o*DFB, calibrated at *o*C_6F_2**H_4** = 6.96 ppm, 298 K]: δ = 2.65–2.34 (m, 8 H, C^1**H**^{A}, C^1**H**^{B}, $C^{1'}$**H**^{A} and $C^{1'}$**H**^{B}), 2.48–2.33 (m, 8 H, C**H** protons of the Cy groups), 2. 0–1.25 (m, 80 H, C**H_2** protons of the Cy groups), 1.83 (t, 2 H, C^2**H_2**, $^3J_{H,P}$ = 3.8 Hz) ppm.

^{13}C NMR [75.48 MHz, *o*DFB, 298 K]: δ = 123.1 (1 C, **C**^{3}N), 33.3–32.3 (8 C, **C**H carbon atoms of the Cy groups), 29.4–25.2 (40 C, **C**H_2 carbon atoms of the Cy groups), 15.5 and 14.8 (4 C, **C**^{1} and **C**$^{1'}$), –3.5 (1 C, **C**^{2}) ppm.

^{19}F NMR [282.45 MHz, *o*DFB, 298 K]: δ = –75.3 (s, 36 F, [Al(OC(C**F_3**)$_3$)$_4$]$^-$), –113.9 (m, Ph**F**), –139.5 (m, *o*C_6**F_2**H_4), –203.7 (t, 1 F, **F**–Ga, $^2J_{F,PI}$ = 27.3 Hz) ppm.

^{27}Al NMR [78.22 MHz, *o*DFB, 298 K]: δ = 35.0 (s, 1 Al, [**Al**(OC(CF_3)$_3$)$_4$]$^-$) ppm.

^{31}P NMR [121.52 MHz, *o*DFB, 298 K]: δ = 30.3 (unknown impurity), 15.6 (m, 2 P, **P**^{II}), 11.6 (unknown impurity), 9.8 (m, 2 P, **P**^{I}) ppm.

^{71}Ga NMR [91.54 MHz, *o*DFB, 298 K]: signal probably too broad to be detected, due to the quadrupolar relaxation of ^{71}Ga.

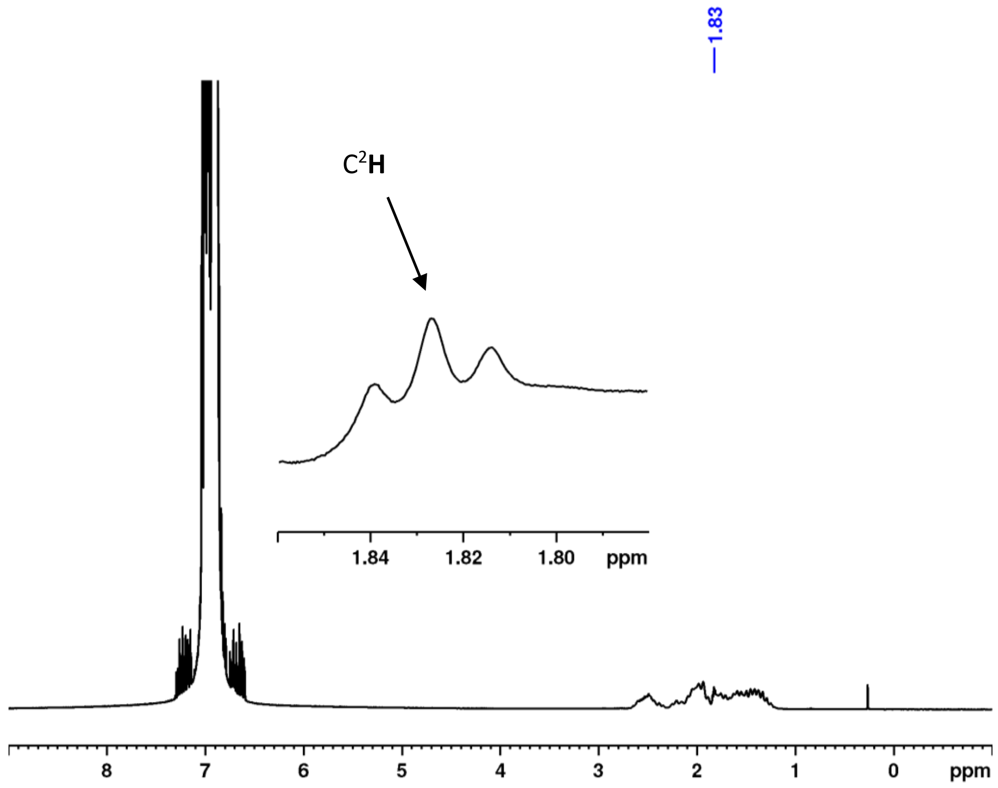

Figure S 137: ^{1}H NMR spectrum (300.18 MHz, *o*DFB, 298 K) of the crystals obtained upon layering an equimolar solution of [{Ga(dcpe)}$_2$][*pf*]$_2$ and NC–CH$_2$F in *o*DFB with *n*-pentane.

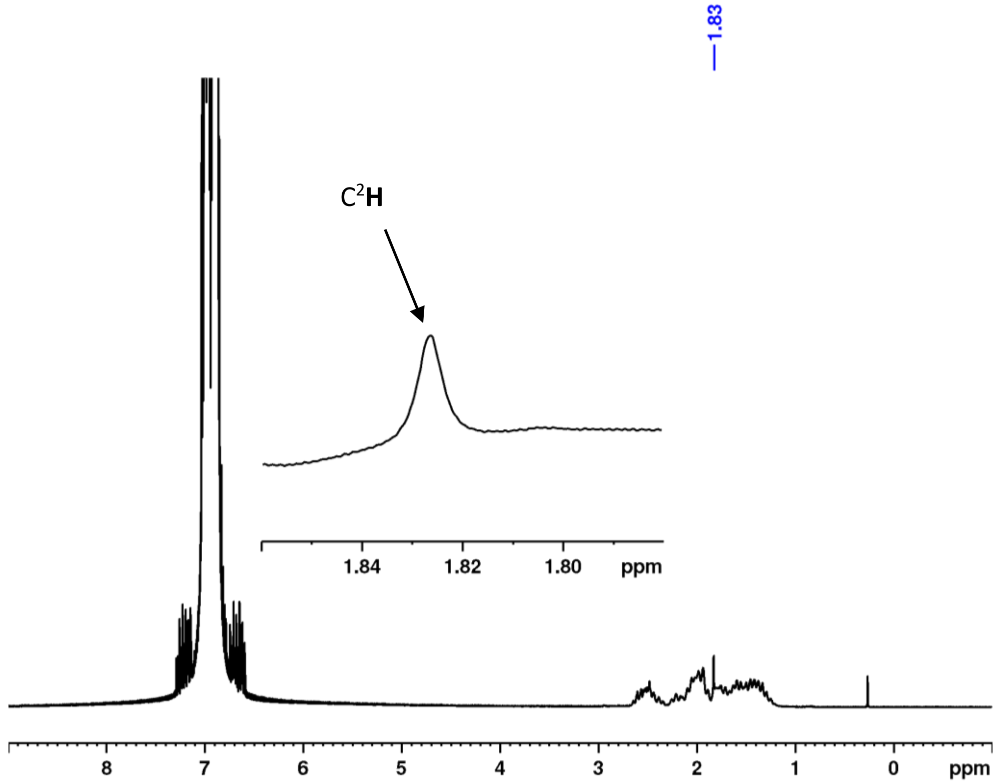

Figure S 138: ^{1}H{^{31}P} NMR spectrum (300.18 MHz, *o*DFB, 298 K) of the crystals obtained upon layering an equimolar solution of [{Ga(dcpe)}$_2$][*pf*]$_2$ and NC–CH$_2$F in *o*DFB with *n*-pentane.

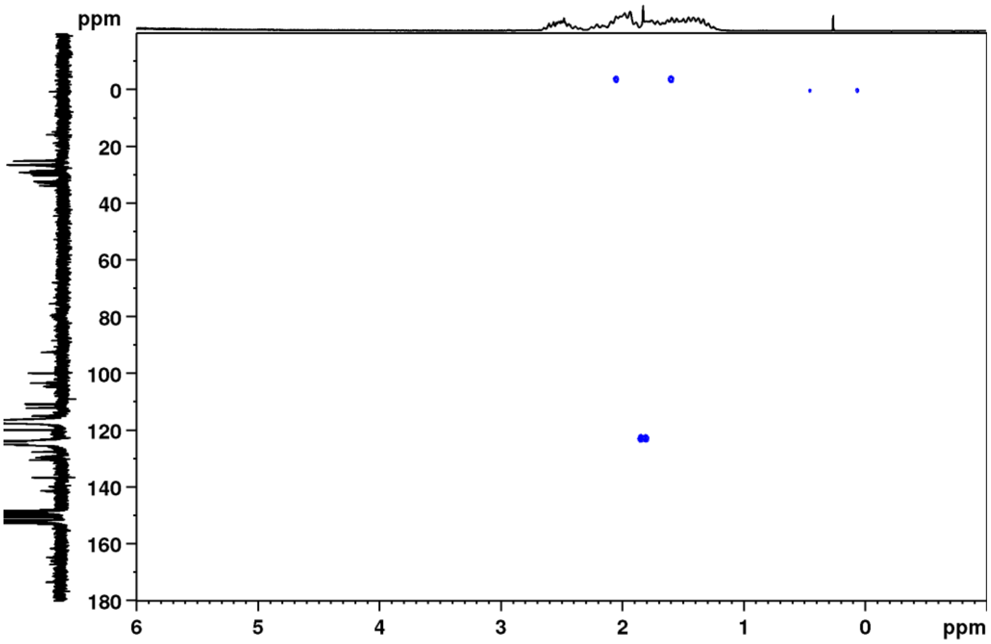

Figure S 139: $^{1}H,^{13}C$-HMBC NMR spectrum (300.18 MHz, *o*DFB, 298 K, optimized for J = 8 Hz) of the crystals obtained upon layering an equimolar solution of [{Ga(dcpe)}$_2$][*pf*]$_2$ and NC–CH_2F in *o*DFB with *n*-pentane.

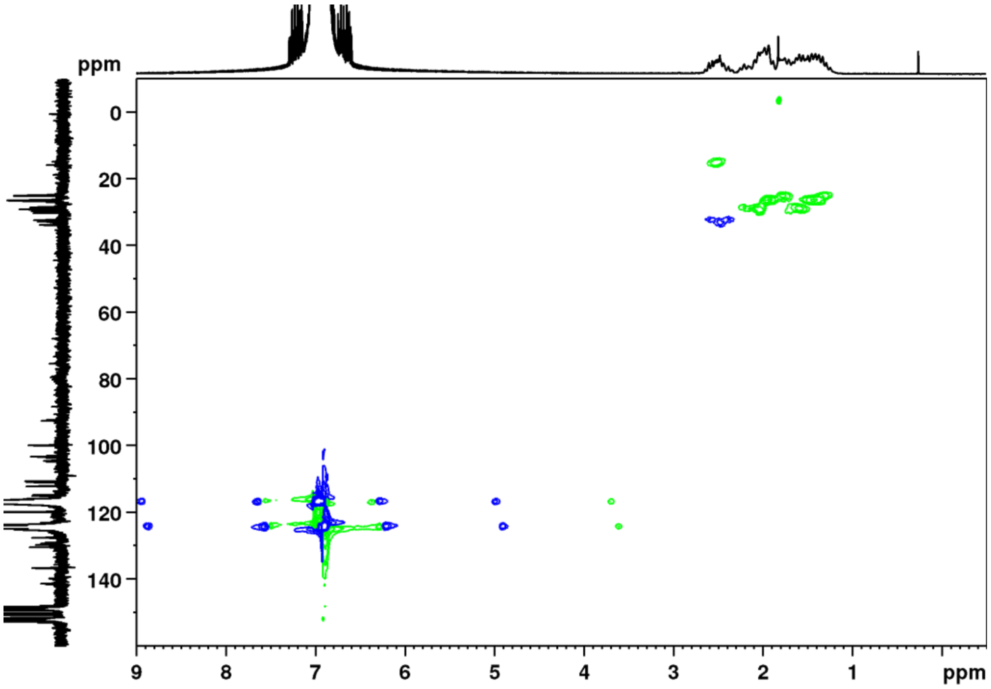

Figure S 140: Edited $^{1}H,^{13}C$-HSQC NMR spectrum (300.18 MHz, *o*DFB, 298 K, optimized for J = 145 Hz) of the crystals obtained upon layering an equimolar solution of [{Ga(dcpe)}$_2$][*pf*]$_2$ and NC–CH_2F in *o*DFB with *n*-pentane.

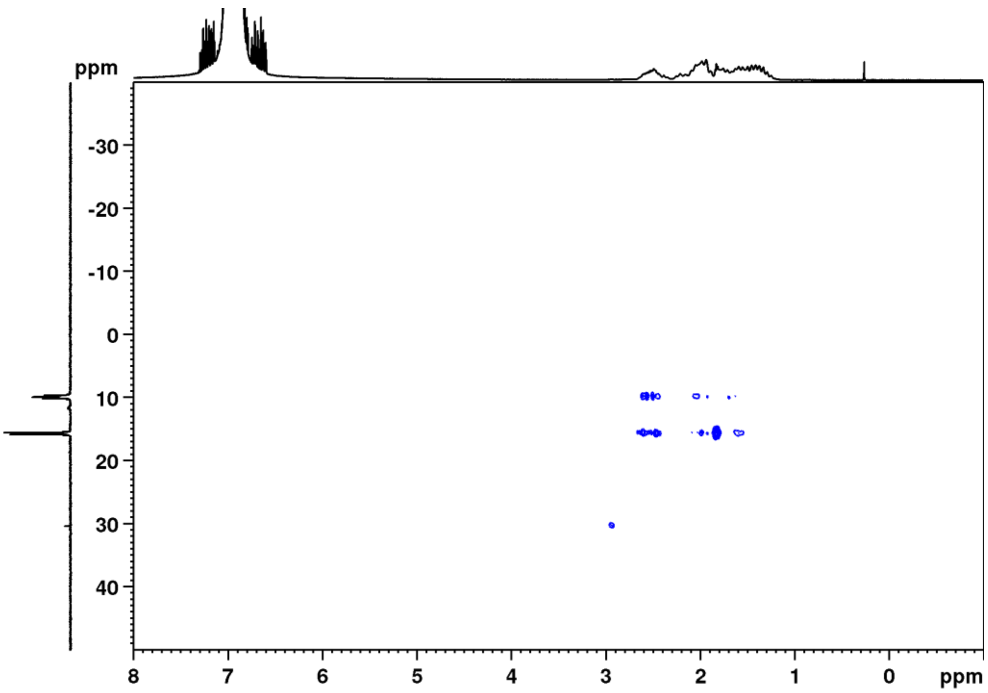

Figure S 141: $^{1}H,^{31}P$-HMBC NMR spectrum (300.18 MHz, *o*DFB, 298 K, optimized for *J* = 15 Hz) of the crystals obtained upon layering an equimolar solution of [{Ga(dcpe)}$_2$][*pf*]$_2$ and NC–CH_2F in *o*DFB with *n*-pentane.

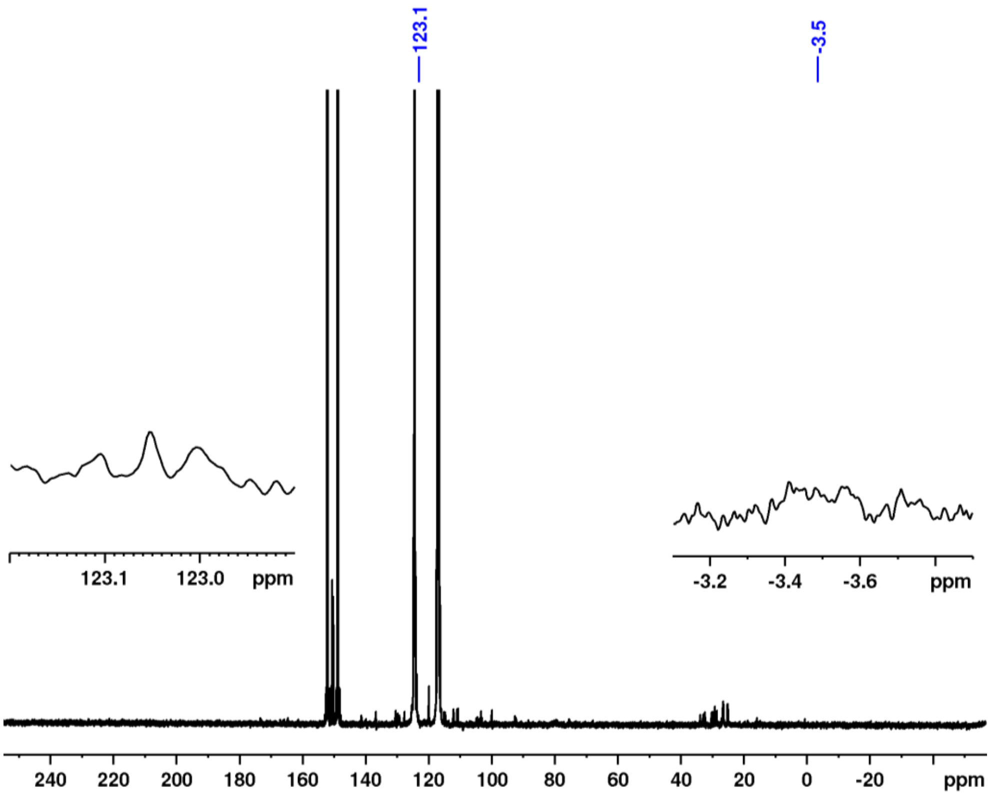

Figure S 142: $^{13}C\{^{1}H\}$ NMR spectrum (75.48 MHz, *o*DFB, 298 K) of the crystals obtained upon layering an equimolar solution of [{Ga(dcpe)}$_2$][*pf*]$_2$ and NC–CH_2F in *o*DFB with *n*-pentane.

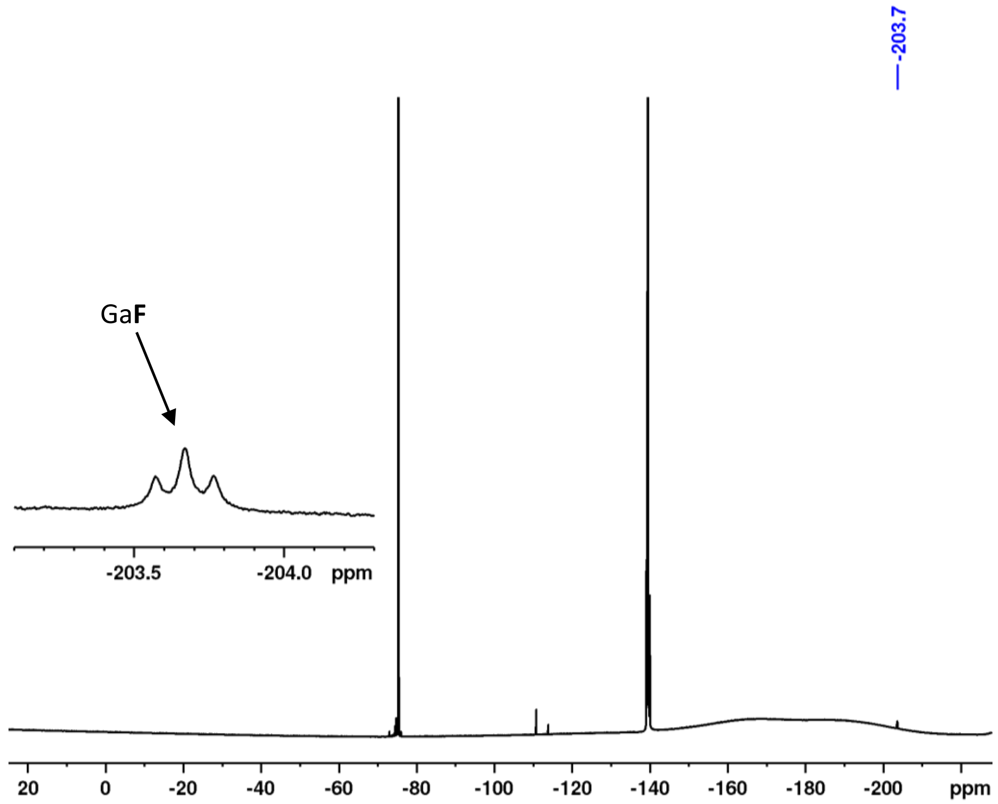

Figure S 143: ^{19}F NMR spectrum (282.45 MHz, *o*DFB, 298 K) of the crystals obtained upon layering an equimolar solution of $[\{Ga(dcpe)\}_2][pf]_2$ and NC–CH_2F in *o*DFB with *n*-pentane.

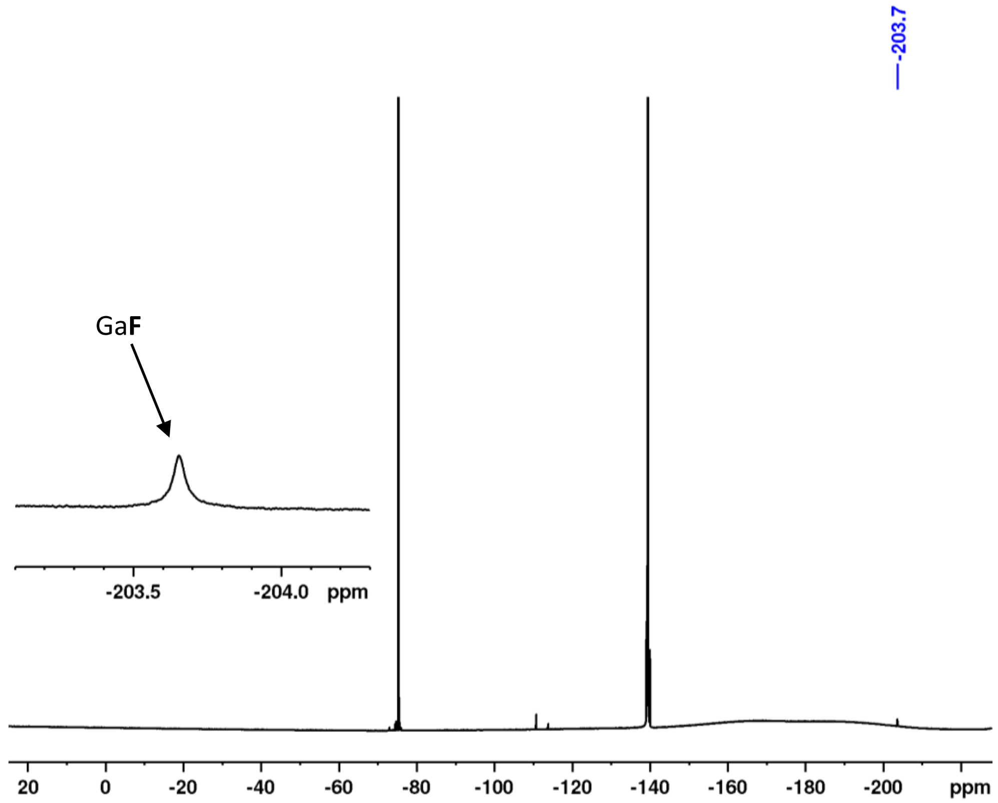

Figure S 144: $^{19}F\{^{31}P\}$ NMR spectrum (282.45 MHz, *o*DFB, 298 K) of the crystals obtained upon layering an equimolar solution of $[\{Ga(dcpe)\}_2][pf]_2$ and NC–CH_2F in *o*DFB with *n*-pentane.

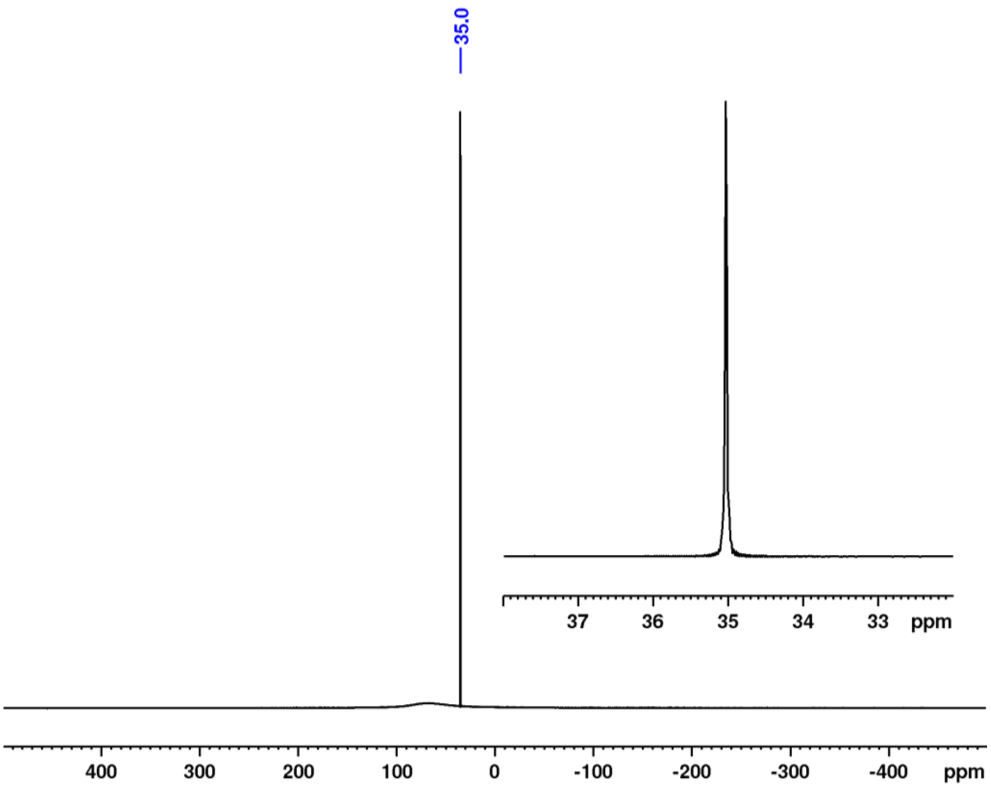

Figure S 145: ^{27}Al NMR spectrum (78.22 MHz, *o*DFB, 298 K) of the crystals obtained upon layering an equimolar solution of [{Ga(dcpe)}$_2$][*pf*]$_2$ and NC–CH$_2$F in *o*DFB with *n*-pentane.

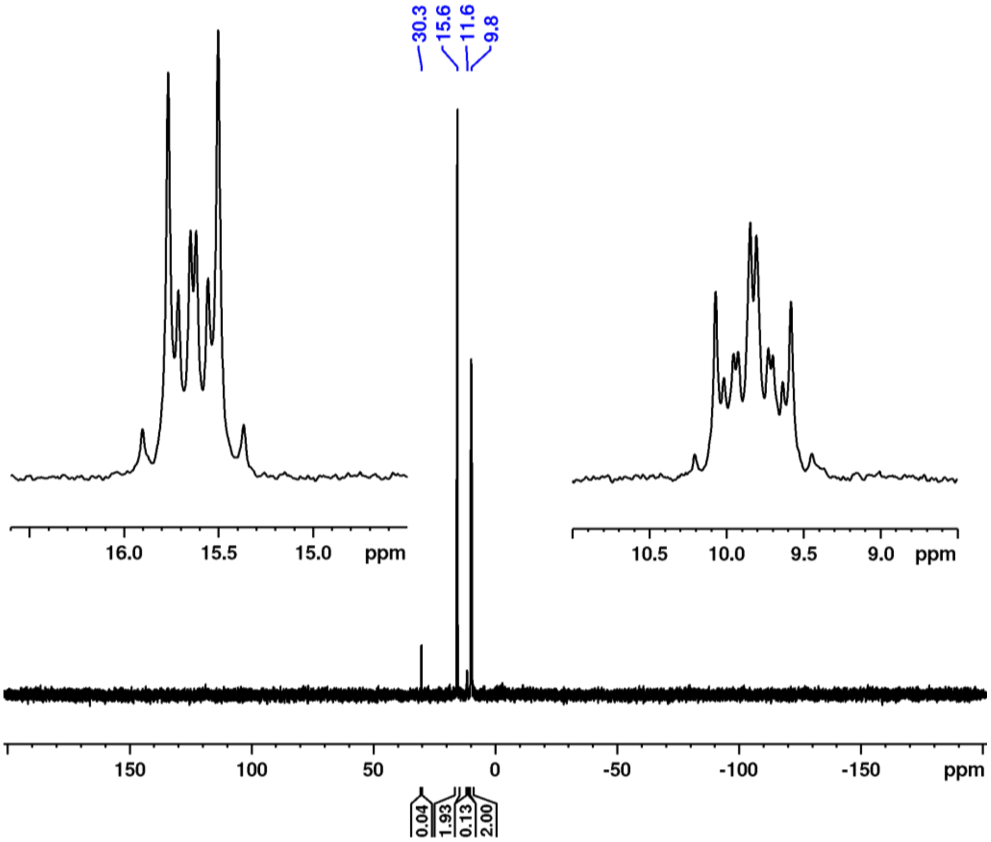

Figure S 146: ^{31}P{^{1}H} NMR spectrum (78.22 MHz, *o*DFB, 298 K) of the crystals obtained upon layering an equimolar solution of [{Ga(dcpe)}$_2$][*pf*]$_2$ and NC–CH$_2$F in *o*DFB with *n*-pentane.

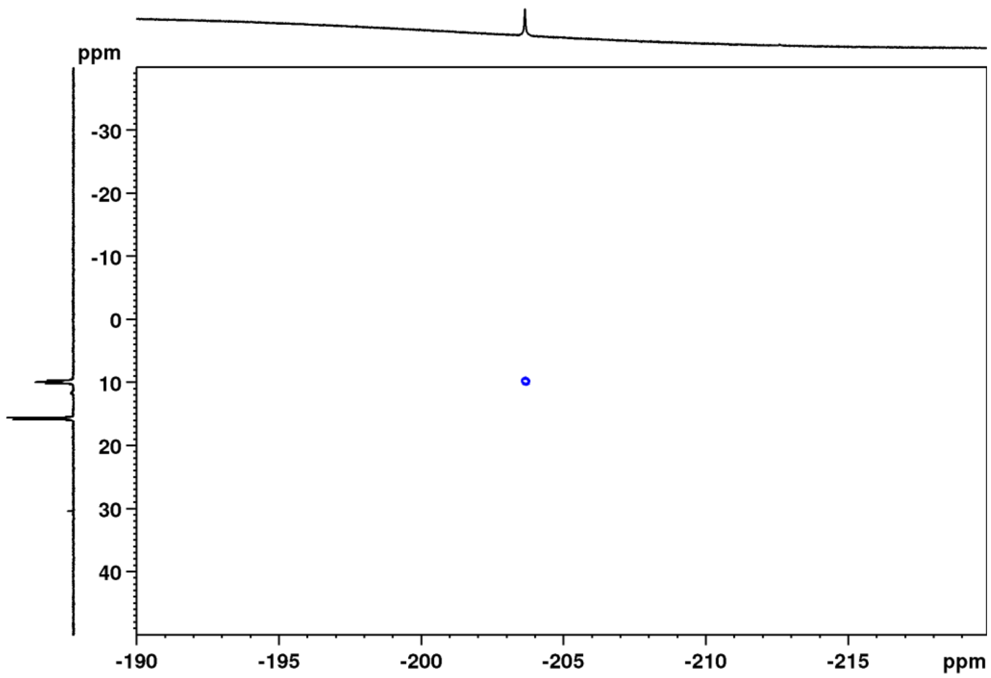

Figure S 147: $^{19}F,^{31}P$-HMBC NMR spectrum (282.45 MHz, *o*DFB, 298 K, optimized for $J = 30$ Hz) of the crystals obtained upon layering an equimolar solution of $[\{Ga(dcpe)\}_2][pf]_2$ and NC–CH_2F in *o*DFB with *n*-pentane.

4.13 [F{Ga(dcpe)}$_2$(C$_5$H$_6$F)][*pf*]$_2$

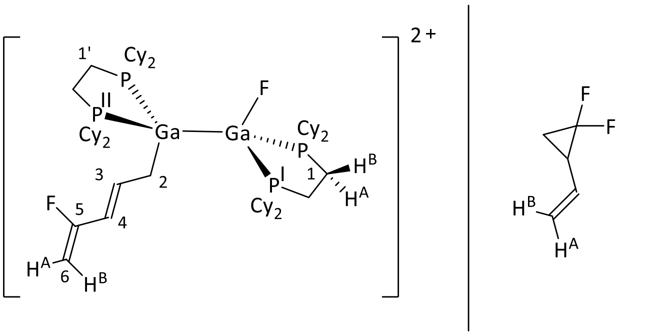

^{1}H NMR [400.17 MHz, *o*DFB, calibrated at *o*$C_6F_2\mathbf{H}_4$ = 6.96 ppm, 298 K]: δ = 6.16 (m, 1 H, $C^3\mathbf{H}$), 5.95 (m, 1 H, $C^4\mathbf{H}$), 5.44, 5.15 and 5.06 (m, olefinic protons in the starting material), 4.65 (m, 1 H, $C^6\mathbf{H}^A$), 4.41 (m, 1 H, $C^6\mathbf{H}^B$), 2.63–2.41 (m, 8 H, $C^1\mathbf{H}^A$, $C^1\mathbf{H}^B$, $C^{1'}\mathbf{H}^A$ and $C^{1'}\mathbf{H}^B$), 2.47–2.34 (m, 8 H, C**H** protons of the Cy groups), 2.21–1.28 (m, 80 H, $C\mathbf{H}_2$ protons of the Cy groups), 2.21 (m, 2 H, $C^2\mathbf{H}_2$) ppm.

^{13}C NMR [100.62 MHz, *o*DFB, 298 K]: δ = 160.9 (1 C, $\mathbf{C}^5$), 130.9 (1 C, $\mathbf{C}^3$), 121.1 (1 C, $\mathbf{C}^4$), 91.9 (1 C, $\mathbf{C}^6$), 32.8–32.6 (8 C, **C**H carbon atoms of the Cy groups), 30.0–24.9 (40 C, $\mathbf{C}H_2$ carbon atoms of the Cy groups), 20.0 (1 C, $\mathbf{C}^2$), 15.8 and 14.8 (4 C, $\mathbf{C}^1$ and $\mathbf{C}^{1'}$) ppm.

^{19}F NMR [376.54 MHz, *o*DFB, 298 K]: δ = –75.3 (s, 36 F, $[Al(OC(C\mathbf{F}_3)_3)_4]^-$), –111.7 (ddd, 1 F, $\mathbf{C}^5$F, $^3J_{F,C6HB}$ = 50.3 Hz, $^3J_{F,C4H}$ = 24.9 Hz, $^3J_{F,C6HA}$ = 17.1 Hz), –113.9 (m, Ph**F**), –128.7 and –142.0 (m, 2 F, $C\mathbf{F}_2$-group in the starting material), –139.5 (m, *o*$C_6\mathbf{F}_2H_4$), –204.0 (t, 1 F, Ga**F**, $^2J_{F,PI}$ = 27.0 Hz) ppm.

^{27}Al NMR [78.22 MHz, *o*DFB, 298 K]: δ = 35.0 (s, 1 Al, $[\mathbf{Al}(OC(CF_3)_3)_4]^-$) ppm.

^{31}P NMR [161.99 MHz, *o*DFB, 298 K]: δ = 15.5 (m, 2 P, $\mathbf{P}^{II}$), 15.1 (unknown impurity), 13.4 (unknown impurity), 9.2 (m, 2 P, $\mathbf{P}^{I}$), –1.5 (unknown impurity) ppm.

^{71}Ga NMR [122.04 MHz, *o*DFB, 298 K]: signal probably too broad to be detected, due to the quadrupolar relaxation of ^{71}Ga.

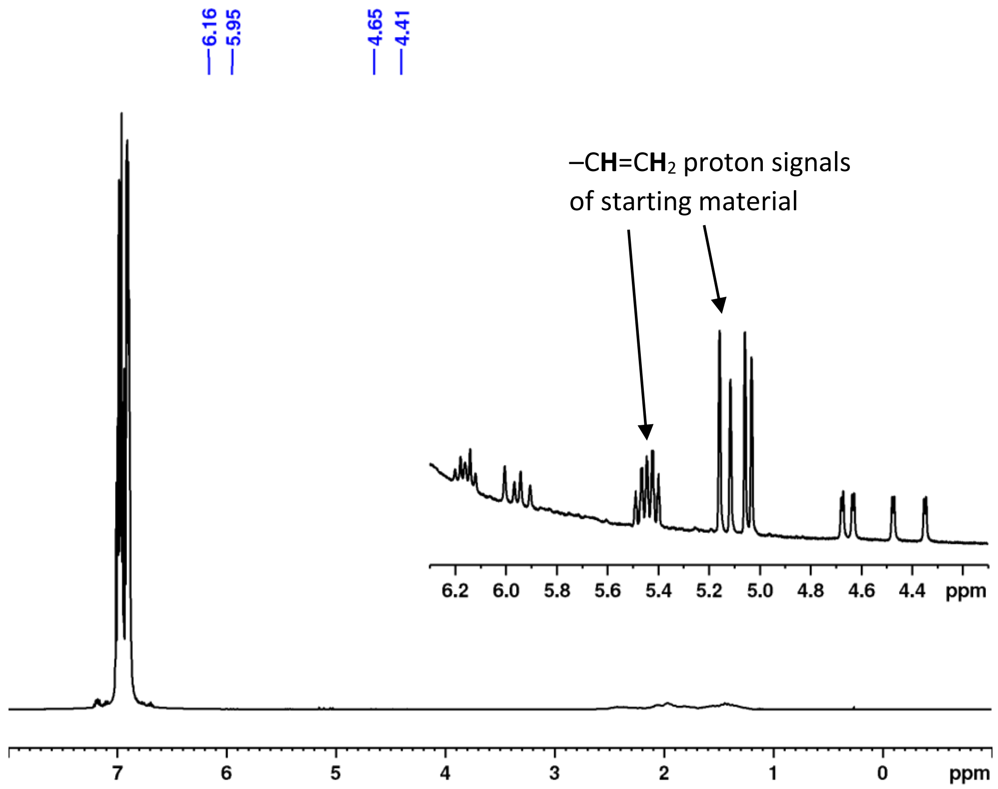

Figure S 148: ^{1}H NMR spectrum (400.17 MHz, *o*DFB, 298 K) of a mixture of [{Ga(dcpe)}$_2$][*pf*]$_2$ and 1,1-difluorovinyl-cyclopropane in *o*DFB.

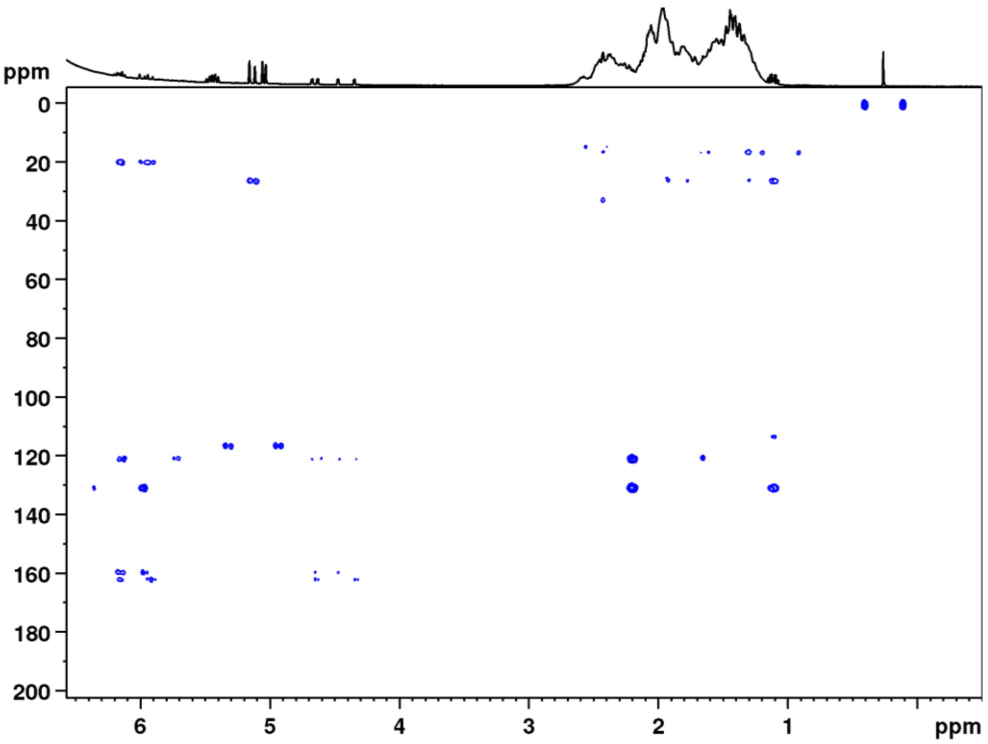

Figure S 149: ^{1}H,^{13}C-HMBC NMR spectrum (400.17 MHz, *o*DFB, 298 K, optimized for J = 8 Hz) of a mixture of [{Ga(dcpe)}$_2$][*pf*]$_2$ and 1,1-difluorovinylcyclopropane in *o*DFB.

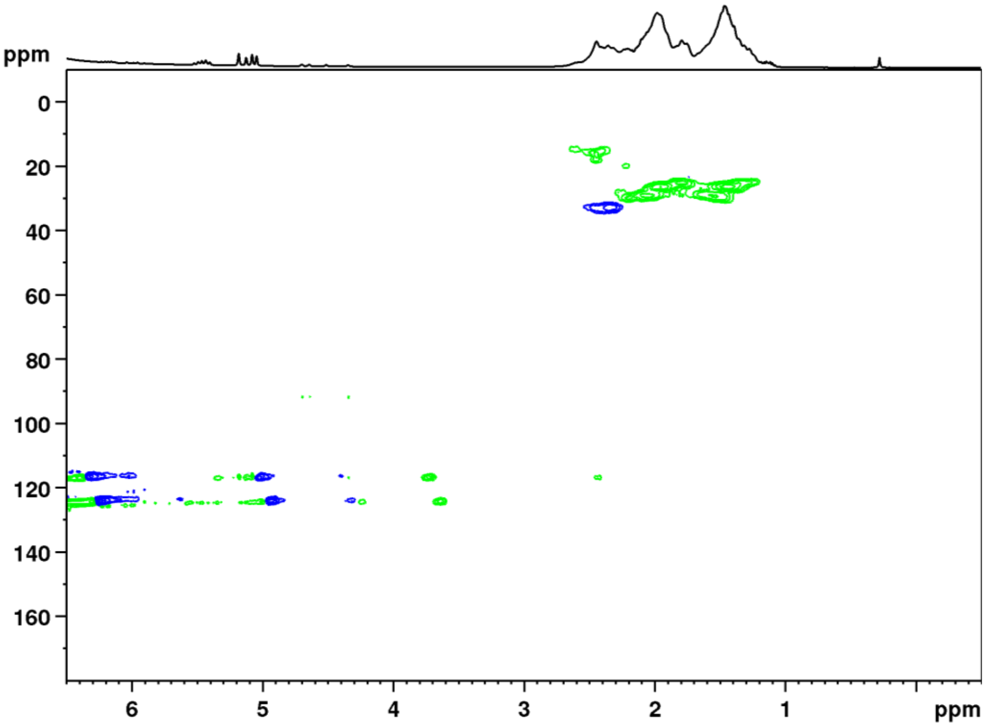

Figure S 150: Edited $^{1}H,^{13}C$-HSQC NMR spectrum (300.18 MHz, *o*DFB, 298 K, optimized for J = 145 Hz) of a mixture of [{Ga(dcpe)}$_2$][*pf*]$_2$ and 1,1-difluorovinylcyclopropane in *o*DFB.

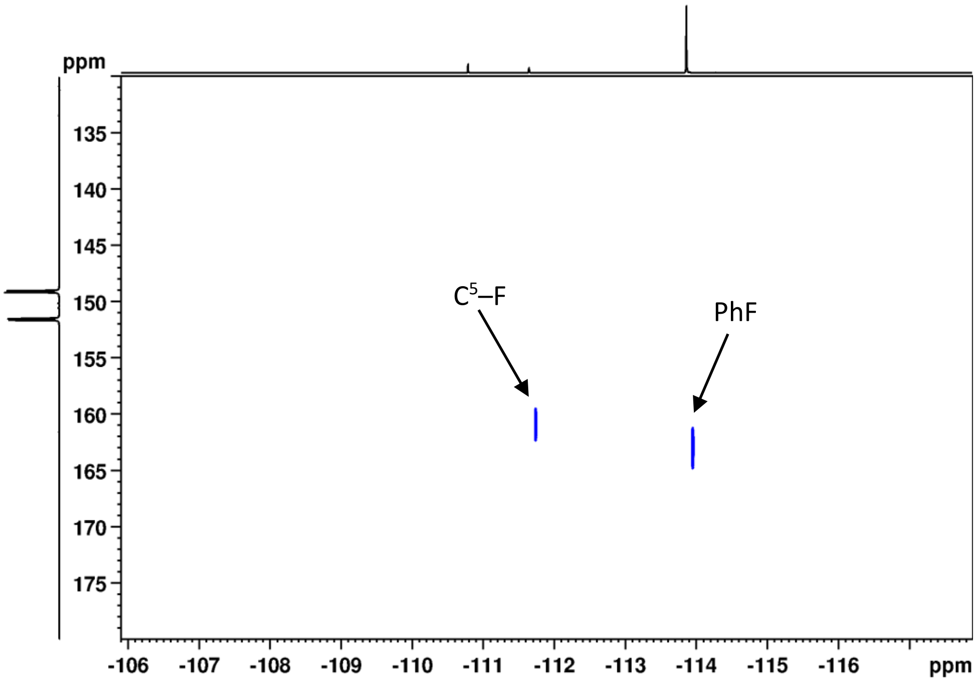

Figure S 151: $^{19}F,^{13}C$-HSQC NMR spectrum (376.54 MHz, *o*DFB, 298 K, optimized for J = 280 Hz) of a mixture of [{Ga(dcpe)}$_2$][*pf*]$_2$ and 1,1-difluorovinylcyclopropane in *o*DFB.

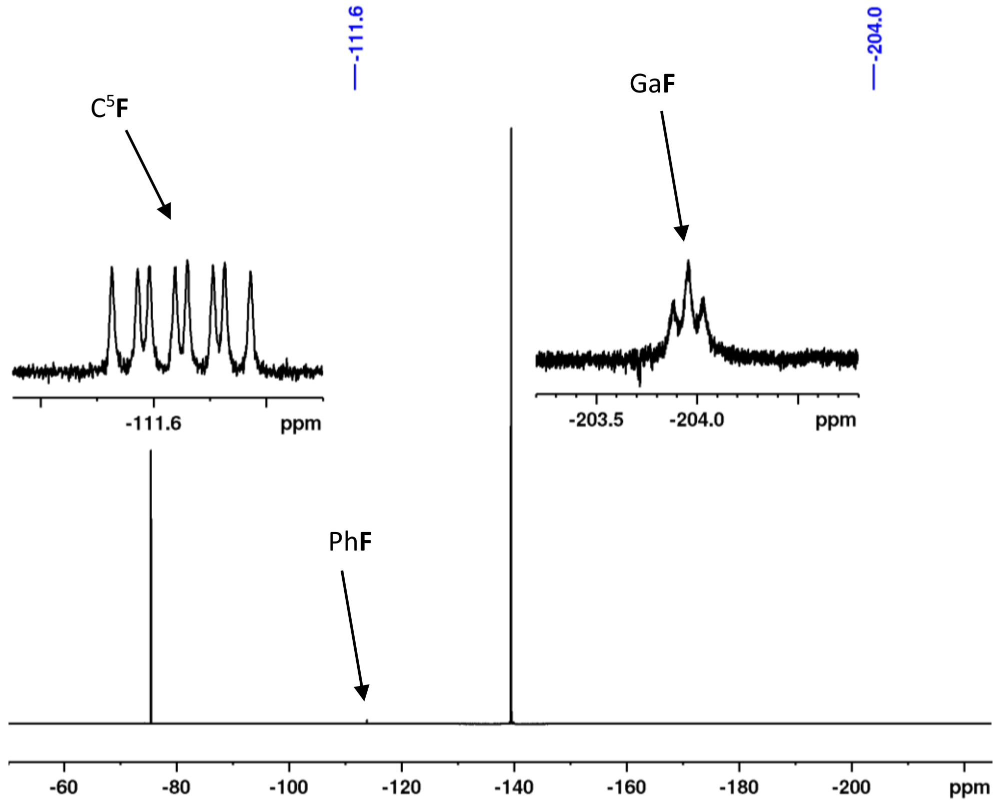

Figure S 152: ^{19}F NMR spectrum (376.54 MHz, *o*DFB, 298 K) of a mixture of [{Ga(dcpe)}$_2$][*pf*]$_2$ and 1,1-difluorovinyl-cyclopropane in *o*DFB.

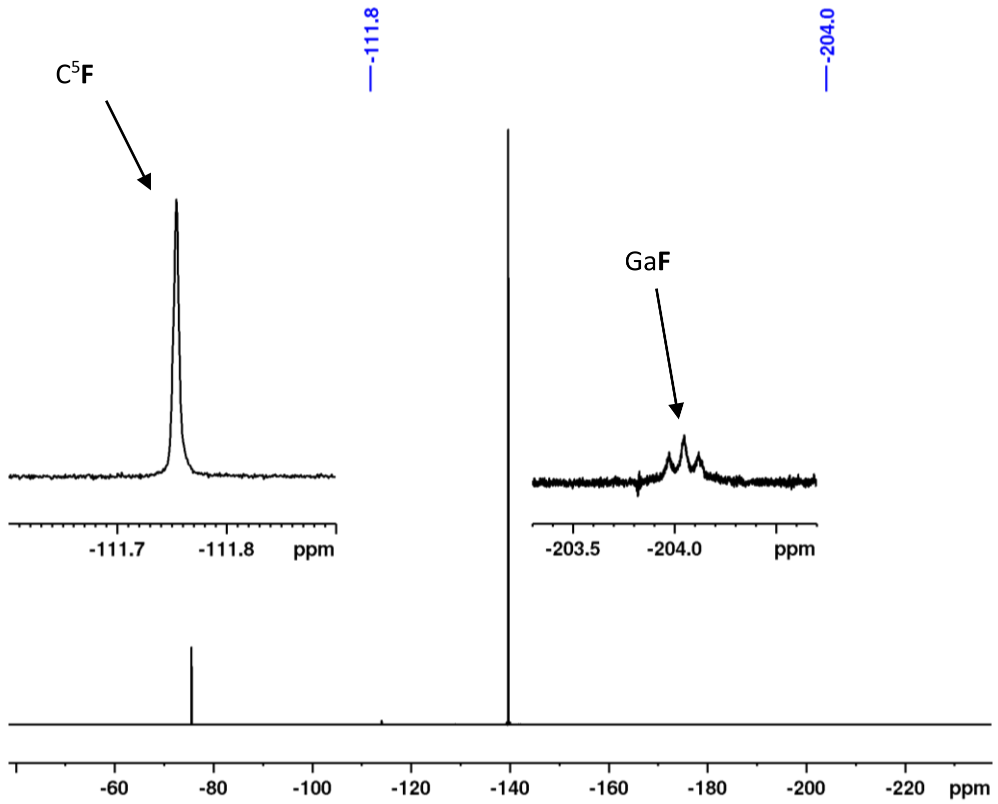

Figure S 153: $^{19}F\{^1H\}$ NMR spectrum (376.54 MHz, *o*DFB, 298 K) of a mixture of [{Ga(dcpe)}$_2$][*pf*]$_2$ and 1,1-difluorovinyl-cyclopropane in *o*DFB.

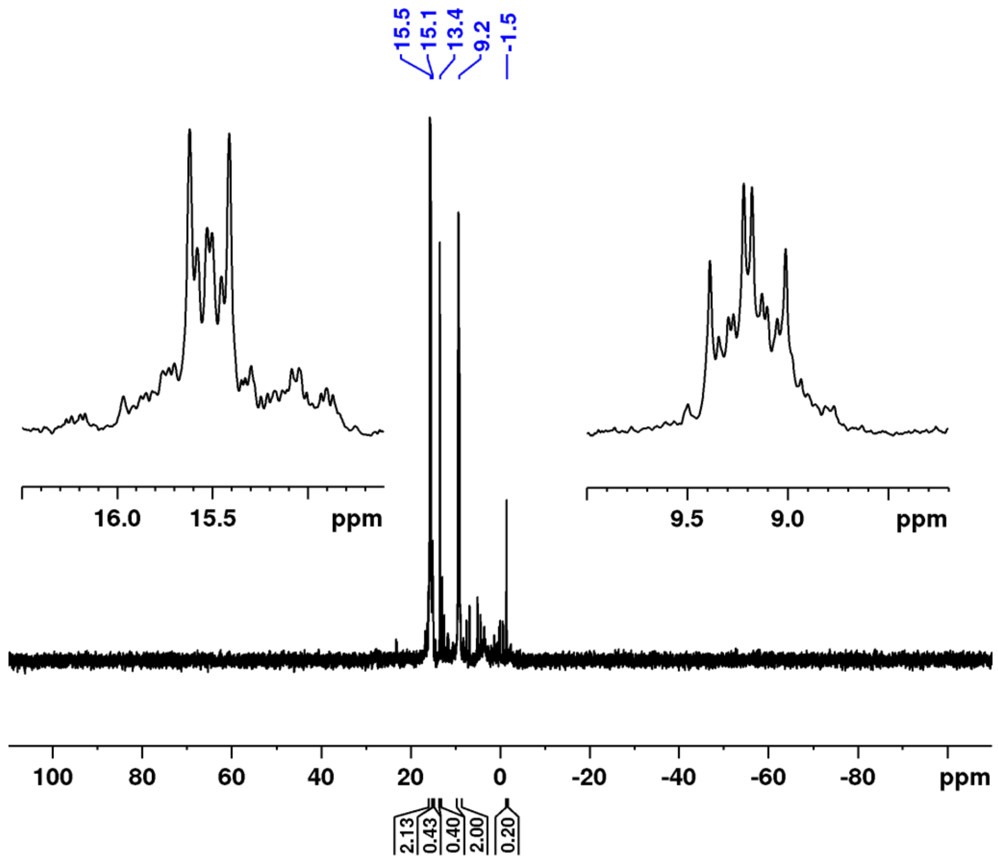

Figure S 154: $^{31}P\{^{1}H\}$ NMR spectrum (161.99 MHz, *o*DFB, 298 K) of a mixture of $[\{Ga(dcpe)\}_2][pf]_2$ and 1,1-difluorovinyl-cyclopropane in *o*DFB.

4.14 [F{Ga(dcpe)}$_2$(COOCH$_2$CHO)][*pf*]$_2$

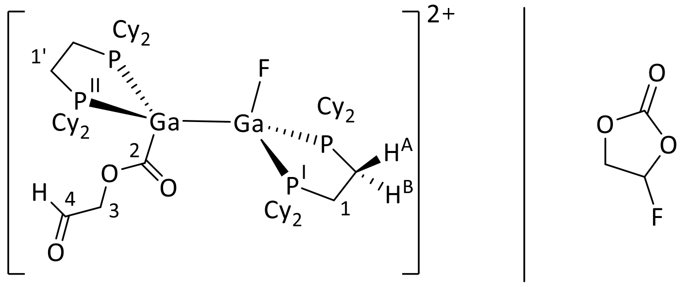

^{1}H NMR [300.18 MHz, *o*DFB, calibrated at *o*C_6F_2**H_4** = 6.96 ppm, 298 K]: δ = 9.53 (s, 1 H, C^4**H**), 6.09, 4.38 and 4.29 (m, 3 H, FEC starting material), 4.94 (s, 2 H, C^3**H**$_2$), 2.61–2.35 (m, 8 H, C**H** protons of the Cy groups), 2.25–1.32 (m, 80 H, C**H**$_2$ protons of the Cy groups), 2.57–2.54 (m, 8 H, C^1**H**^{A}, C^1**H**^{B}, C$^{1'}$**H**^{A} and C$^{1'}$**H**^{B}) ppm.

^{13}C NMR [100.62 MHz, *o*DFB, 298 K]: δ = 193.3 (1 C, **C**^{4}), 189.7 (1 C, **C**^{2}), 67.2 (1 C, **C**^{3}), 33.9–31.9 (8 C, **C**H carbon atoms of the Cy groups), 28.9–24.9 (40 C, **C**H$_2$ carbon atoms of the Cy groups), 16.1 and 14.8 (4 C, **C**^{1} and **C**$^{1'}$) ppm.

^{19}F NMR [282.45 MHz, *o*DFB, 298 K]: δ = –75.3 (s, 36 F, [Al(OC(C**F**$_3$)$_3$)$_4$]$^-$), –113.9 (m, Ph**F**), –121.8 (m, 1 F, FEC starting material), –139.5 (m, *o*C_6**F_2**H_4), –203.6 (t, 1 F, Ga**F**, $^2J_{F,PI}$ = 26.8 Hz) ppm.

^{27}Al NMR [78.22 MHz, *o*DFB, 298 K]: δ = 35.0 (s, 1 Al, [**Al**(OC(CF$_3$)$_3$)$_4$]$^-$) ppm.

^{31}P NMR [121.51 MHz, *o*DFB, 298 K]: δ = 30.6 (unknown impurity), 18.1 (m, 2 P, **P**^{II}), 9.9 (m, 2 P, **P**^{I}) ppm.

^{71}Ga NMR [91.54 MHz, *o*DFB, 298 K]: signal probably too broad to be detected, due to the quadrupolar relaxation of ^{71}Ga.

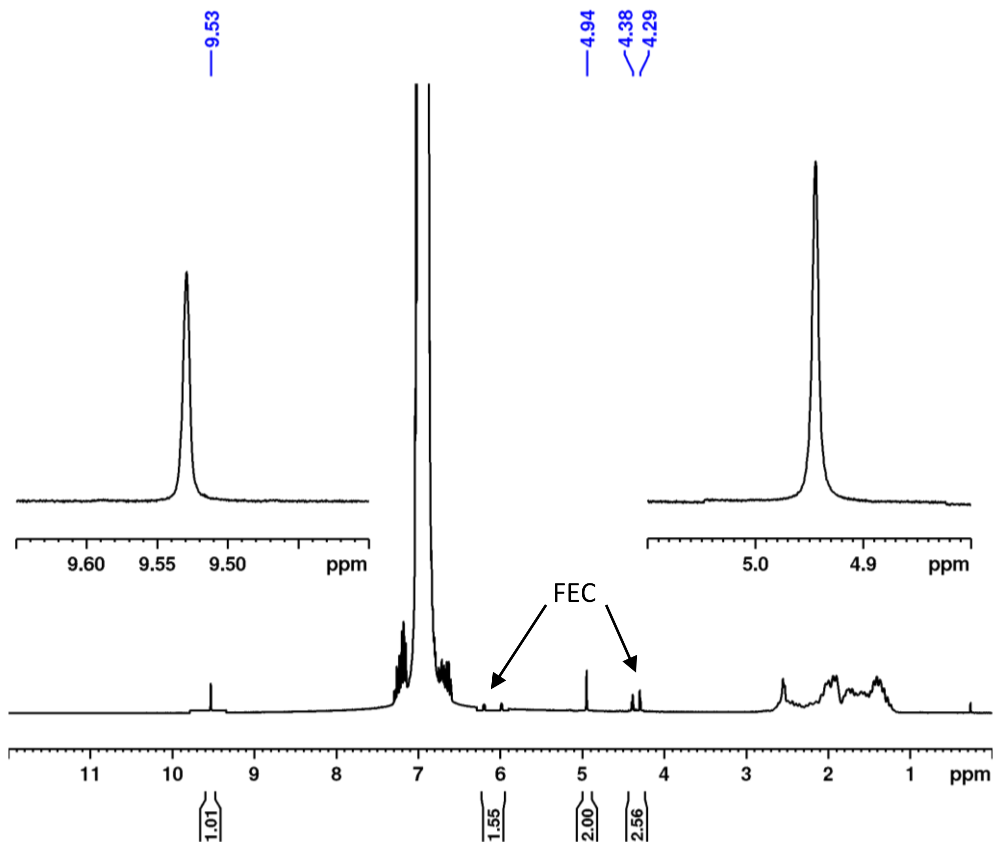

Figure S 155: ^{1}H NMR spectrum (300.18 MHz, *o*DFB, 298 K) of $[F\{Ga(dcpe)\}_2(COOCH_2CHO)][pf]_2$.

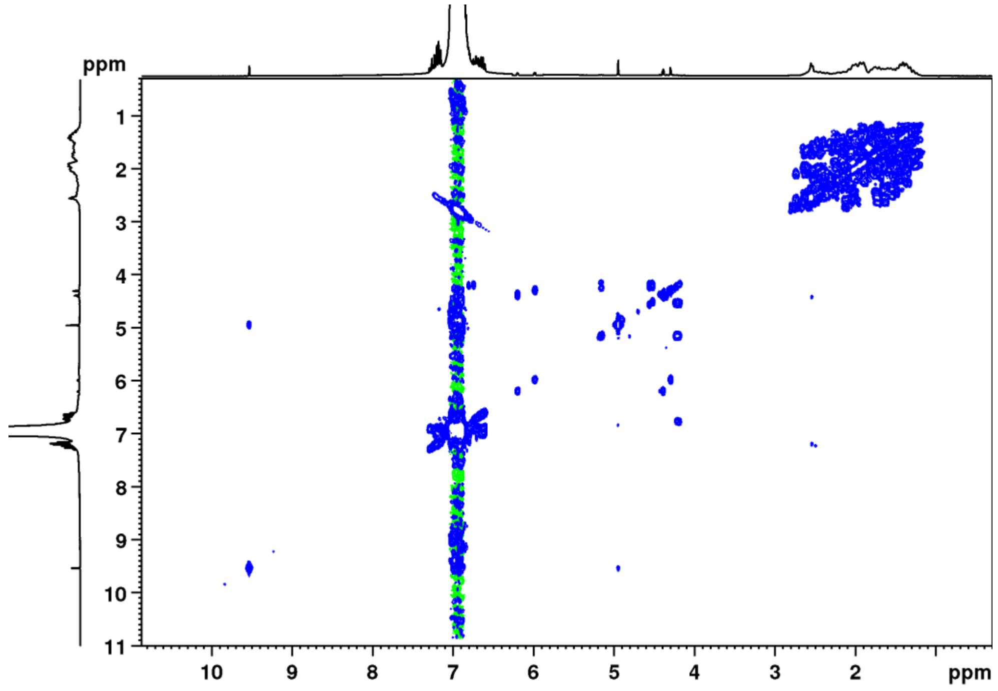

Figure S 156: ^{1}H-COSY NMR spectrum (300.18 MHz, *o*DFB, 298 K) of $[F\{Ga(dcpe)\}_2(COOCH_2CHO)][pf]_2$.

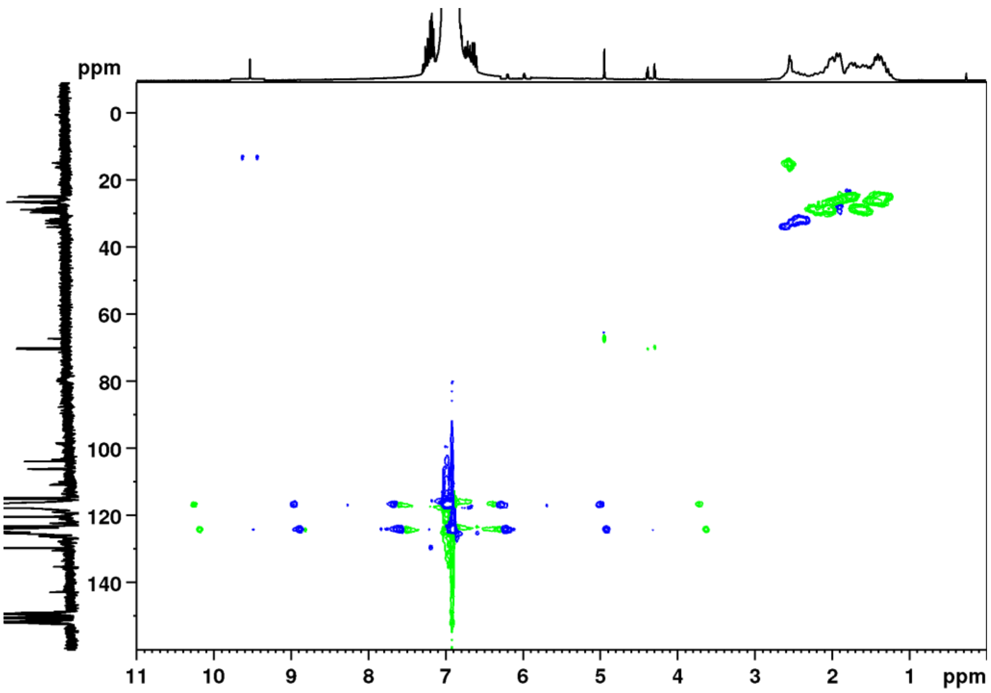

Figure S 157: Edited ^{1}H,^{13}C-HSQC NMR spectrum (300.18 MHz, *o*DFB, 298 K, optimized for *J* = 145 Hz) of [F{Ga(dcpe)}$_2$(COOCH$_2$CHO)][*pf*]$_2$.

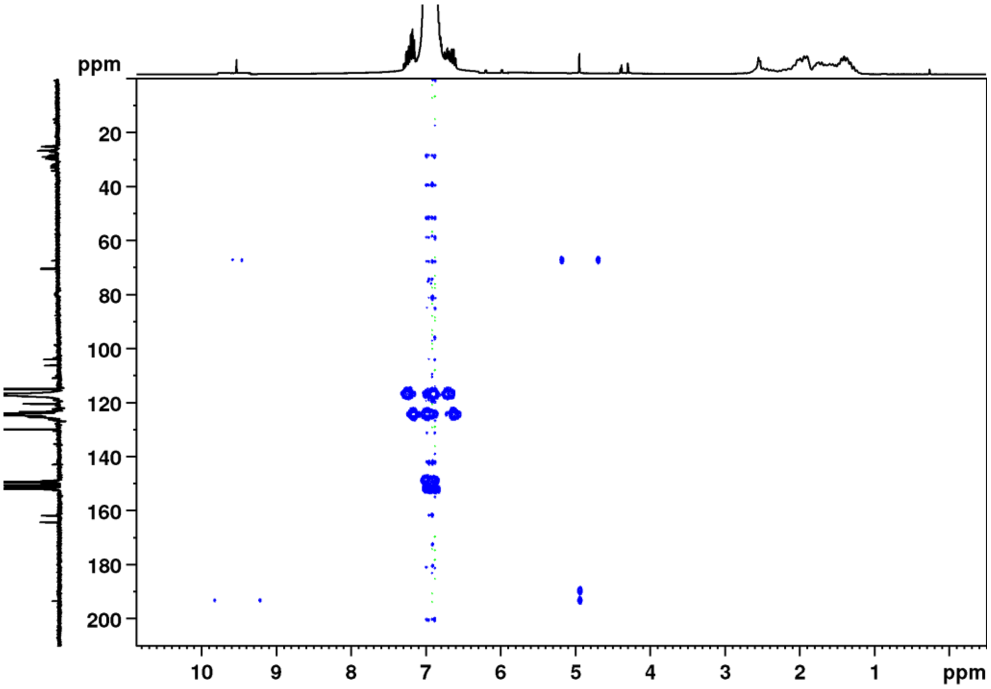

Figure S 158: ^{1}H,^{13}C-HMBC NMR spectrum (300.18 MHz, *o*DFB, 298 K, optimized for *J* = 10 Hz) of [F{Ga(dcpe)}$_2$(COOCH$_2$CHO)][*pf*]$_2$.

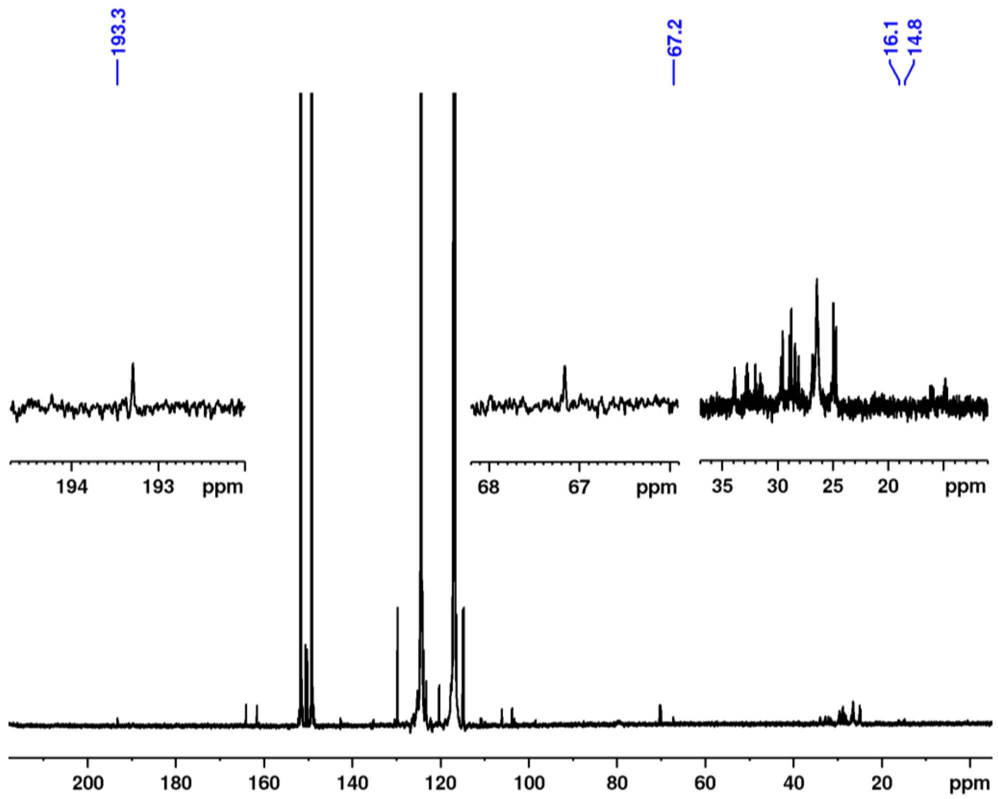

Figure S 159: $^{13}C\{^{1}H\}$ NMR spectrum (100.62 MHz, *o*DFB, 298 K) of [F{Ga(dcpe)}$_2$(COOCH$_2$CHO)][*pf*]$_2$.

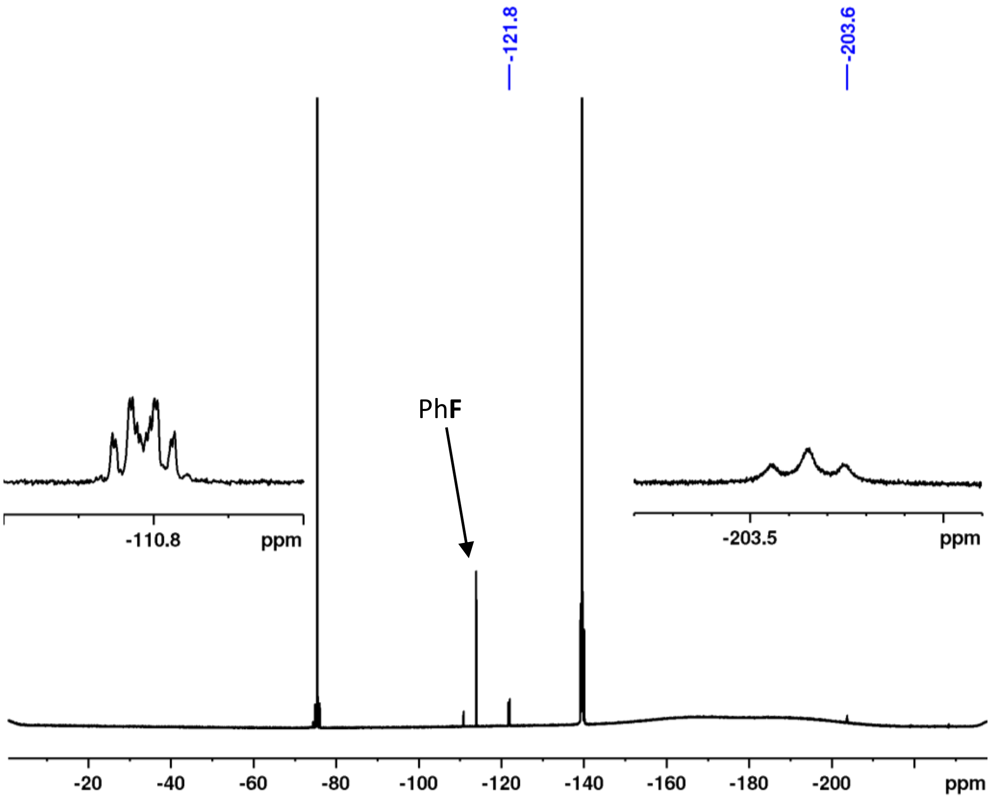

Figure S 160: ^{19}F NMR spectrum (282.45 MHz, *o*DFB, 298 K) of [F{Ga(dcpe)}$_2$(COOCH$_2$CHO)][*pf*]$_2$.

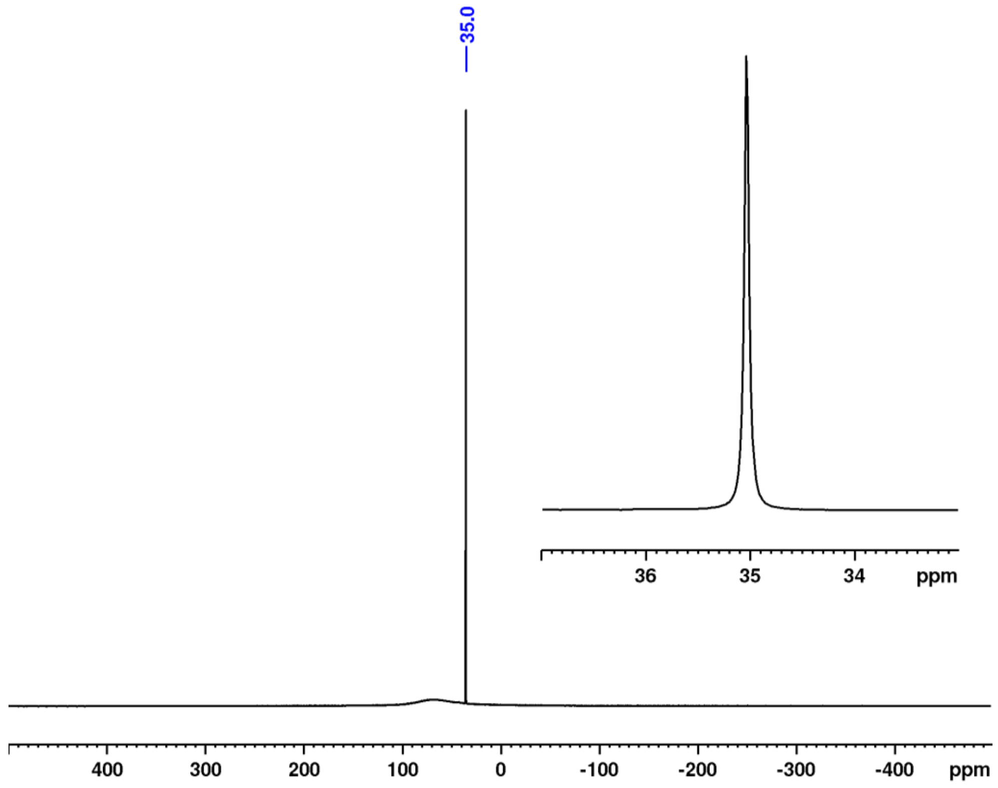

Figure S 161: ^{27}Al NMR spectrum (78.22 MHz, *o*DFB, 298 K) of [F{Ga(dcpe)}$_2$(COOCH$_2$CHO)][*pf*]$_2$.

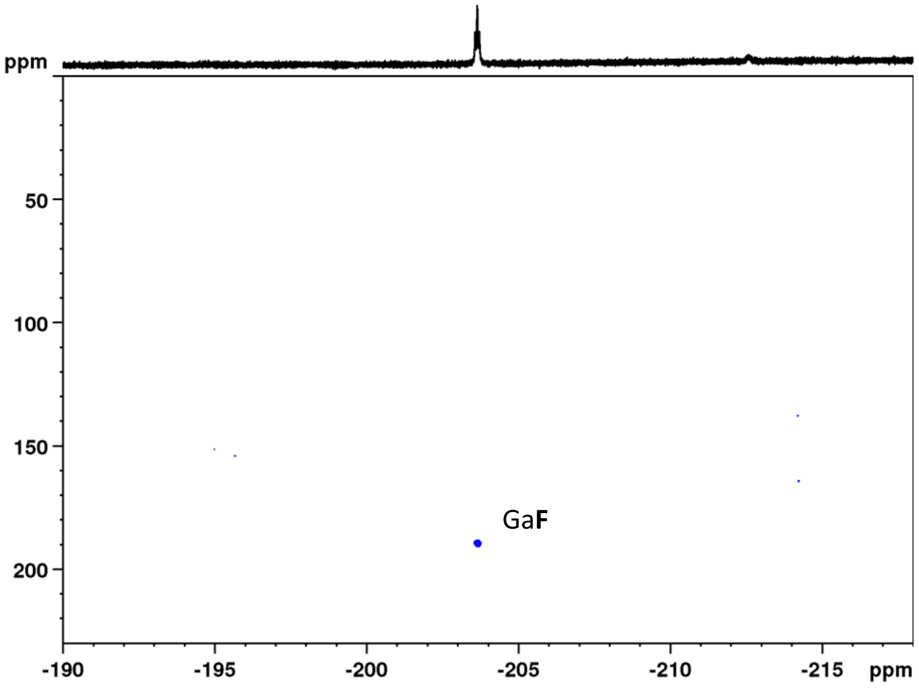

Figure S 162: ^{19}F,^{13}C-HMBC NMR spectrum (376.54 MHz, *o*DFB, 298 K, optimized for J = 20 Hz) of [F{Ga(dcpe)}$_2$(COOCH$_2$CHO)][*pf*]$_2$.

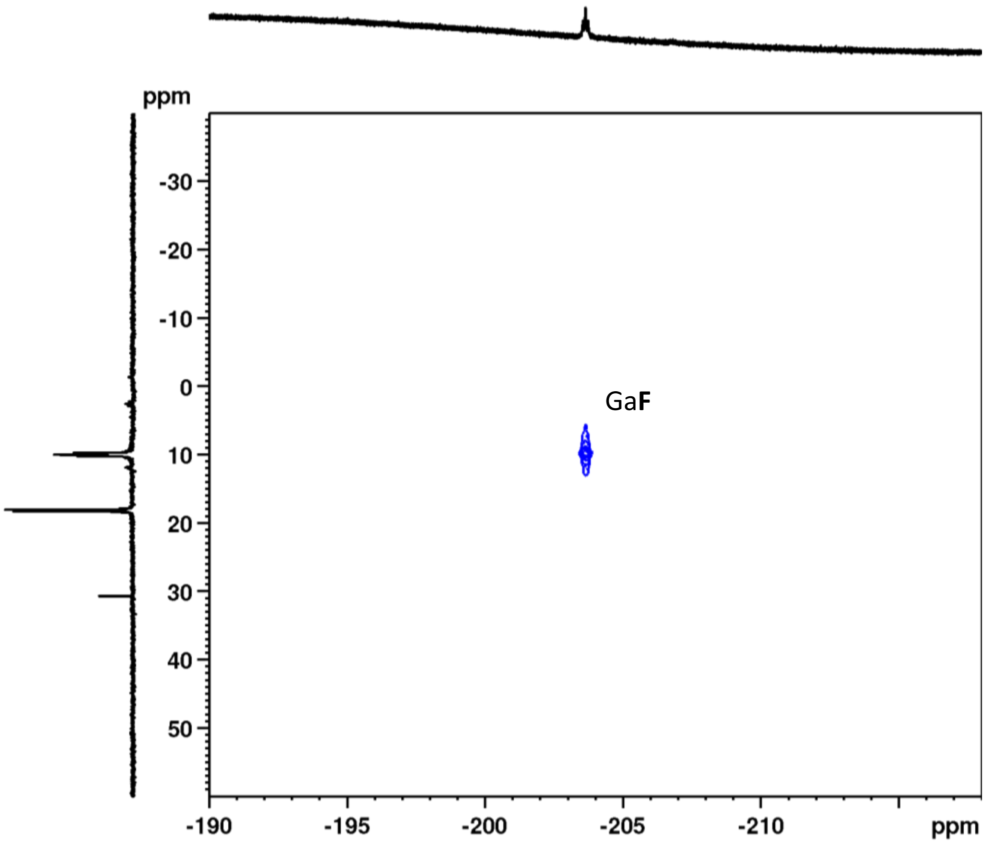

Figure S 163: ^{19}F,^{31}P-HMBC NMR spectrum (282.45 MHz, *o*DFB, 298 K, optimized for $J = 30$ Hz) of $[F\{Ga(dcpe)\}_2(COOCH_2CHO)][pf]_2$.

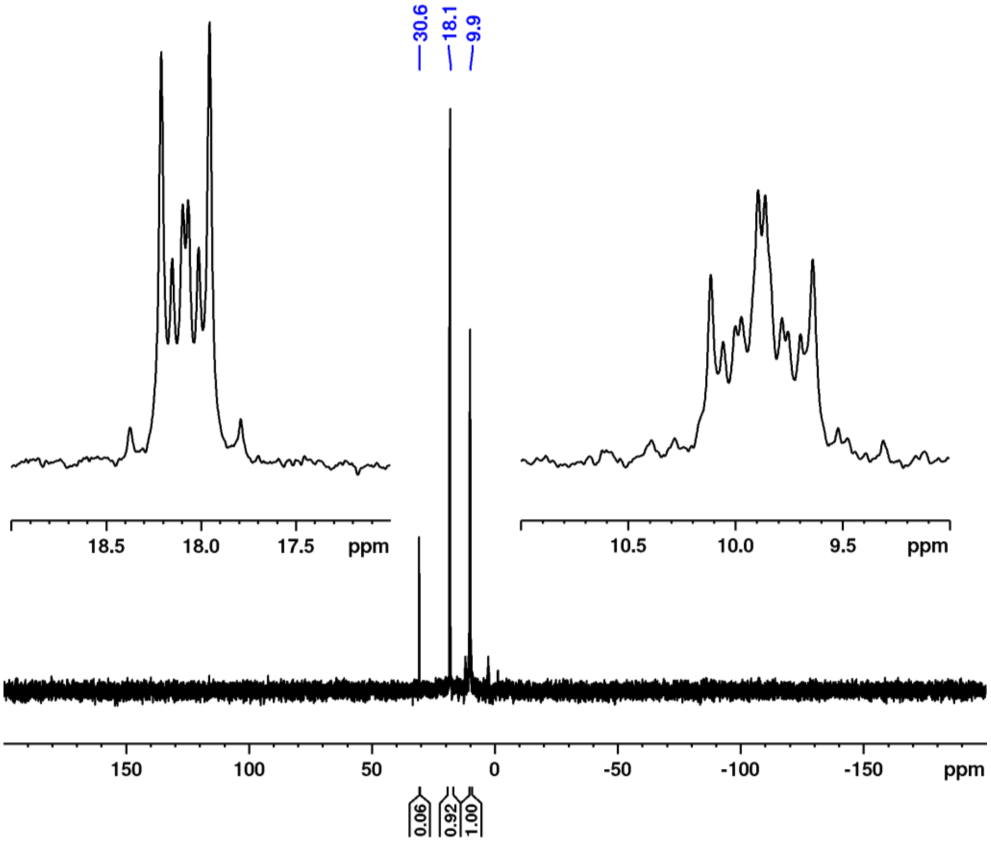

Figure S 164: $^{31}P\{^1H\}$ NMR spectrum (121.51 MHz, *o*DFB, 298 K) of $[F\{Ga(dcpe)\}_2(COOCH_2CHO)][pf]_2$.

4.15 [F{Ga(dcpe)}$_2$(C$_6$F$_2$H$_3$)][*pf*]$_{5/3}$[*alfal*]$_{1/3}$

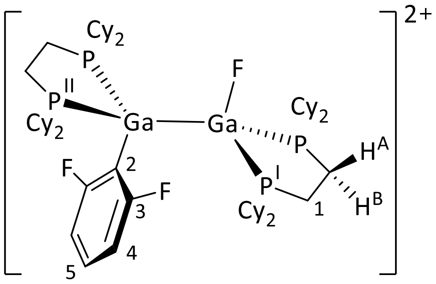

^{1}H NMR [400.17 MHz, 3FB, calibrated at 1,2,3-C_6F_3**H_3** = 6.86 ppm, 298 K]: δ = 7.66 (m, 1 H, C^5**H**), 7.27 (m, 2 H, C^4**H**), 2.74–2.60 (m, 8 H, C^1**H**^{A}, C^1**H**^{B}, C$^{1'}$**H**^{A} and C$^{1'}$**H**^{B}), 2.61–2.49 (m, 8 H, C**H** protons of the Cy groups), 2.40–1.22 (m, 80 H, C**H_2** protons of the Cy groups) ppm.

^{13}C NMR [100.62 MHz, 3FB, 298 K]: δ = 166.4 (2 C, **C**^{3}), 134.4 (1 C, **C**^{5}), 114.0 (1 C, **C**^{2}), 111.9 (2 C, **C**^{4}), 34.9–32.6 (8 C, **C**H carbon atoms of the Cy groups), 29.4–24.9 (40 C, **C**H_2 carbon atoms of the Cy groups), 15.3 and 14.5 (4 C, **C**^{1} and **C**$^{1'}$) ppm.

^{19}F NMR [282.45 MHz, 3FB, 298 K]: δ = –75.7 (s, 36 F, [Al(OC(C**F**$_3$)$_3$)$_4$]$^-$), –75.9 (s, 54 F, [{(**F**$_3$C)$_3$C}Al–F–Al{OC(C**F**$_3$)$_3$}$_3$]$^-$), –88.2 (m, 2 F, C^3**F**), –112.9 (m, Ph**F**), –136.8 and –163.5 (m, 3FB solvent), –184.6 (s, 72 F, [{(F$_3$C)$_3$C}Al–**F**–Al{OC(CF$_3$)$_3$}$_3$]$^-$), –194.8 (t, 1 F, **F**–Ga, $^2J_{F,PI}$ = 27.3 Hz) ppm.

^{27}Al NMR [78.22 MHz, 3FB, 298 K]: δ = 34.8 (s, 1 Al, [**Al**(OC(CF$_3$)$_3$)$_4$]$^-$) ppm.

^{31}P NMR [121.52 MHz, 3FB, 298 K]: δ = 15.8 (m, 2 P, **P**^{II}), 12.0 (unknown impurity), 8.9 (m, 2 P, **P**^{I}) ppm.

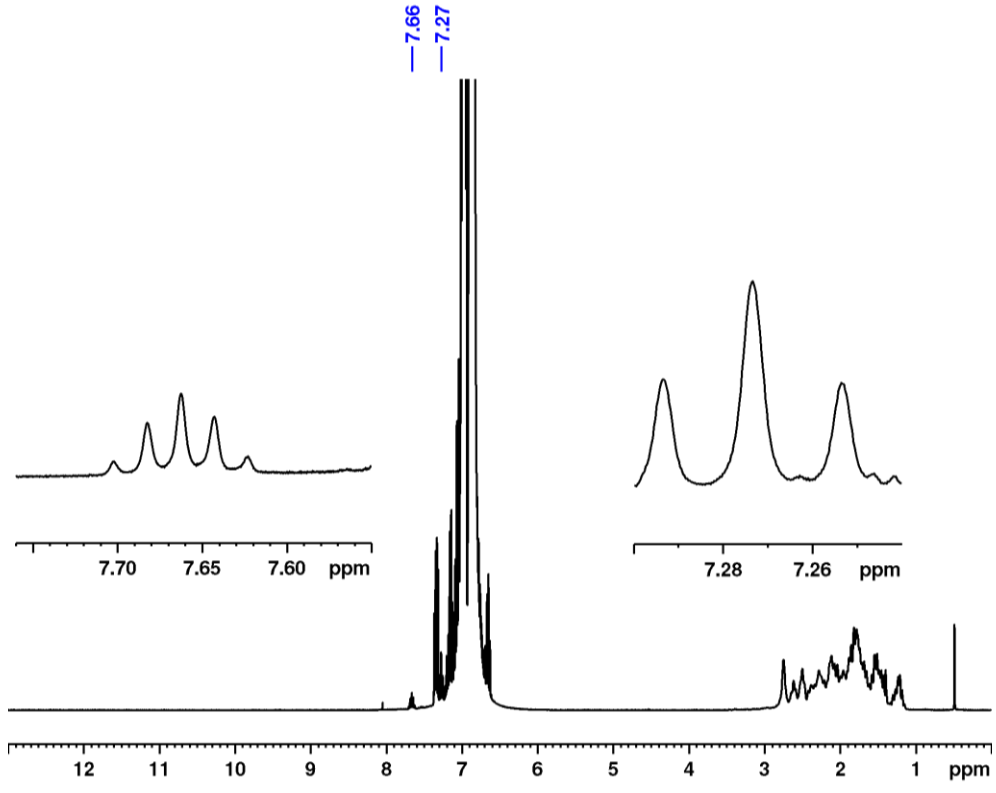

Figure S 165: ^{1}H NMR spectrum (400.17 MHz, 3FB, 298 K) of [{Ga(dcpe)}$_2$][*pf*]$_2$ in 3FB.

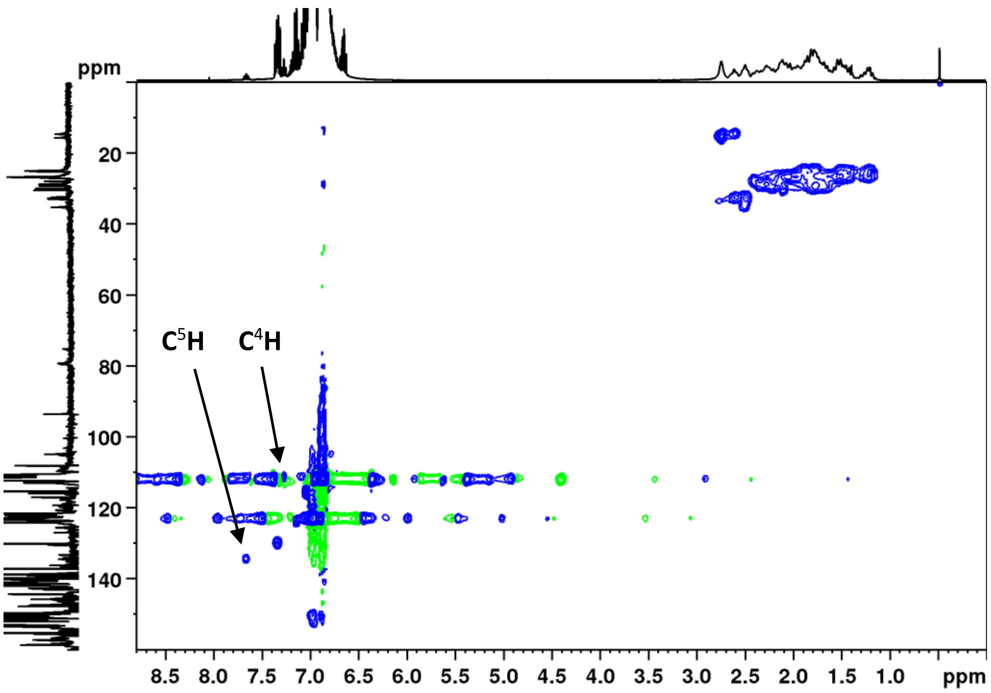

Figure S 166: ^{1}H,^{13}C-HSQC NMR spectrum (400.17 MHz, 3FB, 298 K, optimized for J = 145 Hz) of [{Ga(dcpe)}$_2$][*pf*]$_2$ in 3FB.

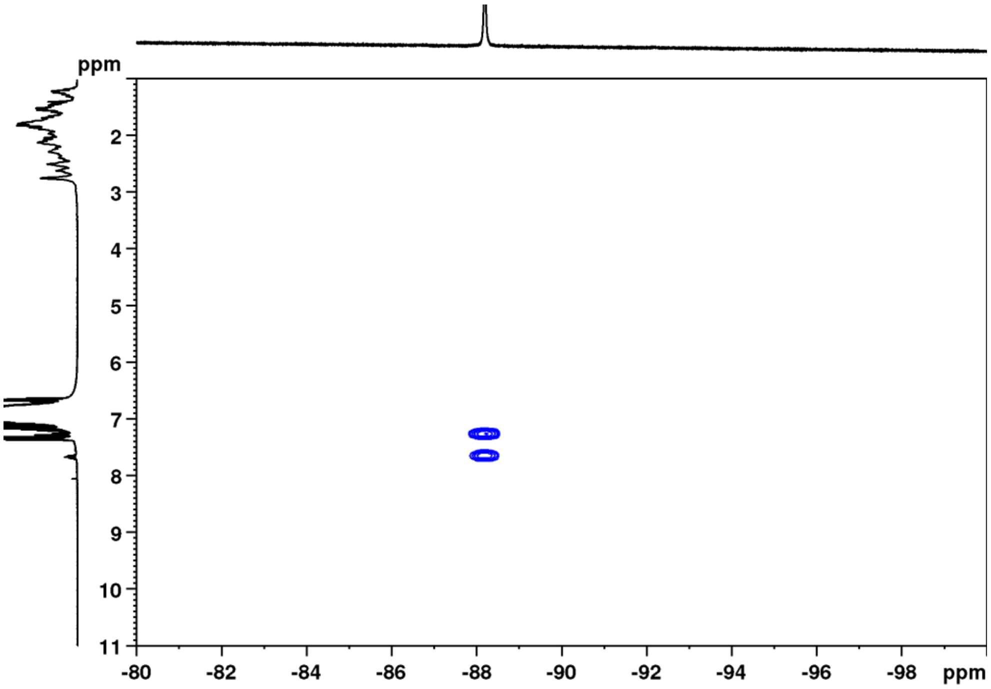

Figure S 167: ^{1}H,^{19}F-COSY NMR spectrum (376.54 MHz, 3FB, 298 K) of [{Ga(dcpe)}$_2$][*pf*]$_2$ in 3FB.

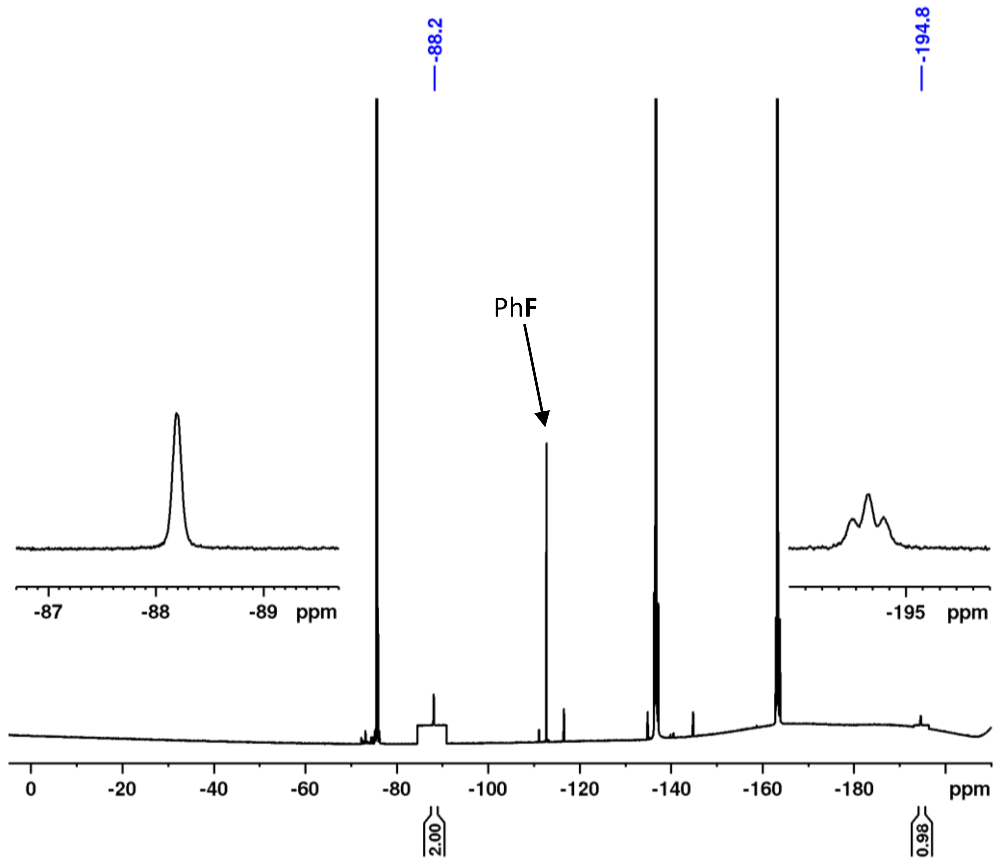

Figure S 168: ^{19}F NMR spectrum (282.45 MHz, 3FB, 298 K) of [{Ga(dcpe)}$_2$][*pf*]$_2$ in 3FB.

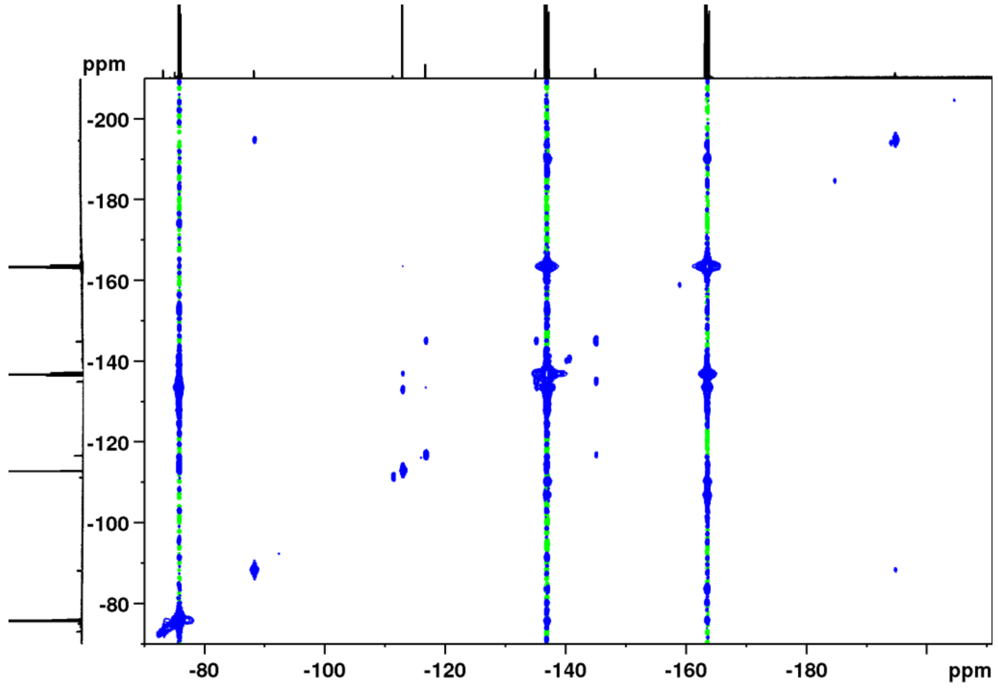

Figure S 169: ^{19}F-COSY NMR spectrum (376.54 MHz, 3FB, 298 K) of [{Ga(dcpe)}$_2$][*pf*]$_2$ in 3FB.

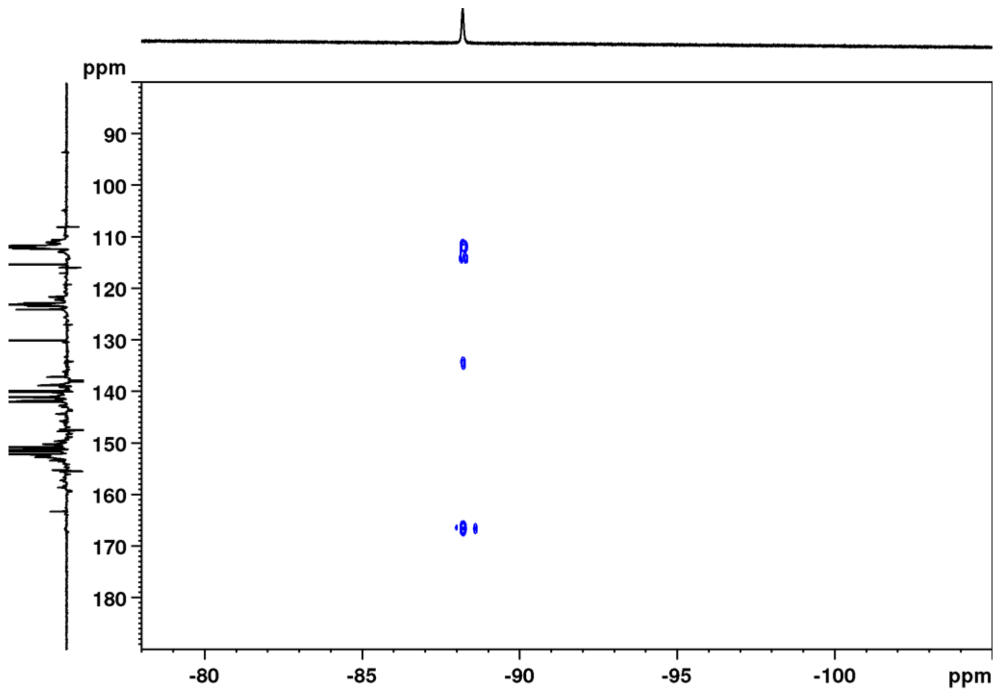

Figure S 170: $^{19}F,^{13}C$-HMBC NMR spectrum (376.54 MHz, 3FB, 298 K, optimized for J = 30 Hz) of [{Ga(dcpe)}$_2$][*pf*]$_2$ in 3FB.

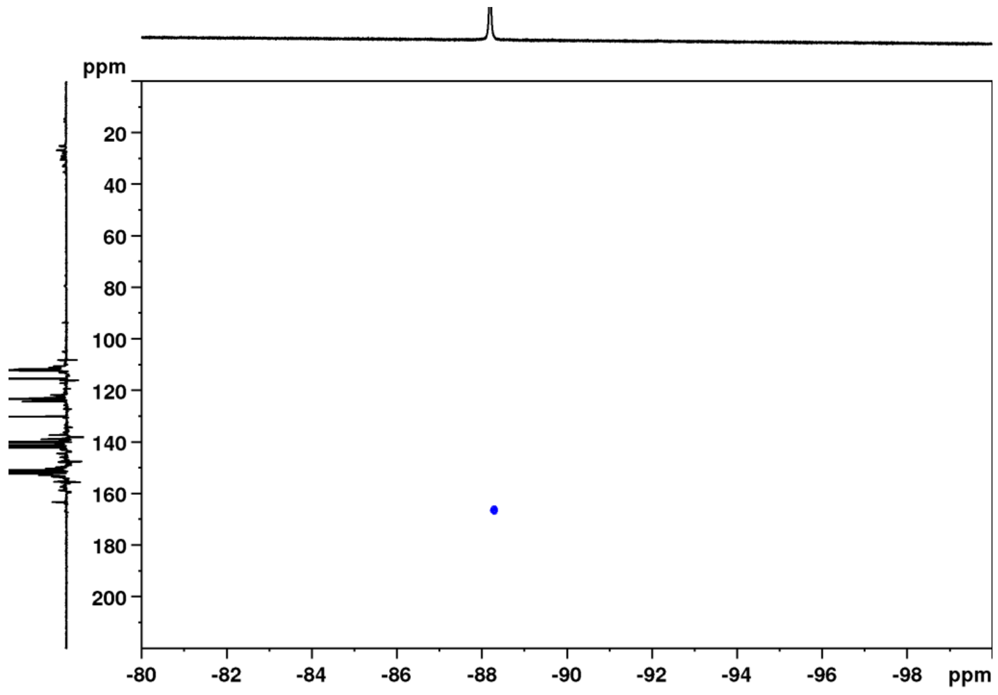

Figure S 171: $^{19}F,^{13}C$-HSQC NMR spectrum (376.54 MHz, 3FB, 298 K, optimized for J = 250 Hz) of [{Ga(dcpe)}$_2$][*pf*]$_2$ in 3FB.

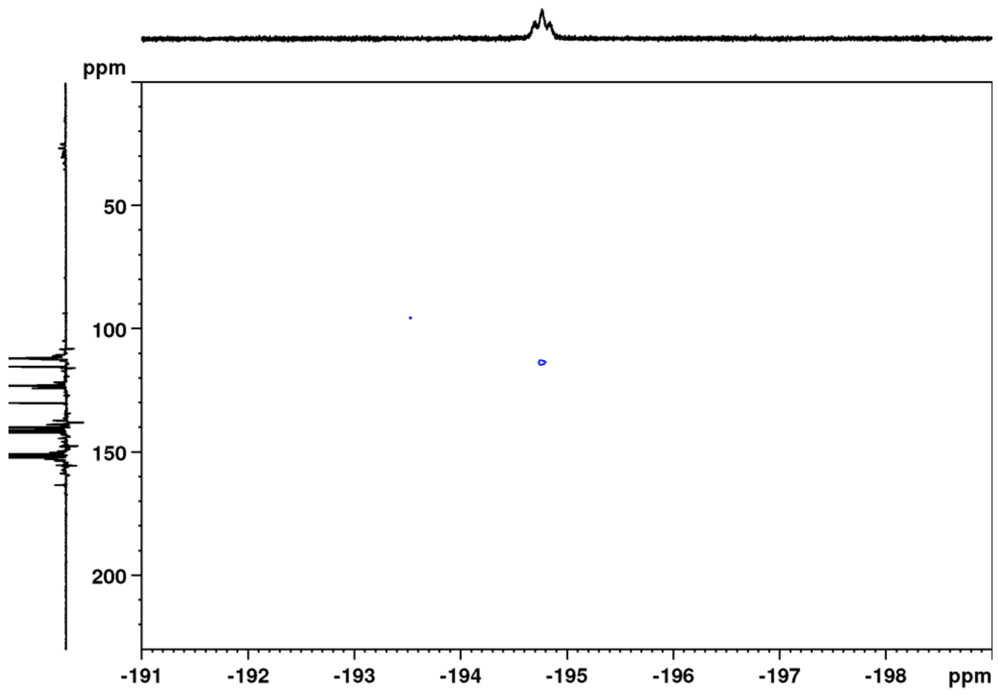

Figure S 172: ^{19}F,^{13}C-HMBC NMR spectrum (376.54 MHz, 3FB, 298 K, optimized for J = 20 Hz) of [{Ga(dcpe)}$_2$][*pf*]$_2$ in 3FB.

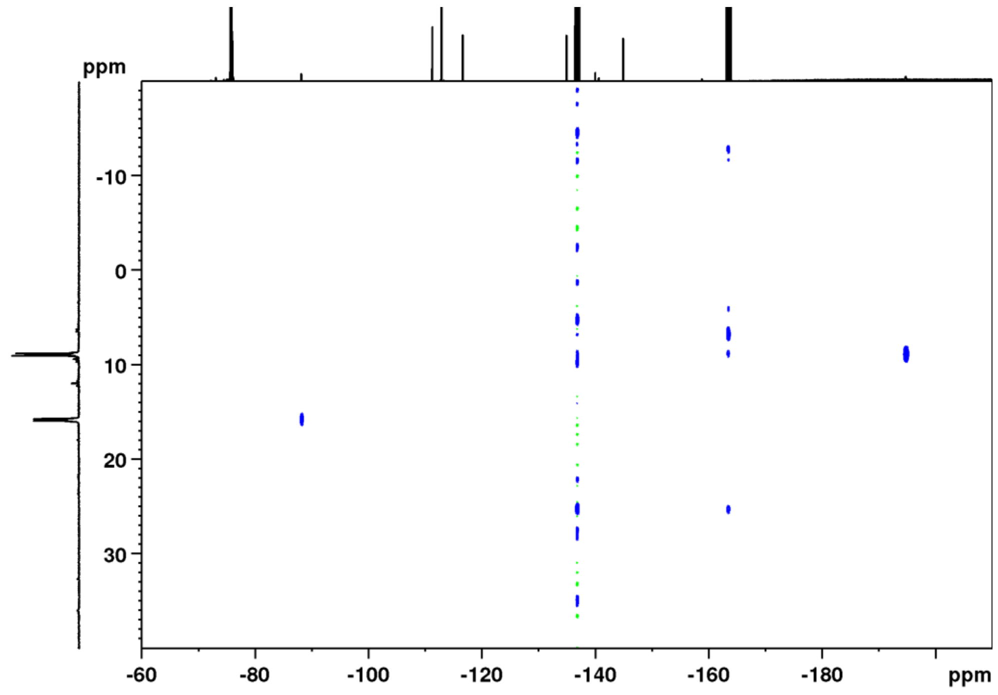

Figure S 173: ^{19}F,^{31}P-COSY NMR spectrum (376.54 MHz, 3FB, 298 K) of [{Ga(dcpe)}$_2$][*pf*]$_2$ in 3FB.

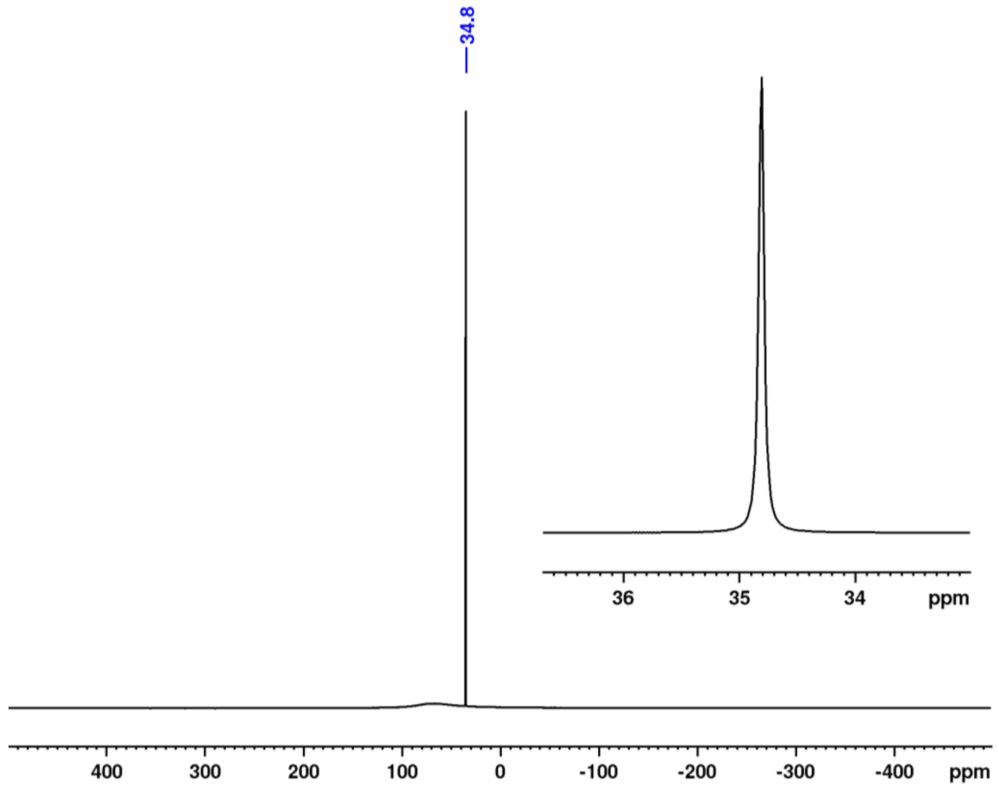

Figure S 174: ^{27}Al NMR spectrum (78.22 MHz, 3FB, 298 K) of [{Ga(dcpe)}$_2$][*pf*]$_2$ in 3FB.

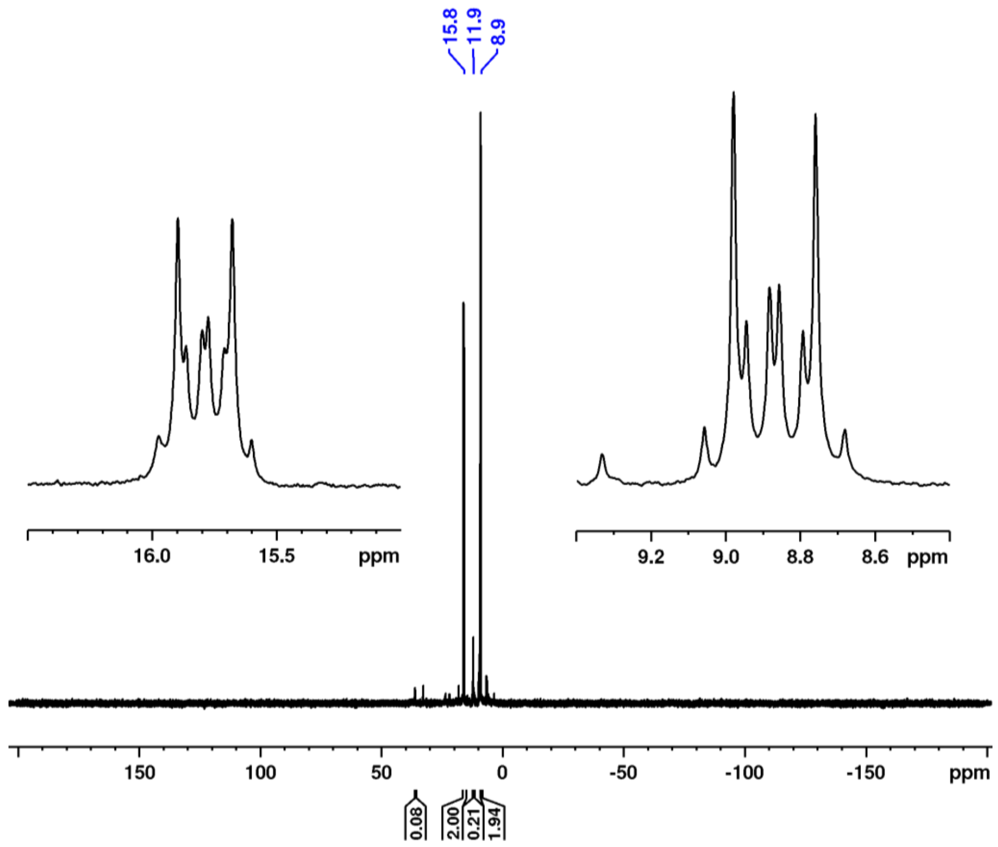

Figure S 175: ^{31}P{^{1}H} NMR spectrum (121.52 MHz, 3FB, 298 K) of [{Ga(dcpe)}$_2$][*pf*]$_2$ in 3FB.

4.16 Unselective Bond Activation Reactions

4.16.1 Reaction Between $[\{Ga(dcpe)\}_2]^{2+}$ and $PhCF_3$

The NMR signals at ca. −200 ppm in the ^{19}F NMR spectrum (**Figure S 176**) of a mixture of $\mathbf{1}^{2+}$ and $PhCF_3$ probably indicate the presence of species with a P–Ga–**F** moiety and thus suggest that $\mathbf{1}^{2+}$ does cleave C–F bonds in this substrate, however in a somewhat unselective reaction and under the formation of a multitude of different reaction products as shown by the ^{19}F and $^{31}P\{^1H\}$ NMR spectra.

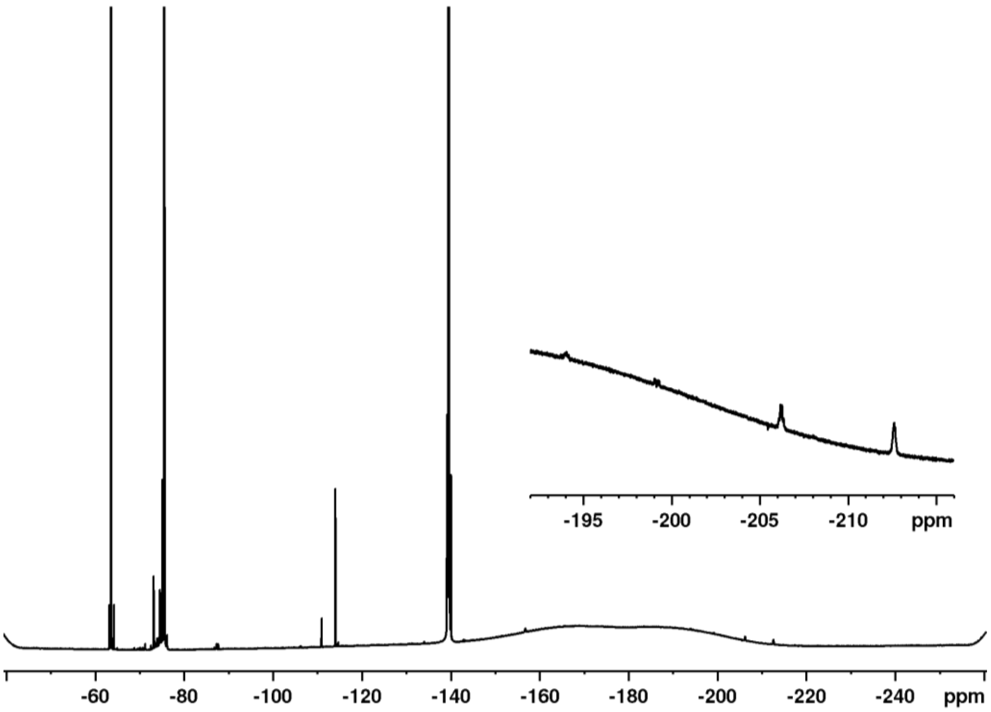

Figure S 176: ^{19}F NMR spectrum (282.45 MHz, *o*DFB, 298 K) of a mixture of $[\{Ga(dcpe)\}_2][pf]_2$ and a excess $PhCF_3$ in *o*DFB.

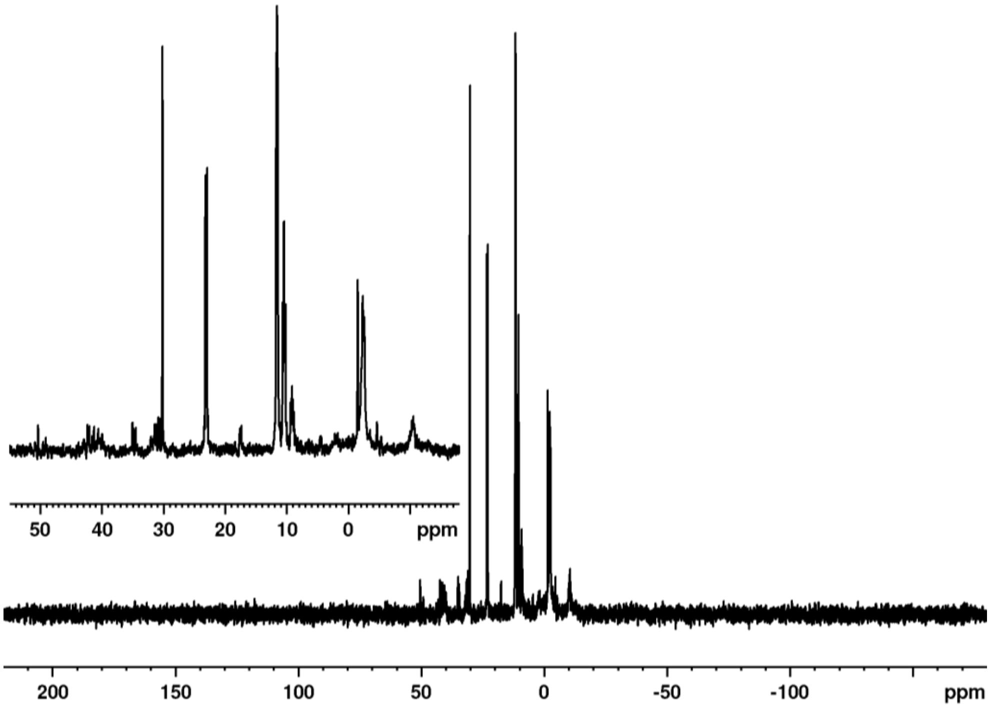

Figure S 177: $^{31}P\{^1H\}$ NMR spectrum (121.52 MHz, *o*DFB, 298 K) of a mixture of $[\{Ga(dcpe)\}_2][pf]_2$ and a excess $PhCF_3$ in *o*DFB.

4.16.2 Reaction Between [{Ga(dcpe)}$_2$]$^{2+}$ and 4FB

When heating a solution of [{Ga(dcpe)}$_2$][*pf*]$_2$ in 4FB for 3 days at 40 °C, the ^{19}F and ^{31}P NMR spectra indicate the formation of at least four different species with FGaP$_2$ moieties, which is confirmed by the ^{19}F,^{31}P-COSY NMR spectrum (**Figure S 178**–**Figure S 180**). If **1**$^{2+}$ merely activated the C–F bonds in 4FB, only two isomeric products would have been expected. Thus, the results suggest that the products undergo consecutive reactions.

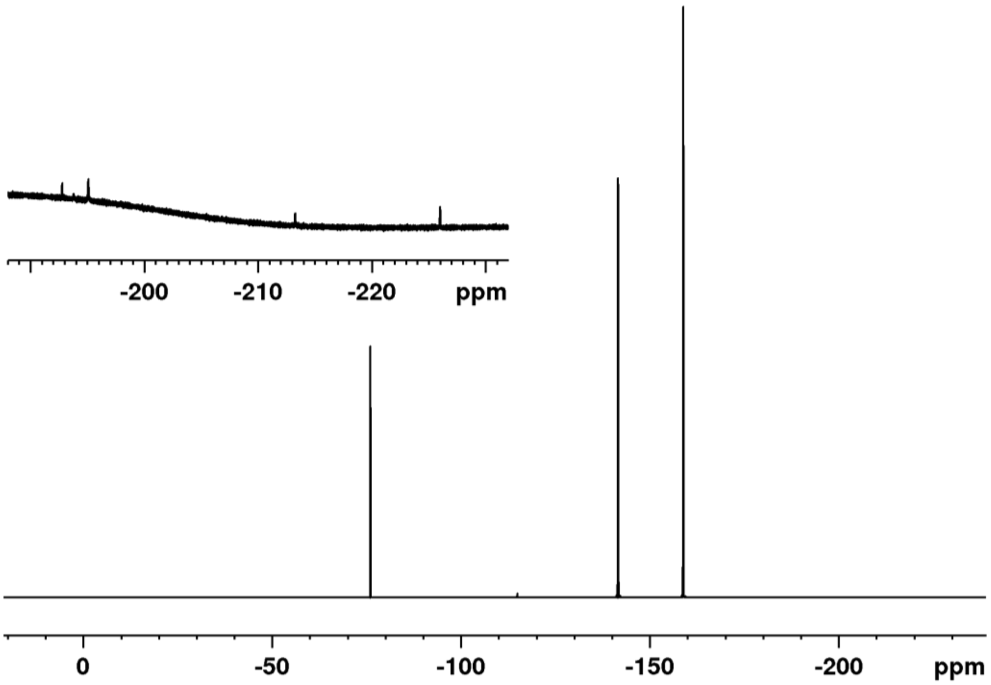

Figure S 178: ^{19}F{^{31}P} NMR spectrum (282.45 MHz, 4FB, 298 K) of [{Ga(dcpe)}$_2$][*pf*]$_2$ in 4FB, after 3 d at 40 °C.

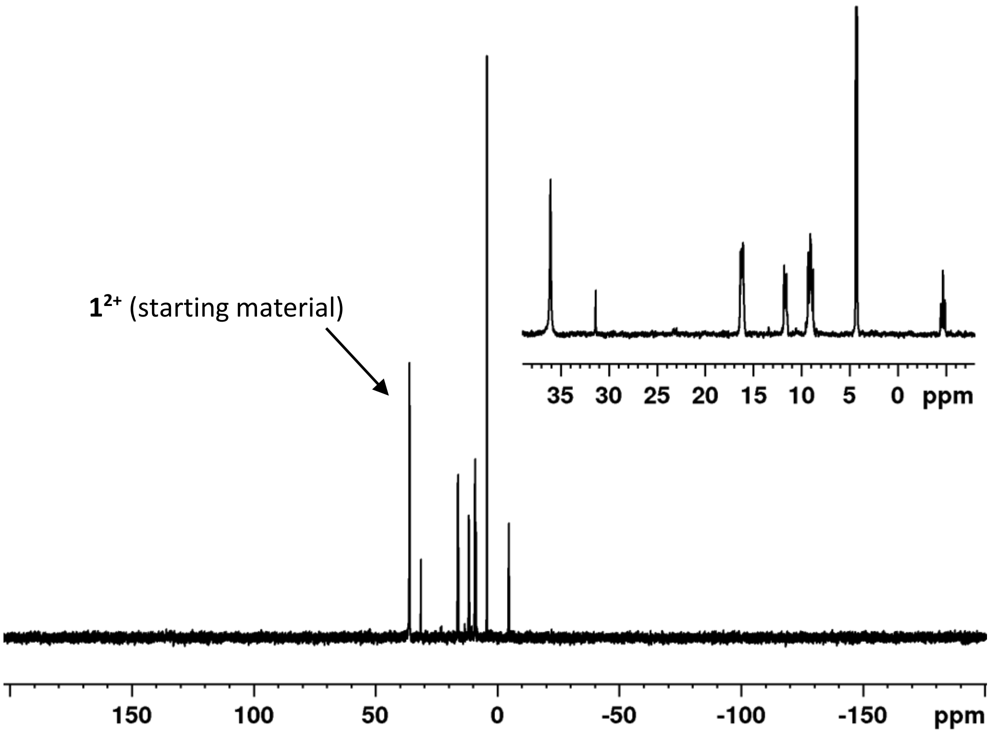

Figure S 179: ^{31}P{^{1}H} NMR spectrum (121.51 MHz, 4FB, 298 K) of [{Ga(dcpe)}$_2$][*pf*]$_2$ in 4FB, after 3 d at 40 °C.

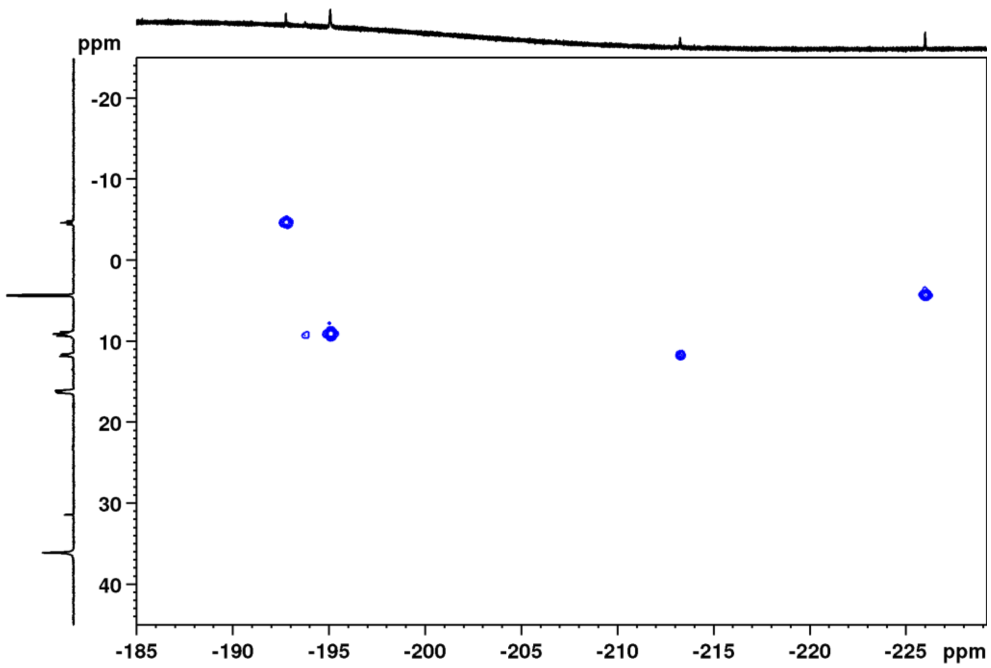

Figure S 180: ^{19}F,^{31}P-COSY NMR spectrum (282.45 MHz, 4FB, 298 K) of [{Ga(dcpe)}$_2$][*pf*]$_2$ in 4FB, after 3 d at 40 °C.

5 Vibrational Spectroscopy

5.1 [{Ga(dcpe)}$_2$(C$_6$H$_{10}$)][*pf*]$_2$

FT-IR: $\tilde{\nu}$ [cm^{-1}] = 2934 (vw), 2859 (vw), 1637 (vw), 1508 (vw), 1451 (vw), 1414 (vw), 1351 (vw), 1297 (w), 1274 (m), 1238 (s), 1213 (vs), 1168 (m), 1049 (vw), 971 (vs), 890 (vw), 852 (vw), 830 (vw), 755 (vw), 726 (vs), 680 (vw), 644 (vw), 571 (vw), 559 (vw).

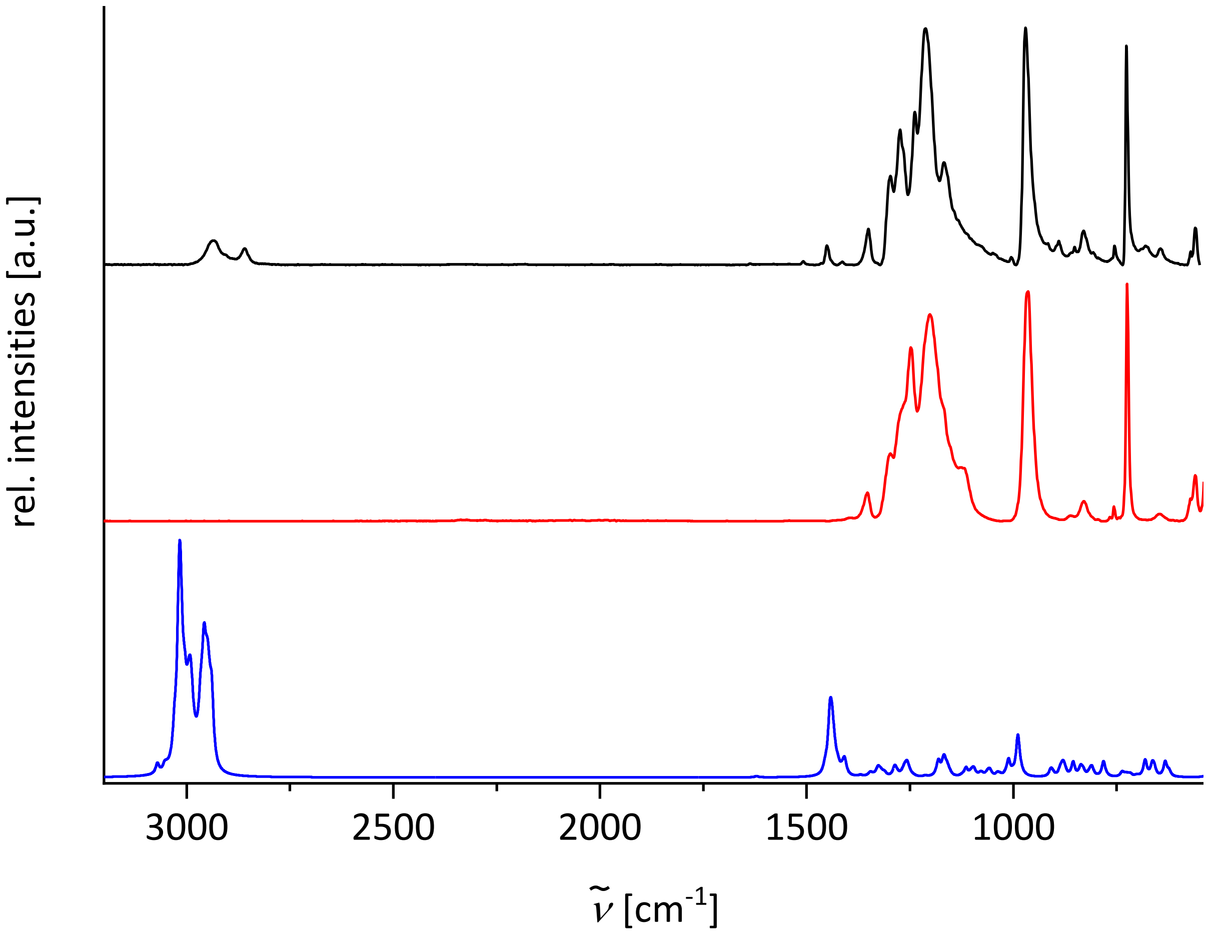

Figure S 181: Comparison of the experimental IR spectra of [{Ga(dcpe)}$_2$(C$_6$H$_{10}$)][*pf*]$_2$ (black) and NO[*pf*] (red) with the calculated IR spectrum (RI-BP86(D3BJ)/def2-TZVPP) of [{Ga(dcpe)}$_2$(C$_6$H$_{10}$)]$^{2+}$ (blue).

Table S 11: IR bands and the assigned vibration modes in [{Ga(dcpe)}$_2$(C_6H_{10})][*pf*]$_2$.

IR	Assignment
2934 (vw)	C–H (dcpe)[a]
2859 (vw)	C–H (dcpe)[a]
1637 (vw)	C=C (H_5C_3=C_3H_5 fragment)[a]
1508 (vw)	-
1451 (vw)	C–C, C–H (dcpe)[a]
1414 (vw)	C–H (dcpe)[a]
1351 (vw)	C–C, C–F ([*pf*]$^-$)[b]
1297 (w)	C–C, C–F, C–O, Al–O ([*pf*]$^-$)[a], C–H (dcpe)[a]
1274 (m)	C–C, C–F ([*pf*]$^-$)[b]
1238 (s)	C–C, C–F ([*pf*]$^-$)[b]
1213 (vs)	C–C, C–O, C–F ([*pf*]$^-$)[a]
1168 (m)	C–C, C–F ([*pf*]$^-$)[a]
1049 (vw)	C–C, C–H (dcpe)[a], C–H (H_5C_3=C_3H_5 fragment)[a]
971 (vs)	C–C, C–F ([*pf*]$^-$)[b]
890 (vw)	C–C, C–H (dcpe)[a]
852 (vw)	C–C, C–H (dcpe)[a]
830 (vw)	Al–O, C–C ([*pf*]$^-$)[b], C–C, C–H (dcpe)[a]
755 (vw)	C–C, C–F, Al–O ([*pf*]$^-$)[a], C–C, C–H (dcpe)[a]
726 (vs)	C–C, C–F, Al–O ([*pf*]$^-$)[a]
680 (vw)	C–C, CH_2 (H_5C_3=C_3H_5 fragment)[a]
644 (vw)	C–C, C–P (dcpe)[a]
571 (vw)	Al–O, C–C ([*pf*]$^-$)[b]
559 (vw)	Al–O, C–C ([*pf*]$^-$)[b]

a) Calculated at RI-BP86(D3BJ)/def2-TZVPP level of theory in the gas phase (section 6). b) Assignments based on literature values.[34–36]

5.2 [H{Ga(dcpe)}$_2$(OEt)][*pf*]$_2$

FT-IR: $\tilde{\nu}$ [cm^{-1}] = 2942 (vw), 2859 (vw), 1863 (vw), 1451 (vw), 1415 (vw), 1352 (vw), 1298 (w), 1274 (m), 1240 (m), 1213 (vs), 1164 (w), 1099 (vw), 1064 (vw), 1003 (vw), 971 (vs), 914 (vw), 888 (vw), 851 (vw), 833 (vw), 755 (vw), 726 (vs), 658 (vw), 643 (vw), 587 (vw), 571 (vw), 560 (vw).

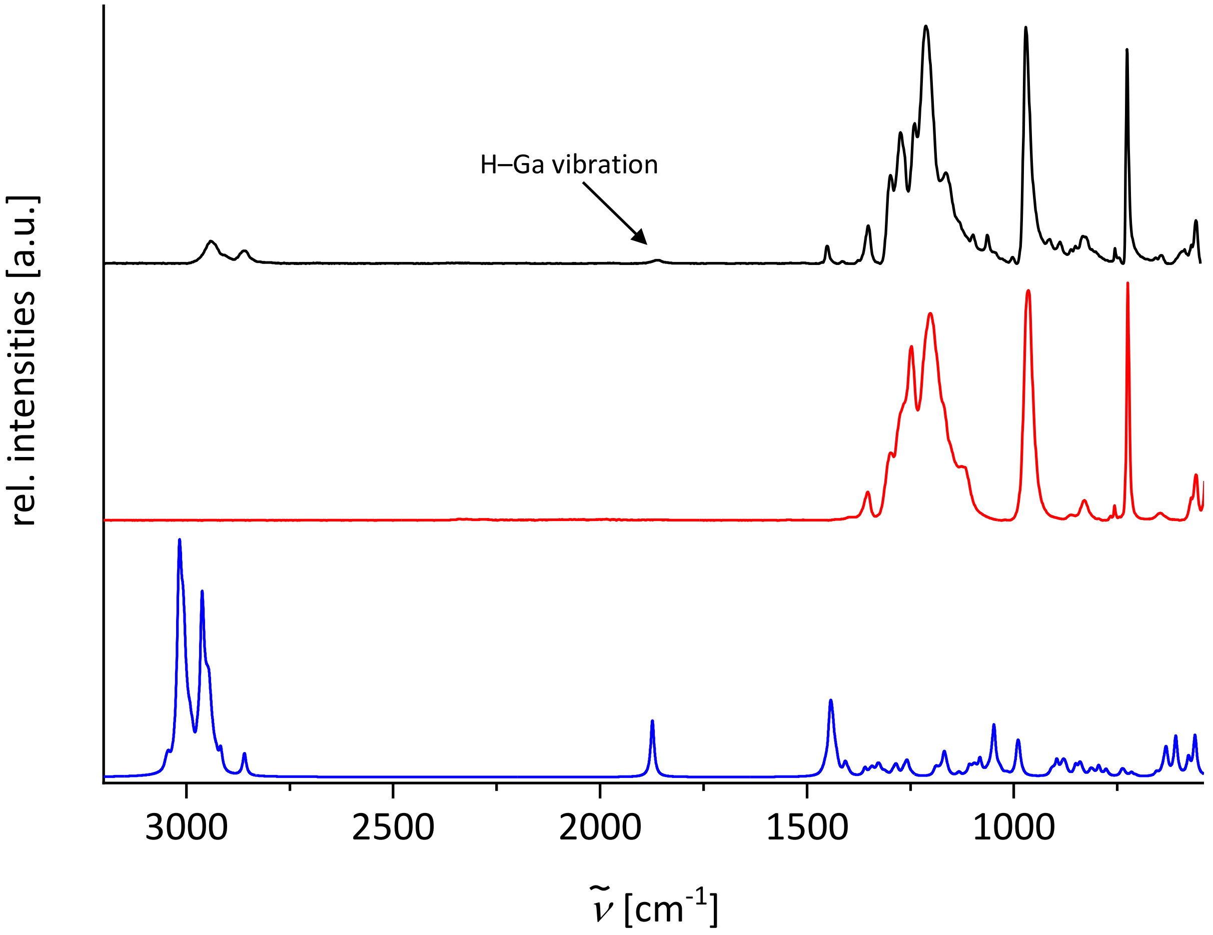

Figure S 182: Comparison of the experimental IR spectra of [H{Ga(dcpe)}$_2$(OEt)][*pf*]$_2$ (black) and NO[*pf*] (red) with the calculated IR spectrum (RI-BP86(D3BJ)/def2-TZVPP) of [H{Ga(dcpe)}$_2$(OEt)]$^{2+}$ (blue).

Table S 12: IR bands and the assigned vibration modes in [H{Ga(dcpe)}$_2$(OEt)][*pf*]$_2$.

IR	Assignment
2942 (vw)	C–H (dcpe)[a]
2859 (vw)	C–H (dcpe)[a]
1863 (vw)	Ga–H[a]
1451 (vw)	C–C, C–H (dcpe)[a]
1415 (vw)	C–H (dcpe)[a]
1352 (vw)	C–C, C–F ([*pf*]$^-$)[b]
1298 (w)	C–C, C–F, C–O, Al–O ([*pf*]$^-$)[a], C–H (dcpe)[a]
1274 (m)	C–C, C–F ([*pf*]$^-$)[b]
1240 (m)	C–C, C–F ([*pf*]$^-$)[b]
1213 (vs)	C–C, C–O, C–F ([*pf*]$^-$)[a]
1164 (w)	C–C, C–F ([*pf*]$^-$)[a]
1099 (vw)	C–C, C–H (dcpe)[a]
1064 (vw)	C–O, C–C (–OCH_2CH_3)[a]
1003 (vw)	C–C, C–H (dcpe)[a]
971 (vs)	C–C, C–F ([*pf*]$^-$)[b]
914 (vw)	C–C, C–F, C–O ([*pf*]$^-$)[a], C–H (dcpe)[a]
888 (vw)	C–C, C–H (dcpe)[a]
851 (vw)	C–C, C–H (dcpe)[a]
833 (vw)	Al–O, C–C ([*pf*]$^-$)[b], C–C, C–H (dcpe)[a]
755 (vw)	C–C, C–F, Al–O ([*pf*]$^-$)[a], C–C, C–H (dcpe)[a]
726 (vs)	C–C, C–F, Al–O ([*pf*]$^-$)[a]
658 (vw)	C–C, C–P (dcpe)[a]
643 (vw)	C–C, C–P (dcpe)[a]
587 (vw)	O–Ga, C–O, C–C (–OCH_2CH_3)[a]
571 (vw)	Al–O, C–C ([*pf*]$^-$)[b]
560 (vw)	Al–O, C–C ([*pf*]$^-$)[b]

a) Calculated at RI-BP86(D3BJ)/def2-TZVPP level of theory in the gas phase (section 6). b) Assignments based on literature values.[34–36]

5.3 [H{Ga(dcpe)}$_2$(OPh)][*pf*]$_2$

FT-IR: $\tilde{\nu}$ [cm^{-1}] = 2940 (vw), 2861 (vw), 1842 (vw), 1592 (vw), 1509 (vw), 1483 (vw), 1452 (vw), 1413 (vw), 1351 (vw), 1297 (w), 1274 (m), 1239 (s), 1210 (vs), 1166 (m), 1074 (vw), 1049 (vw), 1004 (vw), 970 (vs), 920 (vw), 889 (vw), 850 (vw), 831 (vw), 800 (vw), 756 (vw), 726 (vs), 695 (vw), 659 (vw), 645 (vw), 597 (vw), 571 (vw), 559 (vw).

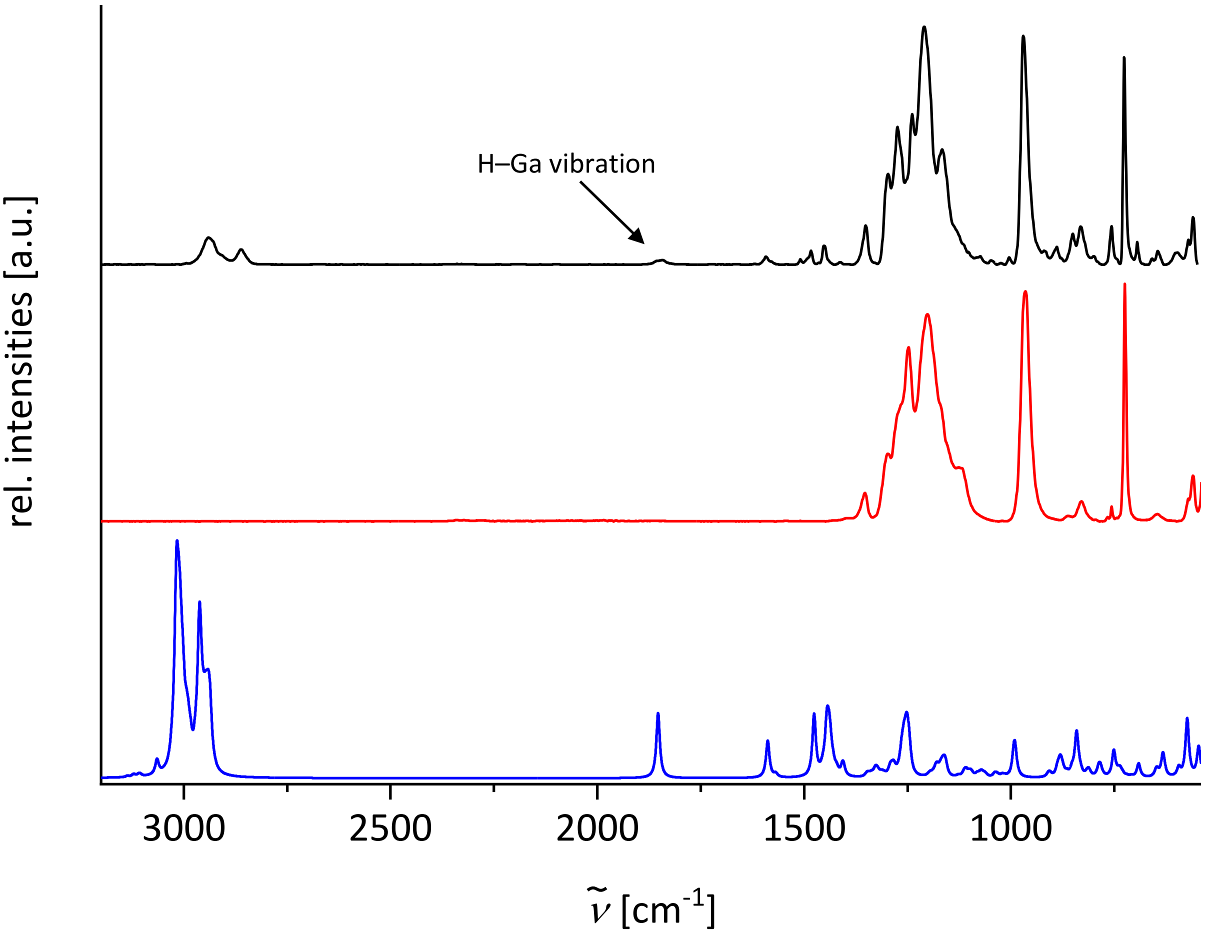

Figure S 183: Comparison of the experimental IR spectra of [H{Ga(dcpe)}$_2$(OPh)][*pf*]$_2$ (black) and NO[*pf*] (red) with the calculated IR spectrum (RI-BP86(D3BJ)/def2-TZVPP) of [H{Ga(dcpe)}$_2$(OPh)]$^{2+}$ (blue).

Table S 13: IR bands and the assigned vibration modes in [H{Ga(dcpe)}$_2$(OPh)][*pf*]$_2$.

IR	Assignment
2940 (vw)	C–H (dcpe)[a]
2861 (vw)	C–H (dcpe)[a]
1842 (vw)	Ga–H[a]
1592 (vw)	C–C, C–O (–C_6H_5)[a]
1509 (vw)	-
1483 (vw)	C–H, C–C, C–O (–C_6H_5)[a]
1452 (vw)	C–C, C–H (dcpe)[a]
1413 (vw)	C–H (dcpe)[a]
1351 (vw)	C–C, C–F ([*pf*]$^-$)[b]
1297 (w)	C–C, C–F, C–O, Al–O ([*pf*]$^-$)[a], C–H (dcpe)[a]
1274 (m)	C–C, C–F ([*pf*]$^-$)[b]
1239 (s)	C–C, C–F ([*pf*]$^-$)[b]
1210 (vs)	C–C, C–O, C–F ([*pf*]$^-$)[a]
1166 (m)	C–C, C–F ([*pf*]$^-$)[a]
1074 (vw)	C–C, C–H (dcpe)[a]
1049 (vw)	C–C, C–H (dcpe)[a]
1003 (vw)	C–C, C–H (dcpe)[a]
970 (vs)	C–C, C–F ([*pf*]$^-$)[b]
920 (vw)	C–C, C–F, C–O ([*pf*]$^-$)[a]
889 (vw)	C–C, C–H (dcpe)[a]
850 (vw)	C–C, C–H (dcpe)[a]
831 (vw)	Al–O, C–C ([*pf*]$^-$)[b], C–C, C–H (dcpe)[a]
800 (vw)	C–H (–C_6H_5)[a]
756 (vw)	C–C, C–F, Al–O ([*pf*]$^-$)[a], C–C, C–H (dcpe)[a]
726 (vs)	C–C, C–F, Al–O ([*pf*]$^-$)[a]
695 (vw)	C–H (–C_6H_5)[a]
659 (vw)	Ga–O, C–C (–C_6H_5)[a], C–C, C–P (dcpe)[a]
645 (vw)	C–C, C–P (dcpe)[a]
597 (vw)	Ga–O, Ga–H, C–C (–C_6H_5)[a]
571 (vw)	Al–O, C–C ([*pf*]$^-$)[b]
559 (vw)	Al–O, C–C ([*pf*]$^-$)[b]

a) Calculated at RI-BP86(D3BJ)/def2-TZVPP level of theory in the gas phase (section 6). b) Assignments based on literature values.[34–36]

5.4 [H{Ga(dcpe)}$_2$(NH*n*Bu)][*pf*]$_2$

FT-IR: $\tilde{\nu}$ [cm^{-1}] = 2942 (vw), 2863 (vw), 1862 (vw), 1453 (vw), 1417 (vw), 1351 (w), 1297 (m), 1273 (s), 1240 (s), 1211 (vs), 1164 (m), 1002 (vw), 970 (vs), 918 (vw), 889 (vw), 853 (vw), 832 (vw), 755 (vw), 726 (vs), 645 (vw), 612 (vw), 595 (vw), 571 (vw), 559 (vw).

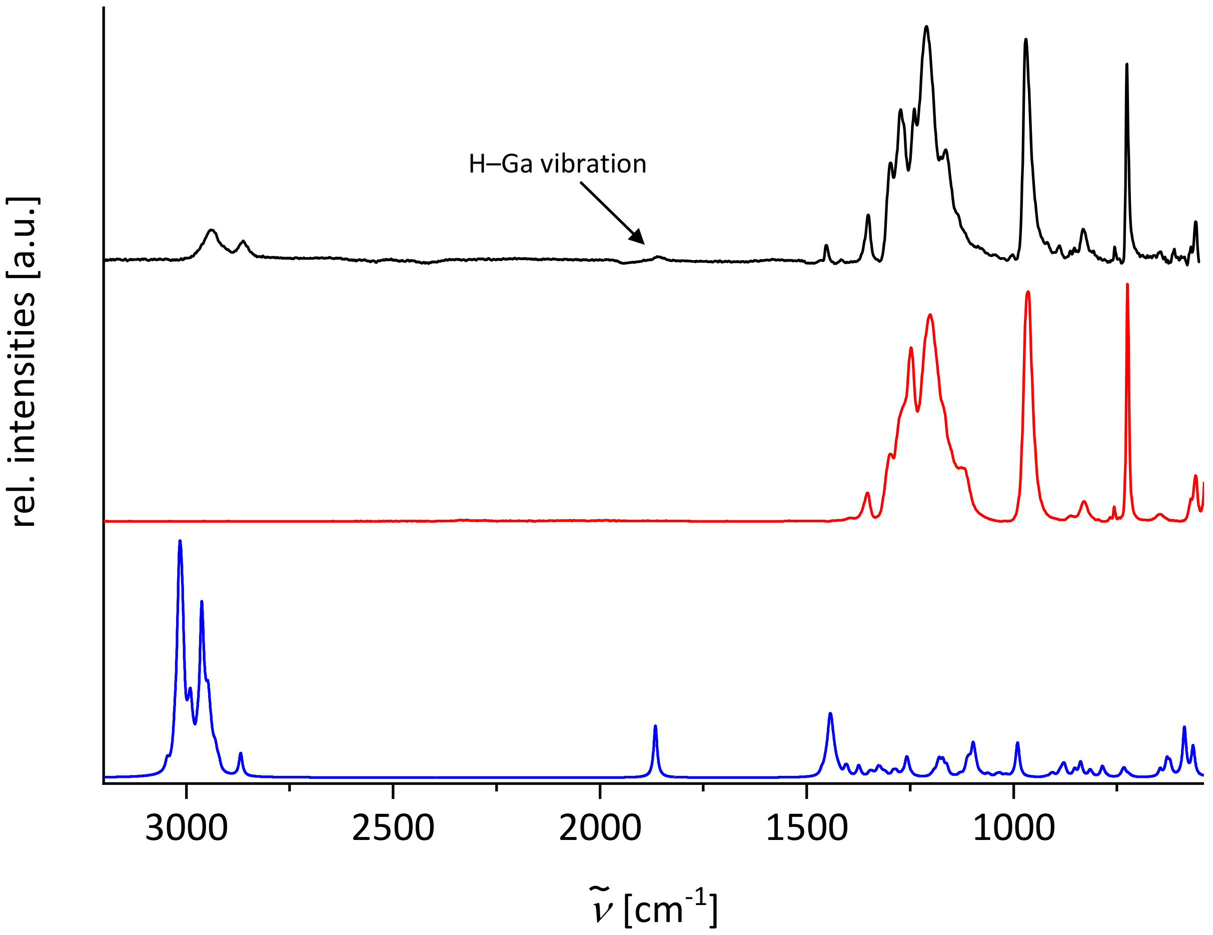

Figure S 184: Comparison of the experimental IR spectra of [H{Ga(dcpe)}$_2$(NH*n*Bu)][*pf*]$_2$ (black) and NO[*pf*] (red) with the calculated IR spectrum (RI-BP86(D3BJ)/def2-TZVPP) of [H{Ga(dcpe)}$_2$(NH*n*Bu)]$^{2+}$ (blue).

Table S 14: IR bands and the assigned vibration modes in [H{Ga(dcpe)}$_2$(NH*n*Bu)][*pf*]$_2$.

IR	Assignment
2942 (vw)	C–H (dcpe)[a]
2863 (vw),	C–H (dcpe)[a]
1862 (vw)	Ga–H[a]
1453 (vw)	C–C, C–H (dcpe)[a], C–C, C–H (–NH*n*Bu)[a]
1417 (vw)	C–H (dcpe)[a]
1351 (w)	C–C, C–F ([*pf*]$^-$)[b]
1297 (m)	C–C, C–F, C–O, Al–O ([*pf*]$^-$)[a], C–H (dcpe)[a]
1273 (s)	C–C, C–F ([*pf*]$^-$)[b]
1240 (s)	C–C, C–F ([*pf*]$^-$)[b]
1211 (vs)	C–C, C–O, C–F ([*pf*]$^-$)[a]
1164 (m)	C–C, C–F ([*pf*]$^-$)[a]
1002 (vw)	C–C, C–H (dcpe)[a]
970 (vs)	C–C, C–F ([*pf*]$^-$)[b]
918 (vw)	C–C, C–F, C–O ([*pf*]$^-$)[a], C–H (dcpe)[a]
889 (vw)	C–C, C–H (dcpe)[a]
853 (vw)	C–C, C–H (dcpe)[a]
832 (vw)	Al–O, C–C ([*pf*]$^-$)[b], C–C, C–H (dcpe)[a]
755 (vw)	C–C, C–F, Al–O ([*pf*]$^-$)[a], C–C, C–H (dcpe)[a]
726 (vs)	C–C, C–F, Al–O ([*pf*]$^-$)[a], C–H, C–C (–NH*n*Bu)[a]
645 (vw)	C–C, C–P (dcpe)[a]
612 (vw)	Ga–N, N–H, N–C (–NH*n*Bu)[a]
595 (vw)	Ga–H, C–C, C–P (dcpe)[a]
571 (vw)	Al–O, C–C ([*pf*]$^-$)[b]
559 (vw)	Al–O, C–C ([*pf*]$^-$)[b]

a) Calculated at RI-BP86(D3BJ)/def2-TZVPP level of theory in the gas phase (section 6). b) Assignments based on literature values.[34–36]

5.5 [H{Ga(dcpe)}$_2$(NHPh)][*pf*]$_2$

FT-IR: $\tilde{\nu}$ [cm^{-1}] = 2940 (vw), 2864 (vw), 1854 (vw), 1597 (vw), 1493 (vw), 1452 (vw), 1419 (vw), 1351 (vw), 1298 (w), 1275 (m), 1240 (s), 1212 (vs), 1168 (m), 1074 (vw), 1045 (vw), 1003 (vw), 971 (vs), 918 (vw), 888 (vw), 864 (vw), 849 (vw), 831 (vw), 749 (vw), 727 (vs), 690 (vw), 659 (vw), 648 (vw), 607 (vw), 571 (vw), 560 (vw).

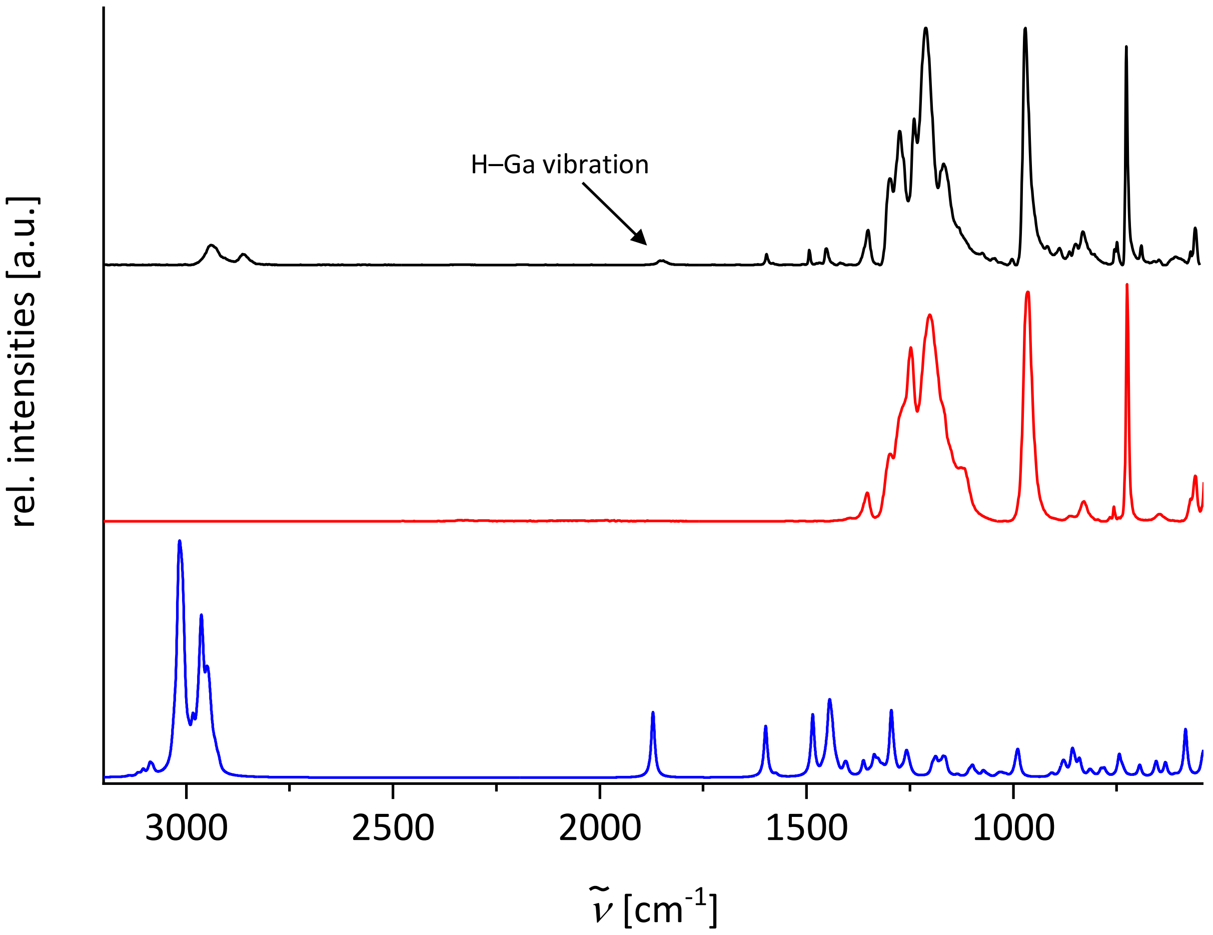

Figure S 185: Comparison of the experimental IR spectra of [H{Ga(dcpe)}$_2$(NHPh)][*pf*]$_2$ (black) and NO[*pf*] (red) with the calculated IR spectrum (RI-BP86(D3BJ)/def2-TZVPP) of [H{Ga(dcpe)}$_2$(NHPh)]$^{2+}$ (blue).

Table S 15: IR bands and the assigned vibration modes in [H{Ga(dcpe)}$_2$(NHPh)][*pf*]$_2$.

IR	Assignment
2940 (vw)	C–H (dcpe)[a]
2864 (vw),	C–H (dcpe)[a]
1854 (vw)	Ga–H[a]
1597 (vw)	C–C, C–N (–C_6H_5)[a]
1493 (vw)	C–H, C–C, C–N (–C_6H_5)[a]
1452 (vw)	C–C, C–H (dcpe)[a]
1419 (vw)	C–H (dcpe)[a]
1351 (vw)	C–C, C–F ([*pf*]$^-$)[b]
1298 (w)	C–C, C–F, C–O, Al–O ([*pf*]$^-$)[a], C–H (dcpe)[a]
1275 (m)	C–C, C–F ([*pf*]$^-$)[b]
1240 (s)	C–C, C–F ([*pf*]$^-$)[b]
1212 (vs)	C–C, C–O, C–F ([*pf*]$^-$)[a]
1168 (m)	C–C, C–F ([*pf*]$^-$)[a]
1074 (vw)	C–C, C–H (dcpe)[a]
1045 (vw)	C–C, C–H (dcpe)[a]
1003 (vw)	C–C, C–H (dcpe)[a]
971 (vs)	C–C, C–F ([*pf*]$^-$)[b]
918 (vw)	C–C, C–F, C–O ([*pf*]$^-$)[a]
888 (vw)	C–C, C–H (dcpe)[a]
864 (vw)	C–C, C–H (dcpe)[a]
849 (vw)	C–C, C–H (dcpe)[a]
831 (vw)	Al–O, C–C ([*pf*]$^-$)[b], C–C, C–H (dcpe)[a]
749 (vw)	C–C, C–F, Al–O ([*pf*]$^-$)[a], C–C, C–H (dcpe)[a]
727 (vs)	C–C, C–F, Al–O ([*pf*]$^-$)[a]
690 (vw)	C–H (–C_6H_5)[a]
659 (vw)	Ga–N, N–H, C–C (–C_6H_5)[a], C–C, C–P (dcpe)[a]
648 (vw)	C–C, C–P (dcpe)[a]
607 (vw)	Ga–N, Ga–H, N–H, C–C (–C_6H_5)[a]
571 (vw)	Al–O, C–C ([*pf*]$^-$)[b]
560 (vw)	Al–O, C–C ([*pf*]$^-$)[b]

a) Calculated at RI-BP86(D3BJ)/def2-TZVPP level of theory in the gas phase (section 6). b) Assignments based on literature values.[34–36]

5.6 [H{Ga(dcpe)}$_2$(NPh$_2$)][*pf*]$_2$

FT-IR: $\tilde{\nu}$ [cm^{-1}] = 2937 (vw), 2861 (vw), 1834 (vw), 1585 (vw), 1483 (vw), 1451 (vw), 1351 (vw), 1298 (w), 1274 (m), 1239 (s), 1211 (vs), 1166 (w), 1134 (w), 1002 (vw), 971 (vs), 927 (vw), 915 (vw), 890 (vw), 869 (vw), 830 (vw), 752 (vw), 726 (vs), 703 (vw), 694 (vw), 605 (vw), 571 (vw), 559 (vw).

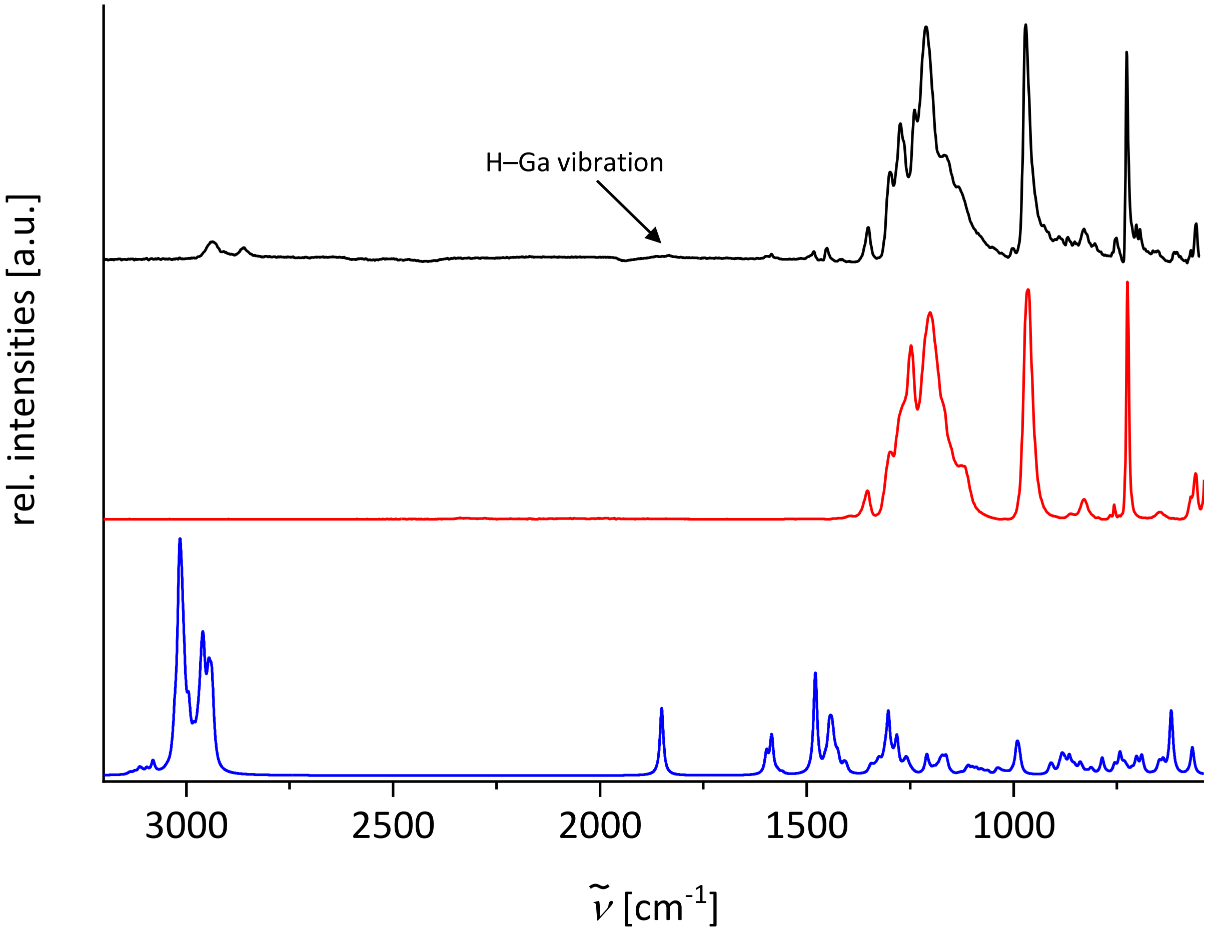

Figure S 186: Comparison of the experimental IR spectra of [H{Ga(dcpe)}$_2$(NPh$_2$)][*pf*]$_2$ (black) and NO[*pf*] (red) with the calculated IR spectrum (RI-BP86(D3BJ)/def2-TZVPP) of [H{Ga(dcpe)}$_2$(NPh$_2$)]$^{2+}$ (blue).

Table S 16: IR bands and the assigned vibration modes in $[H\{Ga(dcpe)\}_2(NPh_2)][pf]_2$.

IR	Assignment
2937 (vw)	C–H (dcpe)[a]
2861 (vw)	C–H (dcpe)[a]
1834 (vw)	Ga–H[a]
1585 (vw)	C–C, C–N ($-C_6H_5$)[a]
1483 (vw)	C–H, C–C, C–N ($-C_6H_5$)[a]
1451 (vw)	C–C, C–H (dcpe)[a], C–C, C–H ($-C_6H_5$)[a]
1351 (vw)	C–C, C–F ($[pf]^-$)[b]
1298 (w)	C–C, C–F, C–O, Al–O ($[pf]^-$)[a], C–H (dcpe)[a], C–H ($-C_6H_5$)[a]
1274 (m)	C–C, C–F ($[pf]^-$)[b]
1239 (s)	C–C, C–F ($[pf]^-$)[b]
1211 (vs)	C–C, C–O, C–F ($[pf]^-$)[a]
1166 (w)	C–C, C–F ($[pf]^-$)[a]
1134 (w)	C–C, C–H (dcpe)[a]
1002 (vw)	C–C, C–H (dcpe)[a]
971 (vs)	C–C, C–F ($[pf]^-$)[b]
927 (vw)	-
915 (vw)	C–C, C–F, C–O ($[pf]^-$)[a], C–H (dcpe)[a], C–H ($-C_6H_5$)[a]
890 (vw)	C–C, C–H (dcpe)[a]
869 (vw)	C–C, C–H (dcpe)[a]
830 (vw)	Al–O, C–C ($[pf]^-$)[b], C–C, C–H (dcpe)[a]
752 (vw)	C–C, C–F, Al–O ($[pf]^-$)[a], C–C, C–H (dcpe)[a]
726 (vs)	C–C, C–F, Al–O ($[pf]^-$)[a]
703 (vw)	C–C, C–P (dcpe)[a], C–H ($-C_6H_5$)[a]
694 (vw)	C–H ($-C_6H_5$)[a]
605 (vw)	Ga–H, Ga–N, C–C ($-C_6H_5$)[a]
571 (vw)	Al–O, C–C ($[pf]^-$)[b]
559 (vw)	Al–O, C–C ($[pf]^-$)[b]

a) Calculated at RI-BP86(D3BJ)/def2-TZVPP level of theory in the gas phase (section 6). b) Assignments based on literature values.[34–36]

5.7 [H{Ga(dcpe)}$_2$(CH_2CN)][*pf*]$_2$

FT-IR: $\tilde{\nu}$ [cm^{-1}] = 2940 (vw), 2862 (vw), 2223 (vw), 1851 (vw), 1509 (vw), 1453 (vw), 1351 (vw), 1296 (w), 1272 (m), 1238 (s), 1208 (vs), 1177 (m), 1164 (m), 1134 (w), 1102 (vw), 1085 (vw), 1000 (vw), 969 (vs), 918 (vw), 890 (vw), 853 (vw), 832 (vw), 755 (vw), 725 (s), 650 (vw), 571 (vw), 560 (vw).

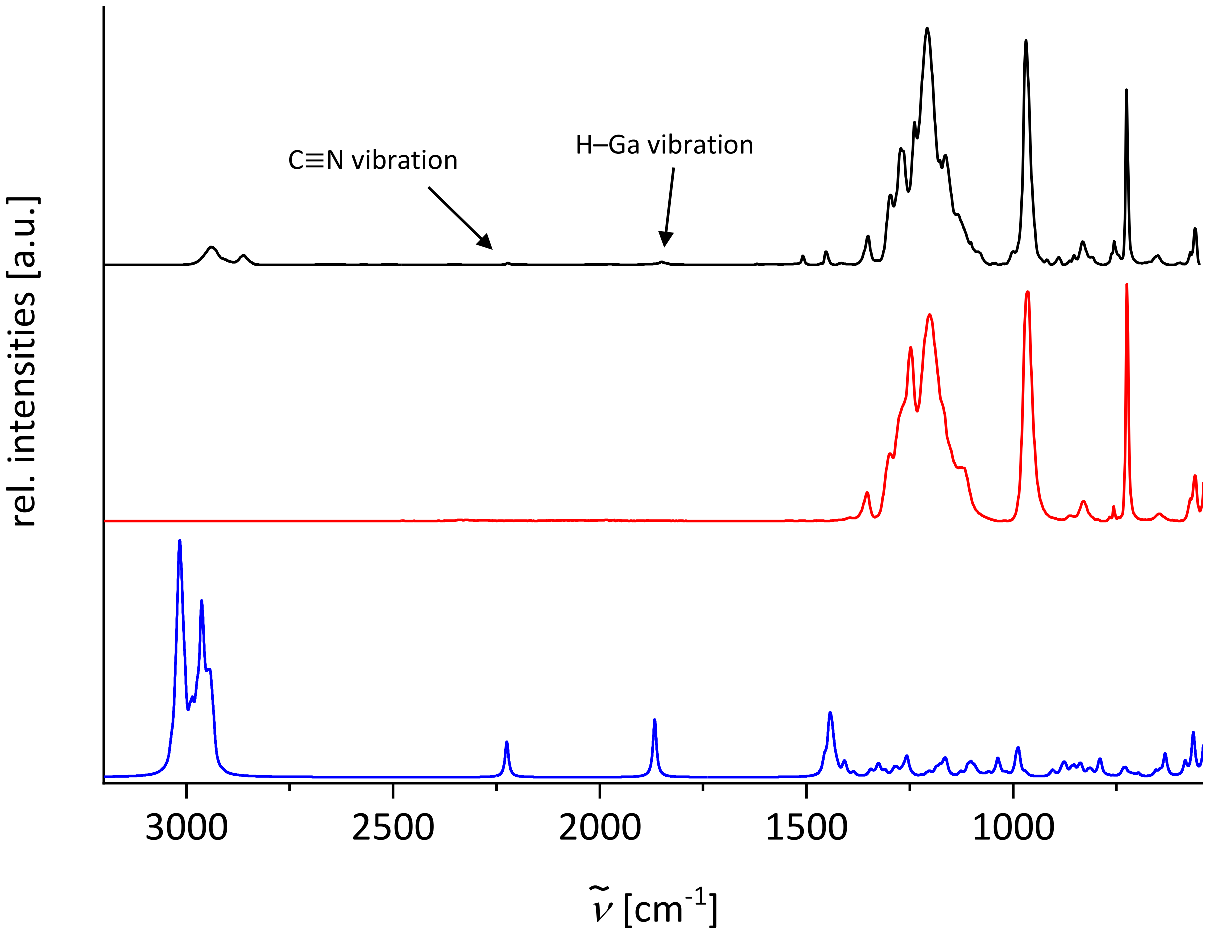

Figure S 187: Comparison of the experimental IR spectra of [H{Ga(dcpe)}$_2$(CH_2CN)][*pf*]$_2$ (black) and NO[*pf*] (red) with the calculated IR spectrum (RI-BP86(D3BJ)/def2-TZVPP) of [H{Ga(dcpe)}$_2$(CH_2CN)]$^{2+}$ (blue).

Table S 17: IR bands and the assigned vibration modes in [H{Ga(dcpe)}$_2$(CH_2CN)][*pf*]$_2$.

IR	Assignment
2940 (vw)	C–H (dcpe)[a]
2862 (vw)	C–H (dcpe)[a]
2223 (vw)	C≡N (CH_2CN fragment)[a]
1851 (vw)	Ga–H[a]
1509 (vw)	-
1453 (vw)	C–C, C–H (dcpe)[a]
1351 (vw)	C–C, C–F ([*pf*]$^-$)[b]
1296 (w)	C–C, C–F, C–O, Al–O ([*pf*]$^-$)[a], C–H (dcpe)[a]
1272 (m)	C–C, C–F ([*pf*]$^-$)[b]
1238 (s)	C–C, C–F ([*pf*]$^-$)[b]
1208 (vs)	C–C, C–O, C–F ([*pf*]$^-$)[a]
1177 (m)	C–C, C–F ([*pf*]$^-$)[a], C–H (dcpe)[a]
1164 (m)	C–C, C–F ([*pf*]$^-$)[a]
1134 (w)	-
1102 (vw)	C–C, C–H (dcpe)[a], CH_2 (CH_2CN fragment)[a]
1085 (vw)	C–C, C–H (dcpe)[a], CH_2 (CH_2CN fragment)[a]
1000 (vw)	C–C, C–H (dcpe)[a]
969 (s)	C–C, C–F ([*pf*]$^-$)[b]
918 (vw)	C–C, C–F, C–O ([*pf*]$^-$)[a], C–H (dcpe)[a]
890 (vw)	C–C, C–H (dcpe)[a]
853 (vw)	C–C, C–H (dcpe)[a]
832 (vw)	Al–O, C–C ([*pf*]$^-$)[b], C–C, C–H (dcpe)[a]
755 (vw)	C–C, C–F, Al–O ([*pf*]$^-$)[a], C–C, C–H (dcpe)[a]
725 (s)	C–C, C–F, Al–O ([*pf*]$^-$)[a]
650 (vw)	C–C, C–P (dcpe)[a]
571 (vw)	Al–O, C–C ([*pf*]$^-$)[b]
560 (vw)	Al–O, C–C ([*pf*]$^-$)[b]

a) Calculated at RI-BP86(D3BJ)/def2-TZVPP level of theory in the gas phase (section 6). b) Assignments based on literature values.[34–36]

5.8 [F{Ga(dcpe)}$_2$(CH_2CN)][*pf*]$_2$

FT-IR: $\tilde{\nu}$ [cm^{-1}] = 2940 (vw), 2864 (vw), 2221 (vw), 1508 (vw), 1453 (vw), 1418 (vw), 1351 (vw), 1298 (w), 1273 (s), 1240 (s), 1210 (vs), 1177 (m), 1164 (m), 1079 (vw), 1003 (vw), 970 (vs), 919 (vw), 890 (vw), 863 (vw), 852 (vw), 831 (vw), 755 (vw), 726 (vs), 657 (vw), 649 (vw), 583 (vw), 571 (vw), 560 (w).

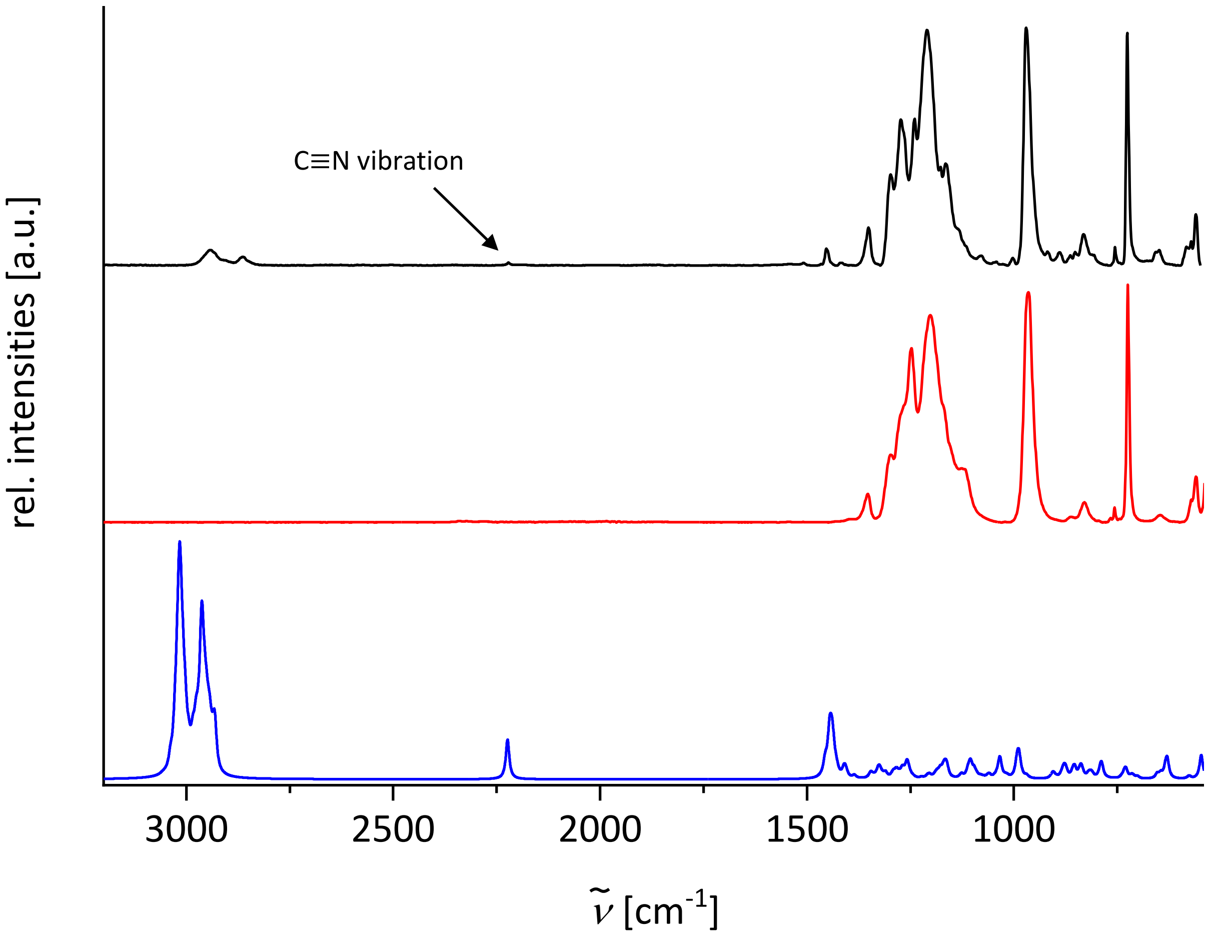

Figure S 188: Comparison of the experimental IR spectra of [F{Ga(dcpe)}$_2$(CH_2CN)][*pf*]$_2$ (black) and NO[*pf*] (red) with the calculated IR spectrum (RI-BP86(D3BJ)/def2-TZVPP) of [F{Ga(dcpe)}$_2$(CH_2CN)]$^{2+}$ (blue).

Table S 18: IR bands and the assigned vibration modes in [F{Ga(dcpe)}$_2$(CH$_2$CN)][*pf*]$_2$.

IR	Assignment
2940 (vw)	C–H (dcpe)[a]
2864 (vw)	C–H (dcpe)[a]
2221 (vw)	C≡N (CH$_2$CN fragment)[a]
1508 (vw)	-
1453 (vw)	C–C, C–H (dcpe)[a]
1418 (vw)	C–H (dcpe)[a]
1351 (vw)	C–C, C–F ([*pf*]$^-$)[b]
1298 (w)	C–C, C–F, C–O, Al–O ([*pf*]$^-$)[a], C–H (dcpe)[a]
1273 (s)	C–C, C–F ([*pf*]$^-$)[b]
1240 (s)	C–C, C–F ([*pf*]$^-$)[b]
1210 (vs)	C–C, C–O, C–F ([*pf*]$^-$)[a]
1177 (m)	C–C, C–F ([*pf*]$^-$)[a], C–H (dcpe)[a]
1164 (m)	C–C, C–F ([*pf*]$^-$)[a]
1079 (vw)	C–C, C–H (dcpe)[a], CH$_2$ (CH$_2$CN fragment)[a]
1003 (vw)	C–C, C–H (dcpe)[a]
970 (vs)	C–C, C–F ([*pf*]$^-$)[b]
919 (vw)	C–C, C–F, C–O ([*pf*]$^-$)[a], C–H (dcpe)[a]
890 (vw)	C–C, C–H (dcpe)[a]
863 (vw)	C–C, C–H (dcpe)[a]
852 (vw)	C–C, C–H (dcpe)[a]
831 (vw)	Al–O, C–C ([*pf*]$^-$)[b], C–C, C–H (dcpe)[a]
755 (vw)	C–C, C–F, Al–O ([*pf*]$^-$)[a], C–C, C–H (dcpe)[a]
726 (vs)	C–C, C–F, Al–O ([*pf*]$^-$)[a]
657 (vw)	C–C, C–P (dcpe)[a]
649 (vw)	C–C, C–P (dcpe)[a]
583 (vw)	Ga–C, C–C, C–N (CH$_2$CN fragment)[a]
571 (vw)	Al–O, C–C ([*pf*]$^-$)[b]
560 (w)	Al–O, C–C ([*pf*]$^-$)[b]

a) Calculated at RI-BP86(D3BJ)/def2-TZVPP level of theory in the gas phase (section 6). b) Assignments based on literature values.[34–36]

5.9 [F{Ga(dcpe)}$_2$(COOCH$_2$CHO)][*pf*]$_2$

FT-IR: $\tilde{\nu}$ [cm^{-1}] = 2943 (vw), 2862 (vw), 1835 (vw), 1751 (vw), 1674 (vw), 1452 (vw), 1414 (vw), 1352 (w), 1297 (m), 1274 (s), 1240 (s), 1213 (vs), 1164 (m), 1077 (vw), 1043 (vw), 1003 (vw), 971 (vs), 918 (vw), 889 (vw), 834 (vw), 756 (vw), 726 (vs), 641 (vw), 586 (vw), 572 (vw), 560 (w).

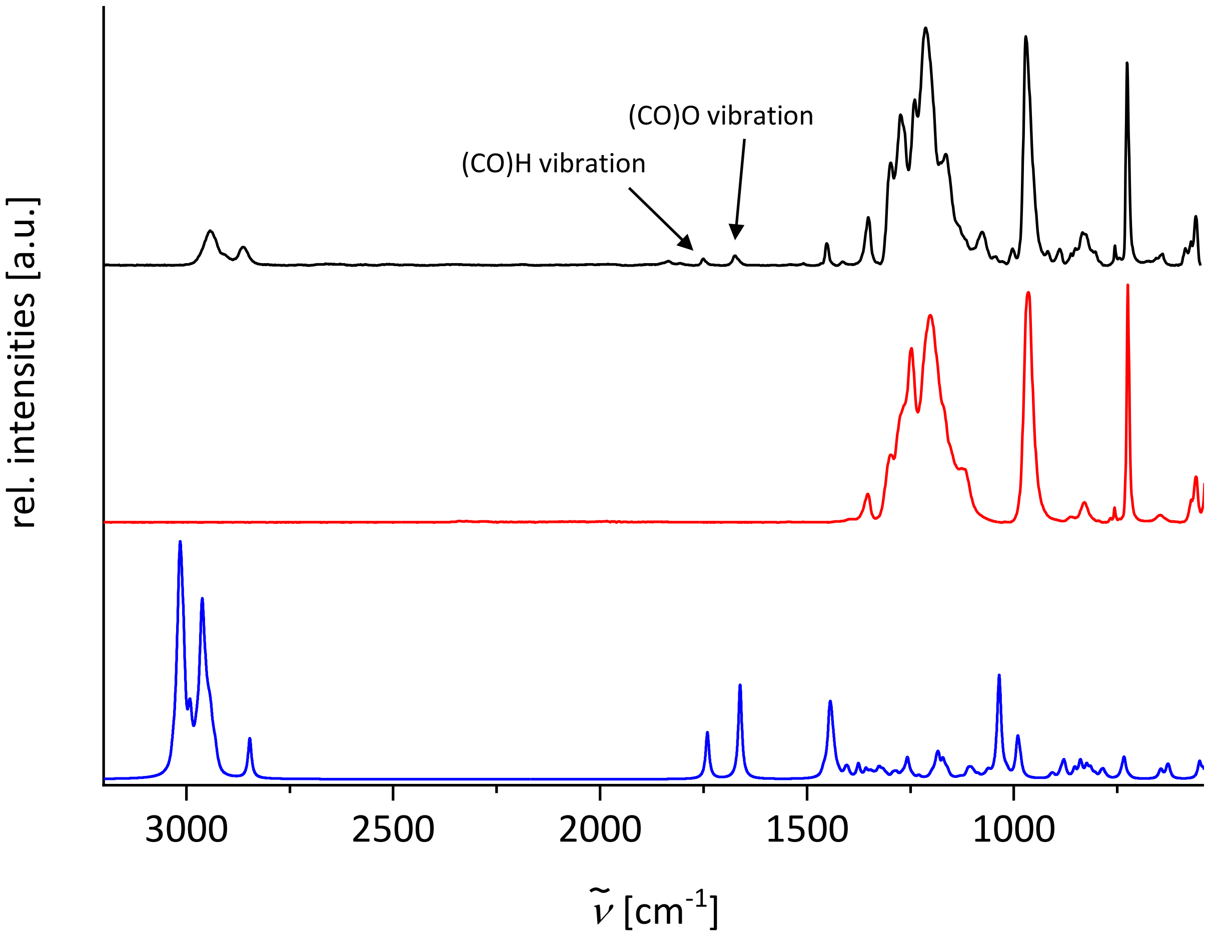

Figure S 189: Comparison of the experimental IR spectra of [F{Ga(dcpe)}$_2$(COOCH$_2$CHO)][*pf*]$_2$ (black) and NO[*pf*] (red) with the calculated IR spectrum (RI-BP86(D3BJ)/def2-TZVPP) of [F{Ga(dcpe)}$_2$(COOCH$_2$CHO)]$^{2+}$ (blue).

Table S 19: IR bands and the assigned vibration modes in [F{Ga(dcpe)}$_2$(COOCH$_2$CHO)][*pf*]$_2$.

IR	Assignment
2943 (vw)	C–H (dcpe)[a]
2862 (vw)	C–H (dcpe)[a]
1835 (vw)	-
1751 (vw)	(C=O)H (COOCH$_2$CHO fragment)[a]
1674 (vw)	(C=O)O (COOCH$_2$CHO fragment)[a]
1452 (vw)	C–C, C–H (dcpe)[a]
1414 (vw)	C–H (dcpe)[a]
1352 (w)	C–C, C–F ([*pf*]$^-$)[b]
1297 (m)	C–C, C–F, C–O, Al–O ([*pf*]$^-$)[a], C–H (dcpe)[a]
1274 (s)	C–C, C–F ([*pf*]$^-$)[b]
1240 (s)	C–C, C–F ([*pf*]$^-$)[b], CH$_2$ (COOCH$_2$CHO fragment)[a]
1213 (vs)	C–C, C–O, C–F ([*pf*]$^-$)[a]
1164 (m)	C–C, C–F ([*pf*]$^-$)[a]
1077 (vw)	C–C, C–H (dcpe)[a]
1043 (vw)	C–C, C–H (dcpe)[a], C–O, C–C (COOCH$_2$CHO fragment)[a]
1003 (vw)	C–C, C–H (dcpe)[a]
971 (vs)	C–C, C–F ([*pf*]$^-$)[b]
918 (vw)	C–C, C–F, C–O ([*pf*]$^-$)[a], C–H (dcpe)[a]
889 (vw)	C–C, C–H (dcpe)[a]
834 (vw)	Al–O, C–C ([*pf*]$^-$)[b], C–C, C–H (dcpe)[a]
756 (vw)	C–C, C–F, Al–O ([*pf*]$^-$)[a], C–C, C–H (dcpe)[a]
726 (vs)	C–C, C–F, Al–O ([*pf*]$^-$)[a]
641 (vw)	C–C, C–P (dcpe)[a]
586 (vw)	Ga–C, C–C, C–N (CH$_2$CN fragment)[a]
572 (vw)	Al–O, C–C ([*pf*]$^-$)[b]
560 (w)	Al–O, C–C ([*pf*]$^-$)[b]

a) Calculated at RI-BP86(D3BJ)/def2-TZVPP level of theory in the gas phase (section 6). b) Assignments based on literature values.[34–36]

5.10 [F{Ga(dcpe)}$_2$(C$_6$F$_2$H$_3$)][*pf*]$_{5/3}$[*alfal*]$_{1/3}$

FT-IR: $\tilde{\nu}$ [cm^{-1}] = 2938 (vw), 2865 (vw), 1608 (vw), 1453 (vw), 1418 (vw), 1351 (vw), 1298 (w), 1274 (m), 1240 (s), 1210 (vs), 1167 (m), 1087 (vw), 1004 (vw), 970 (vs), 918 (vw), 890 (vw), 866 (vw), 852 (vw), 824 (vw), 803 (vw), 787 (vw), 755 (vw), 726 (vs), 649 (vw), 571 (vw), 560 (vw).

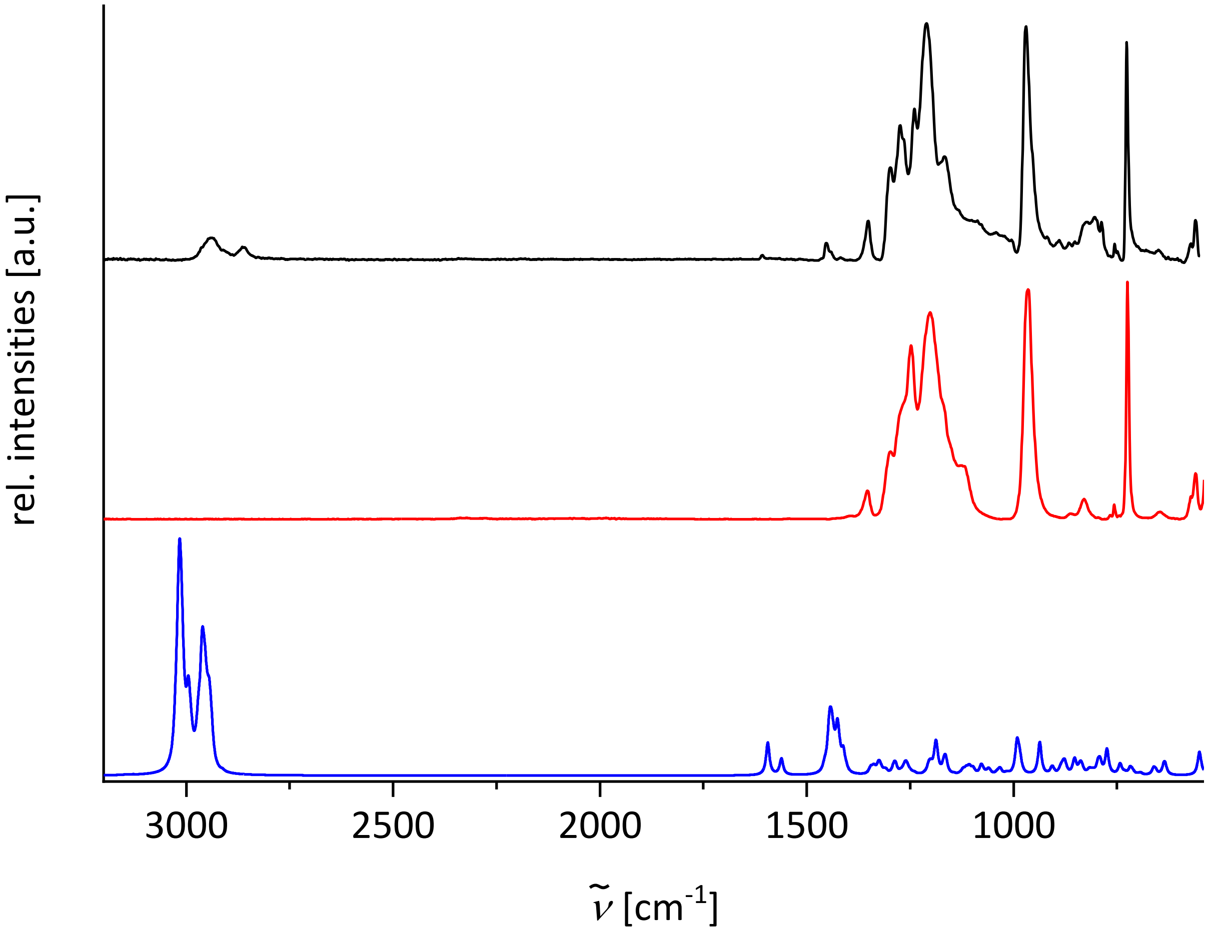

Figure S 190: Comparison of the experimental IR spectra of [F{Ga(dcpe)}$_2$(C$_6$F$_2$H$_3$)][*pf*]$_{5/3}$[*alfal*]$_{1/3}$ (black) and NO[*pf*] (red) with the calculated IR spectrum (RI-BP86(D3BJ)/def2-TZVPP) of [F{Ga(dcpe)}$_2$(C$_6$F$_2$H$_3$)]$^{2+}$ (blue).

Table S 20: IR bands and the assigned vibration modes in $[F\{Ga(dcpe)\}_2(C_6F_2H_3)][pf]_{5/3}[alfal]_{1/3}$.

IR	Assignment
2938 (vw)	C–H (dcpe)[a]
2865 (vw)	C–H (dcpe)[a]
1608 (vw)	C–C ($–C_6F_2H_3$)[a]
1453 (vw)	C–C, C–H (dcpe)[a]
1418 (vw)	C–H (dcpe)[a]
1351 (vw)	C–C, C–F ($[pf]^-$)[b]
1298 (w)	C–C, C–F, C–O, Al–O ($[pf]^-$)[a], C–H (dcpe)[a]
1274 (m)	C–C, C–F ($[pf]^-$)[b]
1240 (s)	C–C, C–F ($[pf]^-$)[b]
1210 (vs)	C–C, C–O, C–F ($[pf]^-$)[a]
1167 (m)	C–C, C–F ($[pf]^-$)[a]
1087 (vw)	C–C, C–H (dcpe)[a]
1004 (vw)	C–C, C–H (dcpe)[a]
970 (vs)	C–C, C–F ($[pf]^-$)[b]
918 (vw)	C–C, C–F, C–O ($[pf]^-$)[a], C–H (dcpe)[a]
890 (vw)	C–C, C–H (dcpe)[a]
866 (vw)	C–H ($–C_6F_2H_3$)[a]
852 (vw)	C–C, C–H (dcpe)[a]
824 (vw)	Al–O, C–C ($[pf]^-$)[b], C–C, C–H (dcpe)[a]
803 (vw)	C–C, C–H (dcpe)[a]
787 (vw)	C–C, C–H (dcpe)[a]
755 (vw)	C–C, C–F, Al–O ($[pf]^-$)[a], C–C, C–H (dcpe)[a]
726 (vs)	C–C, C–F, Al–O ($[pf]^-$)[a]
649 (vw)	C–C, C–P (dcpe)[a]
571 (vw)	Al–O, C–C ($[pf]^-$)[b]
560 (vw)	Al–O, C–C ($[pf]^-$)[b], Ga–F[a]

a) Calculated at RI-BP86(D3BJ)/def2-TZVPP level of theory in the gas phase (section 6). b) Assignments based on literature values.[34–36]

6 Quantum Chemical Calculations

6.1 EDA-NOCV Analysis

6.1.1 [{Ga(dcpe)}$_2$]$^{2+}$ + HOMe

Energies	Orbital Interaction	Interacting Fragments: [{Ga(dcpe)}$_2$]$^{2+}$ + HOMe
ΔE_{int}		−16.8
$\Delta E_{\mathrm{Pauli}}$		38.7
$\Delta E_{\mathrm{disp}}{}^{\mathrm{I}}$		−11.1<br>(20.0 %)
$\Delta E_{\mathrm{elstat}}{}^{\mathrm{I}}$		−25.9<br>(46.6 %)
$\Delta E_{\mathrm{orb}}{}^{\mathrm{I}}$		−18.6<br>(33.5 %)
$\Delta E_{\mathrm{orb(1)}}{}^{\mathrm{II}}$	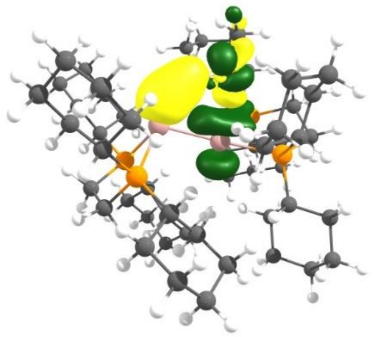<br>p(HOMO and HOMO–1) → σ*(LUMO)<br>and<br>s(HOMO) → σ*(LUMO)	−9.41<br>(50.7 %)<br>$\lvert \nu_1$=0.33$\rvert$
$\Delta E_{\mathrm{orb(2)}}{}^{\mathrm{II}}$	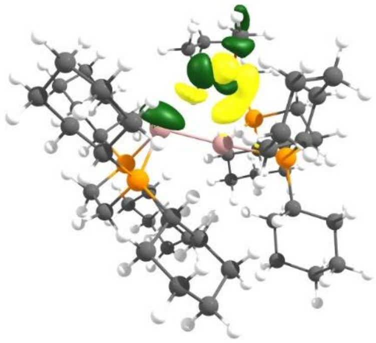<br>s(HOMO) → σ*(LUMO)	−3.07<br>(16.5 %)<br>$\lvert \nu_2$=0.16$\rvert$
$\Delta E_{\mathrm{orb(rest)}}{}^{\mathrm{II}}$		−6.07<br>(32.7 %)

Figure S 191: Results of the EDA-NOCV analysis of the **1**$^{2+}$/HOMe adduct. The shape of the deformation densities $\Delta\rho_{(1)}$ and $\Delta\rho_{(2)}$, corresponding to $\Delta E_{\mathrm{orb(1)}}$ and $\Delta E_{\mathrm{orb(2)}}$, are shown exemplarily (isosurface values: 0.0008 au). The eigenvalues ν_1 and ν_2 give the size of the charge migration in e. The direction of the charge flow of the deformation densities is green→yellow. All calculations were performed at the BP86(D3BJ)/def2-TZVPP level of theory; energy values are given in kcal mol^{-1} instead of the typically recommended kJ mol^{-1} for better comparability with other EDA-NOCV analyses in the literature. I) Values in parentheses show the contribution to the total attractive interaction. II) Values in parentheses show the contribution to the total orbital interaction ΔE_{orb}.

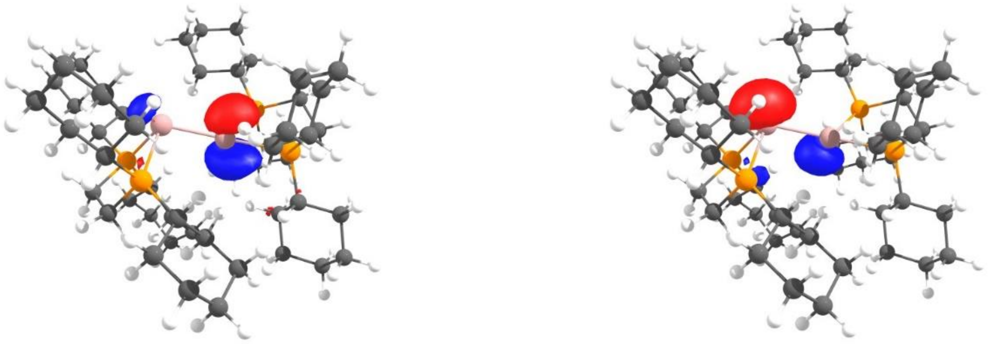

Figure S 192: LUMO (left) and HOMO (right) of the conformer of $\mathbf{1}^{2+}$ interacting with HOMe (contour value: 0.06 au).

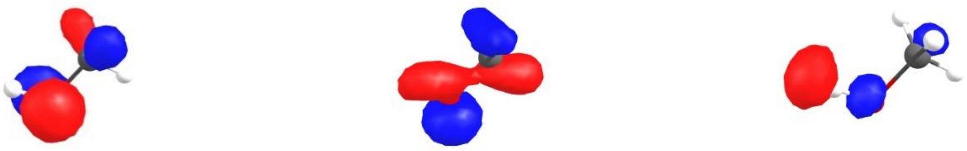

Figure S 193: HOMO (left), HOMO−1 (middle) and LUMO (right) of HOMe interacting with $\mathbf{1}^{2+}$ (contour value: 0.08 au).

6.1.2 $[\{Ga(dcpe)\}_2]^{2+}$ + $HNMe_2$

Energies	Orbital Interaction	Interacting Fragments: $[\{Ga(dcpe)\}_2]^{2+}$ + $HNMe_2$
ΔE_{int}		−37.1
ΔE_{Pauli}		97.3
$\Delta E_{disp}{}^{I}$		−19.4 (14.4 %)
$\Delta E_{elstat}{}^{I}$		−68.1 (50.6 %)
$\Delta E_{orb}{}^{I}$		−47.0 (34.9 %)
$\Delta E_{orb(1)}{}^{II}$	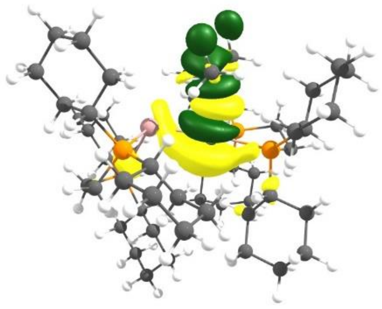 *p*(HOMO) → σ*(LUMO)	−31.6 (67.3 %) $\lvert v_1$=0.55$\rvert$
$\Delta E_{orb(2)}{}^{II}$	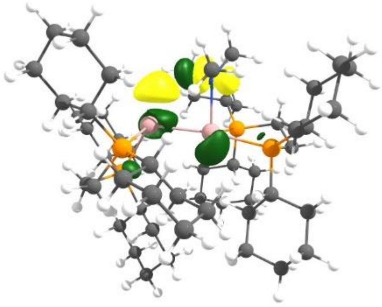 *s*(HOMO) → σ*(LUMO and LUMO+1)	−2.79 (5.9 %) $\lvert v_2$=0.20$\rvert$
$\Delta E_{orb(rest)}{}^{II}$		−12.6 (26.8 %)

Figure S 194: Results of the EDA-NOCV analysis of the **1**$^{2+}$/$HNMe_2$ adduct. The shape of the deformation densities $\Delta\rho_{(1)}$ and $\Delta\rho_{(2)}$, corresponding to $\Delta E_{orb(1)}$ and $\Delta E_{orb(2)}$, are shown exemplarily (isosurface values: 0.0008 au). The eigenvalue v_1 and v_2 give the size of the charge migration in e. The direction of the charge flow of the deformation densities is green→yellow. All calculations were performed at the BP86(D3BJ)/def2-TZVPP level of theory; energy values are given in kcal mol^{-1} instead of the typically recommended kJ mol^{-1} for better comparability with other EDA-NOCV analyses in the literature. I) Values in parentheses show the contribution to the total attractive interaction. II) Values in parentheses show the contribution to the total orbital interaction ΔE_{orb}.

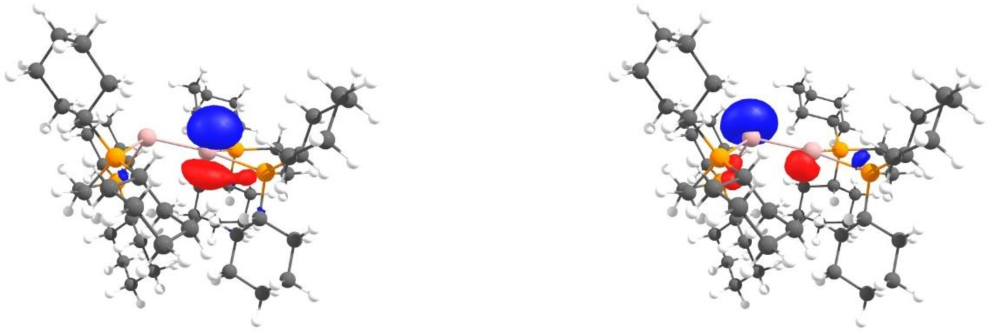

Figure S 195: LUMO (left) and HOMO (right) of $\mathbf{1}^{2+}$ interacting with $HNMe_2$ (contour value: 0.06 au).

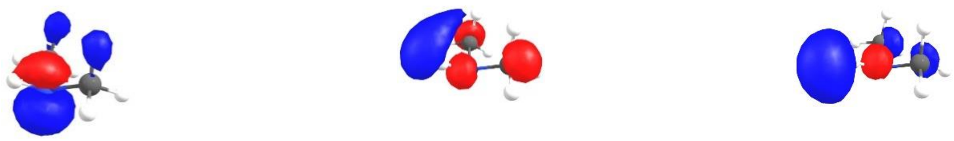

Figure S 196: HOMO (left), LUMO (middle) and LUMO+1 (right) of $HNMe_2$ interacting with $\mathbf{1}^{2+}$ (contour value: 0.08 au for the HOMO and 0.05 for the LUMO and LUMO+1).

6.1.3 Summary of the EDA-NOCV Analysis

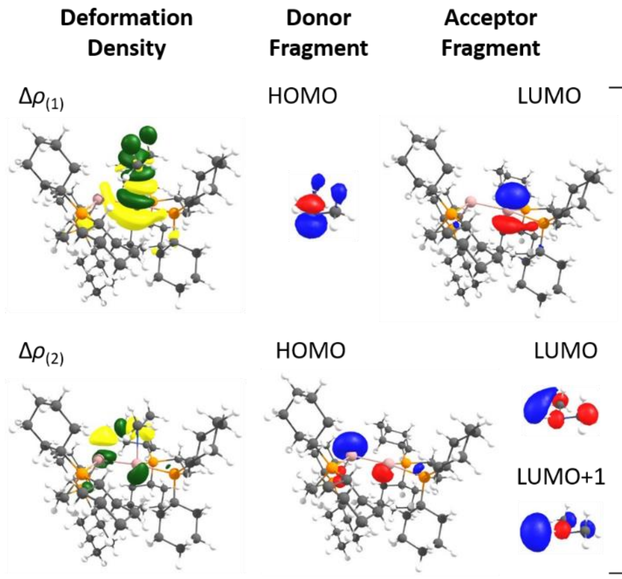

Energies	Orbital Interaction (for E = N)	E = N, n = 2	E = O, n = 1
ΔE_{int}		−37.1	−16.8
ΔE_{Pauli}		97.3	38.7
ΔE_{disp}[i]		−19.4 (14.4 %)	−11.1 (20.0 %)
ΔE_{elstat}[i]		−68.1 (50.6 %)	−25.9 (46.6 %)
ΔE_{orb}[i]		−47.0 (34.9 %)	−18.6 (33.5 %)
$\Delta E_{\text{orb(1)}}$[ii]	*p*(HOMO) → σ*(LUMO)	−31.6 (67.3 %) $\lvert v_1 = 0.55\rvert$	−9.41 (50.7 %) $\lvert v_1 = 0.33\rvert$
$\Delta E_{\text{orb(2)}}$[ii]	*s*(HOMO) → σ*(LUMO and LUMO+1)	−2.79 (5.9 %) $\lvert v_2 = 0.20\rvert$	−3.07 (16.5 %) $\lvert v_2 = 0.16\rvert$
$\Delta E_{\text{orb(rest)}}$[ii]		−12.6 (26.8 %)	−6.07 (32.7 %)

Figure S 197: EDA-NOCV results for $\mathbf{1}^{2+}$/HOMe and $\mathbf{1}^{2+}$/$HNMe_2$ using the digallene and HOMe or $HNMe_2$ as interacting fragments. The shape of the deformation densities $\Delta\rho_{(1)-(2)}$, corresponding to $\Delta E_{\text{orb(1)–(2)}}$, and the fragment orbitals of $\mathbf{1}^{2+}$ and $HNMe_2$ are shown exemplarily (isosurface values: 0.0008 au for $\Delta\rho_{(1)-(2)}$, 0.06 au for the molecular orbitals of $\mathbf{1}^{2+}$, 0.08 au for the HOMO of $HNMe_2$ and 0.05 au for the LUMO(+1) of $HNMe_2$). The eigenvalues $|\boldsymbol{\nu}_1|$ and $|\boldsymbol{\nu}_2|$ give the size of the charge migration in e. The direction of the charge flow of the deformation densities is green → yellow. All calculations were performed at the RI-BP86(D3BJ)/def2-TZVPP level of theory; energy values are given in kcal mol^{-1} instead of kJ mol^{-1} for better comparability with other EDA-NOCV analyses in the literature. [i] Values in parentheses show the contribution to the total attractive interaction. [ii] Values in parentheses show the contribution to the total orbital interaction ΔE_{orb}.

6.2 Optimized Geometries

6.2.1 [{Ga(dcpe)}$_2$]$^{2+}$ (symmetric)

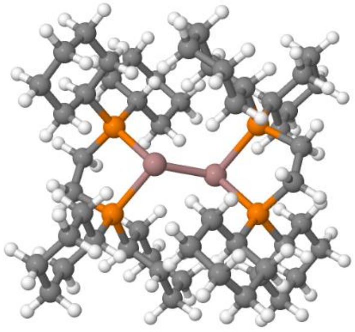

```
Method: (RI-)BP86(D3BJ)/def2-TZVPP
Symmetry: ci

Cartesian coordinates in Ångström:
Ga    0.1584484    0.1534239   -1.1905059
P    -1.3652058   -1.2914867   -2.4666176
P    -1.1570485    1.9737759   -2.1938223
C    -0.4055903   -2.5017094   -3.4750943
H     0.0137175   -3.1910370   -2.7201302
C     0.7774284   -1.8139854   -4.1772143
H     0.3983797   -1.0681914   -4.8945718
H     1.3853137   -1.2638291   -3.4389707
C     1.6369421   -2.8384969   -4.9271827
H     2.4573215   -2.3237125   -5.4458349
H     2.1033284   -3.5184107   -4.1955495
C     0.7964228   -3.6508002   -5.9177769
H     1.4224353   -4.4011099   -6.4187605
H     0.4178687   -2.9827826   -6.7090721
C    -0.3847753   -4.3297025   -5.2182058
H    -0.0074669   -5.0787867   -4.5024945
H    -1.0026757   -4.8750705   -5.9441454
C    -1.2559371   -3.3139886   -4.4651322
H    -2.0724400   -3.8332543   -3.9439414
H    -1.7239068   -2.6284249   -5.1903522
C    -2.6657171   -2.2562157   -1.5764739
H    -3.2235232   -2.7836203   -2.3699495
C    -2.0268698   -3.2921656   -0.6413073
H    -1.3664001   -3.9690668   -1.2015310
H    -1.3954349   -2.7542711    0.0885046
C    -3.0975920   -4.1009116    0.1012702
H    -3.6605394   -4.7053368   -0.6277519
H    -2.6147581   -4.8138831    0.7833757
C    -4.0650254   -3.1897617    0.8612423
H    -4.8399171   -3.7880660    1.3587033
H    -3.5200136   -2.6566134    1.6591413
C    -4.7045200   -2.1628740   -0.0761652
H    -5.3644830   -1.4861432    0.4837940
H    -5.3400993   -2.6824384   -0.8111666
C    -3.6402011   -1.3425909   -0.8155913
H    -4.1313574   -0.6447126   -1.5082629
H    -3.0682788   -0.7371619   -0.0919635
C    -2.2768733   -0.1987435   -3.6519421
H    -1.6511970   -0.1231405   -4.5501529
H    -3.2082265   -0.6974642   -3.9543526
C    -2.5822252    1.1998735   -3.0871682
H    -3.3999249    1.1447597   -2.3567889
H    -2.9131415    1.8716254   -3.8919112
C    -0.2024027    2.9069757   -3.4711712
```

H	-0.9307959	3.5464945	-3.9982197
C	0.8620315	3.7893758	-2.8036160
H	1.5165659	3.1414189	-2.1990898
H	0.3952760	4.5062417	-2.1138338
C	1.7135001	4.5328172	-3.8385279
H	1.0797234	5.2510921	-4.3830650
H	2.4813043	5.1250992	-3.3213488
C	2.3584435	3.5618131	-4.8301376
H	3.0726883	2.9133793	-4.2950171
H	2.9391186	4.1130444	-5.5813525
C	1.2972885	2.6950072	-5.5117519
H	1.7648615	1.9723505	-6.1946067
H	0.6438438	3.3317551	-6.1297452
C	0.4404669	1.9442961	-4.4853007
H	1.0727291	1.2227947	-3.9378114
H	-0.3318476	1.3627282	-5.0068547
C	-1.8515357	3.2195422	-1.0190245
H	-0.9492830	3.6574844	-0.5561574
C	-2.6815558	2.5745549	0.1015426
H	-2.1064295	1.7726876	0.5903233
H	-3.5795471	2.1006479	-0.3278081
C	-3.1125700	3.6326256	1.1234161
H	-3.7177061	3.1615350	1.9106383
H	-2.2125436	4.0385890	1.6175169
C	-3.8870729	4.7735577	0.4560522
H	-4.8394234	4.3816513	0.0629060
H	-4.1497221	5.5412483	1.1962304
C	-3.0832667	5.3970526	-0.6888454
H	-2.1883685	5.8966208	-0.2818060
H	-3.6713709	6.1749670	-1.1940597
C	-2.6396492	4.3436065	-1.7147138
H	-3.5270173	3.9139782	-2.2071143
H	-2.0360973	4.8186529	-2.5001920
Ga	-0.1584484	-0.1534239	1.1905059
P	1.3652058	1.2914867	2.4666176
P	1.1570485	-1.9737759	2.1938223
C	0.4055903	2.5017094	3.4750943
H	-0.0137175	3.1910370	2.7201302
C	-0.7774284	1.8139854	4.1772143
H	-0.3983797	1.0681914	4.8945718
H	-1.3853137	1.2638291	3.4389707
C	-1.6369421	2.8384969	4.9271827
H	-2.4573215	2.3237125	5.4458349
H	-2.1033284	3.5184107	4.1955495
C	-0.7964228	3.6508002	5.9177769
H	-1.4224353	4.4011099	6.4187605
H	-0.4178687	2.9827826	6.7090721
C	0.3847753	4.3297025	5.2182058
H	0.0074669	5.0787867	4.5024945
H	1.0026757	4.8750705	5.9441454
C	1.2559371	3.3139886	4.4651322
H	2.0724400	3.8332543	3.9439414
H	1.7239068	2.6284249	5.1903522
C	2.6657171	2.2562157	1.5764739
H	3.2235232	2.7836203	2.3699495
C	2.0268698	3.2921656	0.6413073
H	1.3664001	3.9690668	1.2015310
H	1.3954349	2.7542711	-0.0885046
C	3.0975920	4.1009116	-0.1012702
H	3.6605394	4.7053368	0.6277519
H	2.6147581	4.8138831	-0.7833757
C	4.0650254	3.1897617	-0.8612423

```
H     4.8399171    3.7880660   -1.3587033
H     3.5200136    2.6566134   -1.6591413
C     4.7045200    2.1628740    0.0761652
H     5.3644830    1.4861432   -0.4837940
H     5.3400993    2.6824384    0.8111666
C     3.6402011    1.3425909    0.8155913
H     4.1313574    0.6447126    1.5082629
H     3.0682788    0.7371619    0.0919635
C     2.2768733    0.1987435    3.6519421
H     1.6511970    0.1231405    4.5501529
H     3.2082265    0.6974642    3.9543526
C     2.5822252   -1.1998735    3.0871682
H     3.3999249   -1.1447597    2.3567889
H     2.9131415   -1.8716254    3.8919112
C     0.2024027   -2.9069757    3.4711712
H     0.9307959   -3.5464945    3.9982197
C    -0.8620315   -3.7893758    2.8036160
H    -1.5165659   -3.1414189    2.1990898
H    -0.3952760   -4.5062417    2.1138338
C    -1.7135001   -4.5328172    3.8385279
H    -1.0797234   -5.2510921    4.3830650
H    -2.4813043   -5.1250992    3.3213488
C    -2.3584435   -3.5618131    4.8301376
H    -3.0726883   -2.9133793    4.2950171
H    -2.9391186   -4.1130444    5.5813525
C    -1.2972885   -2.6950072    5.5117519
H    -1.7648615   -1.9723505    6.1946067
H    -0.6438438   -3.3317551    6.1297452
C    -0.4404669   -1.9442961    4.4853007
H    -1.0727291   -1.2227947    3.9378114
H     0.3318476   -1.3627282    5.0068547
C     1.8515357   -3.2195422    1.0190245
H     0.9492830   -3.6574844    0.5561574
C     2.6815558   -2.5745549   -0.1015426
H     2.1064295   -1.7726876   -0.5903233
H     3.5795471   -2.1006479    0.3278081
C     3.1125700   -3.6326256   -1.1234161
H     3.7177061   -3.1615350   -1.9106383
H     2.2125436   -4.0385890   -1.6175169
C     3.8870729   -4.7735577   -0.4560522
H     4.8394234   -4.3816513   -0.0629060
H     4.1497221   -5.5412483   -1.1962304
C     3.0832667   -5.3970526    0.6888454
H     2.1883685   -5.8966208    0.2818060
H     3.6713709   -6.1749670    1.1940597
C     2.6396492   -4.3436065    1.7147138
H     3.5270173   -3.9139782    2.2071143
H     2.0360973   -4.8186529    2.5001920

SCF energy GEOOPT =  -7256.171633101 H
ZPE =   3596. kJ/mol
FREEH energy =  3772.42 kJ/mol
FREEH entropy =   1.53132 kJ/mol/K

$vibrational spectrum
#  mode     symmetry     wave number   IR intensity    selection rules
#                         cm**(-1)        km/mol          IR     RAMAN
     1                      -0.00         0.00000         -       -
     2                      -0.00         0.00000         -       -
     3                      -0.00         0.00000         -       -
     4                      -0.00         0.00000         -       -
     5                      -0.00         0.00000         -       -
```

6		0.00	0.00000	-	-
7	au	10.92	0.02834	YES	NO
8	au	19.05	0.11990	YES	NO
9	ag	23.26	0.00000	NO	YES
10	au	27.23	0.30003	YES	NO
11	ag	29.95	0.00000	NO	YES
12	au	31.16	0.00344	YES	NO
13	ag	32.42	0.00000	NO	YES
14	au	35.04	0.11615	YES	NO
15	ag	38.82	0.00000	NO	YES
16	au	39.84	0.59894	YES	NO
17	ag	43.19	0.00000	NO	YES
18	au	45.36	0.03676	YES	NO
19	ag	46.46	0.00000	NO	YES
20	ag	52.19	0.00000	NO	YES
21	au	52.20	0.19501	YES	NO
22	ag	54.53	0.00000	NO	YES
23	au	56.54	0.55763	YES	NO
24	ag	62.69	0.00000	NO	YES
25	au	63.88	0.34385	YES	NO
26	ag	65.74	0.00000	NO	YES
27	au	70.23	0.76885	YES	NO
28	ag	71.99	0.00000	NO	YES
29	au	72.25	0.32231	YES	NO
30	au	74.90	0.57779	YES	NO
31	au	80.79	3.14781	YES	NO
32	ag	81.29	0.00000	NO	YES
33	ag	87.50	0.00000	NO	YES
34	au	87.57	0.23786	YES	NO
35	ag	89.44	0.00000	NO	YES
36	ag	122.40	0.00000	NO	YES
37	ag	123.60	0.00000	NO	YES
38	au	124.91	0.84170	YES	NO
39	au	132.09	0.45545	YES	NO
40	ag	141.86	0.00000	NO	YES
41	ag	143.22	0.00000	NO	YES
42	au	144.12	4.33625	YES	NO
43	ag	146.32	0.00000	NO	YES
44	au	147.91	6.62281	YES	NO
45	au	148.81	0.65115	YES	NO
46	ag	166.04	0.00000	NO	YES
47	au	179.11	0.78762	YES	NO
48	ag	179.57	0.00000	NO	YES
49	au	183.48	1.41280	YES	NO
50	ag	191.57	0.00000	NO	YES
51	au	195.42	5.29589	YES	NO
52	ag	200.26	0.00000	NO	YES
53	au	207.44	4.94899	YES	NO
54	ag	215.88	0.00000	NO	YES
55	au	216.74	0.22183	YES	NO
56	ag	217.07	0.00000	NO	YES
57	au	224.30	4.17799	YES	NO
58	ag	226.65	0.00000	NO	YES
59	au	228.02	0.46864	YES	NO
60	ag	235.18	0.00000	NO	YES
61	au	236.39	0.55059	YES	NO
62	au	238.71	0.88127	YES	NO
63	ag	238.87	0.00000	NO	YES
64	ag	244.15	0.00000	NO	YES
65	au	245.14	1.03950	YES	NO
66	ag	256.15	0.00000	NO	YES
67	au	273.65	1.91869	YES	NO

68	ag	276.04	0.00000	NO	YES
69	au	284.53	1.33108	YES	NO
70	ag	290.38	0.00000	NO	YES
71	au	306.25	0.96603	YES	NO
72	ag	307.29	0.00000	NO	YES
73	ag	314.10	0.00000	NO	YES
74	au	315.57	0.32591	YES	NO
75	au	329.88	1.75673	YES	NO
76	ag	330.40	0.00000	NO	YES
77	ag	333.67	0.00000	NO	YES
78	au	335.00	0.05537	YES	NO
79	au	341.51	5.85328	YES	NO
80	ag	343.01	0.00000	NO	YES
81	au	367.31	14.62782	YES	NO
82	ag	369.83	0.00000	NO	YES
83	ag	374.37	0.00000	NO	YES
84	au	375.80	1.16583	YES	NO
85	au	393.92	5.18499	YES	NO
86	ag	396.84	0.00000	NO	YES
87	ag	414.68	0.00000	NO	YES
88	au	415.23	3.60602	YES	NO
89	ag	423.10	0.00000	NO	YES
90	au	423.31	5.46848	YES	NO
91	au	425.89	4.09585	YES	NO
92	ag	426.38	0.00000	NO	YES
93	au	430.08	0.45619	YES	NO
94	ag	430.13	0.00000	NO	YES
95	au	431.18	0.03908	YES	NO
96	ag	431.49	0.00000	NO	YES
97	ag	432.06	0.00000	NO	YES
98	au	432.72	1.74943	YES	NO
99	au	434.81	0.95999	YES	NO
100	ag	434.82	0.00000	NO	YES
101	au	444.37	13.81677	YES	NO
102	ag	446.52	0.00000	NO	YES
103	ag	465.70	0.00000	NO	YES
104	au	466.28	7.59054	YES	NO
105	au	490.95	6.25621	YES	NO
106	ag	491.56	0.00000	NO	YES
107	au	493.95	10.03835	YES	NO
108	ag	494.12	0.00000	NO	YES
109	au	502.40	3.70124	YES	NO
110	ag	503.34	0.00000	NO	YES
111	au	516.65	27.94310	YES	NO
112	ag	516.84	0.00000	NO	YES
113	au	623.10	42.73833	YES	NO
114	ag	623.88	0.00000	NO	YES
115	au	639.50	24.26470	YES	NO
116	ag	639.97	0.00000	NO	YES
117	au	713.20	1.68828	YES	NO
118	ag	713.57	0.00000	NO	YES
119	au	721.58	5.54511	YES	NO
120	ag	722.01	0.00000	NO	YES
121	ag	732.56	0.00000	NO	YES
122	au	732.90	16.20991	YES	NO
123	ag	734.74	0.00000	NO	YES
124	au	735.00	8.60167	YES	NO
125	au	773.38	7.95127	YES	NO
126	ag	773.53	0.00000	NO	YES
127	ag	773.86	0.00000	NO	YES
128	au	774.01	3.17722	YES	NO
129	ag	774.86	0.00000	NO	YES

130	au	774.89	1.40374	YES	NO
131	au	775.47	2.43028	YES	NO
132	ag	775.62	0.00000	NO	YES
133	au	776.60	28.46908	YES	NO
134	ag	776.95	0.00000	NO	YES
135	au	809.92	0.17957	YES	NO
136	ag	810.27	0.00000	NO	YES
137	ag	811.30	0.00000	NO	YES
138	au	811.40	4.20853	YES	NO
139	au	812.26	4.75511	YES	NO
140	ag	812.51	0.00000	NO	YES
141	ag	814.14	0.00000	NO	YES
142	au	814.48	15.99134	YES	NO
143	ag	832.51	0.00000	NO	YES
144	au	833.13	3.84603	YES	NO
145	ag	836.07	0.00000	NO	YES
146	au	836.43	10.18262	YES	NO
147	au	836.96	5.30188	YES	NO
148	ag	837.01	0.00000	NO	YES
149	au	839.42	19.73473	YES	NO
150	ag	840.08	0.00000	NO	YES
151	au	850.21	36.73168	YES	NO
152	ag	852.64	0.00000	NO	YES
153	au	876.19	5.13054	YES	NO
154	ag	876.37	0.00000	NO	YES
155	au	876.74	3.12768	YES	NO
156	ag	876.76	0.00000	NO	YES
157	ag	876.93	0.00000	NO	YES
158	au	877.24	11.18007	YES	NO
159	au	877.97	8.07199	YES	NO
160	ag	878.32	0.00000	NO	YES
161	au	878.94	5.78210	YES	NO
162	ag	878.95	0.00000	NO	YES
163	au	879.90	8.96516	YES	NO
164	ag	880.35	0.00000	NO	YES
165	ag	882.20	0.00000	NO	YES
166	au	883.60	1.09277	YES	NO
167	au	885.11	4.89421	YES	NO
168	ag	886.60	0.00000	NO	YES
169	ag	903.16	0.00000	NO	YES
170	au	903.70	8.47169	YES	NO
171	au	904.02	5.11335	YES	NO
172	ag	904.74	0.00000	NO	YES
173	ag	909.39	0.00000	NO	YES
174	au	909.90	6.07043	YES	NO
175	au	910.90	1.41352	YES	NO
176	ag	911.08	0.00000	NO	YES
177	au	982.84	0.86840	YES	NO
178	ag	982.93	0.00000	NO	YES
179	ag	987.57	0.00000	NO	YES
180	au	987.72	15.41783	YES	NO
181	ag	989.22	0.00000	NO	YES
182	au	989.29	17.30737	YES	NO
183	ag	989.77	0.00000	NO	YES
184	au	990.28	19.90490	YES	NO
185	au	991.57	15.27192	YES	NO
186	ag	992.41	0.00000	NO	YES
187	ag	1015.67	0.00000	NO	YES
188	au	1015.85	5.89493	YES	NO
189	au	1016.31	1.04427	YES	NO
190	ag	1016.59	0.00000	NO	YES
191	au	1020.35	1.42697	YES	NO

192	ag	1020.35	0.00000	NO	YES
193	ag	1022.43	0.00000	NO	YES
194	au	1022.45	0.97360	YES	NO
195	au	1031.60	1.32464	YES	NO
196	ag	1031.80	0.00000	NO	YES
197	ag	1032.53	0.00000	NO	YES
198	au	1032.84	5.56758	YES	NO
199	ag	1039.07	0.00000	NO	YES
200	au	1039.10	6.77623	YES	NO
201	ag	1039.84	0.00000	NO	YES
202	au	1039.99	0.93500	YES	NO
203	au	1059.05	7.48427	YES	NO
204	ag	1059.16	0.00000	NO	YES
205	ag	1059.33	0.00000	NO	YES
206	au	1059.39	2.76014	YES	NO
207	au	1064.34	4.56036	YES	NO
208	ag	1066.25	0.00000	NO	YES
209	ag	1068.98	0.00000	NO	YES
210	au	1070.18	0.28314	YES	NO
211	ag	1071.70	0.00000	NO	YES
212	au	1071.75	0.11032	YES	NO
213	au	1071.84	0.05899	YES	NO
214	ag	1071.85	0.00000	NO	YES
215	au	1074.64	1.49684	YES	NO
216	ag	1074.70	0.00000	NO	YES
217	ag	1075.43	0.00000	NO	YES
218	au	1076.36	0.38739	YES	NO
219	au	1078.90	7.78022	YES	NO
220	ag	1079.64	0.00000	NO	YES
221	ag	1091.79	0.00000	NO	YES
222	au	1091.82	23.95138	YES	NO
223	au	1094.46	14.53236	YES	NO
224	ag	1094.60	0.00000	NO	YES
225	au	1100.84	0.56918	YES	NO
226	ag	1101.07	0.00000	NO	YES
227	ag	1105.68	0.00000	NO	YES
228	au	1106.58	20.88214	YES	NO
229	au	1126.37	16.46788	YES	NO
230	ag	1129.00	0.00000	NO	YES
231	au	1160.24	13.52143	YES	NO
232	ag	1160.58	0.00000	NO	YES
233	ag	1161.08	0.00000	NO	YES
234	au	1161.42	6.73747	YES	NO
235	ag	1169.02	0.00000	NO	YES
236	au	1169.64	18.15012	YES	NO
237	au	1171.57	19.69385	YES	NO
238	ag	1171.64	0.00000	NO	YES
239	au	1174.89	18.44753	YES	NO
240	ag	1175.06	0.00000	NO	YES
241	au	1177.91	20.65658	YES	NO
242	ag	1178.63	0.00000	NO	YES
243	au	1188.58	3.18668	YES	NO
244	ag	1189.07	0.00000	NO	YES
245	ag	1194.84	0.00000	NO	YES
246	au	1195.39	4.27526	YES	NO
247	au	1237.41	0.77382	YES	NO
248	ag	1237.64	0.00000	NO	YES
249	au	1244.37	0.63581	YES	NO
250	ag	1245.01	0.00000	NO	YES
251	ag	1245.28	0.00000	NO	YES
252	au	1245.63	1.39429	YES	NO
253	au	1245.93	0.29623	YES	NO

254	ag	1246.29	0.00000	NO	YES
255	au	1249.06	0.66203	YES	NO
256	ag	1249.10	0.00000	NO	YES
257	ag	1253.52	0.00000	NO	YES
258	au	1254.15	2.01793	YES	NO
259	ag	1255.06	0.00000	NO	YES
260	au	1255.79	13.01262	YES	NO
261	au	1257.13	12.93416	YES	NO
262	ag	1257.36	0.00000	NO	YES
263	au	1257.48	5.22470	YES	NO
264	ag	1257.68	0.00000	NO	YES
265	au	1258.20	1.14098	YES	NO
266	ag	1258.39	0.00000	NO	YES
267	au	1259.46	8.47516	YES	NO
268	ag	1259.73	0.00000	NO	YES
269	ag	1262.09	0.00000	NO	YES
270	au	1263.18	1.34468	YES	NO
271	au	1264.65	5.95954	YES	NO
272	ag	1265.47	0.00000	NO	YES
273	au	1266.21	5.34130	YES	NO
274	ag	1267.18	0.00000	NO	YES
275	ag	1280.78	0.00000	NO	YES
276	au	1280.82	2.51856	YES	NO
277	ag	1283.77	0.00000	NO	YES
278	au	1283.84	5.02350	YES	NO
279	au	1288.61	3.27386	YES	NO
280	ag	1289.23	0.00000	NO	YES
281	ag	1290.32	0.00000	NO	YES
282	au	1290.82	8.98306	YES	NO
283	ag	1309.11	0.00000	NO	YES
284	au	1309.17	1.57998	YES	NO
285	au	1309.98	4.48452	YES	NO
286	ag	1310.23	0.00000	NO	YES
287	au	1314.09	2.10566	YES	NO
288	ag	1314.40	0.00000	NO	YES
289	ag	1315.02	0.00000	NO	YES
290	au	1315.35	6.06773	YES	NO
291	ag	1321.62	0.00000	NO	YES
292	au	1322.07	0.53272	YES	NO
293	ag	1323.10	0.00000	NO	YES
294	au	1323.13	0.57774	YES	NO
295	ag	1323.33	0.00000	NO	YES
296	au	1323.77	10.72592	YES	NO
297	au	1324.41	8.28567	YES	NO
298	ag	1324.44	0.00000	NO	YES
299	au	1324.81	1.36301	YES	NO
300	ag	1325.57	0.00000	NO	YES
301	au	1325.72	2.14091	YES	NO
302	ag	1326.22	0.00000	NO	YES
303	ag	1326.97	0.00000	NO	YES
304	au	1327.11	11.45636	YES	NO
305	au	1327.71	2.69993	YES	NO
306	ag	1328.00	0.00000	NO	YES
307	au	1336.06	0.39128	YES	NO
308	ag	1336.18	0.00000	NO	YES
309	au	1337.08	1.04546	YES	NO
310	ag	1337.09	0.00000	NO	YES
311	au	1338.13	2.05205	YES	NO
312	ag	1338.19	0.00000	NO	YES
313	ag	1339.15	0.00000	NO	YES
314	au	1339.16	1.44737	YES	NO
315	au	1345.57	4.31126	YES	NO

316	ag	1345.59	0.00000	NO	YES
317	ag	1346.12	0.00000	NO	YES
318	au	1346.17	2.88933	YES	NO
319	ag	1348.73	0.00000	NO	YES
320	au	1348.89	2.34969	YES	NO
321	au	1350.18	0.97879	YES	NO
322	ag	1350.40	0.00000	NO	YES
323	au	1400.47	15.36926	YES	NO
324	ag	1400.52	0.00000	NO	YES
325	au	1405.73	14.25230	YES	NO
326	ag	1405.83	0.00000	NO	YES
327	au	1423.24	10.53652	YES	NO
328	ag	1423.42	0.00000	NO	YES
329	ag	1428.22	0.00000	NO	YES
330	au	1428.76	12.54397	YES	NO
331	ag	1430.80	0.00000	NO	YES
332	au	1431.17	3.73989	YES	NO
333	au	1433.32	1.58401	YES	NO
334	ag	1433.44	0.00000	NO	YES
335	ag	1434.55	0.00000	NO	YES
336	au	1435.02	12.40472	YES	NO
337	au	1435.08	7.17288	YES	NO
338	ag	1435.40	0.00000	NO	YES
339	au	1436.26	2.04622	YES	NO
340	ag	1437.29	0.00000	NO	YES
341	ag	1437.76	0.00000	NO	YES
342	au	1437.81	15.76739	YES	NO
343	au	1438.40	4.37829	YES	NO
344	ag	1438.47	0.00000	NO	YES
345	au	1438.59	3.80738	YES	NO
346	ag	1438.67	0.00000	NO	YES
347	ag	1439.19	0.00000	NO	YES
348	au	1439.36	11.93457	YES	NO
349	au	1440.13	3.51942	YES	NO
350	ag	1440.13	0.00000	NO	YES
351	au	1442.03	70.47082	YES	NO
352	ag	1442.46	0.00000	NO	YES
353	ag	1442.93	0.00000	NO	YES
354	au	1443.36	11.79264	YES	NO
355	ag	1444.42	0.00000	NO	YES
356	au	1444.64	15.16248	YES	NO
357	au	1446.72	38.19992	YES	NO
358	ag	1446.78	0.00000	NO	YES
359	ag	1452.54	0.00000	NO	YES
360	au	1452.86	3.38830	YES	NO
361	au	1453.24	7.52717	YES	NO
362	ag	1454.27	0.00000	NO	YES
363	ag	1454.59	0.00000	NO	YES
364	au	1454.74	1.48202	YES	NO
365	ag	1455.77	0.00000	NO	YES
366	au	1456.07	1.73107	YES	NO
367	ag	2919.84	0.00000	NO	YES
368	au	2919.86	26.35208	YES	NO
369	ag	2921.46	0.00000	NO	YES
370	au	2921.51	74.21241	YES	NO
371	au	2925.79	75.48958	YES	NO
372	ag	2926.39	0.00000	NO	YES
373	au	2927.35	9.09713	YES	NO
374	ag	2927.39	0.00000	NO	YES
375	ag	2930.28	0.00000	NO	YES
376	au	2930.29	30.14789	YES	NO
377	ag	2931.85	0.00000	NO	YES

378	au	2931.89	20.11262	YES	NO
379	ag	2933.08	0.00000	NO	YES
380	au	2933.25	10.85575	YES	NO
381	ag	2936.41	0.00000	NO	YES
382	au	2936.56	24.71459	YES	NO
383	ag	2937.30	0.00000	NO	YES
384	au	2937.31	25.21273	YES	NO
385	ag	2942.21	0.00000	NO	YES
386	au	2942.22	18.08738	YES	NO
387	au	2945.02	14.94226	YES	NO
388	ag	2945.04	0.00000	NO	YES
389	ag	2945.33	0.00000	NO	YES
390	au	2945.39	23.75846	YES	NO
391	au	2945.61	5.14390	YES	NO
392	ag	2945.67	0.00000	NO	YES
393	au	2946.21	2.94310	YES	NO
394	ag	2946.24	0.00000	NO	YES
395	au	2948.69	22.30099	YES	NO
396	ag	2948.70	0.00000	NO	YES
397	au	2955.88	3.73836	YES	NO
398	ag	2955.90	0.00000	NO	YES
399	au	2956.11	7.28972	YES	NO
400	ag	2956.11	0.00000	NO	YES
401	au	2956.97	21.45592	YES	NO
402	ag	2956.98	0.00000	NO	YES
403	au	2959.26	21.26361	YES	NO
404	ag	2959.26	0.00000	NO	YES
405	ag	2959.70	0.00000	NO	YES
406	au	2959.74	10.13724	YES	NO
407	au	2959.84	69.00169	YES	NO
408	ag	2959.87	0.00000	NO	YES
409	ag	2960.80	0.00000	NO	YES
410	au	2960.83	36.03664	YES	NO
411	au	2962.98	99.88988	YES	NO
412	ag	2963.08	0.00000	NO	YES
413	ag	2963.30	0.00000	NO	YES
414	au	2963.31	85.60392	YES	NO
415	ag	2977.08	0.00000	NO	YES
416	au	2977.16	106.69336	YES	NO
417	ag	2978.21	0.00000	NO	YES
418	au	2978.32	29.29696	YES	NO
419	au	2982.42	13.86777	YES	NO
420	ag	2982.43	0.00000	NO	YES
421	au	2988.48	9.64088	YES	NO
422	ag	2988.48	0.00000	NO	YES
423	ag	2998.26	0.00000	NO	YES
424	au	2998.29	43.23556	YES	NO
425	ag	3002.08	0.00000	NO	YES
426	au	3002.16	47.67971	YES	NO
427	au	3003.92	28.02357	YES	NO
428	ag	3003.93	0.00000	NO	YES
429	ag	3004.82	0.00000	NO	YES
430	au	3004.90	54.64863	YES	NO
431	ag	3005.78	0.00000	NO	YES
432	au	3005.81	96.75875	YES	NO
433	au	3006.38	10.01856	YES	NO
434	ag	3006.43	0.00000	NO	YES
435	ag	3009.93	0.00000	NO	YES
436	au	3009.95	43.64844	YES	NO
437	au	3010.06	8.90268	YES	NO
438	ag	3010.08	0.00000	NO	YES
439	ag	3010.90	0.00000	NO	YES

440	au	3010.91	15.39461	YES	NO
441	ag	3011.52	0.00000	NO	YES
442	au	3011.56	44.16445	YES	NO
443	au	3011.98	85.84790	YES	NO
444	ag	3012.00	0.00000	NO	YES
445	ag	3012.32	0.00000	NO	YES
446	au	3012.35	43.77723	YES	NO
447	au	3015.21	50.55656	YES	NO
448	ag	3015.23	0.00000	NO	YES
449	au	3015.59	61.33338	YES	NO
450	ag	3015.64	0.00000	NO	YES
451	au	3016.03	67.42986	YES	NO
452	ag	3016.06	0.00000	NO	YES
453	ag	3017.04	0.00000	NO	YES
454	au	3017.06	36.72299	YES	NO
455	ag	3018.37	0.00000	NO	YES
456	au	3018.38	42.26232	YES	NO
457	au	3019.44	67.69109	YES	NO
458	ag	3019.49	0.00000	NO	YES
459	au	3029.42	0.67149	YES	NO
460	ag	3029.43	0.00000	NO	YES
461	au	3044.33	2.35926	YES	NO
462	ag	3044.34	0.00000	NO	YES

$end

Double hybrid single point energy = -7249.051030759942 H
COSMO energy + OC correction = -7256.3196247836 H (in *o*DFB)
COSMO energy + OC correction = -7256.326891604 H (in 3FB)

6.2.2 [{Ga(dcpe)}$_2$]$^{2+}$ (asymmetric)

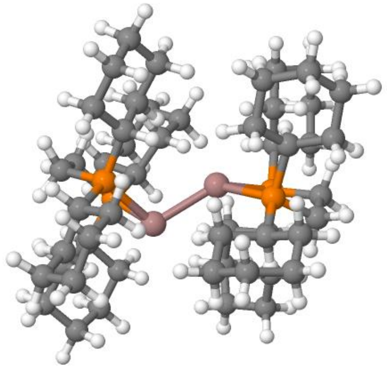

Method: (RI-)BP86(D3BJ)/def2-TZVPP
Symmetry: c1

Cartesian coordinates in Ångström:

Ga	-2.1400311	17.4157585	-2.1819865
Ga	-4.5460621	17.2346598	-1.9146004
P	-1.6632199	18.9426330	-4.0809401
P	-6.0110963	19.0210663	-1.0697684
P	-1.6140671	15.6725487	-3.8598852
P	-5.6234988	15.9465445	-0.1330542
C	-5.1965751	20.7684785	-5.4294914
H	-5.3764832	21.4337763	-4.5668411
H	-6.1681912	20.3301913	-5.6972823
C	-0.3916335	14.4182724	-3.2756488
H	-0.0557914	13.8667918	-4.1707010
C	-4.5907092	14.8986651	0.9777028

H	-4.1660664	15.6514304	1.6679367
C	-4.0089464	15.6744279	-5.3422317
H	-3.4969453	16.0635864	-6.2376481
H	-4.2795328	16.5368188	-4.7148302
C	-7.1233732	19.5967900	-2.4198998
H	-6.4522031	20.1110999	-3.1268611
C	-7.1248073	18.2939274	0.2223841
H	-7.4507682	19.0865096	0.9097815
H	-8.0232661	17.9358787	-0.2987108
C	-3.2664390	22.1716644	-6.2498909
H	-2.8585507	22.7315324	-7.1021727
H	-3.3610124	22.8903097	-5.4191166
C	-2.2824049	21.0650539	-5.8464520
H	-1.3132297	21.5074148	-5.5796826
H	-2.1074264	20.4046353	-6.7106081
C	-4.2250528	19.6574012	-5.0141282
H	-4.1330709	18.9361558	-5.8422565
H	-4.6149555	19.0887350	-4.1542362
C	1.8311912	14.0575184	-2.1127721
H	2.2519512	13.5118704	-2.9728276
H	2.6723555	14.5702784	-1.6266851
C	-8.1576587	14.5547093	-0.1931210
H	-8.5953271	15.3906443	0.3713985
H	-7.8310659	13.8093784	0.5453320
C	-5.1658278	20.4798264	-0.3288040
H	-5.9543750	21.2014050	-0.0549506
C	-0.4188468	20.7996692	-2.4538526
H	-1.1713188	21.5425712	-2.7532516
H	-0.8590420	20.2151593	-1.6257994
C	0.8452317	21.5089904	-1.9559834
H	1.2204274	22.1782025	-2.7470105
H	0.5905929	22.1494009	-1.1001168
C	-7.7311198	18.3829296	-3.1477303
H	-6.9264300	17.7171623	-3.5061168
H	-8.3387022	17.7939098	-2.4398235
C	-4.6439829	21.5990958	-6.5913954
H	-4.5648276	20.9621474	-7.4876627
H	-5.3416991	22.4083270	-6.8450063
C	-8.2039444	20.5884335	-1.9596638
H	-7.7439412	21.4684974	-1.4884716
H	-8.8404086	20.1126962	-1.1959635
C	-4.2329740	21.1132941	-1.3735998
H	-4.8013122	21.4224105	-2.2627284
H	-3.5110926	20.3438956	-1.7011842
C	-2.6560209	13.5482807	-5.4597232
H	-1.9734203	12.8715113	-4.9301123
H	-2.1186626	13.9105876	-6.3512891
C	2.2562850	19.5768782	-2.7500699
H	2.6872497	20.1648867	-3.5765987
H	3.0159487	18.8362460	-2.4647185
C	-3.6200649	21.2783280	1.5127516
H	-4.3406199	22.0335184	1.8655240
H	-3.0485033	20.9566868	2.3938774
C	-9.6998104	19.8110695	-3.8520693
H	-10.3016302	20.1402608	-4.7094709
H	-10.3932646	19.3016655	-3.1624948
C	-5.3656494	13.8644368	1.8133298
H	-6.1889021	14.3417514	2.3631008

H	-5.8168142	13.1208006	1.1386271
C	1.1751165	13.0650604	-1.1486260
H	1.9031356	12.3074018	-0.8299335
H	0.8580871	13.5990070	-0.2367557
C	-8.6244231	18.8224168	-4.3138629
H	-7.9982495	19.2965747	-5.0864541
H	-9.0828364	17.9394403	-4.7799424
C	1.9360184	20.5048607	-1.5756098
H	1.5964334	19.9033012	-0.7156242
H	2.8423670	21.0323433	-1.2503889
C	-2.6925043	21.9054780	0.4682023
H	-2.1783577	22.7780182	0.8923478
H	-1.9089851	21.1785786	0.1963142
C	-6.3549659	13.8844282	-1.8636954
H	-5.9520761	13.1113129	-1.1914092
H	-5.5087380	14.2590248	-2.4628106
C	-4.3871817	20.0807020	0.9372437
H	-3.6748399	19.2768776	0.6794076
H	-5.0712589	19.6789026	1.6982539
C	-6.9552791	15.0274457	-1.0245302
H	-7.3252054	15.7883322	-1.7394502
C	-3.4695963	22.3063617	-0.7884630
C	-8.6369475	12.7954972	-1.9581457
H	-9.4065718	12.3896954	-2.6280147
H	-8.3333083	11.9685872	-1.2954832
C	-9.2185982	13.9343642	-1.1140847
H	-10.0626024	13.5744840	-0.5105740
H	-9.6203403	14.7164914	-1.7800985
C	-1.0492210	13.4381985	-2.2919280
H	-1.9161868	12.9408688	-2.7483733
H	-1.4276175	14.0159508	-1.4352853
C	-4.4177737	13.1500474	2.7867202
H	-4.0397098	13.8776446	3.5233576
H	-4.9787064	12.3945685	3.3532548
C	-5.2660924	14.9147786	-5.7785587
H	-5.9316979	15.5883049	-6.3367869
H	-5.8199277	14.5983752	-4.8787311
C	-3.2394944	12.5061833	2.0498102
H	-3.6140581	11.7029922	1.3935709
H	-2.5590478	12.0293805	2.7677258
C	-3.4140588	14.2566676	0.2270626
H	-3.7936597	13.5396397	-0.5174389
H	-2.8676637	15.0366854	-0.3269356
C	-9.0782433	21.0195237	-3.1456844
H	-9.8604037	21.7053769	-2.7938777
H	-8.4608502	21.5873361	-3.8608553
C	-3.9125368	12.7803022	-5.8991068
H	-3.6217983	11.9377685	-6.5408906
H	-4.3899962	12.3432266	-5.0060112
C	-7.4247483	13.2649977	-2.7695793
H	-6.9917792	12.4293317	-3.3369752
H	-7.7468637	14.0162338	-3.5102714
C	-4.9121461	13.6868383	-6.6231110
H	-5.8198407	13.1233644	-6.8781822
H	-4.4745877	14.0197078	-7.5785319
C	-0.0422863	12.3959458	-1.7920151
H	-0.5358706	11.7198855	-1.0793822
H	0.2829493	11.7699352	-2.6387057

```
C    -2.4810579   13.5371166    1.2076273
H    -2.0146935   14.2840904    1.8708815
H    -1.6620324   13.0547242    0.6564453
C    -0.1167236   19.8424688   -3.6198206
H     0.2022710   20.4261396   -4.4990009
C     1.0012244   18.8525143   -3.2506245
H     1.2551666   18.2235328   -4.1154483
H     0.6323137   18.1764832   -2.4573726
C     0.8249104   15.0976860   -2.6217495
H     1.3209659   15.7808495   -3.3251397
H     0.4720543   15.7139145   -1.7758713
C    -0.7622388   16.5134592   -5.2720334
H     0.3005441   16.5467460   -5.0027341
H    -0.8424538   15.8734469   -6.1620186
C    -1.2802671   17.9248138   -5.5801269
H    -2.2217611   17.8682858   -6.1420251
H    -0.5610084   18.4649697   -6.2116665
C    -3.0514304   14.7453703   -4.5793520
H    -3.5821631   14.3692372   -3.6860414
C    -2.8454304   20.2393408   -4.6745010
H    -2.9663055   20.9082800   -3.8074152
C    -6.4598837   17.1463493    0.9919752
H    -7.1932291   16.6265419    1.6242413
H    -5.6723263   17.5279323    1.6559347
H    -4.1835599   23.1084638   -0.5425269
H    -2.7916944   22.7191057   -1.5490877

SCF energy GEOOPT =  -7256.165972908 H
ZPE =   3596. kJ/mol
FREEH energy =  3772.30 kJ/mol
FREEH entropy =   1.54579 kJ/mol/K

$vibrational spectrum
#  mode     symmetry     wave number   IR intensity    selection rules
#                         cm**(-1)        km/mol          IR     RAMAN
     1                        -0.00         0.00000         -        -
     2                        -0.00         0.00000         -        -
     3                        -0.00         0.00000         -        -
     4                         0.00         0.00000         -        -
     5                         0.00         0.00000         -        -
     6                         0.00         0.00000         -        -
     7         a               5.60         0.02921        YES      YES
     8         a              14.49         0.09237        YES      YES
     9         a              18.34         0.04973        YES      YES
    10         a              21.38         0.00934        YES      YES
    11         a              24.72         0.18559        YES      YES
    12         a              31.02         0.02820        YES      YES
    13         a              32.99         0.01686        YES      YES
    14         a              37.52         0.54758        YES      YES
    15         a              38.20         0.15959        YES      YES
    16         a              40.60         0.01272        YES      YES
    17         a              44.19         0.05895        YES      YES
    18         a              45.66         0.07682        YES      YES
    19         a              48.35         0.36329        YES      YES
    20         a              50.21         0.40208        YES      YES
    21         a              54.17         0.12647        YES      YES
    22         a              55.24         0.02185        YES      YES
    23         a              56.88         0.23762        YES      YES
```

24	a	59.30	0.25587	YES	YES
25	a	62.06	0.70547	YES	YES
26	a	66.43	0.01693	YES	YES
27	a	68.31	0.53916	YES	YES
28	a	70.30	0.18098	YES	YES
29	a	70.82	0.21743	YES	YES
30	a	74.78	0.56183	YES	YES
31	a	75.26	1.11375	YES	YES
32	a	79.80	0.11096	YES	YES
33	a	83.53	0.02828	YES	YES
34	a	90.88	0.72598	YES	YES
35	a	93.76	0.24452	YES	YES
36	a	115.11	0.25430	YES	YES
37	a	118.54	0.14634	YES	YES
38	a	126.71	0.64539	YES	YES
39	a	129.74	0.63618	YES	YES
40	a	134.60	0.70480	YES	YES
41	a	138.19	0.38911	YES	YES
42	a	140.39	1.71561	YES	YES
43	a	143.44	4.06558	YES	YES
44	a	150.66	0.47706	YES	YES
45	a	155.70	2.19328	YES	YES
46	a	166.59	0.28568	YES	YES
47	a	176.91	1.16162	YES	YES
48	a	179.03	3.79459	YES	YES
49	a	184.19	1.58206	YES	YES
50	a	193.58	2.16127	YES	YES
51	a	196.46	4.36579	YES	YES
52	a	209.26	0.93483	YES	YES
53	a	210.63	0.09032	YES	YES
54	a	215.98	0.19627	YES	YES
55	a	220.80	0.23868	YES	YES
56	a	223.90	0.03107	YES	YES
57	a	227.56	0.60939	YES	YES
58	a	228.75	1.06302	YES	YES
59	a	229.14	0.28396	YES	YES
60	a	233.69	0.43944	YES	YES
61	a	235.62	0.43678	YES	YES
62	a	238.03	0.47443	YES	YES
63	a	240.15	0.49777	YES	YES
64	a	246.43	0.29210	YES	YES
65	a	251.03	0.75064	YES	YES
66	a	258.23	0.91817	YES	YES
67	a	267.43	0.44555	YES	YES
68	a	278.76	0.08445	YES	YES
69	a	281.44	0.75155	YES	YES
70	a	288.00	1.83350	YES	YES
71	a	296.79	0.93188	YES	YES
72	a	305.22	0.49218	YES	YES
73	a	310.22	0.07571	YES	YES
74	a	313.49	1.44016	YES	YES
75	a	328.62	0.91241	YES	YES
76	a	329.81	0.12971	YES	YES
77	a	331.70	1.32254	YES	YES
78	a	337.31	1.69886	YES	YES
79	a	337.95	0.55834	YES	YES
80	a	340.05	1.90703	YES	YES
81	a	363.40	9.12894	YES	YES

82	a	376.74	3.22200	YES	YES
83	a	379.32	0.76792	YES	YES
84	a	379.90	0.77747	YES	YES
85	a	389.82	2.36932	YES	YES
86	a	393.68	2.82144	YES	YES
87	a	407.96	0.38918	YES	YES
88	a	416.71	2.44917	YES	YES
89	a	420.58	1.01770	YES	YES
90	a	422.53	4.32763	YES	YES
91	a	427.58	2.16925	YES	YES
92	a	429.00	0.65612	YES	YES
93	a	429.95	0.19550	YES	YES
94	a	430.66	0.01708	YES	YES
95	a	431.02	0.30943	YES	YES
96	a	432.67	0.67428	YES	YES
97	a	432.81	0.60428	YES	YES
98	a	433.82	0.15674	YES	YES
99	a	436.12	3.72126	YES	YES
100	a	436.66	0.11765	YES	YES
101	a	444.56	3.04352	YES	YES
102	a	455.79	7.20500	YES	YES
103	a	461.94	4.84136	YES	YES
104	a	467.55	5.59207	YES	YES
105	a	491.98	7.41526	YES	YES
106	a	492.20	0.10203	YES	YES
107	a	494.40	5.33297	YES	YES
108	a	495.79	2.38117	YES	YES
109	a	501.32	2.86295	YES	YES
110	a	502.25	1.06203	YES	YES
111	a	510.42	11.50638	YES	YES
112	a	512.90	11.64469	YES	YES
113	a	630.74	10.57444	YES	YES
114	a	631.24	31.47386	YES	YES
115	a	640.53	9.45194	YES	YES
116	a	659.99	13.02658	YES	YES
117	a	698.16	3.22720	YES	YES
118	a	713.97	1.86963	YES	YES
119	a	722.12	2.26615	YES	YES
120	a	726.16	6.80034	YES	YES
121	a	731.76	3.41117	YES	YES
122	a	734.13	6.27124	YES	YES
123	a	734.64	10.98773	YES	YES
124	a	737.72	4.82574	YES	YES
125	a	770.86	17.96929	YES	YES
126	a	772.45	0.19428	YES	YES
127	a	772.61	1.62363	YES	YES
128	a	773.40	0.46258	YES	YES
129	a	773.77	2.81775	YES	YES
130	a	774.07	0.23767	YES	YES
131	a	775.53	0.31499	YES	YES
132	a	775.81	0.35677	YES	YES
133	a	777.98	2.70472	YES	YES
134	a	778.93	20.97526	YES	YES
135	a	806.30	0.79658	YES	YES
136	a	808.02	0.79539	YES	YES
137	a	809.13	4.07129	YES	YES
138	a	810.40	8.67380	YES	YES
139	a	811.33	3.63027	YES	YES

140	a	811.62	4.71022	YES	YES
141	a	812.52	5.24530	YES	YES
142	a	817.55	2.69117	YES	YES
143	a	832.22	7.85337	YES	YES
144	a	832.46	2.72644	YES	YES
145	a	836.42	1.46023	YES	YES
146	a	837.14	3.03395	YES	YES
147	a	837.34	0.94584	YES	YES
148	a	838.18	5.21479	YES	YES
149	a	839.17	6.95602	YES	YES
150	a	839.48	2.78899	YES	YES
151	a	847.05	26.03745	YES	YES
152	a	853.14	16.74959	YES	YES
153	a	873.95	6.11511	YES	YES
154	a	874.46	0.48082	YES	YES
155	a	874.83	1.81762	YES	YES
156	a	875.21	3.82529	YES	YES
157	a	875.78	1.36512	YES	YES
158	a	876.35	3.37225	YES	YES
159	a	877.55	4.60848	YES	YES
160	a	878.41	1.47950	YES	YES
161	a	878.87	2.74180	YES	YES
162	a	879.09	0.88281	YES	YES
163	a	879.79	0.71525	YES	YES
164	a	881.11	9.04059	YES	YES
165	a	881.34	6.24871	YES	YES
166	a	883.05	0.92634	YES	YES
167	a	884.58	3.84416	YES	YES
168	a	884.90	0.53337	YES	YES
169	a	902.88	3.48941	YES	YES
170	a	903.32	2.69034	YES	YES
171	a	904.05	3.71855	YES	YES
172	a	905.61	0.28919	YES	YES
173	a	906.74	4.24244	YES	YES
174	a	909.35	2.12428	YES	YES
175	a	909.98	0.47250	YES	YES
176	a	911.31	1.56442	YES	YES
177	a	986.04	4.32259	YES	YES
178	a	986.16	2.45655	YES	YES
179	a	987.42	3.67968	YES	YES
180	a	987.69	15.11743	YES	YES
181	a	988.33	17.20171	YES	YES
182	a	989.15	6.68161	YES	YES
183	a	989.68	6.00834	YES	YES
184	a	990.43	21.00824	YES	YES
185	a	991.52	5.89036	YES	YES
186	a	996.64	0.56748	YES	YES
187	a	1012.85	0.63239	YES	YES
188	a	1014.55	3.07582	YES	YES
189	a	1015.26	2.25135	YES	YES
190	a	1016.17	1.74140	YES	YES
191	a	1016.63	0.47621	YES	YES
192	a	1019.00	1.08577	YES	YES
193	a	1019.62	0.23971	YES	YES
194	a	1021.81	0.54438	YES	YES
195	a	1028.77	0.73605	YES	YES
196	a	1030.11	1.22312	YES	YES
197	a	1032.03	1.86296	YES	YES

198	a	1036.06	2.10696	YES	YES
199	a	1036.67	1.40700	YES	YES
200	a	1038.52	1.13307	YES	YES
201	a	1038.61	2.89361	YES	YES
202	a	1040.00	2.26759	YES	YES
203	a	1055.59	2.77414	YES	YES
204	a	1057.41	0.30216	YES	YES
205	a	1058.55	4.84573	YES	YES
206	a	1059.15	1.95294	YES	YES
207	a	1060.35	1.79098	YES	YES
208	a	1063.53	0.60971	YES	YES
209	a	1066.19	4.35659	YES	YES
210	a	1067.41	0.30958	YES	YES
211	a	1069.87	0.02893	YES	YES
212	a	1069.93	0.37413	YES	YES
213	a	1071.15	0.19897	YES	YES
214	a	1071.67	0.03775	YES	YES
215	a	1072.36	0.12016	YES	YES
216	a	1072.43	0.04906	YES	YES
217	a	1072.89	0.26693	YES	YES
218	a	1075.09	0.09984	YES	YES
219	a	1078.01	4.34237	YES	YES
220	a	1081.79	4.52295	YES	YES
221	a	1089.43	3.86274	YES	YES
222	a	1090.54	7.07676	YES	YES
223	a	1093.24	9.49564	YES	YES
224	a	1094.23	1.96142	YES	YES
225	a	1101.30	2.22160	YES	YES
226	a	1101.58	2.91683	YES	YES
227	a	1107.94	7.84187	YES	YES
228	a	1113.84	15.63624	YES	YES
229	a	1122.90	7.30113	YES	YES
230	a	1130.44	3.03421	YES	YES
231	a	1157.93	4.01837	YES	YES
232	a	1159.10	3.10454	YES	YES
233	a	1160.75	13.21422	YES	YES
234	a	1165.25	7.77947	YES	YES
235	a	1167.53	17.44087	YES	YES
236	a	1168.08	7.28312	YES	YES
237	a	1168.39	5.37125	YES	YES
238	a	1170.60	2.99027	YES	YES
239	a	1173.24	12.73883	YES	YES
240	a	1175.38	5.68750	YES	YES
241	a	1179.25	9.18478	YES	YES
242	a	1180.03	4.56104	YES	YES
243	a	1188.37	0.34325	YES	YES
244	a	1188.90	1.60629	YES	YES
245	a	1191.56	2.38867	YES	YES
246	a	1210.40	1.93410	YES	YES
247	a	1234.79	1.16516	YES	YES
248	a	1236.88	0.90999	YES	YES
249	a	1243.95	0.50754	YES	YES
250	a	1244.63	1.01203	YES	YES
251	a	1245.27	0.27179	YES	YES
252	a	1245.75	0.90102	YES	YES
253	a	1246.84	0.20829	YES	YES
254	a	1247.95	0.43148	YES	YES
255	a	1249.45	0.57365	YES	YES

256	a	1250.21	0.11833	YES	YES
257	a	1252.32	1.44104	YES	YES
258	a	1252.91	3.55024	YES	YES
259	a	1253.36	1.00007	YES	YES
260	a	1253.69	1.25921	YES	YES
261	a	1255.91	1.42298	YES	YES
262	a	1256.13	0.31522	YES	YES
263	a	1256.32	9.90419	YES	YES
264	a	1257.42	1.01349	YES	YES
265	a	1257.97	6.70051	YES	YES
266	a	1258.76	1.19905	YES	YES
267	a	1260.01	6.13873	YES	YES
268	a	1260.59	0.99778	YES	YES
269	a	1261.64	1.77143	YES	YES
270	a	1263.38	1.66106	YES	YES
271	a	1263.91	1.88849	YES	YES
272	a	1265.21	0.16958	YES	YES
273	a	1269.30	1.40793	YES	YES
274	a	1270.16	2.92880	YES	YES
275	a	1281.34	3.42211	YES	YES
276	a	1281.71	0.31525	YES	YES
277	a	1282.67	2.91932	YES	YES
278	a	1284.20	2.80575	YES	YES
279	a	1285.87	3.64522	YES	YES
280	a	1288.07	1.72686	YES	YES
281	a	1288.24	3.67588	YES	YES
282	a	1290.27	4.92735	YES	YES
283	a	1304.23	0.74014	YES	YES
284	a	1306.93	0.19187	YES	YES
285	a	1309.11	2.42583	YES	YES
286	a	1309.59	1.37583	YES	YES
287	a	1311.51	1.66381	YES	YES
288	a	1312.57	0.11554	YES	YES
289	a	1313.72	1.79367	YES	YES
290	a	1315.77	1.30302	YES	YES
291	a	1319.11	3.70483	YES	YES
292	a	1320.13	5.59232	YES	YES
293	a	1321.78	1.87052	YES	YES
294	a	1322.18	0.15669	YES	YES
295	a	1322.62	4.05250	YES	YES
296	a	1322.98	2.27002	YES	YES
297	a	1323.36	0.23910	YES	YES
298	a	1323.75	2.27828	YES	YES
299	a	1324.32	2.24276	YES	YES
300	a	1324.61	1.45002	YES	YES
301	a	1324.75	0.05737	YES	YES
302	a	1325.01	2.15813	YES	YES
303	a	1325.66	1.41917	YES	YES
304	a	1326.87	6.49973	YES	YES
305	a	1327.41	3.11979	YES	YES
306	a	1329.35	2.81036	YES	YES
307	a	1335.85	0.16776	YES	YES
308	a	1336.41	0.38668	YES	YES
309	a	1336.75	0.41510	YES	YES
310	a	1337.03	0.34819	YES	YES
311	a	1337.70	0.09367	YES	YES
312	a	1337.99	0.64814	YES	YES
313	a	1338.29	0.08050	YES	YES

314	a	1338.74	0.48646	YES	YES
315	a	1343.19	2.01883	YES	YES
316	a	1343.88	1.13015	YES	YES
317	a	1345.28	2.63813	YES	YES
318	a	1345.79	1.08132	YES	YES
319	a	1346.29	1.73865	YES	YES
320	a	1348.55	1.57204	YES	YES
321	a	1348.67	0.89093	YES	YES
322	a	1348.81	1.91143	YES	YES
323	a	1401.41	5.96334	YES	YES
324	a	1405.17	10.04516	YES	YES
325	a	1406.99	7.63761	YES	YES
326	a	1409.18	6.72860	YES	YES
327	a	1423.15	3.62521	YES	YES
328	a	1425.75	4.24906	YES	YES
329	a	1425.92	12.68974	YES	YES
330	a	1428.13	0.28349	YES	YES
331	a	1429.10	0.93529	YES	YES
332	a	1430.92	1.07931	YES	YES
333	a	1431.07	0.86349	YES	YES
334	a	1432.52	0.64820	YES	YES
335	a	1434.66	1.09701	YES	YES
336	a	1435.27	5.25743	YES	YES
337	a	1435.58	2.27343	YES	YES
338	a	1436.15	2.30912	YES	YES
339	a	1436.65	3.51683	YES	YES
340	a	1437.14	7.88182	YES	YES
341	a	1437.71	14.47030	YES	YES
342	a	1437.93	0.97428	YES	YES
343	a	1438.16	2.50328	YES	YES
344	a	1438.24	1.87531	YES	YES
345	a	1438.88	4.76994	YES	YES
346	a	1439.22	2.97146	YES	YES
347	a	1439.31	1.73109	YES	YES
348	a	1439.49	5.39297	YES	YES
349	a	1439.79	4.67700	YES	YES
350	a	1441.13	0.37875	YES	YES
351	a	1442.31	8.73856	YES	YES
352	a	1442.62	19.25412	YES	YES
353	a	1443.20	7.03582	YES	YES
354	a	1443.34	33.39448	YES	YES
355	a	1444.27	1.98587	YES	YES
356	a	1444.55	21.12804	YES	YES
357	a	1445.36	15.31385	YES	YES
358	a	1448.10	9.42241	YES	YES
359	a	1452.22	2.69947	YES	YES
360	a	1452.96	0.18968	YES	YES
361	a	1453.60	5.19679	YES	YES
362	a	1454.31	1.84987	YES	YES
363	a	1454.64	1.48648	YES	YES
364	a	1455.21	1.65127	YES	YES
365	a	1456.11	0.11915	YES	YES
366	a	1458.31	0.29506	YES	YES
367	a	2897.57	3.84486	YES	YES
368	a	2907.50	37.12499	YES	YES
369	a	2914.19	60.09382	YES	YES
370	a	2918.03	3.21925	YES	YES
371	a	2918.47	23.85833	YES	YES

372	a	2922.30	9.46689	YES	YES
373	a	2924.25	3.76985	YES	YES
374	a	2924.55	21.30014	YES	YES
375	a	2924.73	9.23600	YES	YES
376	a	2929.38	21.68557	YES	YES
377	a	2933.64	29.80520	YES	YES
378	a	2938.24	20.38795	YES	YES
379	a	2938.82	10.98556	YES	YES
380	a	2940.64	26.49168	YES	YES
381	a	2941.37	10.35642	YES	YES
382	a	2942.13	3.30383	YES	YES
383	a	2942.60	16.05997	YES	YES
384	a	2943.34	8.69451	YES	YES
385	a	2944.07	7.48685	YES	YES
386	a	2944.98	5.67484	YES	YES
387	a	2945.25	4.63707	YES	YES
388	a	2945.68	14.48020	YES	YES
389	a	2946.05	8.37251	YES	YES
390	a	2946.71	9.15552	YES	YES
391	a	2947.32	10.53313	YES	YES
392	a	2948.67	5.47790	YES	YES
393	a	2950.47	3.42387	YES	YES
394	a	2951.11	13.95218	YES	YES
395	a	2951.71	9.63144	YES	YES
396	a	2955.45	4.56821	YES	YES
397	a	2955.72	4.75074	YES	YES
398	a	2956.28	6.09112	YES	YES
399	a	2956.77	4.61390	YES	YES
400	a	2957.49	5.42021	YES	YES
401	a	2959.02	9.68538	YES	YES
402	a	2959.53	6.76642	YES	YES
403	a	2959.70	26.79616	YES	YES
404	a	2959.91	11.29435	YES	YES
405	a	2960.39	26.33106	YES	YES
406	a	2960.45	36.90080	YES	YES
407	a	2961.01	73.42928	YES	YES
408	a	2961.13	19.57895	YES	YES
409	a	2963.06	43.23861	YES	YES
410	a	2964.08	26.64028	YES	YES
411	a	2964.50	25.88806	YES	YES
412	a	2966.90	0.79270	YES	YES
413	a	2972.31	14.41920	YES	YES
414	a	2973.53	9.63789	YES	YES
415	a	2974.49	24.55338	YES	YES
416	a	2980.93	21.24916	YES	YES
417	a	2982.47	16.98522	YES	YES
418	a	2982.80	3.91048	YES	YES
419	a	2984.85	4.51076	YES	YES
420	a	2985.84	10.79564	YES	YES
421	a	2986.90	11.62910	YES	YES
422	a	2988.16	4.13223	YES	YES
423	a	2990.21	29.55315	YES	YES
424	a	2995.41	20.82539	YES	YES
425	a	2997.24	33.82276	YES	YES
426	a	2998.93	21.21414	YES	YES
427	a	3000.29	21.76372	YES	YES
428	a	3001.84	29.07383	YES	YES
429	a	3003.21	7.02976	YES	YES

430	a	3003.33	31.06212	YES	YES
431	a	3003.65	40.65606	YES	YES
432	a	3004.73	30.81451	YES	YES
433	a	3005.72	14.93254	YES	YES
434	a	3007.20	17.92703	YES	YES
435	a	3008.70	16.49923	YES	YES
436	a	3009.01	17.70426	YES	YES
437	a	3010.88	23.03653	YES	YES
438	a	3011.54	17.60275	YES	YES
439	a	3011.73	23.05067	YES	YES
440	a	3013.23	19.79837	YES	YES
441	a	3013.82	18.08133	YES	YES
442	a	3013.87	26.75183	YES	YES
443	a	3014.19	17.53084	YES	YES
444	a	3015.05	16.75186	YES	YES
445	a	3015.49	17.63004	YES	YES
446	a	3015.69	20.97885	YES	YES
447	a	3016.13	25.17473	YES	YES
448	a	3016.54	20.19419	YES	YES
449	a	3017.76	29.13775	YES	YES
450	a	3018.24	18.20136	YES	YES
451	a	3018.44	26.37214	YES	YES
452	a	3018.52	30.26508	YES	YES
453	a	3018.70	38.31006	YES	YES
454	a	3018.78	20.55836	YES	YES
455	a	3019.00	37.35209	YES	YES
456	a	3019.44	20.92608	YES	YES
457	a	3020.41	19.57105	YES	YES
458	a	3022.54	30.77771	YES	YES
459	a	3026.79	2.26441	YES	YES
460	a	3028.45	0.47743	YES	YES
461	a	3037.58	1.65825	YES	YES
462	a	3045.01	1.01003	YES	YES

$end

Double hybrid single point energy = -7249.045868529255 H
COSMO energy + OC correction = -7256.3135866390 H (in *o*DFB)

6.2.3 [Ga(dtbpf)]⁺

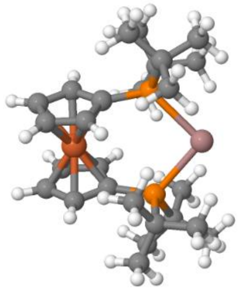

Method: (RI-)BP86(D3BJ)/def2-TZVPP
Symmetry: c1

Cartesian coordinates in Ångström:

Ga	1.3340101	0.7103251	-1.6910945
Fe	-1.4416557	-0.7680499	1.8240354
P	1.4515160	-1.3293270	-0.0156445
P	-0.9233779	1.6106840	-0.6535103

C	2.9173717	-0.9557545	1.1196518
C	4.0636630	-0.3455371	0.2886896
H	4.8444071	0.0013958	0.9807442
H	3.7327066	0.5284629	-0.2924488
H	4.5246076	-1.0543225	-0.4024500
C	2.4584702	0.1432028	2.0950183
H	3.3092300	0.4250096	2.7316312
H	1.6341756	-0.1661113	2.7422596
H	2.1464461	1.0445321	1.5485967
C	3.4302609	-2.1838793	1.8836448
H	4.2044614	-1.8660524	2.5977025
H	3.8891666	-2.9157699	1.2090067
H	2.6410950	-2.6925149	2.4481413
C	1.7810402	-2.8286782	-1.1414207
C	3.1854799	-2.7510500	-1.7577681
H	3.2700432	-3.5339974	-2.5251485
H	3.9744832	-2.9329088	-1.0201034
H	3.3741531	-1.7872250	-2.2496438
C	0.7614803	-2.7257826	-2.2927097
H	0.9589202	-3.5265380	-3.0198778
H	0.8518902	-1.7704069	-2.8358008
H	-0.2724764	-2.8433380	-1.9535066
C	1.5986087	-4.1616458	-0.4045854
H	1.7577205	-4.9868709	-1.1143836
H	0.5896331	-4.2652478	0.0108381
H	2.3188613	-4.2825891	0.4121714
C	-0.5884974	3.4624734	-0.3660202
C	0.7727246	3.5396479	0.3527260
H	1.0555657	4.5952300	0.4741431
H	1.5733367	3.0588489	-0.2335302
H	0.7491529	3.0932857	1.3518268
C	-1.6698529	4.1227565	0.4986562
H	-1.3995880	5.1758674	0.6661545
H	-1.7627377	3.6396077	1.4780634
H	-2.6522692	4.1050725	0.0139263
C	-0.4499524	4.2066556	-1.7021590
H	-0.0901200	5.2248325	-1.4951590
H	-1.4065684	4.3008569	-2.2272933
H	0.2782719	3.7308566	-2.3728276
C	-2.2322232	1.3224847	-1.9881350
C	-1.6307880	1.6438789	-3.3708171
H	-2.3501012	1.3303655	-4.1409708
H	-0.6970428	1.0909244	-3.5536704
H	-1.4275681	2.7064084	-3.5202142
C	-2.5419273	-0.1854654	-1.9921017
H	-3.2586578	-0.3943383	-2.7990347
H	-2.9695624	-0.5444003	-1.0527971
H	-1.6345948	-0.7696246	-2.2015403
C	-3.5125190	2.1422571	-1.7785684
H	-4.2599361	1.8401707	-2.5271535
H	-3.3309539	3.2148138	-1.9130204
H	-3.9527357	1.9955748	-0.7861827
C	0.0665994	-1.8859521	1.0180100
C	-0.0268175	-2.1149682	2.4415982
H	0.7461528	-1.9329534	3.1775001
C	-1.3313847	-2.6141425	2.7403954
H	-1.7051119	-2.8452409	3.7325837
C	-2.0651820	-2.7066996	1.5208739

```
H    -3.0964412   -3.0267461    1.4150410
C    -1.2197451   -2.2527289    0.4644991
H    -1.5069921   -2.1822197   -0.5749297
C    -1.6107927    1.0638279    0.9355171
C    -0.8661686    1.1418669    2.1745331
H     0.1596528    1.4652310    2.2795184
C    -1.7009437    0.6998239    3.2443192
H    -1.4099275    0.6251507    4.2868354
C    -2.9615251    0.3264594    2.6913918
H    -3.8024867   -0.0900873    3.2361817
C    -2.9153492    0.5475124    1.2809657
H    -3.7307844    0.3428104    0.5988296

SCF energy GEOOPT =  -4889.656646528 H
ZPE =    1642. kJ/mol
FREEH energy =  1740.85 kJ/mol
FREEH entropy =    0.91153 kJ/mol/K

$vibrational spectrum
#  mode     symmetry     wave number   IR intensity    selection rules
#                         cm**(-1)        km/mol          IR     RAMAN
     1                      -0.00          0.00000         -        -
     2                      -0.00          0.00000         -        -
     3                      -0.00          0.00000         -        -
     4                       0.00          0.00000         -        -
     5                       0.00          0.00000         -        -
     6                       0.00          0.00000         -        -
     7         a            22.91          0.19788        YES      YES
     8         a            34.17          0.01252        YES      YES
     9         a            51.22          0.01467        YES      YES
    10         a            63.92          0.00047        YES      YES
    11         a            68.40          0.01244        YES      YES
    12         a            81.20          0.02355        YES      YES
    13         a            86.41          0.05064        YES      YES
    14         a            98.40          0.01733        YES      YES
    15         a           100.81          1.61369        YES      YES
    16         a           105.48          0.57629        YES      YES
    17         a           112.36          3.66740        YES      YES
    18         a           118.02          2.48191        YES      YES
    19         a           123.00          0.03746        YES      YES
    20         a           140.70          1.56419        YES      YES
    21         a           144.73          0.17300        YES      YES
    22         a           162.54          4.99398        YES      YES
    23         a           164.93          5.50181        YES      YES
    24         a           176.86          0.23128        YES      YES
    25         a           189.04          0.06970        YES      YES
    26         a           190.76          0.01825        YES      YES
    27         a           202.01          0.70757        YES      YES
    28         a           204.33          0.66039        YES      YES
    29         a           211.05          1.71659        YES      YES
    30         a           214.34          0.83470        YES      YES
    31         a           216.88          3.29612        YES      YES
    32         a           226.66          0.16966        YES      YES
    33         a           238.57          0.05372        YES      YES
    34         a           239.67          0.72940        YES      YES
    35         a           240.06          0.46989        YES      YES
    36         a           245.31          0.95099        YES      YES
    37         a           261.37          0.09870        YES      YES
```

38	a	262.40	0.66625	YES	YES
39	a	262.76	0.52342	YES	YES
40	a	263.10	0.04653	YES	YES
41	a	267.01	0.00424	YES	YES
42	a	270.71	0.17936	YES	YES
43	a	274.91	0.17628	YES	YES
44	a	275.52	0.18438	YES	YES
45	a	299.82	0.58622	YES	YES
46	a	303.14	0.98451	YES	YES
47	a	327.83	0.17024	YES	YES
48	a	329.51	0.25125	YES	YES
49	a	353.00	0.75363	YES	YES
50	a	357.24	1.78391	YES	YES
51	a	360.17	1.70800	YES	YES
52	a	366.88	0.80772	YES	YES
53	a	369.35	0.26028	YES	YES
54	a	374.60	2.54529	YES	YES
55	a	377.97	0.25407	YES	YES
56	a	397.70	2.14447	YES	YES
57	a	399.09	1.55236	YES	YES
58	a	406.74	0.53627	YES	YES
59	a	418.01	3.79568	YES	YES
60	a	424.27	11.97697	YES	YES
61	a	426.15	1.04674	YES	YES
62	a	454.44	26.14071	YES	YES
63	a	455.17	33.41743	YES	YES
64	a	470.70	28.34704	YES	YES
65	a	472.99	5.45387	YES	YES
66	a	493.07	10.82827	YES	YES
67	a	511.09	8.93660	YES	YES
68	a	520.04	1.88306	YES	YES
69	a	554.04	9.22505	YES	YES
70	a	557.94	0.49833	YES	YES
71	a	572.21	0.03280	YES	YES
72	a	576.39	0.61146	YES	YES
73	a	588.92	0.68833	YES	YES
74	a	589.80	5.93916	YES	YES
75	a	612.88	4.14284	YES	YES
76	a	617.67	2.09488	YES	YES
77	a	792.01	1.31164	YES	YES
78	a	792.74	11.44072	YES	YES
79	a	794.05	0.56729	YES	YES
80	a	797.72	9.63041	YES	YES
81	a	799.02	1.52764	YES	YES
82	a	806.92	33.00966	YES	YES
83	a	813.33	0.27220	YES	YES
84	a	818.99	2.35948	YES	YES
85	a	820.82	5.49590	YES	YES
86	a	823.35	2.66079	YES	YES
87	a	840.92	1.68043	YES	YES
88	a	849.98	0.56699	YES	YES
89	a	871.71	1.08083	YES	YES
90	a	875.62	3.12171	YES	YES
91	a	878.54	1.29784	YES	YES
92	a	879.86	1.02529	YES	YES
93	a	912.60	1.00705	YES	YES
94	a	913.17	0.98053	YES	YES
95	a	914.81	0.87345	YES	YES

96	a	915.30	2.91219	YES	YES
97	a	918.08	1.14623	YES	YES
98	a	918.99	1.23487	YES	YES
99	a	920.48	1.24629	YES	YES
100	a	921.34	0.62893	YES	YES
101	a	932.71	0.02879	YES	YES
102	a	933.28	0.05146	YES	YES
103	a	940.17	0.20481	YES	YES
104	a	940.47	0.35373	YES	YES
105	a	987.51	0.34096	YES	YES
106	a	988.01	1.11524	YES	YES
107	a	998.55	1.57478	YES	YES
108	a	999.56	0.85163	YES	YES
109	a	1002.52	0.16138	YES	YES
110	a	1002.77	1.77485	YES	YES
111	a	1006.75	4.88165	YES	YES
112	a	1007.19	5.75941	YES	YES
113	a	1026.50	0.91952	YES	YES
114	a	1030.18	2.88953	YES	YES
115	a	1032.77	13.84986	YES	YES
116	a	1035.61	7.96022	YES	YES
117	a	1052.33	1.31973	YES	YES
118	a	1052.99	1.03629	YES	YES
119	a	1149.12	0.38458	YES	YES
120	a	1149.70	13.65429	YES	YES
121	a	1153.89	74.61034	YES	YES
122	a	1154.44	10.62833	YES	YES
123	a	1158.58	31.19319	YES	YES
124	a	1160.14	1.21805	YES	YES
125	a	1167.88	1.25735	YES	YES
126	a	1171.11	2.81632	YES	YES
127	a	1179.48	1.57039	YES	YES
128	a	1182.18	3.09711	YES	YES
129	a	1189.26	0.58648	YES	YES
130	a	1189.67	0.64676	YES	YES
131	a	1192.52	0.13359	YES	YES
132	a	1192.97	0.09317	YES	YES
133	a	1197.52	0.68292	YES	YES
134	a	1199.92	1.07967	YES	YES
135	a	1291.58	3.30019	YES	YES
136	a	1293.77	0.97520	YES	YES
137	a	1345.62	4.68351	YES	YES
138	a	1347.71	4.78599	YES	YES
139	a	1353.52	0.46528	YES	YES
140	a	1354.05	2.01193	YES	YES
141	a	1356.79	3.96416	YES	YES
142	a	1357.18	9.00760	YES	YES
143	a	1357.25	10.61419	YES	YES
144	a	1358.29	25.15581	YES	YES
145	a	1360.95	0.51661	YES	YES
146	a	1361.40	0.11603	YES	YES
147	a	1378.24	9.63993	YES	YES
148	a	1379.58	0.12004	YES	YES
149	a	1383.48	22.39773	YES	YES
150	a	1384.08	3.82420	YES	YES
151	a	1389.61	8.58764	YES	YES
152	a	1390.17	19.20312	YES	YES
153	a	1403.26	0.99047	YES	YES

154	a	1405.12	0.38824	YES	YES
155	a	1419.90	1.23855	YES	YES
156	a	1421.24	0.16435	YES	YES
157	a	1422.63	0.44963	YES	YES
158	a	1423.35	0.84758	YES	YES
159	a	1429.93	1.63478	YES	YES
160	a	1430.54	1.45825	YES	YES
161	a	1431.03	0.52053	YES	YES
162	a	1431.72	0.68037	YES	YES
163	a	1438.06	2.45102	YES	YES
164	a	1438.56	1.56689	YES	YES
165	a	1440.96	3.46917	YES	YES
166	a	1442.53	0.47142	YES	YES
167	a	1449.16	11.77392	YES	YES
168	a	1449.56	13.00698	YES	YES
169	a	1452.43	0.64157	YES	YES
170	a	1454.21	2.73101	YES	YES
171	a	1454.52	0.13004	YES	YES
172	a	1455.24	5.65510	YES	YES
173	a	1461.31	7.43010	YES	YES
174	a	1461.62	1.60086	YES	YES
175	a	1469.04	0.54332	YES	YES
176	a	1469.66	39.60082	YES	YES
177	a	1473.10	8.52824	YES	YES
178	a	1473.58	10.58823	YES	YES
179	a	2934.53	29.61942	YES	YES
180	a	2935.24	7.93811	YES	YES
181	a	2954.48	10.27662	YES	YES
182	a	2954.99	11.77017	YES	YES
183	a	2964.82	12.22909	YES	YES
184	a	2965.02	9.42190	YES	YES
185	a	2965.23	17.02532	YES	YES
186	a	2965.25	15.24119	YES	YES
187	a	2970.53	23.41089	YES	YES
188	a	2970.56	10.30363	YES	YES
189	a	2971.37	40.15295	YES	YES
190	a	2971.56	14.02125	YES	YES
191	a	3008.10	40.83059	YES	YES
192	a	3008.37	3.13370	YES	YES
193	a	3014.84	43.69602	YES	YES
194	a	3015.29	0.87705	YES	YES
195	a	3024.07	52.64528	YES	YES
196	a	3024.21	3.08250	YES	YES
197	a	3027.29	27.38493	YES	YES
198	a	3027.44	8.75558	YES	YES
199	a	3039.25	13.41654	YES	YES
200	a	3039.47	21.65080	YES	YES
201	a	3041.93	4.12218	YES	YES
202	a	3042.18	5.44432	YES	YES
203	a	3059.84	11.34124	YES	YES
204	a	3060.95	10.99189	YES	YES
205	a	3066.44	5.74832	YES	YES
206	a	3066.63	6.00897	YES	YES
207	a	3068.30	0.70470	YES	YES
208	a	3068.69	1.58205	YES	YES
209	a	3069.41	23.07829	YES	YES
210	a	3069.88	20.57266	YES	YES
211	a	3087.75	5.84663	YES	YES

212	a	3089.73	5.54856	YES	YES
213	a	3096.85	8.43750	YES	YES
214	a	3097.71	8.11361	YES	YES
215	a	3164.17	0.08086	YES	YES
216	a	3164.24	0.04353	YES	YES
217	a	3175.76	0.52228	YES	YES
218	a	3175.97	0.55337	YES	YES
219	a	3190.73	1.72736	YES	YES
220	a	3190.96	0.88210	YES	YES
221	a	3211.58	1.56128	YES	YES
222	a	3212.15	0.55721	YES	YES

$end

6.2.4 [Ga(dcpe)]$^+$

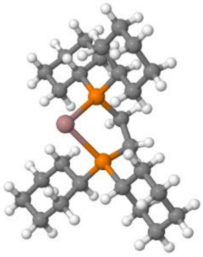

Method: (RI-)BP86(D3BJ)/def2-TZVPP
Symmetry: c2

Cartesian coordinates in Ångström:

Ga	-0.0000000	0.0000000	2.1405337
P	-0.8248189	-1.3619484	0.0539142
P	0.8248189	1.3619484	0.0539142
C	-0.6357848	-3.1979283	-0.0094537
H	-1.1360682	-3.5434903	0.9114336
C	0.8443289	-3.6071087	0.0903907
H	1.3951521	-3.2215623	-0.7837176
H	1.3041363	-3.1544345	0.9815559
C	0.9859215	-5.1342066	0.1317948
H	2.0501133	-5.4057855	0.1672848
H	0.5339179	-5.5106019	1.0640086
C	0.3013669	-5.7929551	-1.0702716
H	0.3855310	-6.8860300	-1.0016948
H	0.8241859	-5.4968287	-1.9952410
C	-1.1708431	-5.3804236	-1.1673308
H	-1.7192636	-5.7675003	-0.2928228
H	-1.6400715	-5.8276164	-2.0545850
C	-1.3230729	-3.8538575	-1.2184240
H	-2.3871924	-3.5794911	-1.2544409
H	-0.8645465	-3.4763636	-2.1468307
C	-2.6423860	-1.0104920	0.0739993
H	-3.0692707	-1.3201210	-0.8949880
C	-3.3311397	-1.7989335	1.2028181
H	-3.2323513	-2.8790424	1.0296238
H	-2.8207352	-1.5842816	2.1582862
C	-4.8133698	-1.4247313	1.3254850
H	-5.3425501	-1.7428587	0.4122133
H	-5.2626818	-1.9833320	2.1579177
C	-5.0006725	0.0818038	1.5237919
H	-6.0690650	0.3294338	1.5821941

```
H    -4.5558601    0.3821582    2.4873921
C    -4.3360965    0.8683862    0.3914939
H    -4.4363324    1.9505390    0.5551098
H    -4.8457052    0.6457362   -0.5605252
C    -2.8550467    0.5047090    0.2534973
H    -2.4075561    1.0602304   -0.5804686
H    -2.3247758    0.8343915    1.1714412
C    -0.1921759   -0.7444434   -1.5816450
H     0.6858801   -1.3633783   -1.8084444
H    -0.9337067   -0.9517217   -2.3657344
C     0.1921759    0.7444434   -1.5816450
H    -0.6858801    1.3633783   -1.8084444
H     0.9337067    0.9517217   -2.3657344
C     2.6423860    1.0104920    0.0739993
H     3.0692707    1.3201210   -0.8949880
C     3.3311397    1.7989335    1.2028181
H     2.8207352    1.5842816    2.1582862
H     3.2323513    2.8790424    1.0296238
C     4.8133698    1.4247313    1.3254850
H     5.3425501    1.7428587    0.4122133
H     5.2626818    1.9833320    2.1579177
C     5.0006725   -0.0818038    1.5237919
H     4.5558601   -0.3821582    2.4873921
H     6.0690650   -0.3294338    1.5821941
C     4.3360965   -0.8683862    0.3914939
H     4.4363324   -1.9505390    0.5551098
H     4.8457052   -0.6457362   -0.5605252
C     2.8550467   -0.5047090    0.2534973
H     2.3247758   -0.8343915    1.1714412
H     2.4075561   -1.0602304   -0.5804686
C     0.6357848    3.1979283   -0.0094537
H     1.1360682    3.5434903    0.9114336
C    -0.8443289    3.6071087    0.0903907
H    -1.3041363    3.1544345    0.9815559
H    -1.3951521    3.2215623   -0.7837176
C    -0.9859215    5.1342066    0.1317948
H    -2.0501133    5.4057855    0.1672848
H    -0.5339179    5.5106019    1.0640086
C    -0.3013669    5.7929551   -1.0702716
H    -0.8241859    5.4968287   -1.9952410
H    -0.3855310    6.8860300   -1.0016948
C     1.1708431    5.3804236   -1.1673308
H     1.7192636    5.7675003   -0.2928228
H     1.6400715    5.8276164   -2.0545850
C     1.3230729    3.8538575   -1.2184240
H     0.8645465    3.4763636   -2.1468307
H     2.3871924    3.5794911   -1.2544409

SCF energy GEOOPT =  -3628.083122169 H
ZPE =   1793. kJ/mol
FREEH energy =  1880.12 kJ/mol
FREEH entropy =   0.87088 kJ/mol/K

$vibrational spectrum
#  mode     symmetry     wave number   IR intensity    selection rules
#                         cm**(-1)        km/mol          IR     RAMAN
     1                        0.00         0.00000         -       -
     2                        0.00         0.00000         -       -
```

3		0.00	0.00000	-	-
4		0.00	0.00000	-	-
5		0.00	0.00000	-	-
6		0.00	0.00000	-	-
7	a	23.11	0.00661	YES	YES
8	b	33.06	0.06666	YES	YES
9	a	37.74	0.01490	YES	YES
10	b	41.10	0.19935	YES	YES
11	a	43.43	0.14321	YES	YES
12	a	44.73	0.12202	YES	YES
13	b	48.82	0.30806	YES	YES
14	b	53.51	0.02964	YES	YES
15	b	66.89	0.41674	YES	YES
16	a	68.14	0.02005	YES	YES
17	a	69.51	0.09832	YES	YES
18	b	70.78	0.00378	YES	YES
19	a	117.66	0.49772	YES	YES
20	b	122.06	0.92535	YES	YES
21	b	127.83	1.56923	YES	YES
22	a	133.13	1.90038	YES	YES
23	a	138.51	1.66315	YES	YES
24	a	171.47	1.95694	YES	YES
25	b	174.93	0.69242	YES	YES
26	a	194.48	2.09142	YES	YES
27	b	197.60	1.49132	YES	YES
28	b	217.60	0.07493	YES	YES
29	a	220.19	0.73480	YES	YES
30	b	225.13	0.32084	YES	YES
31	a	225.31	0.31537	YES	YES
32	b	238.42	0.08895	YES	YES
33	a	240.87	0.11064	YES	YES
34	b	260.42	1.43153	YES	YES
35	a	266.72	1.63134	YES	YES
36	b	299.72	0.59961	YES	YES
37	a	306.46	0.03497	YES	YES
38	a	329.34	0.21523	YES	YES
39	b	329.47	0.57446	YES	YES
40	b	347.20	0.50944	YES	YES
41	b	357.21	5.24551	YES	YES
42	a	359.48	0.04198	YES	YES
43	a	394.93	0.16971	YES	YES
44	b	415.85	3.19822	YES	YES
45	a	422.43	0.04872	YES	YES
46	b	424.06	2.18169	YES	YES
47	a	430.34	0.04491	YES	YES
48	b	430.84	0.00417	YES	YES
49	a	431.88	0.00051	YES	YES
50	b	432.93	0.52317	YES	YES
51	a	442.26	0.31041	YES	YES
52	b	465.65	5.92144	YES	YES
53	a	491.13	0.34154	YES	YES
54	b	491.95	4.77065	YES	YES
55	a	504.50	0.06260	YES	YES
56	b	515.21	19.49590	YES	YES
57	b	629.17	17.07453	YES	YES
58	a	631.36	4.21466	YES	YES
59	b	715.06	4.99029	YES	YES
60	a	723.69	0.04839	YES	YES

61	a	733.94	1.56253	YES	YES
62	b	735.58	7.43648	YES	YES
63	b	761.61	19.02670	YES	YES
64	a	769.81	0.00017	YES	YES
65	b	770.16	0.36372	YES	YES
66	a	773.92	0.02165	YES	YES
67	b	774.07	0.59070	YES	YES
68	a	808.40	1.21964	YES	YES
69	b	810.68	2.87258	YES	YES
70	a	811.29	0.01120	YES	YES
71	b	811.61	7.48938	YES	YES
72	a	830.62	2.12633	YES	YES
73	b	837.33	6.32493	YES	YES
74	a	837.73	0.20426	YES	YES
75	b	839.04	10.97779	YES	YES
76	a	847.88	0.87667	YES	YES
77	b	872.23	3.30381	YES	YES
78	a	873.53	3.20144	YES	YES
79	b	875.89	10.86205	YES	YES
80	a	876.79	0.11450	YES	YES
81	a	877.41	0.48725	YES	YES
82	b	877.65	4.67778	YES	YES
83	b	880.22	0.64513	YES	YES
84	a	882.39	1.65081	YES	YES
85	b	900.81	1.65889	YES	YES
86	a	902.72	0.39748	YES	YES
87	b	907.78	3.46702	YES	YES
88	a	908.21	0.00566	YES	YES
89	b	987.01	1.36911	YES	YES
90	a	987.21	3.49851	YES	YES
91	b	988.85	19.54758	YES	YES
92	a	990.41	7.08185	YES	YES
93	a	992.95	3.11147	YES	YES
94	b	1015.80	0.06209	YES	YES
95	a	1016.05	0.96177	YES	YES
96	b	1021.20	0.48647	YES	YES
97	a	1021.45	0.39336	YES	YES
98	b	1029.04	0.00374	YES	YES
99	a	1030.01	1.36771	YES	YES
100	b	1038.38	5.23720	YES	YES
101	a	1038.75	0.32159	YES	YES
102	b	1058.62	0.89348	YES	YES
103	a	1058.63	0.04103	YES	YES
104	b	1059.28	5.26938	YES	YES
105	a	1066.02	0.04466	YES	YES
106	b	1070.38	1.63959	YES	YES
107	a	1071.68	0.05294	YES	YES
108	b	1072.79	2.57365	YES	YES
109	a	1075.48	0.06778	YES	YES
110	b	1076.21	1.59387	YES	YES
111	a	1087.26	1.35924	YES	YES
112	b	1090.77	6.91920	YES	YES
113	a	1098.70	0.04139	YES	YES
114	b	1105.40	9.11553	YES	YES
115	a	1128.82	0.46273	YES	YES
116	a	1158.22	3.35191	YES	YES
117	b	1159.01	0.54633	YES	YES
118	b	1168.36	27.28763	YES	YES

119	a	1169.24	0.55143	YES	YES
120	b	1173.07	3.54121	YES	YES
121	a	1174.03	1.24330	YES	YES
122	b	1187.91	3.76277	YES	YES
123	a	1189.86	0.48539	YES	YES
124	b	1225.63	2.24782	YES	YES
125	a	1243.51	0.21010	YES	YES
126	b	1246.39	2.55224	YES	YES
127	a	1247.52	0.47355	YES	YES
128	b	1251.09	0.25653	YES	YES
129	a	1252.55	0.00025	YES	YES
130	a	1253.99	0.03421	YES	YES
131	b	1254.02	5.35525	YES	YES
132	b	1257.31	5.33201	YES	YES
133	a	1257.99	2.25084	YES	YES
134	b	1258.97	8.68072	YES	YES
135	a	1259.09	1.53097	YES	YES
136	b	1262.47	4.40384	YES	YES
137	a	1263.08	0.00747	YES	YES
138	a	1282.75	0.68572	YES	YES
139	b	1282.80	3.92766	YES	YES
140	b	1288.61	5.81457	YES	YES
141	a	1288.88	0.76799	YES	YES
142	b	1310.96	3.12029	YES	YES
143	a	1311.56	0.01058	YES	YES
144	b	1314.47	1.20909	YES	YES
145	a	1314.48	0.13065	YES	YES
146	b	1322.26	1.92844	YES	YES
147	a	1322.75	1.00005	YES	YES
148	a	1323.66	1.50123	YES	YES
149	b	1323.67	1.22809	YES	YES
150	b	1324.93	1.99484	YES	YES
151	a	1324.95	0.87471	YES	YES
152	b	1327.28	3.63970	YES	YES
153	a	1327.74	7.93669	YES	YES
154	b	1335.90	0.10096	YES	YES
155	a	1335.96	1.04938	YES	YES
156	a	1337.14	0.00707	YES	YES
157	b	1337.32	0.23492	YES	YES
158	b	1344.78	5.33673	YES	YES
159	a	1344.98	0.97021	YES	YES
160	b	1347.83	2.27450	YES	YES
161	a	1347.87	0.04968	YES	YES
162	b	1406.11	6.73795	YES	YES
163	a	1410.39	1.42430	YES	YES
164	b	1411.48	8.90924	YES	YES
165	a	1413.34	8.15444	YES	YES
166	b	1430.05	2.29886	YES	YES
167	a	1431.54	1.51490	YES	YES
168	a	1434.41	0.00176	YES	YES
169	b	1435.49	6.50186	YES	YES
170	a	1436.11	1.55432	YES	YES
171	b	1436.30	17.67660	YES	YES
172	b	1438.22	6.02175	YES	YES
173	a	1438.32	2.25378	YES	YES
174	b	1439.83	0.49641	YES	YES
175	a	1439.87	0.00024	YES	YES
176	a	1442.89	30.48399	YES	YES

177	b	1443.05	1.79744	YES	YES
178	b	1444.69	16.79287	YES	YES
179	a	1444.83	4.49806	YES	YES
180	a	1452.37	1.22471	YES	YES
181	b	1452.54	4.72776	YES	YES
182	b	1454.87	0.28154	YES	YES
183	a	1455.38	0.00687	YES	YES
184	b	2851.40	142.52655	YES	YES
185	a	2853.79	9.61907	YES	YES
186	b	2928.67	10.21872	YES	YES
187	a	2928.72	1.79025	YES	YES
188	a	2938.10	0.04259	YES	YES
189	b	2938.17	29.02088	YES	YES
190	b	2942.53	14.97135	YES	YES
191	a	2942.57	9.39953	YES	YES
192	b	2942.71	18.36806	YES	YES
193	a	2942.79	1.76327	YES	YES
194	a	2944.69	1.57410	YES	YES
195	b	2944.77	25.66260	YES	YES
196	b	2947.71	17.82401	YES	YES
197	a	2947.81	6.30882	YES	YES
198	a	2952.06	4.69832	YES	YES
199	b	2952.30	12.76826	YES	YES
200	b	2952.57	7.40247	YES	YES
201	a	2952.79	1.85790	YES	YES
202	b	2955.88	0.22918	YES	YES
203	a	2955.93	8.74273	YES	YES
204	b	2958.18	76.46042	YES	YES
205	a	2958.29	1.12222	YES	YES
206	b	2958.89	11.64694	YES	YES
207	a	2958.89	89.91379	YES	YES
208	b	2978.99	6.41121	YES	YES
209	a	2984.72	3.62729	YES	YES
210	b	2991.33	9.04792	YES	YES
211	a	2991.43	44.06557	YES	YES
212	a	3000.85	1.03700	YES	YES
213	b	3000.89	55.97565	YES	YES
214	a	3005.54	4.50975	YES	YES
215	b	3005.57	37.30448	YES	YES
216	a	3007.94	0.07594	YES	YES
217	b	3007.97	66.03052	YES	YES
218	b	3008.22	37.83825	YES	YES
219	a	3008.27	8.56363	YES	YES
220	b	3009.76	37.55844	YES	YES
221	a	3009.80	31.68979	YES	YES
222	b	3011.57	21.96275	YES	YES
223	a	3011.62	33.03292	YES	YES
224	a	3013.08	18.27614	YES	YES
225	b	3013.09	52.21455	YES	YES
226	b	3014.15	43.37986	YES	YES
227	a	3014.18	3.95885	YES	YES
228	b	3014.94	21.83648	YES	YES
229	a	3015.09	37.24130	YES	YES
230	a	3021.88	0.21182	YES	YES
231	b	3038.54	4.65595	YES	YES

$end

6.2.5 $HCCC_3H_5$

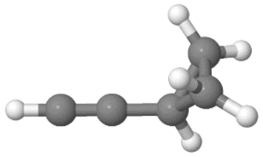

```
Method: (RI-)BP86(D3BJ)/def2-TZVPP
Symmetry: cs

Cartesian coordinates in Ångström:
C    -2.4798527   -0.4282591    0.0000000
C    -1.2697989   -0.5097138    0.0000000
H    -3.5462910   -0.3590792    0.0000000
C     0.1603871   -0.5971183    0.0000000
C     0.9853149    0.4419296    0.7504470
C     0.9853149    0.4419296   -0.7504470
H     0.5589582   -1.6127203    0.0000000
H     1.8709108    0.0851723    1.2728924
H     0.4320339    1.2263757    1.2618571
H     0.4320339    1.2263757   -1.2618571
H     1.8709108    0.0851723   -1.2728924

SCF energy GEOOPT =  -194.1292951041 H
ZPE =   231.1 kJ/mol
FREEH energy =   244.93 kJ/mol
FREEH entropy =   0.29943 kJ/mol/K

$vibrational spectrum
#  mode     symmetry     wave number   IR intensity    selection rules
#                         cm**(-1)        km/mol          IR     RAMAN
     1                        0.00         0.00000         -       -
     2                        0.00         0.00000         -       -
     3                        0.00         0.00000         -       -
     4                        0.00         0.00000         -       -
     5                        0.00         0.00000         -       -
     6                        0.00         0.00000         -       -
     7        a'            194.01         1.39950        YES     YES
     8        a"            200.39         1.62749        YES     YES
     9        a'            491.03         7.84414        YES     YES
    10        a"            502.40         0.96116        YES     YES
    11        a"            576.24        55.38173        YES     YES
    12        a'            638.17        46.98139        YES     YES
    13        a'            740.33         2.18504        YES     YES
    14        a'            793.18         0.95453        YES     YES
    15        a"            799.29         7.92883        YES     YES
    16        a"            861.42         1.72724        YES     YES
    17        a'            948.04        20.44498        YES     YES
    18        a'           1016.07         7.08727        YES     YES
    19        a"           1036.74         5.81888        YES     YES
    20        a"           1072.95         1.43513        YES     YES
    21        a'           1104.93         0.35992        YES     YES
    22        a"           1163.97         0.77694        YES     YES
    23        a'           1185.58         0.52862        YES     YES
    24        a'           1337.13         3.12190        YES     YES
    25        a"           1420.77         2.90184        YES     YES
    26        a'           1450.17         0.99458        YES     YES
    27        a'           2150.14        32.54722        YES     YES
```

28	a"	3071.44	16.74176	YES	YES
29	a'	3073.40	4.98742	YES	YES
30	a'	3077.10	6.83209	YES	YES
31	a"	3154.61	0.11165	YES	YES
32	a'	3169.51	9.21200	YES	YES
33	a'	3405.87	74.89504	YES	YES

$end

Double hybrid single point energy = -193.765731385910 H
COSMO energy + OC correction = -194.1351446828 H (in *o*DFB)

6.2.6 [{Ga(dcpe)}$_2$(HCCC$_3$H$_5$)]$^{2+}$

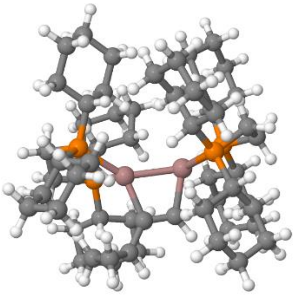

Method: (RI-)BP86(D3BJ)/def2-TZVPP
Symmetry: c1

Cartesian coordinates in Ångström:

Ga	-2.4757386	17.2482330	-2.7111462
Ga	-4.0653228	17.8416555	-0.9742568
P	-1.6232113	18.8844879	-4.2518510
P	-6.0520188	19.1902998	-0.8125894
P	-2.0361340	15.6067784	-4.3885897
P	-5.3065183	16.1961036	0.2639921
C	-5.0922555	20.9731535	-5.3049401
H	-5.2669110	21.4920130	-4.3461119
H	-6.0700144	20.6021877	-5.6432941
C	-1.0588376	14.1822544	-3.7494945
H	-0.8162823	13.5291975	-4.6042466
C	-4.2193203	15.3651721	1.4918713
H	-3.6043160	16.2112457	1.8473038
C	-4.3991076	16.0644342	-5.8031462
H	-3.8347658	16.5996254	-6.5840451
H	-4.6327305	16.7978575	-5.0157469
C	-6.8967885	19.2809399	-2.4522027
H	-6.2150025	19.8918346	-3.0663763
C	-7.2134220	18.2601783	0.2995513
H	-7.7877635	18.9757308	0.9024827
H	-7.9355784	17.7532581	-0.3542856
C	-3.1321013	22.4579217	-5.8815286
H	-2.7111224	23.1403930	-6.6318354
H	-3.2222225	23.0367016	-4.9473219
C	-2.1672018	21.2869028	-5.6523852
H	-1.1957185	21.6647786	-5.3060637
H	-1.9869014	20.7709108	-6.6096668
C	-4.1415757	19.7927071	-5.0724276
H	-4.0578035	19.2185035	-6.0089693

H	-4.5418554	19.1022127	-4.3140093
C	0.9985302	13.4929134	-2.4373836
H	1.3226914	12.7831577	-3.2154399
H	1.9120994	13.8712657	-1.9589193
C	-7.0201526	13.9064543	0.0414317
H	-7.5781934	14.3040177	0.9009391
H	-6.2526164	13.2286385	0.4447032
C	-5.8670952	20.9176675	-0.2084687
H	-6.8222947	21.4299271	-0.4124198
C	-0.2548179	20.4461517	-2.4343129
H	-0.9398402	21.2794415	-2.6481431
H	-0.7422504	19.8110068	-1.6769212
C	1.0647579	20.9871210	-1.8712660
H	1.4971019	21.7113671	-2.5807494
H	0.8637743	21.5398010	-0.9431748
C	-2.2389525	17.8886439	-0.0706494
C	-6.9972448	17.8884351	-3.0960998
H	-6.0005052	17.4218278	-3.1441380
H	-7.6230011	17.2382390	-2.4626023
C	-4.5166655	21.9695616	-6.3155129
H	-4.4414621	21.4827629	-7.3017719
H	-5.1994408	22.8206013	-6.4401971
C	-8.2673544	19.9766587	-2.4022768
H	-8.1761213	20.9878232	-1.9832106
H	-8.9417101	19.4144656	-1.7356498
C	-4.7490013	21.6024079	-1.0184965
H	-4.9844790	21.5806091	-2.0934459
H	-3.8143133	21.0289118	-0.8877821
C	-3.2136059	13.8655468	-6.2889988
H	-2.6300868	13.0447572	-5.8497926
H	-2.5933764	14.3075395	-7.0858770
C	2.3266508	19.0673515	-2.9072472
H	2.8011390	19.7253083	-3.6531850
H	3.0310184	18.2443841	-2.7235526
C	-5.3745855	22.4301142	1.7545758
H	-6.3151605	22.9906024	1.6297543
H	-5.1414943	22.4456194	2.8277055
C	-8.9947299	18.6627820	-4.4460057
H	-9.4116879	18.7435449	-5.4587903
H	-9.7018535	18.0457867	-3.8670476
C	-4.9074261	14.7357317	2.7139943
H	-5.5632922	15.4648525	3.2103371
H	-5.5426847	13.8962103	2.3981907
C	0.1151008	12.7686133	-1.4186788
H	0.6616117	11.9302090	-0.9668426
H	-0.1352136	13.4625416	-0.5984084
C	-7.6329922	17.9634905	-4.4880976
H	-6.9561223	18.5108807	-5.1639539
H	-7.7329929	16.9500126	-4.8991072
C	2.0683639	19.8583191	-1.6222479
H	1.6710467	19.1807587	-0.8477588
H	3.0105232	20.2653970	-1.2319231
C	-4.2626424	23.1145662	0.9532219
H	-4.1572607	24.1600639	1.2717671
H	-3.2994429	22.6246779	1.1713638
C	-5.5594608	14.4902085	-1.9524800
H	-4.7445698	13.8395560	-1.6014058
H	-5.0838966	15.3206159	-2.4966779

C	-5.5897907	20.9796880	1.3010831
H	-4.6904362	20.3894032	1.5323807
H	-6.4213479	20.5321886	1.8642725
C	-6.3326672	15.0371883	-0.7402185
H	-7.1224890	15.7024519	-1.1350630
C	-4.5379413	23.0477848	-0.5521284
C	-7.2086851	12.5724269	-2.1048651
H	-7.9032677	12.0423780	-2.7701584
H	-6.4648142	11.8291506	-1.7734699
C	-7.9565996	13.1171899	-0.8837365
H	-8.4240640	12.2970367	-0.3225489
H	-8.7760022	13.7757897	-1.2164801
C	-1.9414311	13.4119288	-2.7479316
H	-2.8379784	13.0191763	-3.2482377
H	-2.2896088	14.1201209	-1.9768315
C	-3.8449406	14.2289932	3.7002559
H	-3.2807442	15.0901075	4.0948752
H	-4.3382011	13.7567498	4.5605277
C	-5.6842139	15.5029719	-6.4222940
H	-6.2718382	16.3221993	-6.8586279
H	-6.3011300	15.0601441	-5.6233669
C	-2.8767400	13.2480944	3.0304697
H	-3.4270218	12.3404235	2.7316266
H	-2.1103180	12.9242828	3.7472693
C	-3.2627789	14.3798756	0.7978115
H	-3.8358322	13.5273053	0.4001665
H	-2.7699658	14.8755548	-0.0517162
C	-8.8892993	20.0486866	-3.8040438
H	-9.8787335	20.5210124	-3.7413118
H	-8.2693804	20.7034603	-4.4376985
C	-4.5087145	13.3172304	-6.9043399
H	-4.2627451	12.5808119	-7.6809763
H	-5.0735265	12.7755068	-6.1275375
C	-6.4943336	13.6931677	-2.8676859
H	-5.9264557	13.2766004	-3.7129472
H	-7.2417242	14.3788621	-3.3020006
C	-5.3779455	14.4383959	-7.4800488
H	-6.3122586	14.0256134	-7.8832724
H	-4.8535791	14.9080123	-8.3286765
C	-1.1747242	12.2690397	-2.0735534
H	-1.8257935	11.7836164	-1.3326473
H	-0.9313827	11.5011578	-2.8253078
C	-2.2168137	13.8689961	1.7948330
H	-1.5768442	14.7130961	2.1001020
H	-1.5606801	13.1387492	1.3008478
C	-1.7898612	18.2930885	1.2726022
C	-1.4385970	17.3064827	-1.0008804
C	-0.0208047	19.6101876	-3.7086577
H	0.3310614	20.2702980	-4.5195219
C	1.0241267	18.5016556	-3.4856812
H	1.2329273	17.9828261	-4.4328019
H	0.6112291	17.7545621	-2.7894995
C	0.2481436	14.6595417	-3.0907840
H	0.8961545	15.1530360	-3.8283524
H	0.0050968	15.4098768	-2.3218966
C	-1.0059567	16.4794448	-5.6536666
H	0.0312598	16.3680804	-5.3130862
H	-1.0830733	15.9506558	-6.6137717

```
C    -1.3422998   17.9725393   -5.8389890
H    -2.2648361   18.0844946   -6.4232153
H    -0.5434111   18.4674329   -6.4080616
C    -3.5293080   14.9335453   -5.2299584
H    -4.0963797   14.4586929   -4.4117992
C    -2.7533484   20.2891341   -4.6395973
H    -2.8698679   20.7984074   -3.6665693
C    -6.5068302   17.2374763    1.2067845
H    -7.2469711   16.6071431    1.7185427
H    -5.9271141   17.7492938    1.9881686
H    -5.4380731   23.6377036   -0.7888131
H    -3.7108091   23.4995331   -1.1173558
H    -0.8364743   17.8621433    1.5898470
C    -2.7779695   18.6115201    2.3781833
C    -2.0268294   19.7294793    1.7222464
H    -2.5640943   20.3719405    1.0270293
H    -1.2317448   20.2224196    2.2771666
H    -2.5204271   18.3363215    3.3988858
H    -3.8351840   18.5062549    2.1317631
H    -0.3964590   17.0495839   -0.7619430

SCF energy GEOOPT =  -7450.377337511 H
ZPE =   3838. kJ/mol
FREEH energy =  4027.98 kJ/mol
FREEH entropy =   1.60876 kJ/mol/K

$vibrational spectrum
#  mode     symmetry     wave number   IR intensity    selection rules
#                         cm**(-1)        km/mol          IR     RAMAN
     1                       -0.00         0.00000         -        -
     2                       -0.00         0.00000         -        -
     3                        0.00         0.00000         -        -
     4                        0.00         0.00000         -        -
     5                        0.00         0.00000         -        -
     6                        0.00         0.00000         -        -
     7         a             13.83         0.00960        YES      YES
     8         a             19.27         0.00019        YES      YES
     9         a             24.07         0.01285        YES      YES
    10         a             25.42         0.02064        YES      YES
    11         a             30.02         0.01028        YES      YES
    12         a             32.00         0.03532        YES      YES
    13         a             34.71         0.03614        YES      YES
    14         a             39.86         0.01175        YES      YES
    15         a             41.13         0.13570        YES      YES
    16         a             43.96         0.03518        YES      YES
    17         a             45.90         0.01412        YES      YES
    18         a             46.41         0.08010        YES      YES
    19         a             48.86         0.11645        YES      YES
    20         a             50.23         0.09911        YES      YES
    21         a             52.60         0.02668        YES      YES
    22         a             57.21         0.42082        YES      YES
    23         a             58.70         0.07567        YES      YES
    24         a             60.58         0.02312        YES      YES
    25         a             63.41         0.05150        YES      YES
    26         a             65.65         0.27149        YES      YES
    27         a             67.20         0.10318        YES      YES
    28         a             68.20         0.13599        YES      YES
    29         a             70.30         0.01824        YES      YES
```

30	a	72.75	0.05123	YES	YES
31	a	77.25	0.09314	YES	YES
32	a	80.41	0.09975	YES	YES
33	a	85.53	0.48363	YES	YES
34	a	90.30	0.29856	YES	YES
35	a	91.83	0.25467	YES	YES
36	a	97.97	0.15407	YES	YES
37	a	107.45	0.11449	YES	YES
38	a	117.35	1.59591	YES	YES
39	a	122.43	0.18172	YES	YES
40	a	130.29	0.59461	YES	YES
41	a	132.70	1.04868	YES	YES
42	a	135.72	0.71459	YES	YES
43	a	136.56	0.22997	YES	YES
44	a	140.18	0.09158	YES	YES
45	a	142.05	0.85446	YES	YES
46	a	147.03	1.57991	YES	YES
47	a	155.24	0.43138	YES	YES
48	a	159.81	0.86509	YES	YES
49	a	166.21	4.22422	YES	YES
50	a	173.96	0.62185	YES	YES
51	a	176.54	2.95004	YES	YES
52	a	179.82	0.60623	YES	YES
53	a	187.71	2.38952	YES	YES
54	a	194.37	1.59001	YES	YES
55	a	199.79	0.07052	YES	YES
56	a	212.08	0.24064	YES	YES
57	a	213.86	0.15057	YES	YES
58	a	219.50	0.28151	YES	YES
59	a	220.25	0.30793	YES	YES
60	a	223.24	0.37716	YES	YES
61	a	224.65	0.72179	YES	YES
62	a	226.87	0.24842	YES	YES
63	a	229.52	0.08512	YES	YES
64	a	230.83	0.17932	YES	YES
65	a	235.23	0.32792	YES	YES
66	a	238.75	4.70354	YES	YES
67	a	241.34	3.00309	YES	YES
68	a	243.52	6.70382	YES	YES
69	a	245.21	0.78892	YES	YES
70	a	249.64	1.63739	YES	YES
71	a	254.92	0.84787	YES	YES
72	a	263.34	2.83746	YES	YES
73	a	278.05	1.63375	YES	YES
74	a	280.66	0.10844	YES	YES
75	a	285.13	3.88628	YES	YES
76	a	294.81	0.20678	YES	YES
77	a	297.78	0.08692	YES	YES
78	a	305.27	0.33725	YES	YES
79	a	309.59	0.07004	YES	YES
80	a	313.51	0.12300	YES	YES
81	a	330.14	0.06657	YES	YES
82	a	330.64	0.89917	YES	YES
83	a	334.94	0.31849	YES	YES
84	a	336.33	0.28070	YES	YES
85	a	340.07	0.18560	YES	YES
86	a	344.30	0.76580	YES	YES
87	a	370.05	0.03528	YES	YES

88	a	374.95	1.62513	YES	YES
89	a	380.36	0.79713	YES	YES
90	a	382.61	2.67074	YES	YES
91	a	393.04	1.16050	YES	YES
92	a	394.68	0.06588	YES	YES
93	a	406.47	6.54967	YES	YES
94	a	410.94	0.79943	YES	YES
95	a	411.74	1.57526	YES	YES
96	a	414.03	2.16432	YES	YES
97	a	420.90	4.42824	YES	YES
98	a	424.70	2.63789	YES	YES
99	a	428.09	0.47782	YES	YES
100	a	430.12	0.01398	YES	YES
101	a	430.35	0.12212	YES	YES
102	a	430.81	0.24992	YES	YES
103	a	431.41	0.15837	YES	YES
104	a	431.70	0.47551	YES	YES
105	a	433.18	0.41847	YES	YES
106	a	435.66	1.81684	YES	YES
107	a	437.98	0.17807	YES	YES
108	a	445.35	0.61122	YES	YES
109	a	453.84	21.86624	YES	YES
110	a	456.89	10.72690	YES	YES
111	a	462.15	6.28153	YES	YES
112	a	472.11	3.96954	YES	YES
113	a	492.40	4.53112	YES	YES
114	a	494.08	1.92704	YES	YES
115	a	496.17	4.84756	YES	YES
116	a	497.36	0.91533	YES	YES
117	a	501.31	2.07755	YES	YES
118	a	503.53	0.52494	YES	YES
119	a	511.24	22.40132	YES	YES
120	a	514.86	9.79425	YES	YES
121	a	516.68	9.36567	YES	YES
122	a	622.72	26.86636	YES	YES
123	a	626.25	12.94094	YES	YES
124	a	630.01	18.96446	YES	YES
125	a	642.27	11.41750	YES	YES
126	a	656.37	12.72589	YES	YES
127	a	700.90	1.65354	YES	YES
128	a	718.65	1.02599	YES	YES
129	a	723.10	2.06286	YES	YES
130	a	727.66	2.62101	YES	YES
131	a	733.12	9.24385	YES	YES
132	a	736.64	3.93601	YES	YES
133	a	737.81	2.29119	YES	YES
134	a	738.45	2.54109	YES	YES
135	a	765.27	5.99367	YES	YES
136	a	770.45	0.36450	YES	YES
137	a	771.87	0.93471	YES	YES
138	a	773.60	1.37246	YES	YES
139	a	774.18	0.75442	YES	YES
140	a	775.08	0.13148	YES	YES
141	a	776.60	0.34429	YES	YES
142	a	776.73	3.39679	YES	YES
143	a	777.39	0.64708	YES	YES
144	a	777.62	11.03896	YES	YES
145	a	779.20	13.62549	YES	YES

146	a	793.53	12.94623	YES	YES
147	a	803.21	22.01088	YES	YES
148	a	807.61	0.13053	YES	YES
149	a	809.41	2.32168	YES	YES
150	a	809.64	10.08681	YES	YES
151	a	811.38	6.26455	YES	YES
152	a	811.95	0.65018	YES	YES
153	a	813.64	3.15742	YES	YES
154	a	815.39	1.82603	YES	YES
155	a	816.94	2.88968	YES	YES
156	a	830.22	4.36376	YES	YES
157	a	831.65	5.81222	YES	YES
158	a	835.71	7.88385	YES	YES
159	a	837.49	0.16594	YES	YES
160	a	838.25	1.61321	YES	YES
161	a	838.43	2.72638	YES	YES
162	a	839.00	7.11565	YES	YES
163	a	839.45	1.43828	YES	YES
164	a	848.33	17.64114	YES	YES
165	a	850.68	24.46209	YES	YES
166	a	852.67	8.03190	YES	YES
167	a	872.06	0.39868	YES	YES
168	a	872.15	3.64385	YES	YES
169	a	872.61	3.31246	YES	YES
170	a	876.69	0.41723	YES	YES
171	a	877.26	3.69474	YES	YES
172	a	877.59	1.22448	YES	YES
173	a	878.27	1.92314	YES	YES
174	a	878.47	4.85870	YES	YES
175	a	879.48	2.42680	YES	YES
176	a	880.07	4.54643	YES	YES
177	a	880.53	2.72546	YES	YES
178	a	881.30	1.92002	YES	YES
179	a	881.77	1.97747	YES	YES
180	a	883.58	5.70047	YES	YES
181	a	883.75	10.05065	YES	YES
182	a	885.89	4.69474	YES	YES
183	a	886.64	1.43387	YES	YES
184	a	902.96	1.31666	YES	YES
185	a	904.10	1.57417	YES	YES
186	a	904.64	2.62674	YES	YES
187	a	905.29	0.60639	YES	YES
188	a	907.87	1.28549	YES	YES
189	a	908.22	2.93096	YES	YES
190	a	910.27	1.39891	YES	YES
191	a	910.93	0.64703	YES	YES
192	a	961.04	16.93418	YES	YES
193	a	977.72	0.30154	YES	YES
194	a	979.45	0.06382	YES	YES
195	a	986.97	10.18269	YES	YES
196	a	987.93	5.69113	YES	YES
197	a	988.97	3.83580	YES	YES
198	a	989.31	14.84845	YES	YES
199	a	989.79	1.61015	YES	YES
200	a	990.18	1.49901	YES	YES
201	a	991.82	35.15092	YES	YES
202	a	992.73	9.41866	YES	YES
203	a	1003.96	5.97804	YES	YES

204	a	1013.63	0.37888	YES	YES
205	a	1016.08	1.11984	YES	YES
206	a	1016.42	0.59758	YES	YES
207	a	1017.16	0.39985	YES	YES
208	a	1018.04	0.64410	YES	YES
209	a	1019.18	0.28792	YES	YES
210	a	1020.91	0.56549	YES	YES
211	a	1022.57	0.41815	YES	YES
212	a	1026.52	3.76132	YES	YES
213	a	1030.32	0.96424	YES	YES
214	a	1031.39	0.61372	YES	YES
215	a	1032.05	2.19363	YES	YES
216	a	1037.29	0.26161	YES	YES
217	a	1038.87	2.68617	YES	YES
218	a	1039.52	1.11613	YES	YES
219	a	1040.17	0.74148	YES	YES
220	a	1041.71	1.54913	YES	YES
221	a	1056.57	0.63894	YES	YES
222	a	1059.39	1.07025	YES	YES
223	a	1059.61	0.58714	YES	YES
224	a	1060.94	2.05572	YES	YES
225	a	1061.77	2.36259	YES	YES
226	a	1063.82	0.54741	YES	YES
227	a	1065.66	0.23331	YES	YES
228	a	1068.11	1.53045	YES	YES
229	a	1071.18	0.21366	YES	YES
230	a	1071.86	0.13419	YES	YES
231	a	1072.75	0.33115	YES	YES
232	a	1072.80	0.36140	YES	YES
233	a	1073.09	0.28645	YES	YES
234	a	1073.62	0.23352	YES	YES
235	a	1074.21	0.04187	YES	YES
236	a	1074.71	0.00184	YES	YES
237	a	1076.19	4.31691	YES	YES
238	a	1079.90	1.42186	YES	YES
239	a	1081.18	0.29164	YES	YES
240	a	1083.67	11.72254	YES	YES
241	a	1091.64	4.24493	YES	YES
242	a	1092.04	6.25284	YES	YES
243	a	1093.05	6.97128	YES	YES
244	a	1097.13	1.87447	YES	YES
245	a	1101.77	0.72337	YES	YES
246	a	1103.54	2.76897	YES	YES
247	a	1109.95	8.64547	YES	YES
248	a	1116.19	8.41001	YES	YES
249	a	1120.08	1.05634	YES	YES
250	a	1129.65	4.53478	YES	YES
251	a	1159.60	1.75302	YES	YES
252	a	1160.29	4.68550	YES	YES
253	a	1161.04	0.76095	YES	YES
254	a	1161.43	10.07024	YES	YES
255	a	1166.70	8.80013	YES	YES
256	a	1167.41	3.23246	YES	YES
257	a	1168.64	12.48472	YES	YES
258	a	1169.46	10.24627	YES	YES
259	a	1170.72	5.22947	YES	YES
260	a	1173.13	1.17621	YES	YES
261	a	1175.25	7.57124	YES	YES

262	a	1176.80	11.51142	YES	YES
263	a	1179.14	4.45605	YES	YES
264	a	1183.27	5.90328	YES	YES
265	a	1187.61	0.64584	YES	YES
266	a	1189.65	1.71991	YES	YES
267	a	1192.19	0.78102	YES	YES
268	a	1195.78	3.42147	YES	YES
269	a	1215.50	2.32908	YES	YES
270	a	1236.21	0.40066	YES	YES
271	a	1240.95	0.33053	YES	YES
272	a	1242.49	0.51713	YES	YES
273	a	1245.96	0.18865	YES	YES
274	a	1246.45	0.29518	YES	YES
275	a	1246.81	0.15743	YES	YES
276	a	1248.60	1.31763	YES	YES
277	a	1250.13	0.31260	YES	YES
278	a	1251.12	1.46243	YES	YES
279	a	1251.78	0.63668	YES	YES
280	a	1252.25	0.22392	YES	YES
281	a	1255.00	4.69392	YES	YES
282	a	1256.21	0.66559	YES	YES
283	a	1256.24	0.43462	YES	YES
284	a	1256.68	10.54994	YES	YES
285	a	1257.25	3.90728	YES	YES
286	a	1257.50	1.10638	YES	YES
287	a	1258.36	2.58709	YES	YES
288	a	1258.86	0.40925	YES	YES
289	a	1259.91	1.91014	YES	YES
290	a	1260.91	0.60215	YES	YES
291	a	1261.38	4.81270	YES	YES
292	a	1262.46	1.94726	YES	YES
293	a	1263.80	0.61364	YES	YES
294	a	1266.08	2.42149	YES	YES
295	a	1267.87	1.02877	YES	YES
296	a	1270.62	2.76837	YES	YES
297	a	1271.16	1.62396	YES	YES
298	a	1280.80	1.77665	YES	YES
299	a	1282.80	0.95723	YES	YES
300	a	1285.00	4.90465	YES	YES
301	a	1285.82	2.60914	YES	YES
302	a	1287.93	5.01087	YES	YES
303	a	1288.34	1.41211	YES	YES
304	a	1290.49	2.40374	YES	YES
305	a	1293.02	3.32290	YES	YES
306	a	1307.36	0.63914	YES	YES
307	a	1310.49	2.37164	YES	YES
308	a	1311.14	0.51894	YES	YES
309	a	1311.38	2.63642	YES	YES
310	a	1312.09	0.96290	YES	YES
311	a	1312.71	0.94976	YES	YES
312	a	1313.33	1.85154	YES	YES
313	a	1316.53	1.76102	YES	YES
314	a	1318.53	0.26749	YES	YES
315	a	1320.06	2.57328	YES	YES
316	a	1321.57	0.35460	YES	YES
317	a	1322.12	0.69655	YES	YES
318	a	1322.44	0.17591	YES	YES
319	a	1323.69	1.36338	YES	YES

320	a	1323.99	1.99188	YES	YES
321	a	1324.42	0.27758	YES	YES
322	a	1325.23	0.08858	YES	YES
323	a	1325.37	0.90381	YES	YES
324	a	1325.63	0.82327	YES	YES
325	a	1325.76	1.47538	YES	YES
326	a	1326.14	6.59414	YES	YES
327	a	1328.47	7.13484	YES	YES
328	a	1329.37	1.00979	YES	YES
329	a	1330.40	2.39521	YES	YES
330	a	1333.21	1.99416	YES	YES
331	a	1336.31	0.10761	YES	YES
332	a	1336.54	0.60520	YES	YES
333	a	1336.65	0.91694	YES	YES
334	a	1337.43	0.10432	YES	YES
335	a	1337.84	0.03991	YES	YES
336	a	1338.11	0.35143	YES	YES
337	a	1338.63	0.07470	YES	YES
338	a	1339.54	0.31845	YES	YES
339	a	1343.77	0.28517	YES	YES
340	a	1345.04	1.63746	YES	YES
341	a	1345.83	0.20960	YES	YES
342	a	1346.09	0.13541	YES	YES
343	a	1347.27	2.14556	YES	YES
344	a	1347.41	0.94857	YES	YES
345	a	1348.59	0.85388	YES	YES
346	a	1350.26	0.97501	YES	YES
347	a	1400.60	5.18812	YES	YES
348	a	1403.96	5.97110	YES	YES
349	a	1404.08	10.11223	YES	YES
350	a	1405.56	7.87667	YES	YES
351	a	1412.76	5.04213	YES	YES
352	a	1425.78	3.42864	YES	YES
353	a	1428.88	6.49164	YES	YES
354	a	1429.65	0.89834	YES	YES
355	a	1430.34	6.02826	YES	YES
356	a	1430.79	1.87469	YES	YES
357	a	1431.60	1.29854	YES	YES
358	a	1432.18	3.56750	YES	YES
359	a	1433.25	2.52423	YES	YES
360	a	1435.34	3.37201	YES	YES
361	a	1436.14	6.02185	YES	YES
362	a	1436.43	3.35447	YES	YES
363	a	1437.20	8.21757	YES	YES
364	a	1437.33	4.56603	YES	YES
365	a	1437.66	0.10774	YES	YES
366	a	1438.02	16.47081	YES	YES
367	a	1438.11	0.42172	YES	YES
368	a	1438.43	3.45556	YES	YES
369	a	1439.01	8.46649	YES	YES
370	a	1439.20	1.07756	YES	YES
371	a	1439.37	10.94772	YES	YES
372	a	1439.51	9.61186	YES	YES
373	a	1440.29	0.33405	YES	YES
374	a	1440.96	2.93794	YES	YES
375	a	1441.18	4.55381	YES	YES
376	a	1442.88	14.48813	YES	YES
377	a	1443.37	9.79893	YES	YES

378	a	1443.46	9.30311	YES	YES
379	a	1443.72	14.02779	YES	YES
380	a	1444.61	11.25344	YES	YES
381	a	1445.27	12.20184	YES	YES
382	a	1446.08	1.57378	YES	YES
383	a	1446.47	8.75985	YES	YES
384	a	1447.03	9.45148	YES	YES
385	a	1452.96	2.01768	YES	YES
386	a	1454.02	1.10412	YES	YES
387	a	1454.04	2.24916	YES	YES
388	a	1455.19	3.45526	YES	YES
389	a	1455.97	0.89676	YES	YES
390	a	1456.25	0.59531	YES	YES
391	a	1456.98	1.66852	YES	YES
392	a	1457.43	4.22030	YES	YES
393	a	1496.35	27.45574	YES	YES
394	a	2921.12	4.34691	YES	YES
395	a	2926.60	9.26967	YES	YES
396	a	2929.14	29.51824	YES	YES
397	a	2934.55	2.37943	YES	YES
398	a	2935.42	23.25792	YES	YES
399	a	2937.28	11.90585	YES	YES
400	a	2937.44	7.31816	YES	YES
401	a	2939.14	11.90830	YES	YES
402	a	2942.15	4.08589	YES	YES
403	a	2942.35	30.73751	YES	YES
404	a	2942.72	4.51313	YES	YES
405	a	2942.83	16.58556	YES	YES
406	a	2944.04	3.47053	YES	YES
407	a	2944.36	6.03720	YES	YES
408	a	2945.43	10.01771	YES	YES
409	a	2946.01	9.99865	YES	YES
410	a	2946.31	11.75869	YES	YES
411	a	2947.96	9.34052	YES	YES
412	a	2948.80	14.23071	YES	YES
413	a	2949.07	3.06583	YES	YES
414	a	2949.24	16.22504	YES	YES
415	a	2950.30	1.21053	YES	YES
416	a	2950.83	12.04129	YES	YES
417	a	2951.24	17.55054	YES	YES
418	a	2952.90	6.96013	YES	YES
419	a	2953.62	5.11691	YES	YES
420	a	2953.77	9.83545	YES	YES
421	a	2955.26	6.89968	YES	YES
422	a	2955.35	5.43219	YES	YES
423	a	2956.00	6.94049	YES	YES
424	a	2956.41	5.14072	YES	YES
425	a	2956.93	1.90262	YES	YES
426	a	2957.38	9.57626	YES	YES
427	a	2957.56	9.54816	YES	YES
428	a	2958.24	21.50996	YES	YES
429	a	2958.48	22.60510	YES	YES
430	a	2959.83	41.87217	YES	YES
431	a	2959.96	39.88728	YES	YES
432	a	2960.55	21.94560	YES	YES
433	a	2961.10	40.64456	YES	YES
434	a	2962.29	28.45909	YES	YES
435	a	2962.59	25.52496	YES	YES

436	a	2963.36	8.55092	YES	YES
437	a	2965.26	21.99912	YES	YES
438	a	2965.34	17.86916	YES	YES
439	a	2966.80	0.14631	YES	YES
440	a	2969.68	12.90194	YES	YES
441	a	2975.77	9.77234	YES	YES
442	a	2981.03	5.81449	YES	YES
443	a	2981.53	14.95928	YES	YES
444	a	2981.63	45.51620	YES	YES
445	a	2985.80	27.72329	YES	YES
446	a	2986.67	3.05706	YES	YES
447	a	2988.45	37.14291	YES	YES
448	a	2989.16	7.33568	YES	YES
449	a	2989.61	3.02786	YES	YES
450	a	2990.34	4.81962	YES	YES
451	a	2990.64	48.70715	YES	YES
452	a	2997.78	17.10712	YES	YES
453	a	2999.20	34.32927	YES	YES
454	a	3000.30	33.14459	YES	YES
455	a	3001.43	12.99461	YES	YES
456	a	3002.33	25.39084	YES	YES
457	a	3003.68	14.89554	YES	YES
458	a	3005.23	21.15266	YES	YES
459	a	3007.90	14.75811	YES	YES
460	a	3008.05	20.41656	YES	YES
461	a	3008.62	25.21977	YES	YES
462	a	3009.05	15.27233	YES	YES
463	a	3009.20	25.18603	YES	YES
464	a	3009.97	14.56281	YES	YES
465	a	3010.82	11.69681	YES	YES
466	a	3011.21	29.47897	YES	YES
467	a	3011.34	17.58817	YES	YES
468	a	3011.92	34.04993	YES	YES
469	a	3012.54	12.36376	YES	YES
470	a	3013.06	20.20372	YES	YES
471	a	3014.09	17.14155	YES	YES
472	a	3014.69	19.70028	YES	YES
473	a	3015.74	35.12652	YES	YES
474	a	3015.84	26.31170	YES	YES
475	a	3015.94	13.02755	YES	YES
476	a	3016.39	30.20828	YES	YES
477	a	3016.48	26.72726	YES	YES
478	a	3017.40	30.31877	YES	YES
479	a	3017.54	30.26843	YES	YES
480	a	3017.77	25.87916	YES	YES
481	a	3017.83	22.24303	YES	YES
482	a	3017.94	6.78557	YES	YES
483	a	3018.07	37.44029	YES	YES
484	a	3018.68	26.69681	YES	YES
485	a	3020.34	28.29970	YES	YES
486	a	3021.87	33.24974	YES	YES
487	a	3026.87	2.54915	YES	YES
488	a	3030.55	0.46655	YES	YES
489	a	3038.73	1.33430	YES	YES
490	a	3044.59	0.50792	YES	YES
491	a	3049.24	7.49962	YES	YES
492	a	3051.06	15.45352	YES	YES
493	a	3071.00	5.66412	YES	YES

```
   494         a             3142.77          2.52553       YES      YES
   495         a             3161.36          4.77742       YES      YES
$end
```

Double hybrid single point energy = -7442.885925002109 H
COSMO energy + OC correction = -7450.5235382757 H (in *o*DFB)

6.2.7 C_6H_{10}

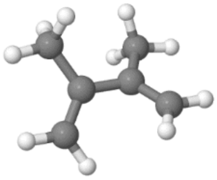

Method: (RI-)BP86(D3BJ)/def2-TZVPP
Symmetry: c2

Cartesian coordinates in Ångström:

C	-0.7326713	-0.1241313	0.2400331
C	0.7326713	0.1241313	0.2400331
C	-1.3477486	-0.6658435	1.3045998
C	-1.4941578	0.2353353	-1.0114284
H	-0.7870871	-0.9724776	2.1868274
H	-2.4259748	-0.8287519	1.3120231
C	1.3477486	0.6658435	1.3045998
C	1.4941578	-0.2353353	-1.0114284
H	0.7870871	0.9724776	2.1868274
H	2.4259748	0.8287519	1.3120231
H	-1.1680258	-0.3741357	-1.8674761
H	-1.3264762	1.2869564	-1.2892828
H	-2.5706840	0.0785652	-0.8752504
H	1.3264762	-1.2869564	-1.2892828
H	2.5706840	-0.0785652	-0.8752504
H	1.1680258	0.3741357	-1.8674761

SCF energy GEOOPT = -234.7340258129 H
ZPE = 359.9 kJ/mol
FREEH energy = 379.48 kJ/mol
FREEH entropy = 0.33996 kJ/mol/K

```
$vibrational spectrum
#  mode     symmetry     wave number   IR intensity    selection rules
#                         cm**(-1)        km/mol          IR     RAMAN
     1                       -0.00          0.00000        -        -
     2                       -0.00          0.00000        -        -
     3                        0.00          0.00000        -        -
     4                        0.00          0.00000        -        -
     5                        0.00          0.00000        -        -
     6                        0.00          0.00000        -        -
     7         a             78.62          0.00018       YES      YES
     8         b            176.14          0.12493       YES      YES
     9         a            206.75          0.24604       YES      YES
    10         a            257.48          0.00303       YES      YES
    11         b            276.17          5.84921       YES      YES
    12         a            358.38          0.63935       YES      YES
```

13	b	444.07	0.58020	YES	YES
14	b	514.18	8.76247	YES	YES
15	a	550.22	3.68092	YES	YES
16	b	685.14	0.68633	YES	YES
17	a	712.13	0.09922	YES	YES
18	a	757.18	1.94561	YES	YES
19	b	884.56	71.81242	YES	YES
20	a	885.07	9.05260	YES	YES
21	b	910.07	0.20203	YES	YES
22	a	952.51	2.37956	YES	YES
23	a	977.23	4.25876	YES	YES
24	b	1001.35	2.32254	YES	YES
25	b	1027.74	0.02229	YES	YES
26	a	1036.47	0.02025	YES	YES
27	b	1182.34	6.44214	YES	YES
28	a	1285.51	3.15694	YES	YES
29	b	1353.23	5.55225	YES	YES
30	a	1360.76	6.56987	YES	YES
31	b	1387.38	1.21752	YES	YES
32	a	1401.40	1.59209	YES	YES
33	b	1427.48	16.70381	YES	YES
34	a	1431.50	3.87081	YES	YES
35	b	1445.66	16.48187	YES	YES
36	a	1452.24	4.83373	YES	YES
37	a	1622.51	17.91000	YES	YES
38	b	1648.53	11.46982	YES	YES
39	b	2955.73	7.57400	YES	YES
40	a	2957.36	36.69059	YES	YES
41	b	3005.02	25.51495	YES	YES
42	a	3007.77	2.91476	YES	YES
43	b	3049.44	37.60871	YES	YES
44	a	3050.71	1.24401	YES	YES
45	b	3067.55	13.08597	YES	YES
46	a	3069.05	5.46481	YES	YES
47	b	3155.29	19.86228	YES	YES
48	a	3156.44	3.88690	YES	YES

$end

Double hybrid single point energy = -234.285983195144 H
COSMO energy + OC correction = -234.7372988397 H (in *o*DFB)

6.2.8 [{Ga(dcpe)}$_2$(C$_6$H$_{10}$)]$^{2+}$

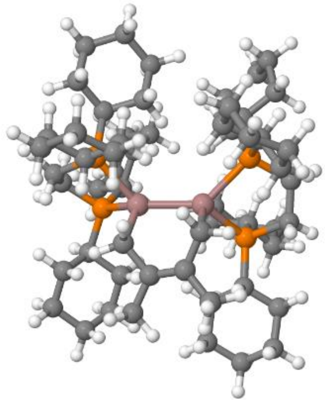

Method: (RI-)BP86(D3BJ)/def2-TZVPP

Symmetry: c1

Cartesian coordinates in Ångström:

Ga	12.6601144	9.8744342	5.2774439
Ga	10.8479666	8.2781817	5.4641419
P	15.0005722	9.8753690	5.8721927
P	12.6433176	12.1215292	6.1802405
P	11.0977186	5.8950774	5.2754690
P	9.2188250	7.7696855	7.1872097
C	6.8140916	8.1563306	5.7495857
H	6.5659380	7.0913600	5.8622400
H	7.4273168	8.2510253	4.8420640
C	11.0462054	3.8759374	3.2261026
H	11.5707852	3.1435024	3.8544833
H	9.9711772	3.7510040	3.4172739
C	11.9245232	9.2764073	2.3931455
C	13.2754858	5.2232039	1.7882246
H	12.7446289	5.9446296	1.1480906
H	14.3479704	5.3603588	1.5902029
C	12.3475495	3.5831456	6.4137761
H	12.9256003	3.3806306	5.4999988
H	11.3806360	3.0732302	6.2994528
C	14.5349992	9.3122023	8.5645502
H	14.6659384	10.3836942	8.7865740
H	13.4797533	9.1754410	8.2799229
C	11.7747477	14.7357069	5.2863243
H	11.5439580	15.0945758	6.2981588
H	12.8283549	14.9796258	5.0912848
C	10.8703472	15.4560524	4.2748696
H	11.1723365	15.1725589	3.2531612
C	16.9367047	9.0492336	7.7732108
H	17.5771782	8.7071443	6.9495350
H	17.1845757	10.1083800	7.9517882
C	10.7397152	8.6274944	2.5583625
C	15.4079727	11.6430525	6.2807316
H	16.3973935	11.8795072	5.8673520
H	15.5043634	11.7034046	7.3725963
C	8.8975460	5.9468552	7.0118265
H	9.4079950	5.4525451	7.8487759
C	12.1523875	5.1006477	6.5658181
H	11.5708258	5.2686240	7.4887809
C	14.3640802	12.6542295	5.7836369
H	14.5682269	13.6494499	6.2015352
H	14.4040633	12.7482633	4.6887325
C	7.6143031	8.6542191	6.9636517
H	7.0220537	8.4527994	7.8717248
C	9.6943217	8.0182742	8.9574276
H	9.7892392	9.1123797	9.0563335
C	16.1720537	9.3386518	4.5562850
H	17.1753377	9.3735403	5.0139169
C	12.8342383	3.8014109	1.4289200
H	13.4401667	3.0748490	1.9955760
H	13.0249903	3.6030546	0.3658096
C	13.1069666	3.0197660	7.6218200
H	13.2586918	1.9400094	7.4907796
H	12.4911183	3.1421046	8.5283969
C	12.3377001	10.3831981	3.3173159
H	13.1802927	10.9494303	2.9035557

H	11.5008379	11.0915171	3.4259768
C	9.3937064	15.1047850	4.4882517
H	8.7723548	15.6099525	3.7369099
H	9.0670088	15.4856715	5.4701533
C	14.2578856	5.2458182	7.9461183
H	13.6979128	5.4634940	8.8713654
H	15.2325151	5.7450409	8.0470522
C	16.8813908	8.3250922	1.8703356
H	15.9129679	8.2770489	1.3462282
H	17.6381138	7.9812887	1.1527275
C	11.3516679	3.5855505	1.7492915
H	10.7389129	4.2471312	1.1154394
H	11.0510793	2.5564902	1.5105220
C	12.8464090	9.0539960	1.2255860
H	13.8772598	8.8921681	1.5769178
H	12.5722602	8.1972664	0.6030456
H	12.8792228	9.9408973	0.5731022
C	16.1661218	10.2709054	3.3351334
H	16.4133457	11.3013908	3.6292232
H	15.1554612	10.2929603	2.9018031
C	12.4465673	12.3137884	8.0037285
H	13.3892272	11.8845637	8.3880166
C	12.3412225	13.7538363	8.5291909
H	11.4025683	14.2012728	8.1698649
H	13.1608089	14.3757992	8.1426570
C	11.0650865	7.3908054	9.2525565
H	11.0063066	6.3022498	9.0894019
C	9.3769824	5.3573404	5.6741575
H	9.3100043	4.2612219	5.6982908
H	8.7440263	5.7041582	4.8449503
C	12.9794807	5.5486752	3.2561014
H	13.2348886	6.5958406	3.4750007
H	13.6102573	4.9151295	3.8988634
C	8.6295191	7.5257194	9.9532792
H	8.4708747	6.4435113	9.8168984
H	7.6642839	8.0146384	9.7666696
C	7.8993992	10.1636996	6.8773701
H	8.5741422	10.3405057	6.0242080
H	8.4305486	10.5072369	7.7772667
C	10.0642577	12.8548105	5.4294536
H	9.7770213	13.1307626	6.4566276
H	9.9269276	11.7658348	5.3445230
C	5.8319250	10.4737974	5.4579963
H	4.8988667	11.0416971	5.3463286
H	6.4241214	10.6578887	4.5456818
C	17.2478573	8.2409277	9.0402541
H	18.3062306	8.3688635	9.3039226
H	17.1065466	7.1684084	8.8274415
C	16.8449979	7.3932882	3.0851613
H	17.8501247	7.3295261	3.5324094
H	16.5742510	6.3723637	2.7810928
C	14.8681394	8.5214202	9.8340748
H	14.6233660	7.4604108	9.6739708
H	14.2310325	8.8709407	10.6576600
C	16.3470975	8.6545245	10.2060255
H	16.5629516	9.7005103	10.4806149
H	16.5702093	8.0484760	11.0941993
C	11.2887332	11.4584062	10.0747048

H	12.2068472	10.9729570	10.4468787
H	10.4454998	10.8605331	10.4507246
C	5.5290358	8.9764677	5.5724581
H	4.8636736	8.8012835	6.4333785
H	4.9900688	8.6217518	4.6836366
C	11.3041155	11.4366320	8.5427192
H	11.4132685	10.4094281	8.1609481
H	10.3437636	11.8134599	8.1579616
C	11.4951294	5.3042791	3.5806986
H	10.9154040	6.0013427	2.9577577
C	12.3416296	13.7585372	10.0636527
H	12.2483827	14.7897911	10.4295303
H	13.3123044	13.3831629	10.4282092
C	13.4928520	5.8292658	6.7534115
H	13.3076820	6.9060063	6.8886232
H	14.0990716	5.7286166	5.8407445
C	6.6121266	10.9705071	6.6781878
H	6.8608911	12.0362911	6.5742636
H	5.9835925	10.8826545	7.5789698
C	14.4489004	3.7318046	7.8137790
H	15.0998108	3.5209533	6.9492211
H	14.9673404	3.3367389	8.6976012
C	11.2114968	12.8877490	10.6211733
H	11.2456149	12.8759143	11.7187417
H	10.2408894	13.3307738	10.3435024
C	10.4320924	7.1507739	11.6932252
H	10.7437227	7.3739336	12.7223265
H	10.3411380	6.0538280	11.6264577
C	15.8570794	7.8909851	4.1468952
H	14.8313421	7.8600117	3.7427995
H	15.8740312	7.2245744	5.0217890
C	11.4957526	7.6329581	10.7033993
H	11.6752247	8.7091959	10.8511175
H	12.4518989	7.1257609	10.8896272
C	9.0732174	7.7897243	11.3986679
H	8.3089912	7.4110584	12.0905051
H	9.1331329	8.8781431	11.5640580
C	17.1653406	9.7742040	2.2805806
H	17.1310571	10.4348508	1.4038025
H	18.1864384	9.8479703	2.6885006
C	9.8667664	8.8675360	3.7544884
H	9.6578767	9.9361988	3.9260300
H	8.9006891	8.3674025	3.6353180
C	15.4510184	8.9234639	7.3933205
H	15.2431450	7.8744994	7.1293173
C	11.5398280	13.2182937	5.1961173
H	11.7935029	12.9137629	4.1643503
C	9.1641084	13.5905649	4.4312812
H	9.3812709	13.2215294	3.4152476
H	8.1107561	13.3503312	4.6335878
H	11.8180020	7.7764976	8.5475243
H	7.8245387	5.7595663	7.1489767
C	10.1336568	7.7264304	1.5080558
H	10.8669713	7.2431289	0.8536952
H	9.4593628	8.3108773	0.8610262
H	9.5085214	6.9396359	1.9551324
H	11.0226184	16.5407134	4.3562086

```
SCF energy GEOOPT =  -7490.973352785 H
ZPE =    3967. kJ/mol
FREEH energy =  4162.64 kJ/mol
FREEH entropy =   1.64570 kJ/mol/K

$vibrational spectrum
#  mode     symmetry     wave number   IR intensity    selection rules
#                         cm**(-1)        km/mol          IR     RAMAN
     1                      -0.00         0.00000          -       -
     2                      -0.00         0.00000          -       -
     3                      -0.00         0.00000          -       -
     4                       0.00         0.00000          -       -
     5                       0.00         0.00000          -       -
     6                       0.00         0.00000          -       -
     7         a            16.49         0.09374         YES     YES
     8         a            21.66         0.01143         YES     YES
     9         a            24.04         0.02387         YES     YES
    10         a            25.92         0.01705         YES     YES
    11         a            29.59         0.01135         YES     YES
    12         a            31.67         0.07051         YES     YES
    13         a            34.92         0.04518         YES     YES
    14         a            36.34         0.03290         YES     YES
    15         a            39.02         0.00360         YES     YES
    16         a            40.96         0.02259         YES     YES
    17         a            42.44         0.15281         YES     YES
    18         a            44.50         0.05784         YES     YES
    19         a            45.26         0.02585         YES     YES
    20         a            49.41         0.20735         YES     YES
    21         a            51.77         0.04295         YES     YES
    22         a            54.40         0.01055         YES     YES
    23         a            58.01         0.05446         YES     YES
    24         a            58.84         0.00701         YES     YES
    25         a            62.25         0.22735         YES     YES
    26         a            66.60         0.33690         YES     YES
    27         a            70.79         0.04763         YES     YES
    28         a            72.48         0.05365         YES     YES
    29         a            76.90         0.00052         YES     YES
    30         a            78.62         0.06519         YES     YES
    31         a            80.13         0.04335         YES     YES
    32         a            86.62         0.03243         YES     YES
    33         a            87.49         0.05855         YES     YES
    34         a            91.90         0.38483         YES     YES
    35         a            93.31         0.25461         YES     YES
    36         a           103.59         0.30471         YES     YES
    37         a           108.45         1.47250         YES     YES
    38         a           110.24         0.53474         YES     YES
    39         a           113.14         0.32487         YES     YES
    40         a           123.07         0.38844         YES     YES
    41         a           123.22         0.11254         YES     YES
    42         a           131.46         1.64917         YES     YES
    43         a           134.97         1.77575         YES     YES
    44         a           136.93         0.25516         YES     YES
    45         a           141.06         0.42402         YES     YES
    46         a           144.28         1.12166         YES     YES
    47         a           148.14         1.68958         YES     YES
    48         a           150.70         1.24861         YES     YES
    49         a           156.45         3.06318         YES     YES
    50         a           159.65         0.79027         YES     YES
```

51	a	166.07	3.36006	YES	YES
52	a	173.58	1.17828	YES	YES
53	a	176.99	1.54698	YES	YES
54	a	179.48	0.71956	YES	YES
55	a	188.61	5.73690	YES	YES
56	a	191.88	1.62359	YES	YES
57	a	195.70	0.99107	YES	YES
58	a	212.32	0.47633	YES	YES
59	a	213.33	0.14964	YES	YES
60	a	214.17	0.33292	YES	YES
61	a	217.04	0.27417	YES	YES
62	a	222.16	1.36390	YES	YES
63	a	222.82	0.39081	YES	YES
64	a	227.00	0.18364	YES	YES
65	a	228.55	0.03574	YES	YES
66	a	230.73	0.03191	YES	YES
67	a	234.94	0.56258	YES	YES
68	a	235.91	0.05115	YES	YES
69	a	240.19	0.56828	YES	YES
70	a	241.76	1.27969	YES	YES
71	a	243.37	0.42524	YES	YES
72	a	248.71	0.52500	YES	YES
73	a	271.11	0.19203	YES	YES
74	a	281.65	0.23630	YES	YES
75	a	283.61	0.33930	YES	YES
76	a	285.01	0.81799	YES	YES
77	a	289.92	0.63383	YES	YES
78	a	295.28	0.11709	YES	YES
79	a	300.62	0.27111	YES	YES
80	a	303.70	1.68162	YES	YES
81	a	311.67	0.22499	YES	YES
82	a	312.74	0.46585	YES	YES
83	a	318.87	5.86196	YES	YES
84	a	331.63	0.62128	YES	YES
85	a	333.59	0.10094	YES	YES
86	a	335.38	0.56167	YES	YES
87	a	339.15	0.06041	YES	YES
88	a	344.25	0.24878	YES	YES
89	a	345.71	0.61578	YES	YES
90	a	365.30	2.65412	YES	YES
91	a	371.53	0.26364	YES	YES
92	a	373.38	0.56479	YES	YES
93	a	377.84	1.12042	YES	YES
94	a	381.32	2.82627	YES	YES
95	a	384.87	0.17145	YES	YES
96	a	395.18	1.61241	YES	YES
97	a	397.02	0.41912	YES	YES
98	a	409.18	0.12341	YES	YES
99	a	409.78	0.69686	YES	YES
100	a	416.36	1.98028	YES	YES
101	a	418.36	1.05075	YES	YES
102	a	424.03	6.05493	YES	YES
103	a	425.17	0.05411	YES	YES
104	a	429.17	0.05034	YES	YES
105	a	430.03	0.03315	YES	YES
106	a	430.24	0.07392	YES	YES
107	a	431.09	0.02063	YES	YES
108	a	433.10	0.08789	YES	YES

109	a	434.95	0.75053	YES	YES
110	a	438.34	0.47030	YES	YES
111	a	439.23	0.44949	YES	YES
112	a	454.92	5.45367	YES	YES
113	a	457.98	4.48553	YES	YES
114	a	469.20	2.93496	YES	YES
115	a	470.96	3.55929	YES	YES
116	a	480.47	0.46177	YES	YES
117	a	493.10	3.76144	YES	YES
118	a	494.74	3.65226	YES	YES
119	a	495.56	1.24302	YES	YES
120	a	497.27	2.39963	YES	YES
121	a	502.62	1.05520	YES	YES
122	a	504.62	2.61864	YES	YES
123	a	512.81	10.52460	YES	YES
124	a	513.74	8.25779	YES	YES
125	a	516.88	0.59686	YES	YES
126	a	532.16	4.47593	YES	YES
127	a	623.25	10.77864	YES	YES
128	a	632.17	20.59625	YES	YES
129	a	633.20	9.75284	YES	YES
130	a	658.54	7.43428	YES	YES
131	a	659.68	8.50201	YES	YES
132	a	663.93	22.21521	YES	YES
133	a	681.18	33.94316	YES	YES
134	a	700.91	2.07137	YES	YES
135	a	703.35	1.03841	YES	YES
136	a	716.07	4.20802	YES	YES
137	a	720.44	2.51727	YES	YES
138	a	723.33	2.29505	YES	YES
139	a	728.17	2.30734	YES	YES
140	a	729.32	2.69660	YES	YES
141	a	736.00	3.66957	YES	YES
142	a	737.22	5.82319	YES	YES
143	a	769.17	0.10314	YES	YES
144	a	771.06	0.14885	YES	YES
145	a	773.92	0.40040	YES	YES
146	a	774.20	0.09924	YES	YES
147	a	774.25	0.07130	YES	YES
148	a	774.48	0.73095	YES	YES
149	a	775.23	0.13010	YES	YES
150	a	777.39	0.01490	YES	YES
151	a	781.68	25.57763	YES	YES
152	a	782.20	5.93954	YES	YES
153	a	806.63	1.65394	YES	YES
154	a	807.79	1.87141	YES	YES
155	a	809.73	5.05624	YES	YES
156	a	810.17	2.39380	YES	YES
157	a	811.03	5.98412	YES	YES
158	a	812.08	3.88418	YES	YES
159	a	815.39	3.59286	YES	YES
160	a	817.30	2.58944	YES	YES
161	a	830.36	4.49551	YES	YES
162	a	831.71	6.29328	YES	YES
163	a	836.60	5.64274	YES	YES
164	a	837.01	10.41758	YES	YES
165	a	837.79	0.72918	YES	YES
166	a	838.36	0.51575	YES	YES

167	a	839.07	0.59615	YES	YES
168	a	839.32	0.68814	YES	YES
169	a	854.93	19.73811	YES	YES
170	a	856.07	9.22351	YES	YES
171	a	873.75	1.00990	YES	YES
172	a	874.64	4.26720	YES	YES
173	a	875.59	0.18762	YES	YES
174	a	876.58	0.82395	YES	YES
175	a	876.77	0.61646	YES	YES
176	a	877.40	3.93083	YES	YES
177	a	877.86	2.33912	YES	YES
178	a	877.94	2.97581	YES	YES
179	a	879.58	7.74743	YES	YES
180	a	879.99	0.62392	YES	YES
181	a	881.44	4.61192	YES	YES
182	a	882.29	2.16019	YES	YES
183	a	882.94	3.91896	YES	YES
184	a	884.13	1.97275	YES	YES
185	a	886.89	6.29510	YES	YES
186	a	887.16	3.56774	YES	YES
187	a	903.41	0.55577	YES	YES
188	a	904.23	0.57097	YES	YES
189	a	904.57	3.52165	YES	YES
190	a	905.31	1.24064	YES	YES
191	a	908.44	3.66251	YES	YES
192	a	909.24	5.61807	YES	YES
193	a	909.37	1.59976	YES	YES
194	a	909.96	1.35123	YES	YES
195	a	910.37	2.05440	YES	YES
196	a	970.72	1.21381	YES	YES
197	a	979.75	0.52171	YES	YES
198	a	981.02	5.37688	YES	YES
199	a	985.01	2.16231	YES	YES
200	a	987.27	9.48155	YES	YES
201	a	987.78	4.04935	YES	YES
202	a	988.26	7.37263	YES	YES
203	a	988.53	11.07148	YES	YES
204	a	989.34	38.05776	YES	YES
205	a	989.70	11.74312	YES	YES
206	a	990.50	1.11015	YES	YES
207	a	991.15	2.37618	YES	YES
208	a	1001.24	5.70401	YES	YES
209	a	1011.59	31.62966	YES	YES
210	a	1012.65	0.50378	YES	YES
211	a	1014.03	0.16444	YES	YES
212	a	1016.06	0.07327	YES	YES
213	a	1016.70	0.15509	YES	YES
214	a	1017.68	0.82640	YES	YES
215	a	1018.58	0.75064	YES	YES
216	a	1019.22	1.18483	YES	YES
217	a	1019.80	0.08600	YES	YES
218	a	1032.29	2.92118	YES	YES
219	a	1032.59	0.28180	YES	YES
220	a	1036.45	0.41773	YES	YES
221	a	1037.13	0.76184	YES	YES
222	a	1038.46	0.74432	YES	YES
223	a	1038.64	3.94980	YES	YES
224	a	1039.88	0.66286	YES	YES

225	a	1040.66	0.25868	YES	YES
226	a	1055.23	4.54768	YES	YES
227	a	1055.60	1.34447	YES	YES
228	a	1058.04	5.83314	YES	YES
229	a	1059.54	0.96587	YES	YES
230	a	1060.10	1.84784	YES	YES
231	a	1060.49	0.23251	YES	YES
232	a	1060.90	2.72514	YES	YES
233	a	1064.35	3.84015	YES	YES
234	a	1064.77	0.02165	YES	YES
235	a	1069.76	0.02489	YES	YES
236	a	1070.54	0.09987	YES	YES
237	a	1072.12	0.19204	YES	YES
238	a	1072.40	0.16727	YES	YES
239	a	1072.60	0.03640	YES	YES
240	a	1073.09	0.34782	YES	YES
241	a	1074.20	0.95049	YES	YES
242	a	1074.95	0.30684	YES	YES
243	a	1077.61	2.88534	YES	YES
244	a	1078.63	0.50528	YES	YES
245	a	1079.40	4.26020	YES	YES
246	a	1085.26	1.07815	YES	YES
247	a	1093.41	2.97799	YES	YES
248	a	1094.06	2.10815	YES	YES
249	a	1095.89	4.98235	YES	YES
250	a	1098.13	8.81324	YES	YES
251	a	1102.58	3.28271	YES	YES
252	a	1103.66	2.61434	YES	YES
253	a	1114.05	12.51638	YES	YES
254	a	1115.50	4.83374	YES	YES
255	a	1122.97	1.65746	YES	YES
256	a	1125.78	1.10577	YES	YES
257	a	1153.88	5.97511	YES	YES
258	a	1159.97	13.28936	YES	YES
259	a	1160.50	2.63715	YES	YES
260	a	1166.32	14.47463	YES	YES
261	a	1166.86	1.51657	YES	YES
262	a	1167.48	7.45985	YES	YES
263	a	1168.16	2.26902	YES	YES
264	a	1169.65	6.66549	YES	YES
265	a	1170.02	7.40207	YES	YES
266	a	1173.78	2.98636	YES	YES
267	a	1180.64	15.51001	YES	YES
268	a	1181.34	3.44870	YES	YES
269	a	1182.06	8.43088	YES	YES
270	a	1182.26	1.83560	YES	YES
271	a	1184.59	2.20214	YES	YES
272	a	1188.38	1.14164	YES	YES
273	a	1212.58	0.65242	YES	YES
274	a	1213.04	1.49405	YES	YES
275	a	1239.10	0.36053	YES	YES
276	a	1239.83	0.21509	YES	YES
277	a	1241.90	0.46657	YES	YES
278	a	1242.33	0.72391	YES	YES
279	a	1246.05	0.34143	YES	YES
280	a	1247.03	0.31993	YES	YES
281	a	1248.62	0.94211	YES	YES
282	a	1249.14	0.12800	YES	YES

283	a	1249.93	0.73217	YES	YES
284	a	1251.06	0.55215	YES	YES
285	a	1251.85	2.12769	YES	YES
286	a	1252.45	2.59710	YES	YES
287	a	1254.09	0.13669	YES	YES
288	a	1255.16	1.50496	YES	YES
289	a	1256.51	3.19205	YES	YES
290	a	1256.68	9.80796	YES	YES
291	a	1257.60	2.26777	YES	YES
292	a	1258.01	5.00716	YES	YES
293	a	1259.05	0.44042	YES	YES
294	a	1259.82	0.95898	YES	YES
295	a	1260.72	1.74496	YES	YES
296	a	1261.69	2.11076	YES	YES
297	a	1261.81	3.40430	YES	YES
298	a	1263.70	4.04530	YES	YES
299	a	1264.43	3.70870	YES	YES
300	a	1266.16	1.65483	YES	YES
301	a	1268.15	1.81073	YES	YES
302	a	1268.62	1.09197	YES	YES
303	a	1270.50	1.21676	YES	YES
304	a	1280.20	4.11518	YES	YES
305	a	1281.60	0.12257	YES	YES
306	a	1283.90	1.54715	YES	YES
307	a	1284.50	2.17130	YES	YES
308	a	1286.41	9.29247	YES	YES
309	a	1287.02	5.53122	YES	YES
310	a	1288.88	1.45374	YES	YES
311	a	1289.27	2.46815	YES	YES
312	a	1305.10	0.20816	YES	YES
313	a	1306.29	0.26338	YES	YES
314	a	1310.44	1.29066	YES	YES
315	a	1311.01	0.50243	YES	YES
316	a	1311.28	0.78648	YES	YES
317	a	1312.17	3.76831	YES	YES
318	a	1313.63	0.94604	YES	YES
319	a	1314.75	0.82332	YES	YES
320	a	1318.40	1.54824	YES	YES
321	a	1319.33	2.82743	YES	YES
322	a	1321.23	1.03170	YES	YES
323	a	1321.58	1.87922	YES	YES
324	a	1322.48	1.06732	YES	YES
325	a	1323.28	1.09475	YES	YES
326	a	1324.00	1.30999	YES	YES
327	a	1324.07	1.08329	YES	YES
328	a	1324.22	0.22426	YES	YES
329	a	1324.51	0.48385	YES	YES
330	a	1324.82	0.57439	YES	YES
331	a	1325.16	0.33281	YES	YES
332	a	1326.80	2.75167	YES	YES
333	a	1327.55	9.36543	YES	YES
334	a	1330.23	0.34203	YES	YES
335	a	1330.55	3.19777	YES	YES
336	a	1336.50	0.13928	YES	YES
337	a	1336.73	0.17128	YES	YES
338	a	1336.87	0.81135	YES	YES
339	a	1337.12	0.49281	YES	YES
340	a	1337.77	0.12572	YES	YES

341	a	1337.89	0.10678	YES	YES
342	a	1338.60	0.05921	YES	YES
343	a	1339.10	0.10639	YES	YES
344	a	1343.44	0.06260	YES	YES
345	a	1343.61	0.20033	YES	YES
346	a	1344.53	2.33615	YES	YES
347	a	1344.75	2.34536	YES	YES
348	a	1345.25	0.24757	YES	YES
349	a	1347.11	1.59981	YES	YES
350	a	1347.31	1.68038	YES	YES
351	a	1347.97	0.68896	YES	YES
352	a	1355.06	0.78943	YES	YES
353	a	1369.65	2.92879	YES	YES
354	a	1402.99	0.95090	YES	YES
355	a	1406.84	3.40229	YES	YES
356	a	1407.25	11.59440	YES	YES
357	a	1409.36	8.46103	YES	YES
358	a	1409.64	10.66404	YES	YES
359	a	1415.29	7.77603	YES	YES
360	a	1422.47	0.08692	YES	YES
361	a	1423.76	10.16142	YES	YES
362	a	1425.39	5.51916	YES	YES
363	a	1427.15	2.12668	YES	YES
364	a	1429.83	3.82391	YES	YES
365	a	1431.25	8.17101	YES	YES
366	a	1432.42	0.36359	YES	YES
367	a	1432.66	0.72911	YES	YES
368	a	1433.50	9.33397	YES	YES
369	a	1433.95	3.54271	YES	YES
370	a	1435.33	1.81190	YES	YES
371	a	1435.43	7.85943	YES	YES
372	a	1435.98	4.56263	YES	YES
373	a	1436.44	2.17668	YES	YES
374	a	1437.11	7.07509	YES	YES
375	a	1437.61	7.54787	YES	YES
376	a	1437.95	9.60613	YES	YES
377	a	1438.34	9.20473	YES	YES
378	a	1438.56	3.89930	YES	YES
379	a	1438.89	0.59795	YES	YES
380	a	1439.13	8.69295	YES	YES
381	a	1439.76	4.64563	YES	YES
382	a	1439.89	10.18077	YES	YES
383	a	1440.03	0.45043	YES	YES
384	a	1440.58	6.01985	YES	YES
385	a	1441.63	13.49025	YES	YES
386	a	1442.15	11.84162	YES	YES
387	a	1442.69	3.47492	YES	YES
388	a	1443.16	23.61681	YES	YES
389	a	1443.63	17.38177	YES	YES
390	a	1444.49	17.78732	YES	YES
391	a	1444.96	13.64962	YES	YES
392	a	1445.52	3.12766	YES	YES
393	a	1445.98	12.14269	YES	YES
394	a	1447.26	1.37556	YES	YES
395	a	1451.61	2.21192	YES	YES
396	a	1453.63	2.62071	YES	YES
397	a	1453.98	4.39606	YES	YES
398	a	1454.38	1.52842	YES	YES

399	a	1454.47	0.45163	YES	YES
400	a	1454.86	1.08551	YES	YES
401	a	1456.71	1.11306	YES	YES
402	a	1456.99	3.10226	YES	YES
403	a	1457.67	1.67649	YES	YES
404	a	1621.38	2.39499	YES	YES
405	a	2926.21	0.52903	YES	YES
406	a	2933.09	3.68719	YES	YES
407	a	2938.16	15.77901	YES	YES
408	a	2938.50	18.43248	YES	YES
409	a	2938.87	38.08872	YES	YES
410	a	2939.19	18.05833	YES	YES
411	a	2939.88	6.02832	YES	YES
412	a	2940.62	35.90173	YES	YES
413	a	2942.25	2.27852	YES	YES
414	a	2942.79	8.12098	YES	YES
415	a	2944.16	4.18949	YES	YES
416	a	2944.58	5.83371	YES	YES
417	a	2944.58	4.22436	YES	YES
418	a	2945.50	11.42107	YES	YES
419	a	2946.03	12.52108	YES	YES
420	a	2946.15	11.07793	YES	YES
421	a	2946.78	19.89235	YES	YES
422	a	2948.03	15.41885	YES	YES
423	a	2948.59	14.57699	YES	YES
424	a	2949.14	15.65197	YES	YES
425	a	2949.87	13.16501	YES	YES
426	a	2950.05	11.64002	YES	YES
427	a	2950.09	10.55898	YES	YES
428	a	2950.54	19.83777	YES	YES
429	a	2951.46	8.85069	YES	YES
430	a	2951.59	11.50107	YES	YES
431	a	2952.99	20.33472	YES	YES
432	a	2954.01	12.19250	YES	YES
433	a	2954.92	6.67086	YES	YES
434	a	2955.41	16.13624	YES	YES
435	a	2956.26	6.76586	YES	YES
436	a	2957.08	20.07831	YES	YES
437	a	2957.19	14.05127	YES	YES
438	a	2957.37	12.86969	YES	YES
439	a	2957.53	25.54882	YES	YES
440	a	2957.67	15.56948	YES	YES
441	a	2958.36	30.52073	YES	YES
442	a	2958.64	20.85563	YES	YES
443	a	2960.22	36.82764	YES	YES
444	a	2962.65	28.82036	YES	YES
445	a	2965.08	16.14306	YES	YES
446	a	2965.49	20.92146	YES	YES
447	a	2966.33	19.12933	YES	YES
448	a	2966.78	8.14881	YES	YES
449	a	2967.61	8.22019	YES	YES
450	a	2967.74	11.86231	YES	YES
451	a	2968.75	14.57127	YES	YES
452	a	2973.80	11.85309	YES	YES
453	a	2973.82	7.12100	YES	YES
454	a	2978.50	11.63512	YES	YES
455	a	2979.09	4.31296	YES	YES
456	a	2981.44	9.64805	YES	YES

457	a	2982.18	6.01551	YES	YES
458	a	2986.58	48.05383	YES	YES
459	a	2987.12	5.09901	YES	YES
460	a	2988.00	2.03421	YES	YES
461	a	2989.63	49.49689	YES	YES
462	a	2991.45	10.60437	YES	YES
463	a	2991.51	20.02657	YES	YES
464	a	2991.56	12.90993	YES	YES
465	a	2991.98	7.50388	YES	YES
466	a	2992.08	10.81959	YES	YES
467	a	2993.13	12.54649	YES	YES
468	a	2994.95	39.04498	YES	YES
469	a	2997.00	32.91840	YES	YES
470	a	2998.77	23.08348	YES	YES
471	a	3002.40	30.54927	YES	YES
472	a	3003.54	6.74468	YES	YES
473	a	3004.74	17.55315	YES	YES
474	a	3005.29	38.21074	YES	YES
475	a	3006.11	25.64183	YES	YES
476	a	3010.16	14.46579	YES	YES
477	a	3010.55	9.79084	YES	YES
478	a	3010.65	15.75189	YES	YES
479	a	3011.35	24.10546	YES	YES
480	a	3012.90	13.63644	YES	YES
481	a	3014.03	39.08969	YES	YES
482	a	3014.24	17.94758	YES	YES
483	a	3014.43	23.47118	YES	YES
484	a	3014.85	22.11121	YES	YES
485	a	3014.88	1.90674	YES	YES
486	a	3015.54	16.62094	YES	YES
487	a	3016.02	26.65750	YES	YES
488	a	3016.30	11.40235	YES	YES
489	a	3016.36	26.39929	YES	YES
490	a	3016.46	47.51240	YES	YES
491	a	3016.71	13.19962	YES	YES
492	a	3017.14	21.32211	YES	YES
493	a	3017.66	15.45451	YES	YES
494	a	3017.88	33.43171	YES	YES
495	a	3018.18	28.15309	YES	YES
496	a	3018.25	19.53407	YES	YES
497	a	3019.34	18.47844	YES	YES
498	a	3019.84	34.60449	YES	YES
499	a	3019.95	34.35528	YES	YES
500	a	3021.75	22.17190	YES	YES
501	a	3026.44	4.83403	YES	YES
502	a	3028.13	11.08196	YES	YES
503	a	3029.19	16.38171	YES	YES
504	a	3029.42	25.58440	YES	YES
505	a	3031.60	7.57417	YES	YES
506	a	3039.37	1.78179	YES	YES
507	a	3039.55	1.82791	YES	YES
508	a	3047.85	6.06601	YES	YES
509	a	3053.39	12.51112	YES	YES
510	a	3070.73	20.59903	YES	YES

$end

Double hybrid single point energy = -7483.398135452510 H
COSMO energy + OC correction = -7491.1196653206 (in *o*DFB)

6.2.9 EtOH

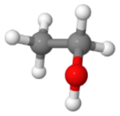

```
Method: (RI-)BP86(D3BJ)/def2-TZVPP
Symmetry: c1

Cartesian coordinates in Ångström:
O    -2.0682522    1.3205384    0.4754789
C    -3.4961656    1.4394881    0.5199099
H    -3.8091119    2.4733390    0.2843073
H    -3.7650506    1.2502773    1.5681229
C    -4.2034914    0.4504537   -0.3980239
H    -3.9510116    0.6394265   -1.4524210
H    -5.2952508    0.5370450   -0.2983930
H    -3.9127273   -0.5800273   -0.1551024
H    -1.7874988    1.4837693   -0.4390388

SCF energy GEOOPT =  -155.1205854950 H
ZPE =   203.6 kJ/mol
FREEH energy =   215.02 kJ/mol
FREEH entropy =   0.27115 kJ/mol/K

$vibrational spectrum
#  mode     symmetry     wave number   IR intensity    selection rules
#                         cm**(-1)        km/mol         IR     RAMAN
     1                      -0.00        0.00000         -        -
     2                       0.00        0.00000         -        -
     3                       0.00        0.00000         -        -
     4                       0.00        0.00000         -        -
     5                       0.00        0.00000         -        -
     6                       0.00        0.00000         -        -
     7         a           256.65        3.13145        YES      YES
     8         a           273.67      103.63565        YES      YES
     9         a           408.33       12.33647        YES      YES
    10         a           781.75        3.21810        YES      YES
    11         a           859.25       11.51120        YES      YES
    12         a          1030.97       73.36766        YES      YES
    13         a          1034.89       47.66579        YES      YES
    14         a          1099.51        3.49889        YES      YES
    15         a          1241.53       10.04921        YES      YES
    16         a          1329.30        1.02629        YES      YES
    17         a          1355.35        3.37722        YES      YES
    18         a          1372.17       41.69523        YES      YES
    19         a          1441.30        7.08622        YES      YES
    20         a          1445.50        2.02265        YES      YES
    21         a          1467.72        1.11946        YES      YES
    22         a          2909.98       67.74161        YES      YES
    23         a          2956.17       17.41557        YES      YES
    24         a          3000.06        7.75822        YES      YES
    25         a          3025.22       48.95406        YES      YES
    26         a          3042.05       30.41449        YES      YES
    27         a          3703.24       13.26086        YES      YES
$end

Double hybrid single point energy = -154.860751543774 H
```

COSMO energy + OC correction = -155.1276140019 H (in *o*DFB)

6.2.10 [H{Ga(dcpe)}$_2$(OEt)]$^{2+}$

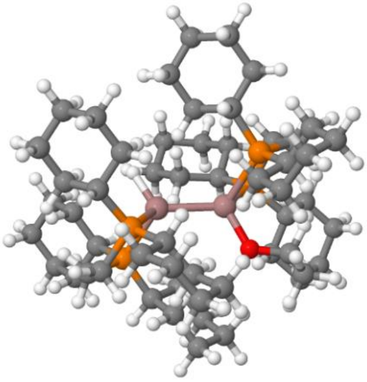

Method: (RI-)BP86(D3BJ)/def2-TZVPP
Symmetry: c1

Cartesian coordinates in Ångström:

Ga	0.0323728	1.3341080	-0.0701687
Ga	-0.1559380	-1.0792664	0.0054747
O	-1.8724623	-1.7197872	0.2040362
C	-2.2175130	-3.0876618	0.3837749
H	-1.3297686	-3.7032286	0.6346682
H	-2.6155682	-3.4949400	-0.5634810
C	-3.2614498	-3.2320431	1.4778943
H	-4.1539462	-2.6439376	1.2293481
H	-2.8738190	-2.8699265	2.4394616
H	-3.5639304	-4.2814022	1.5968842
P	-1.2884495	2.5736011	-1.6331869
P	-1.0654664	2.5667392	1.6762742
C	-2.7125358	1.6480951	-2.3395502
H	-3.2845208	2.3661529	-2.9509961
C	-3.6217002	1.0991734	-1.2283520
H	-3.0298130	0.4477135	-0.5658073
H	-4.0187244	1.9229703	-0.6194247
C	-4.7823240	0.2851909	-1.8111874
H	-5.3939137	-0.1138733	-0.9904078
H	-5.4375958	0.9476906	-2.3995154
C	-4.2660733	-0.8503725	-2.6971290
H	-3.6980083	-1.5553523	-2.0686786
H	-5.1056962	-1.4108793	-3.1293700
C	-3.3622737	-0.3103562	-3.8089048
H	-3.9540456	0.3242758	-4.4879217
H	-2.9657260	-1.1311029	-4.4223919
C	-2.1988719	0.5127912	-3.2423820
H	-1.5857322	0.9138122	-4.0621196
H	-1.5454616	-0.1432538	-2.6408164
C	-0.3567176	3.3277187	-3.0393034
H	0.0015577	2.4598637	-3.6200302
C	-1.2518644	4.1909526	-3.9471377
H	-2.1018769	3.6063069	-4.3253615
H	-1.6712037	5.0239103	-3.3597916
C	-0.4452211	4.7605571	-5.1222529

H	-0.1147916	3.9299868	-5.7677277
H	-1.0971299	5.3948178	-5.7379613
C	0.7747001	5.5476880	-4.6368568
H	0.4382940	6.4356931	-4.0764768
H	1.3510223	5.9212244	-5.4936791
C	1.6631990	4.6843038	-3.7373691
H	2.5171716	5.2644542	-3.3616143
H	2.0828873	3.8551566	-4.3303263
C	0.8778774	4.1075644	-2.5519147
H	1.5179167	3.4583240	-1.9382472
H	0.5575015	4.9399430	-1.9042901
C	-1.9432833	3.9945035	-0.6432179
H	-1.1576254	4.7602772	-0.6904965
H	-2.8251825	4.4104846	-1.1496354
C	-2.2787808	3.6760117	0.8240761
H	-3.2469994	3.1649473	0.8911961
H	-2.3723116	4.6084174	1.3975320
C	-2.0275592	1.6512102	2.9593266
H	-1.2751321	1.0112107	3.4510395
C	-3.0878420	0.7385984	2.3191755
H	-3.7973157	1.3521896	1.7419680
H	-2.6241937	0.0351733	1.6115265
C	-3.8758950	-0.0326904	3.3847018
H	-4.6566763	-0.6293692	2.8939166
H	-3.2048662	-0.7490456	3.8827805
C	-4.4858902	0.9004110	4.4330251
H	-5.2469507	1.5408159	3.9572577
H	-5.0082776	0.3174905	5.2034425
C	-3.4109623	1.7820982	5.0727859
H	-2.6992845	1.1506417	5.6307014
H	-3.8559952	2.4707820	5.8034898
C	-2.6422325	2.5866679	4.0161011
H	-1.8642855	3.1911699	4.5019506
H	-3.3346073	3.2895778	3.5245488
C	0.1627483	3.6223681	2.5544214
H	-0.3913589	4.1833247	3.3251567
C	1.1998470	2.7126278	3.2399724
H	1.6668403	2.0718361	2.4726296
H	0.7062481	2.0444060	3.9610560
C	2.2900741	3.5334343	3.9386704
H	1.8436566	4.0931447	4.7762401
H	3.0340795	2.8548922	4.3796889
C	2.9570983	4.5107386	2.9672589
H	3.4833957	3.9425930	2.1817058
H	3.7188642	5.1047207	3.4893059
C	1.9198258	5.4322642	2.3189775
H	2.4007350	6.1148456	1.6051130
H	1.4562228	6.0643178	3.0934622
C	0.8293328	4.6282234	1.5993269
H	1.2773741	4.0818323	0.7540653
H	0.0749854	5.3122077	1.1834565
P	0.9999592	-2.4398657	-1.6913398
P	1.2366262	-2.3049586	1.6182709
C	-0.0811166	-3.7485714	-2.4095418
H	-0.4368169	-4.3199643	-1.5341717
C	0.6274380	-4.7129874	-3.3743205
H	1.4882898	-5.1970138	-2.8913484
H	1.0167556	-4.1456810	-4.2345831

C	-0.3578341	-5.7778620	-3.8792257
H	0.1561319	-6.4458714	-4.5834031
H	-0.6783054	-6.4034663	-3.0300789
C	-1.5845820	-5.1403830	-4.5392019
H	-2.2888202	-5.9191899	-4.8603610
H	-1.2713644	-4.6074878	-5.4523199
C	-2.2779277	-4.1570119	-3.5916752
H	-2.6965246	-4.7055724	-2.7315559
H	-3.1258725	-3.6682736	-4.0912005
C	-1.3021254	-3.0937354	-3.0758086
H	-0.9624816	-2.4747893	-3.9230430
H	-1.7998981	-2.4190607	-2.3658031
C	1.8122111	-1.5387049	-3.0910497
H	1.0998369	-1.6610489	-3.9255459
C	3.1673862	-2.1304362	-3.5194595
H	3.8802729	-2.0504044	-2.6832855
H	3.0705335	-3.1982029	-3.7559757
C	3.7315471	-1.3653394	-4.7240557
H	3.0653185	-1.5101241	-5.5901605
H	4.7043562	-1.7912891	-5.0041198
C	3.8659678	0.1297111	-4.4215672
H	4.6130407	0.2746328	-3.6235770
H	4.2442256	0.6643996	-5.3029478
C	2.5275212	0.7258937	-3.9752246
H	2.6477645	1.7846775	-3.7066720
H	1.8135579	0.6910529	-4.8154660
C	1.9344335	-0.0362945	-2.7871092
H	0.9447910	0.3714240	-2.5307987
H	2.5686492	0.1108237	-1.8972008
C	2.3078442	-3.3300952	-0.7351056
H	2.6587253	-4.1952240	-1.3129374
H	3.1653999	-2.6523128	-0.6287536
C	1.7944928	-3.7685291	0.6459834
H	2.5734337	-4.3197405	1.1882164
H	0.9289320	-4.4405143	0.5515858
C	0.3226758	-2.9102176	3.0972104
H	-0.4702276	-3.5549207	2.6803590
C	-0.3550803	-1.7133487	3.7906262
H	0.4163775	-0.9966052	4.1203836
H	-0.9981038	-1.1887305	3.0680652
C	-1.1718163	-2.1676773	5.0066631
H	-1.5927356	-1.2890286	5.5157544
H	-2.0283963	-2.7676834	4.6578570
C	-0.3332217	-3.0014037	5.9795917
H	-0.9573047	-3.3453478	6.8149197
H	0.4565184	-2.3694534	6.4188567
C	0.3099583	-4.1958162	5.2697739
H	-0.4752990	-4.8805858	4.9103435
H	0.9323056	-4.7712485	5.9680858
C	1.1660440	-3.7366651	4.0820343
H	1.9936991	-3.1171406	4.4613184
H	1.6157445	-4.6029679	3.5764312
C	2.7640083	-1.4269702	2.2019378
H	2.4501446	-0.9932713	3.1684238
C	3.1579432	-0.2643071	1.2766838
H	2.3008272	0.3980004	1.1020792
H	3.4463742	-0.6603092	0.2877582
C	4.3280937	0.5311864	1.8628576

```
H     4.6041434    1.3427602    1.1751371
H     3.9951141    1.0121600    2.7980040
C     5.5319981   -0.3697281    2.1536383
H     5.9361023   -0.7553726    1.2027845
H     6.3387536    0.2105807    2.6207381
C     5.1384679   -1.5474891    3.0502645
H     4.8458425   -1.1739772    4.0452565
H     5.9958451   -2.2155563    3.2079500
C     3.9721160   -2.3473928    2.4524320
H     3.7074796   -3.1828836    3.1125085
H     4.2971487   -2.7869486    1.4952145
H     1.4544991    2.0030036   -0.1693886

SCF energy GEOOPT =  -7411.352212692 H
ZPE =   3797. kJ/mol
FREEH energy =  3985.92 kJ/mol
FREEH entropy =   1.61294 kJ/mol/K

$vibrational spectrum
#  mode     symmetry     wave number   IR intensity    selection rules
#                         cm**(-1)        km/mol          IR     RAMAN
     1                      -0.00          0.00000         -        -
     2                       0.00          0.00000         -        -
     3                       0.00          0.00000         -        -
     4                       0.00          0.00000         -        -
     5                       0.00          0.00000         -        -
     6                       0.00          0.00000         -        -
     7         a             9.83          0.03113        YES      YES
     8         a            13.87          0.13365        YES      YES
     9         a            23.41          0.01402        YES      YES
    10         a            24.96          0.01587        YES      YES
    11         a            31.05          0.07830        YES      YES
    12         a            33.76          0.03696        YES      YES
    13         a            34.78          0.00637        YES      YES
    14         a            38.25          0.03727        YES      YES
    15         a            40.59          0.08942        YES      YES
    16         a            41.05          0.09038        YES      YES
    17         a            44.26          0.03041        YES      YES
    18         a            49.01          0.31025        YES      YES
    19         a            50.45          0.25772        YES      YES
    20         a            51.90          0.06587        YES      YES
    21         a            53.59          0.06690        YES      YES
    22         a            56.72          0.16422        YES      YES
    23         a            58.62          0.10483        YES      YES
    24         a            61.53          0.10374        YES      YES
    25         a            63.05          0.41311        YES      YES
    26         a            65.20          0.34347        YES      YES
    27         a            66.56          0.73003        YES      YES
    28         a            68.93          0.35784        YES      YES
    29         a            70.40          0.70672        YES      YES
    30         a            74.99          0.92608        YES      YES
    31         a            76.71          0.02888        YES      YES
    32         a            78.53          0.67139        YES      YES
    33         a            84.40          0.16392        YES      YES
    34         a            88.24          0.05423        YES      YES
    35         a            89.62          0.25283        YES      YES
    36         a            97.84          0.31199        YES      YES
    37         a            99.90          2.23602        YES      YES
```

38	a	116.90	1.94676	YES	YES
39	a	120.00	1.76699	YES	YES
40	a	122.40	1.28925	YES	YES
41	a	123.81	6.49059	YES	YES
42	a	129.59	0.61747	YES	YES
43	a	132.83	0.21378	YES	YES
44	a	143.24	1.81364	YES	YES
45	a	143.70	0.40296	YES	YES
46	a	146.27	0.35769	YES	YES
47	a	154.04	1.50628	YES	YES
48	a	161.03	2.89493	YES	YES
49	a	169.91	13.28954	YES	YES
50	a	171.97	1.78199	YES	YES
51	a	174.80	0.20188	YES	YES
52	a	179.57	0.23622	YES	YES
53	a	184.64	2.64478	YES	YES
54	a	188.48	1.88397	YES	YES
55	a	193.13	1.20642	YES	YES
56	a	204.26	3.59864	YES	YES
57	a	206.51	9.10843	YES	YES
58	a	212.42	0.57451	YES	YES
59	a	217.19	0.68591	YES	YES
60	a	220.28	0.04639	YES	YES
61	a	223.19	2.73981	YES	YES
62	a	227.78	2.15820	YES	YES
63	a	231.39	0.10120	YES	YES
64	a	232.86	0.60050	YES	YES
65	a	234.90	0.25510	YES	YES
66	a	237.06	0.24909	YES	YES
67	a	237.72	0.20429	YES	YES
68	a	240.08	0.32756	YES	YES
69	a	241.27	0.71827	YES	YES
70	a	253.14	0.12241	YES	YES
71	a	256.37	0.25645	YES	YES
72	a	272.08	0.72963	YES	YES
73	a	275.34	3.06281	YES	YES
74	a	282.33	6.70057	YES	YES
75	a	291.93	0.07468	YES	YES
76	a	298.97	4.57063	YES	YES
77	a	306.18	0.15647	YES	YES
78	a	308.73	0.15202	YES	YES
79	a	309.82	0.04215	YES	YES
80	a	312.59	0.06036	YES	YES
81	a	330.45	0.44899	YES	YES
82	a	330.94	0.70168	YES	YES
83	a	333.12	0.16757	YES	YES
84	a	335.06	2.17408	YES	YES
85	a	337.26	2.69220	YES	YES
86	a	346.21	3.90936	YES	YES
87	a	349.27	1.30482	YES	YES
88	a	370.03	3.76274	YES	YES
89	a	373.29	0.51788	YES	YES
90	a	374.12	2.01670	YES	YES
91	a	382.97	1.29714	YES	YES
92	a	393.74	0.19970	YES	YES
93	a	402.96	0.04731	YES	YES
94	a	411.59	0.35701	YES	YES
95	a	412.41	1.05553	YES	YES

96	a	421.00	2.77876	YES	YES
97	a	422.88	0.19435	YES	YES
98	a	427.87	0.61447	YES	YES
99	a	428.73	0.12095	YES	YES
100	a	429.89	0.05366	YES	YES
101	a	430.28	0.17555	YES	YES
102	a	430.93	0.33954	YES	YES
103	a	431.22	0.27281	YES	YES
104	a	434.23	0.67609	YES	YES
105	a	434.76	0.81655	YES	YES
106	a	435.68	0.00451	YES	YES
107	a	440.51	3.20291	YES	YES
108	a	446.85	4.11163	YES	YES
109	a	457.34	1.59580	YES	YES
110	a	463.90	3.58077	YES	YES
111	a	466.05	4.52987	YES	YES
112	a	489.60	4.73988	YES	YES
113	a	493.38	2.98461	YES	YES
114	a	493.56	1.34699	YES	YES
115	a	497.98	0.05856	YES	YES
116	a	501.70	0.58460	YES	YES
117	a	503.16	3.16800	YES	YES
118	a	514.91	3.40142	YES	YES
119	a	516.76	18.52331	YES	YES
120	a	562.05	81.91880	YES	YES
121	a	577.87	34.84836	YES	YES
122	a	608.61	80.51144	YES	YES
123	a	631.49	47.66552	YES	YES
124	a	635.84	16.96188	YES	YES
125	a	641.90	5.70406	YES	YES
126	a	655.04	7.48193	YES	YES
127	a	705.80	2.80675	YES	YES
128	a	715.69	6.21369	YES	YES
129	a	716.62	1.67335	YES	YES
130	a	726.90	1.17001	YES	YES
131	a	734.16	8.40476	YES	YES
132	a	736.31	0.73463	YES	YES
133	a	739.06	9.68957	YES	YES
134	a	741.22	0.70151	YES	YES
135	a	770.25	0.52730	YES	YES
136	a	771.28	0.52931	YES	YES
137	a	773.29	0.26489	YES	YES
138	a	773.46	0.65267	YES	YES
139	a	773.84	0.31724	YES	YES
140	a	776.51	0.06923	YES	YES
141	a	777.17	0.20655	YES	YES
142	a	777.38	12.09491	YES	YES
143	a	778.32	0.06245	YES	YES
144	a	785.13	0.57913	YES	YES
145	a	794.83	20.58850	YES	YES
146	a	807.78	1.13301	YES	YES
147	a	808.29	3.39034	YES	YES
148	a	809.01	0.27726	YES	YES
149	a	809.71	3.08494	YES	YES
150	a	812.76	1.25149	YES	YES
151	a	813.25	0.77268	YES	YES
152	a	814.66	2.51363	YES	YES
153	a	814.83	6.87381	YES	YES

154	a	832.93	3.55528	YES	YES
155	a	833.63	0.29133	YES	YES
156	a	837.02	3.35219	YES	YES
157	a	837.41	1.39193	YES	YES
158	a	838.47	5.67875	YES	YES
159	a	839.99	4.59503	YES	YES
160	a	840.28	5.11410	YES	YES
161	a	841.60	6.87229	YES	YES
162	a	850.48	18.57119	YES	YES
163	a	852.07	1.83245	YES	YES
164	a	872.00	1.82885	YES	YES
165	a	873.70	6.22828	YES	YES
166	a	875.18	2.17166	YES	YES
167	a	875.78	0.76768	YES	YES
168	a	876.03	1.74289	YES	YES
169	a	877.22	4.84461	YES	YES
170	a	877.95	0.98370	YES	YES
171	a	878.96	1.11840	YES	YES
172	a	879.46	2.92183	YES	YES
173	a	879.67	5.35609	YES	YES
174	a	880.30	2.65765	YES	YES
175	a	880.81	2.94516	YES	YES
176	a	882.14	3.73990	YES	YES
177	a	882.64	1.72832	YES	YES
178	a	884.66	3.27697	YES	YES
179	a	885.92	4.59479	YES	YES
180	a	896.04	29.46404	YES	YES
181	a	903.00	0.88299	YES	YES
182	a	903.50	1.07668	YES	YES
183	a	903.87	2.14733	YES	YES
184	a	905.43	2.28385	YES	YES
185	a	908.27	4.38560	YES	YES
186	a	908.40	2.39339	YES	YES
187	a	909.84	1.67933	YES	YES
188	a	911.57	2.09098	YES	YES
189	a	978.54	2.04632	YES	YES
190	a	986.14	7.64088	YES	YES
191	a	986.69	3.25243	YES	YES
192	a	987.11	8.16808	YES	YES
193	a	987.51	8.60984	YES	YES
194	a	988.40	16.50649	YES	YES
195	a	991.24	18.91670	YES	YES
196	a	991.28	7.56347	YES	YES
197	a	991.84	15.83146	YES	YES
198	a	993.20	0.98754	YES	YES
199	a	1014.63	1.44909	YES	YES
200	a	1015.22	0.51167	YES	YES
201	a	1016.84	0.75272	YES	YES
202	a	1017.14	0.79972	YES	YES
203	a	1017.43	0.18114	YES	YES
204	a	1018.82	0.42363	YES	YES
205	a	1020.30	0.78255	YES	YES
206	a	1020.99	0.55171	YES	YES
207	a	1030.32	1.15668	YES	YES
208	a	1031.17	2.27162	YES	YES
209	a	1031.80	1.50661	YES	YES
210	a	1033.39	1.50920	YES	YES
211	a	1034.61	3.34470	YES	YES

212	a	1036.58	1.02579	YES	YES
213	a	1037.81	3.28785	YES	YES
214	a	1038.64	1.15443	YES	YES
215	a	1047.95	95.31731	YES	YES
216	a	1053.42	15.05579	YES	YES
217	a	1057.72	2.26636	YES	YES
218	a	1059.92	2.98580	YES	YES
219	a	1060.87	0.94822	YES	YES
220	a	1061.91	1.33074	YES	YES
221	a	1063.35	2.11010	YES	YES
222	a	1065.93	2.22420	YES	YES
223	a	1067.04	0.89053	YES	YES
224	a	1068.62	0.33987	YES	YES
225	a	1071.24	0.12596	YES	YES
226	a	1071.96	0.39626	YES	YES
227	a	1072.35	0.16698	YES	YES
228	a	1072.38	0.07230	YES	YES
229	a	1072.79	0.60819	YES	YES
230	a	1072.84	0.18611	YES	YES
231	a	1074.13	0.06919	YES	YES
232	a	1074.67	0.06605	YES	YES
233	a	1081.84	32.22897	YES	YES
234	a	1087.56	1.25522	YES	YES
235	a	1092.71	8.82700	YES	YES
236	a	1094.80	2.16162	YES	YES
237	a	1096.65	7.87853	YES	YES
238	a	1100.02	1.90960	YES	YES
239	a	1100.39	4.42061	YES	YES
240	a	1106.74	6.13195	YES	YES
241	a	1107.37	5.10904	YES	YES
242	a	1108.09	7.68433	YES	YES
243	a	1116.84	1.10848	YES	YES
244	a	1132.20	6.21579	YES	YES
245	a	1135.40	1.51449	YES	YES
246	a	1159.62	2.38291	YES	YES
247	a	1161.06	4.14697	YES	YES
248	a	1163.49	3.80773	YES	YES
249	a	1164.86	3.80838	YES	YES
250	a	1165.87	3.57725	YES	YES
251	a	1167.36	20.00488	YES	YES
252	a	1169.62	18.83925	YES	YES
253	a	1170.79	3.30170	YES	YES
254	a	1176.93	5.63608	YES	YES
255	a	1181.30	5.28150	YES	YES
256	a	1186.99	6.63437	YES	YES
257	a	1187.39	3.12809	YES	YES
258	a	1189.33	1.22879	YES	YES
259	a	1189.69	2.28087	YES	YES
260	a	1190.53	0.48960	YES	YES
261	a	1191.34	4.97111	YES	YES
262	a	1235.94	0.44199	YES	YES
263	a	1241.15	0.12873	YES	YES
264	a	1242.81	0.17141	YES	YES
265	a	1244.42	0.41637	YES	YES
266	a	1245.52	0.71750	YES	YES
267	a	1246.24	0.68430	YES	YES
268	a	1247.80	0.49561	YES	YES
269	a	1247.89	0.56541	YES	YES

270	a	1248.95	0.93833	YES	YES
271	a	1250.12	0.44709	YES	YES
272	a	1251.63	0.06995	YES	YES
273	a	1253.06	0.54106	YES	YES
274	a	1254.55	0.38407	YES	YES
275	a	1255.92	8.54554	YES	YES
276	a	1256.13	0.32841	YES	YES
277	a	1257.19	3.35991	YES	YES
278	a	1257.52	0.72275	YES	YES
279	a	1257.90	3.52165	YES	YES
280	a	1258.37	2.76273	YES	YES
281	a	1258.65	3.00457	YES	YES
282	a	1259.28	3.56478	YES	YES
283	a	1261.29	5.43867	YES	YES
284	a	1261.57	1.28848	YES	YES
285	a	1262.81	1.34891	YES	YES
286	a	1263.84	1.33536	YES	YES
287	a	1265.18	1.11141	YES	YES
288	a	1266.08	1.63115	YES	YES
289	a	1267.38	6.19833	YES	YES
290	a	1272.31	0.01449	YES	YES
291	a	1280.58	2.17621	YES	YES
292	a	1281.62	2.74599	YES	YES
293	a	1283.04	3.31872	YES	YES
294	a	1284.86	6.79348	YES	YES
295	a	1285.19	1.80745	YES	YES
296	a	1286.17	6.88592	YES	YES
297	a	1290.50	4.77003	YES	YES
298	a	1293.37	4.27057	YES	YES
299	a	1305.71	1.47721	YES	YES
300	a	1307.61	0.70098	YES	YES
301	a	1311.19	2.04087	YES	YES
302	a	1311.51	1.38059	YES	YES
303	a	1312.32	1.21518	YES	YES
304	a	1313.82	1.46353	YES	YES
305	a	1315.52	0.77223	YES	YES
306	a	1315.73	1.07605	YES	YES
307	a	1320.80	1.33202	YES	YES
308	a	1322.88	0.83389	YES	YES
309	a	1323.05	1.13128	YES	YES
310	a	1323.26	0.93384	YES	YES
311	a	1323.68	2.47407	YES	YES
312	a	1323.77	0.41317	YES	YES
313	a	1324.13	2.05302	YES	YES
314	a	1324.16	0.63681	YES	YES
315	a	1324.21	1.21072	YES	YES
316	a	1324.43	0.38340	YES	YES
317	a	1326.35	4.44736	YES	YES
318	a	1327.54	1.23372	YES	YES
319	a	1328.26	1.42056	YES	YES
320	a	1328.66	3.96205	YES	YES
321	a	1329.52	6.45570	YES	YES
322	a	1332.06	2.61448	YES	YES
323	a	1336.43	0.34611	YES	YES
324	a	1336.54	0.25162	YES	YES
325	a	1337.16	0.87047	YES	YES
326	a	1337.51	0.34351	YES	YES
327	a	1338.09	0.51737	YES	YES

328	a	1338.48	0.55926	YES	YES
329	a	1339.92	0.55800	YES	YES
330	a	1340.41	1.59692	YES	YES
331	a	1342.41	8.11193	YES	YES
332	a	1344.43	0.41088	YES	YES
333	a	1344.61	2.16798	YES	YES
334	a	1346.16	1.27972	YES	YES
335	a	1346.45	0.68244	YES	YES
336	a	1346.87	0.91503	YES	YES
337	a	1346.97	1.30371	YES	YES
338	a	1349.26	0.73924	YES	YES
339	a	1350.21	2.04753	YES	YES
340	a	1359.79	15.93628	YES	YES
341	a	1399.94	6.89819	YES	YES
342	a	1406.48	10.00549	YES	YES
343	a	1406.69	6.30828	YES	YES
344	a	1408.80	9.56179	YES	YES
345	a	1423.58	1.69076	YES	YES
346	a	1427.37	7.57959	YES	YES
347	a	1428.28	4.99521	YES	YES
348	a	1429.27	5.33592	YES	YES
349	a	1431.06	2.40379	YES	YES
350	a	1431.18	7.09911	YES	YES
351	a	1432.57	0.85323	YES	YES
352	a	1433.21	2.91213	YES	YES
353	a	1434.44	1.18844	YES	YES
354	a	1435.36	9.70092	YES	YES
355	a	1435.62	5.10993	YES	YES
356	a	1436.35	0.60983	YES	YES
357	a	1436.84	1.83664	YES	YES
358	a	1437.14	1.55001	YES	YES
359	a	1437.33	4.28065	YES	YES
360	a	1437.61	2.17804	YES	YES
361	a	1437.76	5.62090	YES	YES
362	a	1438.24	10.28535	YES	YES
363	a	1438.56	3.46726	YES	YES
364	a	1438.86	11.30964	YES	YES
365	a	1439.41	5.02899	YES	YES
366	a	1439.76	5.24448	YES	YES
367	a	1440.37	0.53216	YES	YES
368	a	1440.68	5.18798	YES	YES
369	a	1441.15	8.22643	YES	YES
370	a	1441.48	15.71927	YES	YES
371	a	1442.91	16.17863	YES	YES
372	a	1443.47	15.31451	YES	YES
373	a	1443.62	15.57944	YES	YES
374	a	1444.50	14.35823	YES	YES
375	a	1445.27	27.71620	YES	YES
376	a	1445.75	4.52912	YES	YES
377	a	1446.89	4.95459	YES	YES
378	a	1447.73	3.62458	YES	YES
379	a	1452.76	1.08680	YES	YES
380	a	1453.79	1.49932	YES	YES
381	a	1454.69	1.97690	YES	YES
382	a	1454.99	4.71093	YES	YES
383	a	1455.60	2.30708	YES	YES
384	a	1455.86	0.50451	YES	YES
385	a	1456.44	0.28255	YES	YES

386	a	1458.49	3.92971	YES	YES
387	a	1464.68	2.16456	YES	YES
388	a	1873.53	116.02075	YES	YES
389	a	2859.58	45.85833	YES	YES
390	a	2917.06	42.76606	YES	YES
391	a	2927.25	3.94727	YES	YES
392	a	2927.82	17.72685	YES	YES
393	a	2933.48	15.59732	YES	YES
394	a	2935.55	3.43637	YES	YES
395	a	2936.40	3.97593	YES	YES
396	a	2937.98	8.02690	YES	YES
397	a	2938.14	5.67695	YES	YES
398	a	2938.23	6.41780	YES	YES
399	a	2940.18	10.15621	YES	YES
400	a	2941.45	6.36579	YES	YES
401	a	2942.59	8.98376	YES	YES
402	a	2942.77	6.75261	YES	YES
403	a	2943.57	9.83091	YES	YES
404	a	2943.69	9.23029	YES	YES
405	a	2944.87	23.67822	YES	YES
406	a	2944.91	6.84541	YES	YES
407	a	2945.14	19.14164	YES	YES
408	a	2946.34	12.46320	YES	YES
409	a	2946.60	14.93082	YES	YES
410	a	2947.62	1.84909	YES	YES
411	a	2947.72	12.67336	YES	YES
412	a	2949.06	7.28434	YES	YES
413	a	2949.55	8.30536	YES	YES
414	a	2950.45	17.61445	YES	YES
415	a	2950.68	3.55373	YES	YES
416	a	2950.79	22.84283	YES	YES
417	a	2952.75	9.28789	YES	YES
418	a	2953.39	9.14339	YES	YES
419	a	2953.90	6.03801	YES	YES
420	a	2954.67	5.13798	YES	YES
421	a	2955.19	4.06635	YES	YES
422	a	2956.40	6.73530	YES	YES
423	a	2956.54	7.62067	YES	YES
424	a	2956.85	4.24835	YES	YES
425	a	2956.89	16.16846	YES	YES
426	a	2957.60	6.59099	YES	YES
427	a	2957.98	6.39375	YES	YES
428	a	2958.73	3.16965	YES	YES
429	a	2959.89	8.76335	YES	YES
430	a	2960.58	31.99795	YES	YES
431	a	2961.11	31.02000	YES	YES
432	a	2961.47	50.02324	YES	YES
433	a	2961.96	10.69781	YES	YES
434	a	2962.11	82.21763	YES	YES
435	a	2963.23	29.91025	YES	YES
436	a	2963.41	34.66156	YES	YES
437	a	2963.91	41.99386	YES	YES
438	a	2970.80	11.49466	YES	YES
439	a	2973.10	20.95409	YES	YES
440	a	2980.89	2.85658	YES	YES
441	a	2985.36	4.36743	YES	YES
442	a	2985.54	32.01239	YES	YES
443	a	2990.19	6.05714	YES	YES

444	a	2991.51	26.83125	YES	YES
445	a	2991.96	7.29953	YES	YES
446	a	2992.37	18.21689	YES	YES
447	a	2997.52	33.65051	YES	YES
448	a	2997.83	1.77262	YES	YES
449	a	2999.28	18.59780	YES	YES
450	a	3003.40	17.32267	YES	YES
451	a	3003.44	24.44318	YES	YES
452	a	3004.42	30.71837	YES	YES
453	a	3005.03	18.79301	YES	YES
454	a	3005.62	17.15359	YES	YES
455	a	3007.12	16.62391	YES	YES
456	a	3007.33	3.10658	YES	YES
457	a	3007.59	15.50157	YES	YES
458	a	3007.69	42.44592	YES	YES
459	a	3008.12	44.36907	YES	YES
460	a	3009.45	15.73188	YES	YES
461	a	3009.70	14.45610	YES	YES
462	a	3010.42	16.53969	YES	YES
463	a	3011.46	22.91429	YES	YES
464	a	3013.00	16.59921	YES	YES
465	a	3013.69	21.48135	YES	YES
466	a	3014.16	19.21156	YES	YES
467	a	3014.62	26.76086	YES	YES
468	a	3015.40	37.93841	YES	YES
469	a	3015.44	12.47552	YES	YES
470	a	3015.94	17.81329	YES	YES
471	a	3016.24	7.77740	YES	YES
472	a	3016.42	38.74599	YES	YES
473	a	3016.93	19.46057	YES	YES
474	a	3017.69	34.41203	YES	YES
475	a	3017.82	23.26810	YES	YES
476	a	3017.92	29.54159	YES	YES
477	a	3018.09	34.14230	YES	YES
478	a	3018.29	20.05418	YES	YES
479	a	3018.79	24.49297	YES	YES
480	a	3019.30	15.95602	YES	YES
481	a	3019.53	19.21951	YES	YES
482	a	3019.80	33.62877	YES	YES
483	a	3024.84	14.96994	YES	YES
484	a	3030.42	0.08546	YES	YES
485	a	3032.93	1.34937	YES	YES
486	a	3038.50	0.67206	YES	YES
487	a	3043.69	22.06002	YES	YES
488	a	3046.48	0.54871	YES	YES
489	a	3048.83	14.11245	YES	YES

$end

Double hybrid single point energy = -7403.975779491784 H
COSMO energy + OC correction = -7411.4994578365 H (in *o*DFB)

6.2.11 PhOH

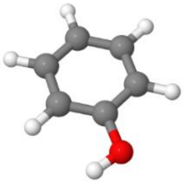

Method: (RI-)BP86(D3BJ)/def2-TZVPP
Symmetry: cs

Cartesian coordinates in Ångström:

O	1.3319757	-2.1255567	0.0000000
C	0.6090030	-0.9583424	0.0000000
C	-0.7912461	-0.9410843	0.0000000
H	-1.3473176	-1.8813969	0.0000000
C	-1.4700835	0.2791366	0.0000000
H	-2.5601197	0.2838856	0.0000000
C	-0.7629095	1.4831124	0.0000000
H	-1.2950572	2.4332757	0.0000000
C	0.6353513	1.4556534	0.0000000
H	1.1985243	2.3890018	0.0000000
C	1.3254354	0.2443727	0.0000000
H	2.4141062	0.2112185	0.0000000
H	0.7123377	-2.8732763	0.0000000

SCF energy GEOOPT = -307.6259677609 H
ZPE = 267.0 kJ/mol
FREEH energy = 281.85 kJ/mol
FREEH entropy = 0.31440 kJ/mol/K

$vibrational spectrum

# mode	symmetry	wave number	IR intensity	selection rules	
#		cm**(-1)	km/mol	IR	RAMAN
1		-0.00	0.00000	-	-
2		-0.00	0.00000	-	-
3		-0.00	0.00000	-	-
4		-0.00	0.00000	-	-
5		0.00	0.00000	-	-
6		0.00	0.00000	-	-
7	a"	219.63	0.80421	YES	YES
8	a"	367.74	91.63266	YES	YES
9	a'	392.77	9.53439	YES	YES
10	a"	405.93	0.30821	YES	YES
11	a"	497.25	14.04645	YES	YES
12	a'	520.76	1.52776	YES	YES
13	a'	614.15	0.39648	YES	YES
14	a"	677.78	25.09697	YES	YES
15	a"	736.51	55.28477	YES	YES
16	a"	792.83	0.00047	YES	YES
17	a'	810.69	19.47492	YES	YES
18	a"	857.22	5.53024	YES	YES
19	a"	927.24	0.09408	YES	YES
20	a"	946.05	0.04918	YES	YES
21	a'	990.81	1.92608	YES	YES
22	a'	1020.19	4.54383	YES	YES
23	a'	1066.46	13.96494	YES	YES
24	a'	1146.20	7.56746	YES	YES

25	a'	1157.10	23.85152	YES	YES
26	a'	1162.36	126.32063	YES	YES
27	a'	1252.23	69.88939	YES	YES
28	a'	1324.93	12.75173	YES	YES
29	a'	1355.44	14.73199	YES	YES
30	a'	1461.74	26.28463	YES	YES
31	a'	1487.50	47.98834	YES	YES
32	a'	1592.84	46.65641	YES	YES
33	a'	1603.87	30.93651	YES	YES
34	a'	3080.24	14.87537	YES	YES
35	a'	3101.93	0.28134	YES	YES
36	a'	3110.00	16.05929	YES	YES
37	a'	3123.93	16.04588	YES	YES
38	a'	3130.49	5.92187	YES	YES
39	a'	3703.45	44.54951	YES	YES

$end

Double hybrid single point energy = -307.083414303859H
COSMO enery + OC correction = -307.6350898506 H (in *o*DFB)

6.2.12 [H{Ga(dcpe)}$_2$(OPh)]$^{2+}$

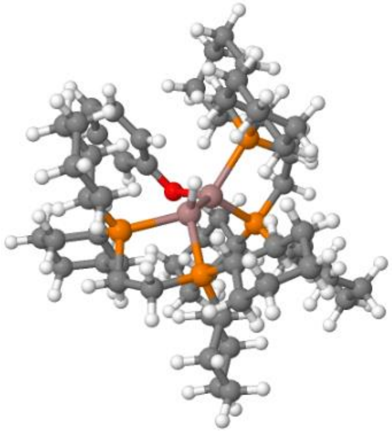

Method: (RI-)BP86(D3BJ)/def2-TZVPP
Symmetry: c1

Cartesian coordinates in Ångström:

Ga	15.2549201	12.9883647	16.3768647
Ga	16.4757343	13.9973158	18.2399182
P	15.1147957	15.1942440	19.8698037
P	17.6601611	13.0440696	20.1162747
P	15.2102155	10.6831385	15.7134324
P	16.1978642	13.4788979	14.2244877
O	17.8443833	15.2119224	17.7839774
C	15.5590980	14.4075585	21.4827759
H	15.3121650	15.1020596	22.2980233
H	14.9074654	13.5329203	21.6025202
C	17.0388906	14.0006610	21.5691934
H	17.2294233	13.4303983	22.4882873
H	17.6687822	14.8967811	21.6150068
C	15.3991064	10.7945304	13.8803365
H	15.6296042	9.8040762	13.4677828
H	14.4054980	11.0740167	13.5054522

C	16.4528269	11.8225998	13.4379225
H	16.4437189	11.9333865	12.3446036
H	17.4594463	11.4836812	13.7164240
C	17.6075596	16.3999801	17.1842860
C	18.6805902	17.2990257	17.0362181
H	19.6622406	17.0085211	17.4094680
C	18.4815339	18.5410328	16.4371416
H	19.3231741	19.2257858	16.3355071
C	17.2148457	18.9198338	15.9763227
H	17.0647581	19.8946105	15.5159078
C	16.1474193	18.0328320	16.1180052
H	15.1543694	18.3116398	15.7651827
C	16.3402410	16.7826240	16.7115268
H	15.4990362	16.0922053	16.8087287
C	13.3040256	14.9578910	19.6402967
H	12.8034123	15.4164823	20.5096945
C	12.9444991	13.4629626	19.5877515
H	13.2136935	12.9627735	20.5284879
H	13.5368931	12.9848515	18.7897428
C	11.4527158	13.2572372	19.3010199
H	11.2385610	12.1814466	19.2369868
H	10.8652016	13.6422871	20.1499549
C	11.0259593	13.9719447	18.0163850
H	9.9501439	13.8335449	17.8446498
H	11.5487715	13.5186024	17.1573698
C	11.3652429	15.4639573	18.0821215
H	11.0894755	15.9660170	17.1446238
H	10.7726941	15.9418784	18.8788426
C	12.8571610	15.6863032	18.3575462
H	13.0754585	16.7606731	18.4295086
H	13.4390014	15.2940203	17.5072034
C	15.4564987	16.9986027	20.0032485
H	15.3206689	17.3608591	18.9707266
C	16.9197507	17.2727791	20.3942905
H	17.0823509	16.9479644	21.4356582
H	17.6077111	16.7033307	19.7565888
C	17.2096345	18.7744438	20.2923827
H	17.1058707	19.0815880	19.2391337
H	18.2532998	18.9687907	20.5751413
C	16.2525117	19.5802648	21.1764322
H	16.4503973	19.3438756	22.2351875
H	16.4400959	20.6559509	21.0592673
C	14.7879607	19.2662764	20.8531006
H	14.5522669	19.6224138	19.8369055
H	14.1177361	19.8038815	21.5374333
C	14.4909463	17.7595752	20.9256571
H	13.4449413	17.5749484	20.6448973
H	14.6127980	17.4077190	21.9634230
C	19.4952630	13.2372517	20.0605677
H	19.8914220	12.6071027	20.8757638
C	19.9632506	14.6869405	20.2795411
H	19.6575215	15.0493257	21.2703865
H	19.4837053	15.3286572	19.5258919
C	21.4899326	14.7867731	20.1559581
H	21.7937628	15.8345812	20.2849353
H	21.9596608	14.2223742	20.9780078
C	21.9891457	14.2417465	18.8152212
H	23.0844088	14.3019680	18.7648914

H	21.5989200	14.8688601	17.9967532
C	21.5256057	12.7979922	18.6069389
H	21.8514665	12.4216502	17.6266941
H	21.9930423	12.1460727	19.3627545
C	20.0005954	12.6916621	18.7118821
H	19.6842359	11.6483418	18.5760754
H	19.5396142	13.2849883	17.9073701
C	17.3134553	11.2575352	20.4082263
H	17.9026050	10.7530677	19.6235080
C	15.8307559	10.9222764	20.1739268
H	15.5140405	11.2729042	19.1784919
H	15.2110531	11.4606640	20.9087741
C	15.5798341	9.4170211	20.3211515
H	14.5085772	9.2062976	20.1981536
H	16.1039023	8.8862146	19.5080316
C	16.0775356	8.8875615	21.6697270
H	15.4735818	9.3301159	22.4788802
H	15.9258110	7.8015484	21.7281211
C	17.5528162	9.2313449	21.8923566
H	18.1732679	8.7049844	21.1484692
H	17.8849614	8.8820866	22.8789801
C	17.7974126	10.7417336	21.7731579
H	17.2467873	11.2557205	22.5772638
H	18.8629735	10.9657865	21.9202706
C	16.4698942	9.5003704	16.3673411
H	15.9976228	9.1018509	17.2829975
C	17.7662278	10.2212756	16.7747131
H	18.2349308	10.6653298	15.8817896
H	17.5366666	11.0614109	17.4478642
C	18.7479170	9.2417630	17.4280595
H	19.6818465	9.7615050	17.6842184
H	18.3158426	8.8806424	18.3770176
C	19.0426501	8.0423131	16.5218286
H	19.7090172	7.3361429	17.0345989
H	19.5843200	8.3887325	15.6263327
C	17.7512416	7.3417360	16.0929619
H	17.2620797	6.8915386	16.9723090
H	17.9716800	6.5170502	15.4021084
C	16.7813480	8.3242622	15.4241985
H	15.8633453	7.8070890	15.1189226
H	17.2498212	8.7146670	14.5061260
C	15.0687885	14.4341455	13.1249663
H	15.6197934	14.5739409	12.1790416
C	14.7911407	15.8089538	13.7542686
H	15.7291048	16.3513849	13.9311573
H	14.3257604	15.6527345	14.7426453
C	13.8478780	16.6421735	12.8804512
H	13.6481242	17.6042206	13.3725930
H	14.3486915	16.8776254	11.9275146
C	12.5422936	15.8930108	12.6028940
H	11.9960178	15.7469784	13.5501343
H	11.8889058	16.4904090	11.9533858
C	12.8207820	14.5310157	11.9615225
H	13.2772039	14.6803967	10.9696689
H	11.8843318	13.9814093	11.7943211
C	13.7625047	13.6788129	12.8238169
H	13.9772528	12.7355335	12.3018593
H	13.2638853	13.4263915	13.7747421

```
C    17.8170476   14.3455977   14.2942927
H    17.5994936   15.2328752   14.9136417
C    18.3585462   14.8230671   12.9386085
H    18.5573732   13.9546449   12.2888533
H    17.6191221   15.4500812   12.4222934
C    19.6539828   15.6196576   13.1523049
H    19.4160432   16.5314296   13.7243168
H    20.0487593   15.9479088   12.1814771
C    20.7032519   14.8001302   13.9098066
H    21.6035768   15.4053259   14.0818550
H    21.0171166   13.9449416   13.2884141
C    20.1531663   14.2848830   15.2434071
H    20.8964137   13.6500263   15.7457530
H    19.9474434   15.1314939   15.9152453
C    18.8553532   13.4956200   15.0448062
H    18.4508294   13.1735381   16.0157582
H    19.0780491   12.5849691   14.4642513
C    13.5158742   10.0215003   15.9980235
H    12.8825458   10.7702383   15.4874226
C    13.1720084   10.0574344   17.4954384
H    13.3636989   11.0611476   17.9040245
H    13.8304192    9.3583065   18.0351778
C    11.7099713    9.6576158   17.7280170
H    11.4958316    9.6506217   18.8061824
H    11.0558553   10.4239051   17.2807011
C    11.3963033    8.2910756   17.1101338
H    11.9661604    7.5111024   17.6418684
H    10.3343610    8.0485552   17.2484181
C    11.7581795    8.2557541   15.6221028
H    11.1130738    8.9562527   15.0670070
H    11.5697589    7.2580819   15.2034581
C    13.2279318    8.6371871   15.3951014
H    13.4662740    8.6177455   14.3222544
H    13.8706822    7.8882548   15.8836904
H    13.7166213   13.3137332   16.2170787

SCF energy GEOOPT =  -7563.875389214 H
ZPE =    3862. kJ/mol
FREEH energy =  4054.64 kJ/mol
FREEH entropy =   1.63198 kJ/mol/K

$vibrational spectrum
#  mode     symmetry     wave number   IR intensity    selection rules
#                         cm**(-1)        km/mol          IR     RAMAN
     1                      -0.00         0.00000         -        -
     2                      -0.00         0.00000         -        -
     3                      -0.00         0.00000         -        -
     4                       0.00         0.00000         -        -
     5                       0.00         0.00000         -        -
     6                       0.00         0.00000         -        -
     7         a            14.37         0.12208        YES      YES
     8         a            17.48         0.00050        YES      YES
     9         a            20.97         0.05192        YES      YES
    10         a            25.46         0.09588        YES      YES
    11         a            32.99         0.11293        YES      YES
    12         a            34.84         0.02998        YES      YES
    13         a            38.03         0.01755        YES      YES
    14         a            40.46         0.02035        YES      YES
```

15	a	41.65	0.06482	YES	YES
16	a	42.17	0.06873	YES	YES
17	a	42.45	0.10838	YES	YES
18	a	44.32	0.02438	YES	YES
19	a	47.60	0.01750	YES	YES
20	a	48.84	0.00366	YES	YES
21	a	51.34	0.41177	YES	YES
22	a	53.96	0.16881	YES	YES
23	a	54.11	0.00690	YES	YES
24	a	58.97	0.09732	YES	YES
25	a	59.08	0.20270	YES	YES
26	a	63.48	0.02023	YES	YES
27	a	63.71	0.32885	YES	YES
28	a	66.08	0.07733	YES	YES
29	a	67.98	0.01477	YES	YES
30	a	71.40	0.01913	YES	YES
31	a	73.75	0.45551	YES	YES
32	a	79.13	0.08182	YES	YES
33	a	81.50	0.17087	YES	YES
34	a	84.82	0.05708	YES	YES
35	a	90.56	0.06441	YES	YES
36	a	95.11	0.61804	YES	YES
37	a	98.92	0.19252	YES	YES
38	a	103.06	0.66303	YES	YES
39	a	119.49	0.70717	YES	YES
40	a	127.89	0.82497	YES	YES
41	a	130.05	0.20481	YES	YES
42	a	131.46	4.25941	YES	YES
43	a	136.64	0.03807	YES	YES
44	a	139.59	2.58519	YES	YES
45	a	142.99	1.86986	YES	YES
46	a	149.30	0.18461	YES	YES
47	a	151.10	6.60128	YES	YES
48	a	155.71	3.36143	YES	YES
49	a	160.16	1.18870	YES	YES
50	a	163.63	7.15696	YES	YES
51	a	172.09	0.11639	YES	YES
52	a	185.24	2.18259	YES	YES
53	a	188.62	6.12267	YES	YES
54	a	190.50	2.94194	YES	YES
55	a	202.83	1.14813	YES	YES
56	a	209.99	1.77112	YES	YES
57	a	212.73	0.74000	YES	YES
58	a	217.01	0.69324	YES	YES
59	a	218.19	0.16176	YES	YES
60	a	221.75	0.15334	YES	YES
61	a	223.84	0.25419	YES	YES
62	a	225.39	0.98682	YES	YES
63	a	226.83	1.25676	YES	YES
64	a	232.66	0.12796	YES	YES
65	a	238.68	0.29912	YES	YES
66	a	239.17	0.11322	YES	YES
67	a	242.12	1.14715	YES	YES
68	a	243.37	3.09789	YES	YES
69	a	245.66	0.41197	YES	YES
70	a	252.72	1.59766	YES	YES
71	a	256.10	0.73484	YES	YES
72	a	257.38	0.73192	YES	YES

73	a	269.65	2.14814	YES	YES
74	a	275.34	0.27150	YES	YES
75	a	279.68	5.07936	YES	YES
76	a	285.25	0.95394	YES	YES
77	a	296.78	4.79977	YES	YES
78	a	306.88	0.04605	YES	YES
79	a	308.92	1.07389	YES	YES
80	a	311.28	0.29714	YES	YES
81	a	311.64	0.53823	YES	YES
82	a	326.90	0.99346	YES	YES
83	a	329.31	0.74084	YES	YES
84	a	333.41	0.27267	YES	YES
85	a	334.28	0.13865	YES	YES
86	a	336.52	1.27827	YES	YES
87	a	339.95	0.63714	YES	YES
88	a	373.48	2.11865	YES	YES
89	a	377.16	2.71033	YES	YES
90	a	378.16	2.21521	YES	YES
91	a	382.39	0.02619	YES	YES
92	a	392.59	0.96445	YES	YES
93	a	401.64	0.20826	YES	YES
94	a	413.40	2.18294	YES	YES
95	a	414.54	1.96006	YES	YES
96	a	415.63	0.90649	YES	YES
97	a	424.50	1.94804	YES	YES
98	a	426.32	0.42489	YES	YES
99	a	428.52	0.22219	YES	YES
100	a	428.87	0.15894	YES	YES
101	a	429.97	0.90279	YES	YES
102	a	431.59	0.51498	YES	YES
103	a	431.89	0.28993	YES	YES
104	a	432.86	0.48976	YES	YES
105	a	433.74	0.20120	YES	YES
106	a	434.25	0.30092	YES	YES
107	a	436.55	0.21021	YES	YES
108	a	440.14	1.68906	YES	YES
109	a	449.60	4.09486	YES	YES
110	a	450.45	1.21797	YES	YES
111	a	457.19	5.26421	YES	YES
112	a	465.44	6.88462	YES	YES
113	a	493.21	2.29356	YES	YES
114	a	494.78	2.85258	YES	YES
115	a	495.13	3.19741	YES	YES
116	a	497.23	2.94347	YES	YES
117	a	502.81	1.00444	YES	YES
118	a	506.53	8.40648	YES	YES
119	a	507.18	5.48359	YES	YES
120	a	509.19	12.50000	YES	YES
121	a	517.94	18.67793	YES	YES
122	a	519.52	10.90098	YES	YES
123	a	546.03	57.47772	YES	YES
124	a	573.77	113.99102	YES	YES
125	a	594.17	17.22989	YES	YES
126	a	629.76	8.52627	YES	YES
127	a	632.00	14.04521	YES	YES
128	a	632.95	26.86790	YES	YES
129	a	645.72	7.04388	YES	YES
130	a	648.79	11.15250	YES	YES

131	a	691.21	27.00282	YES	YES
132	a	708.79	2.61208	YES	YES
133	a	719.65	2.43784	YES	YES
134	a	725.96	0.50672	YES	YES
135	a	730.71	5.70017	YES	YES
136	a	735.67	1.55428	YES	YES
137	a	736.35	8.07450	YES	YES
138	a	738.18	4.43564	YES	YES
139	a	742.56	4.29300	YES	YES
140	a	751.23	51.21416	YES	YES
141	a	768.93	0.08130	YES	YES
142	a	773.78	0.09821	YES	YES
143	a	774.39	0.33472	YES	YES
144	a	775.59	0.07268	YES	YES
145	a	777.09	0.10095	YES	YES
146	a	778.00	0.14462	YES	YES
147	a	778.59	0.66439	YES	YES
148	a	780.48	0.38201	YES	YES
149	a	783.46	15.59284	YES	YES
150	a	787.70	17.40270	YES	YES
151	a	799.48	0.17907	YES	YES
152	a	808.75	1.63222	YES	YES
153	a	810.58	0.46350	YES	YES
154	a	811.06	4.42785	YES	YES
155	a	812.21	1.39783	YES	YES
156	a	812.68	1.91405	YES	YES
157	a	812.90	2.44495	YES	YES
158	a	815.33	1.76295	YES	YES
159	a	816.04	4.36658	YES	YES
160	a	831.45	4.92843	YES	YES
161	a	833.75	3.79553	YES	YES
162	a	837.62	0.50953	YES	YES
163	a	838.14	0.65853	YES	YES
164	a	838.85	9.07150	YES	YES
165	a	839.15	1.53229	YES	YES
166	a	839.57	7.41459	YES	YES
167	a	840.95	3.42159	YES	YES
168	a	841.52	65.97412	YES	YES
169	a	846.88	3.38326	YES	YES
170	a	852.60	11.61394	YES	YES
171	a	864.94	6.30374	YES	YES
172	a	873.84	1.89206	YES	YES
173	a	874.16	2.29598	YES	YES
174	a	874.51	2.59243	YES	YES
175	a	875.95	1.75780	YES	YES
176	a	876.83	2.02479	YES	YES
177	a	878.06	6.41311	YES	YES
178	a	879.23	5.01709	YES	YES
179	a	880.31	0.50365	YES	YES
180	a	880.45	7.09578	YES	YES
181	a	880.70	1.17649	YES	YES
182	a	880.98	2.63982	YES	YES
183	a	881.83	9.12841	YES	YES
184	a	882.54	1.58700	YES	YES
185	a	885.43	6.02367	YES	YES
186	a	887.71	10.56051	YES	YES
187	a	889.10	0.91011	YES	YES
188	a	903.74	0.45379	YES	YES

189	a	904.97	2.48300	YES	YES
190	a	905.90	0.74600	YES	YES
191	a	906.08	1.39444	YES	YES
192	a	907.83	3.73151	YES	YES
193	a	910.22	1.18966	YES	YES
194	a	910.64	2.44890	YES	YES
195	a	911.37	0.19477	YES	YES
196	a	945.26	0.20218	YES	YES
197	a	971.80	0.10457	YES	YES
198	a	981.38	0.68250	YES	YES
199	a	983.94	0.09092	YES	YES
200	a	988.05	2.75681	YES	YES
201	a	988.96	8.13382	YES	YES
202	a	989.42	9.86602	YES	YES
203	a	990.01	6.73740	YES	YES
204	a	990.95	17.82739	YES	YES
205	a	991.44	8.66617	YES	YES
206	a	992.88	8.55059	YES	YES
207	a	993.49	10.83658	YES	YES
208	a	993.75	8.16740	YES	YES
209	a	1014.01	1.46828	YES	YES
210	a	1014.54	0.38227	YES	YES
211	a	1015.86	0.02547	YES	YES
212	a	1016.28	0.76856	YES	YES
213	a	1017.51	0.31597	YES	YES
214	a	1020.36	0.34918	YES	YES
215	a	1020.61	1.39353	YES	YES
216	a	1021.18	0.10967	YES	YES
217	a	1021.71	2.37523	YES	YES
218	a	1029.98	1.43052	YES	YES
219	a	1030.35	0.09072	YES	YES
220	a	1032.23	0.98958	YES	YES
221	a	1034.91	2.50542	YES	YES
222	a	1035.79	1.36481	YES	YES
223	a	1037.66	2.50222	YES	YES
224	a	1039.23	1.13961	YES	YES
225	a	1040.57	3.00839	YES	YES
226	a	1057.71	0.19134	YES	YES
227	a	1059.38	1.63852	YES	YES
228	a	1060.65	0.08090	YES	YES
229	a	1061.71	1.43796	YES	YES
230	a	1061.85	1.46077	YES	YES
231	a	1065.21	3.68459	YES	YES
232	a	1065.94	0.32303	YES	YES
233	a	1068.59	1.01278	YES	YES
234	a	1069.99	0.49856	YES	YES
235	a	1070.89	0.11520	YES	YES
236	a	1071.15	1.88035	YES	YES
237	a	1072.02	0.29406	YES	YES
238	a	1072.30	6.47567	YES	YES
239	a	1072.72	0.26802	YES	YES
240	a	1073.30	0.18108	YES	YES
241	a	1073.49	0.19096	YES	YES
242	a	1074.02	0.04101	YES	YES
243	a	1076.88	2.26470	YES	YES
244	a	1081.89	5.98152	YES	YES
245	a	1094.45	3.81946	YES	YES
246	a	1095.49	2.37376	YES	YES

247	a	1098.37	5.13140	YES	YES
248	a	1101.06	4.28655	YES	YES
249	a	1107.18	4.58716	YES	YES
250	a	1107.86	4.36045	YES	YES
251	a	1110.54	4.56536	YES	YES
252	a	1113.67	7.23815	YES	YES
253	a	1125.66	1.74707	YES	YES
254	a	1128.79	1.83687	YES	YES
255	a	1149.77	0.22048	YES	YES
256	a	1157.20	15.53964	YES	YES
257	a	1160.32	2.17383	YES	YES
258	a	1160.60	13.28223	YES	YES
259	a	1162.47	5.60471	YES	YES
260	a	1164.26	1.46266	YES	YES
261	a	1165.53	13.66032	YES	YES
262	a	1169.00	6.39340	YES	YES
263	a	1170.90	2.07754	YES	YES
264	a	1171.39	6.74027	YES	YES
265	a	1178.17	11.37222	YES	YES
266	a	1180.85	8.79007	YES	YES
267	a	1183.94	5.85775	YES	YES
268	a	1189.54	2.64173	YES	YES
269	a	1194.65	2.23837	YES	YES
270	a	1195.13	3.40185	YES	YES
271	a	1199.20	1.17734	YES	YES
272	a	1201.76	1.12797	YES	YES
273	a	1240.50	0.91407	YES	YES
274	a	1242.03	1.24770	YES	YES
275	a	1245.29	19.74770	YES	YES
276	a	1245.84	6.93045	YES	YES
277	a	1246.40	7.55115	YES	YES
278	a	1247.25	0.49115	YES	YES
279	a	1248.13	6.51768	YES	YES
280	a	1248.58	5.03075	YES	YES
281	a	1249.16	21.38940	YES	YES
282	a	1251.11	1.16190	YES	YES
283	a	1252.01	41.32421	YES	YES
284	a	1253.19	12.95704	YES	YES
285	a	1254.57	10.79304	YES	YES
286	a	1254.89	0.56423	YES	YES
287	a	1256.23	2.92797	YES	YES
288	a	1257.12	4.42639	YES	YES
289	a	1257.76	7.60571	YES	YES
290	a	1258.18	15.92880	YES	YES
291	a	1258.39	8.79545	YES	YES
292	a	1260.81	16.18501	YES	YES
293	a	1261.05	1.02223	YES	YES
294	a	1261.76	6.19704	YES	YES
295	a	1263.72	2.63683	YES	YES
296	a	1264.44	2.93713	YES	YES
297	a	1264.70	13.17240	YES	YES
298	a	1265.14	9.40695	YES	YES
299	a	1269.03	3.07506	YES	YES
300	a	1270.08	1.64963	YES	YES
301	a	1274.56	1.85672	YES	YES
302	a	1279.91	0.40067	YES	YES
303	a	1281.91	7.93124	YES	YES
304	a	1284.05	6.76892	YES	YES

305	a	1285.27	3.48631	YES	YES
306	a	1286.14	2.29432	YES	YES
307	a	1288.66	4.85353	YES	YES
308	a	1291.13	7.24316	YES	YES
309	a	1291.61	4.80365	YES	YES
310	a	1294.26	3.76066	YES	YES
311	a	1305.11	2.71734	YES	YES
312	a	1308.36	1.35844	YES	YES
313	a	1310.38	1.20792	YES	YES
314	a	1311.42	2.89328	YES	YES
315	a	1311.48	0.84555	YES	YES
316	a	1314.84	1.46825	YES	YES
317	a	1316.57	1.45076	YES	YES
318	a	1318.23	0.60925	YES	YES
319	a	1321.62	2.91062	YES	YES
320	a	1321.99	1.92351	YES	YES
321	a	1322.46	0.47433	YES	YES
322	a	1323.02	0.34280	YES	YES
323	a	1323.56	0.37437	YES	YES
324	a	1324.32	2.92488	YES	YES
325	a	1324.46	1.41180	YES	YES
326	a	1324.80	0.56213	YES	YES
327	a	1325.20	0.49959	YES	YES
328	a	1325.60	1.58676	YES	YES
329	a	1325.79	0.15611	YES	YES
330	a	1326.57	2.81818	YES	YES
331	a	1327.95	0.54680	YES	YES
332	a	1328.39	2.22231	YES	YES
333	a	1329.30	6.16966	YES	YES
334	a	1332.56	0.53707	YES	YES
335	a	1336.97	0.44299	YES	YES
336	a	1337.26	4.01899	YES	YES
337	a	1337.41	0.18883	YES	YES
338	a	1337.81	0.33030	YES	YES
339	a	1338.06	0.08250	YES	YES
340	a	1338.20	0.05920	YES	YES
341	a	1338.81	0.32037	YES	YES
342	a	1339.17	0.13151	YES	YES
343	a	1339.60	0.28135	YES	YES
344	a	1344.31	2.35613	YES	YES
345	a	1344.96	0.56582	YES	YES
346	a	1345.09	0.32415	YES	YES
347	a	1346.72	1.68327	YES	YES
348	a	1346.78	2.23283	YES	YES
349	a	1349.50	1.52044	YES	YES
350	a	1350.01	0.47277	YES	YES
351	a	1350.76	0.97957	YES	YES
352	a	1404.79	8.83531	YES	YES
353	a	1404.85	5.43816	YES	YES
354	a	1407.14	8.50251	YES	YES
355	a	1407.84	6.97610	YES	YES
356	a	1419.90	2.02817	YES	YES
357	a	1421.82	8.72899	YES	YES
358	a	1427.30	1.92869	YES	YES
359	a	1428.33	0.85561	YES	YES
360	a	1429.62	3.61514	YES	YES
361	a	1430.34	8.17764	YES	YES
362	a	1432.68	2.24527	YES	YES

363	a	1433.86	1.97238	YES	YES
364	a	1434.46	2.35075	YES	YES
365	a	1436.12	7.34078	YES	YES
366	a	1436.38	2.68026	YES	YES
367	a	1436.87	8.44497	YES	YES
368	a	1437.15	5.42842	YES	YES
369	a	1437.47	5.27098	YES	YES
370	a	1437.81	0.93595	YES	YES
371	a	1438.14	6.71046	YES	YES
372	a	1438.38	4.97397	YES	YES
373	a	1438.73	2.28899	YES	YES
374	a	1439.33	3.66598	YES	YES
375	a	1439.40	5.00650	YES	YES
376	a	1439.59	3.70361	YES	YES
377	a	1440.06	4.44484	YES	YES
378	a	1440.20	10.29502	YES	YES
379	a	1441.08	2.85738	YES	YES
380	a	1441.93	9.75656	YES	YES
381	a	1442.36	2.05498	YES	YES
382	a	1443.00	15.69547	YES	YES
383	a	1443.88	7.22510	YES	YES
384	a	1444.61	14.18938	YES	YES
385	a	1445.04	6.91133	YES	YES
386	a	1445.58	40.87799	YES	YES
387	a	1446.19	1.85346	YES	YES
388	a	1447.31	7.21682	YES	YES
389	a	1451.79	1.75711	YES	YES
390	a	1453.23	2.56015	YES	YES
391	a	1453.81	5.36453	YES	YES
392	a	1454.79	0.76649	YES	YES
393	a	1455.06	0.31950	YES	YES
394	a	1455.48	2.04158	YES	YES
395	a	1456.48	0.36127	YES	YES
396	a	1458.60	2.86447	YES	YES
397	a	1475.84	121.19521	YES	YES
398	a	1568.43	6.91131	YES	YES
399	a	1588.02	72.51622	YES	YES
400	a	1853.18	127.44482	YES	YES
401	a	2924.43	2.81687	YES	YES
402	a	2931.94	3.20659	YES	YES
403	a	2933.65	12.94933	YES	YES
404	a	2935.85	23.96962	YES	YES
405	a	2936.67	26.26373	YES	YES
406	a	2937.07	22.87281	YES	YES
407	a	2937.09	3.61856	YES	YES
408	a	2939.00	11.31471	YES	YES
409	a	2939.57	0.43054	YES	YES
410	a	2939.70	25.35663	YES	YES
411	a	2940.86	12.19668	YES	YES
412	a	2941.46	18.90799	YES	YES
413	a	2941.77	8.42300	YES	YES
414	a	2942.72	17.44220	YES	YES
415	a	2943.99	8.80472	YES	YES
416	a	2944.23	9.41815	YES	YES
417	a	2945.61	6.88700	YES	YES
418	a	2946.67	9.95194	YES	YES
419	a	2946.99	13.60839	YES	YES
420	a	2947.10	9.61891	YES	YES

421	a	2947.15	16.28841	YES	YES
422	a	2947.36	3.78492	YES	YES
423	a	2949.94	24.84121	YES	YES
424	a	2950.51	0.35508	YES	YES
425	a	2951.44	8.37137	YES	YES
426	a	2952.69	12.19059	YES	YES
427	a	2952.94	12.61469	YES	YES
428	a	2953.99	7.20606	YES	YES
429	a	2955.45	2.65718	YES	YES
430	a	2955.77	12.38134	YES	YES
431	a	2956.33	2.77701	YES	YES
432	a	2957.65	8.91904	YES	YES
433	a	2957.86	5.99005	YES	YES
434	a	2958.07	2.82519	YES	YES
435	a	2958.69	11.55284	YES	YES
436	a	2959.12	6.07934	YES	YES
437	a	2960.07	34.96864	YES	YES
438	a	2960.50	16.85739	YES	YES
439	a	2961.36	33.87820	YES	YES
440	a	2961.47	32.38227	YES	YES
441	a	2962.20	22.58178	YES	YES
442	a	2962.42	50.72060	YES	YES
443	a	2962.78	33.58646	YES	YES
444	a	2963.21	28.12372	YES	YES
445	a	2965.13	30.81235	YES	YES
446	a	2968.58	8.77525	YES	YES
447	a	2972.02	9.88181	YES	YES
448	a	2972.50	14.16191	YES	YES
449	a	2983.74	33.46931	YES	YES
450	a	2985.34	0.54146	YES	YES
451	a	2988.00	3.88567	YES	YES
452	a	2989.43	45.22784	YES	YES
453	a	2992.89	21.84662	YES	YES
454	a	2994.57	19.33624	YES	YES
455	a	2994.98	14.57507	YES	YES
456	a	2997.36	2.12854	YES	YES
457	a	3000.84	27.22731	YES	YES
458	a	3001.23	26.01276	YES	YES
459	a	3001.87	34.85838	YES	YES
460	a	3004.08	25.97433	YES	YES
461	a	3005.10	20.81462	YES	YES
462	a	3007.29	5.52585	YES	YES
463	a	3007.37	31.89315	YES	YES
464	a	3007.69	19.04130	YES	YES
465	a	3007.80	24.26779	YES	YES
466	a	3008.80	27.28612	YES	YES
467	a	3009.66	23.12102	YES	YES
468	a	3010.57	7.62176	YES	YES
469	a	3010.66	30.51999	YES	YES
470	a	3011.55	12.85168	YES	YES
471	a	3012.09	34.88854	YES	YES
472	a	3012.11	11.91676	YES	YES
473	a	3012.33	14.64274	YES	YES
474	a	3012.67	20.68037	YES	YES
475	a	3014.48	31.75015	YES	YES
476	a	3015.81	43.73472	YES	YES
477	a	3016.45	22.38832	YES	YES
478	a	3016.62	17.37536	YES	YES

479	a	3016.92	17.21726	YES	YES
480	a	3017.26	19.30054	YES	YES
481	a	3017.37	23.87894	YES	YES
482	a	3017.48	4.99925	YES	YES
483	a	3017.51	44.80340	YES	YES
484	a	3018.19	28.88480	YES	YES
485	a	3018.25	16.05277	YES	YES
486	a	3019.15	31.26831	YES	YES
487	a	3019.33	16.90469	YES	YES
488	a	3019.60	18.74422	YES	YES
489	a	3019.83	21.50981	YES	YES
490	a	3022.24	16.86284	YES	YES
491	a	3027.76	7.03048	YES	YES
492	a	3028.30	12.72725	YES	YES
493	a	3032.32	1.59768	YES	YES
494	a	3035.15	1.21029	YES	YES
495	a	3045.25	0.88040	YES	YES
496	a	3052.77	0.26821	YES	YES
497	a	3064.70	28.66553	YES	YES
498	a	3105.38	3.64997	YES	YES
499	a	3109.11	3.41923	YES	YES
500	a	3121.17	5.02853	YES	YES
501	a	3136.35	2.41675	YES	YES

$end

Double hybrid single point energy = -7556.212272619291 H
COSMO enery + OC correction = -7564.0224598681 H (in *o*DFB)

6.2.13 $HNPh_2$

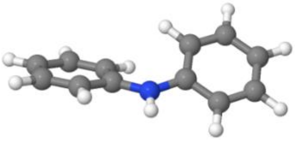

Method: (RI-)BP86(D3BJ)/def2-TZVPP
Symmetry: c2

Cartesian coordinates in Ångström:

H	-2.0277022	2.2948717	2.3545109
C	-2.0994247	1.8853110	1.3466016
C	-3.2673503	2.0661356	0.6014583
H	-4.1158509	2.6023540	1.0239277
C	-3.3267870	1.5538320	-0.6978155
H	-4.2264014	1.6882635	-1.2986907
C	-2.2474930	0.8561895	-1.2332830
H	-2.3082603	0.4434822	-2.2420668
C	-1.0709239	0.6661732	-0.4831060
C	-1.0061567	1.2013719	0.8167076
H	-0.0912977	1.1062499	1.3973107
H	4.1158509	-2.6023540	1.0239277
C	3.2673503	-2.0661356	0.6014583
C	2.0994247	-1.8853110	1.3466016
H	2.0277022	-2.2948717	2.3545109
C	1.0061567	-1.2013719	0.8167076
H	0.0912977	-1.1062499	1.3973107
C	1.0709239	-0.6661732	-0.4831060
C	2.2474930	-0.8561895	-1.2332830

```
H     2.3082603   -0.4434822   -2.2420668
C     3.3267870   -1.5538320   -0.6978155
H     4.2264014   -1.6882635   -1.2986907
N     0.0000000    0.0000000   -1.0809386
H    -0.0000000    0.0000000   -2.0944000

SCF energy GEOOPT =  -518.9110712150 H
ZPE =   505.3 kJ/mol
FREEH energy =   533.10 kJ/mol
FREEH entropy =   0.42326 kJ/mol/K

$vibrational spectrum
#  mode     symmetry     wave number   IR intensity    selection rules
#                         cm**(-1)        km/mol          IR     RAMAN
     1                      -0.00          0.00000        -        -
     2                      -0.00          0.00000        -        -
     3                      -0.00          0.00000        -        -
     4                      -0.00          0.00000        -        -
     5                       0.00          0.00000        -        -
     6                       0.00          0.00000        -        -
     7        b             34.70          0.96053       YES      YES
     8        a             51.79          0.01339       YES      YES
     9        a            105.00          0.01997       YES      YES
    10        b            211.01          4.32617       YES      YES
    11        a            219.71          0.39138       YES      YES
    12        a            304.04          0.21503       YES      YES
    13        b            327.79          3.12441       YES      YES
    14        b            378.67        111.24827       YES      YES
    15        b            401.47          1.80539       YES      YES
    16        a            408.32          0.55764       YES      YES
    17        a            484.76          2.78715       YES      YES
    18        b            494.33         30.92078       YES      YES
    19        b            564.85          3.59097       YES      YES
    20        a            609.55          0.41690       YES      YES
    21        b            614.78          1.48263       YES      YES
    22        a            637.41          1.20126       YES      YES
    23        b            682.06         61.48215       YES      YES
    24        a            690.22          8.03696       YES      YES
    25        b            733.02         80.96426       YES      YES
    26        a            736.27          6.15904       YES      YES
    27        b            798.51          1.72736       YES      YES
    28        a            800.54          0.02004       YES      YES
    29        a            816.78          0.08278       YES      YES
    30        b            859.44          5.43365       YES      YES
    31        a            877.07          1.11833       YES      YES
    32        b            877.24          2.81236       YES      YES
    33        b            932.15          0.08290       YES      YES
    34        a            936.45          0.00015       YES      YES
    35        b            948.79          0.80605       YES      YES
    36        a            957.86          0.18082       YES      YES
    37        b            988.17          6.56880       YES      YES
    38        a            989.33          0.50624       YES      YES
    39        b           1025.11          8.56469       YES      YES
    40        a           1026.52          0.30624       YES      YES
    41        b           1074.64          3.03414       YES      YES
    42        a           1087.08          9.98489       YES      YES
    43        b           1148.31          0.36807       YES      YES
    44        a           1148.73          1.46048       YES      YES
    45        b           1166.17         16.91784       YES      YES
    46        a           1171.82          2.20247       YES      YES
    47        a           1225.69          3.69980       YES      YES
    48        b           1246.25         21.72136       YES      YES
```

49	b	1311.88	117.71877	YES	YES
50	a	1319.09	0.13223	YES	YES
51	a	1347.16	3.36461	YES	YES
52	b	1350.50	89.27851	YES	YES
53	b	1411.06	28.97426	YES	YES
54	a	1455.11	3.14714	YES	YES
55	b	1483.07	103.61249	YES	YES
56	a	1488.09	4.61259	YES	YES
57	b	1510.22	314.38206	YES	YES
58	a	1578.55	8.49564	YES	YES
59	b	1584.83	15.88874	YES	YES
60	b	1594.72	306.55038	YES	YES
61	a	1607.51	6.75258	YES	YES
62	b	3086.10	12.76804	YES	YES
63	a	3086.41	10.85731	YES	YES
64	b	3100.24	1.90992	YES	YES
65	a	3100.81	1.26232	YES	YES
66	b	3109.08	6.13028	YES	YES
67	a	3109.33	35.45085	YES	YES
68	b	3125.59	41.38305	YES	YES
69	a	3126.91	0.70672	YES	YES
70	b	3131.90	2.19791	YES	YES
71	a	3138.72	3.22969	YES	YES
72	a	3534.65	18.05234	YES	YES

$end

Double hybrid single point energy = -517.948290328414 H
COSMO enery + OC correction = –518.9204541244 H (in *o*DFB)

6.2.14 [H{Ga(dcpe)}$_2$(NPh$_2$)]$^{2+}$

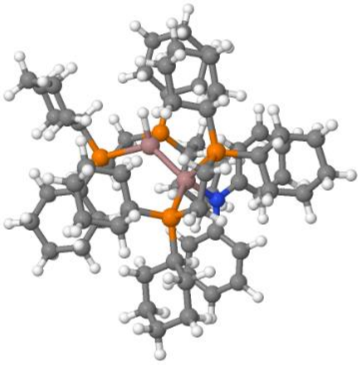

Method: (RI-)BP86(D3BJ)/def2-TZVPP
Symmetry: c1

Cartesian coordinates in Ångström:

Ga	15.8345254	15.7008335	15.7583697
H	16.4129480	17.0787599	15.2417361
Ga	14.1966392	15.7607551	17.5814181
P	17.8143307	14.3320836	16.0266561
P	15.5826409	14.5650722	13.6604906
P	14.1680710	18.0013843	18.6431219
P	11.7891351	16.0762750	17.2400814
N	14.3980450	14.5273292	19.0583049
C	12.4588702	18.6328611	18.3527256
H	12.2142605	19.3632120	19.1370553
H	12.4658475	19.1799942	17.4028188
C	11.4085437	17.5181676	18.3306587
H	10.4257814	17.9211621	18.0533248

H	11.2993219	17.0847562	19.3332583
C	17.9148771	13.2884503	14.4966218
H	18.5661248	13.8196095	13.7904689
H	18.4204071	12.3460432	14.7486401
C	16.5491794	13.0106557	13.8525869
H	15.9532150	12.3378756	14.4824728
H	16.6748876	12.5230014	12.8765371
C	15.3120176	19.2959712	17.9892472
H	15.1050914	20.2025380	18.5833576
C	15.0739640	19.6186751	16.5050313
H	14.0553682	20.0008679	16.3507166
H	15.1721915	18.6961108	15.9137889
C	16.0868456	20.6527882	15.9945391
H	15.9152455	20.8301952	14.9237362
H	15.9130433	21.6151019	16.5026068
C	17.5293612	20.2016478	16.2423760
H	17.7295012	19.2869950	15.6580920
H	18.2332885	20.9662156	15.8873441
C	17.7600735	19.9096601	17.7267052
H	17.6473292	20.8374897	18.3101897
H	18.7860937	19.5558988	17.9007669
C	16.7674818	18.8618582	18.2370358
H	16.9373809	18.6545522	19.3027121
H	16.9425104	17.9190512	17.6931245
C	14.4072000	17.9404515	20.4726231
H	15.3637259	17.4062093	20.5859989
C	13.3244866	17.0824468	21.1482755
H	12.3578836	17.6110723	21.1000365
H	13.2095871	16.1269134	20.6198422
C	13.6877039	16.8520558	22.6193605
H	12.9137613	16.2398219	23.1037411
H	14.6260470	16.2755742	22.6655960
C	13.8503617	18.1871416	23.3538373
H	14.1575286	18.0150263	24.3940607
H	12.8734942	18.6966973	23.3979910
C	14.8650075	19.0960469	22.6520473
H	15.8689512	18.6466123	22.7233092
H	14.9204928	20.0708009	23.1553632
C	14.5287565	19.3056593	21.1658473
H	13.5808289	19.8612316	21.0729690
H	15.3096945	19.9211260	20.6991122
C	11.4597961	16.5839740	15.4888961
H	11.5049749	15.6270966	14.9397184
C	10.0682669	17.2026629	15.2720660
H	9.9940078	18.1411559	15.8447465
H	9.2828310	16.5370500	15.6508767
C	9.8290811	17.5016268	13.7862835
H	9.8094341	16.5512833	13.2270397
H	8.8385779	17.9584969	13.6592890
C	10.9195548	18.4103502	13.2147844
H	10.7496652	18.5862067	12.1441616
H	10.8678850	19.3965956	13.7047997
C	12.3081846	17.8063279	13.4384408
H	13.0910555	18.4816705	13.0665098
H	12.3941526	16.8744844	12.8541340
C	12.5579416	17.4963793	14.9183776
H	12.5804545	18.4437438	15.4757222
H	13.5486302	17.0333980	15.0507562
C	10.5308587	14.7875519	17.7240607
H	11.0379769	14.2092654	18.5117369
C	10.2506199	13.8452643	16.5423637
H	9.8474831	14.4201338	15.6952779

H	11.1924112	13.3920441	16.2055056
C	9.2399808	12.7513919	16.9062377
H	9.0241603	12.1498357	16.0116996
H	9.6918432	12.0753911	17.6453149
C	7.9542029	13.3413332	17.4879751
H	7.2641871	12.5362639	17.7737266
H	7.4354882	13.9401484	16.7207089
C	8.2709151	14.2243820	18.6964359
H	8.7299317	13.6117120	19.4893491
H	7.3522199	14.6536374	19.1187525
C	9.2317823	15.3614060	18.3232736
H	8.7354903	16.0287017	17.6026288
H	9.4492265	15.9599422	19.2170347
C	13.4199517	13.5205602	19.2475783
C	12.6942322	13.3982678	20.4470373
H	12.8865816	14.0929301	21.2630944
C	11.7475259	12.3854494	20.6036509
H	11.2024197	12.3006288	21.5433081
C	11.5013447	11.4788525	19.5685274
H	10.7748183	10.6786641	19.7013951
C	12.1968924	11.6083150	18.3645157
H	12.0122828	10.9089103	17.5494598
C	13.1407385	12.6224803	18.2042015
H	13.6944148	12.7121815	17.2694099
C	15.5066135	14.6056138	19.9014059
C	16.5232143	15.5488639	19.6305683
H	16.4384949	16.1868018	18.7500101
C	17.6451089	15.6783285	20.4474988
H	18.3928082	16.4346966	20.2089434
C	17.8146298	14.8484983	21.5564196
H	18.6866329	14.9476376	22.1998056
C	16.8384759	13.8811878	21.8168339
H	16.9555533	13.2081241	22.6660537
C	15.7069869	13.7553268	21.0136279
H	14.9757270	12.9860255	21.2479094
C	16.5054395	15.6200357	12.4601000
H	17.3197225	16.0248229	13.0877199
C	15.6369205	16.8173836	12.0385245
H	15.2185630	17.3096475	12.9296182
H	14.7865108	16.4613995	11.4353151
C	16.4640011	17.8146251	11.2183117
H	15.8229057	18.6431025	10.8868495
H	17.2380489	18.2543362	11.8687865
C	17.1292735	17.1349607	10.0170142
H	16.3515863	16.7928547	9.3143147
H	17.7469831	17.8576979	9.4674852
C	17.9797392	15.9370105	10.4520815
H	18.4235790	15.4407093	9.5786383
H	18.8196659	16.2895746	11.0739994
C	17.1529782	14.9216889	11.2546477
H	17.7947141	14.0905075	11.5811387
H	16.3809087	14.4862115	10.6049325
C	19.3889776	15.2888991	16.0814460
H	20.1993191	14.5396116	16.0621752
C	19.4665773	16.0780987	17.3983153
H	18.6040927	16.7636852	17.4444086
H	19.3776703	15.4037115	18.2599832
C	20.7637680	16.8898134	17.4848200
H	20.7712813	17.4702764	18.4182706
H	21.6191348	16.1975948	17.5429098
C	20.9323910	17.8113070	16.2738162
H	20.1376532	18.5757196	16.2819883

```
H    21.8865210   18.3512251   16.3358340
C    20.8537150   17.0223018   14.9639288
H    21.7081339   16.3295826   14.8983255
H    20.9316659   17.6979129   14.1010742
C    19.5468401   16.2241546   14.8705048
H    19.5289624   15.6549666   13.9293747
H    18.6919315   16.9197662   14.8441524
C    13.9159245   14.1122878   13.0259928
H    13.3859882   15.0784253   12.9598977
C    13.1775256   13.2358134   14.0548529
H    13.7336281   12.2971133   14.2075770
H    13.1470932   13.7464952   15.0315576
C    11.7620208   12.8967665   13.5710548
H    11.2717710   12.2362313   14.2986805
H    11.1621055   13.8219769   13.5322914
C    11.7769328   12.2469580   12.1853269
H    12.2844893   11.2701577   12.2453885
H    10.7501098   12.0439343   11.8531962
C    12.4991771   13.1351336   11.1711824
H    11.9368104   14.0742623   11.0374036
H    12.5352410   12.6502615   10.1865106
C    13.9248735   13.4662115   11.6294779
H    14.4017221   14.1372132   10.9045993
H    14.5246885   12.5424568   11.6545566
C    17.7734918   13.1720857   17.4510861
H    17.5313262   13.8142768   18.3139436
C    19.0993730   12.4512275   17.7326199
H    19.3828330   11.8302176   16.8661446
H    19.9118521   13.1735786   17.8896289
C    18.9474969   11.5598005   18.9744557
H    19.8936184   11.0367424   19.1683241
H    18.7532097   12.2041411   19.8475125
C    17.8005993   10.5568307   18.8131144
H    17.6940238    9.9551483   19.7257535
H    18.0440285    9.8509217   18.0014259
C    16.4811844   11.2672373   18.4956879
H    16.1806647   11.8849270   19.3543119
H    15.6742179   10.5401458   18.3300043
C    16.6220052   12.1699315   17.2663261
H    15.6836637   12.7124108   17.0786705
H    16.8223452   11.5400041   16.3835508

SCF energy GEOOPT =  -7775.157050149 H
ZPE =   4103. kJ/mol
FREEH energy =  4307.76 kJ/mol
FREEH entropy =   1.69830 kJ/mol/K

$vibrational spectrum
#  mode     symmetry     wave number   IR intensity    selection rules
#                         cm**(-1)        km/mol          IR     RAMAN
     1                      -0.00         0.00000         -        -
     2                       0.00         0.00000         -        -
     3                       0.00         0.00000         -        -
     4                       0.00         0.00000         -        -
     5                       0.00         0.00000         -        -
     6                       0.00         0.00000         -        -
     7        a             16.54         0.05790        YES      YES
     8        a             20.21         0.01515        YES      YES
     9        a             23.70         0.04546        YES      YES
    10        a             28.01         0.00140        YES      YES
    11        a             30.05         0.09282        YES      YES
    12        a             33.82         0.04400        YES      YES
```

13	a	36.43	0.03616	YES	YES
14	a	38.30	0.16492	YES	YES
15	a	42.32	0.01818	YES	YES
16	a	42.73	0.03859	YES	YES
17	a	44.57	0.15007	YES	YES
18	a	46.31	0.22601	YES	YES
19	a	50.64	0.08237	YES	YES
20	a	52.39	0.04584	YES	YES
21	a	54.13	0.09599	YES	YES
22	a	56.17	0.03978	YES	YES
23	a	56.60	0.05212	YES	YES
24	a	58.45	0.11317	YES	YES
25	a	60.78	0.03550	YES	YES
26	a	63.32	0.21647	YES	YES
27	a	68.15	0.10036	YES	YES
28	a	69.32	0.08881	YES	YES
29	a	72.66	0.08128	YES	YES
30	a	74.84	0.20032	YES	YES
31	a	76.81	0.10243	YES	YES
32	a	79.28	0.13917	YES	YES
33	a	80.40	0.08558	YES	YES
34	a	84.35	0.21168	YES	YES
35	a	86.62	0.24487	YES	YES
36	a	90.56	0.05214	YES	YES
37	a	94.81	0.04953	YES	YES
38	a	99.49	1.04265	YES	YES
39	a	105.77	0.14915	YES	YES
40	a	113.17	0.40540	YES	YES
41	a	117.42	0.71260	YES	YES
42	a	123.02	2.58232	YES	YES
43	a	124.35	0.12862	YES	YES
44	a	131.40	1.55619	YES	YES
45	a	136.06	2.43822	YES	YES
46	a	139.04	0.10195	YES	YES
47	a	141.54	4.92556	YES	YES
48	a	147.40	1.64473	YES	YES
49	a	151.59	1.23959	YES	YES
50	a	153.60	1.80427	YES	YES
51	a	154.24	3.11864	YES	YES
52	a	158.85	0.23118	YES	YES
53	a	168.13	1.70193	YES	YES
54	a	168.64	2.16593	YES	YES
55	a	186.03	4.28931	YES	YES
56	a	187.56	5.54166	YES	YES
57	a	191.92	1.89312	YES	YES
58	a	206.70	2.30570	YES	YES
59	a	209.94	0.47234	YES	YES
60	a	211.18	1.18237	YES	YES
61	a	216.97	0.44644	YES	YES
62	a	219.39	0.32143	YES	YES
63	a	220.76	0.42007	YES	YES
64	a	221.99	0.78989	YES	YES
65	a	226.50	2.14264	YES	YES
66	a	229.63	0.25479	YES	YES
67	a	233.80	1.33842	YES	YES
68	a	235.18	0.20633	YES	YES
69	a	237.34	0.15662	YES	YES
70	a	239.35	0.83400	YES	YES
71	a	241.09	1.91189	YES	YES
72	a	245.68	0.54849	YES	YES
73	a	246.56	0.94903	YES	YES
74	a	247.70	1.67635	YES	YES

75	a	254.37	0.48157	YES	YES
76	a	264.39	0.73231	YES	YES
77	a	265.43	2.06273	YES	YES
78	a	273.31	0.29244	YES	YES
79	a	277.68	1.13903	YES	YES
80	a	285.32	0.39141	YES	YES
81	a	291.60	2.90717	YES	YES
82	a	309.90	0.15892	YES	YES
83	a	310.26	0.46942	YES	YES
84	a	311.34	0.10359	YES	YES
85	a	312.79	0.15210	YES	YES
86	a	321.65	6.50432	YES	YES
87	a	329.67	1.02892	YES	YES
88	a	330.54	0.07750	YES	YES
89	a	334.26	0.21032	YES	YES
90	a	334.96	0.06499	YES	YES
91	a	339.09	0.33129	YES	YES
92	a	340.67	1.22070	YES	YES
93	a	363.86	0.47549	YES	YES
94	a	377.40	0.74933	YES	YES
95	a	380.29	1.00266	YES	YES
96	a	387.52	1.36200	YES	YES
97	a	389.67	0.37018	YES	YES
98	a	394.01	8.44521	YES	YES
99	a	399.18	0.63660	YES	YES
100	a	411.34	0.48319	YES	YES
101	a	412.28	0.98786	YES	YES
102	a	416.85	0.71902	YES	YES
103	a	420.03	1.10523	YES	YES
104	a	425.50	0.15888	YES	YES
105	a	426.42	2.59356	YES	YES
106	a	427.44	0.15256	YES	YES
107	a	429.26	0.51917	YES	YES
108	a	430.26	0.27194	YES	YES
109	a	430.48	0.12554	YES	YES
110	a	432.25	0.18105	YES	YES
111	a	432.92	0.13254	YES	YES
112	a	434.11	0.53872	YES	YES
113	a	437.40	0.36346	YES	YES
114	a	441.26	2.46175	YES	YES
115	a	445.26	1.57592	YES	YES
116	a	449.23	2.67741	YES	YES
117	a	451.91	4.04898	YES	YES
118	a	463.81	7.69032	YES	YES
119	a	464.61	0.90238	YES	YES
120	a	492.28	1.07048	YES	YES
121	a	494.14	2.51020	YES	YES
122	a	497.53	1.10048	YES	YES
123	a	497.95	3.83030	YES	YES
124	a	499.47	5.04015	YES	YES
125	a	501.04	10.83095	YES	YES
126	a	503.86	0.87137	YES	YES
127	a	510.39	11.00929	YES	YES
128	a	512.01	12.18936	YES	YES
129	a	514.78	8.32454	YES	YES
130	a	567.63	57.22360	YES	YES
131	a	579.76	0.87807	YES	YES
132	a	614.85	1.49377	YES	YES
133	a	616.51	8.70519	YES	YES
134	a	618.84	125.62923	YES	YES
135	a	638.13	13.38952	YES	YES
136	a	638.33	9.00420	YES	YES

137	a	646.08	12.48219	YES	YES
138	a	649.03	12.53964	YES	YES
139	a	664.74	1.03881	YES	YES
140	a	689.91	36.92177	YES	YES
141	a	700.39	5.39459	YES	YES
142	a	703.01	27.72527	YES	YES
143	a	714.01	7.86276	YES	YES
144	a	724.98	7.08516	YES	YES
145	a	729.96	2.77073	YES	YES
146	a	731.38	7.51011	YES	YES
147	a	731.84	4.99596	YES	YES
148	a	734.69	3.58692	YES	YES
149	a	742.33	29.81982	YES	YES
150	a	743.41	12.37670	YES	YES
151	a	755.53	18.31608	YES	YES
152	a	771.82	0.60138	YES	YES
153	a	772.15	0.18835	YES	YES
154	a	772.84	0.29454	YES	YES
155	a	774.83	0.24220	YES	YES
156	a	775.44	0.07817	YES	YES
157	a	776.28	0.55565	YES	YES
158	a	776.67	0.29962	YES	YES
159	a	781.15	0.25458	YES	YES
160	a	785.41	23.49967	YES	YES
161	a	786.02	10.60684	YES	YES
162	a	797.09	0.42585	YES	YES
163	a	806.79	1.73526	YES	YES
164	a	810.22	2.42059	YES	YES
165	a	811.36	2.06726	YES	YES
166	a	812.15	0.88953	YES	YES
167	a	812.76	2.51553	YES	YES
168	a	813.50	1.88993	YES	YES
169	a	813.63	2.25494	YES	YES
170	a	817.83	1.29924	YES	YES
171	a	823.43	3.69561	YES	YES
172	a	832.54	3.73161	YES	YES
173	a	834.41	1.94774	YES	YES
174	a	836.81	5.32290	YES	YES
175	a	838.41	2.50297	YES	YES
176	a	838.68	1.34390	YES	YES
177	a	839.46	5.43504	YES	YES
178	a	840.37	5.73916	YES	YES
179	a	840.95	1.65252	YES	YES
180	a	849.13	3.65923	YES	YES
181	a	853.43	11.36124	YES	YES
182	a	858.88	6.83855	YES	YES
183	a	865.13	31.41418	YES	YES
184	a	873.88	5.66361	YES	YES
185	a	874.86	2.86405	YES	YES
186	a	875.69	3.02179	YES	YES
187	a	876.88	3.56247	YES	YES
188	a	877.38	1.37457	YES	YES
189	a	878.61	2.66484	YES	YES
190	a	879.56	1.88663	YES	YES
191	a	879.92	4.84511	YES	YES
192	a	880.34	1.11112	YES	YES
193	a	880.91	1.24879	YES	YES
194	a	882.01	2.35475	YES	YES
195	a	882.54	3.69035	YES	YES
196	a	882.91	3.74534	YES	YES
197	a	883.51	6.33401	YES	YES
198	a	884.06	9.81285	YES	YES

199	a	886.59	1.19740	YES	YES
200	a	889.00	8.78367	YES	YES
201	a	904.17	2.19195	YES	YES
202	a	905.28	1.17357	YES	YES
203	a	905.61	2.34507	YES	YES
204	a	906.02	1.77881	YES	YES
205	a	907.25	2.38570	YES	YES
206	a	908.80	6.07999	YES	YES
207	a	909.41	1.92581	YES	YES
208	a	911.84	9.43840	YES	YES
209	a	912.50	1.58492	YES	YES
210	a	943.30	0.56546	YES	YES
211	a	957.28	0.31625	YES	YES
212	a	970.56	0.17517	YES	YES
213	a	980.11	0.17930	YES	YES
214	a	984.61	3.54380	YES	YES
215	a	985.70	6.42650	YES	YES
216	a	985.82	15.57351	YES	YES
217	a	987.80	7.35869	YES	YES
218	a	989.83	6.40578	YES	YES
219	a	990.02	12.99679	YES	YES
220	a	990.68	1.94279	YES	YES
221	a	991.94	9.31683	YES	YES
222	a	992.85	20.77695	YES	YES
223	a	993.20	7.12713	YES	YES
224	a	997.00	0.45783	YES	YES
225	a	1003.54	3.82396	YES	YES
226	a	1013.92	1.51974	YES	YES
227	a	1014.56	0.49628	YES	YES
228	a	1015.65	0.26901	YES	YES
229	a	1017.15	0.25142	YES	YES
230	a	1017.95	0.62188	YES	YES
231	a	1020.37	0.49181	YES	YES
232	a	1021.29	1.24287	YES	YES
233	a	1022.38	0.03065	YES	YES
234	a	1025.55	3.22008	YES	YES
235	a	1031.55	1.07334	YES	YES
236	a	1032.02	3.75871	YES	YES
237	a	1033.17	0.65472	YES	YES
238	a	1036.25	1.00807	YES	YES
239	a	1036.94	2.66989	YES	YES
240	a	1039.20	3.90206	YES	YES
241	a	1040.26	1.97132	YES	YES
242	a	1040.55	0.92423	YES	YES
243	a	1041.03	2.68009	YES	YES
244	a	1057.93	0.08951	YES	YES
245	a	1058.91	0.14320	YES	YES
246	a	1060.56	2.72770	YES	YES
247	a	1061.94	1.88031	YES	YES
248	a	1062.31	0.99530	YES	YES
249	a	1065.98	1.58039	YES	YES
250	a	1067.06	1.06840	YES	YES
251	a	1068.38	0.87421	YES	YES
252	a	1070.41	0.63163	YES	YES
253	a	1071.01	0.11061	YES	YES
254	a	1071.75	0.06106	YES	YES
255	a	1073.20	0.63757	YES	YES
256	a	1073.63	0.06002	YES	YES
257	a	1073.86	0.03613	YES	YES
258	a	1073.88	0.72571	YES	YES
259	a	1074.59	0.04226	YES	YES
260	a	1075.42	2.27087	YES	YES

261	a	1079.10	6.22385	YES	YES
262	a	1079.46	0.75190	YES	YES
263	a	1089.18	9.38482	YES	YES
264	a	1092.66	3.04448	YES	YES
265	a	1097.90	1.12980	YES	YES
266	a	1099.37	6.82218	YES	YES
267	a	1100.38	2.61568	YES	YES
268	a	1107.12	2.84949	YES	YES
269	a	1108.93	8.15849	YES	YES
270	a	1109.19	3.32547	YES	YES
271	a	1114.35	7.01507	YES	YES
272	a	1126.90	3.14806	YES	YES
273	a	1133.80	1.19387	YES	YES
274	a	1150.11	1.19260	YES	YES
275	a	1153.01	0.48724	YES	YES
276	a	1159.92	4.31291	YES	YES
277	a	1160.43	2.43911	YES	YES
278	a	1162.53	10.56694	YES	YES
279	a	1162.97	6.51686	YES	YES
280	a	1164.19	9.76598	YES	YES
281	a	1167.69	3.07932	YES	YES
282	a	1170.31	3.37079	YES	YES
283	a	1171.18	7.25787	YES	YES
284	a	1171.66	10.46504	YES	YES
285	a	1176.58	14.24670	YES	YES
286	a	1178.48	4.74227	YES	YES
287	a	1184.34	9.17017	YES	YES
288	a	1191.37	4.32917	YES	YES
289	a	1192.13	2.48848	YES	YES
290	a	1195.57	3.29714	YES	YES
291	a	1201.94	3.32066	YES	YES
292	a	1202.79	2.51440	YES	YES
293	a	1207.58	2.11912	YES	YES
294	a	1209.84	37.41837	YES	YES
295	a	1239.34	0.95555	YES	YES
296	a	1243.99	0.55303	YES	YES
297	a	1244.51	1.38582	YES	YES
298	a	1246.30	0.08666	YES	YES
299	a	1247.64	1.04935	YES	YES
300	a	1248.18	1.87396	YES	YES
301	a	1248.90	0.44627	YES	YES
302	a	1250.60	0.76833	YES	YES
303	a	1250.94	0.73658	YES	YES
304	a	1253.24	3.75362	YES	YES
305	a	1254.17	1.75052	YES	YES
306	a	1255.07	0.11625	YES	YES
307	a	1255.83	4.29209	YES	YES
308	a	1256.41	1.95883	YES	YES
309	a	1257.59	0.13769	YES	YES
310	a	1258.37	5.27064	YES	YES
311	a	1258.64	3.31423	YES	YES
312	a	1259.68	1.80209	YES	YES
313	a	1259.86	5.96601	YES	YES
314	a	1260.59	2.43288	YES	YES
315	a	1262.12	5.08380	YES	YES
316	a	1264.38	1.84173	YES	YES
317	a	1265.00	0.19545	YES	YES
318	a	1266.20	0.56598	YES	YES
319	a	1266.64	4.79522	YES	YES
320	a	1268.54	0.46349	YES	YES
321	a	1269.37	1.20968	YES	YES
322	a	1274.58	1.85309	YES	YES

323	a	1281.33	5.91174	YES	YES
324	a	1281.75	56.44966	YES	YES
325	a	1283.35	3.42999	YES	YES
326	a	1285.64	0.91997	YES	YES
327	a	1286.26	3.86735	YES	YES
328	a	1287.30	6.22202	YES	YES
329	a	1289.84	3.69848	YES	YES
330	a	1293.80	9.85486	YES	YES
331	a	1295.22	4.60937	YES	YES
332	a	1302.69	113.84761	YES	YES
333	a	1308.19	6.46309	YES	YES
334	a	1309.62	7.55478	YES	YES
335	a	1309.91	3.63870	YES	YES
336	a	1310.39	1.51507	YES	YES
337	a	1310.81	6.53749	YES	YES
338	a	1312.40	3.68327	YES	YES
339	a	1314.08	0.82730	YES	YES
340	a	1315.10	5.41611	YES	YES
341	a	1318.11	2.30606	YES	YES
342	a	1322.26	3.54317	YES	YES
343	a	1322.94	1.35047	YES	YES
344	a	1322.99	1.68009	YES	YES
345	a	1323.34	0.45595	YES	YES
346	a	1323.51	0.73426	YES	YES
347	a	1323.62	1.49030	YES	YES
348	a	1324.15	0.40008	YES	YES
349	a	1324.58	2.23100	YES	YES
350	a	1324.93	0.31194	YES	YES
351	a	1325.81	0.44718	YES	YES
352	a	1326.38	2.94860	YES	YES
353	a	1326.43	2.40137	YES	YES
354	a	1327.36	4.05385	YES	YES
355	a	1328.03	4.93612	YES	YES
356	a	1331.17	0.91601	YES	YES
357	a	1331.98	1.17267	YES	YES
358	a	1334.21	5.81572	YES	YES
359	a	1335.93	0.47450	YES	YES
360	a	1336.46	0.42702	YES	YES
361	a	1337.04	0.35963	YES	YES
362	a	1337.65	0.15104	YES	YES
363	a	1337.77	0.07692	YES	YES
364	a	1337.91	0.66348	YES	YES
365	a	1338.62	0.89621	YES	YES
366	a	1339.04	0.80442	YES	YES
367	a	1342.01	5.99690	YES	YES
368	a	1344.75	4.00328	YES	YES
369	a	1345.12	2.56132	YES	YES
370	a	1345.87	1.95035	YES	YES
371	a	1346.47	1.11228	YES	YES
372	a	1348.06	2.61869	YES	YES
373	a	1349.36	0.60113	YES	YES
374	a	1349.94	0.27561	YES	YES
375	a	1349.98	0.61111	YES	YES
376	a	1404.34	5.23255	YES	YES
377	a	1404.40	8.65664	YES	YES
378	a	1409.81	8.37946	YES	YES
379	a	1409.95	6.35378	YES	YES
380	a	1420.50	2.00391	YES	YES
381	a	1423.78	10.67542	YES	YES
382	a	1425.10	12.96768	YES	YES
383	a	1426.28	4.00902	YES	YES
384	a	1427.70	2.36717	YES	YES

385	a	1431.00	12.97254	YES	YES
386	a	1432.94	1.31013	YES	YES
387	a	1435.24	2.59409	YES	YES
388	a	1435.38	0.76094	YES	YES
389	a	1435.81	6.55177	YES	YES
390	a	1436.10	4.44686	YES	YES
391	a	1436.71	0.47175	YES	YES
392	a	1436.92	2.87415	YES	YES
393	a	1437.31	4.31503	YES	YES
394	a	1437.64	5.37653	YES	YES
395	a	1438.18	2.16975	YES	YES
396	a	1438.31	7.21541	YES	YES
397	a	1438.68	8.85155	YES	YES
398	a	1438.93	1.14098	YES	YES
399	a	1439.20	1.88027	YES	YES
400	a	1439.38	3.22454	YES	YES
401	a	1439.89	5.17657	YES	YES
402	a	1440.04	9.45738	YES	YES
403	a	1440.05	1.33184	YES	YES
404	a	1440.48	12.05818	YES	YES
405	a	1441.86	1.80982	YES	YES
406	a	1442.73	9.33267	YES	YES
407	a	1443.64	12.76936	YES	YES
408	a	1444.27	7.55801	YES	YES
409	a	1444.38	2.13652	YES	YES
410	a	1445.67	15.37502	YES	YES
411	a	1445.88	4.34070	YES	YES
412	a	1446.34	26.09205	YES	YES
413	a	1447.86	5.72846	YES	YES
414	a	1451.91	0.42222	YES	YES
415	a	1453.08	0.06975	YES	YES
416	a	1453.61	2.42404	YES	YES
417	a	1454.10	3.36847	YES	YES
418	a	1455.66	6.60104	YES	YES
419	a	1456.35	0.69311	YES	YES
420	a	1456.78	2.36145	YES	YES
421	a	1457.16	2.15632	YES	YES
422	a	1478.82	185.98085	YES	YES
423	a	1481.43	31.79222	YES	YES
424	a	1559.15	4.26580	YES	YES
425	a	1570.52	4.73107	YES	YES
426	a	1584.86	79.86673	YES	YES
427	a	1597.27	43.45019	YES	YES
428	a	1850.75	141.75479	YES	YES
429	a	2929.69	4.84606	YES	YES
430	a	2934.03	13.77454	YES	YES
431	a	2935.61	4.32757	YES	YES
432	a	2936.62	1.90965	YES	YES
433	a	2937.04	55.22016	YES	YES
434	a	2938.01	7.29435	YES	YES
435	a	2939.10	41.92789	YES	YES
436	a	2939.23	5.66887	YES	YES
437	a	2940.01	18.19973	YES	YES
438	a	2940.12	16.40863	YES	YES
439	a	2940.54	1.81536	YES	YES
440	a	2942.43	3.85946	YES	YES
441	a	2942.98	8.58667	YES	YES
442	a	2944.04	13.18943	YES	YES
443	a	2944.16	12.47078	YES	YES
444	a	2944.85	15.86369	YES	YES
445	a	2945.39	15.57322	YES	YES
446	a	2945.57	16.77727	YES	YES

447	a	2945.98	3.03449	YES	YES
448	a	2946.60	10.85436	YES	YES
449	a	2947.05	4.29063	YES	YES
450	a	2947.35	38.27637	YES	YES
451	a	2948.21	5.73999	YES	YES
452	a	2950.38	17.35534	YES	YES
453	a	2952.86	11.48470	YES	YES
454	a	2953.97	11.32050	YES	YES
455	a	2955.96	2.62553	YES	YES
456	a	2956.85	12.10648	YES	YES
457	a	2956.97	13.09841	YES	YES
458	a	2957.47	14.12160	YES	YES
459	a	2957.50	21.76256	YES	YES
460	a	2958.24	12.89851	YES	YES
461	a	2958.69	30.88269	YES	YES
462	a	2958.88	3.99888	YES	YES
463	a	2960.01	32.87376	YES	YES
464	a	2960.79	31.49216	YES	YES
465	a	2961.13	8.02140	YES	YES
466	a	2962.47	31.21799	YES	YES
467	a	2962.61	38.44517	YES	YES
468	a	2964.45	12.93454	YES	YES
469	a	2966.00	26.73634	YES	YES
470	a	2966.21	22.92360	YES	YES
471	a	2969.47	17.69395	YES	YES
472	a	2969.92	3.85840	YES	YES
473	a	2972.11	17.14425	YES	YES
474	a	2974.19	7.72154	YES	YES
475	a	2976.24	13.94704	YES	YES
476	a	2982.08	37.09502	YES	YES
477	a	2987.15	3.27728	YES	YES
478	a	2987.90	5.62591	YES	YES
479	a	2988.16	1.26924	YES	YES
480	a	2991.84	8.89294	YES	YES
481	a	2993.95	2.16439	YES	YES
482	a	2994.01	4.02503	YES	YES
483	a	2994.25	24.64261	YES	YES
484	a	2994.53	54.55326	YES	YES
485	a	3000.64	18.66530	YES	YES
486	a	3004.88	28.45634	YES	YES
487	a	3005.12	9.91812	YES	YES
488	a	3005.19	32.50134	YES	YES
489	a	3006.76	7.57041	YES	YES
490	a	3008.20	25.22859	YES	YES
491	a	3009.16	19.11384	YES	YES
492	a	3009.35	23.71512	YES	YES
493	a	3010.06	31.73251	YES	YES
494	a	3010.39	26.10916	YES	YES
495	a	3010.99	10.41388	YES	YES
496	a	3011.94	7.86630	YES	YES
497	a	3011.96	23.17570	YES	YES
498	a	3012.90	22.84511	YES	YES
499	a	3013.16	21.39481	YES	YES
500	a	3013.52	23.43276	YES	YES
501	a	3014.39	29.86588	YES	YES
502	a	3014.62	29.01656	YES	YES
503	a	3015.56	24.13635	YES	YES
504	a	3015.74	39.42287	YES	YES
505	a	3016.15	25.51639	YES	YES
506	a	3016.70	28.61573	YES	YES
507	a	3016.76	9.93661	YES	YES
508	a	3016.93	23.35626	YES	YES

509	a	3017.35	32.94431	YES	YES
510	a	3017.55	18.92042	YES	YES
511	a	3017.74	1.17341	YES	YES
512	a	3017.95	7.51506	YES	YES
513	a	3017.97	28.01447	YES	YES
514	a	3019.00	36.48330	YES	YES
515	a	3019.23	22.59793	YES	YES
516	a	3021.26	16.52274	YES	YES
517	a	3021.85	4.44321	YES	YES
518	a	3025.86	23.52711	YES	YES
519	a	3028.38	20.89357	YES	YES
520	a	3028.67	27.53393	YES	YES
521	a	3032.78	1.06262	YES	YES
522	a	3035.71	0.42635	YES	YES
523	a	3045.43	0.73404	YES	YES
524	a	3049.07	1.49088	YES	YES
525	a	3080.64	25.11114	YES	YES
526	a	3095.56	9.49720	YES	YES
527	a	3106.73	4.62486	YES	YES
528	a	3109.54	0.97156	YES	YES
529	a	3112.00	5.13029	YES	YES
530	a	3114.78	6.89120	YES	YES
531	a	3124.80	4.62221	YES	YES
532	a	3133.24	1.27232	YES	YES
533	a	3136.01	2.86186	YES	YES
534	a	3140.54	1.17225	YES	YES

$end

Double hybrid single point energy = -7767.058128041005 H
COSMO enery + OC correction = -7775.3012445274 H (in *o*DFB)

6.2.15 [H{Ga(dcpe)}(NPh$_2$)]$^+$

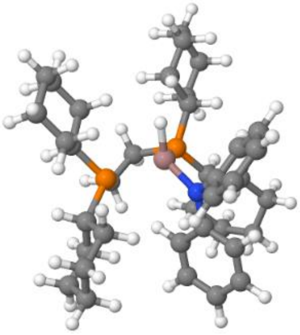

Method: (RI-)BP86(D3BJ)/def2-TZVPP
Symmetry: c1

Cartesian coordinates in Ångström:

Ga	-2.0386955	0.3040672	-0.2318020
P	-3.9287312	-0.7225160	-1.3542358
P	-2.2989890	2.2031226	-1.7299851
C	-3.8757635	-2.5011513	-1.9007240
H	-4.8730374	-2.8822165	-1.6178971
C	-2.8155247	-3.2774499	-1.0931042
H	-1.8212109	-2.8649219	-1.3149448
H	-2.9806459	-3.1405650	-0.0149302
C	-2.8357887	-4.7658206	-1.4559400
H	-2.0486447	-5.2873877	-0.8943187
H	-3.7947870	-5.2106675	-1.1413936
C	-2.6477896	-4.9665880	-2.9625143
H	-2.6800133	-6.0357221	-3.2124330

H	-1.6498424	-4.6014164	-3.2554669
C	-3.7189875	-4.2091236	-3.7527256
H	-4.7117489	-4.6298061	-3.5222767
H	-3.5680238	-4.3364376	-4.8337327
C	-3.7165785	-2.7110636	-3.4158713
H	-4.5252473	-2.2041696	-3.9612920
H	-2.7675156	-2.2753174	-3.7593630
C	-5.4397886	-0.6360091	-0.2894434
H	-6.2555639	-1.0550048	-0.9061847
C	-5.2366071	-1.5329154	0.9482937
H	-5.0324453	-2.5685305	0.6447015
H	-4.3502941	-1.1800263	1.5010791
C	-6.4601009	-1.4952258	1.8721632
H	-7.3225666	-1.9434636	1.3520040
H	-6.2663384	-2.1202387	2.7545838
C	-6.8021963	-0.0626461	2.2876579
H	-7.6946858	-0.0530779	2.9277769
H	-5.9766723	0.3499134	2.8910536
C	-7.0237583	0.8198193	1.0572064
H	-7.2340535	1.8568679	1.3542093
H	-7.9101572	0.4652368	0.5060164
C	-5.8082889	0.7972831	0.1211160
H	-6.0241500	1.4057928	-0.7678604
H	-4.9474339	1.2549134	0.6335003
C	-4.1938516	0.3649057	-2.8312607
H	-3.5230111	-0.0193470	-3.6117228
H	-5.2198315	0.2368053	-3.2045796
C	-3.9049849	1.8591109	-2.5837666
H	-4.6807922	2.3012139	-1.9465791
H	-3.9305221	2.4029260	-3.5375787
C	-0.9869850	2.5502940	-2.9844101
H	-1.1325723	3.6095698	-3.2576356
C	0.3928371	2.4035271	-2.3132192
H	0.5091810	1.3742237	-1.9470926
H	0.4624836	3.0652133	-1.4361174
C	1.5200497	2.7133350	-3.3036583
H	1.4761405	3.7761774	-3.5946836
H	2.4883626	2.5603737	-2.8079063
C	1.4073746	1.8321312	-4.5505076
H	1.5346775	0.7762744	-4.2623144
H	2.2142829	2.0685618	-5.2575037
C	0.0457164	2.0154351	-5.2284538
H	-0.0389664	1.3630992	-6.1088480
H	-0.0466089	3.0517338	-5.5940809
C	-1.1094176	1.7117428	-4.2642978
H	-1.0768803	0.6458806	-4.0056401
H	-2.0719034	1.9084401	-4.7595326
C	-2.5288193	3.8134065	-0.8451637
H	-1.5013188	4.0760891	-0.5353322
C	-3.3921590	3.6841891	0.4194924
H	-3.0007866	2.8938539	1.0738523
H	-4.4132502	3.3859892	0.1302399
C	-3.4598093	5.0197270	1.1715085
H	-4.1004798	4.9096430	2.0573943
H	-2.4531218	5.2729056	1.5424453
C	-3.9728515	6.1466797	0.2705924
H	-5.0218195	5.9447247	-0.0042393
H	-3.9718605	7.1005773	0.8152918
C	-3.1307405	6.2637486	-1.0031780
H	-2.1069748	6.5773065	-0.7413048
H	-3.5365605	7.0404037	-1.6660641
C	-3.0665040	4.9292881	-1.7598542

```
H    -4.0796740    4.6602206   -2.1005705
H    -2.4461173    5.0403605   -2.6598188
N    -0.3448486   -0.5413770   -0.3385837
H     2.1564171   -0.7968461    4.3522880
C     1.6537959   -0.7441819    3.3874702
C     1.3206804    0.4940777    2.8361823
H     1.5672207    1.4132596    3.3670533
C     0.6838223    0.5599606    1.5947014
H     0.4429499    1.5251960    1.1465390
C     0.3569023   -0.6126113    0.9015738
C     0.6893686   -1.8554168    1.4625941
H     0.4322325   -2.7664035    0.9221902
C     1.3400042   -1.9178861    2.6936190
H     1.5938317   -2.8879088    3.1205616
H    -2.4625404    0.6628826    1.2253118
H     2.2506615   -1.2104200   -0.6743726
C     1.6263655   -1.3684482   -1.5513969
C     0.2480018   -1.0593251   -1.4870606
C    -0.5107297   -1.2568690   -2.6561122
H    -1.5723013   -1.0112680   -2.6342938
C     0.0564493   -1.7585350   -3.8255570
H    -0.5680385   -1.9019800   -4.7085291
C     1.4148470   -2.0730106   -3.8718030
H     1.8634294   -2.4676715   -4.7814942
C     2.1888935   -1.8653702   -2.7240456
H     3.2552304   -2.0905455   -2.7421403

SCF energy GEOOPT =  -4147.035923940 H
ZPE =   2297. kJ/mol
FREEH energy =  2413.54 kJ/mol
FREEH entropy =   1.10070 kJ/mol/K

$vibrational spectrum
#  mode     symmetry     wave number   IR intensity    selection rules
#                         cm**(-1)        km/mol          IR     RAMAN
     1                       -0.00         0.00000         -       -
     2                       -0.00         0.00000         -       -
     3                        0.00         0.00000         -       -
     4                        0.00         0.00000         -       -
     5                        0.00         0.00000         -       -
     6                        0.00         0.00000         -       -
     7         a              8.47         0.03686        YES     YES
     8         a             16.66         0.01783        YES     YES
     9         a             27.07         0.52404        YES     YES
    10         a             28.33         0.03075        YES     YES
    11         a             33.97         0.04953        YES     YES
    12         a             41.17         0.02811        YES     YES
    13         a             43.41         0.05671        YES     YES
    14         a             46.07         0.03959        YES     YES
    15         a             47.72         0.18347        YES     YES
    16         a             49.23         0.08881        YES     YES
    17         a             53.19         0.07482        YES     YES
    18         a             65.69         0.48457        YES     YES
    19         a             68.63         0.08934        YES     YES
    20         a             77.96         0.54508        YES     YES
    21         a             79.30         0.75044        YES     YES
    22         a             84.45         0.12926        YES     YES
    23         a             91.90         0.12313        YES     YES
    24         a             99.36         0.24876        YES     YES
    25         a            120.96         2.70227        YES     YES
    26         a            124.99         1.66750        YES     YES
    27         a            137.60         1.05970        YES     YES
```

28	a	141.32	2.21633	YES	YES
29	a	143.29	0.37779	YES	YES
30	a	160.88	0.06179	YES	YES
31	a	166.97	1.02356	YES	YES
32	a	174.75	3.62899	YES	YES
33	a	181.62	1.63936	YES	YES
34	a	200.55	0.91465	YES	YES
35	a	211.07	0.00670	YES	YES
36	a	214.25	0.08731	YES	YES
37	a	223.04	0.27073	YES	YES
38	a	227.92	0.04379	YES	YES
39	a	232.88	0.07506	YES	YES
40	a	235.45	1.14007	YES	YES
41	a	239.23	0.40246	YES	YES
42	a	242.22	0.24653	YES	YES
43	a	247.68	3.85012	YES	YES
44	a	278.62	8.77044	YES	YES
45	a	288.80	1.02179	YES	YES
46	a	294.21	1.11905	YES	YES
47	a	301.48	1.75720	YES	YES
48	a	308.37	0.24093	YES	YES
49	a	324.95	5.80699	YES	YES
50	a	329.67	5.50246	YES	YES
51	a	333.42	2.91632	YES	YES
52	a	343.18	0.94415	YES	YES
53	a	359.41	0.76184	YES	YES
54	a	378.44	6.85410	YES	YES
55	a	386.67	0.45525	YES	YES
56	a	405.55	6.33301	YES	YES
57	a	405.91	0.83007	YES	YES
58	a	410.75	0.76391	YES	YES
59	a	414.24	2.30792	YES	YES
60	a	422.63	2.26787	YES	YES
61	a	428.34	3.85987	YES	YES
62	a	428.67	1.51777	YES	YES
63	a	429.05	1.33140	YES	YES
64	a	430.67	2.10717	YES	YES
65	a	437.49	0.70813	YES	YES
66	a	439.42	2.42348	YES	YES
67	a	461.81	6.88193	YES	YES
68	a	488.70	1.03109	YES	YES
69	a	492.53	4.91458	YES	YES
70	a	497.68	27.12255	YES	YES
71	a	499.10	4.13363	YES	YES
72	a	507.73	11.22555	YES	YES
73	a	510.68	16.83546	YES	YES
74	a	547.87	80.47246	YES	YES
75	a	569.72	23.46812	YES	YES
76	a	607.39	67.59195	YES	YES
77	a	614.83	0.03805	YES	YES
78	a	618.50	4.31164	YES	YES
79	a	626.99	15.29901	YES	YES
80	a	650.11	5.62053	YES	YES
81	a	668.55	1.03782	YES	YES
82	a	691.97	42.74104	YES	YES
83	a	694.65	23.00549	YES	YES
84	a	712.90	0.88099	YES	YES
85	a	713.29	0.50554	YES	YES
86	a	732.45	0.98324	YES	YES
87	a	736.82	3.93426	YES	YES
88	a	739.61	46.83566	YES	YES
89	a	751.94	11.66010	YES	YES

90	a	768.78	0.30614	YES	YES
91	a	771.07	0.24944	YES	YES
92	a	774.53	0.20515	YES	YES
93	a	777.33	0.11969	YES	YES
94	a	785.03	10.16167	YES	YES
95	a	799.04	1.89675	YES	YES
96	a	807.83	1.37949	YES	YES
97	a	809.76	2.33183	YES	YES
98	a	811.04	0.33828	YES	YES
99	a	813.88	5.69412	YES	YES
100	a	816.39	0.81247	YES	YES
101	a	834.84	0.39240	YES	YES
102	a	836.53	1.52393	YES	YES
103	a	837.02	4.37006	YES	YES
104	a	838.78	10.38829	YES	YES
105	a	849.24	7.79070	YES	YES
106	a	857.16	2.95561	YES	YES
107	a	871.78	11.55998	YES	YES
108	a	872.93	10.10646	YES	YES
109	a	874.83	0.91420	YES	YES
110	a	876.91	3.02549	YES	YES
111	a	878.73	4.05154	YES	YES
112	a	880.00	4.34236	YES	YES
113	a	880.62	1.96333	YES	YES
114	a	882.20	3.48837	YES	YES
115	a	883.04	5.83982	YES	YES
116	a	887.01	3.52750	YES	YES
117	a	905.25	1.60957	YES	YES
118	a	905.89	1.84922	YES	YES
119	a	908.50	0.20599	YES	YES
120	a	910.37	0.32425	YES	YES
121	a	916.82	21.86346	YES	YES
122	a	935.95	0.14691	YES	YES
123	a	947.73	0.00964	YES	YES
124	a	965.00	0.36453	YES	YES
125	a	971.76	0.04061	YES	YES
126	a	979.98	0.88908	YES	YES
127	a	987.39	2.13653	YES	YES
128	a	988.92	8.68647	YES	YES
129	a	989.34	17.09849	YES	YES
130	a	990.42	3.91198	YES	YES
131	a	994.62	3.14191	YES	YES
132	a	996.42	0.27486	YES	YES
133	a	1017.52	0.76662	YES	YES
134	a	1018.93	0.19186	YES	YES
135	a	1019.32	0.59686	YES	YES
136	a	1021.46	0.21862	YES	YES
137	a	1023.59	4.28036	YES	YES
138	a	1028.75	5.11142	YES	YES
139	a	1031.62	1.56921	YES	YES
140	a	1032.74	0.76184	YES	YES
141	a	1035.03	1.05702	YES	YES
142	a	1038.63	0.48631	YES	YES
143	a	1061.95	1.18303	YES	YES
144	a	1063.34	0.34389	YES	YES
145	a	1064.92	0.06681	YES	YES
146	a	1067.40	0.39977	YES	YES
147	a	1070.95	0.41619	YES	YES
148	a	1071.20	0.00731	YES	YES
149	a	1071.98	7.15160	YES	YES
150	a	1073.27	0.12551	YES	YES
151	a	1073.91	0.07500	YES	YES

152	a	1081.34	8.75272	YES	YES
153	a	1085.57	3.63613	YES	YES
154	a	1092.85	1.22788	YES	YES
155	a	1097.99	7.95054	YES	YES
156	a	1106.07	2.01665	YES	YES
157	a	1107.91	3.94634	YES	YES
158	a	1127.29	2.30044	YES	YES
159	a	1146.91	1.71826	YES	YES
160	a	1151.72	0.08446	YES	YES
161	a	1158.96	4.08726	YES	YES
162	a	1162.27	5.16237	YES	YES
163	a	1165.19	2.13914	YES	YES
164	a	1167.95	7.81724	YES	YES
165	a	1168.33	7.21252	YES	YES
166	a	1169.23	5.22173	YES	YES
167	a	1178.74	10.21795	YES	YES
168	a	1186.42	11.55140	YES	YES
169	a	1192.65	3.97687	YES	YES
170	a	1194.87	11.16162	YES	YES
171	a	1207.32	45.15775	YES	YES
172	a	1244.30	1.93097	YES	YES
173	a	1246.58	0.34239	YES	YES
174	a	1248.87	0.49019	YES	YES
175	a	1250.45	2.68488	YES	YES
176	a	1251.27	1.62489	YES	YES
177	a	1254.66	3.38331	YES	YES
178	a	1257.27	2.83753	YES	YES
179	a	1258.17	9.39902	YES	YES
180	a	1259.83	1.56996	YES	YES
181	a	1260.26	6.22012	YES	YES
182	a	1263.27	2.16734	YES	YES
183	a	1265.83	2.95733	YES	YES
184	a	1270.69	2.06966	YES	YES
185	a	1272.03	0.09714	YES	YES
186	a	1284.78	57.17092	YES	YES
187	a	1286.17	2.99301	YES	YES
188	a	1287.94	1.85112	YES	YES
189	a	1289.22	3.16593	YES	YES
190	a	1290.90	1.15858	YES	YES
191	a	1302.79	70.48134	YES	YES
192	a	1304.04	68.54780	YES	YES
193	a	1311.65	1.69433	YES	YES
194	a	1316.43	0.65913	YES	YES
195	a	1318.10	2.18516	YES	YES
196	a	1318.34	1.25582	YES	YES
197	a	1323.42	0.60428	YES	YES
198	a	1323.87	0.33164	YES	YES
199	a	1324.13	1.15653	YES	YES
200	a	1325.00	0.16565	YES	YES
201	a	1327.23	6.14073	YES	YES
202	a	1329.77	4.24311	YES	YES
203	a	1330.56	2.98972	YES	YES
204	a	1333.70	1.53451	YES	YES
205	a	1334.57	1.78461	YES	YES
206	a	1337.75	0.02159	YES	YES
207	a	1338.79	0.09869	YES	YES
208	a	1339.75	0.26408	YES	YES
209	a	1340.59	0.67809	YES	YES
210	a	1344.94	1.26412	YES	YES
211	a	1345.43	5.27586	YES	YES
212	a	1346.68	1.52673	YES	YES
213	a	1348.03	1.04929	YES	YES

214	a	1350.27	0.36689	YES	YES
215	a	1402.25	10.64019	YES	YES
216	a	1405.06	7.82134	YES	YES
217	a	1429.94	3.10376	YES	YES
218	a	1431.38	1.11258	YES	YES
219	a	1432.88	2.64635	YES	YES
220	a	1435.94	3.67515	YES	YES
221	a	1436.13	3.95857	YES	YES
222	a	1436.74	5.15367	YES	YES
223	a	1437.59	1.04811	YES	YES
224	a	1438.15	7.22300	YES	YES
225	a	1438.49	7.30288	YES	YES
226	a	1438.87	3.21257	YES	YES
227	a	1440.17	3.52690	YES	YES
228	a	1440.52	3.67395	YES	YES
229	a	1441.02	8.71665	YES	YES
230	a	1443.16	12.25194	YES	YES
231	a	1444.27	28.81546	YES	YES
232	a	1445.29	6.15605	YES	YES
233	a	1446.49	5.87304	YES	YES
234	a	1447.88	1.61688	YES	YES
235	a	1453.96	1.29087	YES	YES
236	a	1454.88	3.62062	YES	YES
237	a	1455.83	3.83879	YES	YES
238	a	1457.38	0.87483	YES	YES
239	a	1478.53	120.75911	YES	YES
240	a	1481.14	76.31145	YES	YES
241	a	1562.05	6.33230	YES	YES
242	a	1576.78	6.19735	YES	YES
243	a	1586.81	67.89705	YES	YES
244	a	1598.84	54.40354	YES	YES
245	a	1945.97	85.30339	YES	YES
246	a	2924.52	4.91929	YES	YES
247	a	2930.06	9.30618	YES	YES
248	a	2931.74	1.95246	YES	YES
249	a	2940.18	9.96719	YES	YES
250	a	2942.63	11.27738	YES	YES
251	a	2943.50	23.66285	YES	YES
252	a	2944.76	48.75525	YES	YES
253	a	2944.88	3.73426	YES	YES
254	a	2945.81	9.60808	YES	YES
255	a	2946.55	17.70666	YES	YES
256	a	2947.97	12.60091	YES	YES
257	a	2948.24	11.71112	YES	YES
258	a	2953.51	1.24335	YES	YES
259	a	2954.88	4.78266	YES	YES
260	a	2956.28	1.30665	YES	YES
261	a	2958.59	3.93398	YES	YES
262	a	2959.00	19.43977	YES	YES
263	a	2960.08	91.64648	YES	YES
264	a	2961.41	15.77413	YES	YES
265	a	2962.23	24.17542	YES	YES
266	a	2964.11	16.41615	YES	YES
267	a	2967.61	30.79144	YES	YES
268	a	2971.21	18.55152	YES	YES
269	a	2975.97	10.76359	YES	YES
270	a	2980.78	2.53633	YES	YES
271	a	2992.65	4.98245	YES	YES
272	a	3003.96	24.02910	YES	YES
273	a	3004.89	36.35496	YES	YES
274	a	3005.87	31.72155	YES	YES
275	a	3006.32	28.18822	YES	YES

276	a	3008.59	14.55472	YES	YES
277	a	3008.67	7.80635	YES	YES
278	a	3009.25	16.48764	YES	YES
279	a	3010.25	41.76204	YES	YES
280	a	3010.57	22.13824	YES	YES
281	a	3011.39	26.66006	YES	YES
282	a	3012.27	35.17953	YES	YES
283	a	3012.86	17.62738	YES	YES
284	a	3013.12	24.81900	YES	YES
285	a	3013.90	27.91701	YES	YES
286	a	3015.13	50.98714	YES	YES
287	a	3015.71	1.48280	YES	YES
288	a	3017.66	11.24042	YES	YES
289	a	3021.59	25.49408	YES	YES
290	a	3024.62	2.67542	YES	YES
291	a	3027.60	1.18437	YES	YES
292	a	3033.19	5.09482	YES	YES
293	a	3044.53	0.80465	YES	YES
294	a	3085.39	20.12450	YES	YES
295	a	3090.78	16.46139	YES	YES
296	a	3100.13	27.06604	YES	YES
297	a	3103.75	2.04303	YES	YES
298	a	3108.63	7.23877	YES	YES
299	a	3112.93	4.93438	YES	YES
300	a	3119.44	13.18970	YES	YES
301	a	3131.02	1.56081	YES	YES
302	a	3131.34	11.66769	YES	YES
303	a	3135.87	3.67807	YES	YES

$end

Double hybrid single point energy = -4142.522672211521 H
COSMO enery + OC correction = -4147.0879874357 H (in *o*DFB)

6.2.16 H_2NPh

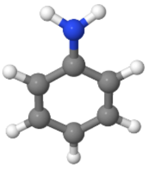

Method: (RI-)BP86(D3BJ)/def2-TZVPP
Symmetry: c1

Cartesian coordinates in Ångström:

H	1.3133090	-0.0242037	2.0620512
C	0.7657024	0.0113832	1.1184513
C	-0.6357611	0.1335595	1.1366725
N	-1.3342593	0.1509879	2.3460866
C	-1.3231472	0.1721490	-0.0902798
H	-2.4109544	0.2624532	-0.0929077
C	-0.6261103	0.0909604	-1.2943070
H	-1.1798836	0.1248201	-2.2329902
C	0.7662424	-0.0304241	-1.3080256
H	1.3071670	-0.0924302	-2.2511231
C	1.4529476	-0.0690118	-0.0912804
H	2.5393659	-0.1613606	-0.0808145
H	-0.8006918	0.4680206	3.1490065
H	-2.2533766	0.5795064	2.3084402

SCF energy GEOOPT = -287.7508036819 H

ZPE = 298.9 kJ/mol
FREEH energy = 314.54 kJ/mol
FREEH entropy = 0.31853 kJ/mol/K

```
$vibrational spectrum
#  mode     symmetry     wave number   IR intensity    selection rules
#                         cm**(-1)        km/mol         IR     RAMAN
     1                     -0.00         0.00000         -        -
     2                     -0.00         0.00000         -        -
     3                     -0.00         0.00000         -        -
     4                     -0.00         0.00000         -        -
     5                      0.00         0.00000         -        -
     6                      0.00         0.00000         -        -
     7         a          211.51         4.96821        YES      YES
     8         a          306.11        17.15264        YES      YES
     9         a          371.34         0.00787        YES      YES
    10         a          402.40         0.19282        YES      YES
    11         a          487.72        74.28704        YES      YES
    12         a          521.57         9.07873        YES      YES
    13         a          568.60       237.47743        YES      YES
    14         a          617.35         0.32415        YES      YES
    15         a          682.82        31.08616        YES      YES
    16         a          737.71        54.80911        YES      YES
    17         a          795.91         0.06249        YES      YES
    18         a          815.35         2.44832        YES      YES
    19         a          852.69         7.90059        YES      YES
    20         a          926.13         0.00049        YES      YES
    21         a          941.52         0.04377        YES      YES
    22         a          988.27         1.38143        YES      YES
    23         a         1024.43         3.42000        YES      YES
    24         a         1036.98         3.65303        YES      YES
    25         a         1106.00         3.59680        YES      YES
    26         a         1146.73         1.28524        YES      YES
    27         a         1166.39         8.92545        YES      YES
    28         a         1278.28        53.50489        YES      YES
    29         a         1324.18         0.01238        YES      YES
    30         a         1351.06         7.18185        YES      YES
    31         a         1460.65         1.19007        YES      YES
    32         a         1490.58        57.15474        YES      YES
    33         a         1582.05         4.23351        YES      YES
    34         a         1597.43         5.02515        YES      YES
    35         a         1612.36       146.50477        YES      YES
    36         a         3085.38        18.81532        YES      YES
    37         a         3086.32         5.11989        YES      YES
    38         a         3103.39         2.44230        YES      YES
    39         a         3108.63        33.67040        YES      YES
    40         a         3127.10        13.04485        YES      YES
    41         a         3479.48        12.26652        YES      YES
    42         a         3578.56        11.70990        YES      YES
$end
```

Double hybrid single point energy = -287.221982147440 H
COSMO enery + OC correction = -287.7602991828 H (in *o*DFB)

6.2.17 [H{Ga(dcpe)}$_2$(HNPh)]$^{2+}$

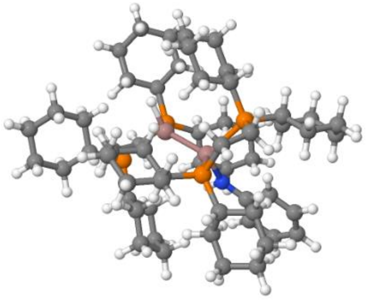

Method: (RI-)BP86(D3BJ)/def2-TZVPP
Symmetry: c1

Cartesian coordinates in Ångström:

Ga	-0.3577689	0.2200689	-1.7938063
P	-2.2846772	-0.8764139	-2.8843464
P	-1.4422902	2.3512686	-2.4680828
C	-2.1382314	-2.2197539	-4.1750213
H	-2.9501106	-2.9155689	-3.8974830
C	-0.8013214	-2.9681117	-4.0446411
H	0.0278603	-2.2691839	-4.2263625
H	-0.6851664	-3.3374578	-3.0175710
C	-0.7168948	-4.1279273	-5.0430651
H	0.2654966	-4.6129214	-4.9576215
H	-1.4688744	-4.8924026	-4.7859644
C	-0.9485268	-3.6438731	-6.4761932
H	-0.9024127	-4.4886739	-7.1760230
H	-0.1404178	-2.9506298	-6.7610463
C	-2.2988998	-2.9342166	-6.5946026
H	-3.1106034	-3.6506963	-6.3877299
H	-2.4596597	-2.5677726	-7.6176863
C	-2.4068302	-1.7549059	-5.6173141
H	-3.4047180	-1.3029630	-5.6970167
H	-1.6760540	-0.9867963	-5.9102559
C	-3.4121638	-1.6162947	-1.6170186
H	-4.2835931	-1.9763084	-2.1934003
C	-2.7263204	-2.8249262	-0.9574203
H	-2.4369711	-3.5660659	-1.7149102
H	-1.7929691	-2.4821330	-0.4780837
C	-3.6323534	-3.4748140	0.0935245
H	-4.5205725	-3.8954723	-0.4045600
H	-3.1087506	-4.3225151	0.5558876
C	-4.0708684	-2.4598945	1.1508996
H	-4.7311740	-2.9359786	1.8879925
H	-3.1859843	-2.0981077	1.7017178
C	-4.7768772	-1.2696067	0.4977781
H	-5.0594215	-0.5232476	1.2530532
H	-5.7142719	-1.6124556	0.0310023
C	-3.8982957	-0.6022250	-0.5693763
H	-4.4716791	0.1982820	-1.0554315
H	-3.0262965	-0.1370166	-0.0815991
C	-3.2265467	0.5073565	-3.6673321
H	-2.8055150	0.6315014	-4.6728735
H	-4.2729371	0.1958062	-3.7983148
C	-3.1575651	1.8386311	-2.9103100
H	-3.7145960	1.7847077	-1.9670707

H	-3.6227804	2.6322560	-3.5102079
C	-0.7562479	3.3364339	-3.8801721
H	-1.1660383	4.3488331	-3.7195190
C	0.7732563	3.4078965	-3.7553324
H	1.1889798	2.3944666	-3.8421632
H	1.0481621	3.7856385	-2.7611196
C	1.3754510	4.2983235	-4.8465131
H	1.0457850	5.3397693	-4.6966245
H	2.4703183	4.2969856	-4.7541104
C	0.9517613	3.8180644	-6.2372929
H	1.3699532	2.8147600	-6.4165698
H	1.3670194	4.4781427	-7.0103725
C	-0.5749018	3.7659867	-6.3582742
H	-0.8722481	3.3934838	-7.3481459
H	-0.9863699	4.7849839	-6.2710371
C	-1.2014476	2.8747070	-5.2750504
H	-0.8775464	1.8389769	-5.4414044
H	-2.2973777	2.9025195	-5.3602130
C	-1.6868852	3.5965925	-1.1163811
H	-0.7057292	4.0926750	-1.0249009
C	-2.0218375	2.9309087	0.2259404
H	-1.2348135	2.2101332	0.4970825
H	-2.9525309	2.3482727	0.1308914
C	-2.1948926	3.9739919	1.3359241
H	-2.4706530	3.4701943	2.2724794
H	-1.2260404	4.4716695	1.5149905
C	-3.2318979	5.0375440	0.9681103
H	-4.2264553	4.5682329	0.8907097
H	-3.3028276	5.7887217	1.7659796
C	-2.8771385	5.7027972	-0.3631789
H	-1.9288394	6.2554451	-0.2611694
H	-3.6407623	6.4405386	-0.6435704
C	-2.7407577	4.6611017	-1.4809608
H	-3.7169143	4.1732534	-1.6281785
H	-2.4866844	5.1581446	-2.4267569
Ga	-0.0812018	-0.2558546	0.6094572
P	1.3575473	1.2063474	1.9175657
P	1.4119326	-2.0564789	1.2988553
C	0.5094694	2.3083497	3.1349859
H	-0.0209226	3.0468571	2.5128334
C	-0.5419713	1.5328304	3.9502244
H	-0.0466174	0.7292081	4.5195143
H	-1.2582050	1.0434756	3.2757568
C	-1.2690887	2.4562220	4.9369194
H	-1.9778355	1.8651655	5.5329843
H	-1.8691120	3.1884124	4.3725620
C	-0.2875808	3.2023093	5.8440180
H	-0.8311191	3.8820809	6.5135127
H	0.2365552	2.4799149	6.4914973
C	0.7388765	3.9808545	5.0176558
H	0.2271860	4.7688005	4.4407326
H	1.4594937	4.4897821	5.6719262
C	1.4945112	3.0597122	4.0494948
H	2.2075620	3.6486094	3.4560622
H	2.0833881	2.3332366	4.6321292
C	2.5371414	2.2569364	0.9655522
H	3.1042412	2.8313197	1.7171511
C	1.7727320	3.2382718	0.0645539

H	1.0910927	3.8597285	0.6633716
H	1.1490372	2.6545739	-0.6359715
C	2.7423342	4.1181482	-0.7317641
H	3.3232120	4.7416091	-0.0332188
H	2.1791974	4.8167881	-1.3665488
C	3.6933644	3.2622509	-1.5725259
H	4.3895459	3.9007595	-2.1318969
H	3.1135697	2.6942810	-2.3186575
C	4.4691768	2.2857247	-0.6861250
H	5.1201591	1.6422638	-1.2940787
H	5.1320532	2.8490119	-0.0100409
C	3.5308152	1.4110630	0.1537057
H	4.1262362	0.7738704	0.8204276
H	2.9623755	0.7385710	-0.5101660
C	2.3881530	0.0697827	2.9449752
H	1.7968948	-0.1303356	3.8468712
H	3.2916219	0.6057779	3.2679387
C	2.7768147	-1.2404209	2.2476857
H	3.5782209	-1.0510869	1.5267608
H	3.1716633	-1.9601453	2.9791373
C	0.6361473	-3.2193542	2.5186646
H	1.4462142	-3.9185020	2.7944718
C	-0.5229099	-4.0126932	1.8963366
H	-1.2621274	-3.2970085	1.5037748
H	-0.1675233	-4.6108636	1.0476654
C	-1.2019128	-4.9296438	2.9209872
H	-0.4927405	-5.7128288	3.2338748
H	-2.0436365	-5.4484721	2.4411301
C	-1.6743366	-4.1482844	4.1476898
H	-2.4544473	-3.4288743	3.8475287
H	-2.1357844	-4.8258758	4.8780627
C	-0.5052453	-3.3963949	4.7858339
H	-0.8436709	-2.8095718	5.6508564
H	0.2316460	-4.1211495	5.1680230
C	0.1797665	-2.4622255	3.7798870
H	-0.5215121	-1.6647827	3.4841413
H	1.0390329	-1.9799347	4.2651786
C	2.2249335	-3.0987431	-0.0067798
H	2.5109301	-4.0333574	0.5072300
C	1.2223624	-3.4306037	-1.1277840
H	0.3084539	-3.8796096	-0.7191497
H	0.9281096	-2.4877133	-1.6177414
C	1.8608871	-4.3576267	-2.1663503
H	1.1237150	-4.6159192	-2.9364129
H	2.1491273	-5.3056308	-1.6833743
C	3.0900275	-3.6983752	-2.7922187
H	2.7808552	-2.7913700	-3.3379177
H	3.5542240	-4.3665923	-3.5296388
C	4.1053681	-3.3300575	-1.7085969
H	4.5011261	-4.2515052	-1.2519664
H	4.9671387	-2.8043568	-2.1426045
C	3.4918979	-2.4581035	-0.6036135
H	3.2323492	-1.4707621	-1.0203921
H	4.2492720	-2.2878293	0.1724656
N	1.2975045	-0.0508660	-2.6576718
H	2.0826975	-0.1134294	-2.0159124
C	1.6951012	-0.0516111	-3.9827400
C	0.7475999	-0.0339118	-5.0194856

```
C     3.0602548   -0.0771181   -4.3395501
C     3.4446713   -0.0886995   -5.6781671
C     2.4883507   -0.0753187   -6.7001834
C     1.1352811   -0.0476533   -6.3575385
H    -0.3090093   -0.0132006   -4.7534280
H     3.8172485   -0.0843958   -3.5525892
H     4.5051075   -0.1077600   -5.9284573
H     2.7952073   -0.0853717   -7.7441192
H     0.3752207   -0.0340838   -7.1396044
H    -1.3268391   -0.4590205    1.5540712

SCF energy GEOOPT =  -7543.978421672 H
ZPE =    3896. kJ/mol
FREEH energy =  4089.00 kJ/mol
FREEH entropy =   1.62556 kJ/mol/K

$vibrational spectrum
#  mode     symmetry     wave number   IR intensity    selection rules
#                         cm**(-1)        km/mol          IR     RAMAN
     1                      -0.00         0.00000         -        -
     2                      -0.00         0.00000         -        -
     3                      -0.00         0.00000         -        -
     4                       0.00         0.00000         -        -
     5                       0.00         0.00000         -        -
     6                       0.00         0.00000         -        -
     7         a             6.99         0.03919        YES      YES
     8         a            19.21         0.25651        YES      YES
     9         a            22.49         0.17209        YES      YES
    10         a            23.37         0.22137        YES      YES
    11         a            28.05         0.17723        YES      YES
    12         a            32.91         0.02007        YES      YES
    13         a            36.59         0.02516        YES      YES
    14         a            40.08         0.19591        YES      YES
    15         a            41.95         0.08727        YES      YES
    16         a            44.13         0.09592        YES      YES
    17         a            47.77         0.03305        YES      YES
    18         a            49.43         0.01026        YES      YES
    19         a            51.62         0.06249        YES      YES
    20         a            52.21         0.27895        YES      YES
    21         a            59.47         0.06207        YES      YES
    22         a            61.51         0.13039        YES      YES
    23         a            62.98         0.06066        YES      YES
    24         a            65.28         0.25711        YES      YES
    25         a            68.29         0.13794        YES      YES
    26         a            70.64         0.20848        YES      YES
    27         a            71.85         0.34657        YES      YES
    28         a            73.69         0.15649        YES      YES
    29         a            76.55         0.03894        YES      YES
    30         a            79.03         0.31724        YES      YES
    31         a            83.99         0.05082        YES      YES
    32         a            86.10         0.13586        YES      YES
    33         a            89.71         0.54068        YES      YES
    34         a            91.55         0.11609        YES      YES
    35         a            97.41         0.26707        YES      YES
    36         a            99.44         0.20004        YES      YES
    37         a           101.25         0.02267        YES      YES
    38         a           110.16         0.17984        YES      YES
    39         a           125.70         1.13262        YES      YES
```

40	a	131.18	4.83505	YES	YES
41	a	132.68	0.30397	YES	YES
42	a	134.77	1.04255	YES	YES
43	a	138.59	0.61203	YES	YES
44	a	139.26	2.64451	YES	YES
45	a	144.27	5.76288	YES	YES
46	a	148.15	8.84151	YES	YES
47	a	153.71	1.48441	YES	YES
48	a	157.96	0.22623	YES	YES
49	a	160.39	0.52481	YES	YES
50	a	165.45	0.16492	YES	YES
51	a	174.21	0.33868	YES	YES
52	a	176.74	1.85479	YES	YES
53	a	181.23	4.14171	YES	YES
54	a	184.08	0.35722	YES	YES
55	a	197.36	0.32850	YES	YES
56	a	206.04	0.83374	YES	YES
57	a	207.02	2.22443	YES	YES
58	a	213.02	0.03604	YES	YES
59	a	217.67	0.17050	YES	YES
60	a	219.94	0.32970	YES	YES
61	a	225.94	0.79475	YES	YES
62	a	227.45	0.43681	YES	YES
63	a	228.27	0.13737	YES	YES
64	a	230.17	0.23476	YES	YES
65	a	235.61	0.49005	YES	YES
66	a	238.32	0.25868	YES	YES
67	a	238.77	0.30827	YES	YES
68	a	241.27	0.65406	YES	YES
69	a	243.77	0.44377	YES	YES
70	a	244.54	0.19899	YES	YES
71	a	250.96	1.53613	YES	YES
72	a	257.55	1.21210	YES	YES
73	a	284.28	1.87694	YES	YES
74	a	286.55	0.50257	YES	YES
75	a	288.98	0.67684	YES	YES
76	a	294.48	0.43448	YES	YES
77	a	300.94	2.60916	YES	YES
78	a	304.51	4.84018	YES	YES
79	a	311.08	0.53489	YES	YES
80	a	312.30	0.42567	YES	YES
81	a	322.59	0.06877	YES	YES
82	a	331.30	0.15656	YES	YES
83	a	332.49	0.98365	YES	YES
84	a	335.62	0.00237	YES	YES
85	a	337.08	0.37009	YES	YES
86	a	343.08	1.06613	YES	YES
87	a	346.16	1.06641	YES	YES
88	a	358.24	0.44935	YES	YES
89	a	360.62	2.89208	YES	YES
90	a	369.69	11.69039	YES	YES
91	a	379.14	3.61430	YES	YES
92	a	389.35	8.17781	YES	YES
93	a	392.90	2.66808	YES	YES
94	a	394.27	33.44073	YES	YES
95	a	409.12	1.25058	YES	YES
96	a	412.97	0.97156	YES	YES
97	a	414.37	1.13955	YES	YES

98	a	420.98	4.31988	YES	YES
99	a	422.84	0.99753	YES	YES
100	a	426.24	0.64847	YES	YES
101	a	427.83	3.90918	YES	YES
102	a	428.83	0.37355	YES	YES
103	a	429.52	0.91658	YES	YES
104	a	430.13	0.19506	YES	YES
105	a	431.13	0.28432	YES	YES
106	a	431.93	0.16483	YES	YES
107	a	434.51	0.86030	YES	YES
108	a	434.85	2.28398	YES	YES
109	a	436.25	4.97476	YES	YES
110	a	437.41	0.93755	YES	YES
111	a	440.66	2.06651	YES	YES
112	a	463.01	4.02905	YES	YES
113	a	464.22	8.60119	YES	YES
114	a	489.63	9.09634	YES	YES
115	a	490.23	2.52568	YES	YES
116	a	492.04	1.49988	YES	YES
117	a	494.08	2.48425	YES	YES
118	a	498.94	10.02031	YES	YES
119	a	500.87	0.45719	YES	YES
120	a	501.68	4.95004	YES	YES
121	a	512.32	19.39213	YES	YES
122	a	513.04	4.73268	YES	YES
123	a	534.87	61.01441	YES	YES
124	a	542.21	27.12580	YES	YES
125	a	583.29	95.77225	YES	YES
126	a	609.07	3.05780	YES	YES
127	a	631.47	20.58625	YES	YES
128	a	633.09	7.76564	YES	YES
129	a	653.49	11.73197	YES	YES
130	a	654.30	13.87589	YES	YES
131	a	657.31	6.87652	YES	YES
132	a	694.18	23.91454	YES	YES
133	a	708.18	0.38865	YES	YES
134	a	710.11	0.89125	YES	YES
135	a	714.91	0.55416	YES	YES
136	a	721.12	1.38181	YES	YES
137	a	729.53	0.89697	YES	YES
138	a	730.77	2.63880	YES	YES
139	a	735.85	6.64305	YES	YES
140	a	736.67	5.61223	YES	YES
141	a	743.75	42.29349	YES	YES
142	a	771.26	0.02328	YES	YES
143	a	771.71	0.27093	YES	YES
144	a	772.21	0.07467	YES	YES
145	a	772.53	0.53392	YES	YES
146	a	772.95	0.53582	YES	YES
147	a	774.69	0.25401	YES	YES
148	a	775.02	0.12117	YES	YES
149	a	776.30	0.05634	YES	YES
150	a	779.92	13.63233	YES	YES
151	a	787.64	12.86914	YES	YES
152	a	801.00	0.59014	YES	YES
153	a	807.59	1.95966	YES	YES
154	a	809.42	0.53801	YES	YES
155	a	810.88	1.52311	YES	YES

156	a	811.52	2.04469	YES	YES
157	a	812.61	0.51968	YES	YES
158	a	812.95	0.56062	YES	YES
159	a	815.09	5.73857	YES	YES
160	a	816.00	4.05661	YES	YES
161	a	834.37	0.76556	YES	YES
162	a	836.22	1.22251	YES	YES
163	a	836.73	1.09108	YES	YES
164	a	838.66	1.23216	YES	YES
165	a	838.99	7.51446	YES	YES
166	a	839.52	6.91888	YES	YES
167	a	840.52	8.83620	YES	YES
168	a	842.13	6.96023	YES	YES
169	a	851.41	10.82165	YES	YES
170	a	853.01	9.99388	YES	YES
171	a	855.93	3.88716	YES	YES
172	a	857.68	40.04492	YES	YES
173	a	872.76	2.82493	YES	YES
174	a	873.38	1.76740	YES	YES
175	a	874.67	2.19444	YES	YES
176	a	875.67	5.12129	YES	YES
177	a	876.45	1.22596	YES	YES
178	a	878.18	6.43858	YES	YES
179	a	878.63	3.22699	YES	YES
180	a	879.70	4.78606	YES	YES
181	a	880.14	0.61385	YES	YES
182	a	880.93	4.58200	YES	YES
183	a	881.36	0.93312	YES	YES
184	a	882.37	1.56865	YES	YES
185	a	883.91	0.23135	YES	YES
186	a	884.34	3.28429	YES	YES
187	a	885.74	0.83597	YES	YES
188	a	886.84	3.10706	YES	YES
189	a	904.59	2.20287	YES	YES
190	a	905.24	0.52538	YES	YES
191	a	907.25	1.73271	YES	YES
192	a	907.47	2.06568	YES	YES
193	a	908.93	0.10011	YES	YES
194	a	909.93	0.32800	YES	YES
195	a	910.55	1.16062	YES	YES
196	a	912.86	0.46954	YES	YES
197	a	939.86	0.10227	YES	YES
198	a	965.24	0.03491	YES	YES
199	a	985.46	0.76172	YES	YES
200	a	986.54	11.88332	YES	YES
201	a	987.36	9.29724	YES	YES
202	a	988.23	6.55873	YES	YES
203	a	989.36	11.74308	YES	YES
204	a	990.10	0.77138	YES	YES
205	a	990.78	13.50808	YES	YES
206	a	993.18	9.25696	YES	YES
207	a	995.48	5.67958	YES	YES
208	a	999.68	0.64602	YES	YES
209	a	1001.19	0.81898	YES	YES
210	a	1017.17	1.16361	YES	YES
211	a	1017.35	0.78946	YES	YES
212	a	1018.98	1.23747	YES	YES
213	a	1019.43	0.05254	YES	YES

214	a	1019.66	0.29975	YES	YES
215	a	1020.67	0.32755	YES	YES
216	a	1021.90	0.34902	YES	YES
217	a	1024.26	0.82628	YES	YES
218	a	1026.61	2.95142	YES	YES
219	a	1028.07	0.66489	YES	YES
220	a	1031.31	3.58059	YES	YES
221	a	1033.88	2.06202	YES	YES
222	a	1034.59	0.55218	YES	YES
223	a	1035.92	1.07432	YES	YES
224	a	1037.78	1.15544	YES	YES
225	a	1040.13	1.06693	YES	YES
226	a	1040.47	0.15367	YES	YES
227	a	1059.79	1.55156	YES	YES
228	a	1062.81	0.80825	YES	YES
229	a	1063.33	0.64225	YES	YES
230	a	1064.89	0.30300	YES	YES
231	a	1066.17	0.26098	YES	YES
232	a	1067.18	1.50226	YES	YES
233	a	1069.29	0.89131	YES	YES
234	a	1071.52	3.45619	YES	YES
235	a	1072.14	0.32341	YES	YES
236	a	1072.54	2.06043	YES	YES
237	a	1072.85	1.80264	YES	YES
238	a	1073.41	0.13936	YES	YES
239	a	1073.53	0.22591	YES	YES
240	a	1074.08	0.61437	YES	YES
241	a	1074.41	2.04340	YES	YES
242	a	1074.91	0.07754	YES	YES
243	a	1075.79	0.03179	YES	YES
244	a	1088.30	1.50558	YES	YES
245	a	1089.24	3.63106	YES	YES
246	a	1093.05	0.29836	YES	YES
247	a	1093.31	0.27203	YES	YES
248	a	1097.23	4.67621	YES	YES
249	a	1098.44	13.38985	YES	YES
250	a	1102.04	3.82056	YES	YES
251	a	1105.24	1.20764	YES	YES
252	a	1106.08	2.15645	YES	YES
253	a	1107.08	6.14691	YES	YES
254	a	1132.97	0.79753	YES	YES
255	a	1135.54	3.85826	YES	YES
256	a	1149.78	0.90294	YES	YES
257	a	1160.71	2.56039	YES	YES
258	a	1162.58	5.39816	YES	YES
259	a	1163.32	6.13995	YES	YES
260	a	1164.76	12.88571	YES	YES
261	a	1165.39	1.48614	YES	YES
262	a	1169.48	1.94378	YES	YES
263	a	1169.78	11.26549	YES	YES
264	a	1170.36	7.86888	YES	YES
265	a	1171.60	1.72021	YES	YES
266	a	1175.89	6.65197	YES	YES
267	a	1177.97	6.04334	YES	YES
268	a	1179.86	3.98881	YES	YES
269	a	1184.56	0.05940	YES	YES
270	a	1186.22	18.52997	YES	YES
271	a	1189.26	12.79226	YES	YES

272	a	1191.05	3.91259	YES	YES
273	a	1195.75	16.54925	YES	YES
274	a	1219.91	0.91128	YES	YES
275	a	1243.44	0.38277	YES	YES
276	a	1243.80	0.56275	YES	YES
277	a	1245.92	0.20484	YES	YES
278	a	1246.32	1.02436	YES	YES
279	a	1247.26	0.28597	YES	YES
280	a	1249.01	1.41903	YES	YES
281	a	1249.17	1.72390	YES	YES
282	a	1250.72	0.45286	YES	YES
283	a	1251.50	3.05675	YES	YES
284	a	1251.71	1.29888	YES	YES
285	a	1253.16	6.72913	YES	YES
286	a	1255.02	2.09646	YES	YES
287	a	1256.09	9.36401	YES	YES
288	a	1257.52	1.37112	YES	YES
289	a	1257.79	5.53277	YES	YES
290	a	1258.32	2.16586	YES	YES
291	a	1258.45	4.07680	YES	YES
292	a	1258.82	10.74530	YES	YES
293	a	1259.51	1.16957	YES	YES
294	a	1259.83	6.36838	YES	YES
295	a	1260.37	1.86473	YES	YES
296	a	1263.33	3.97732	YES	YES
297	a	1264.06	1.94073	YES	YES
298	a	1268.48	2.69575	YES	YES
299	a	1268.70	1.76292	YES	YES
300	a	1270.24	0.48214	YES	YES
301	a	1271.12	1.80073	YES	YES
302	a	1272.77	0.99566	YES	YES
303	a	1283.87	0.98483	YES	YES
304	a	1284.76	3.56905	YES	YES
305	a	1287.20	2.09735	YES	YES
306	a	1288.84	4.64662	YES	YES
307	a	1290.07	4.01880	YES	YES
308	a	1290.38	7.48114	YES	YES
309	a	1294.08	3.85134	YES	YES
310	a	1294.84	87.26953	YES	YES
311	a	1295.79	32.05357	YES	YES
312	a	1308.17	1.20380	YES	YES
313	a	1308.42	1.09905	YES	YES
314	a	1312.03	0.38838	YES	YES
315	a	1314.65	1.93509	YES	YES
316	a	1315.66	0.77068	YES	YES
317	a	1315.76	1.09499	YES	YES
318	a	1316.39	1.18560	YES	YES
319	a	1317.11	6.27917	YES	YES
320	a	1320.94	0.30243	YES	YES
321	a	1322.44	0.66882	YES	YES
322	a	1323.05	2.00909	YES	YES
323	a	1323.62	1.27145	YES	YES
324	a	1323.90	1.52158	YES	YES
325	a	1324.42	1.25372	YES	YES
326	a	1324.88	0.66752	YES	YES
327	a	1324.92	2.49158	YES	YES
328	a	1325.59	0.13565	YES	YES
329	a	1326.04	2.30528	YES	YES

330	a	1327.01	3.55694	YES	YES
331	a	1327.07	2.52888	YES	YES
332	a	1329.00	2.97766	YES	YES
333	a	1329.33	6.25326	YES	YES
334	a	1330.22	0.59189	YES	YES
335	a	1332.38	0.63309	YES	YES
336	a	1335.82	12.64312	YES	YES
337	a	1336.71	19.40163	YES	YES
338	a	1337.92	0.29471	YES	YES
339	a	1338.20	1.64830	YES	YES
340	a	1339.86	0.01570	YES	YES
341	a	1340.33	0.50942	YES	YES
342	a	1341.56	0.15929	YES	YES
343	a	1342.87	0.27426	YES	YES
344	a	1342.97	1.00555	YES	YES
345	a	1345.94	1.08821	YES	YES
346	a	1346.69	0.52847	YES	YES
347	a	1347.73	0.05198	YES	YES
348	a	1347.89	2.64813	YES	YES
349	a	1348.55	0.46691	YES	YES
350	a	1349.68	0.19750	YES	YES
351	a	1350.54	0.27877	YES	YES
352	a	1353.66	0.15680	YES	YES
353	a	1362.32	29.98006	YES	YES
354	a	1402.03	8.28984	YES	YES
355	a	1404.17	11.98628	YES	YES
356	a	1407.78	6.96800	YES	YES
357	a	1408.54	5.82445	YES	YES
358	a	1423.89	1.34129	YES	YES
359	a	1425.15	1.98571	YES	YES
360	a	1426.39	6.86694	YES	YES
361	a	1429.72	2.34603	YES	YES
362	a	1431.13	3.23807	YES	YES
363	a	1432.20	2.05710	YES	YES
364	a	1432.77	1.10254	YES	YES
365	a	1432.93	3.05789	YES	YES
366	a	1434.24	3.96315	YES	YES
367	a	1435.51	1.83641	YES	YES
368	a	1436.69	7.01535	YES	YES
369	a	1437.14	3.41666	YES	YES
370	a	1437.41	1.70193	YES	YES
371	a	1437.51	6.48449	YES	YES
372	a	1437.86	1.99961	YES	YES
373	a	1438.22	2.81595	YES	YES
374	a	1438.32	3.78531	YES	YES
375	a	1438.58	7.53715	YES	YES
376	a	1438.93	9.84029	YES	YES
377	a	1439.21	6.39206	YES	YES
378	a	1439.54	6.96618	YES	YES
379	a	1440.53	5.90767	YES	YES
380	a	1441.40	3.90488	YES	YES
381	a	1442.74	5.24261	YES	YES
382	a	1443.55	19.37506	YES	YES
383	a	1443.72	26.17320	YES	YES
384	a	1443.86	6.77742	YES	YES
385	a	1443.99	3.07524	YES	YES
386	a	1445.55	17.44716	YES	YES
387	a	1446.13	25.40871	YES	YES

388	a	1447.26	8.33403	YES	YES
389	a	1447.82	18.84813	YES	YES
390	a	1452.60	4.37893	YES	YES
391	a	1453.57	3.32452	YES	YES
392	a	1454.08	1.86840	YES	YES
393	a	1455.07	2.68975	YES	YES
394	a	1455.37	0.42791	YES	YES
395	a	1456.07	2.08333	YES	YES
396	a	1456.87	0.36077	YES	YES
397	a	1457.32	0.46822	YES	YES
398	a	1461.34	8.09668	YES	YES
399	a	1485.02	123.74588	YES	YES
400	a	1573.90	4.90074	YES	YES
401	a	1598.75	103.48911	YES	YES
402	a	1871.26	132.63731	YES	YES
403	a	2922.05	15.55462	YES	YES
404	a	2923.80	3.20595	YES	YES
405	a	2928.15	3.78411	YES	YES
406	a	2928.62	5.31884	YES	YES
407	a	2930.66	20.41845	YES	YES
408	a	2932.33	2.04218	YES	YES
409	a	2936.92	18.64300	YES	YES
410	a	2937.74	4.83957	YES	YES
411	a	2940.51	11.55764	YES	YES
412	a	2941.88	29.17370	YES	YES
413	a	2943.75	18.57693	YES	YES
414	a	2944.93	3.77744	YES	YES
415	a	2945.04	3.10873	YES	YES
416	a	2945.65	9.13689	YES	YES
417	a	2945.72	13.71881	YES	YES
418	a	2946.00	22.99128	YES	YES
419	a	2947.44	2.47792	YES	YES
420	a	2947.61	27.50599	YES	YES
421	a	2949.05	16.53958	YES	YES
422	a	2950.29	8.70973	YES	YES
423	a	2950.64	6.39791	YES	YES
424	a	2950.90	14.45446	YES	YES
425	a	2951.05	8.25327	YES	YES
426	a	2951.42	12.69916	YES	YES
427	a	2951.60	10.28678	YES	YES
428	a	2952.49	7.84444	YES	YES
429	a	2953.26	19.10029	YES	YES
430	a	2956.71	6.69119	YES	YES
431	a	2957.50	4.81606	YES	YES
432	a	2958.09	2.01576	YES	YES
433	a	2959.06	0.65246	YES	YES
434	a	2959.37	16.07025	YES	YES
435	a	2959.90	10.99962	YES	YES
436	a	2960.31	1.24175	YES	YES
437	a	2961.32	34.16651	YES	YES
438	a	2961.65	36.03176	YES	YES
439	a	2961.94	17.46237	YES	YES
440	a	2963.48	56.29388	YES	YES
441	a	2964.51	48.61855	YES	YES
442	a	2965.06	40.47399	YES	YES
443	a	2965.97	10.24961	YES	YES
444	a	2966.16	18.46880	YES	YES
445	a	2968.63	39.22507	YES	YES

446	a	2972.27	24.24426	YES	YES
447	a	2973.46	12.29547	YES	YES
448	a	2973.80	0.91762	YES	YES
449	a	2980.00	9.20098	YES	YES
450	a	2982.36	2.11569	YES	YES
451	a	2983.56	32.84738	YES	YES
452	a	2984.49	23.97838	YES	YES
453	a	2986.28	5.53465	YES	YES
454	a	2990.67	3.50537	YES	YES
455	a	2993.16	4.92859	YES	YES
456	a	2994.34	23.41609	YES	YES
457	a	3003.29	28.00268	YES	YES
458	a	3006.19	37.51877	YES	YES
459	a	3006.29	3.61712	YES	YES
460	a	3006.33	23.32577	YES	YES
461	a	3007.84	19.49740	YES	YES
462	a	3008.13	24.73956	YES	YES
463	a	3008.69	27.07875	YES	YES
464	a	3008.78	10.11682	YES	YES
465	a	3008.98	6.47253	YES	YES
466	a	3009.07	27.12135	YES	YES
467	a	3009.76	24.27477	YES	YES
468	a	3010.07	9.74635	YES	YES
469	a	3010.30	19.64373	YES	YES
470	a	3011.92	9.25471	YES	YES
471	a	3011.94	19.76634	YES	YES
472	a	3012.42	16.44853	YES	YES
473	a	3012.69	17.10797	YES	YES
474	a	3013.13	35.31934	YES	YES
475	a	3013.34	14.76329	YES	YES
476	a	3014.04	17.33042	YES	YES
477	a	3015.69	34.88127	YES	YES
478	a	3015.94	34.14006	YES	YES
479	a	3016.61	35.88679	YES	YES
480	a	3016.91	25.48875	YES	YES
481	a	3017.69	30.19720	YES	YES
482	a	3018.41	22.70458	YES	YES
483	a	3018.60	44.97284	YES	YES
484	a	3018.78	28.47614	YES	YES
485	a	3019.22	19.45754	YES	YES
486	a	3019.44	21.86784	YES	YES
487	a	3019.98	5.13293	YES	YES
488	a	3020.95	54.27220	YES	YES
489	a	3021.06	8.52143	YES	YES
490	a	3026.61	17.26543	YES	YES
491	a	3028.19	8.86876	YES	YES
492	a	3028.63	6.29084	YES	YES
493	a	3031.18	13.95385	YES	YES
494	a	3032.30	9.64803	YES	YES
495	a	3034.82	0.32561	YES	YES
496	a	3038.37	0.22130	YES	YES
497	a	3048.27	0.57137	YES	YES
498	a	3063.19	0.39000	YES	YES
499	a	3081.42	13.65875	YES	YES
500	a	3087.99	20.25649	YES	YES
501	a	3104.28	11.46131	YES	YES
502	a	3116.82	5.54143	YES	YES
503	a	3138.33	2.12619	YES	YES

504	a	3491.34	15.62770	YES	YES

$end

Double hybrid single point energy = -7536.336316713699 H
COSMO enery + OC correction = -7544.1277460103 H (in *o*DFB)

6.2.18 H_2N*n*Bu

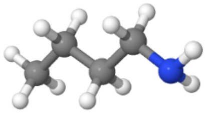

Method: (RI-)BP86(D3BJ)/def2-TZVPP
Symmetry: c1

Cartesian coordinates in Ångström:

N	-1.2439487	-1.9609154	-0.0850574
C	0.2091963	-1.7514658	0.0058493
H	-1.7320070	-1.0641260	-0.0902246
H	-1.5734254	-2.4581697	0.7449915
C	0.9489535	-3.0866512	-0.0104457
H	0.5245099	-1.1771113	0.9011477
H	0.5160770	-1.1575826	-0.8696770
C	2.4709193	-2.9339773	0.0341031
H	0.6494581	-3.6447751	-0.9114307
H	0.6190101	-3.6934686	0.8512992
C	3.2048810	-4.2760478	0.0284190
H	2.7550881	-2.3609787	0.9319221
H	2.7991429	-2.3299016	-0.8275767
H	2.9195667	-4.8865335	0.8975024
H	4.2947238	-4.1423259	0.0578309
H	2.9629745	-4.8550895	-0.8744430

SCF energy GEOOPT = -213.9066145926 H
ZPE = 381.6 kJ/mol
FREEH energy = 400.35 kJ/mol
FREEH entropy = 0.33937 kJ/mol/K

$vibrational spectrum

# mode	symmetry	wave number	IR intensity	selection rules	
#		cm**(-1)	km/mol	IR	RAMAN
1		0.00	0.00000	-	-
2		0.00	0.00000	-	-
3		0.00	0.00000	-	-
4		0.00	0.00000	-	-
5		0.00	0.00000	-	-
6		0.00	0.00000	-	-
7	a	102.56	2.39019	YES	YES
8	a	110.25	1.48207	YES	YES
9	a	172.97	0.99504	YES	YES
10	a	238.76	6.19505	YES	YES
11	a	243.23	29.60202	YES	YES
12	a	385.53	0.04480	YES	YES
13	a	421.65	8.91091	YES	YES
14	a	722.19	3.56525	YES	YES
15	a	777.61	16.61253	YES	YES

16	a	822.99	111.37202	YES	YES
17	a	880.43	1.30093	YES	YES
18	a	925.72	5.42881	YES	YES
19	a	956.72	3.28780	YES	YES
20	a	1033.10	0.13483	YES	YES
21	a	1047.56	3.24407	YES	YES
22	a	1071.53	8.23842	YES	YES
23	a	1125.95	5.72198	YES	YES
24	a	1187.49	0.19968	YES	YES
25	a	1227.12	1.51849	YES	YES
26	a	1270.77	1.04424	YES	YES
27	a	1285.65	0.69096	YES	YES
28	a	1293.31	1.39709	YES	YES
29	a	1341.18	0.76899	YES	YES
30	a	1363.82	3.34678	YES	YES
31	a	1377.87	11.58551	YES	YES
32	a	1439.30	0.20509	YES	YES
33	a	1446.29	0.41544	YES	YES
34	a	1452.38	7.48408	YES	YES
35	a	1459.91	4.93648	YES	YES
36	a	1470.78	2.77310	YES	YES
37	a	1604.96	24.12132	YES	YES
38	a	2859.16	81.16524	YES	YES
39	a	2928.61	9.52036	YES	YES
40	a	2943.11	33.69859	YES	YES
41	a	2958.58	30.52485	YES	YES
42	a	2963.40	41.08585	YES	YES
43	a	2966.81	10.24408	YES	YES
44	a	2991.76	50.11259	YES	YES
45	a	3023.37	55.95006	YES	YES
46	a	3030.03	37.88536	YES	YES
47	a	3396.74	2.22330	YES	YES
48	a	3477.05	0.13320	YES	YES

$end

Double hybrid single point energy = -213.511849899828 H
COSMO enery + OC correction = -213.9128469055 H (in *o*DFB)

6.2.19 [H{Ga(dcpe)}$_2$(HN*n*Bu)]$^{2+}$

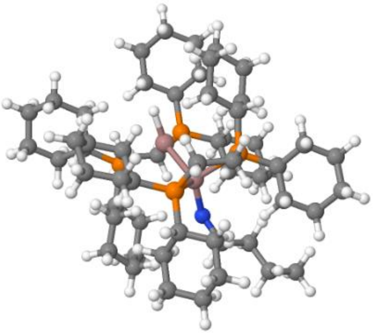

Method: (RI-)BP86(D3BJ)/def2-TZVPP
Symmetry: c1

Cartesian coordinates in Ångström:
Ga -0.3504018 0.5347406 -1.8842889

P	-2.1571665	-0.7530468	-2.9618482
P	-1.7289064	2.5081166	-2.3988973
C	-1.6754000	-1.8717089	-4.3582907
H	-0.8181624	-2.4367936	-3.9491927
C	-1.1687409	-1.0688390	-5.5681739
H	-1.9987365	-0.4803652	-5.9901046
H	-0.3934011	-0.3565410	-5.2584388
C	-0.6339525	-2.0072081	-6.6560027
H	-0.2866561	-1.4156081	-7.5139757
H	0.2468375	-2.5437918	-6.2672616
C	-1.7010451	-3.0152104	-7.0901876
H	-1.2907347	-3.7046550	-7.8399183
H	-2.5306757	-2.4795185	-7.5801108
C	-2.2394640	-3.7989536	-5.8904173
H	-1.4356283	-4.4276525	-5.4724771
H	-3.0403060	-4.4829855	-6.2022341
C	-2.7650917	-2.8700508	-4.7857382
H	-3.1038083	-3.4740791	-3.9336671
H	-3.6428537	-2.3151479	-5.1559638
C	-3.1621406	-1.7743768	-1.7992771
H	-3.9257855	-2.2534424	-2.4359107
C	-2.3098557	-2.8765719	-1.1521375
H	-1.7975875	-3.4761277	-1.9190570
H	-1.5210639	-2.4017577	-0.5416740
C	-3.1811333	-3.7743390	-0.2653843
H	-3.9119140	-4.3016898	-0.8994317
H	-2.5626879	-4.5526349	0.2015717
C	-3.9240292	-2.9574970	0.7964237
H	-4.5655372	-3.6130395	1.4003438
H	-3.1958172	-2.5039258	1.4896762
C	-4.7545497	-1.8413070	0.1593004
H	-5.2407109	-1.2329005	0.9341684
H	-5.5630466	-2.2835001	-0.4449608
C	-3.8959906	-0.9395417	-0.7373766
H	-4.5425694	-0.1932998	-1.2179846
H	-3.1608972	-0.3958305	-0.1216639
C	-3.3388057	0.5023042	-3.6268001
H	-3.0200198	0.7188179	-4.6527630
H	-4.3426461	0.0588324	-3.6897406
C	-3.3869137	1.8036921	-2.8049998
H	-3.8874023	1.6346544	-1.8425402
H	-3.9746151	2.5624773	-3.3397060
C	-1.2263720	3.5796989	-3.8177079
H	-1.9088778	4.4465011	-3.7873042
C	0.2122159	4.0737414	-3.5855436
H	0.8729790	3.2058763	-3.4441890
H	0.2567191	4.6553551	-2.6543965
C	0.7168789	4.9266147	-4.7542144
H	0.1421731	5.8659405	-4.7980957
H	1.7632781	5.2079618	-4.5717636
C	0.5793308	4.1849866	-6.0861477
H	1.2287698	3.2941515	-6.0805623
H	0.9264011	4.8195366	-6.9122268
C	-0.8725526	3.7611427	-6.3234309
H	-0.9697223	3.2144152	-7.2714317
H	-1.5042962	4.6595090	-6.4147374
C	-1.4048135	2.8886175	-5.1791978
H	-0.8723928	1.9247135	-5.1801859

H	-2.4676181	2.6669728	-5.3515084
C	-2.0198126	3.6763192	-0.9930777
H	-1.0878046	4.2654202	-0.9466799
C	-2.1774515	2.9383933	0.3432208
H	-1.2998113	2.3001388	0.5218952
H	-3.0467126	2.2616170	0.3006551
C	-2.3540712	3.9228087	1.5039066
H	-2.5043317	3.3643039	2.4382265
H	-1.4218913	4.5013472	1.6257126
C	-3.5145605	4.8907661	1.2583426
H	-4.4624246	4.3278547	1.2461933
H	-3.5902492	5.6100593	2.0846916
C	-3.3424176	5.6261773	-0.0732460
H	-2.4489144	6.2701865	-0.0277638
H	-4.1969338	6.2898812	-0.2613371
C	-3.1949441	4.6403680	-1.2402938
H	-4.1273742	4.0621644	-1.3386803
H	-3.0588298	5.1911734	-2.1810658
Ga	-0.1797497	-0.0455239	0.5046913
P	1.4370681	1.3112729	1.7274523
P	1.1686041	-1.9637917	1.1614292
C	0.7535672	2.3550759	3.0940415
H	0.2291113	3.1677529	2.5616480
C	-0.2837057	1.5953425	3.9367392
H	0.2066490	0.7432625	4.4338299
H	-1.0659194	1.1752627	3.2893502
C	-0.8944716	2.5078712	5.0078241
H	-1.6096824	1.9339802	5.6130071
H	-1.4710124	3.3074897	4.5143334
C	0.1844542	3.1352608	5.8943333
H	-0.2719923	3.8130490	6.6278939
H	0.6890009	2.3436381	6.4728117
C	1.2190279	3.8858759	5.0521676
H	0.7376500	4.7442024	4.5551780
H	2.0130547	4.2983477	5.6892467
C	1.8449697	2.9765439	3.9848273
H	2.5654981	3.5505114	3.3864578
H	2.4099245	2.1720962	4.4831226
C	2.5098752	2.4403236	0.7371377
H	3.0866363	3.0105577	1.4853561
C	1.6603981	3.4280258	-0.0783096
H	0.9690367	3.9770973	0.5781318
H	1.0515507	2.8557926	-0.7982596
C	2.5611776	4.4033135	-0.8442451
H	3.1334699	5.0114059	-0.1249877
H	1.9462527	5.1070763	-1.4211716
C	3.5244369	3.6467177	-1.7624173
H	4.1712648	4.3507541	-2.3025034
H	2.9426330	3.0950053	-2.5184290
C	4.3755506	2.6562327	-0.9628954
H	5.0363511	2.0860989	-1.6303816
H	5.0319158	3.2112228	-0.2732874
C	3.5074336	1.6836866	-0.1530209
H	4.1543956	1.0383608	0.4571919
H	2.9466989	1.0380171	-0.8447071
C	2.5904257	0.0948187	2.5116638
H	2.1866167	-0.1221222	3.5079146
H	3.5653085	0.5780692	2.6647553

C	2.7635527	-1.2017417	1.7030462
H	3.3336870	-1.0043165	0.7846548
H	3.3348592	-1.9379911	2.2850031
C	0.5477978	-2.9564250	2.5999789
H	1.4137141	-3.5473113	2.9453766
C	-0.5647713	-3.9105259	2.1386244
H	-1.3626096	-3.3107530	1.6754208
H	-0.1904412	-4.5984752	1.3687894
C	-1.1529086	-4.7155298	3.3030418
H	-0.3836042	-5.4001290	3.6953633
H	-1.9701601	-5.3477873	2.9287684
C	-1.6444878	-3.8019152	4.4271661
H	-2.4841684	-3.1874632	4.0612315
H	-2.0336884	-4.3997424	5.2618176
C	-0.5165814	-2.8872843	4.9078520
H	-0.8741012	-2.2081477	5.6941505
H	0.2830675	-3.4964311	5.3596201
C	0.0642438	-2.0612713	3.7536694
H	-0.7041492	-1.3709724	3.3716927
H	0.8911226	-1.4452755	4.1305040
C	1.5907667	-3.1943847	-0.1481358
H	0.6315878	-3.7089464	-0.3296183
C	2.0045001	-2.5325365	-1.4684992
H	1.2338877	-1.8194724	-1.7947673
H	2.9334443	-1.9545320	-1.3229502
C	2.2338200	-3.5908528	-2.5543129
H	2.5557276	-3.1015569	-3.4831365
H	1.2720430	-4.0854784	-2.7744450
C	3.2568907	-4.6409855	-2.1121917
H	4.2427388	-4.1608787	-1.9966350
H	3.3746065	-5.4077800	-2.8894860
C	2.8490624	-5.2891795	-0.7861473
H	1.9191336	-5.8644138	-0.9275160
H	3.6122603	-6.0063064	-0.4553874
C	2.6245506	-4.2382386	0.3098535
H	3.5788347	-3.7327723	0.5290334
H	2.3014651	-4.7281392	1.2388041
H	-1.4463623	-0.1328703	1.4381754
N	1.2964759	0.6535832	-2.7608036
C	1.6908676	0.8026922	-4.1532467
H	2.0155203	0.1488032	-2.2481745
C	2.2820706	-0.4626011	-4.7729214
H	2.4313737	1.6209148	-4.2592303
H	0.8212198	1.1275785	-4.7417012
C	2.6643840	-0.2984161	-6.2443496
H	1.5597528	-1.2881302	-4.6596727
H	3.1735429	-0.7565557	-4.1917152
C	3.2982312	-1.5566215	-6.8375390
H	3.3587048	0.5504668	-6.3471307
H	1.7659371	-0.0253964	-6.8221033
H	4.2231037	-1.8216162	-6.3062355
H	3.5516199	-1.4201857	-7.8961213
H	2.6169083	-2.4167791	-6.7674876

SCF energy GEOOPT = -7470.128410288 H
ZPE = 3976. kJ/mol
FREEH energy = 4172.32 kJ/mol
FREEH entropy = 1.67823 kJ/mol/K

$vibrational spectrum

# mode	symmetry	wave number cm**(-1)	IR intensity km/mol	selection rules IR	RAMAN
1		0.00	0.00000	-	-
2		0.00	0.00000	-	-
3		0.00	0.00000	-	-
4		0.00	0.00000	-	-
5		0.00	0.00000	-	-
6		0.00	0.00000	-	-
7	a	2.19	0.07543	YES	YES
8	a	13.92	0.01323	YES	YES
9	a	18.50	0.03970	YES	YES
10	a	22.34	0.04428	YES	YES
11	a	23.19	0.09033	YES	YES
12	a	27.83	0.11344	YES	YES
13	a	31.07	0.02150	YES	YES
14	a	36.67	0.01145	YES	YES
15	a	38.84	0.16138	YES	YES
16	a	40.25	0.03127	YES	YES
17	a	43.17	0.02154	YES	YES
18	a	44.01	0.02768	YES	YES
19	a	46.77	0.17609	YES	YES
20	a	49.09	0.16373	YES	YES
21	a	52.63	0.06986	YES	YES
22	a	54.05	0.14223	YES	YES
23	a	56.57	0.04852	YES	YES
24	a	60.55	0.07518	YES	YES
25	a	62.98	0.06500	YES	YES
26	a	64.90	0.14052	YES	YES
27	a	69.43	0.22564	YES	YES
28	a	70.46	0.16222	YES	YES
29	a	70.80	0.04169	YES	YES
30	a	76.70	0.06604	YES	YES
31	a	79.23	0.17023	YES	YES
32	a	84.39	1.25185	YES	YES
33	a	84.85	0.03629	YES	YES
34	a	88.30	0.69645	YES	YES
35	a	93.04	0.16282	YES	YES
36	a	97.08	0.44843	YES	YES
37	a	100.26	1.01313	YES	YES
38	a	103.20	0.17958	YES	YES
39	a	112.82	3.59552	YES	YES
40	a	126.50	0.89926	YES	YES
41	a	130.75	0.29552	YES	YES
42	a	134.48	1.16383	YES	YES
43	a	138.39	4.40123	YES	YES
44	a	142.66	2.98650	YES	YES
45	a	145.46	2.38872	YES	YES
46	a	146.47	0.09310	YES	YES
47	a	148.51	0.50094	YES	YES
48	a	149.82	0.65943	YES	YES
49	a	155.05	1.26754	YES	YES
50	a	158.90	1.07139	YES	YES
51	a	162.29	1.06123	YES	YES
52	a	176.61	3.17161	YES	YES
53	a	176.92	4.28651	YES	YES
54	a	182.72	0.12558	YES	YES

55	a	184.00	0.47903	YES	YES
56	a	193.40	1.71034	YES	YES
57	a	201.66	2.03702	YES	YES
58	a	205.13	0.88681	YES	YES
59	a	206.46	1.91763	YES	YES
60	a	209.72	1.55855	YES	YES
61	a	215.66	1.69194	YES	YES
62	a	216.99	0.28809	YES	YES
63	a	218.83	0.15764	YES	YES
64	a	222.84	0.96547	YES	YES
65	a	225.18	1.77567	YES	YES
66	a	229.12	1.58724	YES	YES
67	a	233.85	0.05170	YES	YES
68	a	237.09	0.08482	YES	YES
69	a	238.12	1.25665	YES	YES
70	a	240.16	0.10683	YES	YES
71	a	243.43	0.82408	YES	YES
72	a	247.22	0.15926	YES	YES
73	a	248.36	0.35374	YES	YES
74	a	249.30	0.72130	YES	YES
75	a	268.05	3.13407	YES	YES
76	a	277.51	2.42452	YES	YES
77	a	279.15	1.33900	YES	YES
78	a	290.61	0.29522	YES	YES
79	a	293.46	0.03445	YES	YES
80	a	299.99	0.99635	YES	YES
81	a	306.49	0.67674	YES	YES
82	a	314.00	0.14916	YES	YES
83	a	315.71	0.26356	YES	YES
84	a	329.86	0.35995	YES	YES
85	a	330.85	0.55906	YES	YES
86	a	333.91	0.42558	YES	YES
87	a	336.30	0.45735	YES	YES
88	a	341.46	2.15113	YES	YES
89	a	342.83	3.43717	YES	YES
90	a	353.98	46.06172	YES	YES
91	a	368.49	0.93491	YES	YES
92	a	371.97	3.28294	YES	YES
93	a	373.97	0.73496	YES	YES
94	a	377.48	1.73206	YES	YES
95	a	391.96	0.52676	YES	YES
96	a	394.28	0.94041	YES	YES
97	a	400.97	0.71445	YES	YES
98	a	410.57	1.34733	YES	YES
99	a	414.11	0.84797	YES	YES
100	a	420.84	3.83739	YES	YES
101	a	421.65	8.17643	YES	YES
102	a	423.69	5.31365	YES	YES
103	a	425.69	2.14960	YES	YES
104	a	428.28	0.88575	YES	YES
105	a	429.13	0.01198	YES	YES
106	a	430.89	0.95105	YES	YES
107	a	431.62	0.51626	YES	YES
108	a	432.34	0.19726	YES	YES
109	a	433.22	0.44806	YES	YES
110	a	433.36	1.72724	YES	YES
111	a	433.74	0.55776	YES	YES
112	a	439.73	10.64875	YES	YES

113	a	443.85	6.39601	YES	YES
114	a	448.78	2.95253	YES	YES
115	a	466.73	4.01466	YES	YES
116	a	468.82	5.17781	YES	YES
117	a	489.56	4.17018	YES	YES
118	a	491.03	1.06685	YES	YES
119	a	492.09	0.19750	YES	YES
120	a	494.54	3.79355	YES	YES
121	a	501.71	4.65142	YES	YES
122	a	504.92	0.37659	YES	YES
123	a	516.18	19.33789	YES	YES
124	a	517.84	6.00528	YES	YES
125	a	565.79	68.72152	YES	YES
126	a	586.79	114.26534	YES	YES
127	a	621.70	28.12270	YES	YES
128	a	629.20	32.48395	YES	YES
129	a	629.58	4.04739	YES	YES
130	a	645.38	13.20552	YES	YES
131	a	645.88	3.72649	YES	YES
132	a	711.13	0.25681	YES	YES
133	a	712.19	0.46855	YES	YES
134	a	718.30	1.93431	YES	YES
135	a	721.32	1.01120	YES	YES
136	a	723.74	4.50925	YES	YES
137	a	731.56	6.04835	YES	YES
138	a	732.48	10.09255	YES	YES
139	a	735.34	3.15945	YES	YES
140	a	737.06	6.18313	YES	YES
141	a	771.50	0.14192	YES	YES
142	a	772.42	0.07042	YES	YES
143	a	773.28	0.38047	YES	YES
144	a	774.30	0.07314	YES	YES
145	a	774.43	0.20463	YES	YES
146	a	774.90	0.68767	YES	YES
147	a	775.42	0.56503	YES	YES
148	a	775.91	0.69700	YES	YES
149	a	781.82	2.16467	YES	YES
150	a	784.89	19.77638	YES	YES
151	a	786.25	4.30808	YES	YES
152	a	809.45	0.51574	YES	YES
153	a	810.46	0.81213	YES	YES
154	a	811.20	2.20369	YES	YES
155	a	812.23	1.55172	YES	YES
156	a	814.04	0.98412	YES	YES
157	a	814.73	2.33022	YES	YES
158	a	815.24	5.27291	YES	YES
159	a	816.25	4.49269	YES	YES
160	a	832.81	0.29423	YES	YES
161	a	833.88	0.63741	YES	YES
162	a	835.99	2.44684	YES	YES
163	a	836.85	2.93318	YES	YES
164	a	837.26	5.39145	YES	YES
165	a	837.66	10.26714	YES	YES
166	a	838.76	5.82954	YES	YES
167	a	838.94	7.82008	YES	YES
168	a	852.20	15.98092	YES	YES
169	a	853.23	0.91984	YES	YES
170	a	874.31	2.95580	YES	YES

171	a	875.76	6.24705	YES	YES
172	a	875.93	0.55714	YES	YES
173	a	876.84	4.51629	YES	YES
174	a	877.18	2.16322	YES	YES
175	a	877.76	3.18839	YES	YES
176	a	878.60	1.21205	YES	YES
177	a	879.42	2.76086	YES	YES
178	a	879.83	0.67019	YES	YES
179	a	880.41	4.56147	YES	YES
180	a	880.57	4.04943	YES	YES
181	a	882.31	2.74465	YES	YES
182	a	883.52	1.17814	YES	YES
183	a	884.25	1.24633	YES	YES
184	a	886.63	5.34638	YES	YES
185	a	888.51	1.39733	YES	YES
186	a	889.44	3.66904	YES	YES
187	a	903.10	1.84374	YES	YES
188	a	903.48	1.32988	YES	YES
189	a	905.04	0.74004	YES	YES
190	a	905.89	2.86180	YES	YES
191	a	908.36	1.05893	YES	YES
192	a	909.60	0.39559	YES	YES
193	a	910.02	3.37624	YES	YES
194	a	911.29	0.19664	YES	YES
195	a	920.62	1.72736	YES	YES
196	a	984.44	0.09764	YES	YES
197	a	984.77	0.27783	YES	YES
198	a	988.10	5.42248	YES	YES
199	a	988.41	8.53399	YES	YES
200	a	989.16	13.08871	YES	YES
201	a	989.82	12.83165	YES	YES
202	a	990.45	14.75028	YES	YES
203	a	991.19	21.67335	YES	YES
204	a	991.94	6.63646	YES	YES
205	a	992.30	1.45835	YES	YES
206	a	992.58	1.38297	YES	YES
207	a	1015.66	0.32919	YES	YES
208	a	1015.99	0.21147	YES	YES
209	a	1016.80	0.88432	YES	YES
210	a	1017.67	0.31002	YES	YES
211	a	1018.18	1.19444	YES	YES
212	a	1018.27	0.72809	YES	YES
213	a	1021.96	1.40446	YES	YES
214	a	1024.20	0.60796	YES	YES
215	a	1031.66	3.06044	YES	YES
216	a	1032.73	0.94678	YES	YES
217	a	1033.44	1.61098	YES	YES
218	a	1034.67	2.12373	YES	YES
219	a	1038.85	2.08855	YES	YES
220	a	1039.75	0.56818	YES	YES
221	a	1041.29	1.06061	YES	YES
222	a	1042.14	0.89230	YES	YES
223	a	1042.23	1.07318	YES	YES
224	a	1058.49	1.95933	YES	YES
225	a	1060.04	0.51655	YES	YES
226	a	1060.18	1.32639	YES	YES
227	a	1061.64	1.14129	YES	YES
228	a	1063.73	2.86878	YES	YES

229	a	1065.96	0.29544	YES	YES
230	a	1066.76	0.17275	YES	YES
231	a	1069.37	0.01897	YES	YES
232	a	1071.44	0.02377	YES	YES
233	a	1071.70	0.25371	YES	YES
234	a	1072.53	0.14803	YES	YES
235	a	1072.71	0.17808	YES	YES
236	a	1073.32	0.04482	YES	YES
237	a	1073.92	0.37273	YES	YES
238	a	1076.48	0.06355	YES	YES
239	a	1076.77	0.38491	YES	YES
240	a	1079.28	3.17240	YES	YES
241	a	1084.94	0.84939	YES	YES
242	a	1088.75	5.92608	YES	YES
243	a	1092.51	12.26678	YES	YES
244	a	1094.65	13.74165	YES	YES
245	a	1097.23	3.42463	YES	YES
246	a	1098.11	49.44453	YES	YES
247	a	1099.65	4.11219	YES	YES
248	a	1102.45	0.42367	YES	YES
249	a	1104.84	6.60302	YES	YES
250	a	1107.15	1.47420	YES	YES
251	a	1107.93	18.76234	YES	YES
252	a	1113.01	24.89204	YES	YES
253	a	1128.91	3.92788	YES	YES
254	a	1131.53	1.73191	YES	YES
255	a	1160.02	2.89225	YES	YES
256	a	1161.26	8.21645	YES	YES
257	a	1161.83	6.68405	YES	YES
258	a	1162.75	4.93860	YES	YES
259	a	1170.36	0.32144	YES	YES
260	a	1170.69	20.51722	YES	YES
261	a	1170.89	4.51218	YES	YES
262	a	1172.03	5.86122	YES	YES
263	a	1179.14	26.94920	YES	YES
264	a	1180.35	1.77379	YES	YES
265	a	1182.96	2.70784	YES	YES
266	a	1183.93	10.86663	YES	YES
267	a	1192.07	6.75036	YES	YES
268	a	1192.76	1.05531	YES	YES
269	a	1194.46	0.39825	YES	YES
270	a	1195.30	1.83866	YES	YES
271	a	1199.07	1.26874	YES	YES
272	a	1235.55	0.70349	YES	YES
273	a	1241.18	0.05537	YES	YES
274	a	1243.67	0.03092	YES	YES
275	a	1245.13	1.14838	YES	YES
276	a	1245.93	0.44834	YES	YES
277	a	1246.35	0.18715	YES	YES
278	a	1246.93	0.15312	YES	YES
279	a	1247.36	1.24365	YES	YES
280	a	1248.47	0.21429	YES	YES
281	a	1249.11	0.03400	YES	YES
282	a	1251.22	0.45378	YES	YES
283	a	1253.09	3.70085	YES	YES
284	a	1254.90	5.10305	YES	YES
285	a	1255.43	2.56414	YES	YES
286	a	1256.93	5.76287	YES	YES

287	a	1257.64	5.67568	YES	YES
288	a	1258.07	5.05875	YES	YES
289	a	1258.24	10.22663	YES	YES
290	a	1258.68	4.02350	YES	YES
291	a	1259.58	0.28015	YES	YES
292	a	1259.96	0.33508	YES	YES
293	a	1260.15	2.30873	YES	YES
294	a	1260.47	6.43389	YES	YES
295	a	1260.93	0.50797	YES	YES
296	a	1263.46	0.32688	YES	YES
297	a	1266.96	1.10827	YES	YES
298	a	1268.12	0.16737	YES	YES
299	a	1268.65	1.20227	YES	YES
300	a	1268.87	2.30941	YES	YES
301	a	1275.49	1.51740	YES	YES
302	a	1282.16	2.30283	YES	YES
303	a	1283.66	5.29784	YES	YES
304	a	1283.99	1.70446	YES	YES
305	a	1285.22	2.74574	YES	YES
306	a	1286.39	0.49492	YES	YES
307	a	1289.89	5.25118	YES	YES
308	a	1290.80	0.29949	YES	YES
309	a	1291.22	0.77459	YES	YES
310	a	1292.16	5.59719	YES	YES
311	a	1308.38	5.14179	YES	YES
312	a	1309.07	0.95013	YES	YES
313	a	1310.95	0.67152	YES	YES
314	a	1312.54	0.90739	YES	YES
315	a	1313.00	1.43083	YES	YES
316	a	1313.83	1.02800	YES	YES
317	a	1314.72	1.92801	YES	YES
318	a	1317.68	2.13395	YES	YES
319	a	1320.62	0.35155	YES	YES
320	a	1321.07	2.92140	YES	YES
321	a	1322.29	1.73501	YES	YES
322	a	1322.67	3.50645	YES	YES
323	a	1323.03	1.06288	YES	YES
324	a	1323.50	1.10834	YES	YES
325	a	1323.85	1.71236	YES	YES
326	a	1324.13	0.84935	YES	YES
327	a	1324.80	0.97956	YES	YES
328	a	1325.18	0.27703	YES	YES
329	a	1325.80	0.64284	YES	YES
330	a	1325.96	0.40845	YES	YES
331	a	1326.84	3.21584	YES	YES
332	a	1327.59	4.97924	YES	YES
333	a	1327.81	1.83274	YES	YES
334	a	1329.13	3.75031	YES	YES
335	a	1332.34	1.16547	YES	YES
336	a	1335.82	0.07753	YES	YES
337	a	1336.67	0.37276	YES	YES
338	a	1337.54	0.55455	YES	YES
339	a	1337.80	0.16176	YES	YES
340	a	1338.58	0.45108	YES	YES
341	a	1338.79	1.21715	YES	YES
342	a	1339.24	0.04462	YES	YES
343	a	1339.66	0.64189	YES	YES
344	a	1342.76	4.57936	YES	YES

345	a	1345.86	2.79261	YES	YES
346	a	1346.04	0.69993	YES	YES
347	a	1347.53	2.54583	YES	YES
348	a	1347.73	1.32148	YES	YES
349	a	1348.33	0.91257	YES	YES
350	a	1348.92	0.38095	YES	YES
351	a	1349.60	1.36045	YES	YES
352	a	1350.97	0.71346	YES	YES
353	a	1370.87	4.40343	YES	YES
354	a	1374.56	23.13883	YES	YES
355	a	1401.61	7.66530	YES	YES
356	a	1402.04	7.20561	YES	YES
357	a	1406.29	8.98139	YES	YES
358	a	1406.55	6.81786	YES	YES
359	a	1420.20	4.87199	YES	YES
360	a	1423.66	3.44558	YES	YES
361	a	1424.95	3.30754	YES	YES
362	a	1425.90	3.42108	YES	YES
363	a	1427.91	2.56979	YES	YES
364	a	1429.61	3.70809	YES	YES
365	a	1431.18	9.03151	YES	YES
366	a	1432.80	2.87705	YES	YES
367	a	1433.55	6.31957	YES	YES
368	a	1434.68	6.09806	YES	YES
369	a	1435.38	3.52575	YES	YES
370	a	1435.89	1.07121	YES	YES
371	a	1436.85	2.03710	YES	YES
372	a	1436.93	6.67144	YES	YES
373	a	1437.51	2.92460	YES	YES
374	a	1437.85	3.87562	YES	YES
375	a	1437.91	4.12006	YES	YES
376	a	1438.09	5.38274	YES	YES
377	a	1438.73	0.39111	YES	YES
378	a	1438.79	3.83552	YES	YES
379	a	1439.29	4.52389	YES	YES
380	a	1440.19	4.69147	YES	YES
381	a	1440.33	14.96685	YES	YES
382	a	1440.77	5.87333	YES	YES
383	a	1441.16	5.18992	YES	YES
384	a	1441.32	11.75279	YES	YES
385	a	1443.00	14.40758	YES	YES
386	a	1443.18	8.74594	YES	YES
387	a	1444.09	28.94602	YES	YES
388	a	1444.71	3.04568	YES	YES
389	a	1445.03	12.08303	YES	YES
390	a	1446.36	7.67701	YES	YES
391	a	1446.62	10.67950	YES	YES
392	a	1448.15	16.95385	YES	YES
393	a	1451.21	7.43093	YES	YES
394	a	1452.20	3.73014	YES	YES
395	a	1453.13	0.46800	YES	YES
396	a	1453.55	3.13722	YES	YES
397	a	1454.17	8.70618	YES	YES
398	a	1455.13	0.80230	YES	YES
399	a	1456.23	2.07228	YES	YES
400	a	1456.82	1.05692	YES	YES
401	a	1458.15	0.77180	YES	YES
402	a	1458.73	1.13354	YES	YES

403	a	1463.61	10.95688	YES	YES
404	a	1865.87	122.50089	YES	YES
405	a	2868.57	53.88000	YES	YES
406	a	2921.46	19.29032	YES	YES
407	a	2924.68	2.16374	YES	YES
408	a	2928.81	23.98330	YES	YES
409	a	2928.98	1.94137	YES	YES
410	a	2931.19	13.77813	YES	YES
411	a	2933.78	11.49430	YES	YES
412	a	2937.19	13.13193	YES	YES
413	a	2940.06	3.94079	YES	YES
414	a	2940.30	1.36994	YES	YES
415	a	2940.37	5.12469	YES	YES
416	a	2940.48	7.88931	YES	YES
417	a	2941.97	7.01718	YES	YES
418	a	2942.01	11.80053	YES	YES
419	a	2943.90	7.36478	YES	YES
420	a	2944.29	7.38998	YES	YES
421	a	2944.70	11.67057	YES	YES
422	a	2944.91	8.78037	YES	YES
423	a	2946.17	8.11662	YES	YES
424	a	2947.33	21.53460	YES	YES
425	a	2947.65	31.16288	YES	YES
426	a	2948.00	9.58816	YES	YES
427	a	2948.29	12.64638	YES	YES
428	a	2948.51	11.19485	YES	YES
429	a	2948.71	5.11070	YES	YES
430	a	2951.11	6.50043	YES	YES
431	a	2951.13	11.38367	YES	YES
432	a	2952.64	10.38208	YES	YES
433	a	2954.25	15.58048	YES	YES
434	a	2954.34	14.46757	YES	YES
435	a	2955.65	3.02232	YES	YES
436	a	2956.15	5.50400	YES	YES
437	a	2956.75	8.24517	YES	YES
438	a	2957.05	3.76634	YES	YES
439	a	2957.92	3.74353	YES	YES
440	a	2958.20	9.81240	YES	YES
441	a	2958.55	4.35613	YES	YES
442	a	2959.22	4.80906	YES	YES
443	a	2959.69	12.41252	YES	YES
444	a	2959.82	4.93073	YES	YES
445	a	2960.45	8.14426	YES	YES
446	a	2961.92	39.02368	YES	YES
447	a	2962.21	59.44169	YES	YES
448	a	2962.55	74.95838	YES	YES
449	a	2962.66	32.71708	YES	YES
450	a	2963.36	22.92574	YES	YES
451	a	2963.74	36.17072	YES	YES
452	a	2964.84	0.92431	YES	YES
453	a	2964.86	12.91722	YES	YES
454	a	2964.89	53.73350	YES	YES
455	a	2970.93	20.35036	YES	YES
456	a	2973.09	19.05981	YES	YES
457	a	2975.38	15.54873	YES	YES
458	a	2982.20	4.07889	YES	YES
459	a	2982.73	19.19970	YES	YES
460	a	2985.73	6.94686	YES	YES

461	a	2988.44	11.04750	YES	YES
462	a	2989.07	49.82076	YES	YES
463	a	2989.85	28.84831	YES	YES
464	a	2990.45	4.68081	YES	YES
465	a	2992.10	15.76521	YES	YES
466	a	2992.45	23.71587	YES	YES
467	a	2996.43	42.33856	YES	YES
468	a	3003.54	15.56252	YES	YES
469	a	3006.74	15.18632	YES	YES
470	a	3007.41	27.00976	YES	YES
471	a	3007.81	7.00336	YES	YES
472	a	3007.94	32.90393	YES	YES
473	a	3008.54	18.66651	YES	YES
474	a	3009.18	13.39872	YES	YES
475	a	3009.46	26.71916	YES	YES
476	a	3010.14	9.72042	YES	YES
477	a	3010.38	24.57941	YES	YES
478	a	3010.80	29.03852	YES	YES
479	a	3011.24	22.38524	YES	YES
480	a	3011.50	18.41253	YES	YES
481	a	3011.95	20.78984	YES	YES
482	a	3012.77	21.19134	YES	YES
483	a	3012.95	4.28746	YES	YES
484	a	3013.55	44.41612	YES	YES
485	a	3013.79	12.79268	YES	YES
486	a	3014.69	32.57277	YES	YES
487	a	3014.96	31.24617	YES	YES
488	a	3015.72	20.62466	YES	YES
489	a	3016.22	41.22999	YES	YES
490	a	3016.38	41.62667	YES	YES
491	a	3016.38	6.36714	YES	YES
492	a	3016.59	24.33022	YES	YES
493	a	3016.89	6.47615	YES	YES
494	a	3017.61	23.45630	YES	YES
495	a	3017.82	24.30295	YES	YES
496	a	3018.00	13.62918	YES	YES
497	a	3018.13	29.40637	YES	YES
498	a	3018.45	15.97433	YES	YES
499	a	3018.73	22.41265	YES	YES
500	a	3020.13	35.02180	YES	YES
501	a	3020.93	29.44446	YES	YES
502	a	3021.09	29.72766	YES	YES
503	a	3027.34	19.27462	YES	YES
504	a	3027.62	36.12253	YES	YES
505	a	3028.71	0.41250	YES	YES
506	a	3032.87	0.38376	YES	YES
507	a	3045.44	1.20848	YES	YES
508	a	3046.31	22.20679	YES	YES
509	a	3051.88	0.12270	YES	YES
510	a	3473.48	2.39057	YES	YES

$end

Double hybrid single point energy = -7462.609810716432 H
COSMO enery + OC correction = -7470.2746861071 H (in *o*DFB)

6.2.20 HOMe

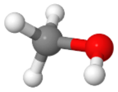

```
Method: (RI-)BP86(D3BJ)/def2-TZVPP
Symmetry: cs

Cartesian coordinates in Ångström:
C    -0.3322226    0.1433998    0.0000000
O     1.0961566    0.1316259    0.0000000
H    -0.7564967   -0.3393027    0.8971508
H    -0.7564967   -0.3393027   -0.8971508
H    -0.6380192    1.1962799    0.0000000
H     1.3870785   -0.7927002    0.0000000

SCF energy GEOOPT =  -115.7836368041 H
ZPE =   130.4 kJ/mol
FREEH energy =   139.26 kJ/mol
FREEH entropy =   0.23919 kJ/mol/K

$vibrational spectrum
#  mode     symmetry     wave number   IR intensity    selection rules
#                         cm**(-1)        km/mol         IR      RAMAN
     1                       -0.00         0.00000        -        -
     2                        0.00         0.00000        -        -
     3                        0.00         0.00000        -        -
     4                        0.00         0.00000        -        -
     5                        0.00         0.00000        -        -
     6                        0.00         0.00000        -        -
     7        a"            292.69       108.23880       YES      YES
     8        a'           1008.65       114.53489       YES      YES
     9        a'           1052.66         0.49233       YES      YES
    10        a"           1130.93         0.15557       YES      YES
    11        a'           1335.47        21.72847       YES      YES
    12        a'           1429.72         4.03162       YES      YES
    13        a"           1444.98         1.85912       YES      YES
    14        a'           1461.40         3.88303       YES      YES
    15        a'           2914.46        65.88322       YES      YES
    16        a"           2960.91        64.43070       YES      YES
    17        a'           3042.99        26.49602       YES      YES
    18        a'           3724.49        19.80111       YES      YES
$end

Double hybrid single point energy = -115.598907709007 H
COSMO energy + OC correction = -115.7907813795 H (in oDFB)
```

6.2.21 [{Ga(dcpe)}$_2$(HOMe)]$^{2+}$

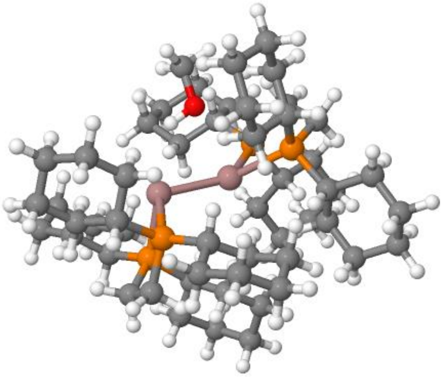

Method: (RI-)BP86(D3BJ)/def2-TZVPP
Symmetry: c1

Cartesian coordinates in Ångström:

Ga	-1.8119855	17.4103192	-2.1630161
Ga	-4.2436345	17.3728123	-1.5945255
P	-1.5290345	18.9837592	-4.0637196
P	-5.9971214	19.0173815	-1.0124205
P	-1.7108377	15.7323112	-3.9584634
P	-5.4821849	15.9715273	-0.0045377
C	-5.0516407	20.9789514	-5.1882256
H	-5.1920936	21.6071693	-4.2911890
H	-6.0411899	20.5764885	-5.4479432
C	-0.6125365	14.3247353	-3.4883585
H	-0.4275565	13.7314807	-4.3997147
C	-4.4645193	14.9601372	1.1512123
H	-4.1058610	15.7201128	1.8659821
C	-4.2057120	16.0520236	-5.2145283
H	-3.7393128	16.4520898	-6.1294472
H	-4.3243075	16.8946394	-4.5163122
C	-7.0164667	19.3428217	-2.5128393
H	-6.3596414	19.9540568	-3.1525289
C	-7.1392749	18.2198505	0.2110051
H	-7.5942311	18.9861143	0.8524465
H	-7.9553884	17.7644772	-0.3672143
C	-3.1138803	22.3733920	-6.0021287
H	-2.7198029	22.9670441	-6.8378898
H	-3.1648051	23.0503713	-5.1333686
C	-2.1450924	21.2254401	-5.6876755
H	-1.1584854	21.6332634	-5.4316486
H	-2.0123450	20.6065467	-6.5895735
C	-4.0940745	19.8272099	-4.8583204
H	-4.0518985	19.1453129	-5.7225514
H	-4.4646876	19.2275874	-4.0111860
C	1.6183272	13.6748803	-2.4715615
H	1.8936070	13.0545339	-3.3398531
H	2.5567196	14.0738229	-2.0629261
C	-7.9658170	14.5144221	-0.1479962
H	-8.4526535	15.3611405	0.3568554
H	-7.6575937	13.8116643	0.6385467
C	-5.4275139	20.6539281	-0.3815072
H	-6.2554323	21.3604413	-0.5638130
C	-0.0816977	20.7295752	-2.4732603
H	-0.8384217	21.5033177	-2.6690066

H	-0.4560928	20.1223201	-1.6294426
C	1.2469979	21.3855821	-2.0783913
H	1.5618296	22.0752621	-2.8783060
H	1.0999592	21.9980780	-1.1780664
C	-7.3151629	18.0280756	-3.2530974
H	-6.3771317	17.4828862	-3.4587235
H	-7.9265428	17.3769185	-2.6055854
C	-4.5149512	21.8511480	-6.3268003
H	-4.4790416	21.2559575	-7.2540471
H	-5.2002680	22.6873916	-6.5192328
C	-8.3050523	20.1365911	-2.2431627
H	-8.0786084	21.0890036	-1.7439640
H	-8.9550077	19.5648628	-1.5610462
C	-4.2075443	21.0845541	-1.2173296
H	-4.4738424	21.1179067	-2.2834737
H	-3.4179881	20.3211887	-1.1057800
C	-3.0942226	13.8113674	-5.5666870
H	-2.4464404	13.0405702	-5.1289784
H	-2.5954674	14.1647546	-6.4838688
C	2.5175889	19.4517406	-3.0831903
H	2.8816210	20.0626865	-3.9252446
H	3.2807831	18.6823509	-2.9025408
C	-4.5633746	22.0125052	1.5629121
H	-5.3555722	22.7708148	1.4541221
H	-4.3120610	21.9763923	2.6316740
C	-9.3589923	19.0897695	-4.2987711
H	-9.8684089	19.3013188	-5.2482450
H	-10.0580723	18.4834445	-3.6991921
C	-5.2239968	13.8830115	1.9447084
H	-6.0815788	14.3204435	2.4749662
H	-5.6253648	13.1301798	1.2495156
C	0.9027479	12.8085806	-1.4313041
H	1.5415368	11.9670709	-1.1320304
H	0.7242296	13.4069657	-0.5214629
C	-8.0822781	18.2819977	-4.5550935
H	-7.4304020	18.8307394	-5.2538268
H	-8.3212723	17.3232779	-5.0356832
C	2.3405387	20.3390921	-1.8482417
H	2.0700851	19.7110272	-0.9827114
H	3.2893032	20.8302633	-1.5946368
C	-3.3418777	22.4288613	0.7367143
H	-2.9857934	23.4165828	1.0579553
H	-2.5169199	21.7203584	0.9211542
C	-6.0675874	13.8285859	-1.7029245
H	-5.6723770	13.0941585	-0.9850112
H	-5.2062061	14.2022321	-2.2793720
C	-5.1033024	20.6457913	1.1194992
H	-4.3435406	19.8750053	1.3085881
H	-5.9958551	20.3873728	1.7069134
C	-6.7348492	14.9868467	-0.9386040
H	-7.0874515	15.7071322	-1.6998894
C	-3.6682113	22.4430498	-0.7595005
C	-8.3118072	12.6680044	-1.8522175
H	-9.0366237	12.2094079	-2.5378273
H	-8.0159772	11.8809719	-1.1394067
C	-8.9639753	13.8252060	-1.0891361
H	-9.8269927	13.4685194	-0.5111511
H	-9.3528299	14.5656015	-1.8085131

C	-1.3248017	13.4503050	-2.4427483
H	-2.2727533	13.0594865	-2.8394292
H	-1.5780954	14.0881126	-1.5810051
C	-4.2786010	13.1927438	2.9383203
H	-3.9505818	13.9248792	3.6944371
H	-4.8266356	12.4097063	3.4794231
C	-5.5670948	15.4495711	-5.5731252
H	-6.2042381	16.2177546	-6.0336251
H	-6.0720536	15.1353038	-4.6437725
C	-3.0542920	12.6026199	2.2313846
H	-3.3789585	11.7993271	1.5491680
H	-2.3821726	12.1360141	2.9636679
C	-3.2326101	14.3798726	0.4376652
H	-3.5575455	13.6630596	-0.3317195
H	-2.6946420	15.1869593	-0.0844195
C	-9.0568983	20.3954238	-3.5567464
H	-9.9861061	20.9414018	-3.3460395
H	-8.4458633	21.0521108	-4.1971699
C	-4.4574984	13.2013324	-5.9277891
H	-4.3153337	12.3746194	-6.6366774
H	-4.8995843	12.7612632	-5.0182538
C	-7.0776686	13.1401813	-2.6273233
H	-6.5954125	12.2958992	-3.1395901
H	-7.3888066	13.8490952	-3.4129401
C	-5.4164456	14.2452171	-6.5079477
H	-6.3965810	13.7908237	-6.7067213
H	-5.0316616	14.5911452	-7.4812091
C	-0.4345173	12.2952969	-1.9711914
H	-0.9653694	11.7121852	-1.2050405
H	-0.2530730	11.6090696	-2.8140957
C	-2.3058712	13.6740862	1.4324446
H	-1.8849239	14.4211631	2.1259200
H	-1.4535369	13.2342036	0.8954139
H	-2.2208677	17.8683061	0.1342929
C	0.0822109	19.8152882	-3.7009254
H	0.3411709	20.4289684	-4.5792511
C	1.1987258	18.7793366	-3.4825395
H	1.3464588	18.1802807	-4.3927140
H	0.8895575	18.0818437	-2.6829230
C	0.7321576	14.8358361	-2.9401896
H	1.2650050	15.4280342	-3.6973462
H	0.5328669	15.5122896	-2.0903671
C	-0.8637590	16.5464892	-5.3879966
H	0.2093096	16.4897006	-5.1662194
H	-1.0316077	15.9478382	-6.2943474
C	-1.2814628	18.0059826	-5.6206571
H	-2.2420387	18.0448106	-6.1502350
H	-0.5444486	18.5154134	-6.2572134
C	-3.2837648	14.9896795	-4.5996958
H	-3.7703341	14.6151396	-3.6812440
C	-2.6867230	20.3519751	-4.5413410
H	-2.7521901	20.9729457	-3.6328034
C	-6.4200486	17.1582981	1.0535687
H	-7.1320782	16.6206344	1.6949690
H	-5.6749405	17.6272903	1.7101121
H	-4.4177150	23.2235102	-0.9675410
H	-2.7761150	22.7016091	-1.3483566
O	-3.0294685	18.0068265	0.6887852

```
C    -2.6784068   17.9531993    2.0845794
H    -2.2056620   16.9948012    2.3431264
H    -3.6029612   18.0619846    2.6633262
H    -2.0017904   18.7768018    2.3510021

SCF energy GEOOPT =  -7371.970295588 H
ZPE =   3730. kJ/mol
FREEH energy =  3918.92 kJ/mol
FREEH entropy =   1.62593 kJ/mol/K

$vibrational spectrum
#  mode     symmetry     wave number   IR intensity    selection rules
#                         cm**(-1)       km/mol          IR     RAMAN
     1                      -0.00         0.00000         -       -
     2                      -0.00         0.00000         -       -
     3                      -0.00         0.00000         -       -
     4                       0.00         0.00000         -       -
     5                       0.00         0.00000         -       -
     6                       0.00         0.00000         -       -
     7         a             6.25         1.53960        YES     YES
     8         a            15.26         0.34833        YES     YES
     9         a            19.52         0.64436        YES     YES
    10         a            23.31         0.17285        YES     YES
    11         a            31.79         0.06641        YES     YES
    12         a            33.49         0.08692        YES     YES
    13         a            34.55         0.18631        YES     YES
    14         a            36.98         0.04047        YES     YES
    15         a            39.94         0.03587        YES     YES
    16         a            40.90         0.34395        YES     YES
    17         a            43.20         0.34500        YES     YES
    18         a            44.72         0.96493        YES     YES
    19         a            46.95         0.34157        YES     YES
    20         a            49.83         0.23032        YES     YES
    21         a            50.64         0.17764        YES     YES
    22         a            54.19         0.04556        YES     YES
    23         a            56.37         0.07736        YES     YES
    24         a            58.11         0.14972        YES     YES
    25         a            60.20         0.22543        YES     YES
    26         a            63.62         0.86252        YES     YES
    27         a            66.48         0.21892        YES     YES
    28         a            69.28         0.62718        YES     YES
    29         a            71.09         0.35062        YES     YES
    30         a            71.71         0.25229        YES     YES
    31         a            74.22         2.90120        YES     YES
    32         a            78.21         0.14690        YES     YES
    33         a            79.77         0.92765        YES     YES
    34         a            82.14         1.28135        YES     YES
    35         a            87.84         0.27066        YES     YES
    36         a            91.14         1.23426        YES     YES
    37         a            97.01         0.31757        YES     YES
    38         a           100.10         0.23767        YES     YES
    39         a           110.97         3.06116        YES     YES
    40         a           119.79         1.51391        YES     YES
    41         a           120.44         2.24276        YES     YES
    42         a           128.50         0.22078        YES     YES
    43         a           130.35         0.87992        YES     YES
    44         a           134.03         0.11439        YES     YES
    45         a           139.51         0.19662        YES     YES
```

46	a	140.56	1.07432	YES	YES
47	a	144.19	0.68493	YES	YES
48	a	151.12	0.89169	YES	YES
49	a	151.69	4.97875	YES	YES
50	a	157.02	0.77162	YES	YES
51	a	167.93	0.91369	YES	YES
52	a	177.62	0.64599	YES	YES
53	a	180.38	3.58522	YES	YES
54	a	186.48	1.13986	YES	YES
55	a	193.02	2.85323	YES	YES
56	a	197.81	2.77559	YES	YES
57	a	200.68	5.13366	YES	YES
58	a	211.43	0.13561	YES	YES
59	a	215.01	0.64650	YES	YES
60	a	217.75	0.11293	YES	YES
61	a	221.99	0.16656	YES	YES
62	a	223.22	0.14393	YES	YES
63	a	226.11	0.59392	YES	YES
64	a	227.36	0.24359	YES	YES
65	a	230.63	0.08722	YES	YES
66	a	231.82	0.15805	YES	YES
67	a	232.60	1.05696	YES	YES
68	a	239.69	0.45491	YES	YES
69	a	243.14	0.01031	YES	YES
70	a	244.78	1.70453	YES	YES
71	a	250.40	0.80219	YES	YES
72	a	254.42	2.21962	YES	YES
73	a	266.50	0.90740	YES	YES
74	a	279.22	0.69533	YES	YES
75	a	281.85	0.92617	YES	YES
76	a	287.49	0.42560	YES	YES
77	a	297.89	3.42716	YES	YES
78	a	304.86	0.47606	YES	YES
79	a	308.65	0.11220	YES	YES
80	a	313.98	1.28411	YES	YES
81	a	329.12	1.21500	YES	YES
82	a	329.51	0.11422	YES	YES
83	a	331.69	0.58309	YES	YES
84	a	336.87	2.31680	YES	YES
85	a	337.35	0.46159	YES	YES
86	a	338.72	1.61801	YES	YES
87	a	363.08	7.85183	YES	YES
88	a	377.53	0.20875	YES	YES
89	a	379.36	5.14416	YES	YES
90	a	381.64	0.81915	YES	YES
91	a	389.32	0.31724	YES	YES
92	a	393.12	3.04411	YES	YES
93	a	405.74	0.10218	YES	YES
94	a	416.12	1.04414	YES	YES
95	a	420.66	2.68434	YES	YES
96	a	422.12	4.55116	YES	YES
97	a	425.82	3.97612	YES	YES
98	a	429.00	1.06993	YES	YES
99	a	429.76	0.24454	YES	YES
100	a	430.95	0.07283	YES	YES
101	a	432.03	0.63816	YES	YES
102	a	432.26	0.14316	YES	YES
103	a	432.52	0.96756	YES	YES

104	a	433.44	0.59618	YES	YES
105	a	436.33	2.16542	YES	YES
106	a	436.56	0.70427	YES	YES
107	a	447.87	2.73532	YES	YES
108	a	456.43	5.30822	YES	YES
109	a	462.80	4.21487	YES	YES
110	a	468.34	3.94310	YES	YES
111	a	490.76	5.67951	YES	YES
112	a	492.00	3.26298	YES	YES
113	a	494.42	4.44574	YES	YES
114	a	494.87	0.36001	YES	YES
115	a	501.74	0.76781	YES	YES
116	a	502.66	1.53895	YES	YES
117	a	509.11	8.92466	YES	YES
118	a	513.15	9.21575	YES	YES
119	a	532.36	16.25014	YES	YES
120	a	629.46	13.79927	YES	YES
121	a	630.30	24.33945	YES	YES
122	a	641.26	11.00818	YES	YES
123	a	660.58	12.56488	YES	YES
124	a	699.80	1.94537	YES	YES
125	a	714.32	1.21510	YES	YES
126	a	719.05	1.89873	YES	YES
127	a	728.59	8.78978	YES	YES
128	a	731.53	3.68670	YES	YES
129	a	733.62	4.00887	YES	YES
130	a	735.25	10.11832	YES	YES
131	a	740.07	4.57649	YES	YES
132	a	771.41	16.96430	YES	YES
133	a	773.05	0.10569	YES	YES
134	a	773.09	0.17235	YES	YES
135	a	773.78	5.00919	YES	YES
136	a	774.12	0.55089	YES	YES
137	a	774.84	0.18401	YES	YES
138	a	775.66	0.06032	YES	YES
139	a	775.83	0.65907	YES	YES
140	a	777.42	0.27469	YES	YES
141	a	779.49	20.47598	YES	YES
142	a	806.15	0.57435	YES	YES
143	a	808.05	1.52303	YES	YES
144	a	810.11	11.76834	YES	YES
145	a	810.50	5.55919	YES	YES
146	a	811.92	3.63403	YES	YES
147	a	812.94	3.78128	YES	YES
148	a	813.26	0.62075	YES	YES
149	a	817.05	2.91999	YES	YES
150	a	831.95	7.24674	YES	YES
151	a	832.10	1.67029	YES	YES
152	a	836.90	2.42083	YES	YES
153	a	837.50	3.12527	YES	YES
154	a	837.93	2.19095	YES	YES
155	a	838.27	0.42606	YES	YES
156	a	839.00	4.37873	YES	YES
157	a	840.02	10.52088	YES	YES
158	a	847.39	26.31287	YES	YES
159	a	849.56	15.15883	YES	YES
160	a	873.67	4.87200	YES	YES
161	a	874.26	2.10008	YES	YES

162	a	874.63	1.82371	YES	YES
163	a	876.50	1.83477	YES	YES
164	a	876.96	2.07686	YES	YES
165	a	877.18	3.04762	YES	YES
166	a	877.94	3.37976	YES	YES
167	a	878.40	1.45225	YES	YES
168	a	879.12	3.68947	YES	YES
169	a	879.89	0.27201	YES	YES
170	a	880.15	0.68783	YES	YES
171	a	881.41	2.22495	YES	YES
172	a	881.94	1.26698	YES	YES
173	a	882.79	11.72797	YES	YES
174	a	883.39	2.58232	YES	YES
175	a	885.60	2.56710	YES	YES
176	a	902.73	2.75182	YES	YES
177	a	903.57	3.72397	YES	YES
178	a	904.93	2.62198	YES	YES
179	a	905.45	1.70353	YES	YES
180	a	907.10	1.58161	YES	YES
181	a	909.46	0.96052	YES	YES
182	a	910.08	0.69549	YES	YES
183	a	911.01	0.98651	YES	YES
184	a	974.91	123.00962	YES	YES
185	a	983.75	2.04422	YES	YES
186	a	985.73	2.60443	YES	YES
187	a	987.61	5.86262	YES	YES
188	a	988.20	7.20959	YES	YES
189	a	988.54	14.04278	YES	YES
190	a	989.31	17.73301	YES	YES
191	a	989.69	2.67082	YES	YES
192	a	990.85	23.75586	YES	YES
193	a	991.20	0.20106	YES	YES
194	a	994.96	0.75530	YES	YES
195	a	1013.72	0.57176	YES	YES
196	a	1014.90	3.44640	YES	YES
197	a	1015.60	0.43645	YES	YES
198	a	1017.16	1.06557	YES	YES
199	a	1017.49	0.46765	YES	YES
200	a	1018.54	0.94107	YES	YES
201	a	1020.98	0.55531	YES	YES
202	a	1021.65	0.30296	YES	YES
203	a	1030.11	0.97709	YES	YES
204	a	1030.70	0.88204	YES	YES
205	a	1032.88	1.95863	YES	YES
206	a	1035.84	0.71612	YES	YES
207	a	1037.10	1.81050	YES	YES
208	a	1037.65	2.83849	YES	YES
209	a	1038.29	1.51314	YES	YES
210	a	1040.92	1.87601	YES	YES
211	a	1056.46	2.63148	YES	YES
212	a	1057.30	1.98825	YES	YES
213	a	1057.95	2.05723	YES	YES
214	a	1059.46	2.04123	YES	YES
215	a	1060.86	1.91363	YES	YES
216	a	1063.31	1.50190	YES	YES
217	a	1065.25	2.31445	YES	YES
218	a	1067.41	2.00445	YES	YES
219	a	1070.21	0.42902	YES	YES

220	a	1070.88	0.83484	YES	YES
221	a	1071.16	0.88178	YES	YES
222	a	1071.73	0.03344	YES	YES
223	a	1072.10	8.95161	YES	YES
224	a	1072.60	11.33716	YES	YES
225	a	1072.75	0.08600	YES	YES
226	a	1074.06	0.28187	YES	YES
227	a	1074.53	0.09466	YES	YES
228	a	1075.37	3.39942	YES	YES
229	a	1080.63	3.67218	YES	YES
230	a	1089.64	5.44004	YES	YES
231	a	1090.07	1.52970	YES	YES
232	a	1092.41	4.62920	YES	YES
233	a	1094.22	8.51879	YES	YES
234	a	1100.65	1.30785	YES	YES
235	a	1104.08	1.61069	YES	YES
236	a	1109.12	6.49690	YES	YES
237	a	1112.91	12.30384	YES	YES
238	a	1118.62	5.94842	YES	YES
239	a	1121.14	0.53504	YES	YES
240	a	1130.34	2.04150	YES	YES
241	a	1158.55	6.02212	YES	YES
242	a	1159.90	4.41960	YES	YES
243	a	1161.46	15.76034	YES	YES
244	a	1165.96	7.83686	YES	YES
245	a	1166.98	11.68896	YES	YES
246	a	1167.71	6.05372	YES	YES
247	a	1168.80	10.89216	YES	YES
248	a	1170.19	4.47206	YES	YES
249	a	1175.09	4.65140	YES	YES
250	a	1177.19	16.96248	YES	YES
251	a	1178.18	3.13603	YES	YES
252	a	1179.44	4.86324	YES	YES
253	a	1187.00	1.25522	YES	YES
254	a	1190.00	0.56995	YES	YES
255	a	1191.11	1.46681	YES	YES
256	a	1209.43	1.67681	YES	YES
257	a	1232.61	0.87608	YES	YES
258	a	1236.93	0.88393	YES	YES
259	a	1244.00	0.58210	YES	YES
260	a	1244.99	1.45321	YES	YES
261	a	1245.90	1.20322	YES	YES
262	a	1246.77	0.34208	YES	YES
263	a	1246.94	0.19512	YES	YES
264	a	1247.76	0.48470	YES	YES
265	a	1249.50	0.15019	YES	YES
266	a	1250.14	0.11053	YES	YES
267	a	1250.52	1.22232	YES	YES
268	a	1253.55	1.29892	YES	YES
269	a	1253.74	1.78534	YES	YES
270	a	1254.35	2.75629	YES	YES
271	a	1255.23	7.15428	YES	YES
272	a	1255.68	4.20947	YES	YES
273	a	1256.93	0.79435	YES	YES
274	a	1257.46	2.21013	YES	YES
275	a	1258.30	5.12989	YES	YES
276	a	1259.09	0.80612	YES	YES
277	a	1259.79	0.85738	YES	YES

278	a	1261.07	0.51991	YES	YES
279	a	1261.67	3.15998	YES	YES
280	a	1262.36	1.86073	YES	YES
281	a	1264.67	1.06996	YES	YES
282	a	1266.48	0.89826	YES	YES
283	a	1267.44	2.52374	YES	YES
284	a	1269.25	1.29722	YES	YES
285	a	1281.16	4.57116	YES	YES
286	a	1282.01	0.56006	YES	YES
287	a	1283.01	0.77376	YES	YES
288	a	1284.53	2.70061	YES	YES
289	a	1284.69	6.03769	YES	YES
290	a	1287.60	3.67320	YES	YES
291	a	1289.89	3.23359	YES	YES
292	a	1291.60	4.98024	YES	YES
293	a	1306.11	0.72122	YES	YES
294	a	1306.81	0.96315	YES	YES
295	a	1308.99	1.20885	YES	YES
296	a	1310.36	0.58986	YES	YES
297	a	1311.77	2.60866	YES	YES
298	a	1314.20	1.09517	YES	YES
299	a	1315.13	1.78249	YES	YES
300	a	1315.87	0.31930	YES	YES
301	a	1319.18	5.10475	YES	YES
302	a	1319.81	2.94184	YES	YES
303	a	1321.29	1.64766	YES	YES
304	a	1321.88	11.95030	YES	YES
305	a	1322.08	19.01400	YES	YES
306	a	1322.35	2.80368	YES	YES
307	a	1322.87	17.97604	YES	YES
308	a	1323.58	1.52971	YES	YES
309	a	1324.51	0.42538	YES	YES
310	a	1324.53	4.08699	YES	YES
311	a	1324.82	2.33748	YES	YES
312	a	1325.17	0.36083	YES	YES
313	a	1326.19	17.51951	YES	YES
314	a	1327.39	4.08470	YES	YES
315	a	1328.13	1.35737	YES	YES
316	a	1328.72	17.60001	YES	YES
317	a	1330.04	8.62839	YES	YES
318	a	1335.90	0.38870	YES	YES
319	a	1336.12	0.42032	YES	YES
320	a	1336.55	0.69807	YES	YES
321	a	1337.05	0.24154	YES	YES
322	a	1337.34	0.35965	YES	YES
323	a	1337.84	0.21313	YES	YES
324	a	1338.30	0.00554	YES	YES
325	a	1338.79	0.58304	YES	YES
326	a	1343.54	0.83587	YES	YES
327	a	1343.82	0.91475	YES	YES
328	a	1344.34	2.95963	YES	YES
329	a	1345.68	0.41756	YES	YES
330	a	1346.26	0.86160	YES	YES
331	a	1347.24	0.80268	YES	YES
332	a	1348.28	2.95600	YES	YES
333	a	1349.59	1.31049	YES	YES
334	a	1400.99	5.78308	YES	YES
335	a	1404.01	9.16138	YES	YES

336	a	1405.38	6.08923	YES	YES
337	a	1407.30	8.05003	YES	YES
338	a	1419.75	0.77427	YES	YES
339	a	1423.67	5.90766	YES	YES
340	a	1425.49	4.52130	YES	YES
341	a	1427.47	6.80716	YES	YES
342	a	1428.36	1.78650	YES	YES
343	a	1429.34	0.95611	YES	YES
344	a	1430.84	1.08901	YES	YES
345	a	1432.64	1.26687	YES	YES
346	a	1433.48	0.82327	YES	YES
347	a	1434.14	3.00860	YES	YES
348	a	1434.73	2.11254	YES	YES
349	a	1435.47	3.64139	YES	YES
350	a	1435.94	1.67720	YES	YES
351	a	1436.54	8.73481	YES	YES
352	a	1436.62	7.21009	YES	YES
353	a	1437.92	5.20808	YES	YES
354	a	1437.96	0.83770	YES	YES
355	a	1438.22	5.84432	YES	YES
356	a	1438.61	3.07054	YES	YES
357	a	1438.84	15.11555	YES	YES
358	a	1439.17	0.56547	YES	YES
359	a	1439.24	2.84105	YES	YES
360	a	1439.60	10.27745	YES	YES
361	a	1439.83	2.05835	YES	YES
362	a	1441.02	2.88299	YES	YES
363	a	1441.68	0.50943	YES	YES
364	a	1442.05	6.17978	YES	YES
365	a	1443.00	34.28170	YES	YES
366	a	1443.20	3.88323	YES	YES
367	a	1443.73	17.81368	YES	YES
368	a	1444.35	4.11826	YES	YES
369	a	1444.91	19.64006	YES	YES
370	a	1446.04	3.90139	YES	YES
371	a	1447.09	8.29684	YES	YES
372	a	1452.50	0.07532	YES	YES
373	a	1453.43	3.57501	YES	YES
374	a	1453.62	4.03260	YES	YES
375	a	1454.35	0.34997	YES	YES
376	a	1454.56	2.14250	YES	YES
377	a	1455.07	2.27555	YES	YES
378	a	1455.99	1.84550	YES	YES
379	a	1457.78	0.63305	YES	YES
380	a	1462.65	5.67388	YES	YES
381	a	2911.80	27.96639	YES	YES
382	a	2914.27	57.25676	YES	YES
383	a	2919.56	18.47430	YES	YES
384	a	2920.64	0.68475	YES	YES
385	a	2925.16	12.25340	YES	YES
386	a	2926.41	5.34982	YES	YES
387	a	2929.58	5.60501	YES	YES
388	a	2931.22	21.99346	YES	YES
389	a	2938.43	14.32021	YES	YES
390	a	2938.69	26.48850	YES	YES
391	a	2939.76	22.22796	YES	YES
392	a	2941.57	18.17863	YES	YES
393	a	2941.77	5.14619	YES	YES

394	a	2942.66	10.45755	YES	YES
395	a	2942.71	3.38795	YES	YES
396	a	2943.31	8.99932	YES	YES
397	a	2943.76	10.96942	YES	YES
398	a	2944.78	6.08522	YES	YES
399	a	2945.27	2.44186	YES	YES
400	a	2945.64	11.20805	YES	YES
401	a	2946.45	9.43417	YES	YES
402	a	2946.68	8.81524	YES	YES
403	a	2948.48	4.10737	YES	YES
404	a	2948.92	10.91657	YES	YES
405	a	2949.08	8.68776	YES	YES
406	a	2949.27	20.99628	YES	YES
407	a	2951.19	11.32572	YES	YES
408	a	2951.71	16.24649	YES	YES
409	a	2952.24	3.76174	YES	YES
410	a	2954.31	37.64141	YES	YES
411	a	2955.21	4.16885	YES	YES
412	a	2955.78	4.99838	YES	YES
413	a	2955.83	10.12128	YES	YES
414	a	2956.39	6.96326	YES	YES
415	a	2957.47	5.90783	YES	YES
416	a	2958.46	23.39598	YES	YES
417	a	2958.80	23.64967	YES	YES
418	a	2959.50	26.87666	YES	YES
419	a	2959.68	43.94515	YES	YES
420	a	2959.87	32.30996	YES	YES
421	a	2960.20	38.44115	YES	YES
422	a	2960.68	25.68580	YES	YES
423	a	2960.78	10.83420	YES	YES
424	a	2962.73	31.16430	YES	YES
425	a	2964.34	25.18479	YES	YES
426	a	2964.46	27.21234	YES	YES
427	a	2965.87	19.74209	YES	YES
428	a	2974.49	11.01509	YES	YES
429	a	2975.73	16.30828	YES	YES
430	a	2977.91	12.18207	YES	YES
431	a	2981.68	13.50270	YES	YES
432	a	2983.00	25.08076	YES	YES
433	a	2985.12	6.12918	YES	YES
434	a	2985.56	16.38886	YES	YES
435	a	2985.81	3.98719	YES	YES
436	a	2987.97	6.69916	YES	YES
437	a	2991.15	34.40746	YES	YES
438	a	2992.37	9.97127	YES	YES
439	a	2993.49	23.79679	YES	YES
440	a	2994.92	28.78164	YES	YES
441	a	2998.38	22.05057	YES	YES
442	a	2998.97	44.84770	YES	YES
443	a	2999.72	22.07762	YES	YES
444	a	3000.74	19.56322	YES	YES
445	a	3002.60	18.20167	YES	YES
446	a	3003.52	34.36819	YES	YES
447	a	3005.81	12.63885	YES	YES
448	a	3007.19	16.06485	YES	YES
449	a	3007.45	31.89802	YES	YES
450	a	3007.55	6.42729	YES	YES
451	a	3008.92	18.81915	YES	YES

452	a	3009.59	23.83447	YES	YES
453	a	3010.21	23.39358	YES	YES
454	a	3011.25	18.32836	YES	YES
455	a	3012.37	17.35961	YES	YES
456	a	3012.99	32.12803	YES	YES
457	a	3013.26	15.55026	YES	YES
458	a	3013.79	16.05229	YES	YES
459	a	3015.49	26.37769	YES	YES
460	a	3015.79	28.75807	YES	YES
461	a	3016.33	17.65614	YES	YES
462	a	3016.67	13.05374	YES	YES
463	a	3016.97	56.04565	YES	YES
464	a	3017.06	14.64269	YES	YES
465	a	3017.47	38.10863	YES	YES
466	a	3017.63	21.56489	YES	YES
467	a	3018.16	34.63575	YES	YES
468	a	3018.34	22.02954	YES	YES
469	a	3018.61	18.12990	YES	YES
470	a	3018.75	25.18441	YES	YES
471	a	3019.43	22.38270	YES	YES
472	a	3022.78	31.73705	YES	YES
473	a	3023.33	14.40830	YES	YES
474	a	3028.11	0.78431	YES	YES
475	a	3029.92	1.90293	YES	YES
476	a	3030.78	7.96827	YES	YES
477	a	3039.33	0.79364	YES	YES
478	a	3044.02	1.01348	YES	YES
479	a	3052.34	12.76965	YES	YES
480	a	3288.70	487.20134	YES	YES

$end

Double hybrid single point energy = -7364.660701520127 H
COSMO energy + OC correction = -7372.1170406266 H (in *o*DFB)

6.2.22 [{Ga(dcpe)}$_2$(H–OMe)]$^{2+}$ (Transition State)

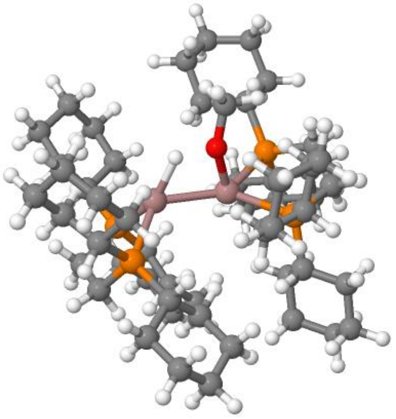

Method: (RI-)BP86(D3BJ)/def2-TZVPP
Symmetry: c1

Cartesian coordinates in Ångström:

Ga	-1.9641607	17.3490663	-2.4150484
Ga	-4.1368328	17.4807333	-1.2484970
P	-1.5266385	18.9597120	-4.1514037
P	-5.9796118	19.0243290	-0.8948943

P	-1.7395523	15.6889084	-4.1003547
P	-5.4158143	15.9939341	0.1729033
C	-5.0698684	20.9411338	-5.2145810
H	-5.2108249	21.5454305	-4.3015593
H	-6.0574655	20.5374195	-5.4789288
C	-0.7031874	14.2724543	-3.5428056
H	-0.5071504	13.6295474	-4.4168548
C	-4.4473090	14.9679248	1.3575942
H	-4.1163338	15.7145713	2.1007651
C	-4.2162174	16.1086780	-5.3339683
H	-3.7269013	16.5483399	-6.2180188
H	-4.3363767	16.9134849	-4.5941356
C	-6.9604940	19.2970814	-2.4317054
H	-6.2875432	19.8924989	-3.0699057
C	-7.1368568	18.2101896	0.3078029
H	-7.6149788	18.9742388	0.9349516
H	-7.9348414	17.7458800	-0.2881931
C	-3.1470835	22.3729293	-6.0011587
H	-2.7610778	22.9893487	-6.8240139
H	-3.2018445	23.0290913	-5.1168539
C	-2.1665482	21.2275020	-5.7155469
H	-1.1830246	21.6372255	-5.4498582
H	-2.0288148	20.6316990	-6.6324250
C	-4.1031459	19.7887026	-4.9152154
H	-4.0580825	19.1281405	-5.7957429
H	-4.4647565	19.1700365	-4.0789319
C	1.4519802	13.6149973	-2.3757850
H	1.7425136	12.9229698	-3.1826256
H	2.3850763	14.0094307	-1.9512497
C	-7.8519603	14.4983208	-0.1647643
H	-8.3846186	15.3221659	0.3315977
H	-7.5804752	13.7790037	0.6203391
C	-5.4739601	20.6827089	-0.2769962
H	-6.3456192	21.3482206	-0.3977114
C	-0.1408447	20.6395918	-2.4483671
H	-0.8934058	21.4197225	-2.6341721
H	-0.5372227	19.9953562	-1.6441069
C	1.1735562	21.2818177	-1.9877176
H	1.5087621	22.0073360	-2.7466152
H	0.9969397	21.8529399	-1.0658550
C	-7.2426337	17.9669776	-3.1494371
H	-6.3011425	17.4189642	-3.3203100
H	-7.8716883	17.3323042	-2.5025385
C	-4.5443020	21.8445187	-6.3336980
H	-4.5056989	21.2718099	-7.2749281
H	-5.2375887	22.6787939	-6.5048242
C	-8.2540778	20.0986455	-2.2101145
H	-8.0371808	21.0626583	-1.7297712
H	-8.9189979	19.5442060	-1.5279369
C	-4.3204260	21.1908870	-1.1654261
H	-4.6398430	21.2345599	-2.2171142
H	-3.4897761	20.4680699	-1.1035422
C	-3.1415699	13.8504644	-5.7589673
H	-2.5242996	13.0497351	-5.3294884
H	-2.6102948	14.2193518	-6.6515825
C	2.4828801	19.3992547	-3.0389144
H	2.8679111	20.0484482	-3.8420591
H	3.2445192	18.6256447	-2.8701781

C	-4.5765380	22.0410483	1.6539323
H	-5.4120262	22.7581239	1.6059661
H	-4.2660695	21.9937578	2.7065138
C	-9.2648668	19.0060046	-4.2627699
H	-9.7539752	19.1958430	-5.2274386
H	-9.9767389	18.4131414	-3.6647384
C	-5.2379685	13.8784134	2.1024875
H	-6.1212592	14.3059629	2.5974202
H	-5.6027533	13.1330871	1.3793764
C	0.6543783	12.8549230	-1.3127042
H	1.2468151	12.0197544	-0.9162158
H	0.4496847	13.5280802	-0.4625959
C	-7.9830494	18.1937159	-4.4721995
H	-7.3161084	18.7265016	-5.1691318
H	-8.2118683	17.2246074	-4.9363706
C	2.2644953	20.2297063	-1.7713619
H	1.9708758	19.5628168	-0.9434691
H	3.2029392	20.7124200	-1.4680195
C	-3.4244464	22.5384476	0.7747459
H	-3.1038893	23.5369571	1.1001381
H	-2.5558783	21.8706252	0.8994401
C	-5.8472217	13.8895096	-1.6156161
H	-5.4861240	13.1407971	-0.8945835
H	-4.9579070	14.2962824	-2.1233306
C	-5.0678623	20.6584112	1.2044478
H	-4.2581507	19.9264472	1.3348421
H	-5.9120676	20.3372839	1.8317079
C	-6.5838022	15.0133571	-0.8644680
H	-6.9002529	15.7482717	-1.6263352
C	-3.8304306	22.5669414	-0.7018535
C	-8.0561375	12.6956086	-1.9368838
H	-8.7280047	12.2439068	-2.6787432
H	-7.7901465	11.8941328	-1.2284515
C	-8.7765675	13.8192792	-1.1850999
H	-9.6671104	13.4314989	-0.6726946
H	-9.1336067	14.5729781	-1.9072372
C	-1.4989140	13.4810544	-2.4872925
H	-2.4267600	13.0816331	-2.9210347
H	-1.7950719	14.1784817	-1.6855774
C	-4.3356889	13.1807940	3.1305087
H	-4.0464001	13.9059515	3.9085901
H	-4.9040814	12.3892709	3.6371327
C	-5.5775642	15.5465584	-5.7536869
H	-6.1879256	16.3468134	-6.1946140
H	-6.1120555	15.2013922	-4.8528030
C	-3.0775239	12.6036509	2.4739362
H	-3.3668561	11.8031538	1.7726838
H	-2.4356738	12.1358205	3.2320757
C	-3.1864164	14.3917933	0.6862654
H	-3.4834657	13.6742737	-0.0931300
H	-2.6270208	15.1945368	0.1805537
C	-8.9784764	20.3277244	-3.5441326
H	-9.9115892	20.8782593	-3.3648167
H	-8.3540074	20.9702033	-4.1859956
C	-4.5096805	13.2902016	-6.1753829
H	-4.3700930	12.4880493	-6.9123268
H	-4.9865050	12.8281027	-5.2951205
C	-6.7853962	13.2110775	-2.6195857

H	-6.2569627	12.3901883	-3.1245976
H	-7.0609276	13.9376230	-3.4022007
C	-5.4248845	14.3822354	-6.7374497
H	-6.4087022	13.9615697	-6.9855211
H	-5.0021032	14.7607536	-7.6825480
C	-0.6688760	12.3417956	-1.8854608
H	-1.2583215	11.8325561	-1.1095243
H	-0.4654550	11.5900911	-2.6649738
C	-2.2996201	13.6842666	1.7159689
H	-1.9124002	14.4287238	2.4314356
H	-1.4239315	13.2512269	1.2122115
H	-1.8469573	17.7996677	-0.7192333
C	0.0660061	19.7807271	-3.7101931
H	0.3432033	20.4326191	-4.5550920
C	1.1798063	18.7386706	-3.5049016
H	1.3569046	18.1812637	-4.4364062
H	0.8497260	18.0082756	-2.7453683
C	0.6347975	14.7706184	-2.9661492
H	1.2223467	15.2875210	-3.7378901
H	0.4246289	15.5117093	-2.1751136
C	-0.8434909	16.5238324	-5.4852336
H	0.2208642	16.4584565	-5.2256049
H	-0.9812400	15.9448535	-6.4090619
C	-1.2451983	17.9940483	-5.7095282
H	-2.1882169	18.0482623	-6.2684436
H	-0.4841573	18.5014812	-6.3185134
C	-3.3216278	15.0012886	-4.7578128
H	-3.8194779	14.6092824	-3.8533338
C	-2.6993686	20.3203465	-4.5920843
H	-2.7687071	20.9129492	-3.6640770
C	-6.4397559	17.1544688	1.1815906
H	-7.1771577	16.6011609	1.7794564
H	-5.7454608	17.6346847	1.8858173
H	-4.6297083	23.3105504	-0.8517353
H	-2.9855085	22.8857157	-1.3293219
O	-2.8405888	18.2049840	0.1376718
C	-2.5867143	17.9413751	1.5162619
H	-2.1347596	16.9467709	1.6584626
H	-3.5141682	17.9913169	2.1066742
H	-1.8951251	18.6988019	1.9112447

SCF energy GEOOPT = -7371.954494974 H
ZPE = 3716. kJ/mol
FREEH energy = 3902.07 kJ/mol
FREEH entropy = 1.59765 kJ/mol/K

$vibrational spectrum

# mode	symmetry	wave number	IR intensity	selection rules	
#		cm**(-1)	km/mol	IR	RAMAN
1	a	-1057.34	0.00000	YES	YES
2		-0.00	0.00000	-	-
3		-0.00	0.00000	-	-
4		-0.00	0.00000	-	-
5		0.00	0.00000	-	-
6		0.00	0.00000	-	-
7		0.00	0.00000	-	-
8	a	7.20	0.05992	YES	YES
9	a	17.25	0.04854	YES	YES

10	a	18.39	0.01895	YES	YES
11	a	28.48	0.04829	YES	YES
12	a	28.83	0.07213	YES	YES
13	a	31.81	0.00032	YES	YES
14	a	32.88	0.01586	YES	YES
15	a	35.80	0.05993	YES	YES
16	a	38.27	0.00376	YES	YES
17	a	39.20	0.08458	YES	YES
18	a	41.54	0.50280	YES	YES
19	a	43.66	0.14789	YES	YES
20	a	48.25	0.00846	YES	YES
21	a	49.81	0.08091	YES	YES
22	a	52.55	0.03439	YES	YES
23	a	54.23	0.15399	YES	YES
24	a	56.92	0.18868	YES	YES
25	a	60.10	0.18651	YES	YES
26	a	62.13	0.30989	YES	YES
27	a	64.88	0.21432	YES	YES
28	a	66.30	0.25012	YES	YES
29	a	68.25	0.13176	YES	YES
30	a	71.76	0.08433	YES	YES
31	a	73.78	0.04257	YES	YES
32	a	77.45	0.10898	YES	YES
33	a	82.00	0.85072	YES	YES
34	a	85.08	0.15286	YES	YES
35	a	94.26	0.86105	YES	YES
36	a	101.49	0.25817	YES	YES
37	a	106.79	1.45812	YES	YES
38	a	116.80	7.97934	YES	YES
39	a	119.17	3.60925	YES	YES
40	a	125.46	0.42274	YES	YES
41	a	129.05	0.81485	YES	YES
42	a	134.82	0.09815	YES	YES
43	a	136.43	1.42465	YES	YES
44	a	138.69	0.55216	YES	YES
45	a	141.14	1.17132	YES	YES
46	a	145.11	4.03154	YES	YES
47	a	153.27	1.72132	YES	YES
48	a	156.12	1.77727	YES	YES
49	a	163.27	0.76515	YES	YES
50	a	165.61	1.80963	YES	YES
51	a	170.68	0.90730	YES	YES
52	a	181.98	2.95855	YES	YES
53	a	186.35	1.07813	YES	YES
54	a	188.50	0.88038	YES	YES
55	a	200.33	1.87779	YES	YES
56	a	207.64	0.20573	YES	YES
57	a	212.88	0.65752	YES	YES
58	a	213.72	3.98114	YES	YES
59	a	216.26	0.72366	YES	YES
60	a	220.43	0.57269	YES	YES
61	a	221.65	0.15720	YES	YES
62	a	223.25	0.42654	YES	YES
63	a	227.27	0.08705	YES	YES
64	a	228.09	0.23138	YES	YES
65	a	229.20	0.23272	YES	YES
66	a	233.59	0.09217	YES	YES
67	a	237.23	0.74996	YES	YES

68	a	240.08	0.00759	YES	YES
69	a	244.40	0.65370	YES	YES
70	a	246.70	0.95463	YES	YES
71	a	256.87	0.43956	YES	YES
72	a	270.52	21.11884	YES	YES
73	a	280.18	5.37174	YES	YES
74	a	282.08	1.12257	YES	YES
75	a	286.73	8.26621	YES	YES
76	a	290.01	17.13334	YES	YES
77	a	299.24	2.15802	YES	YES
78	a	307.02	0.02916	YES	YES
79	a	309.69	0.28166	YES	YES
80	a	313.84	0.08388	YES	YES
81	a	327.89	0.27271	YES	YES
82	a	329.70	1.51948	YES	YES
83	a	331.15	0.31005	YES	YES
84	a	336.81	5.27547	YES	YES
85	a	339.67	1.55253	YES	YES
86	a	342.99	0.74974	YES	YES
87	a	360.46	34.86679	YES	YES
88	a	370.50	14.04369	YES	YES
89	a	380.34	2.67966	YES	YES
90	a	382.19	5.58838	YES	YES
91	a	383.74	0.44909	YES	YES
92	a	393.23	0.40054	YES	YES
93	a	397.79	0.93906	YES	YES
94	a	406.09	0.16384	YES	YES
95	a	413.42	0.75242	YES	YES
96	a	419.84	1.89799	YES	YES
97	a	421.95	4.67648	YES	YES
98	a	425.97	3.64803	YES	YES
99	a	428.26	0.24275	YES	YES
100	a	429.13	0.54225	YES	YES
101	a	430.16	0.10672	YES	YES
102	a	430.82	0.08320	YES	YES
103	a	431.25	0.16310	YES	YES
104	a	432.12	0.88240	YES	YES
105	a	433.37	0.67282	YES	YES
106	a	436.78	2.11260	YES	YES
107	a	437.28	0.73057	YES	YES
108	a	445.29	3.57317	YES	YES
109	a	457.23	4.75126	YES	YES
110	a	462.74	5.62404	YES	YES
111	a	469.00	3.09015	YES	YES
112	a	491.94	5.19050	YES	YES
113	a	493.52	3.27750	YES	YES
114	a	494.35	1.94316	YES	YES
115	a	496.33	1.69952	YES	YES
116	a	502.43	2.27018	YES	YES
117	a	503.26	0.26890	YES	YES
118	a	510.05	8.82438	YES	YES
119	a	515.01	11.90509	YES	YES
120	a	629.22	7.28395	YES	YES
121	a	630.18	34.66088	YES	YES
122	a	641.41	9.97628	YES	YES
123	a	657.31	12.85658	YES	YES
124	a	700.30	1.51020	YES	YES
125	a	715.71	1.58414	YES	YES

126	a	720.27	1.82603	YES	YES
127	a	731.70	6.98011	YES	YES
128	a	731.88	3.47500	YES	YES
129	a	735.43	3.17566	YES	YES
130	a	738.06	5.36189	YES	YES
131	a	740.24	4.11928	YES	YES
132	a	767.93	30.94780	YES	YES
133	a	770.29	1.96973	YES	YES
134	a	772.94	0.38235	YES	YES
135	a	773.77	0.13565	YES	YES
136	a	774.50	0.18602	YES	YES
137	a	774.84	1.11287	YES	YES
138	a	775.13	0.18326	YES	YES
139	a	775.54	0.19607	YES	YES
140	a	775.91	0.51585	YES	YES
141	a	781.28	16.98602	YES	YES
142	a	792.03	12.71965	YES	YES
143	a	805.20	0.78691	YES	YES
144	a	808.57	2.24736	YES	YES
145	a	809.76	10.11781	YES	YES
146	a	810.23	8.47190	YES	YES
147	a	811.98	2.62825	YES	YES
148	a	813.04	2.61834	YES	YES
149	a	813.81	1.11511	YES	YES
150	a	816.49	3.26968	YES	YES
151	a	831.58	6.56804	YES	YES
152	a	832.58	2.94039	YES	YES
153	a	836.77	2.84838	YES	YES
154	a	837.60	0.40234	YES	YES
155	a	837.95	5.14731	YES	YES
156	a	838.55	1.16832	YES	YES
157	a	838.99	4.25794	YES	YES
158	a	839.30	7.13700	YES	YES
159	a	847.55	15.85901	YES	YES
160	a	850.91	16.57577	YES	YES
161	a	871.21	1.10944	YES	YES
162	a	873.00	4.68778	YES	YES
163	a	874.31	1.59924	YES	YES
164	a	876.23	2.00106	YES	YES
165	a	876.78	5.11338	YES	YES
166	a	877.28	0.33935	YES	YES
167	a	877.65	3.17873	YES	YES
168	a	878.11	1.37209	YES	YES
169	a	878.99	3.33482	YES	YES
170	a	879.88	3.01380	YES	YES
171	a	880.42	0.87239	YES	YES
172	a	880.71	3.65413	YES	YES
173	a	881.61	1.78135	YES	YES
174	a	883.22	3.72809	YES	YES
175	a	883.52	11.23844	YES	YES
176	a	885.97	0.47393	YES	YES
177	a	902.72	1.70895	YES	YES
178	a	903.90	3.07186	YES	YES
179	a	904.95	2.74607	YES	YES
180	a	905.33	1.79222	YES	YES
181	a	907.86	1.27622	YES	YES
182	a	909.79	1.47166	YES	YES
183	a	910.15	0.76125	YES	YES

184	a	910.53	0.77204	YES	YES
185	a	979.78	1.50594	YES	YES
186	a	981.67	0.21353	YES	YES
187	a	987.34	0.55318	YES	YES
188	a	987.74	16.66157	YES	YES
189	a	988.30	7.48471	YES	YES
190	a	988.77	18.43162	YES	YES
191	a	988.89	4.79610	YES	YES
192	a	989.66	0.08541	YES	YES
193	a	990.71	2.06883	YES	YES
194	a	991.01	27.12745	YES	YES
195	a	1010.42	101.15655	YES	YES
196	a	1012.92	8.22496	YES	YES
197	a	1015.01	3.76374	YES	YES
198	a	1015.98	0.11937	YES	YES
199	a	1016.60	3.75092	YES	YES
200	a	1017.65	1.04948	YES	YES
201	a	1018.31	1.98393	YES	YES
202	a	1020.93	0.60727	YES	YES
203	a	1021.65	0.15180	YES	YES
204	a	1029.00	0.96861	YES	YES
205	a	1029.73	1.09654	YES	YES
206	a	1031.94	1.96229	YES	YES
207	a	1034.02	2.36082	YES	YES
208	a	1036.91	2.06029	YES	YES
209	a	1038.15	1.83229	YES	YES
210	a	1038.69	1.79186	YES	YES
211	a	1040.21	1.09526	YES	YES
212	a	1057.19	2.58296	YES	YES
213	a	1058.10	1.91384	YES	YES
214	a	1058.79	1.00834	YES	YES
215	a	1060.05	0.92709	YES	YES
216	a	1060.89	2.18893	YES	YES
217	a	1064.59	1.49157	YES	YES
218	a	1064.86	0.33585	YES	YES
219	a	1067.66	1.67264	YES	YES
220	a	1069.63	0.12004	YES	YES
221	a	1070.42	0.18911	YES	YES
222	a	1071.01	1.02111	YES	YES
223	a	1072.13	0.20754	YES	YES
224	a	1072.58	0.04015	YES	YES
225	a	1072.64	0.24740	YES	YES
226	a	1073.79	0.05040	YES	YES
227	a	1074.05	0.01818	YES	YES
228	a	1075.74	5.13393	YES	YES
229	a	1080.86	3.58163	YES	YES
230	a	1091.13	1.12193	YES	YES
231	a	1091.75	4.89483	YES	YES
232	a	1091.92	4.80964	YES	YES
233	a	1094.89	8.77123	YES	YES
234	a	1101.47	0.77604	YES	YES
235	a	1104.72	1.72850	YES	YES
236	a	1109.66	7.74998	YES	YES
237	a	1113.49	10.36528	YES	YES
238	a	1120.71	5.16083	YES	YES
239	a	1120.99	2.07396	YES	YES
240	a	1128.73	1.62093	YES	YES
241	a	1129.92	1.07862	YES	YES

242	a	1159.15	6.75570	YES	YES
243	a	1160.02	0.56568	YES	YES
244	a	1161.51	12.07117	YES	YES
245	a	1165.36	11.16790	YES	YES
246	a	1166.60	2.22400	YES	YES
247	a	1167.24	9.10596	YES	YES
248	a	1169.12	15.96158	YES	YES
249	a	1170.33	3.37324	YES	YES
250	a	1175.82	7.34067	YES	YES
251	a	1177.91	16.30863	YES	YES
252	a	1178.86	3.10739	YES	YES
253	a	1180.95	6.13960	YES	YES
254	a	1186.87	0.51476	YES	YES
255	a	1188.86	0.97534	YES	YES
256	a	1190.88	1.93825	YES	YES
257	a	1211.10	1.89297	YES	YES
258	a	1235.63	0.51776	YES	YES
259	a	1238.77	0.91097	YES	YES
260	a	1244.24	0.94713	YES	YES
261	a	1245.03	0.97354	YES	YES
262	a	1246.34	1.00851	YES	YES
263	a	1246.74	0.39574	YES	YES
264	a	1247.11	0.62435	YES	YES
265	a	1248.43	0.35562	YES	YES
266	a	1249.99	0.07518	YES	YES
267	a	1250.45	0.88525	YES	YES
268	a	1250.66	0.20033	YES	YES
269	a	1253.27	2.08247	YES	YES
270	a	1254.31	0.49500	YES	YES
271	a	1255.28	9.32361	YES	YES
272	a	1255.52	1.47616	YES	YES
273	a	1256.27	3.62227	YES	YES
274	a	1257.31	1.26886	YES	YES
275	a	1257.74	3.84674	YES	YES
276	a	1258.48	4.90509	YES	YES
277	a	1259.91	0.31341	YES	YES
278	a	1260.17	1.94401	YES	YES
279	a	1260.56	2.74286	YES	YES
280	a	1261.91	0.30602	YES	YES
281	a	1262.50	0.83495	YES	YES
282	a	1266.11	0.01640	YES	YES
283	a	1266.82	0.67611	YES	YES
284	a	1268.19	1.75939	YES	YES
285	a	1268.99	2.30849	YES	YES
286	a	1280.61	2.36675	YES	YES
287	a	1281.37	4.46809	YES	YES
288	a	1282.45	0.28526	YES	YES
289	a	1284.94	3.57779	YES	YES
290	a	1285.10	3.37695	YES	YES
291	a	1287.33	3.42050	YES	YES
292	a	1290.44	2.04579	YES	YES
293	a	1291.64	6.61407	YES	YES
294	a	1306.56	0.74374	YES	YES
295	a	1308.10	0.68738	YES	YES
296	a	1309.94	1.17419	YES	YES
297	a	1310.68	1.05769	YES	YES
298	a	1313.04	1.66704	YES	YES
299	a	1313.26	0.46531	YES	YES

300	a	1314.48	1.25469	YES	YES
301	a	1317.39	0.32884	YES	YES
302	a	1320.02	2.15417	YES	YES
303	a	1321.26	0.53947	YES	YES
304	a	1321.91	1.82241	YES	YES
305	a	1322.07	1.48783	YES	YES
306	a	1322.63	0.45109	YES	YES
307	a	1323.22	9.70332	YES	YES
308	a	1324.20	2.94021	YES	YES
309	a	1324.24	1.23881	YES	YES
310	a	1324.64	0.80797	YES	YES
311	a	1324.94	3.86403	YES	YES
312	a	1325.14	5.10252	YES	YES
313	a	1326.84	7.66334	YES	YES
314	a	1327.70	7.81703	YES	YES
315	a	1328.88	12.80254	YES	YES
316	a	1328.99	3.83313	YES	YES
317	a	1330.50	2.95842	YES	YES
318	a	1333.65	92.29254	YES	YES
319	a	1336.22	0.09521	YES	YES
320	a	1336.49	0.20053	YES	YES
321	a	1336.64	2.52915	YES	YES
322	a	1337.43	3.22959	YES	YES
323	a	1337.56	0.48372	YES	YES
324	a	1338.20	0.46696	YES	YES
325	a	1338.52	1.54386	YES	YES
326	a	1339.27	3.42166	YES	YES
327	a	1343.32	0.99307	YES	YES
328	a	1344.00	2.77893	YES	YES
329	a	1344.55	0.26217	YES	YES
330	a	1345.67	0.98039	YES	YES
331	a	1346.53	1.78696	YES	YES
332	a	1346.78	1.28256	YES	YES
333	a	1348.91	1.74540	YES	YES
334	a	1349.50	1.00699	YES	YES
335	a	1398.69	6.41410	YES	YES
336	a	1404.73	9.01512	YES	YES
337	a	1405.19	9.93548	YES	YES
338	a	1406.01	3.79330	YES	YES
339	a	1416.64	8.46702	YES	YES
340	a	1424.89	3.78708	YES	YES
341	a	1426.74	4.78051	YES	YES
342	a	1430.26	6.79484	YES	YES
343	a	1430.96	0.91976	YES	YES
344	a	1431.57	0.34589	YES	YES
345	a	1431.89	1.33955	YES	YES
346	a	1432.60	0.09327	YES	YES
347	a	1433.27	4.21216	YES	YES
348	a	1433.92	2.08714	YES	YES
349	a	1434.49	0.06599	YES	YES
350	a	1434.91	9.64740	YES	YES
351	a	1435.45	1.96902	YES	YES
352	a	1436.00	10.98018	YES	YES
353	a	1436.50	10.94070	YES	YES
354	a	1437.51	1.96525	YES	YES
355	a	1438.16	8.33885	YES	YES
356	a	1438.20	3.93505	YES	YES
357	a	1438.46	4.61280	YES	YES

358	a	1438.63	4.35165	YES	YES
359	a	1438.97	3.66733	YES	YES
360	a	1439.12	4.97317	YES	YES
361	a	1439.32	4.94368	YES	YES
362	a	1439.61	3.55378	YES	YES
363	a	1439.65	2.30936	YES	YES
364	a	1441.08	7.25625	YES	YES
365	a	1441.83	3.93680	YES	YES
366	a	1442.62	6.77885	YES	YES
367	a	1443.15	35.80344	YES	YES
368	a	1444.14	7.55985	YES	YES
369	a	1444.61	2.07034	YES	YES
370	a	1444.86	25.58122	YES	YES
371	a	1445.13	7.19669	YES	YES
372	a	1446.53	9.60285	YES	YES
373	a	1450.79	1.66620	YES	YES
374	a	1452.36	0.64301	YES	YES
375	a	1453.53	3.62030	YES	YES
376	a	1454.39	1.26879	YES	YES
377	a	1454.69	4.38660	YES	YES
378	a	1455.29	1.25107	YES	YES
379	a	1455.41	5.68938	YES	YES
380	a	1456.44	0.58983	YES	YES
381	a	1458.07	4.15160	YES	YES
382	a	2924.38	21.41184	YES	YES
383	a	2926.87	37.17627	YES	YES
384	a	2928.32	5.95618	YES	YES
385	a	2928.80	59.60198	YES	YES
386	a	2930.66	7.01232	YES	YES
387	a	2931.75	7.70536	YES	YES
388	a	2931.90	19.08792	YES	YES
389	a	2932.61	1.20233	YES	YES
390	a	2938.77	10.51096	YES	YES
391	a	2939.45	0.69848	YES	YES
392	a	2939.75	7.74688	YES	YES
393	a	2941.21	21.08619	YES	YES
394	a	2941.94	4.13524	YES	YES
395	a	2942.62	7.32746	YES	YES
396	a	2943.11	8.59553	YES	YES
397	a	2943.19	18.84330	YES	YES
398	a	2943.91	14.13355	YES	YES
399	a	2944.46	11.59728	YES	YES
400	a	2945.34	9.76448	YES	YES
401	a	2945.72	6.46232	YES	YES
402	a	2945.92	13.54803	YES	YES
403	a	2946.14	6.11828	YES	YES
404	a	2947.20	11.43114	YES	YES
405	a	2949.73	0.86261	YES	YES
406	a	2949.92	9.38537	YES	YES
407	a	2950.05	22.77988	YES	YES
408	a	2950.15	4.06202	YES	YES
409	a	2951.47	28.57850	YES	YES
410	a	2952.05	8.70128	YES	YES
411	a	2953.53	0.62213	YES	YES
412	a	2955.24	4.45110	YES	YES
413	a	2955.41	7.57802	YES	YES
414	a	2955.42	8.99412	YES	YES
415	a	2956.20	7.70647	YES	YES

416	a	2957.32	5.42526	YES	YES
417	a	2958.07	12.31387	YES	YES
418	a	2958.68	14.72924	YES	YES
419	a	2959.37	11.47001	YES	YES
420	a	2959.79	34.38437	YES	YES
421	a	2959.98	32.86785	YES	YES
422	a	2960.30	41.57005	YES	YES
423	a	2960.98	16.72947	YES	YES
424	a	2961.72	18.32178	YES	YES
425	a	2961.83	58.14074	YES	YES
426	a	2962.66	22.97244	YES	YES
427	a	2965.01	25.22910	YES	YES
428	a	2966.19	19.04644	YES	YES
429	a	2971.49	15.39705	YES	YES
430	a	2974.78	11.44347	YES	YES
431	a	2978.40	22.86103	YES	YES
432	a	2979.35	6.09883	YES	YES
433	a	2984.74	3.73619	YES	YES
434	a	2985.90	28.70768	YES	YES
435	a	2985.96	4.03512	YES	YES
436	a	2988.97	6.07821	YES	YES
437	a	2991.16	14.27318	YES	YES
438	a	2991.74	37.61671	YES	YES
439	a	2992.37	17.41710	YES	YES
440	a	2994.48	23.36883	YES	YES
441	a	2995.49	18.71208	YES	YES
442	a	2997.13	23.65118	YES	YES
443	a	2997.56	21.11691	YES	YES
444	a	2999.20	43.57171	YES	YES
445	a	3000.31	5.83795	YES	YES
446	a	3001.28	18.83906	YES	YES
447	a	3002.09	34.46485	YES	YES
448	a	3004.05	32.78734	YES	YES
449	a	3005.85	15.71079	YES	YES
450	a	3007.38	27.81319	YES	YES
451	a	3007.49	10.15276	YES	YES
452	a	3009.18	15.06322	YES	YES
453	a	3009.44	23.06888	YES	YES
454	a	3009.68	22.16669	YES	YES
455	a	3010.88	25.65647	YES	YES
456	a	3010.96	18.59929	YES	YES
457	a	3011.68	23.43593	YES	YES
458	a	3011.96	17.77802	YES	YES
459	a	3012.64	22.47167	YES	YES
460	a	3014.10	17.87893	YES	YES
461	a	3014.48	26.95070	YES	YES
462	a	3014.68	11.03760	YES	YES
463	a	3014.81	23.06187	YES	YES
464	a	3016.36	25.39532	YES	YES
465	a	3017.06	30.45275	YES	YES
466	a	3017.10	9.87280	YES	YES
467	a	3017.34	23.55986	YES	YES
468	a	3017.48	43.26205	YES	YES
469	a	3017.89	24.60695	YES	YES
470	a	3018.02	37.90353	YES	YES
471	a	3018.08	18.81995	YES	YES
472	a	3018.23	24.38528	YES	YES
473	a	3019.02	25.20187	YES	YES

474	a	3019.12	21.34789	YES	YES
475	a	3022.59	32.33831	YES	YES
476	a	3024.71	12.25449	YES	YES
477	a	3026.35	5.42856	YES	YES
478	a	3029.25	0.82805	YES	YES
479	a	3036.12	1.37438	YES	YES
480	a	3044.05	0.61742	YES	YES

$end

Double hybrid single point energy = -7364.642355164008 H
COSMO energy + OC correction = -7372.1015196706 H (in *o*DFB)

6.2.23 [H{Ga(dcpe)}$_2$(OMe)]$^{2+}$ (*syn*-periplanar)

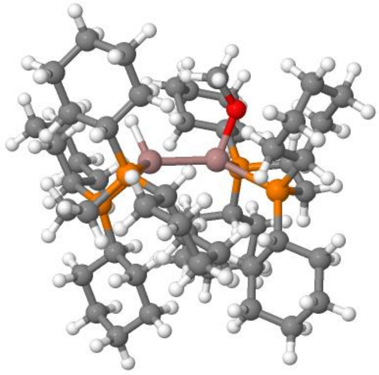

Method: (RI-)BP86(D3BJ)/def2-TZVPP
Symmetry: c1

Cartesian coordinates in Ångström:

Ga	-2.2158517	17.2831098	-2.4356251
Ga	-4.0345940	17.6984071	-0.8965123
P	-1.4944858	18.9056695	-4.0399729
P	-6.0683501	19.0357245	-0.9210266
P	-2.0147850	15.6292530	-4.1487876
P	-5.3277291	16.0474393	0.2517611
C	-4.9660946	21.0367638	-5.0213262
H	-5.0968088	21.6286673	-4.0995381
H	-5.9619635	20.6630962	-5.2992568
C	-1.0591847	14.1488840	-3.6004569
H	-0.9287955	13.5107523	-4.4905190
C	-4.3115518	15.1731415	1.5078591
H	-4.0455329	16.0065604	2.1826357
C	-4.3683780	16.1766537	-5.5465217
H	-3.7860433	16.6313511	-6.3640920
H	-4.5337670	16.9626217	-4.7938066
C	-7.0141523	19.2621958	-2.4848092
H	-6.3777616	19.9292476	-3.0884723
C	-7.2077398	18.1234456	0.2240937
H	-7.8001343	18.8475001	0.7985037
H	-7.9142130	17.5707440	-0.4109002
C	-3.0007145	22.4288738	-5.7692064
H	-2.5943732	23.0545979	-6.5748830
H	-3.0428527	23.0643331	-4.8694142
C	-2.0525181	21.2510682	-5.5095413
H	-1.0585149	21.6264595	-5.2323359
H	-1.9293881	20.6753508	-6.4413668

C	-4.0336882	19.8521488	-4.7374093
H	-4.0127320	19.2037621	-5.6275263
H	-4.4177356	19.2355986	-3.9092187
C	1.0488206	13.2705276	-2.5072911
H	1.2538306	12.5759749	-3.3379999
H	2.0246496	13.5662191	-2.0989786
C	-7.5726024	14.2900174	-0.1818520
H	-8.2077057	15.0291782	0.3271381
H	-7.2381617	13.5784723	0.5864461
C	-5.7191970	20.7168574	-0.2514299
H	-6.5912021	21.3417495	-0.5093444
C	0.0224999	20.3177934	-2.2203479
H	-0.7263347	21.1218979	-2.2740566
H	-0.3365079	19.5926386	-1.4709977
C	1.3735726	20.8852572	-1.7679845
H	1.6829412	21.6841532	-2.4611888
H	1.2598236	21.3537242	-0.7808492
C	-7.1673015	17.9307638	-3.2382713
H	-6.1806731	17.4657774	-3.3963020
H	-7.7532253	17.2287726	-2.6211595
C	-4.4090223	21.9446487	-6.1211276
H	-4.3794455	21.3891332	-7.0729916
H	-5.0788348	22.8000315	-6.2802885
C	-8.3763727	19.9490284	-2.2810785
H	-8.2530603	20.9128069	-1.7686642
H	-9.0113164	19.3223830	-1.6342856
C	-4.4802255	21.2708280	-0.9821458
H	-4.6509582	21.2808149	-2.0694666
H	-3.6311404	20.5921104	-0.7951255
C	-3.3414018	13.8892490	-5.9543690
H	-2.8083419	13.0481192	-5.4913682
H	-2.7060924	14.2565627	-6.7770295
C	2.5786300	19.1035398	-3.0892035
H	2.9356880	19.8288120	-3.8382224
H	3.3296257	18.3029383	-3.0466743
C	-5.1712181	22.1482835	1.7528349
H	-6.0221555	22.8250839	1.5708622
H	-5.0104032	22.1344064	2.8392366
C	-9.2416555	18.8334174	-4.3822240
H	-9.7190849	19.0048084	-5.3561558
H	-9.9185497	18.1721149	-3.8162687
C	-5.0415355	14.0857654	2.3127435
H	-5.9677003	14.4796448	2.7548059
H	-5.3311968	13.2662646	1.6360809
C	0.2109702	12.5592679	-1.4415965
H	0.7359750	11.6669040	-1.0761232
H	0.0824610	13.2277187	-0.5733818
C	-7.8941532	18.1309043	-4.5736961
H	-7.2558123	18.7319359	-5.2421675
H	-8.0357178	17.1594460	-5.0651279
C	2.4486286	19.7956879	-1.7294707
H	2.1849761	19.0489824	-0.9619879
H	3.4135419	20.2255136	-1.4299621
C	-3.9305224	22.6884474	1.0345445
H	-3.7057488	23.7066071	1.3789149
H	-3.0580353	22.0670201	1.2961347
C	-5.4721553	13.9584362	-1.5770769
H	-5.0612032	13.2296382	-0.8617900

H	-4.6102601	14.4757354	-2.0263442
C	-5.5309024	20.7350654	1.2751455
H	-4.7297296	20.0293498	1.5404332
H	-6.4499393	20.3979034	1.7758161
C	-6.3546262	14.9689361	-0.8262582
H	-6.7324084	15.6844837	-1.5806110
C	-4.1237386	22.6757767	-0.4849457
C	-7.5191597	12.5437282	-2.0247924
H	-8.1142372	12.0530906	-2.8065100
H	-7.1879514	11.7469460	-1.3391296
C	-8.3834776	13.5484196	-1.2544464
H	-9.2367656	13.0396999	-0.7865033
H	-8.8062368	14.2842570	-1.9595066
C	-1.9024142	13.3966930	-2.5538727
H	-2.8645480	13.0857365	-2.9855492
H	-2.1310440	14.0912390	-1.7282677
C	-4.1209511	13.5294805	3.4081893
H	-3.9062572	14.3270925	4.1377224
H	-4.6436983	12.7348925	3.9575649
C	-5.7014123	15.6783835	-6.1148541
H	-6.2424088	16.5164337	-6.5743180
H	-6.3290903	15.3121111	-5.2851688
C	-2.8067238	13.0032327	2.8220394
H	-3.0182326	12.1343042	2.1762853
H	-2.1522371	12.6408044	3.6258567
C	-2.9986314	14.6427073	0.9030049
H	-3.2242806	13.8455996	0.1782700
H	-2.4845459	15.4469278	0.3542093
C	-9.0806375	20.1560540	-3.6285838
H	-10.0586957	20.6271195	-3.4630334
H	-8.4925021	20.8613267	-4.2383849
C	-4.6807164	13.4029928	-6.5259134
H	-4.4962278	12.6231240	-7.2766327
H	-5.2626170	12.9297966	-5.7182542
C	-6.2891443	13.2195344	-2.6411123
H	-5.6571993	12.4774451	-3.1507183
H	-6.6133977	13.9412098	-3.4105919
C	-5.4893201	14.5530043	-7.1314967
H	-6.4572398	14.1856118	-7.4976204
H	-4.9563444	14.9529279	-8.0097979
C	-1.1629614	12.1759245	-1.9954624
H	-1.7790209	11.7025759	-1.2177216
H	-1.0416087	11.4281394	-2.7956205
C	-2.0902488	14.0814516	2.0022146
H	-1.7795981	14.9051777	2.6649832
H	-1.1722503	13.6780261	1.5516210
H	-0.8962599	17.0163306	-1.6061685
C	0.1460093	19.6021063	-3.5788404
H	0.4041730	20.3401362	-4.3568370
C	1.2377983	18.5178857	-3.5515004
H	1.3535412	18.0689903	-4.5489960
H	0.9314350	17.7150920	-2.8619248
C	0.3289478	14.5140933	-3.0451637
H	0.9435925	14.9904984	-3.8218612
H	0.2110006	15.2479649	-2.2331022
C	-1.0051420	16.4749502	-5.4478497
H	0.0385978	16.3381387	-5.1376453
H	-1.1253003	15.9440526	-6.4025601

```
C    -1.3073472   17.9728855   -5.6286594
H    -2.2482757   18.1108012   -6.1762956
H    -0.5175747   18.4447810   -6.2292266
C    -3.5661580   15.0177894   -4.9348326
H    -4.1508627   14.6146939   -4.0907480
C    -2.6097043   20.3295157   -4.4095443
H    -2.6491915   20.8919830   -3.4607607
C    -6.4692261   17.1627124    1.1709370
H    -7.1870991   16.5842588    1.7676437
H    -5.8252390   17.7205848    1.8651816
H    -4.9295107   23.3751099   -0.7609974
H    -3.2155959   23.0278437   -0.9951645
O    -3.4036223   18.3230524    0.7509333
C    -2.0939221   18.8397073    0.9162888
H    -1.3159184   18.1447801    0.5552496
H    -1.9191227   19.0184736    1.9874218
H    -1.9535345   19.8040949    0.3916721

SCF energy GEOOPT =  -7372.004530091 H
ZPE =   3723. kJ/mol
FREEH energy =  3909.84 kJ/mol
FREEH entropy =   1.60252 kJ/mol/K

$vibrational spectrum
#  mode     symmetry     wave number   IR intensity    selection rules
#                         cm**(-1)        km/mol          IR     RAMAN
     1                       -0.00          0.00000        -        -
     2                       -0.00          0.00000        -        -
     3                       -0.00          0.00000        -        -
     4                       -0.00          0.00000        -        -
     5                        0.00          0.00000        -        -
     6                        0.00          0.00000        -        -
     7         a             13.67          0.00971       YES      YES
     8         a             16.77          0.03287       YES      YES
     9         a             22.29          0.21157       YES      YES
    10         a             25.92          0.04627       YES      YES
    11         a             28.34          0.01253       YES      YES
    12         a             31.10          0.03592       YES      YES
    13         a             31.72          0.00952       YES      YES
    14         a             33.97          0.04075       YES      YES
    15         a             37.00          0.00258       YES      YES
    16         a             37.45          0.03296       YES      YES
    17         a             40.87          0.09205       YES      YES
    18         a             45.65          0.16917       YES      YES
    19         a             48.41          0.13924       YES      YES
    20         a             49.07          0.30146       YES      YES
    21         a             51.62          0.08865       YES      YES
    22         a             52.39          0.28866       YES      YES
    23         a             55.08          0.08083       YES      YES
    24         a             56.56          0.52287       YES      YES
    25         a             61.74          0.04716       YES      YES
    26         a             64.10          0.01402       YES      YES
    27         a             66.42          0.08312       YES      YES
    28         a             67.59          0.05736       YES      YES
    29         a             70.19          0.38319       YES      YES
    30         a             74.29          0.34530       YES      YES
    31         a             75.64          0.01558       YES      YES
    32         a             76.89          0.03015       YES      YES
```

33	a	87.30	0.05203	YES	YES
34	a	89.72	0.36347	YES	YES
35	a	90.59	0.50448	YES	YES
36	a	100.56	0.23022	YES	YES
37	a	101.99	1.63297	YES	YES
38	a	105.46	0.35346	YES	YES
39	a	115.30	0.04324	YES	YES
40	a	122.74	0.74004	YES	YES
41	a	134.90	0.69249	YES	YES
42	a	135.60	1.42525	YES	YES
43	a	137.09	0.22789	YES	YES
44	a	140.24	0.25561	YES	YES
45	a	140.75	0.80586	YES	YES
46	a	149.00	13.35085	YES	YES
47	a	156.33	0.60313	YES	YES
48	a	159.09	2.01414	YES	YES
49	a	164.82	1.66219	YES	YES
50	a	166.85	0.09794	YES	YES
51	a	184.32	6.02239	YES	YES
52	a	188.05	9.21271	YES	YES
53	a	190.49	0.48172	YES	YES
54	a	193.80	0.82361	YES	YES
55	a	204.75	0.41398	YES	YES
56	a	210.19	0.72449	YES	YES
57	a	216.05	0.23875	YES	YES
58	a	219.15	0.13982	YES	YES
59	a	221.04	0.12856	YES	YES
60	a	223.97	0.01240	YES	YES
61	a	226.36	0.11709	YES	YES
62	a	227.73	0.44588	YES	YES
63	a	229.70	0.25470	YES	YES
64	a	231.84	0.55491	YES	YES
65	a	235.14	2.98040	YES	YES
66	a	236.67	0.08316	YES	YES
67	a	238.14	0.75935	YES	YES
68	a	240.11	7.70163	YES	YES
69	a	242.82	1.94168	YES	YES
70	a	244.06	0.44277	YES	YES
71	a	264.04	4.32160	YES	YES
72	a	281.22	1.22237	YES	YES
73	a	283.31	0.59140	YES	YES
74	a	285.99	0.25816	YES	YES
75	a	290.67	0.30674	YES	YES
76	a	303.60	1.87962	YES	YES
77	a	306.63	1.79620	YES	YES
78	a	310.02	0.46018	YES	YES
79	a	314.16	0.94563	YES	YES
80	a	329.62	0.71115	YES	YES
81	a	330.73	0.16843	YES	YES
82	a	334.46	0.31341	YES	YES
83	a	339.32	1.33027	YES	YES
84	a	340.35	1.28036	YES	YES
85	a	340.67	1.66968	YES	YES
86	a	370.26	5.65073	YES	YES
87	a	380.72	2.93075	YES	YES
88	a	381.50	0.75598	YES	YES
89	a	384.24	0.63871	YES	YES
90	a	394.50	0.49782	YES	YES

91	a	398.44	0.31866	YES	YES
92	a	406.17	0.38784	YES	YES
93	a	411.14	1.05609	YES	YES
94	a	419.52	0.75618	YES	YES
95	a	420.69	4.26226	YES	YES
96	a	425.20	3.77331	YES	YES
97	a	427.55	0.34446	YES	YES
98	a	429.73	0.07290	YES	YES
99	a	430.13	0.48961	YES	YES
100	a	430.59	0.06228	YES	YES
101	a	431.59	0.36110	YES	YES
102	a	433.24	0.12265	YES	YES
103	a	433.73	0.49263	YES	YES
104	a	435.75	1.17331	YES	YES
105	a	437.17	0.29415	YES	YES
106	a	445.51	2.26132	YES	YES
107	a	459.08	3.59002	YES	YES
108	a	460.27	4.86673	YES	YES
109	a	471.92	2.60237	YES	YES
110	a	492.22	11.72945	YES	YES
111	a	494.62	2.83101	YES	YES
112	a	496.65	10.55358	YES	YES
113	a	497.51	2.88098	YES	YES
114	a	499.15	2.74060	YES	YES
115	a	502.83	23.13260	YES	YES
116	a	508.37	11.04129	YES	YES
117	a	512.18	15.71572	YES	YES
118	a	514.62	11.80014	YES	YES
119	a	535.92	87.57757	YES	YES
120	a	593.10	87.93940	YES	YES
121	a	628.65	15.79248	YES	YES
122	a	633.07	24.23107	YES	YES
123	a	643.34	12.56273	YES	YES
124	a	661.97	12.18023	YES	YES
125	a	701.44	1.58488	YES	YES
126	a	718.36	1.95393	YES	YES
127	a	721.36	1.44313	YES	YES
128	a	732.52	10.56100	YES	YES
129	a	734.83	3.57795	YES	YES
130	a	735.26	4.27900	YES	YES
131	a	738.07	1.18874	YES	YES
132	a	741.83	4.62980	YES	YES
133	a	771.29	1.17445	YES	YES
134	a	773.27	0.06404	YES	YES
135	a	773.50	0.90531	YES	YES
136	a	774.04	0.44873	YES	YES
137	a	775.00	0.83359	YES	YES
138	a	776.26	0.76379	YES	YES
139	a	776.29	1.30173	YES	YES
140	a	776.36	1.03160	YES	YES
141	a	778.47	10.56374	YES	YES
142	a	787.93	13.77815	YES	YES
143	a	807.08	0.18150	YES	YES
144	a	809.42	2.54842	YES	YES
145	a	810.74	10.33254	YES	YES
146	a	810.89	5.31528	YES	YES
147	a	812.00	1.05261	YES	YES
148	a	813.44	1.58462	YES	YES

149	a	815.02	2.53531	YES	YES
150	a	818.15	2.62075	YES	YES
151	a	832.56	6.06336	YES	YES
152	a	832.83	4.82516	YES	YES
153	a	837.01	5.99056	YES	YES
154	a	837.66	2.02235	YES	YES
155	a	838.32	0.64968	YES	YES
156	a	838.97	1.73538	YES	YES
157	a	839.22	4.23328	YES	YES
158	a	839.44	5.94719	YES	YES
159	a	848.54	12.34419	YES	YES
160	a	854.49	15.35280	YES	YES
161	a	872.38	3.24800	YES	YES
162	a	873.37	1.48229	YES	YES
163	a	873.64	2.00548	YES	YES
164	a	876.42	1.90593	YES	YES
165	a	877.33	3.98521	YES	YES
166	a	877.99	2.49155	YES	YES
167	a	878.55	3.13079	YES	YES
168	a	879.12	6.29623	YES	YES
169	a	879.72	1.51512	YES	YES
170	a	880.02	1.74880	YES	YES
171	a	880.68	2.15485	YES	YES
172	a	882.42	1.99042	YES	YES
173	a	883.26	5.37986	YES	YES
174	a	883.79	4.30122	YES	YES
175	a	885.88	0.72173	YES	YES
176	a	887.26	7.10020	YES	YES
177	a	903.90	1.58393	YES	YES
178	a	904.63	1.27805	YES	YES
179	a	904.79	1.43216	YES	YES
180	a	905.18	2.64559	YES	YES
181	a	907.88	2.03141	YES	YES
182	a	909.36	0.99123	YES	YES
183	a	909.96	0.79225	YES	YES
184	a	911.02	0.83106	YES	YES
185	a	980.58	1.20837	YES	YES
186	a	983.67	0.33769	YES	YES
187	a	988.44	12.56234	YES	YES
188	a	988.60	1.17845	YES	YES
189	a	989.38	12.63901	YES	YES
190	a	989.57	14.15640	YES	YES
191	a	990.24	16.47343	YES	YES
192	a	990.46	3.77503	YES	YES
193	a	991.99	4.79839	YES	YES
194	a	992.08	10.20653	YES	YES
195	a	1013.59	0.35714	YES	YES
196	a	1014.66	0.58887	YES	YES
197	a	1016.65	0.11474	YES	YES
198	a	1017.04	1.18528	YES	YES
199	a	1017.82	1.07543	YES	YES
200	a	1018.44	0.34920	YES	YES
201	a	1019.22	1.26932	YES	YES
202	a	1021.89	0.51654	YES	YES
203	a	1030.37	0.81045	YES	YES
204	a	1031.33	2.59831	YES	YES
205	a	1031.82	0.57771	YES	YES
206	a	1033.29	0.92198	YES	YES

207	a	1037.11	2.93978	YES	YES
208	a	1039.22	4.04384	YES	YES
209	a	1039.46	0.74148	YES	YES
210	a	1040.07	1.93433	YES	YES
211	a	1050.25	142.45659	YES	YES
212	a	1056.77	3.48891	YES	YES
213	a	1057.86	2.54762	YES	YES
214	a	1059.51	3.19832	YES	YES
215	a	1061.01	1.27856	YES	YES
216	a	1061.40	1.75046	YES	YES
217	a	1064.82	0.74740	YES	YES
218	a	1065.20	1.80743	YES	YES
219	a	1067.93	1.26455	YES	YES
220	a	1070.23	0.06751	YES	YES
221	a	1071.34	0.27056	YES	YES
222	a	1071.83	0.10016	YES	YES
223	a	1072.01	0.26527	YES	YES
224	a	1072.55	0.14340	YES	YES
225	a	1073.37	0.12689	YES	YES
226	a	1073.38	0.23369	YES	YES
227	a	1074.44	0.03507	YES	YES
228	a	1082.47	2.70958	YES	YES
229	a	1083.44	3.22053	YES	YES
230	a	1092.96	1.38174	YES	YES
231	a	1093.87	4.92411	YES	YES
232	a	1094.45	5.53424	YES	YES
233	a	1097.47	7.76073	YES	YES
234	a	1101.88	1.23162	YES	YES
235	a	1104.73	1.81484	YES	YES
236	a	1109.07	9.61422	YES	YES
237	a	1116.33	8.10066	YES	YES
238	a	1122.01	0.82833	YES	YES
239	a	1130.93	1.68954	YES	YES
240	a	1133.87	0.29321	YES	YES
241	a	1145.50	1.72268	YES	YES
242	a	1159.39	10.38021	YES	YES
243	a	1160.71	1.01520	YES	YES
244	a	1161.77	14.37185	YES	YES
245	a	1165.12	6.64013	YES	YES
246	a	1167.21	7.37849	YES	YES
247	a	1167.73	5.84905	YES	YES
248	a	1169.42	13.75082	YES	YES
249	a	1170.72	2.46537	YES	YES
250	a	1177.78	10.56223	YES	YES
251	a	1180.57	10.30516	YES	YES
252	a	1181.46	4.82694	YES	YES
253	a	1181.56	5.05293	YES	YES
254	a	1187.23	0.53695	YES	YES
255	a	1188.82	1.48459	YES	YES
256	a	1192.97	1.92675	YES	YES
257	a	1213.33	2.85293	YES	YES
258	a	1239.35	0.26360	YES	YES
259	a	1241.54	0.29405	YES	YES
260	a	1243.07	0.81564	YES	YES
261	a	1245.82	0.08574	YES	YES
262	a	1246.50	1.50150	YES	YES
263	a	1247.30	0.58655	YES	YES
264	a	1247.73	0.36465	YES	YES

265	a	1248.49	0.45602	YES	YES
266	a	1250.07	0.40803	YES	YES
267	a	1250.61	0.03687	YES	YES
268	a	1252.30	1.30249	YES	YES
269	a	1253.60	1.64400	YES	YES
270	a	1254.92	8.83010	YES	YES
271	a	1255.22	2.15334	YES	YES
272	a	1256.28	4.48624	YES	YES
273	a	1256.71	1.47529	YES	YES
274	a	1257.27	3.49200	YES	YES
275	a	1257.51	4.21583	YES	YES
276	a	1258.77	2.40073	YES	YES
277	a	1261.02	1.72320	YES	YES
278	a	1261.34	0.82972	YES	YES
279	a	1261.63	2.20498	YES	YES
280	a	1263.91	1.12687	YES	YES
281	a	1263.97	0.75256	YES	YES
282	a	1266.35	0.78366	YES	YES
283	a	1267.38	0.62963	YES	YES
284	a	1270.67	2.10057	YES	YES
285	a	1272.15	1.45931	YES	YES
286	a	1281.36	2.28102	YES	YES
287	a	1282.53	2.99103	YES	YES
288	a	1282.91	1.42219	YES	YES
289	a	1285.14	3.02992	YES	YES
290	a	1286.72	3.75650	YES	YES
291	a	1287.38	2.55108	YES	YES
292	a	1290.13	3.62474	YES	YES
293	a	1292.30	3.33301	YES	YES
294	a	1307.01	0.14317	YES	YES
295	a	1309.14	0.36093	YES	YES
296	a	1310.05	0.71172	YES	YES
297	a	1310.92	0.57161	YES	YES
298	a	1311.95	0.63642	YES	YES
299	a	1312.25	0.45150	YES	YES
300	a	1315.89	2.18233	YES	YES
301	a	1316.96	2.11873	YES	YES
302	a	1319.14	1.26254	YES	YES
303	a	1321.40	1.58258	YES	YES
304	a	1321.76	0.49441	YES	YES
305	a	1322.35	0.75835	YES	YES
306	a	1322.82	1.31131	YES	YES
307	a	1323.78	0.72493	YES	YES
308	a	1323.98	0.59810	YES	YES
309	a	1324.21	0.99986	YES	YES
310	a	1324.71	0.51730	YES	YES
311	a	1325.22	0.24332	YES	YES
312	a	1325.83	6.10722	YES	YES
313	a	1326.80	1.53852	YES	YES
314	a	1329.84	3.27143	YES	YES
315	a	1330.13	3.35849	YES	YES
316	a	1330.39	6.40849	YES	YES
317	a	1333.21	3.13587	YES	YES
318	a	1336.60	0.23247	YES	YES
319	a	1336.70	0.36243	YES	YES
320	a	1336.88	0.37078	YES	YES
321	a	1337.60	0.11709	YES	YES
322	a	1337.82	0.54453	YES	YES

323	a	1338.47	0.17277	YES	YES
324	a	1338.77	0.02058	YES	YES
325	a	1339.44	0.31647	YES	YES
326	a	1343.85	1.59938	YES	YES
327	a	1344.72	1.08683	YES	YES
328	a	1345.04	1.06288	YES	YES
329	a	1346.22	1.45741	YES	YES
330	a	1346.43	0.27702	YES	YES
331	a	1347.39	2.44078	YES	YES
332	a	1347.87	1.66425	YES	YES
333	a	1350.06	1.21840	YES	YES
334	a	1399.80	5.80908	YES	YES
335	a	1403.95	8.07592	YES	YES
336	a	1404.28	7.77922	YES	YES
337	a	1407.56	11.63272	YES	YES
338	a	1424.66	4.62402	YES	YES
339	a	1426.44	1.89962	YES	YES
340	a	1427.69	8.22370	YES	YES
341	a	1430.31	2.32282	YES	YES
342	a	1430.91	0.93519	YES	YES
343	a	1431.47	0.99985	YES	YES
344	a	1432.10	3.29455	YES	YES
345	a	1433.22	2.77630	YES	YES
346	a	1433.39	6.45783	YES	YES
347	a	1434.52	0.51186	YES	YES
348	a	1435.93	0.16700	YES	YES
349	a	1436.24	12.31759	YES	YES
350	a	1436.62	11.98082	YES	YES
351	a	1436.72	1.59054	YES	YES
352	a	1437.23	3.91169	YES	YES
353	a	1437.69	12.15745	YES	YES
354	a	1438.00	0.63549	YES	YES
355	a	1438.31	1.65602	YES	YES
356	a	1438.58	6.57113	YES	YES
357	a	1438.81	6.05648	YES	YES
358	a	1439.33	7.75234	YES	YES
359	a	1439.88	5.21921	YES	YES
360	a	1440.02	5.27888	YES	YES
361	a	1440.22	3.16411	YES	YES
362	a	1440.96	2.61958	YES	YES
363	a	1441.94	11.80994	YES	YES
364	a	1443.08	3.56343	YES	YES
365	a	1443.48	17.45311	YES	YES
366	a	1444.56	13.84874	YES	YES
367	a	1444.71	11.91952	YES	YES
368	a	1445.54	17.38057	YES	YES
369	a	1446.17	6.28639	YES	YES
370	a	1446.61	13.92127	YES	YES
371	a	1448.40	13.44967	YES	YES
372	a	1450.74	11.04633	YES	YES
373	a	1452.94	1.17700	YES	YES
374	a	1453.80	0.61294	YES	YES
375	a	1454.44	3.68781	YES	YES
376	a	1455.23	5.22411	YES	YES
377	a	1455.90	1.36592	YES	YES
378	a	1456.61	3.09951	YES	YES
379	a	1457.10	1.12465	YES	YES
380	a	1462.61	2.12343	YES	YES

381	a	1851.55	123.98370	YES	YES
382	a	2883.01	79.07857	YES	YES
383	a	2916.06	1.03873	YES	YES
384	a	2932.18	34.38402	YES	YES
385	a	2935.10	15.93751	YES	YES
386	a	2936.59	3.99629	YES	YES
387	a	2936.69	10.08623	YES	YES
388	a	2938.55	8.42888	YES	YES
389	a	2939.86	17.55450	YES	YES
390	a	2940.61	8.53310	YES	YES
391	a	2940.92	20.31790	YES	YES
392	a	2941.28	9.40348	YES	YES
393	a	2942.49	9.21289	YES	YES
394	a	2942.75	35.06665	YES	YES
395	a	2943.24	13.61662	YES	YES
396	a	2943.37	9.64351	YES	YES
397	a	2944.06	12.45966	YES	YES
398	a	2944.54	14.32974	YES	YES
399	a	2945.52	8.85303	YES	YES
400	a	2945.75	12.08495	YES	YES
401	a	2945.97	15.34049	YES	YES
402	a	2946.62	10.86743	YES	YES
403	a	2947.35	33.62176	YES	YES
404	a	2948.06	11.13053	YES	YES
405	a	2949.12	4.99893	YES	YES
406	a	2950.09	5.89848	YES	YES
407	a	2950.89	10.68869	YES	YES
408	a	2951.73	32.43566	YES	YES
409	a	2952.61	3.48920	YES	YES
410	a	2953.13	7.45483	YES	YES
411	a	2953.57	13.25456	YES	YES
412	a	2954.57	3.51624	YES	YES
413	a	2954.69	6.98526	YES	YES
414	a	2955.63	13.02183	YES	YES
415	a	2956.71	3.16816	YES	YES
416	a	2957.30	5.23603	YES	YES
417	a	2957.50	8.70705	YES	YES
418	a	2958.00	6.17297	YES	YES
419	a	2958.73	30.80892	YES	YES
420	a	2959.32	21.27013	YES	YES
421	a	2959.83	32.20574	YES	YES
422	a	2959.90	4.50024	YES	YES
423	a	2961.27	33.09142	YES	YES
424	a	2961.52	41.72176	YES	YES
425	a	2961.62	32.69811	YES	YES
426	a	2962.66	26.09935	YES	YES
427	a	2963.45	26.71109	YES	YES
428	a	2964.47	22.06157	YES	YES
429	a	2965.46	10.03753	YES	YES
430	a	2968.10	14.37950	YES	YES
431	a	2973.86	10.76785	YES	YES
432	a	2980.79	21.77811	YES	YES
433	a	2984.91	5.94967	YES	YES
434	a	2986.53	29.66956	YES	YES
435	a	2987.00	5.57897	YES	YES
436	a	2987.70	7.12929	YES	YES
437	a	2989.66	20.37846	YES	YES
438	a	2990.54	12.91760	YES	YES

439	a	2990.60	7.19401	YES	YES
440	a	2991.69	36.66699	YES	YES
441	a	2994.25	26.74557	YES	YES
442	a	2995.06	28.82181	YES	YES
443	a	2995.82	31.45671	YES	YES
444	a	2999.10	45.58245	YES	YES
445	a	2999.65	17.85049	YES	YES
446	a	3000.35	12.22830	YES	YES
447	a	3001.61	13.76796	YES	YES
448	a	3001.84	41.59052	YES	YES
449	a	3004.43	9.80673	YES	YES
450	a	3005.31	24.91836	YES	YES
451	a	3007.02	34.44019	YES	YES
452	a	3007.96	19.87315	YES	YES
453	a	3010.51	12.15011	YES	YES
454	a	3011.79	18.93922	YES	YES
455	a	3012.34	15.05369	YES	YES
456	a	3012.78	22.57406	YES	YES
457	a	3013.26	18.90103	YES	YES
458	a	3013.72	16.56635	YES	YES
459	a	3014.05	31.55844	YES	YES
460	a	3015.07	12.90009	YES	YES
461	a	3015.78	22.80124	YES	YES
462	a	3016.06	20.77391	YES	YES
463	a	3016.42	33.74918	YES	YES
464	a	3017.00	18.53257	YES	YES
465	a	3017.05	10.90581	YES	YES
466	a	3017.21	10.38385	YES	YES
467	a	3017.30	44.13823	YES	YES
468	a	3017.78	24.93726	YES	YES
469	a	3018.27	22.23148	YES	YES
470	a	3018.71	23.02440	YES	YES
471	a	3018.87	28.09711	YES	YES
472	a	3019.25	32.86639	YES	YES
473	a	3019.46	12.82211	YES	YES
474	a	3019.48	36.94312	YES	YES
475	a	3019.88	22.25176	YES	YES
476	a	3020.86	27.58072	YES	YES
477	a	3032.00	1.01280	YES	YES
478	a	3032.13	0.39360	YES	YES
479	a	3040.52	0.42455	YES	YES
480	a	3045.90	0.33397	YES	YES

$end

Double hybrid single point energy = -7364.706089042699 H
COSMO energy + OC correction = -7372.1518871473 H (in *o*DFB)

6.2.24 [H{Ga(dcpe)}$_2$(OMe)]$^{2+}$ (*anti*-periplanar)

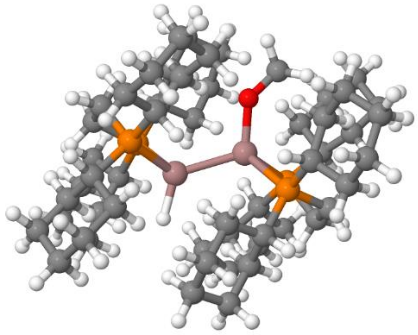

Method: (RI-)BP86(D3BJ)/def2-TZVPP
Symmetry: c1

Cartesian coordinates in Ångström:

Ga	-0.4456779	0.4751610	-1.8104128
P	-2.2353459	-0.8420844	-2.8723823
P	-1.7966751	2.4564738	-2.3957018
C	-1.7587144	-2.0742969	-4.1599087
H	-1.3510281	-2.9177435	-3.5759854
C	-0.6256943	-1.5382383	-5.0477971
H	-0.9667972	-0.6304669	-5.5747542
H	0.2234017	-1.2494053	-4.4151436
C	-0.1925927	-2.5891611	-6.0775198
H	0.5854898	-2.1668002	-6.7280748
H	0.2710701	-3.4363170	-5.5451355
C	-1.3768703	-3.0949919	-6.9058224
H	-1.0451573	-3.8723573	-7.6068071
H	-1.7735749	-2.2699854	-7.5206309
C	-2.4903900	-3.6365975	-6.0045981
H	-2.1276355	-4.5255948	-5.4629993
H	-3.3499829	-3.9647419	-6.6044044
C	-2.9517591	-2.5821529	-4.9883681
H	-3.7321568	-3.0015421	-4.3375978
H	-3.4051637	-1.7370133	-5.5307679
C	-3.3241518	-1.7480039	-1.6868659
H	-4.1089510	-2.2094650	-2.3114421
C	-2.5416942	-2.8651740	-0.9773913
H	-2.0906780	-3.5517815	-1.7071973
H	-1.7110675	-2.4093093	-0.4108421
C	-3.4504425	-3.6446598	-0.0196573
H	-4.2266215	-4.1633683	-0.6048933
H	-2.8698693	-4.4289059	0.4837965
C	-4.1151700	-2.7174309	1.0007003
H	-4.7810467	-3.2904623	1.6594861
H	-3.3436449	-2.2656731	1.6467694
C	-4.8927130	-1.6017543	0.2997817
H	-5.3293878	-0.9124576	1.0354866
H	-5.7358504	-2.0375266	-0.2601019
C	-4.0009383	-0.8145612	-0.6690207
H	-4.6116128	-0.0637003	-1.1875916
H	-3.2261155	-0.2763157	-0.0988598
C	-3.3057493	0.4148954	-3.7010487
H	-2.8632998	0.5768200	-4.6933178
H	-4.3067470	-0.0088827	-3.8629799
C	-3.4159795	1.7492463	-2.9411171

H	-4.0250436	1.6282971	-2.0372790
H	-3.9237732	2.4953513	-3.5681863
C	-1.2807140	3.5910350	-3.7566368
H	-2.1072782	4.3174100	-3.8447323
C	-0.0026286	4.3466396	-3.3639795
H	0.7847832	3.6173884	-3.1183263
H	-0.1789565	4.9371246	-2.4537856
C	0.4870920	5.2598952	-4.4931643
H	-0.2391942	6.0751079	-4.6421815
H	1.4316120	5.7341244	-4.1926515
C	0.6605578	4.4866884	-5.8027087
H	1.4569163	3.7332523	-5.6837828
H	0.9899400	5.1636548	-6.6017911
C	-0.6449736	3.7948787	-6.2035784
H	-0.5142975	3.2254628	-7.1338682
H	-1.4134457	4.5575522	-6.4090337
C	-1.1509924	2.8546604	-5.1011504
H	-0.4560181	2.0086023	-4.9940647
H	-2.1245523	2.4381162	-5.3960037
C	-2.1541614	3.5298694	-0.9339037
H	-1.1696867	3.9667497	-0.6925705
C	-2.6081123	2.7076418	0.2813451
H	-1.8903537	1.9017852	0.4925868
H	-3.5703525	2.2179477	0.0616378
C	-2.7749982	3.6039814	1.5129770
H	-3.1136402	2.9994036	2.3658358
H	-1.7900877	4.0179935	1.7899518
C	-3.7482702	4.7536852	1.2380921
H	-4.7563332	4.3419068	1.0665290
H	-3.8254231	5.4070494	2.1173978
C	-3.3133140	5.5639440	0.0135248
H	-2.3573203	6.0699022	0.2277413
H	-4.0425356	6.3556357	-0.2043078
C	-3.1382852	4.6755401	-1.2270256
H	-4.1146529	4.2535553	-1.5155626
H	-2.7903914	5.2853040	-2.0716541
Ga	-0.1466534	-0.0759411	0.5586913
P	1.4685320	1.3195417	1.6816957
P	1.2759277	-1.9414209	1.1126302
C	0.7725985	2.3860259	3.0204019
H	0.1491428	3.1151148	2.4727744
C	-0.1543688	1.5915276	3.9561453
H	0.4303017	0.8171709	4.4789764
H	-0.9254621	1.0691162	3.3730188
C	-0.7962639	2.5164999	4.9978593
H	-1.4320479	1.9249838	5.6708521
H	-1.4610364	3.2295870	4.4829474
C	0.2622481	3.2868222	5.7919321
H	-0.2187089	3.9683005	6.5061603
H	0.8577278	2.5777079	6.3904421
C	1.1909901	4.0685836	4.8592002
H	0.6158740	4.8531006	4.3398902
H	1.9735644	4.5833078	5.4327980
C	1.8447177	3.1545838	3.8126280
H	2.4765815	3.7524545	3.1416936
H	2.5070327	2.4350539	4.3211069
C	2.5110346	2.4123607	0.6288408
H	3.1857005	2.9230492	1.3379630

C	1.6623268	3.4746047	-0.0852303
H	1.0395847	4.0297043	0.6317328
H	0.9766637	2.9613899	-0.7822778
C	2.5627866	4.4419282	-0.8628419
H	3.1884354	4.9984761	-0.1467954
H	1.9482376	5.1906137	-1.3811164
C	3.4580746	3.6966859	-1.8562463
H	4.1304181	4.4014187	-2.3636189
H	2.8311676	3.2431532	-2.6405361
C	4.2683102	2.5941521	-1.1688342
H	4.8496624	2.0283106	-1.9095916
H	4.9988262	3.0522917	-0.4824426
C	3.3709137	1.6313700	-0.3781370
H	4.0030503	0.8993114	0.1432269
H	2.7141488	1.0763993	-1.0680240
C	2.6424939	0.1393329	2.4899869
H	2.2351343	-0.0590286	3.4890435
H	3.6080671	0.6442455	2.6330220
C	2.8449451	-1.1764578	1.7170735
H	3.4582065	-1.0013654	0.8242743
H	3.3831552	-1.9040062	2.3406435
C	0.6358442	-3.0015272	2.4802320
H	1.4334806	-3.7364100	2.6843733
C	-0.6266403	-3.7380705	2.0086394
H	-1.3739971	-2.9832177	1.7185504
H	-0.4125903	-4.3400659	1.1140333
C	-1.2124168	-4.6215518	3.1152023
H	-0.5035706	-5.4322600	3.3485909
H	-2.1296635	-5.1052067	2.7508701
C	-1.4973071	-3.8072580	4.3793450
H	-2.2822329	-3.0625940	4.1647875
H	-1.8904477	-4.4575879	5.1718007
C	-0.2335962	-3.0903737	4.8608309
H	-0.4481013	-2.4793680	5.7483822
H	0.5164867	-3.8367186	5.1685726
C	0.3631040	-2.1991067	3.7636412
H	-0.3373765	-1.3804581	3.5331363
H	1.2915380	-1.7417170	4.1322962
C	1.6894013	-3.0531695	-0.2988363
H	0.6937289	-3.3232813	-0.6961542
C	2.4446263	-2.3009496	-1.4056159
H	1.9460949	-1.3589659	-1.6729542
H	3.4533047	-2.0427016	-1.0438721
C	2.5828817	-3.1936358	-2.6444997
H	3.1289786	-2.6529923	-3.4298257
H	1.5766912	-3.4011164	-3.0489790
C	3.2874780	-4.5109699	-2.3048176
H	4.3304829	-4.2964686	-2.0198131
H	3.3358896	-5.1607367	-3.1891479
C	2.5886660	-5.2417686	-1.1534090
H	1.5897975	-5.5751168	-1.4819517
H	3.1441479	-6.1488915	-0.8798002
C	2.4242685	-4.3475329	0.0853470
H	3.4154525	-4.0952194	0.4959883
H	1.8833384	-4.8972090	0.8673423
H	-1.3856706	-0.2019092	1.5251757
O	1.1704618	0.3650755	-2.6845243
C	1.6657027	0.8898073	-3.8982912

```
H     2.6908815    0.5163962   -4.0425656
H     1.7085280    1.9910511   -3.8913881
H     1.0736735    0.5835860   -4.7763724

SCF energy GEOOPT =  -7372.010905670 H
ZPE =   3726. kJ/mol
FREEH energy =  3911.68 kJ/mol
FREEH entropy =   1.59300 kJ/mol/K

$vibrational spectrum
#  mode     symmetry     wave number   IR intensity    selection rules
#                         cm**(-1)        km/mol          IR     RAMAN
     1                      -0.00          0.00000         -       -
     2                      -0.00          0.00000         -       -
     3                      -0.00          0.00000         -       -
     4                       0.00          0.00000         -       -
     5                       0.00          0.00000         -       -
     6                       0.00          0.00000         -       -
     7         a             8.48          0.04936        YES     YES
     8         a            14.89          0.08854        YES     YES
     9         a            17.63          0.00701        YES     YES
    10         a            24.14          0.01466        YES     YES
    11         a            30.12          0.11460        YES     YES
    12         a            32.15          0.03317        YES     YES
    13         a            32.41          0.45213        YES     YES
    14         a            34.08          0.04856        YES     YES
    15         a            35.04          0.07867        YES     YES
    16         a            38.32          0.04036        YES     YES
    17         a            42.58          0.10129        YES     YES
    18         a            44.33          0.04744        YES     YES
    19         a            47.02          0.09557        YES     YES
    20         a            49.80          0.19612        YES     YES
    21         a            56.05          0.20461        YES     YES
    22         a            56.70          0.20916        YES     YES
    23         a            59.14          0.31883        YES     YES
    24         a            64.13          0.91898        YES     YES
    25         a            65.63          0.09683        YES     YES
    26         a            67.45          0.03034        YES     YES
    27         a            70.95          0.26819        YES     YES
    28         a            73.57          0.20976        YES     YES
    29         a            76.37          0.41931        YES     YES
    30         a            78.00          0.52739        YES     YES
    31         a            80.61          0.41129        YES     YES
    32         a            84.27          0.86956        YES     YES
    33         a            87.92          0.52482        YES     YES
    34         a            89.22          0.38833        YES     YES
    35         a            99.21          0.39471        YES     YES
    36         a           109.00          4.66238        YES     YES
    37         a           117.76          6.29863        YES     YES
    38         a           127.21          0.90162        YES     YES
    39         a           128.70          2.85107        YES     YES
    40         a           135.16          3.41814        YES     YES
    41         a           136.93          0.32856        YES     YES
    42         a           139.85          7.48980        YES     YES
    43         a           143.64          2.44170        YES     YES
    44         a           148.10          2.03261        YES     YES
    45         a           150.34          1.95029        YES     YES
    46         a           151.46          0.04919        YES     YES
```

47	a	156.33	0.12454	YES	YES
48	a	163.50	1.20178	YES	YES
49	a	172.60	4.15603	YES	YES
50	a	180.10	3.61001	YES	YES
51	a	181.84	3.40159	YES	YES
52	a	186.14	0.07775	YES	YES
53	a	192.51	1.01204	YES	YES
54	a	200.99	0.60121	YES	YES
55	a	202.61	1.69135	YES	YES
56	a	207.52	3.81194	YES	YES
57	a	208.63	1.94701	YES	YES
58	a	213.59	0.09578	YES	YES
59	a	215.36	0.29438	YES	YES
60	a	216.45	0.43551	YES	YES
61	a	223.99	2.34974	YES	YES
62	a	230.04	0.71904	YES	YES
63	a	232.70	1.24403	YES	YES
64	a	234.79	3.51414	YES	YES
65	a	236.58	0.36540	YES	YES
66	a	238.64	1.09283	YES	YES
67	a	240.09	1.00109	YES	YES
68	a	244.00	0.01874	YES	YES
69	a	247.35	0.14879	YES	YES
70	a	248.16	2.46622	YES	YES
71	a	272.68	11.91526	YES	YES
72	a	277.39	2.90355	YES	YES
73	a	279.50	2.39438	YES	YES
74	a	287.19	0.39450	YES	YES
75	a	293.22	1.48567	YES	YES
76	a	305.27	0.41876	YES	YES
77	a	307.19	0.20962	YES	YES
78	a	313.99	0.11213	YES	YES
79	a	315.23	0.39370	YES	YES
80	a	329.53	0.62912	YES	YES
81	a	331.93	0.60798	YES	YES
82	a	335.15	0.26313	YES	YES
83	a	335.57	0.17789	YES	YES
84	a	342.78	0.66073	YES	YES
85	a	344.80	0.72129	YES	YES
86	a	370.02	1.34196	YES	YES
87	a	373.24	2.77780	YES	YES
88	a	375.30	2.08002	YES	YES
89	a	377.67	1.51988	YES	YES
90	a	394.19	0.15461	YES	YES
91	a	398.85	0.00869	YES	YES
92	a	411.93	1.17359	YES	YES
93	a	414.34	1.60562	YES	YES
94	a	422.66	6.25326	YES	YES
95	a	423.34	0.02725	YES	YES
96	a	424.97	1.34288	YES	YES
97	a	427.10	1.00235	YES	YES
98	a	429.81	0.07470	YES	YES
99	a	430.50	0.00912	YES	YES
100	a	431.10	0.48510	YES	YES
101	a	431.73	0.19035	YES	YES
102	a	432.50	0.72559	YES	YES
103	a	433.45	0.37911	YES	YES
104	a	434.13	0.93738	YES	YES

105	a	434.85	0.57315	YES	YES
106	a	443.25	4.30902	YES	YES
107	a	450.32	4.24208	YES	YES
108	a	467.15	1.72318	YES	YES
109	a	470.16	3.69328	YES	YES
110	a	489.71	2.73789	YES	YES
111	a	491.47	7.69657	YES	YES
112	a	493.06	2.89303	YES	YES
113	a	494.74	4.11827	YES	YES
114	a	498.92	17.09237	YES	YES
115	a	503.55	4.83682	YES	YES
116	a	505.25	4.18255	YES	YES
117	a	518.08	12.02334	YES	YES
118	a	519.43	17.77384	YES	YES
119	a	564.14	79.00738	YES	YES
120	a	579.52	114.85975	YES	YES
121	a	627.74	26.95999	YES	YES
122	a	628.61	2.45451	YES	YES
123	a	643.75	7.89807	YES	YES
124	a	645.30	9.45183	YES	YES
125	a	713.06	0.34076	YES	YES
126	a	716.13	1.11749	YES	YES
127	a	719.26	0.59804	YES	YES
128	a	724.59	0.91099	YES	YES
129	a	733.07	5.94769	YES	YES
130	a	735.41	10.00391	YES	YES
131	a	736.07	4.59507	YES	YES
132	a	736.18	4.57963	YES	YES
133	a	771.67	0.25308	YES	YES
134	a	772.21	0.24413	YES	YES
135	a	773.05	0.24319	YES	YES
136	a	773.44	0.50548	YES	YES
137	a	775.58	0.16187	YES	YES
138	a	775.99	0.46494	YES	YES
139	a	776.71	0.24165	YES	YES
140	a	778.28	0.19455	YES	YES
141	a	783.21	15.88498	YES	YES
142	a	785.69	8.10021	YES	YES
143	a	810.19	0.03639	YES	YES
144	a	810.34	0.76956	YES	YES
145	a	812.05	2.44689	YES	YES
146	a	812.36	0.47558	YES	YES
147	a	813.45	1.02449	YES	YES
148	a	814.23	0.99565	YES	YES
149	a	815.83	3.99690	YES	YES
150	a	816.49	7.97624	YES	YES
151	a	832.89	0.91490	YES	YES
152	a	833.97	0.39487	YES	YES
153	a	836.12	1.83532	YES	YES
154	a	837.07	1.34516	YES	YES
155	a	837.22	4.09565	YES	YES
156	a	837.54	5.98211	YES	YES
157	a	838.04	15.20416	YES	YES
158	a	839.19	7.10155	YES	YES
159	a	852.80	15.60341	YES	YES
160	a	854.41	2.91697	YES	YES
161	a	875.39	0.19507	YES	YES
162	a	875.58	0.21919	YES	YES

163	a	876.08	6.18139	YES	YES
164	a	876.79	4.60052	YES	YES
165	a	877.33	7.45190	YES	YES
166	a	877.86	2.75848	YES	YES
167	a	878.46	3.15316	YES	YES
168	a	879.72	0.18525	YES	YES
169	a	880.03	2.12936	YES	YES
170	a	880.30	5.89256	YES	YES
171	a	880.48	2.47176	YES	YES
172	a	881.97	1.52068	YES	YES
173	a	883.43	1.57286	YES	YES
174	a	884.85	1.13420	YES	YES
175	a	886.51	5.55840	YES	YES
176	a	888.01	0.20411	YES	YES
177	a	902.91	0.55783	YES	YES
178	a	903.81	3.64716	YES	YES
179	a	904.19	3.30892	YES	YES
180	a	905.04	0.92465	YES	YES
181	a	908.39	0.70486	YES	YES
182	a	908.97	4.14596	YES	YES
183	a	910.39	0.27488	YES	YES
184	a	911.15	1.39096	YES	YES
185	a	983.49	0.23413	YES	YES
186	a	984.98	0.34548	YES	YES
187	a	987.58	5.13368	YES	YES
188	a	987.75	5.40778	YES	YES
189	a	988.62	19.67645	YES	YES
190	a	989.98	11.92508	YES	YES
191	a	990.55	5.00946	YES	YES
192	a	990.66	14.67510	YES	YES
193	a	992.44	9.35783	YES	YES
194	a	993.36	2.82774	YES	YES
195	a	1016.02	0.26948	YES	YES
196	a	1016.19	1.42591	YES	YES
197	a	1016.69	1.38422	YES	YES
198	a	1016.97	0.15075	YES	YES
199	a	1018.64	1.09626	YES	YES
200	a	1020.81	0.96486	YES	YES
201	a	1021.58	0.31734	YES	YES
202	a	1021.95	1.46412	YES	YES
203	a	1031.12	2.23470	YES	YES
204	a	1032.02	1.57347	YES	YES
205	a	1033.16	1.57832	YES	YES
206	a	1034.52	2.31257	YES	YES
207	a	1038.84	2.73899	YES	YES
208	a	1039.78	1.09809	YES	YES
209	a	1040.45	1.36614	YES	YES
210	a	1042.04	0.60756	YES	YES
211	a	1059.15	0.60549	YES	YES
212	a	1059.99	2.89478	YES	YES
213	a	1060.44	0.83555	YES	YES
214	a	1061.00	1.37822	YES	YES
215	a	1064.72	3.59641	YES	YES
216	a	1067.05	1.50990	YES	YES
217	a	1068.48	0.70942	YES	YES
218	a	1069.48	0.34745	YES	YES
219	a	1070.31	0.52054	YES	YES
220	a	1071.44	0.15166	YES	YES

221	a	1072.02	44.90064	YES	YES
222	a	1072.41	13.36897	YES	YES
223	a	1072.90	75.99447	YES	YES
224	a	1074.11	5.14944	YES	YES
225	a	1074.56	7.59259	YES	YES
226	a	1074.98	1.43866	YES	YES
227	a	1077.34	0.19523	YES	YES
228	a	1082.77	2.43195	YES	YES
229	a	1084.85	3.02284	YES	YES
230	a	1093.80	9.81462	YES	YES
231	a	1095.76	3.61062	YES	YES
232	a	1095.99	8.10579	YES	YES
233	a	1099.31	2.20477	YES	YES
234	a	1103.23	1.56940	YES	YES
235	a	1105.23	3.80943	YES	YES
236	a	1107.45	5.95845	YES	YES
237	a	1108.86	7.85456	YES	YES
238	a	1127.74	0.34359	YES	YES
239	a	1130.00	4.58258	YES	YES
240	a	1132.14	0.69019	YES	YES
241	a	1144.19	5.85046	YES	YES
242	a	1160.52	2.05328	YES	YES
243	a	1160.59	14.92733	YES	YES
244	a	1161.48	2.15558	YES	YES
245	a	1161.89	1.06560	YES	YES
246	a	1170.30	6.69928	YES	YES
247	a	1171.26	1.38852	YES	YES
248	a	1172.20	26.52885	YES	YES
249	a	1172.64	1.66165	YES	YES
250	a	1178.07	20.37216	YES	YES
251	a	1180.26	11.37091	YES	YES
252	a	1182.40	4.21784	YES	YES
253	a	1187.74	8.54784	YES	YES
254	a	1188.98	0.47458	YES	YES
255	a	1190.39	3.05919	YES	YES
256	a	1193.75	1.17300	YES	YES
257	a	1194.53	5.50021	YES	YES
258	a	1242.58	0.15355	YES	YES
259	a	1243.50	0.05296	YES	YES
260	a	1244.12	1.33110	YES	YES
261	a	1245.01	0.20438	YES	YES
262	a	1245.58	0.25960	YES	YES
263	a	1246.27	0.45539	YES	YES
264	a	1246.95	0.44573	YES	YES
265	a	1247.92	0.36416	YES	YES
266	a	1249.43	0.36404	YES	YES
267	a	1250.71	0.26971	YES	YES
268	a	1254.05	1.65393	YES	YES
269	a	1256.56	8.76748	YES	YES
270	a	1256.76	2.10783	YES	YES
271	a	1256.97	2.43015	YES	YES
272	a	1257.55	6.47632	YES	YES
273	a	1257.87	3.06161	YES	YES
274	a	1258.44	0.86861	YES	YES
275	a	1258.66	3.01071	YES	YES
276	a	1258.93	3.50932	YES	YES
277	a	1259.47	2.07721	YES	YES
278	a	1260.20	8.61272	YES	YES

279	a	1261.56	2.97457	YES	YES
280	a	1262.99	0.38052	YES	YES
281	a	1264.48	1.16801	YES	YES
282	a	1266.00	4.54454	YES	YES
283	a	1267.20	0.72726	YES	YES
284	a	1269.87	2.28319	YES	YES
285	a	1271.11	0.33297	YES	YES
286	a	1280.51	2.24386	YES	YES
287	a	1282.86	2.33042	YES	YES
288	a	1284.62	0.88715	YES	YES
289	a	1287.15	3.92920	YES	YES
290	a	1288.32	5.61404	YES	YES
291	a	1290.44	3.22668	YES	YES
292	a	1291.60	4.85978	YES	YES
293	a	1293.66	2.18734	YES	YES
294	a	1309.13	1.00233	YES	YES
295	a	1309.94	0.86131	YES	YES
296	a	1310.70	3.20264	YES	YES
297	a	1310.82	3.31541	YES	YES
298	a	1312.70	0.43581	YES	YES
299	a	1312.77	2.22061	YES	YES
300	a	1316.44	0.82495	YES	YES
301	a	1317.46	0.75823	YES	YES
302	a	1320.73	0.18566	YES	YES
303	a	1321.90	0.25552	YES	YES
304	a	1322.76	3.42756	YES	YES
305	a	1323.38	0.43111	YES	YES
306	a	1323.70	2.03511	YES	YES
307	a	1324.25	0.43172	YES	YES
308	a	1324.93	1.64472	YES	YES
309	a	1325.47	3.35901	YES	YES
310	a	1325.86	0.80964	YES	YES
311	a	1326.19	0.30624	YES	YES
312	a	1326.53	0.43513	YES	YES
313	a	1327.01	8.29842	YES	YES
314	a	1327.24	0.96900	YES	YES
315	a	1327.62	3.59859	YES	YES
316	a	1330.10	2.66957	YES	YES
317	a	1336.18	0.08726	YES	YES
318	a	1336.76	0.10420	YES	YES
319	a	1337.40	0.33438	YES	YES
320	a	1338.35	0.96582	YES	YES
321	a	1338.66	0.20803	YES	YES
322	a	1339.11	0.55096	YES	YES
323	a	1339.41	0.29832	YES	YES
324	a	1340.85	1.28279	YES	YES
325	a	1340.97	0.44649	YES	YES
326	a	1345.22	2.30700	YES	YES
327	a	1347.49	1.06358	YES	YES
328	a	1347.89	2.52613	YES	YES
329	a	1348.22	0.54960	YES	YES
330	a	1348.49	1.48530	YES	YES
331	a	1350.33	2.86205	YES	YES
332	a	1350.47	0.20187	YES	YES
333	a	1351.21	0.42429	YES	YES
334	a	1400.87	7.17604	YES	YES
335	a	1402.89	8.90374	YES	YES
336	a	1405.50	7.82089	YES	YES

337	a	1407.94	7.15514	YES	YES
338	a	1421.57	4.03511	YES	YES
339	a	1426.49	1.99104	YES	YES
340	a	1427.95	3.88018	YES	YES
341	a	1428.84	8.31541	YES	YES
342	a	1429.50	2.28813	YES	YES
343	a	1430.23	1.45651	YES	YES
344	a	1432.11	3.29822	YES	YES
345	a	1432.51	1.85403	YES	YES
346	a	1434.67	4.41009	YES	YES
347	a	1435.16	8.93614	YES	YES
348	a	1435.45	6.46889	YES	YES
349	a	1436.10	1.10577	YES	YES
350	a	1436.68	1.76644	YES	YES
351	a	1436.84	3.80849	YES	YES
352	a	1437.71	4.15213	YES	YES
353	a	1437.80	3.53365	YES	YES
354	a	1438.29	1.87945	YES	YES
355	a	1438.60	3.24384	YES	YES
356	a	1439.01	5.02453	YES	YES
357	a	1439.25	9.76717	YES	YES
358	a	1439.86	3.74856	YES	YES
359	a	1440.51	11.82070	YES	YES
360	a	1440.74	4.28634	YES	YES
361	a	1440.82	5.43397	YES	YES
362	a	1442.20	10.63878	YES	YES
363	a	1442.89	31.49450	YES	YES
364	a	1443.31	8.19390	YES	YES
365	a	1443.49	8.90984	YES	YES
366	a	1444.27	10.76263	YES	YES
367	a	1444.98	8.89636	YES	YES
368	a	1446.65	4.41889	YES	YES
369	a	1446.91	14.43772	YES	YES
370	a	1447.47	35.92700	YES	YES
371	a	1449.80	6.29901	YES	YES
372	a	1452.61	2.13894	YES	YES
373	a	1453.15	1.65668	YES	YES
374	a	1454.21	1.90209	YES	YES
375	a	1454.89	1.75235	YES	YES
376	a	1455.85	1.95044	YES	YES
377	a	1456.53	1.07876	YES	YES
378	a	1458.08	1.86265	YES	YES
379	a	1462.04	2.34049	YES	YES
380	a	1465.52	3.24180	YES	YES
381	a	1862.36	115.80736	YES	YES
382	a	2918.40	79.56815	YES	YES
383	a	2921.15	2.04653	YES	YES
384	a	2925.03	16.82508	YES	YES
385	a	2927.27	11.26665	YES	YES
386	a	2929.14	15.85301	YES	YES
387	a	2930.33	2.65863	YES	YES
388	a	2931.31	9.28574	YES	YES
389	a	2934.48	18.96584	YES	YES
390	a	2938.15	20.14765	YES	YES
391	a	2939.45	12.18412	YES	YES
392	a	2939.76	4.20860	YES	YES
393	a	2941.54	6.89997	YES	YES
394	a	2941.67	3.52231	YES	YES

395	a	2941.71	4.63965	YES	YES
396	a	2941.98	14.16840	YES	YES
397	a	2942.64	8.18397	YES	YES
398	a	2943.32	6.09396	YES	YES
399	a	2944.53	7.34908	YES	YES
400	a	2945.02	10.08823	YES	YES
401	a	2946.21	8.89297	YES	YES
402	a	2946.28	12.66457	YES	YES
403	a	2946.34	12.73293	YES	YES
404	a	2948.52	3.36777	YES	YES
405	a	2950.45	20.88611	YES	YES
406	a	2951.88	10.44980	YES	YES
407	a	2952.36	15.56598	YES	YES
408	a	2952.97	18.67266	YES	YES
409	a	2953.96	14.73074	YES	YES
410	a	2954.14	1.60568	YES	YES
411	a	2955.21	2.07571	YES	YES
412	a	2955.61	10.66285	YES	YES
413	a	2956.00	5.45646	YES	YES
414	a	2956.13	3.67740	YES	YES
415	a	2956.35	5.69902	YES	YES
416	a	2957.69	7.33503	YES	YES
417	a	2958.28	4.63235	YES	YES
418	a	2958.55	2.23462	YES	YES
419	a	2960.16	1.40410	YES	YES
420	a	2960.25	65.26874	YES	YES
421	a	2960.83	14.25147	YES	YES
422	a	2961.12	23.53239	YES	YES
423	a	2961.21	56.95752	YES	YES
424	a	2961.53	25.98866	YES	YES
425	a	2962.02	30.78283	YES	YES
426	a	2963.88	36.47480	YES	YES
427	a	2964.30	54.87648	YES	YES
428	a	2964.94	15.74581	YES	YES
429	a	2965.32	52.44659	YES	YES
430	a	2968.88	37.72457	YES	YES
431	a	2973.46	16.41816	YES	YES
432	a	2982.42	6.59198	YES	YES
433	a	2986.66	5.44345	YES	YES
434	a	2989.82	3.62703	YES	YES
435	a	2989.92	2.19456	YES	YES
436	a	2991.50	25.92342	YES	YES
437	a	2992.18	25.18393	YES	YES
438	a	2995.34	36.88628	YES	YES
439	a	3001.70	23.95635	YES	YES
440	a	3004.34	17.30285	YES	YES
441	a	3004.51	34.44536	YES	YES
442	a	3004.92	8.57257	YES	YES
443	a	3005.09	26.38593	YES	YES
444	a	3005.93	6.31548	YES	YES
445	a	3006.64	23.80757	YES	YES
446	a	3006.97	35.70381	YES	YES
447	a	3007.60	13.24434	YES	YES
448	a	3008.05	19.98264	YES	YES
449	a	3008.48	22.42441	YES	YES
450	a	3009.60	12.59321	YES	YES
451	a	3009.92	8.12357	YES	YES
452	a	3010.32	21.98652	YES	YES

453	a	3010.95	20.11791	YES	YES
454	a	3011.19	10.47783	YES	YES
455	a	3011.26	16.97647	YES	YES
456	a	3011.84	22.26582	YES	YES
457	a	3012.02	24.11317	YES	YES
458	a	3012.28	16.49707	YES	YES
459	a	3012.55	31.16123	YES	YES
460	a	3013.05	24.86589	YES	YES
461	a	3013.59	28.58590	YES	YES
462	a	3014.72	21.39237	YES	YES
463	a	3015.12	31.64343	YES	YES
464	a	3015.55	33.91429	YES	YES
465	a	3015.95	20.18911	YES	YES
466	a	3016.48	23.11119	YES	YES
467	a	3016.98	11.37011	YES	YES
468	a	3017.02	19.62455	YES	YES
469	a	3017.52	28.36143	YES	YES
470	a	3017.67	42.71655	YES	YES
471	a	3018.09	37.43928	YES	YES
472	a	3018.32	13.23608	YES	YES
473	a	3018.51	22.18842	YES	YES
474	a	3019.13	29.52022	YES	YES
475	a	3020.56	30.76140	YES	YES
476	a	3023.59	8.75858	YES	YES
477	a	3029.02	0.13199	YES	YES
478	a	3033.76	0.07636	YES	YES
479	a	3046.82	0.79962	YES	YES
480	a	3047.73	0.20685	YES	YES

$end

Double hybrid single point energy = -7364.706635170493 H
COSMO energy + OC correction = -7372.1585533757 H (in *o*DFB)

6.2.25 HNMe₂

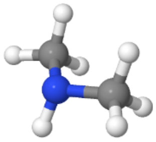

Method: (RI-)BP86(D3BJ)/def2-TZVPP
Symmetry: cs

Cartesian coordinates in Ångström:

C	-0.0969023	0.0657129	-1.2133541
N	0.7117511	0.1035964	0.0000000
H	-0.8018585	-0.7916757	-1.2628324
H	0.5560872	0.0278495	-2.0950110
H	-0.6952222	0.9865342	-1.2801125
H	1.3639130	-0.6805431	0.0000000
C	-0.0969023	0.0657129	1.2133541
H	-0.6952222	0.9865342	1.2801125
H	0.5560872	0.0278495	2.0950110
H	-0.8018585	-0.7916757	1.2628324

SCF energy GEOOPT = -135.2311371841 H

ZPE =   235.2 kJ/mol
FREEH energy =   246.91 kJ/mol
FREEH entropy =   0.27271 kJ/mol/K

$vibrational spectrum

# mode	symmetry	wave number	IR intensity	selection rules	
#		cm**(-1)	km/mol	IR	RAMAN
1		-0.00	0.00000	-	-
2		-0.00	0.00000	-	-
3		-0.00	0.00000	-	-
4		-0.00	0.00000	-	-
5		0.00	0.00000	-	-
6		0.00	0.00000	-	-
7	a"	233.23	0.11153	YES	YES
8	a'	254.62	2.36465	YES	YES
9	a'	370.40	5.64611	YES	YES
10	a'	746.47	113.48381	YES	YES
11	a'	924.45	2.82346	YES	YES
12	a"	1000.70	7.68542	YES	YES
13	a"	1066.51	0.34888	YES	YES
14	a"	1138.20	30.28528	YES	YES
15	a'	1145.76	6.44304	YES	YES
16	a'	1227.54	0.05264	YES	YES
17	a"	1392.92	1.73733	YES	YES
18	a'	1418.20	0.97813	YES	YES
19	a"	1424.37	5.05181	YES	YES
20	a"	1437.53	4.00793	YES	YES
21	a'	1443.98	11.31507	YES	YES
22	a"	1468.85	11.50502	YES	YES
23	a'	1469.53	2.04840	YES	YES
24	a"	2846.93	54.30767	YES	YES
25	a'	2850.24	171.27428	YES	YES
26	a'	2977.24	48.55393	YES	YES
27	a"	2979.11	23.72549	YES	YES
28	a"	3031.76	25.87661	YES	YES
29	a'	3032.00	31.57215	YES	YES
30	a'	3436.51	0.02461	YES	YES

$end

Double hybrid single point energy = -134.984427786201 H
COSMO energy + OC correction = -135.2358620943 H (in *o*DFB)

6.2.26 $[\{Ga(dcpe)\}_2(HNMe_2)]^{2+}$

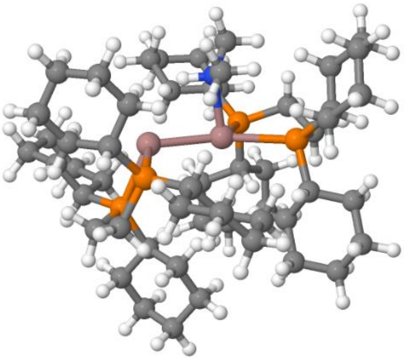

Method: (RI-)BP86(D3BJ)/def2-TZVPP
Symmetry: c1

Cartesian coordinates in Ångström:

Ga	-1.7258303	17.0346588	-2.2548376
Ga	-3.9345195	17.8363015	-1.2072985
P	-1.3018074	18.6806405	-4.1041886
P	-6.0566986	19.2243020	-0.7583028
P	-2.0167654	15.5039435	-4.1996961
P	-5.1097076	16.2387197	0.2998412
C	-4.6636462	21.1014218	-4.7965196
H	-4.6712646	21.6470233	-3.8372245
H	-5.7058399	20.8218118	-5.0108186
C	-1.0777293	13.9734356	-3.7411925
H	-0.9829010	13.3531276	-4.6472682
C	-4.1592851	15.2541655	1.5436604
H	-3.9549393	16.0018511	2.3316039
C	-4.3741265	16.0806758	-5.5953406
H	-3.7742403	16.5140707	-6.4117251
H	-4.5243699	16.8773710	-4.8505465
C	-6.9420654	19.3557229	-2.3813662
H	-6.2852125	20.0115562	-2.9763764
C	-7.1394183	18.1474796	0.3090928
H	-7.8130082	18.7946260	0.8870580
H	-7.7737444	17.5507401	-0.3600825
C	-2.6570585	22.3748407	-5.6460601
H	-2.2662104	22.9986278	-6.4612472
H	-2.5754470	22.9762975	-4.7253312
C	-1.7896169	21.1169763	-5.4999210
H	-0.7515369	21.4054705	-5.2883717
H	-1.7802111	20.5684102	-6.4557443
C	-3.8081291	19.8382793	-4.6461783
H	-3.8899301	19.2416050	-5.5689959
H	-4.1778217	19.2005967	-3.8270061
C	1.0577316	13.0450180	-2.7314234
H	1.2331125	12.3710206	-3.5856910
H	2.0476558	13.3217700	-2.3435788
C	-7.3748992	14.5247761	-0.2193446
H	-8.0158411	15.2852999	0.2486968
H	-7.0956819	13.8172042	0.5730753
C	-6.2430421	20.9139113	-0.0079540
H	-7.3358776	21.0704668	0.0115477
C	0.5193571	20.1412860	-2.5892033
H	-0.1626289	21.0030191	-2.6446664
H	0.1869005	19.5160713	-1.7410225
C	1.9520998	20.6218696	-2.3285908
H	2.2518832	21.3161212	-3.1302751
H	1.9848787	21.1950559	-1.3914020
C	-7.0158261	18.0007806	-3.1017944
H	-6.0101192	17.5529037	-3.1770917
H	-7.6356489	17.3052804	-2.5115025
C	-4.1250512	22.0212728	-5.8962138
H	-4.2185719	21.5138292	-6.8704099
H	-4.7322354	22.9343032	-5.9611884
C	-8.3323504	20.0081407	-2.2936417
H	-8.2727066	20.9961189	-1.8192458
H	-8.9895064	19.3885301	-1.6612754
C	-5.6200469	22.0052234	-0.8924088
H	-6.0480654	21.9687633	-1.9038005
H	-4.5399280	21.8181951	-1.0053041

C	-3.4118967	13.7583095	-5.9636316
H	-2.9120357	12.9074500	-5.4823441
H	-2.7573984	14.0872090	-6.7873727
C	2.8544693	18.6145548	-3.5610715
H	3.1952216	19.2243467	-4.4136278
H	3.5316315	17.7512066	-3.5044358
C	-5.9872327	22.3873747	2.0285642
H	-7.0719883	22.5687972	2.0957925
H	-5.5992464	22.4276866	3.0554697
C	-9.0229481	18.8079469	-4.4174948
H	-9.4347156	18.9409906	-5.4268681
H	-9.7173552	18.1379467	-3.8837573
C	-4.9226169	14.0942173	2.2070844
H	-5.8784716	14.4409101	2.6241301
H	-5.1582569	13.3335466	1.4476586
C	0.2453508	12.3100875	-1.6618409
H	0.7717574	11.4019968	-1.3389847
H	0.1506649	12.9542539	-0.7708259
C	-7.6436501	18.1482089	-4.4924029
H	-6.9748274	18.7542153	-5.1254568
H	-7.7165846	17.1617933	-4.9670891
C	2.9312443	19.4459392	-2.2779231
H	2.6892770	18.8057689	-1.4127423
H	3.9553446	19.8100185	-2.1214245
C	-5.3391371	23.4706576	1.1610913
H	-5.5415511	24.4643731	1.5817278
H	-4.2429299	23.3448801	1.1765445
C	-5.2287352	14.1263162	-1.5291599
H	-4.8479503	13.4005059	-0.7951665
H	-4.3487985	14.6218762	-1.9670670
C	-5.7448011	20.9904377	1.4415807
H	-4.6660562	20.7773283	1.4716754
H	-6.2388052	20.2278938	2.0623864
C	-6.1132204	15.1645905	-0.8212628
H	-6.4421295	15.8761942	-1.5990847
C	-5.8418817	23.3965063	-0.2835052
C	-7.2916342	12.7414612	-2.0191097
H	-7.8654150	12.2431725	-2.8118593
H	-7.0038969	11.9562042	-1.3011827
C	-8.1611882	13.7844342	-1.3104164
H	-9.0479032	13.3112546	-0.8677904
H	-8.5315303	14.5152711	-2.0493727
C	-1.8882077	13.2065667	-2.6810992
H	-2.8746681	12.9267080	-3.0780843
H	-2.0665751	13.8858408	-1.8288714
C	-4.0622273	13.4551247	3.3067663
H	-3.9005154	14.1901710	4.1123622
H	-4.6097597	12.6157489	3.7563329
C	-5.7121758	15.6111829	-6.1779646
H	-6.2237097	16.4583376	-6.6548299
H	-6.3633660	15.2697033	-5.3556444
C	-2.7100727	12.9855533	2.7608974
H	-2.8733870	12.1723589	2.0341479
H	-2.1014309	12.5612169	3.5703369
C	-2.8076455	14.7794411	0.9784597
H	-2.9896257	14.0445611	0.1797304
H	-2.2651719	15.6122282	0.5026945
C	-8.9549353	20.1514179	-3.6892758

```
H    -9.9557320   20.5948791   -3.5999774
H    -8.3503210   20.8602812   -4.2788366
C    -4.7588282   13.3039913   -6.5436167
H    -4.5895018   12.5052272   -7.2783299
H    -5.3674741   12.8638839   -5.7368890
C    -6.0294969   13.3826468   -2.6029756
H    -5.3952645   12.6217010   -3.0807292
H    -6.3180658   14.0953165   -3.3938698
C    -5.5202854   14.4690201   -7.1789011
H    -6.4930749   14.1282013   -7.5582316
H    -4.9586102   14.8412432   -8.0516221
C    -1.1499341   11.9593155   -2.1837630
H    -1.7460578   11.4670086   -1.4020180
H    -1.0617709   11.2361599   -3.0105809
C    -1.9575747   14.1309668    2.0758259
H    -1.6891533   14.8941879    2.8255882
H    -1.0128583   13.7713817    1.6447101
H    -1.8130001   18.1814262    0.0162343
C     0.4286085   19.2981747   -3.8711689
H     0.6693420   19.9328191   -4.7400671
C     1.4257478   18.1267159   -3.8310543
H     1.3996828   17.5712079   -4.7791534
H     1.1199555   17.4244427   -3.0353572
C     0.3311927   14.3047204   -3.2185295
H     0.9282611   14.8003159   -3.9967207
H     0.2421706   15.0207482   -2.3822381
C    -1.0497315   16.2744722   -5.5811737
H     0.0058985   16.0693013   -5.3655927
H    -1.2873768   15.7544559   -6.5197085
C    -1.2776776   17.7858443   -5.7284655
H    -2.2499141   17.9799542   -6.1987553
H    -0.5105993   18.2267576   -6.3810510
C    -3.6023469   14.9114381   -4.9641203
H    -4.1925092   14.5398522   -4.1124734
C    -2.3359437   20.1982038   -4.3938466
H    -2.2853522   20.7428906   -3.4338254
C    -6.3437210   17.2289090    1.2460147
H    -7.0199679   16.5683500    1.8065507
H    -5.7795946   17.8162439    1.9843082
H    -6.9187837   23.6292934   -0.3072982
H    -5.3463064   24.1531690   -0.9069612
C    -2.2815466   20.1569621   -0.1048142
N    -2.6081191   18.7754133    0.3084661
H    -2.1481779   20.1839051   -1.1929324
H    -1.3603085   20.5130807    0.3776814
H    -3.1039693   20.8249254    0.1666805
C    -2.7579142   18.6452042    1.7724935
H    -3.7030645   19.0921368    2.0966380
H    -1.9334517   19.1461964    2.3014893
H    -2.7504981   17.5851036    2.0415767

SCF energy GEOOPT =  -7391.428226926 H
ZPE =   3841. kJ/mol
FREEH energy =  4030.99 kJ/mol
FREEH entropy =   1.61213 kJ/mol/K

$vibrational spectrum
#  mode     symmetry     wave number   IR intensity    selection rules
```

#		cm**(-1)	km/mol	IR	RAMAN
1		-0.00	0.00000	-	-
2		-0.00	0.00000	-	-
3		0.00	0.00000	-	-
4		0.00	0.00000	-	-
5		0.00	0.00000	-	-
6		0.00	0.00000	-	-
7	a	18.66	0.14979	YES	YES
8	a	22.34	0.01017	YES	YES
9	a	25.53	0.05268	YES	YES
10	a	28.75	0.05300	YES	YES
11	a	29.59	0.04876	YES	YES
12	a	30.42	0.19426	YES	YES
13	a	33.19	0.46063	YES	YES
14	a	34.94	0.12526	YES	YES
15	a	36.76	0.36551	YES	YES
16	a	43.11	0.05738	YES	YES
17	a	44.44	0.09375	YES	YES
18	a	48.16	0.07110	YES	YES
19	a	49.86	0.14801	YES	YES
20	a	51.71	0.06836	YES	YES
21	a	56.48	0.04133	YES	YES
22	a	57.63	0.14580	YES	YES
23	a	61.94	0.17619	YES	YES
24	a	63.75	0.25773	YES	YES
25	a	65.92	0.20222	YES	YES
26	a	67.34	0.08439	YES	YES
27	a	71.38	0.00582	YES	YES
28	a	72.04	0.91230	YES	YES
29	a	76.60	0.41810	YES	YES
30	a	76.68	0.29042	YES	YES
31	a	77.96	0.03560	YES	YES
32	a	80.74	0.08201	YES	YES
33	a	86.42	0.24066	YES	YES
34	a	89.69	0.45159	YES	YES
35	a	97.38	0.09045	YES	YES
36	a	98.97	0.15795	YES	YES
37	a	101.45	0.21573	YES	YES
38	a	113.25	0.58810	YES	YES
39	a	116.94	1.68843	YES	YES
40	a	123.79	1.35533	YES	YES
41	a	128.04	0.88674	YES	YES
42	a	128.55	0.05934	YES	YES
43	a	134.32	0.78123	YES	YES
44	a	134.66	2.04414	YES	YES
45	a	138.01	0.75412	YES	YES
46	a	142.88	1.56577	YES	YES
47	a	146.39	0.35021	YES	YES
48	a	148.65	3.65529	YES	YES
49	a	169.93	0.43328	YES	YES
50	a	173.87	1.15587	YES	YES
51	a	178.65	1.32124	YES	YES
52	a	180.92	2.02199	YES	YES
53	a	182.06	2.72553	YES	YES
54	a	192.11	0.62156	YES	YES
55	a	199.57	0.20281	YES	YES
56	a	204.35	4.67018	YES	YES
57	a	209.24	0.29587	YES	YES

58	a	212.46	0.28784	YES	YES
59	a	214.63	1.03849	YES	YES
60	a	216.13	0.73653	YES	YES
61	a	217.27	0.88460	YES	YES
62	a	220.02	0.44937	YES	YES
63	a	222.31	0.91637	YES	YES
64	a	225.34	0.13382	YES	YES
65	a	226.51	0.10005	YES	YES
66	a	232.27	0.64292	YES	YES
67	a	235.00	0.17408	YES	YES
68	a	235.59	0.58273	YES	YES
69	a	238.81	0.17937	YES	YES
70	a	239.50	0.41971	YES	YES
71	a	241.60	2.98301	YES	YES
72	a	245.89	0.48364	YES	YES
73	a	254.07	1.14574	YES	YES
74	a	265.21	1.26297	YES	YES
75	a	277.06	1.31121	YES	YES
76	a	278.90	0.77856	YES	YES
77	a	287.27	0.49141	YES	YES
78	a	295.20	0.21816	YES	YES
79	a	301.90	0.80841	YES	YES
80	a	311.95	1.96025	YES	YES
81	a	313.26	0.48217	YES	YES
82	a	327.62	1.66673	YES	YES
83	a	329.91	1.06443	YES	YES
84	a	330.76	0.49890	YES	YES
85	a	334.29	0.70553	YES	YES
86	a	335.05	0.76359	YES	YES
87	a	337.88	0.71689	YES	YES
88	a	352.68	10.30649	YES	YES
89	a	364.71	9.44839	YES	YES
90	a	376.29	8.81088	YES	YES
91	a	379.51	3.18798	YES	YES
92	a	381.19	0.68686	YES	YES
93	a	388.55	1.36191	YES	YES
94	a	390.30	1.34853	YES	YES
95	a	407.96	0.28122	YES	YES
96	a	412.32	1.30471	YES	YES
97	a	413.19	2.30592	YES	YES
98	a	420.02	4.02498	YES	YES
99	a	422.84	3.62189	YES	YES
100	a	425.76	5.88927	YES	YES
101	a	429.19	2.34791	YES	YES
102	a	430.70	0.08840	YES	YES
103	a	431.04	0.29303	YES	YES
104	a	431.28	0.35083	YES	YES
105	a	433.03	0.57244	YES	YES
106	a	433.70	0.71435	YES	YES
107	a	433.88	1.03567	YES	YES
108	a	434.39	1.54326	YES	YES
109	a	438.27	0.23124	YES	YES
110	a	440.73	5.11308	YES	YES
111	a	456.60	5.91229	YES	YES
112	a	459.86	4.32480	YES	YES
113	a	469.93	3.05790	YES	YES
114	a	492.03	3.02986	YES	YES
115	a	492.68	4.73084	YES	YES

116	a	493.67	5.17796	YES	YES
117	a	496.36	0.84615	YES	YES
118	a	500.49	0.38292	YES	YES
119	a	501.07	1.67898	YES	YES
120	a	508.12	8.54312	YES	YES
121	a	511.48	9.96819	YES	YES
122	a	625.82	4.93595	YES	YES
123	a	626.61	26.94271	YES	YES
124	a	640.55	9.64961	YES	YES
125	a	662.76	11.74261	YES	YES
126	a	700.63	1.88155	YES	YES
127	a	711.25	8.24136	YES	YES
128	a	712.31	1.29671	YES	YES
129	a	726.40	4.71417	YES	YES
130	a	727.31	6.27768	YES	YES
131	a	729.64	2.91697	YES	YES
132	a	730.80	12.10351	YES	YES
133	a	732.42	6.13835	YES	YES
134	a	771.65	13.07358	YES	YES
135	a	772.72	3.41216	YES	YES
136	a	772.98	2.20721	YES	YES
137	a	773.72	0.23463	YES	YES
138	a	774.15	3.45197	YES	YES
139	a	775.16	0.85802	YES	YES
140	a	776.19	0.08390	YES	YES
141	a	776.31	0.49448	YES	YES
142	a	778.06	0.81117	YES	YES
143	a	785.81	16.68392	YES	YES
144	a	805.06	0.49758	YES	YES
145	a	808.02	1.44619	YES	YES
146	a	809.22	6.16303	YES	YES
147	a	810.11	7.15391	YES	YES
148	a	811.33	4.12460	YES	YES
149	a	812.33	2.47731	YES	YES
150	a	815.01	2.83481	YES	YES
151	a	816.91	1.87520	YES	YES
152	a	831.59	2.75723	YES	YES
153	a	832.23	6.43774	YES	YES
154	a	834.64	9.92299	YES	YES
155	a	837.19	2.19637	YES	YES
156	a	838.02	1.24460	YES	YES
157	a	838.68	1.84871	YES	YES
158	a	839.57	5.50798	YES	YES
159	a	839.74	4.86563	YES	YES
160	a	847.19	18.27678	YES	YES
161	a	851.32	15.35399	YES	YES
162	a	873.98	2.19495	YES	YES
163	a	875.04	8.25337	YES	YES
164	a	875.64	6.08716	YES	YES
165	a	875.95	5.10526	YES	YES
166	a	877.01	3.87825	YES	YES
167	a	877.24	19.32819	YES	YES
168	a	877.31	7.19934	YES	YES
169	a	878.04	9.20632	YES	YES
170	a	878.15	13.56387	YES	YES
171	a	879.26	4.27762	YES	YES
172	a	880.31	11.45761	YES	YES
173	a	881.04	2.09509	YES	YES

174	a	882.58	5.06544	YES	YES
175	a	883.32	0.55738	YES	YES
176	a	884.96	1.60732	YES	YES
177	a	885.17	2.31494	YES	YES
178	a	886.55	2.20124	YES	YES
179	a	903.17	3.68465	YES	YES
180	a	903.90	1.52852	YES	YES
181	a	904.18	3.40607	YES	YES
182	a	904.87	1.71341	YES	YES
183	a	908.83	0.75901	YES	YES
184	a	909.85	0.90112	YES	YES
185	a	910.26	0.35133	YES	YES
186	a	910.85	1.49291	YES	YES
187	a	984.37	1.89994	YES	YES
188	a	985.16	1.31055	YES	YES
189	a	986.48	7.95531	YES	YES
190	a	986.96	4.53844	YES	YES
191	a	988.29	12.17938	YES	YES
192	a	989.28	11.15557	YES	YES
193	a	990.12	16.25831	YES	YES
194	a	990.35	4.37912	YES	YES
195	a	991.25	2.55959	YES	YES
196	a	992.15	10.74737	YES	YES
197	a	997.88	7.01106	YES	YES
198	a	1014.06	1.70186	YES	YES
199	a	1015.51	2.52809	YES	YES
200	a	1016.02	2.62125	YES	YES
201	a	1017.25	1.34720	YES	YES
202	a	1018.03	0.57207	YES	YES
203	a	1019.44	0.09700	YES	YES
204	a	1020.81	0.49201	YES	YES
205	a	1022.38	0.79703	YES	YES
206	a	1024.71	60.56621	YES	YES
207	a	1030.87	1.13890	YES	YES
208	a	1031.97	1.95738	YES	YES
209	a	1034.74	1.50687	YES	YES
210	a	1035.44	1.19449	YES	YES
211	a	1038.75	2.95485	YES	YES
212	a	1039.22	0.91116	YES	YES
213	a	1040.41	0.79923	YES	YES
214	a	1042.33	0.79179	YES	YES
215	a	1044.05	2.87738	YES	YES
216	a	1056.33	1.49764	YES	YES
217	a	1056.94	0.56389	YES	YES
218	a	1057.41	2.80223	YES	YES
219	a	1058.16	2.09926	YES	YES
220	a	1060.74	0.71153	YES	YES
221	a	1063.09	0.74365	YES	YES
222	a	1064.63	0.72264	YES	YES
223	a	1066.35	2.46423	YES	YES
224	a	1070.17	0.50926	YES	YES
225	a	1071.30	0.84680	YES	YES
226	a	1072.45	0.21025	YES	YES
227	a	1072.74	0.65574	YES	YES
228	a	1073.32	0.21228	YES	YES
229	a	1074.11	0.29225	YES	YES
230	a	1074.62	0.14842	YES	YES
231	a	1074.92	0.32489	YES	YES

232	a	1075.71	2.66847	YES	YES
233	a	1077.88	4.06963	YES	YES
234	a	1088.79	6.31465	YES	YES
235	a	1090.06	7.29650	YES	YES
236	a	1090.97	4.23563	YES	YES
237	a	1092.84	16.21477	YES	YES
238	a	1098.24	1.55927	YES	YES
239	a	1104.13	8.63242	YES	YES
240	a	1104.32	1.45872	YES	YES
241	a	1113.35	3.19311	YES	YES
242	a	1113.88	16.45601	YES	YES
243	a	1122.34	3.84202	YES	YES
244	a	1126.75	2.71597	YES	YES
245	a	1158.34	5.38425	YES	YES
246	a	1159.74	5.70828	YES	YES
247	a	1160.38	9.23616	YES	YES
248	a	1165.64	10.07507	YES	YES
249	a	1168.06	17.54369	YES	YES
250	a	1168.95	3.61084	YES	YES
251	a	1169.98	7.42783	YES	YES
252	a	1170.61	17.22483	YES	YES
253	a	1173.84	5.25802	YES	YES
254	a	1177.05	3.48714	YES	YES
255	a	1177.65	22.65599	YES	YES
256	a	1180.66	5.72557	YES	YES
257	a	1182.74	3.68899	YES	YES
258	a	1185.83	0.98686	YES	YES
259	a	1187.32	10.70242	YES	YES
260	a	1192.91	1.22029	YES	YES
261	a	1212.15	1.27946	YES	YES
262	a	1229.59	21.18449	YES	YES
263	a	1234.83	0.94673	YES	YES
264	a	1236.52	0.51250	YES	YES
265	a	1244.13	0.77950	YES	YES
266	a	1244.81	0.83932	YES	YES
267	a	1245.41	0.22075	YES	YES
268	a	1245.81	0.30507	YES	YES
269	a	1246.94	2.33780	YES	YES
270	a	1248.58	1.99911	YES	YES
271	a	1250.16	0.24943	YES	YES
272	a	1250.56	0.88321	YES	YES
273	a	1251.23	3.06631	YES	YES
274	a	1253.49	0.74068	YES	YES
275	a	1254.32	1.84788	YES	YES
276	a	1255.29	6.50585	YES	YES
277	a	1255.92	3.32850	YES	YES
278	a	1256.30	5.77044	YES	YES
279	a	1256.48	4.53832	YES	YES
280	a	1257.13	1.91125	YES	YES
281	a	1257.73	2.99286	YES	YES
282	a	1258.57	5.28107	YES	YES
283	a	1259.41	2.35554	YES	YES
284	a	1260.56	1.76352	YES	YES
285	a	1261.12	1.52242	YES	YES
286	a	1263.33	0.15119	YES	YES
287	a	1264.18	2.37773	YES	YES
288	a	1264.85	1.38214	YES	YES
289	a	1269.20	0.96127	YES	YES

290	a	1269.65	3.68636	YES	YES
291	a	1281.65	1.25791	YES	YES
292	a	1282.47	3.74195	YES	YES
293	a	1283.41	1.08804	YES	YES
294	a	1284.19	1.28466	YES	YES
295	a	1289.81	4.00846	YES	YES
296	a	1290.11	3.16269	YES	YES
297	a	1291.25	3.36887	YES	YES
298	a	1291.81	5.20020	YES	YES
299	a	1306.03	0.93143	YES	YES
300	a	1307.42	1.79491	YES	YES
301	a	1309.37	1.84119	YES	YES
302	a	1310.31	0.58733	YES	YES
303	a	1311.82	2.81075	YES	YES
304	a	1313.01	0.41382	YES	YES
305	a	1314.41	0.57997	YES	YES
306	a	1316.10	1.99462	YES	YES
307	a	1318.58	3.30716	YES	YES
308	a	1319.31	0.65832	YES	YES
309	a	1320.40	1.90857	YES	YES
310	a	1320.71	0.67037	YES	YES
311	a	1322.59	5.52138	YES	YES
312	a	1322.94	1.54340	YES	YES
313	a	1323.08	0.11142	YES	YES
314	a	1323.64	4.29399	YES	YES
315	a	1324.19	0.73413	YES	YES
316	a	1324.29	0.54791	YES	YES
317	a	1324.60	3.27337	YES	YES
318	a	1324.90	1.36287	YES	YES
319	a	1325.03	1.94604	YES	YES
320	a	1326.33	4.18216	YES	YES
321	a	1327.11	9.64892	YES	YES
322	a	1331.17	3.31135	YES	YES
323	a	1335.79	0.63966	YES	YES
324	a	1335.90	0.12768	YES	YES
325	a	1336.49	0.08523	YES	YES
326	a	1336.70	0.02764	YES	YES
327	a	1337.28	0.22898	YES	YES
328	a	1338.14	0.14392	YES	YES
329	a	1338.28	0.02753	YES	YES
330	a	1338.69	0.14232	YES	YES
331	a	1344.50	2.28371	YES	YES
332	a	1345.04	0.57862	YES	YES
333	a	1345.67	0.44707	YES	YES
334	a	1346.78	1.47948	YES	YES
335	a	1347.78	1.90265	YES	YES
336	a	1348.00	2.56772	YES	YES
337	a	1348.63	0.51272	YES	YES
338	a	1349.17	1.35692	YES	YES
339	a	1388.80	1.89619	YES	YES
340	a	1400.74	4.86144	YES	YES
341	a	1405.95	6.72142	YES	YES
342	a	1407.32	10.90810	YES	YES
343	a	1411.23	5.07494	YES	YES
344	a	1415.05	0.87921	YES	YES
345	a	1419.53	1.14484	YES	YES
346	a	1424.98	2.24071	YES	YES
347	a	1426.40	3.88453	YES	YES

348	a	1428.48	4.95254	YES	YES
349	a	1429.40	1.29435	YES	YES
350	a	1430.71	0.84065	YES	YES
351	a	1432.70	1.28356	YES	YES
352	a	1433.28	3.36612	YES	YES
353	a	1433.37	4.40318	YES	YES
354	a	1433.83	2.21236	YES	YES
355	a	1435.30	4.54689	YES	YES
356	a	1436.38	9.18380	YES	YES
357	a	1436.99	4.50379	YES	YES
358	a	1437.48	12.95243	YES	YES
359	a	1437.94	5.24829	YES	YES
360	a	1438.10	2.88209	YES	YES
361	a	1438.21	9.29383	YES	YES
362	a	1438.29	2.37335	YES	YES
363	a	1438.70	4.61021	YES	YES
364	a	1438.88	6.05800	YES	YES
365	a	1439.35	1.93252	YES	YES
366	a	1439.49	0.63228	YES	YES
367	a	1439.89	10.40047	YES	YES
368	a	1440.65	6.70235	YES	YES
369	a	1441.00	3.36198	YES	YES
370	a	1441.53	4.64334	YES	YES
371	a	1443.13	14.35918	YES	YES
372	a	1443.78	11.79025	YES	YES
373	a	1444.18	2.04242	YES	YES
374	a	1444.42	0.98330	YES	YES
375	a	1445.20	37.02306	YES	YES
376	a	1445.94	6.96795	YES	YES
377	a	1446.35	19.09513	YES	YES
378	a	1447.32	10.79802	YES	YES
379	a	1451.33	3.97218	YES	YES
380	a	1452.74	1.59359	YES	YES
381	a	1454.05	2.58515	YES	YES
382	a	1454.36	4.21771	YES	YES
383	a	1455.07	2.38987	YES	YES
384	a	1455.92	4.06532	YES	YES
385	a	1456.19	4.41234	YES	YES
386	a	1456.75	0.21494	YES	YES
387	a	1457.74	0.80322	YES	YES
388	a	1462.40	11.01122	YES	YES
389	a	1464.85	11.42838	YES	YES
390	a	2917.56	18.26983	YES	YES
391	a	2917.89	14.65584	YES	YES
392	a	2919.90	38.19273	YES	YES
393	a	2923.42	17.28672	YES	YES
394	a	2924.43	3.44008	YES	YES
395	a	2924.64	6.00178	YES	YES
396	a	2927.94	13.96246	YES	YES
397	a	2934.00	2.58056	YES	YES
398	a	2935.31	18.44945	YES	YES
399	a	2936.16	22.17460	YES	YES
400	a	2937.17	12.87229	YES	YES
401	a	2938.49	8.24233	YES	YES
402	a	2938.93	5.15041	YES	YES
403	a	2939.19	9.78630	YES	YES
404	a	2940.29	12.10209	YES	YES
405	a	2940.75	8.25358	YES	YES

406	a	2941.33	18.37509	YES	YES
407	a	2942.06	9.99401	YES	YES
408	a	2943.31	5.76017	YES	YES
409	a	2944.58	8.77909	YES	YES
410	a	2944.86	8.94364	YES	YES
411	a	2945.39	22.65149	YES	YES
412	a	2946.13	4.65660	YES	YES
413	a	2947.81	12.36248	YES	YES
414	a	2948.05	11.24865	YES	YES
415	a	2950.24	5.84271	YES	YES
416	a	2952.39	5.43206	YES	YES
417	a	2954.06	12.07743	YES	YES
418	a	2954.15	8.92731	YES	YES
419	a	2955.17	4.44291	YES	YES
420	a	2955.27	5.04315	YES	YES
421	a	2955.65	6.20788	YES	YES
422	a	2957.45	20.25062	YES	YES
423	a	2957.79	20.51680	YES	YES
424	a	2958.08	20.41925	YES	YES
425	a	2958.98	34.02993	YES	YES
426	a	2959.07	50.70016	YES	YES
427	a	2959.20	20.80946	YES	YES
428	a	2960.09	1.01507	YES	YES
429	a	2960.30	15.66191	YES	YES
430	a	2960.44	28.33548	YES	YES
431	a	2961.23	42.83187	YES	YES
432	a	2962.04	35.63080	YES	YES
433	a	2962.68	4.27541	YES	YES
434	a	2964.12	37.57442	YES	YES
435	a	2965.42	17.86185	YES	YES
436	a	2967.76	35.25633	YES	YES
437	a	2970.03	12.67851	YES	YES
438	a	2975.39	33.53201	YES	YES
439	a	2977.84	3.53874	YES	YES
440	a	2978.15	20.34100	YES	YES
441	a	2978.69	16.87074	YES	YES
442	a	2980.56	15.21925	YES	YES
443	a	2981.38	3.24605	YES	YES
444	a	2985.71	4.24805	YES	YES
445	a	2986.91	30.25477	YES	YES
446	a	2989.40	4.49368	YES	YES
447	a	2992.10	9.91150	YES	YES
448	a	2993.35	37.51400	YES	YES
449	a	2993.99	14.71248	YES	YES
450	a	2994.46	14.92008	YES	YES
451	a	2997.26	34.13463	YES	YES
452	a	2998.30	25.88181	YES	YES
453	a	2999.85	24.88774	YES	YES
454	a	3001.86	29.50637	YES	YES
455	a	3003.77	26.49894	YES	YES
456	a	3004.34	24.64181	YES	YES
457	a	3006.07	20.52408	YES	YES
458	a	3007.00	15.70649	YES	YES
459	a	3007.54	17.70820	YES	YES
460	a	3009.11	23.89652	YES	YES
461	a	3010.04	18.63669	YES	YES
462	a	3010.70	15.59706	YES	YES
463	a	3011.47	30.36626	YES	YES

464	a	3013.22	12.83235	YES	YES
465	a	3013.31	28.01802	YES	YES
466	a	3013.47	28.14380	YES	YES
467	a	3014.07	20.82273	YES	YES
468	a	3014.26	31.08357	YES	YES
469	a	3014.34	13.21454	YES	YES
470	a	3015.24	36.60132	YES	YES
471	a	3015.37	30.45388	YES	YES
472	a	3015.96	22.40527	YES	YES
473	a	3016.07	27.06624	YES	YES
474	a	3016.37	8.73783	YES	YES
475	a	3016.40	35.12253	YES	YES
476	a	3016.76	35.80073	YES	YES
477	a	3016.81	38.90446	YES	YES
478	a	3017.06	17.10766	YES	YES
479	a	3018.40	17.12772	YES	YES
480	a	3018.63	25.32452	YES	YES
481	a	3019.59	31.54686	YES	YES
482	a	3022.93	6.89743	YES	YES
483	a	3026.03	28.18112	YES	YES
484	a	3026.87	14.20454	YES	YES
485	a	3030.02	1.55661	YES	YES
486	a	3034.95	2.57477	YES	YES
487	a	3045.10	4.67908	YES	YES
488	a	3046.30	1.08948	YES	YES
489	a	3051.29	2.63124	YES	YES
490	a	3081.73	4.24589	YES	YES
491	a	3089.95	1.89414	YES	YES
492	a	3190.84	102.14833	YES	YES

$end

Double hybrid single point energy = -7384.056600270266 H
COSMO energy + OC correction = -7391.5761338392 H (in *o*DFB)

6.2.27 [{Ga(dcpe)}$_2$(H–NMe$_2$)]$^{2+}$ (Transition State)

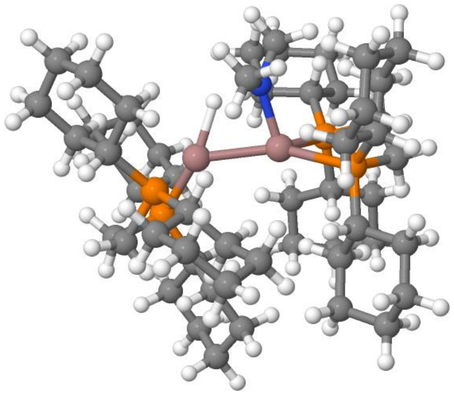

Method: (RI-)BP86(D3BJ)/def2-TZVPP
Symmetry: c1

Cartesian coordinates in Ångström:

Ga	-2.0256823	17.2434061	-2.4121215
Ga	-4.0487714	17.8432785	-1.0786100
P	-1.3692115	18.8005491	-4.1497622
P	-6.1216389	19.1393855	-0.8116180

P	-1.9671263	15.5780233	-4.1448727
P	-5.2276509	16.1703847	0.2581906
C	-4.7808495	21.1123884	-4.9087987
H	-4.8058860	21.6793054	-3.9626378
H	-5.8149146	20.8034382	-5.1208417
C	-0.9936881	14.1146283	-3.5833382
H	-0.8243243	13.4671184	-4.4593531
C	-4.2926112	15.1766279	1.4992391
H	-4.0573756	15.9332431	2.2693406
C	-4.3444771	16.0917411	-5.5190929
H	-3.7604669	16.5172476	-6.3507025
H	-4.5009657	16.8998763	-4.7884619
C	-7.0001637	19.3398575	-2.4222615
H	-6.3270823	19.9925852	-3.0025855
C	-7.2314661	18.1038641	0.2606272
H	-7.8846488	18.7739748	0.8357891
H	-7.8822042	17.5224563	-0.4064810
C	-2.7998493	22.4149026	-5.7773756
H	-2.4199591	23.0292080	-6.6046969
H	-2.7382896	23.0387394	-4.8703485
C	-1.9020671	21.1830003	-5.5984042
H	-0.8726083	21.5003257	-5.3850610
H	-1.8728130	20.6138479	-6.5418683
C	-3.8974841	19.8728478	-4.7249147
H	-3.9603331	19.2535078	-5.6343107
H	-4.2552720	19.2468924	-3.8921436
C	1.1259094	13.3574251	-2.4104615
H	1.3863040	12.6513929	-3.2155490
H	2.0751760	13.7088691	-1.9838299
C	-7.5097819	14.5039414	-0.3093191
H	-8.1491340	15.2806378	0.1334615
H	-7.2619054	13.8018813	0.4988453
C	-6.1064218	20.8046595	-0.0195797
H	-7.1674349	21.1035408	0.0418171
C	0.4094154	20.3704926	-2.6743499
H	-0.2844843	21.2152029	-2.7956367
H	0.0802980	19.8055630	-1.7874664
C	1.8338346	20.8888570	-2.4414710
H	2.1352018	21.5207552	-3.2927289
H	1.8477818	21.5347021	-1.5526603
C	-7.1137639	18.0031981	-3.1721128
H	-6.1190208	17.5374366	-3.2685961
H	-7.7404377	17.3077399	-2.5887852
C	-4.2571841	22.0184801	-6.0270916
H	-4.3325180	21.4866141	-6.9898377
H	-4.8858797	22.9145690	-6.1165679
C	-8.3674267	20.0334882	-2.3012917
H	-8.2669632	21.0069229	-1.8027429
H	-9.0364287	19.4205774	-1.6750856
C	-5.3624398	21.8230196	-0.8995147
H	-5.8548250	21.9060153	-1.8788393
H	-4.3392739	21.4621521	-1.0893422
C	-3.3387667	13.7807972	-5.8537394
H	-2.8140601	12.9503654	-5.3625260
H	-2.7002920	14.1105283	-6.6895887
C	2.7709427	18.7979079	-3.4958596
H	3.1189297	19.3376973	-4.3915790
H	3.4525053	17.9468737	-3.3600539

C	-5.4909682	22.1271680	2.0523505
H	-6.5177885	22.4995465	2.1979765
H	-5.0420652	22.0506758	3.0519946
C	-9.1157561	18.8972562	-4.4404432
H	-9.5377589	19.0630054	-5.4406547
H	-9.8218794	18.2366613	-3.9104334
C	-5.0814947	14.0494850	2.1887670
H	-6.0256547	14.4279202	2.6048943
H	-5.3411672	13.2787825	1.4472358
C	0.2914476	12.6360940	-1.3488306
H	0.8443548	11.7766617	-0.9471275
H	0.1118660	13.3203176	-0.5019349
C	-7.7573186	18.1987274	-4.5498630
H	-7.0805438	18.7993706	-5.1796111
H	-7.8657199	17.2251895	-5.0453499
C	2.8248634	19.7325266	-2.2847446
H	2.5802720	19.1634812	-1.3721988
H	3.8435992	20.1191917	-2.1491884
C	-4.7108912	23.1168010	1.1823444
H	-4.7068938	24.1098362	1.6507366
H	-3.6562186	22.7995866	1.1185208
C	-5.3410868	14.0450923	-1.5518079
H	-5.0058442	13.3202410	-0.7952837
H	-4.4342116	14.5133911	-1.9629405
C	-5.5461956	20.7349265	1.4103403
H	-4.5363463	20.2989044	1.3835807
H	-6.1636535	20.0657958	2.0275742
C	-6.2216908	15.1135994	-0.8859854
H	-6.5131271	15.8236256	-1.6804137
C	-5.3066042	23.2001986	-0.2257711
C	-7.4141121	12.6968942	-2.0868483
H	-7.9766232	12.2027664	-2.8901991
H	-7.1628009	11.9129541	-1.3540209
C	-8.2809967	13.7643849	-1.4118072
H	-9.1873990	13.3126723	-0.9870691
H	-8.6187122	14.4935128	-2.1678676
C	-1.8251618	13.3559040	-2.5335433
H	-2.7676700	12.9987642	-2.9725139
H	-2.0927334	14.0652605	-1.7315798
C	-4.2285799	13.4153026	3.2974086
H	-4.0483709	14.1641444	4.0859417
H	-4.7905259	12.5967311	3.7670570
C	-5.6796705	15.5883505	-6.0781332
H	-6.2074969	16.4174904	-6.5685929
H	-6.3189627	15.2573147	-5.2428183
C	-2.8882000	12.9066454	2.7568299
H	-3.0709507	12.0783264	2.0519544
H	-2.2842117	12.4911580	3.5742896
C	-2.9604003	14.6534962	0.9293603
H	-3.1711479	13.8962052	0.1605345
H	-2.4041742	15.4611011	0.4280723
C	-9.0004795	20.2241418	-3.6864520
H	-9.9872168	20.6937032	-3.5773458
H	-8.3821467	20.9271324	-4.2686143
C	-4.6821448	13.2902059	-6.4125076
H	-4.5030342	12.4853536	-7.1380137
H	-5.2718317	12.8483538	-5.5934705
C	-6.1225041	13.3051137	-2.6412941

```
H    -5.4894042   12.5256229   -3.0897345
H    -6.3721607   14.0132240   -3.4488983
C    -5.4761016   14.4288997   -7.0574555
H    -6.4461125   14.0602371   -7.4170746
H    -4.9344082   14.7954132   -7.9450530
C    -1.0505957   12.1811496   -1.9267266
H    -1.6653640   11.7002155   -1.1523592
H    -0.8773804   11.4199602   -2.7043385
C    -2.1158491   14.0185359    2.0390001
H    -1.8271528   14.7966523    2.7649871
H    -1.1814661   13.6273396    1.6126770
H    -1.7003524   17.7075359   -0.7624499
C     0.3479935   19.4363417   -3.8919296
H     0.6014371   20.0070193   -4.8016934
C     1.3494506   18.2771838   -3.7446139
H     1.3441699   17.6481793   -4.6459227
H     1.0342799   17.6401884   -2.9006294
C     0.3642750   14.5490630   -3.0033317
H     0.9767711   15.0378468   -3.7738328
H     0.1874547   15.3000845   -2.2134012
C    -1.0224221   16.3451817   -5.5381520
H     0.0379747   16.1799452   -5.3120981
H    -1.2395938   15.7911440   -6.4619611
C    -1.2999425   17.8452126   -5.7348218
H    -2.2698476   17.9923765   -6.2260677
H    -0.5367598   18.2873747   -6.3907531
C    -3.5464789   14.9473536   -4.8731737
H    -4.1206510   14.5821114   -4.0074777
C    -2.4357657   20.2773117   -4.4754924
H    -2.4013758   20.8400441   -3.5254498
C    -6.4568785   17.1680809    1.1999165
H    -7.1480883   16.5154633    1.7507356
H    -5.8858130   17.7428644    1.9428152
H    -6.3265456   23.6132459   -0.1664450
H    -4.7270033   23.8911302   -0.8532578
C    -2.1954621   19.9322735   -0.1537631
N    -2.6369330   18.5803541    0.1693040
H    -2.2599876   20.0974036   -1.2403809
H    -1.1466070   20.0820694    0.1502028
H    -2.7955593   20.7125070    0.3409814
C    -2.4736031   18.2853730    1.5868589
H    -3.1910906   18.8270451    2.2295611
H    -1.4613341   18.5680346    1.9203884
H    -2.5907663   17.2123283    1.7682893

SCF energy GEOOPT =  -7391.395640946 H
ZPE =   3824. kJ/mol
FREEH energy =  4011.90 kJ/mol
FREEH entropy =   1.58614 kJ/mol/K

$vibrational spectrum
#  mode     symmetry     wave number   IR intensity    selection rules
#                         cm**(-1)        km/mol          IR     RAMAN
     1        a          -1003.77         0.00000        YES      YES
     2                      -0.00         0.00000         -        -
     3                      -0.00         0.00000         -        -
     4                       0.00         0.00000         -        -
     5                       0.00         0.00000         -        -
```

6		0.00	0.00000	-	-
7		0.00	0.00000	-	-
8	a	18.08	0.01380	YES	YES
9	a	24.73	0.00885	YES	YES
10	a	29.19	0.01476	YES	YES
11	a	31.43	0.07469	YES	YES
12	a	32.19	0.24835	YES	YES
13	a	34.48	0.00676	YES	YES
14	a	36.36	0.00426	YES	YES
15	a	36.61	0.05822	YES	YES
16	a	40.18	0.01818	YES	YES
17	a	42.03	0.06750	YES	YES
18	a	42.32	0.28864	YES	YES
19	a	48.60	0.14675	YES	YES
20	a	50.37	0.05779	YES	YES
21	a	53.44	0.03306	YES	YES
22	a	58.49	0.05571	YES	YES
23	a	58.93	0.32634	YES	YES
24	a	63.13	0.07042	YES	YES
25	a	65.61	0.23378	YES	YES
26	a	68.50	0.15551	YES	YES
27	a	70.72	0.24636	YES	YES
28	a	71.37	0.07209	YES	YES
29	a	74.08	0.00585	YES	YES
30	a	76.27	0.21764	YES	YES
31	a	77.63	0.08370	YES	YES
32	a	80.98	0.10425	YES	YES
33	a	84.96	0.38563	YES	YES
34	a	90.71	0.15634	YES	YES
35	a	93.78	0.21917	YES	YES
36	a	102.66	0.29652	YES	YES
37	a	107.87	0.72815	YES	YES
38	a	111.94	3.52961	YES	YES
39	a	119.45	0.47506	YES	YES
40	a	123.22	1.55184	YES	YES
41	a	129.15	0.19398	YES	YES
42	a	133.56	0.30463	YES	YES
43	a	136.98	2.12737	YES	YES
44	a	138.97	0.62098	YES	YES
45	a	141.64	1.04786	YES	YES
46	a	143.12	0.50798	YES	YES
47	a	148.81	2.33713	YES	YES
48	a	153.68	5.89137	YES	YES
49	a	162.24	1.21536	YES	YES
50	a	175.87	0.27516	YES	YES
51	a	178.71	0.78575	YES	YES
52	a	181.18	0.47646	YES	YES
53	a	185.83	0.43244	YES	YES
54	a	193.71	1.76101	YES	YES
55	a	198.67	1.09385	YES	YES
56	a	203.66	0.04192	YES	YES
57	a	215.15	0.35215	YES	YES
58	a	216.77	0.32098	YES	YES
59	a	217.72	0.37013	YES	YES
60	a	220.11	0.04433	YES	YES
61	a	224.03	0.42493	YES	YES
62	a	225.51	0.17910	YES	YES
63	a	228.46	0.60634	YES	YES

64	a	233.09	2.66520	YES	YES
65	a	236.37	0.63223	YES	YES
66	a	237.80	0.47436	YES	YES
67	a	238.50	0.46703	YES	YES
68	a	239.53	0.12121	YES	YES
69	a	241.94	1.32669	YES	YES
70	a	243.42	0.08947	YES	YES
71	a	251.20	0.24789	YES	YES
72	a	257.91	2.90135	YES	YES
73	a	259.13	1.99153	YES	YES
74	a	273.08	1.66472	YES	YES
75	a	281.18	1.94509	YES	YES
76	a	282.58	8.08458	YES	YES
77	a	284.93	0.27149	YES	YES
78	a	293.64	6.22395	YES	YES
79	a	298.59	1.17030	YES	YES
80	a	305.85	0.03665	YES	YES
81	a	313.29	0.11250	YES	YES
82	a	314.43	0.31899	YES	YES
83	a	329.87	0.28146	YES	YES
84	a	330.73	4.61159	YES	YES
85	a	332.60	6.11213	YES	YES
86	a	334.15	5.66281	YES	YES
87	a	336.40	7.55875	YES	YES
88	a	338.88	2.11558	YES	YES
89	a	339.98	0.51978	YES	YES
90	a	371.55	5.26539	YES	YES
91	a	379.89	2.59414	YES	YES
92	a	383.85	1.36796	YES	YES
93	a	385.82	0.55850	YES	YES
94	a	393.00	0.70122	YES	YES
95	a	397.02	0.75462	YES	YES
96	a	409.94	0.12325	YES	YES
97	a	413.93	0.46312	YES	YES
98	a	421.41	2.07416	YES	YES
99	a	422.24	3.13473	YES	YES
100	a	425.01	5.05845	YES	YES
101	a	429.23	0.83315	YES	YES
102	a	431.00	0.08499	YES	YES
103	a	431.21	0.46519	YES	YES
104	a	431.42	0.19798	YES	YES
105	a	431.55	0.03144	YES	YES
106	a	433.75	0.79190	YES	YES
107	a	434.15	0.46618	YES	YES
108	a	435.65	1.87384	YES	YES
109	a	438.98	0.51904	YES	YES
110	a	444.30	4.82853	YES	YES
111	a	459.93	6.65485	YES	YES
112	a	461.10	5.49638	YES	YES
113	a	472.57	2.98345	YES	YES
114	a	482.80	15.80817	YES	YES
115	a	493.07	6.71100	YES	YES
116	a	493.79	3.22385	YES	YES
117	a	495.63	1.88734	YES	YES
118	a	498.26	1.56642	YES	YES
119	a	502.74	2.09405	YES	YES
120	a	503.81	0.65265	YES	YES
121	a	512.10	7.45972	YES	YES

122	a	514.45	14.49791	YES	YES
123	a	631.30	5.96323	YES	YES
124	a	632.49	28.16366	YES	YES
125	a	643.07	10.04812	YES	YES
126	a	665.62	12.97772	YES	YES
127	a	703.13	1.77380	YES	YES
128	a	715.75	1.49607	YES	YES
129	a	719.97	4.07854	YES	YES
130	a	729.19	11.42723	YES	YES
131	a	731.37	2.16906	YES	YES
132	a	733.58	0.48378	YES	YES
133	a	734.05	5.06120	YES	YES
134	a	736.37	8.20017	YES	YES
135	a	771.42	0.78300	YES	YES
136	a	772.94	7.12861	YES	YES
137	a	773.33	1.59127	YES	YES
138	a	774.23	2.25697	YES	YES
139	a	775.46	11.48230	YES	YES
140	a	775.93	0.09780	YES	YES
141	a	777.25	0.96642	YES	YES
142	a	777.38	0.69449	YES	YES
143	a	777.98	0.38089	YES	YES
144	a	788.32	15.96222	YES	YES
145	a	805.83	0.76636	YES	YES
146	a	809.45	2.08691	YES	YES
147	a	810.25	4.85076	YES	YES
148	a	811.94	4.42130	YES	YES
149	a	812.62	3.35638	YES	YES
150	a	812.93	6.32084	YES	YES
151	a	814.55	2.52148	YES	YES
152	a	817.52	2.67459	YES	YES
153	a	832.44	2.78042	YES	YES
154	a	832.97	6.16466	YES	YES
155	a	835.24	9.95082	YES	YES
156	a	837.64	1.46378	YES	YES
157	a	838.05	1.41296	YES	YES
158	a	838.51	2.42932	YES	YES
159	a	839.28	3.78302	YES	YES
160	a	840.56	6.77839	YES	YES
161	a	848.49	23.75262	YES	YES
162	a	855.00	16.16191	YES	YES
163	a	873.39	1.67039	YES	YES
164	a	873.88	3.06088	YES	YES
165	a	875.92	0.11415	YES	YES
166	a	876.74	1.81483	YES	YES
167	a	877.12	3.68294	YES	YES
168	a	877.51	2.88517	YES	YES
169	a	878.31	2.10365	YES	YES
170	a	879.49	4.06327	YES	YES
171	a	879.67	9.02049	YES	YES
172	a	880.46	4.72819	YES	YES
173	a	881.29	0.31125	YES	YES
174	a	882.40	4.77061	YES	YES
175	a	883.20	2.05049	YES	YES
176	a	885.29	2.54379	YES	YES
177	a	885.54	2.30820	YES	YES
178	a	886.16	4.61398	YES	YES
179	a	903.25	5.76602	YES	YES

180	a	903.67	3.03476	YES	YES
181	a	904.76	6.61273	YES	YES
182	a	906.49	1.28441	YES	YES
183	a	908.81	2.70457	YES	YES
184	a	910.39	1.52038	YES	YES
185	a	910.47	0.18351	YES	YES
186	a	911.27	1.87706	YES	YES
187	a	914.44	58.08535	YES	YES
188	a	918.89	35.62032	YES	YES
189	a	983.30	0.97052	YES	YES
190	a	984.12	0.12522	YES	YES
191	a	987.37	13.27886	YES	YES
192	a	987.67	1.86460	YES	YES
193	a	988.59	3.93152	YES	YES
194	a	989.38	23.08946	YES	YES
195	a	989.78	10.53240	YES	YES
196	a	990.80	4.64098	YES	YES
197	a	991.22	0.84322	YES	YES
198	a	991.99	16.66784	YES	YES
199	a	1013.67	0.61032	YES	YES
200	a	1015.84	1.37903	YES	YES
201	a	1016.23	0.80252	YES	YES
202	a	1017.97	0.46721	YES	YES
203	a	1018.63	0.22030	YES	YES
204	a	1019.45	0.56583	YES	YES
205	a	1020.32	0.47985	YES	YES
206	a	1022.46	0.45504	YES	YES
207	a	1030.35	1.19237	YES	YES
208	a	1032.06	1.32470	YES	YES
209	a	1032.96	7.81077	YES	YES
210	a	1034.82	1.19745	YES	YES
211	a	1035.36	1.14039	YES	YES
212	a	1038.25	2.82401	YES	YES
213	a	1039.29	1.24885	YES	YES
214	a	1039.83	0.83773	YES	YES
215	a	1041.81	1.29249	YES	YES
216	a	1057.73	1.58264	YES	YES
217	a	1058.71	2.58475	YES	YES
218	a	1058.89	1.04771	YES	YES
219	a	1059.72	1.38476	YES	YES
220	a	1061.85	0.83375	YES	YES
221	a	1064.77	0.91998	YES	YES
222	a	1066.14	0.59314	YES	YES
223	a	1067.80	3.42299	YES	YES
224	a	1068.48	1.32107	YES	YES
225	a	1070.27	0.47428	YES	YES
226	a	1071.86	0.85110	YES	YES
227	a	1072.59	0.11654	YES	YES
228	a	1073.08	0.23101	YES	YES
229	a	1073.49	0.13640	YES	YES
230	a	1073.94	0.09653	YES	YES
231	a	1074.76	0.10838	YES	YES
232	a	1075.98	0.42950	YES	YES
233	a	1077.69	3.50314	YES	YES
234	a	1080.33	3.63244	YES	YES
235	a	1091.39	4.38976	YES	YES
236	a	1092.74	4.46518	YES	YES
237	a	1093.85	4.63942	YES	YES

238	a	1095.16	6.93460	YES	YES
239	a	1099.92	2.56680	YES	YES
240	a	1103.98	2.82103	YES	YES
241	a	1105.94	6.97734	YES	YES
242	a	1114.05	9.09445	YES	YES
243	a	1114.66	6.52098	YES	YES
244	a	1123.14	3.57098	YES	YES
245	a	1129.70	2.34323	YES	YES
246	a	1159.22	5.52303	YES	YES
247	a	1160.46	11.13027	YES	YES
248	a	1160.99	3.76283	YES	YES
249	a	1161.86	11.67637	YES	YES
250	a	1165.94	11.87524	YES	YES
251	a	1168.98	10.70648	YES	YES
252	a	1169.87	11.54485	YES	YES
253	a	1170.55	4.35107	YES	YES
254	a	1171.73	9.96430	YES	YES
255	a	1176.07	9.32801	YES	YES
256	a	1179.04	14.44674	YES	YES
257	a	1179.56	8.89354	YES	YES
258	a	1183.03	5.24822	YES	YES
259	a	1185.88	0.69846	YES	YES
260	a	1188.01	1.20842	YES	YES
261	a	1192.08	0.96375	YES	YES
262	a	1210.70	4.17849	YES	YES
263	a	1213.37	1.79143	YES	YES
264	a	1238.43	1.24489	YES	YES
265	a	1239.54	0.88564	YES	YES
266	a	1245.30	1.01375	YES	YES
267	a	1245.49	0.12308	YES	YES
268	a	1245.80	0.61556	YES	YES
269	a	1246.48	1.73578	YES	YES
270	a	1246.75	0.74791	YES	YES
271	a	1248.77	1.31637	YES	YES
272	a	1249.93	0.35334	YES	YES
273	a	1250.92	0.20507	YES	YES
274	a	1253.16	1.15010	YES	YES
275	a	1254.00	0.95130	YES	YES
276	a	1255.14	1.60775	YES	YES
277	a	1256.04	3.69798	YES	YES
278	a	1256.93	1.97987	YES	YES
279	a	1257.49	2.38472	YES	YES
280	a	1258.03	3.89877	YES	YES
281	a	1258.36	8.26500	YES	YES
282	a	1258.56	9.87092	YES	YES
283	a	1259.90	2.32366	YES	YES
284	a	1261.18	0.16338	YES	YES
285	a	1261.84	3.60313	YES	YES
286	a	1262.14	0.22275	YES	YES
287	a	1263.29	1.62956	YES	YES
288	a	1265.38	1.62203	YES	YES
289	a	1265.96	0.99272	YES	YES
290	a	1269.97	1.36117	YES	YES
291	a	1270.90	1.88674	YES	YES
292	a	1282.19	0.52658	YES	YES
293	a	1282.70	4.76764	YES	YES
294	a	1283.64	5.58651	YES	YES
295	a	1284.71	2.11288	YES	YES

296	a	1288.88	16.05398	YES	YES
297	a	1289.02	0.57270	YES	YES
298	a	1291.38	10.72662	YES	YES
299	a	1291.91	4.17384	YES	YES
300	a	1301.02	210.64880	YES	YES
301	a	1305.44	12.21933	YES	YES
302	a	1309.97	0.82208	YES	YES
303	a	1310.44	0.55372	YES	YES
304	a	1310.73	1.91990	YES	YES
305	a	1313.32	0.28085	YES	YES
306	a	1314.72	1.78616	YES	YES
307	a	1314.88	1.00357	YES	YES
308	a	1317.37	2.02882	YES	YES
309	a	1319.11	2.15151	YES	YES
310	a	1321.09	1.98876	YES	YES
311	a	1321.32	0.39284	YES	YES
312	a	1322.83	1.03122	YES	YES
313	a	1323.06	0.10332	YES	YES
314	a	1323.72	1.38428	YES	YES
315	a	1324.21	1.99493	YES	YES
316	a	1324.37	0.77822	YES	YES
317	a	1324.56	0.35449	YES	YES
318	a	1324.78	0.34317	YES	YES
319	a	1325.41	4.51989	YES	YES
320	a	1325.91	1.99207	YES	YES
321	a	1326.58	0.60761	YES	YES
322	a	1327.36	6.44362	YES	YES
323	a	1329.48	6.51371	YES	YES
324	a	1331.22	2.76064	YES	YES
325	a	1336.27	0.64137	YES	YES
326	a	1336.47	0.23112	YES	YES
327	a	1336.89	0.22988	YES	YES
328	a	1337.09	0.12258	YES	YES
329	a	1337.69	0.38459	YES	YES
330	a	1338.15	0.06275	YES	YES
331	a	1338.46	0.16458	YES	YES
332	a	1338.78	0.36243	YES	YES
333	a	1344.53	1.97063	YES	YES
334	a	1345.16	0.41617	YES	YES
335	a	1345.84	0.40024	YES	YES
336	a	1346.58	1.66653	YES	YES
337	a	1347.46	1.91793	YES	YES
338	a	1348.24	1.63845	YES	YES
339	a	1349.24	0.65981	YES	YES
340	a	1349.89	0.85577	YES	YES
341	a	1386.06	0.02916	YES	YES
342	a	1401.75	4.63931	YES	YES
343	a	1406.64	8.50010	YES	YES
344	a	1407.40	9.42878	YES	YES
345	a	1410.68	6.91361	YES	YES
346	a	1413.75	3.76659	YES	YES
347	a	1416.29	13.28476	YES	YES
348	a	1426.49	2.26076	YES	YES
349	a	1428.35	6.12862	YES	YES
350	a	1430.59	3.98429	YES	YES
351	a	1431.12	5.36906	YES	YES
352	a	1432.53	2.74139	YES	YES
353	a	1432.95	3.39631	YES	YES

354	a	1434.12	6.31737	YES	YES
355	a	1434.94	3.34989	YES	YES
356	a	1435.76	6.31311	YES	YES
357	a	1436.33	0.45765	YES	YES
358	a	1436.61	7.34502	YES	YES
359	a	1437.13	4.96437	YES	YES
360	a	1437.77	0.83903	YES	YES
361	a	1437.92	9.87422	YES	YES
362	a	1438.23	2.85473	YES	YES
363	a	1438.44	6.39942	YES	YES
364	a	1438.76	6.92368	YES	YES
365	a	1439.41	5.50323	YES	YES
366	a	1439.61	3.15012	YES	YES
367	a	1439.73	4.58599	YES	YES
368	a	1440.13	0.61229	YES	YES
369	a	1440.35	9.39123	YES	YES
370	a	1440.55	3.31373	YES	YES
371	a	1441.06	2.79690	YES	YES
372	a	1441.61	11.24558	YES	YES
373	a	1442.16	9.87265	YES	YES
374	a	1443.34	5.18750	YES	YES
375	a	1444.01	6.62368	YES	YES
376	a	1444.74	23.29059	YES	YES
377	a	1445.03	7.46765	YES	YES
378	a	1445.80	16.76780	YES	YES
379	a	1446.31	18.36208	YES	YES
380	a	1446.51	1.92845	YES	YES
381	a	1449.85	13.17156	YES	YES
382	a	1452.99	1.39572	YES	YES
383	a	1454.39	2.44594	YES	YES
384	a	1455.18	0.95561	YES	YES
385	a	1455.77	1.49929	YES	YES
386	a	1455.78	2.25218	YES	YES
387	a	1456.63	1.95877	YES	YES
388	a	1456.91	1.00335	YES	YES
389	a	1459.67	6.11863	YES	YES
390	a	1468.18	5.92344	YES	YES
391	a	2905.04	69.06273	YES	YES
392	a	2922.64	55.77028	YES	YES
393	a	2925.72	10.07298	YES	YES
394	a	2927.78	31.96957	YES	YES
395	a	2928.70	2.22662	YES	YES
396	a	2929.88	2.02991	YES	YES
397	a	2934.33	1.28530	YES	YES
398	a	2934.75	7.32161	YES	YES
399	a	2937.81	12.40812	YES	YES
400	a	2938.02	2.49207	YES	YES
401	a	2938.32	18.58836	YES	YES
402	a	2939.06	12.02479	YES	YES
403	a	2940.74	13.96181	YES	YES
404	a	2940.77	17.67807	YES	YES
405	a	2941.89	5.81109	YES	YES
406	a	2942.00	6.81174	YES	YES
407	a	2942.96	6.50154	YES	YES
408	a	2943.83	8.89335	YES	YES
409	a	2943.91	14.42347	YES	YES
410	a	2944.02	9.96110	YES	YES
411	a	2944.68	10.76860	YES	YES

412	a	2946.54	6.86168	YES	YES
413	a	2946.84	13.92211	YES	YES
414	a	2947.75	12.02198	YES	YES
415	a	2949.41	15.35664	YES	YES
416	a	2950.41	10.77238	YES	YES
417	a	2951.53	2.72364	YES	YES
418	a	2953.59	7.72335	YES	YES
419	a	2954.07	12.87825	YES	YES
420	a	2954.95	5.74495	YES	YES
421	a	2955.33	19.07724	YES	YES
422	a	2955.90	12.02628	YES	YES
423	a	2956.29	4.45841	YES	YES
424	a	2956.79	5.70868	YES	YES
425	a	2958.41	12.91778	YES	YES
426	a	2959.37	6.78213	YES	YES
427	a	2959.66	43.06035	YES	YES
428	a	2959.91	6.61447	YES	YES
429	a	2959.97	55.33774	YES	YES
430	a	2960.46	6.73630	YES	YES
431	a	2960.67	23.78993	YES	YES
432	a	2960.72	58.81309	YES	YES
433	a	2960.85	26.18850	YES	YES
434	a	2962.12	26.19695	YES	YES
435	a	2962.33	18.31335	YES	YES
436	a	2964.47	20.95190	YES	YES
437	a	2964.60	7.68335	YES	YES
438	a	2964.84	22.89019	YES	YES
439	a	2966.86	23.42641	YES	YES
440	a	2976.75	39.97499	YES	YES
441	a	2977.37	15.64900	YES	YES
442	a	2978.40	8.33819	YES	YES
443	a	2979.00	4.06906	YES	YES
444	a	2980.27	6.31470	YES	YES
445	a	2981.22	20.77055	YES	YES
446	a	2983.90	12.56904	YES	YES
447	a	2986.37	4.55571	YES	YES
448	a	2990.96	3.44061	YES	YES
449	a	2995.71	8.09418	YES	YES
450	a	2996.20	39.25208	YES	YES
451	a	2996.80	19.21053	YES	YES
452	a	2997.64	12.00953	YES	YES
453	a	2999.22	52.94647	YES	YES
454	a	3000.06	46.75004	YES	YES
455	a	3000.41	1.47046	YES	YES
456	a	3001.43	6.01901	YES	YES
457	a	3001.74	16.68617	YES	YES
458	a	3003.36	30.66515	YES	YES
459	a	3004.29	25.35040	YES	YES
460	a	3005.90	25.44982	YES	YES
461	a	3006.73	6.84873	YES	YES
462	a	3008.89	5.30635	YES	YES
463	a	3009.52	11.23768	YES	YES
464	a	3009.93	21.13973	YES	YES
465	a	3011.01	25.56474	YES	YES
466	a	3011.22	27.14267	YES	YES
467	a	3012.41	34.08726	YES	YES
468	a	3012.44	16.48597	YES	YES
469	a	3012.76	14.25000	YES	YES

470	a	3013.47	16.33417	YES	YES
471	a	3013.71	20.45281	YES	YES
472	a	3014.38	18.42817	YES	YES
473	a	3015.02	39.20151	YES	YES
474	a	3015.10	14.40145	YES	YES
475	a	3016.19	36.22953	YES	YES
476	a	3016.31	18.53647	YES	YES
477	a	3016.33	16.67242	YES	YES
478	a	3017.06	19.66181	YES	YES
479	a	3017.22	33.47753	YES	YES
480	a	3017.27	23.02353	YES	YES
481	a	3017.32	18.55538	YES	YES
482	a	3017.95	32.92742	YES	YES
483	a	3018.33	34.67332	YES	YES
484	a	3018.81	23.07596	YES	YES
485	a	3019.02	32.88364	YES	YES
486	a	3019.79	25.08372	YES	YES
487	a	3022.68	31.52937	YES	YES
488	a	3026.74	4.13485	YES	YES
489	a	3032.25	1.16804	YES	YES
490	a	3036.27	2.13494	YES	YES
491	a	3047.92	0.64829	YES	YES
492	a	3065.35	10.47450	YES	YES

$end

Double hybrid single point energy = -7384.020186848819 H
COSMO energy + OC correction = -7391.5418957544 H (in *o*DFB)

6.2.28 [H{Ga(dcpe)}$_2$(NMe$_2$)]$^{2+}$ (*syn*-periplanar)

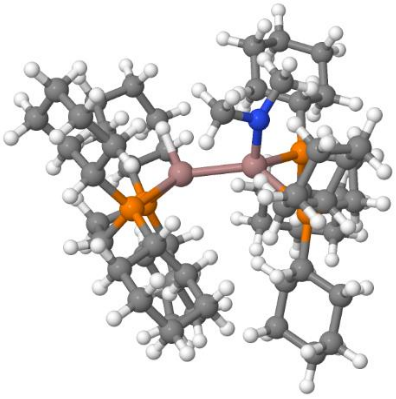

Method: (RI-)BP86(D3BJ)/def2-TZVPP
Symmetry: c1

Cartesian coordinates in Ångström:

Ga	-2.2612947	17.3197851	-2.4499839
Ga	-3.9591223	17.8820169	-0.7778190
P	-1.4583895	18.8840162	-4.1033400
P	-6.1088426	19.0941386	-0.8646001
P	-2.0892834	15.6264007	-4.1575050
P	-5.2472343	16.1862959	0.3610997
C	-4.8851483	21.1371128	-4.9473292
H	-4.9581765	21.7298272	-4.0203110
H	-5.9033831	20.7986329	-5.1869290
C	-1.0904247	14.1753542	-3.5987928

H	-0.9854736	13.5141804	-4.4751947
C	-4.3191423	15.2165810	1.6220649
H	-4.1606271	15.9558562	2.4260288
C	-4.4100453	16.0889600	-5.6347781
H	-3.7971851	16.4969164	-6.4544198
H	-4.6037425	16.9169070	-4.9351496
C	-6.9891149	19.2858330	-2.4742939
H	-6.3465548	19.9781725	-3.0421815
C	-7.2573838	18.1108541	0.2155158
H	-7.9288442	18.7995688	0.7449856
H	-7.8864668	17.5075186	-0.4530738
C	-2.9067594	22.4682724	-5.7660029
H	-2.5102233	23.0821888	-6.5855953
H	-2.8979473	23.1040234	-4.8654445
C	-1.9862929	21.2622375	-5.5393088
H	-0.9734051	21.6073036	-5.2926449
H	-1.9092663	20.6861748	-6.4759510
C	-3.9831607	19.9214859	-4.6999026
H	-4.0172822	19.2750670	-5.5914500
H	-4.3553416	19.3159254	-3.8583236
C	1.0722295	13.3480237	-2.5764146
H	1.2527635	12.6391228	-3.4007484
H	2.0598507	13.6646852	-2.2147059
C	-7.4681711	14.4767029	-0.2674780
H	-8.1304848	15.2269634	0.1866122
H	-7.2039539	13.7669207	0.5283612
C	-5.9810761	20.7835334	-0.1378693
H	-6.9593546	21.2628706	-0.3140341
C	0.3380155	20.4241092	-2.6162752
H	-0.3739591	21.2578297	-2.7043704
H	0.0499513	19.8425854	-1.7275308
C	1.7610765	20.9624766	-2.4223493
H	2.0263402	21.6086845	-3.2749489
H	1.7922611	21.5972910	-1.5264050
C	-7.0487770	17.9553402	-3.2410107
H	-6.0387533	17.5246976	-3.3372252
H	-7.6509624	17.2309809	-2.6668116
C	-4.3409129	22.0278476	-6.0672609
H	-4.3627320	21.4723049	-7.0193984
H	-4.9893568	22.9036490	-6.2025556
C	-8.3861080	19.9185206	-2.3487671
H	-8.3311731	20.8840909	-1.8288095
H	-9.0328701	19.2643669	-1.7417582
C	-4.8953892	21.5728122	-0.8913515
H	-5.1286597	21.6150837	-1.9654533
H	-3.9401279	21.0362775	-0.7832026
C	-3.3813557	13.7760774	-5.8705501
H	-2.8793346	12.9592229	-5.3353883
H	-2.7064089	14.0851217	-6.6857196
C	2.6970843	18.8974910	-3.5306835
H	3.0118868	19.4531134	-4.4289798
H	3.3942609	18.0550220	-3.4246455
C	-5.5739493	22.1882050	1.9203266
H	-6.5344760	22.7183284	1.8126384
H	-5.3579902	22.1480495	2.9966259
C	-9.0794894	18.7888654	-4.5071999
H	-9.5017345	18.9517935	-5.5077819
H	-9.7638530	18.0959804	-3.9900239

C	-5.0558552	14.0094750	2.2259837
H	-6.0304534	14.3116266	2.6345531
H	-5.2525816	13.2672613	1.4371253
C	0.2897100	12.6466003	-1.4629273
H	0.8415746	11.7678703	-1.1039018
H	0.1888563	13.3296524	-0.6024464
C	-7.6963927	18.1404967	-4.6184614
H	-7.0405287	18.7707081	-5.2414339
H	-7.7703105	17.1691868	-5.1237873
C	2.7722857	19.8183456	-2.3099244
H	2.5630345	19.2355318	-1.3974619
H	3.7890006	20.2180579	-2.1997387
C	-4.4792915	22.9625143	1.1800121
H	-4.4060560	23.9857348	1.5716797
H	-3.5037673	22.4827390	1.3664596
C	-5.2809634	14.1160970	-1.5194417
H	-4.9066049	13.3884661	-0.7839255
H	-4.3982517	14.6306159	-1.9277047
C	-5.7132322	20.7607177	1.3760911
H	-4.7810591	20.2060984	1.5520483
H	-6.5252382	20.2392439	1.9035027
C	-6.1944985	15.1353582	-0.8237349
H	-6.5033658	15.8621404	-1.5963066
C	-4.7499369	22.9910684	-0.3270848
C	-7.3115372	12.7091152	-2.0774062
H	-7.8561362	12.2092539	-2.8894816
H	-7.0328117	11.9225567	-1.3575350
C	-8.2134747	13.7359162	-1.3862184
H	-9.1065484	13.2487851	-0.9726812
H	-8.5717693	14.4678887	-2.1300307
C	-1.8767611	13.4389327	-2.4996059
H	-2.8557981	13.1130428	-2.8792080
H	-2.0700056	14.1499589	-1.6785105
C	-4.1967770	13.3598696	3.3202718
H	-4.0779711	14.0692686	4.1553338
H	-4.7227988	12.4851085	3.7257772
C	-5.7203346	15.5605906	-6.2299514
H	-6.2390769	16.3738708	-6.7550958
H	-6.3842393	15.2376716	-5.4107644
C	-2.8173081	12.9600500	2.7871431
H	-2.9361064	12.1736257	2.0226679
H	-2.2130520	12.5216688	3.5923341
C	-2.9309361	14.8237982	1.0779218
H	-3.0557229	14.1210692	0.2397467
H	-2.4152214	15.7097632	0.6765262
C	-9.0176608	20.1085267	-3.7344592
H	-10.0221197	20.5386245	-3.6244039
H	-8.4234605	20.8430713	-4.3024529
C	-4.6978881	13.2656882	-6.4726862
H	-4.4848935	12.4442243	-7.1696891
H	-5.3195722	12.8407061	-5.6682085
C	-6.0429305	13.3725130	-2.6210563
H	-5.3862536	12.6247243	-3.0896486
H	-6.3224911	14.0890534	-3.4120835
C	-5.4694654	14.3839479	-7.1764702
H	-6.4218441	14.0019958	-7.5675911
H	-4.8934280	14.7352162	-8.0484514
C	-1.1013244	12.2363301	-1.9511454

```
H    -1.6782031   11.7700427   -1.1397351
H    -1.0045864   11.4756071   -2.7424928
C    -2.0890917   14.1594955    2.1721525
H    -1.8717780   14.9015429    2.9577758
H    -1.1196261   13.8528716    1.7549930
H    -0.9423044   17.0023829   -1.6284749
C     0.2604639   19.5072686   -3.8470858
H     0.4904538   20.0972932   -4.7514498
C     1.2770049   18.3565303   -3.7466282
H     1.2563521   17.7452021   -4.6599741
H     0.9950042   17.7011362   -2.9073083
C     0.3138912   14.5707520   -3.1095340
H     0.8900963   15.0372595   -3.9206492
H     0.2191283   15.3211732   -2.3099334
C    -1.1304337   16.4334977   -5.5166173
H    -0.0733643   16.2745487   -5.2726193
H    -1.3233925   15.8964592   -6.4559636
C    -1.4159810   17.9333519   -5.6917554
H    -2.3921734   18.0826760   -6.1696065
H    -0.6633917   18.3851177   -6.3527659
C    -3.6314532   14.9662017   -4.9284689
H    -4.2380089   14.6176652   -4.0784538
C    -2.5323849   20.3523159   -4.4247966
H    -2.5208556   20.9105896   -3.4721791
C    -6.5221633   17.2050402    1.2180331
H    -7.2341876   16.5631811    1.7540472
H    -5.9960376   17.8077986    1.9709084
H    -5.6744567   23.5579412   -0.5237362
H    -3.9424666   23.5159395   -0.8566981
C    -1.8697156   19.5979851    0.1646023
N    -3.0433954   18.8543141    0.5785126
H    -1.9745774   19.9344565   -0.8775088
H    -0.9265804   19.0182845    0.2328791
H    -1.7406693   20.5056191    0.7831061
C    -2.9618446   18.4562117    1.9694331
H    -2.7192544   19.3263006    2.6060878
H    -2.1887499   17.6877825    2.1746322
H    -3.9241803   18.0668014    2.3288928

SCF energy GEOOPT =  -7391.436447529 H
ZPE =   3828. kJ/mol
FREEH energy =  4017.63 kJ/mol
FREEH entropy =   1.59978 kJ/mol/K

$vibrational spectrum
#  mode     symmetry     wave number   IR intensity    selection rules
#                         cm**(-1)        km/mol          IR     RAMAN
     1                      -0.00         0.00000         -        -
     2                       0.00         0.00000         -        -
     3                       0.00         0.00000         -        -
     4                       0.00         0.00000         -        -
     5                       0.00         0.00000         -        -
     6                       0.00         0.00000         -        -
     7         a            16.53         0.01412        YES      YES
     8         a            20.70         0.01592        YES      YES
     9         a            27.87         0.01660        YES      YES
    10         a            29.57         0.06911        YES      YES
    11         a            30.31         0.05954        YES      YES
```

12	a	32.62	0.00599	YES	YES
13	a	33.25	0.06466	YES	YES
14	a	36.25	0.01295	YES	YES
15	a	39.67	0.08195	YES	YES
16	a	41.72	0.00762	YES	YES
17	a	43.12	0.02144	YES	YES
18	a	47.50	0.03721	YES	YES
19	a	50.93	0.12058	YES	YES
20	a	51.59	0.01832	YES	YES
21	a	55.38	0.09815	YES	YES
22	a	59.57	0.29302	YES	YES
23	a	60.30	0.06459	YES	YES
24	a	64.37	0.28426	YES	YES
25	a	65.65	0.05342	YES	YES
26	a	68.90	0.20792	YES	YES
27	a	71.73	0.05530	YES	YES
28	a	73.54	0.20772	YES	YES
29	a	75.36	0.09285	YES	YES
30	a	79.11	0.04985	YES	YES
31	a	80.08	0.15056	YES	YES
32	a	86.03	0.23939	YES	YES
33	a	89.55	0.42411	YES	YES
34	a	91.25	0.41919	YES	YES
35	a	97.39	0.43250	YES	YES
36	a	105.16	0.22018	YES	YES
37	a	109.42	0.24660	YES	YES
38	a	113.93	0.12465	YES	YES
39	a	119.41	0.46399	YES	YES
40	a	124.64	1.16886	YES	YES
41	a	132.42	0.35069	YES	YES
42	a	135.67	0.06100	YES	YES
43	a	139.64	0.20396	YES	YES
44	a	140.20	2.49116	YES	YES
45	a	142.99	0.78952	YES	YES
46	a	150.18	6.89901	YES	YES
47	a	153.79	0.83942	YES	YES
48	a	156.91	0.35989	YES	YES
49	a	165.45	1.44843	YES	YES
50	a	174.90	2.04961	YES	YES
51	a	182.39	3.44573	YES	YES
52	a	184.95	4.17759	YES	YES
53	a	189.05	1.78265	YES	YES
54	a	200.96	0.47279	YES	YES
55	a	205.89	1.67717	YES	YES
56	a	215.00	0.15539	YES	YES
57	a	216.66	0.22739	YES	YES
58	a	220.20	0.04582	YES	YES
59	a	220.92	0.10604	YES	YES
60	a	225.35	0.14251	YES	YES
61	a	226.28	0.19903	YES	YES
62	a	227.67	0.06022	YES	YES
63	a	228.09	0.29994	YES	YES
64	a	234.85	1.33577	YES	YES
65	a	236.54	0.69792	YES	YES
66	a	238.43	0.64635	YES	YES
67	a	239.16	0.57344	YES	YES
68	a	239.49	0.69157	YES	YES
69	a	241.36	0.79176	YES	YES

70	a	246.16	4.98629	YES	YES
71	a	249.92	0.07885	YES	YES
72	a	265.94	6.88793	YES	YES
73	a	269.60	6.90911	YES	YES
74	a	280.56	1.86980	YES	YES
75	a	285.23	0.35938	YES	YES
76	a	286.83	0.77476	YES	YES
77	a	291.51	0.41875	YES	YES
78	a	300.52	6.21497	YES	YES
79	a	305.13	0.29451	YES	YES
80	a	312.79	0.19377	YES	YES
81	a	313.82	0.39262	YES	YES
82	a	321.64	7.01370	YES	YES
83	a	330.17	1.28734	YES	YES
84	a	331.46	0.03100	YES	YES
85	a	336.29	0.38301	YES	YES
86	a	336.67	0.32782	YES	YES
87	a	338.25	0.38795	YES	YES
88	a	340.23	1.49878	YES	YES
89	a	372.91	4.78591	YES	YES
90	a	377.05	2.75923	YES	YES
91	a	381.62	0.76693	YES	YES
92	a	384.09	0.46398	YES	YES
93	a	396.44	0.50819	YES	YES
94	a	397.59	0.81387	YES	YES
95	a	406.14	0.88456	YES	YES
96	a	412.25	0.88795	YES	YES
97	a	419.24	3.41129	YES	YES
98	a	420.57	1.89687	YES	YES
99	a	423.40	3.08003	YES	YES
100	a	428.45	0.41272	YES	YES
101	a	430.42	0.34787	YES	YES
102	a	430.96	0.34513	YES	YES
103	a	431.41	0.33576	YES	YES
104	a	431.51	0.09818	YES	YES
105	a	433.04	0.95618	YES	YES
106	a	433.79	0.44037	YES	YES
107	a	434.61	0.57058	YES	YES
108	a	437.20	0.10698	YES	YES
109	a	445.95	2.66855	YES	YES
110	a	458.61	5.02401	YES	YES
111	a	459.49	3.86615	YES	YES
112	a	470.52	2.62773	YES	YES
113	a	491.56	3.10891	YES	YES
114	a	493.86	7.59046	YES	YES
115	a	497.51	3.91555	YES	YES
116	a	499.38	1.34739	YES	YES
117	a	500.13	0.68424	YES	YES
118	a	504.70	0.78229	YES	YES
119	a	511.41	16.93639	YES	YES
120	a	511.88	2.02866	YES	YES
121	a	519.47	14.08933	YES	YES
122	a	543.64	98.78605	YES	YES
123	a	604.65	88.70916	YES	YES
124	a	630.39	11.41967	YES	YES
125	a	632.98	30.18241	YES	YES
126	a	645.30	12.28083	YES	YES
127	a	663.57	12.16345	YES	YES

128	a	701.38	1.18881	YES	YES
129	a	718.71	1.35273	YES	YES
130	a	720.06	1.41025	YES	YES
131	a	730.07	14.08577	YES	YES
132	a	732.78	2.04814	YES	YES
133	a	733.34	3.19573	YES	YES
134	a	736.60	2.69779	YES	YES
135	a	737.94	2.83431	YES	YES
136	a	771.52	0.99345	YES	YES
137	a	774.06	0.33866	YES	YES
138	a	774.17	0.27609	YES	YES
139	a	774.38	0.77779	YES	YES
140	a	775.14	0.13935	YES	YES
141	a	775.67	0.42998	YES	YES
142	a	777.05	0.49564	YES	YES
143	a	777.47	0.17555	YES	YES
144	a	779.36	14.27643	YES	YES
145	a	789.16	13.98312	YES	YES
146	a	806.89	0.72389	YES	YES
147	a	810.42	1.84002	YES	YES
148	a	811.03	3.47801	YES	YES
149	a	811.36	9.48879	YES	YES
150	a	813.06	2.67873	YES	YES
151	a	813.48	0.15612	YES	YES
152	a	814.19	4.69020	YES	YES
153	a	818.89	1.92006	YES	YES
154	a	833.78	3.94271	YES	YES
155	a	833.93	7.37934	YES	YES
156	a	836.97	6.58529	YES	YES
157	a	837.56	0.23329	YES	YES
158	a	838.36	0.92107	YES	YES
159	a	839.05	2.93748	YES	YES
160	a	839.56	6.00455	YES	YES
161	a	839.73	5.34326	YES	YES
162	a	850.73	14.43111	YES	YES
163	a	853.86	14.20203	YES	YES
164	a	874.21	2.88993	YES	YES
165	a	874.47	1.43498	YES	YES
166	a	876.34	3.09933	YES	YES
167	a	876.89	5.89994	YES	YES
168	a	877.08	1.93144	YES	YES
169	a	878.02	1.11764	YES	YES
170	a	878.27	2.87657	YES	YES
171	a	878.94	2.78829	YES	YES
172	a	879.68	4.09500	YES	YES
173	a	880.14	1.14086	YES	YES
174	a	880.99	2.59922	YES	YES
175	a	881.56	3.80291	YES	YES
176	a	883.73	3.66341	YES	YES
177	a	884.09	4.45706	YES	YES
178	a	885.94	2.53476	YES	YES
179	a	886.54	4.15893	YES	YES
180	a	904.28	1.19340	YES	YES
181	a	905.30	2.04731	YES	YES
182	a	905.90	3.41592	YES	YES
183	a	906.21	2.47708	YES	YES
184	a	908.99	0.62398	YES	YES
185	a	910.69	0.79362	YES	YES

186	a	910.74	0.64402	YES	YES
187	a	911.49	1.11878	YES	YES
188	a	953.62	74.17394	YES	YES
189	a	978.17	0.37533	YES	YES
190	a	985.48	1.94464	YES	YES
191	a	988.24	8.72641	YES	YES
192	a	988.57	9.05747	YES	YES
193	a	989.27	5.37462	YES	YES
194	a	989.74	15.36093	YES	YES
195	a	990.63	8.70407	YES	YES
196	a	991.42	8.66895	YES	YES
197	a	991.80	10.91108	YES	YES
198	a	993.51	4.07102	YES	YES
199	a	1014.78	0.38119	YES	YES
200	a	1016.17	0.46065	YES	YES
201	a	1017.58	0.26241	YES	YES
202	a	1018.12	0.18919	YES	YES
203	a	1018.51	0.43836	YES	YES
204	a	1019.18	0.38988	YES	YES
205	a	1019.70	0.38114	YES	YES
206	a	1021.87	0.53445	YES	YES
207	a	1031.15	1.21026	YES	YES
208	a	1031.91	0.65889	YES	YES
209	a	1033.20	1.53352	YES	YES
210	a	1034.27	0.70304	YES	YES
211	a	1038.29	3.35733	YES	YES
212	a	1039.17	1.15593	YES	YES
213	a	1040.92	1.25999	YES	YES
214	a	1041.39	0.97846	YES	YES
215	a	1047.49	4.18236	YES	YES
216	a	1058.16	2.05081	YES	YES
217	a	1059.02	1.96789	YES	YES
218	a	1059.73	0.22678	YES	YES
219	a	1061.48	1.19239	YES	YES
220	a	1062.19	1.64558	YES	YES
221	a	1065.03	1.52875	YES	YES
222	a	1066.06	0.06303	YES	YES
223	a	1068.27	1.19447	YES	YES
224	a	1070.75	0.06384	YES	YES
225	a	1071.55	0.16470	YES	YES
226	a	1072.11	0.91248	YES	YES
227	a	1072.58	0.13077	YES	YES
228	a	1072.89	0.14593	YES	YES
229	a	1073.35	0.17490	YES	YES
230	a	1074.18	0.11220	YES	YES
231	a	1074.53	0.02722	YES	YES
232	a	1077.06	2.07860	YES	YES
233	a	1077.96	0.78812	YES	YES
234	a	1082.73	2.94513	YES	YES
235	a	1091.38	1.01207	YES	YES
236	a	1094.75	4.18568	YES	YES
237	a	1095.37	4.05481	YES	YES
238	a	1096.56	8.81212	YES	YES
239	a	1100.95	3.74641	YES	YES
240	a	1104.42	1.73318	YES	YES
241	a	1106.43	8.35011	YES	YES
242	a	1111.77	1.23680	YES	YES
243	a	1114.39	6.25653	YES	YES

244	a	1122.44	1.54262	YES	YES
245	a	1132.09	2.23630	YES	YES
246	a	1160.01	8.34953	YES	YES
247	a	1161.21	0.19224	YES	YES
248	a	1162.33	12.68851	YES	YES
249	a	1166.52	5.56588	YES	YES
250	a	1168.74	9.25052	YES	YES
251	a	1169.70	13.27361	YES	YES
252	a	1170.52	7.80665	YES	YES
253	a	1171.60	2.70827	YES	YES
254	a	1176.29	46.02505	YES	YES
255	a	1178.66	20.71535	YES	YES
256	a	1180.07	13.88026	YES	YES
257	a	1181.64	14.34197	YES	YES
258	a	1182.30	6.47827	YES	YES
259	a	1187.23	0.39375	YES	YES
260	a	1189.49	1.15230	YES	YES
261	a	1192.27	2.02662	YES	YES
262	a	1211.42	1.14141	YES	YES
263	a	1227.52	11.97718	YES	YES
264	a	1238.80	0.03483	YES	YES
265	a	1242.06	0.33759	YES	YES
266	a	1244.55	0.83179	YES	YES
267	a	1246.25	0.49905	YES	YES
268	a	1246.49	0.03120	YES	YES
269	a	1247.19	1.01558	YES	YES
270	a	1247.87	1.22719	YES	YES
271	a	1248.36	2.03540	YES	YES
272	a	1250.48	0.26772	YES	YES
273	a	1251.01	0.72859	YES	YES
274	a	1251.70	0.12031	YES	YES
275	a	1255.07	1.67807	YES	YES
276	a	1255.39	5.64829	YES	YES
277	a	1256.34	4.68137	YES	YES
278	a	1256.69	4.93897	YES	YES
279	a	1257.34	1.59188	YES	YES
280	a	1257.90	5.75662	YES	YES
281	a	1258.25	2.54837	YES	YES
282	a	1259.53	3.94554	YES	YES
283	a	1261.22	0.90583	YES	YES
284	a	1261.81	1.71983	YES	YES
285	a	1262.86	1.69918	YES	YES
286	a	1263.33	0.79842	YES	YES
287	a	1264.42	0.64975	YES	YES
288	a	1266.31	0.17899	YES	YES
289	a	1267.32	1.93108	YES	YES
290	a	1269.77	1.50826	YES	YES
291	a	1272.69	2.10741	YES	YES
292	a	1281.85	2.69644	YES	YES
293	a	1282.95	0.90876	YES	YES
294	a	1284.89	4.40685	YES	YES
295	a	1285.50	1.81385	YES	YES
296	a	1288.03	4.07244	YES	YES
297	a	1290.10	4.03807	YES	YES
298	a	1290.93	2.59859	YES	YES
299	a	1294.02	3.32593	YES	YES
300	a	1305.59	1.12092	YES	YES
301	a	1309.21	0.28295	YES	YES

302	a	1311.32	1.03524	YES	YES
303	a	1311.57	0.50136	YES	YES
304	a	1313.73	1.28122	YES	YES
305	a	1314.51	0.28110	YES	YES
306	a	1316.89	2.85493	YES	YES
307	a	1317.88	0.66156	YES	YES
308	a	1320.06	1.47845	YES	YES
309	a	1321.25	1.27476	YES	YES
310	a	1322.67	0.65502	YES	YES
311	a	1322.92	0.64386	YES	YES
312	a	1323.40	0.16522	YES	YES
313	a	1324.29	0.89992	YES	YES
314	a	1324.52	2.90359	YES	YES
315	a	1324.65	1.38461	YES	YES
316	a	1324.83	0.33367	YES	YES
317	a	1324.91	0.63856	YES	YES
318	a	1325.44	0.35345	YES	YES
319	a	1325.99	5.81293	YES	YES
320	a	1327.07	2.15886	YES	YES
321	a	1329.86	0.02327	YES	YES
322	a	1330.19	10.23770	YES	YES
323	a	1331.48	3.55197	YES	YES
324	a	1336.50	0.23621	YES	YES
325	a	1336.83	0.48612	YES	YES
326	a	1337.19	0.21387	YES	YES
327	a	1337.26	0.18536	YES	YES
328	a	1338.02	0.45582	YES	YES
329	a	1338.64	0.18499	YES	YES
330	a	1338.99	0.01486	YES	YES
331	a	1339.49	0.34716	YES	YES
332	a	1344.79	2.10792	YES	YES
333	a	1345.69	0.15369	YES	YES
334	a	1346.61	1.00929	YES	YES
335	a	1346.68	1.29728	YES	YES
336	a	1347.10	0.78163	YES	YES
337	a	1348.09	3.04170	YES	YES
338	a	1348.27	0.24954	YES	YES
339	a	1350.67	1.05170	YES	YES
340	a	1392.38	1.00009	YES	YES
341	a	1401.22	5.38438	YES	YES
342	a	1406.22	7.40065	YES	YES
343	a	1407.77	11.22470	YES	YES
344	a	1409.65	6.90632	YES	YES
345	a	1417.20	3.30535	YES	YES
346	a	1421.96	0.57270	YES	YES
347	a	1425.82	3.95719	YES	YES
348	a	1426.91	5.40883	YES	YES
349	a	1427.70	17.49663	YES	YES
350	a	1428.40	0.74470	YES	YES
351	a	1431.98	1.98341	YES	YES
352	a	1432.69	1.67719	YES	YES
353	a	1433.36	0.60108	YES	YES
354	a	1433.78	8.61088	YES	YES
355	a	1434.41	1.68516	YES	YES
356	a	1435.25	6.29393	YES	YES
357	a	1435.85	2.20580	YES	YES
358	a	1437.10	4.38992	YES	YES
359	a	1437.46	8.39541	YES	YES

360	a	1437.63	4.26654	YES	YES
361	a	1438.22	2.77485	YES	YES
362	a	1438.28	1.14714	YES	YES
363	a	1438.69	5.11645	YES	YES
364	a	1438.85	8.21180	YES	YES
365	a	1439.02	9.19217	YES	YES
366	a	1439.55	6.37182	YES	YES
367	a	1439.76	4.05342	YES	YES
368	a	1440.14	4.89881	YES	YES
369	a	1440.39	3.18816	YES	YES
370	a	1440.94	2.86527	YES	YES
371	a	1441.34	3.74663	YES	YES
372	a	1442.18	13.81878	YES	YES
373	a	1443.68	5.61568	YES	YES
374	a	1444.15	17.18584	YES	YES
375	a	1444.92	15.81289	YES	YES
376	a	1445.49	26.38094	YES	YES
377	a	1446.45	13.50167	YES	YES
378	a	1446.90	17.29553	YES	YES
379	a	1447.09	7.42831	YES	YES
380	a	1452.77	1.60000	YES	YES
381	a	1453.18	8.45653	YES	YES
382	a	1454.44	2.56094	YES	YES
383	a	1454.67	0.53078	YES	YES
384	a	1455.07	1.68372	YES	YES
385	a	1456.04	1.42059	YES	YES
386	a	1456.40	0.40103	YES	YES
387	a	1457.28	11.59937	YES	YES
388	a	1458.21	0.56876	YES	YES
389	a	1470.69	7.25522	YES	YES
390	a	1833.27	127.02437	YES	YES
391	a	2860.01	74.03283	YES	YES
392	a	2862.96	111.20182	YES	YES
393	a	2912.84	68.44554	YES	YES
394	a	2925.65	19.04608	YES	YES
395	a	2930.28	32.69731	YES	YES
396	a	2933.82	1.19338	YES	YES
397	a	2935.79	2.40915	YES	YES
398	a	2937.38	16.41486	YES	YES
399	a	2937.62	2.43748	YES	YES
400	a	2938.96	16.69317	YES	YES
401	a	2940.51	11.84111	YES	YES
402	a	2940.81	10.06200	YES	YES
403	a	2941.66	5.84066	YES	YES
404	a	2941.80	7.91781	YES	YES
405	a	2942.33	19.77930	YES	YES
406	a	2942.36	10.88573	YES	YES
407	a	2942.68	8.81304	YES	YES
408	a	2944.43	6.11703	YES	YES
409	a	2944.79	31.99632	YES	YES
410	a	2945.45	7.08596	YES	YES
411	a	2945.64	1.75968	YES	YES
412	a	2946.72	13.54409	YES	YES
413	a	2948.36	12.84171	YES	YES
414	a	2948.89	4.32671	YES	YES
415	a	2949.26	9.55752	YES	YES
416	a	2951.92	12.88917	YES	YES
417	a	2952.53	15.26399	YES	YES

418	a	2952.69	22.44758	YES	YES
419	a	2952.71	9.61462	YES	YES
420	a	2953.72	4.19991	YES	YES
421	a	2953.81	5.15659	YES	YES
422	a	2955.24	4.12386	YES	YES
423	a	2956.06	20.54041	YES	YES
424	a	2956.43	4.25135	YES	YES
425	a	2957.52	4.79671	YES	YES
426	a	2958.12	37.77411	YES	YES
427	a	2959.01	31.49399	YES	YES
428	a	2959.27	0.15845	YES	YES
429	a	2960.14	30.56616	YES	YES
430	a	2960.56	33.62445	YES	YES
431	a	2961.01	29.17747	YES	YES
432	a	2961.18	26.72716	YES	YES
433	a	2961.39	37.01447	YES	YES
434	a	2961.65	8.49829	YES	YES
435	a	2963.55	25.56552	YES	YES
436	a	2963.70	25.82928	YES	YES
437	a	2964.39	26.57505	YES	YES
438	a	2965.51	15.15896	YES	YES
439	a	2967.54	8.45165	YES	YES
440	a	2968.97	20.02799	YES	YES
441	a	2979.51	7.87050	YES	YES
442	a	2980.28	36.45353	YES	YES
443	a	2981.60	10.06399	YES	YES
444	a	2984.87	25.97707	YES	YES
445	a	2986.20	2.44823	YES	YES
446	a	2987.53	6.86685	YES	YES
447	a	2988.39	4.51922	YES	YES
448	a	2990.44	30.74299	YES	YES
449	a	2990.90	4.01198	YES	YES
450	a	2992.88	18.39795	YES	YES
451	a	2995.71	29.19536	YES	YES
452	a	2996.78	15.25555	YES	YES
453	a	2999.78	28.08736	YES	YES
454	a	3001.50	19.60491	YES	YES
455	a	3001.94	24.43801	YES	YES
456	a	3004.08	0.99159	YES	YES
457	a	3004.20	44.15457	YES	YES
458	a	3004.60	39.78787	YES	YES
459	a	3007.55	8.61268	YES	YES
460	a	3007.78	0.69608	YES	YES
461	a	3008.54	30.30354	YES	YES
462	a	3009.33	5.97155	YES	YES
463	a	3009.40	35.49740	YES	YES
464	a	3009.95	38.59176	YES	YES
465	a	3011.56	15.74455	YES	YES
466	a	3012.02	4.52816	YES	YES
467	a	3012.05	27.64161	YES	YES
468	a	3014.11	20.04595	YES	YES
469	a	3014.21	36.20679	YES	YES
470	a	3014.30	14.60371	YES	YES
471	a	3015.07	13.65757	YES	YES
472	a	3015.53	22.82132	YES	YES
473	a	3015.56	18.24580	YES	YES
474	a	3016.17	26.88393	YES	YES
475	a	3016.66	15.00838	YES	YES

476	a	3016.68	30.98375	YES	YES
477	a	3017.15	30.57763	YES	YES
478	a	3017.41	23.73343	YES	YES
479	a	3017.42	32.71107	YES	YES
480	a	3017.98	18.59733	YES	YES
481	a	3018.27	33.86313	YES	YES
482	a	3018.59	20.49646	YES	YES
483	a	3019.09	9.53882	YES	YES
484	a	3019.17	16.59865	YES	YES
485	a	3019.21	48.86617	YES	YES
486	a	3023.97	22.03718	YES	YES
487	a	3025.17	25.36665	YES	YES
488	a	3025.84	8.26700	YES	YES
489	a	3030.91	1.45399	YES	YES
490	a	3034.79	0.41767	YES	YES
491	a	3038.87	1.16910	YES	YES
492	a	3049.81	0.24298	YES	YES

$end

Double hybrid single point energy = -7384.071958556270 H
COSMO energy + OC correction = -7391.5829919471 H (in *o*DFB)

6.2.29 [H{Ga(dcpe)}$_2$(NMe$_2$)]$^{2+}$ (*anti*-periplanar)

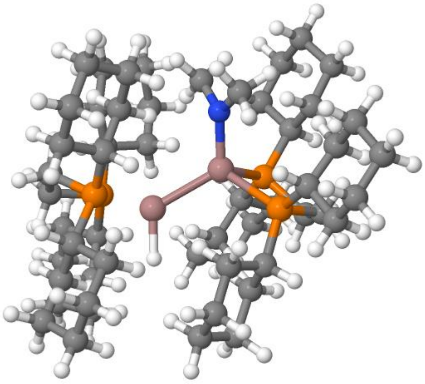

Method: (RI-)BP86(D3BJ)/def2-TZVPP
Symmetry: c1

Cartesian coordinates in Ångström:

Ga	-0.4319512	0.2874977	-2.0002729
P	-2.5051482	-0.7183509	-2.8853319
P	-1.5769251	2.4707777	-2.4372908
C	-2.4128390	-1.9507929	-4.2736453
H	-1.5771060	-2.6123144	-3.9873320
C	-2.0545197	-1.2758985	-5.6105533
H	-2.9092437	-0.6662713	-5.9430817
H	-1.1997097	-0.5973785	-5.4991655
C	-1.7770812	-2.3369872	-6.6817644
H	-1.5182694	-1.8446637	-7.6290378
H	-0.8978022	-2.9324587	-6.3853292
C	-2.9914292	-3.2528650	-6.8654138
H	-2.7685321	-4.0408441	-7.5969459
H	-3.8251407	-2.6659872	-7.2844778
C	-3.4284453	-3.8776960	-5.5371201
H	-2.6497875	-4.5744085	-5.1846498

H	-4.3408254	-4.4739803	-5.6733548
C	-3.6708467	-2.8213053	-4.4467343
H	-3.9421448	-3.3245343	-3.5097904
H	-4.5229609	-2.1818190	-4.7290325
C	-3.5062490	-1.4970484	-1.5495479
H	-4.4258256	-1.8552060	-2.0423043
C	-2.7519812	-2.7054707	-0.9725854
H	-2.5062854	-3.4257451	-1.7661500
H	-1.7919462	-2.3547551	-0.5556849
C	-3.5727055	-3.3841923	0.1294438
H	-4.4953718	-3.7966906	-0.3097335
H	-3.0169226	-4.2401827	0.5350999
C	-3.9304578	-2.3909991	1.2374337
H	-4.5284162	-2.8849546	2.0150239
H	-3.0044871	-2.0375172	1.7217865
C	-4.6911583	-1.1894144	0.6703229
H	-4.9108218	-0.4610861	1.4632343
H	-5.6637332	-1.5262684	0.2767422
C	-3.9065055	-0.4968274	-0.4520459
H	-4.5216325	0.3065031	-0.8800105
H	-3.0013384	-0.0330022	-0.0300673
C	-3.5209535	0.6802007	-3.5350893
H	-3.2410175	0.7911761	-4.5886163
H	-4.5790353	0.3813926	-3.5180078
C	-3.3328597	2.0127936	-2.8024793
H	-3.8428112	1.9930112	-1.8312899
H	-3.7898237	2.8244436	-3.3850965
C	-0.9691904	3.3890900	-3.9213263
H	-1.7631849	4.1129851	-4.1739717
C	0.3167115	4.1657908	-3.5903425
H	1.0734955	3.4661396	-3.2040701
H	0.1236333	4.8984353	-2.7943987
C	0.8729185	4.8820670	-4.8272758
H	0.1694440	5.6734771	-5.1326395
H	1.8131919	5.3855693	-4.5633612
C	1.0857691	3.9136734	-5.9936001
H	1.8661296	3.1814962	-5.7259920
H	1.4564730	4.4557985	-6.8735196
C	-0.2104222	3.1746229	-6.3342181
H	-0.0452518	2.4575724	-7.1501600
H	-0.9579786	3.8976678	-6.6989071
C	-0.7737768	2.4351505	-5.1147241
H	-0.0907810	1.6250830	-4.8146534
H	-1.7294207	1.9627363	-5.3818895
C	-1.7420539	3.7165696	-1.0800930
H	-0.7226529	4.1085834	-0.9421839
C	-2.1755333	3.0612020	0.2407669
H	-1.4694785	2.2614464	0.5168064
H	-3.1565427	2.5754400	0.1179471
C	-2.2773953	4.1023440	1.3621882
H	-2.6243669	3.6170325	2.2850659
H	-1.2718530	4.5051979	1.5741694
C	-3.1989298	5.2635796	0.9804811
H	-4.2290965	4.8895760	0.8611864
H	-3.2283725	6.0047198	1.7902466
C	-2.7386457	5.9171391	-0.3246054
H	-1.7453717	6.3730482	-0.1789555
H	-3.4174838	6.7316099	-0.6106449

C	-2.6621482	4.8910761	-1.4623289
H	-3.6758265	4.5138891	-1.6695908
H	-2.3067708	5.3749702	-2.3824584
Ga	-0.0241109	-0.1489400	0.4049604
P	1.3371784	1.2913427	1.8182660
P	1.4906804	-1.9355583	1.0625166
C	0.3879500	2.2295949	3.1009832
H	-0.2111137	2.9486512	2.5192686
C	-0.5865638	1.3159454	3.8663577
H	-0.0168534	0.5441876	4.4090458
H	-1.2422065	0.7865279	3.1620820
C	-1.4137140	2.1183722	4.8789690
H	-2.0724687	1.4358742	5.4333459
H	-2.0717713	2.8161922	4.3355778
C	-0.5201342	2.9074330	5.8389445
H	-1.1335724	3.5025457	6.5284409
H	0.0574503	2.2049781	6.4624642
C	0.4439496	3.8142966	5.0705128
H	-0.1286260	4.5833265	4.5260674
H	1.1081624	4.3500112	5.7620957
C	1.2909223	3.0183704	4.0669814
H	1.9503975	3.7038597	3.5176533
H	1.9408093	2.3183789	4.6167658
C	2.4566636	2.5060588	1.0011374
H	2.9288993	3.0694672	1.8234687
C	1.6423762	3.4827009	0.1415928
H	0.8617596	3.9674005	0.7460715
H	1.1235934	2.9060279	-0.6443114
C	2.5464304	4.5405077	-0.5003989
H	2.9809731	5.1682609	0.2938180
H	1.9451333	5.2106349	-1.1308230
C	3.6687155	3.8963641	-1.3190829
H	4.3412696	4.6702868	-1.7122626
H	3.2364984	3.3905398	-2.1968057
C	4.4665694	2.8884496	-0.4844062
H	5.2201692	2.3866535	-1.1065475
H	5.0206795	3.4277340	0.3006232
C	3.5699322	1.8345273	0.1828447
H	4.1869667	1.1966145	0.8306865
H	3.1221000	1.1784232	-0.5798326
C	2.4338183	0.1447853	2.7657548
H	1.8704755	-0.1372667	3.6637680
H	3.3177475	0.7019500	3.1064062
C	2.8618514	-1.1047441	1.9849648
H	3.6177552	-0.8436875	1.2337370
H	3.3230789	-1.8357878	2.6632144
C	0.7662145	-3.1624506	2.2379356
H	1.6168563	-3.7831325	2.5683681
C	-0.2536500	-4.0535260	1.5140198
H	-1.0529942	-3.4099405	1.1158360
H	0.2100789	-4.5587422	0.6549221
C	-0.8637787	-5.0888560	2.4662622
H	-0.0772622	-5.7891243	2.7909404
H	-1.6092177	-5.6902764	1.9269663
C	-1.4919267	-4.4188332	3.6908716
H	-2.3472331	-3.8012415	3.3711941
H	-1.8921338	-5.1776141	4.3762449
C	-0.4724112	-3.5364206	4.4155975

```
H    -0.9394007   -3.0264110    5.2693644
H     0.3304941   -4.1678935    4.8292900
C     0.1420961   -2.4926119    3.4736308
H    -0.6347330   -1.7840011    3.1449555
H     0.9001581   -1.9122826    4.0179748
C     2.3163190   -2.9011145   -0.2761866
H     1.4832309   -3.3934896   -0.8077937
C     3.0210412   -1.9678708   -1.2742871
H     2.2998951   -1.2433982   -1.6831304
H     3.8096113   -1.3980057   -0.7564424
C     3.6685894   -2.7758343   -2.4073084
H     4.1946865   -2.0933760   -3.0897467
H     2.8757087   -3.2628577   -2.9985455
C     4.6258820   -3.8420629   -1.8684952
H     5.4731927   -3.3511091   -1.3616600
H     5.0532033   -4.4234769   -2.6963697
C     3.9116443   -4.7700567   -0.8829846
H     3.1292716   -5.3378950   -1.4131767
H     4.6099070   -5.5109188   -0.4707986
C     3.2672516   -3.9833513    0.2664565
H     4.0597296   -3.5045930    0.8645087
H     2.7341095   -4.6726568    0.9352595
H    -1.2639688   -0.3961241    1.3525492
N     0.9958478    0.0143071   -3.1965964
C     1.0728735   -0.9310349   -4.2882967
C     2.1051330    0.9393743   -3.1922278
H     2.0640201   -1.4175339   -4.3079050
H     0.9348868   -0.4634866   -5.2849690
H     0.3265170   -1.7264446   -4.1805237
H     2.1580591    1.5584070   -4.1104986
H     3.0773252    0.4149089   -3.1083628
H     2.0295136    1.6286227   -2.3416010

SCF energy GEOOPT =  -7391.442612933 H
ZPE =   3830. kJ/mol
FREEH energy =  4019.26 kJ/mol
FREEH entropy =   1.59770 kJ/mol/K

$vibrational spectrum
#  mode     symmetry     wave number   IR intensity    selection rules
#                         cm**(-1)        km/mol          IR     RAMAN
     1                       -0.00         0.00000         -       -
     2                       -0.00         0.00000         -       -
     3                       -0.00         0.00000         -       -
     4                       -0.00         0.00000         -       -
     5                        0.00         0.00000         -       -
     6                        0.00         0.00000         -       -
     7         a              9.05         0.01399        YES     YES
     8         a              9.84         0.00824        YES     YES
     9         a             19.79         0.03319        YES     YES
    10         a             25.84         0.02719        YES     YES
    11         a             29.44         0.01923        YES     YES
    12         a             32.78         0.06258        YES     YES
    13         a             37.81         0.03414        YES     YES
    14         a             42.28         0.12496        YES     YES
    15         a             45.03         0.03541        YES     YES
    16         a             46.82         0.02124        YES     YES
    17         a             51.31         0.03503        YES     YES
```

18	a	54.09	0.09223	YES	YES
19	a	56.75	0.44180	YES	YES
20	a	57.95	0.07058	YES	YES
21	a	62.02	0.26274	YES	YES
22	a	63.19	0.07373	YES	YES
23	a	63.81	0.09560	YES	YES
24	a	66.81	0.35266	YES	YES
25	a	70.12	0.05939	YES	YES
26	a	72.10	0.29088	YES	YES
27	a	73.40	0.73812	YES	YES
28	a	76.15	0.23688	YES	YES
29	a	79.03	0.01945	YES	YES
30	a	80.81	0.40115	YES	YES
31	a	85.56	0.02311	YES	YES
32	a	85.81	0.16380	YES	YES
33	a	90.37	0.59131	YES	YES
34	a	95.38	0.80932	YES	YES
35	a	95.91	0.09460	YES	YES
36	a	102.79	0.69369	YES	YES
37	a	118.45	5.48594	YES	YES
38	a	127.36	5.06718	YES	YES
39	a	130.24	1.96451	YES	YES
40	a	131.32	1.34233	YES	YES
41	a	135.18	2.68171	YES	YES
42	a	139.42	0.17538	YES	YES
43	a	142.66	4.59605	YES	YES
44	a	145.03	1.43964	YES	YES
45	a	149.08	2.57547	YES	YES
46	a	152.37	0.91293	YES	YES
47	a	164.61	2.16238	YES	YES
48	a	165.93	2.44654	YES	YES
49	a	168.51	4.58038	YES	YES
50	a	175.56	1.17096	YES	YES
51	a	181.00	0.10150	YES	YES
52	a	182.29	0.61744	YES	YES
53	a	185.95	0.47589	YES	YES
54	a	199.20	0.50284	YES	YES
55	a	202.40	5.45543	YES	YES
56	a	206.06	0.02334	YES	YES
57	a	210.22	3.51884	YES	YES
58	a	214.91	0.36401	YES	YES
59	a	217.39	1.78771	YES	YES
60	a	221.79	1.73905	YES	YES
61	a	222.69	0.92152	YES	YES
62	a	226.41	0.13651	YES	YES
63	a	228.01	0.52029	YES	YES
64	a	234.29	0.03381	YES	YES
65	a	234.95	1.04171	YES	YES
66	a	239.68	0.09980	YES	YES
67	a	240.53	6.25896	YES	YES
68	a	242.09	1.77564	YES	YES
69	a	243.45	0.53179	YES	YES
70	a	254.56	3.16121	YES	YES
71	a	260.40	0.24754	YES	YES
72	a	271.41	0.50272	YES	YES
73	a	272.67	2.09504	YES	YES
74	a	275.91	5.57581	YES	YES
75	a	277.80	0.06929	YES	YES

76	a	283.26	2.90059	YES	YES
77	a	290.61	0.21152	YES	YES
78	a	303.05	0.07579	YES	YES
79	a	307.19	0.43162	YES	YES
80	a	313.46	0.22451	YES	YES
81	a	316.13	2.12685	YES	YES
82	a	323.15	2.09170	YES	YES
83	a	330.80	0.14962	YES	YES
84	a	334.84	0.83898	YES	YES
85	a	335.62	0.38422	YES	YES
86	a	336.43	0.25084	YES	YES
87	a	342.11	0.61150	YES	YES
88	a	346.17	0.13906	YES	YES
89	a	369.36	2.57962	YES	YES
90	a	371.05	2.70785	YES	YES
91	a	374.45	2.25470	YES	YES
92	a	375.78	1.05462	YES	YES
93	a	393.14	0.28894	YES	YES
94	a	396.99	0.29712	YES	YES
95	a	413.82	1.13694	YES	YES
96	a	414.60	1.30141	YES	YES
97	a	421.47	0.70067	YES	YES
98	a	423.54	1.62683	YES	YES
99	a	423.68	3.68755	YES	YES
100	a	426.31	0.69102	YES	YES
101	a	428.93	0.31613	YES	YES
102	a	430.55	0.14877	YES	YES
103	a	430.86	0.12847	YES	YES
104	a	432.17	1.19864	YES	YES
105	a	433.00	0.19526	YES	YES
106	a	433.59	0.41133	YES	YES
107	a	434.67	0.22810	YES	YES
108	a	435.48	0.85255	YES	YES
109	a	445.09	7.21658	YES	YES
110	a	447.99	1.47838	YES	YES
111	a	466.65	4.39764	YES	YES
112	a	476.56	2.54807	YES	YES
113	a	491.08	7.26187	YES	YES
114	a	491.26	6.17795	YES	YES
115	a	492.56	2.46293	YES	YES
116	a	495.49	1.76780	YES	YES
117	a	499.30	8.95046	YES	YES
118	a	507.07	0.30462	YES	YES
119	a	516.35	11.06291	YES	YES
120	a	519.79	5.08802	YES	YES
121	a	523.75	16.16126	YES	YES
122	a	534.59	70.83148	YES	YES
123	a	599.54	108.06236	YES	YES
124	a	626.06	14.35235	YES	YES
125	a	631.77	26.62702	YES	YES
126	a	642.11	6.81501	YES	YES
127	a	645.32	7.27117	YES	YES
128	a	715.17	0.97844	YES	YES
129	a	716.02	0.32447	YES	YES
130	a	722.98	2.38875	YES	YES
131	a	723.25	0.13442	YES	YES
132	a	732.28	3.88375	YES	YES
133	a	733.43	3.51709	YES	YES

134	a	734.59	13.50630	YES	YES
135	a	735.97	1.23497	YES	YES
136	a	771.38	0.15399	YES	YES
137	a	771.66	0.25597	YES	YES
138	a	772.23	0.45823	YES	YES
139	a	772.41	1.46351	YES	YES
140	a	774.09	0.06022	YES	YES
141	a	774.49	0.17274	YES	YES
142	a	777.71	2.86026	YES	YES
143	a	777.84	0.03996	YES	YES
144	a	781.79	19.98085	YES	YES
145	a	782.60	4.48159	YES	YES
146	a	807.26	1.15069	YES	YES
147	a	808.69	1.44040	YES	YES
148	a	809.97	2.03688	YES	YES
149	a	811.89	0.13418	YES	YES
150	a	812.19	1.36628	YES	YES
151	a	814.56	2.05216	YES	YES
152	a	815.28	0.28068	YES	YES
153	a	815.59	11.12728	YES	YES
154	a	833.66	0.31722	YES	YES
155	a	834.10	0.28254	YES	YES
156	a	836.59	1.59777	YES	YES
157	a	837.04	9.82254	YES	YES
158	a	838.33	5.94544	YES	YES
159	a	838.75	6.85074	YES	YES
160	a	838.92	9.61898	YES	YES
161	a	840.16	3.82269	YES	YES
162	a	853.45	14.64241	YES	YES
163	a	855.03	3.83994	YES	YES
164	a	873.54	2.58489	YES	YES
165	a	874.24	3.04388	YES	YES
166	a	875.36	5.09348	YES	YES
167	a	876.18	3.63035	YES	YES
168	a	877.28	1.97577	YES	YES
169	a	877.80	6.19398	YES	YES
170	a	878.24	3.91686	YES	YES
171	a	878.66	2.82927	YES	YES
172	a	879.45	1.58696	YES	YES
173	a	879.91	1.65013	YES	YES
174	a	880.24	3.15518	YES	YES
175	a	880.98	2.97577	YES	YES
176	a	883.59	0.52533	YES	YES
177	a	884.12	0.69644	YES	YES
178	a	885.84	4.44990	YES	YES
179	a	887.63	1.18153	YES	YES
180	a	902.77	2.00673	YES	YES
181	a	903.41	1.30349	YES	YES
182	a	905.27	1.31850	YES	YES
183	a	906.29	3.42871	YES	YES
184	a	908.01	2.86638	YES	YES
185	a	909.24	1.05273	YES	YES
186	a	910.41	0.33540	YES	YES
187	a	911.16	1.82617	YES	YES
188	a	968.43	71.11219	YES	YES
189	a	986.23	7.07844	YES	YES
190	a	987.68	2.78156	YES	YES
191	a	987.85	13.76513	YES	YES

192	a	988.38	1.41387	YES	YES
193	a	988.49	12.20099	YES	YES
194	a	989.91	20.85252	YES	YES
195	a	991.11	21.40667	YES	YES
196	a	991.85	8.22820	YES	YES
197	a	997.46	0.07127	YES	YES
198	a	1005.69	0.19640	YES	YES
199	a	1013.83	0.91386	YES	YES
200	a	1017.12	1.31348	YES	YES
201	a	1018.42	0.21789	YES	YES
202	a	1019.12	0.51377	YES	YES
203	a	1019.43	0.18106	YES	YES
204	a	1019.85	1.29709	YES	YES
205	a	1020.74	0.85686	YES	YES
206	a	1021.30	0.66064	YES	YES
207	a	1029.20	2.19583	YES	YES
208	a	1031.69	2.91503	YES	YES
209	a	1033.07	1.05127	YES	YES
210	a	1033.76	1.80354	YES	YES
211	a	1039.44	0.08339	YES	YES
212	a	1039.81	0.59203	YES	YES
213	a	1040.69	4.81085	YES	YES
214	a	1041.20	0.61393	YES	YES
215	a	1054.08	2.18155	YES	YES
216	a	1058.64	1.61929	YES	YES
217	a	1059.60	2.23694	YES	YES
218	a	1060.18	1.94953	YES	YES
219	a	1063.35	3.41503	YES	YES
220	a	1063.74	2.02589	YES	YES
221	a	1064.51	0.45457	YES	YES
222	a	1067.22	0.16150	YES	YES
223	a	1068.99	0.39848	YES	YES
224	a	1069.83	0.20707	YES	YES
225	a	1071.26	0.08068	YES	YES
226	a	1072.71	0.24312	YES	YES
227	a	1073.07	0.04940	YES	YES
228	a	1074.66	0.30980	YES	YES
229	a	1075.49	0.04513	YES	YES
230	a	1076.23	0.06369	YES	YES
231	a	1076.54	0.33027	YES	YES
232	a	1079.86	0.23264	YES	YES
233	a	1085.68	2.14698	YES	YES
234	a	1088.29	3.06429	YES	YES
235	a	1093.64	3.87448	YES	YES
236	a	1096.23	2.06237	YES	YES
237	a	1098.06	8.77148	YES	YES
238	a	1099.41	6.82436	YES	YES
239	a	1101.07	0.65892	YES	YES
240	a	1102.25	0.77378	YES	YES
241	a	1106.63	4.58423	YES	YES
242	a	1107.95	9.77002	YES	YES
243	a	1112.11	0.61110	YES	YES
244	a	1133.31	3.90147	YES	YES
245	a	1137.73	1.81657	YES	YES
246	a	1158.44	4.41971	YES	YES
247	a	1160.16	3.41662	YES	YES
248	a	1161.89	7.07013	YES	YES
249	a	1162.48	1.75229	YES	YES

250	a	1169.64	3.70927	YES	YES
251	a	1170.87	15.47370	YES	YES
252	a	1171.04	1.35520	YES	YES
253	a	1173.35	12.24797	YES	YES
254	a	1178.87	15.18971	YES	YES
255	a	1181.21	8.82462	YES	YES
256	a	1182.60	8.24875	YES	YES
257	a	1182.97	20.39734	YES	YES
258	a	1187.68	0.38229	YES	YES
259	a	1188.44	41.15881	YES	YES
260	a	1190.03	1.86197	YES	YES
261	a	1190.81	6.87744	YES	YES
262	a	1194.74	1.77155	YES	YES
263	a	1242.78	1.55506	YES	YES
264	a	1243.41	11.10183	YES	YES
265	a	1243.72	1.36017	YES	YES
266	a	1244.69	0.36021	YES	YES
267	a	1245.94	1.63515	YES	YES
268	a	1246.54	1.18610	YES	YES
269	a	1247.06	0.62971	YES	YES
270	a	1247.94	0.55532	YES	YES
271	a	1248.21	3.70201	YES	YES
272	a	1250.49	0.30924	YES	YES
273	a	1250.62	0.54507	YES	YES
274	a	1254.48	2.91551	YES	YES
275	a	1255.51	0.79723	YES	YES
276	a	1256.68	6.98029	YES	YES
277	a	1257.60	8.89980	YES	YES
278	a	1257.89	1.32728	YES	YES
279	a	1258.49	2.76138	YES	YES
280	a	1258.68	4.18229	YES	YES
281	a	1259.21	2.61300	YES	YES
282	a	1259.82	6.49921	YES	YES
283	a	1260.35	5.33025	YES	YES
284	a	1261.36	5.08507	YES	YES
285	a	1262.57	1.13154	YES	YES
286	a	1262.84	0.01842	YES	YES
287	a	1263.81	3.60641	YES	YES
288	a	1264.80	1.99050	YES	YES
289	a	1268.10	2.26076	YES	YES
290	a	1273.04	2.42807	YES	YES
291	a	1274.39	0.60734	YES	YES
292	a	1282.56	2.28850	YES	YES
293	a	1283.77	2.89380	YES	YES
294	a	1285.07	1.16176	YES	YES
295	a	1286.73	3.18104	YES	YES
296	a	1289.99	3.21139	YES	YES
297	a	1290.62	1.64298	YES	YES
298	a	1290.76	6.02242	YES	YES
299	a	1296.33	5.99330	YES	YES
300	a	1305.87	2.83067	YES	YES
301	a	1310.04	2.14450	YES	YES
302	a	1312.24	0.68413	YES	YES
303	a	1312.61	1.12877	YES	YES
304	a	1313.22	0.21488	YES	YES
305	a	1316.11	1.85283	YES	YES
306	a	1316.66	3.90826	YES	YES
307	a	1317.77	1.44573	YES	YES

308	a	1321.94	0.67387	YES	YES
309	a	1322.54	1.96722	YES	YES
310	a	1322.93	0.67818	YES	YES
311	a	1323.28	1.25949	YES	YES
312	a	1323.34	1.29076	YES	YES
313	a	1323.45	2.09828	YES	YES
314	a	1324.30	0.37889	YES	YES
315	a	1325.25	1.42673	YES	YES
316	a	1325.60	0.56952	YES	YES
317	a	1325.75	0.61149	YES	YES
318	a	1326.37	1.98190	YES	YES
319	a	1327.35	1.87706	YES	YES
320	a	1327.69	5.37184	YES	YES
321	a	1328.35	0.20042	YES	YES
322	a	1329.01	5.73254	YES	YES
323	a	1329.53	4.07948	YES	YES
324	a	1335.97	0.05909	YES	YES
325	a	1336.64	1.30195	YES	YES
326	a	1337.49	0.41062	YES	YES
327	a	1338.50	0.23570	YES	YES
328	a	1338.86	0.12070	YES	YES
329	a	1338.99	0.62266	YES	YES
330	a	1339.28	0.42359	YES	YES
331	a	1340.55	0.39150	YES	YES
332	a	1345.59	1.34089	YES	YES
333	a	1346.70	4.69473	YES	YES
334	a	1347.53	1.36588	YES	YES
335	a	1348.49	0.41054	YES	YES
336	a	1348.77	0.42440	YES	YES
337	a	1348.87	0.92776	YES	YES
338	a	1349.90	0.77000	YES	YES
339	a	1352.33	1.82450	YES	YES
340	a	1397.92	0.64477	YES	YES
341	a	1403.17	7.07710	YES	YES
342	a	1404.57	6.29849	YES	YES
343	a	1407.96	8.85265	YES	YES
344	a	1408.94	9.06718	YES	YES
345	a	1415.38	2.98081	YES	YES
346	a	1418.86	3.33552	YES	YES
347	a	1422.50	6.60775	YES	YES
348	a	1424.40	6.75508	YES	YES
349	a	1426.72	2.23433	YES	YES
350	a	1429.37	9.85263	YES	YES
351	a	1430.41	2.39225	YES	YES
352	a	1432.38	2.53025	YES	YES
353	a	1433.19	7.60249	YES	YES
354	a	1434.86	2.87113	YES	YES
355	a	1435.30	0.88895	YES	YES
356	a	1436.46	5.42431	YES	YES
357	a	1436.90	3.45267	YES	YES
358	a	1437.00	0.34938	YES	YES
359	a	1437.33	4.01271	YES	YES
360	a	1437.58	4.23154	YES	YES
361	a	1437.94	1.38778	YES	YES
362	a	1438.34	13.33886	YES	YES
363	a	1438.72	7.03858	YES	YES
364	a	1439.55	14.19308	YES	YES
365	a	1440.03	3.82712	YES	YES

366	a	1440.22	1.88468	YES	YES
367	a	1440.71	2.31546	YES	YES
368	a	1441.23	3.07389	YES	YES
369	a	1441.53	3.76584	YES	YES
370	a	1441.68	5.58501	YES	YES
371	a	1443.15	4.90972	YES	YES
372	a	1443.25	20.46121	YES	YES
373	a	1443.71	18.06070	YES	YES
374	a	1444.54	24.51118	YES	YES
375	a	1445.03	0.71581	YES	YES
376	a	1446.17	4.42899	YES	YES
377	a	1446.45	9.20071	YES	YES
378	a	1446.86	5.12112	YES	YES
379	a	1447.16	21.57831	YES	YES
380	a	1452.88	1.96933	YES	YES
381	a	1453.29	4.90524	YES	YES
382	a	1454.18	0.71460	YES	YES
383	a	1455.13	2.93011	YES	YES
384	a	1456.55	0.56133	YES	YES
385	a	1457.26	0.70000	YES	YES
386	a	1458.21	0.44205	YES	YES
387	a	1458.48	0.77056	YES	YES
388	a	1465.66	14.92902	YES	YES
389	a	1477.44	3.86046	YES	YES
390	a	1854.82	139.34323	YES	YES
391	a	2856.32	99.90682	YES	YES
392	a	2867.81	92.35058	YES	YES
393	a	2893.07	57.58221	YES	YES
394	a	2926.70	16.65871	YES	YES
395	a	2930.28	11.17571	YES	YES
396	a	2932.15	23.17573	YES	YES
397	a	2933.20	24.45870	YES	YES
398	a	2934.46	12.43544	YES	YES
399	a	2937.27	2.78762	YES	YES
400	a	2938.74	4.54428	YES	YES
401	a	2940.26	12.40687	YES	YES
402	a	2941.16	11.37082	YES	YES
403	a	2941.45	1.04835	YES	YES
404	a	2942.83	11.05419	YES	YES
405	a	2942.98	28.41240	YES	YES
406	a	2944.33	10.72344	YES	YES
407	a	2944.39	2.12985	YES	YES
408	a	2945.28	7.96550	YES	YES
409	a	2946.58	16.02939	YES	YES
410	a	2947.48	9.96576	YES	YES
411	a	2947.59	4.45921	YES	YES
412	a	2948.91	20.15540	YES	YES
413	a	2948.96	6.21928	YES	YES
414	a	2949.72	11.68917	YES	YES
415	a	2949.98	6.96375	YES	YES
416	a	2950.82	19.85763	YES	YES
417	a	2953.79	13.68142	YES	YES
418	a	2954.49	12.98701	YES	YES
419	a	2955.40	9.82639	YES	YES
420	a	2955.43	3.72379	YES	YES
421	a	2956.35	1.28053	YES	YES
422	a	2956.83	10.29520	YES	YES
423	a	2957.44	9.94695	YES	YES

424	a	2958.09	3.37244	YES	YES
425	a	2958.29	11.29687	YES	YES
426	a	2958.74	3.75676	YES	YES
427	a	2959.62	33.20406	YES	YES
428	a	2960.03	18.24975	YES	YES
429	a	2960.18	27.36597	YES	YES
430	a	2960.40	51.28841	YES	YES
431	a	2961.03	6.04874	YES	YES
432	a	2962.55	27.53674	YES	YES
433	a	2962.67	26.64481	YES	YES
434	a	2963.69	43.90390	YES	YES
435	a	2965.29	15.27656	YES	YES
436	a	2965.84	14.17771	YES	YES
437	a	2966.62	40.31024	YES	YES
438	a	2968.58	14.22606	YES	YES
439	a	2969.62	24.90111	YES	YES
440	a	2969.66	28.82453	YES	YES
441	a	2972.81	1.98077	YES	YES
442	a	2974.89	13.20535	YES	YES
443	a	2984.49	5.23776	YES	YES
444	a	2986.59	8.91693	YES	YES
445	a	2987.51	0.23148	YES	YES
446	a	2988.50	9.36526	YES	YES
447	a	2989.55	17.93802	YES	YES
448	a	2989.96	3.51315	YES	YES
449	a	2993.07	22.85716	YES	YES
450	a	2996.40	28.27553	YES	YES
451	a	3003.64	21.47018	YES	YES
452	a	3004.03	29.46031	YES	YES
453	a	3005.26	15.65401	YES	YES
454	a	3005.65	19.15109	YES	YES
455	a	3005.80	35.45882	YES	YES
456	a	3006.86	42.06148	YES	YES
457	a	3007.54	24.33928	YES	YES
458	a	3008.23	0.65096	YES	YES
459	a	3008.49	11.82922	YES	YES
460	a	3009.54	30.15516	YES	YES
461	a	3009.87	0.41960	YES	YES
462	a	3010.21	30.22052	YES	YES
463	a	3011.83	4.94159	YES	YES
464	a	3012.16	23.36343	YES	YES
465	a	3012.26	31.05064	YES	YES
466	a	3012.41	5.30443	YES	YES
467	a	3012.62	0.82356	YES	YES
468	a	3012.89	23.04754	YES	YES
469	a	3013.80	30.22432	YES	YES
470	a	3014.15	13.31669	YES	YES
471	a	3014.20	19.27561	YES	YES
472	a	3014.49	29.02720	YES	YES
473	a	3014.54	35.47919	YES	YES
474	a	3014.95	32.13051	YES	YES
475	a	3015.12	32.83340	YES	YES
476	a	3015.43	40.83230	YES	YES
477	a	3016.79	21.58312	YES	YES
478	a	3017.03	21.52214	YES	YES
479	a	3017.65	19.67203	YES	YES
480	a	3018.04	30.31175	YES	YES
481	a	3018.54	36.00476	YES	YES

```
   482        a           3018.59        15.92670       YES     YES
   483        a           3018.85        46.16208       YES     YES
   484        a           3019.55        35.89690       YES     YES
   485        a           3019.74        23.47137       YES     YES
   486        a           3026.66         8.06505       YES     YES
   487        a           3029.43        12.33665       YES     YES
   488        a           3032.90         0.05092       YES     YES
   489        a           3034.22         0.27472       YES     YES
   490        a           3041.05        11.91545       YES     YES
   491        a           3047.07         0.36222       YES     YES
   492        a           3052.85         0.22714       YES     YES
$end
```

Double hybrid single point energy = -7384.069502233423 H
COSMO energy + OC correction = -7391.5848093672 H (in *o*DFB)

6.2.30 H_2NMe

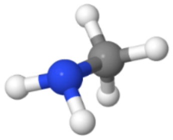

```
Method: (RI-)BP86(D3BJ)/def2-TZVPP
Symmetry: cs

Cartesian coordinates in Ångström:
N     0.8114119   -0.4324804    0.0000000
H     0.7951481   -1.0462683   -0.8154029
H     0.7951481   -1.0462683    0.8154029
H    -0.3368083    1.0919505   -0.8832561
C    -0.3701413    0.4398886    0.0000000
H    -0.3368083    1.0919505    0.8832561
H    -1.3475198   -0.0791260    0.0000000

SCF energy GEOOPT =    -95.908012557 H
ZPE =   163.2 kJ/mol
FREEH energy =   172.28 kJ/mol
FREEH entropy =   0.24131 kJ/mol/K

$vibrational spectrum
#  mode     symmetry     wave number   IR intensity    selection rules
#                         cm**(-1)        km/mol          IR      RAMAN
     1                       -0.00         0.00000        -        -
     2                       -0.00         0.00000        -        -
     3                       -0.00         0.00000        -        -
     4                        0.00         0.00000        -        -
     5                        0.00         0.00000        -        -
     6                        0.00         0.00000        -        -
     7        a"            294.96        34.07441       YES     YES
     8        a'            813.22       150.45328       YES     YES
     9        a"            947.78         0.11579       YES     YES
    10        a'           1032.70         7.78533       YES     YES
    11        a'           1135.50         5.17228       YES     YES
    12        a"           1308.43         0.09957       YES     YES
    13        a'           1409.23         1.81784       YES     YES
    14        a'           1448.62         6.85647       YES     YES
    15        a"           1470.62         3.74869       YES     YES
    16        a'           1614.08        18.01347       YES     YES
```

17	a'	2890.88	98.57229	YES	YES
18	a'	2991.90	33.70520	YES	YES
19	a"	3030.35	29.67839	YES	YES
20	a'	3408.05	1.18933	YES	YES
21	a"	3485.24	0.12228	YES	YES

$end

Double hybrid single point energy = -95.736887274557 H
COSMO energy + OC correction = -95.9143077467 H (in *o*DFB)

6.2.31 [{Ga(dcpe)}$_2$(H$_2$NMe)]$^{2+}$

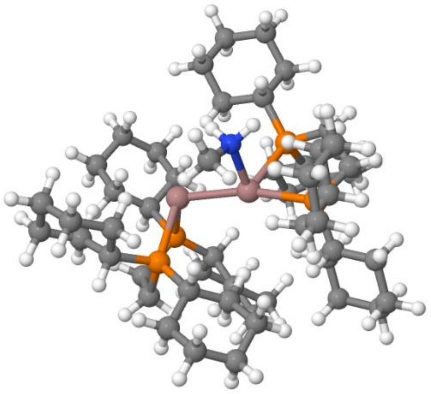

Method: (RI-)BP86(D3BJ)/def2-TZVPP
Symmetry: c1

Cartesian coordinates in Ångström:

Ga	-1.6948511	17.0992273	-2.1994605
Ga	-4.0025619	17.7956493	-1.3240355
P	-1.2894867	18.7293649	-4.0781792
P	-6.1204656	19.1686005	-0.8783539
P	-1.9911950	15.5513854	-4.1497530
P	-5.1966180	16.1925726	0.1608376
C	-4.6646780	21.1224957	-4.8222783
H	-4.7051063	21.6613720	-3.8604689
H	-5.6967421	20.8297341	-5.0668674
C	-1.0448804	14.0205184	-3.7088526
H	-0.9426572	13.4118495	-4.6221492
C	-4.1794163	15.3205405	1.4341067
H	-3.9115399	16.1490099	2.1195776
C	-4.3692830	16.1278446	-5.5094255
H	-3.7869396	16.5596516	-6.3392183
H	-4.4999762	16.9251682	-4.7615829
C	-7.0260494	19.3617623	-2.4772514
H	-6.3636489	20.0086867	-3.0757972
C	-7.2208321	18.1117594	0.1875387
H	-7.8866040	18.7637326	0.7693564
H	-7.8619441	17.5234497	-0.4828954
C	-2.6542707	22.4290778	-5.6033164
H	-2.2484688	23.0668012	-6.4002296
H	-2.6080026	23.0217682	-4.6744708
C	-1.7736196	21.1820217	-5.4451191
H	-0.7467041	21.4841156	-5.2013577
H	-1.7285923	20.6445345	-6.4063494
C	-3.7968291	19.8696216	-4.6584598

H	-3.8491392	19.2806883	-5.5881421
H	-4.1816633	19.2207189	-3.8550042
C	1.0974477	13.1023135	-2.7024851
H	1.2812829	12.4380944	-3.5625976
H	2.0837007	13.3847040	-2.3094131
C	-7.4350667	14.4158147	-0.2603095
H	-8.0904233	15.1842648	0.1737494
H	-7.1362438	13.7523770	0.5638774
C	-6.1840622	20.8402015	-0.0771873
H	-7.2605468	21.0800892	-0.0274264
C	0.5585916	20.2185782	-2.6139596
H	-0.1407930	21.0669077	-2.6559996
H	0.2702922	19.5979269	-1.7470056
C	1.9920714	20.7231687	-2.4079661
H	2.2580297	21.4032719	-3.2334294
H	2.0464110	21.3164932	-1.4845738
C	-7.1440777	18.0159058	-3.2114997
H	-6.1481494	17.5540810	-3.3218828
H	-7.7557193	17.3206273	-2.6122664
C	-4.1090567	22.0580894	-5.8997179
H	-4.1672049	21.5572360	-6.8800588
H	-4.7269583	22.9631232	-5.9750441
C	-8.3950311	20.0502253	-2.3471499
H	-8.2935577	21.0306667	-1.8629348
H	-9.0529401	19.4423536	-1.7043537
C	-5.4934132	21.9039376	-0.9445681
H	-5.9475996	21.9332098	-1.9446530
H	-4.4359051	21.6269273	-1.0933445
C	-3.4113576	13.8114983	-5.9053698
H	-2.8968862	12.9600004	-5.4408352
H	-2.7763140	14.1493293	-6.7405381
C	2.8789809	18.6951157	-3.6210072
H	3.1905328	19.2856283	-4.4979751
H	3.5646375	17.8386292	-3.5621105
C	-5.7573327	22.2284658	1.9936423
H	-6.8212962	22.4971786	2.0910820
H	-5.3459502	22.2061546	3.0119559
C	-9.1693741	18.8870147	-4.4602555
H	-9.6064437	19.0378966	-5.4563318
H	-9.8643318	18.2296049	-3.9117687
C	-4.9230312	14.2573152	2.2619959
H	-5.8518397	14.6648648	2.6851359
H	-5.2091118	13.4256395	1.5998366
C	0.2891873	12.3499270	-1.6420151
H	0.8233115	11.4440768	-1.3256210
H	0.1860572	12.9851801	-0.7454901
C	-7.8091268	18.1947464	-4.5815820
H	-7.1443787	18.7928038	-5.2265175
H	-7.9198129	17.2162310	-5.0655782
C	2.9859972	19.5592498	-2.3616857
H	2.7792333	18.9383610	-1.4737185
H	4.0104040	19.9377888	-2.2470039
C	-5.0386507	23.2781233	1.1407394
H	-5.1461888	24.2722373	1.5939837
H	-3.9569873	23.0598631	1.1260052
C	-5.2871537	13.9877541	-1.5455662
H	-4.9175596	13.2991784	-0.7708219
H	-4.4010829	14.4653760	-1.9898100

C	-5.6478612	20.8335555	1.3630020
H	-4.5864234	20.5378888	1.3606444
H	-6.1903003	20.0983650	1.9755218
C	-6.1884484	15.0497181	-0.8967241
H	-6.5298417	15.7175488	-1.7095393
C	-5.5781073	23.2901869	-0.2924067
C	-7.3233510	12.5558412	-1.9854033
H	-7.8909911	12.0203806	-2.7580122
H	-7.0245767	11.8017545	-1.2391255
C	-8.2090575	13.6111409	-1.3147728
H	-9.0838951	13.1391066	-0.8479973
H	-8.5978711	14.3032060	-2.0808709
C	-1.8509819	13.2341442	-2.6601537
H	-2.8314981	12.9458696	-3.0656221
H	-2.0426522	13.9026294	-1.8028122
C	-4.0171027	13.7240818	3.3812478
H	-3.8083280	14.5385378	4.0944685
H	-4.5511805	12.9468182	3.9442910
C	-5.7205776	15.6600499	-6.0602596
H	-6.2424167	16.5076065	-6.5246699
H	-6.3525467	15.3191054	-5.2224541
C	-2.6978478	13.1765191	2.8270386
H	-2.9041326	12.2885287	2.2067134
H	-2.0536300	12.8379552	3.6492589
C	-2.8693460	14.7537290	0.8571883
H	-3.1056402	13.9296981	0.1684371
H	-2.3407742	15.5115305	0.2542883
C	-9.0486429	20.2231163	-3.7248127
H	-10.0351775	20.6910255	-3.6067195
H	-8.4408615	20.9206881	-4.3244148
C	-4.7687058	13.3548176	-6.4590120
H	-4.6121495	12.5630323	-7.2040610
H	-5.3559778	12.9045457	-5.6423833
C	-6.0683639	13.1898204	-2.5934626
H	-5.4201661	12.4187584	-3.0343730
H	-6.3605838	13.8646946	-3.4154376
C	-5.5515448	14.5199451	-7.0682048
H	-6.5327653	14.1765229	-7.4228511
H	-5.0150529	14.8974058	-7.9543522
C	-1.1011587	11.9903883	-2.1710967
H	-1.6944337	11.4848785	-1.3955668
H	-1.0031890	11.2755567	-3.0040206
C	-1.9697447	14.2248379	1.9790498
H	-1.6554808	15.0649121	2.6212222
H	-1.0520598	13.8032885	1.5457156
H	-2.3213871	17.8595793	0.6741574
C	0.4423931	19.3548349	-3.8796708
H	0.6647725	19.9762988	-4.7629469
C	1.4471744	18.1900931	-3.8403920
H	1.4010174	17.6134201	-4.7749194
H	1.1676297	17.5033505	-3.0218647
C	0.3597370	14.3593398	-3.1792259
H	0.9536726	14.8691221	-3.9506762
H	0.2619556	15.0661507	-2.3359893
C	-1.0423349	16.3163606	-5.5468501
H	0.0159589	16.1147215	-5.3407902
H	-1.2877543	15.7912258	-6.4805073
C	-1.2755799	17.8261868	-5.6988925

```
H    -2.2509222   18.0148813   -6.1643679
H    -0.5138942   18.2667493   -6.3580072
C    -3.5861731   14.9578169   -4.8956490
H    -4.1644984   14.5810478   -4.0373804
C    -2.3350325   20.2418700   -4.3644656
H    -2.3113450   20.7681281   -3.3932939
C    -6.4290916   17.1806663    1.1140334
H    -7.1064535   16.5183485    1.6707781
H    -5.8657168   17.7586837    1.8614412
H    -6.6306114   23.6162529   -0.2831576
H    -5.0324429   24.0190488   -0.9070450
C    -2.2042563   19.8430475    0.1433139
N    -2.9777945   18.6172381    0.4560714
H    -1.5641163   19.6293650   -0.7204928
H    -1.5770079   20.1649883    0.9836730
H    -2.8959170   20.6490446   -0.1207840
H    -3.5611167   18.7679738    1.2829516

SCF energy GEOOPT =  -7352.105335593 H
ZPE =   3767. kJ/mol
FREEH energy =  3954.72 kJ/mol
FREEH entropy =   1.59936 kJ/mol/K

$vibrational spectrum
#  mode     symmetry     wave number   IR intensity    selection rules
#                         cm**(-1)        km/mol          IR     RAMAN
     1                      -0.00         0.00000         -        -
     2                      -0.00         0.00000         -        -
     3                       0.00         0.00000         -        -
     4                       0.00         0.00000         -        -
     5                       0.00         0.00000         -        -
     6                       0.00         0.00000         -        -
     7         a            16.23         0.22135        YES      YES
     8         a            20.88         0.06237        YES      YES
     9         a            25.26         0.09180        YES      YES
    10         a            26.56         0.07986        YES      YES
    11         a            28.02         0.20497        YES      YES
    12         a            30.78         0.10174        YES      YES
    13         a            32.94         0.29996        YES      YES
    14         a            36.07         0.04715        YES      YES
    15         a            38.19         0.25691        YES      YES
    16         a            41.75         0.31886        YES      YES
    17         a            43.14         0.06384        YES      YES
    18         a            44.99         0.05934        YES      YES
    19         a            47.36         0.01611        YES      YES
    20         a            52.51         0.03330        YES      YES
    21         a            54.14         0.64127        YES      YES
    22         a            54.90         0.01028        YES      YES
    23         a            59.79         0.12703        YES      YES
    24         a            64.57         0.28157        YES      YES
    25         a            66.40         0.16393        YES      YES
    26         a            67.03         0.23916        YES      YES
    27         a            67.22         0.11051        YES      YES
    28         a            69.34         0.21062        YES      YES
    29         a            72.82         0.39113        YES      YES
    30         a            75.82         0.68630        YES      YES
    31         a            77.13         0.01907        YES      YES
    32         a            78.15         0.08544        YES      YES
```

33	a	85.39	0.19111	YES	YES
34	a	88.25	0.22431	YES	YES
35	a	95.90	0.13422	YES	YES
36	a	98.48	0.14151	YES	YES
37	a	106.23	0.06411	YES	YES
38	a	111.75	0.77487	YES	YES
39	a	122.52	0.39648	YES	YES
40	a	125.19	5.30601	YES	YES
41	a	126.85	1.04697	YES	YES
42	a	130.47	0.72721	YES	YES
43	a	134.63	0.67678	YES	YES
44	a	134.92	1.67292	YES	YES
45	a	138.53	1.94778	YES	YES
46	a	142.21	0.86226	YES	YES
47	a	145.78	0.37465	YES	YES
48	a	153.21	4.64600	YES	YES
49	a	171.25	0.40020	YES	YES
50	a	174.54	4.32645	YES	YES
51	a	176.68	0.17007	YES	YES
52	a	181.31	0.81268	YES	YES
53	a	188.58	0.39448	YES	YES
54	a	197.53	0.67351	YES	YES
55	a	200.85	1.58376	YES	YES
56	a	204.10	1.06001	YES	YES
57	a	212.30	0.41320	YES	YES
58	a	214.67	0.04285	YES	YES
59	a	215.18	0.08903	YES	YES
60	a	219.63	0.16622	YES	YES
61	a	220.44	0.17440	YES	YES
62	a	224.91	0.21053	YES	YES
63	a	225.90	0.06576	YES	YES
64	a	231.16	0.42753	YES	YES
65	a	233.34	1.35299	YES	YES
66	a	236.01	0.44157	YES	YES
67	a	236.94	0.02441	YES	YES
68	a	237.97	0.84043	YES	YES
69	a	240.06	2.34918	YES	YES
70	a	241.71	0.32926	YES	YES
71	a	251.61	2.30554	YES	YES
72	a	263.27	1.10406	YES	YES
73	a	277.58	1.74147	YES	YES
74	a	278.54	0.82877	YES	YES
75	a	284.97	0.38534	YES	YES
76	a	294.26	0.27435	YES	YES
77	a	302.29	0.51405	YES	YES
78	a	306.68	9.01597	YES	YES
79	a	311.43	0.72706	YES	YES
80	a	313.05	1.08363	YES	YES
81	a	329.54	0.97210	YES	YES
82	a	329.70	1.55374	YES	YES
83	a	330.85	0.11431	YES	YES
84	a	334.08	1.04019	YES	YES
85	a	334.95	0.80464	YES	YES
86	a	337.23	1.52812	YES	YES
87	a	364.40	9.58716	YES	YES
88	a	373.48	2.83747	YES	YES
89	a	379.18	1.97091	YES	YES
90	a	381.84	0.46839	YES	YES

91	a	388.17	2.95600	YES	YES
92	a	390.55	0.83345	YES	YES
93	a	408.23	0.48233	YES	YES
94	a	412.45	0.65741	YES	YES
95	a	418.12	3.03278	YES	YES
96	a	421.34	6.45582	YES	YES
97	a	422.93	2.38630	YES	YES
98	a	429.17	3.17502	YES	YES
99	a	430.12	0.05573	YES	YES
100	a	431.12	0.22648	YES	YES
101	a	431.45	0.22193	YES	YES
102	a	431.84	0.06423	YES	YES
103	a	432.58	0.42691	YES	YES
104	a	433.02	0.51144	YES	YES
105	a	434.50	1.33983	YES	YES
106	a	437.20	1.06762	YES	YES
107	a	440.67	3.89113	YES	YES
108	a	455.81	7.81970	YES	YES
109	a	460.09	4.37784	YES	YES
110	a	472.04	2.97626	YES	YES
111	a	490.78	4.62915	YES	YES
112	a	492.73	3.50336	YES	YES
113	a	494.20	6.71412	YES	YES
114	a	497.95	0.95050	YES	YES
115	a	500.73	1.48870	YES	YES
116	a	501.32	0.44628	YES	YES
117	a	509.20	7.61708	YES	YES
118	a	511.24	11.81022	YES	YES
119	a	543.35	60.35823	YES	YES
120	a	625.32	8.56712	YES	YES
121	a	626.74	23.32068	YES	YES
122	a	640.12	8.87224	YES	YES
123	a	661.92	14.16050	YES	YES
124	a	699.41	1.93120	YES	YES
125	a	712.22	1.41991	YES	YES
126	a	714.61	5.46094	YES	YES
127	a	726.83	9.03296	YES	YES
128	a	729.65	2.51270	YES	YES
129	a	730.63	2.14083	YES	YES
130	a	731.27	8.77528	YES	YES
131	a	732.41	8.39678	YES	YES
132	a	770.98	5.52112	YES	YES
133	a	771.83	13.22419	YES	YES
134	a	772.71	0.53778	YES	YES
135	a	773.92	2.02551	YES	YES
136	a	774.51	1.54069	YES	YES
137	a	775.46	0.42907	YES	YES
138	a	776.15	0.79582	YES	YES
139	a	777.21	0.31884	YES	YES
140	a	777.25	0.13001	YES	YES
141	a	783.71	17.82291	YES	YES
142	a	805.74	0.33856	YES	YES
143	a	808.30	0.72540	YES	YES
144	a	809.58	6.98341	YES	YES
145	a	810.59	6.31218	YES	YES
146	a	811.16	3.65436	YES	YES
147	a	812.41	2.88106	YES	YES
148	a	814.73	2.71554	YES	YES

149	a	816.72	3.08406	YES	YES
150	a	831.72	1.53955	YES	YES
151	a	832.04	7.04219	YES	YES
152	a	835.39	10.05322	YES	YES
153	a	837.43	1.90226	YES	YES
154	a	838.06	1.38050	YES	YES
155	a	838.47	0.88715	YES	YES
156	a	839.49	6.83283	YES	YES
157	a	840.07	3.88796	YES	YES
158	a	847.09	20.66483	YES	YES
159	a	851.42	17.13545	YES	YES
160	a	873.94	1.25220	YES	YES
161	a	875.30	0.51309	YES	YES
162	a	875.89	0.48227	YES	YES
163	a	876.05	2.92532	YES	YES
164	a	876.41	6.90804	YES	YES
165	a	877.15	1.66401	YES	YES
166	a	877.74	4.30663	YES	YES
167	a	878.27	4.16149	YES	YES
168	a	878.64	4.47533	YES	YES
169	a	879.23	0.86597	YES	YES
170	a	880.69	1.81098	YES	YES
171	a	882.18	2.97876	YES	YES
172	a	882.95	3.38914	YES	YES
173	a	884.20	2.76932	YES	YES
174	a	884.64	0.77332	YES	YES
175	a	886.08	6.15371	YES	YES
176	a	902.73	1.03771	YES	YES
177	a	903.10	2.00311	YES	YES
178	a	904.15	5.83441	YES	YES
179	a	904.87	0.82481	YES	YES
180	a	908.38	0.70121	YES	YES
181	a	909.60	1.08832	YES	YES
182	a	910.32	0.21906	YES	YES
183	a	910.63	1.17040	YES	YES
184	a	962.25	44.98869	YES	YES
185	a	980.98	34.50761	YES	YES
186	a	984.59	2.08445	YES	YES
187	a	985.96	1.41184	YES	YES
188	a	986.69	10.10111	YES	YES
189	a	987.22	3.25057	YES	YES
190	a	988.89	11.56024	YES	YES
191	a	989.49	17.38269	YES	YES
192	a	990.47	16.70270	YES	YES
193	a	991.22	4.69364	YES	YES
194	a	991.89	1.88321	YES	YES
195	a	992.50	7.27720	YES	YES
196	a	1008.99	4.05306	YES	YES
197	a	1013.56	0.91062	YES	YES
198	a	1015.22	1.42053	YES	YES
199	a	1016.47	1.32334	YES	YES
200	a	1017.25	0.58720	YES	YES
201	a	1017.68	0.43951	YES	YES
202	a	1018.80	0.15474	YES	YES
203	a	1020.15	0.25378	YES	YES
204	a	1022.54	0.46810	YES	YES
205	a	1030.78	1.21245	YES	YES
206	a	1031.84	0.66412	YES	YES

207	a	1032.03	2.71026	YES	YES
208	a	1034.77	0.79764	YES	YES
209	a	1038.39	1.10503	YES	YES
210	a	1038.57	2.66122	YES	YES
211	a	1039.32	1.08969	YES	YES
212	a	1042.04	1.20486	YES	YES
213	a	1054.99	1.63342	YES	YES
214	a	1056.27	1.55296	YES	YES
215	a	1056.87	0.78581	YES	YES
216	a	1058.03	2.48468	YES	YES
217	a	1059.41	1.43886	YES	YES
218	a	1062.35	1.02771	YES	YES
219	a	1063.92	0.68057	YES	YES
220	a	1066.25	2.56257	YES	YES
221	a	1069.05	0.76641	YES	YES
222	a	1070.22	1.04559	YES	YES
223	a	1072.33	0.49369	YES	YES
224	a	1072.76	0.08231	YES	YES
225	a	1073.47	1.05153	YES	YES
226	a	1073.61	0.69529	YES	YES
227	a	1073.83	0.84542	YES	YES
228	a	1074.70	0.07805	YES	YES
229	a	1075.09	0.60975	YES	YES
230	a	1077.71	3.43423	YES	YES
231	a	1088.46	5.54599	YES	YES
232	a	1089.68	6.00696	YES	YES
233	a	1091.59	6.61926	YES	YES
234	a	1092.67	9.46958	YES	YES
235	a	1098.28	2.31402	YES	YES
236	a	1102.85	3.36042	YES	YES
237	a	1104.20	6.11624	YES	YES
238	a	1113.98	9.83582	YES	YES
239	a	1121.83	4.65805	YES	YES
240	a	1126.95	2.65990	YES	YES
241	a	1157.85	5.61434	YES	YES
242	a	1159.43	3.10082	YES	YES
243	a	1159.68	12.77337	YES	YES
244	a	1165.59	9.64513	YES	YES
245	a	1167.84	17.55711	YES	YES
246	a	1168.41	2.75284	YES	YES
247	a	1169.49	2.32317	YES	YES
248	a	1169.65	14.46692	YES	YES
249	a	1173.82	5.65978	YES	YES
250	a	1177.64	4.73743	YES	YES
251	a	1178.39	20.26052	YES	YES
252	a	1179.35	12.63541	YES	YES
253	a	1182.88	8.22331	YES	YES
254	a	1183.32	5.27672	YES	YES
255	a	1185.80	0.29789	YES	YES
256	a	1192.17	1.11106	YES	YES
257	a	1210.36	1.48895	YES	YES
258	a	1234.56	1.15279	YES	YES
259	a	1235.32	0.61611	YES	YES
260	a	1242.34	0.57534	YES	YES
261	a	1245.40	0.35214	YES	YES
262	a	1245.46	0.58586	YES	YES
263	a	1245.74	0.59419	YES	YES
264	a	1246.25	1.92283	YES	YES

265	a	1247.91	0.36432	YES	YES
266	a	1249.07	1.21413	YES	YES
267	a	1250.45	1.27478	YES	YES
268	a	1250.84	1.83716	YES	YES
269	a	1253.29	0.92893	YES	YES
270	a	1253.91	0.90815	YES	YES
271	a	1255.20	2.70260	YES	YES
272	a	1255.48	1.47876	YES	YES
273	a	1256.38	1.10363	YES	YES
274	a	1256.45	4.34157	YES	YES
275	a	1256.81	2.21868	YES	YES
276	a	1257.53	8.84767	YES	YES
277	a	1258.02	4.25849	YES	YES
278	a	1258.23	4.63080	YES	YES
279	a	1259.93	3.53067	YES	YES
280	a	1260.83	1.30522	YES	YES
281	a	1262.26	0.98891	YES	YES
282	a	1263.03	1.34074	YES	YES
283	a	1263.94	1.74757	YES	YES
284	a	1267.68	3.08338	YES	YES
285	a	1269.32	1.68933	YES	YES
286	a	1281.02	2.91001	YES	YES
287	a	1281.94	0.78480	YES	YES
288	a	1282.39	1.51933	YES	YES
289	a	1283.84	2.29873	YES	YES
290	a	1288.30	2.92643	YES	YES
291	a	1289.97	2.64613	YES	YES
292	a	1290.26	4.97088	YES	YES
293	a	1291.52	5.23339	YES	YES
294	a	1302.26	1.00534	YES	YES
295	a	1304.55	0.54009	YES	YES
296	a	1306.85	1.11876	YES	YES
297	a	1308.97	0.83993	YES	YES
298	a	1309.64	3.48028	YES	YES
299	a	1310.66	0.66958	YES	YES
300	a	1311.55	3.24953	YES	YES
301	a	1313.48	0.36143	YES	YES
302	a	1315.93	2.00209	YES	YES
303	a	1318.33	2.54954	YES	YES
304	a	1318.69	0.83785	YES	YES
305	a	1320.01	0.51781	YES	YES
306	a	1321.29	0.93337	YES	YES
307	a	1321.92	6.30496	YES	YES
308	a	1322.29	0.87368	YES	YES
309	a	1323.05	2.40399	YES	YES
310	a	1323.38	1.74975	YES	YES
311	a	1323.73	0.64890	YES	YES
312	a	1324.30	0.49382	YES	YES
313	a	1324.64	3.58500	YES	YES
314	a	1325.07	2.38454	YES	YES
315	a	1325.23	1.17778	YES	YES
316	a	1326.45	3.87035	YES	YES
317	a	1326.99	9.45711	YES	YES
318	a	1330.22	3.48892	YES	YES
319	a	1335.44	0.23100	YES	YES
320	a	1335.88	0.65997	YES	YES
321	a	1336.80	0.08528	YES	YES
322	a	1336.94	0.07916	YES	YES

323	a	1337.65	0.21335	YES	YES
324	a	1338.09	0.18982	YES	YES
325	a	1338.21	0.07739	YES	YES
326	a	1338.58	0.11328	YES	YES
327	a	1344.45	0.39732	YES	YES
328	a	1344.96	1.72762	YES	YES
329	a	1345.96	0.09664	YES	YES
330	a	1346.08	2.53739	YES	YES
331	a	1347.09	1.97982	YES	YES
332	a	1347.68	2.36176	YES	YES
333	a	1348.63	0.86353	YES	YES
334	a	1349.33	1.39427	YES	YES
335	a	1397.76	3.82370	YES	YES
336	a	1400.76	4.67439	YES	YES
337	a	1406.12	6.33701	YES	YES
338	a	1408.52	11.66183	YES	YES
339	a	1410.89	6.35539	YES	YES
340	a	1425.05	0.86596	YES	YES
341	a	1426.74	5.18918	YES	YES
342	a	1428.46	3.00972	YES	YES
343	a	1429.33	4.48892	YES	YES
344	a	1431.16	0.41443	YES	YES
345	a	1432.80	2.79017	YES	YES
346	a	1433.23	3.08009	YES	YES
347	a	1433.73	3.83739	YES	YES
348	a	1433.99	0.47055	YES	YES
349	a	1435.26	0.92522	YES	YES
350	a	1435.53	14.68979	YES	YES
351	a	1435.89	8.91379	YES	YES
352	a	1436.31	4.91830	YES	YES
353	a	1437.13	9.78807	YES	YES
354	a	1437.74	8.48982	YES	YES
355	a	1437.85	3.56906	YES	YES
356	a	1438.09	8.02981	YES	YES
357	a	1438.57	2.72392	YES	YES
358	a	1439.20	0.51910	YES	YES
359	a	1439.33	1.16714	YES	YES
360	a	1439.70	2.25090	YES	YES
361	a	1440.11	4.98978	YES	YES
362	a	1440.39	0.54143	YES	YES
363	a	1440.89	11.29145	YES	YES
364	a	1441.94	11.54227	YES	YES
365	a	1442.75	7.30038	YES	YES
366	a	1443.05	9.77779	YES	YES
367	a	1444.40	10.56642	YES	YES
368	a	1444.72	4.96265	YES	YES
369	a	1445.11	30.21774	YES	YES
370	a	1445.54	15.17975	YES	YES
371	a	1446.58	1.74281	YES	YES
372	a	1447.86	7.69779	YES	YES
373	a	1452.77	0.56791	YES	YES
374	a	1454.03	1.95297	YES	YES
375	a	1454.12	2.86234	YES	YES
376	a	1455.13	1.95140	YES	YES
377	a	1455.57	0.13302	YES	YES
378	a	1455.83	2.50312	YES	YES
379	a	1456.94	0.07442	YES	YES
380	a	1457.87	2.13802	YES	YES

381	a	1463.24	7.07275	YES	YES
382	a	1560.21	62.19501	YES	YES
383	a	2890.68	8.71050	YES	YES
384	a	2917.05	18.95698	YES	YES
385	a	2919.45	1.20191	YES	YES
386	a	2922.86	7.05672	YES	YES
387	a	2923.77	39.02213	YES	YES
388	a	2924.46	23.93406	YES	YES
389	a	2926.23	2.71688	YES	YES
390	a	2926.47	11.85234	YES	YES
391	a	2933.46	6.50771	YES	YES
392	a	2934.01	12.45186	YES	YES
393	a	2934.86	24.71307	YES	YES
394	a	2935.72	12.19062	YES	YES
395	a	2936.62	11.27806	YES	YES
396	a	2938.95	7.53992	YES	YES
397	a	2939.33	23.14275	YES	YES
398	a	2941.31	9.33770	YES	YES
399	a	2941.40	9.36448	YES	YES
400	a	2942.36	15.62894	YES	YES
401	a	2942.78	9.87553	YES	YES
402	a	2943.24	6.24636	YES	YES
403	a	2944.18	4.39547	YES	YES
404	a	2944.27	11.89500	YES	YES
405	a	2945.14	17.80932	YES	YES
406	a	2946.15	9.40827	YES	YES
407	a	2947.61	10.53817	YES	YES
408	a	2948.42	13.48082	YES	YES
409	a	2950.42	5.59787	YES	YES
410	a	2952.57	7.26605	YES	YES
411	a	2953.69	8.68698	YES	YES
412	a	2955.00	4.60775	YES	YES
413	a	2955.23	5.48143	YES	YES
414	a	2955.29	6.23960	YES	YES
415	a	2956.15	22.60525	YES	YES
416	a	2957.56	22.99057	YES	YES
417	a	2957.78	4.82587	YES	YES
418	a	2959.05	29.26288	YES	YES
419	a	2959.20	29.03018	YES	YES
420	a	2959.32	33.14835	YES	YES
421	a	2959.60	32.06276	YES	YES
422	a	2960.02	32.20257	YES	YES
423	a	2960.35	3.95885	YES	YES
424	a	2960.46	27.39159	YES	YES
425	a	2961.88	30.14222	YES	YES
426	a	2962.56	29.29043	YES	YES
427	a	2963.61	26.26666	YES	YES
428	a	2963.74	33.60999	YES	YES
429	a	2972.94	5.64270	YES	YES
430	a	2973.86	11.28746	YES	YES
431	a	2975.39	30.58596	YES	YES
432	a	2975.86	3.87282	YES	YES
433	a	2977.98	17.47017	YES	YES
434	a	2981.11	15.11457	YES	YES
435	a	2981.65	10.87421	YES	YES
436	a	2984.76	5.61968	YES	YES
437	a	2988.90	22.85045	YES	YES
438	a	2989.01	12.24120	YES	YES

439	a	2992.66	32.26213	YES	YES
440	a	2993.60	17.48344	YES	YES
441	a	2995.96	10.80779	YES	YES
442	a	2997.12	23.45433	YES	YES
443	a	2997.41	33.43826	YES	YES
444	a	2999.03	29.93624	YES	YES
445	a	2999.09	10.24735	YES	YES
446	a	2999.48	34.65296	YES	YES
447	a	3001.86	29.08028	YES	YES
448	a	3004.67	23.97699	YES	YES
449	a	3006.01	15.80497	YES	YES
450	a	3007.54	21.94467	YES	YES
451	a	3008.17	3.07422	YES	YES
452	a	3009.24	32.07375	YES	YES
453	a	3010.24	23.22657	YES	YES
454	a	3010.60	20.74896	YES	YES
455	a	3011.57	29.08041	YES	YES
456	a	3012.49	21.00684	YES	YES
457	a	3012.87	28.28500	YES	YES
458	a	3013.06	12.26461	YES	YES
459	a	3013.12	31.73124	YES	YES
460	a	3013.40	20.12159	YES	YES
461	a	3013.95	18.85896	YES	YES
462	a	3014.57	21.72693	YES	YES
463	a	3015.34	35.84804	YES	YES
464	a	3015.78	28.80317	YES	YES
465	a	3015.95	23.34658	YES	YES
466	a	3016.02	20.62877	YES	YES
467	a	3016.80	17.89782	YES	YES
468	a	3016.83	41.01864	YES	YES
469	a	3017.30	37.09610	YES	YES
470	a	3017.35	12.95872	YES	YES
471	a	3017.40	26.83963	YES	YES
472	a	3018.79	31.63110	YES	YES
473	a	3019.22	11.82197	YES	YES
474	a	3020.16	27.79339	YES	YES
475	a	3020.61	22.69690	YES	YES
476	a	3025.23	17.29416	YES	YES
477	a	3030.56	1.20418	YES	YES
478	a	3033.66	2.64505	YES	YES
479	a	3046.30	1.30097	YES	YES
480	a	3056.81	2.90364	YES	YES
481	a	3076.35	0.87004	YES	YES
482	a	3351.88	4.26478	YES	YES
483	a	3437.06	11.90029	YES	YES

$end

Double hybrid single point energy = -7344.811774872377 H
COSMO energy + OC correction = -7352.2564056133 H (in *o*DFB)

6.2.32 [{Ga(dcpe)}$_2$(H–NHMe)]$^{2+}$ (Transition State)

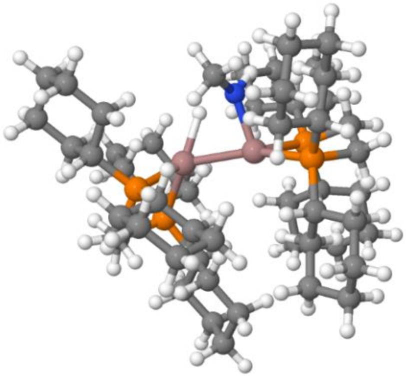

```
Method: (RI-)BP86(D3BJ)/def2-TZVPP
Symmetry: c1

Cartesian coordinates in Ångström:
Ga   -2.0531665   17.2450493   -2.4514219
Ga   -4.1289985   17.8199364   -1.1955610
P    -1.3831561   18.8123770   -4.1678759
P    -6.1614362   19.1351211   -0.8778519
P    -1.9788077   15.5890675   -4.1912354
P    -5.2802737   16.1641784    0.1549283
C    -4.7964182   21.1139823   -4.9643404
H    -4.8532989   21.6628575   -4.0088512
H    -5.8211373   20.8000759   -5.2112229
C    -1.0014541   14.1223936   -3.6462048
H    -0.8186570   13.4941046   -4.5336745
C    -4.2268885   15.3189732    1.4092706
H    -3.9700139   16.1563390    2.0871312
C    -4.3782210   16.1134842   -5.5234908
H    -3.8159551   16.5536448   -6.3626987
H    -4.5210760   16.9101873   -4.7777104
C    -7.0418251   19.3750658   -2.4796428
H    -6.3625495   20.0224103   -3.0586996
C    -7.2859765   18.0976416    0.1748768
H    -7.9415121   18.7618092    0.7542875
H    -7.9337087   17.5251832   -0.5030289
C    -2.8039019   22.4506383   -5.7470292
H    -2.4052064   23.0860917   -6.5491372
H    -2.7751799   23.0549917   -4.8252863
C    -1.9003386   21.2230580   -5.5682303
H    -0.8805786   21.5450098   -5.3185482
H    -1.8383590   20.6751451   -6.5226937
C    -3.9080963   19.8781531   -4.7789617
H    -3.9422178   19.2768618   -5.7017198
H    -4.2846022   19.2343903   -3.9685927
C     1.1178950   13.3592337   -2.4774011
H     1.3910490   12.6762500   -3.2979977
H     2.0605721   13.7095862   -2.0355010
C    -7.4773063   14.3403021   -0.2607091
H    -8.1464217   15.0890689    0.1863541
H    -7.1519758   13.6790211    0.5552987
C    -6.0636516   20.7811773   -0.0577674
H    -7.0948389   21.1744527   -0.0547565
C     0.3640045   20.3688964   -2.6502486
H    -0.3287152   21.2142308   -2.7742556
```

H	0.0203862	19.7921814	-1.7758325
C	1.7834405	20.8858865	-2.3850158
H	2.0980190	21.5301333	-3.2221121
H	1.7808071	21.5190789	-1.4871366
C	-7.1754831	18.0422287	-3.2356699
H	-6.1857240	17.5682785	-3.3446215
H	-7.8003448	17.3474964	-2.6493839
C	-4.2492359	22.0468763	-6.0485398
H	-4.2910034	21.5339890	-7.0235401
H	-4.8835161	22.9389411	-6.1386761
C	-8.3982279	20.0864852	-2.3449766
H	-8.2793100	21.0563808	-1.8432455
H	-9.0708348	19.4806975	-1.7157199
C	-5.1771019	21.7302444	-0.8826079
H	-5.5929141	21.8526832	-1.8931143
H	-4.1786855	21.2790717	-1.0036118
C	-3.3644387	13.8166884	-5.9175737
H	-2.8181200	12.9838915	-5.4546275
H	-2.7515496	14.1685569	-6.7634318
C	2.7437261	18.8111554	-3.4523361
H	3.1059410	19.3638839	-4.3343881
H	3.4249054	17.9599021	-3.3161786
C	-5.4551310	22.0377249	2.0553259
H	-6.4536081	22.4950659	2.1456052
H	-5.0725889	21.9208823	3.0783856
C	-9.1795617	18.9666938	-4.4800973
H	-9.6093970	19.1403216	-5.4756188
H	-9.8887927	18.3138804	-3.9446357
C	-4.9330657	14.2388404	2.2474422
H	-5.8648983	14.6250389	2.6839027
H	-5.2091960	13.3995148	1.5903753
C	0.2835950	12.6034556	-1.4399792
H	0.8419511	11.7391730	-1.0566694
H	0.0926252	13.2640477	-0.5770136
C	-7.8313842	18.2509529	-4.6057433
H	-7.1537639	18.8453830	-5.2404708
H	-7.9576953	17.2806641	-5.1034567
C	2.7739609	19.7291070	-2.2275850
H	2.5149693	19.1468763	-1.3274263
H	3.7892535	20.1160013	-2.0689112
C	-4.5466710	22.9629676	1.2404777
H	-4.4920996	23.9512260	1.7158147
H	-3.5186838	22.5631961	1.2381393
C	-5.3345703	13.9793336	-1.5752329
H	-4.9367497	13.2947008	-0.8110093
H	-4.4679537	14.4871725	-2.0246904
C	-5.5945912	20.6560742	1.4019321
H	-4.6292549	20.1262895	1.4319213
H	-6.3075624	20.0473728	1.9769586
C	-6.2547630	15.0113460	-0.9053989
H	-6.6212512	15.6809076	-1.7061376
C	-5.0457422	23.0979720	-0.2009060
C	-7.3370275	12.4977298	-2.0028953
H	-7.8997389	11.9535306	-2.7730246
H	-7.0090754	11.7459084	-1.2666592
C	-8.2420857	13.5236323	-1.3125945
H	-9.0981327	13.0240938	-0.8397237
H	-8.6587995	14.2111492	-2.0680030

C	-1.8333397	13.3334362	-2.6193644
H	-2.7696078	12.9778725	-3.0724855
H	-2.1124965	14.0207302	-1.8027124
C	-3.9977517	13.7294897	3.3534029
H	-3.8002626	14.5494593	4.0632379
H	-4.5028838	12.9384093	3.9238873
C	-5.7241935	15.6079056	-6.0536377
H	-6.2712288	16.4389687	-6.5188911
H	-6.3373343	15.2613602	-5.2046581
C	-2.6724207	13.2169999	2.7802360
H	-2.8640679	12.3230379	2.1634815
H	-2.0079303	12.8964058	3.5934937
C	-2.9119190	14.7867294	0.8104029
H	-3.1383617	13.9534378	0.1295328
H	-2.4129082	15.5623902	0.2091477
C	-9.0398113	20.2902907	-3.7242444
H	-10.0191240	20.7729242	-3.6058993
H	-8.4166129	20.9859933	-4.3098849
C	-4.7174785	13.3218173	-6.4485454
H	-4.5511006	12.5301237	-7.1913302
H	-5.2804124	12.8617177	-5.6206006
C	-6.1061218	13.1691108	-2.6205701
H	-5.4435327	12.4176842	-3.0742550
H	-6.4253138	13.8420962	-3.4341681
C	-5.5390675	14.4629074	-7.0538094
H	-6.5159156	14.0908250	-7.3907499
H	-5.0261993	14.8462069	-7.9513385
C	-1.0509423	12.1511910	-2.0371729
H	-1.6653608	11.6451846	-1.2788541
H	-0.8660206	11.4115804	-2.8327390
C	-1.9844068	14.2835286	1.9216214
H	-1.6845577	15.1339507	2.5562276
H	-1.0611384	13.8856110	1.4775233
H	-1.8210778	17.5408187	-0.7348835
C	0.3269275	19.4501949	-3.8816674
H	0.5938970	20.0335460	-4.7792526
C	1.3280276	18.2909688	-3.7327605
H	1.3383730	17.6735561	-4.6421752
H	1.0001255	17.6423420	-2.9024180
C	0.3478168	14.5571444	-3.0461205
H	0.9605176	15.0712896	-3.7998057
H	0.1572446	15.2864004	-2.2393196
C	-1.0329169	16.3681497	-5.5777465
H	0.0269060	16.1989195	-5.3511886
H	-1.2491119	15.8230962	-6.5070653
C	-1.3068590	17.8705039	-5.7613258
H	-2.2747774	18.0230965	-6.2549538
H	-0.5408833	18.3176368	-6.4104878
C	-3.5614634	14.9637754	-4.9127169
H	-4.1206325	14.5787996	-4.0449351
C	-2.4565042	20.2873564	-4.4810271
H	-2.4509603	20.8245028	-3.5157053
C	-6.5141095	17.1503290	1.1043865
H	-7.2054933	16.4926888	1.6486124
H	-5.9443472	17.7186498	1.8528791
H	-6.0284386	23.5965879	-0.2024446
H	-4.3713637	23.7368986	-0.7875394
C	-2.1525014	19.6656260	0.1762575

```
N    -2.7670660   18.3388059    0.2033590
H    -1.9703397   19.9466302   -0.8729373
H    -1.1815368   19.6693804    0.6926800
H    -2.7771391   20.4536958    0.6205728
H    -2.8489349   17.9886158    1.1593874

SCF energy GEOOPT =  -7352.070844895 H
ZPE =   3750. kJ/mol
FREEH energy =  3935.62 kJ/mol
FREEH entropy =   1.57842 kJ/mol/K

$vibrational spectrum
#  mode     symmetry     wave number   IR intensity    selection rules
#                         cm**(-1)        km/mol         IR     RAMAN
     1        a           -1046.19        0.00000        YES     YES
     2                       -0.00        0.00000         -       -
     3                       -0.00        0.00000         -       -
     4                       -0.00        0.00000         -       -
     5                       -0.00        0.00000         -       -
     6                       -0.00        0.00000         -       -
     7                        0.00        0.00000         -       -
     8        a              16.43        0.02547        YES     YES
     9        a              21.06        0.02424        YES     YES
    10        a              25.88        0.13202        YES     YES
    11        a              26.76        0.07917        YES     YES
    12        a              28.21        0.03598        YES     YES
    13        a              32.22        0.02795        YES     YES
    14        a              32.78        0.02982        YES     YES
    15        a              38.02        0.02038        YES     YES
    16        a              38.81        0.09128        YES     YES
    17        a              40.66        0.02383        YES     YES
    18        a              41.17        0.02015        YES     YES
    19        a              44.59        0.20055        YES     YES
    20        a              46.16        0.18174        YES     YES
    21        a              52.16        0.04118        YES     YES
    22        a              55.62        0.08855        YES     YES
    23        a              57.22        0.02278        YES     YES
    24        a              60.86        0.20170        YES     YES
    25        a              62.46        0.06022        YES     YES
    26        a              63.51        0.03694        YES     YES
    27        a              67.15        0.09590        YES     YES
    28        a              68.46        0.25699        YES     YES
    29        a              70.01        0.28283        YES     YES
    30        a              71.68        0.02948        YES     YES
    31        a              72.01        0.09523        YES     YES
    32        a              75.80        0.07047        YES     YES
    33        a              83.52        0.42742        YES     YES
    34        a              86.24        0.11542        YES     YES
    35        a              93.25        0.00067        YES     YES
    36        a              96.56        0.03541        YES     YES
    37        a             107.62        0.24511        YES     YES
    38        a             114.87        5.33925        YES     YES
    39        a             119.16        0.63287        YES     YES
    40        a             129.63        0.18398        YES     YES
    41        a             131.67        1.37365        YES     YES
    42        a             136.61        0.28993        YES     YES
    43        a             137.81        2.27813        YES     YES
    44        a             139.03        3.24593        YES     YES
```

45	a	143.27	0.24076	YES	YES
46	a	146.83	0.24174	YES	YES
47	a	152.05	1.44997	YES	YES
48	a	158.12	5.66077	YES	YES
49	a	170.90	0.25166	YES	YES
50	a	176.97	1.07059	YES	YES
51	a	181.77	0.90270	YES	YES
52	a	187.08	0.65490	YES	YES
53	a	190.04	1.01062	YES	YES
54	a	199.68	1.40165	YES	YES
55	a	203.38	0.07640	YES	YES
56	a	214.51	0.51080	YES	YES
57	a	215.60	0.24660	YES	YES
58	a	216.47	0.26918	YES	YES
59	a	219.47	0.06919	YES	YES
60	a	224.34	0.21194	YES	YES
61	a	225.20	1.97534	YES	YES
62	a	225.83	0.51869	YES	YES
63	a	230.45	2.00776	YES	YES
64	a	234.40	0.04678	YES	YES
65	a	236.43	0.44468	YES	YES
66	a	237.74	0.03996	YES	YES
67	a	239.77	0.74669	YES	YES
68	a	240.65	1.12925	YES	YES
69	a	242.57	0.53943	YES	YES
70	a	249.19	1.25677	YES	YES
71	a	258.16	0.82161	YES	YES
72	a	273.58	0.83254	YES	YES
73	a	280.50	0.13681	YES	YES
74	a	283.80	0.61166	YES	YES
75	a	288.31	0.58700	YES	YES
76	a	297.18	1.64256	YES	YES
77	a	305.21	0.64130	YES	YES
78	a	309.61	9.55543	YES	YES
79	a	312.28	0.37725	YES	YES
80	a	314.10	0.63785	YES	YES
81	a	329.31	0.63731	YES	YES
82	a	330.75	0.37770	YES	YES
83	a	333.15	0.92994	YES	YES
84	a	336.00	1.09250	YES	YES
85	a	338.71	1.35850	YES	YES
86	a	340.09	0.49491	YES	YES
87	a	371.72	4.62928	YES	YES
88	a	381.60	2.78260	YES	YES
89	a	383.07	1.58579	YES	YES
90	a	384.55	1.04137	YES	YES
91	a	394.34	1.04848	YES	YES
92	a	396.93	1.13867	YES	YES
93	a	408.29	0.02313	YES	YES
94	a	413.27	0.42972	YES	YES
95	a	419.05	1.57532	YES	YES
96	a	421.19	3.54438	YES	YES
97	a	422.88	3.49170	YES	YES
98	a	428.56	1.99110	YES	YES
99	a	430.45	0.06278	YES	YES
100	a	430.72	0.50762	YES	YES
101	a	431.31	0.07211	YES	YES
102	a	432.22	0.13538	YES	YES

103	a	432.70	0.32875	YES	YES
104	a	433.75	0.44346	YES	YES
105	a	435.42	2.75932	YES	YES
106	a	437.98	0.79189	YES	YES
107	a	443.86	8.57315	YES	YES
108	a	457.09	17.57950	YES	YES
109	a	459.90	21.87072	YES	YES
110	a	460.51	15.32680	YES	YES
111	a	473.38	3.12916	YES	YES
112	a	492.98	6.53204	YES	YES
113	a	493.67	3.24644	YES	YES
114	a	495.76	1.93132	YES	YES
115	a	499.14	1.64548	YES	YES
116	a	502.32	1.88688	YES	YES
117	a	503.99	1.41439	YES	YES
118	a	512.23	6.84789	YES	YES
119	a	513.85	14.70729	YES	YES
120	a	598.81	44.70600	YES	YES
121	a	629.86	7.12861	YES	YES
122	a	631.17	27.87278	YES	YES
123	a	642.20	9.87434	YES	YES
124	a	664.76	15.71267	YES	YES
125	a	701.26	1.78848	YES	YES
126	a	716.04	1.55631	YES	YES
127	a	721.65	3.78280	YES	YES
128	a	729.69	11.19535	YES	YES
129	a	732.65	2.50134	YES	YES
130	a	733.66	0.25849	YES	YES
131	a	735.85	5.00178	YES	YES
132	a	736.56	8.29960	YES	YES
133	a	769.49	0.31082	YES	YES
134	a	771.98	7.84043	YES	YES
135	a	772.47	0.50174	YES	YES
136	a	774.03	2.47018	YES	YES
137	a	774.80	9.75771	YES	YES
138	a	775.54	0.27339	YES	YES
139	a	776.59	1.90407	YES	YES
140	a	777.26	0.95337	YES	YES
141	a	777.62	0.13281	YES	YES
142	a	787.02	18.02917	YES	YES
143	a	806.85	0.45398	YES	YES
144	a	809.42	1.76733	YES	YES
145	a	810.28	5.60838	YES	YES
146	a	811.83	6.90053	YES	YES
147	a	812.42	0.67069	YES	YES
148	a	812.51	6.49502	YES	YES
149	a	814.78	2.93124	YES	YES
150	a	817.87	3.32159	YES	YES
151	a	831.84	4.31783	YES	YES
152	a	832.69	3.41114	YES	YES
153	a	834.79	11.58587	YES	YES
154	a	837.43	0.66935	YES	YES
155	a	837.83	2.10898	YES	YES
156	a	838.47	1.62693	YES	YES
157	a	839.18	3.40763	YES	YES
158	a	840.32	6.53387	YES	YES
159	a	848.39	21.70145	YES	YES
160	a	854.67	18.50930	YES	YES

161	a	872.68	3.31255	YES	YES
162	a	873.34	1.30084	YES	YES
163	a	875.66	0.35086	YES	YES
164	a	875.90	1.68813	YES	YES
165	a	876.74	5.05051	YES	YES
166	a	877.17	1.08198	YES	YES
167	a	878.45	2.63461	YES	YES
168	a	879.04	10.11234	YES	YES
169	a	879.37	1.40623	YES	YES
170	a	879.64	2.53659	YES	YES
171	a	881.73	0.96605	YES	YES
172	a	882.20	5.08248	YES	YES
173	a	883.23	2.64980	YES	YES
174	a	884.76	4.16394	YES	YES
175	a	885.36	0.56864	YES	YES
176	a	886.47	5.40907	YES	YES
177	a	903.03	1.67509	YES	YES
178	a	903.17	1.76562	YES	YES
179	a	904.32	5.80780	YES	YES
180	a	905.22	1.20546	YES	YES
181	a	907.96	0.74850	YES	YES
182	a	909.15	1.31653	YES	YES
183	a	910.33	0.83201	YES	YES
184	a	910.91	0.78888	YES	YES
185	a	960.27	55.64081	YES	YES
186	a	983.30	1.50320	YES	YES
187	a	983.55	0.09965	YES	YES
188	a	986.52	12.20726	YES	YES
189	a	987.46	2.52428	YES	YES
190	a	988.48	5.55337	YES	YES
191	a	989.39	15.45875	YES	YES
192	a	989.66	9.78165	YES	YES
193	a	990.83	1.75324	YES	YES
194	a	991.79	12.56933	YES	YES
195	a	991.84	13.46162	YES	YES
196	a	1013.99	0.98245	YES	YES
197	a	1015.33	1.35467	YES	YES
198	a	1016.19	0.92281	YES	YES
199	a	1017.29	1.64990	YES	YES
200	a	1018.47	0.37728	YES	YES
201	a	1019.46	0.61889	YES	YES
202	a	1019.94	0.40284	YES	YES
203	a	1020.26	2.26891	YES	YES
204	a	1022.15	0.47557	YES	YES
205	a	1029.99	1.30840	YES	YES
206	a	1031.55	0.87260	YES	YES
207	a	1032.76	2.30068	YES	YES
208	a	1033.91	1.68606	YES	YES
209	a	1037.70	2.15088	YES	YES
210	a	1038.90	1.55028	YES	YES
211	a	1039.18	0.43428	YES	YES
212	a	1040.61	0.72835	YES	YES
213	a	1043.13	29.73353	YES	YES
214	a	1056.64	1.29175	YES	YES
215	a	1057.38	2.32292	YES	YES
216	a	1058.53	2.31050	YES	YES
217	a	1059.40	1.25613	YES	YES
218	a	1060.83	1.83054	YES	YES

219	a	1064.42	0.24943	YES	YES
220	a	1065.03	0.66881	YES	YES
221	a	1067.67	2.14121	YES	YES
222	a	1069.39	0.06045	YES	YES
223	a	1071.18	0.41805	YES	YES
224	a	1072.22	0.21263	YES	YES
225	a	1072.71	0.13381	YES	YES
226	a	1073.18	0.12360	YES	YES
227	a	1073.62	0.11102	YES	YES
228	a	1074.40	0.09475	YES	YES
229	a	1075.12	0.23311	YES	YES
230	a	1076.83	3.13070	YES	YES
231	a	1080.37	3.65900	YES	YES
232	a	1090.55	3.51650	YES	YES
233	a	1092.49	4.17840	YES	YES
234	a	1093.45	4.62450	YES	YES
235	a	1094.38	7.86730	YES	YES
236	a	1099.60	1.65246	YES	YES
237	a	1099.85	5.25678	YES	YES
238	a	1103.15	2.69137	YES	YES
239	a	1106.60	8.05722	YES	YES
240	a	1115.07	8.72900	YES	YES
241	a	1121.45	4.56881	YES	YES
242	a	1129.63	2.26988	YES	YES
243	a	1158.92	5.67239	YES	YES
244	a	1160.70	6.08412	YES	YES
245	a	1161.52	9.72025	YES	YES
246	a	1165.82	8.49460	YES	YES
247	a	1167.94	7.43908	YES	YES
248	a	1169.29	13.91376	YES	YES
249	a	1170.16	1.99938	YES	YES
250	a	1170.58	9.79873	YES	YES
251	a	1176.07	10.17433	YES	YES
252	a	1178.08	18.51323	YES	YES
253	a	1179.24	5.96339	YES	YES
254	a	1179.48	2.68016	YES	YES
255	a	1186.01	0.75528	YES	YES
256	a	1188.51	0.54307	YES	YES
257	a	1191.52	0.81527	YES	YES
258	a	1210.49	2.26112	YES	YES
259	a	1236.88	2.28995	YES	YES
260	a	1239.10	0.97532	YES	YES
261	a	1242.93	0.81540	YES	YES
262	a	1245.21	0.50694	YES	YES
263	a	1245.50	0.34832	YES	YES
264	a	1245.91	2.11624	YES	YES
265	a	1246.48	0.04058	YES	YES
266	a	1248.67	1.91204	YES	YES
267	a	1249.44	0.66246	YES	YES
268	a	1250.44	1.08231	YES	YES
269	a	1252.66	1.05673	YES	YES
270	a	1254.04	0.85833	YES	YES
271	a	1254.68	1.51679	YES	YES
272	a	1255.94	3.17916	YES	YES
273	a	1256.75	3.91810	YES	YES
274	a	1257.36	2.63558	YES	YES
275	a	1257.63	3.48334	YES	YES
276	a	1258.01	27.91069	YES	YES

277	a	1258.44	5.55269	YES	YES
278	a	1259.24	10.04352	YES	YES
279	a	1260.76	0.54116	YES	YES
280	a	1261.05	8.41523	YES	YES
281	a	1261.60	3.30753	YES	YES
282	a	1262.96	6.05882	YES	YES
283	a	1264.74	10.06762	YES	YES
284	a	1265.39	3.96324	YES	YES
285	a	1267.35	98.52854	YES	YES
286	a	1269.31	31.41225	YES	YES
287	a	1271.07	26.41526	YES	YES
288	a	1280.85	3.40239	YES	YES
289	a	1282.30	0.91586	YES	YES
290	a	1282.45	1.36042	YES	YES
291	a	1284.34	2.84114	YES	YES
292	a	1288.68	3.93567	YES	YES
293	a	1289.26	1.69772	YES	YES
294	a	1290.99	3.23699	YES	YES
295	a	1291.33	4.09502	YES	YES
296	a	1300.45	2.88269	YES	YES
297	a	1307.33	0.79216	YES	YES
298	a	1309.01	0.70428	YES	YES
299	a	1310.40	2.12377	YES	YES
300	a	1310.66	0.68586	YES	YES
301	a	1311.53	0.49528	YES	YES
302	a	1314.44	0.99463	YES	YES
303	a	1314.78	1.63351	YES	YES
304	a	1317.21	1.93731	YES	YES
305	a	1318.74	1.00737	YES	YES
306	a	1320.44	0.49845	YES	YES
307	a	1321.75	0.80548	YES	YES
308	a	1322.20	0.97015	YES	YES
309	a	1322.86	1.05550	YES	YES
310	a	1323.43	0.62656	YES	YES
311	a	1323.77	4.13879	YES	YES
312	a	1324.07	1.95411	YES	YES
313	a	1324.52	0.41549	YES	YES
314	a	1324.76	0.82846	YES	YES
315	a	1325.22	3.98632	YES	YES
316	a	1326.12	1.38082	YES	YES
317	a	1326.60	0.89575	YES	YES
318	a	1327.29	7.21782	YES	YES
319	a	1329.29	7.30623	YES	YES
320	a	1330.38	3.41472	YES	YES
321	a	1335.99	0.28557	YES	YES
322	a	1336.62	0.74750	YES	YES
323	a	1336.93	0.12338	YES	YES
324	a	1337.34	0.17120	YES	YES
325	a	1337.81	0.30593	YES	YES
326	a	1338.21	0.12627	YES	YES
327	a	1338.38	0.05332	YES	YES
328	a	1338.81	0.15885	YES	YES
329	a	1344.69	0.31105	YES	YES
330	a	1345.05	1.58979	YES	YES
331	a	1345.90	0.27795	YES	YES
332	a	1346.18	2.04993	YES	YES
333	a	1347.56	1.64850	YES	YES
334	a	1348.16	1.61604	YES	YES

335	a	1348.83	1.04759	YES	YES
336	a	1349.60	0.94660	YES	YES
337	a	1401.62	4.54861	YES	YES
338	a	1403.83	4.87302	YES	YES
339	a	1406.28	7.18507	YES	YES
340	a	1407.22	10.91900	YES	YES
341	a	1410.14	6.29248	YES	YES
342	a	1425.00	2.30875	YES	YES
343	a	1426.51	7.59813	YES	YES
344	a	1427.39	5.69530	YES	YES
345	a	1430.32	4.75325	YES	YES
346	a	1431.70	4.78263	YES	YES
347	a	1433.23	1.97670	YES	YES
348	a	1433.70	0.30597	YES	YES
349	a	1434.01	4.25045	YES	YES
350	a	1434.43	3.85039	YES	YES
351	a	1434.85	4.09462	YES	YES
352	a	1435.69	2.89871	YES	YES
353	a	1436.06	8.04994	YES	YES
354	a	1436.51	9.92217	YES	YES
355	a	1437.51	11.89256	YES	YES
356	a	1437.57	3.99946	YES	YES
357	a	1438.11	3.23517	YES	YES
358	a	1438.48	7.37153	YES	YES
359	a	1438.70	2.82439	YES	YES
360	a	1439.19	3.50697	YES	YES
361	a	1439.41	5.82995	YES	YES
362	a	1439.78	10.90425	YES	YES
363	a	1439.86	1.56970	YES	YES
364	a	1440.22	0.71880	YES	YES
365	a	1440.55	0.51905	YES	YES
366	a	1442.01	16.46946	YES	YES
367	a	1442.39	5.89422	YES	YES
368	a	1443.26	10.55799	YES	YES
369	a	1443.86	6.07051	YES	YES
370	a	1444.47	37.96068	YES	YES
371	a	1444.75	7.20540	YES	YES
372	a	1445.80	12.94878	YES	YES
373	a	1446.27	14.65566	YES	YES
374	a	1447.41	3.65083	YES	YES
375	a	1452.70	0.86803	YES	YES
376	a	1453.61	2.14538	YES	YES
377	a	1454.06	1.67959	YES	YES
378	a	1454.73	0.92821	YES	YES
379	a	1455.04	4.17342	YES	YES
380	a	1456.28	1.71049	YES	YES
381	a	1456.84	0.32084	YES	YES
382	a	1457.03	3.09514	YES	YES
383	a	1467.82	3.20028	YES	YES
384	a	2898.72	6.60381	YES	YES
385	a	2916.42	0.81922	YES	YES
386	a	2927.22	11.94831	YES	YES
387	a	2928.20	31.44495	YES	YES
388	a	2929.57	1.89406	YES	YES
389	a	2933.83	8.43966	YES	YES
390	a	2937.64	18.62938	YES	YES
391	a	2938.64	10.17110	YES	YES
392	a	2938.74	34.81137	YES	YES

393	a	2939.37	9.09783	YES	YES
394	a	2940.58	14.19675	YES	YES
395	a	2941.10	2.34714	YES	YES
396	a	2941.25	16.20021	YES	YES
397	a	2942.27	20.27313	YES	YES
398	a	2942.48	13.54146	YES	YES
399	a	2943.23	13.34716	YES	YES
400	a	2943.25	13.89362	YES	YES
401	a	2943.68	5.16651	YES	YES
402	a	2944.02	6.90746	YES	YES
403	a	2944.22	9.03494	YES	YES
404	a	2946.27	8.62206	YES	YES
405	a	2946.77	7.96218	YES	YES
406	a	2947.01	10.45747	YES	YES
407	a	2948.98	14.50956	YES	YES
408	a	2949.58	13.29585	YES	YES
409	a	2951.48	2.84455	YES	YES
410	a	2951.87	21.08139	YES	YES
411	a	2952.57	6.52021	YES	YES
412	a	2953.48	10.41425	YES	YES
413	a	2954.97	5.48076	YES	YES
414	a	2955.96	2.94916	YES	YES
415	a	2956.11	13.14969	YES	YES
416	a	2956.97	5.10942	YES	YES
417	a	2957.38	9.55009	YES	YES
418	a	2958.24	4.08415	YES	YES
419	a	2958.41	0.45486	YES	YES
420	a	2959.54	29.05626	YES	YES
421	a	2959.60	12.71473	YES	YES
422	a	2959.89	20.87491	YES	YES
423	a	2960.11	2.32410	YES	YES
424	a	2960.20	56.20132	YES	YES
425	a	2960.65	36.77762	YES	YES
426	a	2961.25	40.69386	YES	YES
427	a	2962.40	43.65838	YES	YES
428	a	2962.55	27.80517	YES	YES
429	a	2963.57	27.42760	YES	YES
430	a	2963.67	8.46950	YES	YES
431	a	2972.65	0.47374	YES	YES
432	a	2973.87	9.69142	YES	YES
433	a	2976.09	37.23628	YES	YES
434	a	2982.14	5.87877	YES	YES
435	a	2982.99	17.03971	YES	YES
436	a	2984.62	16.51861	YES	YES
437	a	2986.16	5.35004	YES	YES
438	a	2990.95	2.99947	YES	YES
439	a	2994.23	3.27455	YES	YES
440	a	2994.94	39.34386	YES	YES
441	a	2996.15	17.65098	YES	YES
442	a	2997.26	19.56592	YES	YES
443	a	2998.80	26.67392	YES	YES
444	a	2999.63	30.23685	YES	YES
445	a	2999.79	26.12256	YES	YES
446	a	3000.47	48.06044	YES	YES
447	a	3000.59	19.66116	YES	YES
448	a	3001.65	9.14461	YES	YES
449	a	3002.38	13.03577	YES	YES
450	a	3003.52	19.03990	YES	YES

451	a	3004.80	29.43485	YES	YES
452	a	3007.20	7.37226	YES	YES
453	a	3007.98	12.06132	YES	YES
454	a	3009.81	18.74131	YES	YES
455	a	3010.03	18.60788	YES	YES
456	a	3010.66	26.00725	YES	YES
457	a	3010.72	20.49506	YES	YES
458	a	3011.49	12.50265	YES	YES
459	a	3011.69	20.78231	YES	YES
460	a	3012.05	30.18189	YES	YES
461	a	3013.29	18.78409	YES	YES
462	a	3013.63	18.72037	YES	YES
463	a	3013.87	17.93605	YES	YES
464	a	3014.92	29.62980	YES	YES
465	a	3015.04	22.53125	YES	YES
466	a	3015.24	25.55326	YES	YES
467	a	3015.93	19.61403	YES	YES
468	a	3016.38	26.95114	YES	YES
469	a	3017.21	20.81423	YES	YES
470	a	3017.33	0.42908	YES	YES
471	a	3017.34	29.15247	YES	YES
472	a	3017.47	42.63310	YES	YES
473	a	3018.12	17.96137	YES	YES
474	a	3018.15	58.73378	YES	YES
475	a	3018.45	22.36869	YES	YES
476	a	3018.55	29.48580	YES	YES
477	a	3019.29	26.38023	YES	YES
478	a	3020.26	25.37807	YES	YES
479	a	3027.86	4.43886	YES	YES
480	a	3032.00	0.98483	YES	YES
481	a	3036.77	2.79732	YES	YES
482	a	3047.34	0.68772	YES	YES
483	a	3426.56	1.84873	YES	YES

$end

Double hybrid single point energy = -7344.774077423213 H
COSMO energy + OC correction = -7352.2183821669 H (in *o*DFB)

6.2.33 [H{Ga(dcpe)}$_2$(NHMe)]$^{2+}$ (*syn*-periplanar)

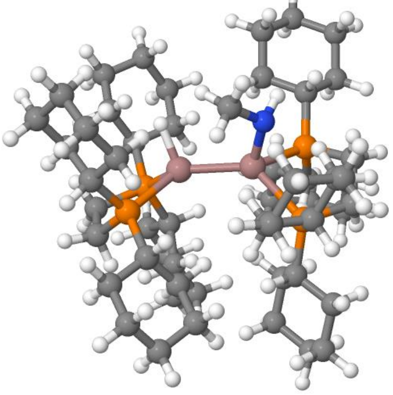

Method: (RI-)BP86(D3BJ)/def2-TZVPP
Symmetry: c1

Cartesian coordinates in Ångström:

Ga	-2.2404231	17.3527965	-2.5024946
Ga	-3.9384769	17.8124576	-0.8264875
P	-1.5198820	18.9345226	-4.1588329
P	-6.0235467	19.0968101	-0.9057996
P	-2.0427539	15.6620429	-4.1927030
P	-5.2552851	16.1445058	0.2898787
C	-5.0026325	21.0570142	-5.1053997
H	-5.1182909	21.6503023	-4.1825330
H	-6.0027416	20.6825706	-5.3659617
C	-1.0555195	14.2100012	-3.6191397
H	-0.9400076	13.5446237	-4.4909558
C	-4.2894758	15.2201477	1.5563731
H	-4.0640036	16.0072157	2.3007764
C	-4.3845548	16.1242744	-5.6398948
H	-3.7892398	16.5486654	-6.4642289
H	-4.5730826	16.9405313	-4.9257858
C	-6.9452785	19.2696748	-2.4938106
H	-6.3078639	19.9342803	-3.0987076
C	-7.1708954	18.1760613	0.2281006
H	-7.7807574	18.8970186	0.7878587
H	-7.8606830	17.6082491	-0.4115956
C	-3.0514738	22.4508668	-5.8874313
H	-2.6581811	23.0734043	-6.7020417
H	-3.0820147	23.0909687	-4.9904140
C	-2.0968110	21.2767520	-5.6346244
H	-1.1010723	21.6567478	-5.3702938
H	-1.9820901	20.6974214	-6.5653638
C	-4.0639014	19.8746215	-4.8344012
H	-4.0525038	19.2273115	-5.7256620
H	-4.4338770	19.2555435	-4.0018226
C	1.0860083	13.3817061	-2.5526867
H	1.2768267	12.6636663	-3.3666884
H	2.0689401	13.6972795	-2.1775979
C	-7.5084336	14.4256874	-0.2228102
H	-8.1465929	15.1778919	0.2625136
H	-7.2163318	13.7086001	0.5569794
C	-5.7836365	20.8038161	-0.2543383
H	-6.7132015	21.3541767	-0.4790854
C	0.0797224	20.4165989	-2.4611778
H	-0.6652623	21.2234648	-2.5260621
H	-0.2528731	19.7326034	-1.6638401
C	1.4514133	20.9940923	-2.0915336
H	1.7414878	21.7507564	-2.8384506
H	1.3794148	21.5166840	-1.1278808
C	-7.0617949	17.9223138	-3.2238398
H	-6.0661444	17.4652206	-3.3444030
H	-7.6587538	17.2278232	-2.6088888
C	-4.4640678	21.9627185	-6.2161579
H	-4.4480779	21.4040908	-7.1665454
H	-5.1378413	22.8163904	-6.3678972
C	-8.3233231	19.9368150	-2.3368281
H	-8.2295058	20.9130332	-1.8427057
H	-8.9631832	19.3126434	-1.6922385
C	-4.6244480	21.4564915	-1.0312333
H	-4.8397611	21.4591790	-2.1105275
H	-3.7175217	20.8467000	-0.8864423

C	-3.3353872	13.8276332	-5.9259642
H	-2.8116905	13.0097908	-5.4134737
H	-2.6812241	14.1591377	-6.7491413
C	2.5879298	19.1296913	-3.3571483
H	2.9245684	19.8088169	-4.1571977
H	3.3313586	18.3229565	-3.2988848
C	-5.2804336	22.2768869	1.7322927
H	-6.1870530	22.8856720	1.5807078
H	-5.0815130	22.2770146	2.8125492
C	-9.1187364	18.7683061	-4.4382304
H	-9.5733567	18.9147414	-5.4270887
H	-9.7995513	18.1059308	-3.8781870
C	-5.0438673	14.0914259	2.2793530
H	-5.9890291	14.4599238	2.7022222
H	-5.3005457	13.3063370	1.5512135
C	0.2812319	12.6955349	-1.4456533
H	0.8232417	11.8188927	-1.0670460
H	0.1678438	13.3887484	-0.5948168
C	-7.7558745	18.0843061	-4.5813780
H	-7.1096081	18.6808133	-5.2459038
H	-7.8702182	17.1010267	-5.0556461
C	2.5171021	19.8962762	-2.0335550
H	2.2755527	19.1956423	-1.2169008
H	3.4969348	20.3303706	-1.7946271
C	-4.1111104	22.9103153	0.9718874
H	-3.9520546	23.9427184	1.3105330
H	-3.1851268	22.3568772	1.1998989
C	-5.3690563	14.0544379	-1.5506585
H	-4.9880520	13.3191880	-0.8256311
H	-4.4895151	14.5596777	-1.9777110
C	-5.5424255	20.8394122	1.2645939
H	-4.6765368	20.1990696	1.4953977
H	-6.4133829	20.4294856	1.7969598
C	-6.2560928	15.0793896	-0.8273138
H	-6.5938903	15.8010914	-1.5927888
C	-4.3587553	22.8837165	-0.5394166
C	-7.4280229	12.6742216	-2.0579313
H	-8.0059250	12.1909346	-2.8569115
H	-7.1337038	11.8747276	-1.3587613
C	-8.2977846	13.6983561	-1.3208563
H	-9.1769057	13.2093879	-0.8805177
H	-8.6807058	14.4403778	-2.0420274
C	-1.8647689	13.4829901	-2.5293603
H	-2.8355628	13.1534664	-2.9265994
H	-2.0763247	14.2010470	-1.7190587
C	-4.1640296	13.4851849	3.3815910
H	-3.9819314	14.2462119	4.1578732
H	-4.7051565	12.6639989	3.8705922
C	-5.7005166	15.5907342	-6.2168487
H	-6.2381319	16.4048730	-6.7212374
H	-6.3441642	15.2513830	-5.3881371
C	-2.8257287	12.9908628	2.8230672
H	-3.0081296	12.1540979	2.1280171
H	-2.2020980	12.5910001	3.6335138
C	-2.9483863	14.7238064	0.9814301
H	-3.1458004	13.9635230	0.2106851
H	-2.4160806	15.5517390	0.4875290
C	-8.9988392	20.1061584	-3.7042876

```
H    -9.9886927   20.5625774   -3.5706526
H    -8.4087109   20.8112198   -4.3122860
C    -4.6577474   13.3095388   -6.5084748
H    -4.4502402   12.4993824   -7.2201779
H    -5.2577093   12.8675776   -5.6964573
C    -6.1682486   13.3271896   -2.6363461
H    -5.5340565   12.5740163   -3.1265118
H    -6.4579821   14.0528020   -3.4156349
C    -5.4565263   14.4276805   -7.1820583
H    -6.4126412   14.0394737   -7.5576192
H    -4.9030163   14.7953339   -8.0618026
C    -1.1022684   12.2846185   -1.9541268
H    -1.6950712   11.8285402   -1.1482767
H    -0.9937106   11.5149540   -2.7351817
C    -2.0815232   14.1088545    2.0854626
H    -1.7966419   14.8974772    2.8010707
H    -1.1462608   13.7297269    1.6501200
H    -0.8875651   17.0935471   -1.7162480
C     0.1435833   19.6311720   -3.7830646
H     0.3815599   20.3239059   -4.6080485
C     1.2246177   18.5370149   -3.7377352
H     1.3002078   18.0353780   -4.7135635
H     0.9345684   17.7751129   -2.9967998
C     0.3424981   14.6026965   -3.1099844
H     0.9338742   15.0589087   -3.9161812
H     0.2385773   15.3609101   -2.3188228
C    -1.0630727   16.4812765   -5.5309527
H    -0.0119355   16.3549051   -5.2433386
H    -1.2042610   15.9298179   -6.4711161
C    -1.3774501   17.9731831   -5.7346960
H    -2.3340379   18.0953068   -6.2580607
H    -0.6067959   18.4366214   -6.3660206
C    -3.5841311   15.0001149   -4.9623843
H    -4.1771677   14.6322575   -4.1089778
C    -2.6376302   20.3575665   -4.5251099
H    -2.6697662   20.9238371   -3.5780054
C    -6.4377716   17.2308668    1.1938400
H    -7.1570970   16.6334723    1.7700343
H    -5.8326868   17.8054641    1.9095944
H    -5.2264955   23.5183364   -0.7820595
H    -3.5009591   23.3062034   -1.0818803
C    -1.8742136   19.2851738    0.6504671
N    -3.1392568   18.5561858    0.7466058
H    -1.9688098   20.0998562   -0.0827303
H    -1.0089417   18.6695977    0.3418573
H    -1.6229071   19.7571558    1.6123003
H    -3.0897945   17.8877873    1.5145886

SCF energy GEOOPT =  -7352.112926291 H
ZPE =   3755. kJ/mol
FREEH energy =  3941.71 kJ/mol
FREEH entropy =   1.59813 kJ/mol/K

$vibrational spectrum
#  mode     symmetry     wave number   IR intensity    selection rules
#                         cm**(-1)        km/mol          IR     RAMAN
     1                       -0.00         0.00000         -       -
     2                       -0.00         0.00000         -       -
```

3		0.00	0.00000	-	-
4		0.00	0.00000	-	-
5		0.00	0.00000	-	-
6		0.00	0.00000	-	-
7	a	13.06	0.01524	YES	YES
8	a	19.06	0.01838	YES	YES
9	a	19.55	0.05192	YES	YES
10	a	25.69	0.10762	YES	YES
11	a	28.30	0.05892	YES	YES
12	a	30.60	0.00777	YES	YES
13	a	31.73	0.03825	YES	YES
14	a	34.64	0.01312	YES	YES
15	a	35.70	0.02247	YES	YES
16	a	40.07	0.04126	YES	YES
17	a	42.88	0.03613	YES	YES
18	a	43.74	0.02572	YES	YES
19	a	46.25	0.14204	YES	YES
20	a	48.50	0.17445	YES	YES
21	a	52.05	0.04204	YES	YES
22	a	55.25	0.01713	YES	YES
23	a	56.84	0.26223	YES	YES
24	a	60.28	0.31486	YES	YES
25	a	63.98	0.06028	YES	YES
26	a	65.74	0.08230	YES	YES
27	a	68.04	0.18140	YES	YES
28	a	68.94	0.04797	YES	YES
29	a	72.79	0.10657	YES	YES
30	a	77.08	0.08317	YES	YES
31	a	77.31	0.00403	YES	YES
32	a	86.62	0.69970	YES	YES
33	a	88.07	0.26467	YES	YES
34	a	93.16	0.21131	YES	YES
35	a	99.60	0.54662	YES	YES
36	a	104.39	0.46239	YES	YES
37	a	109.51	0.87505	YES	YES
38	a	113.06	0.44949	YES	YES
39	a	121.54	0.16880	YES	YES
40	a	128.96	0.90864	YES	YES
41	a	134.26	0.58806	YES	YES
42	a	135.07	1.98248	YES	YES
43	a	138.29	0.35899	YES	YES
44	a	140.27	0.11306	YES	YES
45	a	142.80	0.22085	YES	YES
46	a	155.10	1.09477	YES	YES
47	a	157.03	3.23679	YES	YES
48	a	164.56	0.99407	YES	YES
49	a	168.86	0.28709	YES	YES
50	a	173.80	0.68491	YES	YES
51	a	184.68	4.30860	YES	YES
52	a	187.42	8.19623	YES	YES
53	a	189.39	0.35808	YES	YES
54	a	195.42	0.20854	YES	YES
55	a	206.98	0.22842	YES	YES
56	a	211.18	0.61002	YES	YES
57	a	216.15	0.41162	YES	YES
58	a	219.40	0.01412	YES	YES
59	a	221.63	0.09547	YES	YES
60	a	224.00	0.80961	YES	YES

61	a	225.66	0.28437	YES	YES
62	a	227.12	0.23839	YES	YES
63	a	229.72	0.37811	YES	YES
64	a	231.73	0.74709	YES	YES
65	a	233.73	7.88959	YES	YES
66	a	235.65	0.07567	YES	YES
67	a	238.49	3.15040	YES	YES
68	a	239.30	2.29245	YES	YES
69	a	242.81	3.99481	YES	YES
70	a	245.45	0.65035	YES	YES
71	a	264.90	4.77958	YES	YES
72	a	280.92	0.92344	YES	YES
73	a	284.13	0.39886	YES	YES
74	a	286.44	0.39698	YES	YES
75	a	290.49	0.04329	YES	YES
76	a	301.69	3.46518	YES	YES
77	a	305.04	1.20697	YES	YES
78	a	311.15	0.06724	YES	YES
79	a	313.78	0.61086	YES	YES
80	a	329.76	0.86536	YES	YES
81	a	330.40	0.07368	YES	YES
82	a	334.57	0.37232	YES	YES
83	a	338.14	0.40888	YES	YES
84	a	338.76	0.87022	YES	YES
85	a	339.23	1.86346	YES	YES
86	a	370.12	6.79023	YES	YES
87	a	379.81	3.28856	YES	YES
88	a	380.99	0.96305	YES	YES
89	a	382.90	0.32559	YES	YES
90	a	394.97	0.57025	YES	YES
91	a	397.42	1.64887	YES	YES
92	a	406.27	0.18389	YES	YES
93	a	411.47	1.02712	YES	YES
94	a	418.86	12.63003	YES	YES
95	a	420.33	1.73457	YES	YES
96	a	424.60	14.02239	YES	YES
97	a	425.53	11.48983	YES	YES
98	a	427.97	1.59789	YES	YES
99	a	430.34	0.11775	YES	YES
100	a	430.59	0.64365	YES	YES
101	a	431.15	0.68704	YES	YES
102	a	432.02	0.58134	YES	YES
103	a	433.57	2.69814	YES	YES
104	a	434.21	2.72091	YES	YES
105	a	435.00	1.55539	YES	YES
106	a	437.75	0.03462	YES	YES
107	a	445.21	2.91807	YES	YES
108	a	459.51	3.58027	YES	YES
109	a	459.84	4.81412	YES	YES
110	a	472.37	3.15435	YES	YES
111	a	490.79	20.97163	YES	YES
112	a	492.73	3.97891	YES	YES
113	a	496.32	1.50305	YES	YES
114	a	497.64	5.35982	YES	YES
115	a	499.39	1.11598	YES	YES
116	a	505.47	1.56171	YES	YES
117	a	511.18	17.84261	YES	YES
118	a	513.18	4.76922	YES	YES

119	a	522.53	73.39220	YES	YES
120	a	568.13	18.71127	YES	YES
121	a	595.51	92.34724	YES	YES
122	a	629.75	15.07885	YES	YES
123	a	633.16	25.66810	YES	YES
124	a	644.17	13.13525	YES	YES
125	a	662.50	12.55148	YES	YES
126	a	702.52	1.27118	YES	YES
127	a	718.68	1.90348	YES	YES
128	a	720.54	1.53852	YES	YES
129	a	731.71	11.97592	YES	YES
130	a	734.31	3.03793	YES	YES
131	a	734.62	4.46768	YES	YES
132	a	737.09	0.45953	YES	YES
133	a	739.97	4.73044	YES	YES
134	a	771.23	1.26002	YES	YES
135	a	773.71	0.19052	YES	YES
136	a	774.23	0.88793	YES	YES
137	a	774.50	1.01344	YES	YES
138	a	775.18	0.29386	YES	YES
139	a	775.67	0.30158	YES	YES
140	a	776.10	0.82998	YES	YES
141	a	776.88	0.10653	YES	YES
142	a	778.31	13.49558	YES	YES
143	a	786.79	14.34061	YES	YES
144	a	806.99	0.44905	YES	YES
145	a	809.94	1.78057	YES	YES
146	a	810.47	7.45756	YES	YES
147	a	811.48	7.63385	YES	YES
148	a	812.32	1.42424	YES	YES
149	a	813.61	1.81123	YES	YES
150	a	814.72	2.64992	YES	YES
151	a	818.28	2.28661	YES	YES
152	a	832.94	2.66903	YES	YES
153	a	833.37	8.69537	YES	YES
154	a	836.43	7.41936	YES	YES
155	a	837.91	1.65029	YES	YES
156	a	838.21	0.04859	YES	YES
157	a	838.90	4.47371	YES	YES
158	a	839.25	0.97177	YES	YES
159	a	839.90	7.53164	YES	YES
160	a	849.52	13.27580	YES	YES
161	a	854.77	13.32904	YES	YES
162	a	872.06	3.23690	YES	YES
163	a	874.13	1.37229	YES	YES
164	a	874.53	2.08645	YES	YES
165	a	876.51	1.83721	YES	YES
166	a	876.91	6.06761	YES	YES
167	a	877.84	1.37480	YES	YES
168	a	878.94	2.18887	YES	YES
169	a	879.51	5.74080	YES	YES
170	a	879.82	0.80946	YES	YES
171	a	880.06	2.03234	YES	YES
172	a	881.04	3.97037	YES	YES
173	a	881.92	3.29492	YES	YES
174	a	883.85	6.34410	YES	YES
175	a	884.29	2.41294	YES	YES
176	a	886.41	1.04518	YES	YES

177	a	887.55	6.41196	YES	YES
178	a	904.33	1.71074	YES	YES
179	a	905.09	2.00442	YES	YES
180	a	905.24	1.55310	YES	YES
181	a	905.57	2.05072	YES	YES
182	a	908.08	0.90642	YES	YES
183	a	909.72	1.28325	YES	YES
184	a	910.14	0.76230	YES	YES
185	a	911.28	0.89241	YES	YES
186	a	981.25	1.19497	YES	YES
187	a	984.36	0.45311	YES	YES
188	a	988.49	9.34219	YES	YES
189	a	988.77	1.35521	YES	YES
190	a	989.12	16.66683	YES	YES
191	a	989.60	14.37203	YES	YES
192	a	990.46	0.34479	YES	YES
193	a	990.79	17.49661	YES	YES
194	a	991.67	2.09873	YES	YES
195	a	992.18	11.97436	YES	YES
196	a	1013.84	0.33531	YES	YES
197	a	1015.79	0.63664	YES	YES
198	a	1016.98	0.19861	YES	YES
199	a	1017.33	0.80454	YES	YES
200	a	1017.95	0.88921	YES	YES
201	a	1019.09	0.05453	YES	YES
202	a	1019.50	1.00388	YES	YES
203	a	1022.23	0.44538	YES	YES
204	a	1030.54	0.92882	YES	YES
205	a	1032.09	2.38855	YES	YES
206	a	1032.14	0.38835	YES	YES
207	a	1033.77	0.94635	YES	YES
208	a	1037.48	2.65938	YES	YES
209	a	1039.36	2.72853	YES	YES
210	a	1039.99	0.63515	YES	YES
211	a	1040.98	3.12839	YES	YES
212	a	1043.36	9.60030	YES	YES
213	a	1054.66	54.07545	YES	YES
214	a	1057.87	4.07489	YES	YES
215	a	1058.74	2.74592	YES	YES
216	a	1059.26	1.80939	YES	YES
217	a	1061.06	1.76248	YES	YES
218	a	1061.76	1.37713	YES	YES
219	a	1064.17	1.97821	YES	YES
220	a	1065.55	0.48356	YES	YES
221	a	1068.16	1.33097	YES	YES
222	a	1070.21	0.01486	YES	YES
223	a	1071.23	0.20751	YES	YES
224	a	1071.94	0.22735	YES	YES
225	a	1072.30	0.10028	YES	YES
226	a	1072.78	0.11212	YES	YES
227	a	1073.46	0.21152	YES	YES
228	a	1073.64	0.12646	YES	YES
229	a	1074.45	0.02735	YES	YES
230	a	1081.15	2.14223	YES	YES
231	a	1083.17	3.32355	YES	YES
232	a	1091.91	0.55531	YES	YES
233	a	1094.67	3.73907	YES	YES
234	a	1094.80	6.97107	YES	YES

235	a	1095.25	3.26076	YES	YES
236	a	1096.84	6.09489	YES	YES
237	a	1101.43	1.62336	YES	YES
238	a	1104.63	1.88736	YES	YES
239	a	1107.84	9.35838	YES	YES
240	a	1115.26	6.81823	YES	YES
241	a	1122.38	1.02380	YES	YES
242	a	1130.93	2.16160	YES	YES
243	a	1159.70	9.53899	YES	YES
244	a	1160.93	0.95784	YES	YES
245	a	1162.01	14.11315	YES	YES
246	a	1166.03	8.34633	YES	YES
247	a	1168.00	6.23658	YES	YES
248	a	1168.89	8.61962	YES	YES
249	a	1169.81	11.26014	YES	YES
250	a	1170.95	2.66975	YES	YES
251	a	1178.30	11.12916	YES	YES
252	a	1178.92	11.41817	YES	YES
253	a	1180.88	4.22772	YES	YES
254	a	1181.50	5.05089	YES	YES
255	a	1187.28	0.61978	YES	YES
256	a	1189.22	1.13851	YES	YES
257	a	1193.19	1.87705	YES	YES
258	a	1211.43	1.73404	YES	YES
259	a	1239.63	0.10810	YES	YES
260	a	1241.74	0.33125	YES	YES
261	a	1244.43	0.47552	YES	YES
262	a	1245.89	0.37637	YES	YES
263	a	1246.35	0.06283	YES	YES
264	a	1247.11	2.38286	YES	YES
265	a	1247.76	0.27426	YES	YES
266	a	1248.06	1.12308	YES	YES
267	a	1250.27	0.47844	YES	YES
268	a	1251.31	0.05074	YES	YES
269	a	1253.01	0.96306	YES	YES
270	a	1254.31	1.55424	YES	YES
271	a	1254.72	6.87572	YES	YES
272	a	1255.55	3.46171	YES	YES
273	a	1256.68	2.87635	YES	YES
274	a	1257.04	4.90427	YES	YES
275	a	1257.64	4.52340	YES	YES
276	a	1258.00	1.55423	YES	YES
277	a	1259.30	3.38612	YES	YES
278	a	1261.51	1.36578	YES	YES
279	a	1261.77	1.37095	YES	YES
280	a	1262.90	1.16881	YES	YES
281	a	1264.13	0.99827	YES	YES
282	a	1264.24	0.69482	YES	YES
283	a	1266.50	0.72751	YES	YES
284	a	1267.74	0.66453	YES	YES
285	a	1270.77	1.67145	YES	YES
286	a	1272.46	1.68568	YES	YES
287	a	1282.07	2.09707	YES	YES
288	a	1282.53	1.06509	YES	YES
289	a	1283.96	4.57174	YES	YES
290	a	1285.33	2.58965	YES	YES
291	a	1286.34	3.28989	YES	YES
292	a	1288.79	4.24545	YES	YES

293	a	1290.19	3.08198	YES	YES
294	a	1293.93	3.38999	YES	YES
295	a	1305.98	0.84565	YES	YES
296	a	1309.27	0.62128	YES	YES
297	a	1310.24	0.26226	YES	YES
298	a	1312.34	0.33847	YES	YES
299	a	1312.81	0.85381	YES	YES
300	a	1313.39	0.61040	YES	YES
301	a	1316.55	2.29768	YES	YES
302	a	1317.69	1.36559	YES	YES
303	a	1319.17	1.66090	YES	YES
304	a	1320.65	1.89860	YES	YES
305	a	1322.33	0.96815	YES	YES
306	a	1322.62	1.07910	YES	YES
307	a	1322.80	0.02660	YES	YES
308	a	1323.99	0.79367	YES	YES
309	a	1324.24	1.75199	YES	YES
310	a	1324.35	0.55871	YES	YES
311	a	1324.85	0.14429	YES	YES
312	a	1325.22	0.66297	YES	YES
313	a	1325.57	5.41296	YES	YES
314	a	1327.82	2.47051	YES	YES
315	a	1329.98	1.00818	YES	YES
316	a	1330.47	7.45585	YES	YES
317	a	1330.65	3.80282	YES	YES
318	a	1334.01	4.41345	YES	YES
319	a	1336.53	0.77798	YES	YES
320	a	1336.71	0.31791	YES	YES
321	a	1336.83	0.13001	YES	YES
322	a	1337.33	0.34577	YES	YES
323	a	1337.87	0.52665	YES	YES
324	a	1338.53	0.26582	YES	YES
325	a	1338.75	0.09483	YES	YES
326	a	1339.44	0.40676	YES	YES
327	a	1339.90	4.87912	YES	YES
328	a	1344.65	1.00302	YES	YES
329	a	1345.63	0.47804	YES	YES
330	a	1345.87	0.66566	YES	YES
331	a	1345.98	1.33690	YES	YES
332	a	1346.65	0.71212	YES	YES
333	a	1347.98	2.56569	YES	YES
334	a	1348.26	1.14088	YES	YES
335	a	1350.49	1.16860	YES	YES
336	a	1399.34	6.19625	YES	YES
337	a	1404.85	6.15291	YES	YES
338	a	1405.92	8.16714	YES	YES
339	a	1406.74	9.03977	YES	YES
340	a	1408.17	9.09583	YES	YES
341	a	1424.79	3.97839	YES	YES
342	a	1426.76	3.48544	YES	YES
343	a	1427.66	3.46430	YES	YES
344	a	1430.83	0.44813	YES	YES
345	a	1431.80	6.44166	YES	YES
346	a	1432.54	0.07969	YES	YES
347	a	1433.19	9.01860	YES	YES
348	a	1433.82	2.14040	YES	YES
349	a	1434.55	1.02597	YES	YES
350	a	1435.90	3.15967	YES	YES

351	a	1436.05	8.91439	YES	YES
352	a	1436.64	3.04412	YES	YES
353	a	1437.08	7.29205	YES	YES
354	a	1437.94	1.81063	YES	YES
355	a	1437.96	3.04950	YES	YES
356	a	1438.23	2.78077	YES	YES
357	a	1438.43	5.42380	YES	YES
358	a	1438.62	8.63649	YES	YES
359	a	1438.99	5.43230	YES	YES
360	a	1439.58	2.93505	YES	YES
361	a	1439.70	4.69702	YES	YES
362	a	1439.81	11.29393	YES	YES
363	a	1440.17	2.62606	YES	YES
364	a	1440.93	5.47087	YES	YES
365	a	1441.65	10.53512	YES	YES
366	a	1443.09	4.09481	YES	YES
367	a	1443.53	5.08206	YES	YES
368	a	1444.39	22.25218	YES	YES
369	a	1444.96	20.06291	YES	YES
370	a	1445.22	23.14111	YES	YES
371	a	1446.50	3.88085	YES	YES
372	a	1446.80	17.91878	YES	YES
373	a	1448.75	8.62222	YES	YES
374	a	1452.73	1.21825	YES	YES
375	a	1454.39	0.56307	YES	YES
376	a	1454.90	2.73928	YES	YES
377	a	1455.17	5.55823	YES	YES
378	a	1456.17	1.85819	YES	YES
379	a	1456.75	0.94432	YES	YES
380	a	1457.60	3.32125	YES	YES
381	a	1462.64	3.46762	YES	YES
382	a	1466.01	9.20408	YES	YES
383	a	1833.13	133.87607	YES	YES
384	a	2893.70	71.75160	YES	YES
385	a	2911.59	4.59087	YES	YES
386	a	2930.76	34.00797	YES	YES
387	a	2931.80	2.26216	YES	YES
388	a	2936.14	16.76775	YES	YES
389	a	2938.38	11.47690	YES	YES
390	a	2938.97	2.88728	YES	YES
391	a	2939.57	6.09448	YES	YES
392	a	2940.16	8.67621	YES	YES
393	a	2940.57	21.75679	YES	YES
394	a	2940.86	5.59534	YES	YES
395	a	2941.91	18.98209	YES	YES
396	a	2943.31	8.07263	YES	YES
397	a	2944.31	33.34274	YES	YES
398	a	2944.47	5.00342	YES	YES
399	a	2945.05	7.58088	YES	YES
400	a	2945.30	31.11434	YES	YES
401	a	2945.51	18.10188	YES	YES
402	a	2946.72	17.97027	YES	YES
403	a	2946.73	13.22332	YES	YES
404	a	2946.96	11.32714	YES	YES
405	a	2949.36	0.60755	YES	YES
406	a	2949.93	8.64906	YES	YES
407	a	2950.81	15.33211	YES	YES
408	a	2951.87	13.48180	YES	YES

409	a	2952.28	17.86836	YES	YES
410	a	2952.59	5.55290	YES	YES
411	a	2953.07	23.94268	YES	YES
412	a	2953.59	2.77639	YES	YES
413	a	2953.88	0.14990	YES	YES
414	a	2954.49	1.94244	YES	YES
415	a	2955.41	7.34431	YES	YES
416	a	2956.23	4.82400	YES	YES
417	a	2956.70	3.27267	YES	YES
418	a	2957.41	12.22944	YES	YES
419	a	2957.76	6.65628	YES	YES
420	a	2958.66	35.14979	YES	YES
421	a	2958.72	10.14928	YES	YES
422	a	2959.24	24.02076	YES	YES
423	a	2960.04	33.92284	YES	YES
424	a	2960.84	37.50457	YES	YES
425	a	2961.58	39.97150	YES	YES
426	a	2962.08	14.12875	YES	YES
427	a	2962.19	33.50803	YES	YES
428	a	2962.77	31.51597	YES	YES
429	a	2964.93	18.90694	YES	YES
430	a	2967.33	4.36853	YES	YES
431	a	2968.06	29.04247	YES	YES
432	a	2968.49	17.22373	YES	YES
433	a	2975.67	11.35782	YES	YES
434	a	2981.13	20.22040	YES	YES
435	a	2982.38	5.52959	YES	YES
436	a	2986.29	28.32497	YES	YES
437	a	2987.41	8.18373	YES	YES
438	a	2987.52	5.87200	YES	YES
439	a	2990.60	7.56805	YES	YES
440	a	2991.11	23.05483	YES	YES
441	a	2993.04	12.86274	YES	YES
442	a	2994.10	37.48991	YES	YES
443	a	2997.07	23.83709	YES	YES
444	a	2997.75	32.49980	YES	YES
445	a	2998.98	27.20066	YES	YES
446	a	3000.01	14.03837	YES	YES
447	a	3001.09	30.02644	YES	YES
448	a	3002.17	18.88108	YES	YES
449	a	3002.71	19.22932	YES	YES
450	a	3002.78	11.00817	YES	YES
451	a	3003.82	32.87980	YES	YES
452	a	3003.85	28.42400	YES	YES
453	a	3007.63	2.39541	YES	YES
454	a	3008.59	37.24642	YES	YES
455	a	3009.13	20.68405	YES	YES
456	a	3011.93	12.06375	YES	YES
457	a	3012.08	21.26029	YES	YES
458	a	3013.25	29.09458	YES	YES
459	a	3013.46	7.19991	YES	YES
460	a	3013.59	42.62319	YES	YES
461	a	3013.81	17.59633	YES	YES
462	a	3014.86	16.76990	YES	YES
463	a	3014.92	13.17261	YES	YES
464	a	3015.80	20.96909	YES	YES
465	a	3016.37	26.00309	YES	YES
466	a	3016.73	15.94471	YES	YES

467	a	3016.76	24.97975	YES	YES
468	a	3017.43	27.39311	YES	YES
469	a	3017.54	19.66513	YES	YES
470	a	3017.74	34.92666	YES	YES
471	a	3017.95	14.82439	YES	YES
472	a	3018.18	31.86835	YES	YES
473	a	3018.87	27.18620	YES	YES
474	a	3018.96	26.34761	YES	YES
475	a	3019.03	23.31033	YES	YES
476	a	3019.38	23.73948	YES	YES
477	a	3020.58	28.07251	YES	YES
478	a	3022.74	28.99436	YES	YES
479	a	3030.82	1.32532	YES	YES
480	a	3033.11	0.41347	YES	YES
481	a	3038.34	0.91511	YES	YES
482	a	3047.22	0.35003	YES	YES
483	a	3449.44	1.85490	YES	YES

$end

Double hybrid single point energy = -7344.826402651739 H
COSMO energy + OC correction = -7352.2602829504 H (in *o*DFB)

6.2.34 [H{Ga(dcpe)}$_2$(NHMe)]$^{2+}$ (*anti*-periplanar)

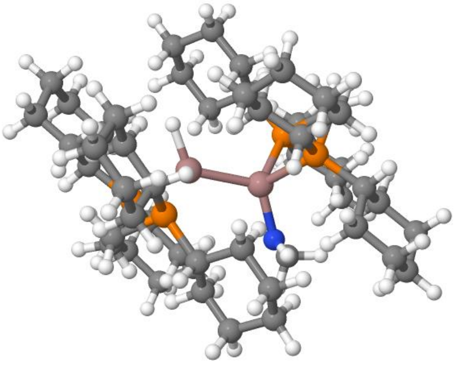

Method: (RI-)BP86(D3BJ)/def2-TZVPP
Symmetry: c1

Cartesian coordinates in Ångström:

Ga	-0.4142791	0.4626268	-1.8559953
P	-2.2574730	-0.7467801	-2.9294231
P	-1.7697999	2.4803512	-2.2749942
C	-1.8506332	-1.8721661	-4.3480381
H	-0.9534140	-2.4132043	-3.9983810
C	-1.4619962	-1.0745551	-5.6051027
H	-2.3553211	-0.5621491	-5.9958337
H	-0.7244845	-0.2983691	-5.3668262
C	-0.9300745	-2.0169808	-6.6908169
H	-0.6568640	-1.4353538	-7.5817969
H	-0.0048332	-2.4976031	-6.3321448
C	-1.9704530	-3.0848598	-7.0429163
H	-1.5636219	-3.7843039	-7.7852075
H	-2.8397794	-2.5996748	-7.5161522
C	-2.4313797	-3.8486011	-5.7979351
H	-1.5920453	-4.4427376	-5.3996540
H	-3.2241348	-4.5638566	-6.0556955

C	-2.9353300	-2.9079024	-4.6917324
H	-3.2154634	-3.5008173	-3.8113147
H	-3.8445275	-2.3857273	-5.0319074
C	-3.2614481	-1.7334192	-1.7399249
H	-4.0566563	-2.1848909	-2.3579563
C	-2.4292057	-2.8630459	-1.1152232
H	-1.9561027	-3.4791218	-1.8939366
H	-1.6109674	-2.4138157	-0.5256858
C	-3.3071340	-3.7333372	-0.2075081
H	-4.0671033	-4.2390593	-0.8247264
H	-2.7010526	-4.5288919	0.2457868
C	-4.0015593	-2.8964550	0.8713956
H	-4.6536259	-3.5331659	1.4840341
H	-3.2459706	-2.4722923	1.5532632
C	-4.8061050	-1.7495259	0.2559057
H	-5.2528571	-1.1268500	1.0430801
H	-5.6430676	-2.1602180	-0.3316399
C	-3.9365889	-0.8755744	-0.6575729
H	-4.5657686	-0.1028217	-1.1188621
H	-3.1664248	-0.3630714	-0.0584750
C	-3.4156278	0.5430792	-3.5756119
H	-3.1020499	0.7466382	-4.6053898
H	-4.4261850	0.1132866	-3.6297833
C	-3.4375812	1.8483161	-2.7641869
H	-3.9924825	1.7103339	-1.8277335
H	-3.9592954	2.6316488	-3.3313735
C	-1.1869750	3.5312379	-3.6789841
H	-1.9979287	4.2518821	-3.8806552
C	0.0759476	4.3019428	-3.2647772
H	0.8359001	3.5792660	-2.9270041
H	-0.1392163	4.9565683	-2.4088047
C	0.6540383	5.1220746	-4.4223024
H	-0.0535620	5.9232667	-4.6895986
H	1.5769375	5.6184770	-4.0912843
C	0.9184905	4.2442238	-5.6474466
H	1.6944323	3.4980788	-5.4044912
H	1.3155865	4.8502927	-6.4722276
C	-0.3606525	3.5262322	-6.0833851
H	-0.1676383	2.8779513	-6.9491815
H	-1.1046724	4.2708830	-6.4094204
C	-0.9485408	2.6859305	-4.9435727
H	-0.2562145	1.8661910	-4.6948807
H	-1.8890551	2.2263174	-5.2757961
C	-2.0751332	3.6099508	-0.8467360
H	-1.0822857	4.0404939	-0.6320516
C	-2.5224091	2.8295641	0.3993673
H	-1.7962433	2.0377680	0.6371917
H	-3.4795019	2.3208402	0.2005063
C	-2.6992349	3.7690607	1.5973335
H	-3.0451680	3.1942621	2.4675621
H	-1.7171794	4.1931603	1.8685643
C	-3.6667618	4.9125167	1.2798613
H	-4.6746064	4.4998980	1.1087340
H	-3.7502928	5.5895223	2.1404999
C	-3.2149731	5.6861948	0.0381120
H	-2.2544700	6.1866442	0.2450900
H	-3.9338078	6.4797894	-0.2058138
C	-3.0458129	4.7585657	-1.1730382

H	-4.0275793	4.3404715	-1.4476057
H	-2.6899037	5.3335688	-2.0390224
Ga	-0.1569887	-0.1000656	0.5293830
P	1.4328812	1.3085398	1.7029803
P	1.2734177	-1.9484945	1.1713810
C	0.7040265	2.4180787	2.9885253
H	0.0906227	3.1289404	2.4077706
C	-0.2398004	1.6454394	3.9271446
H	0.3325793	0.8766422	4.4712954
H	-1.0047759	1.1157198	3.3424451
C	-0.8911561	2.5885866	4.9467892
H	-1.5312640	2.0073749	5.6246597
H	-1.5525892	3.2916999	4.4152437
C	0.1581550	3.3762816	5.7356145
H	-0.3313085	4.0682374	6.4338103
H	0.7526024	2.6809676	6.3511432
C	1.0897287	4.1448831	4.7950968
H	0.5133770	4.9126531	4.2525308
H	1.8626341	4.6787888	5.3642855
C	1.7605903	3.2115550	3.7773003
H	2.3970317	3.7971099	3.0996425
H	2.4212963	2.5090083	4.3108961
C	2.5419371	2.3507187	0.6648930
H	3.2249917	2.8439465	1.3778842
C	1.7460919	3.4344918	-0.0757458
H	1.1628275	4.0426210	0.6310092
H	1.0193267	2.9406861	-0.7448697
C	2.6823087	4.3313365	-0.8942727
H	3.3423403	4.8800735	-0.2037073
H	2.0983181	5.0916248	-1.4300547
C	3.5350630	3.5149508	-1.8689289
H	4.2288868	4.1725476	-2.4094615
H	2.8841357	3.0622172	-2.6371673
C	4.3076095	2.4091719	-1.1451408
H	4.8624715	1.7931214	-1.8658438
H	5.0590978	2.8684814	-0.4829420
C	3.3823461	1.5115178	-0.3119357
H	3.9939673	0.7850676	0.2400327
H	2.7145973	0.9424613	-0.9814496
C	2.5594750	0.1479116	2.6006154
H	2.0952540	-0.0302816	3.5784694
H	3.5150217	0.6571439	2.7884642
C	2.8029094	-1.1829405	1.8709300
H	3.4852116	-1.0348842	1.0251111
H	3.2816112	-1.9045591	2.5474035
C	0.5918766	-3.0240330	2.5075560
H	1.4127603	-3.7069150	2.7865122
C	-0.5872446	-3.8408308	1.9620496
H	-1.3442240	-3.1362679	1.5860162
H	-0.2692494	-4.4569522	1.1089017
C	-1.2181178	-4.7232160	3.0445694
H	-0.4942892	-5.4946629	3.3530122
H	-2.0815406	-5.2573573	2.6235931
C	-1.6360453	-3.8956844	4.2623967
H	-2.4355986	-3.1938834	3.9712608
H	-2.0584610	-4.5470580	5.0388069
C	-0.4474021	-3.1086445	4.8194677
H	-0.7565384	-2.4920885	5.6746601

H	0.3125086	-3.8111842	5.1982054
C	0.1836976	-2.2102850	3.7479627
H	-0.5372546	-1.4334448	3.4467821
H	1.0580542	-1.6965970	4.1712362
C	1.8087378	-3.0544550	-0.2019213
H	0.8544371	-3.4217446	-0.6206799
C	2.5325314	-2.2665065	-1.3057745
H	1.9487137	-1.3875734	-1.6190231
H	3.4867127	-1.8799319	-0.9123543
C	2.8346775	-3.1818506	-2.4989853
H	3.3680202	-2.6146838	-3.2736457
H	1.8826946	-3.5123797	-2.9494312
C	3.6514874	-4.4053836	-2.0717589
H	4.6460484	-4.0732695	-1.7312993
H	3.8234138	-5.0682254	-2.9304332
C	2.9593892	-5.1733234	-0.9420502
H	2.0187508	-5.6108232	-1.3162476
H	3.5824583	-6.0140299	-0.6085574
C	2.6410659	-4.2647743	0.2540557
H	3.5812428	-3.9092587	0.7065641
H	2.1117755	-4.8395477	1.0260038
H	-1.3883801	-0.2212259	1.5106832
N	1.1724095	0.5783629	-2.8815773
C	1.5489027	-0.1893099	-4.0600464
H	1.5297627	1.5297918	-2.9407049
H	2.6442042	-0.2849299	-4.1313303
H	1.2017534	0.2367572	-5.0200071
H	1.1463823	-1.2088625	-3.9936487

SCF energy GEOOPT = -7352.121960061 H
ZPE = 3757. kJ/mol
FREEH energy = 3943.07 kJ/mol
FREEH entropy = 1.57436 kJ/mol/K

$vibrational spectrum

# mode	symmetry	wave number	IR intensity	selection rules	
#		cm**(-1)	km/mol	IR	RAMAN
1		-0.00	0.00000	-	-
2		-0.00	0.00000	-	-
3		0.00	0.00000	-	-
4		0.00	0.00000	-	-
5		0.00	0.00000	-	-
6		0.00	0.00000	-	-
7	a	13.18	0.05696	YES	YES
8	a	18.88	0.06316	YES	YES
9	a	20.92	0.03706	YES	YES
10	a	31.27	0.07194	YES	YES
11	a	31.51	0.04055	YES	YES
12	a	34.07	0.01710	YES	YES
13	a	36.29	0.03634	YES	YES
14	a	39.02	0.11667	YES	YES
15	a	40.27	0.16775	YES	YES
16	a	44.74	0.01836	YES	YES
17	a	45.69	0.01110	YES	YES
18	a	46.83	0.05664	YES	YES
19	a	51.88	0.01390	YES	YES
20	a	52.68	0.22944	YES	YES
21	a	55.79	0.27861	YES	YES

22	a	59.95	0.04516	YES	YES
23	a	65.32	0.05601	YES	YES
24	a	66.82	0.58411	YES	YES
25	a	68.88	0.73118	YES	YES
26	a	70.04	0.05000	YES	YES
27	a	73.65	0.58385	YES	YES
28	a	75.36	0.36792	YES	YES
29	a	79.57	0.40187	YES	YES
30	a	80.94	0.10087	YES	YES
31	a	86.14	0.05058	YES	YES
32	a	87.51	0.37687	YES	YES
33	a	90.79	0.10978	YES	YES
34	a	93.09	0.48269	YES	YES
35	a	97.89	0.39707	YES	YES
36	a	99.05	0.12956	YES	YES
37	a	114.18	1.04532	YES	YES
38	a	125.44	0.54767	YES	YES
39	a	131.02	0.61563	YES	YES
40	a	134.90	3.54586	YES	YES
41	a	138.86	2.25605	YES	YES
42	a	141.47	3.46410	YES	YES
43	a	145.40	1.11562	YES	YES
44	a	149.76	4.57283	YES	YES
45	a	149.96	1.04205	YES	YES
46	a	152.57	0.36129	YES	YES
47	a	157.77	2.80824	YES	YES
48	a	168.66	2.30062	YES	YES
49	a	176.32	4.33584	YES	YES
50	a	179.56	0.83876	YES	YES
51	a	183.08	0.15312	YES	YES
52	a	188.25	0.46298	YES	YES
53	a	196.99	1.18955	YES	YES
54	a	200.00	0.90093	YES	YES
55	a	204.94	1.01368	YES	YES
56	a	206.58	6.88632	YES	YES
57	a	209.18	5.95326	YES	YES
58	a	215.85	0.22121	YES	YES
59	a	218.82	1.03447	YES	YES
60	a	222.67	0.18222	YES	YES
61	a	225.45	1.13603	YES	YES
62	a	225.91	4.37486	YES	YES
63	a	229.71	0.15653	YES	YES
64	a	237.36	0.49025	YES	YES
65	a	239.47	0.14519	YES	YES
66	a	240.97	0.21270	YES	YES
67	a	243.05	0.88357	YES	YES
68	a	245.45	0.56933	YES	YES
69	a	246.57	1.76251	YES	YES
70	a	256.98	0.48095	YES	YES
71	a	264.03	9.84299	YES	YES
72	a	275.93	2.41544	YES	YES
73	a	278.96	0.83434	YES	YES
74	a	284.32	1.82211	YES	YES
75	a	290.93	0.43303	YES	YES
76	a	305.33	0.56860	YES	YES
77	a	309.27	0.13286	YES	YES
78	a	315.87	0.22799	YES	YES
79	a	316.92	0.22867	YES	YES

80	a	331.33	1.09608	YES	YES
81	a	331.53	0.06482	YES	YES
82	a	334.01	0.11619	YES	YES
83	a	335.43	0.22498	YES	YES
84	a	344.05	0.26051	YES	YES
85	a	345.27	0.14057	YES	YES
86	a	365.62	11.14694	YES	YES
87	a	371.63	6.25412	YES	YES
88	a	374.03	1.42440	YES	YES
89	a	374.71	1.19899	YES	YES
90	a	391.74	8.06072	YES	YES
91	a	395.75	8.66266	YES	YES
92	a	402.02	23.66808	YES	YES
93	a	413.32	2.50033	YES	YES
94	a	414.20	0.77553	YES	YES
95	a	423.22	3.46773	YES	YES
96	a	423.53	3.40708	YES	YES
97	a	424.59	0.58347	YES	YES
98	a	426.51	1.25347	YES	YES
99	a	429.56	0.07507	YES	YES
100	a	430.97	0.09033	YES	YES
101	a	431.30	0.15889	YES	YES
102	a	432.56	0.32512	YES	YES
103	a	432.64	1.28103	YES	YES
104	a	433.32	0.14006	YES	YES
105	a	434.14	0.20576	YES	YES
106	a	436.12	1.91953	YES	YES
107	a	447.06	4.30747	YES	YES
108	a	449.68	2.40821	YES	YES
109	a	468.98	3.62109	YES	YES
110	a	472.39	3.17455	YES	YES
111	a	490.83	5.02148	YES	YES
112	a	492.65	0.00828	YES	YES
113	a	493.24	2.72018	YES	YES
114	a	494.60	3.28494	YES	YES
115	a	503.63	6.27547	YES	YES
116	a	506.78	0.33012	YES	YES
117	a	518.96	25.23624	YES	YES
118	a	519.59	1.10579	YES	YES
119	a	557.90	109.58800	YES	YES
120	a	559.91	24.51131	YES	YES
121	a	580.01	93.64282	YES	YES
122	a	623.93	18.34388	YES	YES
123	a	630.00	15.41247	YES	YES
124	a	641.21	7.80246	YES	YES
125	a	645.64	9.53860	YES	YES
126	a	715.54	1.00589	YES	YES
127	a	717.39	0.63783	YES	YES
128	a	722.70	1.04674	YES	YES
129	a	723.99	0.94078	YES	YES
130	a	731.14	5.07921	YES	YES
131	a	734.85	4.76378	YES	YES
132	a	735.08	11.44074	YES	YES
133	a	736.73	3.71671	YES	YES
134	a	771.93	1.08358	YES	YES
135	a	772.70	0.13946	YES	YES
136	a	772.88	0.22021	YES	YES
137	a	773.77	0.17863	YES	YES

138	a	774.23	0.75622	YES	YES
139	a	775.36	0.33160	YES	YES
140	a	775.82	0.40730	YES	YES
141	a	776.72	3.87919	YES	YES
142	a	780.32	14.34438	YES	YES
143	a	782.16	7.40993	YES	YES
144	a	808.78	0.97292	YES	YES
145	a	810.27	1.31372	YES	YES
146	a	810.96	2.07448	YES	YES
147	a	812.12	0.93073	YES	YES
148	a	812.94	0.63719	YES	YES
149	a	813.49	1.08407	YES	YES
150	a	815.90	2.59229	YES	YES
151	a	816.42	9.49127	YES	YES
152	a	833.28	0.34614	YES	YES
153	a	834.12	0.83263	YES	YES
154	a	836.52	5.28193	YES	YES
155	a	837.63	5.26132	YES	YES
156	a	837.82	2.34000	YES	YES
157	a	838.27	4.34033	YES	YES
158	a	839.06	15.61576	YES	YES
159	a	839.71	3.32589	YES	YES
160	a	851.26	11.41990	YES	YES
161	a	855.21	6.98213	YES	YES
162	a	874.82	0.57198	YES	YES
163	a	875.34	3.62131	YES	YES
164	a	875.73	9.47763	YES	YES
165	a	876.06	0.26152	YES	YES
166	a	876.94	4.52538	YES	YES
167	a	878.05	2.18530	YES	YES
168	a	878.35	2.78633	YES	YES
169	a	878.88	0.71326	YES	YES
170	a	879.08	3.63562	YES	YES
171	a	880.03	5.77626	YES	YES
172	a	880.54	3.22706	YES	YES
173	a	880.86	1.33211	YES	YES
174	a	882.24	0.39595	YES	YES
175	a	883.49	2.53666	YES	YES
176	a	886.02	4.91578	YES	YES
177	a	887.79	0.17765	YES	YES
178	a	902.96	0.62947	YES	YES
179	a	903.43	3.89479	YES	YES
180	a	904.78	1.68437	YES	YES
181	a	905.57	1.57520	YES	YES
182	a	908.64	0.66255	YES	YES
183	a	909.12	2.40653	YES	YES
184	a	910.84	1.20925	YES	YES
185	a	911.34	1.79063	YES	YES
186	a	986.91	2.23578	YES	YES
187	a	987.77	2.50933	YES	YES
188	a	988.27	7.70608	YES	YES
189	a	988.75	4.13736	YES	YES
190	a	989.75	0.38287	YES	YES
191	a	990.30	29.11852	YES	YES
192	a	991.00	24.89025	YES	YES
193	a	991.74	6.83956	YES	YES
194	a	993.49	1.09459	YES	YES
195	a	995.05	0.25206	YES	YES

196	a	1014.26	1.49383	YES	YES
197	a	1016.94	0.52609	YES	YES
198	a	1017.35	0.98510	YES	YES
199	a	1017.71	0.29060	YES	YES
200	a	1017.85	0.58544	YES	YES
201	a	1020.48	0.94291	YES	YES
202	a	1021.68	0.58413	YES	YES
203	a	1022.92	0.87809	YES	YES
204	a	1030.00	2.63012	YES	YES
205	a	1031.76	2.02435	YES	YES
206	a	1032.18	0.42416	YES	YES
207	a	1033.28	3.00082	YES	YES
208	a	1038.99	2.95138	YES	YES
209	a	1039.82	2.04481	YES	YES
210	a	1040.64	0.31976	YES	YES
211	a	1041.39	1.36733	YES	YES
212	a	1049.44	4.23801	YES	YES
213	a	1058.45	1.55275	YES	YES
214	a	1059.98	3.60312	YES	YES
215	a	1060.65	1.06778	YES	YES
216	a	1061.69	0.72970	YES	YES
217	a	1064.10	3.54344	YES	YES
218	a	1066.35	0.67717	YES	YES
219	a	1067.83	0.41798	YES	YES
220	a	1069.15	0.52407	YES	YES
221	a	1070.95	0.22540	YES	YES
222	a	1071.57	0.11172	YES	YES
223	a	1072.29	0.18734	YES	YES
224	a	1072.86	0.11764	YES	YES
225	a	1073.81	0.15777	YES	YES
226	a	1075.99	0.31293	YES	YES
227	a	1076.35	0.06327	YES	YES
228	a	1076.74	0.26887	YES	YES
229	a	1079.47	49.79106	YES	YES
230	a	1085.09	4.34676	YES	YES
231	a	1085.89	8.71019	YES	YES
232	a	1092.09	5.74019	YES	YES
233	a	1096.18	8.46435	YES	YES
234	a	1096.48	11.83959	YES	YES
235	a	1098.11	5.51692	YES	YES
236	a	1100.49	1.67294	YES	YES
237	a	1101.74	0.52782	YES	YES
238	a	1102.45	3.51342	YES	YES
239	a	1107.01	1.09751	YES	YES
240	a	1107.68	16.38346	YES	YES
241	a	1132.89	4.97365	YES	YES
242	a	1135.18	1.01760	YES	YES
243	a	1159.36	4.67753	YES	YES
244	a	1160.40	4.77478	YES	YES
245	a	1160.83	6.05294	YES	YES
246	a	1161.91	4.50430	YES	YES
247	a	1169.85	3.21983	YES	YES
248	a	1170.54	0.88851	YES	YES
249	a	1171.07	19.35950	YES	YES
250	a	1172.04	9.53170	YES	YES
251	a	1175.88	14.41953	YES	YES
252	a	1179.68	9.39865	YES	YES
253	a	1185.73	3.96394	YES	YES

254	a	1186.92	10.10942	YES	YES
255	a	1188.97	3.82505	YES	YES
256	a	1191.08	0.26614	YES	YES
257	a	1193.29	2.76085	YES	YES
258	a	1197.07	1.72009	YES	YES
259	a	1242.25	0.10378	YES	YES
260	a	1242.61	0.10476	YES	YES
261	a	1244.10	0.11191	YES	YES
262	a	1244.46	1.21337	YES	YES
263	a	1245.14	0.55798	YES	YES
264	a	1245.83	0.88716	YES	YES
265	a	1247.10	0.17938	YES	YES
266	a	1247.48	0.41091	YES	YES
267	a	1249.75	0.21608	YES	YES
268	a	1250.61	0.20803	YES	YES
269	a	1254.92	1.43007	YES	YES
270	a	1256.37	3.44881	YES	YES
271	a	1256.99	5.82021	YES	YES
272	a	1257.46	2.68309	YES	YES
273	a	1257.73	1.69063	YES	YES
274	a	1257.89	3.21610	YES	YES
275	a	1258.51	3.60833	YES	YES
276	a	1259.19	4.05894	YES	YES
277	a	1259.56	3.19429	YES	YES
278	a	1259.95	2.90055	YES	YES
279	a	1260.14	8.92403	YES	YES
280	a	1260.39	0.08837	YES	YES
281	a	1262.18	4.10683	YES	YES
282	a	1263.53	2.12841	YES	YES
283	a	1265.66	2.63314	YES	YES
284	a	1268.22	1.07840	YES	YES
285	a	1269.31	1.14326	YES	YES
286	a	1271.71	0.28337	YES	YES
287	a	1281.25	2.95741	YES	YES
288	a	1283.62	1.72921	YES	YES
289	a	1283.67	2.20628	YES	YES
290	a	1285.79	4.83743	YES	YES
291	a	1289.43	4.59400	YES	YES
292	a	1290.22	1.45543	YES	YES
293	a	1291.06	2.05548	YES	YES
294	a	1291.34	5.08737	YES	YES
295	a	1307.36	3.85204	YES	YES
296	a	1310.21	0.16558	YES	YES
297	a	1310.75	2.37981	YES	YES
298	a	1311.47	1.00567	YES	YES
299	a	1312.74	2.79435	YES	YES
300	a	1313.62	0.53697	YES	YES
301	a	1315.14	2.97121	YES	YES
302	a	1318.95	1.51285	YES	YES
303	a	1321.74	0.55630	YES	YES
304	a	1322.12	1.02250	YES	YES
305	a	1322.55	4.08926	YES	YES
306	a	1323.36	0.14761	YES	YES
307	a	1323.58	0.70979	YES	YES
308	a	1323.96	1.15996	YES	YES
309	a	1324.98	1.08925	YES	YES
310	a	1325.25	0.38232	YES	YES
311	a	1325.84	0.03511	YES	YES

312	a	1326.19	0.40371	YES	YES
313	a	1326.76	3.88293	YES	YES
314	a	1327.18	5.17627	YES	YES
315	a	1327.76	4.10502	YES	YES
316	a	1328.96	1.85044	YES	YES
317	a	1329.98	3.80078	YES	YES
318	a	1333.78	0.68120	YES	YES
319	a	1336.01	0.21863	YES	YES
320	a	1337.45	0.59458	YES	YES
321	a	1337.72	1.82100	YES	YES
322	a	1337.98	0.33205	YES	YES
323	a	1338.65	0.23739	YES	YES
324	a	1339.09	0.83313	YES	YES
325	a	1340.25	0.18846	YES	YES
326	a	1342.31	0.46625	YES	YES
327	a	1345.29	2.76383	YES	YES
328	a	1347.11	5.05841	YES	YES
329	a	1347.27	0.51000	YES	YES
330	a	1348.13	2.30226	YES	YES
331	a	1348.28	0.68133	YES	YES
332	a	1349.40	0.94815	YES	YES
333	a	1349.62	0.62961	YES	YES
334	a	1350.70	0.22653	YES	YES
335	a	1351.00	1.26936	YES	YES
336	a	1400.65	7.27909	YES	YES
337	a	1402.46	6.71721	YES	YES
338	a	1405.28	9.35745	YES	YES
339	a	1407.22	7.51114	YES	YES
340	a	1412.39	7.91367	YES	YES
341	a	1423.49	2.86220	YES	YES
342	a	1426.31	6.70967	YES	YES
343	a	1427.52	5.85706	YES	YES
344	a	1427.80	4.77295	YES	YES
345	a	1429.27	4.13259	YES	YES
346	a	1429.82	7.15112	YES	YES
347	a	1433.03	2.83753	YES	YES
348	a	1434.21	1.50307	YES	YES
349	a	1434.95	10.12951	YES	YES
350	a	1436.24	2.61546	YES	YES
351	a	1436.48	2.52136	YES	YES
352	a	1437.22	1.70915	YES	YES
353	a	1437.55	6.44797	YES	YES
354	a	1437.87	1.32266	YES	YES
355	a	1437.99	3.79083	YES	YES
356	a	1438.37	6.38451	YES	YES
357	a	1438.62	5.74157	YES	YES
358	a	1439.04	2.45811	YES	YES
359	a	1439.11	1.56585	YES	YES
360	a	1439.87	2.07312	YES	YES
361	a	1440.25	17.45951	YES	YES
362	a	1440.78	6.85760	YES	YES
363	a	1441.32	10.55094	YES	YES
364	a	1441.56	2.35474	YES	YES
365	a	1441.89	3.94042	YES	YES
366	a	1443.30	42.69597	YES	YES
367	a	1443.63	9.39922	YES	YES
368	a	1444.65	7.03402	YES	YES
369	a	1445.25	8.89473	YES	YES

370	a	1445.98	10.48476	YES	YES
371	a	1446.77	5.67297	YES	YES
372	a	1447.59	19.04086	YES	YES
373	a	1448.27	14.53188	YES	YES
374	a	1452.90	1.12424	YES	YES
375	a	1454.07	3.01550	YES	YES
376	a	1454.33	0.24374	YES	YES
377	a	1454.75	0.32539	YES	YES
378	a	1456.34	1.17562	YES	YES
379	a	1456.83	1.16136	YES	YES
380	a	1457.77	0.87404	YES	YES
381	a	1460.89	1.36208	YES	YES
382	a	1475.18	9.13092	YES	YES
383	a	1850.65	128.76158	YES	YES
384	a	2887.74	97.27812	YES	YES
385	a	2924.31	10.80559	YES	YES
386	a	2927.08	10.11451	YES	YES
387	a	2927.41	6.15867	YES	YES
388	a	2929.20	12.54097	YES	YES
389	a	2929.52	1.91347	YES	YES
390	a	2931.66	37.33274	YES	YES
391	a	2932.61	18.74660	YES	YES
392	a	2934.51	20.13013	YES	YES
393	a	2938.30	9.97860	YES	YES
394	a	2938.37	5.15675	YES	YES
395	a	2941.20	3.55331	YES	YES
396	a	2941.74	2.51351	YES	YES
397	a	2941.93	10.72545	YES	YES
398	a	2943.70	10.99582	YES	YES
399	a	2943.89	7.38341	YES	YES
400	a	2944.32	6.38397	YES	YES
401	a	2944.57	9.40848	YES	YES
402	a	2944.68	7.44135	YES	YES
403	a	2946.04	16.19604	YES	YES
404	a	2946.19	9.93202	YES	YES
405	a	2946.45	8.37807	YES	YES
406	a	2946.78	8.15968	YES	YES
407	a	2948.51	15.25829	YES	YES
408	a	2948.94	8.38645	YES	YES
409	a	2951.91	7.78619	YES	YES
410	a	2953.00	25.99473	YES	YES
411	a	2953.36	18.93662	YES	YES
412	a	2954.07	7.45101	YES	YES
413	a	2954.60	25.98856	YES	YES
414	a	2955.22	1.54096	YES	YES
415	a	2955.53	0.75927	YES	YES
416	a	2956.93	3.12134	YES	YES
417	a	2957.94	2.93914	YES	YES
418	a	2958.04	8.14540	YES	YES
419	a	2958.37	1.96615	YES	YES
420	a	2958.76	10.39753	YES	YES
421	a	2959.13	17.57008	YES	YES
422	a	2959.20	4.33785	YES	YES
423	a	2959.55	7.02952	YES	YES
424	a	2960.15	36.27481	YES	YES
425	a	2960.93	44.22231	YES	YES
426	a	2961.34	14.01919	YES	YES
427	a	2962.62	16.90774	YES	YES

428	a	2962.99	38.04725	YES	YES
429	a	2963.57	45.13520	YES	YES
430	a	2963.88	38.37826	YES	YES
431	a	2964.62	88.43989	YES	YES
432	a	2965.74	12.97536	YES	YES
433	a	2967.24	24.01887	YES	YES
434	a	2985.38	5.08838	YES	YES
435	a	2987.08	6.13730	YES	YES
436	a	2989.41	0.99116	YES	YES
437	a	2990.07	5.16179	YES	YES
438	a	2990.36	42.69260	YES	YES
439	a	2991.16	5.47570	YES	YES
440	a	2992.95	6.54320	YES	YES
441	a	2992.98	49.04665	YES	YES
442	a	3004.56	34.85778	YES	YES
443	a	3005.36	33.45883	YES	YES
444	a	3005.65	5.39509	YES	YES
445	a	3006.20	13.80717	YES	YES
446	a	3007.05	20.22608	YES	YES
447	a	3007.93	19.87746	YES	YES
448	a	3008.48	25.81059	YES	YES
449	a	3009.57	23.04022	YES	YES
450	a	3009.74	10.86644	YES	YES
451	a	3010.71	17.64337	YES	YES
452	a	3011.07	26.29329	YES	YES
453	a	3011.18	16.61707	YES	YES
454	a	3011.58	14.98764	YES	YES
455	a	3011.74	5.59400	YES	YES
456	a	3012.19	22.79574	YES	YES
457	a	3012.29	16.01065	YES	YES
458	a	3012.35	24.54931	YES	YES
459	a	3013.38	30.36500	YES	YES
460	a	3013.90	24.59655	YES	YES
461	a	3014.15	30.47819	YES	YES
462	a	3014.47	27.30404	YES	YES
463	a	3014.83	38.88035	YES	YES
464	a	3014.91	25.03679	YES	YES
465	a	3015.30	31.65112	YES	YES
466	a	3015.41	15.05351	YES	YES
467	a	3016.03	29.09563	YES	YES
468	a	3016.17	18.80169	YES	YES
469	a	3016.33	21.27225	YES	YES
470	a	3016.58	20.51668	YES	YES
471	a	3017.16	16.42660	YES	YES
472	a	3017.28	38.86483	YES	YES
473	a	3017.94	32.05939	YES	YES
474	a	3018.23	20.25547	YES	YES
475	a	3018.95	31.92498	YES	YES
476	a	3019.46	22.89707	YES	YES
477	a	3019.58	29.52935	YES	YES
478	a	3031.74	6.53947	YES	YES
479	a	3034.60	0.01085	YES	YES
480	a	3035.29	0.20344	YES	YES
481	a	3049.61	0.35914	YES	YES
482	a	3054.92	0.19877	YES	YES
483	a	3458.78	8.66488	YES	YES

$end

```
Double hybrid single point energy = -7344.830305190681 H
COSMO energy + OC correction = -7352.2689154038 H (in oDFB)
```

6.2.35 H_3CCN

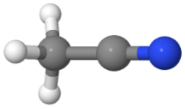

```
Method: (RI-)BP86(D3BJ)/def2-TZVPP
Symmetry: c3v

Cartesian coordinates in Ångström:
N     0.0000000    0.0000000   -2.1284736
C     0.0000000    0.0000000   -0.9665286
C     0.0000000    0.0000000    0.4884740
H     0.5147647    0.8915986    0.8688108
H     0.5147647   -0.8915986    0.8688108
H    -1.0295294    0.0000000    0.8688108

SCF energy GEOOPT =  -132.8168663825 H
ZPE =   115.3 kJ/mol
FREEH energy =   124.93 kJ/mol
FREEH entropy =   0.24344 kJ/mol/K

$vibrational spectrum
#  mode     symmetry     wave number   IR intensity    selection rules
#                         cm**(-1)        km/mol         IR     RAMAN
     1                      -0.00         0.00000        -        -
     2                      -0.00         0.00000        -        -
     3                      -0.00         0.00000        -        -
     4                      -0.00         0.00000        -        -
     5                       0.00         0.00000        -        -
     6                       0.00         0.00000        -        -
     7         e           370.13         0.15852       YES      YES
     8         e           370.13         0.15852       YES      YES
     9         a1          919.25         0.42873       YES      YES
    10         e          1021.94         2.03865       YES      YES
    11         e          1021.94         2.03865       YES      YES
    12         a1         1360.91         2.99554       YES      YES
    13         e          1425.33        10.59818       YES      YES
    14         e          1425.33        10.59818       YES      YES
    15         a1         2278.74         9.01912       YES      YES
    16         a1         2982.13         2.81479       YES      YES
    17         e          3054.39         0.66764       YES      YES
    18         e          3054.39         0.66764       YES      YES
$end

Double hybrid single point energy = -132.585848102054 H
COSMO energy + OC correction = -132.8271645470 H (in oDFB)
```

6.2.36 [{Ga(dcpe)}$_2$(NCCH$_3$)]$^{2+}$ (Adduct)

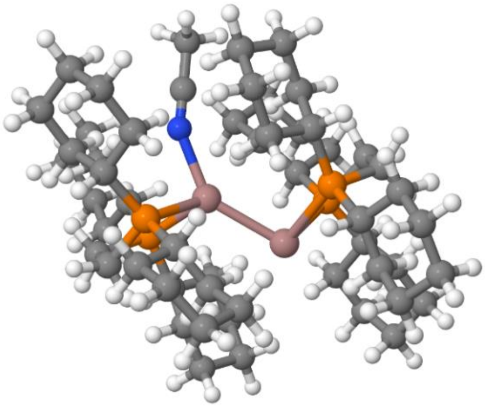

```
Method: (RI-)BP86(D3BJ)/def2-TZVPP
Symmetry: c1

Cartesian coordinates in Ångström:
H      3.8327870   -5.9231862    1.2048901
H      4.0369300   -5.3225199   -1.2284679
C      3.1039520   -5.2509532    0.7324309
C      3.7219330   -4.5700295   -0.4929734
H      2.2581246   -5.8817755    0.4131005
H      4.6337065   -4.0294297   -0.1880471
H      1.0574737   -4.6638004   -6.3890685
H      0.0860716   -5.1159353   -4.1536618
C      2.7375181   -3.5820192   -1.1279402
C      2.6039308   -4.2263017    1.7613500
H     -1.1639671   -5.2980441   -5.3836499
H      1.8707671   -4.1368808   -1.5262756
H      2.1352665   -4.7455672    2.6081667
C     -0.5132481   -4.5607036   -4.8945480
H      3.1983455   -3.0717339   -1.9880560
C      0.4193237   -3.9051078   -5.9167353
H      3.4636037   -3.6678963    2.1657762
H      1.9663780   -3.2754098   -4.5362106
C      1.2781510   -2.8164492   -5.2661678
C      1.6168996   -3.2422475    1.1127252
H     -0.1813626   -3.4563646   -6.7247397
H      1.9018812   -2.3165543   -6.0206015
C      2.2405155   -2.5545647   -0.1068512
H      0.7411075   -3.8109410    0.7552918
H     -1.2327983   -5.4366679    4.4133880
C     -1.3773808   -3.5277509   -4.1555194
H     -0.6626965   -4.5517885    2.1686326
H      3.0815895   -1.9175558    0.2186562
H     -1.9991083   -4.0401057   -3.4100332
H      0.1759453   -2.9609766   -2.7678159
H     -1.3639024   -3.8529593   -1.1408962
H     -2.6805486   -4.7468760    0.7811730
H      0.7876171   -3.7124503    4.0075316
C     -1.8857782   -4.6767005    3.9544228
H     -2.6909770   -5.2243070    3.4443316
C     -0.4870901   -2.4603888   -3.4963638
H     -3.6798004   -4.6291530   -0.6639926
C      0.4047883   -1.7796355   -4.5484804
C     -1.0835239   -3.8740504    2.9248285
```

H	1.4993539	-1.8785081	-0.5586228
C	-3.1470458	-4.0302058	0.0924067
H	4.4312211	-0.5362516	-3.5292408
H	-2.0640863	-3.0407793	-4.8670774
C	0.0273936	-3.0389137	3.5767714
C	-2.0632833	-3.1969255	-0.6017390
H	2.6598224	-1.9187645	3.9732423
P	0.8780830	-2.0098146	2.2894072
H	-0.6627938	-3.6576686	6.2473919
H	1.0239250	-1.0028606	-4.0846099
C	2.3053271	-1.2372087	3.1864422
H	-0.2312074	-1.2771732	-5.2945144
C	4.2679327	-0.0388482	-2.5637038
H	-1.4741012	-2.6518040	0.1565851
H	3.1145676	-1.1482998	2.4496487
C	-2.4569103	-3.7674021	5.0445892
H	-3.0050916	-4.3605828	5.7885266
H	-1.7638093	-3.1857473	2.3967957
H	-3.2473607	-2.7162497	-2.3433777
C	-1.3412528	-2.9671307	5.7203691
H	4.7432356	0.9508389	-2.5880126
C	-2.6975255	-2.1729184	-1.5555935
C	-4.1459199	-3.1364944	0.8327741
C	-0.5377168	-2.1513819	4.7003269
H	-4.9284890	-3.7476334	1.3018023
C	2.8538393	0.1076935	-2.3135560
P	-1.3966546	-1.2235489	-2.4554870
H	4.7322228	-0.6382138	-1.7686918
H	0.2767132	-1.6231263	5.2148583
N	1.7113864	0.2196234	-2.1224077
H	-3.6281961	-2.6048546	1.6488158
H	2.8829318	0.7224564	0.3438068
H	-3.1863389	-3.0720683	4.5960296
H	5.2076079	1.5648623	-0.0762535
H	3.8581704	0.6744732	1.8183037
H	-5.3831176	-2.6243554	-0.8674958
C	1.9706220	0.1395785	3.7820183
H	-1.7540245	-2.2922087	6.4828606
C	-4.7710840	-2.1064002	-0.1111578
Ga	-0.7701968	-0.2197092	1.5583652
C	3.3899882	1.3533211	1.0931605
Ga	-0.0703444	0.1861247	-0.8756856
C	-3.6989652	-1.2675216	-0.8185139
H	-1.1905765	-1.3814890	4.2523217
H	2.8883441	0.6554503	4.0980695
H	1.3477043	0.0253019	4.6778898
C	-2.3296106	-0.1005434	-3.5947993
H	-1.7176987	0.0052323	-4.4981817
H	0.7602262	1.2345443	-4.2231069
H	-3.2671192	-0.5892167	-3.8962342
C	4.4772397	2.2111537	0.4329276
H	1.3700097	2.1863360	-6.4153975
H	-3.1598382	-0.6573453	-0.0738068
H	2.9105362	2.7479347	-4.5234529
C	0.1873603	2.0913070	-4.6073953
P	1.0182116	1.2314266	2.6231964
H	-4.1845196	-0.5791243	-1.5240045
H	-0.6965223	1.6851384	-5.1178363

H	3.4180949	2.7143518	-1.3876271
H	-5.4506175	-1.4413957	0.4392364
H	5.0403431	2.7397143	1.2183260
C	1.0450101	2.8663507	-5.6158380
C	3.8858686	3.2381674	-0.5359923
C	2.3394860	2.2324556	1.7847715
C	2.2486868	3.5278896	-4.9403575
C	-2.6235209	1.2815708	-2.9848125
H	4.6799980	3.8708835	-0.9553157
H	1.5713168	2.9652701	-2.2734601
P	-1.1693510	2.0454412	-2.1326022
H	2.8444632	4.0808703	-5.6784280
H	2.8404467	2.7872361	2.5961773
H	1.1863465	2.6814544	0.0236322
C	-0.2463536	2.9839459	-3.4325360
H	-0.5580642	1.0419422	5.2006089
H	-3.4145833	1.2060405	-2.2277474
C	1.7351273	3.2423173	0.8023428
H	0.4290573	3.6422288	-6.0980649
C	0.9425415	3.7099444	-2.7854875
H	-1.6417967	1.2093399	3.8139607
C	2.8260159	4.1022737	0.1536271
C	-0.9899213	1.7745531	4.4988684
C	0.1412918	2.4555793	3.7089084
H	-2.9873337	1.9704619	-3.7601555
C	1.7919283	4.4598293	-3.8159652
H	1.5612510	2.5922920	5.3392622
H	-2.1060517	1.8427559	0.6367865
H	2.6547948	4.9189531	-3.3132747
C	1.0487547	3.2741530	4.6410135
H	-0.9593103	3.7343540	-3.8149464
H	3.3081484	4.7167475	0.9303484
H	2.3775848	4.8058998	-0.5605040
H	0.9995225	3.8841981	1.3082798
C	-2.6672434	2.6618041	0.1582309
C	-1.8396413	3.3092526	-0.9635965
H	-0.3335887	3.1447627	2.9862088
H	0.5855255	4.4056886	-2.0137380
H	1.8315400	3.7954447	4.0727114
H	-3.5781254	2.2086931	-0.2660323
H	-0.9292234	3.7296221	-0.5010272
H	1.2035798	5.2870164	-4.2440308
C	-1.8037101	2.8044150	5.2910872
H	-0.4801899	2.9680376	6.9935706
H	-2.5894415	2.2928914	5.8639923
C	-0.9070095	3.6279819	6.2201896
C	0.2288531	4.2969231	5.4413385
C	-2.6185028	4.4541591	-1.6322574
C	-3.0697233	3.7081143	1.2028006
H	-2.3166800	3.4765312	4.5831786
H	-3.5164257	4.0460801	-2.1240770
H	-3.6708180	3.2313837	1.9894639
H	-2.0166510	4.9376879	-2.4139563
H	-2.1581058	4.0928416	1.6922562
H	0.8925733	4.8477983	6.1217045
H	-0.1927924	5.0405701	4.7445958
H	-1.5005101	4.3846993	6.7507253
C	-3.8357071	4.8690168	0.5612148

```
C    -3.0365962    5.4972452   -0.5848163
H    -4.7983318    4.4963405    0.1743666
H    -2.1299467    5.9776070   -0.1805447
H    -3.6191527    6.2913757   -1.0708659
H    -4.0781295    5.6302229    1.3149251

SCF energy GEOOPT =  -7389.015250247 H
ZPE =    3713. kJ/mol
FREEH energy =  3904.02 kJ/mol
FREEH entropy =   1.64445 kJ/mol/K

$vibrational spectrum
#  mode     symmetry     wave number   IR intensity    selection rules
#                         cm**(-1)        km/mol          IR     RAMAN
     1                      -0.00         0.00000         -        -
     2                      -0.00         0.00000         -        -
     3                      -0.00         0.00000         -        -
     4                       0.00         0.00000         -        -
     5                       0.00         0.00000         -        -
     6                       0.00         0.00000         -        -
     7         a            11.04         0.17045        YES      YES
     8         a            16.32         0.15208        YES      YES
     9         a            17.54         0.11819        YES      YES
    10         a            18.95         0.01731        YES      YES
    11         a            26.98         0.15613        YES      YES
    12         a            29.22         0.01719        YES      YES
    13         a            30.20         0.05451        YES      YES
    14         a            31.01         0.01518        YES      YES
    15         a            32.71         0.72630        YES      YES
    16         a            39.18         0.91399        YES      YES
    17         a            40.63         1.57450        YES      YES
    18         a            41.12         0.34352        YES      YES
    19         a            43.77         0.43168        YES      YES
    20         a            47.15         0.25658        YES      YES
    21         a            50.14         0.03738        YES      YES
    22         a            52.70         0.34492        YES      YES
    23         a            54.73         0.37656        YES      YES
    24         a            57.97         0.03109        YES      YES
    25         a            60.25         0.26230        YES      YES
    26         a            62.46         0.36001        YES      YES
    27         a            66.25         0.45064        YES      YES
    28         a            67.93         0.23453        YES      YES
    29         a            70.71         0.93116        YES      YES
    30         a            71.32         1.47860        YES      YES
    31         a            73.28         0.26025        YES      YES
    32         a            77.35         0.40620        YES      YES
    33         a            78.70         1.21327        YES      YES
    34         a            83.03         0.18218        YES      YES
    35         a            85.94         0.30696        YES      YES
    36         a            88.32         0.13052        YES      YES
    37         a            92.54         1.50831        YES      YES
    38         a            99.24         0.10139        YES      YES
    39         a           115.93         1.75986        YES      YES
    40         a           121.62         0.22429        YES      YES
    41         a           122.88         0.04690        YES      YES
    42         a           134.22         0.85096        YES      YES
    43         a           136.36         1.59996        YES      YES
    44         a           137.84         1.11617        YES      YES
```

45	a	140.17	2.39376	YES	YES
46	a	143.09	5.84359	YES	YES
47	a	144.77	0.84082	YES	YES
48	a	146.93	10.17361	YES	YES
49	a	151.86	2.33326	YES	YES
50	a	156.49	4.70701	YES	YES
51	a	167.96	2.04806	YES	YES
52	a	173.36	9.16797	YES	YES
53	a	178.57	6.11277	YES	YES
54	a	179.93	2.64277	YES	YES
55	a	185.67	5.25842	YES	YES
56	a	192.81	1.09153	YES	YES
57	a	196.44	0.38573	YES	YES
58	a	201.32	4.10800	YES	YES
59	a	206.80	3.83860	YES	YES
60	a	212.41	0.40264	YES	YES
61	a	212.94	3.88114	YES	YES
62	a	214.71	0.15692	YES	YES
63	a	217.52	1.89979	YES	YES
64	a	222.26	5.88319	YES	YES
65	a	224.67	2.13769	YES	YES
66	a	228.01	1.65892	YES	YES
67	a	237.42	0.12255	YES	YES
68	a	238.02	1.08845	YES	YES
69	a	239.53	0.56656	YES	YES
70	a	242.26	1.14816	YES	YES
71	a	244.09	0.17446	YES	YES
72	a	245.57	0.44825	YES	YES
73	a	275.89	1.48622	YES	YES
74	a	277.33	0.25900	YES	YES
75	a	284.53	2.40615	YES	YES
76	a	285.58	0.57121	YES	YES
77	a	301.60	0.13424	YES	YES
78	a	304.07	0.33098	YES	YES
79	a	313.59	0.65336	YES	YES
80	a	315.11	0.24616	YES	YES
81	a	328.11	0.79246	YES	YES
82	a	331.00	0.87656	YES	YES
83	a	332.86	0.30096	YES	YES
84	a	334.74	0.28206	YES	YES
85	a	337.61	1.13706	YES	YES
86	a	342.53	0.58078	YES	YES
87	a	361.60	8.07565	YES	YES
88	a	366.51	0.97584	YES	YES
89	a	373.36	0.81210	YES	YES
90	a	374.88	0.53638	YES	YES
91	a	383.14	12.63024	YES	YES
92	a	387.69	4.89915	YES	YES
93	a	390.39	0.16375	YES	YES
94	a	391.67	0.68134	YES	YES
95	a	411.93	1.44290	YES	YES
96	a	412.45	1.45777	YES	YES
97	a	420.94	4.96436	YES	YES
98	a	422.22	5.06264	YES	YES
99	a	422.76	1.09740	YES	YES
100	a	423.58	1.21618	YES	YES
101	a	428.84	0.11955	YES	YES
102	a	430.03	0.33348	YES	YES

103	a	430.56	0.08246	YES	YES
104	a	431.52	0.33218	YES	YES
105	a	431.70	1.04034	YES	YES
106	a	431.83	0.08586	YES	YES
107	a	432.16	0.63116	YES	YES
108	a	432.66	0.31761	YES	YES
109	a	440.10	7.15937	YES	YES
110	a	446.55	2.28896	YES	YES
111	a	462.79	3.41953	YES	YES
112	a	467.03	3.98920	YES	YES
113	a	489.10	5.81351	YES	YES
114	a	490.65	2.39762	YES	YES
115	a	491.26	0.65030	YES	YES
116	a	492.72	4.68429	YES	YES
117	a	499.87	0.74035	YES	YES
118	a	503.51	0.49111	YES	YES
119	a	512.05	13.47999	YES	YES
120	a	516.46	9.28730	YES	YES
121	a	622.43	25.36394	YES	YES
122	a	623.52	4.45110	YES	YES
123	a	638.87	11.91122	YES	YES
124	a	641.13	7.67969	YES	YES
125	a	707.03	0.62582	YES	YES
126	a	713.53	0.45330	YES	YES
127	a	717.49	4.85351	YES	YES
128	a	721.52	1.68133	YES	YES
129	a	729.98	3.52772	YES	YES
130	a	730.93	9.44736	YES	YES
131	a	731.31	6.66421	YES	YES
132	a	734.40	6.39204	YES	YES
133	a	770.99	0.26264	YES	YES
134	a	771.59	0.67557	YES	YES
135	a	771.90	0.20835	YES	YES
136	a	772.41	0.58460	YES	YES
137	a	773.16	0.36483	YES	YES
138	a	773.91	0.95177	YES	YES
139	a	774.81	0.57613	YES	YES
140	a	775.18	2.34013	YES	YES
141	a	776.96	22.80596	YES	YES
142	a	779.27	10.34266	YES	YES
143	a	808.74	0.36445	YES	YES
144	a	810.48	2.49583	YES	YES
145	a	810.52	0.11502	YES	YES
146	a	811.32	1.15814	YES	YES
147	a	812.36	1.63075	YES	YES
148	a	813.40	0.40968	YES	YES
149	a	813.99	6.63603	YES	YES
150	a	815.61	7.29276	YES	YES
151	a	831.87	2.19264	YES	YES
152	a	832.80	1.30739	YES	YES
153	a	834.98	4.96372	YES	YES
154	a	835.95	2.71548	YES	YES
155	a	836.56	3.21609	YES	YES
156	a	837.27	1.71049	YES	YES
157	a	838.04	13.73575	YES	YES
158	a	839.45	8.25296	YES	YES
159	a	850.37	27.27812	YES	YES
160	a	852.34	1.30023	YES	YES

161	a	873.45	1.17193	YES	YES
162	a	875.62	3.90918	YES	YES
163	a	876.39	0.88578	YES	YES
164	a	876.51	6.75812	YES	YES
165	a	876.80	3.13342	YES	YES
166	a	878.05	0.94586	YES	YES
167	a	878.33	5.86233	YES	YES
168	a	878.95	1.31628	YES	YES
169	a	879.37	2.34908	YES	YES
170	a	879.93	1.24101	YES	YES
171	a	880.15	5.84410	YES	YES
172	a	880.27	3.30428	YES	YES
173	a	882.46	2.41413	YES	YES
174	a	883.77	0.64970	YES	YES
175	a	885.38	4.03002	YES	YES
176	a	887.81	1.10722	YES	YES
177	a	902.30	2.36985	YES	YES
178	a	902.83	3.52526	YES	YES
179	a	903.50	2.60865	YES	YES
180	a	904.32	0.83550	YES	YES
181	a	907.56	1.19126	YES	YES
182	a	909.34	1.54656	YES	YES
183	a	910.38	1.72970	YES	YES
184	a	910.67	0.34938	YES	YES
185	a	932.17	0.43422	YES	YES
186	a	982.13	0.14147	YES	YES
187	a	984.31	0.94694	YES	YES
188	a	986.40	4.75739	YES	YES
189	a	988.15	13.75894	YES	YES
190	a	989.12	0.83541	YES	YES
191	a	989.72	20.80771	YES	YES
192	a	989.87	12.22206	YES	YES
193	a	990.71	4.85598	YES	YES
194	a	991.61	6.86562	YES	YES
195	a	992.65	4.55477	YES	YES
196	a	1002.23	0.89821	YES	YES
197	a	1007.44	5.42490	YES	YES
198	a	1013.24	3.31561	YES	YES
199	a	1014.46	1.40880	YES	YES
200	a	1016.75	1.29445	YES	YES
201	a	1018.19	0.53754	YES	YES
202	a	1018.52	0.68631	YES	YES
203	a	1020.43	0.27087	YES	YES
204	a	1022.88	0.68101	YES	YES
205	a	1023.41	0.36632	YES	YES
206	a	1031.71	3.07133	YES	YES
207	a	1032.59	0.26412	YES	YES
208	a	1033.18	0.93755	YES	YES
209	a	1034.12	2.96976	YES	YES
210	a	1039.22	2.91094	YES	YES
211	a	1040.44	0.21525	YES	YES
212	a	1040.52	2.09336	YES	YES
213	a	1041.20	0.67474	YES	YES
214	a	1058.08	2.85228	YES	YES
215	a	1058.87	1.43348	YES	YES
216	a	1059.57	1.69961	YES	YES
217	a	1059.92	1.39199	YES	YES
218	a	1062.30	3.04161	YES	YES

219	a	1065.74	0.51069	YES	YES
220	a	1067.11	0.11708	YES	YES
221	a	1069.75	0.16830	YES	YES
222	a	1070.59	0.12167	YES	YES
223	a	1072.22	0.05840	YES	YES
224	a	1072.59	0.00682	YES	YES
225	a	1073.04	0.31122	YES	YES
226	a	1073.68	0.48004	YES	YES
227	a	1074.73	1.13909	YES	YES
228	a	1076.56	0.21897	YES	YES
229	a	1077.51	0.29882	YES	YES
230	a	1078.17	1.13672	YES	YES
231	a	1082.06	2.69488	YES	YES
232	a	1090.12	7.79223	YES	YES
233	a	1091.91	12.23608	YES	YES
234	a	1093.57	8.03715	YES	YES
235	a	1098.39	2.05766	YES	YES
236	a	1100.34	0.93264	YES	YES
237	a	1101.64	0.97704	YES	YES
238	a	1104.49	5.36447	YES	YES
239	a	1107.21	11.03337	YES	YES
240	a	1124.07	11.63256	YES	YES
241	a	1127.04	1.35806	YES	YES
242	a	1158.35	7.42808	YES	YES
243	a	1159.54	6.83392	YES	YES
244	a	1159.85	3.22663	YES	YES
245	a	1161.79	5.95514	YES	YES
246	a	1168.15	10.65917	YES	YES
247	a	1168.57	1.00158	YES	YES
248	a	1170.25	10.04213	YES	YES
249	a	1172.12	7.96248	YES	YES
250	a	1174.59	9.73472	YES	YES
251	a	1176.88	11.24920	YES	YES
252	a	1179.45	9.75179	YES	YES
253	a	1183.26	6.95754	YES	YES
254	a	1189.06	2.72511	YES	YES
255	a	1191.55	0.63133	YES	YES
256	a	1194.31	1.64600	YES	YES
257	a	1198.25	2.32678	YES	YES
258	a	1234.18	0.41521	YES	YES
259	a	1237.77	0.39402	YES	YES
260	a	1243.45	0.04152	YES	YES
261	a	1244.27	0.15548	YES	YES
262	a	1244.82	0.39815	YES	YES
263	a	1245.42	0.40421	YES	YES
264	a	1245.54	0.46982	YES	YES
265	a	1247.16	0.45115	YES	YES
266	a	1247.89	1.34272	YES	YES
267	a	1250.24	0.15418	YES	YES
268	a	1252.05	1.28962	YES	YES
269	a	1253.77	7.97641	YES	YES
270	a	1254.32	3.95728	YES	YES
271	a	1255.91	2.70525	YES	YES
272	a	1256.69	5.39216	YES	YES
273	a	1256.93	4.04681	YES	YES
274	a	1257.06	2.15039	YES	YES
275	a	1257.83	7.69904	YES	YES
276	a	1258.68	0.92121	YES	YES

277	a	1259.09	1.39188	YES	YES
278	a	1260.14	1.42491	YES	YES
279	a	1260.22	0.94037	YES	YES
280	a	1261.12	2.66522	YES	YES
281	a	1261.70	0.21682	YES	YES
282	a	1264.77	3.19777	YES	YES
283	a	1266.52	1.89847	YES	YES
284	a	1267.06	1.37175	YES	YES
285	a	1268.37	1.86610	YES	YES
286	a	1278.86	1.87667	YES	YES
287	a	1281.54	0.71697	YES	YES
288	a	1283.92	3.06850	YES	YES
289	a	1284.21	1.61503	YES	YES
290	a	1289.38	1.90572	YES	YES
291	a	1290.34	0.07081	YES	YES
292	a	1290.73	5.79364	YES	YES
293	a	1291.93	3.17407	YES	YES
294	a	1308.59	2.04245	YES	YES
295	a	1309.38	2.04030	YES	YES
296	a	1310.31	0.90135	YES	YES
297	a	1312.20	2.82115	YES	YES
298	a	1312.41	1.13888	YES	YES
299	a	1314.83	1.81148	YES	YES
300	a	1315.66	3.96219	YES	YES
301	a	1319.55	1.44840	YES	YES
302	a	1320.33	0.33170	YES	YES
303	a	1320.58	2.25934	YES	YES
304	a	1322.13	0.27325	YES	YES
305	a	1322.79	1.00106	YES	YES
306	a	1323.21	2.02243	YES	YES
307	a	1323.44	1.51742	YES	YES
308	a	1323.77	3.17285	YES	YES
309	a	1324.64	0.87178	YES	YES
310	a	1324.93	1.31225	YES	YES
311	a	1325.49	2.29581	YES	YES
312	a	1325.99	4.12602	YES	YES
313	a	1326.28	3.10543	YES	YES
314	a	1326.69	0.20691	YES	YES
315	a	1327.16	5.55126	YES	YES
316	a	1328.23	0.54560	YES	YES
317	a	1328.62	1.22676	YES	YES
318	a	1333.06	0.16355	YES	YES
319	a	1333.49	0.55111	YES	YES
320	a	1336.49	0.31666	YES	YES
321	a	1337.25	1.58991	YES	YES
322	a	1337.59	0.37003	YES	YES
323	a	1337.92	0.33741	YES	YES
324	a	1338.36	0.21048	YES	YES
325	a	1340.70	1.09588	YES	YES
326	a	1340.97	1.08275	YES	YES
327	a	1346.82	3.27231	YES	YES
328	a	1347.08	0.94191	YES	YES
329	a	1347.37	2.69850	YES	YES
330	a	1348.00	1.15520	YES	YES
331	a	1348.24	1.28145	YES	YES
332	a	1350.00	0.76104	YES	YES
333	a	1350.34	0.51318	YES	YES
334	a	1353.63	6.34592	YES	YES

335	a	1400.48	8.51202	YES	YES
336	a	1400.71	12.89912	YES	YES
337	a	1401.06	10.73491	YES	YES
338	a	1405.42	13.60924	YES	YES
339	a	1405.79	7.61343	YES	YES
340	a	1407.04	5.52467	YES	YES
341	a	1422.83	3.21318	YES	YES
342	a	1424.33	3.26873	YES	YES
343	a	1425.97	4.97678	YES	YES
344	a	1427.32	2.14480	YES	YES
345	a	1429.03	0.64849	YES	YES
346	a	1429.74	5.57236	YES	YES
347	a	1430.76	3.97042	YES	YES
348	a	1432.68	0.56241	YES	YES
349	a	1433.20	2.14272	YES	YES
350	a	1434.10	7.09930	YES	YES
351	a	1434.53	6.10331	YES	YES
352	a	1434.99	1.44202	YES	YES
353	a	1435.49	4.11297	YES	YES
354	a	1437.15	5.05355	YES	YES
355	a	1437.21	5.11225	YES	YES
356	a	1437.81	3.51687	YES	YES
357	a	1437.94	10.04702	YES	YES
358	a	1438.09	1.37502	YES	YES
359	a	1438.50	1.56461	YES	YES
360	a	1438.66	2.73257	YES	YES
361	a	1439.42	2.21633	YES	YES
362	a	1440.23	2.04055	YES	YES
363	a	1440.50	2.51062	YES	YES
364	a	1441.59	6.55677	YES	YES
365	a	1441.90	6.11511	YES	YES
366	a	1442.38	43.16783	YES	YES
367	a	1442.73	21.62341	YES	YES
368	a	1443.36	9.80062	YES	YES
369	a	1444.44	0.42610	YES	YES
370	a	1444.69	14.33882	YES	YES
371	a	1446.43	2.58603	YES	YES
372	a	1446.62	21.51272	YES	YES
373	a	1452.18	5.21164	YES	YES
374	a	1452.37	0.61299	YES	YES
375	a	1453.24	9.15532	YES	YES
376	a	1453.53	0.54686	YES	YES
377	a	1454.60	1.35437	YES	YES
378	a	1455.63	2.32705	YES	YES
379	a	1455.81	0.49203	YES	YES
380	a	1457.90	1.25697	YES	YES
381	a	2242.26	20.31840	YES	YES
382	a	2911.62	16.82176	YES	YES
383	a	2916.85	2.00186	YES	YES
384	a	2922.93	10.57171	YES	YES
385	a	2923.17	8.28668	YES	YES
386	a	2923.46	15.33175	YES	YES
387	a	2924.47	23.35140	YES	YES
388	a	2925.24	18.67567	YES	YES
389	a	2926.66	7.61476	YES	YES
390	a	2927.53	1.14034	YES	YES
391	a	2931.48	15.22161	YES	YES
392	a	2933.07	18.01906	YES	YES

393	a	2933.61	26.37113	YES	YES
394	a	2936.35	13.36636	YES	YES
395	a	2936.43	14.96044	YES	YES
396	a	2940.19	10.69268	YES	YES
397	a	2940.84	3.25615	YES	YES
398	a	2941.27	4.07313	YES	YES
399	a	2942.47	12.23144	YES	YES
400	a	2942.49	9.05098	YES	YES
401	a	2942.54	11.93925	YES	YES
402	a	2942.60	1.24862	YES	YES
403	a	2942.81	4.46334	YES	YES
404	a	2943.61	9.88514	YES	YES
405	a	2943.79	12.42787	YES	YES
406	a	2944.90	7.48269	YES	YES
407	a	2945.53	10.65785	YES	YES
408	a	2946.23	6.35159	YES	YES
409	a	2951.99	10.43736	YES	YES
410	a	2952.02	12.61697	YES	YES
411	a	2953.54	3.28905	YES	YES
412	a	2953.92	2.36763	YES	YES
413	a	2955.43	3.84467	YES	YES
414	a	2955.56	9.06041	YES	YES
415	a	2956.00	6.35357	YES	YES
416	a	2956.37	3.28986	YES	YES
417	a	2957.64	27.05426	YES	YES
418	a	2958.11	14.68455	YES	YES
419	a	2958.91	25.88212	YES	YES
420	a	2959.16	53.43262	YES	YES
421	a	2959.32	12.36222	YES	YES
422	a	2959.70	20.75652	YES	YES
423	a	2960.69	48.27405	YES	YES
424	a	2961.10	25.01620	YES	YES
425	a	2961.12	19.88236	YES	YES
426	a	2963.66	38.64074	YES	YES
427	a	2963.70	30.17184	YES	YES
428	a	2966.48	22.51114	YES	YES
429	a	2973.13	28.85297	YES	YES
430	a	2973.58	17.40027	YES	YES
431	a	2973.85	40.92784	YES	YES
432	a	2975.77	4.91662	YES	YES
433	a	2975.90	14.11282	YES	YES
434	a	2978.32	31.17717	YES	YES
435	a	2980.97	39.35276	YES	YES
436	a	2983.83	6.05436	YES	YES
437	a	2984.57	11.80997	YES	YES
438	a	2987.69	7.34172	YES	YES
439	a	2989.42	26.04757	YES	YES
440	a	2993.93	17.07463	YES	YES
441	a	2995.58	32.50940	YES	YES
442	a	3000.80	47.32400	YES	YES
443	a	3002.19	8.17463	YES	YES
444	a	3002.62	24.55513	YES	YES
445	a	3004.17	19.00315	YES	YES
446	a	3004.39	31.57926	YES	YES
447	a	3005.40	14.99110	YES	YES
448	a	3005.83	29.03149	YES	YES
449	a	3006.45	16.81195	YES	YES
450	a	3008.29	25.08170	YES	YES

451	a	3008.90	27.28031	YES	YES
452	a	3009.03	22.17084	YES	YES
453	a	3009.41	12.17312	YES	YES
454	a	3009.77	28.32911	YES	YES
455	a	3011.35	13.21771	YES	YES
456	a	3011.47	20.83219	YES	YES
457	a	3011.75	47.52653	YES	YES
458	a	3012.18	14.30558	YES	YES
459	a	3012.62	19.90995	YES	YES
460	a	3013.60	41.76386	YES	YES
461	a	3013.66	23.18659	YES	YES
462	a	3014.76	21.78947	YES	YES
463	a	3014.91	24.88707	YES	YES
464	a	3015.19	30.65217	YES	YES
465	a	3015.35	30.07934	YES	YES
466	a	3016.20	37.72091	YES	YES
467	a	3016.51	3.50030	YES	YES
468	a	3016.52	30.46247	YES	YES
469	a	3016.66	38.64847	YES	YES
470	a	3016.81	31.72181	YES	YES
471	a	3017.21	18.03515	YES	YES
472	a	3018.93	21.80220	YES	YES
473	a	3020.34	26.40154	YES	YES
474	a	3023.94	2.41472	YES	YES
475	a	3032.53	0.13029	YES	YES
476	a	3037.42	14.26913	YES	YES
477	a	3041.49	1.61676	YES	YES
478	a	3049.71	4.61990	YES	YES
479	a	3049.98	0.27075	YES	YES
480	a	3055.60	2.36994	YES	YES

$end

Double hybrid single point energy = -7381.656574387431 H
COSMO energy + OC correction = -7389.1647565056 H (in *o*DFB)

6.2.37 [H{Ga(dcpe)}$_2$(NCCH$_3$)]$^{3+}$

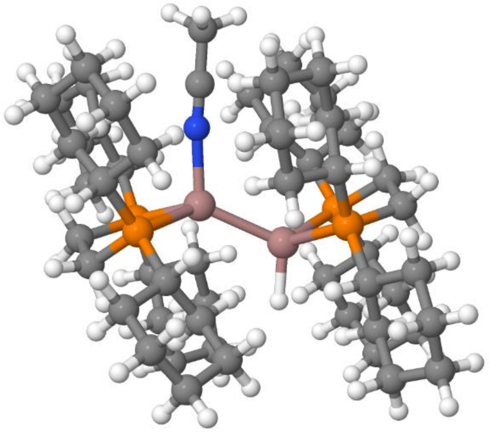

Method: (RI-)BP86(D3BJ)/def2-TZVPP
Symmetry: c1

Cartesian coordinates in Ångström:
H 4.1188786 -5.7757600 1.2725987

H	4.1636458	-5.2676800	-1.1878541
C	3.3171391	-5.1860870	0.8097278
C	3.8204953	-4.5116032	-0.4698988
H	2.5112396	-5.8955047	0.5621926
H	4.6970456	-3.8856381	-0.2332486
H	0.9871171	-4.7132818	-6.4374935
H	-0.0422708	-5.1787089	-4.2300284
C	2.7274686	-3.6418513	-1.1003257
C	2.7929500	-4.1570999	1.8215036
H	-1.2754355	-5.2791582	-5.4849631
H	1.8958198	-4.2858942	-1.4325249
H	2.4106188	-4.6677697	2.7159207
C	-0.6030021	-4.5819251	-4.9684186
H	3.1042565	-3.1335905	-2.0014455
C	0.3719023	-3.9406226	-5.9589162
H	3.6272650	-3.5165659	2.1492002
H	1.9205670	-3.4097533	-4.5380663
C	1.2655020	-2.9058068	-5.2686774
C	1.6960671	-3.2853386	1.1826523
H	-0.1938873	-3.4502588	-6.7674109
H	1.9219386	-2.4157178	-6.0009413
C	2.1849104	-2.6098438	-0.1054880
H	0.8447669	-3.9349203	0.9163903
H	-1.3864951	-5.3632216	4.3771306
C	-1.4421211	-3.5319593	-4.2234956
H	-0.7636142	-4.4588008	2.1561619
H	2.9719192	-1.8745494	0.1371954
H	-2.0963422	-4.0325307	-3.4977932
H	0.1003363	-3.0493481	-2.7851314
H	-1.4520798	-3.8870454	-1.1531621
H	-2.7574469	-4.7040218	0.8100729
H	0.7215689	-3.7553787	4.0256788
C	-1.9951173	-4.5565723	3.9388201
H	-2.8273047	-5.0454864	3.4138694
C	-0.5188708	-2.5139130	-3.5273563
H	-3.7762480	-4.5688257	-0.6161769
C	0.4244818	-1.8459948	-4.5433409
C	-1.1486073	-3.7757597	2.9263920
H	1.3538022	-2.0464496	-0.5567490
C	-3.2115135	-3.9786538	0.1227145
H	4.1335338	-0.3953198	-3.8136948
H	-2.0946566	-3.0042064	-4.9377303
C	0.0047157	-3.0255179	3.6102233
C	-2.1097369	-3.1968671	-0.6050280
H	2.7253390	-1.9079495	4.0206110
P	0.9586354	-2.0673489	2.3561031
H	-0.7223225	-3.6609114	6.2587370
H	1.0692451	-1.1085018	-4.0474478
C	2.3528568	-1.2336655	3.2369013
H	-0.1707755	-1.2966355	-5.2893907
C	4.1235700	0.0948817	-2.8298675
H	-1.4792984	-2.6690188	0.1330390
H	3.1606848	-1.1273998	2.5006784
C	-2.5164841	-3.6424038	5.0492072
H	-3.0956445	-4.2230573	5.7783854
H	-1.7874583	-3.0367165	2.4166843
H	-3.3201881	-2.6949262	-2.3224627
C	-1.3602235	-2.9231436	5.7465513

H	4.5080831	1.1191355	-2.9349495
C	-2.7317776	-2.1615728	-1.5558167
C	-4.1671889	-3.0438804	0.8693909
C	-0.5069106	-2.1261706	4.7501868
H	-4.9664873	-3.6243088	1.3477473
C	2.7774922	0.1335354	-2.3201147
P	-1.4358796	-1.2738483	-2.5103488
H	4.7715225	-0.4654002	-2.1422332
H	0.3374223	-1.6674598	5.2829087
N	1.6852120	0.1642907	-1.9335571
H	-3.6252243	-2.5297074	1.6807995
H	2.9409126	0.7155449	0.4283489
H	-3.2085886	-2.8985472	4.6196963
H	5.2351911	1.5810069	-0.0319642
H	3.9212448	0.7183636	1.9016900
H	-5.4133229	-2.4900506	-0.8118334
C	1.9944415	0.1330650	3.8494157
H	-1.7351576	-2.2436360	6.5235759
C	-4.7671683	-1.9954842	-0.0694070
Ga	-0.2332692	-0.2234258	1.3755388
C	3.4372771	1.3716046	1.1637764
Ga	-0.1567291	0.1711132	-1.0515268
C	-3.6767769	-1.2058997	-0.8065773
H	-1.1118723	-1.3098106	4.3215805
H	2.9054625	0.6378410	4.2001165
H	1.3484540	0.0026573	4.7262736
C	-2.3164313	-0.1071220	-3.6393132
H	-1.6904505	-0.0111304	-4.5344010
H	0.7927447	1.2043058	-4.2320437
H	-3.2587612	-0.5748360	-3.9579604
C	4.5077908	2.2302527	0.4763928
H	1.4436926	2.1425896	-6.4171985
H	-3.0979991	-0.6090723	-0.0805098
H	2.9720929	2.6962924	-4.5109340
C	0.2372342	2.0708687	-4.6246679
P	1.0865629	1.2692320	2.7106912
H	-4.1517807	-0.5063325	-1.5073973
H	-0.6460342	1.6764225	-5.1449473
H	3.4284341	2.7033807	-1.3421679
H	-5.4090245	-1.2992497	0.4865860
H	5.0770986	2.7717343	1.2475302
C	1.1189707	2.8303672	-5.6248668
C	3.8974872	3.2401903	-0.4983513
C	2.3776392	2.2545902	1.8415864
C	2.3207076	3.4814848	-4.9371811
C	-2.6014666	1.2796765	-3.0322281
H	4.6831373	3.8739016	-0.9301869
H	1.6059624	2.9471196	-2.2670863
P	-1.1635839	2.0659525	-2.1811845
H	2.9310793	4.0235500	-5.6704650
H	2.8737237	2.8299838	2.6417653
H	1.2040487	2.6728487	0.0756226
C	-0.1957848	2.9778296	-3.4587783
H	-0.5927732	0.9934027	5.2052714
H	-3.4024843	1.2185237	-2.2844158
C	1.7516544	3.2442431	0.8477199
H	0.5177149	3.6094449	-6.1189072
C	0.9918807	3.6934993	-2.7963778

```
H    -1.6489278    1.1316944    3.7912780
C     2.8354800    4.1043972    0.1867426
C    -1.0308542    1.7106268    4.4924025
C     0.0979901    2.4374423    3.7390876
H    -2.9532098    1.9643982   -3.8166168
C     1.8633517    4.4247365   -3.8230972
H     1.4780667    2.6139974    5.4044143
H    -1.8488474    1.8698610    0.6742406
H     2.7246623    4.8769399   -3.3125282
C     0.9629953    3.2809854    4.6941968
H    -0.8964565    3.7355994   -3.8504720
H     3.3148317    4.7298891    0.9555756
H     2.3785912    4.7990198   -0.5299646
H     1.0137855    3.8853699    1.3503542
C    -2.5225804    2.6040959    0.2055939
C    -1.8284526    3.2924457   -0.9809189
H    -0.3703929    3.1080235    2.9960646
H     0.6316230    4.4014018   -2.0376257
H     1.7409774    3.8285846    4.1441680
H    -3.4018193    2.0421680   -0.1488402
H    -0.9235736    3.7949762   -0.5967300
H     1.2909709    5.2569757   -4.2612986
C    -1.8929453    2.7164628    5.2676387
H    -0.6187435    2.9158267    7.0050230
H    -2.6752384    2.1767269    5.8178712
C    -1.0454320    3.5627529    6.2212146
C     0.0858252    4.2727979    5.4737454
C    -2.7392879    4.3495218   -1.6327892
C    -2.9732852    3.6414115    1.2414832
H    -2.4096472    3.3741011    4.5493403
H    -3.6239067    3.8540442   -2.0638711
H    -3.4877711    3.1335266    2.0687261
H    -2.2218465    4.8598181   -2.4568896
H    -2.0803321    4.1267743    1.6722109
H     0.7163979    4.8400054    6.1709559
H    -0.3391777    5.0059826    4.7685692
H    -1.6761389    4.2979387    6.7373663
C    -3.8763102    4.7052987    0.6098379
C    -3.1928771    5.3769200   -0.5844889
H    -4.8164659    4.2345916    0.2796722
H    -2.3157560    5.9476206   -0.2379369
H    -3.8680031    6.1016531   -1.0576454
H    -4.1544901    5.4583680    1.3583787
H    -1.7122031   -0.2441420    1.8963738

SCF energy GEOOPT =  -7389.282422237 H
ZPE =   3737. kJ/mol
FREEH energy =  3927.11 kJ/mol
FREEH entropy =   1.65571 kJ/mol/K

$vibrational spectrum
#  mode     symmetry     wave number   IR intensity    selection rules
#                         cm**(-1)        km/mol          IR     RAMAN
     1                      -0.00         0.00000        -       -
     2                      -0.00         0.00000        -       -
     3                      -0.00         0.00000        -       -
     4                      -0.00         0.00000        -       -
     5                       0.00         0.00000        -       -
```

6		0.00	0.00000	-	-
7	a	2.69	0.10980	YES	YES
8	a	10.91	0.11068	YES	YES
9	a	17.28	0.01268	YES	YES
10	a	20.79	0.14548	YES	YES
11	a	23.01	0.11202	YES	YES
12	a	24.02	0.01023	YES	YES
13	a	28.48	0.09094	YES	YES
14	a	30.91	0.59768	YES	YES
15	a	32.27	0.01015	YES	YES
16	a	38.10	0.56407	YES	YES
17	a	38.80	0.23526	YES	YES
18	a	41.69	0.35775	YES	YES
19	a	45.27	0.77405	YES	YES
20	a	46.47	0.19549	YES	YES
21	a	48.09	0.53787	YES	YES
22	a	50.18	0.71096	YES	YES
23	a	52.54	0.43739	YES	YES
24	a	54.38	0.55758	YES	YES
25	a	56.95	0.16974	YES	YES
26	a	60.11	0.22225	YES	YES
27	a	62.61	1.90390	YES	YES
28	a	66.33	0.36761	YES	YES
29	a	68.77	0.41876	YES	YES
30	a	69.60	0.22589	YES	YES
31	a	77.11	0.84634	YES	YES
32	a	77.47	0.64284	YES	YES
33	a	80.42	0.51804	YES	YES
34	a	85.21	0.11242	YES	YES
35	a	88.43	0.63852	YES	YES
36	a	91.41	0.32132	YES	YES
37	a	92.31	0.11825	YES	YES
38	a	97.50	0.10281	YES	YES
39	a	126.98	1.00825	YES	YES
40	a	129.85	0.80450	YES	YES
41	a	133.31	0.32762	YES	YES
42	a	140.90	0.51658	YES	YES
43	a	144.26	0.25997	YES	YES
44	a	146.04	1.94283	YES	YES
45	a	147.39	1.44931	YES	YES
46	a	150.08	0.60602	YES	YES
47	a	152.26	2.82430	YES	YES
48	a	155.77	1.87918	YES	YES
49	a	167.39	6.18944	YES	YES
50	a	172.83	0.08065	YES	YES
51	a	182.33	7.36120	YES	YES
52	a	184.31	3.95930	YES	YES
53	a	186.95	7.78444	YES	YES
54	a	188.77	2.01591	YES	YES
55	a	196.47	0.61719	YES	YES
56	a	198.11	0.10042	YES	YES
57	a	198.38	3.05677	YES	YES
58	a	207.23	4.73707	YES	YES
59	a	210.28	0.92336	YES	YES
60	a	212.45	0.04082	YES	YES
61	a	215.12	0.12514	YES	YES
62	a	223.34	1.84805	YES	YES
63	a	225.09	0.11688	YES	YES

64	a	225.96	1.91828	YES	YES
65	a	236.39	1.48879	YES	YES
66	a	236.88	0.04817	YES	YES
67	a	238.06	0.40940	YES	YES
68	a	240.18	0.71465	YES	YES
69	a	242.10	0.09455	YES	YES
70	a	242.84	0.81896	YES	YES
71	a	247.02	1.88941	YES	YES
72	a	270.67	2.09640	YES	YES
73	a	276.31	1.65678	YES	YES
74	a	278.75	1.06980	YES	YES
75	a	287.97	2.15973	YES	YES
76	a	295.51	0.19714	YES	YES
77	a	306.73	0.43757	YES	YES
78	a	307.61	0.14928	YES	YES
79	a	315.06	0.23598	YES	YES
80	a	315.81	0.37124	YES	YES
81	a	329.31	0.66066	YES	YES
82	a	331.16	0.50460	YES	YES
83	a	335.93	0.16035	YES	YES
84	a	336.31	0.33670	YES	YES
85	a	345.57	0.37325	YES	YES
86	a	347.06	0.46212	YES	YES
87	a	370.24	1.97341	YES	YES
88	a	375.06	3.19456	YES	YES
89	a	376.74	2.12502	YES	YES
90	a	379.33	1.18304	YES	YES
91	a	392.52	3.33800	YES	YES
92	a	394.50	0.26650	YES	YES
93	a	396.08	0.91113	YES	YES
94	a	397.64	8.77844	YES	YES
95	a	410.72	1.61465	YES	YES
96	a	413.06	1.09479	YES	YES
97	a	419.35	0.88215	YES	YES
98	a	420.19	4.95365	YES	YES
99	a	421.12	2.50939	YES	YES
100	a	424.60	1.49718	YES	YES
101	a	428.04	0.17195	YES	YES
102	a	428.67	0.34977	YES	YES
103	a	429.77	0.13099	YES	YES
104	a	430.79	0.11289	YES	YES
105	a	431.37	0.01450	YES	YES
106	a	431.79	0.34693	YES	YES
107	a	432.42	0.76770	YES	YES
108	a	433.89	0.91483	YES	YES
109	a	444.17	6.09477	YES	YES
110	a	446.80	1.16022	YES	YES
111	a	466.07	2.48567	YES	YES
112	a	466.91	4.72608	YES	YES
113	a	489.96	3.30614	YES	YES
114	a	491.44	0.08357	YES	YES
115	a	492.50	0.76949	YES	YES
116	a	493.92	4.74816	YES	YES
117	a	501.98	7.22879	YES	YES
118	a	505.73	0.88560	YES	YES
119	a	516.80	26.43094	YES	YES
120	a	520.65	9.57280	YES	YES
121	a	549.12	49.29588	YES	YES

122	a	563.33	120.26402	YES	YES
123	a	624.66	19.45455	YES	YES
124	a	628.69	10.66223	YES	YES
125	a	641.73	7.82588	YES	YES
126	a	645.04	8.00806	YES	YES
127	a	713.40	0.56187	YES	YES
128	a	714.81	0.25352	YES	YES
129	a	720.68	1.12976	YES	YES
130	a	721.15	1.14509	YES	YES
131	a	732.82	6.10507	YES	YES
132	a	735.23	6.65538	YES	YES
133	a	736.26	2.28959	YES	YES
134	a	736.91	7.39179	YES	YES
135	a	768.73	0.18200	YES	YES
136	a	770.13	0.51607	YES	YES
137	a	770.97	0.36476	YES	YES
138	a	772.22	0.82707	YES	YES
139	a	774.00	0.32698	YES	YES
140	a	774.09	0.28135	YES	YES
141	a	774.74	0.46106	YES	YES
142	a	774.99	0.11488	YES	YES
143	a	780.27	16.53449	YES	YES
144	a	783.97	8.98794	YES	YES
145	a	807.19	1.67729	YES	YES
146	a	808.55	0.53925	YES	YES
147	a	809.46	2.91225	YES	YES
148	a	810.23	0.91629	YES	YES
149	a	811.33	0.55224	YES	YES
150	a	813.39	2.07537	YES	YES
151	a	814.86	4.32840	YES	YES
152	a	815.47	9.53925	YES	YES
153	a	831.62	0.82155	YES	YES
154	a	833.02	1.02348	YES	YES
155	a	834.40	2.22362	YES	YES
156	a	835.50	1.33656	YES	YES
157	a	836.00	0.90176	YES	YES
158	a	836.24	6.00929	YES	YES
159	a	836.80	9.42485	YES	YES
160	a	838.83	8.93812	YES	YES
161	a	850.94	13.65923	YES	YES
162	a	852.96	3.08177	YES	YES
163	a	871.39	3.11961	YES	YES
164	a	873.22	3.21313	YES	YES
165	a	873.40	1.74934	YES	YES
166	a	874.41	3.97709	YES	YES
167	a	875.00	4.13656	YES	YES
168	a	876.14	4.76623	YES	YES
169	a	876.54	1.81927	YES	YES
170	a	877.86	1.46700	YES	YES
171	a	878.68	1.94501	YES	YES
172	a	879.00	2.76445	YES	YES
173	a	879.25	3.43352	YES	YES
174	a	881.40	1.52946	YES	YES
175	a	882.28	3.35719	YES	YES
176	a	883.25	1.99138	YES	YES
177	a	885.74	6.82372	YES	YES
178	a	887.47	0.55817	YES	YES
179	a	901.76	2.41806	YES	YES

180	a	902.86	1.65885	YES	YES
181	a	903.27	1.00119	YES	YES
182	a	904.21	2.45030	YES	YES
183	a	907.57	0.66674	YES	YES
184	a	908.04	4.34551	YES	YES
185	a	909.19	0.47269	YES	YES
186	a	910.46	1.27499	YES	YES
187	a	951.64	3.37034	YES	YES
188	a	983.00	0.34218	YES	YES
189	a	983.90	0.21194	YES	YES
190	a	986.56	4.99248	YES	YES
191	a	987.00	3.69872	YES	YES
192	a	987.21	26.84156	YES	YES
193	a	988.05	25.24001	YES	YES
194	a	988.20	16.37286	YES	YES
195	a	988.68	2.61190	YES	YES
196	a	990.19	7.81189	YES	YES
197	a	991.23	1.18801	YES	YES
198	a	999.43	5.14225	YES	YES
199	a	1002.57	4.64041	YES	YES
200	a	1010.80	2.29891	YES	YES
201	a	1012.96	0.87861	YES	YES
202	a	1013.42	1.84653	YES	YES
203	a	1014.82	1.23601	YES	YES
204	a	1016.62	1.39184	YES	YES
205	a	1018.99	0.91762	YES	YES
206	a	1020.52	1.39154	YES	YES
207	a	1020.98	1.32277	YES	YES
208	a	1030.10	3.92577	YES	YES
209	a	1031.27	2.12827	YES	YES
210	a	1032.71	0.75251	YES	YES
211	a	1033.12	3.61027	YES	YES
212	a	1038.06	3.82851	YES	YES
213	a	1039.29	0.50716	YES	YES
214	a	1039.74	2.42329	YES	YES
215	a	1040.16	0.39987	YES	YES
216	a	1056.92	1.91240	YES	YES
217	a	1058.10	1.10714	YES	YES
218	a	1059.28	0.69393	YES	YES
219	a	1059.89	1.59116	YES	YES
220	a	1062.67	4.14489	YES	YES
221	a	1064.17	0.18975	YES	YES
222	a	1067.48	0.06054	YES	YES
223	a	1068.40	0.92207	YES	YES
224	a	1069.32	0.28342	YES	YES
225	a	1069.65	0.38753	YES	YES
226	a	1071.16	0.16383	YES	YES
227	a	1071.54	0.03936	YES	YES
228	a	1073.36	0.35022	YES	YES
229	a	1073.70	0.35537	YES	YES
230	a	1075.44	0.26780	YES	YES
231	a	1075.99	0.25730	YES	YES
232	a	1084.21	1.21120	YES	YES
233	a	1085.87	1.31492	YES	YES
234	a	1093.29	13.38399	YES	YES
235	a	1094.97	3.02032	YES	YES
236	a	1097.42	9.70465	YES	YES
237	a	1098.57	1.38705	YES	YES

238	a	1100.86	1.61118	YES	YES
239	a	1102.99	0.40429	YES	YES
240	a	1106.34	1.02704	YES	YES
241	a	1107.31	15.30181	YES	YES
242	a	1132.59	3.92706	YES	YES
243	a	1134.83	0.47359	YES	YES
244	a	1159.42	4.57156	YES	YES
245	a	1161.01	13.33008	YES	YES
246	a	1161.30	6.70682	YES	YES
247	a	1161.91	2.27479	YES	YES
248	a	1169.52	3.58449	YES	YES
249	a	1170.19	7.39722	YES	YES
250	a	1170.99	7.30741	YES	YES
251	a	1172.22	9.55930	YES	YES
252	a	1179.24	32.47502	YES	YES
253	a	1180.44	1.66599	YES	YES
254	a	1184.01	1.78481	YES	YES
255	a	1184.62	13.27847	YES	YES
256	a	1189.39	3.87524	YES	YES
257	a	1191.81	2.18098	YES	YES
258	a	1192.47	1.26583	YES	YES
259	a	1195.87	2.43710	YES	YES
260	a	1241.53	0.57079	YES	YES
261	a	1242.71	0.16898	YES	YES
262	a	1243.24	0.26521	YES	YES
263	a	1244.39	0.44107	YES	YES
264	a	1244.88	0.41437	YES	YES
265	a	1245.50	0.96055	YES	YES
266	a	1246.05	0.10643	YES	YES
267	a	1246.93	0.28574	YES	YES
268	a	1247.06	0.23500	YES	YES
269	a	1248.40	0.28396	YES	YES
270	a	1252.11	0.54172	YES	YES
271	a	1253.04	5.79266	YES	YES
272	a	1255.02	9.27971	YES	YES
273	a	1255.38	3.79953	YES	YES
274	a	1255.81	1.76846	YES	YES
275	a	1256.14	2.93954	YES	YES
276	a	1257.05	5.33330	YES	YES
277	a	1257.93	12.35897	YES	YES
278	a	1258.00	6.27695	YES	YES
279	a	1258.82	3.32009	YES	YES
280	a	1259.06	2.45572	YES	YES
281	a	1259.17	2.26119	YES	YES
282	a	1261.13	1.80692	YES	YES
283	a	1261.57	1.12764	YES	YES
284	a	1264.78	3.59799	YES	YES
285	a	1266.86	1.87253	YES	YES
286	a	1268.85	0.08387	YES	YES
287	a	1269.26	2.88430	YES	YES
288	a	1279.77	1.15507	YES	YES
289	a	1280.53	3.11175	YES	YES
290	a	1281.15	4.26078	YES	YES
291	a	1283.29	3.46127	YES	YES
292	a	1286.57	1.94189	YES	YES
293	a	1288.16	2.29865	YES	YES
294	a	1288.99	1.33385	YES	YES
295	a	1290.38	5.32764	YES	YES

296	a	1304.03	3.80735	YES	YES
297	a	1305.33	2.21130	YES	YES
298	a	1307.59	1.53042	YES	YES
299	a	1308.33	2.16386	YES	YES
300	a	1308.88	0.84042	YES	YES
301	a	1310.31	2.31314	YES	YES
302	a	1313.54	1.31168	YES	YES
303	a	1313.85	1.10500	YES	YES
304	a	1319.38	2.10045	YES	YES
305	a	1320.58	2.48756	YES	YES
306	a	1321.30	1.20786	YES	YES
307	a	1321.81	1.42794	YES	YES
308	a	1322.75	2.19567	YES	YES
309	a	1322.97	0.57604	YES	YES
310	a	1323.44	5.11534	YES	YES
311	a	1323.59	4.61636	YES	YES
312	a	1324.17	0.20619	YES	YES
313	a	1324.54	1.03934	YES	YES
314	a	1325.13	4.03948	YES	YES
315	a	1325.94	2.57212	YES	YES
316	a	1326.18	4.50637	YES	YES
317	a	1326.93	1.32295	YES	YES
318	a	1327.07	0.26499	YES	YES
319	a	1327.93	0.55295	YES	YES
320	a	1333.58	0.64836	YES	YES
321	a	1335.80	0.28213	YES	YES
322	a	1336.35	0.47043	YES	YES
323	a	1338.55	0.23783	YES	YES
324	a	1340.21	1.07203	YES	YES
325	a	1340.52	0.15425	YES	YES
326	a	1340.92	0.32633	YES	YES
327	a	1341.84	0.34384	YES	YES
328	a	1344.54	2.34873	YES	YES
329	a	1346.01	3.05589	YES	YES
330	a	1347.25	3.32458	YES	YES
331	a	1348.28	15.48195	YES	YES
332	a	1348.50	2.23045	YES	YES
333	a	1349.54	3.50593	YES	YES
334	a	1350.86	0.09647	YES	YES
335	a	1351.16	2.05863	YES	YES
336	a	1351.78	3.56336	YES	YES
337	a	1392.83	19.46811	YES	YES
338	a	1394.34	13.00949	YES	YES
339	a	1397.53	9.32805	YES	YES
340	a	1398.45	8.86693	YES	YES
341	a	1402.24	8.66243	YES	YES
342	a	1403.82	7.25918	YES	YES
343	a	1423.38	1.69984	YES	YES
344	a	1424.39	2.15904	YES	YES
345	a	1425.69	5.70229	YES	YES
346	a	1427.73	1.60157	YES	YES
347	a	1428.31	5.68162	YES	YES
348	a	1429.49	1.20930	YES	YES
349	a	1430.05	4.64516	YES	YES
350	a	1433.15	4.69265	YES	YES
351	a	1433.91	0.75411	YES	YES
352	a	1434.95	2.73462	YES	YES
353	a	1435.15	10.43588	YES	YES

354	a	1435.49	0.87869	YES	YES
355	a	1436.68	1.82096	YES	YES
356	a	1437.06	4.65995	YES	YES
357	a	1437.28	8.76531	YES	YES
358	a	1437.67	5.57947	YES	YES
359	a	1437.76	0.17019	YES	YES
360	a	1438.04	9.75867	YES	YES
361	a	1438.30	3.28859	YES	YES
362	a	1439.01	0.90698	YES	YES
363	a	1439.43	5.96598	YES	YES
364	a	1440.16	10.04165	YES	YES
365	a	1440.60	5.62710	YES	YES
366	a	1441.05	16.29894	YES	YES
367	a	1442.64	40.35143	YES	YES
368	a	1443.13	7.96470	YES	YES
369	a	1443.38	24.76266	YES	YES
370	a	1443.53	8.33493	YES	YES
371	a	1444.50	2.84819	YES	YES
372	a	1445.49	22.71556	YES	YES
373	a	1445.97	16.65999	YES	YES
374	a	1446.66	21.84109	YES	YES
375	a	1452.03	0.58467	YES	YES
376	a	1452.61	6.06900	YES	YES
377	a	1452.91	1.51470	YES	YES
378	a	1453.27	1.26798	YES	YES
379	a	1454.44	3.48251	YES	YES
380	a	1455.23	0.30224	YES	YES
381	a	1455.97	0.79971	YES	YES
382	a	1456.49	0.81234	YES	YES
383	a	1902.60	84.55765	YES	YES
384	a	2293.84	73.58213	YES	YES
385	a	2910.49	29.03188	YES	YES
386	a	2914.67	37.48746	YES	YES
387	a	2919.93	30.74067	YES	YES
388	a	2924.84	47.90194	YES	YES
389	a	2925.42	30.95678	YES	YES
390	a	2927.74	3.05506	YES	YES
391	a	2929.23	1.68929	YES	YES
392	a	2933.13	16.02234	YES	YES
393	a	2933.28	20.05101	YES	YES
394	a	2938.20	11.48184	YES	YES
395	a	2938.47	18.78221	YES	YES
396	a	2939.17	12.41519	YES	YES
397	a	2939.64	6.48630	YES	YES
398	a	2942.52	8.09812	YES	YES
399	a	2942.61	10.87667	YES	YES
400	a	2944.42	7.05634	YES	YES
401	a	2944.74	10.00884	YES	YES
402	a	2945.19	32.88438	YES	YES
403	a	2945.65	7.90562	YES	YES
404	a	2946.87	16.99671	YES	YES
405	a	2947.69	9.63728	YES	YES
406	a	2948.05	6.69941	YES	YES
407	a	2948.34	12.44489	YES	YES
408	a	2948.50	2.88506	YES	YES
409	a	2950.33	7.26610	YES	YES
410	a	2951.47	0.71774	YES	YES
411	a	2954.66	15.43545	YES	YES

412	a	2955.52	17.27139	YES	YES
413	a	2955.80	6.18407	YES	YES
414	a	2956.27	8.19542	YES	YES
415	a	2958.18	4.54051	YES	YES
416	a	2959.09	2.82199	YES	YES
417	a	2960.34	2.66822	YES	YES
418	a	2960.50	11.14519	YES	YES
419	a	2962.41	18.05911	YES	YES
420	a	2963.72	3.12539	YES	YES
421	a	2965.04	3.90529	YES	YES
422	a	2965.14	40.31762	YES	YES
423	a	2965.62	5.24588	YES	YES
424	a	2966.27	22.82924	YES	YES
425	a	2966.77	24.03822	YES	YES
426	a	2967.03	23.59368	YES	YES
427	a	2967.50	15.39093	YES	YES
428	a	2967.85	38.17229	YES	YES
429	a	2969.36	39.61260	YES	YES
430	a	2969.56	2.04423	YES	YES
431	a	2971.31	30.89530	YES	YES
432	a	2972.57	13.01138	YES	YES
433	a	2974.32	45.87219	YES	YES
434	a	2975.09	49.27940	YES	YES
435	a	2984.92	39.77438	YES	YES
436	a	2985.34	42.79219	YES	YES
437	a	2987.20	4.13205	YES	YES
438	a	2989.66	3.60730	YES	YES
439	a	2991.77	1.94811	YES	YES
440	a	2992.40	0.19540	YES	YES
441	a	2994.57	24.30164	YES	YES
442	a	2999.98	18.19253	YES	YES
443	a	3002.41	19.17149	YES	YES
444	a	3004.26	28.32797	YES	YES
445	a	3005.13	22.43274	YES	YES
446	a	3007.24	22.31279	YES	YES
447	a	3007.37	15.88113	YES	YES
448	a	3009.20	20.23619	YES	YES
449	a	3009.96	14.62171	YES	YES
450	a	3010.18	18.38728	YES	YES
451	a	3010.28	22.14357	YES	YES
452	a	3011.23	27.25405	YES	YES
453	a	3012.42	10.09669	YES	YES
454	a	3013.52	19.22856	YES	YES
455	a	3013.57	15.81879	YES	YES
456	a	3014.14	12.36752	YES	YES
457	a	3016.69	8.85541	YES	YES
458	a	3017.73	15.41752	YES	YES
459	a	3018.75	20.37128	YES	YES
460	a	3019.21	18.29665	YES	YES
461	a	3019.41	12.20671	YES	YES
462	a	3020.16	17.95252	YES	YES
463	a	3020.18	18.39108	YES	YES
464	a	3021.24	22.32831	YES	YES
465	a	3021.96	23.65811	YES	YES
466	a	3022.19	14.69546	YES	YES
467	a	3022.49	24.15391	YES	YES
468	a	3024.09	14.91420	YES	YES
469	a	3024.28	7.71537	YES	YES

470	a	3024.93	19.87233	YES	YES
471	a	3025.08	9.57162	YES	YES
472	a	3026.20	15.70653	YES	YES
473	a	3026.53	10.50150	YES	YES
474	a	3026.58	16.69208	YES	YES
475	a	3027.07	17.28520	YES	YES
476	a	3027.59	11.30923	YES	YES
477	a	3027.91	10.98590	YES	YES
478	a	3032.00	0.70264	YES	YES
479	a	3035.86	1.17796	YES	YES
480	a	3047.52	0.48495	YES	YES
481	a	3051.13	0.71917	YES	YES
482	a	3051.30	7.04220	YES	YES
483	a	3055.98	5.36226	YES	YES

$end

Double hybrid single point energy = -7381.932133400864 H
COSMO energy + OC correction = -7389.6084596274 H (in *o*DFB)

6.2.38 [{Ga(dcpe)}$_2$(NCCH$_2$)]$^+$

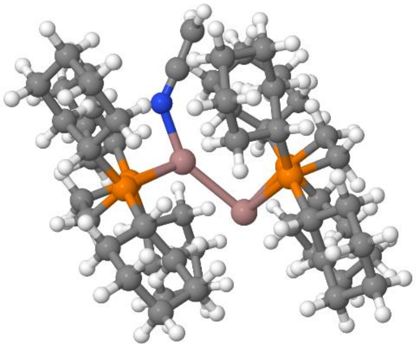

Method: (RI-)BP86(D3BJ)/def2-TZVPP
Symmetry: c1

Cartesian coordinates in Ångström:

H	4.2060833	-5.6009288	1.1513931
H	4.0229966	-5.2802880	-1.3350992
C	3.3302005	-5.0978517	0.7177284
C	3.6787217	-4.4951751	-0.6473819
H	2.5543454	-5.8712280	0.5900715
H	4.5167153	-3.7881496	-0.5297092
H	1.0212229	-4.5941597	-6.4578179
H	0.0171062	-5.0985900	-4.2459427
C	2.4787530	-3.7511810	-1.2411187
C	2.8166828	-4.0245416	1.6883741
H	-1.2251518	-5.2013259	-5.4953108
H	1.6741110	-4.4750775	-1.4600001
H	2.5554634	-4.4810394	2.6539925
C	-0.5593071	-4.5007027	-4.9718433
H	2.7479486	-3.2803840	-2.1980317
C	0.4052848	-3.8340341	-5.9572631
H	3.6283338	-3.3058132	1.8830673
H	1.9549549	-3.3182314	-4.5347350
C	1.2923718	-2.8047629	-5.2511637
C	1.6112783	-3.2803614	1.0922945

H	-0.1745078	-3.3301677	-6.7488095
H	1.9467604	-2.2960482	-5.9727607
C	1.9506606	-2.6869712	-0.2768648
H	0.7929533	-4.0074087	0.9507003
H	-1.3145092	-5.4116737	4.3053668
C	-1.3986275	-3.4695948	-4.2029216
H	-0.8752328	-4.3880955	2.0849318
H	2.7030959	-1.8888370	-0.1691378
H	-2.0458137	-3.9901724	-3.4843269
H	0.1446742	-3.0015747	-2.7717674
H	-1.3703905	-3.8612982	-1.1327771
H	-2.7237467	-4.7584672	0.7670995
H	0.7922893	-3.8067442	3.8393114
C	-1.9439268	-4.5744199	3.9608638
H	-2.8296512	-5.0218986	3.4867545
C	-0.4878409	-2.4550800	-3.4936339
H	-3.6973837	-4.6266492	-0.6963773
C	0.4525112	-1.7667689	-4.4984364
C	-1.1729691	-3.7483030	2.9273245
H	1.0540206	-2.2056936	-0.6953286
C	-3.1727822	-4.0354189	0.0727090
H	4.4162783	-1.3018952	-2.3549180
H	-2.0618971	-2.9363639	-4.9041765
C	0.0529536	-3.0477872	3.5285952
C	-2.0735585	-3.2046493	-0.5991225
H	2.6933787	-1.8983997	3.8614048
P	0.8633717	-1.9981208	2.2203513
H	-0.4581913	-3.8256907	6.1995879
H	1.0945737	-1.0427163	-3.9829764
C	2.2940450	-1.2115016	3.1002544
H	-0.1485808	-1.2025614	-5.2302408
C	4.0783944	-0.3144696	-2.0508787
H	-1.4913724	-2.6695898	0.1703693
H	3.0744252	-1.0805467	2.3391081
C	-2.3504213	-3.7151262	5.1602904
H	-2.8791880	-4.3230136	5.9075630
H	-1.8382560	-2.9701195	2.5171242
H	-3.2317204	-2.7041550	-2.3509970
C	-1.1227676	-3.0487896	5.7861181
H	4.8190521	0.4472174	-1.8242989
C	-2.6843869	-2.1694001	-1.5552663
C	-4.1798185	-3.1414486	0.8025276
C	-0.3471949	-2.2161837	4.7578233
H	-4.9746276	-3.7516369	1.2536883
C	2.7807635	-0.0157545	-2.0617903
P	-1.3631516	-1.2149193	-2.4260333
H	0.5460896	-1.7903793	5.2349620
N	1.6046199	0.2581612	-2.1318763
H	-3.6717034	-2.6186493	1.6290310
H	2.9710963	0.7748466	0.3776873
H	-3.0573407	-2.9369720	4.8265779
H	5.2433165	1.7041616	0.0570097
H	3.8632611	0.7847182	1.9028764
H	-5.3827416	-2.6052745	-0.9153626
C	1.9353250	0.1433884	3.7281892
H	-1.4173524	-2.4077880	6.6293297
C	-4.7801630	-2.0981537	-0.1431601
Ga	-0.8252823	-0.2256753	1.5087606

C	3.3969788	1.4346489	1.1501430
Ga	0.0665655	0.1735444	-0.8772133
C	-3.6878117	-1.2635992	-0.8228167
H	-0.9726789	-1.3707136	4.4233933
H	2.8451483	0.6710961	4.0486497
H	1.3154645	0.0002411	4.6215577
C	-2.3101422	-0.0987333	-3.5642758
H	-1.7029712	0.0020378	-4.4712799
H	0.7732505	1.1897627	-4.1926217
H	-3.2512355	-0.5867539	-3.8567416
C	4.4745497	2.3310643	0.5297647
H	1.3783415	2.0925004	-6.4144914
H	-3.1526900	-0.6684187	-0.0628736
H	2.9360876	2.6327317	-4.5364521
C	0.2074711	2.0403491	-4.5989886
P	0.9638362	1.2278347	2.5775225
H	-4.1540079	-0.5611026	-1.5274672
H	-0.6858165	1.6428920	-5.1009884
H	3.4679843	2.7441549	-1.3334190
H	-5.4640381	-1.4332128	0.4031143
H	4.9794493	2.8965008	1.3307371
C	1.0716844	2.7887932	-5.6210815
C	3.8775448	3.3115141	-0.4819713
C	2.2838411	2.2747487	1.7878012
C	2.2964595	3.4253169	-4.9588440
C	-2.5912880	1.2878626	-2.9592439
H	4.6598511	3.9676150	-0.8885027
H	1.6195116	2.8873628	-2.3026894
P	-1.1202920	2.0312887	-2.1125224
H	2.9009581	3.9588989	-5.7052925
H	2.7315574	2.8560840	2.6117037
H	1.1849742	2.6652831	-0.0196438
C	-0.2070560	2.9551926	-3.4335281
H	-0.5866486	1.0130345	5.1868529
H	-3.3791360	1.2201531	-2.1976940
C	1.6803359	3.2525479	0.7736948
H	0.4706590	3.5755977	-6.1073770
C	1.0066431	3.6524129	-2.8016886
H	-1.6767962	1.1598435	3.8052433
C	2.7629777	4.1457334	0.1566570
C	-1.0357224	1.7383755	4.4875067
C	0.0811171	2.4343600	3.6922246
H	-2.9522206	1.9774801	-3.7355364
C	1.8653375	4.3780139	-3.8412663
H	1.5010347	2.5788401	5.3206251
H	-1.9610391	1.9301750	0.6838582
H	2.7422273	4.8183618	-3.3451363
C	0.9785610	3.2590385	4.6276887
H	-0.9051546	3.7193719	-3.8178024
H	3.1923839	4.7843191	0.9462370
H	2.3161032	4.8253876	-0.5817820
H	0.9004595	3.8697175	1.2453506
C	-2.5660925	2.7079917	0.1911133
C	-1.7988581	3.3316232	-0.9844084
H	-0.4069270	3.1249035	2.9796029
H	0.6756214	4.3592780	-2.0281109
H	1.7542096	3.7932398	4.0625750
H	-3.4698970	2.1986540	-0.1827876

```
H    -0.8897697    3.8011800   -0.5697115
H     1.2928636    5.2159123   -4.2728628
C    -1.8620237    2.7527878    5.2854085
H    -0.5382746    2.9284334    6.9852597
H    -2.6404460    2.2293804    5.8587459
C    -0.9759351    3.5864901    6.2157850
C     0.1510372    4.2693823    5.4357030
C    -2.6359807    4.4225901   -1.6698698
C    -2.9784325    3.7808723    1.2030482
H    -2.3853076    3.4210743    4.5810483
H    -3.5347576    3.9656452   -2.1156235
H    -3.5383046    3.3170394    2.0275210
H    -2.0749697    4.8900171   -2.4909154
H    -2.0686106    4.2196413    1.6483531
H     0.8097881    4.8271915    6.1164334
H    -0.2824998    5.0095422    4.7422017
H    -1.5771472    4.3352064    6.7506211
C    -3.8039789    4.8870595    0.5386027
C    -3.0593814    5.4947058   -0.6550561
H    -4.7615602    4.4633070    0.1926000
H    -2.1580740    6.0185076   -0.2949672
H    -3.6821405    6.2510914   -1.1529724
H    -4.0548825    5.6699235    1.2680027

SCF energy GEOOPT =  -7388.658098428 H
ZPE =   3682. kJ/mol
FREEH energy =  3869.39 kJ/mol
FREEH entropy =   1.60240 kJ/mol/K

$vibrational spectrum
#  mode     symmetry     wave number   IR intensity    selection rules
#                         cm**(-1)        km/mol         IR     RAMAN
     1                        0.00         0.00000        -       -
     2                        0.00         0.00000        -       -
     3                        0.00         0.00000        -       -
     4                        0.00         0.00000        -       -
     5                        0.00         0.00000        -       -
     6                        0.00         0.00000        -       -
     7        a               8.36         0.01238       YES     YES
     8        a              19.00         0.03842       YES     YES
     9        a              21.11         0.01550       YES     YES
    10        a              24.33         0.10127       YES     YES
    11        a              27.97         0.03512       YES     YES
    12        a              30.10         0.04731       YES     YES
    13        a              33.07         0.04188       YES     YES
    14        a              34.60         0.01031       YES     YES
    15        a              37.59         0.02700       YES     YES
    16        a              41.20         0.02008       YES     YES
    17        a              46.03         0.13906       YES     YES
    18        a              47.63         0.02666       YES     YES
    19        a              49.19         0.23283       YES     YES
    20        a              51.77         0.00711       YES     YES
    21        a              53.27         0.15932       YES     YES
    22        a              55.57         0.07191       YES     YES
    23        a              58.31         0.21820       YES     YES
    24        a              62.30         0.30818       YES     YES
    25        a              65.75         0.09454       YES     YES
    26        a              66.60         0.35787       YES     YES
```

27	a	71.45	0.44835	YES	YES
28	a	74.66	0.11105	YES	YES
29	a	77.98	0.09238	YES	YES
30	a	81.38	0.29176	YES	YES
31	a	84.45	0.45010	YES	YES
32	a	87.27	0.14690	YES	YES
33	a	88.83	0.46635	YES	YES
34	a	93.36	0.06790	YES	YES
35	a	96.50	0.27750	YES	YES
36	a	101.77	0.14987	YES	YES
37	a	106.72	0.76448	YES	YES
38	a	120.35	1.90824	YES	YES
39	a	121.40	0.82824	YES	YES
40	a	127.46	4.48056	YES	YES
41	a	131.53	6.93188	YES	YES
42	a	136.71	0.67787	YES	YES
43	a	138.93	0.74282	YES	YES
44	a	142.08	4.89232	YES	YES
45	a	145.82	0.61032	YES	YES
46	a	148.88	0.53943	YES	YES
47	a	154.49	2.03495	YES	YES
48	a	156.55	0.89917	YES	YES
49	a	164.18	4.03260	YES	YES
50	a	171.23	0.23384	YES	YES
51	a	172.96	2.15207	YES	YES
52	a	176.13	10.76303	YES	YES
53	a	180.69	6.85801	YES	YES
54	a	193.71	1.71419	YES	YES
55	a	194.53	0.04865	YES	YES
56	a	194.89	0.64021	YES	YES
57	a	209.64	7.64374	YES	YES
58	a	211.66	0.18368	YES	YES
59	a	212.52	0.05689	YES	YES
60	a	216.36	0.16673	YES	YES
61	a	218.03	1.12153	YES	YES
62	a	225.19	0.48167	YES	YES
63	a	229.45	0.07234	YES	YES
64	a	231.71	0.01633	YES	YES
65	a	236.61	0.34023	YES	YES
66	a	238.00	0.14018	YES	YES
67	a	239.93	0.14477	YES	YES
68	a	241.84	0.22889	YES	YES
69	a	245.68	0.06159	YES	YES
70	a	250.36	1.01456	YES	YES
71	a	274.54	1.70342	YES	YES
72	a	279.37	4.69241	YES	YES
73	a	284.37	1.02950	YES	YES
74	a	285.47	1.03375	YES	YES
75	a	293.40	0.67302	YES	YES
76	a	299.99	0.79917	YES	YES
77	a	302.69	0.59879	YES	YES
78	a	315.34	0.54393	YES	YES
79	a	316.50	0.12198	YES	YES
80	a	329.57	0.82912	YES	YES
81	a	332.44	0.63706	YES	YES
82	a	334.43	0.44585	YES	YES
83	a	336.27	0.31422	YES	YES
84	a	339.17	2.12832	YES	YES

85	a	342.71	1.22910	YES	YES
86	a	353.02	49.68105	YES	YES
87	a	360.73	2.88552	YES	YES
88	a	368.01	1.93345	YES	YES
89	a	374.53	1.81363	YES	YES
90	a	376.48	1.03263	YES	YES
91	a	390.21	0.92831	YES	YES
92	a	390.42	0.13054	YES	YES
93	a	411.86	1.34120	YES	YES
94	a	414.79	1.02058	YES	YES
95	a	422.25	3.80011	YES	YES
96	a	423.12	5.86940	YES	YES
97	a	424.44	2.45413	YES	YES
98	a	428.96	1.14272	YES	YES
99	a	429.75	0.01456	YES	YES
100	a	430.75	0.05740	YES	YES
101	a	431.87	0.03494	YES	YES
102	a	432.91	2.96812	YES	YES
103	a	433.23	0.32998	YES	YES
104	a	433.55	1.10524	YES	YES
105	a	434.64	0.67169	YES	YES
106	a	434.74	0.79516	YES	YES
107	a	437.11	3.27061	YES	YES
108	a	441.76	7.93086	YES	YES
109	a	445.44	3.05305	YES	YES
110	a	465.99	3.05010	YES	YES
111	a	466.35	3.63454	YES	YES
112	a	490.56	9.18240	YES	YES
113	a	491.88	1.78813	YES	YES
114	a	492.68	3.07325	YES	YES
115	a	493.65	6.68367	YES	YES
116	a	500.34	0.38848	YES	YES
117	a	502.87	0.59134	YES	YES
118	a	511.12	12.14257	YES	YES
119	a	513.75	70.98069	YES	YES
120	a	515.02	8.71459	YES	YES
121	a	572.02	6.95182	YES	YES
122	a	622.63	17.29078	YES	YES
123	a	627.81	7.83157	YES	YES
124	a	641.54	15.99571	YES	YES
125	a	642.52	7.08046	YES	YES
126	a	704.01	0.54944	YES	YES
127	a	712.65	0.77736	YES	YES
128	a	714.51	5.12142	YES	YES
129	a	722.67	2.65273	YES	YES
130	a	727.97	7.06944	YES	YES
131	a	731.40	3.87697	YES	YES
132	a	731.56	11.70064	YES	YES
133	a	734.71	9.81970	YES	YES
134	a	772.18	0.46728	YES	YES
135	a	774.13	0.17985	YES	YES
136	a	774.27	0.61226	YES	YES
137	a	774.94	0.40276	YES	YES
138	a	776.26	1.64375	YES	YES
139	a	776.67	0.14034	YES	YES
140	a	777.17	0.31517	YES	YES
141	a	777.27	0.42708	YES	YES
142	a	780.26	35.73009	YES	YES

143	a	780.98	0.37201	YES	YES
144	a	809.85	0.11112	YES	YES
145	a	811.51	1.78503	YES	YES
146	a	811.70	0.85294	YES	YES
147	a	812.63	0.82061	YES	YES
148	a	814.26	1.19354	YES	YES
149	a	814.88	0.42464	YES	YES
150	a	815.21	5.70451	YES	YES
151	a	816.70	4.88090	YES	YES
152	a	833.40	1.28291	YES	YES
153	a	833.89	3.43393	YES	YES
154	a	836.50	4.36143	YES	YES
155	a	837.19	1.50411	YES	YES
156	a	837.94	2.84639	YES	YES
157	a	838.50	5.35820	YES	YES
158	a	839.89	13.58608	YES	YES
159	a	840.31	10.82938	YES	YES
160	a	851.51	28.70672	YES	YES
161	a	853.08	0.20090	YES	YES
162	a	876.66	2.79129	YES	YES
163	a	876.85	4.11505	YES	YES
164	a	877.49	2.20812	YES	YES
165	a	877.71	3.01792	YES	YES
166	a	878.92	3.68171	YES	YES
167	a	879.43	2.78543	YES	YES
168	a	879.91	3.08613	YES	YES
169	a	880.38	2.46198	YES	YES
170	a	881.15	1.03925	YES	YES
171	a	881.54	8.96426	YES	YES
172	a	882.11	2.93745	YES	YES
173	a	882.76	2.11941	YES	YES
174	a	883.55	1.68606	YES	YES
175	a	885.25	1.62257	YES	YES
176	a	887.17	2.25474	YES	YES
177	a	889.60	1.11989	YES	YES
178	a	902.54	2.89789	YES	YES
179	a	903.36	0.97476	YES	YES
180	a	903.87	3.85706	YES	YES
181	a	904.40	1.08880	YES	YES
182	a	908.70	1.07625	YES	YES
183	a	909.60	1.73332	YES	YES
184	a	910.98	0.87330	YES	YES
185	a	912.65	1.57215	YES	YES
186	a	960.07	0.17067	YES	YES
187	a	981.97	0.15206	YES	YES
188	a	985.81	0.55182	YES	YES
189	a	989.67	4.98939	YES	YES
190	a	989.97	13.93376	YES	YES
191	a	990.22	10.68666	YES	YES
192	a	990.63	5.04033	YES	YES
193	a	991.52	3.96756	YES	YES
194	a	992.42	17.53034	YES	YES
195	a	993.69	7.63572	YES	YES
196	a	994.84	2.72562	YES	YES
197	a	1016.02	1.58893	YES	YES
198	a	1017.46	0.60827	YES	YES
199	a	1017.83	1.53613	YES	YES
200	a	1019.20	0.80389	YES	YES

201	a	1020.19	0.29597	YES	YES
202	a	1021.95	0.65387	YES	YES
203	a	1024.17	0.39784	YES	YES
204	a	1025.04	0.01570	YES	YES
205	a	1031.45	1.92852	YES	YES
206	a	1032.64	0.61037	YES	YES
207	a	1033.48	0.65147	YES	YES
208	a	1034.01	2.21483	YES	YES
209	a	1039.34	2.69002	YES	YES
210	a	1039.68	1.23939	YES	YES
211	a	1040.39	0.26867	YES	YES
212	a	1041.74	1.10701	YES	YES
213	a	1058.01	3.33407	YES	YES
214	a	1058.55	1.08899	YES	YES
215	a	1058.81	1.04893	YES	YES
216	a	1059.32	1.07407	YES	YES
217	a	1063.46	3.25100	YES	YES
218	a	1066.15	0.68848	YES	YES
219	a	1067.78	0.11300	YES	YES
220	a	1070.61	0.00755	YES	YES
221	a	1071.29	0.09451	YES	YES
222	a	1072.69	0.22468	YES	YES
223	a	1072.95	0.04536	YES	YES
224	a	1074.15	0.60789	YES	YES
225	a	1074.59	0.37788	YES	YES
226	a	1075.94	0.61511	YES	YES
227	a	1076.78	0.56102	YES	YES
228	a	1077.92	0.29003	YES	YES
229	a	1079.90	1.09534	YES	YES
230	a	1081.54	1.96213	YES	YES
231	a	1088.61	3.67067	YES	YES
232	a	1091.87	16.37181	YES	YES
233	a	1094.23	4.50247	YES	YES
234	a	1098.77	3.00091	YES	YES
235	a	1101.23	1.54035	YES	YES
236	a	1102.36	0.57925	YES	YES
237	a	1105.29	2.03966	YES	YES
238	a	1107.01	10.39354	YES	YES
239	a	1120.78	10.76792	YES	YES
240	a	1124.04	2.56564	YES	YES
241	a	1159.32	3.44152	YES	YES
242	a	1159.47	4.61985	YES	YES
243	a	1159.89	15.41339	YES	YES
244	a	1160.55	3.60660	YES	YES
245	a	1161.27	4.64051	YES	YES
246	a	1167.56	7.40924	YES	YES
247	a	1168.27	4.00385	YES	YES
248	a	1170.32	12.27034	YES	YES
249	a	1171.00	3.04172	YES	YES
250	a	1174.51	10.30460	YES	YES
251	a	1176.92	11.19188	YES	YES
252	a	1180.01	12.84548	YES	YES
253	a	1183.60	7.20032	YES	YES
254	a	1190.60	1.88566	YES	YES
255	a	1193.31	2.98764	YES	YES
256	a	1193.45	0.68197	YES	YES
257	a	1197.08	2.33382	YES	YES
258	a	1232.77	0.83812	YES	YES

259	a	1235.13	0.41073	YES	YES
260	a	1244.01	1.30001	YES	YES
261	a	1244.34	0.18825	YES	YES
262	a	1245.39	0.38450	YES	YES
263	a	1246.75	0.88597	YES	YES
264	a	1247.81	0.17648	YES	YES
265	a	1248.17	0.17837	YES	YES
266	a	1249.35	1.96108	YES	YES
267	a	1251.31	0.21794	YES	YES
268	a	1252.98	7.56795	YES	YES
269	a	1253.47	0.37801	YES	YES
270	a	1255.13	6.51139	YES	YES
271	a	1256.24	2.34094	YES	YES
272	a	1256.40	2.65545	YES	YES
273	a	1256.78	1.70170	YES	YES
274	a	1257.83	1.01738	YES	YES
275	a	1258.21	1.64767	YES	YES
276	a	1259.18	1.67558	YES	YES
277	a	1259.35	0.96178	YES	YES
278	a	1260.28	5.34496	YES	YES
279	a	1261.19	1.94981	YES	YES
280	a	1261.65	1.58937	YES	YES
281	a	1262.82	0.46980	YES	YES
282	a	1265.75	3.40902	YES	YES
283	a	1268.40	0.30481	YES	YES
284	a	1269.33	1.83063	YES	YES
285	a	1272.29	2.08553	YES	YES
286	a	1280.33	1.84192	YES	YES
287	a	1282.54	0.55252	YES	YES
288	a	1284.96	2.35444	YES	YES
289	a	1285.89	0.55464	YES	YES
290	a	1289.84	1.19984	YES	YES
291	a	1291.17	3.59592	YES	YES
292	a	1292.13	2.13917	YES	YES
293	a	1293.55	2.38058	YES	YES
294	a	1309.03	1.67912	YES	YES
295	a	1309.40	1.83804	YES	YES
296	a	1312.83	1.58024	YES	YES
297	a	1313.18	0.57929	YES	YES
298	a	1314.71	0.77900	YES	YES
299	a	1317.88	0.84639	YES	YES
300	a	1319.33	3.00659	YES	YES
301	a	1320.31	2.49449	YES	YES
302	a	1321.27	0.41242	YES	YES
303	a	1321.50	0.09460	YES	YES
304	a	1322.15	0.58538	YES	YES
305	a	1322.48	0.07224	YES	YES
306	a	1322.65	1.03679	YES	YES
307	a	1323.25	1.70501	YES	YES
308	a	1323.78	0.61461	YES	YES
309	a	1324.25	0.25662	YES	YES
310	a	1325.43	1.11881	YES	YES
311	a	1326.42	1.79149	YES	YES
312	a	1327.38	3.83447	YES	YES
313	a	1327.52	8.55380	YES	YES
314	a	1328.18	5.57987	YES	YES
315	a	1329.26	1.02985	YES	YES
316	a	1330.31	0.55438	YES	YES

317	a	1332.68	0.06767	YES	YES
318	a	1333.07	0.16055	YES	YES
319	a	1334.25	0.59946	YES	YES
320	a	1334.62	0.79896	YES	YES
321	a	1335.78	0.03913	YES	YES
322	a	1336.51	0.29498	YES	YES
323	a	1337.53	1.82755	YES	YES
324	a	1339.09	0.80082	YES	YES
325	a	1340.24	1.38907	YES	YES
326	a	1341.05	1.54172	YES	YES
327	a	1345.57	2.35582	YES	YES
328	a	1346.62	0.33136	YES	YES
329	a	1346.71	0.05409	YES	YES
330	a	1347.02	2.21338	YES	YES
331	a	1348.91	0.87945	YES	YES
332	a	1349.23	1.20449	YES	YES
333	a	1349.58	0.10978	YES	YES
334	a	1381.53	11.58657	YES	YES
335	a	1400.73	8.18573	YES	YES
336	a	1401.48	4.46362	YES	YES
337	a	1407.51	8.33116	YES	YES
338	a	1407.85	3.92834	YES	YES
339	a	1424.39	2.53724	YES	YES
340	a	1426.36	6.61957	YES	YES
341	a	1428.55	3.38746	YES	YES
342	a	1429.70	7.51180	YES	YES
343	a	1429.87	0.65096	YES	YES
344	a	1431.57	2.96681	YES	YES
345	a	1433.02	1.17213	YES	YES
346	a	1433.32	0.55123	YES	YES
347	a	1434.78	4.99678	YES	YES
348	a	1435.08	0.57740	YES	YES
349	a	1435.51	0.64270	YES	YES
350	a	1435.79	5.35100	YES	YES
351	a	1436.02	4.80703	YES	YES
352	a	1436.61	7.22181	YES	YES
353	a	1436.80	1.99155	YES	YES
354	a	1437.40	2.10805	YES	YES
355	a	1437.68	3.57367	YES	YES
356	a	1437.80	5.32060	YES	YES
357	a	1438.41	3.23729	YES	YES
358	a	1438.83	4.03039	YES	YES
359	a	1439.31	3.22146	YES	YES
360	a	1439.41	18.67496	YES	YES
361	a	1439.82	2.53694	YES	YES
362	a	1441.67	10.62463	YES	YES
363	a	1442.12	11.78374	YES	YES
364	a	1442.50	10.89927	YES	YES
365	a	1442.83	30.39603	YES	YES
366	a	1444.20	1.92727	YES	YES
367	a	1444.68	12.77868	YES	YES
368	a	1445.99	4.11053	YES	YES
369	a	1446.48	20.31315	YES	YES
370	a	1447.58	12.26820	YES	YES
371	a	1452.72	3.23056	YES	YES
372	a	1453.31	0.06922	YES	YES
373	a	1453.56	1.51732	YES	YES
374	a	1454.32	4.54150	YES	YES

375	a	1455.17	1.18986	YES	YES
376	a	1456.17	1.28453	YES	YES
377	a	1456.71	0.75146	YES	YES
378	a	1460.50	0.47860	YES	YES
379	a	2071.47	607.14620	YES	YES
380	a	2916.01	1.34859	YES	YES
381	a	2919.60	6.58325	YES	YES
382	a	2920.22	13.32571	YES	YES
383	a	2926.75	20.16079	YES	YES
384	a	2926.95	5.97164	YES	YES
385	a	2927.02	1.45124	YES	YES
386	a	2928.96	11.26390	YES	YES
387	a	2931.02	14.91110	YES	YES
388	a	2931.12	16.10228	YES	YES
389	a	2933.48	3.37728	YES	YES
390	a	2934.71	19.34369	YES	YES
391	a	2935.65	5.73534	YES	YES
392	a	2936.65	8.26467	YES	YES
393	a	2938.12	6.56213	YES	YES
394	a	2938.42	18.80177	YES	YES
395	a	2939.18	11.50739	YES	YES
396	a	2939.36	4.99288	YES	YES
397	a	2939.87	13.65561	YES	YES
398	a	2940.19	13.90757	YES	YES
399	a	2940.49	27.38354	YES	YES
400	a	2941.27	11.31462	YES	YES
401	a	2941.74	41.21185	YES	YES
402	a	2941.94	9.98226	YES	YES
403	a	2943.31	18.40214	YES	YES
404	a	2943.54	0.06405	YES	YES
405	a	2943.62	16.55639	YES	YES
406	a	2943.99	12.47996	YES	YES
407	a	2944.33	26.11666	YES	YES
408	a	2945.13	16.17696	YES	YES
409	a	2947.03	12.08899	YES	YES
410	a	2947.99	6.46032	YES	YES
411	a	2948.06	10.74664	YES	YES
412	a	2949.41	7.62176	YES	YES
413	a	2950.34	6.03208	YES	YES
414	a	2950.88	16.16445	YES	YES
415	a	2951.43	3.15391	YES	YES
416	a	2951.84	14.32265	YES	YES
417	a	2952.60	61.03961	YES	YES
418	a	2952.92	33.50585	YES	YES
419	a	2954.00	25.87531	YES	YES
420	a	2954.67	17.22058	YES	YES
421	a	2956.77	19.18730	YES	YES
422	a	2957.38	16.68750	YES	YES
423	a	2957.86	13.14760	YES	YES
424	a	2957.93	78.36515	YES	YES
425	a	2962.72	15.36136	YES	YES
426	a	2966.94	20.56729	YES	YES
427	a	2967.74	18.76208	YES	YES
428	a	2968.51	16.77617	YES	YES
429	a	2970.85	32.89411	YES	YES
430	a	2977.03	9.23395	YES	YES
431	a	2979.82	8.64386	YES	YES
432	a	2981.06	31.81130	YES	YES

433	a	2981.72	16.82592	YES	YES
434	a	2982.58	28.23065	YES	YES
435	a	2982.97	45.69970	YES	YES
436	a	2990.08	28.67169	YES	YES
437	a	2992.50	16.88197	YES	YES
438	a	2995.59	35.79485	YES	YES
439	a	2998.04	36.79890	YES	YES
440	a	2999.55	29.08118	YES	YES
441	a	2999.88	20.12157	YES	YES
442	a	3000.24	11.78404	YES	YES
443	a	3000.42	32.84976	YES	YES
444	a	3000.75	13.51836	YES	YES
445	a	3001.12	32.71348	YES	YES
446	a	3001.21	31.82124	YES	YES
447	a	3001.43	68.81630	YES	YES
448	a	3001.93	54.06008	YES	YES
449	a	3002.55	39.83597	YES	YES
450	a	3003.21	49.24012	YES	YES
451	a	3003.37	18.11529	YES	YES
452	a	3003.95	11.83994	YES	YES
453	a	3004.23	80.80571	YES	YES
454	a	3004.42	11.45543	YES	YES
455	a	3004.55	11.95875	YES	YES
456	a	3004.69	54.68585	YES	YES
457	a	3005.26	52.83435	YES	YES
458	a	3006.62	39.24522	YES	YES
459	a	3006.84	8.18055	YES	YES
460	a	3007.14	30.71010	YES	YES
461	a	3007.22	26.21482	YES	YES
462	a	3007.54	46.73533	YES	YES
463	a	3007.62	67.23811	YES	YES
464	a	3007.76	41.44485	YES	YES
465	a	3007.95	18.85769	YES	YES
466	a	3009.19	39.24496	YES	YES
467	a	3009.75	31.14506	YES	YES
468	a	3012.36	34.58718	YES	YES
469	a	3015.30	19.37585	YES	YES
470	a	3020.17	10.39010	YES	YES
471	a	3022.77	4.15877	YES	YES
472	a	3027.48	1.27586	YES	YES
473	a	3033.57	3.04408	YES	YES
474	a	3041.89	1.99891	YES	YES
475	a	3048.10	0.73069	YES	YES
476	a	3103.00	5.09220	YES	YES
477	a	3187.44	0.04737	YES	YES

$end

Double hybrid single point energy = -7381.294959206796 H
COSMO energy + OC correction = -7388.7022664540 H (in *o*DFB)

6.2.39 [H{Ga(dcpe)}$_2$(NCCH$_2$)]$^{2+}$

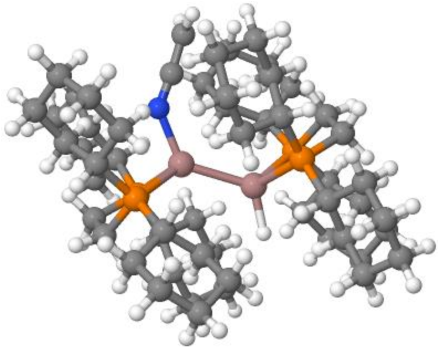

Method: (RI-)BP86(D3BJ)/def2-TZVPP
Symmetry: c1

Cartesian coordinates in Ångström:

H	4.2808739	-5.5815338	1.1275304
H	4.0255017	-5.2702199	-1.3525707
C	3.3871276	-5.0937917	0.7160191
C	3.6896304	-4.4885499	-0.6583477
H	2.6200074	-5.8787684	0.6141868
H	4.5217501	-3.7712153	-0.5657639
H	0.9324843	-4.6628959	-6.4982749
H	-0.0866674	-5.1460991	-4.2890946
C	2.4626207	-3.7670745	-1.2238067
C	2.8905605	-4.0250999	1.6999639
H	-1.3290385	-5.2166275	-5.5390809
H	1.6644827	-4.5039059	-1.4190590
H	2.6610180	-4.4813158	2.6737225
C	-0.6461978	-4.5336716	-5.0158288
H	2.6977445	-3.2950717	-2.1888206
C	0.3333964	-3.8888744	-6.0003778
H	3.6976822	-3.2948127	1.8675227
H	1.8953986	-3.4077400	-4.5778907
C	1.2448231	-2.8805891	-5.2954722
C	1.6568125	-3.3019192	1.1309864
H	-0.2323918	-3.3742870	-6.7944415
H	1.9102648	-2.3876027	-6.0170619
C	1.9396208	-2.7060197	-0.2521244
H	0.8414114	-4.0381977	1.0265083
H	-1.4143390	-5.3187375	4.2970336
C	-1.4649815	-3.4837362	-4.2496173
H	-0.9145987	-4.2927477	2.0935572
H	2.6788694	-1.8925275	-0.1695486
H	-2.1242180	-3.9871244	-3.5294891
H	0.0879860	-3.0450000	-2.8128607
H	-1.4252077	-3.8578895	-1.1491058
H	-2.7604270	-4.6998883	0.7880314
H	0.7447151	-3.8014026	3.8857672
C	-2.0088371	-4.4550342	3.9584475
H	-2.9047428	-4.8638883	3.4706601
C	-0.5273197	-2.4895912	-3.5431404
H	-3.7562140	-4.5539134	-0.6549581
C	0.4298532	-1.8194185	-4.5458556
C	-1.1992586	-3.6491814	2.9373576
H	1.0183905	-2.2540261	-0.6500673
C	-3.2039313	-3.9694224	0.0984081

H	4.4685316	-1.3183699	-2.1657596
H	-2.1138423	-2.9364570	-4.9529429
C	0.0449221	-3.0072406	3.5699659
C	-2.0956567	-3.1764458	-0.6055113
H	2.8045863	-1.8777823	3.8623810
P	0.9518924	-2.0397490	2.2827303
H	-0.5175986	-3.7770239	6.2219574
H	1.0901088	-1.1094700	-4.0322082
C	2.3694351	-1.1981924	3.1166065
H	-0.1575387	-1.2438384	-5.2793939
C	4.1038600	-0.3297823	-1.8970940
H	-1.4788286	-2.6523891	0.1451268
H	3.1296641	-1.0466561	2.3382286
C	-2.3942818	-3.5923403	5.1620827
H	-2.9478778	-4.1895867	5.8984106
H	-1.8307683	-2.8441829	2.5288139
H	-3.2908570	-2.6670316	-2.3273506
C	-1.1509380	-2.9761859	5.8073584
H	4.8260638	0.4439491	-1.6511649
C	-2.7054690	-2.1356970	-1.5570053
C	-4.1752899	-3.0455499	0.8393094
C	-0.3328345	-2.1613977	4.7964637
H	-4.9811753	-3.6330881	1.2988940
C	2.8145714	-0.0409266	-2.0034095
P	-1.4010638	-1.2405439	-2.4994409
H	0.5712288	-1.7769995	5.2883485
N	1.6447601	0.2294497	-2.1746737
H	-3.6464425	-2.5392127	1.6632394
H	3.0491627	0.8168773	0.4394127
H	-3.0743111	-2.7885312	4.8332616
H	5.2900726	1.8217706	0.1180168
H	3.9344388	0.8782901	1.9711158
H	-5.3949778	-2.4737513	-0.8545007
C	1.9980276	0.1440617	3.7720741
H	-1.4299360	-2.3309651	6.6516105
C	-4.7605203	-1.9860796	-0.0968910
Ga	-0.2553249	-0.2067347	1.2690768
C	3.4490231	1.5000278	1.2066528
Ga	0.0257186	0.1844336	-1.1368210
C	-3.6586396	-1.1895878	-0.8079335
H	-0.9243029	-1.2926622	4.4636777
H	2.9063765	0.6611700	4.1114372
H	1.3726623	-0.0183165	4.6580177
C	-2.3116077	-0.0897311	-3.6224151
H	-1.6970815	0.0039654	-4.5255238
H	0.7560734	1.1902861	-4.2990440
H	-3.2581567	-0.5599775	-3.9241712
C	4.4963950	2.4264994	0.5769272
H	1.3405369	2.1180309	-6.5135456
H	-3.0879399	-0.6024575	-0.0692548
H	2.9456264	2.6012965	-4.6607672
C	0.1989381	2.0585227	-4.6811250
P	1.0460185	1.2712768	2.6597773
H	-4.1224035	-0.4803840	-1.5064294
H	-0.7104714	1.6879026	-5.1747065
H	3.4852999	2.7847094	-1.2983493
H	-5.4114274	-1.2974874	0.4590257
H	4.9782377	3.0165504	1.3731940

C	1.0616638	2.8076410	-5.7051059
C	3.8698212	3.3730404	-0.4490769
C	2.3083557	2.3209607	1.8258346
C	2.3086481	3.4108885	-5.0539484
C	-2.5808765	1.2999539	-3.0166718
H	4.6317950	4.0504729	-0.8566521
H	1.6670664	2.8407339	-2.3902840
P	-1.1141069	2.0646267	-2.1924078
H	2.9056651	3.9483900	-5.8023759
H	2.7353527	2.9389121	2.6344809
H	1.2037686	2.6393234	-0.0015052
C	-0.1753729	2.9696349	-3.4978530
H	-0.6348710	0.9153824	5.1529854
H	-3.3669228	1.2435319	-2.2524729
C	1.6659805	3.2550727	0.7901175
H	0.4659099	3.6105826	-6.1691692
C	1.0624364	3.6268044	-2.8676122
H	-1.6831468	1.0756793	3.7370063
C	2.7245623	4.1772197	0.1729274
C	-1.0784262	1.6455304	4.4563059
C	0.0476060	2.3997280	3.7287167
H	-2.9428146	1.9848092	-3.7960898
C	1.9175367	4.3516249	-3.9120670
H	1.4091796	2.5371883	5.4109185
H	-1.6204342	2.0062996	0.7013631
H	2.8110905	4.7655060	-3.4240673
C	0.8953872	3.2214112	4.7160455
H	-0.8593705	3.7573227	-3.8599807
H	3.1240028	4.8380551	0.9589337
H	2.2602343	4.8339380	-0.5741637
H	0.8599265	3.8489763	1.2463591
C	-2.3594411	2.6782968	0.2397764
C	-1.7677360	3.3288589	-1.0198350
H	-0.4222234	3.0907965	3.0060538
H	0.7571957	4.3299489	-2.0804684
H	1.6749054	3.7892182	4.1901475
H	-3.2222771	2.0482395	-0.0323662
H	-0.8660718	3.8879408	-0.7156724
H	1.3549090	5.2087748	-4.3161032
C	-1.9566464	2.6188357	5.2528197
H	-0.6972895	2.7811673	7.0037000
H	-2.7386108	2.0575764	5.7823782
C	-1.1249526	3.4470709	6.2360051
C	0.0069342	4.1851383	5.5164451
C	-2.7583436	4.3155715	-1.6619379
C	-2.8066797	3.7430393	1.2470556
H	-2.4739000	3.2924335	4.5497289
H	-3.6449614	3.7651030	-2.0154131
H	-3.2538987	3.2556772	2.1248738
H	-2.3109199	4.8007626	-2.5403831
H	-1.9174624	4.2901044	1.6059049
H	0.6264839	4.7395299	6.2341847
H	-0.4201800	4.9327828	4.8278209
H	-1.7656548	4.1627391	6.7680742
C	-3.7887001	4.7357021	0.6172708
C	-3.1973874	5.3759871	-0.6422661
H	-4.7209256	4.2079236	0.3569574
H	-2.3265260	5.9939736	-0.3682474

```
H    -3.9257426    6.0528731   -1.1083457
H    -4.0635765    5.5113107    1.3444603
H    -1.7060317   -0.2532995    1.8807152

SCF energy GEOOPT =  -7389.022242704 H
ZPE =   3707. kJ/mol
FREEH energy =  3894.43 kJ/mol
FREEH entropy =   1.60092 kJ/mol/K

$vibrational spectrum
#  mode     symmetry     wave number   IR intensity    selection rules
#                         cm**(-1)        km/mol          IR     RAMAN
     1                      -0.00         0.00000          -       -
     2                      -0.00         0.00000          -       -
     3                      -0.00         0.00000          -       -
     4                      -0.00         0.00000          -       -
     5                       0.00         0.00000          -       -
     6                       0.00         0.00000          -       -
     7         a            11.23         0.00549        YES     YES
     8         a            16.73         0.03494        YES     YES
     9         a            17.88         0.07428        YES     YES
    10         a            21.85         0.01761        YES     YES
    11         a            25.42         0.05421        YES     YES
    12         a            26.91         0.08986        YES     YES
    13         a            32.98         0.02932        YES     YES
    14         a            33.59         0.05959        YES     YES
    15         a            37.61         0.01558        YES     YES
    16         a            41.79         0.04525        YES     YES
    17         a            43.54         0.00885        YES     YES
    18         a            47.29         0.02278        YES     YES
    19         a            48.13         0.38920        YES     YES
    20         a            49.19         0.00324        YES     YES
    21         a            52.65         0.10861        YES     YES
    22         a            53.09         0.00872        YES     YES
    23         a            57.26         0.22952        YES     YES
    24         a            59.32         0.01888        YES     YES
    25         a            62.58         0.38008        YES     YES
    26         a            65.81         0.19576        YES     YES
    27         a            73.03         0.20475        YES     YES
    28         a            74.80         0.43853        YES     YES
    29         a            78.65         0.14656        YES     YES
    30         a            81.49         0.22634        YES     YES
    31         a            87.31         0.66956        YES     YES
    32         a            88.65         0.13545        YES     YES
    33         a            91.68         0.29426        YES     YES
    34         a            94.17         0.53041        YES     YES
    35         a            95.81         0.13429        YES     YES
    36         a           101.75         0.19909        YES     YES
    37         a           109.38         0.41685        YES     YES
    38         a           126.00         1.55360        YES     YES
    39         a           127.58         8.90352        YES     YES
    40         a           131.65         1.63863        YES     YES
    41         a           136.24         0.16664        YES     YES
    42         a           141.77         0.87605        YES     YES
    43         a           143.73         0.18714        YES     YES
    44         a           146.83         2.53194        YES     YES
    45         a           148.83         0.46852        YES     YES
    46         a           151.17         1.70521        YES     YES
```

47	a	156.97	0.06847	YES	YES
48	a	159.67	2.46826	YES	YES
49	a	169.27	5.69423	YES	YES
50	a	180.83	2.52538	YES	YES
51	a	182.13	10.84525	YES	YES
52	a	185.09	4.42864	YES	YES
53	a	187.43	1.24288	YES	YES
54	a	193.08	0.69328	YES	YES
55	a	196.94	0.57825	YES	YES
56	a	203.83	5.02741	YES	YES
57	a	210.58	1.63557	YES	YES
58	a	212.36	1.00021	YES	YES
59	a	214.69	1.19107	YES	YES
60	a	216.33	0.81127	YES	YES
61	a	225.89	0.18995	YES	YES
62	a	227.91	3.45663	YES	YES
63	a	229.56	0.03693	YES	YES
64	a	235.20	0.61070	YES	YES
65	a	238.32	0.39008	YES	YES
66	a	239.99	0.13003	YES	YES
67	a	242.50	0.59175	YES	YES
68	a	244.11	0.09898	YES	YES
69	a	251.95	0.37591	YES	YES
70	a	256.47	0.70663	YES	YES
71	a	277.76	1.09913	YES	YES
72	a	282.84	1.13215	YES	YES
73	a	289.95	2.34569	YES	YES
74	a	296.79	0.86468	YES	YES
75	a	305.43	2.75385	YES	YES
76	a	305.89	0.31828	YES	YES
77	a	307.60	4.61582	YES	YES
78	a	316.66	0.60533	YES	YES
79	a	318.71	0.26249	YES	YES
80	a	329.87	0.46371	YES	YES
81	a	331.85	0.59624	YES	YES
82	a	336.51	0.36422	YES	YES
83	a	337.23	0.62103	YES	YES
84	a	345.75	0.45298	YES	YES
85	a	347.88	0.35925	YES	YES
86	a	372.42	3.41652	YES	YES
87	a	374.63	0.70831	YES	YES
88	a	377.99	1.09743	YES	YES
89	a	379.28	2.39736	YES	YES
90	a	394.69	1.85452	YES	YES
91	a	396.69	0.68171	YES	YES
92	a	403.41	20.83287	YES	YES
93	a	411.22	1.79757	YES	YES
94	a	414.61	1.41675	YES	YES
95	a	420.78	3.20638	YES	YES
96	a	423.03	4.92628	YES	YES
97	a	424.38	0.55701	YES	YES
98	a	428.33	0.64816	YES	YES
99	a	429.15	0.31117	YES	YES
100	a	429.62	0.04556	YES	YES
101	a	430.79	0.10780	YES	YES
102	a	432.00	0.73518	YES	YES
103	a	433.18	0.28211	YES	YES
104	a	433.76	0.52425	YES	YES

105	a	433.84	1.58996	YES	YES
106	a	436.07	1.26474	YES	YES
107	a	438.13	5.42644	YES	YES
108	a	445.43	4.64664	YES	YES
109	a	446.80	2.03624	YES	YES
110	a	466.90	2.40034	YES	YES
111	a	467.78	4.26567	YES	YES
112	a	491.79	4.77139	YES	YES
113	a	493.13	1.09442	YES	YES
114	a	494.52	2.33593	YES	YES
115	a	495.97	2.44535	YES	YES
116	a	503.07	1.88400	YES	YES
117	a	505.37	0.79286	YES	YES
118	a	516.27	22.51812	YES	YES
119	a	519.21	9.61455	YES	YES
120	a	540.57	41.25714	YES	YES
121	a	563.02	97.48711	YES	YES
122	a	572.62	61.92296	YES	YES
123	a	593.76	52.03943	YES	YES
124	a	626.15	19.59199	YES	YES
125	a	631.31	9.86985	YES	YES
126	a	645.01	9.07462	YES	YES
127	a	649.15	8.93380	YES	YES
128	a	712.30	0.21430	YES	YES
129	a	714.96	0.44816	YES	YES
130	a	721.76	1.78918	YES	YES
131	a	723.69	1.67458	YES	YES
132	a	733.95	5.40887	YES	YES
133	a	736.42	4.25239	YES	YES
134	a	737.96	5.74549	YES	YES
135	a	738.60	8.53023	YES	YES
136	a	770.27	0.36371	YES	YES
137	a	773.76	0.11463	YES	YES
138	a	775.13	0.31709	YES	YES
139	a	775.63	0.13919	YES	YES
140	a	776.48	0.46993	YES	YES
141	a	777.00	0.15693	YES	YES
142	a	777.14	0.89919	YES	YES
143	a	777.47	0.02707	YES	YES
144	a	783.60	16.93783	YES	YES
145	a	787.14	8.95613	YES	YES
146	a	810.44	0.33068	YES	YES
147	a	810.66	0.52262	YES	YES
148	a	811.29	2.37975	YES	YES
149	a	812.31	1.07693	YES	YES
150	a	813.64	0.50099	YES	YES
151	a	815.25	1.04574	YES	YES
152	a	816.92	6.08378	YES	YES
153	a	817.77	5.30200	YES	YES
154	a	833.11	1.31824	YES	YES
155	a	834.71	0.67090	YES	YES
156	a	835.45	3.75664	YES	YES
157	a	837.25	0.41494	YES	YES
158	a	837.63	0.51257	YES	YES
159	a	837.85	5.65617	YES	YES
160	a	839.89	10.35759	YES	YES
161	a	840.18	13.57023	YES	YES
162	a	852.96	16.12540	YES	YES

163	a	854.85	3.53062	YES	YES
164	a	875.15	3.41515	YES	YES
165	a	875.46	2.04287	YES	YES
166	a	876.13	2.89803	YES	YES
167	a	876.97	4.97362	YES	YES
168	a	877.74	3.12065	YES	YES
169	a	878.32	4.56333	YES	YES
170	a	878.95	3.69168	YES	YES
171	a	879.94	1.90662	YES	YES
172	a	880.00	1.58368	YES	YES
173	a	880.80	3.02540	YES	YES
174	a	882.19	3.45581	YES	YES
175	a	883.34	1.21510	YES	YES
176	a	883.62	1.86816	YES	YES
177	a	885.04	3.92701	YES	YES
178	a	887.84	4.57904	YES	YES
179	a	889.31	0.25737	YES	YES
180	a	902.65	2.28579	YES	YES
181	a	903.71	2.01818	YES	YES
182	a	905.27	1.90138	YES	YES
183	a	905.81	1.66850	YES	YES
184	a	909.19	0.13285	YES	YES
185	a	909.70	4.90124	YES	YES
186	a	911.51	0.70498	YES	YES
187	a	911.88	0.96095	YES	YES
188	a	952.60	0.26736	YES	YES
189	a	983.34	0.44251	YES	YES
190	a	983.63	0.24508	YES	YES
191	a	988.29	2.25570	YES	YES
192	a	988.51	25.09414	YES	YES
193	a	989.80	2.72270	YES	YES
194	a	989.96	14.23521	YES	YES
195	a	990.67	4.62358	YES	YES
196	a	991.44	19.48097	YES	YES
197	a	993.07	9.04642	YES	YES
198	a	994.04	0.15354	YES	YES
199	a	1015.05	0.31919	YES	YES
200	a	1015.68	2.14128	YES	YES
201	a	1016.26	0.91420	YES	YES
202	a	1016.95	0.60617	YES	YES
203	a	1018.72	0.45732	YES	YES
204	a	1019.11	1.50550	YES	YES
205	a	1022.47	1.02462	YES	YES
206	a	1022.85	0.70987	YES	YES
207	a	1030.64	2.71477	YES	YES
208	a	1032.10	1.59791	YES	YES
209	a	1033.35	0.95635	YES	YES
210	a	1033.84	2.85170	YES	YES
211	a	1038.68	2.94665	YES	YES
212	a	1039.41	1.23870	YES	YES
213	a	1039.86	0.74865	YES	YES
214	a	1041.31	1.36549	YES	YES
215	a	1058.31	2.16153	YES	YES
216	a	1058.59	1.84565	YES	YES
217	a	1060.86	0.70514	YES	YES
218	a	1062.78	1.46137	YES	YES
219	a	1065.46	5.23285	YES	YES
220	a	1066.67	0.16563	YES	YES

221	a	1069.10	0.05816	YES	YES
222	a	1070.04	0.10405	YES	YES
223	a	1071.32	0.07198	YES	YES
224	a	1071.67	0.16340	YES	YES
225	a	1072.06	0.17782	YES	YES
226	a	1074.01	0.31442	YES	YES
227	a	1074.40	0.34927	YES	YES
228	a	1074.83	0.20730	YES	YES
229	a	1075.16	0.12486	YES	YES
230	a	1075.70	0.34749	YES	YES
231	a	1083.32	1.58400	YES	YES
232	a	1084.18	1.85760	YES	YES
233	a	1094.27	7.62050	YES	YES
234	a	1095.95	2.32968	YES	YES
235	a	1099.96	10.64796	YES	YES
236	a	1100.69	0.23779	YES	YES
237	a	1103.54	1.61025	YES	YES
238	a	1105.32	0.95518	YES	YES
239	a	1107.03	3.93578	YES	YES
240	a	1108.47	8.62123	YES	YES
241	a	1128.71	4.73079	YES	YES
242	a	1130.72	0.77857	YES	YES
243	a	1160.50	6.67204	YES	YES
244	a	1161.41	4.24201	YES	YES
245	a	1161.60	5.22536	YES	YES
246	a	1163.09	6.12992	YES	YES
247	a	1170.14	8.23309	YES	YES
248	a	1170.36	3.96397	YES	YES
249	a	1171.16	8.82881	YES	YES
250	a	1171.76	4.10878	YES	YES
251	a	1175.76	15.07442	YES	YES
252	a	1179.36	27.05370	YES	YES
253	a	1181.55	8.22854	YES	YES
254	a	1185.99	4.90874	YES	YES
255	a	1187.64	12.69548	YES	YES
256	a	1191.54	3.96734	YES	YES
257	a	1191.99	0.47207	YES	YES
258	a	1194.30	1.72977	YES	YES
259	a	1195.46	3.27001	YES	YES
260	a	1242.45	0.27499	YES	YES
261	a	1243.03	0.12167	YES	YES
262	a	1244.10	0.44108	YES	YES
263	a	1244.76	0.09275	YES	YES
264	a	1246.09	0.60319	YES	YES
265	a	1246.85	0.56113	YES	YES
266	a	1248.00	0.39839	YES	YES
267	a	1248.12	0.51480	YES	YES
268	a	1250.74	0.09082	YES	YES
269	a	1251.07	0.65133	YES	YES
270	a	1253.58	1.24671	YES	YES
271	a	1254.82	8.42573	YES	YES
272	a	1255.60	3.74924	YES	YES
273	a	1256.14	5.15624	YES	YES
274	a	1256.91	4.26439	YES	YES
275	a	1257.41	5.46437	YES	YES
276	a	1257.84	0.72470	YES	YES
277	a	1258.26	4.18004	YES	YES
278	a	1258.98	0.40007	YES	YES

279	a	1259.26	8.20287	YES	YES
280	a	1259.91	1.31725	YES	YES
281	a	1260.59	0.58734	YES	YES
282	a	1261.88	1.97244	YES	YES
283	a	1263.34	1.64937	YES	YES
284	a	1267.51	1.52566	YES	YES
285	a	1269.39	1.30173	YES	YES
286	a	1270.42	1.76099	YES	YES
287	a	1271.07	3.08390	YES	YES
288	a	1280.50	3.24887	YES	YES
289	a	1281.43	1.89518	YES	YES
290	a	1284.04	1.08169	YES	YES
291	a	1284.81	0.70054	YES	YES
292	a	1288.63	4.44846	YES	YES
293	a	1289.77	0.43712	YES	YES
294	a	1290.68	1.79373	YES	YES
295	a	1292.06	3.88919	YES	YES
296	a	1306.79	3.21636	YES	YES
297	a	1307.01	0.53313	YES	YES
298	a	1311.18	2.31396	YES	YES
299	a	1311.42	0.33972	YES	YES
300	a	1314.07	3.69632	YES	YES
301	a	1314.94	0.22134	YES	YES
302	a	1316.35	0.47941	YES	YES
303	a	1320.76	0.47077	YES	YES
304	a	1321.06	2.35658	YES	YES
305	a	1321.46	0.05686	YES	YES
306	a	1323.24	1.16345	YES	YES
307	a	1323.59	4.68813	YES	YES
308	a	1323.66	0.03685	YES	YES
309	a	1323.86	0.28264	YES	YES
310	a	1324.92	0.63849	YES	YES
311	a	1325.36	1.61266	YES	YES
312	a	1325.51	1.57335	YES	YES
313	a	1325.79	0.45212	YES	YES
314	a	1326.72	0.73990	YES	YES
315	a	1326.83	9.16302	YES	YES
316	a	1327.59	1.08014	YES	YES
317	a	1328.71	3.42152	YES	YES
318	a	1332.19	1.79506	YES	YES
319	a	1333.51	0.17413	YES	YES
320	a	1335.78	0.17229	YES	YES
321	a	1336.10	0.11924	YES	YES
322	a	1338.38	0.46076	YES	YES
323	a	1338.75	0.11718	YES	YES
324	a	1339.36	0.17559	YES	YES
325	a	1339.62	1.33956	YES	YES
326	a	1340.82	0.07691	YES	YES
327	a	1340.97	2.29969	YES	YES
328	a	1343.19	0.43943	YES	YES
329	a	1343.31	2.30723	YES	YES
330	a	1347.62	1.62447	YES	YES
331	a	1347.96	0.51927	YES	YES
332	a	1348.82	2.78864	YES	YES
333	a	1349.28	0.62849	YES	YES
334	a	1349.95	0.49057	YES	YES
335	a	1351.25	0.19198	YES	YES
336	a	1381.57	10.21342	YES	YES

337	a	1399.20	8.38878	YES	YES
338	a	1399.83	9.70367	YES	YES
339	a	1404.43	8.38718	YES	YES
340	a	1404.80	5.83344	YES	YES
341	a	1421.22	5.27162	YES	YES
342	a	1426.84	4.24637	YES	YES
343	a	1429.52	2.65217	YES	YES
344	a	1429.84	6.13653	YES	YES
345	a	1431.06	1.17816	YES	YES
346	a	1432.25	1.89623	YES	YES
347	a	1433.64	0.50358	YES	YES
348	a	1433.89	3.34480	YES	YES
349	a	1435.24	0.34342	YES	YES
350	a	1435.57	6.13379	YES	YES
351	a	1436.04	1.00590	YES	YES
352	a	1436.20	5.53508	YES	YES
353	a	1436.80	4.60999	YES	YES
354	a	1437.09	7.16711	YES	YES
355	a	1437.80	4.11861	YES	YES
356	a	1438.01	2.50431	YES	YES
357	a	1438.22	2.78752	YES	YES
358	a	1438.79	3.84708	YES	YES
359	a	1439.37	5.86697	YES	YES
360	a	1439.74	2.48541	YES	YES
361	a	1440.57	3.00927	YES	YES
362	a	1440.73	7.75564	YES	YES
363	a	1440.96	13.02761	YES	YES
364	a	1442.04	11.87110	YES	YES
365	a	1442.51	13.37451	YES	YES
366	a	1443.40	16.60368	YES	YES
367	a	1443.81	47.43123	YES	YES
368	a	1445.05	4.10491	YES	YES
369	a	1446.05	23.47953	YES	YES
370	a	1446.29	3.92505	YES	YES
371	a	1446.91	11.90303	YES	YES
372	a	1447.50	23.80676	YES	YES
373	a	1453.00	0.29845	YES	YES
374	a	1453.13	0.06157	YES	YES
375	a	1453.57	1.99863	YES	YES
376	a	1454.48	3.04404	YES	YES
377	a	1456.00	3.99604	YES	YES
378	a	1456.92	0.24928	YES	YES
379	a	1457.46	0.49162	YES	YES
380	a	1459.38	0.24362	YES	YES
381	a	1867.57	139.71361	YES	YES
382	a	2077.12	547.19144	YES	YES
383	a	2924.32	18.47446	YES	YES
384	a	2929.42	10.14548	YES	YES
385	a	2929.78	18.45179	YES	YES
386	a	2931.34	4.21365	YES	YES
387	a	2932.66	7.97343	YES	YES
388	a	2932.81	5.84123	YES	YES
389	a	2933.96	9.81079	YES	YES
390	a	2934.90	4.86481	YES	YES
391	a	2937.50	18.06705	YES	YES
392	a	2939.98	8.54595	YES	YES
393	a	2940.23	4.40687	YES	YES
394	a	2941.13	11.20883	YES	YES

395	a	2943.26	6.24397	YES	YES
396	a	2943.71	5.14764	YES	YES
397	a	2944.12	9.48998	YES	YES
398	a	2944.31	20.75182	YES	YES
399	a	2944.65	6.87548	YES	YES
400	a	2945.75	14.96055	YES	YES
401	a	2946.11	28.94160	YES	YES
402	a	2946.44	7.18829	YES	YES
403	a	2949.18	6.92309	YES	YES
404	a	2949.28	6.55420	YES	YES
405	a	2949.58	1.20845	YES	YES
406	a	2949.82	10.50284	YES	YES
407	a	2950.13	3.25028	YES	YES
408	a	2952.46	14.84699	YES	YES
409	a	2953.51	13.01016	YES	YES
410	a	2954.16	12.82241	YES	YES
411	a	2954.33	4.17103	YES	YES
412	a	2955.27	2.13549	YES	YES
413	a	2955.90	6.56709	YES	YES
414	a	2956.08	4.13346	YES	YES
415	a	2956.13	3.00441	YES	YES
416	a	2957.45	7.30317	YES	YES
417	a	2957.76	7.06576	YES	YES
418	a	2959.24	4.32358	YES	YES
419	a	2959.49	4.26332	YES	YES
420	a	2960.22	25.53129	YES	YES
421	a	2960.27	4.75853	YES	YES
422	a	2961.42	43.73713	YES	YES
423	a	2961.72	33.75332	YES	YES
424	a	2961.93	46.68269	YES	YES
425	a	2962.10	33.24319	YES	YES
426	a	2962.61	35.07992	YES	YES
427	a	2964.75	29.91353	YES	YES
428	a	2965.04	48.28599	YES	YES
429	a	2965.47	55.28317	YES	YES
430	a	2966.65	11.89291	YES	YES
431	a	2983.25	38.91665	YES	YES
432	a	2984.48	18.05848	YES	YES
433	a	2985.71	22.97730	YES	YES
434	a	2986.58	32.89809	YES	YES
435	a	2988.83	29.48173	YES	YES
436	a	2989.51	5.12958	YES	YES
437	a	2990.60	3.11836	YES	YES
438	a	2995.61	26.62841	YES	YES
439	a	2996.66	14.76410	YES	YES
440	a	3002.49	17.56921	YES	YES
441	a	3004.44	25.81075	YES	YES
442	a	3004.79	20.78252	YES	YES
443	a	3005.56	5.47639	YES	YES
444	a	3005.65	55.93203	YES	YES
445	a	3007.61	5.30477	YES	YES
446	a	3009.39	2.46568	YES	YES
447	a	3009.47	29.72431	YES	YES
448	a	3009.60	41.97878	YES	YES
449	a	3009.65	14.46393	YES	YES
450	a	3009.97	8.96409	YES	YES
451	a	3010.14	16.36517	YES	YES
452	a	3010.43	25.43481	YES	YES

453	a	3011.63	20.32851	YES	YES
454	a	3012.83	13.85840	YES	YES
455	a	3012.93	11.17429	YES	YES
456	a	3013.38	2.76740	YES	YES
457	a	3013.60	35.61051	YES	YES
458	a	3014.12	24.28807	YES	YES
459	a	3014.41	33.84361	YES	YES
460	a	3015.40	21.69225	YES	YES
461	a	3015.66	31.30947	YES	YES
462	a	3016.98	27.09649	YES	YES
463	a	3017.09	19.39263	YES	YES
464	a	3017.32	21.89653	YES	YES
465	a	3017.83	24.93708	YES	YES
466	a	3018.06	37.50253	YES	YES
467	a	3018.32	18.13964	YES	YES
468	a	3018.66	31.65551	YES	YES
469	a	3018.93	19.98061	YES	YES
470	a	3019.01	18.85214	YES	YES
471	a	3019.64	25.10141	YES	YES
472	a	3019.99	31.96307	YES	YES
473	a	3020.27	26.73792	YES	YES
474	a	3026.75	2.96340	YES	YES
475	a	3031.27	0.25869	YES	YES
476	a	3032.71	0.39040	YES	YES
477	a	3048.55	0.42539	YES	YES
478	a	3050.04	0.12071	YES	YES
479	a	3102.08	12.81029	YES	YES
480	a	3186.53	1.44490	YES	YES

$end

Double hybrid single point energy = -7381.665875854929 H
COSMO energy + OC correction = -7389.1715293695 H (in *o*DFB)

6.2.40 $[H\{Ga(dcpe)\}_2(NCCH_2)]^{2+}$/$[H\{Ga(dcpe)\}_2(CH_2CN)]^{2+}$ Transition State

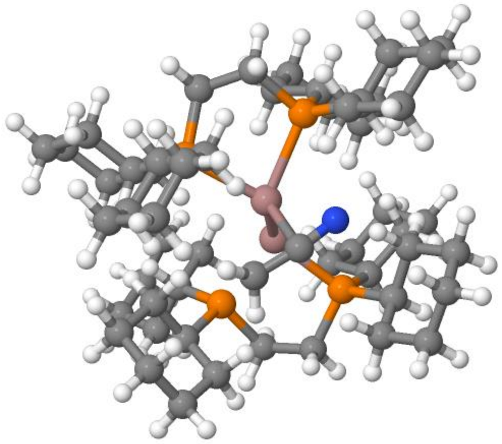

Method: (RI-)BP86(D3BJ)/def2-TZVPP
Symmetry: c1

Cartesian coordinates in Ångström:

H	5.2915549	-3.2460622	-0.3110634
H	4.1049615	-5.0150475	-1.6584701

C	4.3567741	-3.7005312	0.0448585
C	3.5370039	-4.2368656	-1.1311961
H	4.6473914	-4.5341409	0.7050132
H	3.3545590	-3.4270148	-1.8531589
H	-0.0817156	-5.0506955	-6.6053857
H	-0.7993063	-5.3257524	-4.2502537
C	2.1908036	-4.7913963	-0.6588945
C	3.5787244	-2.6689213	0.8755135
H	-2.2156901	-5.3631507	-5.3014215
H	2.3501477	-5.6945768	-0.0469032
H	4.2004257	-2.3526121	1.7235338
C	-1.4146153	-4.7108395	-4.9278053
H	1.5812918	-5.1014181	-1.5196079
C	-0.5481909	-4.2039603	-6.0842989
H	3.3767403	-1.7736634	0.2632800
H	1.2400822	-3.7498889	-4.9387313
C	0.5229678	-3.2266629	-5.5915355
C	2.2432534	-3.2597886	1.3605481
H	-1.1891065	-3.6976578	-6.8249689
H	1.0998419	-2.8280000	-6.4373352
C	1.4126843	-3.7542509	0.1590838
H	2.4573428	-4.1110082	2.0317216
H	-0.7034318	-5.5622400	4.5638908
C	-2.0308293	-3.5574564	-4.1209365
H	0.1639957	-4.8943752	2.3577318
H	1.1848506	-2.8919057	-0.4841801
H	-2.6105658	-3.9684023	-3.2835784
H	-0.2277670	-3.1719816	-2.9758623
H	-1.7561201	-3.8340961	-1.1459169
H	-2.8813685	-4.4506425	0.9926734
H	0.9964186	-3.5387241	4.2444037
C	-1.3735528	-5.0300139	3.8696396
H	-1.9292753	-5.8035929	3.3216886
C	-0.9232054	-2.6126564	-3.6234415
H	-4.0733140	-4.1760861	-0.2717895
C	-0.1038409	-2.0677372	-4.8069844
C	-0.5296136	-4.2213329	2.8763318
H	0.4545997	-4.1733439	0.4853959
C	-3.3226262	-3.6706472	0.3568117
H	3.3525038	0.7434511	-0.8080478
H	-2.7364056	-2.9967107	-4.7559311
C	0.2415515	-3.0892377	3.5737451
C	-2.2431890	-3.0570107	-0.5405185
H	2.8119737	-1.7756767	4.2312676
P	1.2489862	-2.0782651	2.3806798
H	-0.8829642	-3.4892948	6.1399519
H	0.6761110	-1.3833724	-4.4520627
C	2.4347242	-1.1210278	3.4324845
H	-0.7661115	-1.5032922	-5.4834904
C	2.6853396	0.7066550	-1.6660081
H	-1.4550979	-2.6097082	0.0890627
H	3.2869402	-0.8843694	2.7835299
C	-2.3255528	-4.1356329	4.6644853
H	-2.8989916	-4.7331933	5.3852703
H	-1.1904387	-3.7817224	2.1124984
H	-3.5687706	-2.4256094	-2.1263831
C	-1.5462145	-3.0355144	5.3859074
H	2.8413474	1.4600555	-2.4338031

C	-2.8383574	-1.9618879	-1.4416617
C	-4.0028343	-2.5942269	1.2055875
C	-0.7072024	-2.2073761	4.4046265
H	-4.7805224	-3.0399714	1.8402294
C	2.2601670	-0.5375549	-2.0608040
P	-1.5180218	-1.2551389	-2.5196286
H	-0.1389643	-1.4550273	4.9658857
N	1.6739981	-1.5652007	-2.2558733
H	-3.2579107	-2.1421537	1.8826933
H	3.4893018	0.9912663	1.0810160
H	-3.0586761	-3.6774606	3.9800255
H	5.5697417	2.3435295	1.0397109
H	4.0480293	1.3026278	2.7266861
H	-5.4222030	-1.9357822	-0.2854997
C	1.8655986	0.1761636	4.0262762
H	-2.2272011	-2.3668672	5.9305444
C	-4.6085523	-1.5026734	0.3181696
Ga	0.0442114	-0.3417470	1.2216177
C	3.6318951	1.7847557	1.8310022
Ga	0.2308223	-0.0193940	-1.2062241
C	-3.5628110	-0.8827220	-0.6189420
H	-1.3742415	-1.6606258	3.7194742
H	2.6698488	0.7559987	4.4999440
H	1.1283117	-0.0355005	4.8093564
C	-2.3462722	-0.0017006	-3.5896217
H	-1.8012935	0.0007812	-4.5411369
H	1.3650508	1.0247833	-4.0648203
H	-3.3728458	-0.3246083	-3.8133569
C	4.6268600	2.8354156	1.3146704
H	1.9727958	1.5626186	-6.3852564
H	-2.8262623	-0.3241535	-0.0194744
H	3.3706873	2.6567258	-4.6271622
C	0.7272563	1.7239272	-4.6271970
P	1.0172409	1.2454642	2.7803022
H	-4.0573166	-0.1645133	-1.2879582
H	-0.0899715	1.1247288	-5.0517022
H	3.9711708	2.9810510	-0.7460360
H	-5.0626974	-0.7120558	0.9315780
H	4.8659570	3.5257728	2.1395230
C	1.5478397	2.3569496	-5.7568487
C	4.0748564	3.6382379	0.1308980
C	2.2779708	2.4342981	2.1498993
C	2.6506959	3.2624832	-5.2036818
C	-2.3617286	1.4106262	-2.9821571
H	4.7912772	4.4205932	-0.1526684
H	1.9071139	3.2339469	-2.4564133
P	-0.7551779	1.9970695	-2.2785248
H	3.2181564	3.7156026	-6.0270660
H	2.4333761	3.1479422	2.9768583
H	1.5215591	2.4821174	0.1294012
C	0.1609801	2.7923675	-3.6717655
H	-1.1008518	0.6571347	4.8579813
H	-3.0829231	1.4603201	-2.1560694
C	1.7189488	3.2018749	0.9421432
H	0.8802653	2.9474714	-6.4048891
C	1.2397817	3.7615974	-3.1539441
H	-1.8902299	0.9690129	3.3120024
C	2.7156438	4.2620209	0.4610910

```
C    -1.4236911    1.4603428    4.1763474
C    -0.1955785    2.2715854    3.7306647
H    -2.6916348    2.1399616   -3.7346683
C     2.0594814    4.3535122   -4.3073987
H     0.8345709    2.2535789    5.6415109
H    -1.2827431    2.0447930    0.6382547
H     2.8522662    4.9935517   -3.8966864
C     0.4514503    2.9980683    4.9253166
H    -0.5974821    3.3784182   -4.2196534
H     2.8392905    5.0249698    1.2463681
H     2.3079132    4.7833824   -0.4166437
H     0.7604919    3.6738326    1.2034854
C    -1.9378319    2.7785869    0.1418463
C    -1.2480850    3.3411465   -1.1112735
H    -0.5304747    3.0277838    3.0007200
H     0.7698558    4.5730341   -2.5810717
H     1.3092992    3.6045795    4.6042786
H    -2.8520595    2.2336004   -0.1421654
H    -0.2932191    3.7927994   -0.7987772
H     1.4111894    5.0083068   -4.9118908
C    -2.4374534    2.3514659    4.9056822
H    -1.4924576    2.3552247    6.8518326
H    -3.2855123    1.7391530    5.2417229
C    -1.7987968    3.0871354    6.0861895
C    -0.5766578    3.8905928    5.6352728
C    -2.1036661    4.4271224   -1.7904940
C    -2.3124812    3.9031002    1.1144160
H    -2.8458019    3.0890992    4.1953784
H    -3.0370491    3.9791643   -2.1656465
H    -2.8398101    3.4787697    1.9800050
H    -1.5780883    4.8452502   -2.6600850
H    -1.3892208    4.3682606    1.5010357
H    -0.0982317    4.3839355    6.4920448
H    -0.8937175    4.6921212    4.9479749
H    -2.5331046    3.7484692    6.5649109
C    -3.1620902    4.9824407    0.4381951
C    -2.4525468    5.5427799   -0.7969194
H    -4.1319238    4.5510447    0.1405297
H    -1.5277575    6.0586479   -0.4902342
H    -3.0791617    6.2941285   -1.2956272
H    -3.3851055    5.7885768    1.1496643
H    -1.4494762   -0.4624652    1.7043705

SCF energy GEOOPT =  -7388.976138557 H
ZPE =    3708. kJ/mol
FREEH energy =  3893.01 kJ/mol
FREEH entropy =   1.55326 kJ/mol/K

$vibrational spectrum
#  mode     symmetry     wave number   IR intensity    selection rules
#                         cm**(-1)        km/mol          IR     RAMAN
     1        a           -228.98         0.00000        YES     YES
     2                      -0.00         0.00000         -       -
     3                      -0.00         0.00000         -       -
     4                       0.00         0.00000         -       -
     5                       0.00         0.00000         -       -
     6                       0.00         0.00000         -       -
     7                       0.00         0.00000         -       -
```

8	a	16.58	0.05439	YES	YES
9	a	18.07	0.01092	YES	YES
10	a	25.32	0.01540	YES	YES
11	a	28.83	0.01172	YES	YES
12	a	33.46	0.02270	YES	YES
13	a	35.75	0.02350	YES	YES
14	a	36.85	0.04538	YES	YES
15	a	42.03	0.03745	YES	YES
16	a	46.97	0.12346	YES	YES
17	a	48.36	0.02026	YES	YES
18	a	50.16	0.30591	YES	YES
19	a	54.23	0.22881	YES	YES
20	a	57.20	0.17441	YES	YES
21	a	59.69	0.11637	YES	YES
22	a	60.03	0.26627	YES	YES
23	a	63.33	0.22653	YES	YES
24	a	64.37	0.05589	YES	YES
25	a	66.49	0.38451	YES	YES
26	a	70.92	0.05081	YES	YES
27	a	72.14	0.26966	YES	YES
28	a	73.85	0.12105	YES	YES
29	a	77.36	0.55656	YES	YES
30	a	79.83	0.02897	YES	YES
31	a	80.86	0.13635	YES	YES
32	a	84.69	0.14067	YES	YES
33	a	89.33	0.03911	YES	YES
34	a	93.28	0.24551	YES	YES
35	a	99.48	0.40428	YES	YES
36	a	106.12	0.44873	YES	YES
37	a	119.15	1.95722	YES	YES
38	a	126.32	1.13894	YES	YES
39	a	132.04	3.85191	YES	YES
40	a	135.04	2.44264	YES	YES
41	a	136.37	4.01668	YES	YES
42	a	137.86	4.96993	YES	YES
43	a	144.22	2.46148	YES	YES
44	a	148.73	0.71857	YES	YES
45	a	150.64	0.05754	YES	YES
46	a	153.28	1.59094	YES	YES
47	a	162.60	0.32474	YES	YES
48	a	167.74	3.99140	YES	YES
49	a	173.22	1.30775	YES	YES
50	a	177.74	1.03006	YES	YES
51	a	178.19	2.65693	YES	YES
52	a	182.11	0.33127	YES	YES
53	a	186.23	1.08204	YES	YES
54	a	197.26	1.57062	YES	YES
55	a	205.34	2.73413	YES	YES
56	a	212.12	1.05044	YES	YES
57	a	213.01	0.43204	YES	YES
58	a	215.28	0.15189	YES	YES
59	a	216.31	1.43961	YES	YES
60	a	222.60	0.76969	YES	YES
61	a	224.86	0.68799	YES	YES
62	a	227.87	0.08683	YES	YES
63	a	229.69	4.74695	YES	YES
64	a	235.32	1.80574	YES	YES
65	a	238.67	13.30771	YES	YES

66	a	241.05	0.98600	YES	YES
67	a	242.80	2.29745	YES	YES
68	a	250.84	1.90377	YES	YES
69	a	253.75	0.07359	YES	YES
70	a	259.18	0.76829	YES	YES
71	a	267.03	11.25898	YES	YES
72	a	272.20	4.27052	YES	YES
73	a	281.85	2.75414	YES	YES
74	a	291.07	1.80282	YES	YES
75	a	295.78	0.18072	YES	YES
76	a	304.06	0.43689	YES	YES
77	a	305.41	0.40840	YES	YES
78	a	312.88	0.23472	YES	YES
79	a	317.25	0.37029	YES	YES
80	a	330.62	0.43181	YES	YES
81	a	331.69	0.76989	YES	YES
82	a	333.34	0.09396	YES	YES
83	a	335.32	0.39137	YES	YES
84	a	340.70	0.20174	YES	YES
85	a	342.70	0.24853	YES	YES
86	a	362.29	1.78170	YES	YES
87	a	368.37	2.58502	YES	YES
88	a	373.13	0.77181	YES	YES
89	a	376.80	7.71845	YES	YES
90	a	388.17	12.24959	YES	YES
91	a	395.49	0.53455	YES	YES
92	a	397.97	0.77708	YES	YES
93	a	402.71	8.72768	YES	YES
94	a	414.62	1.17221	YES	YES
95	a	419.46	0.53869	YES	YES
96	a	422.27	0.99750	YES	YES
97	a	424.07	4.70756	YES	YES
98	a	424.42	0.84945	YES	YES
99	a	428.18	0.91599	YES	YES
100	a	428.49	2.84953	YES	YES
101	a	429.26	0.77253	YES	YES
102	a	430.68	0.67901	YES	YES
103	a	430.85	1.17378	YES	YES
104	a	432.53	0.25392	YES	YES
105	a	433.79	0.62429	YES	YES
106	a	434.99	0.78329	YES	YES
107	a	436.32	8.32842	YES	YES
108	a	440.93	0.70134	YES	YES
109	a	446.13	1.37144	YES	YES
110	a	462.19	5.32299	YES	YES
111	a	469.85	4.72845	YES	YES
112	a	490.12	2.22231	YES	YES
113	a	491.42	10.19027	YES	YES
114	a	494.07	3.43781	YES	YES
115	a	495.13	1.76617	YES	YES
116	a	498.29	5.94146	YES	YES
117	a	505.33	0.15801	YES	YES
118	a	513.40	15.57864	YES	YES
119	a	519.35	9.15554	YES	YES
120	a	530.77	66.07700	YES	YES
121	a	547.03	2.15148	YES	YES
122	a	607.09	99.83427	YES	YES
123	a	623.30	15.94097	YES	YES

124	a	633.48	19.46049	YES	YES
125	a	641.79	7.33998	YES	YES
126	a	647.36	82.34632	YES	YES
127	a	654.69	9.04490	YES	YES
128	a	713.99	1.31070	YES	YES
129	a	716.41	0.24381	YES	YES
130	a	717.57	1.56693	YES	YES
131	a	720.94	1.75363	YES	YES
132	a	730.47	2.96733	YES	YES
133	a	732.90	7.00383	YES	YES
134	a	735.02	3.87573	YES	YES
135	a	736.27	5.37152	YES	YES
136	a	768.00	0.09934	YES	YES
137	a	770.54	0.56004	YES	YES
138	a	771.76	0.10626	YES	YES
139	a	772.61	0.34640	YES	YES
140	a	773.50	0.73675	YES	YES
141	a	774.53	0.16101	YES	YES
142	a	775.49	1.31788	YES	YES
143	a	776.53	0.11871	YES	YES
144	a	782.84	14.36521	YES	YES
145	a	785.73	9.98678	YES	YES
146	a	808.16	2.11561	YES	YES
147	a	809.31	0.19734	YES	YES
148	a	809.97	1.52397	YES	YES
149	a	810.22	5.10458	YES	YES
150	a	811.28	0.66119	YES	YES
151	a	812.96	0.45547	YES	YES
152	a	814.52	4.67721	YES	YES
153	a	815.44	5.47672	YES	YES
154	a	832.06	1.85099	YES	YES
155	a	835.18	0.66928	YES	YES
156	a	836.58	1.03296	YES	YES
157	a	837.15	8.22161	YES	YES
158	a	838.09	4.25016	YES	YES
159	a	838.84	9.43402	YES	YES
160	a	839.72	1.55026	YES	YES
161	a	840.96	8.78715	YES	YES
162	a	850.78	11.83575	YES	YES
163	a	853.44	5.28595	YES	YES
164	a	872.31	2.66158	YES	YES
165	a	874.17	0.16602	YES	YES
166	a	874.67	3.97741	YES	YES
167	a	875.42	5.79281	YES	YES
168	a	876.50	3.02104	YES	YES
169	a	877.55	1.47554	YES	YES
170	a	878.03	1.94981	YES	YES
171	a	878.90	5.10208	YES	YES
172	a	879.23	4.85748	YES	YES
173	a	879.66	3.50267	YES	YES
174	a	880.56	3.87456	YES	YES
175	a	881.92	0.32739	YES	YES
176	a	882.45	1.43922	YES	YES
177	a	884.00	0.41656	YES	YES
178	a	884.85	4.71661	YES	YES
179	a	886.06	0.93085	YES	YES
180	a	902.66	1.87217	YES	YES
181	a	903.87	2.76485	YES	YES

182	a	904.74	0.90471	YES	YES
183	a	907.37	2.81041	YES	YES
184	a	908.40	2.92474	YES	YES
185	a	908.73	0.70340	YES	YES
186	a	910.32	0.50761	YES	YES
187	a	912.52	0.58433	YES	YES
188	a	985.35	9.49039	YES	YES
189	a	986.41	1.31399	YES	YES
190	a	987.53	5.42192	YES	YES
191	a	987.94	11.00392	YES	YES
192	a	989.50	0.90989	YES	YES
193	a	989.69	18.39010	YES	YES
194	a	991.01	23.23180	YES	YES
195	a	992.83	1.63162	YES	YES
196	a	994.32	6.77948	YES	YES
197	a	995.14	0.70125	YES	YES
198	a	1006.78	0.85315	YES	YES
199	a	1015.73	0.82286	YES	YES
200	a	1016.52	1.62297	YES	YES
201	a	1017.61	0.16392	YES	YES
202	a	1019.07	1.80805	YES	YES
203	a	1019.59	0.59221	YES	YES
204	a	1019.90	0.64717	YES	YES
205	a	1021.30	0.60236	YES	YES
206	a	1025.10	0.91692	YES	YES
207	a	1028.57	0.37349	YES	YES
208	a	1029.37	1.73760	YES	YES
209	a	1032.09	2.46103	YES	YES
210	a	1032.82	1.70620	YES	YES
211	a	1035.18	0.53110	YES	YES
212	a	1038.65	1.34951	YES	YES
213	a	1039.19	0.74326	YES	YES
214	a	1040.11	2.34457	YES	YES
215	a	1057.46	1.11772	YES	YES
216	a	1059.75	2.62705	YES	YES
217	a	1060.91	1.93739	YES	YES
218	a	1063.18	1.50015	YES	YES
219	a	1064.00	0.73251	YES	YES
220	a	1066.84	0.51767	YES	YES
221	a	1068.42	0.19579	YES	YES
222	a	1069.93	0.70176	YES	YES
223	a	1072.07	0.11876	YES	YES
224	a	1072.32	0.35904	YES	YES
225	a	1072.35	0.32645	YES	YES
226	a	1074.22	0.36243	YES	YES
227	a	1074.27	0.40255	YES	YES
228	a	1075.47	0.17530	YES	YES
229	a	1075.63	0.07348	YES	YES
230	a	1077.42	0.09364	YES	YES
231	a	1082.93	4.15086	YES	YES
232	a	1084.22	5.05216	YES	YES
233	a	1091.30	1.90231	YES	YES
234	a	1092.04	7.55948	YES	YES
235	a	1095.66	0.98652	YES	YES
236	a	1097.92	4.79352	YES	YES
237	a	1099.24	3.74300	YES	YES
238	a	1101.00	4.48948	YES	YES
239	a	1103.80	4.30261	YES	YES

240	a	1106.41	1.00773	YES	YES
241	a	1108.19	7.36071	YES	YES
242	a	1131.66	4.80161	YES	YES
243	a	1133.97	1.23953	YES	YES
244	a	1159.12	2.73973	YES	YES
245	a	1160.48	4.11680	YES	YES
246	a	1162.69	3.64619	YES	YES
247	a	1164.06	3.06934	YES	YES
248	a	1169.59	5.03028	YES	YES
249	a	1170.83	3.72159	YES	YES
250	a	1171.23	11.09583	YES	YES
251	a	1171.67	3.66521	YES	YES
252	a	1177.70	16.32450	YES	YES
253	a	1179.33	3.13655	YES	YES
254	a	1182.46	7.48657	YES	YES
255	a	1187.71	19.19518	YES	YES
256	a	1189.19	4.06182	YES	YES
257	a	1190.59	3.48929	YES	YES
258	a	1191.52	12.15455	YES	YES
259	a	1201.92	1.91956	YES	YES
260	a	1242.67	0.48884	YES	YES
261	a	1243.47	0.29721	YES	YES
262	a	1243.94	0.29080	YES	YES
263	a	1245.36	0.22652	YES	YES
264	a	1246.00	0.31164	YES	YES
265	a	1247.66	0.67033	YES	YES
266	a	1248.03	1.72578	YES	YES
267	a	1249.09	1.00348	YES	YES
268	a	1250.12	0.22506	YES	YES
269	a	1250.78	0.26930	YES	YES
270	a	1253.62	8.98142	YES	YES
271	a	1255.03	1.78151	YES	YES
272	a	1256.83	5.99769	YES	YES
273	a	1257.27	7.19603	YES	YES
274	a	1258.10	2.75965	YES	YES
275	a	1258.32	1.15331	YES	YES
276	a	1259.07	9.05650	YES	YES
277	a	1259.86	1.23705	YES	YES
278	a	1260.26	5.56089	YES	YES
279	a	1260.56	6.82994	YES	YES
280	a	1261.28	1.93827	YES	YES
281	a	1261.82	2.22868	YES	YES
282	a	1262.89	4.05567	YES	YES
283	a	1264.72	2.01121	YES	YES
284	a	1266.93	0.75497	YES	YES
285	a	1269.06	0.20598	YES	YES
286	a	1269.81	1.61847	YES	YES
287	a	1270.15	2.15520	YES	YES
288	a	1280.95	2.60946	YES	YES
289	a	1282.18	5.02016	YES	YES
290	a	1285.19	1.05624	YES	YES
291	a	1289.03	0.60532	YES	YES
292	a	1289.88	5.30254	YES	YES
293	a	1290.15	1.87570	YES	YES
294	a	1291.52	3.11196	YES	YES
295	a	1294.38	3.95774	YES	YES
296	a	1306.06	1.22692	YES	YES
297	a	1309.31	0.95473	YES	YES

298	a	1310.28	0.36749	YES	YES
299	a	1310.98	2.10207	YES	YES
300	a	1313.43	0.46809	YES	YES
301	a	1314.74	2.21768	YES	YES
302	a	1316.48	1.69003	YES	YES
303	a	1320.10	2.50681	YES	YES
304	a	1322.20	0.29237	YES	YES
305	a	1323.00	3.38206	YES	YES
306	a	1323.36	2.10145	YES	YES
307	a	1323.57	1.21803	YES	YES
308	a	1323.89	0.24615	YES	YES
309	a	1324.53	3.10598	YES	YES
310	a	1325.23	1.09643	YES	YES
311	a	1325.43	3.74716	YES	YES
312	a	1325.94	0.38234	YES	YES
313	a	1326.38	0.58019	YES	YES
314	a	1326.50	0.69125	YES	YES
315	a	1326.93	1.99245	YES	YES
316	a	1328.18	3.06753	YES	YES
317	a	1328.94	2.20503	YES	YES
318	a	1329.84	2.03397	YES	YES
319	a	1330.12	1.38200	YES	YES
320	a	1336.09	0.05756	YES	YES
321	a	1336.63	1.04156	YES	YES
322	a	1338.05	0.38751	YES	YES
323	a	1338.42	0.19332	YES	YES
324	a	1338.72	0.33758	YES	YES
325	a	1339.12	0.58592	YES	YES
326	a	1340.58	0.04769	YES	YES
327	a	1344.81	1.13048	YES	YES
328	a	1345.06	1.38750	YES	YES
329	a	1347.03	1.45854	YES	YES
330	a	1347.33	2.79588	YES	YES
331	a	1348.76	0.27337	YES	YES
332	a	1348.92	0.60509	YES	YES
333	a	1350.01	1.13078	YES	YES
334	a	1350.61	0.88754	YES	YES
335	a	1352.97	0.21285	YES	YES
336	a	1373.97	11.64455	YES	YES
337	a	1402.56	6.27073	YES	YES
338	a	1405.16	8.28182	YES	YES
339	a	1406.01	10.53186	YES	YES
340	a	1409.15	6.41648	YES	YES
341	a	1421.91	2.08641	YES	YES
342	a	1424.21	7.44658	YES	YES
343	a	1425.38	1.88942	YES	YES
344	a	1429.70	4.86024	YES	YES
345	a	1431.10	3.97963	YES	YES
346	a	1432.04	2.73451	YES	YES
347	a	1433.14	2.98676	YES	YES
348	a	1434.60	4.62765	YES	YES
349	a	1435.52	1.35850	YES	YES
350	a	1435.63	2.36077	YES	YES
351	a	1436.89	5.36742	YES	YES
352	a	1437.46	10.78074	YES	YES
353	a	1437.73	1.13539	YES	YES
354	a	1437.76	17.23381	YES	YES
355	a	1438.00	4.61736	YES	YES

356	a	1438.18	3.90856	YES	YES
357	a	1438.34	0.55100	YES	YES
358	a	1438.65	5.45014	YES	YES
359	a	1439.66	0.23992	YES	YES
360	a	1440.05	3.70395	YES	YES
361	a	1441.14	8.80207	YES	YES
362	a	1441.72	8.67844	YES	YES
363	a	1441.82	4.27126	YES	YES
364	a	1442.89	7.44974	YES	YES
365	a	1443.23	21.55321	YES	YES
366	a	1443.84	4.89833	YES	YES
367	a	1444.29	7.82911	YES	YES
368	a	1445.35	30.85699	YES	YES
369	a	1446.33	8.51947	YES	YES
370	a	1446.57	4.88195	YES	YES
371	a	1447.80	8.36316	YES	YES
372	a	1448.87	9.38718	YES	YES
373	a	1453.27	0.43702	YES	YES
374	a	1453.75	2.69591	YES	YES
375	a	1454.78	6.20617	YES	YES
376	a	1454.92	0.46243	YES	YES
377	a	1455.68	0.86834	YES	YES
378	a	1457.06	1.07241	YES	YES
379	a	1458.13	0.92432	YES	YES
380	a	1461.11	0.36640	YES	YES
381	a	1880.40	110.69460	YES	YES
382	a	2029.99	190.33166	YES	YES
383	a	2925.97	4.04183	YES	YES
384	a	2928.62	3.37066	YES	YES
385	a	2930.58	3.22346	YES	YES
386	a	2932.23	22.07006	YES	YES
387	a	2935.35	7.59021	YES	YES
388	a	2936.42	4.16421	YES	YES
389	a	2938.59	16.25524	YES	YES
390	a	2939.24	6.20425	YES	YES
391	a	2942.58	36.70198	YES	YES
392	a	2943.47	8.03407	YES	YES
393	a	2944.46	14.50900	YES	YES
394	a	2944.84	6.99054	YES	YES
395	a	2945.01	4.85441	YES	YES
396	a	2945.58	14.63007	YES	YES
397	a	2946.42	6.15302	YES	YES
398	a	2948.06	5.53857	YES	YES
399	a	2948.10	10.19987	YES	YES
400	a	2948.47	15.04220	YES	YES
401	a	2948.55	13.35249	YES	YES
402	a	2948.60	21.04498	YES	YES
403	a	2948.90	2.90618	YES	YES
404	a	2953.99	10.41242	YES	YES
405	a	2954.52	11.82686	YES	YES
406	a	2955.05	34.61624	YES	YES
407	a	2955.92	15.51649	YES	YES
408	a	2956.11	7.36957	YES	YES
409	a	2956.23	6.92118	YES	YES
410	a	2956.95	9.28403	YES	YES
411	a	2957.80	8.46302	YES	YES
412	a	2958.33	3.21930	YES	YES
413	a	2958.99	10.09542	YES	YES

414	a	2959.62	20.81588	YES	YES
415	a	2960.51	2.00653	YES	YES
416	a	2960.54	25.98376	YES	YES
417	a	2961.62	29.77778	YES	YES
418	a	2962.40	41.03785	YES	YES
419	a	2962.75	25.83954	YES	YES
420	a	2963.20	31.92540	YES	YES
421	a	2964.16	21.44231	YES	YES
422	a	2965.97	15.96271	YES	YES
423	a	2967.29	26.58282	YES	YES
424	a	2968.39	30.22383	YES	YES
425	a	2969.03	7.49894	YES	YES
426	a	2970.30	18.29627	YES	YES
427	a	2973.22	4.90060	YES	YES
428	a	2974.48	5.27788	YES	YES
429	a	2974.63	26.94765	YES	YES
430	a	2982.04	11.51414	YES	YES
431	a	2984.05	6.80874	YES	YES
432	a	2984.41	2.42252	YES	YES
433	a	2986.34	21.08734	YES	YES
434	a	2987.81	14.77380	YES	YES
435	a	2992.87	4.26869	YES	YES
436	a	2994.33	16.87775	YES	YES
437	a	3002.94	33.06273	YES	YES
438	a	3003.12	15.68172	YES	YES
439	a	3004.57	26.65347	YES	YES
440	a	3005.38	19.31742	YES	YES
441	a	3005.70	18.88408	YES	YES
442	a	3007.08	15.20663	YES	YES
443	a	3007.78	17.47990	YES	YES
444	a	3008.74	13.19730	YES	YES
445	a	3009.09	25.69844	YES	YES
446	a	3009.55	26.75927	YES	YES
447	a	3009.88	27.63994	YES	YES
448	a	3010.01	29.01149	YES	YES
449	a	3010.05	24.25302	YES	YES
450	a	3010.57	6.78388	YES	YES
451	a	3010.75	20.51854	YES	YES
452	a	3010.88	24.26646	YES	YES
453	a	3011.39	21.12291	YES	YES
454	a	3011.56	4.54373	YES	YES
455	a	3011.80	31.48693	YES	YES
456	a	3012.64	21.08601	YES	YES
457	a	3012.91	37.95109	YES	YES
458	a	3013.79	39.33653	YES	YES
459	a	3015.06	15.39724	YES	YES
460	a	3015.54	3.22864	YES	YES
461	a	3016.00	28.31340	YES	YES
462	a	3017.05	22.87534	YES	YES
463	a	3017.27	36.33760	YES	YES
464	a	3017.64	15.82221	YES	YES
465	a	3018.87	33.57349	YES	YES
466	a	3019.33	28.07003	YES	YES
467	a	3019.46	15.99799	YES	YES
468	a	3019.93	36.65678	YES	YES
469	a	3021.86	15.82313	YES	YES
470	a	3022.06	51.69754	YES	YES
471	a	3022.72	28.81721	YES	YES

472	a	3028.04	14.76083	YES	YES
473	a	3030.24	0.34917	YES	YES
474	a	3031.06	11.29452	YES	YES
475	a	3036.49	1.00838	YES	YES
476	a	3046.04	8.08065	YES	YES
477	a	3046.71	0.46992	YES	YES
478	a	3051.78	0.67159	YES	YES
479	a	3084.28	3.59878	YES	YES
480	a	3170.42	1.74430	YES	YES

$end

Double hybrid single point energy = -7381.619781965132 H
COSMO energy + OC correction = -7389.1253556530 H (in *o*DFB)

6.2.41 $[H\{Ga(dcpe)\}_2(CH_2CN)]^{2+}$

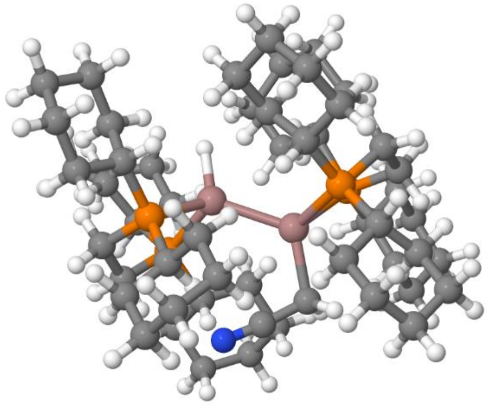

Method: (RI-)BP86(D3BJ)/def2-TZVPP
Symmetry: c1

Cartesian coordinates in Ångström:

Ga	0.0199873	-0.0612427	-1.3450973
Ga	-0.3302978	0.0476855	1.0856514
H	0.7041359	0.8507132	1.9624954
N	-3.2099155	-1.8367576	-0.9866473
C	-2.4876837	-1.3175793	-1.7428988
C	-1.5381803	-0.6311238	-2.5666329
H	-2.0005441	0.2703448	-2.9884595
H	-1.2053950	-1.2669977	-3.3942253
P	-2.5363604	0.7732642	1.7439526
P	-0.8042075	-2.0292114	2.2259281
C	-2.6293400	2.0499099	3.0727474
H	-3.7004016	2.0944059	3.3323552
C	-1.8420607	1.6735366	4.3377236
H	-2.0740534	0.6542663	4.6672376
H	-0.7678819	1.6898820	4.0999807
C	-2.1707357	2.6555283	5.4861142
H	-3.0238073	2.2731622	6.0631755
H	-1.3163576	2.6831926	6.1764220
C	-2.4817109	4.0768069	4.9737411
H	-2.2445043	4.8175374	5.7472934
H	-3.5588259	4.1829600	4.7711387
C	-1.6988511	4.3573115	3.6909369

H	-1.8047434	5.4046679	3.3795595
H	-0.6269909	4.1964556	3.8825264
C	-2.1705296	3.4334859	2.5486829
H	-1.3535310	3.3067715	1.8244188
H	-3.0014917	3.9050505	2.0066711
C	-3.6383013	1.2794454	0.3564356
H	-3.8037422	0.3226696	-0.1653785
C	-5.0064430	1.8200321	0.8049986
H	-5.4950154	1.1166345	1.4943281
H	-4.8718776	2.7665619	1.3517149
C	-5.9019577	2.0625224	-0.4187806
H	-6.1244234	1.0952202	-0.8972471
H	-6.8639013	2.4806372	-0.0933377
C	-5.2311203	2.9937966	-1.4338004
H	-5.1103219	3.9953380	-0.9888598
H	-5.8745020	3.1196493	-2.3148078
C	-3.8561116	2.4622912	-1.8550444
H	-3.3722133	3.1605909	-2.5531042
H	-3.9851280	1.5089630	-2.3949612
C	-2.9556992	2.2342251	-0.6370513
H	-1.9807998	1.8202088	-0.9433999
H	-2.7458527	3.1988240	-0.1515765
C	-3.3086236	-0.7682419	2.4133787
H	-4.1696416	-0.5031779	3.0425194
H	-3.6917425	-1.3120442	1.5367728
C	-2.3220673	-1.6465023	3.1975807
H	-2.8039331	-2.5846137	3.5003574
H	-1.9880060	-1.1493999	4.1155972
C	0.4989309	-2.5364340	3.4301837
H	1.3639859	-2.7856790	2.7911117
C	0.9046227	-1.3511010	4.3269800
H	0.0401371	-1.0403480	4.9362429
H	1.1844364	-0.4854346	3.7110948
C	2.0533679	-1.7435862	5.2643643
H	2.9516054	-1.9582804	4.6620652
H	2.3052485	-0.8916898	5.9109042
C	1.6978520	-2.9737406	6.1035072
H	0.8654277	-2.7248250	6.7823009
H	2.5451634	-3.2572678	6.7419552
C	1.2915354	-4.1483785	5.2104749
H	2.1576307	-4.4675050	4.6068911
H	0.9987593	-5.0152130	5.8182092
C	0.1382103	-3.7756408	4.2682678
H	-0.7664470	-3.5653739	4.8612839
H	-0.0982909	-4.6266972	3.6172276
C	-1.2368484	-3.4251345	1.0967439
H	-1.7443312	-2.9202643	0.2590448
C	0.0409679	-4.0814126	0.5507438
H	0.6822701	-3.3095480	0.0942880
H	0.6202374	-4.5273477	1.3744873
C	-0.2982516	-5.1723052	-0.4721855
H	0.6264546	-5.6553275	-0.8198160
H	-0.7687504	-4.7061067	-1.3524469
C	-1.2606783	-6.2078664	0.1139757
H	-1.5019215	-6.9682898	-0.6407749
H	-0.7697467	-6.7382194	0.9466954
C	-2.5389176	-5.5319802	0.6146974
H	-3.2257795	-6.2723422	1.0461859

H	-3.0660713	-5.0660167	-0.2332170
C	-2.2320207	-4.4537871	1.6625658
H	-3.1678999	-3.9551665	1.9485799
H	-1.8288921	-4.9282446	2.5689796
P	2.0965431	-1.1068465	-2.1204522
P	1.0477063	2.0036030	-2.1109876
C	1.9242499	-2.2389910	-3.5655832
H	2.9376337	-2.6225239	-3.7740419
C	1.4260742	-1.4996670	-4.8211106
H	2.1618028	-0.7432681	-5.1287540
H	0.4920257	-0.9604528	-4.5930533
C	1.1789325	-2.4752372	-5.9794701
H	0.7800680	-1.9226307	-6.8408861
H	2.1422628	-2.9028153	-6.3008157
C	0.2324382	-3.6077777	-5.5733155
H	0.0837305	-4.2961622	-6.4153996
H	-0.7613273	-3.1922394	-5.3328130
C	0.7781040	-4.3642035	-4.3599234
H	1.7260455	-4.8562742	-4.6305993
H	0.0879101	-5.1619206	-4.0532477
C	1.0191699	-3.4202856	-3.1771754
H	0.0510287	-3.0355036	-2.8163599
H	1.4626813	-3.9745514	-2.3392081
C	3.0822503	-2.0028803	-0.8419030
H	2.5114906	-2.9288066	-0.6593580
C	4.4918132	-2.3904913	-1.3243727
H	5.0613866	-1.4788084	-1.5654039
H	4.4384529	-2.9881037	-2.2447182
C	5.2377922	-3.1733465	-0.2344965
H	4.7248237	-4.1343546	-0.0656698
H	6.2486787	-3.4167177	-0.5877334
C	5.3000675	-2.3875514	1.0782100
H	5.9037099	-1.4765754	0.9324571
H	5.8103957	-2.9779115	1.8507478
C	3.8976835	-1.9959101	1.5516303
H	3.3299685	-2.9099430	1.7958992
H	3.9476440	-1.3995410	2.4731802
C	3.1357787	-1.2140388	0.4758629
H	3.6364070	-0.2449226	0.3113448
H	2.1179064	-0.9808981	0.8225066
C	3.1753953	0.2939804	-2.6690427
H	3.7484422	0.6140562	-1.7875005
H	3.9023482	-0.0860773	-3.3997734
C	2.3903013	1.4753023	-3.2519917
H	1.9171335	1.2008091	-4.2027708
H	3.0625163	2.3201449	-3.4531201
C	0.0640373	3.2958749	-2.9848493
H	-0.7341987	3.5590333	-2.2695053
C	-0.5968280	2.7003486	-4.2439514
H	-1.1924304	1.8147675	-3.9832169
H	0.1844444	2.3631088	-4.9438244
C	-1.4757137	3.7408950	-4.9499417
H	-1.9083228	3.2977784	-5.8572302
H	-2.3223378	4.0022637	-4.2941204
C	-0.6843588	5.0069821	-5.2871487
H	0.0950630	4.7657885	-6.0286145
H	-1.3416368	5.7498258	-5.7579269
C	-0.0311420	5.5955082	-4.0344316

```
H    -0.8134104    5.9347202   -3.3353566
H     0.5665379    6.4813785   -4.2878893
C     0.8618863    4.5681351   -3.3249042
H     1.2877193    5.0103685   -2.4154370
H     1.7075464    4.3059498   -3.9807511
C     1.8883350    2.6910826   -0.6158975
H     2.1213746    1.7874407   -0.0216540
C     0.8842848    3.5137132    0.2074826
H     0.5840185    4.4075992   -0.3607922
H    -0.0302525    2.9234431    0.3748588
C     1.4995306    3.9377151    1.5442688
H     0.7863843    4.5654690    2.0953319
H     1.6691959    3.0362067    2.1560075
C     2.8210173    4.6851375    1.3403772
H     3.2648550    4.9399000    2.3119942
H     2.6250570    5.6412517    0.8278494
C     3.8074855    3.8593452    0.5091335
H     4.7365653    4.4206451    0.3422058
H     4.0850183    2.9487898    1.0665562
C     3.2025208    3.4541972   -0.8424886
H     3.0196609    4.3540714   -1.4469676
H     3.9223342    2.8404966   -1.4026912

SCF energy GEOOPT =  -7389.023362915 H
ZPE =   3710. kJ/mol
FREEH energy =  3895.05 kJ/mol
FREEH entropy =   1.58077 kJ/mol/K

$vibrational spectrum
#  mode     symmetry     wave number   IR intensity    selection rules
#                         cm**(-1)        km/mol          IR     RAMAN
     1        a            -10.47          0.00000        YES     YES
     2                      -0.00          0.00000         -       -
     3                      -0.00          0.00000         -       -
     4                      -0.00          0.00000         -       -
     5                      -0.00          0.00000         -       -
     6                       0.00          0.00000         -       -
     7                       0.00          0.00000         -       -
     8        a             11.13          0.08455        YES     YES
     9        a             13.96          0.12439        YES     YES
    10        a             22.23          0.03388        YES     YES
    11        a             23.71          0.28455        YES     YES
    12        a             28.69          0.02911        YES     YES
    13        a             30.29          0.07123        YES     YES
    14        a             34.68          0.01816        YES     YES
    15        a             36.45          0.17222        YES     YES
    16        a             39.27          0.07820        YES     YES
    17        a             40.95          0.21247        YES     YES
    18        a             43.60          0.81858        YES     YES
    19        a             50.39          0.04783        YES     YES
    20        a             53.44          0.12709        YES     YES
    21        a             53.85          0.04676        YES     YES
    22        a             54.71          0.07015        YES     YES
    23        a             58.25          0.13116        YES     YES
    24        a             59.72          0.13099        YES     YES
    25        a             62.02          0.18066        YES     YES
    26        a             65.98          0.05522        YES     YES
    27        a             67.17          0.55673        YES     YES
```

28	a	69.92	0.61878	YES	YES
29	a	74.81	0.22388	YES	YES
30	a	78.55	0.11105	YES	YES
31	a	79.58	0.12076	YES	YES
32	a	84.51	0.35526	YES	YES
33	a	89.06	0.49323	YES	YES
34	a	92.39	1.64375	YES	YES
35	a	96.01	3.34835	YES	YES
36	a	103.42	1.32246	YES	YES
37	a	105.58	5.88786	YES	YES
38	a	116.93	0.71250	YES	YES
39	a	118.06	1.00136	YES	YES
40	a	129.94	1.27381	YES	YES
41	a	134.36	1.36442	YES	YES
42	a	136.44	0.71643	YES	YES
43	a	138.02	2.02268	YES	YES
44	a	140.97	0.40926	YES	YES
45	a	148.56	1.68006	YES	YES
46	a	151.62	0.73842	YES	YES
47	a	155.24	0.77378	YES	YES
48	a	160.53	0.67750	YES	YES
49	a	160.88	2.43685	YES	YES
50	a	161.64	0.63294	YES	YES
51	a	179.31	2.64260	YES	YES
52	a	185.11	1.64012	YES	YES
53	a	187.17	5.07442	YES	YES
54	a	191.93	5.08994	YES	YES
55	a	194.76	0.02181	YES	YES
56	a	200.09	1.71458	YES	YES
57	a	202.87	1.85663	YES	YES
58	a	208.82	0.38559	YES	YES
59	a	213.89	0.23541	YES	YES
60	a	219.43	0.68460	YES	YES
61	a	223.59	0.31407	YES	YES
62	a	226.07	0.86823	YES	YES
63	a	228.95	0.00906	YES	YES
64	a	232.00	0.24515	YES	YES
65	a	233.45	1.43775	YES	YES
66	a	236.65	0.35845	YES	YES
67	a	237.78	0.01276	YES	YES
68	a	239.29	0.37449	YES	YES
69	a	241.55	0.63427	YES	YES
70	a	256.47	0.16693	YES	YES
71	a	261.07	0.25803	YES	YES
72	a	275.38	1.02539	YES	YES
73	a	292.93	0.29772	YES	YES
74	a	297.33	0.36096	YES	YES
75	a	306.09	0.61988	YES	YES
76	a	307.72	0.05854	YES	YES
77	a	312.61	0.14829	YES	YES
78	a	313.26	0.25703	YES	YES
79	a	327.59	0.45036	YES	YES
80	a	330.13	0.54591	YES	YES
81	a	332.33	0.31850	YES	YES
82	a	335.83	0.25589	YES	YES
83	a	337.18	0.01415	YES	YES
84	a	341.94	0.54365	YES	YES
85	a	344.96	1.31853	YES	YES

86	a	374.06	0.31937	YES	YES
87	a	376.66	0.82423	YES	YES
88	a	379.84	1.11721	YES	YES
89	a	385.03	0.84863	YES	YES
90	a	387.47	0.61311	YES	YES
91	a	390.69	0.62303	YES	YES
92	a	402.94	1.93259	YES	YES
93	a	408.42	3.14689	YES	YES
94	a	411.71	2.19376	YES	YES
95	a	416.92	1.72292	YES	YES
96	a	421.08	3.25693	YES	YES
97	a	424.02	0.72109	YES	YES
98	a	426.02	0.59719	YES	YES
99	a	428.90	0.43274	YES	YES
100	a	430.40	0.15733	YES	YES
101	a	431.07	0.07016	YES	YES
102	a	431.72	0.07078	YES	YES
103	a	431.92	0.07955	YES	YES
104	a	434.17	3.12873	YES	YES
105	a	436.62	2.67659	YES	YES
106	a	449.82	1.44674	YES	YES
107	a	453.44	3.74704	YES	YES
108	a	464.07	0.42414	YES	YES
109	a	465.04	8.38768	YES	YES
110	a	469.88	1.66073	YES	YES
111	a	491.32	2.46765	YES	YES
112	a	495.98	0.51558	YES	YES
113	a	498.53	2.72194	YES	YES
114	a	502.28	1.42980	YES	YES
115	a	503.64	4.37536	YES	YES
116	a	514.12	17.28476	YES	YES
117	a	514.79	2.38378	YES	YES
118	a	525.10	14.46596	YES	YES
119	a	536.47	83.71877	YES	YES
120	a	563.90	82.22459	YES	YES
121	a	583.74	25.88896	YES	YES
122	a	628.62	3.89062	YES	YES
123	a	631.95	33.25149	YES	YES
124	a	633.97	8.06655	YES	YES
125	a	644.99	8.07147	YES	YES
126	a	654.77	9.42883	YES	YES
127	a	697.12	6.53588	YES	YES
128	a	708.95	3.25577	YES	YES
129	a	716.62	4.12345	YES	YES
130	a	726.30	5.51461	YES	YES
131	a	727.91	7.49798	YES	YES
132	a	733.37	10.18336	YES	YES
133	a	737.06	1.14423	YES	YES
134	a	740.44	0.96315	YES	YES
135	a	758.18	1.30682	YES	YES
136	a	768.38	0.39226	YES	YES
137	a	769.84	0.31009	YES	YES
138	a	770.34	0.11026	YES	YES
139	a	772.83	0.35587	YES	YES
140	a	774.00	0.13488	YES	YES
141	a	774.41	0.03474	YES	YES
142	a	775.98	0.21666	YES	YES
143	a	788.23	22.95114	YES	YES

144	a	792.29	15.57284	YES	YES
145	a	806.86	0.82134	YES	YES
146	a	809.12	1.23768	YES	YES
147	a	809.97	1.08165	YES	YES
148	a	810.32	2.90723	YES	YES
149	a	812.26	3.76578	YES	YES
150	a	815.21	0.69785	YES	YES
151	a	815.67	4.12627	YES	YES
152	a	819.36	5.85401	YES	YES
153	a	833.65	2.88794	YES	YES
154	a	834.37	2.57588	YES	YES
155	a	835.28	6.87645	YES	YES
156	a	836.27	0.82939	YES	YES
157	a	837.06	1.25534	YES	YES
158	a	837.70	4.74145	YES	YES
159	a	839.86	6.49324	YES	YES
160	a	843.20	4.25273	YES	YES
161	a	852.22	14.86251	YES	YES
162	a	859.30	10.90356	YES	YES
163	a	871.86	2.37132	YES	YES
164	a	871.95	3.28022	YES	YES
165	a	872.99	4.36333	YES	YES
166	a	874.69	2.10272	YES	YES
167	a	875.76	4.81552	YES	YES
168	a	876.35	3.30608	YES	YES
169	a	877.73	4.00245	YES	YES
170	a	878.48	1.41167	YES	YES
171	a	879.33	1.90990	YES	YES
172	a	880.44	0.55574	YES	YES
173	a	881.49	3.85956	YES	YES
174	a	881.94	2.62274	YES	YES
175	a	882.56	1.10092	YES	YES
176	a	885.20	2.26973	YES	YES
177	a	885.86	0.68694	YES	YES
178	a	896.29	1.26542	YES	YES
179	a	902.70	3.57146	YES	YES
180	a	903.39	1.17032	YES	YES
181	a	904.28	1.48465	YES	YES
182	a	905.50	3.35843	YES	YES
183	a	905.89	1.26973	YES	YES
184	a	906.68	0.58285	YES	YES
185	a	908.64	1.11539	YES	YES
186	a	910.27	0.58552	YES	YES
187	a	969.30	3.26640	YES	YES
188	a	970.62	3.28542	YES	YES
189	a	985.34	12.16884	YES	YES
190	a	985.98	9.44630	YES	YES
191	a	986.49	1.11663	YES	YES
192	a	986.99	14.57976	YES	YES
193	a	988.17	7.33692	YES	YES
194	a	991.00	3.97136	YES	YES
195	a	992.34	15.15368	YES	YES
196	a	992.64	7.22690	YES	YES
197	a	993.36	0.82341	YES	YES
198	a	1013.99	1.02946	YES	YES
199	a	1014.85	1.05884	YES	YES
200	a	1016.27	0.86289	YES	YES
201	a	1016.48	0.52123	YES	YES

202	a	1017.98	0.39990	YES	YES
203	a	1018.97	0.63737	YES	YES
204	a	1020.49	1.83688	YES	YES
205	a	1022.24	0.46492	YES	YES
206	a	1030.97	3.62095	YES	YES
207	a	1032.03	1.84090	YES	YES
208	a	1033.14	3.06160	YES	YES
209	a	1037.01	24.96528	YES	YES
210	a	1038.52	4.46223	YES	YES
211	a	1039.78	0.57076	YES	YES
212	a	1040.81	0.55683	YES	YES
213	a	1042.58	0.66729	YES	YES
214	a	1045.67	2.52023	YES	YES
215	a	1057.02	0.86343	YES	YES
216	a	1058.12	1.73056	YES	YES
217	a	1059.87	2.31304	YES	YES
218	a	1060.69	0.64538	YES	YES
219	a	1060.93	1.87892	YES	YES
220	a	1064.38	0.32090	YES	YES
221	a	1066.36	0.64442	YES	YES
222	a	1070.06	0.10162	YES	YES
223	a	1070.87	0.85287	YES	YES
224	a	1071.47	0.04394	YES	YES
225	a	1071.85	0.01722	YES	YES
226	a	1071.97	0.05557	YES	YES
227	a	1072.37	0.35120	YES	YES
228	a	1075.18	0.38725	YES	YES
229	a	1075.61	0.34063	YES	YES
230	a	1078.26	2.06412	YES	YES
231	a	1085.04	1.32071	YES	YES
232	a	1088.95	4.57355	YES	YES
233	a	1090.23	1.80627	YES	YES
234	a	1093.97	7.04545	YES	YES
235	a	1095.62	6.22755	YES	YES
236	a	1100.83	0.18860	YES	YES
237	a	1101.86	17.25973	YES	YES
238	a	1105.20	1.85525	YES	YES
239	a	1107.62	3.75270	YES	YES
240	a	1110.06	13.21061	YES	YES
241	a	1112.38	0.89606	YES	YES
242	a	1126.17	5.95468	YES	YES
243	a	1128.14	2.41295	YES	YES
244	a	1160.52	8.94683	YES	YES
245	a	1161.00	4.54210	YES	YES
246	a	1162.92	5.70778	YES	YES
247	a	1164.37	7.90189	YES	YES
248	a	1166.00	7.47231	YES	YES
249	a	1168.31	2.21784	YES	YES
250	a	1169.38	2.70243	YES	YES
251	a	1170.13	6.94136	YES	YES
252	a	1177.48	13.58214	YES	YES
253	a	1184.77	6.77345	YES	YES
254	a	1185.88	3.76903	YES	YES
255	a	1188.10	0.43570	YES	YES
256	a	1188.94	3.85168	YES	YES
257	a	1201.10	3.22123	YES	YES
258	a	1203.21	4.55256	YES	YES
259	a	1208.83	3.65685	YES	YES

260	a	1221.04	1.71488	YES	YES
261	a	1239.45	0.33839	YES	YES
262	a	1242.58	0.11606	YES	YES
263	a	1243.47	0.13303	YES	YES
264	a	1244.27	0.32266	YES	YES
265	a	1245.80	0.46184	YES	YES
266	a	1247.26	0.33906	YES	YES
267	a	1248.43	0.58517	YES	YES
268	a	1249.65	0.70462	YES	YES
269	a	1250.45	0.47469	YES	YES
270	a	1251.81	0.91012	YES	YES
271	a	1252.94	2.09095	YES	YES
272	a	1253.99	0.08947	YES	YES
273	a	1255.36	7.19118	YES	YES
274	a	1256.48	9.96820	YES	YES
275	a	1256.58	1.01466	YES	YES
276	a	1257.34	3.27718	YES	YES
277	a	1257.76	8.49618	YES	YES
278	a	1259.05	1.33365	YES	YES
279	a	1259.79	2.34126	YES	YES
280	a	1260.34	2.02385	YES	YES
281	a	1261.61	0.61562	YES	YES
282	a	1263.65	5.49989	YES	YES
283	a	1264.87	2.52622	YES	YES
284	a	1265.11	0.32143	YES	YES
285	a	1268.78	2.18208	YES	YES
286	a	1270.22	4.32669	YES	YES
287	a	1276.81	2.77195	YES	YES
288	a	1279.89	2.76956	YES	YES
289	a	1281.12	2.58911	YES	YES
290	a	1281.80	3.43381	YES	YES
291	a	1283.61	0.85033	YES	YES
292	a	1286.06	1.82584	YES	YES
293	a	1287.30	7.43350	YES	YES
294	a	1289.33	2.59232	YES	YES
295	a	1292.73	2.23733	YES	YES
296	a	1305.99	1.62170	YES	YES
297	a	1308.52	2.62105	YES	YES
298	a	1309.24	0.78395	YES	YES
299	a	1309.96	2.79902	YES	YES
300	a	1310.57	1.49915	YES	YES
301	a	1311.58	0.47699	YES	YES
302	a	1312.50	0.56539	YES	YES
303	a	1317.56	1.76329	YES	YES
304	a	1319.87	0.67922	YES	YES
305	a	1321.42	0.52272	YES	YES
306	a	1321.88	0.98335	YES	YES
307	a	1322.76	1.00115	YES	YES
308	a	1322.86	0.60426	YES	YES
309	a	1323.11	1.21627	YES	YES
310	a	1323.69	1.39806	YES	YES
311	a	1323.86	0.91512	YES	YES
312	a	1324.34	1.66257	YES	YES
313	a	1325.00	2.31921	YES	YES
314	a	1325.46	1.71278	YES	YES
315	a	1325.47	0.74165	YES	YES
316	a	1325.83	4.26516	YES	YES
317	a	1327.25	1.68266	YES	YES

318	a	1328.24	3.16990	YES	YES
319	a	1329.64	4.89310	YES	YES
320	a	1335.59	0.50023	YES	YES
321	a	1335.97	0.10953	YES	YES
322	a	1336.14	0.34072	YES	YES
323	a	1336.65	0.24297	YES	YES
324	a	1337.72	0.06388	YES	YES
325	a	1337.84	0.50891	YES	YES
326	a	1338.18	0.33793	YES	YES
327	a	1338.37	0.75897	YES	YES
328	a	1340.09	0.53642	YES	YES
329	a	1343.43	1.10039	YES	YES
330	a	1344.32	5.11520	YES	YES
331	a	1344.48	1.42002	YES	YES
332	a	1345.38	2.03311	YES	YES
333	a	1345.67	0.67160	YES	YES
334	a	1347.03	1.06061	YES	YES
335	a	1349.21	0.77411	YES	YES
336	a	1386.02	7.79515	YES	YES
337	a	1404.87	9.21756	YES	YES
338	a	1407.87	7.80805	YES	YES
339	a	1408.39	5.54545	YES	YES
340	a	1410.46	7.55934	YES	YES
341	a	1424.13	1.34453	YES	YES
342	a	1424.79	4.19172	YES	YES
343	a	1427.52	5.26321	YES	YES
344	a	1428.79	3.83284	YES	YES
345	a	1431.51	2.23636	YES	YES
346	a	1432.49	4.93544	YES	YES
347	a	1433.24	0.65377	YES	YES
348	a	1434.32	10.32519	YES	YES
349	a	1435.12	3.99486	YES	YES
350	a	1435.83	1.36902	YES	YES
351	a	1436.77	4.16310	YES	YES
352	a	1437.04	1.01942	YES	YES
353	a	1437.44	3.28896	YES	YES
354	a	1437.68	1.13883	YES	YES
355	a	1437.90	6.61429	YES	YES
356	a	1437.92	8.43854	YES	YES
357	a	1438.36	2.49661	YES	YES
358	a	1439.28	3.43219	YES	YES
359	a	1439.38	6.81243	YES	YES
360	a	1439.80	2.20078	YES	YES
361	a	1439.87	7.83737	YES	YES
362	a	1441.10	8.60127	YES	YES
363	a	1441.23	6.61065	YES	YES
364	a	1442.93	10.02272	YES	YES
365	a	1443.00	18.83950	YES	YES
366	a	1444.15	6.46514	YES	YES
367	a	1444.51	8.18514	YES	YES
368	a	1445.11	10.94470	YES	YES
369	a	1445.52	19.19745	YES	YES
370	a	1446.11	2.42204	YES	YES
371	a	1447.08	7.15596	YES	YES
372	a	1453.50	0.30102	YES	YES
373	a	1453.90	3.50259	YES	YES
374	a	1454.09	1.30588	YES	YES
375	a	1454.30	0.30775	YES	YES

376	a	1455.22	5.89345	YES	YES
377	a	1456.05	1.30425	YES	YES
378	a	1456.69	7.63711	YES	YES
379	a	1457.60	8.96361	YES	YES
380	a	1465.43	0.40571	YES	YES
381	a	1867.04	111.60849	YES	YES
382	a	2224.90	68.61718	YES	YES
383	a	2911.16	2.39687	YES	YES
384	a	2933.44	12.62811	YES	YES
385	a	2935.06	39.58662	YES	YES
386	a	2937.72	6.58846	YES	YES
387	a	2938.06	9.43971	YES	YES
388	a	2940.59	16.91624	YES	YES
389	a	2940.99	31.76515	YES	YES
390	a	2941.52	5.36939	YES	YES
391	a	2941.94	9.05833	YES	YES
392	a	2942.41	0.37801	YES	YES
393	a	2942.71	27.97717	YES	YES
394	a	2943.58	14.12530	YES	YES
395	a	2945.11	5.63420	YES	YES
396	a	2945.47	5.62989	YES	YES
397	a	2945.72	9.42568	YES	YES
398	a	2946.14	8.06996	YES	YES
399	a	2947.92	11.85030	YES	YES
400	a	2948.07	10.69535	YES	YES
401	a	2948.27	8.10865	YES	YES
402	a	2948.92	19.38412	YES	YES
403	a	2950.79	20.26057	YES	YES
404	a	2951.01	10.73189	YES	YES
405	a	2952.29	3.30903	YES	YES
406	a	2954.33	12.04411	YES	YES
407	a	2954.61	5.61825	YES	YES
408	a	2954.79	6.21382	YES	YES
409	a	2957.05	5.84647	YES	YES
410	a	2958.01	18.02697	YES	YES
411	a	2958.50	5.36679	YES	YES
412	a	2958.94	4.75452	YES	YES
413	a	2959.20	9.15696	YES	YES
414	a	2959.38	24.53555	YES	YES
415	a	2959.48	20.50944	YES	YES
416	a	2959.60	11.59604	YES	YES
417	a	2960.96	5.78084	YES	YES
418	a	2962.82	23.65998	YES	YES
419	a	2963.26	34.22165	YES	YES
420	a	2963.58	45.78461	YES	YES
421	a	2963.75	52.96193	YES	YES
422	a	2964.92	38.85302	YES	YES
423	a	2965.27	31.71324	YES	YES
424	a	2966.94	1.39211	YES	YES
425	a	2969.69	16.64651	YES	YES
426	a	2972.65	9.59786	YES	YES
427	a	2973.02	21.98947	YES	YES
428	a	2973.73	17.90642	YES	YES
429	a	2975.86	28.43609	YES	YES
430	a	2976.16	7.27809	YES	YES
431	a	2979.35	17.16803	YES	YES
432	a	2982.47	2.04425	YES	YES
433	a	2985.25	47.86327	YES	YES

434	a	2986.21	18.55400	YES	YES
435	a	2991.34	35.56827	YES	YES
436	a	2992.43	15.09761	YES	YES
437	a	2995.16	3.07094	YES	YES
438	a	3002.90	7.91160	YES	YES
439	a	3003.18	27.33745	YES	YES
440	a	3003.54	8.70260	YES	YES
441	a	3003.60	20.18948	YES	YES
442	a	3003.82	2.38001	YES	YES
443	a	3006.48	25.42453	YES	YES
444	a	3006.85	14.87199	YES	YES
445	a	3007.84	17.90801	YES	YES
446	a	3010.20	31.12294	YES	YES
447	a	3010.39	14.02503	YES	YES
448	a	3010.92	1.23269	YES	YES
449	a	3010.95	34.23985	YES	YES
450	a	3011.13	21.78044	YES	YES
451	a	3011.32	11.64393	YES	YES
452	a	3013.33	14.41469	YES	YES
453	a	3013.73	19.46109	YES	YES
454	a	3014.23	32.62825	YES	YES
455	a	3014.32	10.71102	YES	YES
456	a	3014.86	9.93301	YES	YES
457	a	3014.96	18.44706	YES	YES
458	a	3015.84	21.05623	YES	YES
459	a	3016.21	13.67480	YES	YES
460	a	3016.29	23.52738	YES	YES
461	a	3016.51	39.58563	YES	YES
462	a	3016.67	17.12743	YES	YES
463	a	3017.16	16.66026	YES	YES
464	a	3017.73	30.38357	YES	YES
465	a	3018.37	38.65352	YES	YES
466	a	3019.45	18.86425	YES	YES
467	a	3019.52	19.78499	YES	YES
468	a	3019.94	23.24554	YES	YES
469	a	3020.33	13.31013	YES	YES
470	a	3020.74	26.94753	YES	YES
471	a	3023.61	26.84224	YES	YES
472	a	3025.03	54.79850	YES	YES
473	a	3025.15	6.06383	YES	YES
474	a	3027.00	3.51489	YES	YES
475	a	3028.99	1.68146	YES	YES
476	a	3029.14	17.40739	YES	YES
477	a	3036.87	25.11073	YES	YES
478	a	3048.19	0.26798	YES	YES
479	a	3052.09	1.05950	YES	YES
480	a	3059.79	1.05876	YES	YES

$end

Double hybrid single point energy = -7381.673797476506 H
COSMO energy + OC correction = -7389.1728423134 H (in *o*DFB)

6.2.42 [{Ga(dcpe)}$_2$(NCCH$_3$)]$^{2+}$ (Cycloadduct)

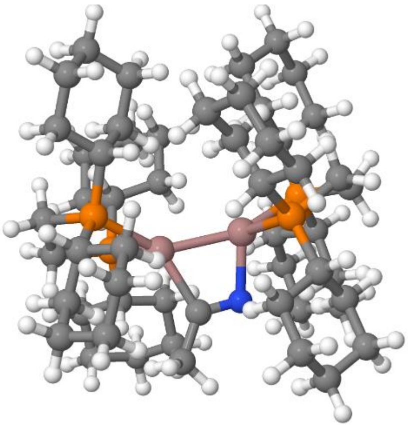

Method: (RI-)BP86(D3BJ)/def2-TZVPP
Symmetry: c1

Cartesian coordinates in Ångström:

Ga	-2.5834570	17.2714063	-2.7598019
Ga	-4.1137959	17.8080044	-0.9661316
P	-1.6836030	18.9272054	-4.2632371
P	-6.1013092	19.1454404	-0.8396918
P	-1.9733328	15.6297384	-4.3807046
P	-5.3730103	16.1819797	0.2722999
C	-5.1788785	20.9089881	-5.4322688
H	-5.3791270	21.4459691	-4.4885870
H	-6.1434565	20.5111605	-5.7774343
C	-0.9842700	14.2625659	-3.6503505
H	-0.6752760	13.5865987	-4.4651986
C	-4.3203994	15.3526485	1.5339900
H	-3.7593520	16.2128949	1.9457844
C	-4.3558563	16.0457371	-5.7628236
H	-3.8191668	16.6169958	-6.5376046
H	-4.5988445	16.7526533	-4.9533725
C	-6.9699211	19.3048086	-2.4537488
H	-6.2617599	19.8747851	-3.0778699
C	-7.2621331	18.2861899	0.3239928
H	-7.7872468	19.0267948	0.9425499
H	-8.0255658	17.8012248	-0.2993275
C	-3.2379243	22.4183332	-6.0156772
H	-2.8188819	23.0899615	-6.7767117
H	-3.3530934	23.0182916	-5.0977347
C	-2.2548670	21.2719888	-5.7433293
H	-1.2952117	21.6750606	-5.3920221
H	-2.0511388	20.7355663	-6.6844279
C	-4.2095508	19.7533530	-5.1554581
H	-4.0993863	19.1553715	-6.0742134
H	-4.6108447	19.0763647	-4.3847665
C	1.0241504	13.7201241	-2.2018783
H	1.4175989	12.9887063	-2.9263213
H	1.8938484	14.1584457	-1.6941017
C	-7.1281580	13.9191502	0.0133633
H	-7.6994959	14.3288430	0.8582871
H	-6.3872393	13.2233152	0.4350258
C	-5.6882632	20.8157324	-0.1966072
H	-6.6089163	21.4208707	-0.1590603
C	-0.4492312	20.5998933	-2.4398949

H	-1.1365274	21.4071819	-2.7312437
H	-0.9600817	19.9935067	-1.6754206
C	0.8309474	21.1966509	-1.8420078
H	1.2830408	21.8901729	-2.5696699
H	0.5734264	21.7962817	-0.9578240
C	-2.1491013	17.8578505	-0.2404696
C	-7.1536272	17.9186719	-3.0968425
H	-6.1828767	17.4013510	-3.1614930
H	-7.8022607	17.3006074	-2.4538391
C	-4.6060873	21.8920647	-6.4576262
H	-4.5064065	21.3834287	-7.4305839
H	-5.3032371	22.7261689	-6.6131296
C	-8.2943056	20.0808347	-2.3795514
H	-8.1309841	21.0833586	-1.9598094
H	-8.9884628	19.5596304	-1.7000913
C	-4.6797095	21.4663956	-1.1650257
H	-5.1372776	21.6081661	-2.1550689
H	-3.8288502	20.7732725	-1.3024289
C	-3.1110187	13.8961469	-6.3198956
H	-2.4922309	13.0856888	-5.9098420
H	-2.5197916	14.3786551	-7.1152302
C	2.1753768	19.2464610	-2.7044857
H	2.6751756	19.8675579	-3.4657297
H	2.8819779	18.4490622	-2.4366272
C	-4.5776877	22.0301929	1.7394900
H	-5.4229363	22.7250947	1.8677601
H	-4.1345658	21.8903934	2.7347860
C	-9.1246488	18.8087385	-4.4123533
H	-9.5532481	18.9145184	-5.4178984
H	-9.8555837	18.2310999	-3.8225635
C	-5.0464193	14.6679190	2.7042047
H	-5.7523673	15.3599969	3.1842497
H	-5.6345447	13.8179546	2.3315110
C	0.1152738	13.0092932	-1.1965082
H	0.6678490	12.2170615	-0.6740407
H	-0.2061921	13.7332120	-0.4291810
C	-7.8051306	18.0327169	-4.4790705
H	-7.1085255	18.5444420	-5.1626394
H	-7.9679110	17.0278103	-4.8925397
C	1.8418498	20.1048862	-1.4814019
H	1.4198997	19.4616360	-0.6917465
H	2.7557077	20.5548804	-1.0713745
C	-3.5577499	22.6399869	0.7736410
H	-3.2081217	23.6091811	1.1527852
H	-2.6703742	21.9862210	0.7165798
C	-5.5960138	14.4647827	-1.9364439
H	-4.8053870	13.7987671	-1.5584606
H	-5.0874784	15.2822245	-2.4706526
C	-5.1016687	20.6853610	1.2218373
H	-4.2697062	19.9589406	1.1950167
H	-5.8571775	20.2826411	1.9121966
C	-6.3940992	15.0331823	-0.7494519
H	-7.1564898	15.7148849	-1.1702057
C	-4.1572204	22.8022566	-0.6256182
C	-7.2841174	12.5834680	-2.1335552
H	-7.9704333	12.0683767	-2.8188483
H	-6.5698694	11.8231364	-1.7768900
C	-8.0559850	13.1509404	-0.9379563

H	-8.5605043	12.3446154	-0.3889878
H	-8.8482431	13.8285995	-1.2971911
C	-1.8902805	13.4992547	-2.6632228
H	-2.7393885	13.0438607	-3.1925821
H	-2.3116504	14.2245958	-1.9459380
C	-4.0212294	14.1641296	3.7304290
H	-3.5023994	15.0279354	4.1780417
H	-4.5450088	13.6550944	4.5506816
C	-5.6350445	15.4635634	-6.3741214
H	-6.2513632	16.2758042	-6.7837055
H	-6.2273157	14.9880307	-5.5756648
C	-2.9942286	13.2283996	3.0847313
H	-3.5037860	12.3159144	2.7330750
H	-2.2562522	12.9039257	3.8303207
C	-3.2951922	14.4180138	0.8677720
H	-3.8234960	13.5636821	0.4153653
H	-2.7721232	14.9467481	0.0567443
C	-8.9333990	20.1872094	-3.7716591
H	-9.8949032	20.7121254	-3.6949370
H	-8.2884787	20.8072001	-4.4152228
C	-4.4003035	13.3233140	-6.9259543
H	-4.1472994	12.6138520	-7.7250136
H	-4.9338670	12.7459288	-6.1527555
C	-6.5191645	13.6835939	-2.8769986
H	-5.9329994	13.2507838	-3.7012729
H	-7.2362753	14.3842207	-3.3377111
C	-5.3142621	14.4307395	-7.4596648
H	-6.2423929	13.9978219	-7.8559617
H	-4.8200498	14.9347084	-8.3066589
C	-1.1149402	12.4240591	-1.8937422
H	-1.7864099	11.9456203	-1.1663205
H	-0.7997322	11.6332214	-2.5931139
C	-2.2891198	13.9020551	1.9031695
H	-1.6844754	14.7495026	2.2656486
H	-1.5908202	13.2038046	1.4217407
C	-1.6475158	18.3168475	1.0962370
N	-1.4094893	17.3633908	-1.1532249
C	-0.1304652	19.7017997	-3.6521629
H	0.2613535	20.3265223	-4.4733655
C	0.9090008	18.6205411	-3.3011372
H	1.1688471	18.0394901	-4.1983355
H	0.4682534	17.9293475	-2.5651558
C	0.2649306	14.8260128	-2.9443166
H	0.9320477	15.3093553	-3.6717187
H	-0.0434995	15.5999834	-2.2216736
C	-0.9304567	16.5235472	-5.6186015
H	0.0968177	16.4479621	-5.2399136
H	-0.9563399	15.9873660	-6.5773178
C	-1.3089378	18.0043844	-5.8264815
H	-2.2106326	18.0825268	-6.4481869
H	-0.5045671	18.5212976	-6.3676306
C	-3.4445416	14.9264676	-5.2308608
H	-3.9875635	14.4139050	-4.4181123
C	-2.8391045	20.2899686	-4.7142004
H	-2.9841966	20.8245033	-3.7589965
C	-6.5583169	17.2447701	1.2138614
H	-7.3009787	16.6231857	1.7325411
H	-5.9592986	17.7432580	1.9897168

```
H    -4.9841936   23.5292913   -0.5899439
H    -3.4122553   23.2112255   -1.3226914
H    -0.5816117   18.0840829    1.2242410
H    -2.2253819   17.8623014    1.9141540
H    -1.7905340   19.4051611    1.1858095

SCF energy GEOOPT =  -7389.017622100 H
ZPE =   3716. kJ/mol
FREEH energy =  3903.63 kJ/mol
FREEH entropy =   1.61005 kJ/mol/K

$vibrational spectrum
#  mode     symmetry     wave number   IR intensity    selection rules
#                         cm**(-1)        km/mol          IR     RAMAN
     1                      -0.00         0.00000         -        -
     2                       0.00         0.00000         -        -
     3                       0.00         0.00000         -        -
     4                       0.00         0.00000         -        -
     5                       0.00         0.00000         -        -
     6                       0.00         0.00000         -        -
     7         a            11.64         0.04883        YES      YES
     8         a            18.41         0.05663        YES      YES
     9         a            21.52         0.07816        YES      YES
    10         a            22.56         0.01737        YES      YES
    11         a            26.88         0.01137        YES      YES
    12         a            28.74         0.05278        YES      YES
    13         a            30.10         0.18842        YES      YES
    14         a            33.62         0.06499        YES      YES
    15         a            36.52         0.01367        YES      YES
    16         a            36.90         0.02061        YES      YES
    17         a            38.84         0.04729        YES      YES
    18         a            44.13         0.04945        YES      YES
    19         a            45.04         0.07364        YES      YES
    20         a            45.96         0.01488        YES      YES
    21         a            49.75         0.09731        YES      YES
    22         a            51.24         0.11849        YES      YES
    23         a            55.61         0.41745        YES      YES
    24         a            55.92         0.06585        YES      YES
    25         a            59.80         0.29108        YES      YES
    26         a            62.30         0.20116        YES      YES
    27         a            63.19         0.29464        YES      YES
    28         a            66.15         0.05564        YES      YES
    29         a            68.65         0.15890        YES      YES
    30         a            73.53         0.16444        YES      YES
    31         a            75.77         0.21415        YES      YES
    32         a            77.51         0.30471        YES      YES
    33         a            85.75         0.74210        YES      YES
    34         a            87.45         0.48940        YES      YES
    35         a            91.53         0.19794        YES      YES
    36         a            94.28         1.12795        YES      YES
    37         a           101.24         0.26515        YES      YES
    38         a           115.91         0.33491        YES      YES
    39         a           124.07         1.00492        YES      YES
    40         a           129.60         0.39453        YES      YES
    41         a           134.33         0.29132        YES      YES
    42         a           135.30         1.52134        YES      YES
    43         a           137.78         1.79887        YES      YES
    44         a           140.25         0.31831        YES      YES
```

45	a	142.48	0.52844	YES	YES
46	a	152.83	0.15218	YES	YES
47	a	156.72	1.60496	YES	YES
48	a	165.35	0.22765	YES	YES
49	a	170.99	0.31276	YES	YES
50	a	179.10	2.62641	YES	YES
51	a	182.92	0.18755	YES	YES
52	a	187.07	0.37662	YES	YES
53	a	195.83	2.31462	YES	YES
54	a	206.57	7.78124	YES	YES
55	a	209.25	1.81686	YES	YES
56	a	211.14	1.08994	YES	YES
57	a	216.57	1.80918	YES	YES
58	a	216.88	0.07632	YES	YES
59	a	220.38	1.09136	YES	YES
60	a	222.23	2.62803	YES	YES
61	a	224.29	0.55063	YES	YES
62	a	225.32	0.82179	YES	YES
63	a	228.67	0.08217	YES	YES
64	a	230.37	6.28211	YES	YES
65	a	232.64	1.09148	YES	YES
66	a	236.79	0.69141	YES	YES
67	a	239.76	0.66549	YES	YES
68	a	242.85	0.19815	YES	YES
69	a	246.37	0.83440	YES	YES
70	a	255.52	1.92106	YES	YES
71	a	275.91	2.12425	YES	YES
72	a	279.20	0.28839	YES	YES
73	a	283.40	0.17379	YES	YES
74	a	292.15	8.04355	YES	YES
75	a	294.68	20.28974	YES	YES
76	a	302.51	6.05629	YES	YES
77	a	305.14	2.10399	YES	YES
78	a	311.42	0.18314	YES	YES
79	a	314.42	0.82262	YES	YES
80	a	328.78	0.48296	YES	YES
81	a	329.82	0.91340	YES	YES
82	a	333.53	0.23195	YES	YES
83	a	336.30	0.20017	YES	YES
84	a	340.63	0.37825	YES	YES
85	a	342.90	1.22076	YES	YES
86	a	371.86	0.72960	YES	YES
87	a	374.20	1.98574	YES	YES
88	a	375.70	3.44159	YES	YES
89	a	380.29	1.28208	YES	YES
90	a	393.46	0.43381	YES	YES
91	a	394.68	1.20676	YES	YES
92	a	404.47	7.98572	YES	YES
93	a	407.11	0.85185	YES	YES
94	a	410.46	2.18055	YES	YES
95	a	413.13	0.80890	YES	YES
96	a	420.62	3.68634	YES	YES
97	a	422.77	1.94610	YES	YES
98	a	424.31	2.39302	YES	YES
99	a	427.33	0.33749	YES	YES
100	a	429.48	0.01243	YES	YES
101	a	429.89	0.20643	YES	YES
102	a	430.18	0.13319	YES	YES

103	a	430.53	0.26534	YES	YES
104	a	432.02	0.46596	YES	YES
105	a	432.36	0.73197	YES	YES
106	a	434.98	0.95184	YES	YES
107	a	438.34	0.20933	YES	YES
108	a	445.28	2.14306	YES	YES
109	a	456.43	4.02726	YES	YES
110	a	461.35	5.21779	YES	YES
111	a	471.14	4.00949	YES	YES
112	a	491.64	3.50925	YES	YES
113	a	493.24	4.44995	YES	YES
114	a	494.80	4.83915	YES	YES
115	a	496.21	0.26583	YES	YES
116	a	502.83	1.85369	YES	YES
117	a	503.90	0.59769	YES	YES
118	a	513.64	6.52394	YES	YES
119	a	514.93	14.05281	YES	YES
120	a	620.44	60.47473	YES	YES
121	a	625.48	10.88697	YES	YES
122	a	631.60	20.49765	YES	YES
123	a	640.49	10.75620	YES	YES
124	a	656.33	12.50079	YES	YES
125	a	699.29	2.13160	YES	YES
126	a	718.30	1.82624	YES	YES
127	a	725.90	1.59696	YES	YES
128	a	729.23	4.12438	YES	YES
129	a	732.89	8.74392	YES	YES
130	a	736.91	1.83415	YES	YES
131	a	739.69	3.44434	YES	YES
132	a	741.68	3.46878	YES	YES
133	a	770.22	1.44274	YES	YES
134	a	771.05	0.99663	YES	YES
135	a	772.22	0.57036	YES	YES
136	a	772.95	1.42184	YES	YES
137	a	773.88	0.03860	YES	YES
138	a	775.28	0.26823	YES	YES
139	a	776.17	11.34837	YES	YES
140	a	776.26	0.77799	YES	YES
141	a	777.19	3.33710	YES	YES
142	a	778.46	12.12734	YES	YES
143	a	805.73	0.24284	YES	YES
144	a	808.15	4.01350	YES	YES
145	a	808.81	9.93945	YES	YES
146	a	810.96	3.80589	YES	YES
147	a	811.72	0.10916	YES	YES
148	a	813.05	3.35571	YES	YES
149	a	814.88	1.66549	YES	YES
150	a	816.58	4.48479	YES	YES
151	a	830.34	4.68730	YES	YES
152	a	831.32	5.81545	YES	YES
153	a	836.19	1.06114	YES	YES
154	a	837.71	3.01418	YES	YES
155	a	838.07	1.75566	YES	YES
156	a	838.62	0.89778	YES	YES
157	a	838.93	6.64348	YES	YES
158	a	839.92	5.67576	YES	YES
159	a	847.54	15.12049	YES	YES
160	a	852.50	13.94880	YES	YES

161	a	868.99	3.76531	YES	YES
162	a	870.92	1.37949	YES	YES
163	a	872.91	3.09562	YES	YES
164	a	875.80	0.48271	YES	YES
165	a	876.51	1.56802	YES	YES
166	a	877.66	3.12820	YES	YES
167	a	878.07	3.68536	YES	YES
168	a	878.99	2.68405	YES	YES
169	a	879.11	2.43245	YES	YES
170	a	880.51	0.77731	YES	YES
171	a	880.83	3.84672	YES	YES
172	a	881.26	0.82880	YES	YES
173	a	882.47	3.93982	YES	YES
174	a	883.39	11.88328	YES	YES
175	a	884.92	5.85091	YES	YES
176	a	886.33	0.26103	YES	YES
177	a	901.93	2.63465	YES	YES
178	a	902.95	0.84110	YES	YES
179	a	903.79	0.85107	YES	YES
180	a	904.26	4.49654	YES	YES
181	a	906.67	3.52487	YES	YES
182	a	908.94	1.21146	YES	YES
183	a	910.37	2.01090	YES	YES
184	a	911.06	0.48362	YES	YES
185	a	934.72	19.88313	YES	YES
186	a	969.23	4.37165	YES	YES
187	a	977.07	0.39112	YES	YES
188	a	977.70	0.06903	YES	YES
189	a	986.95	2.29292	YES	YES
190	a	987.35	14.55294	YES	YES
191	a	988.24	11.16761	YES	YES
192	a	988.59	8.03933	YES	YES
193	a	989.23	2.99420	YES	YES
194	a	990.08	10.46381	YES	YES
195	a	991.09	9.01993	YES	YES
196	a	991.91	22.08582	YES	YES
197	a	1013.18	0.44136	YES	YES
198	a	1014.79	2.46083	YES	YES
199	a	1015.46	0.07175	YES	YES
200	a	1016.49	0.67777	YES	YES
201	a	1017.23	0.22193	YES	YES
202	a	1019.76	1.11092	YES	YES
203	a	1019.91	0.40207	YES	YES
204	a	1022.30	0.47809	YES	YES
205	a	1027.63	1.28919	YES	YES
206	a	1028.53	0.91838	YES	YES
207	a	1030.28	0.98566	YES	YES
208	a	1035.43	1.59409	YES	YES
209	a	1037.72	3.60574	YES	YES
210	a	1038.06	1.69660	YES	YES
211	a	1039.73	2.92895	YES	YES
212	a	1040.24	0.39369	YES	YES
213	a	1054.35	9.02646	YES	YES
214	a	1057.50	11.81945	YES	YES
215	a	1059.68	1.99364	YES	YES
216	a	1060.10	7.36869	YES	YES
217	a	1060.38	7.25884	YES	YES
218	a	1062.29	32.89306	YES	YES

219	a	1064.99	7.19078	YES	YES
220	a	1066.47	2.04223	YES	YES
221	a	1069.62	0.52206	YES	YES
222	a	1069.98	0.50998	YES	YES
223	a	1071.74	0.18915	YES	YES
224	a	1071.82	0.08648	YES	YES
225	a	1071.90	0.46554	YES	YES
226	a	1072.54	0.32464	YES	YES
227	a	1072.71	0.32999	YES	YES
228	a	1073.37	0.03068	YES	YES
229	a	1073.94	0.12033	YES	YES
230	a	1077.35	5.50797	YES	YES
231	a	1081.65	3.10621	YES	YES
232	a	1087.42	6.61228	YES	YES
233	a	1091.29	4.78125	YES	YES
234	a	1093.81	0.79464	YES	YES
235	a	1095.03	4.66224	YES	YES
236	a	1102.12	0.83567	YES	YES
237	a	1102.52	3.40850	YES	YES
238	a	1110.75	9.35711	YES	YES
239	a	1115.08	8.44616	YES	YES
240	a	1121.97	1.06202	YES	YES
241	a	1129.82	3.78224	YES	YES
242	a	1158.50	4.23515	YES	YES
243	a	1159.58	1.52537	YES	YES
244	a	1160.90	7.34004	YES	YES
245	a	1165.83	7.14365	YES	YES
246	a	1166.61	7.89086	YES	YES
247	a	1167.34	10.12959	YES	YES
248	a	1168.89	12.33977	YES	YES
249	a	1170.16	3.93332	YES	YES
250	a	1172.41	7.03849	YES	YES
251	a	1174.37	9.06243	YES	YES
252	a	1178.05	5.92831	YES	YES
253	a	1179.13	7.14767	YES	YES
254	a	1186.99	0.36300	YES	YES
255	a	1188.62	1.54203	YES	YES
256	a	1191.33	0.95365	YES	YES
257	a	1210.19	1.19245	YES	YES
258	a	1236.27	0.70496	YES	YES
259	a	1240.76	0.35702	YES	YES
260	a	1241.30	0.35680	YES	YES
261	a	1245.38	0.20002	YES	YES
262	a	1246.63	0.07369	YES	YES
263	a	1247.16	0.58296	YES	YES
264	a	1248.29	0.16263	YES	YES
265	a	1249.02	1.88257	YES	YES
266	a	1249.66	0.72900	YES	YES
267	a	1252.24	1.42641	YES	YES
268	a	1252.63	0.40892	YES	YES
269	a	1254.75	3.06917	YES	YES
270	a	1255.56	2.53107	YES	YES
271	a	1255.73	5.79036	YES	YES
272	a	1256.05	0.33122	YES	YES
273	a	1256.26	1.50557	YES	YES
274	a	1256.95	3.91914	YES	YES
275	a	1257.21	2.59761	YES	YES
276	a	1258.47	1.62621	YES	YES

277	a	1258.95	0.27685	YES	YES
278	a	1260.52	1.43049	YES	YES
279	a	1261.26	3.96993	YES	YES
280	a	1262.30	2.10936	YES	YES
281	a	1263.53	0.31881	YES	YES
282	a	1265.02	0.63844	YES	YES
283	a	1267.26	1.60679	YES	YES
284	a	1269.60	1.02687	YES	YES
285	a	1272.32	1.85443	YES	YES
286	a	1280.29	1.67195	YES	YES
287	a	1281.48	0.49510	YES	YES
288	a	1283.29	3.67597	YES	YES
289	a	1284.10	7.17397	YES	YES
290	a	1284.62	0.88898	YES	YES
291	a	1287.14	4.24037	YES	YES
292	a	1289.37	3.00596	YES	YES
293	a	1291.77	3.31856	YES	YES
294	a	1304.57	0.47937	YES	YES
295	a	1309.65	1.66302	YES	YES
296	a	1310.14	1.09657	YES	YES
297	a	1311.14	0.93730	YES	YES
298	a	1312.86	1.87702	YES	YES
299	a	1313.29	0.68876	YES	YES
300	a	1317.99	0.20683	YES	YES
301	a	1319.53	3.32505	YES	YES
302	a	1320.44	0.77630	YES	YES
303	a	1320.74	0.47853	YES	YES
304	a	1321.78	0.75437	YES	YES
305	a	1321.96	0.14792	YES	YES
306	a	1323.58	1.66583	YES	YES
307	a	1323.97	1.55751	YES	YES
308	a	1324.27	1.09512	YES	YES
309	a	1324.50	1.35778	YES	YES
310	a	1325.07	2.96560	YES	YES
311	a	1325.33	1.90501	YES	YES
312	a	1325.85	1.53331	YES	YES
313	a	1326.48	3.65094	YES	YES
314	a	1327.52	7.40155	YES	YES
315	a	1329.11	1.41456	YES	YES
316	a	1331.88	9.49989	YES	YES
317	a	1336.13	0.29362	YES	YES
318	a	1336.34	0.06874	YES	YES
319	a	1336.78	0.16597	YES	YES
320	a	1337.28	0.50013	YES	YES
321	a	1337.75	0.43464	YES	YES
322	a	1338.57	0.22034	YES	YES
323	a	1338.84	0.67371	YES	YES
324	a	1339.05	0.48232	YES	YES
325	a	1340.00	0.30231	YES	YES
326	a	1342.13	1.08958	YES	YES
327	a	1343.83	0.13193	YES	YES
328	a	1344.86	0.66695	YES	YES
329	a	1345.37	1.49325	YES	YES
330	a	1345.61	0.96377	YES	YES
331	a	1346.64	2.65293	YES	YES
332	a	1347.01	0.50245	YES	YES
333	a	1347.47	0.88242	YES	YES
334	a	1350.01	0.95150	YES	YES

335	a	1400.47	7.40521	YES	YES
336	a	1404.44	7.58656	YES	YES
337	a	1404.66	8.31742	YES	YES
338	a	1406.93	17.30723	YES	YES
339	a	1409.16	11.69148	YES	YES
340	a	1415.63	11.40858	YES	YES
341	a	1423.34	5.25614	YES	YES
342	a	1426.79	5.32552	YES	YES
343	a	1426.98	0.73240	YES	YES
344	a	1429.40	1.71175	YES	YES
345	a	1431.14	1.94379	YES	YES
346	a	1431.70	1.73698	YES	YES
347	a	1432.20	3.32962	YES	YES
348	a	1433.31	2.76844	YES	YES
349	a	1435.16	3.72702	YES	YES
350	a	1435.47	2.56385	YES	YES
351	a	1435.82	4.28664	YES	YES
352	a	1436.56	6.12995	YES	YES
353	a	1436.72	4.77669	YES	YES
354	a	1436.91	8.18518	YES	YES
355	a	1437.63	2.77624	YES	YES
356	a	1438.17	14.26703	YES	YES
357	a	1438.33	5.81959	YES	YES
358	a	1438.69	4.84349	YES	YES
359	a	1438.82	2.26762	YES	YES
360	a	1438.96	4.10287	YES	YES
361	a	1439.55	2.54551	YES	YES
362	a	1439.98	8.53234	YES	YES
363	a	1440.17	3.98706	YES	YES
364	a	1441.90	9.10643	YES	YES
365	a	1442.69	14.19176	YES	YES
366	a	1443.30	6.40466	YES	YES
367	a	1443.83	23.19729	YES	YES
368	a	1444.23	13.73167	YES	YES
369	a	1444.53	18.53765	YES	YES
370	a	1444.87	0.99718	YES	YES
371	a	1445.24	2.35466	YES	YES
372	a	1447.77	10.07392	YES	YES
373	a	1452.69	3.31411	YES	YES
374	a	1453.56	2.05996	YES	YES
375	a	1453.75	1.05667	YES	YES
376	a	1453.92	1.02397	YES	YES
377	a	1455.31	1.39905	YES	YES
378	a	1455.99	3.59897	YES	YES
379	a	1456.60	2.31805	YES	YES
380	a	1459.29	1.34333	YES	YES
381	a	1570.28	146.99778	YES	YES
382	a	2909.35	19.22158	YES	YES
383	a	2912.87	3.40088	YES	YES
384	a	2920.28	3.04743	YES	YES
385	a	2923.79	13.00602	YES	YES
386	a	2929.76	21.51867	YES	YES
387	a	2934.00	29.63977	YES	YES
388	a	2934.19	12.50547	YES	YES
389	a	2936.36	4.21166	YES	YES
390	a	2937.06	13.24185	YES	YES
391	a	2937.53	11.53104	YES	YES
392	a	2938.28	6.83827	YES	YES

393	a	2938.98	34.98136	YES	YES
394	a	2942.13	12.82656	YES	YES
395	a	2943.52	11.44762	YES	YES
396	a	2944.60	8.48432	YES	YES
397	a	2945.21	12.79226	YES	YES
398	a	2946.12	3.84136	YES	YES
399	a	2946.13	12.19369	YES	YES
400	a	2946.52	0.94840	YES	YES
401	a	2946.66	15.65936	YES	YES
402	a	2946.85	6.90356	YES	YES
403	a	2947.73	14.81066	YES	YES
404	a	2948.29	11.08144	YES	YES
405	a	2948.42	18.15623	YES	YES
406	a	2948.79	2.90097	YES	YES
407	a	2949.61	32.83775	YES	YES
408	a	2951.45	11.90182	YES	YES
409	a	2951.65	7.46363	YES	YES
410	a	2953.53	3.00920	YES	YES
411	a	2953.94	1.79586	YES	YES
412	a	2955.28	2.72302	YES	YES
413	a	2955.96	4.97095	YES	YES
414	a	2956.12	7.45572	YES	YES
415	a	2957.27	18.63838	YES	YES
416	a	2957.82	1.95141	YES	YES
417	a	2958.70	3.51228	YES	YES
418	a	2959.09	10.52463	YES	YES
419	a	2959.49	26.54050	YES	YES
420	a	2959.65	20.45039	YES	YES
421	a	2959.90	4.77894	YES	YES
422	a	2960.57	32.96611	YES	YES
423	a	2960.70	17.45429	YES	YES
424	a	2961.37	33.50358	YES	YES
425	a	2961.78	59.60477	YES	YES
426	a	2962.47	18.96868	YES	YES
427	a	2962.58	29.12400	YES	YES
428	a	2964.74	1.47168	YES	YES
429	a	2965.17	20.87522	YES	YES
430	a	2978.06	7.59093	YES	YES
431	a	2979.97	34.22705	YES	YES
432	a	2980.24	4.95763	YES	YES
433	a	2984.13	15.79671	YES	YES
434	a	2985.82	4.56028	YES	YES
435	a	2986.25	6.97892	YES	YES
436	a	2987.62	39.27811	YES	YES
437	a	2987.75	29.71745	YES	YES
438	a	2989.66	11.45682	YES	YES
439	a	2990.99	24.62464	YES	YES
440	a	2994.55	20.48665	YES	YES
441	a	2995.88	17.56796	YES	YES
442	a	2997.44	35.23137	YES	YES
443	a	2999.81	22.07126	YES	YES
444	a	3000.23	31.80287	YES	YES
445	a	3002.76	7.53422	YES	YES
446	a	3003.43	22.21170	YES	YES
447	a	3003.72	30.78953	YES	YES
448	a	3005.02	21.09763	YES	YES
449	a	3005.71	21.90740	YES	YES
450	a	3006.20	20.62735	YES	YES

451	a	3006.69	15.93016	YES	YES
452	a	3009.13	16.32788	YES	YES
453	a	3009.18	21.75649	YES	YES
454	a	3009.25	21.89816	YES	YES
455	a	3010.18	17.31359	YES	YES
456	a	3010.23	11.34524	YES	YES
457	a	3010.87	21.45153	YES	YES
458	a	3012.33	29.04403	YES	YES
459	a	3012.44	20.51608	YES	YES
460	a	3014.47	19.86286	YES	YES
461	a	3015.01	19.46602	YES	YES
462	a	3015.33	17.80355	YES	YES
463	a	3016.32	19.65524	YES	YES
464	a	3016.45	28.57043	YES	YES
465	a	3016.86	28.16573	YES	YES
466	a	3017.06	25.94595	YES	YES
467	a	3017.29	23.17833	YES	YES
468	a	3017.57	24.33446	YES	YES
469	a	3018.14	20.79165	YES	YES
470	a	3018.39	25.53827	YES	YES
471	a	3018.44	8.94328	YES	YES
472	a	3018.57	39.11859	YES	YES
473	a	3019.09	24.00755	YES	YES
474	a	3019.20	33.03766	YES	YES
475	a	3024.31	20.64434	YES	YES
476	a	3025.68	11.62290	YES	YES
477	a	3029.67	0.47216	YES	YES
478	a	3036.43	1.18054	YES	YES
479	a	3038.76	10.67019	YES	YES
480	a	3043.87	0.27687	YES	YES

$end

Double hybrid single point energy = -7381.660502283365 H
COSMO energy + OC correction = -7389.1667075968 H (in *o*DFB)

6.2.43 PhCN

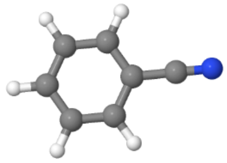

Method: /def2-TZVPP
Symmetry: c2v

Cartesian coordinates in Ångström:

N	0.0000000	0.0000000	-3.6582627
C	0.0000000	0.0000000	1.7296030
C	0.0000000	0.0000000	-2.4927647
C	-1.2113566	0.0000000	1.0321792
C	1.2113566	0.0000000	1.0321792
C	-1.2185765	0.0000000	-0.3605540
C	1.2185765	0.0000000	-0.3605540
C	0.0000000	0.0000000	-1.0634635
H	0.0000000	0.0000000	2.8192397

```
H    -2.1554361    0.0000000    1.5757986
H     2.1554361    0.0000000    1.5757986
H    -2.1559489    0.0000000   -0.9141959
H     2.1559489    0.0000000   -0.9141959

SCF energy GEOOPT =  -324.6460562709 H
ZPE =   253.2 kJ/mol
FREEH energy =   269.61 kJ/mol
FREEH entropy =   0.32567 kJ/mol/K

$vibrational spectrum
#  mode     symmetry     wave number   IR intensity    selection rules
#                         cm**(-1)        km/mol          IR     RAMAN
     1                      -0.00         0.00000         -        -
     2                      -0.00         0.00000         -        -
     3                       0.00         0.00000         -        -
     4                       0.00         0.00000         -        -
     5                       0.00         0.00000         -        -
     6                       0.00         0.00000         -        -
     7        b2           138.99         1.70591        YES      YES
     8        b1           160.11         4.11861        YES      YES
     9        b2           374.94         0.42485        YES      YES
    10        a2           392.80         0.00000        NO       YES
    11        a1           454.45         0.00079        YES      YES
    12        b1           551.14         0.11741        YES      YES
    13        b2           551.53        16.48498        YES      YES
    14        b1           621.31         0.13006        YES      YES
    15        b2           686.21        35.42973        YES      YES
    16        b2           754.65        36.42336        YES      YES
    17        a1           757.11         1.74200        YES      YES
    18        a2           831.75         0.00000        NO       YES
    19        b2           914.93         3.27362        YES      YES
    20        a2           958.30         0.00000        NO       YES
    21        b2           980.33         0.05600        YES      YES
    22        a1           994.20         0.26028        YES      YES
    23        a1          1025.33         3.62139        YES      YES
    24        b1          1077.25         4.54144        YES      YES
    25        b1          1155.83         0.04640        YES      YES
    26        a1          1168.45         0.72867        YES      YES
    27        a1          1195.11         0.48480        YES      YES
    28        b1          1301.83         0.05302        YES      YES
    29        b1          1337.25         1.28358        YES      YES
    30        b1          1437.06         6.27502        YES      YES
    31        a1          1480.55         7.40501        YES      YES
    32        b1          1570.04         1.11182        YES      YES
    33        a1          1596.68         0.67923        YES      YES
    34        a1          2244.73        24.68242        YES      YES
    35        a1          3106.37         0.01611        YES      YES
    36        b1          3116.29         3.85991        YES      YES
    37        a1          3124.49         8.65743        YES      YES
    38        b1          3133.31         7.45263        YES      YES
    39        a1          3136.05         2.73495        YES      YES
$end

Double hybrid single point energy = -324.058476872803 H
COSMO energy + OC correction = -324.6556969616 H (in oDFB)
```

6.2.44 [{Ga(dcpe)}$_2$(NCPh)]$^{2+}$ (Cycloadduct)

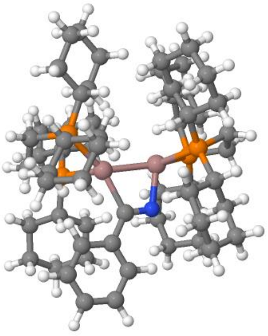

```
Method: (RI-)BP86(D3BJ)/def2-TZVPP
Symmetry: c1

Cartesian coordinates in Ångström:
Ga   -2.6146066   17.2977245   -2.8358805
Ga   -4.0171652   17.8008193   -0.9181935
P    -1.6946771   18.9507195   -4.3222075
P    -5.9901436   19.1732293   -0.7851658
P    -2.0204154   15.6569466   -4.4652595
P    -5.2984871   16.1834038    0.3161008
C    -5.1922988   20.9174197   -5.4917933
H    -5.4067317   21.4370364   -4.5415497
H    -6.1491880   20.5132905   -5.8509989
C    -1.0538266   14.2788812   -3.7183955
H    -0.8123914   13.5569378   -4.5163896
C    -4.2340206   15.3661608    1.5716640
H    -3.6791365   16.2348941    1.9710942
C    -4.4087033   16.0667524   -5.8446826
H    -3.8820059   16.6620833   -6.6084475
H    -4.6628212   16.7537776   -5.0217827
C    -6.8519986   19.2675532   -2.4129537
H    -6.1424769   19.8188769   -3.0520920
C    -7.1526825   18.3236009    0.3861963
H    -7.6602704   19.0701551    1.0120122
H    -7.9295870   17.8525608   -0.2310245
C    -3.2611992   22.4549437   -6.0392825
H    -2.8445616   23.1417847   -6.7879660
H    -3.3881255   23.0403497   -5.1135168
C    -2.2679141   21.3149027   -5.7766475
H    -1.3146838   21.7220462   -5.4125064
H    -2.0521377   20.7936069   -6.7235882
C    -4.2115491   19.7695182   -5.2257000
H    -4.0878342   19.1873824   -6.1528615
H    -4.6109037   19.0753801   -4.4693176
C     0.9754112   13.6964748   -2.3132584
H     1.3037432   12.9086167   -3.0105323
H     1.8852796   14.1120385   -1.8591501
C    -7.0524864   13.9190915    0.0897527
H    -7.6225331   14.3318506    0.9340718
H    -6.3033904   13.2337927    0.5141092
C    -5.6592973   20.8919872   -0.2240014
H    -6.6081434   21.4523359   -0.2619462
C    -0.5111549   20.5423670   -2.4125675
H    -1.1895716   21.3647648   -2.6819743
H    -1.0456183   19.8972772   -1.6971306
```

C	0.7462556	21.1036003	-1.7377181
H	1.2224135	21.8391841	-2.4062728
H	0.4558246	21.6478416	-0.8284129
C	-2.0249977	17.6418403	-0.2943698
C	-7.0356622	17.8653155	-3.0164747
H	-6.0665282	17.3437210	-3.0606096
H	-7.6910186	17.2686432	-2.3607303
C	-4.6216730	21.9219604	-6.4976760
H	-4.5107808	21.4294030	-7.4776695
H	-5.3257274	22.7517198	-6.6451229
C	-8.1781226	20.0437598	-2.3669417
H	-8.0190908	21.0593524	-1.9789541
H	-8.8729406	19.5425572	-1.6731521
C	-4.6456346	21.5303727	-1.1951189
H	-5.0588631	21.5668261	-2.2139037
H	-3.7476615	20.8869280	-1.2363398
C	-3.1304256	13.9488780	-6.4436439
H	-2.4974761	13.1410825	-6.0505546
H	-2.5488948	14.4573376	-7.2298954
C	2.1267773	19.2148900	-2.6709947
H	2.6477752	19.8866137	-3.3726724
H	2.8300486	18.4054241	-2.4314942
C	-4.7332670	22.3138725	1.6583419
H	-5.6346208	22.9465918	1.6955894
H	-4.3315889	22.2803209	2.6800745
C	-9.0012250	18.7134424	-4.3660766
H	-9.4260806	18.7907208	-5.3758601
H	-9.7325568	18.1493739	-3.7637959
C	-4.9353724	14.6862184	2.7584808
H	-5.6661870	15.3632614	3.2228974
H	-5.4904139	13.8024274	2.4138519
C	0.0677856	13.0871723	-1.2422555
H	0.5986602	12.2981302	-0.6930058
H	-0.1970372	13.8665762	-0.5081078
C	-7.6788333	17.9412007	-4.4051528
H	-6.9803874	18.4378434	-5.0981872
H	-7.8354948	16.9253201	-4.7932054
C	1.7450527	19.9915156	-1.4079744
H	1.2929991	19.3012680	-0.6763902
H	2.6414427	20.4147291	-0.9354889
C	-3.7113651	22.9289378	0.6974589
H	-3.4584929	23.9493780	1.0139994
H	-2.7765118	22.3450190	0.7386695
C	-5.5353621	14.4567614	-1.8718142
H	-4.7427484	13.7966893	-1.4879223
H	-5.0288416	15.2702247	-2.4143100
C	-5.1237988	20.8981830	1.2170051
H	-4.2300921	20.2541192	1.2617128
H	-5.8664987	20.4733172	1.9082797
C	-6.3321524	15.0319380	-0.6871885
H	-7.1007801	15.7061038	-1.1073757
C	-4.2422030	22.9373122	-0.7388956
C	-7.2115977	12.5656165	-2.0480632
H	-7.8994659	12.0416317	-2.7250600
H	-6.4903698	11.8121397	-1.6909470
C	-7.9793890	13.1360702	-0.8507764
H	-8.4739331	12.3293671	-0.2932989
H	-8.7794761	13.8047047	-1.2095941

C	-1.9573566	13.6022976	-2.6666391
H	-2.8457360	13.1664906	-3.1456082
H	-2.3163858	14.3778250	-1.9688579
C	-3.8827406	14.2592278	3.7925514
H	-3.3958798	15.1601460	4.2010946
H	-4.3775921	13.7576568	4.6351724
C	-5.6786787	15.4732582	-6.4645431
H	-6.3122960	16.2811313	-6.8556879
H	-6.2585747	14.9702530	-5.6732290
C	-2.8222895	13.3451513	3.1695970
H	-3.2959308	12.3997438	2.8566047
H	-2.0650948	13.0795232	3.9194506
C	-3.1923125	14.4433188	0.9170426
H	-3.6967500	13.5566265	0.5003308
H	-2.6960843	14.9607379	0.0828959
C	-8.8159602	20.1088341	-3.7621469
H	-9.7793510	20.6323499	-3.7002646
H	-8.1726004	20.7135321	-4.4213432
C	-4.4100962	13.3657511	-7.0599357
H	-4.1455447	12.6766118	-7.8729446
H	-4.9331885	12.7645727	-6.2978741
C	-6.4570060	13.6647278	-2.8041452
H	-5.8714257	13.2290280	-3.6276203
H	-7.1798215	14.3580331	-3.2670568
C	-5.3428569	14.4679501	-7.5709760
H	-6.2644811	14.0278103	-7.9744446
H	-4.8584155	14.9968685	-8.4083792
C	-1.2088966	12.5268043	-1.8724750
H	-1.8780399	12.1163933	-1.1024972
H	-0.9525599	11.6897352	-2.5414216
C	-2.1555315	14.0046770	1.9581032
H	-1.5859237	14.8910475	2.2817031
H	-1.4334261	13.3186185	1.4942900
N	-1.3610527	17.2111870	-1.2987242
C	-0.1552253	19.7114068	-3.6632849
H	0.2431872	20.3807174	-4.4448555
C	0.8867031	18.6206241	-3.3491976
H	1.1792251	18.1007071	-4.2735343
H	0.4320898	17.8797356	-2.6723988
C	0.2491696	14.8044484	-3.0852836
H	0.9144381	15.2116721	-3.8593917
H	0.0057748	15.6249603	-2.3904796
C	-0.9518484	16.5499589	-5.6833126
H	0.0681480	16.4646055	-5.2876609
H	-0.9654660	16.0202123	-6.6457508
C	-1.3130613	18.0356913	-5.8882571
H	-2.2092386	18.1262043	-6.5165432
H	-0.4991013	18.5464897	-6.4208411
C	-3.4796886	14.9524326	-5.3344336
H	-4.0160507	14.4153475	-4.5325824
C	-2.8504319	20.3138755	-4.7656766
H	-3.0081845	20.8338131	-3.8044591
C	-6.4571351	17.2688308	1.2663480
H	-7.2044095	16.6611477	1.7948395
H	-5.8400677	17.7556468	2.0358594
H	-5.1170047	23.6039273	-0.8049859
H	-3.4883915	23.3428573	-1.4284856
H	-2.4466665	18.7447924	4.2085264

```
C    -1.8119027   18.3580000    3.4119399
C    -2.2927738   18.2745200    2.1058292
H    -3.3108252   18.5908086    1.8678785
C    -1.4873690   17.7825492    1.0696614
C    -0.1738132   17.3723301    1.3647281
H     0.4412472   16.9839370    0.5534320
C     0.3080149   17.4485574    2.6681850
H     1.3235227   17.1235628    2.8930524
C    -0.5088298   17.9398215    3.6949003
H    -0.1274742   17.9970841    4.7137933

SCF energy GEOOPT =  -7580.864035474 H
ZPE =   3854. kJ/mol
FREEH energy =  4049.59 kJ/mol
FREEH entropy =   1.67928 kJ/mol/K

$vibrational spectrum
#  mode     symmetry     wave number   IR intensity    selection rules
#                         cm**(-1)        km/mol          IR     RAMAN
     1                      -0.00         0.00000         -        -
     2                      -0.00         0.00000         -        -
     3                      -0.00         0.00000         -        -
     4                      -0.00         0.00000         -        -
     5                       0.00         0.00000         -        -
     6                       0.00         0.00000         -        -
     7         a             7.75         0.00619        YES      YES
     8         a            13.40         0.09086        YES      YES
     9         a            18.43         0.00352        YES      YES
    10         a            20.89         0.03009        YES      YES
    11         a            24.74         0.02910        YES      YES
    12         a            26.38         0.12933        YES      YES
    13         a            27.78         0.02095        YES      YES
    14         a            30.05         0.02127        YES      YES
    15         a            33.33         0.02486        YES      YES
    16         a            35.83         0.04031        YES      YES
    17         a            38.22         0.01987        YES      YES
    18         a            40.52         0.02642        YES      YES
    19         a            42.20         0.04865        YES      YES
    20         a            45.63         0.18416        YES      YES
    21         a            46.46         0.18356        YES      YES
    22         a            48.69         0.03436        YES      YES
    23         a            52.81         0.16873        YES      YES
    24         a            55.01         0.08504        YES      YES
    25         a            55.70         0.11780        YES      YES
    26         a            57.05         0.03195        YES      YES
    27         a            59.97         0.41309        YES      YES
    28         a            63.61         0.09653        YES      YES
    29         a            65.60         0.08564        YES      YES
    30         a            65.98         0.04874        YES      YES
    31         a            70.22         0.18271        YES      YES
    32         a            71.60         0.05726        YES      YES
    33         a            75.46         0.10575        YES      YES
    34         a            77.19         0.03395        YES      YES
    35         a            81.75         0.36612        YES      YES
    36         a            86.85         0.24274        YES      YES
    37         a            92.76         0.11737        YES      YES
    38         a            96.41         0.40643        YES      YES
    39         a           113.09         3.26649        YES      YES
```

40	a	118.10	1.22642	YES	YES
41	a	125.66	0.95438	YES	YES
42	a	130.18	0.88873	YES	YES
43	a	134.53	0.87259	YES	YES
44	a	137.20	3.56828	YES	YES
45	a	140.75	7.18866	YES	YES
46	a	141.10	0.32798	YES	YES
47	a	142.60	1.00126	YES	YES
48	a	154.44	0.38677	YES	YES
49	a	159.51	0.63061	YES	YES
50	a	166.23	0.04489	YES	YES
51	a	169.89	0.24687	YES	YES
52	a	177.31	2.40164	YES	YES
53	a	183.68	0.17317	YES	YES
54	a	185.32	1.77338	YES	YES
55	a	196.07	1.40303	YES	YES
56	a	208.69	0.22794	YES	YES
57	a	208.75	0.76648	YES	YES
58	a	214.03	7.66613	YES	YES
59	a	215.74	2.39558	YES	YES
60	a	218.24	1.32669	YES	YES
61	a	218.32	1.05743	YES	YES
62	a	222.45	2.00970	YES	YES
63	a	225.08	0.63814	YES	YES
64	a	225.92	1.21017	YES	YES
65	a	227.32	2.93720	YES	YES
66	a	228.84	2.31464	YES	YES
67	a	232.37	0.39843	YES	YES
68	a	236.06	2.74666	YES	YES
69	a	238.29	1.30823	YES	YES
70	a	242.42	4.61060	YES	YES
71	a	243.08	0.77607	YES	YES
72	a	250.80	0.77389	YES	YES
73	a	258.81	1.12669	YES	YES
74	a	277.11	2.01187	YES	YES
75	a	280.36	1.42892	YES	YES
76	a	284.54	2.34507	YES	YES
77	a	293.51	0.19973	YES	YES
78	a	302.77	1.05196	YES	YES
79	a	305.85	0.84726	YES	YES
80	a	310.62	0.11076	YES	YES
81	a	316.18	0.43289	YES	YES
82	a	328.74	0.43756	YES	YES
83	a	330.21	0.74291	YES	YES
84	a	333.63	0.51183	YES	YES
85	a	337.04	0.15885	YES	YES
86	a	341.46	0.62057	YES	YES
87	a	343.70	0.71282	YES	YES
88	a	372.07	0.49329	YES	YES
89	a	374.09	3.01619	YES	YES
90	a	377.15	2.00344	YES	YES
91	a	381.94	0.73794	YES	YES
92	a	393.36	0.01771	YES	YES
93	a	395.89	1.87321	YES	YES
94	a	399.03	0.57088	YES	YES
95	a	408.91	0.02117	YES	YES
96	a	410.50	1.03481	YES	YES
97	a	411.98	2.46471	YES	YES

98	a	419.26	0.86001	YES	YES
99	a	420.70	3.25155	YES	YES
100	a	423.69	2.16842	YES	YES
101	a	427.52	0.17604	YES	YES
102	a	429.21	0.08798	YES	YES
103	a	429.92	0.13045	YES	YES
104	a	430.53	0.18695	YES	YES
105	a	431.35	0.12593	YES	YES
106	a	431.54	0.34676	YES	YES
107	a	432.54	0.31461	YES	YES
108	a	434.71	1.45114	YES	YES
109	a	437.94	0.22064	YES	YES
110	a	441.16	0.43239	YES	YES
111	a	450.59	19.80895	YES	YES
112	a	455.02	1.47501	YES	YES
113	a	461.46	6.92356	YES	YES
114	a	471.88	3.14021	YES	YES
115	a	492.45	3.37499	YES	YES
116	a	494.04	2.33118	YES	YES
117	a	495.50	7.27456	YES	YES
118	a	496.60	0.89371	YES	YES
119	a	502.24	0.79951	YES	YES
120	a	504.01	0.95649	YES	YES
121	a	512.41	7.83587	YES	YES
122	a	515.39	12.20482	YES	YES
123	a	547.53	19.43559	YES	YES
124	a	558.25	4.35939	YES	YES
125	a	615.36	1.74054	YES	YES
126	a	623.85	18.24916	YES	YES
127	a	630.36	15.45684	YES	YES
128	a	640.05	11.15209	YES	YES
129	a	654.88	13.03229	YES	YES
130	a	677.47	64.87221	YES	YES
131	a	692.90	24.67630	YES	YES
132	a	699.92	2.21139	YES	YES
133	a	718.66	1.79618	YES	YES
134	a	724.92	1.52062	YES	YES
135	a	728.13	4.18842	YES	YES
136	a	734.16	7.14905	YES	YES
137	a	736.37	5.90203	YES	YES
138	a	739.62	1.83136	YES	YES
139	a	740.25	2.00920	YES	YES
140	a	761.67	39.24727	YES	YES
141	a	771.82	0.82684	YES	YES
142	a	772.32	2.69888	YES	YES
143	a	772.86	2.16813	YES	YES
144	a	773.64	0.83576	YES	YES
145	a	773.91	0.86694	YES	YES
146	a	775.48	0.50646	YES	YES
147	a	775.90	0.60856	YES	YES
148	a	776.45	18.04967	YES	YES
149	a	776.64	3.41854	YES	YES
150	a	779.75	1.81880	YES	YES
151	a	805.90	0.29737	YES	YES
152	a	807.63	3.90388	YES	YES
153	a	809.64	4.53317	YES	YES
154	a	809.74	10.05368	YES	YES
155	a	811.25	1.35726	YES	YES

156	a	812.84	2.31984	YES	YES
157	a	815.06	1.59693	YES	YES
158	a	816.47	3.44464	YES	YES
159	a	829.84	3.94835	YES	YES
160	a	831.61	5.69926	YES	YES
161	a	836.17	2.11903	YES	YES
162	a	836.98	3.90211	YES	YES
163	a	837.56	1.55645	YES	YES
164	a	838.04	2.40473	YES	YES
165	a	839.14	0.17792	YES	YES
166	a	839.95	3.43391	YES	YES
167	a	841.16	4.35342	YES	YES
168	a	847.44	16.28606	YES	YES
169	a	851.38	33.03518	YES	YES
170	a	861.07	48.87098	YES	YES
171	a	871.60	3.60070	YES	YES
172	a	872.13	1.80952	YES	YES
173	a	873.03	3.13287	YES	YES
174	a	875.52	0.23154	YES	YES
175	a	876.56	2.72459	YES	YES
176	a	877.19	2.98020	YES	YES
177	a	878.07	3.74296	YES	YES
178	a	879.23	1.96440	YES	YES
179	a	880.04	0.98904	YES	YES
180	a	880.26	5.76146	YES	YES
181	a	880.81	4.13725	YES	YES
182	a	881.88	0.46927	YES	YES
183	a	883.23	7.47484	YES	YES
184	a	883.61	12.17288	YES	YES
185	a	886.15	1.29105	YES	YES
186	a	886.71	5.52181	YES	YES
187	a	902.35	2.02339	YES	YES
188	a	903.04	0.72675	YES	YES
189	a	904.38	0.78992	YES	YES
190	a	904.61	2.24217	YES	YES
191	a	907.06	3.07922	YES	YES
192	a	909.31	1.96699	YES	YES
193	a	910.75	1.26788	YES	YES
194	a	912.67	1.47855	YES	YES
195	a	922.15	3.84523	YES	YES
196	a	966.37	0.38273	YES	YES
197	a	976.92	0.11731	YES	YES
198	a	977.62	0.19619	YES	YES
199	a	987.16	2.58752	YES	YES
200	a	988.11	18.62243	YES	YES
201	a	988.66	11.02424	YES	YES
202	a	988.92	0.59983	YES	YES
203	a	989.68	1.26238	YES	YES
204	a	990.10	7.53218	YES	YES
205	a	990.77	2.79475	YES	YES
206	a	991.79	30.13130	YES	YES
207	a	992.91	8.16639	YES	YES
208	a	996.45	3.63655	YES	YES
209	a	1012.48	0.23897	YES	YES
210	a	1014.82	0.74251	YES	YES
211	a	1015.46	1.50738	YES	YES
212	a	1015.91	0.88440	YES	YES
213	a	1017.40	0.20708	YES	YES

214	a	1018.44	4.24246	YES	YES
215	a	1019.16	0.76220	YES	YES
216	a	1020.44	0.65390	YES	YES
217	a	1023.17	0.47302	YES	YES
218	a	1028.93	1.08971	YES	YES
219	a	1029.04	0.78920	YES	YES
220	a	1029.98	1.23402	YES	YES
221	a	1035.63	1.06116	YES	YES
222	a	1038.33	2.30037	YES	YES
223	a	1039.19	3.02584	YES	YES
224	a	1039.98	0.52029	YES	YES
225	a	1040.78	0.67748	YES	YES
226	a	1054.85	0.70151	YES	YES
227	a	1058.46	1.52020	YES	YES
228	a	1059.21	0.65266	YES	YES
229	a	1060.19	1.21661	YES	YES
230	a	1060.98	2.36292	YES	YES
231	a	1063.96	0.97734	YES	YES
232	a	1067.38	0.61967	YES	YES
233	a	1068.77	3.55444	YES	YES
234	a	1069.35	0.44096	YES	YES
235	a	1070.95	0.79285	YES	YES
236	a	1071.23	0.38378	YES	YES
237	a	1071.39	0.20614	YES	YES
238	a	1072.10	0.21593	YES	YES
239	a	1072.53	0.23286	YES	YES
240	a	1072.93	0.04083	YES	YES
241	a	1073.39	0.06669	YES	YES
242	a	1075.20	0.51386	YES	YES
243	a	1076.96	3.59787	YES	YES
244	a	1082.74	3.93471	YES	YES
245	a	1090.50	6.50011	YES	YES
246	a	1091.11	5.06570	YES	YES
247	a	1093.76	5.83315	YES	YES
248	a	1096.26	1.64644	YES	YES
249	a	1101.89	0.74791	YES	YES
250	a	1103.36	3.00626	YES	YES
251	a	1110.94	9.14587	YES	YES
252	a	1115.98	6.94657	YES	YES
253	a	1121.38	1.97734	YES	YES
254	a	1128.52	7.18736	YES	YES
255	a	1145.23	19.98258	YES	YES
256	a	1155.35	1.92773	YES	YES
257	a	1158.70	3.78938	YES	YES
258	a	1159.87	2.40481	YES	YES
259	a	1161.14	8.54352	YES	YES
260	a	1164.51	7.40356	YES	YES
261	a	1167.18	8.44408	YES	YES
262	a	1167.93	14.67776	YES	YES
263	a	1168.46	10.30549	YES	YES
264	a	1169.95	4.57379	YES	YES
265	a	1173.45	8.73594	YES	YES
266	a	1174.70	5.65400	YES	YES
267	a	1178.20	6.59271	YES	YES
268	a	1182.30	4.79712	YES	YES
269	a	1188.33	0.50008	YES	YES
270	a	1188.58	1.51232	YES	YES
271	a	1191.31	1.15508	YES	YES

272	a	1198.12	51.34478	YES	YES
273	a	1215.62	1.18459	YES	YES
274	a	1235.70	0.60771	YES	YES
275	a	1240.21	0.25872	YES	YES
276	a	1242.24	0.60219	YES	YES
277	a	1246.28	0.10469	YES	YES
278	a	1246.82	0.04362	YES	YES
279	a	1247.98	0.93438	YES	YES
280	a	1248.18	0.32633	YES	YES
281	a	1249.48	0.30301	YES	YES
282	a	1251.89	1.86251	YES	YES
283	a	1252.07	1.07059	YES	YES
284	a	1252.51	0.17172	YES	YES
285	a	1254.18	3.74640	YES	YES
286	a	1255.23	1.86181	YES	YES
287	a	1255.69	3.88035	YES	YES
288	a	1255.86	2.88307	YES	YES
289	a	1256.09	1.17923	YES	YES
290	a	1257.64	4.63260	YES	YES
291	a	1257.74	1.04150	YES	YES
292	a	1258.84	0.61862	YES	YES
293	a	1259.18	2.52084	YES	YES
294	a	1260.11	0.58726	YES	YES
295	a	1261.12	2.92843	YES	YES
296	a	1261.63	2.49624	YES	YES
297	a	1264.23	0.58929	YES	YES
298	a	1266.09	2.16610	YES	YES
299	a	1270.16	1.01967	YES	YES
300	a	1272.84	0.78097	YES	YES
301	a	1274.93	1.18489	YES	YES
302	a	1280.24	2.27100	YES	YES
303	a	1281.87	0.55278	YES	YES
304	a	1283.00	4.71648	YES	YES
305	a	1284.11	0.86511	YES	YES
306	a	1286.63	9.47215	YES	YES
307	a	1287.91	11.03541	YES	YES
308	a	1288.40	3.12807	YES	YES
309	a	1289.11	3.36356	YES	YES
310	a	1293.52	3.25697	YES	YES
311	a	1305.48	0.45301	YES	YES
312	a	1308.51	0.99636	YES	YES
313	a	1310.20	1.02961	YES	YES
314	a	1310.70	2.57067	YES	YES
315	a	1311.43	0.43827	YES	YES
316	a	1312.65	1.72763	YES	YES
317	a	1319.79	4.25458	YES	YES
318	a	1320.83	0.42689	YES	YES
319	a	1321.16	0.80493	YES	YES
320	a	1321.62	1.19298	YES	YES
321	a	1321.79	0.55191	YES	YES
322	a	1322.19	0.31810	YES	YES
323	a	1323.21	1.77555	YES	YES
324	a	1323.68	0.22227	YES	YES
325	a	1324.16	1.69058	YES	YES
326	a	1324.30	1.05858	YES	YES
327	a	1324.52	2.25509	YES	YES
328	a	1325.67	5.43485	YES	YES
329	a	1326.08	1.98298	YES	YES

330	a	1326.64	0.85297	YES	YES
331	a	1327.48	7.67735	YES	YES
332	a	1329.93	2.54177	YES	YES
333	a	1335.99	0.27131	YES	YES
334	a	1336.16	0.05289	YES	YES
335	a	1337.22	0.28531	YES	YES
336	a	1337.43	0.80618	YES	YES
337	a	1337.82	0.05870	YES	YES
338	a	1338.80	0.14625	YES	YES
339	a	1340.18	0.21180	YES	YES
340	a	1340.31	0.28693	YES	YES
341	a	1340.64	0.54078	YES	YES
342	a	1343.09	0.46939	YES	YES
343	a	1343.85	0.61881	YES	YES
344	a	1345.16	0.72569	YES	YES
345	a	1345.59	1.74650	YES	YES
346	a	1346.12	1.36931	YES	YES
347	a	1346.46	0.19674	YES	YES
348	a	1346.95	0.59747	YES	YES
349	a	1347.41	2.04985	YES	YES
350	a	1347.81	0.39519	YES	YES
351	a	1350.96	0.98199	YES	YES
352	a	1399.11	8.17730	YES	YES
353	a	1404.82	5.09856	YES	YES
354	a	1405.04	8.49429	YES	YES
355	a	1406.79	8.80585	YES	YES
356	a	1422.43	4.94901	YES	YES
357	a	1426.42	5.74802	YES	YES
358	a	1428.24	1.69749	YES	YES
359	a	1429.99	1.95426	YES	YES
360	a	1430.92	1.84559	YES	YES
361	a	1431.54	1.66806	YES	YES
362	a	1432.48	2.34334	YES	YES
363	a	1433.40	5.02661	YES	YES
364	a	1434.12	1.35605	YES	YES
365	a	1435.49	13.23432	YES	YES
366	a	1435.54	2.18751	YES	YES
367	a	1436.26	7.09974	YES	YES
368	a	1436.75	6.44417	YES	YES
369	a	1437.12	12.14075	YES	YES
370	a	1437.56	1.59732	YES	YES
371	a	1437.81	18.93916	YES	YES
372	a	1438.29	13.12858	YES	YES
373	a	1438.39	5.85883	YES	YES
374	a	1438.64	1.62179	YES	YES
375	a	1438.95	4.10754	YES	YES
376	a	1439.20	1.49616	YES	YES
377	a	1439.67	1.10215	YES	YES
378	a	1440.07	9.53426	YES	YES
379	a	1440.14	0.28935	YES	YES
380	a	1441.73	7.61241	YES	YES
381	a	1442.59	13.09108	YES	YES
382	a	1443.14	7.60383	YES	YES
383	a	1444.26	19.14151	YES	YES
384	a	1444.41	24.28871	YES	YES
385	a	1444.83	10.01835	YES	YES
386	a	1445.31	3.00283	YES	YES
387	a	1445.52	1.89464	YES	YES

388	a	1447.16	7.01395	YES	YES
389	a	1452.64	2.99373	YES	YES
390	a	1453.71	0.89146	YES	YES
391	a	1454.28	1.30738	YES	YES
392	a	1454.39	1.58652	YES	YES
393	a	1455.31	0.65185	YES	YES
394	a	1455.50	1.69826	YES	YES
395	a	1456.48	2.28440	YES	YES
396	a	1457.97	2.89634	YES	YES
397	a	1470.15	6.58998	YES	YES
398	a	1532.69	204.55797	YES	YES
399	a	1570.09	3.29367	YES	YES
400	a	1591.40	3.64772	YES	YES
401	a	2915.15	17.81610	YES	YES
402	a	2923.20	6.97812	YES	YES
403	a	2925.67	3.90415	YES	YES
404	a	2930.20	21.08774	YES	YES
405	a	2935.37	16.19873	YES	YES
406	a	2936.72	29.31586	YES	YES
407	a	2936.86	9.70919	YES	YES
408	a	2937.19	4.51753	YES	YES
409	a	2937.79	11.68794	YES	YES
410	a	2939.64	4.61418	YES	YES
411	a	2941.15	29.07998	YES	YES
412	a	2941.86	23.20970	YES	YES
413	a	2944.14	5.42600	YES	YES
414	a	2944.33	14.85713	YES	YES
415	a	2944.83	6.18690	YES	YES
416	a	2944.98	0.84031	YES	YES
417	a	2945.03	6.89373	YES	YES
418	a	2945.10	20.13895	YES	YES
419	a	2945.53	8.96280	YES	YES
420	a	2946.00	10.46734	YES	YES
421	a	2947.03	9.18594	YES	YES
422	a	2947.84	3.31432	YES	YES
423	a	2948.11	11.99320	YES	YES
424	a	2948.42	10.51576	YES	YES
425	a	2948.62	36.92738	YES	YES
426	a	2949.52	6.15745	YES	YES
427	a	2950.36	15.91153	YES	YES
428	a	2953.61	2.38302	YES	YES
429	a	2954.32	5.16578	YES	YES
430	a	2955.42	4.11694	YES	YES
431	a	2955.58	3.23634	YES	YES
432	a	2955.60	8.52831	YES	YES
433	a	2956.82	9.27851	YES	YES
434	a	2957.03	6.80251	YES	YES
435	a	2957.28	10.49631	YES	YES
436	a	2957.51	10.83631	YES	YES
437	a	2958.38	10.86317	YES	YES
438	a	2958.53	7.62423	YES	YES
439	a	2959.83	38.61263	YES	YES
440	a	2960.03	18.37519	YES	YES
441	a	2960.49	26.33113	YES	YES
442	a	2960.75	27.46290	YES	YES
443	a	2961.18	50.48096	YES	YES
444	a	2961.87	31.80652	YES	YES
445	a	2963.18	21.23101	YES	YES

446	a	2965.50	2.44056	YES	YES
447	a	2965.71	18.79355	YES	YES
448	a	2975.29	9.32380	YES	YES
449	a	2977.63	6.62752	YES	YES
450	a	2982.22	28.07900	YES	YES
451	a	2983.09	9.00082	YES	YES
452	a	2985.42	3.05829	YES	YES
453	a	2985.68	39.01598	YES	YES
454	a	2986.90	7.34201	YES	YES
455	a	2987.15	48.27328	YES	YES
456	a	2990.36	20.99202	YES	YES
457	a	2990.43	11.70657	YES	YES
458	a	2998.04	11.44414	YES	YES
459	a	2998.14	38.78326	YES	YES
460	a	2998.51	21.36786	YES	YES
461	a	2998.83	18.03067	YES	YES
462	a	3000.27	33.78475	YES	YES
463	a	3001.63	11.02408	YES	YES
464	a	3002.79	11.32667	YES	YES
465	a	3002.87	37.96063	YES	YES
466	a	3005.37	17.20145	YES	YES
467	a	3005.47	25.66549	YES	YES
468	a	3007.29	15.86372	YES	YES
469	a	3008.11	16.60747	YES	YES
470	a	3009.24	7.97941	YES	YES
471	a	3009.35	18.07402	YES	YES
472	a	3009.41	28.93645	YES	YES
473	a	3009.90	21.95604	YES	YES
474	a	3010.55	17.71222	YES	YES
475	a	3011.26	15.60025	YES	YES
476	a	3011.86	38.46661	YES	YES
477	a	3012.60	21.81239	YES	YES
478	a	3012.98	21.10047	YES	YES
479	a	3015.10	17.96490	YES	YES
480	a	3015.62	29.33294	YES	YES
481	a	3015.73	17.19967	YES	YES
482	a	3015.88	20.00029	YES	YES
483	a	3016.22	28.05243	YES	YES
484	a	3016.78	27.48328	YES	YES
485	a	3016.92	27.87592	YES	YES
486	a	3017.41	32.28939	YES	YES
487	a	3017.77	16.72226	YES	YES
488	a	3018.34	4.66958	YES	YES
489	a	3018.45	39.49187	YES	YES
490	a	3018.85	24.58698	YES	YES
491	a	3019.57	32.30783	YES	YES
492	a	3021.48	34.99377	YES	YES
493	a	3024.25	4.36706	YES	YES
494	a	3029.77	0.56138	YES	YES
495	a	3036.09	1.26717	YES	YES
496	a	3044.41	0.21472	YES	YES
497	a	3072.26	8.36753	YES	YES
498	a	3108.45	0.43096	YES	YES
499	a	3115.43	0.87896	YES	YES
500	a	3122.79	7.13447	YES	YES
501	a	3132.02	7.28845	YES	YES

$end

Double hybrid single point energy = -7573.145370452997 H
COSMO energy + OC correction = -7581.0105805499 H (in *o*DFB)

6.2.45 H_2FCCN

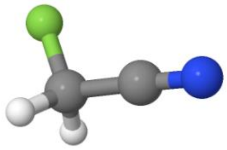

Method: (RI-)BP86(D3BJ)/def2-TZVPP
Symmetry: cs

Cartesian coordinates in Ångström:

N	-0.0600457	-2.1499227	0.0000000
C	-0.0391259	-0.9886156	0.0000000
C	-0.0414465	0.4763755	0.0000000
F	1.2600929	0.9710033	0.0000000
H	-0.5597374	0.8455798	0.8976042
H	-0.5597374	0.8455798	-0.8976042

SCF energy GEOOPT = -232.0867825213 H
ZPE = 97.52 kJ/mol
FREEH energy = 108.25 kJ/mol
FREEH entropy = 0.27596 kJ/mol/K

$vibrational spectrum

# mode	symmetry	wave number cm**(-1)	IR intensity km/mol	selection rules IR	RAMAN
1		-0.00	0.00000	-	-
2		-0.00	0.00000	-	-
3		0.00	0.00000	-	-
4		0.00	0.00000	-	-
5		0.00	0.00000	-	-
6		0.00	0.00000	-	-
7	a'	214.83	8.08432	YES	YES
8	a"	339.26	0.57497	YES	YES
9	a'	556.41	1.34017	YES	YES
10	a'	906.66	15.43937	YES	YES
11	a"	994.26	1.31910	YES	YES
12	a'	1025.25	122.97639	YES	YES
13	a"	1219.37	0.41011	YES	YES
14	a'	1349.71	14.43360	YES	YES
15	a'	1432.49	5.21986	YES	YES
16	a'	2274.65	1.60430	YES	YES
17	a'	2973.94	11.90277	YES	YES
18	a"	3017.98	3.26866	YES	YES

$end

Double hybrid single point energy = -231.741278146058 H
COSMO energy + OC correction = -232.0965627414 H (in *o*DFB)

6.2.46 [{Ga(dcpe)}$_2$(NCCH$_2$F)]$^{2+}$ (Cycloadduct)

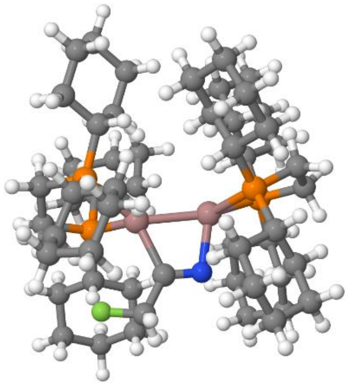

```
Method: (RI-)BP86(D3BJ)/def2-TZVPP
Symmetry: c1

Cartesian coordinates in Ångström:
Ga   -2.5521115   17.3150639   -2.7691630
Ga   -4.1244429   17.7255718   -0.9651381
P    -1.6899779   18.9662100   -4.2961851
P    -6.0515547   19.1329047   -0.8218948
P    -1.9392492   15.6643324   -4.3785231
P    -5.3920394   16.1375517    0.2958301
C    -5.2072895   20.9049292   -5.4647284
H    -5.4055227   21.4494370   -4.5249071
H    -6.1708335   20.4954148   -5.7988055
C    -0.9453558   14.3064476   -3.6384378
H    -0.6297108   13.6263169   -4.4471077
C    -4.3468883   15.2824605    1.5424883
H    -3.7787398   16.1335862    1.9595940
C    -4.3327670   16.0614038   -5.7494171
H    -3.8027927   16.6237861   -6.5351575
H    -4.5716597   16.7777131   -4.9471034
C    -6.9288296   19.2829998   -2.4335375
H    -6.2229441   19.8479491   -3.0648970
C    -7.2161051   18.3010023    0.3573281
H    -7.7160832   19.0554387    0.9796839
H    -7.9979633   17.8321546   -0.2555491
C    -3.2822665   22.4232009   -6.0798323
H    -2.8746457   23.0892368   -6.8518732
H    -3.3942248   23.0325921   -5.1677896
C    -2.2879521   21.2875371   -5.8029116
H    -1.3288055   21.7019980   -5.4636151
H    -2.0876093   20.7418900   -6.7393899
C    -4.2261436   19.7603809   -5.1840048
H    -4.1169151   19.1541154   -6.0974074
H    -4.6162681   19.0874773   -4.4039716
C     1.0574698   13.7897940   -2.1711494
H     1.4593598   13.0516764   -2.8840142
H     1.9216663   14.2386110   -1.6631627
C    -7.1927268   13.9138446    0.0227347
H    -7.7547316   14.3253598    0.8729594
H    -6.4621061   13.2024899    0.4361648
C    -5.6053626   20.8116119   -0.2258776
H    -6.5058003   21.4465945   -0.2649090
C    -0.4780132   20.6398923   -2.4623089
H    -1.1796566   21.4381652   -2.7441289
```

H	-0.9776951	20.0168098	-1.7033495
C	0.7926415	21.2518050	-1.8596954
H	1.2324964	21.9597824	-2.5807779
H	0.5260671	21.8389321	-0.9697819
C	-2.1818545	17.8148707	-0.2425798
C	-7.1272940	17.8968668	-3.0706691
H	-6.1613411	17.3713772	-3.1409478
H	-7.7759115	17.2864782	-2.4206115
C	-4.6500420	21.8818138	-6.5045916
H	-4.5546925	21.3640239	-7.4731406
H	-5.3547161	22.7090297	-6.6626728
C	-8.2490142	20.0662779	-2.3521487
H	-8.0783396	21.0691698	-1.9366004
H	-8.9407946	19.5503094	-1.6663527
C	-4.5327234	21.3798405	-1.1783160
H	-4.9388047	21.4819325	-2.1958286
H	-3.6984685	20.6565568	-1.2383092
C	-3.0880275	13.9075417	-6.2889627
H	-2.4645681	13.1033497	-5.8736582
H	-2.5034005	14.3818335	-7.0940949
C	2.1677109	19.3328767	-2.7432915
H	2.6554636	19.9701422	-3.4988844
H	2.8883255	18.5450544	-2.4847667
C	-4.5515840	22.0920974	1.6938688
H	-5.3781772	22.8189827	1.7442770
H	-4.1602365	21.9911764	2.7149734
C	-9.1024032	18.7934395	-4.3749427
H	-9.5375764	18.8988159	-5.3777049
H	-9.8323149	18.2215922	-3.7783285
C	-5.0835457	14.5962479	2.7058025
H	-5.7766700	15.2955206	3.1938986
H	-5.6851167	13.7577189	2.3285664
C	0.1452740	13.0862700	-1.1635370
H	0.6987038	12.3039417	-0.6272820
H	-0.1868539	13.8169676	-0.4070608
C	-7.7878574	18.0098199	-4.4486186
H	-7.0933198	18.5152058	-5.1390134
H	-7.9592758	17.0044279	-4.8573680
C	1.8217082	20.1731734	-1.5111262
H	1.4117310	19.5141519	-0.7278837
H	2.7285322	20.6336957	-1.0973239
C	-3.4671553	22.6191345	0.7497085
H	-3.1037862	23.5959115	1.0951684
H	-2.6010082	21.9356635	0.7700895
C	-5.6576368	14.4539489	-1.9275085
H	-4.8748302	13.7735428	-1.5583642
H	-5.1392701	15.2683922	-2.4572353
C	-5.0916420	20.7361162	1.2231086
H	-4.2872619	19.9848502	1.2851653
H	-5.8948076	20.3980884	1.8940250
C	-6.4431735	15.0233604	-0.7314516
H	-7.1958993	15.7215870	-1.1416438
C	-3.9935507	22.7262985	-0.6839392
C	-7.3749611	12.6005912	-2.1359941
H	-8.0708881	12.1022932	-2.8239984
H	-6.6717820	11.8261491	-1.7878470
C	-8.1348913	13.1696161	-0.9334393
H	-8.6511011	12.3664697	-0.3907635

H	-8.9170158	13.8630972	-1.2846662
C	-1.8529013	13.5461105	-2.6498747
H	-2.6941377	13.0785263	-3.1809818
H	-2.2864708	14.2750443	-1.9432910
C	-4.0631535	14.0697728	3.7255568
H	-3.5316389	14.9227942	4.1779519
H	-4.5922864	13.5607880	4.5424161
C	-5.6153087	15.4694568	-6.3439998
H	-6.2361520	16.2754735	-6.7589260
H	-6.2007279	15.0029666	-5.5352578
C	-3.0514436	13.1245957	3.0695152
H	-3.5745553	12.2232503	2.7089741
H	-2.3172178	12.7818315	3.8106420
C	-3.3382511	14.3387347	0.8646355
H	-3.8760415	13.4951779	0.4030537
H	-2.8090224	14.8679216	0.0571814
C	-8.8984431	20.1723670	-3.7394228
H	-9.8564358	20.7026329	-3.6560407
H	-8.2555792	20.7873608	-4.3896928
C	-4.3804274	13.3248765	-6.8787117
H	-4.1317402	12.6062840	-7.6709145
H	-4.9069812	12.7558012	-6.0945997
C	-6.5949084	13.6950450	-2.8722518
H	-6.0170991	13.2608885	-3.7016721
H	-7.3025888	14.4106104	-3.3245801
C	-5.3006298	14.4238831	-7.4190433
H	-6.2308878	13.9843377	-7.8028242
H	-4.8139754	14.9182069	-8.2760276
C	-1.0756996	12.4849871	-1.8631205
H	-1.7494908	12.0100845	-1.1356169
H	-0.7498898	11.6887645	-2.5513982
C	-2.3376971	13.7995132	1.8937253
H	-1.7237058	14.6357980	2.2664796
H	-1.6480315	13.0970918	1.4056818
C	-1.7195026	18.1866991	1.1378931
N	-1.3800345	17.4207620	-1.1545487
C	-0.1468227	19.7585613	-3.6835339
H	0.2326058	20.3969685	-4.4999051
C	0.9113038	18.6915352	-3.3448399
H	1.1797696	18.1243051	-4.2482323
H	0.4842634	17.9856663	-2.6149489
C	0.2977367	14.8828266	-2.9318661
H	0.9664416	15.3609394	-3.6613671
H	-0.0177315	15.6637392	-2.2196722
C	-0.9081743	16.5552567	-5.6280434
H	0.1206706	16.4935748	-5.2508657
H	-0.9306114	16.0105409	-6.5819976
C	-1.3026506	18.0305352	-5.8489683
H	-2.2045118	18.0935211	-6.4721736
H	-0.5034624	18.5514718	-6.3940024
C	-3.4159590	14.9503143	-5.2101340
H	-3.9526734	14.4473473	-4.3870215
C	-2.8576362	20.3136281	-4.7580126
H	-3.0007943	20.8589886	-3.8088166
C	-6.5251052	17.2481491	1.2441100
H	-7.2757649	16.6569145	1.7861609
H	-5.8894280	17.7351954	1.9977018
H	-4.7969751	23.4789130	-0.7265725

```
H    -3.2030723   23.0747403   -1.3640531
H    -1.0042421   17.4528352    1.5353313
F    -2.8180683   18.2429620    2.0274046
H    -1.2538923   19.1842786    1.1337748

SCF energy GEOOPT =  -7488.302837555 H
ZPE =   3699. kJ/mol
FREEH energy =  3887.69 kJ/mol
FREEH entropy =   1.62124 kJ/mol/K

$vibrational spectrum
#  mode     symmetry     wave number   IR intensity    selection rules
#                         cm**(-1)        km/mol          IR     RAMAN
     1                      -0.00         0.00000         -        -
     2                       0.00         0.00000         -        -
     3                       0.00         0.00000         -        -
     4                       0.00         0.00000         -        -
     5                       0.00         0.00000         -        -
     6                       0.00         0.00000         -        -
     7        a             11.04         0.04657        YES      YES
     8        a             19.01         0.02054        YES      YES
     9        a             21.18         0.07094        YES      YES
    10        a             25.81         0.07839        YES      YES
    11        a             27.57         0.00798        YES      YES
    12        a             29.75         0.10162        YES      YES
    13        a             33.03         0.09048        YES      YES
    14        a             34.30         0.06264        YES      YES
    15        a             36.10         0.00486        YES      YES
    16        a             36.84         0.01987        YES      YES
    17        a             38.90         0.08083        YES      YES
    18        a             44.48         0.02804        YES      YES
    19        a             44.57         0.11666        YES      YES
    20        a             47.61         0.14258        YES      YES
    21        a             50.20         0.17083        YES      YES
    22        a             51.17         0.09446        YES      YES
    23        a             54.56         0.45560        YES      YES
    24        a             56.04         0.06377        YES      YES
    25        a             60.56         0.18380        YES      YES
    26        a             62.42         0.02893        YES      YES
    27        a             62.60         0.25701        YES      YES
    28        a             64.54         0.13095        YES      YES
    29        a             69.65         0.02303        YES      YES
    30        a             73.41         0.14117        YES      YES
    31        a             74.29         0.07196        YES      YES
    32        a             75.97         0.15662        YES      YES
    33        a             79.22         0.87701        YES      YES
    34        a             85.09         1.05198        YES      YES
    35        a             87.43         1.27958        YES      YES
    36        a             89.35         2.62818        YES      YES
    37        a             94.37         1.75938        YES      YES
    38        a            100.08         0.40083        YES      YES
    39        a            116.63         0.89660        YES      YES
    40        a            121.08         2.38122        YES      YES
    41        a            128.45         0.81147        YES      YES
    42        a            133.62         0.12681        YES      YES
    43        a            136.35         1.10313        YES      YES
    44        a            138.74         0.63274        YES      YES
    45        a            141.02         1.08176        YES      YES
```

46	a	145.88	0.81124	YES	YES
47	a	151.58	0.74003	YES	YES
48	a	157.22	1.04059	YES	YES
49	a	169.05	0.10018	YES	YES
50	a	174.62	1.05325	YES	YES
51	a	179.81	4.87393	YES	YES
52	a	181.78	0.50883	YES	YES
53	a	188.97	2.03496	YES	YES
54	a	195.69	2.04836	YES	YES
55	a	203.25	16.50382	YES	YES
56	a	210.68	0.21181	YES	YES
57	a	210.99	0.74236	YES	YES
58	a	216.31	0.35232	YES	YES
59	a	218.16	0.72211	YES	YES
60	a	220.03	0.90581	YES	YES
61	a	222.29	0.89488	YES	YES
62	a	225.37	0.14558	YES	YES
63	a	226.23	0.97301	YES	YES
64	a	228.27	0.54445	YES	YES
65	a	232.07	0.46106	YES	YES
66	a	235.62	3.36270	YES	YES
67	a	239.41	11.44549	YES	YES
68	a	240.51	3.79904	YES	YES
69	a	243.80	1.47100	YES	YES
70	a	246.77	1.80963	YES	YES
71	a	255.22	3.68381	YES	YES
72	a	276.33	2.15434	YES	YES
73	a	280.83	0.45677	YES	YES
74	a	286.45	4.26987	YES	YES
75	a	293.30	0.53426	YES	YES
76	a	300.67	0.13113	YES	YES
77	a	304.99	0.73241	YES	YES
78	a	311.52	0.07235	YES	YES
79	a	313.97	0.20627	YES	YES
80	a	328.89	0.56967	YES	YES
81	a	329.89	0.78250	YES	YES
82	a	333.64	0.47187	YES	YES
83	a	336.73	0.24057	YES	YES
84	a	341.34	0.50041	YES	YES
85	a	343.92	1.11945	YES	YES
86	a	372.64	1.73448	YES	YES
87	a	373.54	1.22898	YES	YES
88	a	377.10	2.51088	YES	YES
89	a	380.81	1.64066	YES	YES
90	a	390.47	3.24006	YES	YES
91	a	394.11	0.15230	YES	YES
92	a	395.50	1.09697	YES	YES
93	a	407.70	0.03172	YES	YES
94	a	410.29	2.22757	YES	YES
95	a	413.18	0.89031	YES	YES
96	a	417.56	2.61846	YES	YES
97	a	421.61	4.49483	YES	YES
98	a	423.47	1.55824	YES	YES
99	a	427.39	0.32617	YES	YES
100	a	429.24	0.06926	YES	YES
101	a	429.90	0.18830	YES	YES
102	a	430.40	0.11883	YES	YES
103	a	430.88	0.13885	YES	YES

104	a	432.09	0.55920	YES	YES
105	a	432.40	0.49226	YES	YES
106	a	435.28	1.07877	YES	YES
107	a	438.33	0.15359	YES	YES
108	a	445.29	2.09526	YES	YES
109	a	456.44	2.77022	YES	YES
110	a	461.96	5.26678	YES	YES
111	a	471.11	3.48836	YES	YES
112	a	491.54	3.45433	YES	YES
113	a	494.36	1.03947	YES	YES
114	a	495.09	4.63276	YES	YES
115	a	497.01	1.28770	YES	YES
116	a	502.87	2.30888	YES	YES
117	a	503.93	0.34942	YES	YES
118	a	513.41	7.67307	YES	YES
119	a	514.92	17.64458	YES	YES
120	a	520.99	20.61261	YES	YES
121	a	606.87	40.11725	YES	YES
122	a	625.32	14.54826	YES	YES
123	a	632.07	18.73891	YES	YES
124	a	640.32	10.67507	YES	YES
125	a	656.48	12.27509	YES	YES
126	a	700.00	2.03304	YES	YES
127	a	718.27	1.79974	YES	YES
128	a	725.55	1.42758	YES	YES
129	a	729.43	4.01786	YES	YES
130	a	732.95	8.52051	YES	YES
131	a	737.29	2.01283	YES	YES
132	a	739.91	2.98774	YES	YES
133	a	742.14	3.18440	YES	YES
134	a	769.67	1.34280	YES	YES
135	a	771.69	0.36136	YES	YES
136	a	772.63	0.28845	YES	YES
137	a	773.51	1.92108	YES	YES
138	a	774.24	0.21068	YES	YES
139	a	775.67	6.35646	YES	YES
140	a	775.83	0.76139	YES	YES
141	a	776.76	6.33322	YES	YES
142	a	776.94	1.34446	YES	YES
143	a	779.28	13.05515	YES	YES
144	a	806.14	0.28681	YES	YES
145	a	807.93	4.65098	YES	YES
146	a	809.59	9.03000	YES	YES
147	a	810.64	3.86132	YES	YES
148	a	811.67	0.55682	YES	YES
149	a	813.12	2.90886	YES	YES
150	a	815.09	1.71304	YES	YES
151	a	816.48	4.27359	YES	YES
152	a	830.41	4.61857	YES	YES
153	a	831.18	5.58577	YES	YES
154	a	836.16	0.71900	YES	YES
155	a	837.94	2.38294	YES	YES
156	a	838.13	2.17121	YES	YES
157	a	838.46	0.15102	YES	YES
158	a	839.54	9.64054	YES	YES
159	a	839.77	3.99700	YES	YES
160	a	847.22	14.66472	YES	YES
161	a	853.52	12.94217	YES	YES

162	a	870.02	1.35649	YES	YES
163	a	870.27	4.51746	YES	YES
164	a	872.36	3.09281	YES	YES
165	a	876.32	0.80112	YES	YES
166	a	877.09	0.67772	YES	YES
167	a	877.81	2.85575	YES	YES
168	a	878.13	3.78912	YES	YES
169	a	878.98	0.61217	YES	YES
170	a	879.66	3.58359	YES	YES
171	a	880.54	0.25346	YES	YES
172	a	880.70	4.87685	YES	YES
173	a	881.26	0.60219	YES	YES
174	a	882.90	3.66972	YES	YES
175	a	883.39	13.16561	YES	YES
176	a	884.62	5.36428	YES	YES
177	a	886.26	0.50193	YES	YES
178	a	902.26	3.14353	YES	YES
179	a	902.90	0.80348	YES	YES
180	a	904.16	3.77421	YES	YES
181	a	905.08	1.00476	YES	YES
182	a	907.55	3.11901	YES	YES
183	a	908.92	0.73722	YES	YES
184	a	910.26	2.01023	YES	YES
185	a	910.88	0.57899	YES	YES
186	a	913.93	8.75965	YES	YES
187	a	964.91	39.70716	YES	YES
188	a	976.66	0.12321	YES	YES
189	a	976.81	1.05981	YES	YES
190	a	987.20	7.39540	YES	YES
191	a	987.68	10.31316	YES	YES
192	a	988.31	3.17486	YES	YES
193	a	988.89	14.25355	YES	YES
194	a	989.03	5.35307	YES	YES
195	a	990.12	8.98375	YES	YES
196	a	991.79	28.19100	YES	YES
197	a	992.08	5.54762	YES	YES
198	a	1012.99	0.31585	YES	YES
199	a	1014.84	2.74412	YES	YES
200	a	1015.25	0.59961	YES	YES
201	a	1016.33	0.79062	YES	YES
202	a	1017.22	0.37342	YES	YES
203	a	1019.75	0.95742	YES	YES
204	a	1020.20	0.71403	YES	YES
205	a	1022.21	0.40005	YES	YES
206	a	1028.28	0.88263	YES	YES
207	a	1028.41	1.88952	YES	YES
208	a	1030.00	2.10841	YES	YES
209	a	1034.85	14.99987	YES	YES
210	a	1037.17	8.17843	YES	YES
211	a	1039.67	6.83955	YES	YES
212	a	1040.00	30.95353	YES	YES
213	a	1040.18	2.23442	YES	YES
214	a	1040.94	23.80902	YES	YES
215	a	1055.49	0.83361	YES	YES
216	a	1058.36	1.05195	YES	YES
217	a	1059.70	0.30245	YES	YES
218	a	1060.50	1.31524	YES	YES
219	a	1062.36	1.87588	YES	YES

220	a	1065.65	1.09873	YES	YES
221	a	1066.32	0.86192	YES	YES
222	a	1069.54	0.93002	YES	YES
223	a	1070.44	0.00074	YES	YES
224	a	1071.82	0.08711	YES	YES
225	a	1071.86	0.13581	YES	YES
226	a	1072.07	0.26512	YES	YES
227	a	1072.58	0.10590	YES	YES
228	a	1073.05	0.25646	YES	YES
229	a	1073.24	0.12428	YES	YES
230	a	1073.65	0.06797	YES	YES
231	a	1078.50	3.27039	YES	YES
232	a	1081.76	3.86986	YES	YES
233	a	1087.90	7.25058	YES	YES
234	a	1090.96	4.50832	YES	YES
235	a	1094.50	4.63006	YES	YES
236	a	1096.04	0.72434	YES	YES
237	a	1102.40	0.70843	YES	YES
238	a	1103.18	3.51713	YES	YES
239	a	1111.09	8.96747	YES	YES
240	a	1116.19	10.23880	YES	YES
241	a	1122.01	0.99429	YES	YES
242	a	1129.77	3.41844	YES	YES
243	a	1158.56	4.48129	YES	YES
244	a	1159.55	2.37934	YES	YES
245	a	1160.97	7.73403	YES	YES
246	a	1165.60	5.77571	YES	YES
247	a	1166.41	9.46561	YES	YES
248	a	1167.41	10.00385	YES	YES
249	a	1168.75	12.88994	YES	YES
250	a	1170.18	3.55211	YES	YES
251	a	1172.86	9.01239	YES	YES
252	a	1174.49	9.66305	YES	YES
253	a	1178.87	7.73827	YES	YES
254	a	1181.41	5.00557	YES	YES
255	a	1185.88	0.13053	YES	YES
256	a	1187.20	0.42703	YES	YES
257	a	1189.08	1.80617	YES	YES
258	a	1192.16	0.84020	YES	YES
259	a	1215.37	2.17140	YES	YES
260	a	1237.00	0.67301	YES	YES
261	a	1240.94	0.34187	YES	YES
262	a	1241.84	0.82418	YES	YES
263	a	1245.70	0.20176	YES	YES
264	a	1246.41	0.06121	YES	YES
265	a	1246.77	0.46986	YES	YES
266	a	1248.94	0.86805	YES	YES
267	a	1249.21	1.37881	YES	YES
268	a	1252.15	1.21377	YES	YES
269	a	1252.85	0.86159	YES	YES
270	a	1253.80	0.20390	YES	YES
271	a	1254.52	3.38976	YES	YES
272	a	1255.14	2.65831	YES	YES
273	a	1255.46	4.49090	YES	YES
274	a	1256.10	0.76303	YES	YES
275	a	1256.44	2.55854	YES	YES
276	a	1257.06	4.03779	YES	YES
277	a	1257.55	1.01668	YES	YES

278	a	1258.72	1.50209	YES	YES
279	a	1259.40	1.60586	YES	YES
280	a	1260.66	2.41872	YES	YES
281	a	1261.34	3.16593	YES	YES
282	a	1262.71	1.73945	YES	YES
283	a	1263.51	0.70717	YES	YES
284	a	1265.42	1.02140	YES	YES
285	a	1269.22	1.25366	YES	YES
286	a	1270.91	2.14242	YES	YES
287	a	1272.13	1.77136	YES	YES
288	a	1280.84	2.42288	YES	YES
289	a	1281.18	0.89839	YES	YES
290	a	1283.23	4.43186	YES	YES
291	a	1284.96	1.03343	YES	YES
292	a	1286.55	5.57863	YES	YES
293	a	1288.18	3.77221	YES	YES
294	a	1288.66	3.28858	YES	YES
295	a	1291.73	3.27273	YES	YES
296	a	1299.35	6.24905	YES	YES
297	a	1305.85	0.75811	YES	YES
298	a	1309.69	1.21552	YES	YES
299	a	1311.43	0.85361	YES	YES
300	a	1312.48	2.15842	YES	YES
301	a	1314.79	0.56317	YES	YES
302	a	1316.19	2.31929	YES	YES
303	a	1317.87	0.55244	YES	YES
304	a	1319.77	2.66425	YES	YES
305	a	1320.53	1.34236	YES	YES
306	a	1321.12	0.55601	YES	YES
307	a	1321.53	0.04636	YES	YES
308	a	1321.82	0.82882	YES	YES
309	a	1323.56	1.21777	YES	YES
310	a	1324.04	1.62471	YES	YES
311	a	1324.24	0.97246	YES	YES
312	a	1324.42	1.36407	YES	YES
313	a	1325.18	1.78126	YES	YES
314	a	1325.44	4.13329	YES	YES
315	a	1325.84	2.13479	YES	YES
316	a	1327.23	6.40042	YES	YES
317	a	1330.01	2.30616	YES	YES
318	a	1332.29	1.12655	YES	YES
319	a	1336.17	0.17266	YES	YES
320	a	1336.41	0.26667	YES	YES
321	a	1336.63	0.16672	YES	YES
322	a	1337.14	0.39307	YES	YES
323	a	1337.42	1.00644	YES	YES
324	a	1338.17	0.10700	YES	YES
325	a	1338.34	0.24778	YES	YES
326	a	1338.76	0.42202	YES	YES
327	a	1339.94	0.18980	YES	YES
328	a	1341.43	1.11965	YES	YES
329	a	1343.63	0.13894	YES	YES
330	a	1344.59	2.14134	YES	YES
331	a	1344.89	0.61633	YES	YES
332	a	1345.36	0.41884	YES	YES
333	a	1346.59	2.90252	YES	YES
334	a	1347.03	0.67953	YES	YES
335	a	1347.35	0.87868	YES	YES

336	a	1349.86	0.96861	YES	YES
337	a	1400.70	6.63602	YES	YES
338	a	1403.85	8.87075	YES	YES
339	a	1404.60	8.01749	YES	YES
340	a	1407.30	9.71820	YES	YES
341	a	1412.67	10.04530	YES	YES
342	a	1423.67	5.30208	YES	YES
343	a	1427.05	4.44249	YES	YES
344	a	1428.35	1.62968	YES	YES
345	a	1430.33	2.07207	YES	YES
346	a	1430.47	3.64805	YES	YES
347	a	1431.89	3.02089	YES	YES
348	a	1432.54	1.36521	YES	YES
349	a	1433.39	3.22049	YES	YES
350	a	1434.71	2.42577	YES	YES
351	a	1435.07	5.84985	YES	YES
352	a	1435.93	7.14829	YES	YES
353	a	1436.34	6.89878	YES	YES
354	a	1436.61	5.16285	YES	YES
355	a	1436.76	4.80711	YES	YES
356	a	1437.70	0.91540	YES	YES
357	a	1438.06	11.02221	YES	YES
358	a	1438.14	3.27803	YES	YES
359	a	1438.70	3.80005	YES	YES
360	a	1439.08	4.09397	YES	YES
361	a	1439.53	1.95599	YES	YES
362	a	1439.99	9.41174	YES	YES
363	a	1440.18	8.23962	YES	YES
364	a	1440.33	1.63138	YES	YES
365	a	1441.44	7.61258	YES	YES
366	a	1442.65	12.46337	YES	YES
367	a	1443.46	10.97033	YES	YES
368	a	1443.66	21.09438	YES	YES
369	a	1444.42	12.04306	YES	YES
370	a	1444.79	19.18149	YES	YES
371	a	1444.89	5.71627	YES	YES
372	a	1445.03	2.31336	YES	YES
373	a	1447.61	10.23565	YES	YES
374	a	1452.57	4.14909	YES	YES
375	a	1453.53	0.27959	YES	YES
376	a	1454.11	0.61372	YES	YES
377	a	1454.67	2.93586	YES	YES
378	a	1455.25	0.78177	YES	YES
379	a	1456.30	3.93426	YES	YES
380	a	1456.60	2.31104	YES	YES
381	a	1458.88	1.57548	YES	YES
382	a	1558.53	110.99801	YES	YES
383	a	2912.34	21.36487	YES	YES
384	a	2922.60	3.63564	YES	YES
385	a	2929.49	21.21774	YES	YES
386	a	2932.31	16.88844	YES	YES
387	a	2935.29	28.00346	YES	YES
388	a	2935.97	7.94119	YES	YES
389	a	2936.90	10.93507	YES	YES
390	a	2938.27	22.93360	YES	YES
391	a	2938.64	10.33116	YES	YES
392	a	2939.04	13.74503	YES	YES
393	a	2939.44	4.58042	YES	YES

394	a	2941.19	10.97826	YES	YES
395	a	2942.82	17.51430	YES	YES
396	a	2944.55	11.35046	YES	YES
397	a	2944.97	4.74586	YES	YES
398	a	2945.15	6.97742	YES	YES
399	a	2945.46	8.42599	YES	YES
400	a	2946.07	12.98743	YES	YES
401	a	2946.21	3.86888	YES	YES
402	a	2947.22	7.98334	YES	YES
403	a	2947.97	15.59569	YES	YES
404	a	2948.58	8.19608	YES	YES
405	a	2948.89	5.60544	YES	YES
406	a	2949.50	8.26514	YES	YES
407	a	2950.46	31.81641	YES	YES
408	a	2951.00	22.20218	YES	YES
409	a	2951.49	9.94434	YES	YES
410	a	2953.04	1.83296	YES	YES
411	a	2954.95	2.47691	YES	YES
412	a	2955.28	2.31368	YES	YES
413	a	2956.06	2.70482	YES	YES
414	a	2956.48	7.50830	YES	YES
415	a	2957.29	10.38425	YES	YES
416	a	2957.40	20.42551	YES	YES
417	a	2958.08	7.08116	YES	YES
418	a	2958.10	2.77590	YES	YES
419	a	2958.45	3.66110	YES	YES
420	a	2959.40	1.86921	YES	YES
421	a	2960.04	19.47679	YES	YES
422	a	2960.15	38.47447	YES	YES
423	a	2960.80	23.93108	YES	YES
424	a	2961.46	37.97970	YES	YES
425	a	2961.66	63.86324	YES	YES
426	a	2961.81	17.17821	YES	YES
427	a	2962.91	21.14058	YES	YES
428	a	2963.91	12.57410	YES	YES
429	a	2964.34	1.92759	YES	YES
430	a	2966.15	20.60434	YES	YES
431	a	2978.33	8.10029	YES	YES
432	a	2980.56	31.93261	YES	YES
433	a	2981.23	4.66322	YES	YES
434	a	2984.35	12.71779	YES	YES
435	a	2985.62	5.51430	YES	YES
436	a	2986.70	24.53379	YES	YES
437	a	2987.12	50.76129	YES	YES
438	a	2987.34	11.83979	YES	YES
439	a	2987.45	13.56269	YES	YES
440	a	2989.63	9.21686	YES	YES
441	a	2991.04	31.96067	YES	YES
442	a	2997.76	21.68391	YES	YES
443	a	2998.39	32.59202	YES	YES
444	a	3000.23	31.97986	YES	YES
445	a	3001.66	14.62855	YES	YES
446	a	3002.71	8.00225	YES	YES
447	a	3003.20	25.16282	YES	YES
448	a	3003.84	9.69151	YES	YES
449	a	3004.04	32.17444	YES	YES
450	a	3004.26	38.95748	YES	YES
451	a	3006.52	16.68191	YES	YES

452	a	3007.32	15.35457	YES	YES
453	a	3008.57	18.19159	YES	YES
454	a	3009.38	32.65254	YES	YES
455	a	3009.91	2.17467	YES	YES
456	a	3010.33	37.66731	YES	YES
457	a	3010.48	18.00753	YES	YES
458	a	3010.74	23.43234	YES	YES
459	a	3013.04	16.01405	YES	YES
460	a	3014.72	22.07104	YES	YES
461	a	3015.10	21.08720	YES	YES
462	a	3016.46	16.72450	YES	YES
463	a	3016.53	34.59639	YES	YES
464	a	3017.08	14.14342	YES	YES
465	a	3017.25	21.03488	YES	YES
466	a	3017.31	51.30093	YES	YES
467	a	3017.41	12.86176	YES	YES
468	a	3017.83	20.20198	YES	YES
469	a	3018.42	20.20915	YES	YES
470	a	3018.54	27.92990	YES	YES
471	a	3018.64	16.92846	YES	YES
472	a	3018.92	26.18963	YES	YES
473	a	3019.05	28.46485	YES	YES
474	a	3019.41	22.64386	YES	YES
475	a	3022.63	6.62352	YES	YES
476	a	3024.70	26.22837	YES	YES
477	a	3027.37	4.44302	YES	YES
478	a	3029.46	0.43871	YES	YES
479	a	3037.63	0.82462	YES	YES
480	a	3043.60	0.26242	YES	YES

$end

Double hybrid single point energy = -7480.832967431653 H
COSMO energy + OC correction = -7488.4523583825 H (in *o*DFB)

6.2.47 [F{Ga(dcpe)}$_2$(CH$_2$CN)]$^{2+}$

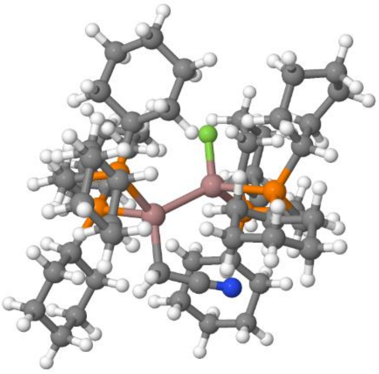

Method: (RI-)BP86(D3BJ)/def2-TZVPP
Symmetry: c1

Cartesian coordinates in Ångström:

Ga	0.0523128	-0.0611468	-1.4363081
Ga	-0.4675508	-0.0577757	0.9659102
F	0.7627414	0.8759795	1.9567170
N	-3.2844698	-1.5128843	-0.9568105

C	-2.4955561	-1.2088434	-1.7620902
C	-1.4398079	-0.8109386	-2.6437100
H	-1.7903145	-0.0521451	-3.3527200
H	-1.0761332	-1.6733460	-3.2148335
P	-2.5943668	0.7493809	1.7997769
P	-0.9179814	-2.1320087	2.1521298
C	-2.5299213	1.9821496	3.1663745
H	-3.5875843	2.1444382	3.4342595
C	-1.7846327	1.4897388	4.4168485
H	-2.1433169	0.5031679	4.7321609
H	-0.7189551	1.3812819	4.1686041
C	-1.9887793	2.4842345	5.5847605
H	-2.8641602	2.1829766	6.1762712
H	-1.1214190	2.4147688	6.2556035
C	-2.1627218	3.9371630	5.0991776
H	-1.8531029	4.6368203	5.8853809
H	-3.2253298	4.1506078	4.9039872
C	-1.3583255	4.1645521	3.8194623
H	-1.3668139	5.2229751	3.5280638
H	-0.3063852	3.8997195	4.0027067
C	-1.9132353	3.3091443	2.6606420
H	-1.1005914	3.0885432	1.9557964
H	-2.6761676	3.8738687	2.1072993
C	-3.7267061	1.3657216	0.4821357
H	-4.0280895	0.4338063	-0.0236043
C	-4.9906191	2.0623472	1.0127120
H	-5.5159211	1.4175131	1.7320290
H	-4.7122109	2.9833609	1.5485949
C	-5.9233006	2.4183527	-0.1541629
H	-6.2876436	1.4887244	-0.6199363
H	-6.8071408	2.9448607	0.2301949
C	-5.2080859	3.2722805	-1.2062678
H	-4.9405339	4.2472618	-0.7661007
H	-5.8845714	3.4839813	-2.0450249
C	-3.9368140	2.5824824	-1.7141103
H	-3.4149914	3.2247921	-2.4383009
H	-4.2091325	1.6565418	-2.2472193
C	-2.9959576	2.2333220	-0.5571008
H	-2.1069165	1.6979971	-0.9306048
H	-2.6288491	3.1580497	-0.0857826
C	-3.3823916	-0.7902927	2.4522934
H	-4.2089832	-0.5203204	3.1241648
H	-3.8167506	-1.2922129	1.5755187
C	-2.3953308	-1.7245513	3.1742588
H	-2.9000047	-2.6540880	3.4669102
H	-2.0153032	-1.2658182	4.0940883
C	0.4147020	-2.6914758	3.2945177
H	1.2571990	-2.9271970	2.6199346
C	0.8512707	-1.5284538	4.2085283
H	0.0025130	-1.2297573	4.8451944
H	1.1232158	-0.6496388	3.6083701
C	2.0132151	-1.9548486	5.1145321
H	2.8998533	-2.1587224	4.4923564
H	2.2818967	-1.1205982	5.7770389
C	1.6644122	-3.2032711	5.9287772
H	0.8447086	-2.9676856	6.6275675
H	2.5203465	-3.5081066	6.5455631
C	1.2369110	-4.3532304	5.0134399

H	2.0900892	-4.6587653	4.3848868
H	0.9527108	-5.2351673	5.6032739
C	0.0665569	-3.9527593	4.1041309
H	-0.8256032	-3.7565094	4.7204862
H	-0.1850617	-4.7848841	3.4341494
C	-1.4273693	-3.4672152	0.9853775
H	-1.9753852	-2.9169792	0.2029230
C	-0.1885929	-4.1049679	0.3355160
H	0.4463529	-3.3188164	-0.1072042
H	0.4204975	-4.6093122	1.1022425
C	-0.6004558	-5.1247217	-0.7330472
H	0.2953824	-5.6000966	-1.1562186
H	-1.1042134	-4.5963868	-1.5595051
C	-1.5508415	-6.1808049	-0.1631241
H	-1.8502026	-6.8834217	-0.9522770
H	-1.0226301	-6.7751743	0.6005977
C	-2.7849414	-5.5243925	0.4597652
H	-3.4542475	-6.2840865	0.8850779
H	-3.3576961	-4.9996693	-0.3219613
C	-2.3958915	-4.5198804	1.5526734
H	-3.3030590	-4.0343617	1.9369219
H	-1.9355525	-5.0547734	2.3957948
P	2.1873046	-1.0310820	-2.0765617
P	1.0060268	2.0362554	-2.1703428
C	2.1471265	-2.2544570	-3.4527841
H	3.1998918	-2.5285688	-3.6382578
C	1.5790252	-1.6359469	-4.7438541
H	2.2207734	-0.8094559	-5.0804805
H	0.5838248	-1.2059028	-4.5459924
C	1.4658551	-2.6855388	-5.8574313
H	1.0153404	-2.2248653	-6.7469461
H	2.4772221	-3.0096679	-6.1508236
C	0.6544362	-3.9022079	-5.4048349
H	0.6006354	-4.6428421	-6.2132968
H	-0.3848985	-3.5960716	-5.1944833
C	1.2662072	-4.5305613	-4.1504706
H	2.2691405	-4.9206132	-4.3871394
H	0.6685983	-5.3881112	-3.8126192
C	1.3781261	-3.5098461	-3.0131128
H	0.3664401	-3.2207937	-2.6843921
H	1.8672971	-3.9661922	-2.1409758
C	3.1083178	-1.7759483	-0.6642370
H	2.4382869	-2.5781127	-0.3073337
C	4.4550116	-2.4031235	-1.0623061
H	5.1162813	-1.6203387	-1.4686363
H	4.3199719	-3.1517788	-1.8548692
C	5.1229116	-3.0539883	0.1580636
H	4.5074829	-3.9079752	0.4868315
H	6.0970409	-3.4670143	-0.1359115
C	5.2832319	-2.0642154	1.3158120
H	5.9917965	-1.2714003	1.0249255
H	5.7234918	-2.5685764	2.1864019
C	3.9418830	-1.4289913	1.6908839
H	3.2650443	-2.2065442	2.0835246
H	4.0660692	-0.6891239	2.4929512
C	3.2791668	-0.7630955	0.4801917
H	3.9188246	0.0693179	0.1436555
H	2.3169781	-0.3207263	0.7747767

```
C     3.2039602    0.3866077   -2.6904141
H     3.7787141    0.7621427   -1.8336928
H     3.9310959    0.0081230   -3.4217855
C     2.3635315    1.5166864   -3.3014849
H     1.8945956    1.1961905   -4.2409818
H     3.0006606    2.3806934   -3.5320327
C    -0.0069003    3.2875183   -3.0693411
H    -0.7377917    3.6313311   -2.3175674
C    -0.7852206    2.6339214   -4.2265234
H    -1.4159545    1.8212919   -3.8410699
H    -0.0791151    2.1827425   -4.9426120
C    -1.6555477    3.6632130   -4.9600978
H    -2.1644531    3.1756419   -5.8026991
H    -2.4465464    4.0139208   -4.2769596
C    -0.8344613    4.8624059   -5.4396581
H    -0.1113898    4.5322397   -6.2036853
H    -1.4886903    5.5966598   -5.9278110
C    -0.0838415    5.5115700   -4.2747264
H    -0.8074051    5.9304322   -3.5560861
H     0.5292913    6.3519120   -4.6269766
C     0.8137802    4.4983447   -3.5519394
H     1.3204118    4.9809619   -2.7063364
H     1.5994896    4.1558101   -4.2439315
C     1.8104314    2.7865874   -0.6852708
H     2.0041047    1.9223952   -0.0252515
C     0.8031799    3.6859900    0.0505986
H     0.5564889    4.5547839   -0.5794978
H    -0.1358933    3.1372751    0.2188969
C     1.3888425    4.1681993    1.3816109
H     0.6762035    4.8456920    1.8705811
H     1.5135889    3.2970782    2.0432081
C     2.7332837    4.8729028    1.1771781
H     3.1557134    5.1681005    2.1469286
H     2.5773796    5.8049168    0.6091872
C     3.7224561    3.9813699    0.4207984
H     4.6680634    4.5125335    0.2474471
H     3.9628600    3.0967760    1.0337445
C     3.1429539    3.5151583   -0.9222854
H     2.9879034    4.3853830   -1.5769394
H     3.8672487    2.8642793   -1.4320713

SCF energy GEOOPT =  -7488.371066174 H
ZPE =   3699. kJ/mol
FREEH energy =  3889.25 kJ/mol
FREEH entropy =   1.62075 kJ/mol/K

$vibrational spectrum
#  mode     symmetry     wave number   IR intensity    selection rules
#                         cm**(-1)        km/mol          IR     RAMAN
     1                      -0.00         0.00000         -       -
     2                      -0.00         0.00000         -       -
     3                       0.00         0.00000         -       -
     4                       0.00         0.00000         -       -
     5                       0.00         0.00000         -       -
     6                       0.00         0.00000         -       -
     7         a             8.19         0.25380        YES     YES
     8         a            17.77         0.18374        YES     YES
     9         a            22.37         0.01370        YES     YES
```

10	a	26.50	0.18655	YES	YES
11	a	28.28	0.01829	YES	YES
12	a	31.39	0.03500	YES	YES
13	a	33.09	0.10348	YES	YES
14	a	35.05	0.03976	YES	YES
15	a	38.99	0.19782	YES	YES
16	a	42.59	0.03348	YES	YES
17	a	43.42	0.26720	YES	YES
18	a	45.22	0.11617	YES	YES
19	a	50.18	0.01243	YES	YES
20	a	51.84	0.25191	YES	YES
21	a	55.16	0.19058	YES	YES
22	a	56.19	0.04764	YES	YES
23	a	58.77	0.10960	YES	YES
24	a	60.59	0.13406	YES	YES
25	a	65.69	0.21966	YES	YES
26	a	66.37	0.29034	YES	YES
27	a	68.86	0.45061	YES	YES
28	a	73.11	0.17902	YES	YES
29	a	75.45	0.12209	YES	YES
30	a	78.73	0.40498	YES	YES
31	a	80.96	0.34750	YES	YES
32	a	82.24	0.82107	YES	YES
33	a	84.89	1.09657	YES	YES
34	a	91.07	1.18113	YES	YES
35	a	96.96	1.40001	YES	YES
36	a	98.92	2.14479	YES	YES
37	a	107.80	2.06718	YES	YES
38	a	112.30	4.97500	YES	YES
39	a	120.52	0.27270	YES	YES
40	a	124.28	0.47097	YES	YES
41	a	133.31	0.57413	YES	YES
42	a	134.35	10.84920	YES	YES
43	a	136.63	1.53407	YES	YES
44	a	138.31	0.64870	YES	YES
45	a	141.50	3.65389	YES	YES
46	a	145.70	0.66451	YES	YES
47	a	149.29	1.75406	YES	YES
48	a	154.67	23.43716	YES	YES
49	a	157.24	3.39992	YES	YES
50	a	162.39	5.20780	YES	YES
51	a	163.10	2.90105	YES	YES
52	a	165.79	2.98678	YES	YES
53	a	179.54	2.80361	YES	YES
54	a	185.29	1.02191	YES	YES
55	a	188.12	4.56231	YES	YES
56	a	191.96	8.27372	YES	YES
57	a	195.28	3.40283	YES	YES
58	a	205.70	2.05613	YES	YES
59	a	206.85	0.44875	YES	YES
60	a	209.04	0.34941	YES	YES
61	a	217.72	2.19188	YES	YES
62	a	221.98	0.40939	YES	YES
63	a	224.37	1.01377	YES	YES
64	a	224.89	1.08318	YES	YES
65	a	230.61	0.17201	YES	YES
66	a	231.89	0.65457	YES	YES
67	a	233.45	1.58254	YES	YES

68	a	237.14	0.47228	YES	YES
69	a	239.25	0.02813	YES	YES
70	a	240.65	0.17074	YES	YES
71	a	242.48	1.04636	YES	YES
72	a	252.42	0.08238	YES	YES
73	a	266.53	0.79249	YES	YES
74	a	276.98	1.05972	YES	YES
75	a	291.69	0.74123	YES	YES
76	a	295.16	0.36458	YES	YES
77	a	306.12	0.37206	YES	YES
78	a	307.19	0.63249	YES	YES
79	a	311.77	0.48419	YES	YES
80	a	314.40	0.30561	YES	YES
81	a	329.89	0.65916	YES	YES
82	a	330.33	0.68091	YES	YES
83	a	331.71	1.70331	YES	YES
84	a	336.64	0.14403	YES	YES
85	a	338.49	1.43836	YES	YES
86	a	341.45	0.28758	YES	YES
87	a	349.57	3.42887	YES	YES
88	a	370.51	0.16228	YES	YES
89	a	378.42	0.91924	YES	YES
90	a	381.16	0.71376	YES	YES
91	a	386.85	1.00989	YES	YES
92	a	388.61	1.79929	YES	YES
93	a	390.25	0.10024	YES	YES
94	a	403.18	2.04781	YES	YES
95	a	408.49	2.73048	YES	YES
96	a	414.38	2.64052	YES	YES
97	a	417.09	3.14654	YES	YES
98	a	424.28	1.51184	YES	YES
99	a	425.06	1.65655	YES	YES
100	a	427.04	0.09366	YES	YES
101	a	429.88	0.64396	YES	YES
102	a	430.60	0.17615	YES	YES
103	a	430.94	0.06081	YES	YES
104	a	431.60	0.05371	YES	YES
105	a	432.38	0.00130	YES	YES
106	a	435.27	1.45117	YES	YES
107	a	436.19	3.36931	YES	YES
108	a	448.53	0.43789	YES	YES
109	a	457.13	3.79996	YES	YES
110	a	465.40	0.95053	YES	YES
111	a	466.78	5.18802	YES	YES
112	a	469.56	1.30836	YES	YES
113	a	492.13	2.55476	YES	YES
114	a	497.73	0.36520	YES	YES
115	a	499.37	0.31496	YES	YES
116	a	503.25	1.32462	YES	YES
117	a	504.62	0.43496	YES	YES
118	a	514.43	12.52952	YES	YES
119	a	516.24	4.94661	YES	YES
120	a	525.67	1.08589	YES	YES
121	a	547.15	46.28633	YES	YES
122	a	576.43	5.60555	YES	YES
123	a	628.29	3.65725	YES	YES
124	a	629.24	29.88220	YES	YES
125	a	633.48	16.90612	YES	YES

126	a	644.93	9.01299	YES	YES
127	a	653.60	8.68930	YES	YES
128	a	701.14	4.37901	YES	YES
129	a	710.99	3.55426	YES	YES
130	a	715.48	5.59097	YES	YES
131	a	728.74	11.25163	YES	YES
132	a	729.59	3.74708	YES	YES
133	a	732.76	9.91982	YES	YES
134	a	738.32	1.61807	YES	YES
135	a	740.55	1.20429	YES	YES
136	a	760.20	1.20740	YES	YES
137	a	770.33	0.16069	YES	YES
138	a	771.33	0.15714	YES	YES
139	a	773.58	0.11898	YES	YES
140	a	773.88	0.08189	YES	YES
141	a	775.15	0.02981	YES	YES
142	a	775.77	0.26180	YES	YES
143	a	776.58	0.05069	YES	YES
144	a	787.40	22.14841	YES	YES
145	a	790.94	15.14475	YES	YES
146	a	808.17	0.79425	YES	YES
147	a	808.58	0.45040	YES	YES
148	a	810.08	2.40455	YES	YES
149	a	812.76	5.20379	YES	YES
150	a	813.61	1.19413	YES	YES
151	a	815.27	0.45593	YES	YES
152	a	815.84	4.08620	YES	YES
153	a	819.81	6.56057	YES	YES
154	a	834.00	2.80715	YES	YES
155	a	834.13	2.44898	YES	YES
156	a	836.10	4.03590	YES	YES
157	a	836.82	3.26224	YES	YES
158	a	837.18	3.58750	YES	YES
159	a	838.55	4.83510	YES	YES
160	a	839.82	7.05459	YES	YES
161	a	842.85	3.12030	YES	YES
162	a	853.19	18.50714	YES	YES
163	a	857.75	9.72857	YES	YES
164	a	872.96	2.24542	YES	YES
165	a	873.62	2.92854	YES	YES
166	a	874.27	5.09883	YES	YES
167	a	875.20	1.61870	YES	YES
168	a	876.74	3.48055	YES	YES
169	a	877.97	3.88430	YES	YES
170	a	878.05	4.23904	YES	YES
171	a	879.58	2.99681	YES	YES
172	a	879.87	1.54533	YES	YES
173	a	880.94	0.25250	YES	YES
174	a	881.17	4.45138	YES	YES
175	a	881.73	1.11849	YES	YES
176	a	883.07	0.90024	YES	YES
177	a	884.94	3.63472	YES	YES
178	a	886.53	0.24663	YES	YES
179	a	897.05	1.86496	YES	YES
180	a	903.43	2.51891	YES	YES
181	a	904.01	2.84687	YES	YES
182	a	904.37	1.84342	YES	YES
183	a	905.62	2.74945	YES	YES

184	a	906.17	1.64522	YES	YES
185	a	907.95	0.75680	YES	YES
186	a	909.50	1.29411	YES	YES
187	a	910.44	0.50849	YES	YES
188	a	967.86	3.11439	YES	YES
189	a	970.79	2.39095	YES	YES
190	a	982.11	3.16169	YES	YES
191	a	985.99	4.25643	YES	YES
192	a	986.81	9.55953	YES	YES
193	a	987.14	11.17813	YES	YES
194	a	988.60	14.03296	YES	YES
195	a	990.79	9.23372	YES	YES
196	a	991.39	3.08788	YES	YES
197	a	992.78	15.26908	YES	YES
198	a	993.35	4.75655	YES	YES
199	a	1014.25	0.91028	YES	YES
200	a	1015.80	1.24008	YES	YES
201	a	1016.43	0.29166	YES	YES
202	a	1017.58	0.47064	YES	YES
203	a	1018.17	0.51522	YES	YES
204	a	1019.13	0.78987	YES	YES
205	a	1020.24	1.63992	YES	YES
206	a	1022.97	0.54069	YES	YES
207	a	1031.24	4.89095	YES	YES
208	a	1032.24	3.54864	YES	YES
209	a	1033.80	10.40113	YES	YES
210	a	1034.53	23.49940	YES	YES
211	a	1039.39	1.10010	YES	YES
212	a	1039.42	1.70798	YES	YES
213	a	1040.64	0.66268	YES	YES
214	a	1042.39	0.79305	YES	YES
215	a	1046.38	2.06981	YES	YES
216	a	1059.07	0.74978	YES	YES
217	a	1059.09	2.91215	YES	YES
218	a	1060.26	2.25510	YES	YES
219	a	1061.07	0.28624	YES	YES
220	a	1061.94	1.97673	YES	YES
221	a	1063.99	0.66028	YES	YES
222	a	1065.49	0.50780	YES	YES
223	a	1070.86	0.11809	YES	YES
224	a	1071.25	0.42047	YES	YES
225	a	1071.47	0.03194	YES	YES
226	a	1071.53	0.02685	YES	YES
227	a	1072.40	0.61409	YES	YES
228	a	1072.62	0.03625	YES	YES
229	a	1074.80	0.27975	YES	YES
230	a	1075.13	0.09584	YES	YES
231	a	1079.30	3.56293	YES	YES
232	a	1086.88	1.39628	YES	YES
233	a	1090.51	2.92625	YES	YES
234	a	1092.67	1.87266	YES	YES
235	a	1096.10	3.23544	YES	YES
236	a	1096.25	9.78016	YES	YES
237	a	1102.99	0.52102	YES	YES
238	a	1104.05	22.43872	YES	YES
239	a	1106.17	0.73506	YES	YES
240	a	1107.59	8.48199	YES	YES
241	a	1108.27	2.16430	YES	YES

242	a	1111.43	9.83363	YES	YES
243	a	1125.88	5.64191	YES	YES
244	a	1128.35	2.76797	YES	YES
245	a	1160.96	9.09199	YES	YES
246	a	1162.28	7.41003	YES	YES
247	a	1163.53	5.06071	YES	YES
248	a	1165.96	8.70485	YES	YES
249	a	1167.39	6.43667	YES	YES
250	a	1168.07	1.32077	YES	YES
251	a	1170.43	6.12683	YES	YES
252	a	1171.08	6.28559	YES	YES
253	a	1177.95	13.63029	YES	YES
254	a	1183.98	5.60379	YES	YES
255	a	1186.94	3.72221	YES	YES
256	a	1189.00	2.99147	YES	YES
257	a	1192.55	2.38715	YES	YES
258	a	1203.12	4.15489	YES	YES
259	a	1205.43	2.64377	YES	YES
260	a	1209.25	3.97394	YES	YES
261	a	1222.94	2.26784	YES	YES
262	a	1240.28	0.58314	YES	YES
263	a	1244.01	0.10376	YES	YES
264	a	1244.30	0.62091	YES	YES
265	a	1244.42	0.36352	YES	YES
266	a	1245.27	0.68092	YES	YES
267	a	1247.62	0.44490	YES	YES
268	a	1248.86	0.62240	YES	YES
269	a	1250.29	0.67470	YES	YES
270	a	1251.71	0.61093	YES	YES
271	a	1252.21	1.06634	YES	YES
272	a	1253.11	1.53035	YES	YES
273	a	1253.86	2.24691	YES	YES
274	a	1256.28	2.12064	YES	YES
275	a	1256.69	8.91701	YES	YES
276	a	1257.59	1.22204	YES	YES
277	a	1258.12	4.37253	YES	YES
278	a	1258.73	8.97000	YES	YES
279	a	1259.45	2.09295	YES	YES
280	a	1260.23	1.47355	YES	YES
281	a	1260.80	0.79099	YES	YES
282	a	1260.89	2.65481	YES	YES
283	a	1266.47	1.41675	YES	YES
284	a	1266.57	4.27314	YES	YES
285	a	1269.09	1.54757	YES	YES
286	a	1270.27	2.47228	YES	YES
287	a	1270.49	9.28955	YES	YES
288	a	1277.46	4.31557	YES	YES
289	a	1281.81	1.73080	YES	YES
290	a	1283.09	5.16355	YES	YES
291	a	1283.92	5.62349	YES	YES
292	a	1285.41	0.85593	YES	YES
293	a	1287.83	0.60196	YES	YES
294	a	1290.06	6.21091	YES	YES
295	a	1291.37	1.81235	YES	YES
296	a	1294.00	4.12104	YES	YES
297	a	1308.39	1.32456	YES	YES
298	a	1309.41	1.03056	YES	YES
299	a	1310.16	3.35511	YES	YES

300	a	1310.55	3.37591	YES	YES
301	a	1311.59	0.49960	YES	YES
302	a	1313.69	0.69881	YES	YES
303	a	1314.47	0.86181	YES	YES
304	a	1318.16	2.27429	YES	YES
305	a	1320.78	1.26771	YES	YES
306	a	1321.96	0.44801	YES	YES
307	a	1322.63	1.04548	YES	YES
308	a	1322.93	3.16168	YES	YES
309	a	1323.06	0.20243	YES	YES
310	a	1323.61	1.15882	YES	YES
311	a	1324.46	0.94557	YES	YES
312	a	1324.59	0.26721	YES	YES
313	a	1324.71	2.79394	YES	YES
314	a	1325.01	1.03923	YES	YES
315	a	1325.43	0.73835	YES	YES
316	a	1325.86	4.13952	YES	YES
317	a	1327.02	1.50254	YES	YES
318	a	1327.86	4.95600	YES	YES
319	a	1328.75	0.45490	YES	YES
320	a	1329.76	5.07738	YES	YES
321	a	1336.02	0.86282	YES	YES
322	a	1336.42	0.02843	YES	YES
323	a	1336.58	0.23010	YES	YES
324	a	1337.23	0.19998	YES	YES
325	a	1337.91	0.34326	YES	YES
326	a	1338.25	0.68907	YES	YES
327	a	1338.96	0.52757	YES	YES
328	a	1339.27	0.12070	YES	YES
329	a	1341.15	0.41578	YES	YES
330	a	1343.40	2.20514	YES	YES
331	a	1345.44	3.95405	YES	YES
332	a	1345.58	1.74411	YES	YES
333	a	1346.68	1.83770	YES	YES
334	a	1347.17	1.84908	YES	YES
335	a	1349.65	0.52872	YES	YES
336	a	1350.97	0.24163	YES	YES
337	a	1385.48	5.62842	YES	YES
338	a	1405.31	7.44008	YES	YES
339	a	1407.68	5.47027	YES	YES
340	a	1409.10	7.10351	YES	YES
341	a	1411.31	9.97230	YES	YES
342	a	1425.04	1.78501	YES	YES
343	a	1428.52	5.51423	YES	YES
344	a	1428.83	5.45957	YES	YES
345	a	1429.25	0.15308	YES	YES
346	a	1432.11	3.39980	YES	YES
347	a	1434.76	7.79498	YES	YES
348	a	1434.83	1.28618	YES	YES
349	a	1435.58	2.83749	YES	YES
350	a	1435.82	2.88690	YES	YES
351	a	1436.05	4.48776	YES	YES
352	a	1437.34	3.33190	YES	YES
353	a	1437.42	5.21537	YES	YES
354	a	1438.05	2.06533	YES	YES
355	a	1438.20	8.73586	YES	YES
356	a	1438.34	0.25181	YES	YES
357	a	1438.96	2.21621	YES	YES

358	a	1439.25	5.22477	YES	YES
359	a	1439.59	7.11098	YES	YES
360	a	1439.68	5.62042	YES	YES
361	a	1439.91	10.85779	YES	YES
362	a	1440.29	2.74941	YES	YES
363	a	1440.50	8.89950	YES	YES
364	a	1442.53	5.76258	YES	YES
365	a	1442.98	14.42935	YES	YES
366	a	1443.65	9.24418	YES	YES
367	a	1444.77	9.66047	YES	YES
368	a	1445.11	7.88105	YES	YES
369	a	1445.66	31.69980	YES	YES
370	a	1446.20	1.90405	YES	YES
371	a	1447.79	11.07419	YES	YES
372	a	1449.50	3.10971	YES	YES
373	a	1453.82	2.44803	YES	YES
374	a	1454.17	4.24223	YES	YES
375	a	1454.63	2.72055	YES	YES
376	a	1454.93	1.19151	YES	YES
377	a	1456.49	14.12508	YES	YES
378	a	1457.10	2.81312	YES	YES
379	a	1457.89	2.14374	YES	YES
380	a	1458.67	0.75134	YES	YES
381	a	1466.93	1.08934	YES	YES
382	a	2223.75	77.97516	YES	YES
383	a	2929.66	5.23740	YES	YES
384	a	2930.93	64.18470	YES	YES
385	a	2933.10	10.00028	YES	YES
386	a	2934.36	22.02737	YES	YES
387	a	2935.45	3.67204	YES	YES
388	a	2937.33	10.19922	YES	YES
389	a	2939.95	11.09885	YES	YES
390	a	2941.16	6.40389	YES	YES
391	a	2942.18	13.75533	YES	YES
392	a	2943.24	7.44074	YES	YES
393	a	2943.31	2.18109	YES	YES
394	a	2944.42	8.00835	YES	YES
395	a	2944.46	11.84195	YES	YES
396	a	2944.71	10.20063	YES	YES
397	a	2944.92	3.24191	YES	YES
398	a	2946.16	9.61504	YES	YES
399	a	2947.53	10.28745	YES	YES
400	a	2948.21	13.80290	YES	YES
401	a	2950.11	15.52811	YES	YES
402	a	2951.08	20.14381	YES	YES
403	a	2951.64	7.48734	YES	YES
404	a	2952.48	12.69640	YES	YES
405	a	2953.03	10.54183	YES	YES
406	a	2954.06	8.50176	YES	YES
407	a	2954.94	2.67472	YES	YES
408	a	2955.51	22.66561	YES	YES
409	a	2955.82	9.35210	YES	YES
410	a	2956.86	26.84709	YES	YES
411	a	2957.40	5.76011	YES	YES
412	a	2957.84	5.24925	YES	YES
413	a	2959.01	10.13183	YES	YES
414	a	2959.10	1.63484	YES	YES
415	a	2959.94	14.84593	YES	YES

416	a	2960.88	5.53817	YES	YES
417	a	2961.53	56.91082	YES	YES
418	a	2962.12	37.23793	YES	YES
419	a	2962.59	23.93391	YES	YES
420	a	2963.25	46.31108	YES	YES
421	a	2964.26	51.93359	YES	YES
422	a	2964.32	14.65401	YES	YES
423	a	2965.81	30.54734	YES	YES
424	a	2968.53	12.04987	YES	YES
425	a	2971.61	17.23299	YES	YES
426	a	2972.80	19.79783	YES	YES
427	a	2976.38	23.13314	YES	YES
428	a	2976.65	12.20651	YES	YES
429	a	2977.81	19.25279	YES	YES
430	a	2978.19	7.18033	YES	YES
431	a	2983.02	10.78820	YES	YES
432	a	2983.86	24.11309	YES	YES
433	a	2986.08	14.81474	YES	YES
434	a	2988.28	2.98643	YES	YES
435	a	2992.52	1.58100	YES	YES
436	a	2994.52	29.79771	YES	YES
437	a	3000.92	17.03058	YES	YES
438	a	3002.03	23.86553	YES	YES
439	a	3003.11	5.44867	YES	YES
440	a	3003.33	8.68810	YES	YES
441	a	3004.11	24.40784	YES	YES
442	a	3005.58	9.87300	YES	YES
443	a	3007.56	28.69391	YES	YES
444	a	3008.17	17.53395	YES	YES
445	a	3009.22	20.80724	YES	YES
446	a	3009.68	21.45867	YES	YES
447	a	3010.68	16.33278	YES	YES
448	a	3010.89	20.26606	YES	YES
449	a	3012.54	15.12842	YES	YES
450	a	3013.56	19.86282	YES	YES
451	a	3013.95	29.10610	YES	YES
452	a	3014.08	16.66886	YES	YES
453	a	3014.19	18.14812	YES	YES
454	a	3014.80	12.93289	YES	YES
455	a	3015.25	14.62535	YES	YES
456	a	3015.42	19.22539	YES	YES
457	a	3016.15	18.23043	YES	YES
458	a	3016.16	25.00730	YES	YES
459	a	3016.25	6.94923	YES	YES
460	a	3016.32	26.37760	YES	YES
461	a	3016.65	13.77411	YES	YES
462	a	3016.82	28.43493	YES	YES
463	a	3016.86	37.96702	YES	YES
464	a	3017.98	23.85088	YES	YES
465	a	3018.21	12.28067	YES	YES
466	a	3018.86	20.53599	YES	YES
467	a	3019.36	21.73841	YES	YES
468	a	3020.03	17.67993	YES	YES
469	a	3020.92	27.98614	YES	YES
470	a	3021.63	10.34625	YES	YES
471	a	3022.74	39.94889	YES	YES
472	a	3024.97	27.83245	YES	YES
473	a	3028.08	18.89936	YES	YES

474	a	3028.40	20.89781	YES	YES
475	a	3029.81	1.20114	YES	YES
476	a	3033.98	0.71745	YES	YES
477	a	3038.28	22.06289	YES	YES
478	a	3044.76	0.37350	YES	YES
479	a	3053.73	1.48818	YES	YES
480	a	3060.18	1.20992	YES	YES

$end

Double hybrid single point energy = -7480.928580306914 H
COSMO energy + OC correction = -7488.5208407909 H (in *o*DFB)

6.2.48 $PhCF_3$

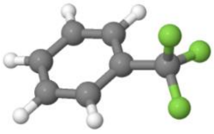

Method: (RI-)BP86(D3BJ)/def2-TZVPP
Symmetry: cs

Cartesian coordinates in Ångström:

H	0.2910558	3.1833279	0.0000000
C	0.1992900	2.0974380	0.0000000
C	0.1421281	1.4014188	-1.2098584
H	0.1897234	1.9415917	-2.1548306
C	0.0271671	0.0111957	-1.2132341
H	-0.0125869	-0.5368621	-2.1529977
C	-0.0279844	-0.6824439	0.0000000
C	-0.2024647	-2.1788288	0.0000000
C	0.0271671	0.0111957	1.2132341
H	-0.0125869	-0.5368621	2.1529977
C	0.1421281	1.4014188	1.2098584
H	0.1897234	1.9415917	2.1548306
F	0.3550461	-2.7630774	-1.0949976
F	-1.5234809	-2.5303479	0.0000000
F	0.3550461	-2.7630774	1.0949976

SCF energy GEOOPT = -569.5771654083 H
ZPE = 267.9 kJ/mol
FREEH energy = 289.23 kJ/mol
FREEH entropy = 0.38620 kJ/mol/K

$vibrational spectrum

# mode	symmetry	wave number	IR intensity	selection rules	
#		cm**(-1)	km/mol	IR	RAMAN
1		-0.00	0.00000	-	-
2		-0.00	0.00000	-	-
3		0.00	0.00000	-	-
4		0.00	0.00000	-	-
5		0.00	0.00000	-	-
6		0.00	0.00000	-	-
7	a"	12.87	0.02318	YES	YES
8	a'	124.90	0.00782	YES	YES
9	a"	193.18	0.39717	YES	YES

10	a'	308.73	1.26777	YES	YES
11	a'	332.25	2.60556	YES	YES
12	a"	379.78	1.09550	YES	YES
13	a"	396.08	0.02203	YES	YES
14	a'	467.23	1.09931	YES	YES
15	a"	550.54	0.24497	YES	YES
16	a'	575.86	6.05153	YES	YES
17	a"	614.45	0.13361	YES	YES
18	a'	642.29	15.36338	YES	YES
19	a'	689.87	43.57840	YES	YES
20	a'	748.09	0.48491	YES	YES
21	a'	761.06	42.25706	YES	YES
22	a"	833.35	0.15703	YES	YES
23	a'	915.26	9.33371	YES	YES
24	a"	957.63	0.12081	YES	YES
25	a'	976.03	0.53357	YES	YES
26	a'	996.66	4.95089	YES	YES
27	a'	1020.86	47.73945	YES	YES
28	a'	1051.16	85.85488	YES	YES
29	a"	1061.17	99.37602	YES	YES
30	a'	1065.36	228.14582	YES	YES
31	a"	1118.57	112.95958	YES	YES
32	a"	1155.09	0.00004	YES	YES
33	a'	1172.90	4.63586	YES	YES
34	a'	1279.42	304.47713	YES	YES
35	a"	1310.44	2.52929	YES	YES
36	a"	1346.62	0.00110	YES	YES
37	a"	1443.93	15.18811	YES	YES
38	a'	1488.03	0.00372	YES	YES
39	a"	1587.47	0.06439	YES	YES
40	a'	1603.67	4.02029	YES	YES
41	a'	3104.04	0.15539	YES	YES
42	a"	3114.81	6.27986	YES	YES
43	a'	3123.57	11.16749	YES	YES
44	a"	3133.52	7.60687	YES	YES
45	a'	3135.72	2.32210	YES	YES

$end

Double hybrid single point energy = -568.722549723201 H
COSMO energy + OC correction = -569.5796058811 H (in *o*DFB)

6.2.49 [F{Ga(dcpe)}$_2$(CF$_2$Ph)]$^{2+}$

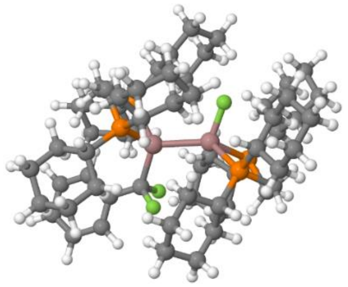

Method: (RI-)BP86(D3BJ)/def2-TZVPP
Symmetry: c1

Cartesian coordinates in Ångström:

H	10.6218165	12.1116328	3.4682765
H	11.7929127	10.8954102	3.9793082
C	10.9192142	11.0587691	3.3336167
H	11.9422566	8.0090661	7.9496918
H	12.0781181	11.5027162	1.5539175
H	9.5084396	10.4101711	4.8320678
C	11.2820225	10.8139202	1.8667981
C	11.8402865	6.9122121	7.9926040
H	12.7434915	6.5341049	8.4897926
C	9.7730406	10.1476299	3.7987592
H	10.6425195	6.9758191	9.8087555
H	9.3517049	8.1418591	8.0541656
H	9.7017317	12.0237937	1.0082324
H	8.6185321	9.1820895	6.0746129
H	10.5280343	7.8993386	5.6840835
H	6.8057113	10.6796790	5.2833554
H	12.6218439	6.6457187	5.9780813
C	10.5930681	6.5467245	8.7990382
H	8.1565095	11.3241651	2.9815157
H	11.6753164	9.7905952	1.7624339
C	10.0537085	10.9801462	0.9693772
H	7.2207173	9.0057821	7.1346362
C	11.7460033	6.3528958	6.5719846
C	7.5813285	8.8283238	6.1125804
C	9.3206018	7.0420836	8.0999186
C	8.5498264	10.2950612	2.8837844
H	10.1168593	9.1024774	3.7818768
C	10.4728191	6.8126857	5.8539208
C	6.7106461	9.5978185	5.1116765
H	13.5269085	9.1596818	0.1129849
H	10.3049053	10.7788841	-0.0810135
H	4.8042480	11.1765291	3.8565327
H	14.4504052	9.2941479	-1.3863185
C	8.9180381	10.0500799	1.4104382
H	8.4390770	6.7720458	8.6964755
H	10.5388096	5.4529234	8.9244249
C	9.2194587	6.4734865	6.6693574
H	4.0111300	9.6204809	3.5881581
C	13.6312028	8.7453007	-0.9032442
H	12.0601992	10.0153774	-1.7146069
H	5.6522358	9.3443576	5.2565769
H	11.7504663	5.2504890	6.6264030
H	10.4139150	6.3541705	4.8561785
C	4.5054724	10.4604466	3.0786816
H	15.3361674	6.2493776	3.7196960
P	7.1024794	9.2373001	3.3412115
H	14.4045123	5.7581353	5.1332160
P	7.6582493	7.0056045	5.8223850
C	12.3264088	8.9497280	-1.6736349
H	2.6128666	11.4328724	2.6967283
H	8.0433051	10.1866311	0.7591590
C	14.6851736	5.4557232	4.1113894
H	9.2407491	9.0017969	1.3015219
H	6.7412834	6.1884016	7.8486473
H	10.9661691	8.5664673	-0.0360749
C	3.4979884	11.1210214	2.1257005
H	14.8882590	7.0979276	-0.2356392

H	12.8788510	6.3171126	3.2348065
H	9.1279744	5.3755342	6.7323992
C	5.7349875	9.9685399	2.2956045
C	13.9675807	7.2563499	-0.8142442
H	5.1941362	8.0214023	7.2942952
C	6.3201992	6.2151771	6.8289127
C	11.1687398	8.1675273	-1.0413060
H	16.3084114	4.2014196	4.8247618
H	12.7017311	6.7782280	0.8770063
C	13.4232553	5.3640055	3.2432768
H	12.4597637	8.6224189	-2.7174889
C	15.4310363	4.1209977	4.1692597
H	3.9431259	12.0407155	1.7123922
Ga	9.7227287	6.2259376	2.2650558
C	5.0133592	7.0168699	6.8852882
Ga	7.5764530	6.8363907	3.3712293
C	12.8266180	6.4600127	-0.1681167
H	6.2228730	10.8634540	1.8713506
H	2.5816142	9.3035030	1.3868862
H	10.2629999	8.3115235	-1.6433817
H	4.9259825	8.1120832	1.5063720
C	3.1039222	10.1851956	0.9798097
H	14.1615876	6.8627928	-1.8253385
H	4.3093191	6.2740576	8.7925642
H	13.7322096	5.1652040	2.2049085
C	5.3554928	9.0475742	1.1244290
H	7.0441853	4.2118980	6.3781258
H	15.8128817	3.8694533	3.1658411
H	12.1465609	4.4743144	4.7327124
C	6.0961630	4.7681867	6.3630810
C	11.4957712	6.6702279	-0.9079876
H	14.2010473	3.2022648	5.6954848
C	3.9695227	6.2894259	7.7440489
C	12.5017318	4.2274195	3.7193639
C	14.5074832	3.0019132	4.6554699
H	5.4392944	3.9582082	8.2618567
H	4.6136307	7.1384031	5.8702921
H	2.3947452	10.6878854	0.3091045
H	13.0950667	5.3957401	-0.1667656
C	4.3389490	9.7255796	0.2000380
H	9.1378170	6.0876705	-2.2757110
C	5.0512774	4.0650650	7.2356116
H	6.2578147	8.7686263	0.5646814
P	10.0894041	5.7514407	-0.1170199
H	3.0295565	6.8580257	7.7287994
H	5.7459613	4.7734627	5.3226538
P	10.9509617	4.1279276	2.7121798
H	11.5687775	6.2263668	-1.9180293
H	8.6764267	4.0535586	4.6252344
C	13.2531758	2.8834958	3.7774864
H	10.1618424	3.5684414	5.4644239
H	15.0341745	2.0380119	4.6647589
C	3.7395831	4.8546328	7.2601709
C	8.6646248	5.9299893	-1.2899469
H	4.8086711	10.5944872	-0.2902196
C	9.3639945	3.2080155	4.7976936
H	4.0533891	9.0285812	-0.5999392
H	8.4730295	8.0683177	-0.8641433

H	7.1668407	7.6089614	-2.9423882
H	12.4975497	3.9148084	0.8949702
H	4.8848032	3.0480015	6.8545976
H	13.5540353	2.5871056	2.7596525
H	7.0404131	5.0036285	-3.3865439
H	8.3407306	3.8094164	-1.6934315
H	3.0055153	4.3529655	7.9045861
C	11.4810090	3.5345209	1.0448354
C	7.8357358	7.1783191	-0.9326757
H	12.6074052	2.0849781	4.1666318
H	8.1806125	2.3763723	6.4160522
C	9.9513981	2.7674769	3.4474961
C	7.7619570	4.6867899	-1.3753587
H	3.3094055	4.8755850	6.2459958
C	10.5568540	3.9623356	-0.1059252
C	6.7184028	7.4004044	-1.9572039
C	8.6032838	2.0508684	5.4546365
C	6.6160786	4.9157270	-2.3730688
H	8.1899417	3.0481267	2.2103045
H	11.0108749	3.7049069	-1.0738734
H	6.7403258	2.3011857	4.3900654
H	10.6710841	1.9522277	3.6325542
C	8.8683187	2.2327561	2.4978391
H	6.1406402	8.2951802	-1.6869547
H	11.5532765	2.4383480	1.0729595
C	5.8061249	6.1753372	-2.0522783
H	7.3969634	7.0222609	0.0635122
H	9.6094370	3.4120894	-0.0421950
C	7.5023441	1.5199421	4.5322121
H	9.3144450	1.2426614	5.6901039
H	7.3466270	4.4669178	-0.3793641
H	5.9658051	4.0304289	-2.3839847
H	5.0369205	6.3315340	-2.8205283
H	9.3290501	1.8480392	1.5769235
C	8.0715494	1.1042296	3.1707469
H	6.9971826	0.6649695	5.0012175
H	5.2780958	6.0394005	-1.0955689
H	7.2648761	0.7783248	2.5001724
H	8.7346365	0.2343445	3.3064254
F	11.0502247	7.4693041	2.5274624
C	6.0540757	5.5954319	2.7420869
C	4.6264866	5.6600221	3.1956724
C	4.0742302	6.8755504	3.5985252
H	4.7157695	7.7531736	3.6501348
C	2.7240153	6.9752028	3.9342449
H	2.3046701	7.9323472	4.2435792
C	1.9160916	5.8381589	3.8861928
H	0.8623841	5.9047858	4.1536183
C	2.4644273	4.6106185	3.4987324
H	1.8358196	3.7216700	3.4646774
C	3.8105212	4.5195166	3.1474103
H	4.2323952	3.5657221	2.8361596
F	6.0550873	5.6681215	1.3293899
F	6.5499209	4.3025569	3.0009163

SCF energy GEOOPT = -7825.823699134 H
ZPE = 3873. kJ/mol
FREEH energy = 4073.69 kJ/mol

FREEH entropy =   1.67067 kJ/mol/K

```
$vibrational spectrum
#  mode     symmetry     wave number   IR intensity    selection rules
#                         cm**(-1)        km/mol          IR     RAMAN
     1                      -0.00          0.00000         -       -
     2                      -0.00          0.00000         -       -
     3                      -0.00          0.00000         -       -
     4                       0.00          0.00000         -       -
     5                       0.00          0.00000         -       -
     6                       0.00          0.00000         -       -
     7         a            19.96          0.02023        YES     YES
     8         a            21.18          0.02784        YES     YES
     9         a            24.16          0.21327        YES     YES
    10         a            27.97          0.15694        YES     YES
    11         a            30.20          0.10534        YES     YES
    12         a            32.44          0.01504        YES     YES
    13         a            35.29          0.20832        YES     YES
    14         a            38.17          0.46469        YES     YES
    15         a            43.64          0.31631        YES     YES
    16         a            45.37          0.29239        YES     YES
    17         a            46.53          0.09207        YES     YES
    18         a            48.42          0.41836        YES     YES
    19         a            51.16          0.07086        YES     YES
    20         a            54.44          0.06640        YES     YES
    21         a            54.93          0.37636        YES     YES
    22         a            58.43          0.18580        YES     YES
    23         a            61.66          0.33108        YES     YES
    24         a            62.12          0.22022        YES     YES
    25         a            65.93          0.31718        YES     YES
    26         a            68.73          0.40070        YES     YES
    27         a            70.28          0.24320        YES     YES
    28         a            71.69          0.01533        YES     YES
    29         a            73.98          1.06352        YES     YES
    30         a            76.35          0.26340        YES     YES
    31         a            77.69          0.27796        YES     YES
    32         a            80.29          0.13869        YES     YES
    33         a            84.54          0.17184        YES     YES
    34         a            88.62          0.50805        YES     YES
    35         a            90.65          0.04085        YES     YES
    36         a            94.03          0.03840        YES     YES
    37         a            95.83          0.04033        YES     YES
    38         a            98.21          0.26322        YES     YES
    39         a           105.95          2.14146        YES     YES
    40         a           116.56          1.66424        YES     YES
    41         a           124.72          0.70318        YES     YES
    42         a           132.64          2.51567        YES     YES
    43         a           133.92          0.25727        YES     YES
    44         a           135.98          0.95105        YES     YES
    45         a           138.14          2.57217        YES     YES
    46         a           139.55          1.99600        YES     YES
    47         a           145.95         11.09043        YES     YES
    48         a           147.71         11.88490        YES     YES
    49         a           155.19          2.94868        YES     YES
    50         a           158.98          0.97633        YES     YES
    51         a           162.71          5.43749        YES     YES
    52         a           163.85          3.77627        YES     YES
    53         a           171.13          7.06300        YES     YES
```

54	a	172.11	0.18426	YES	YES
55	a	176.41	7.05075	YES	YES
56	a	182.18	0.32211	YES	YES
57	a	184.48	11.07904	YES	YES
58	a	196.37	1.47196	YES	YES
59	a	206.58	0.65764	YES	YES
60	a	210.20	1.54930	YES	YES
61	a	212.31	1.00270	YES	YES
62	a	216.18	1.22472	YES	YES
63	a	217.50	0.36842	YES	YES
64	a	221.24	0.96629	YES	YES
65	a	224.82	0.10353	YES	YES
66	a	226.81	0.79998	YES	YES
67	a	229.49	1.06268	YES	YES
68	a	231.20	0.35729	YES	YES
69	a	232.33	1.18637	YES	YES
70	a	237.40	1.94311	YES	YES
71	a	238.77	0.35645	YES	YES
72	a	246.86	0.93850	YES	YES
73	a	248.53	0.17612	YES	YES
74	a	250.13	1.73592	YES	YES
75	a	255.21	1.27692	YES	YES
76	a	265.63	10.42489	YES	YES
77	a	268.23	1.08263	YES	YES
78	a	288.51	3.22834	YES	YES
79	a	293.14	0.06925	YES	YES
80	a	294.00	0.76610	YES	YES
81	a	296.96	0.47246	YES	YES
82	a	305.42	0.75175	YES	YES
83	a	307.24	0.01726	YES	YES
84	a	308.91	1.02678	YES	YES
85	a	317.67	0.22316	YES	YES
86	a	324.79	4.86414	YES	YES
87	a	330.22	0.28241	YES	YES
88	a	332.06	1.49063	YES	YES
89	a	335.20	0.56995	YES	YES
90	a	337.79	0.14701	YES	YES
91	a	343.82	0.93069	YES	YES
92	a	345.36	0.34979	YES	YES
93	a	361.86	2.96794	YES	YES
94	a	362.16	1.09391	YES	YES
95	a	376.71	1.47401	YES	YES
96	a	378.71	1.01692	YES	YES
97	a	383.97	6.22287	YES	YES
98	a	388.55	0.20784	YES	YES
99	a	394.72	1.60285	YES	YES
100	a	402.59	0.57406	YES	YES
101	a	412.66	1.72182	YES	YES
102	a	417.57	1.16667	YES	YES
103	a	420.12	3.40600	YES	YES
104	a	423.18	0.96979	YES	YES
105	a	427.33	4.18024	YES	YES
106	a	428.41	0.98607	YES	YES
107	a	428.50	0.08972	YES	YES
108	a	430.51	0.12865	YES	YES
109	a	430.91	0.10126	YES	YES
110	a	431.05	0.42793	YES	YES
111	a	431.92	0.48950	YES	YES

112	a	434.18	3.04486	YES	YES
113	a	434.83	0.66271	YES	YES
114	a	438.30	3.61332	YES	YES
115	a	439.72	0.35973	YES	YES
116	a	441.13	5.01798	YES	YES
117	a	463.05	2.64441	YES	YES
118	a	465.33	4.06584	YES	YES
119	a	490.11	2.44868	YES	YES
120	a	492.45	3.96304	YES	YES
121	a	493.56	4.68638	YES	YES
122	a	496.83	5.31252	YES	YES
123	a	500.59	0.23742	YES	YES
124	a	502.86	1.30560	YES	YES
125	a	512.24	9.46436	YES	YES
126	a	515.02	4.76749	YES	YES
127	a	532.56	2.02783	YES	YES
128	a	542.18	48.29741	YES	YES
129	a	550.76	2.39581	YES	YES
130	a	616.92	0.30295	YES	YES
131	a	630.80	21.01384	YES	YES
132	a	631.53	14.47184	YES	YES
133	a	651.58	10.79893	YES	YES
134	a	653.92	9.38973	YES	YES
135	a	663.70	11.45993	YES	YES
136	a	700.09	32.75912	YES	YES
137	a	706.36	1.06533	YES	YES
138	a	710.57	1.34977	YES	YES
139	a	712.59	1.17774	YES	YES
140	a	719.04	1.78025	YES	YES
141	a	728.68	1.31338	YES	YES
142	a	729.76	1.98607	YES	YES
143	a	734.90	8.05891	YES	YES
144	a	735.29	4.59893	YES	YES
145	a	758.64	57.98659	YES	YES
146	a	771.45	0.09696	YES	YES
147	a	771.62	0.52748	YES	YES
148	a	772.06	0.33064	YES	YES
149	a	773.09	0.13413	YES	YES
150	a	773.19	0.16349	YES	YES
151	a	773.95	0.14421	YES	YES
152	a	776.25	0.37322	YES	YES
153	a	779.22	0.24458	YES	YES
154	a	784.30	17.93154	YES	YES
155	a	786.14	9.50639	YES	YES
156	a	807.09	2.60510	YES	YES
157	a	808.16	0.40248	YES	YES
158	a	810.60	3.66346	YES	YES
159	a	810.94	1.08578	YES	YES
160	a	812.06	1.16280	YES	YES
161	a	812.62	0.62357	YES	YES
162	a	814.13	2.94495	YES	YES
163	a	815.89	4.44690	YES	YES
164	a	834.71	6.77724	YES	YES
165	a	835.66	0.88792	YES	YES
166	a	836.02	0.44044	YES	YES
167	a	837.20	2.95191	YES	YES
168	a	837.59	1.61922	YES	YES
169	a	838.46	0.51259	YES	YES

170	a	838.68	5.70112	YES	YES
171	a	839.83	7.46091	YES	YES
172	a	840.77	7.77272	YES	YES
173	a	851.01	11.55142	YES	YES
174	a	853.22	5.80855	YES	YES
175	a	869.80	82.40759	YES	YES
176	a	873.85	1.78313	YES	YES
177	a	874.23	4.19043	YES	YES
178	a	875.10	4.98542	YES	YES
179	a	876.15	3.34806	YES	YES
180	a	877.48	1.60922	YES	YES
181	a	878.59	4.64180	YES	YES
182	a	879.09	2.84340	YES	YES
183	a	879.69	0.14920	YES	YES
184	a	880.18	9.32778	YES	YES
185	a	881.32	5.13925	YES	YES
186	a	881.50	2.64032	YES	YES
187	a	881.68	10.27362	YES	YES
188	a	884.49	1.46251	YES	YES
189	a	884.95	1.08066	YES	YES
190	a	887.20	2.81344	YES	YES
191	a	889.73	3.21402	YES	YES
192	a	896.90	17.78564	YES	YES
193	a	903.93	1.88751	YES	YES
194	a	904.44	0.28432	YES	YES
195	a	906.71	2.28103	YES	YES
196	a	907.13	2.24892	YES	YES
197	a	908.45	1.02408	YES	YES
198	a	909.09	1.09321	YES	YES
199	a	911.07	0.67166	YES	YES
200	a	912.71	0.92274	YES	YES
201	a	919.39	7.95168	YES	YES
202	a	961.57	9.30839	YES	YES
203	a	967.58	82.76431	YES	YES
204	a	985.49	8.58221	YES	YES
205	a	986.71	2.02721	YES	YES
206	a	987.94	18.61945	YES	YES
207	a	988.32	2.73944	YES	YES
208	a	989.63	8.64232	YES	YES
209	a	990.04	14.99640	YES	YES
210	a	990.35	0.30778	YES	YES
211	a	993.62	6.08660	YES	YES
212	a	995.13	1.78563	YES	YES
213	a	996.18	4.81309	YES	YES
214	a	999.05	1.17497	YES	YES
215	a	1001.60	4.04964	YES	YES
216	a	1017.01	1.20353	YES	YES
217	a	1017.47	0.47529	YES	YES
218	a	1018.20	0.55424	YES	YES
219	a	1018.93	1.38132	YES	YES
220	a	1019.39	0.63821	YES	YES
221	a	1019.73	0.96426	YES	YES
222	a	1020.29	1.13617	YES	YES
223	a	1024.29	1.04821	YES	YES
224	a	1029.43	0.41155	YES	YES
225	a	1029.68	1.06843	YES	YES
226	a	1030.63	3.92666	YES	YES
227	a	1033.51	1.46312	YES	YES

228	a	1034.80	0.50573	YES	YES
229	a	1037.68	0.86277	YES	YES
230	a	1039.20	0.93092	YES	YES
231	a	1039.86	1.41927	YES	YES
232	a	1040.13	0.38076	YES	YES
233	a	1061.13	1.42585	YES	YES
234	a	1061.97	0.91431	YES	YES
235	a	1063.18	0.25674	YES	YES
236	a	1064.33	0.07441	YES	YES
237	a	1064.68	0.43412	YES	YES
238	a	1066.46	1.26605	YES	YES
239	a	1070.55	0.49723	YES	YES
240	a	1071.83	0.54041	YES	YES
241	a	1072.08	0.00919	YES	YES
242	a	1072.51	0.09931	YES	YES
243	a	1073.18	0.12827	YES	YES
244	a	1073.82	0.17956	YES	YES
245	a	1074.18	0.50610	YES	YES
246	a	1074.49	0.09198	YES	YES
247	a	1074.56	0.62985	YES	YES
248	a	1076.39	0.11537	YES	YES
249	a	1082.88	0.61601	YES	YES
250	a	1086.93	0.71109	YES	YES
251	a	1088.56	5.07823	YES	YES
252	a	1095.52	0.25491	YES	YES
253	a	1096.27	5.93709	YES	YES
254	a	1098.89	2.14037	YES	YES
255	a	1099.94	12.68094	YES	YES
256	a	1105.52	5.11860	YES	YES
257	a	1105.80	0.99917	YES	YES
258	a	1107.43	2.05824	YES	YES
259	a	1108.27	1.66868	YES	YES
260	a	1130.87	1.31912	YES	YES
261	a	1134.35	3.20560	YES	YES
262	a	1156.54	0.59480	YES	YES
263	a	1160.85	7.34982	YES	YES
264	a	1162.22	4.61928	YES	YES
265	a	1164.57	2.18653	YES	YES
266	a	1165.89	0.85791	YES	YES
267	a	1168.17	2.99878	YES	YES
268	a	1169.28	4.52612	YES	YES
269	a	1169.78	11.66695	YES	YES
270	a	1171.26	3.06966	YES	YES
271	a	1171.60	7.15709	YES	YES
272	a	1178.71	9.26095	YES	YES
273	a	1184.78	12.50544	YES	YES
274	a	1185.10	12.27089	YES	YES
275	a	1186.60	0.44965	YES	YES
276	a	1187.57	15.90778	YES	YES
277	a	1189.59	6.67561	YES	YES
278	a	1193.35	17.76332	YES	YES
279	a	1201.59	14.37314	YES	YES
280	a	1220.04	59.46689	YES	YES
281	a	1243.63	0.36846	YES	YES
282	a	1245.13	0.73109	YES	YES
283	a	1246.45	0.28104	YES	YES
284	a	1246.93	0.27194	YES	YES
285	a	1249.12	0.47102	YES	YES

286	a	1250.52	2.97498	YES	YES
287	a	1250.92	1.22552	YES	YES
288	a	1251.22	1.23384	YES	YES
289	a	1252.10	2.14398	YES	YES
290	a	1252.95	5.48571	YES	YES
291	a	1254.12	1.20702	YES	YES
292	a	1255.34	1.51079	YES	YES
293	a	1256.71	5.56689	YES	YES
294	a	1257.04	15.72258	YES	YES
295	a	1257.55	5.28365	YES	YES
296	a	1258.18	1.94187	YES	YES
297	a	1258.67	7.44947	YES	YES
298	a	1259.69	3.33734	YES	YES
299	a	1259.85	5.89458	YES	YES
300	a	1261.07	8.09957	YES	YES
301	a	1262.75	5.27787	YES	YES
302	a	1264.13	0.10678	YES	YES
303	a	1266.82	1.07145	YES	YES
304	a	1267.75	1.92140	YES	YES
305	a	1270.64	0.36953	YES	YES
306	a	1272.50	2.41391	YES	YES
307	a	1272.57	1.01967	YES	YES
308	a	1273.60	0.47877	YES	YES
309	a	1284.13	3.82581	YES	YES
310	a	1286.51	0.58787	YES	YES
311	a	1287.44	0.95694	YES	YES
312	a	1287.98	3.40272	YES	YES
313	a	1290.45	2.39948	YES	YES
314	a	1292.03	3.34813	YES	YES
315	a	1292.46	2.50074	YES	YES
316	a	1293.94	2.66959	YES	YES
317	a	1302.96	5.90392	YES	YES
318	a	1310.98	1.53511	YES	YES
319	a	1313.02	1.53558	YES	YES
320	a	1313.11	0.36068	YES	YES
321	a	1313.93	1.19548	YES	YES
322	a	1314.85	1.06380	YES	YES
323	a	1316.46	2.72889	YES	YES
324	a	1317.43	2.75396	YES	YES
325	a	1319.03	0.43767	YES	YES
326	a	1322.48	0.84104	YES	YES
327	a	1323.29	2.22040	YES	YES
328	a	1323.65	0.12471	YES	YES
329	a	1324.14	0.94206	YES	YES
330	a	1324.37	0.45820	YES	YES
331	a	1324.63	1.21319	YES	YES
332	a	1325.92	0.42063	YES	YES
333	a	1326.38	1.25867	YES	YES
334	a	1326.99	1.71797	YES	YES
335	a	1327.28	0.77718	YES	YES
336	a	1328.65	2.66857	YES	YES
337	a	1328.78	0.99610	YES	YES
338	a	1329.32	0.93016	YES	YES
339	a	1330.04	8.99639	YES	YES
340	a	1332.13	1.08473	YES	YES
341	a	1334.18	1.07425	YES	YES
342	a	1336.34	0.17405	YES	YES
343	a	1338.86	0.09910	YES	YES

344	a	1339.06	0.04797	YES	YES
345	a	1339.27	0.11939	YES	YES
346	a	1339.33	0.63557	YES	YES
347	a	1340.68	1.29987	YES	YES
348	a	1341.16	0.49943	YES	YES
349	a	1344.69	0.64215	YES	YES
350	a	1345.50	1.34673	YES	YES
351	a	1346.43	0.49219	YES	YES
352	a	1346.86	1.47402	YES	YES
353	a	1347.45	0.26297	YES	YES
354	a	1347.90	0.59817	YES	YES
355	a	1349.49	0.21604	YES	YES
356	a	1349.90	0.73973	YES	YES
357	a	1350.83	0.45909	YES	YES
358	a	1352.88	0.08670	YES	YES
359	a	1401.34	11.91138	YES	YES
360	a	1404.73	9.10457	YES	YES
361	a	1404.94	5.93526	YES	YES
362	a	1410.10	7.28904	YES	YES
363	a	1419.92	6.39259	YES	YES
364	a	1422.73	2.95018	YES	YES
365	a	1429.97	2.98655	YES	YES
366	a	1431.53	1.23841	YES	YES
367	a	1432.56	1.44624	YES	YES
368	a	1433.04	1.21014	YES	YES
369	a	1434.83	2.48100	YES	YES
370	a	1435.15	4.65592	YES	YES
371	a	1435.60	1.93704	YES	YES
372	a	1436.79	7.43168	YES	YES
373	a	1437.10	5.56699	YES	YES
374	a	1437.38	3.66717	YES	YES
375	a	1437.67	2.64438	YES	YES
376	a	1437.94	8.85756	YES	YES
377	a	1438.17	6.88349	YES	YES
378	a	1438.46	7.11981	YES	YES
379	a	1438.67	2.13570	YES	YES
380	a	1438.94	3.12669	YES	YES
381	a	1439.17	1.54314	YES	YES
382	a	1439.62	4.08911	YES	YES
383	a	1440.26	4.71428	YES	YES
384	a	1441.84	0.77099	YES	YES
385	a	1442.31	8.05496	YES	YES
386	a	1442.49	14.27439	YES	YES
387	a	1442.97	24.21906	YES	YES
388	a	1443.92	8.88301	YES	YES
389	a	1444.42	31.56291	YES	YES
390	a	1444.70	9.49763	YES	YES
391	a	1444.91	9.51986	YES	YES
392	a	1445.74	29.01911	YES	YES
393	a	1446.33	11.00349	YES	YES
394	a	1447.18	11.56143	YES	YES
395	a	1448.91	9.59701	YES	YES
396	a	1453.77	0.27132	YES	YES
397	a	1454.16	1.97062	YES	YES
398	a	1454.99	1.94122	YES	YES
399	a	1455.61	1.39426	YES	YES
400	a	1455.92	1.16415	YES	YES
401	a	1457.59	0.27567	YES	YES

402	a	1458.73	0.56428	YES	YES
403	a	1461.97	0.99090	YES	YES
404	a	1484.11	5.08500	YES	YES
405	a	1579.28	3.68132	YES	YES
406	a	1600.19	0.69459	YES	YES
407	a	2916.42	6.26900	YES	YES
408	a	2921.53	5.80532	YES	YES
409	a	2930.23	5.39184	YES	YES
410	a	2931.03	14.68870	YES	YES
411	a	2934.43	24.34805	YES	YES
412	a	2935.73	3.65902	YES	YES
413	a	2942.09	4.25525	YES	YES
414	a	2944.39	9.12798	YES	YES
415	a	2944.63	7.40503	YES	YES
416	a	2946.60	1.22474	YES	YES
417	a	2947.35	14.55492	YES	YES
418	a	2948.39	10.09445	YES	YES
419	a	2949.48	12.51999	YES	YES
420	a	2949.53	9.06485	YES	YES
421	a	2950.12	14.75979	YES	YES
422	a	2950.47	28.77868	YES	YES
423	a	2951.28	22.02048	YES	YES
424	a	2951.47	17.45057	YES	YES
425	a	2952.00	10.55388	YES	YES
426	a	2952.38	10.64340	YES	YES
427	a	2952.89	12.98691	YES	YES
428	a	2953.75	4.88542	YES	YES
429	a	2954.14	21.51476	YES	YES
430	a	2954.20	10.68850	YES	YES
431	a	2954.98	11.61924	YES	YES
432	a	2956.99	17.28655	YES	YES
433	a	2958.85	13.05062	YES	YES
434	a	2959.16	14.04582	YES	YES
435	a	2960.04	13.26468	YES	YES
436	a	2960.13	27.32202	YES	YES
437	a	2961.01	32.32756	YES	YES
438	a	2962.68	37.28332	YES	YES
439	a	2965.60	11.47064	YES	YES
440	a	2965.92	23.96285	YES	YES
441	a	2966.52	11.48127	YES	YES
442	a	2966.60	19.83569	YES	YES
443	a	2968.12	15.00274	YES	YES
444	a	2968.77	17.43134	YES	YES
445	a	2972.77	24.00161	YES	YES
446	a	2973.73	13.40606	YES	YES
447	a	2973.84	8.46544	YES	YES
448	a	2974.02	4.89759	YES	YES
449	a	2975.04	20.38527	YES	YES
450	a	2975.44	21.76460	YES	YES
451	a	2977.09	11.90684	YES	YES
452	a	2981.53	3.11857	YES	YES
453	a	2981.80	15.93722	YES	YES
454	a	2982.10	1.77942	YES	YES
455	a	2984.56	15.91794	YES	YES
456	a	2989.47	23.81538	YES	YES
457	a	2992.13	4.53885	YES	YES
458	a	2992.23	8.11560	YES	YES
459	a	2994.71	3.07570	YES	YES

460	a	2997.54	40.66999	YES	YES
461	a	3000.44	42.63464	YES	YES
462	a	3004.13	28.64358	YES	YES
463	a	3005.25	16.50109	YES	YES
464	a	3007.94	18.93529	YES	YES
465	a	3008.39	23.82699	YES	YES
466	a	3008.63	4.42454	YES	YES
467	a	3009.15	30.87450	YES	YES
468	a	3009.33	48.40169	YES	YES
469	a	3010.11	13.91074	YES	YES
470	a	3010.68	22.29604	YES	YES
471	a	3010.94	28.08929	YES	YES
472	a	3011.31	23.13389	YES	YES
473	a	3011.52	10.18581	YES	YES
474	a	3011.77	9.97046	YES	YES
475	a	3012.15	23.51934	YES	YES
476	a	3014.24	44.10720	YES	YES
477	a	3014.45	11.69304	YES	YES
478	a	3015.70	12.59335	YES	YES
479	a	3015.90	7.77179	YES	YES
480	a	3016.59	20.41196	YES	YES
481	a	3017.43	27.36356	YES	YES
482	a	3017.53	9.15752	YES	YES
483	a	3018.00	22.14882	YES	YES
484	a	3018.18	34.46607	YES	YES
485	a	3019.10	25.38566	YES	YES
486	a	3019.46	33.21120	YES	YES
487	a	3019.55	27.90664	YES	YES
488	a	3020.10	22.52043	YES	YES
489	a	3020.25	23.89822	YES	YES
490	a	3021.06	28.75777	YES	YES
491	a	3025.31	23.38345	YES	YES
492	a	3025.96	6.80841	YES	YES
493	a	3027.28	9.19989	YES	YES
494	a	3029.14	16.88608	YES	YES
495	a	3029.63	2.64335	YES	YES
496	a	3031.97	1.04749	YES	YES
497	a	3034.17	5.38219	YES	YES
498	a	3034.39	11.74576	YES	YES
499	a	3036.01	10.78933	YES	YES
500	a	3036.25	6.92957	YES	YES
501	a	3048.72	0.64949	YES	YES
502	a	3053.52	0.60454	YES	YES
503	a	3106.23	3.16567	YES	YES
504	a	3117.31	3.05842	YES	YES
505	a	3120.54	10.24956	YES	YES
506	a	3131.25	1.79720	YES	YES
507	a	3138.09	2.07155	YES	YES

$end

Double hybrid single point energy = -7817.865624002791 H
COSMO energy + OC correction = -7825.9709651389 H (in *o*DFB)

6.2.50 $C_5H_6F_2$

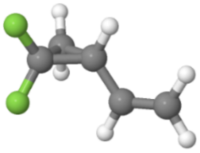

```
Method: (RI-)BP86(D3BJ)/def2-TZVPP
Symmetry: c1

Cartesian coordinates in Ångström:
C     0.0630981    0.0484875    0.4454405
C     1.2402205    0.9378405    0.2018589
C     1.5220016   -0.5062340    0.2863503
F     1.3644021    1.5772606   -0.9950855
F     1.6570946    1.7667589    1.2009047
H     1.7437404   -1.0297111   -0.6420663
H     2.0083795   -0.8862641    1.1820349
H    -0.3081269    0.0603374    1.4711462
C    -0.9365941   -0.2041923   -0.6058872
C    -2.2277632   -0.4600502   -0.3694101
H    -0.5716341   -0.1691482   -1.6358137
H    -2.6275409   -0.4969885    0.6450966
H    -2.9272778   -0.6380965   -1.1845694

SCF energy GEOOPT =  -393.9597861465 H
ZPE =   250.5  kJ/mol
FREEH energy =   268.79 kJ/mol
FREEH entropy =   0.34387 kJ/mol/K

$vibrational spectrum
#  mode     symmetry     wave number   IR intensity    selection rules
#                         cm**(-1)        km/mol         IR      RAMAN
     1                      -0.00         0.00000         -        -
     2                       0.00         0.00000         -        -
     3                       0.00         0.00000         -        -
     4                       0.00         0.00000         -        -
     5                       0.00         0.00000         -        -
     6                       0.00         0.00000         -        -
     7         a            95.73         0.68681        YES      YES
     8         a           156.29         0.83744        YES      YES
     9         a           200.76         0.95789        YES      YES
    10         a           348.65         0.72032        YES      YES
    11         a           375.11         4.05481        YES      YES
    12         a           407.28         0.24746        YES      YES
    13         a           457.85         4.34406        YES      YES
    14         a           555.59         8.26735        YES      YES
    15         a           650.23         0.17736        YES      YES
    16         a           715.28        14.51155        YES      YES
    17         a           805.32        12.62452        YES      YES
    18         a           876.62        22.32296        YES      YES
    19         a           897.22        32.04832        YES      YES
    20         a           913.46        30.16554        YES      YES
    21         a           954.27        68.15588        YES      YES
    22         a           985.43        12.77619        YES      YES
    23         a           993.99        11.26182        YES      YES
    24         a          1012.55        39.45598        YES      YES
    25         a          1071.16        38.33327        YES      YES
```

26	a	1170.62	57.54495	YES	YES
27	a	1190.01	54.65603	YES	YES
28	a	1261.03	61.02189	YES	YES
29	a	1293.06	12.53054	YES	YES
30	a	1345.76	68.56078	YES	YES
31	a	1415.67	17.96741	YES	YES
32	a	1441.68	93.73265	YES	YES
33	a	1650.90	28.75189	YES	YES
34	a	3069.26	6.64961	YES	YES
35	a	3073.63	3.58023	YES	YES
36	a	3076.13	0.72833	YES	YES
37	a	3091.71	4.81359	YES	YES
38	a	3159.42	10.32249	YES	YES
39	a	3171.36	0.62730	YES	YES

$end

Double hybrid single point energy = -393.357223642735 H
COSMO energy + OC correction = -393.9649753282 H (in *o*DFB)

6.2.51 [F{Ga(dcpe)}$_2$(C_5H_6F)]$^{2+}$

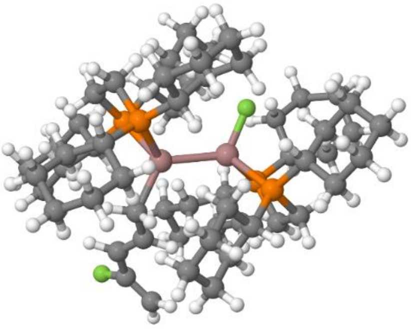

Method: (RI-)BP86(D3BJ)/def2-TZVPP
Symmetry: c1

Cartesian coordinates in Ångström:

C	4.6080890	5.2873209	1.4044631
C	4.2712120	4.8593627	0.0552994
C	4.7071632	3.8019664	-0.6388574
F	3.3641013	5.7017952	-0.5317675
H	5.3682321	3.0783765	-0.1699037
H	4.3653469	3.6183468	-1.6539730
H	3.8416895	5.8782628	1.9095465
C	5.7672542	5.0037497	2.0285078
C	6.1360580	5.3894983	3.4021654
H	6.5113400	4.4282188	1.4709201
H	5.2763774	5.7753166	3.9600855
H	6.5196521	4.5201962	3.9549043
H	9.8433068	12.4135336	2.8109565
H	11.2775784	11.5637879	3.3875931
C	10.3710954	11.4464409	2.7784336
H	12.0703967	8.3695315	8.3057495
H	11.3364484	11.9231133	0.8944828
H	9.1964702	10.6781892	4.4161231
C	10.7421843	11.1099201	1.3322127
C	12.0922601	7.2962658	8.0555895
H	13.0525116	6.9118175	8.4251297
C	9.4738052	10.3669061	3.4003583

H	10.9774802	6.7586387	9.8456065
H	9.4064970	8.1018718	8.4687191
H	8.9150557	11.7784859	0.3755515
H	8.6099949	9.5107264	5.9233135
H	10.5400424	8.6858069	6.2171635
H	6.6870797	10.7153054	4.9616940
H	12.8022892	7.6878055	6.0308022
C	10.9334527	6.5860063	8.7618930
H	7.6794819	11.1293215	2.4980803
H	11.3791787	10.2132365	1.3319656
C	9.4976891	10.8485816	0.4778540
H	7.2525213	9.3192080	7.0344103
C	11.9991649	7.1344846	6.5360018
C	7.6162197	9.0604670	6.0300380
C	9.5655470	7.0381458	8.2276546
C	8.2196311	10.1671096	2.5357744
H	10.0290840	9.4187113	3.4761537
C	10.6407241	7.6085713	6.0083533
C	6.6537213	9.6165054	4.9638500
H	14.5718612	8.1446025	0.3322543
H	9.7819585	10.5540419	-0.5426450
H	5.9841591	11.0583244	1.2526922
H	15.1673437	8.5176835	-1.2864121
C	8.6030477	9.7708876	1.1025163
H	8.7716483	6.4721007	8.7327743
H	11.0266616	5.4966288	8.6156963
C	9.5024034	6.8468419	6.7031438
H	4.9788610	11.2732600	2.6923735
C	14.3335353	8.0566910	-0.7406852
H	13.1046696	9.8533953	-0.7170534
H	5.6222891	9.3274913	5.1987817
H	12.1345903	6.0716650	6.2693664
H	10.6011025	7.4942520	4.9174052
C	5.1355061	10.6358916	1.8064950
H	15.4161161	5.7323821	3.7305814
P	6.9849198	9.0013506	3.2509943
H	14.3675498	5.4681030	5.1236874
P	7.8642029	7.2312781	5.9387541
C	13.0289392	8.8032521	-1.0313997
H	3.6699041	11.7061389	0.6282329
H	7.7088795	9.6085616	0.4832871
C	14.6671224	5.0469200	4.1500889
H	9.1480948	8.8118562	1.1392916
H	6.8212014	6.8741894	8.0496478
H	11.9793383	8.2095156	0.7742119
C	3.8825720	10.6683899	0.9184145
H	15.1365269	6.0368334	-0.8689717
H	13.0089303	6.0042275	3.1013975
H	9.6329742	5.7720731	6.4821499
C	5.4324483	9.1956824	2.2581949
C	14.2120990	6.5770367	-1.1152013
H	5.0690414	8.0314854	6.8036529
C	6.5843185	6.4884975	7.0430537
C	11.8457194	8.1434372	-0.3159201
H	16.1151028	3.6970319	5.0454685
H	13.2097094	5.9154103	0.6829423
C	13.4429464	5.0029596	3.2267674
H	12.8425523	8.8131256	-2.1176974
C	15.2641665	3.6517623	4.3528379
H	4.0948884	10.1186130	-0.0134444
Ga	9.8490825	6.2806103	2.4804616
C	5.1559401	6.9412748	6.6950033

Ga	7.6789329	6.7104221	3.5633549
C	13.0285935	5.9077468	-0.4017369
H	5.6244379	8.5790161	1.3620721
H	2.3863110	10.6593772	2.4817042
H	10.9168027	8.6780794	-0.5591205
H	4.0048398	9.1813429	3.8919963
C	2.6709451	10.0423135	1.6136223
H	14.0760244	6.4828580	-2.2048695
H	4.2578697	6.6035374	8.6329697
H	13.7680447	4.6528205	2.2338809
C	4.2190139	8.5919252	2.9864402
H	7.7398785	4.6774199	7.3912233
H	15.6629721	3.2828376	3.3933996
H	12.0641140	4.4097277	4.7616090
C	6.7297754	4.9577790	7.0592846
C	11.7248867	6.6587120	-0.6965318
H	13.8958067	2.9744807	5.8873583
C	4.1228356	6.2530097	7.5970159
C	12.3895765	4.0270152	3.7770521
C	14.2119099	2.6699613	4.8757320
H	5.8621343	4.5827862	9.0053731
H	4.9337366	6.7056490	5.6432430
H	1.8063726	10.0390806	0.9368806
H	12.9543502	4.8593582	-0.7241139
C	2.9808899	8.6176940	2.0812297
H	9.1395564	7.6213641	-1.1535088
C	5.6759203	4.3002669	7.9567142
H	4.4383910	7.5676301	3.3160301
P	10.2041255	5.9541732	0.0886463
H	3.1129893	6.5602299	7.2929406
H	6.6151538	4.5753224	6.0335977
P	10.8307500	4.0640838	2.7887821
H	11.5201590	6.5812288	-1.7796924
H	8.7905305	4.1328179	4.9518230
C	12.9752028	2.6172051	3.9659138
H	10.2398226	3.3375734	5.5809553
H	14.6356564	1.6611763	4.9718071
C	4.2623769	4.7289204	7.5549394
C	8.8508091	6.5647533	-1.0060785
H	3.1570629	7.9738563	1.2046691
C	9.3397099	3.1747929	4.9708584
H	2.1230334	8.1913730	2.6195716
H	7.5172468	7.0002977	0.6586787
H	6.7231796	8.3259835	-1.3005097
H	12.2373960	3.7126559	0.8715665
H	5.7812490	3.2077033	7.9038138
H	13.2586799	2.2069286	2.9826361
H	8.1123212	7.6174597	-3.4801854
H	9.7828738	5.8928659	-2.8726698
H	3.5209843	4.2629914	8.2171137
C	11.1854144	3.4759972	1.0694539
C	7.4721372	6.5426346	-0.3414476
H	12.2317076	1.9328676	4.3968220
H	8.1849680	2.3820569	6.6248341
C	9.7192026	2.7963552	3.5307670
C	8.7974726	5.8849059	-2.3853460
H	4.0444018	4.3664982	6.5360128
C	10.2829933	4.1102208	-0.0119545
C	6.4441855	7.2612103	-1.2190432
C	8.4642023	2.0867701	5.6042639
C	7.7642416	6.5915425	-3.2761434
H	7.9131785	3.4895458	2.5762826

```
H    10.6293339    3.8119970   -1.0111273
H     6.5520005    2.6864245    4.7874458
H    10.3132512    1.8668824    3.5549470
C     8.4695713    2.5454694    2.6761355
H     5.4561234    7.2254017   -0.7405011
H    11.0932605    2.3814298    1.0388092
C     6.3820583    6.6357261   -2.6160060
H     7.1501080    5.5002042   -0.2017312
H     9.2482626    3.7511718    0.0867015
C     7.2150837    1.8057239    4.7645977
H     9.0584510    1.1641310    5.7008339
H     8.5082621    4.8295599   -2.2563795
H     7.7090851    6.0820145   -4.2475192
H     5.6762757    7.1888774   -3.2494697
H     8.7512431    2.2295598    1.6617322
C     7.5721885    1.4747530    3.3120783
H     6.6393573    0.9815073    5.2060863
H     5.9863249    5.6117629   -2.5248587
H     6.6604340    1.3547447    2.7110657
H     8.0980473    0.5072810    3.2778261
F    11.2356352    7.4193067    2.8648808

SCF energy GEOOPT =  -7650.249603044 H
ZPE =   3853. kJ/mol
FREEH energy =  4051.07 kJ/mol
FREEH entropy =   1.66502 kJ/mol/K

$vibrational spectrum
#  mode     symmetry     wave number   IR intensity    selection rules
#                         cm**(-1)        km/mol         IR     RAMAN
     1                      -0.00          0.00000        -       -
     2                      -0.00          0.00000        -       -
     3                       0.00          0.00000        -       -
     4                       0.00          0.00000        -       -
     5                       0.00          0.00000        -       -
     6                       0.00          0.00000        -       -
     7         a            12.02          0.02844       YES     YES
     8         a            19.62          0.05988       YES     YES
     9         a            24.17          0.07743       YES     YES
    10         a            25.20          0.07423       YES     YES
    11         a            27.90          0.12297       YES     YES
    12         a            28.91          0.51728       YES     YES
    13         a            33.79          0.36760       YES     YES
    14         a            35.76          0.15572       YES     YES
    15         a            39.73          0.17464       YES     YES
    16         a            43.49          0.35141       YES     YES
    17         a            45.55          0.05829       YES     YES
    18         a            46.38          0.07758       YES     YES
    19         a            48.15          0.00820       YES     YES
    20         a            49.98          0.28106       YES     YES
    21         a            53.62          0.27630       YES     YES
    22         a            55.56          0.28718       YES     YES
    23         a            59.29          0.12628       YES     YES
    24         a            63.47          0.47769       YES     YES
    25         a            64.08          0.44776       YES     YES
    26         a            67.36          0.22219       YES     YES
    27         a            68.57          0.20645       YES     YES
    28         a            69.86          0.31632       YES     YES
    29         a            73.74          0.26816       YES     YES
    30         a            77.88          0.02525       YES     YES
    31         a            78.63          0.10270       YES     YES
    32         a            81.76          0.34186       YES     YES
```

33	a	82.40	0.25033	YES	YES
34	a	89.51	0.17921	YES	YES
35	a	90.11	0.43953	YES	YES
36	a	91.56	0.01467	YES	YES
37	a	93.85	0.46626	YES	YES
38	a	97.33	0.38513	YES	YES
39	a	111.79	0.37989	YES	YES
40	a	122.89	0.89179	YES	YES
41	a	130.67	0.18615	YES	YES
42	a	134.84	0.14664	YES	YES
43	a	137.65	0.34757	YES	YES
44	a	140.12	7.14779	YES	YES
45	a	143.27	6.85818	YES	YES
46	a	144.93	7.66518	YES	YES
47	a	147.24	2.86560	YES	YES
48	a	150.25	3.50589	YES	YES
49	a	156.55	0.19789	YES	YES
50	a	159.03	1.68978	YES	YES
51	a	164.13	2.18239	YES	YES
52	a	166.42	12.28945	YES	YES
53	a	170.28	5.51077	YES	YES
54	a	180.36	2.38014	YES	YES
55	a	184.54	1.12332	YES	YES
56	a	188.32	4.24404	YES	YES
57	a	190.52	1.43057	YES	YES
58	a	193.84	3.06661	YES	YES
59	a	201.56	2.85233	YES	YES
60	a	202.68	1.31977	YES	YES
61	a	211.15	5.05122	YES	YES
62	a	212.99	0.75648	YES	YES
63	a	215.54	0.12793	YES	YES
64	a	216.53	1.07504	YES	YES
65	a	219.12	0.35281	YES	YES
66	a	227.96	1.49246	YES	YES
67	a	232.64	0.15231	YES	YES
68	a	234.33	1.04272	YES	YES
69	a	238.47	0.29042	YES	YES
70	a	240.29	1.24877	YES	YES
71	a	241.45	0.74015	YES	YES
72	a	244.30	0.09988	YES	YES
73	a	247.94	0.17804	YES	YES
74	a	253.32	0.53177	YES	YES
75	a	257.29	1.45338	YES	YES
76	a	265.39	12.29263	YES	YES
77	a	278.83	1.39344	YES	YES
78	a	279.30	0.36444	YES	YES
79	a	287.95	0.89642	YES	YES
80	a	291.78	1.43839	YES	YES
81	a	303.41	0.68434	YES	YES
82	a	304.24	0.73778	YES	YES
83	a	312.24	0.51046	YES	YES
84	a	314.79	0.16021	YES	YES
85	a	328.76	0.65496	YES	YES
86	a	330.31	1.36792	YES	YES
87	a	335.48	0.65370	YES	YES
88	a	336.20	0.00701	YES	YES
89	a	341.39	2.10462	YES	YES
90	a	343.85	0.06420	YES	YES
91	a	359.34	1.03740	YES	YES
92	a	374.57	1.86555	YES	YES
93	a	375.49	0.20783	YES	YES
94	a	380.82	0.63341	YES	YES

95	a	383.52	0.87641	YES	YES
96	a	395.10	0.31147	YES	YES
97	a	396.69	0.32679	YES	YES
98	a	410.86	1.28442	YES	YES
99	a	414.91	1.58545	YES	YES
100	a	421.96	2.20149	YES	YES
101	a	425.59	2.42926	YES	YES
102	a	426.88	0.85070	YES	YES
103	a	429.66	0.09425	YES	YES
104	a	430.41	0.27234	YES	YES
105	a	431.36	0.70643	YES	YES
106	a	431.76	0.08336	YES	YES
107	a	432.00	0.47612	YES	YES
108	a	432.61	0.09398	YES	YES
109	a	434.82	0.76240	YES	YES
110	a	435.42	0.74533	YES	YES
111	a	439.16	1.08415	YES	YES
112	a	448.36	1.39764	YES	YES
113	a	451.00	7.54860	YES	YES
114	a	452.17	1.08865	YES	YES
115	a	468.80	2.09679	YES	YES
116	a	470.99	3.21691	YES	YES
117	a	472.93	4.73280	YES	YES
118	a	490.80	2.79114	YES	YES
119	a	493.29	2.30362	YES	YES
120	a	495.57	1.09031	YES	YES
121	a	496.84	2.91457	YES	YES
122	a	506.11	0.68981	YES	YES
123	a	506.65	0.20996	YES	YES
124	a	518.79	14.10855	YES	YES
125	a	519.49	0.43671	YES	YES
126	a	545.95	49.67543	YES	YES
127	a	553.50	2.72576	YES	YES
128	a	599.72	9.89931	YES	YES
129	a	624.34	19.57719	YES	YES
130	a	625.98	14.12693	YES	YES
131	a	642.68	16.13822	YES	YES
132	a	643.22	1.15793	YES	YES
133	a	692.12	27.94458	YES	YES
134	a	696.53	9.03930	YES	YES
135	a	708.18	0.06592	YES	YES
136	a	714.62	0.48742	YES	YES
137	a	720.01	1.98125	YES	YES
138	a	721.85	2.19388	YES	YES
139	a	731.55	6.35416	YES	YES
140	a	732.94	4.97740	YES	YES
141	a	733.95	6.11371	YES	YES
142	a	738.02	5.82761	YES	YES
143	a	771.90	0.17735	YES	YES
144	a	773.01	0.46019	YES	YES
145	a	773.54	0.60354	YES	YES
146	a	774.61	1.02126	YES	YES
147	a	777.55	0.13948	YES	YES
148	a	779.16	0.16268	YES	YES
149	a	780.57	0.73233	YES	YES
150	a	781.84	0.16351	YES	YES
151	a	783.61	19.06807	YES	YES
152	a	785.44	5.49884	YES	YES
153	a	809.84	0.95886	YES	YES
154	a	810.06	2.00276	YES	YES
155	a	811.64	1.81578	YES	YES
156	a	812.30	1.68422	YES	YES

157	a	813.02	0.71868	YES	YES
158	a	813.49	0.31422	YES	YES
159	a	815.48	6.26166	YES	YES
160	a	816.80	8.12653	YES	YES
161	a	817.29	48.45005	YES	YES
162	a	830.83	10.60165	YES	YES
163	a	832.59	1.04987	YES	YES
164	a	833.25	1.55245	YES	YES
165	a	836.24	3.20087	YES	YES
166	a	836.64	4.06280	YES	YES
167	a	837.59	7.16825	YES	YES
168	a	837.91	0.60088	YES	YES
169	a	838.46	9.79789	YES	YES
170	a	838.89	8.71788	YES	YES
171	a	850.79	16.95102	YES	YES
172	a	852.68	4.59367	YES	YES
173	a	875.30	3.66622	YES	YES
174	a	875.82	4.14325	YES	YES
175	a	877.29	2.18540	YES	YES
176	a	877.89	1.11338	YES	YES
177	a	878.12	4.83708	YES	YES
178	a	878.48	10.00352	YES	YES
179	a	878.78	2.04331	YES	YES
180	a	879.57	4.20074	YES	YES
181	a	880.42	0.69554	YES	YES
182	a	880.95	1.36778	YES	YES
183	a	882.52	3.70347	YES	YES
184	a	884.06	2.21653	YES	YES
185	a	885.01	2.05185	YES	YES
186	a	885.86	3.88346	YES	YES
187	a	888.97	1.20165	YES	YES
188	a	890.28	3.08210	YES	YES
189	a	892.79	41.74373	YES	YES
190	a	903.36	1.83942	YES	YES
191	a	904.41	2.92604	YES	YES
192	a	905.62	1.37332	YES	YES
193	a	906.93	3.45398	YES	YES
194	a	908.54	2.79989	YES	YES
195	a	909.58	2.48188	YES	YES
196	a	911.20	0.52519	YES	YES
197	a	912.52	0.06997	YES	YES
198	a	942.63	1.64654	YES	YES
199	a	955.69	32.04286	YES	YES
200	a	970.99	0.09073	YES	YES
201	a	982.32	0.12866	YES	YES
202	a	987.92	2.60495	YES	YES
203	a	988.58	6.56471	YES	YES
204	a	989.00	11.89114	YES	YES
205	a	989.99	28.29702	YES	YES
206	a	990.66	11.72583	YES	YES
207	a	991.09	11.82776	YES	YES
208	a	992.67	6.79615	YES	YES
209	a	993.72	1.12508	YES	YES
210	a	1013.44	1.01085	YES	YES
211	a	1015.31	1.22856	YES	YES
212	a	1016.54	0.89648	YES	YES
213	a	1017.16	0.84454	YES	YES
214	a	1017.40	1.44272	YES	YES
215	a	1019.96	0.92548	YES	YES
216	a	1020.24	1.15754	YES	YES
217	a	1020.43	0.45271	YES	YES
218	a	1031.92	5.19693	YES	YES

219	a	1032.28	0.17996	YES	YES
220	a	1033.44	1.28044	YES	YES
221	a	1033.66	6.49774	YES	YES
222	a	1036.92	26.45030	YES	YES
223	a	1038.88	1.25835	YES	YES
224	a	1040.23	3.80247	YES	YES
225	a	1041.73	1.33397	YES	YES
226	a	1042.47	3.91554	YES	YES
227	a	1059.12	3.15856	YES	YES
228	a	1060.22	0.71720	YES	YES
229	a	1060.65	2.16950	YES	YES
230	a	1060.79	1.42351	YES	YES
231	a	1064.13	3.96972	YES	YES
232	a	1066.43	1.95896	YES	YES
233	a	1067.18	14.36446	YES	YES
234	a	1068.70	0.06746	YES	YES
235	a	1069.08	1.04981	YES	YES
236	a	1070.61	0.08651	YES	YES
237	a	1070.74	0.07434	YES	YES
238	a	1071.27	0.28905	YES	YES
239	a	1071.41	0.42835	YES	YES
240	a	1073.21	0.26887	YES	YES
241	a	1074.46	0.38335	YES	YES
242	a	1075.68	0.08850	YES	YES
243	a	1076.96	0.45900	YES	YES
244	a	1080.83	2.12153	YES	YES
245	a	1085.69	2.53577	YES	YES
246	a	1095.19	7.51068	YES	YES
247	a	1097.46	2.33434	YES	YES
248	a	1100.30	3.26508	YES	YES
249	a	1101.38	3.53240	YES	YES
250	a	1106.91	4.35333	YES	YES
251	a	1107.76	0.82775	YES	YES
252	a	1108.29	5.28698	YES	YES
253	a	1111.89	5.30189	YES	YES
254	a	1122.24	38.11547	YES	YES
255	a	1126.31	2.29612	YES	YES
256	a	1130.82	3.26073	YES	YES
257	a	1159.81	3.40712	YES	YES
258	a	1160.81	9.17624	YES	YES
259	a	1162.66	8.87383	YES	YES
260	a	1163.18	1.08610	YES	YES
261	a	1170.49	8.68719	YES	YES
262	a	1170.98	3.08931	YES	YES
263	a	1171.50	2.38977	YES	YES
264	a	1173.24	13.40238	YES	YES
265	a	1180.83	18.05658	YES	YES
266	a	1182.81	9.20185	YES	YES
267	a	1185.74	12.99800	YES	YES
268	a	1190.20	1.90109	YES	YES
269	a	1190.37	4.70803	YES	YES
270	a	1191.69	36.51603	YES	YES
271	a	1193.87	0.64794	YES	YES
272	a	1197.79	4.91029	YES	YES
273	a	1200.25	3.57572	YES	YES
274	a	1242.19	0.17324	YES	YES
275	a	1242.69	0.12905	YES	YES
276	a	1242.97	0.35847	YES	YES
277	a	1244.23	0.42950	YES	YES
278	a	1244.89	1.06985	YES	YES
279	a	1245.94	0.50427	YES	YES
280	a	1246.83	0.89970	YES	YES

281	a	1247.59	0.59607	YES	YES
282	a	1248.10	0.23120	YES	YES
283	a	1250.39	3.55819	YES	YES
284	a	1251.92	1.00380	YES	YES
285	a	1254.46	3.05115	YES	YES
286	a	1255.80	2.67534	YES	YES
287	a	1256.35	6.25736	YES	YES
288	a	1257.16	6.64035	YES	YES
289	a	1258.18	4.73826	YES	YES
290	a	1258.42	4.01383	YES	YES
291	a	1258.52	7.57073	YES	YES
292	a	1258.80	1.42402	YES	YES
293	a	1259.80	0.80169	YES	YES
294	a	1260.56	1.83034	YES	YES
295	a	1261.82	0.14899	YES	YES
296	a	1262.47	4.58994	YES	YES
297	a	1263.02	0.93905	YES	YES
298	a	1264.52	2.75045	YES	YES
299	a	1269.00	4.02208	YES	YES
300	a	1269.13	0.07456	YES	YES
301	a	1269.97	1.77400	YES	YES
302	a	1279.11	1.70540	YES	YES
303	a	1282.96	0.93498	YES	YES
304	a	1284.11	1.71257	YES	YES
305	a	1286.28	3.44643	YES	YES
306	a	1288.19	2.36423	YES	YES
307	a	1289.76	2.33921	YES	YES
308	a	1291.83	3.84717	YES	YES
309	a	1292.68	5.86955	YES	YES
310	a	1297.04	0.62383	YES	YES
311	a	1306.31	2.24996	YES	YES
312	a	1307.30	0.25357	YES	YES
313	a	1307.54	1.68378	YES	YES
314	a	1309.41	2.78053	YES	YES
315	a	1311.83	12.74760	YES	YES
316	a	1313.35	11.93775	YES	YES
317	a	1314.33	3.75744	YES	YES
318	a	1315.78	10.91006	YES	YES
319	a	1316.53	0.59541	YES	YES
320	a	1319.37	1.36248	YES	YES
321	a	1320.49	0.37532	YES	YES
322	a	1321.40	2.80855	YES	YES
323	a	1322.58	1.08688	YES	YES
324	a	1322.95	2.74687	YES	YES
325	a	1323.45	0.17500	YES	YES
326	a	1324.00	0.38006	YES	YES
327	a	1324.23	2.08893	YES	YES
328	a	1324.53	0.69971	YES	YES
329	a	1325.48	0.38943	YES	YES
330	a	1326.35	1.91842	YES	YES
331	a	1326.73	0.85604	YES	YES
332	a	1326.82	4.67625	YES	YES
333	a	1327.75	1.50871	YES	YES
334	a	1328.45	1.73304	YES	YES
335	a	1333.38	2.22885	YES	YES
336	a	1335.78	0.09623	YES	YES
337	a	1336.40	0.50452	YES	YES
338	a	1337.40	0.22585	YES	YES
339	a	1338.74	0.81551	YES	YES
340	a	1338.87	0.06380	YES	YES
341	a	1339.51	0.39798	YES	YES
342	a	1339.95	0.58863	YES	YES

343	a	1340.93	1.03203	YES	YES
344	a	1343.13	0.07007	YES	YES
345	a	1346.85	3.25532	YES	YES
346	a	1347.93	4.02812	YES	YES
347	a	1348.71	0.59361	YES	YES
348	a	1348.80	1.31478	YES	YES
349	a	1349.21	0.80625	YES	YES
350	a	1350.49	0.03795	YES	YES
351	a	1351.69	0.54408	YES	YES
352	a	1369.83	3.75693	YES	YES
353	a	1396.73	6.57649	YES	YES
354	a	1400.27	7.47413	YES	YES
355	a	1401.06	7.90017	YES	YES
356	a	1404.41	8.61236	YES	YES
357	a	1405.09	7.74705	YES	YES
358	a	1420.38	4.56207	YES	YES
359	a	1427.77	1.46984	YES	YES
360	a	1429.86	2.84527	YES	YES
361	a	1430.68	2.42949	YES	YES
362	a	1431.37	7.59001	YES	YES
363	a	1434.74	1.34814	YES	YES
364	a	1434.99	5.01307	YES	YES
365	a	1436.07	1.68892	YES	YES
366	a	1436.78	1.50782	YES	YES
367	a	1437.10	0.72180	YES	YES
368	a	1437.45	4.12620	YES	YES
369	a	1437.79	7.73437	YES	YES
370	a	1437.94	4.57422	YES	YES
371	a	1438.04	1.66312	YES	YES
372	a	1438.50	3.28289	YES	YES
373	a	1438.80	6.17878	YES	YES
374	a	1438.89	3.01629	YES	YES
375	a	1439.40	4.91811	YES	YES
376	a	1440.39	0.55421	YES	YES
377	a	1440.85	8.07722	YES	YES
378	a	1441.11	3.36986	YES	YES
379	a	1441.45	6.02004	YES	YES
380	a	1442.00	10.63582	YES	YES
381	a	1442.96	25.03742	YES	YES
382	a	1443.42	16.46464	YES	YES
383	a	1444.04	3.49105	YES	YES
384	a	1445.31	16.12042	YES	YES
385	a	1445.86	21.25124	YES	YES
386	a	1446.19	17.40735	YES	YES
387	a	1447.01	26.58493	YES	YES
388	a	1447.17	6.33259	YES	YES
389	a	1447.47	7.09445	YES	YES
390	a	1453.11	0.29011	YES	YES
391	a	1453.32	2.65548	YES	YES
392	a	1454.46	1.21989	YES	YES
393	a	1456.56	0.77821	YES	YES
394	a	1456.97	3.15046	YES	YES
395	a	1458.40	1.22528	YES	YES
396	a	1459.50	2.91119	YES	YES
397	a	1462.23	7.37402	YES	YES
398	a	1637.42	72.65404	YES	YES
399	a	1649.78	33.76049	YES	YES
400	a	2922.54	4.13181	YES	YES
401	a	2926.44	16.53061	YES	YES
402	a	2926.76	6.25940	YES	YES
403	a	2927.46	2.63631	YES	YES
404	a	2928.06	1.74709	YES	YES

405	a	2929.72	7.35077	YES	YES
406	a	2931.02	24.75051	YES	YES
407	a	2933.44	2.89606	YES	YES
408	a	2935.80	16.23027	YES	YES
409	a	2941.34	19.95874	YES	YES
410	a	2941.73	4.55101	YES	YES
411	a	2942.14	9.20750	YES	YES
412	a	2942.49	4.12740	YES	YES
413	a	2943.88	8.51816	YES	YES
414	a	2945.55	8.26399	YES	YES
415	a	2946.43	7.92611	YES	YES
416	a	2946.88	14.02275	YES	YES
417	a	2947.32	10.10665	YES	YES
418	a	2947.71	3.47569	YES	YES
419	a	2948.85	27.08007	YES	YES
420	a	2950.83	8.70520	YES	YES
421	a	2951.98	13.86471	YES	YES
422	a	2952.81	15.45741	YES	YES
423	a	2953.79	9.89498	YES	YES
424	a	2954.42	7.75598	YES	YES
425	a	2955.06	25.35318	YES	YES
426	a	2955.52	4.36649	YES	YES
427	a	2956.10	1.50322	YES	YES
428	a	2956.30	12.29188	YES	YES
429	a	2957.13	14.25851	YES	YES
430	a	2957.15	7.85164	YES	YES
431	a	2958.14	9.38444	YES	YES
432	a	2959.11	7.00886	YES	YES
433	a	2960.21	8.01920	YES	YES
434	a	2960.67	34.43881	YES	YES
435	a	2961.15	21.26149	YES	YES
436	a	2961.45	67.23545	YES	YES
437	a	2962.49	37.75386	YES	YES
438	a	2964.29	42.62618	YES	YES
439	a	2964.63	6.32462	YES	YES
440	a	2965.06	4.76746	YES	YES
441	a	2965.80	54.48352	YES	YES
442	a	2966.11	14.64602	YES	YES
443	a	2966.61	21.49687	YES	YES
444	a	2968.27	27.27178	YES	YES
445	a	2969.57	10.98726	YES	YES
446	a	2971.98	19.77763	YES	YES
447	a	2977.26	4.23381	YES	YES
448	a	2979.08	3.01680	YES	YES
449	a	2979.92	2.72338	YES	YES
450	a	2986.32	4.22744	YES	YES
451	a	2990.71	3.95553	YES	YES
452	a	2991.33	5.29617	YES	YES
453	a	2995.82	21.04796	YES	YES
454	a	2996.82	24.72266	YES	YES
455	a	2999.85	26.11970	YES	YES
456	a	3000.46	36.07859	YES	YES
457	a	3003.85	19.98177	YES	YES
458	a	3003.86	24.19053	YES	YES
459	a	3008.25	24.65930	YES	YES
460	a	3008.40	18.77258	YES	YES
461	a	3008.53	21.92365	YES	YES
462	a	3010.10	20.25917	YES	YES
463	a	3010.41	8.73021	YES	YES
464	a	3011.37	11.44996	YES	YES
465	a	3011.59	11.07319	YES	YES
466	a	3011.99	5.13840	YES	YES

467	a	3012.05	30.89320	YES	YES
468	a	3012.09	28.08250	YES	YES
469	a	3013.02	20.30990	YES	YES
470	a	3013.12	8.99018	YES	YES
471	a	3013.34	31.98237	YES	YES
472	a	3014.50	26.55582	YES	YES
473	a	3014.73	32.34274	YES	YES
474	a	3014.92	17.87148	YES	YES
475	a	3015.80	26.54429	YES	YES
476	a	3015.88	13.29563	YES	YES
477	a	3016.15	17.58725	YES	YES
478	a	3016.30	38.47670	YES	YES
479	a	3016.47	14.94836	YES	YES
480	a	3016.68	15.17172	YES	YES
481	a	3017.35	13.62849	YES	YES
482	a	3017.90	25.76995	YES	YES
483	a	3017.97	21.40578	YES	YES
484	a	3018.35	14.40793	YES	YES
485	a	3019.39	45.15321	YES	YES
486	a	3019.54	26.04398	YES	YES
487	a	3020.48	30.67400	YES	YES
488	a	3020.80	22.25748	YES	YES
489	a	3021.57	3.28102	YES	YES
490	a	3021.93	19.09253	YES	YES
491	a	3022.22	8.28591	YES	YES
492	a	3024.87	29.37357	YES	YES
493	a	3026.44	0.63393	YES	YES
494	a	3038.66	0.03147	YES	YES
495	a	3047.17	1.54317	YES	YES
496	a	3050.29	0.42850	YES	YES
497	a	3052.96	0.05031	YES	YES
498	a	3064.45	14.33180	YES	YES
499	a	3096.93	5.44369	YES	YES
500	a	3108.50	4.68921	YES	YES
501	a	3202.45	0.68883	YES	YES

$end

Double hybrid single point energy = -7642.543931570848 H
COSMO energy + OC correction = -7650.4002375445 H (in *o*DFB)

6.2.52 FEC

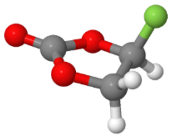

Method: (RI-)BP86(D3BJ)/def2-TZVPP
Symmetry: c1

Cartesian coordinates in Ångström:

O	0.5817457	0.5302533	1.0706794
C	-0.5636870	-0.1845406	1.4087580
O	-0.9499657	-0.3483472	2.5247825
O	-1.1720424	-0.6409267	0.2765452
C	-0.4653763	-0.1532809	-0.8769602
C	0.8490530	0.3745658	-0.2995605
H	-1.0560358	0.6403365	-1.3542362
H	-0.2986483	-0.9799518	-1.5752858

```
H     1.2117472    1.3240592   -0.7097489
F     1.8632095   -0.5621677   -0.4649736

SCF energy GEOOPT =  -441.8789275718 H
ZPE =   169.8 kJ/mol
FREEH energy =   184.48 kJ/mol
FREEH entropy =   0.32062 kJ/mol/K

$vibrational spectrum
#  mode     symmetry     wave number   IR intensity    selection rules
#                         cm**(-1)        km/mol          IR     RAMAN
     1                      -0.00          0.00000         -       -
     2                       0.00          0.00000         -       -
     3                       0.00          0.00000         -       -
     4                       0.00          0.00000         -       -
     5                       0.00          0.00000         -       -
     6                       0.00          0.00000         -       -
     7         a           112.85          2.37579        YES     YES
     8         a           171.86          1.33920        YES     YES
     9         a           378.25          4.61397        YES     YES
    10         a           459.98          3.22752        YES     YES
    11         a           528.56          2.92681        YES     YES
    12         a           709.94          8.10293        YES     YES
    13         a           726.59          9.28333        YES     YES
    14         a           792.52         17.06851        YES     YES
    15         a           835.97         34.11829        YES     YES
    16         a           860.23         11.45584        YES     YES
    17         a           968.09        196.48061        YES     YES
    18         a          1025.52        168.71195        YES     YES
    19         a          1035.18         26.82360        YES     YES
    20         a          1077.86         48.93653        YES     YES
    21         a          1102.45        225.12134        YES     YES
    22         a          1195.56         21.96222        YES     YES
    23         a          1311.51         21.29545        YES     YES
    24         a          1325.85         24.46421        YES     YES
    25         a          1351.85         12.81199        YES     YES
    26         a          1453.37          8.56791        YES     YES
    27         a          1863.71        552.09850        YES     YES
    28         a          2996.20         17.30453        YES     YES
    29         a          3042.79         22.38991        YES     YES
    30         a          3068.89          4.87759        YES     YES
$end
```

Double hybrid single point energy = -441.230407350209 H
COSMO energy + OC correction = -441.8917367036 H (in *o*DFB)

6.2.53 [F{Ga(dcpe)}$_2$(COOCH$_2$CHO)]$^{2+}$

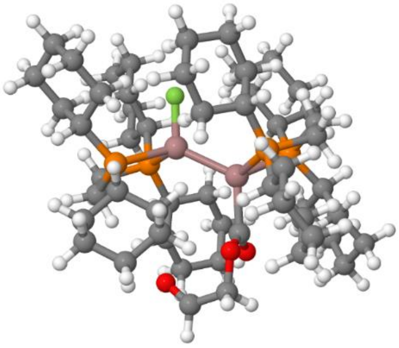

Method: (RI-)BP86(D3BJ)/def2-TZVPP
Symmetry: c1

Cartesian coordinates in Ångström:

C	4.4376790	5.0496805	1.5649686
C	5.0301972	3.8462729	0.8635642
O	6.2127898	3.6928459	0.6391868
H	4.2734928	3.0907378	0.5465040
H	3.7704771	5.5780066	0.8683094
O	5.4330760	6.0002101	1.9791975
C	6.1619057	5.6384268	3.0878099
H	10.1451690	12.4437708	3.0515063
H	11.4946710	11.4666657	3.6319481
C	10.6002540	11.4414977	2.9948536
H	11.9619543	8.1869780	8.3126718
H	11.6560047	11.9110195	1.1583895
H	9.3219867	10.6997923	4.5691852
C	10.9855995	11.1324052	1.5461042
C	11.9336662	7.0979253	8.1443204
H	12.8540375	6.6918037	8.5849870
C	9.6093900	10.4085220	3.5496940
H	10.7164601	6.7505914	9.9145659
H	9.3029659	8.0906581	8.3809804
H	9.2363322	11.9848095	0.5904809
H	8.6646692	9.4144979	6.0260379
H	10.5635497	8.4297895	6.1058729
H	6.8516254	10.7948057	5.0988039
H	12.7547953	7.2851607	6.1321939
C	10.7067472	6.5059204	8.8439870
H	7.8754699	11.3106923	2.6419928
H	11.5464371	10.1858044	1.5236463
C	9.7468696	11.0100723	0.6535899
H	7.2666658	9.2324397	7.0877518
C	11.8967410	6.8226281	6.6387043
C	7.6429792	9.0185200	6.0777497
C	9.3959339	7.0045539	8.2182332
C	8.3686936	10.3227254	2.6453702
H	10.0970590	9.4219926	3.6019565
C	10.5963281	7.3327661	6.0078420
C	6.7474609	9.7026522	5.0302136
H	14.4265628	8.2849117	0.1512185
H	10.0331288	10.7470874	-0.3741713
H	6.1628012	11.2747838	1.3080531
H	15.0097345	8.6242583	-1.4794491
C	8.7642997	9.9685862	1.2032399

H	8.5420393	6.5301605	8.7202175
H	10.7383577	5.4058207	8.7744238
C	9.3789361	6.7117835	6.7073630
H	5.1239099	11.5103808	2.7199704
C	14.1894599	8.1578046	-0.9180743
H	12.9131943	9.9209083	-0.9462775
H	5.6940264	9.4682039	5.2229073
H	11.9761915	5.7350656	6.4665196
H	10.5912751	7.1232278	4.9299353
C	5.2921346	10.8688183	1.8396133
H	15.4172097	5.8244491	3.5748386
P	7.0846334	9.1742348	3.2922394
H	14.4001181	5.5716079	4.9936877
P	7.7949286	7.1846179	5.8881508
C	12.8649238	8.8600710	-1.2288319
H	3.8707501	11.9555865	0.6193826
H	7.8777723	9.8960982	0.5562565
C	14.6796162	5.1414618	4.0180137
H	9.2456901	8.9758147	1.2050245
H	6.5488030	6.8670782	7.8914137
H	11.8382879	8.2957937	0.5964167
C	4.0634666	10.9164119	0.9180904
H	15.0469342	6.1578244	-0.9870776
H	12.9968740	6.0832883	2.9971494
H	9.4268398	5.6191576	6.5622348
C	5.5450429	9.4231675	2.2986749
C	14.1073521	6.6645312	-1.2466693
H	4.9170971	7.9258888	6.3950640
C	6.4321869	6.4375362	6.8809032
C	11.7013108	8.1910334	-0.4905252
H	16.1513040	3.8056189	4.8957564
H	13.1286296	6.0313828	0.5750395
C	13.4352882	5.0840102	3.1227529
H	12.6767036	8.8318424	-2.3144341
C	15.2851110	3.7503037	4.2229756
H	4.2959162	10.3625961	-0.0068252
Ga	9.8733374	6.3417637	2.4139046
C	5.0450263	6.8352321	6.3467025
Ga	7.7346484	6.8706723	3.4879764
C	12.9447054	5.9858663	-0.5086431
H	5.7199051	8.7964407	1.4067426
H	2.5143737	10.9393048	2.4283870
H	10.7567990	8.6927867	-0.7453425
H	4.0844725	9.4886030	3.9001308
C	2.8223864	10.3085258	1.5786135
H	13.9711546	6.5332506	-2.3324619
H	3.9307542	6.5369174	8.1776461
H	13.7389877	4.7253368	2.1260055
C	4.3077873	8.8624966	3.0218668
H	7.5775560	4.6686592	7.4249205
H	15.6631520	3.3716366	3.2589027
H	12.0909369	4.5026917	4.6934897
C	6.6121226	4.9118245	6.9591908
C	11.6188459	6.6904971	-0.8196528
H	13.9524990	3.0877724	5.7947578
C	3.9278835	6.1509057	7.1453478
C	12.3967636	4.1114633	3.7065945
C	14.2472106	2.7721837	4.7800796

H	5.5254098	4.5707005	8.8023602
H	4.9640140	6.5424603	5.2896862
H	1.9811599	10.3074813	0.8727236
H	12.8965078	4.9263122	-0.7973116
C	3.0960618	8.8879585	2.0831308
H	9.0578150	7.5503013	-1.3644775
C	5.4734021	4.2548381	7.7476175
H	4.4942215	7.8446085	3.3863183
P	10.1343377	5.9840011	0.0237595
H	2.9548400	6.4228261	6.7133556
H	6.6252473	4.5059228	5.9375610
P	10.8188889	4.1282834	2.7492811
H	11.3997352	6.5662283	-1.8961690
H	8.7278859	4.1762554	4.9070367
C	12.9910712	2.7054461	3.8984123
H	10.2338200	3.4904653	5.5471093
H	14.6756987	1.7656244	4.8786802
C	4.1071407	4.6306125	7.1685784
C	8.7300095	6.5489292	-1.0337822
H	3.2930638	8.2214333	1.2264741
C	9.3398646	3.2588642	4.9489297
H	2.2154613	8.4863263	2.6030905
H	7.6073559	7.3633316	0.6309311
H	6.5516668	8.2848655	-1.4056194
H	12.1954287	3.7795850	0.8073040
H	5.6112424	3.1647564	7.7399141
H	13.2540327	2.2841277	2.9140908
H	7.6983319	7.2315663	-3.5398965
H	9.4453506	5.5911576	-2.8656160
H	3.3042334	4.1641995	7.7546071
C	11.1510385	3.5324576	1.0306335
C	7.4361546	6.6998749	-0.2301828
H	12.2592717	2.0247724	4.3538334
H	8.2500270	2.4308149	6.6291658
C	9.7234786	2.8658848	3.5149991
C	8.5175878	5.6721624	-2.2806186
H	4.0251238	4.2352670	6.1429716
C	10.2112989	4.1399639	-0.0318589
C	6.3056158	7.2530739	-1.1016089
C	8.5360424	2.1328720	5.6109211
C	7.3909466	6.2474413	-3.1501993
H	7.8605590	3.4670569	2.5804696
H	10.5197642	3.8160673	-1.0352662
H	6.5925353	2.6152973	4.7991054
H	10.3426198	1.9525501	3.5543675
C	8.4757810	2.5610697	2.6757419
H	5.3821517	7.3139048	-0.5069631
H	11.0691829	2.4368011	1.0153476
C	6.0910295	6.3942224	-2.3524101
H	7.1451136	5.7230067	0.1785635
H	9.1794042	3.7886153	0.1171644
C	7.2953974	1.7706952	4.7886000
H	9.1866759	1.2497314	5.7175527
H	8.2412010	4.6540086	-1.9654226
H	7.2356780	5.5996824	-4.0233668
H	5.3052801	6.8291721	-2.9845865
H	8.7533279	2.2532989	1.6583597
C	7.6550235	1.4438626	3.3352130

```
H     6.7782626    0.9171356    5.2473884
H     5.7384222    5.3939706   -2.0515052
H     6.7450027    1.2689371    2.7457892
H     8.2363216    0.5078821    3.2991739
F    11.2426303    7.5012351    2.7888297
O     5.8992199    4.6265959    3.7112602
H     3.8365975    4.6981264    2.4177939

SCF energy GEOOPT =  -7698.133629778 H
ZPE =   3764. kJ/mol
FREEH energy =  3961.56 kJ/mol
FREEH entropy =   1.67005 kJ/mol/K

$vibrational spectrum
#  mode     symmetry     wave number   IR intensity    selection rules
#                         cm**(-1)        km/mol          IR     RAMAN
     1                      -0.00          0.00000         -       -
     2                      -0.00          0.00000         -       -
     3                      -0.00          0.00000         -       -
     4                      -0.00          0.00000         -       -
     5                      -0.00          0.00000         -       -
     6                       0.00          0.00000         -       -
     7         a             8.00          0.11045        YES     YES
     8         a            12.13          0.09012        YES     YES
     9         a            19.37          0.17570        YES     YES
    10         a            25.75          0.02735        YES     YES
    11         a            28.88          0.03589        YES     YES
    12         a            32.56          0.32476        YES     YES
    13         a            34.66          0.23236        YES     YES
    14         a            35.64          0.12259        YES     YES
    15         a            39.86          0.23032        YES     YES
    16         a            41.54          0.14922        YES     YES
    17         a            45.13          0.15870        YES     YES
    18         a            48.77          0.06473        YES     YES
    19         a            50.06          0.44913        YES     YES
    20         a            51.45          0.14460        YES     YES
    21         a            54.86          0.03111        YES     YES
    22         a            56.62          0.23650        YES     YES
    23         a            58.85          0.60398        YES     YES
    24         a            62.25          0.04736        YES     YES
    25         a            65.16          0.82205        YES     YES
    26         a            67.53          0.10166        YES     YES
    27         a            68.95          0.07714        YES     YES
    28         a            71.64          0.05914        YES     YES
    29         a            72.02          0.64482        YES     YES
    30         a            75.32          0.19777        YES     YES
    31         a            76.93          0.03840        YES     YES
    32         a            78.12          0.72638        YES     YES
    33         a            83.13          0.60614        YES     YES
    34         a            87.33          0.72366        YES     YES
    35         a            90.97          0.13226        YES     YES
    36         a            91.77          0.74448        YES     YES
    37         a            92.66          0.15406        YES     YES
    38         a           100.82          0.54889        YES     YES
    39         a           106.70          2.13984        YES     YES
    40         a           113.57          6.01343        YES     YES
    41         a           128.82          3.20338        YES     YES
    42         a           131.64          0.50699        YES     YES
```

43	a	137.65	0.26122	YES	YES
44	a	140.38	3.93184	YES	YES
45	a	142.45	3.11413	YES	YES
46	a	145.61	13.21490	YES	YES
47	a	149.68	0.56410	YES	YES
48	a	152.47	5.49308	YES	YES
49	a	153.86	3.32520	YES	YES
50	a	157.99	10.07889	YES	YES
51	a	162.31	9.81272	YES	YES
52	a	163.95	17.30609	YES	YES
53	a	167.52	0.95503	YES	YES
54	a	174.45	0.47497	YES	YES
55	a	180.43	0.60235	YES	YES
56	a	183.48	2.79373	YES	YES
57	a	190.71	5.55201	YES	YES
58	a	191.19	4.07739	YES	YES
59	a	198.21	1.63327	YES	YES
60	a	205.91	1.72395	YES	YES
61	a	209.65	2.37929	YES	YES
62	a	212.97	1.44225	YES	YES
63	a	214.00	6.24860	YES	YES
64	a	216.38	0.80431	YES	YES
65	a	217.40	1.34977	YES	YES
66	a	227.37	1.52565	YES	YES
67	a	229.08	0.94382	YES	YES
68	a	235.70	0.79508	YES	YES
69	a	238.88	0.82062	YES	YES
70	a	240.81	0.88724	YES	YES
71	a	242.77	2.06036	YES	YES
72	a	245.30	0.02503	YES	YES
73	a	249.81	3.28730	YES	YES
74	a	253.63	0.24885	YES	YES
75	a	257.10	3.16416	YES	YES
76	a	277.31	1.97804	YES	YES
77	a	280.70	0.37492	YES	YES
78	a	283.03	3.11240	YES	YES
79	a	286.56	1.59121	YES	YES
80	a	292.43	0.27259	YES	YES
81	a	302.53	0.86192	YES	YES
82	a	305.20	2.29217	YES	YES
83	a	308.49	0.93467	YES	YES
84	a	312.01	0.37615	YES	YES
85	a	316.03	0.60023	YES	YES
86	a	330.40	0.47364	YES	YES
87	a	331.91	1.55936	YES	YES
88	a	335.88	0.94930	YES	YES
89	a	336.99	0.24263	YES	YES
90	a	337.86	14.69255	YES	YES
91	a	342.38	1.69712	YES	YES
92	a	345.18	0.10130	YES	YES
93	a	374.44	2.41774	YES	YES
94	a	377.05	0.85984	YES	YES
95	a	379.72	1.26025	YES	YES
96	a	381.80	0.92547	YES	YES
97	a	396.21	0.30052	YES	YES
98	a	397.07	0.82714	YES	YES
99	a	413.25	0.70721	YES	YES
100	a	414.84	1.07968	YES	YES

101	a	422.76	0.83002	YES	YES
102	a	423.68	6.62842	YES	YES
103	a	425.27	0.21156	YES	YES
104	a	429.40	0.09008	YES	YES
105	a	430.41	0.22052	YES	YES
106	a	430.79	0.09736	YES	YES
107	a	431.58	0.53792	YES	YES
108	a	432.32	0.01073	YES	YES
109	a	432.83	0.89341	YES	YES
110	a	434.86	0.51510	YES	YES
111	a	436.83	0.94995	YES	YES
112	a	439.25	1.57087	YES	YES
113	a	449.77	5.31637	YES	YES
114	a	450.94	1.83292	YES	YES
115	a	470.94	1.05603	YES	YES
116	a	471.50	4.61495	YES	YES
117	a	492.05	4.80750	YES	YES
118	a	493.58	2.30597	YES	YES
119	a	495.54	3.39831	YES	YES
120	a	496.34	2.83602	YES	YES
121	a	506.42	0.39434	YES	YES
122	a	507.58	0.67944	YES	YES
123	a	518.65	7.93595	YES	YES
124	a	520.94	8.92977	YES	YES
125	a	542.40	16.10770	YES	YES
126	a	551.32	32.91799	YES	YES
127	a	625.37	17.88440	YES	YES
128	a	629.38	16.61304	YES	YES
129	a	643.31	10.58623	YES	YES
130	a	646.14	6.42869	YES	YES
131	a	647.74	3.66353	YES	YES
132	a	710.84	0.01655	YES	YES
133	a	717.39	0.45833	YES	YES
134	a	721.72	1.99980	YES	YES
135	a	723.81	1.47759	YES	YES
136	a	732.53	17.52948	YES	YES
137	a	733.71	16.20925	YES	YES
138	a	734.50	5.23952	YES	YES
139	a	735.20	4.51084	YES	YES
140	a	740.07	7.84905	YES	YES
141	a	771.39	0.23883	YES	YES
142	a	771.97	0.24533	YES	YES
143	a	774.31	0.08672	YES	YES
144	a	776.19	0.31983	YES	YES
145	a	777.05	0.26022	YES	YES
146	a	777.35	2.16711	YES	YES
147	a	780.00	0.26036	YES	YES
148	a	780.31	0.20228	YES	YES
149	a	783.86	13.82362	YES	YES
150	a	789.17	9.51155	YES	YES
151	a	803.59	6.78657	YES	YES
152	a	810.36	0.41694	YES	YES
153	a	810.57	0.23073	YES	YES
154	a	811.19	2.52995	YES	YES
155	a	812.41	0.45294	YES	YES
156	a	812.96	1.24402	YES	YES
157	a	814.81	2.28305	YES	YES
158	a	815.45	12.30243	YES	YES

159	a	816.12	0.23478	YES	YES
160	a	824.13	23.03970	YES	YES
161	a	833.84	0.51442	YES	YES
162	a	835.34	1.42495	YES	YES
163	a	836.15	3.22639	YES	YES
164	a	837.25	1.92063	YES	YES
165	a	837.67	0.82686	YES	YES
166	a	838.95	6.05246	YES	YES
167	a	839.35	16.64136	YES	YES
168	a	840.45	6.17364	YES	YES
169	a	853.13	18.04268	YES	YES
170	a	854.35	0.85356	YES	YES
171	a	876.05	4.19254	YES	YES
172	a	876.41	3.27569	YES	YES
173	a	876.98	1.67780	YES	YES
174	a	877.72	1.66340	YES	YES
175	a	878.57	5.45316	YES	YES
176	a	878.92	8.25192	YES	YES
177	a	879.50	2.26354	YES	YES
178	a	879.94	2.27381	YES	YES
179	a	880.58	4.61848	YES	YES
180	a	881.38	1.80550	YES	YES
181	a	881.87	1.16518	YES	YES
182	a	883.78	2.04792	YES	YES
183	a	884.13	2.92716	YES	YES
184	a	885.22	2.88252	YES	YES
185	a	887.38	3.46285	YES	YES
186	a	889.06	2.02452	YES	YES
187	a	903.53	1.45133	YES	YES
188	a	904.53	2.90804	YES	YES
189	a	905.37	0.57672	YES	YES
190	a	907.79	2.40256	YES	YES
191	a	908.85	2.68620	YES	YES
192	a	909.75	1.60158	YES	YES
193	a	911.48	1.37989	YES	YES
194	a	912.62	0.17241	YES	YES
195	a	975.94	0.04252	YES	YES
196	a	984.36	23.17777	YES	YES
197	a	986.30	1.09211	YES	YES
198	a	988.60	2.41715	YES	YES
199	a	989.16	15.13365	YES	YES
200	a	989.31	8.29126	YES	YES
201	a	990.31	12.83156	YES	YES
202	a	991.34	18.73627	YES	YES
203	a	991.96	20.75295	YES	YES
204	a	993.27	1.71279	YES	YES
205	a	994.17	0.59705	YES	YES
206	a	1014.37	2.16393	YES	YES
207	a	1015.83	2.59932	YES	YES
208	a	1016.34	0.84116	YES	YES
209	a	1016.93	0.70052	YES	YES
210	a	1017.59	0.43994	YES	YES
211	a	1018.86	2.23236	YES	YES
212	a	1020.61	1.48306	YES	YES
213	a	1020.94	2.11752	YES	YES
214	a	1030.31	11.31804	YES	YES
215	a	1032.03	14.72658	YES	YES
216	a	1033.48	2.31293	YES	YES

217	a	1034.47	7.41119	YES	YES
218	a	1035.48	174.08121	YES	YES
219	a	1039.64	16.28910	YES	YES
220	a	1040.14	0.64582	YES	YES
221	a	1040.25	5.45227	YES	YES
222	a	1041.23	0.75670	YES	YES
223	a	1059.15	2.56491	YES	YES
224	a	1059.85	1.79915	YES	YES
225	a	1060.76	1.08327	YES	YES
226	a	1063.01	5.84476	YES	YES
227	a	1065.11	2.20066	YES	YES
228	a	1066.34	0.54034	YES	YES
229	a	1066.96	1.05836	YES	YES
230	a	1068.84	0.15031	YES	YES
231	a	1069.51	0.02600	YES	YES
232	a	1070.71	0.28231	YES	YES
233	a	1071.08	0.07049	YES	YES
234	a	1072.28	0.21083	YES	YES
235	a	1072.56	0.64092	YES	YES
236	a	1073.94	0.03085	YES	YES
237	a	1074.65	0.11399	YES	YES
238	a	1075.15	0.21349	YES	YES
239	a	1075.77	0.54189	YES	YES
240	a	1084.71	2.39653	YES	YES
241	a	1087.67	4.09133	YES	YES
242	a	1096.90	6.87602	YES	YES
243	a	1098.23	0.53149	YES	YES
244	a	1101.72	5.83275	YES	YES
245	a	1102.98	1.50583	YES	YES
246	a	1104.06	7.22744	YES	YES
247	a	1108.24	0.41120	YES	YES
248	a	1108.96	9.75113	YES	YES
249	a	1112.38	8.26335	YES	YES
250	a	1127.32	2.30728	YES	YES
251	a	1133.37	1.65561	YES	YES
252	a	1158.82	3.13012	YES	YES
253	a	1160.05	5.98064	YES	YES
254	a	1161.79	2.69295	YES	YES
255	a	1164.78	6.84452	YES	YES
256	a	1169.69	8.87182	YES	YES
257	a	1170.83	3.23648	YES	YES
258	a	1171.13	8.33565	YES	YES
259	a	1172.61	11.63101	YES	YES
260	a	1182.05	27.46162	YES	YES
261	a	1183.79	8.82133	YES	YES
262	a	1186.12	9.78082	YES	YES
263	a	1187.48	11.52989	YES	YES
264	a	1190.61	2.98289	YES	YES
265	a	1193.48	3.03441	YES	YES
266	a	1197.43	5.80426	YES	YES
267	a	1200.84	3.07340	YES	YES
268	a	1229.44	5.05030	YES	YES
269	a	1244.11	0.28976	YES	YES
270	a	1244.44	0.15130	YES	YES
271	a	1244.68	0.42856	YES	YES
272	a	1245.05	0.68243	YES	YES
273	a	1245.73	1.11919	YES	YES
274	a	1247.29	0.38806	YES	YES

275	a	1248.42	1.38526	YES	YES
276	a	1250.50	0.84703	YES	YES
277	a	1252.14	0.26383	YES	YES
278	a	1253.04	0.05299	YES	YES
279	a	1255.03	3.01675	YES	YES
280	a	1256.16	11.33220	YES	YES
281	a	1256.55	2.26132	YES	YES
282	a	1256.94	2.42846	YES	YES
283	a	1257.17	4.45665	YES	YES
284	a	1257.42	8.26694	YES	YES
285	a	1257.90	2.35928	YES	YES
286	a	1258.91	3.56734	YES	YES
287	a	1259.49	1.68185	YES	YES
288	a	1260.63	0.50818	YES	YES
289	a	1261.11	1.28269	YES	YES
290	a	1261.80	2.74428	YES	YES
291	a	1262.45	0.38694	YES	YES
292	a	1266.39	4.56120	YES	YES
293	a	1270.01	1.42931	YES	YES
294	a	1270.31	1.56554	YES	YES
295	a	1270.61	1.18328	YES	YES
296	a	1273.57	1.44475	YES	YES
297	a	1282.19	4.74696	YES	YES
298	a	1282.80	1.43702	YES	YES
299	a	1284.87	1.84287	YES	YES
300	a	1286.56	1.16096	YES	YES
301	a	1287.32	3.11771	YES	YES
302	a	1289.47	1.72921	YES	YES
303	a	1291.77	2.29666	YES	YES
304	a	1293.28	4.53451	YES	YES
305	a	1306.86	2.66114	YES	YES
306	a	1307.19	1.07759	YES	YES
307	a	1310.39	0.29036	YES	YES
308	a	1311.34	0.79329	YES	YES
309	a	1311.74	3.20233	YES	YES
310	a	1316.22	8.43038	YES	YES
311	a	1317.23	1.36696	YES	YES
312	a	1318.89	1.81749	YES	YES
313	a	1319.09	0.31787	YES	YES
314	a	1321.22	0.59516	YES	YES
315	a	1322.11	0.69739	YES	YES
316	a	1322.70	1.02569	YES	YES
317	a	1323.34	2.36010	YES	YES
318	a	1323.69	0.08541	YES	YES
319	a	1324.18	0.13509	YES	YES
320	a	1324.55	2.64819	YES	YES
321	a	1324.83	0.65163	YES	YES
322	a	1325.73	1.30189	YES	YES
323	a	1326.20	2.98392	YES	YES
324	a	1327.00	2.76920	YES	YES
325	a	1327.26	4.96933	YES	YES
326	a	1329.07	1.90023	YES	YES
327	a	1333.75	2.08678	YES	YES
328	a	1336.00	1.81703	YES	YES
329	a	1336.30	0.04295	YES	YES
330	a	1336.54	0.31566	YES	YES
331	a	1338.02	0.45183	YES	YES
332	a	1338.54	0.51701	YES	YES

333	a	1339.74	0.44922	YES	YES
334	a	1339.96	0.25585	YES	YES
335	a	1340.67	1.61959	YES	YES
336	a	1343.11	2.05202	YES	YES
337	a	1345.60	2.06509	YES	YES
338	a	1346.16	1.63175	YES	YES
339	a	1346.41	1.67621	YES	YES
340	a	1347.10	1.09119	YES	YES
341	a	1348.21	0.06521	YES	YES
342	a	1348.49	2.90504	YES	YES
343	a	1349.38	0.49214	YES	YES
344	a	1350.07	0.09834	YES	YES
345	a	1351.93	0.60122	YES	YES
346	a	1357.37	16.57389	YES	YES
347	a	1375.67	28.49486	YES	YES
348	a	1400.94	7.10422	YES	YES
349	a	1401.33	6.99150	YES	YES
350	a	1404.96	7.37861	YES	YES
351	a	1407.50	8.04254	YES	YES
352	a	1421.65	3.78319	YES	YES
353	a	1425.70	3.46722	YES	YES
354	a	1427.30	1.32112	YES	YES
355	a	1429.41	2.89560	YES	YES
356	a	1432.73	3.23907	YES	YES
357	a	1433.76	9.55126	YES	YES
358	a	1434.80	3.71288	YES	YES
359	a	1435.03	3.72785	YES	YES
360	a	1435.34	2.58343	YES	YES
361	a	1436.64	2.07763	YES	YES
362	a	1437.16	0.72289	YES	YES
363	a	1437.37	2.35909	YES	YES
364	a	1437.44	1.00151	YES	YES
365	a	1437.71	7.66912	YES	YES
366	a	1437.95	1.36615	YES	YES
367	a	1438.42	4.88265	YES	YES
368	a	1438.98	3.87316	YES	YES
369	a	1439.34	9.60457	YES	YES
370	a	1439.96	2.38254	YES	YES
371	a	1440.73	2.29047	YES	YES
372	a	1440.96	2.86318	YES	YES
373	a	1441.12	5.46721	YES	YES
374	a	1441.78	11.72364	YES	YES
375	a	1442.57	36.05818	YES	YES
376	a	1443.03	0.66948	YES	YES
377	a	1443.86	6.08663	YES	YES
378	a	1443.98	13.37471	YES	YES
379	a	1445.05	26.63709	YES	YES
380	a	1446.27	4.85807	YES	YES
381	a	1446.53	35.47478	YES	YES
382	a	1446.73	3.84609	YES	YES
383	a	1447.29	4.53143	YES	YES
384	a	1452.78	3.68581	YES	YES
385	a	1454.14	2.36117	YES	YES
386	a	1455.08	0.43992	YES	YES
387	a	1455.46	2.10367	YES	YES
388	a	1457.19	1.16338	YES	YES
389	a	1457.74	2.21520	YES	YES
390	a	1459.70	3.80214	YES	YES

391	a	1461.15	6.29815	YES	YES
392	a	1661.62	194.43551	YES	YES
393	a	1740.68	96.20546	YES	YES
394	a	2846.76	82.14989	YES	YES
395	a	2922.26	4.66191	YES	YES
396	a	2929.03	20.40690	YES	YES
397	a	2929.67	3.62643	YES	YES
398	a	2931.48	7.95252	YES	YES
399	a	2932.98	15.15982	YES	YES
400	a	2936.36	4.55443	YES	YES
401	a	2936.63	8.48473	YES	YES
402	a	2937.04	9.86514	YES	YES
403	a	2939.87	8.10955	YES	YES
404	a	2940.34	9.16203	YES	YES
405	a	2941.31	12.96967	YES	YES
406	a	2941.83	29.87315	YES	YES
407	a	2943.70	7.93746	YES	YES
408	a	2944.62	7.79815	YES	YES
409	a	2944.79	11.20689	YES	YES
410	a	2946.17	10.06175	YES	YES
411	a	2946.53	10.54129	YES	YES
412	a	2947.28	10.42229	YES	YES
413	a	2948.60	13.75533	YES	YES
414	a	2949.40	13.15102	YES	YES
415	a	2951.97	19.83320	YES	YES
416	a	2952.53	12.77274	YES	YES
417	a	2953.77	10.85592	YES	YES
418	a	2953.98	14.37309	YES	YES
419	a	2954.23	10.00204	YES	YES
420	a	2955.26	14.45047	YES	YES
421	a	2955.46	16.00935	YES	YES
422	a	2955.71	0.82908	YES	YES
423	a	2956.11	5.74783	YES	YES
424	a	2957.10	2.61219	YES	YES
425	a	2957.51	14.54261	YES	YES
426	a	2959.11	15.20298	YES	YES
427	a	2959.97	12.06408	YES	YES
428	a	2960.34	36.43101	YES	YES
429	a	2960.68	30.23658	YES	YES
430	a	2961.04	22.32403	YES	YES
431	a	2961.48	18.01780	YES	YES
432	a	2961.73	20.33328	YES	YES
433	a	2961.95	73.51704	YES	YES
434	a	2964.76	46.80189	YES	YES
435	a	2964.99	20.39142	YES	YES
436	a	2965.27	20.92681	YES	YES
437	a	2966.56	14.60931	YES	YES
438	a	2968.77	27.81104	YES	YES
439	a	2972.98	19.89147	YES	YES
440	a	2974.02	8.00472	YES	YES
441	a	2975.97	16.17209	YES	YES
442	a	2977.03	5.60000	YES	YES
443	a	2979.90	12.48551	YES	YES
444	a	2985.48	10.12404	YES	YES
445	a	2987.27	4.39903	YES	YES
446	a	2989.93	29.96213	YES	YES
447	a	2990.88	5.22581	YES	YES
448	a	2991.09	38.98294	YES	YES

449	a	2992.12	3.57581	YES	YES
450	a	2993.99	28.20045	YES	YES
451	a	3004.00	21.48782	YES	YES
452	a	3006.26	28.12389	YES	YES
453	a	3006.63	41.23760	YES	YES
454	a	3007.11	12.74200	YES	YES
455	a	3007.18	38.40905	YES	YES
456	a	3008.62	6.76709	YES	YES
457	a	3009.62	26.28832	YES	YES
458	a	3010.18	9.05561	YES	YES
459	a	3010.29	11.93389	YES	YES
460	a	3010.82	25.16326	YES	YES
461	a	3011.41	23.38174	YES	YES
462	a	3011.83	12.85598	YES	YES
463	a	3012.90	12.66963	YES	YES
464	a	3013.36	23.82913	YES	YES
465	a	3013.40	23.27549	YES	YES
466	a	3014.36	30.55119	YES	YES
467	a	3014.61	34.44640	YES	YES
468	a	3014.75	22.73835	YES	YES
469	a	3015.08	13.56894	YES	YES
470	a	3015.37	26.70463	YES	YES
471	a	3015.49	22.26594	YES	YES
472	a	3015.62	11.87713	YES	YES
473	a	3016.11	19.43399	YES	YES
474	a	3016.72	15.99665	YES	YES
475	a	3017.22	36.68421	YES	YES
476	a	3017.51	17.98108	YES	YES
477	a	3018.09	18.03530	YES	YES
478	a	3018.55	25.52106	YES	YES
479	a	3018.72	26.10117	YES	YES
480	a	3019.31	20.18773	YES	YES
481	a	3020.59	10.17101	YES	YES
482	a	3022.47	9.63627	YES	YES
483	a	3022.79	15.14078	YES	YES
484	a	3022.81	28.12927	YES	YES
485	a	3024.32	7.10647	YES	YES
486	a	3025.01	0.14704	YES	YES
487	a	3026.18	20.32989	YES	YES
488	a	3027.55	0.38371	YES	YES
489	a	3032.10	26.00099	YES	YES
490	a	3036.66	0.06324	YES	YES
491	a	3050.64	0.49840	YES	YES
492	a	3051.81	0.02822	YES	YES

$end

Double hybrid single point energy = -7690.386609477206 H
COSMO energy + OC correction = -7698.2834970422 H (in *o*DFB)

6.2.54 1,2,3-$C_6F_3H_3$ (3FB)

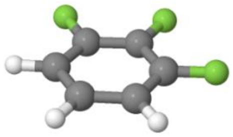

```
Method: (RI-)BP86(D3BJ)/def2-TZVPP
Symmetry: c2v

Cartesian coordinates in Ångström:
H     2.1711364    0.0000000    1.2689662
C     1.2148251    0.0000000    0.7497252
C     0.0000000    0.0000000    1.4385189
H     0.0000000    0.0000000    2.5269849
C    -1.2148251    0.0000000    0.7497252
H    -2.1711364    0.0000000    1.2689662
C    -1.2023417    0.0000000   -0.6408303
F    -2.3582127    0.0000000   -1.3361296
C     0.0000000    0.0000000   -1.3530881
F     0.0000000    0.0000000   -2.6958826
C     1.2023417    0.0000000   -0.6408303
F     2.3582127    0.0000000   -1.3361296

SCF energy GEOOPT =  -530.2114836135 H
ZPE =   193.6 kJ/mol
FREEH energy =   212.08 kJ/mol
FREEH entropy =   0.34268 kJ/mol/K

$vibrational spectrum
#  mode     symmetry     wave number   IR intensity    selection rules
#                         cm**(-1)        km/mol          IR     RAMAN
     1                       -0.00         0.00000         -        -
     2                       -0.00         0.00000         -        -
     3                        0.00         0.00000         -        -
     4                        0.00         0.00000         -        -
     5                        0.00         0.00000         -        -
     6                        0.00         0.00000         -        -
     7         b2           146.42         0.14589        YES      YES
     8         a2           243.97         0.00000        NO       YES
     9         b1           269.71         0.02798        YES      YES
    10         b2           289.90         0.19644        YES      YES
    11         a1           296.51         1.11042        YES      YES
    12         a1           469.94         0.11890        YES      YES
    13         b1           492.45         1.98853        YES      YES
    14         b2           529.24         0.10970        YES      YES
    15         a2           559.45         0.00000        NO       YES
    16         b1           563.72         2.75268        YES      YES
    17         b2           664.54         7.33420        YES      YES
    18         a1           683.65        21.71400        YES      YES
    19         b2           740.73        61.09867        YES      YES
    20         a1           816.41        11.30959        YES      YES
    21         a2           846.13         0.00000        NO       YES
    22         b2           911.60         0.42367        YES      YES
    23         b1          1005.56       136.78447        YES      YES
    24         a1          1054.02        10.34000        YES      YES
    25         b1          1144.05         2.07489        YES      YES
    26         a1          1211.93        21.24624        YES      YES
```

27	b1	1230.29	42.40523	YES	YES
28	a1	1287.96	94.82304	YES	YES
29	b1	1341.64	0.45147	YES	YES
30	b1	1467.47	85.82427	YES	YES
31	a1	1497.54	196.98206	YES	YES
32	b1	1596.81	71.66679	YES	YES
33	a1	1601.97	0.09224	YES	YES
34	a1	3124.28	3.48167	YES	YES
35	b1	3138.97	1.04163	YES	YES
36	a1	3145.00	0.19411	YES	YES

$end

Double hybrid single point energy = -529.424581590831 H
COSMO energy + OC correction = -530.2158225968 H (in 3FB)

6.2.55 [{Ga(dcpe)}$_2$($C_6F_3H_3$)]$^{2+}$ (Adduct)

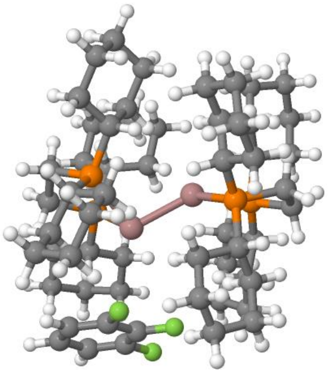

Method: (RI-)BP86(D3BJ)/def2-TZVPP
Symmetry: c1

Cartesian coordinates in Ångström:

Ga	-0.4899183	0.2404897	-1.0682031
Ga	0.6611474	0.0273380	1.0322014
P	-0.4561479	2.3605827	-2.2797401
P	-1.1857843	0.7751259	2.5041997
P	-0.1966905	-0.7552641	-3.2660672
P	0.2190203	-2.1124589	2.1465993
C	-4.2797998	3.5504808	-1.1073975
H	-4.0432956	3.7572072	-0.0496185
H	-5.2302440	2.9986579	-1.1147400
C	1.2542210	-1.8496668	-3.5634953
H	1.2631486	-2.0987482	-4.6385104
C	1.7611922	-3.1152978	2.3053011
H	2.5401539	-2.3371926	2.4074775
C	-2.9805598	-0.8732962	-3.2366454
H	-3.0423731	-0.0299111	-3.9435452
H	-2.8921830	-0.4373799	-2.2253840
C	-2.9232092	1.0245749	1.8867630
H	-2.8083056	1.7930551	1.1027675
C	-1.3147051	-0.4931296	3.8569916
H	-1.3720884	0.0192377	4.8269379
H	-2.2715733	-1.0138421	3.7203457
C	-3.0872037	5.6309585	-1.9134924
H	-3.1894205	6.5537695	-2.4999230

H	-2.8028904	5.9382016	-0.8933873
C	-1.9625401	4.7685325	-2.5042168
H	-1.0152015	5.3244375	-2.4840612
H	-2.1842594	4.5499462	-3.5615113
C	-3.1682164	2.6815190	-1.7046787
H	-3.4511004	2.3927342	-2.7296707
H	-3.0541263	1.7442164	-1.1353179
C	3.7755603	-2.0251590	-3.3617540
H	3.8976587	-2.2838472	-4.4259169
H	4.6798828	-1.4778535	-3.0641977
C	-1.4376737	-4.4559655	2.4153983
H	-1.3653606	-4.3063074	3.5017823
H	-0.6326174	-5.1510091	2.1332094
C	-0.7023416	2.3613582	3.3098133
H	-1.4474910	2.5936330	4.0877604
C	1.3560385	3.9012391	-0.8270599
H	0.4966279	4.4718279	-0.4477073
H	1.4964665	3.0347525	-0.1579071
C	2.6145165	4.7777352	-0.7969611
H	2.4304377	5.6923214	-1.3838000
H	2.8054854	5.1020586	0.2354408
C	-3.4945861	-0.2370538	1.2261843
H	-2.8074700	-0.5890680	0.4404639
H	-3.5668665	-1.0418802	1.9766689
C	-4.4180420	4.8741222	-1.8667872
H	-4.7587324	4.6680141	-2.8947106
H	-5.1931962	5.4978838	-1.4021786
C	-3.8746417	1.5587527	2.9714161
H	-3.4878093	2.4909997	3.4042169
H	-3.9374792	0.8274627	3.7938831
C	-0.7050811	3.4687770	2.2423945
H	-1.7212633	3.6117972	1.8455166
H	-0.0740448	3.1363499	1.4005037
C	-1.8377352	-2.3735177	-4.9422700
H	-0.9582052	-3.0020840	-5.1412056
H	-1.8518750	-1.5898541	-5.7172555
C	3.5508419	3.4990940	-2.7665815
H	3.4159834	4.3400779	-3.4661828
H	4.4092043	2.9208116	-3.1350322
C	1.2007714	3.5477166	4.5043846
H	0.5578103	3.8839661	5.3337250
H	2.2050645	3.4078183	4.9285291
C	-5.8464369	0.5433752	1.7302964
H	-6.8328717	0.7563897	1.2968504
H	-6.0035193	-0.2378555	2.4923066
C	1.8454712	-4.0302493	3.5396812
H	1.7083691	-3.4470338	4.4605140
H	1.0396088	-4.7770193	3.5108804
C	3.6258264	-3.3027681	-2.5330314
H	4.4929258	-3.9592077	-2.6845688
H	3.6205740	-3.0360885	-1.4642046
C	-4.8940689	0.0134766	0.6544700
H	-4.8249716	0.7440993	-0.1676211
H	-5.2829067	-0.9161246	0.2149320
C	3.8301689	4.0375345	-1.3607041
H	4.0840975	3.1994176	-0.6951649
H	4.7031441	4.7034771	-1.3786279
C	1.2254186	4.6171410	3.4084860

H	1.5825905	5.5716428	3.8171219
H	1.9423177	4.3185163	2.6263658
C	-1.3719024	-3.3301680	0.1436167
H	-0.5585178	-3.9813313	-0.2089250
H	-1.2498142	-2.3599892	-0.3662198
C	0.6800563	2.2103363	3.9652266
H	1.3843615	1.8254811	3.2082434
H	0.6393440	1.4639142	4.7727476
C	-1.2537884	-3.1279179	1.6619256
H	-2.0839680	-2.4617782	1.9551883
C	-0.1619929	4.7944365	2.7864044
C	-2.9421893	-5.2755290	0.5443648
H	-3.9324102	-5.6888069	0.3101243
H	-2.2041715	-6.0189470	0.2007744
C	-2.7946141	-5.0795634	2.0565292
H	-2.9091284	-6.0378463	2.5809552
H	-3.6012198	-4.4232745	2.4237225
C	1.1032776	-3.1397560	-2.7388783
H	0.1984192	-3.6881298	-3.0350011
H	0.9737837	-2.8616247	-1.6792422
C	3.1990460	-4.7528560	3.5784207
H	3.9998242	-4.0092467	3.7214468
H	3.2323247	-5.4194996	4.4507657
C	-4.2568629	-1.7140971	-3.3452540
H	-5.1347566	-1.0837478	-3.1470560
H	-4.2401167	-2.4891490	-2.5618962
C	3.4544506	-5.5389528	2.2892666
H	2.7054645	-6.3431915	2.1991473
H	4.4348573	-6.0320891	2.3316541
C	2.0364838	-3.8837287	1.0013100
H	1.2299094	-4.6139179	0.8319573
H	2.0225007	-3.1884355	0.1479538
C	-5.2798042	1.7983551	2.3990252
H	-5.9460373	2.1420743	3.2017962
H	-5.2303363	2.6158644	1.6618543
C	-3.1220815	-3.2094246	-5.0463111
H	-3.1987607	-3.6417764	-6.0528345
H	-3.0543910	-4.0579662	-4.3455174
C	-2.7216670	-3.9609056	-0.2118094
H	-2.7797331	-4.1299025	-1.2975071
H	-3.5273561	-3.2503033	0.0391082
C	-4.3669557	-2.3765759	-4.7232242
H	-5.2657224	-3.0058520	-4.7687304
H	-4.4940495	-1.5970690	-5.4925208
C	2.3334324	-4.0435949	-2.8835822
H	2.2080594	-4.9300550	-2.2455395
H	2.3888486	-4.4129876	-3.9200915
C	3.3746746	-4.6279981	1.0609301
H	4.1952498	-3.8957212	1.0893061
H	3.5116710	-5.2096836	0.1387577
F	4.0830974	0.5995711	-0.4166631
C	1.0801577	3.3837731	-2.2469610
H	0.8702518	4.2408538	-2.9107980
C	2.2944383	2.6186759	-2.7956272
H	2.1061897	2.2821992	-3.8250108
H	2.4575696	1.7197548	-2.1800245
C	2.5552313	-1.1124878	-3.1979641
H	2.6774996	-0.2093121	-3.8115408

H	2.4897556	-0.7762772	-2.1485204
C	-0.1291276	0.6113994	-4.5117101
H	0.9376818	0.7746952	-4.7102931
H	-0.5788199	0.2697213	-5.4544522
C	-0.7937828	1.9215107	-4.0484370
H	-1.8853636	1.8449618	-4.1382519
H	-0.4788357	2.7537094	-4.6931598
C	-1.7376003	-1.7227369	-3.5556367
H	-1.6810700	-2.5215405	-2.7953682
C	-1.8369079	3.4485384	-1.7270134
H	-1.5698638	3.6810697	-0.6805574
C	-0.1561259	-1.5003327	3.8494580
H	-0.3729774	-2.3424628	4.5201944
H	0.7707051	-1.0297721	4.2095138
H	-0.8582387	5.1879595	3.5443572
H	-0.1309469	5.5381728	1.9771168
H	3.7637212	1.2939681	4.0433015
C	4.0679634	0.6272358	3.2393023
C	3.8797457	1.0240725	1.9203456
F	3.3282732	2.2254817	1.6373210
C	4.2750695	0.2103798	0.8569001
C	4.8941775	-1.0088998	1.1371663
F	5.2896555	-1.7743409	0.1009327
C	5.1044364	-1.4234885	2.4477425
H	5.6148862	-2.3668047	2.6295075
C	4.6814133	-0.6015565	3.4948969
H	4.8552134	-0.9115272	4.5235028

SCF energy GEOOPT = -7786.399948175 H
ZPE = 3792. kJ/mol
FREEH energy = 3992.89 kJ/mol
FREEH entropy = 1.71264 kJ/mol/K

$vibrational spectrum

# mode	symmetry	wave number	IR intensity	selection rules	
#		cm**(-1)	km/mol	IR	RAMAN
1		-0.00	0.00000	-	-
2		0.00	0.00000	-	-
3		0.00	0.00000	-	-
4		0.00	0.00000	-	-
5		0.00	0.00000	-	-
6		0.00	0.00000	-	-
7	a	13.82	0.07364	YES	YES
8	a	18.60	0.06536	YES	YES
9	a	22.37	0.03251	YES	YES
10	a	24.29	0.03738	YES	YES
11	a	26.63	0.52926	YES	YES
12	a	31.25	0.23155	YES	YES
13	a	32.02	0.09428	YES	YES
14	a	35.50	0.42678	YES	YES
15	a	37.40	0.53564	YES	YES
16	a	38.00	0.52064	YES	YES
17	a	40.10	0.01692	YES	YES
18	a	42.21	0.44892	YES	YES
19	a	43.20	1.35327	YES	YES
20	a	46.31	0.14886	YES	YES
21	a	47.19	0.11618	YES	YES
22	a	48.40	0.08747	YES	YES

23	a	49.82	0.65420	YES	YES
24	a	51.21	0.16145	YES	YES
25	a	54.04	0.66327	YES	YES
26	a	56.38	0.36059	YES	YES
27	a	59.18	0.14537	YES	YES
28	a	60.83	0.09323	YES	YES
29	a	63.74	0.01338	YES	YES
30	a	64.33	0.13724	YES	YES
31	a	66.67	0.16129	YES	YES
32	a	68.05	0.08057	YES	YES
33	a	69.14	0.08772	YES	YES
34	a	71.09	0.43829	YES	YES
35	a	71.86	0.24739	YES	YES
36	a	74.84	0.24549	YES	YES
37	a	77.01	0.23929	YES	YES
38	a	81.92	0.31438	YES	YES
39	a	84.58	0.67268	YES	YES
40	a	89.58	1.14860	YES	YES
41	a	97.47	0.43360	YES	YES
42	a	113.72	0.38896	YES	YES
43	a	119.48	0.27907	YES	YES
44	a	126.69	1.05442	YES	YES
45	a	133.20	0.16402	YES	YES
46	a	134.94	0.41049	YES	YES
47	a	139.23	0.75101	YES	YES
48	a	141.28	0.10522	YES	YES
49	a	145.09	1.48460	YES	YES
50	a	152.42	0.55273	YES	YES
51	a	157.97	1.33678	YES	YES
52	a	159.04	5.29189	YES	YES
53	a	168.37	0.08160	YES	YES
54	a	174.37	1.86575	YES	YES
55	a	182.04	1.00933	YES	YES
56	a	184.08	2.07451	YES	YES
57	a	191.13	0.61820	YES	YES
58	a	195.38	1.69012	YES	YES
59	a	212.69	0.40824	YES	YES
60	a	213.34	0.04128	YES	YES
61	a	215.62	0.46516	YES	YES
62	a	217.63	0.10892	YES	YES
63	a	220.83	0.43689	YES	YES
64	a	224.31	0.07694	YES	YES
65	a	226.95	0.13266	YES	YES
66	a	227.92	0.76298	YES	YES
67	a	231.25	0.11557	YES	YES
68	a	234.17	0.27411	YES	YES
69	a	241.10	0.18193	YES	YES
70	a	243.71	0.04500	YES	YES
71	a	245.61	0.51152	YES	YES
72	a	246.56	1.14314	YES	YES
73	a	251.22	0.15704	YES	YES
74	a	251.57	0.10723	YES	YES
75	a	271.23	0.82643	YES	YES
76	a	273.05	0.66746	YES	YES
77	a	278.97	0.26917	YES	YES
78	a	282.29	0.37343	YES	YES
79	a	290.38	6.84414	YES	YES
80	a	294.06	1.57701	YES	YES

81	a	296.90	0.65919	YES	YES
82	a	299.17	0.46582	YES	YES
83	a	301.19	0.09464	YES	YES
84	a	309.18	0.53520	YES	YES
85	a	312.65	0.75335	YES	YES
86	a	328.46	2.28795	YES	YES
87	a	328.87	0.11364	YES	YES
88	a	332.46	0.40608	YES	YES
89	a	333.11	0.26592	YES	YES
90	a	338.77	5.01345	YES	YES
91	a	339.91	0.21184	YES	YES
92	a	367.01	2.66491	YES	YES
93	a	373.10	6.70221	YES	YES
94	a	376.14	5.34559	YES	YES
95	a	376.89	0.41957	YES	YES
96	a	388.19	4.96552	YES	YES
97	a	394.90	0.06013	YES	YES
98	a	409.35	0.45299	YES	YES
99	a	412.81	1.12419	YES	YES
100	a	416.89	0.81242	YES	YES
101	a	422.16	4.56653	YES	YES
102	a	423.79	0.97311	YES	YES
103	a	429.06	0.41783	YES	YES
104	a	429.46	1.11052	YES	YES
105	a	430.69	0.38347	YES	YES
106	a	430.96	0.05545	YES	YES
107	a	431.53	0.23962	YES	YES
108	a	432.72	0.56550	YES	YES
109	a	433.08	0.48577	YES	YES
110	a	434.28	1.31251	YES	YES
111	a	437.66	0.35321	YES	YES
112	a	441.52	3.73634	YES	YES
113	a	454.69	5.05073	YES	YES
114	a	461.05	8.84081	YES	YES
115	a	467.22	3.68430	YES	YES
116	a	471.62	0.06916	YES	YES
117	a	492.33	0.27578	YES	YES
118	a	492.61	2.23009	YES	YES
119	a	492.88	5.91932	YES	YES
120	a	494.28	5.86912	YES	YES
121	a	495.43	4.59841	YES	YES
122	a	498.08	1.54977	YES	YES
123	a	502.94	0.66631	YES	YES
124	a	508.48	15.16806	YES	YES
125	a	513.41	10.55653	YES	YES
126	a	529.09	0.01138	YES	YES
127	a	553.46	0.33370	YES	YES
128	a	562.89	2.70682	YES	YES
129	a	624.84	23.07206	YES	YES
130	a	634.60	12.63261	YES	YES
131	a	639.91	11.89811	YES	YES
132	a	656.24	11.02699	YES	YES
133	a	683.38	21.42813	YES	YES
134	a	685.89	17.31273	YES	YES
135	a	695.97	4.47917	YES	YES
136	a	715.48	1.84625	YES	YES
137	a	720.26	4.74160	YES	YES
138	a	723.62	4.31126	YES	YES

139	a	726.85	9.41496	YES	YES
140	a	733.71	5.02470	YES	YES
141	a	735.44	0.67406	YES	YES
142	a	736.91	4.24518	YES	YES
143	a	759.07	71.75391	YES	YES
144	a	768.78	1.14913	YES	YES
145	a	769.99	1.25324	YES	YES
146	a	770.17	11.25731	YES	YES
147	a	771.58	3.52917	YES	YES
148	a	771.99	1.05462	YES	YES
149	a	773.65	2.86353	YES	YES
150	a	775.70	0.18018	YES	YES
151	a	776.93	16.63089	YES	YES
152	a	777.77	0.05285	YES	YES
153	a	779.28	0.29306	YES	YES
154	a	806.52	1.71134	YES	YES
155	a	807.68	2.27186	YES	YES
156	a	809.04	5.33120	YES	YES
157	a	810.30	6.96310	YES	YES
158	a	810.87	0.29670	YES	YES
159	a	813.05	3.92506	YES	YES
160	a	814.17	2.57013	YES	YES
161	a	814.90	6.80555	YES	YES
162	a	815.96	6.27226	YES	YES
163	a	831.34	5.70750	YES	YES
164	a	831.64	6.34133	YES	YES
165	a	836.13	0.58933	YES	YES
166	a	837.35	1.16247	YES	YES
167	a	838.23	1.89685	YES	YES
168	a	838.47	5.11491	YES	YES
169	a	838.81	0.37479	YES	YES
170	a	839.89	6.75768	YES	YES
171	a	847.87	18.31447	YES	YES
172	a	853.07	8.26678	YES	YES
173	a	860.74	1.10345	YES	YES
174	a	871.65	1.65964	YES	YES
175	a	873.53	2.79199	YES	YES
176	a	875.31	0.08796	YES	YES
177	a	876.55	3.64685	YES	YES
178	a	876.91	5.89798	YES	YES
179	a	877.22	1.26033	YES	YES
180	a	877.58	1.57565	YES	YES
181	a	878.42	0.65815	YES	YES
182	a	878.93	4.09007	YES	YES
183	a	879.64	6.42884	YES	YES
184	a	880.73	0.80055	YES	YES
185	a	880.91	2.79805	YES	YES
186	a	881.80	8.67259	YES	YES
187	a	882.11	2.48159	YES	YES
188	a	884.65	6.74062	YES	YES
189	a	885.57	0.24492	YES	YES
190	a	902.39	0.67939	YES	YES
191	a	902.58	2.89883	YES	YES
192	a	903.87	0.83576	YES	YES
193	a	904.14	2.77345	YES	YES
194	a	906.56	4.00806	YES	YES
195	a	909.75	1.97914	YES	YES
196	a	909.96	1.17585	YES	YES

197	a	910.38	0.91093	YES	YES
198	a	946.85	2.44999	YES	YES
199	a	980.34	0.26251	YES	YES
200	a	984.07	3.99604	YES	YES
201	a	986.24	8.54935	YES	YES
202	a	987.32	13.91200	YES	YES
203	a	987.74	8.30600	YES	YES
204	a	988.42	13.23125	YES	YES
205	a	988.89	0.56813	YES	YES
206	a	989.89	1.41283	YES	YES
207	a	990.22	4.89665	YES	YES
208	a	990.89	34.53075	YES	YES
209	a	1000.90	98.87886	YES	YES
210	a	1013.09	2.20512	YES	YES
211	a	1014.72	0.46449	YES	YES
212	a	1015.45	2.08736	YES	YES
213	a	1016.65	0.63845	YES	YES
214	a	1018.21	1.29002	YES	YES
215	a	1019.93	1.22370	YES	YES
216	a	1021.43	0.97613	YES	YES
217	a	1021.74	0.11567	YES	YES
218	a	1029.86	0.82943	YES	YES
219	a	1031.83	1.12411	YES	YES
220	a	1032.72	1.51296	YES	YES
221	a	1035.79	1.69386	YES	YES
222	a	1038.57	1.39020	YES	YES
223	a	1038.97	2.38596	YES	YES
224	a	1040.05	3.12643	YES	YES
225	a	1040.78	1.09810	YES	YES
226	a	1055.89	0.85316	YES	YES
227	a	1056.29	10.10825	YES	YES
228	a	1057.55	0.72793	YES	YES
229	a	1058.43	2.39379	YES	YES
230	a	1059.68	1.52838	YES	YES
231	a	1061.09	1.79783	YES	YES
232	a	1064.50	0.51157	YES	YES
233	a	1067.04	0.33898	YES	YES
234	a	1067.76	2.39959	YES	YES
235	a	1070.37	0.32646	YES	YES
236	a	1071.05	0.13868	YES	YES
237	a	1071.60	0.03605	YES	YES
238	a	1072.50	0.08947	YES	YES
239	a	1073.95	0.24338	YES	YES
240	a	1074.30	0.12119	YES	YES
241	a	1075.16	0.31203	YES	YES
242	a	1075.50	1.58717	YES	YES
243	a	1077.50	0.94182	YES	YES
244	a	1078.99	4.27311	YES	YES
245	a	1089.48	10.42999	YES	YES
246	a	1089.97	5.61933	YES	YES
247	a	1093.50	0.81174	YES	YES
248	a	1095.52	8.61655	YES	YES
249	a	1100.64	2.31652	YES	YES
250	a	1102.09	3.70556	YES	YES
251	a	1109.08	12.27781	YES	YES
252	a	1113.49	9.57926	YES	YES
253	a	1124.09	2.95119	YES	YES
254	a	1128.56	6.53264	YES	YES

255	a	1147.86	1.68490	YES	YES
256	a	1158.08	7.59712	YES	YES
257	a	1158.68	1.61646	YES	YES
258	a	1160.50	6.12638	YES	YES
259	a	1165.25	10.81855	YES	YES
260	a	1166.64	7.02136	YES	YES
261	a	1167.37	6.51811	YES	YES
262	a	1168.87	17.85515	YES	YES
263	a	1170.12	3.60429	YES	YES
264	a	1173.46	11.09114	YES	YES
265	a	1174.42	2.05383	YES	YES
266	a	1178.93	15.47291	YES	YES
267	a	1179.83	2.74198	YES	YES
268	a	1186.63	0.06670	YES	YES
269	a	1188.87	0.82858	YES	YES
270	a	1191.68	1.73708	YES	YES
271	a	1203.22	13.65281	YES	YES
272	a	1209.93	0.83850	YES	YES
273	a	1230.49	29.47514	YES	YES
274	a	1236.18	1.60952	YES	YES
275	a	1237.00	0.21001	YES	YES
276	a	1243.53	1.95589	YES	YES
277	a	1246.01	0.34431	YES	YES
278	a	1246.28	0.06206	YES	YES
279	a	1246.70	0.45376	YES	YES
280	a	1247.75	1.60197	YES	YES
281	a	1249.50	0.06672	YES	YES
282	a	1250.57	1.75693	YES	YES
283	a	1251.78	0.73118	YES	YES
284	a	1252.74	0.35714	YES	YES
285	a	1253.51	3.88688	YES	YES
286	a	1253.83	0.22940	YES	YES
287	a	1254.79	1.22306	YES	YES
288	a	1255.61	2.48661	YES	YES
289	a	1255.77	6.90483	YES	YES
290	a	1256.34	0.35269	YES	YES
291	a	1256.90	2.25421	YES	YES
292	a	1257.20	3.36498	YES	YES
293	a	1258.06	4.17809	YES	YES
294	a	1258.75	1.68608	YES	YES
295	a	1260.59	1.93117	YES	YES
296	a	1261.76	2.09896	YES	YES
297	a	1262.67	3.18118	YES	YES
298	a	1264.37	2.66242	YES	YES
299	a	1266.65	0.32699	YES	YES
300	a	1267.34	1.05643	YES	YES
301	a	1268.27	4.71448	YES	YES
302	a	1280.54	3.23371	YES	YES
303	a	1281.35	1.85969	YES	YES
304	a	1282.30	5.05766	YES	YES
305	a	1283.03	2.24200	YES	YES
306	a	1284.63	34.38267	YES	YES
307	a	1288.62	10.47002	YES	YES
308	a	1288.67	5.66510	YES	YES
309	a	1289.85	1.78743	YES	YES
310	a	1290.17	5.08379	YES	YES
311	a	1305.19	1.80436	YES	YES
312	a	1308.69	0.87495	YES	YES

313	a	1309.48	0.62993	YES	YES
314	a	1310.56	2.38791	YES	YES
315	a	1313.75	0.52287	YES	YES
316	a	1314.04	1.54090	YES	YES
317	a	1315.12	0.59613	YES	YES
318	a	1317.76	0.91331	YES	YES
319	a	1319.29	3.00418	YES	YES
320	a	1320.56	0.63355	YES	YES
321	a	1320.94	0.66667	YES	YES
322	a	1321.83	0.38142	YES	YES
323	a	1321.95	2.79196	YES	YES
324	a	1322.50	0.77542	YES	YES
325	a	1322.98	5.47640	YES	YES
326	a	1323.93	1.03778	YES	YES
327	a	1324.33	1.65428	YES	YES
328	a	1324.90	3.01723	YES	YES
329	a	1325.27	1.98987	YES	YES
330	a	1325.71	1.03272	YES	YES
331	a	1326.41	0.47338	YES	YES
332	a	1328.04	1.66101	YES	YES
333	a	1328.78	7.54193	YES	YES
334	a	1330.63	3.38873	YES	YES
335	a	1335.06	0.50268	YES	YES
336	a	1335.42	0.20240	YES	YES
337	a	1336.36	0.15592	YES	YES
338	a	1336.94	0.09168	YES	YES
339	a	1337.16	0.93988	YES	YES
340	a	1338.51	0.40430	YES	YES
341	a	1339.47	0.41361	YES	YES
342	a	1340.07	0.21330	YES	YES
343	a	1342.78	0.28024	YES	YES
344	a	1343.58	1.30020	YES	YES
345	a	1343.76	0.31543	YES	YES
346	a	1344.79	1.87666	YES	YES
347	a	1345.08	1.72385	YES	YES
348	a	1346.06	0.50204	YES	YES
349	a	1347.25	0.96089	YES	YES
350	a	1348.94	0.72199	YES	YES
351	a	1350.15	0.42149	YES	YES
352	a	1401.08	5.21632	YES	YES
353	a	1404.81	6.10689	YES	YES
354	a	1404.96	10.91607	YES	YES
355	a	1408.04	7.91324	YES	YES
356	a	1419.64	3.18844	YES	YES
357	a	1425.26	5.76167	YES	YES
358	a	1427.67	3.37461	YES	YES
359	a	1429.26	4.40269	YES	YES
360	a	1430.13	2.25649	YES	YES
361	a	1430.61	0.89570	YES	YES
362	a	1430.92	1.83760	YES	YES
363	a	1433.25	1.59873	YES	YES
364	a	1433.35	2.10289	YES	YES
365	a	1434.95	6.97411	YES	YES
366	a	1435.10	4.10804	YES	YES
367	a	1435.42	3.31503	YES	YES
368	a	1436.01	2.95877	YES	YES
369	a	1436.45	1.48500	YES	YES
370	a	1436.83	10.60773	YES	YES

371	a	1437.20	14.15485	YES	YES
372	a	1438.29	2.66461	YES	YES
373	a	1438.57	2.16093	YES	YES
374	a	1439.06	3.37378	YES	YES
375	a	1439.12	3.21533	YES	YES
376	a	1439.44	4.13037	YES	YES
377	a	1439.88	3.60098	YES	YES
378	a	1440.84	1.13260	YES	YES
379	a	1441.45	10.67172	YES	YES
380	a	1442.26	11.53659	YES	YES
381	a	1442.45	0.97896	YES	YES
382	a	1443.25	5.79672	YES	YES
383	a	1443.54	23.36880	YES	YES
384	a	1443.79	2.84544	YES	YES
385	a	1444.34	40.29990	YES	YES
386	a	1444.72	6.86648	YES	YES
387	a	1445.26	6.99984	YES	YES
388	a	1452.20	4.08383	YES	YES
389	a	1452.83	5.36981	YES	YES
390	a	1453.77	1.21517	YES	YES
391	a	1454.13	2.32131	YES	YES
392	a	1454.68	1.71358	YES	YES
393	a	1454.97	1.80201	YES	YES
394	a	1455.97	2.13369	YES	YES
395	a	1457.59	0.15540	YES	YES
396	a	1467.13	59.96759	YES	YES
397	a	1493.37	102.18542	YES	YES
398	a	1595.72	38.74484	YES	YES
399	a	1599.95	8.09981	YES	YES
400	a	2906.23	79.71464	YES	YES
401	a	2923.65	6.17520	YES	YES
402	a	2925.69	25.81122	YES	YES
403	a	2926.94	15.57237	YES	YES
404	a	2930.58	8.02371	YES	YES
405	a	2932.25	5.27004	YES	YES
406	a	2932.31	9.45373	YES	YES
407	a	2935.65	24.40571	YES	YES
408	a	2936.58	15.22563	YES	YES
409	a	2936.92	12.35572	YES	YES
410	a	2937.96	7.75656	YES	YES
411	a	2939.00	20.62902	YES	YES
412	a	2939.69	14.11926	YES	YES
413	a	2939.87	4.09850	YES	YES
414	a	2940.46	0.47549	YES	YES
415	a	2940.99	3.42411	YES	YES
416	a	2942.73	2.23952	YES	YES
417	a	2944.99	10.16371	YES	YES
418	a	2945.28	5.86270	YES	YES
419	a	2946.38	4.71905	YES	YES
420	a	2947.45	15.38540	YES	YES
421	a	2947.76	10.69048	YES	YES
422	a	2949.64	10.07481	YES	YES
423	a	2951.16	14.61290	YES	YES
424	a	2951.64	16.89879	YES	YES
425	a	2951.83	3.23530	YES	YES
426	a	2952.20	12.70162	YES	YES
427	a	2952.54	9.06917	YES	YES
428	a	2954.61	16.01382	YES	YES

429	a	2955.75	9.22594	YES	YES
430	a	2956.01	19.46034	YES	YES
431	a	2956.26	3.93175	YES	YES
432	a	2956.67	24.79091	YES	YES
433	a	2957.05	2.59178	YES	YES
434	a	2957.34	4.08898	YES	YES
435	a	2958.58	25.89049	YES	YES
436	a	2958.59	4.74816	YES	YES
437	a	2959.16	27.44936	YES	YES
438	a	2959.97	17.34939	YES	YES
439	a	2960.84	14.54776	YES	YES
440	a	2961.04	34.57532	YES	YES
441	a	2962.01	15.41602	YES	YES
442	a	2963.52	11.82741	YES	YES
443	a	2965.37	21.98298	YES	YES
444	a	2967.90	32.77388	YES	YES
445	a	2973.24	43.22628	YES	YES
446	a	2974.07	1.83443	YES	YES
447	a	2974.12	12.70932	YES	YES
448	a	2976.56	3.81890	YES	YES
449	a	2978.37	61.18297	YES	YES
450	a	2979.05	5.56606	YES	YES
451	a	2980.14	6.20727	YES	YES
452	a	2984.99	4.64114	YES	YES
453	a	2986.01	19.99754	YES	YES
454	a	2987.04	4.01674	YES	YES
455	a	2988.43	7.46438	YES	YES
456	a	2989.27	45.17671	YES	YES
457	a	2992.04	13.55021	YES	YES
458	a	2992.96	30.95991	YES	YES
459	a	3001.20	20.66176	YES	YES
460	a	3001.68	17.53289	YES	YES
461	a	3001.70	21.03987	YES	YES
462	a	3002.82	21.75911	YES	YES
463	a	3003.60	33.94098	YES	YES
464	a	3004.58	16.12583	YES	YES
465	a	3004.86	23.99746	YES	YES
466	a	3005.25	31.08476	YES	YES
467	a	3005.68	9.44777	YES	YES
468	a	3006.12	28.80285	YES	YES
469	a	3006.99	28.82756	YES	YES
470	a	3008.09	13.86350	YES	YES
471	a	3009.31	12.54867	YES	YES
472	a	3009.90	18.58574	YES	YES
473	a	3010.39	26.15594	YES	YES
474	a	3012.28	18.62396	YES	YES
475	a	3012.28	18.55461	YES	YES
476	a	3012.80	20.61718	YES	YES
477	a	3013.41	32.98388	YES	YES
478	a	3014.32	26.00829	YES	YES
479	a	3014.69	28.12310	YES	YES
480	a	3016.77	26.53832	YES	YES
481	a	3017.22	28.84337	YES	YES
482	a	3017.27	41.07044	YES	YES
483	a	3017.39	18.12035	YES	YES
484	a	3017.41	6.35591	YES	YES
485	a	3018.71	14.87619	YES	YES
486	a	3018.88	21.15699	YES	YES

487	a	3019.08	14.37036	YES	YES
488	a	3019.34	38.82634	YES	YES
489	a	3020.87	32.88084	YES	YES
490	a	3023.53	35.14515	YES	YES
491	a	3025.91	7.61648	YES	YES
492	a	3026.04	29.09705	YES	YES
493	a	3028.97	0.33402	YES	YES
494	a	3037.07	1.56058	YES	YES
495	a	3043.60	0.61988	YES	YES
496	a	3130.10	0.85223	YES	YES
497	a	3142.39	0.15017	YES	YES
498	a	3148.05	0.97311	YES	YES

$end

Double hybrid single point energy = -7778.505759193832 H
COSMO energy + OC correction = -7786.5541536446 H (in 3FB)

6.2.56 [{Ga(dcpe)}$_2$(F–$C_6F_2H_3$)]$^{2+}$ Transition State

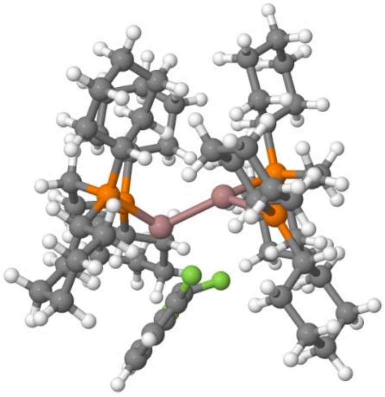

Method: (RI-)BP86(D3BJ)/def2-TZVPP
Symmetry: c1

Cartesian coordinates in Ångström:

Ga	-3.0405172	17.4565679	-2.9570182
Ga	-3.8177700	17.7773865	-0.6776452
P	-1.5441436	18.8702763	-4.2679258
P	-5.9314611	19.0492445	-0.6777892
P	-2.1491963	15.5999253	-4.2487806
P	-5.1899030	16.0192742	0.3275407
C	-4.7630186	21.3688178	-5.1991148
H	-4.7761844	21.9332783	-4.2513035
H	-5.8082175	21.1228818	-5.4362978
C	-1.1199006	14.2608409	-3.5050151
H	-0.8007016	13.6050699	-4.3332240
C	-4.2614913	15.0363071	1.5773264
H	-3.8762560	15.8337641	2.2411236
C	-4.4273451	15.8733957	-5.8325347
H	-3.8149996	16.2923304	-6.6474901
H	-4.6841449	16.7116948	-5.1618628
C	-6.7418474	19.2968006	-2.3258559
H	-6.0179759	19.9265113	-2.8715257
C	-7.1057255	18.0332095	0.3439100
H	-7.7433899	18.7160889	0.9218560

H	-7.7652349	17.4900892	-0.3460679
C	-2.6739602	22.5314787	-6.0319369
H	-2.2393203	23.1092976	-6.8585702
H	-2.5851456	23.1595719	-5.1303143
C	-1.8629408	21.2442405	-5.8221800
H	-0.8190248	21.4952199	-5.5908861
H	-1.8542337	20.6600984	-6.7567570
C	-3.9578861	20.0804714	-5.0001111
H	-4.0272143	19.4669076	-5.9135949
H	-4.3851155	19.4714201	-4.1850159
C	0.9386279	13.7501642	-2.1206524
H	1.3427965	13.0610341	-2.8797079
H	1.8032787	14.2040491	-1.6176757
C	-7.4558132	14.3070045	-0.1930889
H	-8.0612361	15.0032682	0.4039508
H	-7.1116351	13.5194757	0.4914784
C	-5.9448900	20.6904122	0.1618506
H	-7.0169992	20.9336676	0.2704929
C	0.3471805	20.5301467	-2.9234295
H	-0.3155409	21.3727781	-3.1612879
H	0.0418476	20.1426039	-1.9426128
C	1.8026025	21.0089123	-2.8365939
H	2.1030430	21.4537329	-3.7995596
H	1.8762221	21.8079778	-2.0861840
C	-6.9058971	17.9825795	-3.1035175
H	-5.9405176	17.4533286	-3.1589853
H	-7.6077385	17.3245419	-2.5637788
C	-4.1519177	22.2356653	-6.3058004
H	-4.2471012	21.7093419	-7.2697908
H	-4.7142003	23.1735906	-6.4070488
C	-8.0799412	20.0519309	-2.2415200
H	-7.9599642	21.0062391	-1.7122177
H	-8.8017986	19.4525355	-1.6628885
C	-5.2797603	21.7942080	-0.6721802
H	-5.7458274	21.8628411	-1.6658140
H	-4.2213827	21.5435988	-0.8329731
C	-3.2556779	13.6239544	-5.9725269
H	-2.7075348	12.8575568	-5.4075402
H	-2.5888168	13.9585124	-6.7841197
C	2.5996870	18.7101787	-3.4957991
H	2.9526003	19.0473650	-4.4841564
H	3.2342955	17.8592476	-3.2116187
C	-5.4311365	21.9482012	2.2889207
H	-6.4928841	22.1713037	2.4828090
H	-4.9431567	21.8760858	3.2705647
C	-8.7843970	19.0113582	-4.4505210
H	-9.1429779	19.2306944	-5.4651943
H	-9.5521909	18.3753747	-3.9798483
C	-5.1074268	14.0682150	2.4217537
H	-5.9793665	14.5773908	2.8555871
H	-5.4920075	13.2668455	1.7726597
C	0.0827637	12.9693524	-1.1209139
H	0.6749961	12.1736807	-0.6497247
H	-0.2341033	13.6488206	-0.3125333
C	-7.4609143	18.2423323	-4.5081198
H	-6.7200661	18.8208717	-5.0842429
H	-7.5914144	17.2866221	-5.0348101
C	2.7423573	19.8522892	-2.4869759

H	2.5012871	19.4812582	-1.4771818
H	3.7832751	20.2005322	-2.4537996
C	-4.8139312	23.0834167	1.4661405
H	-4.9658347	24.0432744	1.9776077
H	-3.7255427	22.9331624	1.4038118
C	-5.4108415	14.0679266	-1.6811728
H	-4.9813190	13.2751849	-1.0508098
H	-4.5652067	14.6169299	-2.1225312
C	-5.3301365	20.5968572	1.5679211
H	-4.2736587	20.2957393	1.4840543
H	-5.8397728	19.8256473	2.1642398
C	-6.2457580	15.0210234	-0.8174128
H	-6.6333856	15.8008255	-1.4967013
C	-5.3977371	23.1433405	0.0510798
C	-7.5019233	12.7295358	-2.1784763
H	-8.1304343	12.3188582	-2.9799571
H	-7.1676878	11.8713236	-1.5731432
C	-8.3184568	13.6821335	-1.2994645
H	-9.1696434	13.1549007	-0.8482283
H	-8.7431135	14.4859208	-1.9244388
C	-1.9763378	13.4581064	-2.5120782
H	-2.8382048	13.0033736	-3.0204284
H	-2.3784205	14.1567444	-1.7603941
C	-4.2481303	13.4435400	3.5296717
H	-3.9271806	14.2343048	4.2273479
H	-4.8578244	12.7399753	4.1126037
C	-5.6937167	15.2562155	-6.4355766
H	-6.2445427	16.0209894	-7.0004399
H	-6.3567156	14.9258508	-5.6190731
C	-3.0179384	12.7391945	2.9493110
H	-3.3451900	11.8787566	2.3419062
H	-2.3984094	12.3302814	3.7585335
C	-3.0397015	14.3233157	0.9713943
H	-3.3821201	13.5347051	0.2844522
H	-2.4389437	15.0329090	0.3838110
C	-8.6397630	20.3077601	-3.6486903
H	-9.6070858	20.8217941	-3.5693857
H	-7.9622788	20.9966290	-4.1796428
C	-4.5270106	13.0155457	-6.5827604
H	-4.2525528	12.1887980	-7.2514668
H	-5.1364234	12.5765066	-5.7759741
C	-6.2786674	13.4343993	-2.7729018
H	-5.6785064	12.7273287	-3.3642914
H	-6.6121339	14.2240343	-3.4678103
C	-5.3541299	14.0626184	-7.3343894
H	-6.2743212	13.6092479	-7.7264065
H	-4.7853853	14.4180532	-8.2094166
C	-1.1536397	12.3770477	-1.8017834
H	-1.7895742	11.8584506	-1.0699641
H	-0.8420203	11.6170723	-2.5362163
C	-2.1896898	13.6903731	2.0796656
H	-1.7654435	14.4914419	2.7048592
H	-1.3386728	13.1586262	1.6324184
F	-1.0532487	17.9629918	-0.8723778
C	0.2130598	19.4094526	-3.9618861
H	0.5063219	19.8093743	-4.9503089
C	1.1438725	18.2377277	-3.6172707
H	1.0846168	17.4552617	-4.3851464

```
H      0.8142074   17.7910017   -2.6675077
C      0.1216475   14.8472940   -2.8131871
H      0.7526267   15.3792057   -3.5370434
H     -0.2029904   15.5915563   -2.0664034
C     -1.1639756   16.3753695   -5.6086900
H     -0.1095374   16.2232222   -5.3504343
H     -1.3444943   15.8167968   -6.5381052
C     -1.4562283   17.8711483   -5.8265411
H     -2.4244214   17.9978492   -6.3289676
H     -0.6940487   18.3088576   -6.4859338
C     -3.6078600   14.8163714   -5.0698455
H     -4.2272519   14.4523459   -4.2340163
C     -2.4853023   20.4003556   -4.6979871
H     -2.4548486   20.9838126   -3.7620089
C     -6.3824487   17.0526283    1.2760671
H     -7.1033322   16.4184285    1.8087408
H     -5.7987193   17.5901911    2.0369996
H     -6.4612345   23.4283934    0.1045028
H     -4.8964745   23.9231109   -0.5387529
H     -1.8173989   22.0755633    0.7770395
C     -1.7127323   21.0156173    0.9993352
C     -1.8325088   20.1001645   -0.0245267
F     -2.1966781   20.5298574   -1.2749225
C     -1.7438350   18.6964542    0.1608306
C     -1.4425657   18.2975879    1.4892819
F     -1.3291211   16.9624684    1.7123440
C     -1.3419230   19.1839444    2.5444061
H     -1.1433518   18.7976708    3.5428087
C     -1.4536102   20.5586293    2.2987959
H     -1.3303551   21.2699636    3.1119156

SCF energy GEOOPT =  -7786.367203851 H
ZPE =    3789. kJ/mol
FREEH energy =  3987.74 kJ/mol
FREEH entropy =   1.69946 kJ/mol/K

$vibrational spectrum
#  mode     symmetry     wave number   IR intensity    selection rules
#                         cm**(-1)        km/mol          IR     RAMAN
     1        a          -149.44         0.00000        YES     YES
     2                     -0.00         0.00000         -       -
     3                     -0.00         0.00000         -       -
     4                     -0.00         0.00000         -       -
     5                     -0.00         0.00000         -       -
     6                      0.00         0.00000         -       -
     7                      0.00         0.00000         -       -
     8        a             5.14         0.04811        YES     YES
     9        a            11.38         0.06585        YES     YES
    10        a            15.96         0.06286        YES     YES
    11        a            19.57         0.42664        YES     YES
    12        a            25.31         0.02685        YES     YES
    13        a            28.92         0.16692        YES     YES
    14        a            31.38         0.07138        YES     YES
    15        a            32.46         0.51913        YES     YES
    16        a            33.98         0.61527        YES     YES
    17        a            39.18         0.52285        YES     YES
    18        a            41.46         0.44010        YES     YES
    19        a            42.96         0.51115        YES     YES
```

20	a	44.33	0.15345	YES	YES
21	a	49.64	0.08414	YES	YES
22	a	51.12	0.19748	YES	YES
23	a	52.18	2.17137	YES	YES
24	a	53.51	0.11163	YES	YES
25	a	56.32	0.32102	YES	YES
26	a	58.76	0.56001	YES	YES
27	a	60.39	0.28484	YES	YES
28	a	61.36	0.02557	YES	YES
29	a	65.33	1.07441	YES	YES
30	a	69.77	0.42308	YES	YES
31	a	71.67	0.07706	YES	YES
32	a	72.43	0.08690	YES	YES
33	a	73.24	0.02758	YES	YES
34	a	76.34	2.86011	YES	YES
35	a	78.48	0.85151	YES	YES
36	a	80.88	0.42040	YES	YES
37	a	84.79	0.25687	YES	YES
38	a	87.36	0.12720	YES	YES
39	a	93.55	0.59056	YES	YES
40	a	100.96	1.56296	YES	YES
41	a	108.43	0.41276	YES	YES
42	a	115.71	0.27824	YES	YES
43	a	120.62	1.23667	YES	YES
44	a	130.63	1.02476	YES	YES
45	a	133.86	1.69206	YES	YES
46	a	136.37	0.15343	YES	YES
47	a	138.86	0.89584	YES	YES
48	a	146.74	0.17521	YES	YES
49	a	148.19	2.42335	YES	YES
50	a	155.05	0.98369	YES	YES
51	a	161.96	4.23693	YES	YES
52	a	170.33	0.73811	YES	YES
53	a	177.39	0.94250	YES	YES
54	a	182.52	0.39133	YES	YES
55	a	185.48	1.81879	YES	YES
56	a	187.88	3.50293	YES	YES
57	a	191.60	0.73284	YES	YES
58	a	195.96	1.17161	YES	YES
59	a	212.10	0.59527	YES	YES
60	a	215.39	0.20870	YES	YES
61	a	216.61	0.23715	YES	YES
62	a	218.36	0.24355	YES	YES
63	a	220.96	0.22556	YES	YES
64	a	225.67	0.13717	YES	YES
65	a	227.34	0.77935	YES	YES
66	a	233.36	0.82256	YES	YES
67	a	236.56	1.05759	YES	YES
68	a	238.90	0.84310	YES	YES
69	a	240.52	0.23986	YES	YES
70	a	242.03	2.28975	YES	YES
71	a	244.26	5.66321	YES	YES
72	a	245.76	0.08899	YES	YES
73	a	249.09	9.01947	YES	YES
74	a	252.35	1.29733	YES	YES
75	a	255.56	0.19337	YES	YES
76	a	261.90	12.50184	YES	YES
77	a	271.51	0.65823	YES	YES

78	a	276.75	0.19982	YES	YES
79	a	285.45	0.46319	YES	YES
80	a	290.16	0.86927	YES	YES
81	a	293.91	3.73673	YES	YES
82	a	299.76	47.38744	YES	YES
83	a	304.63	0.49117	YES	YES
84	a	310.79	2.13126	YES	YES
85	a	312.06	0.21147	YES	YES
86	a	329.61	0.37685	YES	YES
87	a	329.86	4.93162	YES	YES
88	a	331.94	0.23829	YES	YES
89	a	332.35	1.47099	YES	YES
90	a	335.83	3.01978	YES	YES
91	a	338.36	0.87426	YES	YES
92	a	373.61	15.93649	YES	YES
93	a	374.80	0.93686	YES	YES
94	a	380.19	0.67655	YES	YES
95	a	382.67	0.13224	YES	YES
96	a	391.07	3.28055	YES	YES
97	a	396.98	1.47093	YES	YES
98	a	403.81	1.23393	YES	YES
99	a	414.08	3.49376	YES	YES
100	a	417.00	1.77487	YES	YES
101	a	422.36	5.33945	YES	YES
102	a	424.44	3.32921	YES	YES
103	a	428.01	4.48618	YES	YES
104	a	428.25	25.53819	YES	YES
105	a	430.46	0.41083	YES	YES
106	a	430.95	0.18715	YES	YES
107	a	431.74	2.09222	YES	YES
108	a	432.67	5.86298	YES	YES
109	a	433.25	18.44762	YES	YES
110	a	434.06	34.20768	YES	YES
111	a	435.17	1.01774	YES	YES
112	a	439.34	0.98945	YES	YES
113	a	442.12	11.93535	YES	YES
114	a	459.29	8.20557	YES	YES
115	a	461.17	4.65384	YES	YES
116	a	472.16	8.58703	YES	YES
117	a	478.12	44.96379	YES	YES
118	a	489.24	8.02209	YES	YES
119	a	491.48	6.80334	YES	YES
120	a	493.38	2.54210	YES	YES
121	a	494.96	1.89657	YES	YES
122	a	495.18	4.45044	YES	YES
123	a	500.24	3.67476	YES	YES
124	a	502.03	1.57620	YES	YES
125	a	511.13	3.30243	YES	YES
126	a	512.05	17.19318	YES	YES
127	a	535.15	1.39228	YES	YES
128	a	566.94	5.09832	YES	YES
129	a	606.83	22.89780	YES	YES
130	a	627.13	22.26769	YES	YES
131	a	633.08	12.80131	YES	YES
132	a	641.50	14.89177	YES	YES
133	a	652.84	93.28984	YES	YES
134	a	663.74	12.33404	YES	YES
135	a	697.69	3.77228	YES	YES

136	a	706.24	5.89367	YES	YES
137	a	721.06	10.55964	YES	YES
138	a	721.73	2.44552	YES	YES
139	a	725.93	4.56390	YES	YES
140	a	730.29	16.34611	YES	YES
141	a	731.26	1.43670	YES	YES
142	a	734.25	3.40261	YES	YES
143	a	744.38	60.44258	YES	YES
144	a	747.75	0.85171	YES	YES
145	a	771.45	0.25750	YES	YES
146	a	772.81	3.60524	YES	YES
147	a	773.25	0.08249	YES	YES
148	a	773.36	0.93971	YES	YES
149	a	774.94	0.64726	YES	YES
150	a	776.37	6.89962	YES	YES
151	a	776.67	4.55419	YES	YES
152	a	779.03	1.64768	YES	YES
153	a	780.17	0.01951	YES	YES
154	a	787.42	17.17656	YES	YES
155	a	807.20	0.60665	YES	YES
156	a	808.17	3.73019	YES	YES
157	a	809.54	2.57695	YES	YES
158	a	810.40	2.60208	YES	YES
159	a	811.58	1.85049	YES	YES
160	a	812.02	6.22297	YES	YES
161	a	812.26	5.91823	YES	YES
162	a	817.60	4.87135	YES	YES
163	a	831.14	6.95938	YES	YES
164	a	833.33	3.33995	YES	YES
165	a	833.65	9.42972	YES	YES
166	a	836.25	2.75121	YES	YES
167	a	837.21	1.31908	YES	YES
168	a	838.10	5.19506	YES	YES
169	a	840.00	3.62669	YES	YES
170	a	840.65	3.42700	YES	YES
171	a	848.02	15.57644	YES	YES
172	a	854.23	16.34644	YES	YES
173	a	862.97	0.96770	YES	YES
174	a	874.68	3.94379	YES	YES
175	a	876.17	2.19129	YES	YES
176	a	876.57	2.31929	YES	YES
177	a	876.74	2.49335	YES	YES
178	a	878.18	4.06717	YES	YES
179	a	878.44	4.11355	YES	YES
180	a	879.08	1.78268	YES	YES
181	a	879.29	6.29807	YES	YES
182	a	880.03	3.33874	YES	YES
183	a	880.36	1.03902	YES	YES
184	a	881.26	0.46363	YES	YES
185	a	882.33	8.63225	YES	YES
186	a	882.73	2.42015	YES	YES
187	a	883.56	5.39853	YES	YES
188	a	883.99	1.02540	YES	YES
189	a	887.78	7.43431	YES	YES
190	a	902.12	0.77654	YES	YES
191	a	903.27	1.72536	YES	YES
192	a	904.38	2.74154	YES	YES
193	a	905.02	1.14643	YES	YES

194	a	908.19	1.85629	YES	YES
195	a	909.42	3.35115	YES	YES
196	a	910.60	2.13702	YES	YES
197	a	911.15	1.05661	YES	YES
198	a	922.54	9.40379	YES	YES
199	a	964.43	66.73125	YES	YES
200	a	982.78	0.64485	YES	YES
201	a	984.81	3.07999	YES	YES
202	a	987.71	9.44236	YES	YES
203	a	987.82	8.56475	YES	YES
204	a	988.25	7.41958	YES	YES
205	a	988.66	3.45903	YES	YES
206	a	989.66	21.46842	YES	YES
207	a	989.79	4.48137	YES	YES
208	a	991.68	17.58193	YES	YES
209	a	992.08	8.20394	YES	YES
210	a	1001.00	4.94449	YES	YES
211	a	1012.40	1.67654	YES	YES
212	a	1014.86	2.07115	YES	YES
213	a	1015.30	0.75162	YES	YES
214	a	1016.51	0.91408	YES	YES
215	a	1016.91	0.47731	YES	YES
216	a	1018.26	1.18133	YES	YES
217	a	1018.57	1.21149	YES	YES
218	a	1021.93	0.63742	YES	YES
219	a	1030.77	1.17479	YES	YES
220	a	1032.50	1.20794	YES	YES
221	a	1033.57	0.97394	YES	YES
222	a	1034.27	0.26296	YES	YES
223	a	1038.23	2.89181	YES	YES
224	a	1040.21	1.33879	YES	YES
225	a	1040.75	2.97247	YES	YES
226	a	1041.82	0.98345	YES	YES
227	a	1055.95	0.41821	YES	YES
228	a	1056.93	2.11367	YES	YES
229	a	1058.12	1.14709	YES	YES
230	a	1058.26	6.79761	YES	YES
231	a	1058.82	1.58094	YES	YES
232	a	1062.83	1.76059	YES	YES
233	a	1064.30	0.04843	YES	YES
234	a	1065.09	1.40852	YES	YES
235	a	1067.33	1.98631	YES	YES
236	a	1069.58	0.16241	YES	YES
237	a	1070.33	0.15865	YES	YES
238	a	1070.68	0.27247	YES	YES
239	a	1071.00	0.14725	YES	YES
240	a	1072.10	0.11898	YES	YES
241	a	1072.27	1.08907	YES	YES
242	a	1075.35	0.25381	YES	YES
243	a	1075.82	0.15458	YES	YES
244	a	1076.37	2.37005	YES	YES
245	a	1079.35	2.33313	YES	YES
246	a	1090.05	13.30763	YES	YES
247	a	1091.60	2.50431	YES	YES
248	a	1096.05	15.88035	YES	YES
249	a	1098.77	7.03481	YES	YES
250	a	1101.53	5.51952	YES	YES
251	a	1103.23	2.20027	YES	YES

252	a	1106.75	10.60698	YES	YES
253	a	1116.49	12.42936	YES	YES
254	a	1123.40	1.34610	YES	YES
255	a	1130.86	9.51299	YES	YES
256	a	1137.44	1.02171	YES	YES
257	a	1157.77	5.60152	YES	YES
258	a	1159.41	4.17320	YES	YES
259	a	1160.56	8.19828	YES	YES
260	a	1166.33	6.56346	YES	YES
261	a	1168.19	14.22542	YES	YES
262	a	1169.27	8.35762	YES	YES
263	a	1170.48	5.09376	YES	YES
264	a	1171.24	6.28468	YES	YES
265	a	1172.61	20.27545	YES	YES
266	a	1178.36	2.19617	YES	YES
267	a	1179.54	40.22246	YES	YES
268	a	1185.17	10.24642	YES	YES
269	a	1188.45	2.00990	YES	YES
270	a	1188.73	0.32359	YES	YES
271	a	1192.77	1.60246	YES	YES
272	a	1202.46	19.00034	YES	YES
273	a	1212.22	1.73811	YES	YES
274	a	1226.47	45.19314	YES	YES
275	a	1237.58	0.97917	YES	YES
276	a	1240.61	0.23494	YES	YES
277	a	1241.65	0.63625	YES	YES
278	a	1244.68	1.21778	YES	YES
279	a	1246.21	0.28557	YES	YES
280	a	1247.08	0.62674	YES	YES
281	a	1247.76	1.85792	YES	YES
282	a	1248.58	0.98539	YES	YES
283	a	1251.00	1.21374	YES	YES
284	a	1251.81	1.69147	YES	YES
285	a	1252.41	0.98231	YES	YES
286	a	1253.09	5.01688	YES	YES
287	a	1254.00	0.40924	YES	YES
288	a	1254.88	4.83232	YES	YES
289	a	1255.46	5.44246	YES	YES
290	a	1256.35	6.73982	YES	YES
291	a	1257.10	2.68335	YES	YES
292	a	1257.97	6.75608	YES	YES
293	a	1258.41	1.18140	YES	YES
294	a	1259.06	1.01054	YES	YES
295	a	1259.41	10.96197	YES	YES
296	a	1261.42	3.00575	YES	YES
297	a	1261.79	0.87001	YES	YES
298	a	1262.61	3.61246	YES	YES
299	a	1264.05	1.43383	YES	YES
300	a	1264.49	1.22370	YES	YES
301	a	1268.00	2.03914	YES	YES
302	a	1269.87	1.46876	YES	YES
303	a	1280.32	2.76638	YES	YES
304	a	1280.78	2.92477	YES	YES
305	a	1282.24	2.82851	YES	YES
306	a	1283.45	3.63931	YES	YES
307	a	1286.56	5.94521	YES	YES
308	a	1286.99	1.96813	YES	YES
309	a	1290.21	4.90720	YES	YES

310	a	1290.73	3.86342	YES	YES
311	a	1304.03	0.02677	YES	YES
312	a	1307.14	1.55662	YES	YES
313	a	1309.90	0.59046	YES	YES
314	a	1310.44	2.78469	YES	YES
315	a	1312.97	2.36351	YES	YES
316	a	1313.97	1.53434	YES	YES
317	a	1315.15	1.98774	YES	YES
318	a	1317.38	2.49577	YES	YES
319	a	1318.25	1.76098	YES	YES
320	a	1320.45	1.34332	YES	YES
321	a	1321.03	1.53622	YES	YES
322	a	1321.67	1.77419	YES	YES
323	a	1322.56	1.39780	YES	YES
324	a	1322.96	2.46140	YES	YES
325	a	1323.37	3.98200	YES	YES
326	a	1323.68	3.71926	YES	YES
327	a	1324.47	4.43036	YES	YES
328	a	1325.21	0.31294	YES	YES
329	a	1325.39	2.29154	YES	YES
330	a	1326.34	0.72500	YES	YES
331	a	1326.63	1.17325	YES	YES
332	a	1327.69	1.86472	YES	YES
333	a	1330.82	1.08548	YES	YES
334	a	1332.09	2.94623	YES	YES
335	a	1335.58	0.82215	YES	YES
336	a	1335.74	0.27614	YES	YES
337	a	1336.43	0.43266	YES	YES
338	a	1337.17	0.32783	YES	YES
339	a	1337.94	0.88156	YES	YES
340	a	1338.42	0.38658	YES	YES
341	a	1339.02	0.51614	YES	YES
342	a	1339.37	0.46549	YES	YES
343	a	1341.97	11.41899	YES	YES
344	a	1343.37	0.44898	YES	YES
345	a	1343.82	1.05559	YES	YES
346	a	1345.22	1.57128	YES	YES
347	a	1346.14	2.73810	YES	YES
348	a	1347.13	1.11503	YES	YES
349	a	1348.41	0.89344	YES	YES
350	a	1349.52	0.29182	YES	YES
351	a	1350.55	0.77261	YES	YES
352	a	1386.03	2.40556	YES	YES
353	a	1402.54	4.14435	YES	YES
354	a	1406.26	7.35164	YES	YES
355	a	1407.63	9.40696	YES	YES
356	a	1409.63	8.91475	YES	YES
357	a	1421.48	4.09446	YES	YES
358	a	1423.19	3.85962	YES	YES
359	a	1425.66	5.27491	YES	YES
360	a	1428.12	4.90203	YES	YES
361	a	1429.48	0.29926	YES	YES
362	a	1431.19	2.73928	YES	YES
363	a	1432.72	7.65330	YES	YES
364	a	1433.31	6.85453	YES	YES
365	a	1433.99	1.47464	YES	YES
366	a	1434.58	4.02614	YES	YES
367	a	1435.08	3.12712	YES	YES

368	a	1435.27	1.06835	YES	YES
369	a	1436.34	10.23732	YES	YES
370	a	1436.66	10.58684	YES	YES
371	a	1436.73	3.16952	YES	YES
372	a	1438.10	10.65596	YES	YES
373	a	1438.35	2.18539	YES	YES
374	a	1438.50	1.21473	YES	YES
375	a	1438.68	5.23856	YES	YES
376	a	1439.98	11.77509	YES	YES
377	a	1440.21	2.79508	YES	YES
378	a	1440.25	0.95838	YES	YES
379	a	1440.77	1.99553	YES	YES
380	a	1441.04	7.98539	YES	YES
381	a	1441.55	6.11898	YES	YES
382	a	1442.61	7.57857	YES	YES
383	a	1443.64	3.93980	YES	YES
384	a	1444.11	26.90438	YES	YES
385	a	1445.04	12.81260	YES	YES
386	a	1445.76	12.11694	YES	YES
387	a	1446.12	25.70348	YES	YES
388	a	1446.62	11.39076	YES	YES
389	a	1451.77	50.35413	YES	YES
390	a	1452.35	4.19711	YES	YES
391	a	1453.57	11.77305	YES	YES
392	a	1454.07	3.36613	YES	YES
393	a	1454.58	2.15797	YES	YES
394	a	1455.53	4.22482	YES	YES
395	a	1456.79	3.50328	YES	YES
396	a	1457.66	1.15722	YES	YES
397	a	1458.17	1.50995	YES	YES
398	a	1536.93	12.02578	YES	YES
399	a	1572.07	6.27392	YES	YES
400	a	2913.14	2.95698	YES	YES
401	a	2917.59	78.41114	YES	YES
402	a	2919.07	1.89094	YES	YES
403	a	2919.27	11.05924	YES	YES
404	a	2919.90	64.90231	YES	YES
405	a	2929.35	4.58384	YES	YES
406	a	2935.49	2.23724	YES	YES
407	a	2936.89	15.57665	YES	YES
408	a	2938.11	8.98854	YES	YES
409	a	2939.31	4.42667	YES	YES
410	a	2942.22	3.38822	YES	YES
411	a	2942.36	11.92898	YES	YES
412	a	2944.81	12.11083	YES	YES
413	a	2945.25	11.77649	YES	YES
414	a	2945.65	2.44059	YES	YES
415	a	2945.89	13.83335	YES	YES
416	a	2946.06	16.04890	YES	YES
417	a	2946.34	5.45277	YES	YES
418	a	2947.33	12.01463	YES	YES
419	a	2947.95	7.53270	YES	YES
420	a	2948.53	6.73614	YES	YES
421	a	2948.57	1.00605	YES	YES
422	a	2951.57	10.83062	YES	YES
423	a	2952.18	21.11595	YES	YES
424	a	2952.50	4.08934	YES	YES
425	a	2952.90	11.27811	YES	YES

426	a	2954.61	12.36423	YES	YES
427	a	2955.32	22.48248	YES	YES
428	a	2956.86	2.22453	YES	YES
429	a	2956.89	1.86422	YES	YES
430	a	2957.46	12.19842	YES	YES
431	a	2957.62	16.53669	YES	YES
432	a	2958.26	3.28597	YES	YES
433	a	2959.68	29.29745	YES	YES
434	a	2959.72	24.42843	YES	YES
435	a	2960.49	13.81432	YES	YES
436	a	2960.95	11.38779	YES	YES
437	a	2961.19	15.72034	YES	YES
438	a	2961.65	29.07013	YES	YES
439	a	2962.86	38.75410	YES	YES
440	a	2965.04	16.92164	YES	YES
441	a	2965.63	14.77587	YES	YES
442	a	2966.66	13.67397	YES	YES
443	a	2969.41	31.43174	YES	YES
444	a	2970.59	50.25149	YES	YES
445	a	2972.33	16.05704	YES	YES
446	a	2974.07	13.97298	YES	YES
447	a	2974.61	13.08948	YES	YES
448	a	2978.07	12.73935	YES	YES
449	a	2980.44	27.67560	YES	YES
450	a	2982.26	2.64376	YES	YES
451	a	2983.43	4.84150	YES	YES
452	a	2984.46	8.02731	YES	YES
453	a	2985.55	6.22243	YES	YES
454	a	2987.46	7.68271	YES	YES
455	a	2992.07	22.13477	YES	YES
456	a	2998.57	27.26198	YES	YES
457	a	2999.46	21.19911	YES	YES
458	a	3000.80	28.09124	YES	YES
459	a	3002.01	7.40965	YES	YES
460	a	3006.22	35.28654	YES	YES
461	a	3006.46	17.45248	YES	YES
462	a	3006.92	26.72857	YES	YES
463	a	3007.85	24.92404	YES	YES
464	a	3008.25	19.01957	YES	YES
465	a	3008.50	8.29969	YES	YES
466	a	3009.16	33.01192	YES	YES
467	a	3009.28	14.20836	YES	YES
468	a	3010.97	11.36308	YES	YES
469	a	3012.18	15.36962	YES	YES
470	a	3013.41	16.84390	YES	YES
471	a	3013.84	19.55684	YES	YES
472	a	3014.56	25.09808	YES	YES
473	a	3014.76	28.24371	YES	YES
474	a	3014.92	19.20022	YES	YES
475	a	3015.40	18.51967	YES	YES
476	a	3015.68	25.53449	YES	YES
477	a	3015.82	20.61505	YES	YES
478	a	3016.20	23.47197	YES	YES
479	a	3016.38	22.04685	YES	YES
480	a	3016.77	26.46980	YES	YES
481	a	3017.16	26.72171	YES	YES
482	a	3017.99	14.39976	YES	YES
483	a	3018.14	16.51174	YES	YES

484	a	3018.30	25.32623	YES	YES
485	a	3018.42	42.37943	YES	YES
486	a	3018.92	15.21071	YES	YES
487	a	3019.11	15.78008	YES	YES
488	a	3021.87	23.16229	YES	YES
489	a	3023.50	28.64355	YES	YES
490	a	3023.97	35.88206	YES	YES
491	a	3028.30	4.60709	YES	YES
492	a	3029.52	1.47139	YES	YES
493	a	3034.36	13.56738	YES	YES
494	a	3037.06	1.25476	YES	YES
495	a	3047.19	0.59201	YES	YES
496	a	3130.42	0.68829	YES	YES
497	a	3135.61	0.45851	YES	YES
498	a	3150.37	1.18726	YES	YES

$end

Double hybrid single point energy = -7778.466427138916 H
COSMO energy + OC correction = -7786.5209100887 H (in 3FB)

6.2.57 $[F\{Ga(dcpe)\}_2(C_6F_2H_3)]^{2+}$ (*syn*-periplanar)

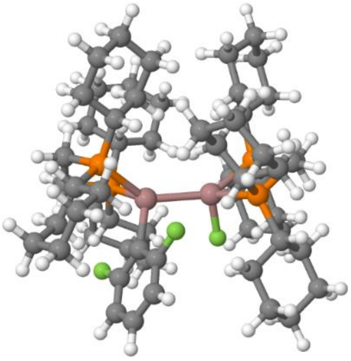

Method: (RI-)BP86(D3BJ)/def2-TZVPP
Symmetry: c1

Cartesian coordinates in Ångström:

Ga	0.5442265	0.2060604	-1.1454217
Ga	0.3126102	0.0421497	1.2935460
P	0.2803816	2.2649656	-2.4194931
P	-1.4630293	0.5749113	2.8894687
P	-0.1138980	-0.9760975	-3.1852066
P	0.2375358	-2.1670839	2.2363857
C	-2.9171949	3.8821162	-0.2826805
H	-2.3470517	4.3278394	0.5488697
H	-3.7937725	3.3880123	0.1622552
C	1.0947084	-2.2419480	-3.7735841
H	0.7026820	-2.6055017	-4.7392021
C	1.7949074	-3.1455003	2.2567399
H	2.3791765	-2.6535766	3.0490758
C	-2.8754849	-0.7088116	-2.8822382
H	-2.8851976	0.0467144	-3.6838903
H	-2.6511020	-0.1774996	-1.9428587
C	-3.1300421	0.8838065	2.1665076
H	-2.9970588	1.8444633	1.6404474

C	-1.5836735	-0.9532082	3.9425838
H	-1.8490794	-0.6434901	4.9623492
H	-2.4238037	-1.5514308	3.5650626
C	-2.1634998	5.6491229	-1.9286868
H	-2.5008436	6.4093833	-2.6457700
H	-1.5561082	6.1758375	-1.1748244
C	-1.2858333	4.6161708	-2.6491928
H	-0.4165338	5.1102847	-3.1052341
H	-1.8650161	4.1630612	-3.4696165
C	-2.0348574	2.8433046	-0.9849211
H	-2.6369079	2.3071314	-1.7381583
H	-1.6759886	2.0883959	-0.2675659
C	3.4810435	-2.7640320	-4.4231644
H	3.1816246	-3.1638284	-5.4058986
H	4.4804836	-2.3276277	-4.5541981
C	-1.7734869	-4.2043136	2.4095578
H	-2.0209780	-3.8158671	3.4071667
H	-1.0270920	-4.9960803	2.5576915
C	-1.1813907	1.9336859	4.1129215
H	-2.0349350	1.8320741	4.8075627
C	2.6575583	3.4272021	-1.5948547
H	2.0697617	4.0995745	-0.9565788
H	2.8295421	2.5008171	-1.0241331
C	4.0076372	4.0691953	-1.9332312
H	3.8400124	5.0449838	-2.4179131
H	4.5531636	4.2710402	-1.0008713
C	-3.5101528	-0.1714880	1.1183992
H	-2.7062917	-0.2741295	0.3709269
H	-3.6196634	-1.1528806	1.6096774
C	-3.3642617	4.9852975	-1.2475204
H	-4.0253044	4.5503781	-2.0155139
H	-3.9609034	5.7364077	-0.7128601
C	-4.2424285	1.0560882	3.2147095
H	-3.9778709	1.8379447	3.9387139
H	-4.3592757	0.1186553	3.7828108
C	-1.2558902	3.3200906	3.4545556
H	-2.2276510	3.4544009	2.9576256
H	-0.4869183	3.3968373	2.6748175
C	-2.0960531	-2.5469186	-4.4600229
H	-1.3392131	-3.3256948	-4.6229508
H	-2.0547189	-1.8743118	-5.3324042
C	4.0541220	2.8466700	-4.1351990
H	3.8859090	3.7724070	-4.7095225
H	4.6345371	2.1770720	-4.7847789
C	0.2866307	2.8655334	5.9485184
H	-0.5178433	2.8116597	6.7001140
H	1.2316653	2.7182648	6.4886525
C	-5.9565692	0.3739695	1.4746070
H	-6.8879500	0.6697025	0.9735840
H	-6.1608438	-0.5916630	1.9661962
C	1.6503875	-4.6326150	2.6215391
H	1.1318159	-4.7516433	3.5834133
H	1.0427381	-5.1446524	1.8598684
C	3.5211263	-3.9029393	-3.4008262
H	4.2167412	-4.6868015	-3.7285805
H	3.9064520	-3.5172908	-2.4420806
C	-4.8392322	0.1868250	0.4438128
H	-4.7082149	1.1161579	-0.1350693

H	-5.1102703	-0.5967449	-0.2749730
C	4.8332647	3.1720486	-2.8583849
H	5.0811568	2.2357629	-2.3322163
H	5.7861379	3.6566026	-3.1095631
C	0.2591603	4.2363240	5.2677901
H	0.3802900	5.0337525	6.0128461
H	1.1144727	4.3152318	4.5759584
C	-0.8921813	-3.5998345	0.0987352
H	-0.0908168	-4.3525637	0.1290398
H	-0.5164479	-2.7686791	-0.5168343
C	0.1101794	1.7367555	4.9249764
H	0.9723473	1.7291368	4.2459229
H	0.0944132	0.7655783	5.4410520
C	-1.1897601	-3.0947543	1.5167481
H	-1.9604386	-2.3116016	1.4133866
C	-1.0456832	4.4320853	4.4912546
C	-2.7328597	-5.3255930	0.3527911
H	-3.6457512	-5.7300240	-0.1043717
H	-2.0172210	-6.1613739	0.4147971
C	-3.0284148	-4.8031349	1.7610462
H	-3.4223636	-5.6071626	2.3968517
H	-3.8151633	-4.0312215	1.7080471
C	1.1240376	-3.4062773	-2.7693076
H	0.1219465	-3.8438039	-2.6532242
H	1.4190462	-3.0026137	-1.7875201
C	3.0352414	-5.2939688	2.6792697
H	3.6150042	-4.8467679	3.5028651
H	2.9196342	-6.3594506	2.9201458
C	-4.2608838	-1.3630570	-2.8162492
H	-5.0234092	-0.5923358	-2.6398396
H	-4.2950063	-2.0482027	-1.9528282
C	3.7967735	-5.1173096	1.3617462
H	3.2631058	-5.6529730	0.5582094
H	4.7908852	-5.5779418	1.4352046
C	2.5532836	-2.9553617	0.9279084
H	1.9650223	-3.4014905	0.1109308
H	2.6656104	-1.8877496	0.6900896
C	-5.5714215	1.4099100	2.5332379
H	-6.3605197	1.4987511	3.2917512
H	-5.4778972	2.4023043	2.0620247
C	-3.4892696	-3.1861240	-4.3823161
H	-3.7002307	-3.7160747	-5.3206816
H	-3.4916480	-3.9473308	-3.5851052
C	-2.1463819	-4.2190567	-0.5278486
H	-1.9089949	-4.6112135	-1.5278967
H	-2.9056939	-3.4305088	-0.6678707
C	-4.5723565	-2.1439319	-4.0957494
H	-5.5548756	-2.6274459	-4.0140074
H	-4.6401511	-1.4422143	-4.9434166
C	2.1242332	-4.4898693	-3.1863705
H	2.1452245	-5.2787990	-2.4209719
H	1.7769989	-4.9666766	-4.1172313
C	3.9233850	-3.6374521	0.9877998
H	4.5480690	-3.1189909	1.7327560
H	4.4309746	-3.5224873	0.0198499
F	2.3602717	-0.0602091	-1.2512322
C	1.8703525	3.0793486	-2.8716632
H	1.5969119	4.0108920	-3.3982040

```
C      2.7017135    2.1948258   -3.8187448
H      2.1543267    2.0202408   -4.7561568
H      2.8688190    1.2192521   -3.3367479
C      2.5034783   -1.6612869   -3.9957172
H      2.4803374   -0.8762496   -4.7644380
H      2.8509223   -1.1951596   -3.0626033
C     -0.1512782    0.3421451   -4.4794368
H      0.8611050    0.3744110   -4.8987361
H     -0.8200884    0.0194473   -5.2897348
C     -0.5607673    1.7360985   -3.9776140
H     -1.6374309    1.7670430   -3.7636426
H     -0.3715062    2.4857031   -4.7584388
C     -1.7858282   -1.7538959   -3.1790267
H     -1.7566945   -2.4588496   -2.3346322
C     -0.8342228    3.5220368   -1.6669781
H     -0.2180904    3.9872596   -0.8825825
C     -0.2913862   -1.7896570    3.9570981
H     -0.4381099   -2.7138273    4.5319495
H      0.5350205   -1.2376464    4.4250522
H     -1.8931171    4.4377348    5.1960605
H     -1.0552462    5.4067982    3.9842014
H      2.7282525    4.4654320    1.7655581
C      2.8045142    3.4213619    2.0616612
C      1.8505860    2.5004833    1.6559324
F      0.7997570    2.9644934    0.8890208
C      1.8399123    1.1485339    1.9848864
C      2.9067633    0.7598332    2.7907743
F      2.9576319   -0.5416930    3.2119382
C      3.9102282    1.6120540    3.2369254
H      4.7093421    1.2286959    3.8682301
C      3.8507423    2.9559237    2.8630508
H      4.6208372    3.6462084    3.2027044

SCF energy GEOOPT =  -7786.496718414 H
ZPE =    3798. kJ/mol
FREEH energy =  3996.20 kJ/mol
FREEH entropy =   1.66860 kJ/mol/K

$vibrational spectrum
#  mode     symmetry     wave number   IR intensity    selection rules
#                         cm**(-1)        km/mol          IR     RAMAN
     1                      -0.00         0.00000         -        -
     2                       0.00         0.00000         -        -
     3                       0.00         0.00000         -        -
     4                       0.00         0.00000         -        -
     5                       0.00         0.00000         -        -
     6                       0.00         0.00000         -        -
     7        a             14.29         0.01854        YES      YES
     8        a             21.58         0.00057        YES      YES
     9        a             24.37         0.01619        YES      YES
    10        a             25.74         0.05350        YES      YES
    11        a             31.98         0.06187        YES      YES
    12        a             32.62         0.00486        YES      YES
    13        a             34.82         0.01171        YES      YES
    14        a             35.71         0.03088        YES      YES
    15        a             38.04         0.06232        YES      YES
    16        a             40.42         0.02464        YES      YES
    17        a             43.47         0.03024        YES      YES
```

18	a	45.93	0.03881	YES	YES
19	a	47.80	0.04216	YES	YES
20	a	49.93	0.03929	YES	YES
21	a	50.49	0.04892	YES	YES
22	a	54.15	0.02313	YES	YES
23	a	56.13	0.18098	YES	YES
24	a	58.08	0.15066	YES	YES
25	a	60.70	0.24253	YES	YES
26	a	64.60	0.13459	YES	YES
27	a	66.35	0.53536	YES	YES
28	a	67.28	0.27107	YES	YES
29	a	69.01	0.33232	YES	YES
30	a	72.00	0.16564	YES	YES
31	a	73.43	0.29025	YES	YES
32	a	75.79	0.57808	YES	YES
33	a	80.31	0.30629	YES	YES
34	a	84.17	0.12258	YES	YES
35	a	92.27	0.41907	YES	YES
36	a	93.66	0.17512	YES	YES
37	a	94.18	0.02214	YES	YES
38	a	103.61	0.01282	YES	YES
39	a	105.74	0.72842	YES	YES
40	a	117.81	3.02022	YES	YES
41	a	118.57	0.46418	YES	YES
42	a	123.98	7.03255	YES	YES
43	a	130.99	1.13293	YES	YES
44	a	132.43	1.50673	YES	YES
45	a	138.12	1.76317	YES	YES
46	a	141.92	3.99881	YES	YES
47	a	143.86	3.72324	YES	YES
48	a	147.16	7.76889	YES	YES
49	a	153.50	2.58727	YES	YES
50	a	158.84	3.41354	YES	YES
51	a	161.48	26.24452	YES	YES
52	a	171.96	2.25829	YES	YES
53	a	176.09	0.93528	YES	YES
54	a	182.83	6.65762	YES	YES
55	a	185.08	8.68697	YES	YES
56	a	188.37	1.96106	YES	YES
57	a	195.88	1.71390	YES	YES
58	a	199.60	3.65987	YES	YES
59	a	214.30	0.50794	YES	YES
60	a	215.40	0.09954	YES	YES
61	a	217.92	0.11891	YES	YES
62	a	221.23	0.20129	YES	YES
63	a	223.19	0.69098	YES	YES
64	a	223.73	0.81950	YES	YES
65	a	228.66	0.34970	YES	YES
66	a	230.30	2.89458	YES	YES
67	a	234.24	3.03431	YES	YES
68	a	235.89	2.81866	YES	YES
69	a	236.33	2.91746	YES	YES
70	a	239.45	1.93926	YES	YES
71	a	241.33	1.10545	YES	YES
72	a	244.31	0.39735	YES	YES
73	a	245.54	1.83443	YES	YES
74	a	249.87	3.40229	YES	YES
75	a	260.96	1.90325	YES	YES

76	a	276.80	9.17427	YES	YES
77	a	280.11	1.35572	YES	YES
78	a	285.18	1.91864	YES	YES
79	a	286.62	0.92576	YES	YES
80	a	293.26	0.84563	YES	YES
81	a	299.43	0.38823	YES	YES
82	a	304.07	1.08283	YES	YES
83	a	313.49	0.75988	YES	YES
84	a	316.47	0.20728	YES	YES
85	a	331.16	0.89782	YES	YES
86	a	332.07	0.23751	YES	YES
87	a	336.17	0.06901	YES	YES
88	a	337.32	0.56969	YES	YES
89	a	339.90	0.72780	YES	YES
90	a	341.38	1.13054	YES	YES
91	a	368.72	0.21914	YES	YES
92	a	377.65	2.43839	YES	YES
93	a	380.11	2.36771	YES	YES
94	a	382.58	0.38974	YES	YES
95	a	387.08	0.05798	YES	YES
96	a	395.52	0.99992	YES	YES
97	a	398.03	0.16472	YES	YES
98	a	406.01	0.83643	YES	YES
99	a	412.48	1.47081	YES	YES
100	a	418.90	1.77541	YES	YES
101	a	422.32	3.80105	YES	YES
102	a	423.34	1.91189	YES	YES
103	a	429.11	0.43571	YES	YES
104	a	429.57	0.05191	YES	YES
105	a	430.67	0.38686	YES	YES
106	a	431.61	0.06078	YES	YES
107	a	431.89	0.06621	YES	YES
108	a	433.32	0.04304	YES	YES
109	a	435.28	1.35301	YES	YES
110	a	435.49	0.96480	YES	YES
111	a	437.81	0.06464	YES	YES
112	a	445.20	1.76359	YES	YES
113	a	460.02	3.30232	YES	YES
114	a	460.71	4.62827	YES	YES
115	a	469.81	2.89710	YES	YES
116	a	488.43	3.58931	YES	YES
117	a	491.60	3.62226	YES	YES
118	a	492.82	1.89283	YES	YES
119	a	496.15	2.90715	YES	YES
120	a	498.01	0.65614	YES	YES
121	a	499.19	0.73505	YES	YES
122	a	503.58	0.31280	YES	YES
123	a	505.31	0.52233	YES	YES
124	a	514.08	5.68759	YES	YES
125	a	515.21	12.57645	YES	YES
126	a	534.91	5.79461	YES	YES
127	a	543.16	45.08466	YES	YES
128	a	548.68	2.92306	YES	YES
129	a	592.97	0.06760	YES	YES
130	a	629.87	20.25023	YES	YES
131	a	634.99	15.42537	YES	YES
132	a	645.44	9.51900	YES	YES
133	a	667.96	13.28744	YES	YES

134	a	691.61	3.44994	YES	YES
135	a	701.87	1.42836	YES	YES
136	a	717.57	3.51196	YES	YES
137	a	718.26	0.50169	YES	YES
138	a	729.81	7.58923	YES	YES
139	a	731.34	2.70685	YES	YES
140	a	733.88	5.38747	YES	YES
141	a	734.58	3.61543	YES	YES
142	a	739.80	4.23161	YES	YES
143	a	742.43	9.82298	YES	YES
144	a	770.37	7.83008	YES	YES
145	a	771.74	42.41666	YES	YES
146	a	772.45	2.13720	YES	YES
147	a	773.72	0.18091	YES	YES
148	a	774.41	0.48651	YES	YES
149	a	775.12	0.65301	YES	YES
150	a	777.03	0.14389	YES	YES
151	a	777.68	0.64065	YES	YES
152	a	779.73	1.33567	YES	YES
153	a	781.79	12.74440	YES	YES
154	a	792.24	13.93851	YES	YES
155	a	806.07	0.93503	YES	YES
156	a	810.41	2.98490	YES	YES
157	a	811.96	1.49315	YES	YES
158	a	812.20	7.56518	YES	YES
159	a	813.21	1.85722	YES	YES
160	a	814.00	1.38249	YES	YES
161	a	815.61	3.93904	YES	YES
162	a	818.98	1.80254	YES	YES
163	a	833.49	4.89512	YES	YES
164	a	833.81	5.70580	YES	YES
165	a	835.86	5.44471	YES	YES
166	a	837.24	3.16451	YES	YES
167	a	837.66	0.77985	YES	YES
168	a	838.36	2.25106	YES	YES
169	a	839.41	5.48929	YES	YES
170	a	840.57	1.72871	YES	YES
171	a	849.54	12.56540	YES	YES
172	a	856.99	15.84330	YES	YES
173	a	868.01	0.24272	YES	YES
174	a	874.19	2.15120	YES	YES
175	a	875.04	2.25587	YES	YES
176	a	875.88	2.29584	YES	YES
177	a	877.16	3.34929	YES	YES
178	a	877.80	3.15004	YES	YES
179	a	878.58	3.94213	YES	YES
180	a	879.15	5.92224	YES	YES
181	a	880.42	4.50100	YES	YES
182	a	880.69	6.12069	YES	YES
183	a	881.36	1.04150	YES	YES
184	a	881.80	2.43933	YES	YES
185	a	882.28	3.07366	YES	YES
186	a	883.83	6.23734	YES	YES
187	a	885.79	0.62833	YES	YES
188	a	887.28	3.26062	YES	YES
189	a	888.04	1.52466	YES	YES
190	a	903.80	2.62781	YES	YES
191	a	904.94	1.23809	YES	YES

192	a	905.09	1.93834	YES	YES
193	a	906.31	2.62049	YES	YES
194	a	909.33	1.50101	YES	YES
195	a	911.21	0.58115	YES	YES
196	a	911.32	1.90415	YES	YES
197	a	911.92	0.75752	YES	YES
198	a	942.76	71.03066	YES	YES
199	a	957.51	0.47905	YES	YES
200	a	975.39	0.39770	YES	YES
201	a	986.63	2.38914	YES	YES
202	a	987.96	9.24864	YES	YES
203	a	989.26	8.74248	YES	YES
204	a	989.83	16.46326	YES	YES
205	a	990.30	3.09160	YES	YES
206	a	991.50	20.90005	YES	YES
207	a	991.58	5.12789	YES	YES
208	a	992.17	4.42108	YES	YES
209	a	995.34	2.96469	YES	YES
210	a	1015.12	0.25111	YES	YES
211	a	1015.60	0.77031	YES	YES
212	a	1015.80	0.20251	YES	YES
213	a	1018.15	0.07009	YES	YES
214	a	1019.18	0.12207	YES	YES
215	a	1020.05	0.70560	YES	YES
216	a	1020.44	0.93650	YES	YES
217	a	1021.23	0.58041	YES	YES
218	a	1030.58	1.04467	YES	YES
219	a	1031.09	2.66151	YES	YES
220	a	1031.67	0.93872	YES	YES
221	a	1034.66	0.95672	YES	YES
222	a	1036.21	1.11558	YES	YES
223	a	1039.02	3.07000	YES	YES
224	a	1040.28	1.37633	YES	YES
225	a	1042.94	1.34895	YES	YES
226	a	1043.97	1.19990	YES	YES
227	a	1058.34	1.56177	YES	YES
228	a	1059.11	1.03795	YES	YES
229	a	1059.39	1.11865	YES	YES
230	a	1060.86	0.76142	YES	YES
231	a	1061.85	1.30290	YES	YES
232	a	1067.55	0.88694	YES	YES
233	a	1068.17	0.33954	YES	YES
234	a	1070.41	0.76889	YES	YES
235	a	1070.80	0.08876	YES	YES
236	a	1071.55	0.11408	YES	YES
237	a	1072.38	0.22405	YES	YES
238	a	1072.98	0.06977	YES	YES
239	a	1073.12	0.04174	YES	YES
240	a	1073.99	0.15535	YES	YES
241	a	1074.23	1.20739	YES	YES
242	a	1074.84	0.75711	YES	YES
243	a	1076.59	12.84528	YES	YES
244	a	1078.24	2.64804	YES	YES
245	a	1085.50	3.67302	YES	YES
246	a	1092.22	1.12004	YES	YES
247	a	1096.59	2.97533	YES	YES
248	a	1098.04	4.97540	YES	YES
249	a	1098.49	8.22835	YES	YES

250	a	1103.92	1.31469	YES	YES
251	a	1105.74	2.72433	YES	YES
252	a	1109.34	10.14496	YES	YES
253	a	1114.78	7.46541	YES	YES
254	a	1121.99	1.12009	YES	YES
255	a	1133.11	2.78814	YES	YES
256	a	1143.86	1.17627	YES	YES
257	a	1160.10	8.59767	YES	YES
258	a	1161.56	0.41004	YES	YES
259	a	1162.59	12.61558	YES	YES
260	a	1166.49	5.53334	YES	YES
261	a	1169.45	11.17065	YES	YES
262	a	1169.85	5.36257	YES	YES
263	a	1171.44	2.91275	YES	YES
264	a	1172.31	6.78709	YES	YES
265	a	1179.66	17.40290	YES	YES
266	a	1182.38	4.26589	YES	YES
267	a	1183.78	24.92495	YES	YES
268	a	1184.96	4.21602	YES	YES
269	a	1187.58	30.08364	YES	YES
270	a	1189.51	9.57147	YES	YES
271	a	1193.05	2.01925	YES	YES
272	a	1195.76	6.24742	YES	YES
273	a	1212.04	27.29383	YES	YES
274	a	1214.41	0.40356	YES	YES
275	a	1239.91	0.26748	YES	YES
276	a	1244.45	0.56449	YES	YES
277	a	1246.10	0.22048	YES	YES
278	a	1246.64	1.24899	YES	YES
279	a	1247.45	0.05759	YES	YES
280	a	1248.19	2.12825	YES	YES
281	a	1249.23	1.04182	YES	YES
282	a	1250.34	0.21646	YES	YES
283	a	1250.57	0.13098	YES	YES
284	a	1252.34	0.86782	YES	YES
285	a	1254.36	0.39630	YES	YES
286	a	1254.58	4.52278	YES	YES
287	a	1255.48	2.57403	YES	YES
288	a	1256.34	5.50154	YES	YES
289	a	1257.26	4.24174	YES	YES
290	a	1257.65	9.32334	YES	YES
291	a	1257.98	4.69496	YES	YES
292	a	1258.63	0.60311	YES	YES
293	a	1259.43	4.03013	YES	YES
294	a	1261.60	2.76131	YES	YES
295	a	1262.00	1.42113	YES	YES
296	a	1263.22	1.95522	YES	YES
297	a	1263.90	2.66236	YES	YES
298	a	1265.85	0.39989	YES	YES
299	a	1267.92	0.80235	YES	YES
300	a	1270.16	1.32660	YES	YES
301	a	1270.91	1.09582	YES	YES
302	a	1274.48	0.75234	YES	YES
303	a	1282.70	2.99275	YES	YES
304	a	1284.16	2.88335	YES	YES
305	a	1286.41	1.18337	YES	YES
306	a	1286.61	4.89244	YES	YES
307	a	1289.98	1.44949	YES	YES

308	a	1291.71	4.11444	YES	YES
309	a	1292.90	4.20056	YES	YES
310	a	1293.14	1.85763	YES	YES
311	a	1306.59	1.84392	YES	YES
312	a	1309.81	1.62694	YES	YES
313	a	1310.47	0.64313	YES	YES
314	a	1313.65	0.59199	YES	YES
315	a	1314.31	2.00242	YES	YES
316	a	1318.26	2.26184	YES	YES
317	a	1319.36	0.78765	YES	YES
318	a	1320.64	0.39255	YES	YES
319	a	1321.57	1.24088	YES	YES
320	a	1321.87	1.11372	YES	YES
321	a	1322.02	1.43232	YES	YES
322	a	1323.02	0.20209	YES	YES
323	a	1323.46	0.21312	YES	YES
324	a	1324.27	1.76412	YES	YES
325	a	1324.33	0.23298	YES	YES
326	a	1324.47	1.82159	YES	YES
327	a	1325.46	4.39448	YES	YES
328	a	1326.17	1.22064	YES	YES
329	a	1326.39	2.84235	YES	YES
330	a	1327.05	1.12887	YES	YES
331	a	1328.57	2.42088	YES	YES
332	a	1331.44	2.58788	YES	YES
333	a	1334.95	0.92079	YES	YES
334	a	1336.63	0.35751	YES	YES
335	a	1336.78	0.52518	YES	YES
336	a	1337.10	0.55801	YES	YES
337	a	1337.79	0.22472	YES	YES
338	a	1338.01	0.73009	YES	YES
339	a	1338.15	1.76674	YES	YES
340	a	1339.30	0.48951	YES	YES
341	a	1339.39	0.22203	YES	YES
342	a	1340.02	6.23453	YES	YES
343	a	1340.25	3.06934	YES	YES
344	a	1344.13	0.45248	YES	YES
345	a	1345.64	0.33672	YES	YES
346	a	1346.53	0.66198	YES	YES
347	a	1346.94	2.72938	YES	YES
348	a	1348.26	1.85989	YES	YES
349	a	1349.22	1.26239	YES	YES
350	a	1349.27	0.18212	YES	YES
351	a	1349.85	0.64817	YES	YES
352	a	1400.73	5.49041	YES	YES
353	a	1405.39	5.58853	YES	YES
354	a	1407.74	5.82291	YES	YES
355	a	1413.20	17.78197	YES	YES
356	a	1420.09	28.40459	YES	YES
357	a	1422.99	7.19985	YES	YES
358	a	1426.65	4.86412	YES	YES
359	a	1430.49	19.10681	YES	YES
360	a	1430.98	13.58918	YES	YES
361	a	1432.18	10.58459	YES	YES
362	a	1432.95	14.62039	YES	YES
363	a	1433.96	1.78167	YES	YES
364	a	1435.41	4.05958	YES	YES
365	a	1435.57	14.67225	YES	YES

366	a	1436.43	1.12739	YES	YES
367	a	1436.57	2.21689	YES	YES
368	a	1437.30	7.98711	YES	YES
369	a	1437.41	10.51015	YES	YES
370	a	1437.53	7.54880	YES	YES
371	a	1437.73	6.58893	YES	YES
372	a	1437.90	2.53109	YES	YES
373	a	1438.42	3.62741	YES	YES
374	a	1439.03	5.26232	YES	YES
375	a	1439.13	8.62817	YES	YES
376	a	1439.73	4.62035	YES	YES
377	a	1439.91	0.56079	YES	YES
378	a	1440.55	7.22506	YES	YES
379	a	1440.84	5.16944	YES	YES
380	a	1441.65	3.85368	YES	YES
381	a	1442.15	3.14436	YES	YES
382	a	1442.86	10.17495	YES	YES
383	a	1444.00	9.47328	YES	YES
384	a	1444.27	18.73414	YES	YES
385	a	1444.93	28.74590	YES	YES
386	a	1445.08	19.55036	YES	YES
387	a	1446.15	17.85016	YES	YES
388	a	1446.91	3.44667	YES	YES
389	a	1447.30	18.33421	YES	YES
390	a	1453.99	1.14354	YES	YES
391	a	1454.70	2.29350	YES	YES
392	a	1454.85	4.97332	YES	YES
393	a	1455.10	3.06451	YES	YES
394	a	1455.91	0.74649	YES	YES
395	a	1456.47	0.32023	YES	YES
396	a	1457.75	0.28124	YES	YES
397	a	1460.09	4.29257	YES	YES
398	a	1563.88	37.29682	YES	YES
399	a	1595.47	63.97794	YES	YES
400	a	2925.74	5.57821	YES	YES
401	a	2929.00	33.67914	YES	YES
402	a	2933.47	33.20744	YES	YES
403	a	2933.53	2.42486	YES	YES
404	a	2934.29	16.66830	YES	YES
405	a	2939.37	16.07112	YES	YES
406	a	2939.98	3.23828	YES	YES
407	a	2940.09	9.66993	YES	YES
408	a	2940.67	33.24031	YES	YES
409	a	2942.28	13.64033	YES	YES
410	a	2943.29	5.05478	YES	YES
411	a	2943.66	8.32814	YES	YES
412	a	2944.38	10.66563	YES	YES
413	a	2944.65	7.95621	YES	YES
414	a	2945.76	8.49341	YES	YES
415	a	2946.07	5.39048	YES	YES
416	a	2947.53	8.55238	YES	YES
417	a	2947.64	12.23615	YES	YES
418	a	2948.94	13.41793	YES	YES
419	a	2949.78	3.15012	YES	YES
420	a	2951.06	11.18948	YES	YES
421	a	2951.55	28.39032	YES	YES
422	a	2953.24	7.35574	YES	YES
423	a	2953.45	6.10926	YES	YES

424	a	2953.80	3.83293	YES	YES
425	a	2954.27	6.81839	YES	YES
426	a	2955.25	19.08781	YES	YES
427	a	2957.67	5.90087	YES	YES
428	a	2958.50	21.11542	YES	YES
429	a	2958.68	33.63360	YES	YES
430	a	2959.59	32.01845	YES	YES
431	a	2959.86	12.75555	YES	YES
432	a	2960.26	29.87773	YES	YES
433	a	2960.43	14.90320	YES	YES
434	a	2960.60	16.44578	YES	YES
435	a	2961.02	15.59923	YES	YES
436	a	2961.59	22.06054	YES	YES
437	a	2961.84	33.07961	YES	YES
438	a	2962.16	13.64120	YES	YES
439	a	2963.21	29.59381	YES	YES
440	a	2964.51	22.87108	YES	YES
441	a	2971.86	5.49005	YES	YES
442	a	2972.48	46.25842	YES	YES
443	a	2973.34	14.96234	YES	YES
444	a	2975.58	40.70541	YES	YES
445	a	2978.19	34.48623	YES	YES
446	a	2978.47	17.85120	YES	YES
447	a	2982.36	11.73001	YES	YES
448	a	2984.73	4.88299	YES	YES
449	a	2987.74	3.63022	YES	YES
450	a	2988.30	1.68454	YES	YES
451	a	2989.24	4.57922	YES	YES
452	a	2989.32	9.25278	YES	YES
453	a	2989.96	6.65594	YES	YES
454	a	2990.29	22.78032	YES	YES
455	a	2995.31	24.85344	YES	YES
456	a	2995.61	11.10194	YES	YES
457	a	3000.41	29.16850	YES	YES
458	a	3003.90	9.63478	YES	YES
459	a	3004.88	37.32459	YES	YES
460	a	3005.72	17.35579	YES	YES
461	a	3006.67	39.02924	YES	YES
462	a	3008.04	4.94330	YES	YES
463	a	3008.24	0.84640	YES	YES
464	a	3008.26	22.47389	YES	YES
465	a	3008.80	20.97811	YES	YES
466	a	3011.33	16.69363	YES	YES
467	a	3011.79	21.85593	YES	YES
468	a	3012.58	8.76612	YES	YES
469	a	3012.72	13.30177	YES	YES
470	a	3013.19	24.17639	YES	YES
471	a	3014.22	22.64913	YES	YES
472	a	3015.45	29.95619	YES	YES
473	a	3015.74	17.82379	YES	YES
474	a	3015.82	17.04889	YES	YES
475	a	3016.16	24.85390	YES	YES
476	a	3016.73	30.65125	YES	YES
477	a	3017.19	13.86589	YES	YES
478	a	3017.30	38.46557	YES	YES
479	a	3017.35	51.76945	YES	YES
480	a	3017.76	30.30393	YES	YES
481	a	3017.85	12.05389	YES	YES

482	a	3017.85	27.96259	YES	YES
483	a	3018.37	17.28963	YES	YES
484	a	3018.86	18.67870	YES	YES
485	a	3019.18	28.46936	YES	YES
486	a	3021.35	17.77788	YES	YES
487	a	3022.79	10.34082	YES	YES
488	a	3026.49	17.81714	YES	YES
489	a	3028.75	22.19647	YES	YES
490	a	3030.14	0.85826	YES	YES
491	a	3031.89	3.40370	YES	YES
492	a	3034.88	2.14184	YES	YES
493	a	3038.77	8.58521	YES	YES
494	a	3041.31	0.90115	YES	YES
495	a	3049.49	0.01605	YES	YES
496	a	3128.30	0.70443	YES	YES
497	a	3139.72	0.08159	YES	YES
498	a	3146.35	0.50251	YES	YES

$end

Double hybrid single point energy = -7778.618007731069 H
COSMO energy + OC correction = -7786.6514250075 H (in 3FB)

6.2.58 $[F\{Ga(dcpe)\}_2(C_6F_2H_3)]^{2+}$ (*anti*-periplanar)

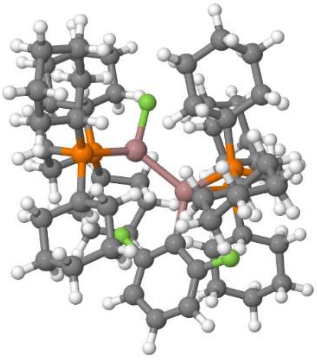

Method: (RI-)BP86(D3BJ)/def2-TZVPP
Symmetry: c1

Cartesian coordinates in Ångström:

Ga	15.5057854	5.0189822	43.3936472
Ga	13.6247904	5.4753221	41.9146561
F	11.7767433	7.2987352	40.0144176
F	16.2198428	5.6628057	40.2203165
F	15.0792802	4.2328426	44.9940404
C	13.9866319	6.4634362	40.2147589
C	13.0580848	7.2402576	39.5236125
C	15.5956023	7.1014265	38.4534156
H	16.6024938	7.0169307	38.0499339
C	14.6147379	7.8760194	37.8277836
H	14.8550941	8.4188855	36.9153946
C	13.3257673	7.9511411	38.3600300
H	12.5430898	8.5388600	37.8846484
C	15.2431400	6.4289691	39.6148371
P	12.2614909	3.5732168	41.2599838
P	11.6503228	6.2521513	43.0602144
C	11.9987699	3.7707045	39.4475006

H	11.9133230	4.8656876	39.3406699
C	10.7421171	3.1532059	38.8179544
H	9.8358170	3.4768102	39.3494028
H	10.7805238	2.0566216	38.8877662
C	10.6587016	3.5696344	37.3410414
H	9.7728929	3.1121867	36.8803417
H	10.5139637	4.6612696	37.2854021
C	11.9252235	3.1811070	36.5696532
H	12.0036911	2.0821221	36.5299574
H	11.8523896	3.5240158	35.5289391
C	13.1859989	3.7527351	37.2275582
H	13.1743738	4.8536966	37.1681216
H	14.0859247	3.4170301	36.6938657
C	13.2747924	3.3419387	38.7010460
H	13.3873616	2.2483480	38.7744171
H	14.1646422	3.7895845	39.1687330
C	12.8089747	1.8731444	41.7011959
H	13.7163472	1.7113887	41.0995365
C	11.7872339	0.7784133	41.3431454
H	10.8392811	0.9689383	41.8709404
H	11.5644655	0.7991648	40.2691162
C	12.3172446	-0.6058579	41.7410136
H	11.5641391	-1.3673865	41.4979535
H	13.2068671	-0.8390455	41.1326497
C	12.6844472	-0.6627379	43.2255766
H	11.7753252	-0.5247882	43.8338765
H	13.0826075	-1.6535987	43.4817219
C	13.7028701	0.4239521	43.5791438
H	14.6479420	0.2199198	43.0488558
H	13.9360921	0.4075498	44.6526058
C	13.1983930	1.8187346	43.1902847
H	12.3205402	2.0595969	43.8121982
H	13.9558747	2.5816671	43.4234985
C	10.6465326	3.8073790	42.1193499
H	9.8611597	3.2727227	41.5692122
H	10.7381087	3.3209433	43.1002078
C	10.2872179	5.2856211	42.2810138
H	9.3699056	5.3993074	42.8745244
H	10.1043175	5.7555511	41.3049030
C	11.0520008	7.9971068	42.9776997
H	10.5999569	8.0333605	41.9723233
C	12.2023708	9.0136349	42.9929349
H	12.9234235	8.7580431	42.2051341
H	12.7360794	8.9580571	43.9545520
C	11.6737591	10.4367957	42.7798929
H	12.5063588	11.1519917	42.8396205
H	11.2643147	10.5155940	41.7595166
C	10.5841539	10.7927249	43.7958373
H	11.0205593	10.8179744	44.8083732
H	10.1968939	11.8011343	43.5987066
C	9.4429739	9.7718979	43.7611363
H	8.9397776	9.8137084	42.7813997
H	8.6820865	10.0160631	44.5147245
C	9.9589763	8.3464709	44.0036435
H	10.3735240	8.2834929	45.0208101
H	9.1283045	7.6288343	43.9501033
C	11.8599827	5.6138557	44.7803075
H	12.4284045	4.6761988	44.6310195

C	12.7705454	6.5501448	45.5978905
H	12.2722987	7.5224791	45.7306728
H	13.7057278	6.7435168	45.0468653
C	13.0801590	5.9380387	46.9681714
H	13.6916893	5.0363114	46.8155933
H	13.6865370	6.6429491	47.5535340
C	11.7987116	5.5815342	47.7272660
H	12.0486070	5.1124959	48.6883869
H	11.2404848	6.5021454	47.9654586
C	10.9071500	4.6499432	46.9010080
H	9.9784863	4.4219857	47.4414081
H	11.4264412	3.6899932	46.7435305
C	10.5680325	5.2685122	45.5380374
H	9.9661761	6.1755316	45.6888259
H	9.9489882	4.5717591	44.9544302
P	17.0268566	6.8391386	43.9419502
P	17.5381957	3.7794136	42.8804639
C	16.9417106	8.0242234	42.5320442
H	16.7577530	7.3636016	41.6682040
C	18.1978790	8.8489290	42.2166961
H	19.0681562	8.1948978	42.0641167
H	18.4407042	9.5108257	43.0606721
C	17.9543439	9.6901508	40.9547439
H	18.8451868	10.2948076	40.7381198
H	17.8154190	9.0128420	40.0954276
C	16.7193065	10.5853452	41.1002784
H	16.9003737	11.3288798	41.8937430
H	16.5531401	11.1532943	40.1750743
C	15.4716138	9.7660815	41.4465063
H	15.2169473	9.1050307	40.6027432
H	14.6088892	10.4286649	41.6012415
C	15.6999302	8.9127834	42.6982073
H	15.8328968	9.5700524	43.5722474
H	14.8195945	8.2861292	42.9052708
C	16.8240830	7.6642441	45.5716165
H	15.8090253	8.0971903	45.5305506
C	17.8350226	8.7929817	45.8388972
H	17.7684435	9.5632296	45.0592375
H	18.8575808	8.3841003	45.7993553
C	17.5914556	9.4215163	47.2182365
H	18.3455542	10.1987581	47.4018181
H	16.6128515	9.9295983	47.2125073
C	17.6140866	8.3711036	48.3320137
H	17.4050299	8.8429872	49.3012741
H	18.6255056	7.9385965	48.4075339
C	16.6031859	7.2546185	48.0564853
H	15.5830953	7.6723702	48.0812169
H	16.6456300	6.4867899	48.8409804
C	16.8429731	6.6015647	46.6898167
H	17.8295746	6.1093511	46.7059898
H	16.0981899	5.8191639	46.4881771
C	18.7100596	6.0888522	43.8917627
H	19.4650627	6.8804808	43.8006195
H	18.8803687	5.5894171	44.8546543
C	18.8257183	5.0911509	42.7303954
H	19.8239108	4.6346603	42.7091465
H	18.6773862	5.5904877	41.7633183
C	17.6674272	2.6362293	41.4328456

```
H    18.2258830    1.7624740   41.8102310
C    16.2520467    2.1808294   41.0398832
H    15.6700000    3.0721673   40.7517613
H    15.7489550    1.7424510   41.9127660
C    16.2836698    1.1889464   39.8752600
H    16.7778817    0.2594034   40.2008064
H    15.2568444    0.9102501   39.5944316
C    17.0307130    1.7765493   38.6748798
H    17.0718925    1.0463909   37.8557888
H    16.4750889    2.6496892   38.2927648
C    18.4464930    2.2088244   39.0702516
H    18.9682735    2.6524479   38.2115038
H    19.0297692    1.3217018   39.3656171
C    18.4312262    3.2144480   40.2303634
H    19.4608745    3.4685144   40.5168013
H    17.9460767    4.1428291   39.8961696
C    17.9530784    2.8253156   44.4029325
H    17.7321296    3.5195677   45.2324111
C    16.9957555    1.6281769   44.5467740
H    15.9574794    1.9836069   44.5318579
H    17.1317406    0.9447307   43.6926906
C    17.2782212    0.8640322   45.8453292
H    17.0382942    1.5150443   46.7015133
H    16.6073009   -0.0036717   45.9124053
C    18.7416545    0.4206847   45.9263254
H    18.9315540   -0.0952385   46.8769307
H    18.9469496   -0.3115753   45.1277724
C    19.6902627    1.6135487   45.7767765
H    20.7371830    1.2817377   45.7951471
H    19.5611639    2.2943174   46.6342985
C    19.4249069    2.3912416   44.4788996
H    19.6656670    1.7505861   43.6144069
H    20.0953654    3.2610844   44.4256711

SCF energy GEOOPT =  -7786.509781137 H
ZPE =   3797. kJ/mol
FREEH energy =  3995.14 kJ/mol
FREEH entropy =   1.67563 kJ/mol/K

$vibrational spectrum
#  mode     symmetry     wave number   IR intensity    selection rules
#                         cm**(-1)        km/mol          IR     RAMAN
     1                      -0.00         0.00000        -        -
     2                       0.00         0.00000        -        -
     3                       0.00         0.00000        -        -
     4                       0.00         0.00000        -        -
     5                       0.00         0.00000        -        -
     6                       0.00         0.00000        -        -
     7         a             9.24         0.01557       YES      YES
     8         a            14.04         0.03080       YES      YES
     9         a            21.84         0.02248       YES      YES
    10         a            27.67         0.16634       YES      YES
    11         a            30.72         0.08833       YES      YES
    12         a            33.11         0.04666       YES      YES
    13         a            35.86         0.06009       YES      YES
    14         a            37.05         0.10373       YES      YES
    15         a            39.39         0.02519       YES      YES
    16         a            42.28         0.10669       YES      YES
```

17	a	44.89	0.39769	YES	YES
18	a	46.01	0.38341	YES	YES
19	a	46.96	0.01385	YES	YES
20	a	52.72	0.14802	YES	YES
21	a	53.37	0.20648	YES	YES
22	a	54.33	0.15677	YES	YES
23	a	55.05	0.23465	YES	YES
24	a	57.71	0.07565	YES	YES
25	a	59.79	0.14960	YES	YES
26	a	63.58	0.20096	YES	YES
27	a	64.99	0.19218	YES	YES
28	a	67.89	0.14474	YES	YES
29	a	69.30	0.25632	YES	YES
30	a	70.25	0.05742	YES	YES
31	a	73.20	0.28569	YES	YES
32	a	76.05	0.27214	YES	YES
33	a	78.54	0.12813	YES	YES
34	a	78.81	0.09322	YES	YES
35	a	85.92	0.23394	YES	YES
36	a	88.40	0.80099	YES	YES
37	a	91.77	0.21903	YES	YES
38	a	101.91	0.48415	YES	YES
39	a	111.54	1.27066	YES	YES
40	a	116.82	0.52846	YES	YES
41	a	121.56	1.12152	YES	YES
42	a	122.47	0.54521	YES	YES
43	a	128.82	0.59950	YES	YES
44	a	133.11	13.87996	YES	YES
45	a	134.56	5.11095	YES	YES
46	a	137.81	1.64007	YES	YES
47	a	145.31	5.65376	YES	YES
48	a	154.87	1.32902	YES	YES
49	a	157.95	0.57647	YES	YES
50	a	160.63	4.49759	YES	YES
51	a	162.20	8.56338	YES	YES
52	a	167.76	8.80044	YES	YES
53	a	173.95	0.45053	YES	YES
54	a	184.24	6.98891	YES	YES
55	a	185.78	8.08973	YES	YES
56	a	189.57	12.70141	YES	YES
57	a	195.97	1.05063	YES	YES
58	a	199.28	5.36061	YES	YES
59	a	208.78	0.28946	YES	YES
60	a	210.98	0.03269	YES	YES
61	a	218.64	0.40917	YES	YES
62	a	220.47	1.57041	YES	YES
63	a	224.69	1.44848	YES	YES
64	a	229.05	0.55385	YES	YES
65	a	230.07	0.24039	YES	YES
66	a	231.68	0.27767	YES	YES
67	a	232.86	1.88275	YES	YES
68	a	233.25	1.68014	YES	YES
69	a	235.02	0.81896	YES	YES
70	a	239.35	0.76055	YES	YES
71	a	239.58	1.70147	YES	YES
72	a	241.75	5.75279	YES	YES
73	a	250.76	0.37811	YES	YES
74	a	253.27	1.05772	YES	YES

75	a	273.10	6.65503	YES	YES
76	a	276.91	1.67477	YES	YES
77	a	283.74	0.15350	YES	YES
78	a	284.64	0.56179	YES	YES
79	a	286.62	2.25824	YES	YES
80	a	306.93	1.09838	YES	YES
81	a	307.90	0.08588	YES	YES
82	a	309.19	0.05069	YES	YES
83	a	310.64	0.46828	YES	YES
84	a	312.83	0.04922	YES	YES
85	a	330.93	0.55180	YES	YES
86	a	332.95	0.48754	YES	YES
87	a	337.05	0.52338	YES	YES
88	a	338.68	0.19807	YES	YES
89	a	339.31	0.58611	YES	YES
90	a	342.45	1.73885	YES	YES
91	a	371.92	0.35346	YES	YES
92	a	377.49	0.72722	YES	YES
93	a	378.91	0.62532	YES	YES
94	a	381.66	0.31478	YES	YES
95	a	387.18	0.95163	YES	YES
96	a	387.41	1.00420	YES	YES
97	a	393.58	0.15983	YES	YES
98	a	405.16	0.19234	YES	YES
99	a	406.04	0.77436	YES	YES
100	a	419.16	1.47759	YES	YES
101	a	420.33	1.53074	YES	YES
102	a	425.84	0.55983	YES	YES
103	a	426.99	0.20211	YES	YES
104	a	430.16	0.07719	YES	YES
105	a	430.26	0.07117	YES	YES
106	a	430.88	0.09349	YES	YES
107	a	431.18	0.13924	YES	YES
108	a	432.65	0.45687	YES	YES
109	a	435.29	0.12582	YES	YES
110	a	444.28	5.50492	YES	YES
111	a	444.64	2.79026	YES	YES
112	a	452.99	2.82139	YES	YES
113	a	464.61	3.88557	YES	YES
114	a	465.42	3.77461	YES	YES
115	a	469.17	3.93706	YES	YES
116	a	487.67	1.93046	YES	YES
117	a	490.91	4.41730	YES	YES
118	a	493.17	1.26075	YES	YES
119	a	494.45	0.62275	YES	YES
120	a	498.09	0.56112	YES	YES
121	a	498.88	0.01750	YES	YES
122	a	501.69	0.24911	YES	YES
123	a	505.86	1.04946	YES	YES
124	a	510.91	5.46908	YES	YES
125	a	511.85	11.36103	YES	YES
126	a	534.22	3.87762	YES	YES
127	a	547.26	4.03587	YES	YES
128	a	550.71	45.96959	YES	YES
129	a	592.24	0.01953	YES	YES
130	a	633.18	15.49844	YES	YES
131	a	636.57	16.26724	YES	YES
132	a	656.62	7.98564	YES	YES

133	a	661.45	12.17719	YES	YES
134	a	692.60	3.87525	YES	YES
135	a	698.44	1.39576	YES	YES
136	a	711.27	5.16079	YES	YES
137	a	716.14	6.47936	YES	YES
138	a	718.40	9.33929	YES	YES
139	a	731.37	4.06743	YES	YES
140	a	740.87	2.04389	YES	YES
141	a	741.86	0.70526	YES	YES
142	a	742.18	12.78774	YES	YES
143	a	742.82	7.19765	YES	YES
144	a	769.31	0.39164	YES	YES
145	a	772.16	0.23581	YES	YES
146	a	772.53	0.05659	YES	YES
147	a	774.10	42.56109	YES	YES
148	a	774.20	8.61989	YES	YES
149	a	774.88	0.39751	YES	YES
150	a	775.10	0.13434	YES	YES
151	a	775.49	0.11917	YES	YES
152	a	779.95	0.03929	YES	YES
153	a	790.62	22.25337	YES	YES
154	a	795.64	20.42728	YES	YES
155	a	806.45	1.73298	YES	YES
156	a	807.47	0.58889	YES	YES
157	a	808.62	1.73390	YES	YES
158	a	809.60	2.82053	YES	YES
159	a	814.82	1.01032	YES	YES
160	a	815.69	2.74621	YES	YES
161	a	817.03	4.54684	YES	YES
162	a	819.00	1.89541	YES	YES
163	a	832.75	2.20322	YES	YES
164	a	834.00	1.23196	YES	YES
165	a	835.47	7.51128	YES	YES
166	a	835.90	3.78878	YES	YES
167	a	838.02	3.05924	YES	YES
168	a	838.92	2.69169	YES	YES
169	a	839.08	4.11694	YES	YES
170	a	840.95	5.89317	YES	YES
171	a	851.85	21.98235	YES	YES
172	a	854.03	10.59386	YES	YES
173	a	871.40	0.05759	YES	YES
174	a	873.90	7.26859	YES	YES
175	a	874.09	0.32752	YES	YES
176	a	875.47	0.39590	YES	YES
177	a	876.84	3.49387	YES	YES
178	a	877.16	7.92906	YES	YES
179	a	877.33	1.92709	YES	YES
180	a	878.08	4.86197	YES	YES
181	a	879.09	1.34760	YES	YES
182	a	879.67	0.20001	YES	YES
183	a	880.82	2.91227	YES	YES
184	a	882.03	1.02722	YES	YES
185	a	882.46	2.56860	YES	YES
186	a	883.21	2.21681	YES	YES
187	a	884.24	1.42908	YES	YES
188	a	884.72	2.40099	YES	YES
189	a	887.18	5.93731	YES	YES
190	a	903.76	0.77767	YES	YES

191	a	904.52	1.19707	YES	YES
192	a	905.09	3.97126	YES	YES
193	a	905.59	1.00626	YES	YES
194	a	906.36	3.60810	YES	YES
195	a	907.47	2.35685	YES	YES
196	a	908.04	4.17447	YES	YES
197	a	909.07	0.32415	YES	YES
198	a	936.60	68.36896	YES	YES
199	a	961.09	0.44149	YES	YES
200	a	982.75	7.26946	YES	YES
201	a	984.92	12.45367	YES	YES
202	a	986.18	10.63621	YES	YES
203	a	986.64	2.18273	YES	YES
204	a	990.42	25.62856	YES	YES
205	a	991.39	4.45664	YES	YES
206	a	991.96	26.23018	YES	YES
207	a	992.65	1.51115	YES	YES
208	a	992.99	9.08339	YES	YES
209	a	997.34	0.54252	YES	YES
210	a	1013.21	0.35284	YES	YES
211	a	1013.61	0.51749	YES	YES
212	a	1015.66	0.69878	YES	YES
213	a	1017.15	0.31017	YES	YES
214	a	1017.55	1.40634	YES	YES
215	a	1018.38	0.18830	YES	YES
216	a	1018.51	0.05476	YES	YES
217	a	1019.32	0.49787	YES	YES
218	a	1031.24	3.24090	YES	YES
219	a	1032.62	4.16107	YES	YES
220	a	1033.06	3.19775	YES	YES
221	a	1033.47	1.18347	YES	YES
222	a	1035.75	0.52067	YES	YES
223	a	1036.95	2.35966	YES	YES
224	a	1039.42	1.52300	YES	YES
225	a	1040.68	1.14660	YES	YES
226	a	1042.29	0.61824	YES	YES
227	a	1056.29	0.20710	YES	YES
228	a	1057.76	3.75477	YES	YES
229	a	1059.86	2.80631	YES	YES
230	a	1060.37	2.61832	YES	YES
231	a	1060.51	1.84687	YES	YES
232	a	1061.91	0.71142	YES	YES
233	a	1063.17	1.30107	YES	YES
234	a	1065.36	0.46317	YES	YES
235	a	1068.72	0.03857	YES	YES
236	a	1069.98	0.96008	YES	YES
237	a	1071.17	0.08541	YES	YES
238	a	1071.32	0.22823	YES	YES
239	a	1071.98	0.79709	YES	YES
240	a	1072.78	0.03600	YES	YES
241	a	1072.89	0.14127	YES	YES
242	a	1073.57	0.22106	YES	YES
243	a	1075.50	3.82224	YES	YES
244	a	1077.86	15.60667	YES	YES
245	a	1080.35	1.50084	YES	YES
246	a	1093.20	1.13353	YES	YES
247	a	1095.25	0.17439	YES	YES
248	a	1097.17	5.53721	YES	YES

249	a	1099.53	6.44824	YES	YES
250	a	1106.39	7.30056	YES	YES
251	a	1107.58	2.18306	YES	YES
252	a	1108.00	5.57717	YES	YES
253	a	1114.58	9.88900	YES	YES
254	a	1121.83	9.03774	YES	YES
255	a	1128.05	1.62960	YES	YES
256	a	1144.23	1.43763	YES	YES
257	a	1162.70	10.84384	YES	YES
258	a	1163.56	1.20114	YES	YES
259	a	1164.76	12.38511	YES	YES
260	a	1165.59	1.75217	YES	YES
261	a	1166.28	6.69971	YES	YES
262	a	1167.61	6.39939	YES	YES
263	a	1169.63	7.01082	YES	YES
264	a	1171.15	0.91131	YES	YES
265	a	1184.35	3.37712	YES	YES
266	a	1184.83	2.46930	YES	YES
267	a	1185.52	12.34603	YES	YES
268	a	1187.46	4.13929	YES	YES
269	a	1188.17	49.06763	YES	YES
270	a	1194.05	3.30459	YES	YES
271	a	1200.15	9.94630	YES	YES
272	a	1200.93	5.73332	YES	YES
273	a	1205.37	14.67637	YES	YES
274	a	1214.00	1.00700	YES	YES
275	a	1238.42	1.38901	YES	YES
276	a	1240.22	1.50167	YES	YES
277	a	1242.17	0.63881	YES	YES
278	a	1244.21	0.06987	YES	YES
279	a	1246.07	0.23584	YES	YES
280	a	1246.69	0.37160	YES	YES
281	a	1247.65	0.82412	YES	YES
282	a	1248.85	0.67034	YES	YES
283	a	1249.15	0.72375	YES	YES
284	a	1249.61	0.55513	YES	YES
285	a	1251.71	0.71342	YES	YES
286	a	1252.33	0.75313	YES	YES
287	a	1253.40	1.22120	YES	YES
288	a	1255.79	2.73287	YES	YES
289	a	1256.68	1.05722	YES	YES
290	a	1257.77	0.84500	YES	YES
291	a	1258.02	3.98914	YES	YES
292	a	1258.30	3.48267	YES	YES
293	a	1260.05	3.11094	YES	YES
294	a	1260.80	5.58234	YES	YES
295	a	1262.25	0.32932	YES	YES
296	a	1262.35	2.90343	YES	YES
297	a	1263.17	4.18023	YES	YES
298	a	1264.38	2.17156	YES	YES
299	a	1265.53	2.07565	YES	YES
300	a	1267.21	1.77761	YES	YES
301	a	1270.40	2.01641	YES	YES
302	a	1273.64	2.63083	YES	YES
303	a	1282.24	0.46624	YES	YES
304	a	1284.25	3.82809	YES	YES
305	a	1284.75	0.34021	YES	YES
306	a	1285.64	0.20170	YES	YES

307	a	1286.24	13.59912	YES	YES
308	a	1287.41	4.28792	YES	YES
309	a	1290.83	9.19269	YES	YES
310	a	1291.50	0.23130	YES	YES
311	a	1306.73	0.77831	YES	YES
312	a	1306.89	1.87020	YES	YES
313	a	1308.44	0.67127	YES	YES
314	a	1309.22	1.01061	YES	YES
315	a	1310.12	1.44300	YES	YES
316	a	1311.21	3.24120	YES	YES
317	a	1312.11	1.09896	YES	YES
318	a	1315.47	0.59387	YES	YES
319	a	1319.87	4.53289	YES	YES
320	a	1322.58	2.85109	YES	YES
321	a	1322.59	1.29786	YES	YES
322	a	1323.13	1.12803	YES	YES
323	a	1323.26	1.20225	YES	YES
324	a	1323.68	0.62751	YES	YES
325	a	1323.86	0.89265	YES	YES
326	a	1324.22	0.32287	YES	YES
327	a	1324.30	1.41536	YES	YES
328	a	1325.02	0.62005	YES	YES
329	a	1325.50	0.77662	YES	YES
330	a	1325.73	6.70933	YES	YES
331	a	1327.19	5.45087	YES	YES
332	a	1327.61	3.36257	YES	YES
333	a	1330.14	1.26490	YES	YES
334	a	1330.89	0.30109	YES	YES
335	a	1335.72	0.63345	YES	YES
336	a	1336.91	0.29003	YES	YES
337	a	1337.02	0.28519	YES	YES
338	a	1337.28	2.38475	YES	YES
339	a	1337.67	2.73700	YES	YES
340	a	1337.98	2.10321	YES	YES
341	a	1338.01	0.83155	YES	YES
342	a	1338.14	3.23808	YES	YES
343	a	1338.32	1.41712	YES	YES
344	a	1343.45	1.03538	YES	YES
345	a	1344.21	3.17429	YES	YES
346	a	1344.57	2.56899	YES	YES
347	a	1345.53	2.11278	YES	YES
348	a	1345.98	1.95878	YES	YES
349	a	1347.40	1.38788	YES	YES
350	a	1348.15	1.03737	YES	YES
351	a	1349.04	0.50213	YES	YES
352	a	1404.84	7.17769	YES	YES
353	a	1410.87	12.84102	YES	YES
354	a	1411.94	7.73056	YES	YES
355	a	1412.18	19.52449	YES	YES
356	a	1420.43	17.28604	YES	YES
357	a	1424.54	0.22894	YES	YES
358	a	1425.27	66.57910	YES	YES
359	a	1426.73	13.41111	YES	YES
360	a	1427.17	3.45751	YES	YES
361	a	1430.33	4.83599	YES	YES
362	a	1430.98	5.73757	YES	YES
363	a	1434.09	1.21825	YES	YES
364	a	1434.38	8.78745	YES	YES

365	a	1435.84	4.14259	YES	YES
366	a	1436.45	3.01188	YES	YES
367	a	1436.55	4.83010	YES	YES
368	a	1436.82	5.43684	YES	YES
369	a	1437.46	2.02185	YES	YES
370	a	1437.92	0.89715	YES	YES
371	a	1438.24	4.73980	YES	YES
372	a	1438.58	0.74332	YES	YES
373	a	1438.70	5.28901	YES	YES
374	a	1439.13	3.91295	YES	YES
375	a	1439.17	8.69751	YES	YES
376	a	1439.29	0.37649	YES	YES
377	a	1439.55	1.45356	YES	YES
378	a	1440.31	6.48279	YES	YES
379	a	1440.66	10.44441	YES	YES
380	a	1440.89	7.01469	YES	YES
381	a	1441.22	10.41532	YES	YES
382	a	1443.12	7.86334	YES	YES
383	a	1443.40	15.63485	YES	YES
384	a	1444.26	4.71950	YES	YES
385	a	1444.36	15.08856	YES	YES
386	a	1445.75	16.20428	YES	YES
387	a	1446.17	11.39947	YES	YES
388	a	1446.34	21.40939	YES	YES
389	a	1448.71	2.32253	YES	YES
390	a	1453.36	1.22355	YES	YES
391	a	1454.67	2.04860	YES	YES
392	a	1454.81	0.82404	YES	YES
393	a	1455.43	1.88260	YES	YES
394	a	1456.48	1.27826	YES	YES
395	a	1457.30	2.92533	YES	YES
396	a	1457.44	1.75436	YES	YES
397	a	1458.04	3.12845	YES	YES
398	a	1561.20	34.17383	YES	YES
399	a	1594.18	68.34051	YES	YES
400	a	2912.30	2.87176	YES	YES
401	a	2934.23	2.92535	YES	YES
402	a	2937.38	4.02973	YES	YES
403	a	2937.97	4.86179	YES	YES
404	a	2939.13	37.22689	YES	YES
405	a	2940.97	9.54726	YES	YES
406	a	2942.10	8.41945	YES	YES
407	a	2942.91	9.21162	YES	YES
408	a	2943.91	8.00352	YES	YES
409	a	2944.35	12.39605	YES	YES
410	a	2944.43	5.09583	YES	YES
411	a	2944.52	6.71840	YES	YES
412	a	2944.82	1.65803	YES	YES
413	a	2944.91	13.77158	YES	YES
414	a	2945.05	13.19068	YES	YES
415	a	2945.84	18.82588	YES	YES
416	a	2945.98	12.65380	YES	YES
417	a	2949.33	0.64010	YES	YES
418	a	2949.62	8.97836	YES	YES
419	a	2949.92	20.80230	YES	YES
420	a	2950.38	11.77413	YES	YES
421	a	2951.25	7.34070	YES	YES
422	a	2953.29	4.73412	YES	YES

423	a	2953.46	12.64133	YES	YES
424	a	2954.30	7.18551	YES	YES
425	a	2954.80	23.38778	YES	YES
426	a	2954.93	24.08358	YES	YES
427	a	2955.09	18.73540	YES	YES
428	a	2955.71	5.77251	YES	YES
429	a	2956.32	5.99201	YES	YES
430	a	2957.21	12.51279	YES	YES
431	a	2957.91	12.84252	YES	YES
432	a	2959.44	25.94817	YES	YES
433	a	2959.61	20.26495	YES	YES
434	a	2959.75	26.64665	YES	YES
435	a	2961.31	7.76580	YES	YES
436	a	2961.95	37.59217	YES	YES
437	a	2962.47	43.14414	YES	YES
438	a	2962.95	51.89613	YES	YES
439	a	2963.61	3.28137	YES	YES
440	a	2965.25	8.83599	YES	YES
441	a	2968.90	27.08825	YES	YES
442	a	2971.15	14.30650	YES	YES
443	a	2971.26	18.97888	YES	YES
444	a	2971.73	15.72197	YES	YES
445	a	2973.45	5.48245	YES	YES
446	a	2978.21	10.73567	YES	YES
447	a	2985.30	7.20854	YES	YES
448	a	2986.70	1.52546	YES	YES
449	a	2988.37	9.23925	YES	YES
450	a	2988.98	22.17587	YES	YES
451	a	2990.85	1.75902	YES	YES
452	a	2992.77	24.06941	YES	YES
453	a	2993.37	3.50591	YES	YES
454	a	2994.33	44.27883	YES	YES
455	a	2995.00	29.09740	YES	YES
456	a	2996.43	24.36631	YES	YES
457	a	2998.63	23.19345	YES	YES
458	a	3004.04	28.68148	YES	YES
459	a	3008.06	3.15747	YES	YES
460	a	3008.94	21.12357	YES	YES
461	a	3008.99	25.26761	YES	YES
462	a	3010.08	12.31097	YES	YES
463	a	3010.22	9.25464	YES	YES
464	a	3010.30	32.23474	YES	YES
465	a	3011.28	12.81149	YES	YES
466	a	3011.37	11.29596	YES	YES
467	a	3011.71	22.03986	YES	YES
468	a	3012.32	20.77529	YES	YES
469	a	3012.58	11.55174	YES	YES
470	a	3013.83	31.32462	YES	YES
471	a	3013.85	10.72064	YES	YES
472	a	3014.34	12.11306	YES	YES
473	a	3014.58	27.93758	YES	YES
474	a	3015.51	25.78339	YES	YES
475	a	3015.98	24.98934	YES	YES
476	a	3016.24	35.54905	YES	YES
477	a	3016.43	21.17998	YES	YES
478	a	3016.54	13.98568	YES	YES
479	a	3016.67	34.69873	YES	YES
480	a	3016.83	11.18488	YES	YES

481	a	3017.25	26.39423	YES	YES
482	a	3017.53	22.27352	YES	YES
483	a	3017.96	17.38459	YES	YES
484	a	3019.07	23.06286	YES	YES
485	a	3019.27	27.03249	YES	YES
486	a	3019.46	12.43774	YES	YES
487	a	3020.00	36.64834	YES	YES
488	a	3022.44	49.66597	YES	YES
489	a	3024.95	2.78208	YES	YES
490	a	3025.18	20.73619	YES	YES
491	a	3027.38	23.34174	YES	YES
492	a	3031.58	0.78983	YES	YES
493	a	3035.62	0.85738	YES	YES
494	a	3039.98	0.30503	YES	YES
495	a	3043.54	0.61801	YES	YES
496	a	3128.92	0.62796	YES	YES
497	a	3140.09	0.03588	YES	YES
498	a	3146.65	0.92999	YES	YES

$end

Double hybrid single point energy = -7778.632643750329 H
COSMO energy + OC correction = -7786.6619437140 H (in 3FB)

6.2.59 [*pf*]⁻

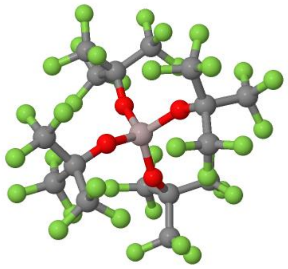

Method: (RI-)BP86(D3BJ)/def2-TZVPP
Symmetry: c1

Cartesian coordinates in Ångström:

Al	-0.3538979	0.1565687	-0.1776142
O	-0.3651207	-1.4200661	-0.9385292
O	1.3077435	0.4931415	0.2588694
O	-0.9052459	1.4480779	-1.2199807
O	-1.4478824	0.0704481	1.1875528
C	2.4787280	0.0170572	0.7294964
F	2.1732888	-2.3863041	0.6888456
C	2.3003778	-1.3368540	1.5299149
C	-0.6357213	2.3208140	-2.2115072
F	-2.0370396	1.3641571	-3.9357280
C	-2.1495954	0.7597648	2.1105070
F	-3.1767061	0.3060471	4.2896849
C	-1.1056434	-2.4678983	-1.3529385
F	1.7605367	2.1181859	-2.4977573
C	0.5831901	1.8529584	-3.1058013
F	0.6080092	2.4605589	-4.3208766
F	0.5239198	0.5239350	-3.3124793
C	-1.9218542	2.4427852	-3.1258117
F	-1.9009519	3.5466442	-3.9192826
F	-3.0344141	2.5031530	-2.3733626
C	-0.3008540	3.7421243	-1.6010587

F	0.2672368	4.5807011	-2.5087016
F	-1.4215301	4.3470653	-1.1413137
F	0.5473965	3.6210674	-0.5662757
F	4.5447235	-0.9994677	-0.1127905
F	1.1875785	-1.2843179	2.2861813
F	3.3481326	-1.6018011	2.3539075
C	3.4801148	-0.2325445	-0.4706422
F	3.9706807	0.9375918	-0.9436404
F	2.8517575	-0.8487838	-1.4861130
F	2.9621223	2.3361913	1.1759681
C	3.0998280	1.1042342	1.6970569
F	2.4678063	1.0965163	2.8940521
F	4.4235227	0.9032641	1.9345091
F	-3.9163509	0.8314247	0.4759204
C	-3.3354219	1.5674902	1.4394931
F	-4.3013684	1.9151982	2.3330737
F	-2.8849524	2.7075209	0.8718573
F	-3.2036503	-2.9614390	-2.5230925
C	-2.2583411	-2.0049851	-2.3302931
F	-2.8745599	-0.9135603	-1.8369723
F	-1.7598515	-1.6780030	-3.5441855
F	-0.8243920	-4.3731859	-2.8710306
C	-0.1459671	-3.4604235	-2.1256851
F	0.6350325	-4.1466107	-1.2587251
F	0.6687284	-2.7835276	-2.9541291
F	-0.8815806	-3.3036117	0.8952258
C	-1.7533767	-3.2329801	-0.1258270
F	-2.8558728	-2.5905232	0.3183296
F	-2.1252826	-4.5038582	-0.4398313
F	-1.9249878	2.6962223	3.5997257
C	-2.7587589	-0.2803365	3.1361237
F	-1.8482589	-1.2148682	3.4606249
F	-3.8247346	-0.9195965	2.6001132
C	-1.2231397	1.7694483	2.8977182
F	-0.4206947	2.4282507	2.0387580
F	-0.4296921	1.1122640	3.7736550

SCF energy GEOOPT = -4750.100769695 H
ZPE = 556.1 kJ/mol
FREEH energy = 704.29 kJ/mol
FREEH entropy = 1.36309 kJ/mol/K

$vibrational spectrum

# mode	symmetry	wave number	IR intensity	selection rules	
#		cm**(-1)	km/mol	IR	RAMAN
1		-0.00	0.00000	-	-
2		-0.00	0.00000	-	-
3		-0.00	0.00000	-	-
4		-0.00	0.00000	-	-
5		0.00	0.00000	-	-
6		0.00	0.00000	-	-
7	a	12.14	0.00088	YES	YES
8	a	14.29	0.00166	YES	YES
9	a	15.82	0.00035	YES	YES
10	a	18.84	0.00147	YES	YES
11	a	28.46	0.06613	YES	YES
12	a	28.97	0.02162	YES	YES
13	a	34.00	0.01822	YES	YES
14	a	35.78	0.01486	YES	YES
15	a	36.96	0.08264	YES	YES
16	a	41.35	0.05066	YES	YES
17	a	44.17	0.05300	YES	YES

18	a	55.79	0.00469	YES	YES
19	a	58.87	0.00450	YES	YES
20	a	67.30	0.11202	YES	YES
21	a	69.15	0.22878	YES	YES
22	a	71.34	0.16263	YES	YES
23	a	72.82	0.13599	YES	YES
24	a	74.95	0.00725	YES	YES
25	a	76.06	0.04382	YES	YES
26	a	76.33	0.08430	YES	YES
27	a	78.77	0.02043	YES	YES
28	a	79.34	0.03030	YES	YES
29	a	83.51	0.06965	YES	YES
30	a	84.65	0.03538	YES	YES
31	a	86.28	0.00412	YES	YES
32	a	88.48	0.05023	YES	YES
33	a	94.11	0.81131	YES	YES
34	a	95.20	0.63727	YES	YES
35	a	96.36	0.92782	YES	YES
36	a	110.00	0.00732	YES	YES
37	a	154.87	0.06074	YES	YES
38	a	155.27	0.07315	YES	YES
39	a	158.98	0.25295	YES	YES
40	a	159.68	0.01893	YES	YES
41	a	161.32	0.61641	YES	YES
42	a	161.86	0.62425	YES	YES
43	a	163.23	0.06244	YES	YES
44	a	164.35	0.70335	YES	YES
45	a	191.66	3.40987	YES	YES
46	a	193.21	2.74368	YES	YES
47	a	193.47	2.56120	YES	YES
48	a	215.42	0.00775	YES	YES
49	a	257.85	0.66938	YES	YES
50	a	258.32	0.59175	YES	YES
51	a	265.91	3.10218	YES	YES
52	a	267.02	0.01450	YES	YES
53	a	271.45	2.72323	YES	YES
54	a	271.69	2.71180	YES	YES
55	a	274.24	3.01085	YES	YES
56	a	275.82	0.02677	YES	YES
57	a	278.41	0.04230	YES	YES
58	a	278.67	0.03023	YES	YES
59	a	278.98	0.03524	YES	YES
60	a	279.30	0.01240	YES	YES
61	a	296.20	8.44749	YES	YES
62	a	296.83	7.21009	YES	YES
63	a	297.10	7.33376	YES	YES
64	a	302.12	0.00409	YES	YES
65	a	305.81	0.05641	YES	YES
66	a	306.09	0.05818	YES	YES
67	a	310.76	0.22834	YES	YES
68	a	311.77	0.02555	YES	YES
69	a	314.22	1.77185	YES	YES
70	a	314.44	1.78833	YES	YES
71	a	316.43	2.02678	YES	YES
72	a	319.06	0.10187	YES	YES
73	a	336.70	0.74181	YES	YES
74	a	336.89	0.86631	YES	YES
75	a	342.79	2.89438	YES	YES
76	a	349.85	8.37095	YES	YES
77	a	350.15	10.01561	YES	YES
78	a	351.47	2.32253	YES	YES
79	a	366.31	32.74171	YES	YES

80	a	377.70	0.25755	YES	YES
81	a	424.78	51.11989	YES	YES
82	a	435.92	60.82615	YES	YES
83	a	437.33	58.54028	YES	YES
84	a	504.36	0.05727	YES	YES
85	a	508.29	1.29690	YES	YES
86	a	508.46	3.10185	YES	YES
87	a	508.64	1.30362	YES	YES
88	a	509.12	1.07345	YES	YES
89	a	509.52	2.02030	YES	YES
90	a	509.85	4.82870	YES	YES
91	a	509.95	7.94918	YES	YES
92	a	510.17	7.26728	YES	YES
93	a	514.60	2.66144	YES	YES
94	a	515.31	1.94413	YES	YES
95	a	515.38	1.75398	YES	YES
96	a	518.41	0.01329	YES	YES
97	a	533.64	22.23075	YES	YES
98	a	539.02	20.82454	YES	YES
99	a	539.14	20.13014	YES	YES
100	a	543.22	0.15474	YES	YES
101	a	543.34	0.24194	YES	YES
102	a	543.60	0.23582	YES	YES
103	a	543.79	0.36454	YES	YES
104	a	544.63	0.45707	YES	YES
105	a	545.53	3.08530	YES	YES
106	a	547.11	13.52608	YES	YES
107	a	547.51	14.58518	YES	YES
108	a	694.41	1.51306	YES	YES
109	a	694.60	1.28448	YES	YES
110	a	695.16	0.28264	YES	YES
111	a	695.38	0.53084	YES	YES
112	a	696.19	9.41710	YES	YES
113	a	696.95	74.73584	YES	YES
114	a	697.29	73.94284	YES	YES
115	a	697.49	72.54285	YES	YES
116	a	711.64	0.00563	YES	YES
117	a	721.86	2.65177	YES	YES
118	a	723.22	4.59454	YES	YES
119	a	723.37	4.39762	YES	YES
120	a	760.70	0.02067	YES	YES
121	a	796.28	8.50174	YES	YES
122	a	805.97	16.93403	YES	YES
123	a	807.32	16.95684	YES	YES
124	a	924.57	15.70989	YES	YES
125	a	924.78	12.93731	YES	YES
126	a	928.60	4.89130	YES	YES
127	a	929.48	0.81151	YES	YES
128	a	932.47	246.83697	YES	YES
129	a	933.23	115.68420	YES	YES
130	a	935.71	322.19670	YES	YES
131	a	935.92	311.33576	YES	YES
132	a	1054.63	11.82460	YES	YES
133	a	1055.80	11.65854	YES	YES
134	a	1057.35	10.69399	YES	YES
135	a	1064.00	0.10254	YES	YES
136	a	1074.06	1.98165	YES	YES
137	a	1074.54	0.43994	YES	YES
138	a	1076.33	4.14625	YES	YES
139	a	1079.23	4.78011	YES	YES
140	a	1079.35	24.17992	YES	YES
141	a	1083.37	27.41318	YES	YES

142	a	1084.13	17.77077	YES	YES
143	a	1085.24	17.79678	YES	YES
144	a	1140.03	7.05863	YES	YES
145	a	1142.66	6.34285	YES	YES
146	a	1143.57	9.34849	YES	YES
147	a	1148.55	1.80739	YES	YES
148	a	1150.79	8.49246	YES	YES
149	a	1156.06	15.55514	YES	YES
150	a	1157.07	10.46109	YES	YES
151	a	1159.34	95.26427	YES	YES
152	a	1168.07	1279.96386	YES	YES
153	a	1168.29	1311.61166	YES	YES
154	a	1169.47	1193.81607	YES	YES
155	a	1182.24	1.80195	YES	YES
156	a	1183.46	90.36447	YES	YES
157	a	1185.04	31.52730	YES	YES
158	a	1186.04	65.71882	YES	YES
159	a	1187.64	97.98562	YES	YES
160	a	1188.29	166.07991	YES	YES
161	a	1190.44	63.67834	YES	YES
162	a	1200.16	130.54824	YES	YES
163	a	1202.35	122.59652	YES	YES
164	a	1207.59	980.05079	YES	YES
165	a	1209.64	912.69425	YES	YES
166	a	1210.71	750.25926	YES	YES
167	a	1217.14	14.11441	YES	YES
168	a	1295.98	250.06913	YES	YES
169	a	1296.76	276.44271	YES	YES
170	a	1298.92	289.83931	YES	YES
171	a	1324.06	2.10289	YES	YES

$end

7 References

[1] I. Krossing, A. Reisinger, *Coord. Chem. Rev.*, 2006, ***250***, 2721–2744.

[2] a) I. Krossing, *Chem. Eur. J.*, 2001, ***7***, 490–502; b) P. J. Malinowski, D. Himmel, I. Krossing, *Angew. Chem. Int. Ed.*, 2016, ***55***, 9259–9261.

[3] J. M. Slattery, A. Higelin, T. Bayer, I. Krossing, *Angew. Chem. Int. Ed.*, 2010, ***49***, 3228–3231.

[4] A. Barthélemy, H. Scherer, M. Daub, A. Bugnet, I. Krossing, *Angew. Chem. Int. Ed.*, 2023, ***62***, e202311648.

[5] a) M. Guillén, S. Liu, C. D. Díaz-Oviedo, M. Klussmann, B. List, *ACS Catal.*, 2024, ***14***, 751–756; b) C. D. Díaz-Oviedo, R. Maji, B. List, *J. Am. Chem. Soc.*, 2021, ***143***, 20598–20604.

[6] J. Cosier, A. M. Glazer, *J. Appl. Crystallogr.*, 1986, ***19***, 105–107.

[7] a) L. Krause, R. Herbst-Irmer, G. M. Sheldrick, D. Stalke, *J. Appl. Crystallogr.*, 2015, ***48***, 3–10; b) Bruker, *SADABS, 2016/2*, Bruker AXS Inc., Madison, Wisconsin, USA; c) Bruker, *SAINT, V8.40A*, Bruker AXS Inc., Madison, Wisconsin, USA.

[8] a) G. M. Sheldrick, *Acta Cryst. A*, 2015, ***71***, 3–8; b) G. M. Sheldrick, *Acta Cryst. C*, 2015, ***71***, 3–8.

[9] C. B. Hübschle, G. M. Sheldrick, B. Dittrich, *J. Appl. Crystallogr.*, 2011, ***44***, 1281–1284.

[10] a) D. Kratzert, J. J. Holstein, I. Krossing, *J. Appl. Crystallogr.*, 2015, ***48***, 933–938; b) D. Kratzert, I. Krossing, *J. Appl. Crystallogr.*, 2018, ***51***, 928–934.

[11] O. V. Dolomanov, L. J. Bourhis, R. J. Gildea, J. A. K. Howard, H. Puschmann, *J. Appl. Crystallogr.*, 2009, ***42***, 339–341.

[12] C. R. Groom, I. J. Bruno, M. P. Lightfoot, S. C. Ward, *Acta Cryst. B*, 2016, ***72***, 171–179.

[13] D. Kratzert, *FinalCif, V66*, can be found under: https://www.xs3.uni-freiburg.de/research/finalcif.

[14] R. K. Harris, E. D. Becker, S. M. Cabral de Menezes, R. Goodfellow, P. Granger, *Magn. Reson. Chem.*, 2002, ***40***, 489–505.

[15] O. Treutler, R. Ahlrichs, *J. Chem. Phys.*, 1995, ***102***, 346–354.

[16] a) R. Ahlrichs, M. Bär, M. Häser, H. Horn, C. Kölmel, *Chem. Phys. Lett.*, 1989, ***162***, 165–169; b) M. von Arnim, R. Ahlrichs, *J. Comput. Chem.*, 1998, ***19***, 1746–1757.

[17] F. Weigend, *Phys. Chem. Chem. Phys.*, 2006, ***8***, 1057–1065.

[18] a) M. Sierka, A. Hogekamp, R. Ahlrichs, *J. Chem. Phys.*, 2003, ***118***, 9136–9148; b) R. Ahlrichs, *Phys. Chem. Chem. Phys.*, 2004, ***6***, 5119.

[19] a) S. Grimme, J. Antony, S. Ehrlich, H. Krieg, *J. Chem. Phys.*, 2010, ***132***, 154104; b) S. Grimme, S. Ehrlich, L. Goerigk, *J. Comput. Chem.*, 2011, ***32***, 1456–1465.

[20] a) Becke, *Phys. Rev. A*, 1988, ***38***, 3098–3100; b) Perdew, *Phys. Rev. B*, 1986, ***33***, 8822–8824; c) J. P. Perdew, *Phys. Rev. B*, 1986, ***34***, 7406.

[21] F. Weigend, R. Ahlrichs, *Phys. Chem. Chem. Phys.*, 2005, ***7***, 3297–3305.

[22] P. Deglmann, F. Furche, R. Ahlrichs, *Chem. Phys. Lett.*, 2002, ***362***, 511–518.

[23] J. C. Sancho-García, C. Adamo, *Phys. Chem. Chem. Phys.*, 2013, ***15***, 14581–14594.

[24] a) F. Neese, *WIREs Comput. Mol. Sci.*, 2012, ***2***, 73–78; b) F. Neese, *WIREs Comput. Mol. Sci.*, 2018, ***8***.

[25] a) S. Kozuch, J. M. L. Martin, *Phys. Chem. Chem. Phys.*, 2011, ***13***, 20104–20107; b) S. Kozuch, J. M. L. Martin, *J. Comput. Chem.*, 2013, ***34***, 2327–2344.

[26] S. Kossmann, F. Neese, *J. Chem. Theory Comput.*, 2010, ***6***, 2325–2338.

[27] A. Hellweg, C. Hättig, S. Höfener, W. Klopper, *Theor. Chem. Acc.*, 2007, ***117***, 587–597.

[28] a) L. Goerigk, A. Hansen, C. Bauer, S. Ehrlich, A. Najibi, S. Grimme, *Phys. Chem. Chem. Phys.*, 2017, ***19***, 32184–32215; b) J. M. L. Martin, G. Santra, *Isr. J. Chem.*, 2020, ***60***, 787–804.

[29] A. Klamt, G. Schüürmann, *J. Chem. Soc., Perkin Trans. 2*, 1993, 799–805.

[30] C. Wohlfarth, in *CRC handbook of chemistry and physics*, ed. W. M. Haynes, D. R. Lide, T. J. Bruno, W. M. Haynes, CRC Press, Boca Raton, 97th edition, 2017, 6-199–6-220.

[31] C. Armbruster, M. Sellin, M. Seiler, T. Würz, F. Oesten, M. Schmucker, T. Sterbak, J. Fischer, V. Radtke, J. Hunger et al., *Nat. Commun.*, 2024, ***15***, 6721.

[32] N. V. Shvydkiy, D. S. Perekalin, *Coord. Chem. Rev.*, 2020, ***411***, 213238.

[33] a) W. Uhl, M. Layh, T. Hildenbrand, *J. Organomet. Chem.*, 1989, ***364***, 289–300; b) G. Linti , R. Frey, M. Schmidt, *Z. Naturforsch. B*, 1994, ***49***, 958–962; c) M. Bernasconi, G. L. Chiarotti, E. Tosatti, *Physical review. B, Condensed matter*, 1995, ***52***, 9988–9998; d) D. S. Brown, A. Decken, A. H. Cowley, *J. Am. Chem. Soc.*, 1995, ***117***, 5421–5422; e) B. Cordero, V. Gómez, A. E. Platero-Prats, M. Revés, J. Echeverría, E. Cremades, F. Barragán, S. Alvarez, *Dalton Trans.*, 2008, 2832–2838; f) A. Barthélemy, H. Scherer, H. Weller, I. Krossing,

Chem. Commun., 2023, ***59***, 1353–1356; g) M. Edwards, J. Hicks, C. McManus, J. M. Goicoechea, S. Aldridge, *Polyhedron*, 2023, ***242***, 116520.

[34] I. Raabe, K. Wagner, K. Guttsche, M. Wang, M. Grätzel, G. Santiso-Quiñones, I. Krossing, *Chem. Eur. J.*, 2009, ***15***, 1966–1976.

[35] J. Bohnenberger, W. Feuerstein, D. Himmel, M. Daub, F. Breher, I. Krossing, *Nat. Commun.*, 2019, ***10***, 624.

[36] M. Schmitt, M. Mayländer, J. Goost, S. Richert, I. Krossing, *Angew. Chem. Int. Ed.*, 2021, ***60***, 14800–14805.
